# Systematic Characterisation of Cellular Localisation and Expression Profiles of Proteins Containing MHC Ligands

**DOI:** 10.1371/journal.pone.0007448

**Published:** 2009-10-14

**Authors:** Agnieszka S. Juncker, Mette V. Larsen, Nils Weinhold, Morten Nielsen, Søren Brunak, Ole Lund

**Affiliations:** Center for Biological Sequence Analysis, Department of Systems Biology, Technical University of Denmark, Lyngby, Denmark; Dana-Farber Cancer Institute, United States of America

## Abstract

**Background:**

Presentation of peptides on Major Histocompatibility Complex (MHC) molecules is the cornerstone in immune system activation and increased knowledge of the characteristics of MHC ligands and their source proteins is highly desirable.

**Methodology/Principal Finding:**

In the present large-scale study, we used a large data set of proteins containing experimentally identified MHC class I or II ligands and examined the proteins according to their expression profiles at the mRNA level and their Gene Ontology (GO) classification within the cellular component ontology. Proteins encoded by highly abundant mRNA were found to be much more likely to be the source of MHC ligands. Of the 2.5% most abundant mRNAs as much as 41% of the proteins encoded by these mRNAs contained MHC class I ligands. For proteins containing MHC class II ligands, the corresponding percentage was 11%. Furthermore, we found that most proteins containing MHC class I ligands were localised to the intracellular parts of the cell including the cytoplasm and nucleus. MHC class II ligand donors were, on the other hand, mostly membrane proteins.

**Conclusions/Significance:**

The results contribute to the ongoing debate concerning the nature of MHC ligand-containing proteins and can be used to extend the existing methods for MHC ligand predictions by including the source protein's localisation and expression profile. Improving the current methods is important in the growing quest for epitopes that can be used for vaccine or diagnostic purposes, especially when it comes to large DNA viruses and cancer.

## Introduction

CD8^+^ T lymphocytes are activated on recognition of MHC class I molecules in complex with peptide ligands, whereas CD4^+^ T lymphocytes are activated by MHC class II molecules in complex with peptide ligands. In both cases, the result is an immune response directed against, e.g., infected or neoplastic cells. Many methods have been developed for predicting which peptides are presented by MHC class I and II molecules [1]–[4], since these peptides could be used for vaccine or diagnostic purposes. The focus of the available methods is, however, to identify the optimal peptides from provided protein sequences, while little effort has been made towards developing schemes for prioritisation of the optimal protein candidates from a pool of proteins. To this end, increased knowledge about the general characteristics of proteins containing MHC ligands is desirable. In this study, we have focused on protein cellular localisation and expression profile.

The main function of MHC class I molecules is thought to be presentation of endogenous foreign peptide antigens. The pathway leading to MHC class I presentation begins when proteins are degraded by the proteasome, which is present in the cytosol. Accordingly, it is a widespread view that MHC class I ligands are predominantly recruited from cytosolic proteins, while transmembrane proteins only rarely donate MHC class I ligands [5]–[7]. In a study from 2004, the source of B*1801-restricted MHC class I ligands was examined. Here it was found that these peptides are derived from proteins of almost all compartments in the cell, although a small over-representation of proteins from the cytoplasmic compartment was found [8]. In an alternative view, Yewdell and Nicchitta suggest that cell-surface proteins and even secreted proteins are equally efficient and probable sources of MHC class I antigenic peptides as cytosolic or nuclear proteins [9].

In contrast to MHC class I molecules, MHC class II molecules are thought to function mainly in the presentation of *exogenous* foreign peptide antigens. Nevertheless, small-scale studies have shown that a large fraction of the presented MHC class II ligands are derived from host cell membrane-bound proteins or host proteins resident in endosomes or lysosomes [7], [10], [11].

Yewdell has proposed that the peptides presented by MHC molecules primarily originate from defective ribosomal products (DRiPs), which are defective forms of gene products that are degraded more rapidly than the standard, functional form [12]. DRiPs would enable MHC class I molecules to monitor protein synthesis rates rather than protein concentrations, and offer the possibility of rapid detection of virus-infected cells. One implication of the DRiP hypothesis is that the correlation between protein concentration and the probability that the protein donates an MHC class I ligand is expected to be weak. In line with this, it has been observed that only a limited correlation exists between the amounts of MHC class I ligands presented by the cells and the relative amounts of source proteins from which these ligands are derived [13]. Other studies have examined the correlation between mRNA levels and the cells surface density of MHC class I ligands originating from the proteins encoded by the mRNA. In one study, mRNA levels were determined using DNA microarrays, while levels of MHC class I molecules with peptide cargo were determined by mass spectrometry in both human renal cell carcinoma and autologous normal tissue [14]. In comparing mRNA levels and corresponding MHC class I ligand presentation ratios between normal versus cancer cells, no clear correlation could be found. In contrast, in a study conducted in mice a moderate correlation was found between mRNA levels and presentation of corresponding MHC class I peptides in normal versus neoplastic mouse thymocytes [15]. Furthermore, the MHC class I peptide repertoire was biased towards peptides derived from proteins encoded by high-abundant mRNA transcripts in mouse thymocytes [15].

In the present study, we aimed at characterising both MHC class I and II ligand-containing proteins with regard to their cellular localisation and mRNA expression profile. This was achieved by using the increased amount of experimentally-verified MHC ligands in combination with the availability of functional annotations and high throughput gene expression data of human proteins. Using a large data set of proteins containing experimentally identified MHC class I or II ligands obtained from the SYFPEITHI database [16], we examined the proteins according to their Gene Ontology (GO) classification within the cellular component ontology [17] and their concentration at the mRNA level [18].

## Results

### Localisation of proteins containing MHC ligands

**Figure 1:**
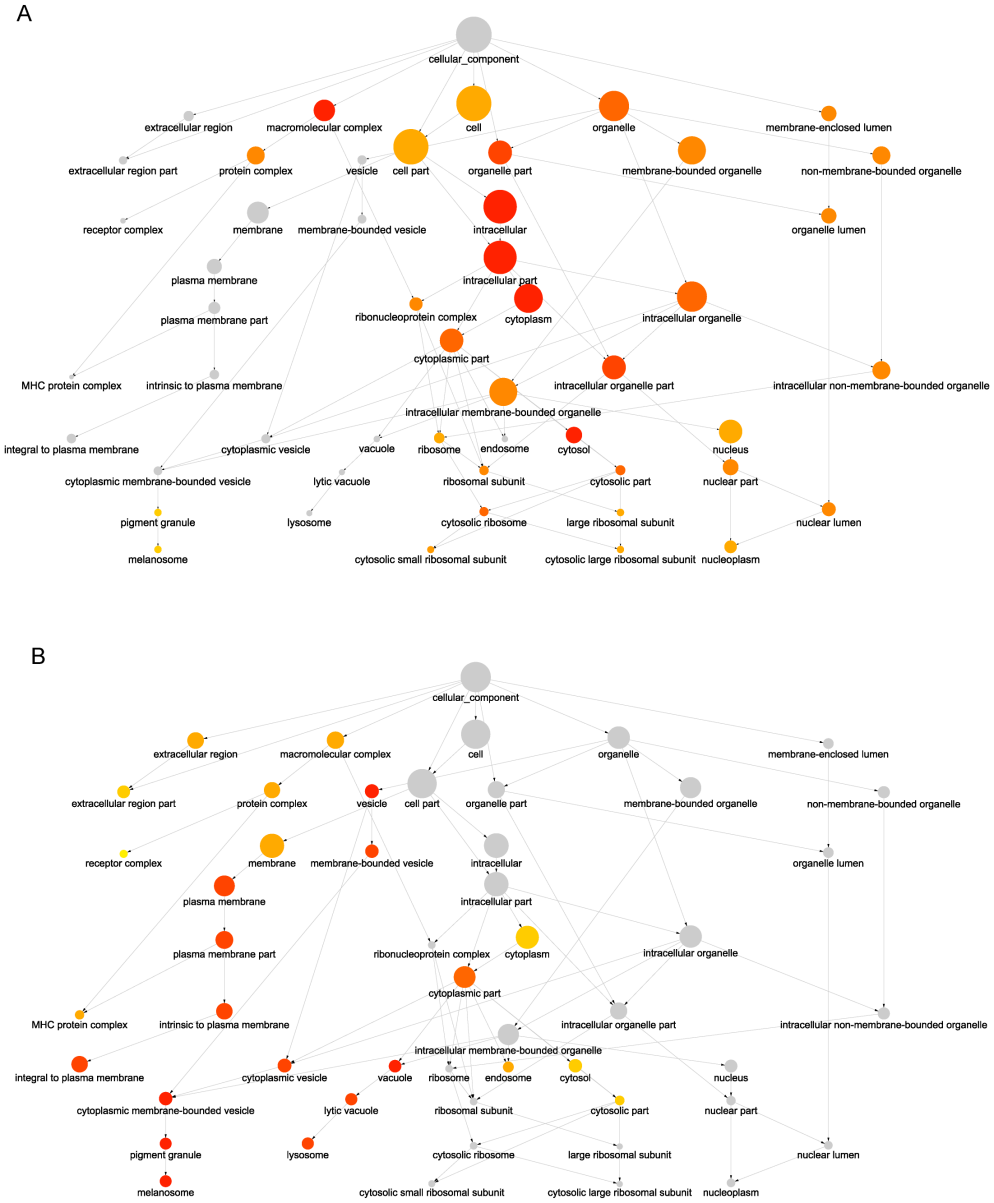
Overview of GO cellular component terms enriched among proteins containing MHC ligands. The results from an enrichment analysis were superimposed onto the GO tree structure; A: MHC class I ligand-containing proteins (MHCI data set) B: MHC class II ligand-containing proteins (MHCII data set). Only nodes representing the most significant terms are included (p-value cut-offs used as inclusion criteria is 1.00*10^−13^ for the MHCI data set and 0.01 for the MHCII data set). The significance level is reflected by the node colour, where red corresponds to the most significant p-values while grey indicates no enrichment. The size of the nodes reflects the number of proteins assigned to this term.

The SYFPEITHI database of MHC ligands and peptide motifs [16] contains no externally imposed bias with respect to protein cellular location, thus making it an ideal data set for investigating the presence of biological bias in proteins containing MHC class I or II ligands. For each MHC ligand in the SYFPEITHI database, we retrieved a possible source protein. We then analysed enrichment of GO terms in the cellular component ontology among the proteins in our two data sets of proteins containing MHC class I (MHCI data set) or MHC class II ligands (MHCII data set) as compared to a background data set of all human proteins with GO assignments. A graphical overview of the results of the analysis is shown in Figure 1, while Table 1 shows a selection of some of the most significantly enriched terms among human proteins containing MHC class I or II ligands (note that proteins can be assigned to more than one compartment resulting in a total higher than 100%).

**Table 1:** Fractions of MHC class I and II ligand-containing proteins that belong to a selection of GO cellular component terms.

Gene Ontology Term	Fraction, MHCI	Corrected P-value, MHCI	Fraction, MHCII	Corrected P-value, MHCII	Fraction, background
cell	**96.8%**	**2.05E-13**	92.4%	0.443	91.1%
intracellular	**86.7%**	**3.07E-60**	61.6%	0.938	65.3%
organelle	**69.3%**	**1.23E-33**	49.2%	0.861	51.5%
cytoplasm	**62.4%**	**8.88E-52**	**52.4%**	**4.42E-03**	40.2%
nucleus	**39.9%**	**3.57E-15**	10.8%	1	28.8%
macromolecular complex	**32.6%**	**2.94E-54**	**25.4%**	**8.58E-04**	14.6%
cytosol	**17.7%**	**3.88E-52**	**12.4%**	**1.42E-03**	5.3%
organelle lumen	**15.1%**	**1.04E-20**	5.9%	0.861	7.0%
ribonucleoprotein complex	**10.6%**	**7.69E-26**	1.6%	0.995	3.6%
cytosolic ribosom	**4.1%**	**3.93E-35**	1.6%	0.125	0.4%
membrane	33.5%	1	**58.9%**	**9.68E-05**	42.8%
plasma membrane	14.5%	1	**40.5%**	**1.81E-09**	19.9%
extracellular region	5.6%	1	**22.7%**	**2.27E-04**	11.8%
integral to plasma membrane	4.5%	1	**22.2%**	**1.04E-09**	7.0%
vesicle	4.1%	0.023	**14.1%**	**7.09E-10**	2.8%
vacoule	1.5%	0.475	**10.3%**	**6.25E-10**	1.4%
lysosome	1.2%	0.705	**9.2%**	**2.12E-09**	1.2%
pigment granule	**2.3%**	**1.65E-09**	**8.6%**	**1.27E-13**	0.5%
melanosome	**2.3%**	**1.65E-09**	**8.6%**	**1.27E-13**	0.5%

Fractions and the corresponding p-values for some of the most significantly enriched terms are marked in bold. The p-values have been corrected for multiple testing by the Benjamini & Hochberg correction method.

From Figure 1 it can be seen that the MHC class I ligand-containing proteins are mainly enriched within terms in the centre and right side of the figure. These include the terms ‘intracellular’, ‘organelle’, ‘cytoplasm’, and ‘nucleus’. As for the proteins containing MHC class II ligands, the proteins are mainly enriched within terms in the left side of Figure 1, including ‘membrane’, ‘plasma membrane’, ‘extracellular region’, ‘vacuole’, and ‘lysosome’. The MHC class II ligand-containing proteins are also slightly enriched within the term ‘cytoplasm’ and some of its child nodes (‘cytoplasmic part’, ‘cytosol’, and ‘cytosolic part’), although this enrichment is not as significant as for the MHC class I ligand-containing proteins.

Table 1 shows that for some of the compartments enriched among the proteins in the MHCII data set (‘membrane’ and ‘extracellular region’), we observe lower fractions among the proteins in the MHCI data set than in the entire human proteome as estimated from the fractions of the background data set. On the other hand, for many cellular compartments overrepresented among MHCI proteins including ‘intracellular’, ‘organelle’, and ‘nucleus’, these are underrepresented among proteins in the MHCII set.

### Expression analysis - proteins containing MHC ligands have elevated mRNA levels

**Figure 2:**
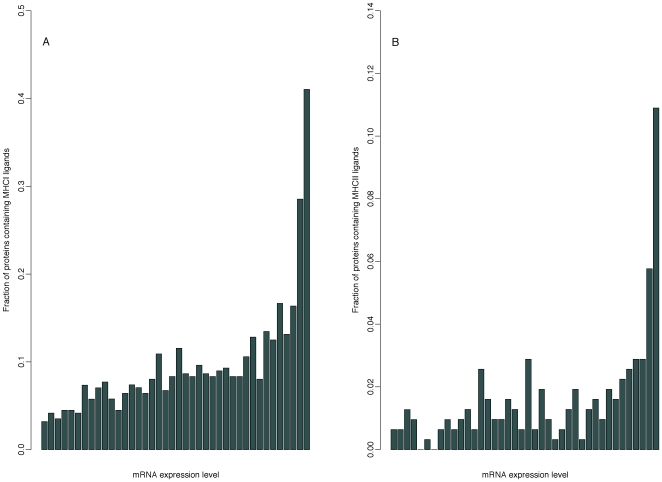
Distribution of MHC ligand-containing proteins relative to their mRNA expression level. The proteins were grouped into bins of equal size, such that each bin contains 2.5% of all proteins in the data set. As a result, each bin comprises an equal number of proteins, increasing in mRNA expression level from left to right. The height of each bar represents the fraction of proteins that contain MHC ligands. A: Fraction of proteins that contain MHC class I ligands versus the mRNA expression level of the proteins according to the GNF gene expression database B: Fraction of proteins that contain MHC class II ligands versus the mRNA expression level of the proteins according to the GNF gene expression database.

Next, we analysed the expression profile of proteins containing MHC class I or II ligands based on their mRNA abundance as determined by DNA microarray analysis obtained from the GNF gene expression database [18]. The data in the GNF expression database were produced using Affymetrix whole genome chips making them appropriate for this type of analysis. In the two histograms in Figure 2, proteins with mRNA expression data are ordered from left to right according to their mRNA level with 2.5% of the proteins in each bar. The height of the bars indicates the fraction of proteins that contain MHC ligands at this level of mRNA expression. Figure 2A shows that proteins encoded by highly abundant mRNA more often contain MHC class I ligands than proteins encoded by less abundant mRNA: In fact, 41% of the proteins encoded by mRNA found in the top 2.5% highest concentrations (the rightmost bar in Figure 2A) contain MHC class I ligands. In contrast, only 3.2% of the proteins encoded by mRNA found in the bottom 2.5% contain MHC class I ligands. Overall, we find that mRNA encoding proteins that contain MHC class I ligands are found in significantly higher concentrations than that of mRNA encoding proteins with no known MHC class I ligands (p<2.2*10^−16^, Wilcoxon rank-sum test). For proteins containing MHC class II ligands similar results are found: Figure 2B demonstrates the existence of a clear correlation between the level of mRNA and the occurrence of MHC class II ligands in the encoded proteins with an extreme increase at the right margin of the plot: For the 2.5% proteins encoded by the most abundant mRNA, the probability that the proteins contain an MHC class II ligand is 11%. Of the 2.5% proteins encoded by the least abundant mRNA only 0.6% contain MHC class II ligands. In general, proteins containing MHC class II ligands are encoded by mRNA found in significantly higher concentrations than that of proteins with no known MHC class II ligands (p<2.2*10^−16^, Wilcoxon rank-sum test).

## Discussion

In the presented study, we have used large data sets of proteins containing MHC class I or II ligands for investigating the source of these important peptides. The proteins have been characterised as regards to their cellular localisation as well as expression profile.

We have found that human proteins containing MHC class I ligands are more frequently localised to intracellular parts of the cell including the cytoplasm and nucleus than expected from the distribution in a background protein data set. In a previous study from 2004, the source of 200 B*1801-restricted MHC class I ligands were examined. It was likewise found that intracellular proteins are the most overrepresented category in terms of MHC class I presentation, although the authors state that “MHC class I peptides can be derived from proteins resident to almost every compartment in the cell and are not particularly biased toward the cytoplasmic compartment” [8]. The difference in the final conclusion of the two studies, concerning whether or not there is a bias towards MHC class I ligand-containing proteins being localised to the cytoplasm, may be found in the size of the studies: The present study includes more than five times as many MHC class I ligand-containing proteins as the earlier study. Another difference between the two studies is that the present study is not limited to proteins containing MHC class I ligands restricted by a single MHC class I allele, but includes all HLA-A and –B restricted ligands in the SYFPEITHI database. In a recent study from 2008, a modest but significant twofold enrichment in proteins located to the cytoplasm and nucleus was found for proteins containing MHC class I ligands [15].

The main pathway leading to MHC class I presentation is initiated when proteins are degraded by the proteasome. Yewdell (2002) points out that: “A common misconception is that proteasomes are located exclusively in the cytosol. In fact proteasomes are present in the nucleus at similar or higher concentration” [19]. In concordance with this observation, we find that proteins located to both the cytoplasm and nucleus are overrepresented as MHC class I ligand donors. Membrane proteins are synthesised directly into the lumen of the ER [20], which complicates their entry into the MHC class I pathway. The fact that some membrane proteins do donate MHC class I ligands is due to different mechanisms. By one mechanism, the MHC class I ligands from membrane proteins originate from the signal sequence that guides the nascent polypeptide chain to the ER. The signal sequence is liberated from the rest of the protein by the signal peptidase and may subsequently enter the lumen of the ER, where further trimming can optimise it for binding to loadable MHC class I molecules [6], [21]. Proteins which fail to fold correctly in the ER, may be transported back into the cytosol by reverse translocation for degradation by the proteasome [22]. In this study, we have only investigated the cellular localisation of *human* proteins containing MHC class I ligands. Based on work on poxvirus [23], Yewdell and Nicchitta (2006) suggest that “cell-surface proteins, and even secreted proteins are equally efficient and probable sources of antigenic peptides as cytosolic or nuclear proteins” [9]. A study concerning an MHC class I ligand in the HIV envelope (env) protein might shed light on the possible difference between the localisation of human and viral proteins containing MHC class I ligands [24]: The env protein is co-translationally translocated into the ER during synthesis, but nevertheless contains several different MHC class I ligands. One of these ligands has the sequence TAVPW**N**ASW. Although the asparagine in the ligand is N-glycosylated inside ER, the authors find that env-specific CTL clones only recognise the non-glycosylated form. In addition, they find that the naturally processed ligand is non-glycosylated. Since this specific ligand has also been shown to be TAP-dependent [25], the results are consistent with the hypothesis that the ligand is derived from cytoplasmic env that have failed to engage the translocation apparatus. It has been suggested that this failure is due to a temporary excess of signal sequence-containing nascent polypeptides over signal recognition particles [6], [24]. It may be a general phenomenon during virus infection that the amount of signal sequence-containing viral polypeptides present simultaneously overloads the translocation apparatus. This would lead to cytosolic localisation of viral membrane proteins, thus making them easily accessible for the proteasome initiating the MHC class I pathway.

For human proteins containing MHC class II ligands, we see opposite result as regards to localisation as compared to human proteins containing MHC class I ligands: More MHC class II ligand-containing proteins are localised to membranes. In particular, plasma membrane proteins are overrepresented as MHC class II donors. Several investigations have previously reported that a large fraction of MHC class II ligands are derived from host cell membrane proteins or host proteins resident in endosomes or lysosomes [7], [10], [11], [26]. The primary function of MHC class II presentation is thought to be activation of CD4^+^ T cells on uptake and degradation of antigen from the extracellular environment. Why then, are so many ligands derived from host cell membrane proteins? Chicz *et al*. (1993) suggest that the self peptides have a physiological role in modulating the immune response [10]: Since overstimulation by antigen can induce peripheral tolerance, the self ligands may serve not only to prevent antigenic peptides with short binding half-lives from binding, but also prevent over-presentation of foreign antigen in vivo. Others have proposed what they call a ‘pseudodimer’ model of T cell activation. In this model, heterodimers consisting of MHC in complex with self-antigen and MHC in complex with foreign peptide antigen, stabilised by CD4, are crucial intermediates for triggering CD4^+^ T lymphocytes [27] .

Regarding the expression profile of human MHC ligand-containing proteins, we find a strong correlation between the abundance of mRNA and the probability that the encoded protein contains an MHC ligand. This is true for proteins containing both MHC class I and II ligands, although the relationship is clearest for proteins containing MHC class I ligands. In a study from 2008 conducted in mice, a similar result was found for MHC class I ligands: Although only 9% of total mRNAs were expressed at high levels, 42% of the mRNAs encoding MHC class I ligands were expressed at high levels [15]. The results from the presents study and the study from 2008 deal with MHC ligands in a purely qualitative manner - does a particular protein contain an MHC ligand or not? Quantitative comparisons of mRNA versus MHC class I peptide levels have also been conducted. They have, however, reached varying results. In a study from 2007, mRNA levels were determined by DNA microarray analysis, while levels of MHC class I in complex with peptides were determined by mass spectrometry for 273 proteins and corresponding MHC class I peptides in samples of renal cell carcinoma and their autologous normal kidney tissue [14]. Next, the correlation between changes in abundance in mRNA levels and changes in MHC class I peptide levels in normal versus cancer cells were examined, but found to be poor (r = 0.32). In another study, the correlation was examined between changes in mRNA levels as determined by real-time PCR and changes in MHC class I peptide levels as determined by mass spectrometry in neoplastic versus normal mouse thymocytes [15]. This analysis included only 47 mRNA and corresponding MHC class I peptide-pairs, but showed a moderate correlation between relative mRNA levels and corresponding MHC class I peptide levels (r = 0.63). The authors of the latter study suggest that the stronger correlation in their case may be caused by estimation of mRNA levels by real-time PCR rather than microarrays [15]. Knowledge of the exact correlation between mRNA levels and corresponding MHC class I peptide levels may not be necessary for selecting proteins with relevance for vaccine or diagnostic use as indicated by the following observations: For CD4^+^ T lymphocytes, as little as about ten MHC class II molecules with peptide cargo are sufficient for activation [28], while for CD8^+^ T lymphocytes only three peptides are needed for induction of cytotoxicity [29]. It may therefore suffice to select proteins encoded by high-abundant mRNA, since the present results show a 41% probability that proteins encoded by the most high-abundant mRNA contain MHC class I ligands and an 11% probability that they contain MHC class II ligands.

Our findings offer the possibility of extending the existing methods for MHC ligand prediction by including the source protein's localisation and expression profile. Improving the current methods is important in the growing quest for epitopes that can be used for vaccine or diagnostic purposes, especially when it comes to large DNA viruses and cancer.

## Materials and Methods

### Data set of proteins containing MHC ligands

In May 2008, 2,164 peptides classified as MHC class I ligands and 860 peptides classified as MHC class II ligands were collected from the SYFPEITHI database [16]. The corresponding source proteins were retrieved from the UniProtKB database [30]. If more than one human protein was the possible origin of a given peptide, the longest one was chosen. 1,372 unique human source proteins were identified for 2,062 MHC class I ligands (the MHCI data set, Data set S1), while 246 unique human source proteins were identified for 699 MHC class II ligands (the MHCII data set, Data set S2).

### Localisation enrichment analysis

The Gene Ontology (GO) project provides a controlled and consistent vocabulary to describe the properties of proteins (gene products) [17]. In order to test for overrepresentation of GO terms among our two protein data sets, we applied a test in hypergeometric distribution as implemented in the Cytoscape plugin BINGO [31]. The p-values were corrected for multiple testing by the Benjamini & Hochberg correction. The analysis was performed separately for the cellular component ontology. For the 1,372 proteins in the MHCI data set, 1,109 had assignments in the cellular component ontology. For the 246 proteins in the MHCII data set, 185 had assignments in the cellular component ontology. The entire human protein set was used as background, and for this set 16,280 proteins with assignments to the cellular component ontology were available. Table S1 lists the output from the Cytoscape plugin BINGO analysis on the MHCI data set, while Table S2 lists the output for the MHCII data set.

### Expression analysis of proteins containing MHC ligands

The GNF gene expression database [18] contains mRNA expression data on 79 distinct human tissues that have been profiled in duplicates using the Affymetrix GeneChip Human Genome U133A array. The data set has previously been shown to display high reproducibility [32]. The data was normalised using robust multi-array averaging (RMA) [33] and bias correction as described in [34]. Among the tissue types in the GNF gene expression database, we only used a subset: Since the ligand data in the SYFPEITHI database has predominantly been obtained from cells belonging to the haematopoietic lineage, our analysis focuses on delineation of expression patterns in haematopoietic tissues (BDCA4 Dendritic Cells, B Lymphoblasts, CD14 Monocytes, CD19 B Cells, CD33 Myeloid Cells, CD34 Cells, CD4 T Cells, CD56 NK Cells, CD71 Early Erythroid, CD8 T Cells, Lymphoma Raji, Tonsil, WHOLEBLOOD(JJV), Bonemarrow, Leukemia, Leukemia Lymphoblastic (molt4), Leukemia Promyelocytic (hl60), Lymph, Lymphnode, Lymphoma Burkitts Daudi, and Thymus). The mRNA expression values for all tissues were present in duplicates and for subsequent analysis the median values for the tissues were calculated.

The MHC ligand-containing proteins in the MHCI and MHCII data sets were integrated with data from the GNF gene expression database [18]: We mapped the 22,283 probes on the HG U133A array to UniProtKB accession numbers, which led to 12,489 unique proteins. If more than one probe mapped to a given protein, the expression value of the mRNA encoding this protein was taken as the median of the expression values for the individual probes. Of the 12,489 unique proteins, 1,206 contained MHC class I ligands and 200 contained MHC class II ligands (Table S3).

### Wilcoxon rank-sum significance test for expression of proteins containing MHC ligands

In order to assess if proteins containing MHC ligands are significantly higher expressed than proteins not known to contain MHC ligands, we used a non-parametric Wilcoxon rank-sum test. We tested for equality of expression distribution between probes mapping to proteins in the MHCI or MHCII data set as compared to the remainder of the probes in the GNF gene expression database.

## Supporting Information

Data set S1 The MHCI data set. The amino acid sequences of the proteins in the MHCI data set in fasta format. The first column of the headings lists the UniProtKB Accession number and ID of the protein, the second column lists the starting position of the MHC class I ligand in the protein, the third column lists the amino acid sequence of the MHC class I ligand.
(2.02 MB DOC)

Data set S2 The MHCII data set. The amino acid sequences of the proteins in the MHCII data set in fasta format. The first column of the heading lists the UniProtKB Accession number and ID of the protein, the second column lists the starting position of the MHC class II ligand in the protein, the third column lists the amino acid sequence of the MHC class II ligand.
(0.56 MB DOC)

Table S1 Localisation analysis of proteins containing MHC class I ligands. The analysis was done using the Cytoscape plugin BINGO on proteins in the MHCI data set. The entire human protein set was used as background. GO-ID: The ID number of the GO cellular component term. p-value: p-value indicating the statistical significance of the difference between the fraction of proteins containing MHC class I ligands assigned to this GO term and the fraction of all proteins within the human protein set assigned to this GO term. corr p-value: The above p-value corrected for multiple testing by the Benjamini & Hochberg correction method. x: The number of MHC class I-containing proteins assigned to this GO term. nX: The number of proteins within the entire human protein set assigned to this GO term. N: The total number of MHC class I-containing proteins with assignments within the GO cellular component ontology. nN: The total number of proteins within the human protein set with assignments within the GO cellular component ontology. Description: Description of the GO term.
(0.20 MB XLS)

Table S2 Localisation analysis of proteins containing MHC class II ligands. The analysis was done using the Cytoscape plugin BINGO on proteins in the MHCII data set. The entire human protein set was used as background. GO-ID: The ID number of the GO cellular component term. p-value: p-value indicating the statistical significance of the difference between the fraction of proteins containing MHC class II ligands assigned to this GO term and the fraction of all proteins within the human protein set assigned to this GO term. corr p-value: The above p-value corrected for multiple testing by the Benjamini & Hochberg correction method. x: The number of MHC class II-containing proteins assigned to this GO term. nX: The number of proteins within the entire human protein set assigned to this GO term. N: The total number of MHC class II-containing proteins with assignments within the GO cellular component ontology. nN: The total number of proteins within the human protein set with assignments within the GO cellular component ontology. Description: Description of the GO term.
(0.07 MB XLS)

Table S3 mRNA expression levels of individual proteins. mRNA expression levels are taken from the GNF gene expression database. UniProtKB Acc: UniProtKB ACC number for the protein. Affy ID: Affymetrix ID for probes mapping to the protein. Expression value: Median expression value of all probes mapping to the protein. MHCI: A “1” in this column indicates that this protein is known to contain an MHC class I ligand, while “0” indicates that this protein is not known to contain an MHC class I ligand. MHCII: A “1” in this column indicates that this protein is known to contain an MHC class II ligand, while “0” indicates that this protein is not known to contain an MHC class II ligand.
(1.54 MB XLS)
