# Supplementary material for: Systematic Characterisation of Cellular Localisation and Expression Profiles of Proteins Containing MHC Ligands
Source: PLoS One. 2009 Oct 14;4(10):e7448. doi: 10.1371/journal.pone.0007448 (PMC2758592; doi:10.1371/journal.pone.0007448)
Supplement: Data set S1 — The MHCI data set. The amino acid sequences of the proteins in the MHCI data set in fasta format. The first column of the headings lists the UniProtKB Accession number and ID of the protein, the second column lists the starting position of the MHC class I ligand in the protein, the third column lists the amino acid sequence of the MHC class I ligand. (2.02 MB DOC) [file pone.0007448.s001.doc]

>sp|Q8NF91|SYNE1_HUMAN 4541 SVYDSVLQK

MATSRGASRCPRDIANVMQRLQDEQEIVQKRTFTKWINSHLAKRKPPMVVDDLFEDMKDG

VKLLALLEVLSGQKLPCEQGRRMKRIHAVANIGTALKFLEGRKIKLVNINSTDIADGRPS

IVLGLMWTIILYFQIEELTSNLPQLQSLSSSASSVDSIVSSETPSPPSKRKVTTKIQGNA

KKALLKWVQYTAGKQTGIEVKDFGKSWRSGVAFHSVIHAIRPELVDLETVKGRSNRENLE

DAFTIAETELGIPRLLDPEDVDVDKPDEKSIMTYVAQFLKHYPDIHNASTDGQEDDEILP

GFPSFANSVQNFKREDRVIFKEMKVWIEQFERDLTRAQMVESNLQDKYQSFKHFRVQYEM

KRKQIEHLIQPLHRDGKLSLDQALVKQSWDRVTSRLFDWHIQLDKSLPAPLGTIGAWLYR

AEVALREEITVQQVHEETANTIQRKLEQHKDLLQNTDAHKRAFHEIYRTRSVNGIPVPPD

QLEDMAERFHFVSSTSELHLMKMEFLELKYRLLSLLVLAESKLKSWIIKYGRRESVEQLL

QNYVSFIENSKFFEQYEVTYQILKQTAEMYVKADGSVEEAENVMKFMNETTAQWRNLSVE

VRSVRSMLEEVISNWDRYGNTVASLQAWLEDAEKMLNQSENAKKDFFRNLPHWIQQHTAM

NDAGNFLIETCDEMVSRDLKQQLLLLNGRWRELFMEVKQYAQADEMDRMKKEYTDCVVTL

SAFATEAHKKLSEPLEVSFMNVKLLIQDLEDIEQRVPVMDAQYKIITKTAHLITKESPQE

EGKEMFATMSKLKEQLTKVKECYSPLLYESQQLLIPLEELEKQMTSFYDSLGKINEIITV

LEREAQSSALFKQKHQELLACQENCKKTLTLIEKGSQSVQKFVTLSNVLKHFDQTRLQRQ

IADIHVAFQSMVKKTGDWKKHVETNSRLMKKFEESRAELEKVLRIAQEGLEEKGDPEELL

RRHTEFFSQLDQRVLNAFLKACDELTDILPEQEQQGLQEAVRKLHKQWKDLQGEAPYHLL

HLKIDVEKNRFLASVEECRTELDRETKLMPQEGSEKIIKEHRVFFSDKGPHHLCEKRLQL

IEELCVKLPVRDPVRDTPGTCHVTLKELRAAIDSTYRKLMEDPDKWKDYTSRFSEFSSWI

STNETQLKGIKGEAIDTANHGEVKRAVEEIRNGVTKRGETLSWLKSRLKVLTEVSSENEA

QKQGDELAKLSSSFKALVTLLSEVEKMLSNFGDCVQYKEIVKNSLEELISGSKEVQEQAE

KILDTENLFEAQQLLLHHQQKTKRISAKKRDVQQQIAQAQQGEGGLPDRGHEELRKLEST

LDGLERSRERQERRIQVTLRKWERFETNKETVVRYLFQTGSSHERFLSFSSLESLSSELE

QTKEFSKRTESIAVQAENLVKEASEIPLGPQNKQLLQQQAKSIKEQVKKLEDTLEEDIKT

MEMVKTKWDHFGSNFETLSVWITEKEKELNALETSSSAMDMQISQIKVTIQEIESKLSSI

VGLEEEAQSFAQFVTTGESARIKAKLTQIRRYGEELREHAQCLEGTILGHLSQQQKFEEN

LRKIQQSVSEFEDKLAVPIKICSSATETYKVLQEHMDLCQALESLSSAITAFSASARKVV

NRDSCVQEAAALQQQYEDILRRAKERQTALENLLAHWQRLEKELSSFLTWLERGEAKASS

PEMDISADRVKVEGELQLIQALQNEVVSQASFYSKLLQLKESLFSVASKDDVKMMKLHLE

QLDERWRDLPQIINKRINFLQSVVAEHQQFDELLLSFSVWIKLFLSELQTTSEISIMDHQ

VALTRHKDHAAEVESKKGELQSLQGHLAKLGSLGRAEDLHLLQGKAEDCFQLFEEASQVV

ERRQLALSHLAEFLQSHASLSGILRQLRQTVEATNSMNKNESDLIEKDLNDALQNAKALE

SAAVSLDGILSKAQYHLKIGSSEQRTSCRATADQLCGEVERIQNLLGTKQSEADALAVLK

KAFQDQKEELLKSIEDIEERTDKERLKEPTRQALQQRLRVFNQLEDELNSHEHELCWLKD

KAKQIAQKDVAFAPEVDREINRLEVTWDDTKRLIHENQGQCCGLIDLMREYQNLKSAVSK

VLENASSVIVTRTTIKDQEDLKWAFSKHETAKNKMNYKQKDLDNFTSKGKHLLSELKKIH

SSDFSLVKTDMESTVDKWLDVSEKLEENMDRLRVSLSIWDDVLSTRDEIEGWSNNCVPQM

AENISNLDNHLRAEELLKEFESEVKNKALRLEELHSKVNDLKELTKNLETPPDLQFIEAD

LMQKLEHAKEITEVAKGTLKDFTAQSTQVEKFINDITTWFTKVEESLMNCAQNETCEALK

KVKDIQKELQSQQSNISSTQENLNSLCRKYHSAELESLGRAMTGLIKKHEAVSQLCSKTQ

ASLQESLEKHFSESMQEFQEWFLGAKAAAKESSDRTGDSKVLEAKLHDLQNILDSVSDGQ

SKLDAVTQEGQTLYAHLSKQIVSSIQEQITKANEEFQAFLKQCLKDKQALQDCASELGSF

EDQHRKLNLWIHEMEERFNTENLGESKQHIPEKKNEVHKVEMFLGELLAARESLDKLSQR

GQLLSEEGHGAGQEGRLCSQLLTSHQNLLRMTKEKLRSCQVALQEHEALEEALQSMWFWV

KAIQDRLACAESTLGSKDTLEKRLSQIQDILLMKGEGEVKLNMAIGKGEQALRSSNKEGQ

RVIQTQLETLKEVWADIMSSSVHAQSTLESVISQWNDYVERKNQLEQWMESVDQKIEHPL

QPQPGLKEKFVLLDHLQSILSEAEDHTRALHRLIAKSRELYEKTEDESFKDTAQEELKTQ

FNDIMTVAKEKMRKVEEIVKDHLMYLDAVHEFTDWLHSAKEELHRWSDMSGDSSATQKKL

SKIKELIDSREIGASRLSRVESLAPEVKQNTTASGCELMHTEMQALRADWKQWEDSVFQT

QSCLENLVSQMALSEQEFSGQVAQLEQALEQFSALLKTWAQQLTLLEGKNTDEEIVECWH

KGQEILDALQKAEPRTEDLKSQLNELCRFSRDLSTYSGKVSGLIKEYNCLCLQASKGCQN

KEQILQQRFRKAFRDFQQWLVNAKITTAKCFDIPQNISEVSTSLQKIQEFLSESENGQHK

LNMMLSKGELLSTLLTKEKAKGIQAKVTAAKEDWKNFHSNLHQKESALENLKIQMKDFEV

SAEPIQDWLSKTEKMVHESSNRLYDLPAKRREQQKLQSVLEEIHCYEPQLNRLKEKAQQL

WEGQAASKSFRHRVSQLSSQYLALSNLTKEKVSRLDRIVAEHNQFSLGIKELQDWMTDAI

HMLDSYCHPTSDKSVLDSRTLKLEALLSVKQEKEIQMKMIVTRGESVLQNTSPEGIPTIQ

QQLQSVKDMWASLLSAGIRCKSQLEGALSKWTSYQDGVRQFSGWMDSMEANLNESERQHA

ELRDKTTMLGKAKLLNEEVLSYSSLLETIEVKGAGMTEHYVTQLELQDLQERYRAIQERA

KEAVTKSEKLVRLHQEYQRDLKAFEVWLGQEQEKLDQYSVLEGDAHTHETTLRDLQELQV

HCAEGQALLNSVLHTREDVIPSGIPQAEDRALESLRQDWQAYQHRLSETRTQFNNVVNKL

RLMEQKFQQVDEWLKTAEEKVSPRTRRQSNRATKEIQLHQMKKWHEEVTAYRDEVEEVGA

RAQEILDESHVNSRMGCQATQLTSRYQALLLQVLEQIKFLEEEIQSLEESESSLSSYSDW

YGSTHKNFKNVATKIDKVDTVMMGKKLKTLEVLLKDMEKGHSLLKSAREKGERAVKYLEE

GEAERLRKEIHDHMEQLKELTSTVRKEHMTLEKGLHLAKEFSDKCKALTQWIAEYQEILH

VPEEPKMELYEKKAQLSKYKSLQQTVLSHEPSVKSVREKGEALLELVQDVTLKDKIDQLQ

SDYQDLCSIGKEHVFSLEAKVKDHEDYNSELQEVEKWLLQMSGRLVAPDLLETSSLETIT

QQLAHHKAMMEEIAGFEDRLNNLQMKGDTLIGQCADHLQAKLKQNVHAHLQGTKDSYSAI

CSTAQRMYQSLEHELQKHVSRQDTLQQCQAWLSAVQPDLEPSPQPPLSRAEAIKQVKHFR

ALQEQARTYLDLLCSMCDLSNASVKTTAKDIQQTEQTIEQKLVQAQNLTQGWEEIKHLKS

ELWIYLQDADQQLQNMKRRHSELELNIAQNMVSQVKDFVKKLQSKQASVNTIIEKVNKLT

KKEESPEHKEINHLNDQWLDLCRQSNNLCLQREEDLQRTRDYHDCMNVVEVFLEKFTTEW

DNLARSDAESTAVHLEALKKLALALQERKYAIEDLKDQKQKMIEHLNLDDKELVKEQTSH

LEQRWFQLEDLIKRKIQVSVTNLEELNVVQSRFQELMEWAEEQQPNIAEALKQSPPPDMA

QNLLMDHLAICSELEAKQMLLKSLIKDADRVMADLGLNERQVIQKALSDAQSHVNCLSDL

VGQRRKYLNKALSEKTQFLMAVFQATSQIQQHERKIMFREHICLLPDDVSKQVKTCKSAQ

ASLKTYQNEVTGLWAQGRELMKEVTEQEKSEVLGKLQELQSVYDSVLQKCSHRLQELEKN

LVSRKHFKEDFDKACHWLKQADIVTFPEINLMNESSELHTQLAKYQNILEQSPEYENLLL

TLQRTGQTILPSLNEVDHSYLSEKLNALPRQFNVIVALAKDKFYKVQEAILARKEYASLI

ELTTQSLSELEAQFLRMSKVPTDLAVEEALSLQDGCRAILDEVAGLGEAVDELNQKKEGF

RSTGQPWQPDKMLHLVTLYHRLKRQTEQRVSLLEDTTSAYQEHEKMCQQLERQLKSVKEE

QSKVNEETLPAEEKLKMYHSLAGSLQDSGIVLKRVTIHLEDLAPHLDPLAYEKARHQIQS

WQGELKLLTSAIGETVTECESRMVQSIDFQTEMSRSLDWLRRVKAELSGPVYLDLNLQDI

QEEIRKIQIHQEEVQSSLRIMNALSHKEKEKFTKAKELISADLEHSLAELSELDGDIQEA

LRTRQATLTEIYSQCQRYYQVFQAANDWLEDAQELLQLAGNGLDVESAEENLKSHMEFFS

TEDQFHSNLEELHSLVATLDPLIKPTGKEDLEQKVASLELRSQRMSRDSGAQVDLLQRCT

AQWHDYQKAREEVIELMNDTEKKLSEFSLLKTSSSHEAEEKLSEHKALVSVVNSFHEKIV

ALEEKASQLEKTGNDASKATLSRSMTTVWQRWTRLRAVAQDQEKILEDAVDEWTGFNNKV

KKATEMIDQLQDKLPGSSAEKASKAELLTLLEYHDTFVLELEQQQSALGMLRQQTLSMLQ

DGAAPTPGEEPPLMQEITAMQDRCLNMQEKVKTNGKLVKQELKDREMVETQINSVKCWVQ

ETKEYLGNPTIEIDAQLEELQILLTEATNHRQNIEKMAEEQKEKYLGLYTILPSELSLQL

AEVALDLKIRDQIQDKIKEVEQSKATSQELSRQIQKLAKDLTTILTKLKAKTDNVVQAKT

DQKVLGEELDGCNSKLMELDAAVQKFLEQNGQLGKPLAKKIGKLTELHQQTIRQAENRLS

KLNQAASHLEEYNEMLELILKWIEKAKVLAHGTIAWNSASQLREQYILHQTLLEESKEID

SELEAMTEKLQYLTSVYCTEKMSQQVAELGRETEELRQMIKIRLQNLQDAAKDMKKFEAE

LKKLQAALEQAQATLTSPEVGRLSLKEQLSHRQHLLSEMESLKPKVQAVQLCQSALRIPE

DVVASLPLCHAALRLQEEASRLQHTAIQQCNIMQAAVVQYEQYEQEMKHLQQLIEGAHRE

IEDKPVATSNIQELQAQISRHEELAQKIKGYQEQIASLNSKCKMLTMKAKHATMLLTVTE

VEGLAEGTEDLDGELLPTPSAHPSVVMMTAGRCHTLLSPVTEESGEEGTNSEISSPPACR

SPSPVANTDASVNQDIAYYQALSAERLQTDAAKIHPSTSASQEFYEPGLEPSATAKLGDL

QRSWETLKNVISEKQRTLYEALERQQKYQDSLQSISTKMEAIELKLSESPEPGRSPESQM

AEHQALMDEILMLQDEINELQSSLAEELVSESCEADPAEQLALQSTLTVLAERMSTIRMK

ASGKRQLLEEKLNDQLEEQRQEQALQRYRCEADELDSWLLSTKATLDTALSPPKEPMDME

AQLMDCQNMLVEIEQKVVALSELSVHNENLLLEGKAHTKDEAEQLAGKLRRLKGSLLELQ

RALHDKQLNMQGTAQEKEESDVDLTATQSPGVQEWLAQARTTWTQQRQSSLQQQKELEQE

LAEQKSLLRSVASRGEEILIQHSAAETSGDAGEKPDVLSQELGMEGEKSSAEDQMRMKWE

SLHQEFSTKQKLLQNVLEQEQEQVLYSRPNRLLSGVPLYKGDVPTQDKSAVTSLLDGLNQ

AFEEVSSQSGGAKRQSIHLEQKLYDGVSATSTWLDDVEERLFVATALLPEETETCLFNQE

ILAKDIKEMSEEMDKNKNLFSQAFPENGDNRDVIEDTLGCLLGRLSLLDSVVNQRCHQMK

ERLQQILNFQNDLKVLFTSLADNKYIILQKLANVFEQPVAEQIEAIQQAEDGLKEFDAGI

IELKRRGDKLQVEQPSMQELSKLQDMYDELMMIIGSRRSGLNQNLTLKSQYERALQDLAD

LLETGQEKMAGDQKIIVSSKEEIQQLLDKHKEYFQGLESHMILTETLFRKIISFAVQKET

QFHTELMAQASAVLKRAHKRGVELEYILETWSHLDEDQQELSRQLEVVESSIPSVGLVEE

NEDRLIDRITLYQHLKSSLNEYQPKLYQVLDDGKRLLISISCSDLESQLNQLGECWLSNT

NKMSKELHRLETILKHWTRYQSESADLIHWLQSAKDRLEFWTQQSVTVPQELEMVRDHLN

AFLEFSKEVDAQSSLKSSVLSTGNQLLRLKKVDTATLRSELSRIDSQWTDLLTNIPAVQE

KLHQLQMDKLPSRHAISEVMSWISLMENVIQKDEDNIKNSIGYKAIHEYLQKYKGFKIDI

NCKQLTVDFVNQSVLQISSQDVESKRSDKTDFAEQLGAMNKSWQILQGLVTEKIQLLEGL

LESWSEYENNVQCLKTWFETQEKRLKQQHRIGDQASVQNALKDCQDLEDLIKAKEKEVEK

IEQNGLALIQNKKEDVSSIVMSTLRELGQTWANLDHMVGQLKILLKSVLDQWSSHKVAFD

KINSYLMEARYSLSRFRLLTGSLEAVQVQVDNLQNLQDDLEKQERSLQKFGSITNQLLKE

CHPPVTETLTNTLKEVNMRWNNLLEEIAEQLQSSKALLQLWQRYKDYSKQCASTVQQQED

RTNELLKAATNKDIADDEVATWIQDCNDLLKGLGTVKDSLFFLHELGEQLKQQVDASAAS

AIQSDQLSLSQHLCALEQALCKQQTSLQAGVLDYETFAKSLEALEAWIVEAEEILQGQDP

SHSSDLSTIQERMEELKGQMLKFSSMAPDLDRLNELGYRLPLNDKEIKRMQNLNRHWSLI

SSQTTERFSKLQSFLLQHQTFLEKCETWMEFLVQTEQKLAVEISGNYQHLLEQQRAHELF

QAEMFSRQQILHSIIIDGQRLLEQGQVDDRDEFNLKLTLLSNQWQGVIRRAQQRRGIIDS

QIRQWQRYREMAEKLRKWLVEVSYLPMSGLGSVPIPLQQARTLFDEVQFKEKVFLRQQGS

YILTVEAGKQLLLSADSGAEAALQAELAEIQEKWKSASMRLEEQKKKLAFLLKDWEKCEK

GIADSLEKLRTFKKKLSQSLPDHHEELHAEQMRCKELENAVGSWTDDLTQLSLLKDTLSA

YISADDISILNERVELLQRQWEELCHQLSLRRQQIGERLNEWAVFSEKNKELCEWLTQME

SKVSQNGDILIEEMIEKLKKDYQEEIAIAQENKIQLQQMGERLAKASHESKASEIEYKLG

KVNDRWQHLLDLIAARVKKLKETLVAVQQLDKNMSSLRTWLAHIESELAKPIVYDSCNSE

EIQRKLNEQQELQRDIEKHSTGVASVLNLCEVLLHDCDACATDAECDSIQQATRNLDRRW

RNICAMSMERRLKIEETWRLWQKFLDDYSRFEDWLKSSERTAAFPSSSGVIYTVAKEELK

KFEAFQRQVHECLTQLELINKQYRRLARENRTDSACSLKQMVHEGNQRWDNLQKRVTSIL

RRLKHFIGQREEFETARDSILVWLTEMDLQLTNIEHFSECDVQAKIKQLKAFQQEISLNH

NKIEQIIAQGEQLIEKSEPLDAAIIEEELDELRRYCQEVFGRVERYHKKLIRLPLPDDEH

DLSDRELELEDSAALSDLHWHDRSADSLLSPQPSSNLSLSLAQPLRSERSGRDTPASVDS

IPLEWDHDYDLSRDLESAMSRALPSEDEEGQDDKDFYLRGAVALSGDHSALESQIRQLGK

ALDDSRFQIQQTENIIRSKTPTGPELDTSYKGYMKLLGECSSSIDSVKRLEHKLKEEEES

LPGFVNLHSTETQTAGVIDRWELLQAQALSKELRMKQNLQKWQQFNSDLNSIWAWLGDTE

EELEQLQRLELSTDIQTIELQIKKLKELQKAVDHRKAIILSINLCSPEFTQADSKESRDL

QDRLSQMNGRWDRVCSLLEEWRGLLQDALMQCQGFHEMSHGLLLMLENIDRRKNEIVPID

SNLDAEILQDHHKQLMQIKHELLESQLRVASLQDMSCQLLVNAEGTDCLEAKEKVHVIGN

RLKLLLKEVSRHIKELEKLLDVSSSQQDLSSWSSADELDTSGSVSPTSGRSTPNRQKTPR

GKCSLSQPGPSVSSPHSRSTKGGSDSSLSEPGPGRSGRGFLFRVLRAALPLQLLLLLLIG

LACLVPMSEEDYSCALSNNFARSFHPMLRYTNGPPPL

>sp|P20929|NEBU_HUMAN 6493 ETITGLRVW

MADDEDYEEVVEYYTEEVVYEEVPGETITKIYETTTTRTSDYEQSETSKPALAQPALAQP

ASAKPVERRKVIRKKVDPSKFMTPYIAHSQKMQDLFSPNKYKEKFEKTKGQPYASTTDTP

ELRRIKKVQDQLSEVKYRMDGDVAKTICHVDEKAKDIEHAKKVSQQVSKVLYKQNWEDTK

DKYLLPPDAPELVQAVKNTAMFSKKLYTEDWEADKSLFYPYNDSPELRRVAQAQKALSDV

AYKKGLAEQQAQFTPLADPPDIEFAKKVTNQVSKQKYKEDYENKIKGKWSETPCFEVANA

RMNADNISTRKYQEDFENMKDQIYFMQTETPEYKMNKKAGVAASKVKYKEDYEKNKGKAD

YNVLPASENPQLRQLKAAGDALSDKLYKENYEKTKAKSINYCETPKFKLDTVLQNFSSDK

KYKDSYLKDILGHYVGSFEDPYHSHCMKVTAQNSDKNYKAEYEEDRGKGFFPQTITQEYE

AIKKLDQCKDHTYKVHPDKTKFTQVTDSPVLLQAQVNSKQLSDLNYKAKHESEKFKCHIP

PDTPAFIQHKVNAYNLSDNLYKQDWEKSKAKKFDIKVDAIPLLAAKANTKNTSDVMYKKD

YEKNKGKMIGVLSINDDPKMLHSLKVAKNQSDRLYKENYEKTKAKSMNYCETPKYQLDTQ

LKNFSEARYKDLYVKDVLGHYVGSMEDPYHTHCMKVAAQNSDKSYKAEYEEDKGKCYFPQ

TITQEYDAIKKLDQCKDHTYKVHPDKTKFTAVTDSPVLLQAQLNTKQLSDLNYKAKHEGE

RFKCHIPADAPQFIQHRVNAYNLSDNVYKQDWEKSKAKKFDIKVDAIPLLAAKANTKNTS

DVMYKKDYEKSKGKMIGALSINDDPKMLHSLKTAKNQSDREYRKDYEKSKTIYTAPLDML

QVTQAKKSQAIASDVDYKHILHSYSYPPDSINVDLAKKAYALQSDVEYKADYNSWMKGCG

WVPFGSLEMEKAKRASDILNEKKYRQHPDTLKFTSIEDAPITVQSKINQAQRSDIAYKAK

GEEIIHNYNLPPDLPQFIQAKVNAYNISENMYKADLKDLSKKGYDLRTDAIPIRAAKAAR

QAASDVQYKKDYEKAKGKMVGFQSLQDDPKLVHYMNVAKIQSDREYKKDYEKTKSKYNTP

HDMFNVVAAKKAQDVVSNVNYKHSLHHYTYLPDAMDLELSKNMMQIQSDNVYKEDYNNWM

KGIGWIPIGSLDVEKVKKAGDALNEKKYRQHPDTLKFTSIVDSPVMVQAKQNTKQVSDIL

YKAKGEDVKHKYTMSPDLPQFLQAKCNAYSISDVCYKRDWHDLIRKGNNVLGDAIPITAA

KASRNIASDYKYKEAYEKSKGKHVGFRSLQDDPKLVHYMNVAKLQSDREYKKNYENTKTS

YHTPGDMVTITAAKMAQDVATNVNYKQPLHHYTYLPDAMSLEHTRNVNQIQSDNVYKDEY

NSFLKGIGWIPIGSLEVEKVKKAGDALNERKYRQHPDTVKFTSVPDSMGMMLAQHNTKQL

SDLNYKVEGEKLKHKYTIDPELPQFIQAKVNALNMSDAHYKADWKKTIRKGYDLRPDAIP

IVAAKSSRNIASDCKYKEAYEKAKGKQVGFLSLQDDPKLVHYMNVAKIQSDREYKKGYEA

SKTKYHTPLDMVSVTAAKKSQEVATNANYRQSYHHYTLLPDALNVEHSRNAMQIQSDNLY

KSDFTNWMKGIGWVPIESLEVEKAKKAGEILSEKKYRQHPEKLKFTYAMDTMEQALNKSN

KLNMDKRLYTEKWNKDKTTIHVMPDTPDILLSRVNQITMSDKLYKAGWEEEKKKGYDLRP

DAIAIKAARASRDIASDYKYKKAYEQAKGKHIGFRSLEDDPKLVHFMQVAKMQSDREYKK

GYEKSKTSFHTPVDMLSVVAAKKSQEVATNANYRNVIHTYNMLPDAMSFELAKNMMQIQS

DNQYKADYADFMKGIGWLPLGSLEAEKNKKAMEIISEKKYRQHPDTLKYSTLMDSMNMVL

AQNNAKIMNEHLYKQAWEADKTKVHIMPDIPQIILAKANAINISDKLYKLSLEESKKKGY

DLRPDAIPIKAAKASRDIASDYKYKYNYEKGKGKMVGFRSLEDDPKLVHSMQVAKMQSDR

EYKKNYENTKTSYHTPADMLSVTAAKDAQANITNTNYKHLIHKYILLPDAMNIELTRNMN

RIQSDNEYKQDYNEWYKGLGWSPAGSLEVEKAKKATEYASDQKYRQHPSNFQFKKLTDSM

DMVLAKQNAHTMNKHLYTIDWNKDKTKIHVMPDTPDILQAKQNQTLYSQKLYKLGWEEAL

KKGYDLPVDAISVQLAKASRDIASDYKYKQGYRKQLGHHVGFRSLQDDPKLVLSMNVAKM

QSEREYKKDFEKWKTKFSSPVDMLGVVLAKKCQELVSDVDYKNYLHQWTCLPDQNDVVQA

KKVYELQSENLYKSDLEWLRGIGWSPLGSLEAEKNKRASEIISEKKYRQPPDRNKFTSIP

DAMDIVLAKTNAKNRSDRLYREAWDKDKTQIHIMPDTPDIVLAKANLINTSDKLYRMGYE

ELKRKGYDLPVDAIPIKAAKASREIASEYKYKEGFRKQLGHHIGARNIEDDPKMMWSMHV

AKIQSDREYKKDFEKWKTKFSSPVDMLGVVLAYKCQTLVSDVDYKNYLHQWTCLPDQSDV

IHARQAYDLQSDNLYKSDLQWLKGIGWMTSGSLEDEKNKRATQILSDHVYRQHPDQFKFS

SLMDSIPMVLAKNNAITMNHRLYTEAWDKDKTTVHIMPDTPEVLLAKQNKVNYSEKLYKL

GLEEAKRKGYDMRVDAIPIKAAKASRDIASEFKYKEGYRKQLGHHIGARAIRDDPKMMWS

MHVAKIQSDREYKKDFEKWKTKFSSPVDMLGVVLAKKCQTLVSDVDYKNYLHQWTCLPDQ

SDVIHARQAYDLQSDNMYKSDLQWMRGIGWVSIGSLDVEKCKRATEILSDKIYRQPPDRF

KFTSVTDSLEQVLAKNNALNMNKRLYTEAWDKDKTQIHIMPDTPEIMLARQNKINYSETL

YKLANEEAKKKGYDLRSDAIPIVAAKASRDVISDYKYKDGYRKQLGHHIGARNIEDDPKM

MWSMHVAKIQSDREYKKDFEKWKTKFSSPVDMLGVVLAKKCQTLVSDVDYKNYLHEWTCL

PDQNDVIHARQAYDLQSDNIYKSDLQWLRGIGWVPIGSMDVVKCKRAAEILSDNIYRQPP

DKLKFTSVTDSLEQVLAKNNALNMNKRLYTEAWDKDKTQVHIMPDTPEIMLARQNKINYS

ESLYRQAMEEAKKEGYDLRSDAIPIVAAKASRDIASDYKYKEAYRKQLGHHIGARAVHDD

PKIMWSLHIAKVQSDREYKKDFEKYKTRYSSPVDMLGIVLAKKCQTLVSDVDYKHPLHEC

ICLPDQNDIIHARKAYDLQSDNLYKSDLEWMKGIGWVPIDSLEVVRAKRAGELLSDTIYR

QRPETLKFTSITDTPEQVLAKNNALNMNKRLYTEAWDNDKKTIHVMPDTPEIMLAKLNRI

NYSDKLYKLALEESKKEGYDLRLDAIPIQAAKASRDIASDYKYKEGYRKQLGHHIGARNI

KDDPKMMWSIHVAKIQSDREYKKEFEKWKTKFSSPVDMLGVVLAKKCQILVSDIDYKHPL

HEWTCLPDQNDVIQARKAYDLQSDAIYKSDLEWLRGIGWVPIGSVEVEKVKRAGEILSDR

KYRQPADQLKFTCITDTPEIVLAKNNALTMSKHLYTEAWDADKTSIHVMPDTPDILLAKS

NSANISQKLYTKGWDESKMKDYDLRADAISIKSAKASRDIASDYKYKEAYEKQKGHHIGA

QSIEDDPKIMCAIHAEKIQSEREYKKEFQKWKTKFSSPVDMLSILLAKKCQTLVTDIYYR

NYLHEWTCMPDQNDIIQAKKAYDLQSDALYKADLEWLRGIGWMPQGSPEVLRVKNAQNIF

CDSVYRTPVVNLKYTSIVDTPEVVLAKSNAENISIPKYREVWDKDKTSIHIMPDTPEINL

ARANALNVSNKLYREGWDEMKAGCDVRLDAIPIQAAKASREIASDYKYKLDHEKQKGHYV

GTLTARDDNKIRWALIADKLQNEREYRLDWAKWKAKIQSPVDMLSILHSKNSQALVSDMD

YRNYLHQWTCMPDQNDVIQAKKAYELQSDNVYKADLEWLRGIGWMPNDSVSVNHAKHAAD

IFSEKKYRTKIETLNFTPVDDRVDYVTAKQSGEILDDIKYRKDWNATKSKYTLTETPLLH

TAQEAARILDQYLYKEGWERQKATGYILPPDAVPFVHAHHCNDVQSELKYKAEHVKQKGH

YVGVPTMRDDPKLVWFEHAGQIQNERLYKEDYHKTKAKINIPADMVSVLAAKQGQTLVSD

IDYRNYLHQWMCHPDQNDVIQARKAYDLQSDNVYRADLEWLRGIGWIPLDSVDHVRVTKN

QEMMSQIKYKKNALENYPNFTSVVDPPEIVLAKINSVNQSDVKYKETFNKAKGKYTFSPD

TPHISHSKDMGKLYSTILYKGAWEGTKAYGYTLDERYIPIVGAKHADLVNSELKYKETYE

KQKGHYLAGKVIGEFPGVVHCLDFQKMRSALNYRKHYEDTKANVHIPNDMMNHVLAKRCQ

YILSDLEYRHYFHQWTSLLEEPNVIRVRNAQEILSDNVYKDDLNWLKGIGCYVWDTPQIL

HAKKSYDLQSQLQYTAAGKENLQNYNLVTDTPLYVTAVQSGINASEVKYKENYHQIKDKY

TTVLETVDYDRTRNLKNLYSSNLYKEAWDRVKATSYILPSSTLSLTHAKNQKHLASHIKY

REEYEKFKALYTLPRSVDDDPNTARCLRVGKLNIDRLYRSVYEKNKMKIHIVPDMVEMVT

AKDSQKKVSEIDYRLRLHEWICHPDLQVNDHVRKVTDQISDIVYKDDLNWLKGIGCYVWD

TPEILHAKHAYDLRDDIKYKAHMLKTRNDYKLVTDTPVYVQAVKSGKQLSDAVYHYDYVH

SVRGKVAPTTKTVDLDRALHAYKLQSSNLYKTSLRTLPTGYRLPGDTPHFKHIKDTRYMS

SYFKYKEAYEHTKAYGYTLGPKDVPFVHVRRVNNVTSERLYRELYHKLKDKIHTTPDPPE

IRQVKKTQEAVSELIYKSDFFKMQGHMISLPYTPQVIHCRYVGDITSDIKYKEDLQVLKG

FGCFLYDTPDMVRSRHLRKLWSNYLYTDKAREMRDKYKVVLDTPEYRKVQELKTHLSELV

YRAAGKKQKSIFTSVPDTPDLLRAKRGQKLQSQYLYVELATKERPHHHAGNQTTALKHAK

DVKDMVSEKKYKIQYEKMKDKYTPVPDTPILIRAKRAYWNASDLRYKETFQKTKGKYHTV

KDALDIVYHRKVTDDISKIKYKENYMSQLGIWRSIPDRPEHFHHRAVTDTVSDVKYKEDL

TWLKGIGCYAYDTPDFTLAEKNKTLYSKYKYKEVFERTKSDFKYVADSPINRHFKYATQL

MNEKKYRADYEQRKDKYHLVVDEPRHLLAKTRSDQISQIKYRKNYEKSKDKFTSIVDTPE

HLRTTKVNKQISDILYKLEYNKAKPRGYTTIHDTPMLLHVRKVKDEVSDLKYKEVYQRNK

SNCTIEPDAVHIKAAKDAYKVNTNLDYKKQYEANKAHWKWTPDRPDFLQAAKSSLQQSDF

EYKLDREFLKGCKLSVTDDKNTVLALRNTLIESDLKYKEKHVKERGTCHAVPDTPQILLA

KTVSNLVSENKYKDHVKKHLAQGSYTTLPETRDTVHVKEVTKHVSDTNYKKKFVKEKGKS

NYSIMLEPPEVKHAMEVAKKQSDVAYRKDAKENLHYTTVADRPDIKKATQAAKQASEVEY

RAKHRKEGSHGLSMLGRPDIEMAKKAAKLSSQVKYRENFDKEKGKTPKYNPKDSQLYKVM

KDANNLASEVKYKADLKKLHKPVTDMKESLIMNHVLNTSQLASSYQYKKKYEKSKGHYHT

IPDNLEQLHLKEATELQSIVKYKEKYEKERGKPMLDFETPTYITAKESQQMQSGKEYRKD

YEESIKGRNLTGLEVTPALLHVKYATKIASEKEYRKDLEESIRGKGLTEMEDTPDMLRAK

NATQILNEKEYKRDLELEVKGRGLNAMANETPDFMRARNATDIASQIKYKQSAEMEKANF

TSVVDTPEIIHAQQVKNLSSQKKYKEDAEKSMSYYETVLDTPEIQRVRENQKNFSLLQYQ

CDLKNSKGKITVVQDTPEILRVKENQKNFSSVLYKEDVSPGTAIGKTPEMMRVKQTQDHI

SSVKYKEAIGQGTPIPDLPEVKRVKETQKHISSVMYKENLGTGIPTTVTPEIERVKRNQE

NFSSVLYKENLGKGIPTPITPEMERVKRNQENFSSVLYKENMGKGTPLPVTPEMERVKHN

QENISSVLYKENVGKATATPVTPEMQRVKRNQENISSVLYKENLGKATPTPFTPEMERVK

RNQENFSSVLYKENMRKATPTPVTPEMERAKRNQENISSVLYSDSFRKQIQGKAAYVLDT

PEMRRVRETQRHISTVKYHEDFEKHKGCFTPVVTDPITERVKKNMQDFSDINYRGIQRKV

VEMEQKRNDQDQETITGLRVWRTNPGSVFDYDPAEDNIQSRSLHMINVQAQRRSREQSRS

ASALSVSGGEEKSEHSEAPDHHLSTYSDGGVFAVSTAYKHAKTTELPQQRSSSVATQQTT

VSSIPSHPSTAGKIFRAMYDYMAADADEVSFKDGDAIINVQAIDEGWMYGTVQRTGRTGM

LPANYVEAI

>sp|Q96PK2|MACF4_HUMAN 2405 KDKLKDATERY

MQKGLLDQDTGLVLLESQVIMSGLIAPETGENLSLEEGIARNLINPQMYQQLRELQDALA

LISRLTESRGPLSVVEAIEKRIISETVGLKILEAHLATGGFSLSPSENCINLEEAFHQGL

ISAWLHSVLESYLRTSKNLIDPNTAEKIGLLDLMQRCIVHQESGFKLLPVKQLAGGMVSL

KSGRKVSIFRAVQEGLIDRQVTVRLLEAQLFAGGIVDPRTGHRLTVEEAVRHNLIDQDMA

CAILIRQLQTGGIIDTVTGQRLTIDEAVSNDLVAAKIALVILESLWSFMGLLWPESGEIL

PITDALEQGIVSTELAHKILSNRQHIKALFLPATTEILSWKKAIESGILDRDLANNLKSI

CIPDVMPHMQLADSAEQNINPGAAVLPCSKSHPKATASQSENLLFQLMTHSYINVQNGQR

LLLLDKELMETLTSRDEYQTSPPKVVEIGHQRQKTPEGLQESANVKISGTFSSGWTVRLP

EFQFSSQNKEYPDREDCTTEKGKKTTVETEDSSVENPEQDLFVEQKERNPNIDALKVINK

VKLEVQRQLIGTQREDQTAVSVRENASRGHLLTIPPAEAEGVPLVVDKDVFSVETPKKEH

QPLRNTSFTCQNEQAHTLETEYIHDETGGSHIKPQSKKLQVQVKKTLGIKLELKSETDGN

VHPLDKKEMLKKTFLAKDDHKESQEAQNIAGGSMMMSEKTDEEDSGREIFLSCSHPLELL

EEATLNVLSAQLLDGGIFHEQTGQKLLLNEAISRGIVPSHTAVKLMEKLNMFQGFFDSQT

CESLTTEEVINEGLMDEKLLHNVLMADKAISGVLDPRTQTLCSVKDAVTVGLLDKETATR

ILERQVVTGGIIDLKRGKKVSVTLASTLGLVDVADQPELINLEKASKGRDAEKTVRERLI

SLQMETTGLIDPDSKAPLTVVQSIDRGLLEREEAVRLLTKQVVDGGIIHHISGMRLSVDN

AFRHGLIGEDLAEKLKRVENLNIHQIFNPETKENISLPKAIKLDLITSDLKREIQEVQAF

TGNFVDLISGQRLTLAEAKKEGLLTNEAVLSPGMMHGIVDPENCRIVPYSELVKKCKIDI

ESGQRYLEVIPFSDIKDGVSDKVLTLSQAIQLGKVDFASTLKVLEAQANTGGIIDTATGK

RLTLASALEEKLVDENMVRIIASHQVLNGGIVDIFSDQRVTLVEAIEKRLISPELANMIQ

IDSSEFSDHRAQIEKQEGIEVCALQNEFLGKDMLIACNQTAEMSCNKVEESERLFQVENQ

SAQEKVKVRVSDGEQAKKSREISLKEFGCKDQRKPRMSSDAKEFISIINPHNLKGKSLGQ

VSLTHPYSECDFKLKEVARNNMGNDTNEEQEKAVTKIEIISHMKQSTSCLDSEEIRENQG

EVILEVQETYCETSGKLPSEQVLQQPMNARVKSKREKREVIVEESIRTCKPAFLSEEKLY

QETAIRDEHDSHIKSQPREMTSSEKGKEADTEMGFSITFKIEESSSQVVPQGISVKHLDA

LTLFSSKQANEGKVNNLSLCLTLKPEENLSREIACGAQSEPFPCMTPRPEGLHYQESDGK

AQVTGPSQISKTDKSFQGTTRQETNYQDSWVTSKTKETKHQISSSNECKEKSYQEVSFDP

ARGLKLEEITVSRPDSKEVRYLEFSDRKDLHHQGSKSDDKLCGTLKSEIATQELTGEKFL

EMANPNVAGLEAGSIEDIVTQRGSRVLGSFLPEKLFKGVSQKENTGQQNAIISPTVLETS

EEKTVSLTVCSAVKTEKTPQEKLRESPGSEQTPFMTAPEGKGNGGVNPEPFRATQNVFTR

QLCLEHDEKLVSYLSLLRNIEMRTKQIQPLELNLAELQDLLCQAKVLERELKDLTTLVSQ

ELECVNQIIISQPQEVPAQLLKALEKDAKNLQKSLSSVSDTWNSRLLHFQNAVEIEKTKV

LNQHTQLEGRLQDLRAWVGNKNLILNSKGSNSEIDVDSLNLCLQQYEDLKQPMAERKAQL

DALAFDIQFFISEHAQDLSPQQNRQMLRLLNELQRSFQDILEQTAAQVDALQGHLQQMEQ

EALVKTLQKQQNTCHQQLEDLCSWVGQAERALAGHQGRTTQQDLSALQKNQSDLKDLQDD

IQNRATSFATVVKDIEGFMEENQTKLSPRELTALREKLHQAKEQYEALQEETRVAQKELE

EAVTSALQQETEKSKAAKELAENKKKIDALLDWVTSVGSSGGQLLTNLPGMEQLSGASLE

KGALDTTDGYMGVNQAPEKLDKQCEMMKARHQELLSQQQNFILATQSAQAFLDQHGHNLT

PEEQQMLQQKLGELKEQYSTSLAQSEAELKQVQTLQDELQKFLQDHKEFESWLERSEKEL

ENMHKGGSSPETLPSLLKRQGSFSEDVISHKGDLRFVTISGQKVLDMENSFKEGKEPSEI

GNLVKDKLKDATERYTALHSKCTRLGSHLNMLLGQYHQFQNSADSLQAWMQACEANVEKL

LSDTVASDPGVLQEQLATTKQLQEELAEHQVPVEKLQKVARDIMEIEGEPAPDHRHVQET

TDSILSHFQSLSYSLAERSSLLQKAIAQSQSVQESLESLLQSIGEVEQNLEGKQVSSLSS

GVIQEALATNMKLKQDIARQKSSLEATREMVTRFMETADSTTAAVLQGKLAEVSQRFEQL

CLQQQEKESSLKKLLPQAEMFEHLSGKLQQFMENKSRMLASGNQPDQDITHFFQQIQELN

LEMEDQQENLDTLEHLVTELSSCGFALDLCQHQDRVQNLRKDFTELQKTVKEREKDASSC

QEQLDEFRKLVRTFQKWLKETEGSIPPTETSMSAKELEKQIEHLKSLLDDWASKGTLVEE

INCKGTSLENLIMEITAPDSQGKTGSILPSVGSSVGSVNGYHTCKDLTEIQCDMSDVNLK

YEKLGGVLHERQESLQAILNRMEEVHKEANSVLQWLESKEEVLKSMDAMSSPTKTETVKA

QAESNKAFLAELEQNSPKIQKVKEALAGLLVTYPNSQEAENWKKIQEELNSRWERATEVT

VARQRQLEESASHLACFQAAESQLRPWLMEKELMMGVLGPLSIDPNMLNAQKQQVQFMLK

EFEARRQQHEQLNEAAQGILTGPGDVSLSTSQVQKELQSINQKWVELTDKLNSRSSQIDQ

AIVKSTQYQELLQDLSEKVRAVGQRLSVQSAISTQPEAVKQQLEETSEIRSDLEQLDHEV

KEAQTLCDELSVLIGEQYLKDELKKRLETVALPLQGLEDLAADRINRLQAALASTQQFQQ

MFDELRTWLDDKQSQQAKNCPISAKLERLQSQLQENEEFQKSLNQHSGSYEVIVAEGESL

LLSVPPGEEKRTLQNQLVELKNHWEELSKKTADRQSRLKDCMQKAQKYQWHVEDLVPWIE

DCKAKMSELRVTLDPVQLESSLLRSKAMLNEVEKRRSLLEILNSAADILINSSEADEDGI

RDEKAGINQNMDAVTEELQAKTGSLEEMTQRLREFQESFKNIEKKVEGAKHQLEIFDALG

SQACSNKNLEKLRAQQEVLQALEPQVDYLRNFTQGLVEDAPDGSDASQLLHQAEVAQQEF

LEVKQRVNSGCVMMENKLEGIGQFHCRVREMFSQLADLDDELDGMGAIGRDTDSLQSQIE

DVRLFLNKIHVLKLDIEASEAECRHMLEEEGTLDLLGLKRELEALNKQCGKLTERGKARQ

EQLELTLGRVEDFYRKLKGLNDATTAAEEAEALQWVVGTEVEIINQQLADFKMFQKEQVD

PLQMKLQQVNGLGQGLIQSAGKDCDVQGLEHDMEEINARWNTLNKKVAQRIAQLQEALLH

CGKFQDALEPLLSWLADTEELIANQKPPSAEYKVVKAQIQEQKLLQRLLDDRKATVDMLQ

AEGGRIAQSAELADREKITGQLESLESRWTELLSKAAARQKQLEDILVLAKQFHETAEPI

SDFLSVTEKKLANSEPVGTQTAKIQQQIIRHKALEEDIENHATDVHQAVKIGQSLSSLTS

PAEQGVLSEKIDSLQARYSEIQDRCCRKAALLDQALSNARLFGEDEVEVLNWLAEVEDKL

SSVFVKDFKQDVLHRQHADHLALNEEIVNRKKNVDQAIKNGQALLKQTTGEEVLLIQEKL

DGIKTRYADITVTSSKALRTLEQARQLATKFQSTYEELTGWLREVEEELATSGGQSPTGE

QIPQFQQRQKELKKEVMEHRLVLDTVNEVSRALLELVPWRAREGLDKLVSDANEQYKLVS

DTIGQRVDEIDAAIQRSQQYEQAADAELAWVAETKRKLMALGPIRLEQDQTTAQLQVQKA

FSIDIIRHKDSMDELFSHRSEIFGTCGEEQKTVLQEKTESLIQQYEAISLLNSERYARLE

RAQVLVNQFWETYEELSPWIEETRALIAQLPSPAIDHEQLRQQQEEMRQLRESIAEHKPH

IDKLLKIGPQLKELNPEEGEMVEEKYQKAENMYAQIKEEVRQRALALDEAVSQSTQITEF

HDKIEPMLETLENLSSRLRMPPLIPAEVDKIRECISDNKSATVELEKLQPSFEALKRRGE

ELIGRSQGADKDLAAKEIQDKLDQMVFFWEDIKARAEEREIKFLDVLELAEKFWYDMAAL

LTTIKDTQDIVHDLESPGIDPSIIKQQVEAAETIKEETDGLHEELEFIRILGADLIFACG

ETEKPEVRKSIDEMNNAWENLNKTWKERLEKLEDAMQAAVQYQDTLQAMFDWLDNTVIKL

CTMPPVGTDLNTVKDQLNEMKEFKVEVYQQQIEMEKLNHQGELMLKKATDETDRDIIREP

LTELKHLWENLGEKIAHRQHKLEGALLALGQFQHALEELMSWLTHTEELLDAQRPISGDP

KVIEVELAKHHVLKNDVLAHQATVETVNKAGNELLESSAGDDASSLRSRLEAMNQCWESV

LQKTEEREQQLQSTLQQAQGFHSEIEDFLLELTRMESQLSASKPTGGLPETAREQLDTHM

ELYSQLKAKEETYNQLLDKGRLMLLSRDDSGSGSKTEQSVALLEQKWHVVSSKMEERKSK

LEEALNLATEFQNSLQEFINWLTLAEQSLNIASPPSLILNTVLSQIEEHKVFANEVNAHR

DQIIELDQTGNQLKFLSQKQDVVLIKNLLVSVQSRWEKVVQRSIERGRSLDDARKRAKQF

HEAWKKLIDWLEDAESHLDSELEISNDPDKIKLQLSKHKEFQKTLGGKQPVYDTTIRTGR

ALKEKTLLPEDTQKLDNFLGEVRDKWDTVCGKSVERQHKLEEALLFSGQFMDALQALVDW

LYKVEPQLAEDQPVHGDLDLVMNLMDAHKVFQKELGKRTGTVQVLKRSGRELIENSRDDT

TWVKGQLQELSTRWDTVCKLSVSKQSRLEQALKQAEVFRDTVHMLLEWLSEAEQTLRFRG

ALPDDTEALQSLIDTHKEFMKKVEEKRVDVNSAVAMGEVILAVCHPDCITTIKHWITIIR

ARFEEVLTWAKQHQQRLETALSELVANAELLEELLAWIQWAETTLIQRDQEPIPQNIDRV

KALIAEHQTFMEEMTRKQPDVDRVTKTYKRKNIEPTHAPFIEKSRSGGRKSLSQPTPPPM

PILSQSEAKNPRINQLSARWQQVWLLALERQRKLNDALDRLEELKEFANFDFDVWRKKYM

RWMNHKKSRVMDFFRRIDKDQDGKITRQEFIDGILASKFPTTKLEMTAVADIFDRDGDGY

IDYYEFVAALHPNKDAYRPTTDADKIEDEVTRQVAQCKCAKRFQVEQIGENKYRFFLGNQ

FGDSQQLRLVRILRSTVMVRVGGGWMALDEFLVKNDPCRARGRTNIELREKFILPEGASQ

GMTPFRSRGRRSKPSSRAASPTRSSSSASQSNHSCTSMPSSPATPASGTKVIPSSGSKLK

RPTPTFHSSRTSLAGDTSNSSSPASTGAKTNRADPKKSASRPGSRAGSRAGSRASSRRGS

DASDFDLLETQSACSDTSESSAAGGQGNSRRGLNKPSKIPTMSKKTTTASPRTPGPKR

>sp|Q09666|AHNK_HUMAN 4419 IDIKGPSL

MEKEETTRELLLPNWQGSGSHGLTIAQRDDGVFVQEVTQNSPAARTGVVKEGDQIVGATI

YFDNLQSGEVTQLLNTMGHHTVGLKLHRKGDRSPEPGQTWTREVFSSCSSEVVLSGDDEE

YQRIYTTKIKPRLKSEDGVEGDLGETQSRTITVTRRVTAYTVDVTGREGAKDIDISSPEF

KIKIPRHELTEISNVDVETQSGKTVIRLPSGSGAASPTGSAVDIRAGAISASGPELQGAG

HSKLQVTMPGIKVGGSGVNVNAKGLDLGGRGGVQVPAVDISSSLGGRAVEVQGPSLESGD

HGKIKFPTMKVPKFGVSTGREGQTPKAGLRVSAPEVSVGHKGGKPGLTIQAPQLEVSVPS

ANIEGLEGKLKGPQITGPSLEGDLGLKGAKPQGHIGVDASAPQIGGSITGPSVEVQAPDI

DVQGPGSKLNVPKMKVPKFSVSGAKGEETGIDVTLPTGEVTVPGVSGDVSLPEIATGGLE

GKMKGTKVKTPEMIIQKPKISMQDVDLSLGSPKLKGDIKVSAPGVQGDVKGPQVALKGSR

VDIETPNLEGTLTGPRLGSPSGKTGTCRISMSEVDLNVAAPKVKGGVDVTLPRVEGKVKV

PEVDVRGPKVDVSAPDVEAHGPEWNLKMPKMKMPTFSTPGAKGEGPDVHMTLPKGDISIS

GPKVNVEAPDVNLEGLGGKLKGPDVKLPDMSVKTPKISMPDVDLHVKGTKVKGEYDVTVP

KLEGELKGPKVDIDAPDVDVHGPDWHLKMPKMKMPKFSVPGFKAEGPEVDVNLPKADVDI

SGPKIDVTAPDVSIEEPEGKLKGPKFKMPEMNIKVPKISMPDVDLHLKGPNVKGEYDVTM

PKVESEIKVPDVELKSAKMDIDVPDVEVQGPDWHLKMPKMKMPKFSMPGFKAEGPEVDVN

LPKADVDISGPKVGVEVPDVNIEGPEGKLKGPKFKMPEMNIKAPKISMPDVDLHMKGPKV

KGEYDMTVPKLEGDLKGPKVDVSAPDVEMQGPDWNLKMPKIKMPKFSMPSLKGEGPEFDV

NLSKANVDISAPKVDTNAPDLSLEGPEGKLKGPKFKMPEMHFRAPKMSLPDVDLDLKGPK

MKGNVDISAPKIEGEMQVPDVDIRGPKVDIKAPDVEGQGLDWSLKIPKMKMPKFSMPSLK

GEGPEVDVNLPKADVVVSGPKVDIEAPDVSLEGPEGKLKGPKFKMPEMHFKTPKISMPDV

DLHLKGPKVKGDVDVSVPKVEGEMKVPDVEIKGPKMDIDAPDVEVQGPDWHLKMPKMKMP

KFSMPGFKGEGREVDVNLPKADIDVSGPKVDVEVPDVSLEGPEGKLKGPKFKMPEMHFKA

PKISMPDVDLNLKGPKLKGDVDVSLPEVEGEMKVPDVDIKGPKVDISAPDVDVHGPDWHL

KMPKVKMPKFSMPGFKGEGPEVDVKLPKADVDVSGPKMDAEVPDVNIEGPDAKLKGPKFK

MPEMSIKPQKISIPDVGLHLKGPKMKGDYDVTVPKVEGEIKAPDVDIKGPKVDINAPDVE

VHGPDWHLKMPKVKMPKFSMPGFKGEGPEVDMNLPKADLGVSGPKVDIDVPDVNLEAPEG

KLKGPKFKMPSMNIQTHKISMPDVGLNLKAPKLKTDVDVSLPKVEGDLKGPEIDVKAPKM

DVNVGDIDIEGPEGKLKGPKFKMPEMHFKAPKISMPDVDLHLKGPKVKGDMDVSVPKVEG

EMKVPDVDIKGPKVDIDAPDVEVHDPDWHLKMPKMKMPKFSMPGFKAEGPEVDVNLPKAD

IDVSGPSVDTDAPDLDIEGPEGKLKGSKFKMPKLNIKAPKVSMPDVDLNLKGPKLKGEID

ASVPELEGDLRGPQVDVKGPFVEAEVPDVDLECPDAKLKGPKFKMPEMHFKAPKISMPDV

DLHLKGPKVKGDADVSVPKLEGDLTGPSVGVEVPDVELECPDAKLKGPKFKMPDMHFKAP

KISMPDVDLHLKGPKVKGDVDVSVPKLEGDLTGPSVGVEVPDVELECPDAKLKGPKFKMP

EMHFKTPKISMPDVDLHLKGPKVKGDMDVSVPKVEGEMKVPDVDIKGPKMDIDAPDVDVH

GPDWHLKMPKMKMPKFSMPGFKAEGPEVDVNLPKADVVVSGPKVDVEVPDVSLEGPEGKL

KGPKLKMPEMHFKAPKISMPDVDLHLKGPKVKGDVDVSLPKLEGDLTGPSVDVEVPDVEL

ECPDAKLKGPKFKMPEMHFKTPKISMPDVNLNLKGPKVKGDMDVSVPKVEGEMKVPDVDI

RGPKVDIDAPDVDVHGPDWHLKMPKMKMPKFSMPGFKGEGPEVDVNLPKADVDVSGPKVD

VEVPDVSLEGPEGKLKGPKFKMPEMHFKTPKISMPDVDFNLKGPKIKGDVDVSAPKLEGE

LKGPELDVKGPKLDADMPEVAVEGPNGKWKTPKFKMPDMHFKAPKISMPDLDLHLKSPKA

KGEVDVDVPKLEGDLKGPHVDVSGPDIDIEGPEGKLKGPKFKMPDMHFKAPNISMPDVDL

NLKGPKIKGDVDVSVPEVEGKLEVPDMNIRGPKVDVNAPDVQAPDWHLKMPKMKMPKFSM

PGFKAEGPEVDVNLPKADVDISGPKVDIEGPDVNIEGPEGKLKGPKLKMPEMNIKAPKIS

MPDFDLHLKGPKVKGDVDVSLPKVEGDLKGPEVDIKGPKVDINAPDVGVQGPDWHLKMPK

VKMPKFSMPGFKGEGPDGDVKLPKADIDVSGPKVDIEGPDVNIEGPEGKLKGPKFKMPEM

NIKAPKISMPDIDLNLKGPKVKGDVDVSLPKVEGDLKGPEVDIKGPKVDIDAPDVDVHGP

DWHLKMPKIKMPKISMPGFKGEGPDVDVNLPKADIDVSGPKVDVECPDVNIEGPEGKWKS

PKFKMPEMHFKTPKISMPDIDLNLTGPKIKGDVDVTGPKVEGDLKGPEVDLKGPKVDIDV

PDVNVQGPDWHLKMPKMKMPKFSMPGFKAEGPEVDVNLPKADVDVSGPKVDVEGPDVNIE

GPEGKLKGPKFKMPEMNIKAPKIPMPDFDLHLKGPKVKGDVDISLPKVEGDLKGPEVDIR

GPQVDIDVPDVGVQGPDWHLKMPKVKMPKFSMPGFKGEGPDVDVNLPKADLDVSGPKVDI

DVPDVNIEGPEGKLKGPKFKMPEMNIKAPKISMPDIDLNLKGPKVKGDMDVSLPKVEGDM

KVPDVDIKGPKVDINAPDVDVQGPDWHLKMPKIKMPKISMPGFKGEGPEVDVNLPKADLD

VSGPKVDVDVPDVNIEGPDAKLKGPKFKMPEMNIKAPKISMPDLDLNLKGPKMKGEVDVS

LANVEGDLKGPALDIKGPKIDVDAPDIDIHGPDAKLKGPKLKMPDMHVNMPKISMPEIDL

NLKGSKLKGDVDVSGPKLEGDIKAPSLDIKGPEVDVSGPKLNIEGKSKKSRFKLPKFNFS

GSKVQTPEVDVKGKKPDIDITGPKVDINAPDVEVQGKVKGSKFKMPFLSISSPKVSMPDV

ELNLKSPKVKGDLDIAGPNLEGDFKGPKVDIKAPEVNLNAPDVDVHGPDWNLKMPKMKMP

KFSVSGLKAEGPDVAVDLPKGDINIEGPSMNIEGPDLNVEGPEGGLKGPKFKMPDMNIKA

PKISMPDIDLNLKGPKVKGDVDISLPKLEGDLKGPEVDIKGPKVDINAPDVDVHGPDWHL

KMPKVKMPKFSMPGFKGEGPEVDVTLPKADIDISGPNVDVDVPDVNIEGPDAKLKGPKFK

MPEMNIKAPKISMPDFDLNLKGPKMKGDVVVSLPKVEGDLKGPEVDIKGPKVDIDTPDIN

IEGSEGKFKGPKFKIPEMHLKAPKISMPDIDLNLKGPKVKGDVDVSLPKMEGDLKGPEVD

IKGPKVDINAPDVDVQGPDWHLKMPKVKMPKFSMPGFKGEGPDVDVNLPKADLDVSGPKV

DIDVPDVNIEGPEGKLKGPKFKMPEMNIKAPKISMPDIDLNLKGPKVKGDMDVSLPKVEG

DMQVPDLDIKGPKVDINAPDVDVRGPDWHLKMPKIKMPKISMPGFKGEGPEVDVNLPKAD

LDVSGPKVDVDVPDVNIEGPDAKLKGPKFKMPEMNIKAPKISMPDFDLHLKGPKVKGDVD

VSLPKMEGDLKAPEVDIKGPKVDIDAPDVDVHGPDWHLKMPKVKMPKFSMPGFKGEGPEV

DVNLPKADIDVSGPKVDIDTPDIDIHGPEGKLKGPKFKMPDLHLKAPKISMPEVDLNLKG

PKMKGDVDVSLPKVEGDLKGPEVDIKGPKVDIDVPDVDVQGPDWHLKMPKVKMPKFSMPG

FKGEGPDVDVNLPKADLDVSGPKVDIDVPDVNIEGPDAKLKGPKFKMPEMNIKAPKISMP

DFDLHLKGPKVKGDVDVSLPKVEGDLKGPEVDIKGPKVDIDAPDVDVHGPDWHLKMPKVK

MPKFSMPGFKGEGPDVDVTLPKADIEISGPKVDIDAPDVSIEGPDAKLKGPKFKMPEMNI

KAPKISMPDIDFNLKGPKVKGDVDVSLPKVEGDLKGPEIDIKGPSLDIDTPDVNIEGPEG

KLKGPKFKMPEMNIKAPKISMPDFDLHLKGPKVKGDVDVSLPKVESDLKGPEVDIEGPEG

KLKGPKFKMPDVHFKSPQISMSDIDLNLKGPKIKGDMDISVPKLEGDLKGPKVDVKGPKV

GIDTPDIDIHGPEGKLKGPKFKMPDLHLKAPKISMPEVDLNLKGPKVKGDMDISLPKVEG

DLKGPEVDIRDPKVDIDVPDVDVQGPDWHLKMPKVKMPKFSMPGFKGEGPDVDVNLPKAD

IDVSGPKVDVDVPDVNIEGPDAKLKGPKFKMPEMSIKAPKISMPDIDLNLKGPKVKGDVD

VTLPKVEGDLKGPEADIKGPKVDINTPDVDVHGPDWHLKMPKVKMPKFSMPGFKGEGPDV

DVSLPKADIDVSGPKVDVDIPDVNIEGPDAKLKGPKFKMPEINIKAPKISIPDVDLDLKG

PKVKGDFDVSVPKVEGTLKGPEVDLKGPRLDFEGPDAKLSGPSLKMPSLEISAPKVTAPD

VDLHLKAPKIGFSGPKLEGGEVDLKGPKVEAPSLDVHMDSPDINIEGPDVKIPKFKKPKF

GFGAKSPKADIKSPSLDVTVPEAELNLETPEISVGGKGKKSKFKMPKIHMSGPKIKAKKQ

GFDLNVPGGEIDASLKAPDVDVNIAGPDAALKVDVKSPKTKKTMFGKMYFPDVEFDIKSP

KFKAEAPLPSPKLEGELQAPDLELSLPAIHVEGLDIKAKAPKVKMPDVDISVPKIEGDLK

GPKVQANLGAPDINIEGLDAKVKTPSFGISAPQVSIPDVNVNLKGPKIKGDVPSVGLEGP

DVDLQGPEAKIKFPKFSMPKIGIPGVKMEGGGAEVHAQLPSLEGDLRGPDVKLEGPDVSL

KGPGVDLPSVNLSMPKVSGPDLDLNLKGPSLKGDLDASVPSMKVHAPGLNLSGVGGKMQV

GGDGVKVPGIDATTKLNVGAPDVTLRGPSLQGDLAVSGDIKCPKVSVGAPDLSLEASEGS

IKLPKMKLPQFGISTPGSDLHVNAKGPQVSGELKGPGVDVNLKGPRISAPNVDFNLEGPK

VKGSLGATGEIKGPTVGGGLPGIGVQGLEGNLQMPGIKSSGCDVNLPGVNVKLPTGQISG

PEIKGGLKGSEVGFHGAAPDISVKGPAFNMASPESDFGINLKGPKIKGGADVSGGVSAPD

ISLGEGHLSVKGSGGEWKGPQVSSALNLDTSKFAGGLHFSGPKVEGGVKGGQIGLQAPGL

SVSGPQGHLESGSGKVTFPKMKIPKFTFSGRELVGREMGVDVHFPKAEASIQAGAGDGEW

EESEVKLKKSKIKMPKFNFSKPKGKGGVTGSPEASISGSKGDLKSSKASLGSLEGEAEAE

ASSPKGKFSLFKSKKPRHRSNSFSDEREFSGPSTPTGTLEFEGGEVSLEGGKVKGKHGKL

KFGTFGGLGSKSKGHYEVTGSDDETGKLQGSGVSLASKKSRLSSSSSNDSGNKVGIQLPE

VELSVSTKKE

>sp|Q9HC84|MUC5B_HUMAN 5026 TFDGTSYTF

MGAPSACRTLVLALAAMLVVPQAETQGPVEPSWGNAGHTMDGGAPTSSPTRRVSFVPPVT

VFPSLSPLNPAHNGRVCSTWGDFHYKTFDGDVFRFPGLCNYVFSEHCRAAYEDFNVQLRR

GLVGSRPVVTRVVIKAQGLVLEASNGSVLINGQREELPYSRTGLLVEQSGDYIKVSIRLV

LTFLWNGEDSALLELDPKYANQTCGLCGDFNGLPAFNEFYAHNARLTPLQFGNLQKLDGP

TEQCPDPLPLPAGNCTDEEGICHRTLLGPAFAECHALVDSTAYLAACAQDLCRCPTCPCA

TFVEYSRQCAHAGGQPRNWRCPELCPRTCPLNMQHQECGSPCTDTCSNPQRAQLCEDHCV

DGCFCPPGSTVLDDITHSGCLPLGQCPCTHGGRTYSPGTSFNTTCSSCTCSGGLWQCQDL

PCPGTCSVQGGAHISTYDEKLYDLHGDCSYVLSKKCADSSFTVLAELRKCGLTDNENCLK

AVTLSLDGGDTAIRVQADGGVFLNSIYTQLPLSAANITLFTPSSFFIVVQTGLGLQLLVQ

LVPLMQVFVRLDPAHQGQMCGLCGNFNQNQADDFTALSGVVEATGAAFANTWKAQAACAN

ARNSFEDPCSLSVENENYARHWCSRLTDPNSAFSRCHSIINPKPFHSNCMFDTCNCERSE

DCLCAALSSYVHACAAKGVQLSDWRDGVCTKYMQNCPKSQRYAYVVDACQPTCRGLSEAD

VTCSVSFVPVDGCTCPAGTFLNDAGACVPAQECPCYAHGTVLAPGEVVHDEGAVCSCTGG

KLSCLGASLQKSTGCAAPMVYLDCSNSSAGTPGAECLRSCHTLDVGCFSTHCVSGCVCPP

GLVSDGSGGCIAEEDCPCVHNEATYKPGETIRVDCNTCTCRNRRWECSHRLCLGTCVAYG

DGHFITFDGDRYSFEGSCEYILAQDYCGDNTTHGTFRIVTENIPCGTTGTTCSKAIKLFV

ESYELILQEGTFKAVARGPGGDPPYKIRYMGIFLVIETHGMAVSWDRKTSVFIRLHQDYK

GRVCGLCGNFDDNAINDFATRSRSVVGDALEFGNSWKLSPSCPDALAPKDPCTANPFRKS

WAQKQCSILHGPTFAACRSQVDSTKYYEACVNDACACDSGGDCECFCTAVAAYAQACHDA

GLCVSWRTPDTCPLFCDFYNPHGGCEWHYQPCGAPCLKTCRNPSGHCLVDLPGLEGCYPK

CPPSQPFFNEDQMKCVAQCGCYDKDGNYYDVGARVPTAENCQSCNCTPSGIQCAHSLEAC

TCTYEDRTYSYQDVIYNTTDGLGACLIAICGSNGTIIRKAVACPGTPATTPFTFTTAWVP

HSTTSPALPVSTVCVREVCRWSSWYNGHRPEPGLGGGDFETFENLRQRGYQVCPVLADIE

CRAAQLPDMPLEELGQQVDCDRMRGLMCANSQQSPPLCHDYELRVLCCEYVPCGPSPAPG

TSPQPSLSASTEPAVPTPTQTTATEKTTLWVTPSIRSTAALTSQTGSSSGPVTVTPSAPG

TTTCQPRCQWTEWFDEDYPKSEQLGGDVESYDKIRAAGGHLCQQPKDIECQAESFPNWTL

AQVGQKVHCDVHFGLVCRNWEQEGVFKMCYNYRIRVLCCSDDHCRGRATTPPPTTELETA

TTTTTQALFSTPQPTSSPGLTRAPPASTTAVPTLSEGLTSPRYTSTLGTATTGGPRQSAG

STEPTVPGVATSTLPTRSALPGTTGSLGTWRPSQPPTLAPTTMATSRARPTGTASTASKE

PLTTSLAPTLTSELSTSQAETSTPRTETTMSPLTNTTTSQGTTRCQPKCEWTEWFDVDFP

TSGVASGDMETFENIRAAGGKMCWAPKSIECRAENYPEVSIDQVGQVLTCSLETGLTCKN

EDQTGRFNMCFNYNVRVLCCDDYSHCPSTLATSSTATPSSTPGTTWILTKPTTTATTTAS

TGSTATASSTQATAGTPHVSTTATTPTVTSSKATPFSSPGTATALPALRSTATTPTATSF

TAIPSSSLGTTWTRLSQTTTPMATMSTATPSSTPETVHTSTVLTTTATTTGATGSVATPS

STPGTAHTTKVLTTTTTGFTATPSSSPGRARTLPVWISTTTTPTTRGSTVTPSSIPGTTH

TPTVLTTTTTTVATGSMATPSSSTQTSGTPPSLTTTATTITATGSTTNPSSTPGTTPIPP

VLTTTATTPAATSSTVTPSSALGTTHTPPVPNTTATTHGRSLSPSSPHTVCTAWTSATSG

ILGTTHITEPSTGTSHTPAATTGTTQHSTPALSSPHPSSRTTESPPSPGTTTPGHTTATS

RTTATATPSKTRTSTLLPSQPTSAPITTVVTMGCEPQCAWSEWLDYSYPMPGPSGGDFDT

YSNIRAAGGAVCEQPLGLECRAQAQPGVPLRELGQVVECSLDFGLVCRNREQVGKFKMCF

NYEIRVFCCNYGHCPSTPATSSTATPSSTPGTTWILTELTTTATTTESTGSTATPTSTLR

TAPPPKVLTTTATTPTVTSSKATPSSSPGTATALPALRSTATTPTATSVTPIPSSSLGTT

WTRLSQTTTPTATMSTATPSSTPETAHTSTVLTATATTTGATGSVATPSSTPGTAHTTKV

PTTTTTGFTATPSSSPGTALTPPVWISTTTTPTTRGSTVTPSSIPGTTHTATVLTTTTTT

VATGSMATPSSSTQTSGTPPSLTTTATTITATGSTTNPSSTPGTRPIPPVLTTTATTPAA

TSSTVTPSSALGTTHTPPVPNTTATTHGRSLSPSSPHTVRTAWTSATSGTLGTTHITEPS

TGTSHTPAATTGTTQHSTPALSSPHPSSRTTESPPSPGTTTPGHTTATSRTTATATPSKT

RTSTLLPSSPTSAPITTVVTMGCEPQCAWSEWLDYSYPMPGPSGGDFDTYSNIRAAGGAV

CEQPLGLECRAQAQPGVPLRELGQVVECSLDFGLVCRNREQVGKFKMCFNYEIRVFCCNY

GHCPSTPATSSTATPSSTPGTTWILTEQTTAATTTATTGSTAIPSSTPGTAPPPKVLTSQ

ATTPTATSSKATSSSSPRTATTLPVLTSTATKSTATSFTPIPSSTLGTTGTSQNRPPHPM

ATMSTIHPSSTPETTHTSTVLTTKATTTRATSSMSTPSSTPGTTWILTELTTAATTTAAL

PHGTPSSTPGTTWILTEPSTTATVTVPTGSTATASSTRATAGTLKVLTSTATTPTVISSR

ATPSSSPGTATALPALRSTATTPTATSVTAIPSSSLGTAWTRLSQTTTPTATMSTATPSS

TPETVHTSTVLTTTATTTRTGSVATPSSTPGTAHTTKVPTTTTTGFTATPSSSPGTALTP

PVWISTTTTPTTRGSTVTPSSIPGTTHTATVLTTTTTTVATGSMATPSSSTQTSGTPPSL

TTTATTITATGSTTNPSSTPGTTPIPPVLTTTATTPAATSSTVTPSSALGTTHTPPVPNT

TATTHGRSLPPSSPHTVPTAWTSATSGILGTTHITEPSTGTSHTPAATTGTTQPSTPALS

SPHPSSRTTESPPSPGTTTPGHTRGTSRTTATATPSKTRTSTLLPSSPTSAPITTVVTTG

CEPQCAWSEWLDYSYPMPGPSGGDFDTYSNIRAAGGAVCEQPLGLECRAQAQPGVPLREL

GQVVECSLDFGLVCRNREQVGKFKMCFNYEIRVFCCNYGHCPSTPATSSTATPSSTPGTT

WILTKLTTTATTTESTGSTATPSSTQGPPAGTPHVSTTATTPTVTSSKATPFSSPGTATA

LPALRSTATTPTATSFTAIPSSSLGTTWTRLSQTTTPMATMSTATPSSTPETVHTSTVLT

TTATTTGATGSVATPSSTPGTAHTTKVPTTTTTGFTVTPSSSPGTARTPPVWISTTTTPT

TSGSTVTPSSIPGTTHTPTVLTTTTQPVATGSMATPSSSTQTSGTPPSLITTATTITATG

STTNPSSTPGTTPIPPELTTTATTPAATSSTVTPSSALGTTHTPPVPNTTATTHGRSLSP

SSPHTVRTAWTSATSGTLGTTHITEPSTGTSHTPAATTGTTTTSTPALSSPHPSSRTTES

PPSPGTTTPGHTTATSRTTATATPSKTRTSTLLPSQPTSAPITTVVTTGCEPQCAWSEWL

DYSYPMPGPSGGDFDTYSNIRAAGGAVCEQPLGLECRAQAQPGVPLGELGQVVECSLDFG

LVCRNREQVGKFKMCFNYEIRVFCCNYGHCPSTPATSSTAMPSSTPGTTWILTELTTTAT

TTASTGSTATPSSTPGTAPPPKVLTSPATTPTATSSKATSSSSPRTATTLPVLTSTATKS

TATSVTPIPSSTLGTTGTLPEQTTTPVATMSTIHPSSTPETTHTSTVLTTKATTRATSST

STPSSTPGTTWILTELTTAATTTAGTGPTATPSSTPGTTWILTELTTTATTTASTGSTAT

LSSTPGTTWILTEPSTTATVTVPTGSTATASSTQATAGTPHVSTTATTPTVTSSKATPSS

SPGTATALPALRSTATTPTATSFTAIPSSSLGTTWTRLSQTTTPTATMSTATPSSTPETV

HTSTVLTTTATTTGATGSVATPSSTPGTAHTTKVPTTTTTGFTATPSSSPGTALTPPVWI

STTTTPTTTTPTTSGSTVTPSSIPGTTHTARVLTTTTTTVATGSMATPSSSTQTSGTPPS

LTTTATTITATGSTTNPSSTPGTTPIPPVLTSMATTPAATSSKATSSSSPRTATTLPVLT

STATKSTATSFTPIPSSTLWTTWTVPAQTTTPMSTMSTIHTSSTPETTHTSTVLTTTATM

TRATNSTATPSSTLGTTRILTELTTTATTTAATGSTATLSSTPGTTWILTEPSTIATVMV

PTGSTATTSSTLGTAHTPKVVTAMATMPTATASTVPSSSTVGTTRTPAVLPSSLPTFSVS

TVSSSVLTTLRPTGFPSSHFSTPCFCRAFGQFFSPGEVIYNKTDRAGCHFYAVCNQHCDI

DRFQGACPTSPPPVSSAPLSSPSPAPGCDNAIPLRQVNETWTLENCTVARCVGDNRVVLL

DPKPVANVTCVNKHLPIKVSDPSQPCDFHYECECICSMWGGSHYSTFDGTSYTFRGNCTY

VLMREIHARFGNLSLYLDNHYCTASATAAAARCPRALSIHYKSMDIVLTVTMVHGKEEGL

ILFDQIPVSSGFSKNGVLVSVLGTTTMRVDIPALGVTVTFNGQVFQARLPYSLFHNNTEG

QCGTCTNNQRDDCLQRDGTTAASCKDMAKTWLVPDSRKDGCWAPTGTPPTASPAAPVSST

PTPTPCPPQPLCDLMLSQVFAECHNLVPPGPFFNACISDHCRGRLEVPCQSLEAYAELCR

ARGVCSDWRGATGGLCDLTCPPTKVYKPCGPIQPATCNSRNQSPQLEGMAEGCFCPEDQI

LFNAHMGICVQACPCVGPDGFPKFPGERWVSNCQSCVCDEGSVSVQCKPLPCDAQGQPPP

CNRPGFVTVTRPRAENPCCPETVCVCNTTTCPQSLPVCPPGQESICTQEEGDCCPTFRCR

PQLCSYNGTFYGVGATFPGALPCHMCTCLSGDTQDPTVQCQEDACNNTTCPQGFEYKRVA

GQCCGECVQTACLTPDGQPVQLNETWVNSHVDNCTVYLCEAEGGVHLLTPQPASCPDVSS

CRGSLRKTGCCYSCEEDSCQVRINTTILWHQGCETEVNITFCEGSCPGASKYSAEAQAMQ

HQCTCCQERRVHEETVPLHCPNGSAILHTYTHVDECGCTPFCVPAPMAPPHTRGFPAQEA

TAV

>sp|Q9NU22|MDN1_HUMAN 4090 EVISSVSEL

MEHFLLEVAAAPLRLIAAKNEKSRSELGRFLAKQVWTPQDRQCVLSTLAQLLLDKDCTVL

VGRQLRPLLLDLLERNAEAIKAGGQINHDLHERLCVSMSKLIGNHPDVLPFALRYFKDTS

PVFQRLFLESSDANPVRYGRRRMKLRDLMEAAFKFLQQEQSVFRELWDWSVCVPLLRSHD

TLVRWYTANCLALVTCMNEEHKLSFLKKIFNSDELIHFRLRLLEEAQLQDLEKALVLANP

EVSLWRKQKELQYLQGHLVSSDLSPRVTAVCGVVLPGQLPAPGELGGNRSSSREQELALR

SYVLVESVCKSLQTLAMAVASQNAVLLEGPIGCGKTSLVEYLAAVTGRTKPPQLLKVQLG

DQTDSKMLLGMYRCTDVPGEFVWQPGTLTQAATMGHWILLEDIDYAPLDVVSVLIPLLEN

GELLIPGRGDCLKVAPGFQFFATRRLLSCGGNWYRPLNSHATLLDKYWTKIHLDNLDKRE

LNEVLQSRYPSLLAVVDHLLDIYIQLTGEKHHSWSDSSVGCEQAPEEVSEARRENKRPTL

EGRELSLRDLLNWCNRIAHSFDSSSLSASLNIFQEALDCFTAMLSEHTSKLKMAEVIGSK

LNISRKKAEFFCQLYKPEIVINELDLQVGRVRLLRKQSEAVHLQREKFTFAATRPSSVLI

EQLAVCVSKGEPVLLVGETGTGKTSTIQYLAHITGHRLRVVNMNQQSDTADLLGGYKPVD

HKLIWLPLREAFEELFAQTFSKKQNFTFLGHIQTCYRQKRWHDLLRLMQHVHKSAVNKDG

KDSETGLLIKEKWEAFGLRLNHAQQQMKMTENTLLFAFVEGTLAQAVKKGEWILLDEINL

AAPEILECLSGLLEGSSGSLVLLDRGDTEPLVRHPDFRLFACMNPATDVGKRNLPPGIRN

RFTELYVEELESKEDLQVLIVDYLKGLSVNKNTVQGIINFYTALRKESGTKLVDGTGHRP

HYSLRTLCRALRFAASNPCGNIQRSLYEGFCLGFLTQLDRASHPIVQKLICQHIVPGNVK

SLLKQPIPEPKGGRLIQVEGYWIAVGDKEPTIDETYILTSSVKLNLRDIVRVVSAGTYPV

LIQGETSVGKTSLIQWLAAATGNHCVRINNHEHTDIQEYIGCYTSDSSGKLVFKEGVLID

AMRKGYWIILDELNLAPTDVLEALNRLLDDNRELLVTETQEVVKAHPRFMLFATQNPPGL

YGGRKVLSRAFRNRFVELHFDELPSSELETILHKRCSLPPSYCSKLVKVMLDLQSYRRSS

SVFAGKQGFITLRDLFRWAERYRLAEPTEKEYDWLQHLANDGYMLLAGRVRKQEEIDVIQ

EVLEKHFKKKLCPQSLFSKENVLKLLGKLSTQISTLECNFGHIVWTEGMRRLAMLVGRAL

EFGEPVLLVGDTGCGKTTICQVFAALANQKLYSVSCHLHMETSDFLGGLRPVRQKPNDKE

EIDTSRLFEWHDGPLVQAMKEDGFFLLDEISLADDSVLERLNSVLEVEKSLVLAEKGSPE

DKDSEIELLTAGKKFRILATMNPGGDFGKKELSPALRNRFTEIWCPQSTSREDLIQIISH

NLRPGLCLGRIDPKGSDIPEVMLDFIDWLTHQEFGRKCVVSIRDILSWVNFMNKMGEEAA

LKRPEIISTVTSFVHAACLVYIDGIGSGVTSSGFGTALLARKECLKFLIKRLAKIVRLTE

YQKNELKIYDRMKAKEFTGIDNLWGIHPFFIPRGPVLHRNNIADYALSAGTTAMNAQRLL

RATKLKKPILLEGSPGVGKTSLVGALAKASGNTLVRINLSEQTDITDLFGADLPVEGGKG

GEFAWRDGPLLAALKAGHWVVLDELNLASQSVLEGLNACFDHRGEIYVPELGMSFQVQHE

KTKIFGCQNPFRQGGGRKGLPRSFLNRFTQVFVDPLTVIDMEFIASTLFPAIEKNIVKKM

VAFNNQIDHEVTVEKKWGQKGGPWEFNLRDLFRWCQLMLVDQSPGCYDPGQHVFLVYGER

MRTEEDKKKVIAVFKDVFGSNSNPYMGTRLFRITPYDVQLGYSVLSRGSCVPHPSRHPLL

LLHQSFQPLESIMKCVQMSWMVILVGPASVGKTSLVQLLAHLTGHTLKIMAMNSAMDTTE

LLGGFEQVDLIRPWRRLLEKVEGTVRALLRDSLLISADDAEVVLRAWSHFLLTYKPKCLG

EGGKAITMEIVNKLEAVLLLMQRLNNKINSYCKAEFAKLVEEFRSFGVKLTQLASGHSHG

TFEWVDSMLVQALKSGDWLLMDNVNFCNPSVLDRLNALLEPGGVLTISERGMIDGSTPTI

TPNPNFRLFLSMDPVHGDISRAMRNRGLEIYISGEGDASTPDNLDLKVLLHSLGLVGNSV

CDILLALHTETRSTVVGSPTSSVSTLIQTAILIVQYLQRGLSLDRAFSEACWEVYVCSQH

SPANRKLVQALLEKHVSSLRAHETWGDSILGMGLWPDSVPSALFATEDSHLSTVRRDGQI

LVYCLNRMSMKTSSWTRSQPFTLQDLEKIMQSPSPENLKFNAVEVNTYWIDEPDVLVMAV

KLLIERATNQDWMLRVKWLYHLAKNIPQGLESIQIHLEASAASLRNFYSHSLSGAVSNVF

KILQPNTTDEFVIPLDPRWNMQALDMIRNLMDFDPQTDQPDQLFALLESAANKTIIYLDR

EKRVFTEANLVSVGSKKLRESVLRMSFEFHQDPESYHTLPHEIVVNLAAFFELCDALVLL

WVQSSQGMVSDASANEILGSLRWRDRFWTVADTVKVDAPGLALLALHWHWVLKHLVHQIP

RLLMNYEDKYYKEVQTVSEHIQNCLGSQTGGFAGIKKLQKFLGRPFPFKDKLVVECFSQL

KVLNKVLAIREQMSALGESGWQEDINRLQVVASQWTLKKSLLQAWGLILRANILEDVSLD

ELKNFVHAQCLELKAKGLSLGFLEKKHDEASSLSHPDLTSVIHLTRSVQLWPAMEYLAML

WRYKVTADFMAQACLRRCSKNQQPQINEEISHLISFCLYHTPVTPQELRDLWSLLHHQKV

SPEEITSLWSELFNSMFMSFWSSTVTTNPEYWLMWNPLPGMQQREAPKSVLDSTLKGPGN

LNRPIFSKCCFEVLTSSWRASPWDVSGLPILSSSHVTLGEWVERTQQLQDISSMLWTNMA

ISSVAEFRRTDSQLQGQVLFRHLAGLAELLPESRRQEYMQNCEQLLLGSSQAFQHVGQTL

GDMAGQEVLPKELLCQLLTSLHHFVGEGESKRSLPEPAQRGSLWVSLGLLQIQTWLPQAR

FDPAVKREYKLNYVKEELHQLQCEWKTRNLSSQLQTGRDLEDEVVVSYSHPHVRLLRQRM

DRLDNLTCHLLKKQAFRPQLPAYESLVQEIHHYVTSIAKAPAVQDLLTRLLQALHIDGPR

SAQVAQSLLKEEASWQQSHHQFRKRLSEEYTFYPDAVSPLQASILQLQHGMRLVASELHT

SLHSSMVGADRLGTLATALLAFPSVGPTFPTYYAHADTLCSVKSEEVLRGLGKLILKRSG

GKELEGKGQKACPTREQLLMNALLYLRSHVLCKGELDQRALQLFRHVCQEIISEWDEQER

IAQEKAEQESGLYRYRSRNSRTALSEEEEEEREFRKQFPLHEKDFADILVQPTLEENKGT

SDGQEEEAGTNPALLSQNSMQAVMLIHQQLCLNFARSLWYQQTLPPHEAKHYLSLFLSCY

QTGASLVTHFYPLMGVELNDRLLGSQLLACTLSHNTLFGEAPSDLMVKPDGPYDFYQHPN

VPEARQCQPVLQGFSEAVSHLLQDWPEHPALEQLLVVMDRIRSFPLSSPISKFLNGLEIL

LAKAQDWEENASRALSLRKHLDLISQMIIRWRKLELNCWSMSLDNTMKRHTEKSTKHWFS

IYQMLEKHMQEQTEEQEDDKQMTLMLLVSTLQAFIEGSSLGEFHVRLQMLLVFHCHVLLM

PQVEGKDSLCSVLWNLYHYYKQFFDRVQAKIVELRSPLEKELKEFVKISKWNDVSFWSIK

QSVEKTHRTLFKFMKKFEAVLSEPCRSSLVESDKEEQPDFLPRPTDGAASELSSIQNLNR

ALRETLLAQPAAGQATIPEWCQGAAPSGLEGELLRRLPKLRKRMRKMCLTFMKESPLPRL

VEGLDQFTGEVISSVSELQSLKVEPSAEKEKQRSEAKHILMQKQRALSDLFKHLAKIGLS

YRKGLAWARSKNPQEMLHLHPLDLQSALSIVSSTQEADSRLLTEISSSWDGCQKYFYRSL

ARHARLNAALATPAKEMGMGNVERCRGFSAHLMKMLVRQRRSLTTLSEQWIILRNLLSCV

QEIHSRLMGPQAYPVAFPPQDGVQQWTERLQHLAMQCQILLEQLSWLLQCCPSVGPAPGH

GNVQVLGQPPGPCLEGPELSKGQLCGVVLDLIPSNLSYPSPIPGSQLPSGCRMRKQDHLW

QQSTTRLTEMLKTIKTVKADVDKIRQQSCETLFHSWKDFEVCSSALSCLSQVSVHLQGLE

SLFILPGMEVEQRDSQMALVESLEYVRGEISKAMADFTTWKTHLLTSDSQGGNQMLDEGF

VEDFSEQMEIAIRAILCAIQNLEERKNEKAEENTDQASPQEDYAGFERLQSGHLTKLLED

DFWADVSTLHVQKIISAISELLERLKSYGEDGTAAKHLFFSQSCSLLVRLVPVLSSYSDL

VLFFLTMSLATHRSTAKLLSVLAQVFTELAQKGFCLPKEFMEDSAGEGATEFHDYEGGGI

GEGEGMKDVSDQIGNEEQVEDTFQKGQEKDKEDPDSKSDIKGEDNAIEMSEDFDGKMHDG

ELEEQEEDDEKSDSEGGDLDKHMGDLNGEEADKLDERLWGDDDEEEDEEEEDNKTEETGP

GMDEEDSELVAKDDNLDSGNSNKDKSQQDKKEEKEEAEADDGGQGEDKINEQIDERDYDE

NEVDPYHGNQEKVPEPEALDLPDDLNLDSEDKNGGEDTDNEEGEEENPLEIKEKPEEAGH

EAEERGETETDQNESQSPQEPEEGPSEDDKAEGEEEMDTGADDQDGDAAQHPEEHSEEQQ

QSVEEKDKEADEEGGENGPADQGFQPQEEEEREDSDTEEQVPEALERKEHASCGQTGVEN

MQNTQAMELAGAAPEKEQGKEEHGSGAADANQAEGHESNFIAQLASQKHTRKNTQSFKRK

PGQADNERSMGDHNERVHKRLRTVDTDSHAEQGPAQQPQAQVEDADAFEHIKQGSDAYDA

QTYDVASKEQQQSAKDSGKDQEEEEIEDTLMDTEEQEEFKAADVEQLKPEEIKSGTTAPL

GFDEMEVEIQTVKTEEDQDPRTDKAHKETENEKPERSRESTIHTAHQFLMDTIFQPFLKD

VNELRQELERQLEMWQPRESGNPEEEKVAAEMWQSYLILTAPLSQRLCEELRLILEPTQA

AKLKGDYRTGKRLNIRKVIPYIASQFRKDKIWLRRTKPSKRQYQICLAIDDSSSMVDNHT

KQLAFESLAVIGNALTLLEVGQIAVCSFGESVKLLHPFHEQFSDYSGSQILRLCKFQQKK

TKIAQFLESVANMFAAAQQLSQNISSETAQLLLVVSDGRGLFLEGKERVLAAVQAARNAN

IFVIFVVLDNPSSRDSILDIKVPIFKGPGEMPEIRSYMEEFPFPYYIILRDVNALPETLS

DALRQWFELVTASDHP

>sp|Q9NU22|MDN1_HUMAN 2715 NEILGSLRW

MEHFLLEVAAAPLRLIAAKNEKSRSELGRFLAKQVWTPQDRQCVLSTLAQLLLDKDCTVL

VGRQLRPLLLDLLERNAEAIKAGGQINHDLHERLCVSMSKLIGNHPDVLPFALRYFKDTS

PVFQRLFLESSDANPVRYGRRRMKLRDLMEAAFKFLQQEQSVFRELWDWSVCVPLLRSHD

TLVRWYTANCLALVTCMNEEHKLSFLKKIFNSDELIHFRLRLLEEAQLQDLEKALVLANP

EVSLWRKQKELQYLQGHLVSSDLSPRVTAVCGVVLPGQLPAPGELGGNRSSSREQELALR

SYVLVESVCKSLQTLAMAVASQNAVLLEGPIGCGKTSLVEYLAAVTGRTKPPQLLKVQLG

DQTDSKMLLGMYRCTDVPGEFVWQPGTLTQAATMGHWILLEDIDYAPLDVVSVLIPLLEN

GELLIPGRGDCLKVAPGFQFFATRRLLSCGGNWYRPLNSHATLLDKYWTKIHLDNLDKRE

LNEVLQSRYPSLLAVVDHLLDIYIQLTGEKHHSWSDSSVGCEQAPEEVSEARRENKRPTL

EGRELSLRDLLNWCNRIAHSFDSSSLSASLNIFQEALDCFTAMLSEHTSKLKMAEVIGSK

LNISRKKAEFFCQLYKPEIVINELDLQVGRVRLLRKQSEAVHLQREKFTFAATRPSSVLI

EQLAVCVSKGEPVLLVGETGTGKTSTIQYLAHITGHRLRVVNMNQQSDTADLLGGYKPVD

HKLIWLPLREAFEELFAQTFSKKQNFTFLGHIQTCYRQKRWHDLLRLMQHVHKSAVNKDG

KDSETGLLIKEKWEAFGLRLNHAQQQMKMTENTLLFAFVEGTLAQAVKKGEWILLDEINL

AAPEILECLSGLLEGSSGSLVLLDRGDTEPLVRHPDFRLFACMNPATDVGKRNLPPGIRN

RFTELYVEELESKEDLQVLIVDYLKGLSVNKNTVQGIINFYTALRKESGTKLVDGTGHRP

HYSLRTLCRALRFAASNPCGNIQRSLYEGFCLGFLTQLDRASHPIVQKLICQHIVPGNVK

SLLKQPIPEPKGGRLIQVEGYWIAVGDKEPTIDETYILTSSVKLNLRDIVRVVSAGTYPV

LIQGETSVGKTSLIQWLAAATGNHCVRINNHEHTDIQEYIGCYTSDSSGKLVFKEGVLID

AMRKGYWIILDELNLAPTDVLEALNRLLDDNRELLVTETQEVVKAHPRFMLFATQNPPGL

YGGRKVLSRAFRNRFVELHFDELPSSELETILHKRCSLPPSYCSKLVKVMLDLQSYRRSS

SVFAGKQGFITLRDLFRWAERYRLAEPTEKEYDWLQHLANDGYMLLAGRVRKQEEIDVIQ

EVLEKHFKKKLCPQSLFSKENVLKLLGKLSTQISTLECNFGHIVWTEGMRRLAMLVGRAL

EFGEPVLLVGDTGCGKTTICQVFAALANQKLYSVSCHLHMETSDFLGGLRPVRQKPNDKE

EIDTSRLFEWHDGPLVQAMKEDGFFLLDEISLADDSVLERLNSVLEVEKSLVLAEKGSPE

DKDSEIELLTAGKKFRILATMNPGGDFGKKELSPALRNRFTEIWCPQSTSREDLIQIISH

NLRPGLCLGRIDPKGSDIPEVMLDFIDWLTHQEFGRKCVVSIRDILSWVNFMNKMGEEAA

LKRPEIISTVTSFVHAACLVYIDGIGSGVTSSGFGTALLARKECLKFLIKRLAKIVRLTE

YQKNELKIYDRMKAKEFTGIDNLWGIHPFFIPRGPVLHRNNIADYALSAGTTAMNAQRLL

RATKLKKPILLEGSPGVGKTSLVGALAKASGNTLVRINLSEQTDITDLFGADLPVEGGKG

GEFAWRDGPLLAALKAGHWVVLDELNLASQSVLEGLNACFDHRGEIYVPELGMSFQVQHE

KTKIFGCQNPFRQGGGRKGLPRSFLNRFTQVFVDPLTVIDMEFIASTLFPAIEKNIVKKM

VAFNNQIDHEVTVEKKWGQKGGPWEFNLRDLFRWCQLMLVDQSPGCYDPGQHVFLVYGER

MRTEEDKKKVIAVFKDVFGSNSNPYMGTRLFRITPYDVQLGYSVLSRGSCVPHPSRHPLL

LLHQSFQPLESIMKCVQMSWMVILVGPASVGKTSLVQLLAHLTGHTLKIMAMNSAMDTTE

LLGGFEQVDLIRPWRRLLEKVEGTVRALLRDSLLISADDAEVVLRAWSHFLLTYKPKCLG

EGGKAITMEIVNKLEAVLLLMQRLNNKINSYCKAEFAKLVEEFRSFGVKLTQLASGHSHG

TFEWVDSMLVQALKSGDWLLMDNVNFCNPSVLDRLNALLEPGGVLTISERGMIDGSTPTI

TPNPNFRLFLSMDPVHGDISRAMRNRGLEIYISGEGDASTPDNLDLKVLLHSLGLVGNSV

CDILLALHTETRSTVVGSPTSSVSTLIQTAILIVQYLQRGLSLDRAFSEACWEVYVCSQH

SPANRKLVQALLEKHVSSLRAHETWGDSILGMGLWPDSVPSALFATEDSHLSTVRRDGQI

LVYCLNRMSMKTSSWTRSQPFTLQDLEKIMQSPSPENLKFNAVEVNTYWIDEPDVLVMAV

KLLIERATNQDWMLRVKWLYHLAKNIPQGLESIQIHLEASAASLRNFYSHSLSGAVSNVF

KILQPNTTDEFVIPLDPRWNMQALDMIRNLMDFDPQTDQPDQLFALLESAANKTIIYLDR

EKRVFTEANLVSVGSKKLRESVLRMSFEFHQDPESYHTLPHEIVVNLAAFFELCDALVLL

WVQSSQGMVSDASANEILGSLRWRDRFWTVADTVKVDAPGLALLALHWHWVLKHLVHQIP

RLLMNYEDKYYKEVQTVSEHIQNCLGSQTGGFAGIKKLQKFLGRPFPFKDKLVVECFSQL

KVLNKVLAIREQMSALGESGWQEDINRLQVVASQWTLKKSLLQAWGLILRANILEDVSLD

ELKNFVHAQCLELKAKGLSLGFLEKKHDEASSLSHPDLTSVIHLTRSVQLWPAMEYLAML

WRYKVTADFMAQACLRRCSKNQQPQINEEISHLISFCLYHTPVTPQELRDLWSLLHHQKV

SPEEITSLWSELFNSMFMSFWSSTVTTNPEYWLMWNPLPGMQQREAPKSVLDSTLKGPGN

LNRPIFSKCCFEVLTSSWRASPWDVSGLPILSSSHVTLGEWVERTQQLQDISSMLWTNMA

ISSVAEFRRTDSQLQGQVLFRHLAGLAELLPESRRQEYMQNCEQLLLGSSQAFQHVGQTL

GDMAGQEVLPKELLCQLLTSLHHFVGEGESKRSLPEPAQRGSLWVSLGLLQIQTWLPQAR

FDPAVKREYKLNYVKEELHQLQCEWKTRNLSSQLQTGRDLEDEVVVSYSHPHVRLLRQRM

DRLDNLTCHLLKKQAFRPQLPAYESLVQEIHHYVTSIAKAPAVQDLLTRLLQALHIDGPR

SAQVAQSLLKEEASWQQSHHQFRKRLSEEYTFYPDAVSPLQASILQLQHGMRLVASELHT

SLHSSMVGADRLGTLATALLAFPSVGPTFPTYYAHADTLCSVKSEEVLRGLGKLILKRSG

GKELEGKGQKACPTREQLLMNALLYLRSHVLCKGELDQRALQLFRHVCQEIISEWDEQER

IAQEKAEQESGLYRYRSRNSRTALSEEEEEEREFRKQFPLHEKDFADILVQPTLEENKGT

SDGQEEEAGTNPALLSQNSMQAVMLIHQQLCLNFARSLWYQQTLPPHEAKHYLSLFLSCY

QTGASLVTHFYPLMGVELNDRLLGSQLLACTLSHNTLFGEAPSDLMVKPDGPYDFYQHPN

VPEARQCQPVLQGFSEAVSHLLQDWPEHPALEQLLVVMDRIRSFPLSSPISKFLNGLEIL

LAKAQDWEENASRALSLRKHLDLISQMIIRWRKLELNCWSMSLDNTMKRHTEKSTKHWFS

IYQMLEKHMQEQTEEQEDDKQMTLMLLVSTLQAFIEGSSLGEFHVRLQMLLVFHCHVLLM

PQVEGKDSLCSVLWNLYHYYKQFFDRVQAKIVELRSPLEKELKEFVKISKWNDVSFWSIK

QSVEKTHRTLFKFMKKFEAVLSEPCRSSLVESDKEEQPDFLPRPTDGAASELSSIQNLNR

ALRETLLAQPAAGQATIPEWCQGAAPSGLEGELLRRLPKLRKRMRKMCLTFMKESPLPRL

VEGLDQFTGEVISSVSELQSLKVEPSAEKEKQRSEAKHILMQKQRALSDLFKHLAKIGLS

YRKGLAWARSKNPQEMLHLHPLDLQSALSIVSSTQEADSRLLTEISSSWDGCQKYFYRSL

ARHARLNAALATPAKEMGMGNVERCRGFSAHLMKMLVRQRRSLTTLSEQWIILRNLLSCV

QEIHSRLMGPQAYPVAFPPQDGVQQWTERLQHLAMQCQILLEQLSWLLQCCPSVGPAPGH

GNVQVLGQPPGPCLEGPELSKGQLCGVVLDLIPSNLSYPSPIPGSQLPSGCRMRKQDHLW

QQSTTRLTEMLKTIKTVKADVDKIRQQSCETLFHSWKDFEVCSSALSCLSQVSVHLQGLE

SLFILPGMEVEQRDSQMALVESLEYVRGEISKAMADFTTWKTHLLTSDSQGGNQMLDEGF

VEDFSEQMEIAIRAILCAIQNLEERKNEKAEENTDQASPQEDYAGFERLQSGHLTKLLED

DFWADVSTLHVQKIISAISELLERLKSYGEDGTAAKHLFFSQSCSLLVRLVPVLSSYSDL

VLFFLTMSLATHRSTAKLLSVLAQVFTELAQKGFCLPKEFMEDSAGEGATEFHDYEGGGI

GEGEGMKDVSDQIGNEEQVEDTFQKGQEKDKEDPDSKSDIKGEDNAIEMSEDFDGKMHDG

ELEEQEEDDEKSDSEGGDLDKHMGDLNGEEADKLDERLWGDDDEEEDEEEEDNKTEETGP

GMDEEDSELVAKDDNLDSGNSNKDKSQQDKKEEKEEAEADDGGQGEDKINEQIDERDYDE

NEVDPYHGNQEKVPEPEALDLPDDLNLDSEDKNGGEDTDNEEGEEENPLEIKEKPEEAGH

EAEERGETETDQNESQSPQEPEEGPSEDDKAEGEEEMDTGADDQDGDAAQHPEEHSEEQQ

QSVEEKDKEADEEGGENGPADQGFQPQEEEEREDSDTEEQVPEALERKEHASCGQTGVEN

MQNTQAMELAGAAPEKEQGKEEHGSGAADANQAEGHESNFIAQLASQKHTRKNTQSFKRK

PGQADNERSMGDHNERVHKRLRTVDTDSHAEQGPAQQPQAQVEDADAFEHIKQGSDAYDA

QTYDVASKEQQQSAKDSGKDQEEEEIEDTLMDTEEQEEFKAADVEQLKPEEIKSGTTAPL

GFDEMEVEIQTVKTEEDQDPRTDKAHKETENEKPERSRESTIHTAHQFLMDTIFQPFLKD

VNELRQELERQLEMWQPRESGNPEEEKVAAEMWQSYLILTAPLSQRLCEELRLILEPTQA

AKLKGDYRTGKRLNIRKVIPYIASQFRKDKIWLRRTKPSKRQYQICLAIDDSSSMVDNHT

KQLAFESLAVIGNALTLLEVGQIAVCSFGESVKLLHPFHEQFSDYSGSQILRLCKFQQKK

TKIAQFLESVANMFAAAQQLSQNISSETAQLLLVVSDGRGLFLEGKERVLAAVQAARNAN

IFVIFVVLDNPSSRDSILDIKVPIFKGPGEMPEIRSYMEEFPFPYYIILRDVNALPETLS

DALRQWFELVTASDHP

>sp|O94833|BPAEA_HUMAN 3725 FIWENIHTL

MHSSSYSYRSSDSVFSNTTSTRTSLDSNENLLLVHCGPTLINSCISFGSESFDGHRLEML

QQIANRVQRDSVICEDKLILAGNALQSDSKRLESGVQFQNEAEIAGYILECENLLRQHVI

DVQILIDGKYYQADQLVQRVAKLRDEIMALRNECSSVYSKGRILTTEQTKLMISGITQSL

NSGFAQTLHPSLTSGLTQSLTPSLTSSSMTSGLSSGMTSRLTPSVTPAYTPGFPSGLVPN

FSSGVEPNSLQTLKLMQIRKPLLKSSLLDQNLTEEEINMKFVQDLLNWVDEMQVQLDRTE

WGSDLPSVESHLENHKNVHRAIEEFESSLKEAKISEIQMTAPLKLTYAEKLHRLESQYAK

LLNTSRNQERHLDTLHNFVSRATNELIWLNEKEEEEVAYDWSERNTNIARKKDYHAELMR

ELDQKEENIKSVQEIAEQLLLENHPARLTIEAYRAAMQTQWSWILQLCQCVEQHIKENTA

YFEFFNDAKEATDYLRNLKDAIQRKYSCDRSSSIHKLEDLVQESMEEKEELLQYKSTIAN

LMGKAKTIIQLKPRNSDCPLKTSIPIKAICDYRQIEITIYKDDECVLANNSHRAKWKVIS

PTGNEAMVPSVCFTVPPPNKEAVDLANRIEQQYQNVLTLWHESHINMKSVVSWHYLINEI

DRIRASNVASIKTMLPGEHQQVLSNLQSRFEDFLEDSQESQVFSGSDITQLEKEVNVCKQ

YYQELLKSAEREEQEESVYNLYISEVRNIRLRLENCEDRLIRQIRTPLERDDLHESVFRI

TEQEKLKKELERLKDDLGTITNKCEEFFSQAAASSSVPTLRSELNVVLQNMNQVYSMSST

YIDKLKTVNLGLKNTQAAEALVKLYETKLCEEEAVIADKNNIENLISTLKQWRSEVDEKR

QVFHALEDELQKAKAISDEMFKTYKERDLDFDWHKEKADQLVERWQNVHVQIDNRLRDLE

GIGKSLKYYRDTYHPLDDWIQQVETTQRKIQENQPENSKTLATQLNQQKMLVSEIEMKQS

KMDECQKYAEQYSATVEDYELQTMTYRAMVDSQQKSPVKRRRMQSSADLIIQEFMDLRTR

YTALVTLMTQYIKFAGDSLKRLEEEEKSLEEEKKEHVEKAKELQKWVSNISKTLKDAEKA

GKPPFSKQKISSEEISTKKEQLSEALQTIQLFLAKHGDKMTDEERNELEKQVKTLQESYN

LLFSESLKQLQESQTSGDVKVEEKIVAERQQEYKEKLQGICDLLTQTENRLIGHQEAFMI

GDGTVELKKYQSKQEELQKDMQGSAQALAEVVKNTENFLKENGEKLSQEDKALIEQKLNE

AKIKCEQLNLKAEQSKKELDKVVTTAIKEETEKVAAVKQLEESKTKIENLLDWLSNVDKD

SERAGTKHKQVIEQNGTHFQEGDGKSAIGEEDEVNGNLLETDVDGQVGTTQENLNQQYQK

VKAQHEKIISQHQAVIIATQSAQVLLEKQGQYLSPEEKEKLQKNMKELKVHYETALAESE

KKMKLTHSLQEELEKFDADYTEFEHWLQQSEQELENLEAGADDINGLMTKLKRQKSFSED

VISHKGDLRYITISGNRVLEAAKSCSKRDGGKVDTSATHREVQRKLDHATDRFRSLYSKC

NVLGNNLKDLVDKYQHYEDASCGLLAGLQACEATASKHLSEPIAVDPKNLQRQLEETKAL

QGQISSQQVAVEKLKKTAEVLLDARGSLLPAKNDIQKTLDDIVGRYEDLSKSVNERNEKL

QITLTRSLSVQDGLDEMLDWMGNVESSLKEQGQVPLNSTALQDIISKNIMLEQDIAGRQS

SINAMNEKVKKFMETTDPSTASSLQAKMKDLSARFSEASHKHKETLAKMEELKTKVELFE

NLSEKLQTFLETKTQALTEVDVPGKDVTELSQYMQESTSEFLEHKKHLEVLHSLLKEISS

HGLPSDKALVLEKTNNLSKKFKEMEDTIKEKKEAVTSCQEQLDAFQVLVKSLKSWIKETT

KKVPIVQPSFGAEDLGKSLEDTKKLQEKWSLKTPEIQKVNNSGISLCNLISAVTTPAKAI

AAVKSGGAVLNGEGTATNTEEFWANKGLTSIKKDMTDISHGYEDLGLLLKDKIAELNTKL

SKLQKAQEESSAMMQWLQKMNKTATKWQQTPAPTDTEAVKTQVEQNKSFEAELKQNVNKV

QELKDKLTELLEENPDTPEAPRWKQMLTEIDSKWQELNQLTIDRQQKLEESSNNLTQFQT

VEAQLKQWLVEKELMVSVLGPLSIDPNMLNTQRQQVQILLQEFATRKPQYEQLTAAGQGI

LSRPGEDPSLRGIVKEQLAAVTQKWDSLTGQLSDRCDWIDQAIVKSTQYQSLLRSLSDKL

SDLDNKLSSSLAVSTHPDAMNQQLETAQKMKQEIQQEKKQIKVAQALCEDLSALVKEEYL

KAELSRQLEGILKSFKDVEQKAENHVQHLQSACASSHQFQQMSRDFQAWLDTKKEEQNKS

HPISAKLDVLESLIKDHKDFSKTLTAQSHMYEKTIAEGENLLLKTQGSEKAALQLQLNTI

KTNWDTFNKQVKERENKLKESLEKALKYKEQVETLWPWIDKCQNNLEEIKFCLDPAEGEN

SIAKLKSLQKEMDQHFGMVELLNNTANSLLSVCEIDKGVVTDENKSLIQKVDMVTEQLHS

KKFCLENMTQKFKEFQEVSKESKRQLQCAKEQLDIHDSLGSQAYSNKYLTMLQTQQKSLQ

ALKHQVDLAKRLAQDLVVEASDSKGTSDVLLQVETIAQEHSTLSQQVDEKCSFLETKLQG

IGHFRNTIREMFSQFAEFDDELDSMAPVGRDAETLQKQKETIKAFLKKLEALMASNDNAN

KTCKMMLATEETSPDLVGIKRDLEALSKQCNKLLDRAQAREEQVEGTIKRLEEFYSKLKE

FSILLQRAEEHEESQGPVGMETETINQQLNMFKVFQKEEIEPLQGKQQDVNWLGQGLIQS

AAKSTSTQGLEHDLDDVNARWKTLNKKVAQRAAQLQEALLHCGRFQDALESLLSWMVDTE

ELVANQKPPSAEFKVVKAQIQEQKLLQRLLDDRKSTVEVIKREGEKIATTAEPADKVKIL

KQLSLLDSRWEALLNKAETRNRQLEGISVVAQQFHETLEPLNEWLTTIEKRLVNCEPIGT

QASKLEEQIAQHKALEDDIINHNKHLHQAVSIGQSLKVLSSREDKDMVQSKLDFSQVWYI

EIQEKSHSRSELLQQALCNAKIFGEDEVELMNWLNEVHDKLSKLSVQDYSTEGLWKQQSE

LRVLQEDILLRKQNVDQALLNGLELLKQTTGDEVLIIQDKLEAIKARYKDITKLSTDVAK

TLEQALQLARRLHSTHEELCTWLDKVEVELLSYETQVLKGEEASQAQMRPKELKKEAKNN

KALLDSLNEVSSALLELVPWRAREGLEKMVAEDNERYRLVSDTITQKVEEVDAAILRSQQ

FDQAADAELSWITETEKKLMSLGDIRLEQDQTSAQLQVQKTFTMEILRHRDIIDDLVKSG

HKIMTACSEEEKQSMKKKLDKVLKNYDTICQINSERYLQLERAQSLVNQFWETYEELWPW

LTETQSIISQLPAPALEYETLRQQQEEHRQLRELIAEHKPHIDKMNKTGPQLLELSPGEG

FSIQEKYVAADTLYSQIKEDVKKRAVALDEAISQSTQFHDKIDQILESLERIVERLRQPP

SISAEVEKIKEQISENKNVSVDMEKLQPLYETLKQRGEEMIARSGGTDKDISAKAVQDKL

DQMVFIWENIHTLVEEREAKLLDVMELAEKFWCDHMSLIVTIKDTQDFIRDLEDPGIDPS

VVKQQQEAAETIREEIDGLQEELDIVINLGSELIAACGEPDKPIVKKSIDELNSAWDSLN

KAWKDRIDKLEEAMQAAVQYQDGLQAVFDWVDIAGGKLASMSPIGTDLETVKQQIEELKQ

FKSEAYQQQIEMERLNHQAELLLKKVTEESDKHTVQDPLMELKLIWDSLEERIINRQHKL

EGALLALGQFQHALDELLAWLTHTEGLLSEQKPVGGDPKAIEIELAKHHVLQNDVLAHQS

TVEAVNKAGNDLIESSAGEEASNLQNKLEVLNQRWQNVLEKTEQRKQQLDGALRQAKGFH

GEIEDLQQWLTDTERHLLASKPLGGLPETAKEQLNVHMEVCAAFEAKEETYKSLMQKGQQ

MLARCPKSAETNIDQDINNLKEKWESVETKLNERKTKLEEALNLAMEFHNSLQDFINWLT

QAEQTLNVASRPSLILDTVLFQIDEHKVFANEVNSHREQIIELDKTGTHLKYFSQKQDVV

LIKNLLISVQSRWEKVVQRLVERGRSLDDARKRAKQFHEAWSKLMEWLEESEKSLDSELE

IANDPDKIKTQLAQHKEFQKSLGAKHSVYDTTNRTGRSLKEKTSLADDNLKLDDMLSELR

DKWDTICGKSVERQNKLEEALLFSGQFTDALQALIDWLYRVEPQLAEDQPVHGDIDLVMN

LIDNHKAFQKELGKRTSSVQALKRSARELIEGSRDDSSWVKVQMQELSTRWETVCALSIS

KQTRLEAALRQAEEFHSVVHALLEWLAEAEQTLRFHGVLPDDEDALRTLIDQHKEFMKKL

EEKRAELNKATTMGDTVLAICHPDSITTIKHWITIIRARFEEVLAWAKQHQQRLASALAG

LIAKQELLEALLAWLQWAETTLTDKDKEVIPQEIEEVKALIAEHQTFMEEMTRKQPDVDK

VTKTYKRRAADPSSLQSHIPVLDKGRAGRKRFPASSLYPSGSQTQIETKNPRVNLLVSKW

QQVWLLALERRRKLNDALDRLEELREFANFDFDIWRKKYMRWMNHKKSRVMDFFRRIDKD

QDGKITRQEFIDGILSSKFPTSRLEMSAVADIFDRDGDGYIDYYEFVAALHPNKDAYKPI

TDADKIEDEVTRQVAKCKCAKRFQVEQIGDNKYRFFLGNQFGDSQQLRLVRILRSTVMVR

VGGGWMALDEFLVKNDPCRAKGRTNMELREKFILADGASQGMAAFRPRGRRSRPSSRGAS

PNRSTSVSSQAAQAASPQVPATTTPKILHPLTRNYGKPWLTNSKMSTPCKAAECSDFPVP

SAEGTPIQGSKLRLPGYLSGKGFHSGEDSGLITTAAARVRTQFADSKKTPSRPGSRAGSK

AGSRASSRRGSDASDFDISEIQSVCSDVETVPQTHRPTPRAGSRPSTAKPSKIPTPQRKS

PASKLDKSSKR

>sp|Q15149|PLEC1_HUMAN 384 DEKSIITY

MVAGMLMPRDQLRAIYEVLFREGVMVAKKDRRPRSLHPHVPGVTNLQVMRAMASLRARGL

VRETFAWCHFFWYLTNEGIAHLRQYLHLPPEIVAASLQRVRRPVAMVMPARRTPHVQAVQ

GPLGSPPKRGPLPTEEQRLYRRKELEEVSPETPVVPATTQRTLARPGPEPAPATDERDRV

QKKTFTKWVNKHLIKAQRHISDLYEDLRDGHNLISLLEVLSGDSLPREKGRMRFHKLQNV

QIALDYLRHRQVKLVNIRNDDIADGNPKLTLGLIWTIILHFQISDIQVSGQSEDMTAKEK

LLLWSQRMVEGYQGLRCDNFTSSWRDGRLFNAIIHRHKPLLIDMNKVYRQTNLENLDQAF

SVAERDLGVTRLLDPEDVDVPQPDEKSIITYVSSLYDAMPRVPDVQDGVRANELQLRWQE

YRELVLLLLQWMRHHTAAFEERRFPSSFEEIEILWSQFLKFKEMELPAKEADKNRSKGIY

QSLEGAVQAGQLKVPPGYHPLDVEKEWGKLHVAILEREKQLRSEFERLECLQRIVTKLQM

EAGLCEEQLNQADALLQSDVRLLAAGKVPQRAGEVERDLDKADSMIRLLFNDVQTLKDGR

HPQGEQMYRRVYRLHERLVAIRTEYNLRLKAGVAAPATQVAQVTLQSVQRRPELEDSTLR

YLQDLLAWVEENQHRVDGAEWGVDLPSVEAQLGSHRGLHQSIEEFRAKIERARSDEGQLS

PATRGAYRDCLGRLDLQYAKLLNSSKARLRSLESLHSFVAAATKELMWLNEKEEEEVGFD

WSDRNTNMTAKKESYSALMRELELKEKKIKELQNAGDRLLREDHPARPTVESFQAALQTQ

WSWMLQLCCCIEAHLKENAAYFQFFSDVREAEGQLQKLQEALRRKYSCDRSATVTRLEDL

LQDAQDEKEQLNEYKGHLSGLAKRAKAVVQLKPRHPAHPMRGRLPLLAVCDYKQVEVTVH

KGDECQLVGPAQPSHWKVLSSSGSEAAVPSVCFLVPPPNQEAQEAVTRLEAQHQALVTLW

HQLHVDMKSLLAWQSLRRDVQLIRSWSLATFRTLKPEEQRQALHSLELHYQAFLRDSQDA

GGFGPEDRLMAEREYGSCSHHYQQLLQSLEQGAQEESRCQRCISELKDIRLQLEACETRT

VHRLRLPLDKEPARECAQRIAEQQKAQAEVEGLGKGVARLSAEAEKVLALPEPSPAAPTL

RSELELTLGKLEQVRSLSAIYLEKLKTISLVIRGTQGAEEVLRAHEEQLKEAQAVPATLP

ELEATKASLKKLRAQAEAQQPTFDALRDELRGAQEVGERLQQRHGERDVEVERWRERVAQ

LLERWQAVLAQTDVRQRELEQLGRQLRYYRESADPLGAWLQDARRRQEQIQAMPLADSQA

VREQLRQEQALLEEIERHGEKVEECQRFAKQYINAIKDYELQLVTYKAQLEPVASPAKKP

KVQSGSESVIQEYVDLRTHYSELTTLTSQYIKFISETLRRMEEEERLAEQQRAEERERLA

EVEAALEKQRQLAEAHAQAKAQAEREAKELQQRMQEEVVRREEAAVDAQQQKRSIQEELQ

QLRQSSEAEIQAKARQAEAAERSRLRIEEEIRVVRLQLEATERQRGGAEGELQALRARAE

EAEAQKRQAQEEAERLRRQVQDESQRKRQAEVELASRVKAEAEAAREKQRALQALEELRL

QAEEAERRLRQAEVERARQVQVALETAQRSAEAELQSKRASFAEKTAQLERSLQEEHVAV

AQLREEAERRAQQQAEAERAREEAERELERWQLKANEALRLRLQAEEVAQQKSLAQAEAE

KQKEEAEREARRRGKAEEQAVRQRELAEQELEKQRQLAEGTAQQRLAAEQELIRLRAETE

QGEQQRQLLEEELARLQREAAAATQKRQELEAELAKVRAEMEVLLASKARAEEESRSTSE

KSKQRLEAEAGRFRELAEEAARLRALAEEAKRQRQLAEEDAARQRAEAERVLAEKLAAIG

EATRLKTEAEIALKEKEAENERLRRLAEDEAFQRRRLEEQAAQHKADIEERLAQLRKASD

SELERQKGLVEDTLRQRRQVEEEILALKASFEKAAAGKAELELELGRIRSNAEDTLRSKE

QAELEAARQRQLAAEEERRRREAEERVQKSLAAEEEAARQRKAALEEVERLKANVEEARR

LRERAEQESARQLQLAQEAAQKRLQAEEKAHAFAVQQKEQELQQTLQQEQSVLDQLRGEA

EAARRAAEEAEEARVQAEREAAQARRQVEEAERLKQSAEEQAQARAQAQAAAEKLRKEAE

QEAARRAQAEQAALRQKQAADAEMEKHKKFAEQTLRQKAQVEQELTTLRLQLEETDHQKN

LLDEELQRLKAEATEAARQRSQVEEELFSVRVQMEELSKLKARIEAENRALILRDKDNTQ

RFLQEEAEKMKQVAEEAARLSVAAQEAARLRQLAEEDLAQQRALAEKMLKEKMQAVQEAT

RLKAEAELLQQQKELAQEQARRLQEDKEQMAQQLAEETQGFQRTLEAERQRQLEMSAEAE

RLKLRVAEMSRAQARAEEDAQRFRKQAEEIGEKLHRTELATQEKVTLVQTLEIQRQQSDH

DAERLREAIAELEREKEKLQQEAKLLQLKSEEMQTVQQEQLLQETQALQQSFLSEKDSLL

QRERFIEQEKAKLEQLFQDEVAKAQQLREEQQRQQQQMEQERQRLVASMEEARRRQHEAE

EGVRRKQEELQQLEQQRRQQEELLAEENQRLREQLQLLEEQHRAALAHSEEVTASQVAAT

KTLPNGRDALDGPAAEAEPEHSFDGLRRKVSAQRLQEAGILSAEELQRLAQGHTTVDELA

RREDVRHYLQGRSSIAGLLLKATNEKLSVYAALQRQLLSPGTALILLEAQAASGFLLDPV

RNRRLTVNEAVKEGVVGPELHHKLLSAERAVTGYKDPYTGQQISLFQAMQKGLIVREHGI

RLLEAQIATGGVIDPVHSHRVPVDVAYRRGYFDEEMNRVLADPSDDTKGFFDPNTHENLT

YLQLLERCVEDPETGLCLLPLTDKAAKGGELVYTDSEARDVFEKATVSAPFGKFQGKTVT

IWEIINSEYFTAEQRRDLLRQFRTGRITVEKIIKIIITVVEEQEQKGRLCFEGLRSLVPA

AELLESRVIDRELYQQLQRGERSVRDVAEVDTVRRALRGANVIAGVWLEEAGQKLSIYNA

LKKDLLPSDMAVALLEAQAGTGHIIDPATSARLTVDEAVRAGLVGPEFHEKLLSAEKAVT

GYRDPYTGQSVSLFQALKKGLIPREQGLRLLDAQLSTGGIVDPSKSHRVPLDVACARGCL

DEETSRALSAPRADAKAYSDPSTGEPATYGELQQRCRPDQLTGLSLLPLSEKAARARQEE

LYSELQARETFEKTPVEVPVGGFKGRTVTVWELISSEYFTAEQRQELLRQFRTGKVTVEK

VIKILITIVEEVETLRQERLSFSGLRAPVPASELLASGVLSRAQFEQLKDGKTTVKDLSE

LGSVRTLLQGSGCLAGIYLEDTKEKVSIYEAMRRGLLRATTAALLLEAQAATGFLVDPVR

NQRLYVHEAVKAGVVGPELHEQLLSAEKAVTGYRDPYSGSTISLFQAMQKGLVLRQHGIR

LLEAQIATGGIIDPVHSHRVPVDVAYQRGYFSEEMNRVLADPSDDTKGFFDPNTHENLTY

RQLLERCVEDPETGLRLLPLKGAEKAEVVETTQVYTEEETRRAFEETQIDIPGGGSHGGS

TMSLWEVMQSDLIPEEQRAQLMADFQAGRVTKERMIIIIIEIIEKTEIIRQQGLASYDYV

RRRLTAEDLFEARIISLETYNLLREGTRSLREALEAESAWCYLYGTGSVAGVYLPGSRQT

LSIYQALKKGLLSAEVARLLLEAQAATGFLLDPVKGERLTVDEAVRKGLVGPELHDRLLS

AERAVTGYRDPYTEQTISLFQAMKKELIPTEEALRLLDAQLATGGIVDPRLGFHLPLEVA

YQRGYLNKDTHDQLSEPSEVRSYVDPSTDERLSYTQLLRRCRRDDGTGQLLLPLSDARKL

TFRGLRKQITMEELVRSQVMDEATALQLREGLTSIEEVTKNLQKFLEGTSCIAGVFVDAT

KERLSVYQAMKKGIIRPGTAFELLEAQAATGYVIDPIKGLKLTVEEAVRMGIVGPEFKDK

LLSAERAVTGYKDPYSGKLISLFQAMKKGLILKDHGIRLLEAQIATGGIIDPEESHRLPV

EVAYKRGLFDEEMNEILTDPSDDTKGFFDPNTEENLTYLQLMERCITDPQTGLCLLPLKE

KKRERKTSSKSSVRKRRVVIVDPETGKEMSVYEAYRKGLIDHQTYLELSEQECEWEEITI

SSSDGVVKSMIIDRRSGRQYDIDDAIAKNLIDRSALDQYRAGTLSITEFADMLSGNAGGF

RSRSSSVGSSSSYPISPAVSRTQLASWSDPTEETGPVAGILDTETLEKVSITEAMHRNLV

DNITGQRLLEAQACTGGIIDPSTGERFPVTDAVNKGLVDKIMVDRINLAQKAFCGFEDPR

TKTKMSAAQALKKGWLYYEAGQRFLEVQYLTGGLIEPDTPGRVPLDEALQRGTVDARTAQ

KLRDVGAYSKYLTCPKTKLKISYKDALDRSMVEEGTGLRLLEAAAQSTKGYYSPYSVSGS

GSTAGSRTGSRTGSRAGSRRGSFDATGSGFSMTFSSSSYSSSGYGRRYASGSSASLGGPE

SAVA

>sp|Q15149|PLEC1_HUMAN 653 ELEDSTLRY

MVAGMLMPRDQLRAIYEVLFREGVMVAKKDRRPRSLHPHVPGVTNLQVMRAMASLRARGL

VRETFAWCHFFWYLTNEGIAHLRQYLHLPPEIVAASLQRVRRPVAMVMPARRTPHVQAVQ

GPLGSPPKRGPLPTEEQRLYRRKELEEVSPETPVVPATTQRTLARPGPEPAPATDERDRV

QKKTFTKWVNKHLIKAQRHISDLYEDLRDGHNLISLLEVLSGDSLPREKGRMRFHKLQNV

QIALDYLRHRQVKLVNIRNDDIADGNPKLTLGLIWTIILHFQISDIQVSGQSEDMTAKEK

LLLWSQRMVEGYQGLRCDNFTSSWRDGRLFNAIIHRHKPLLIDMNKVYRQTNLENLDQAF

SVAERDLGVTRLLDPEDVDVPQPDEKSIITYVSSLYDAMPRVPDVQDGVRANELQLRWQE

YRELVLLLLQWMRHHTAAFEERRFPSSFEEIEILWSQFLKFKEMELPAKEADKNRSKGIY

QSLEGAVQAGQLKVPPGYHPLDVEKEWGKLHVAILEREKQLRSEFERLECLQRIVTKLQM

EAGLCEEQLNQADALLQSDVRLLAAGKVPQRAGEVERDLDKADSMIRLLFNDVQTLKDGR

HPQGEQMYRRVYRLHERLVAIRTEYNLRLKAGVAAPATQVAQVTLQSVQRRPELEDSTLR

YLQDLLAWVEENQHRVDGAEWGVDLPSVEAQLGSHRGLHQSIEEFRAKIERARSDEGQLS

PATRGAYRDCLGRLDLQYAKLLNSSKARLRSLESLHSFVAAATKELMWLNEKEEEEVGFD

WSDRNTNMTAKKESYSALMRELELKEKKIKELQNAGDRLLREDHPARPTVESFQAALQTQ

WSWMLQLCCCIEAHLKENAAYFQFFSDVREAEGQLQKLQEALRRKYSCDRSATVTRLEDL

LQDAQDEKEQLNEYKGHLSGLAKRAKAVVQLKPRHPAHPMRGRLPLLAVCDYKQVEVTVH

KGDECQLVGPAQPSHWKVLSSSGSEAAVPSVCFLVPPPNQEAQEAVTRLEAQHQALVTLW

HQLHVDMKSLLAWQSLRRDVQLIRSWSLATFRTLKPEEQRQALHSLELHYQAFLRDSQDA

GGFGPEDRLMAEREYGSCSHHYQQLLQSLEQGAQEESRCQRCISELKDIRLQLEACETRT

VHRLRLPLDKEPARECAQRIAEQQKAQAEVEGLGKGVARLSAEAEKVLALPEPSPAAPTL

RSELELTLGKLEQVRSLSAIYLEKLKTISLVIRGTQGAEEVLRAHEEQLKEAQAVPATLP

ELEATKASLKKLRAQAEAQQPTFDALRDELRGAQEVGERLQQRHGERDVEVERWRERVAQ

LLERWQAVLAQTDVRQRELEQLGRQLRYYRESADPLGAWLQDARRRQEQIQAMPLADSQA

VREQLRQEQALLEEIERHGEKVEECQRFAKQYINAIKDYELQLVTYKAQLEPVASPAKKP

KVQSGSESVIQEYVDLRTHYSELTTLTSQYIKFISETLRRMEEEERLAEQQRAEERERLA

EVEAALEKQRQLAEAHAQAKAQAEREAKELQQRMQEEVVRREEAAVDAQQQKRSIQEELQ

QLRQSSEAEIQAKARQAEAAERSRLRIEEEIRVVRLQLEATERQRGGAEGELQALRARAE

EAEAQKRQAQEEAERLRRQVQDESQRKRQAEVELASRVKAEAEAAREKQRALQALEELRL

QAEEAERRLRQAEVERARQVQVALETAQRSAEAELQSKRASFAEKTAQLERSLQEEHVAV

AQLREEAERRAQQQAEAERAREEAERELERWQLKANEALRLRLQAEEVAQQKSLAQAEAE

KQKEEAEREARRRGKAEEQAVRQRELAEQELEKQRQLAEGTAQQRLAAEQELIRLRAETE

QGEQQRQLLEEELARLQREAAAATQKRQELEAELAKVRAEMEVLLASKARAEEESRSTSE

KSKQRLEAEAGRFRELAEEAARLRALAEEAKRQRQLAEEDAARQRAEAERVLAEKLAAIG

EATRLKTEAEIALKEKEAENERLRRLAEDEAFQRRRLEEQAAQHKADIEERLAQLRKASD

SELERQKGLVEDTLRQRRQVEEEILALKASFEKAAAGKAELELELGRIRSNAEDTLRSKE

QAELEAARQRQLAAEEERRRREAEERVQKSLAAEEEAARQRKAALEEVERLKANVEEARR

LRERAEQESARQLQLAQEAAQKRLQAEEKAHAFAVQQKEQELQQTLQQEQSVLDQLRGEA

EAARRAAEEAEEARVQAEREAAQARRQVEEAERLKQSAEEQAQARAQAQAAAEKLRKEAE

QEAARRAQAEQAALRQKQAADAEMEKHKKFAEQTLRQKAQVEQELTTLRLQLEETDHQKN

LLDEELQRLKAEATEAARQRSQVEEELFSVRVQMEELSKLKARIEAENRALILRDKDNTQ

RFLQEEAEKMKQVAEEAARLSVAAQEAARLRQLAEEDLAQQRALAEKMLKEKMQAVQEAT

RLKAEAELLQQQKELAQEQARRLQEDKEQMAQQLAEETQGFQRTLEAERQRQLEMSAEAE

RLKLRVAEMSRAQARAEEDAQRFRKQAEEIGEKLHRTELATQEKVTLVQTLEIQRQQSDH

DAERLREAIAELEREKEKLQQEAKLLQLKSEEMQTVQQEQLLQETQALQQSFLSEKDSLL

QRERFIEQEKAKLEQLFQDEVAKAQQLREEQQRQQQQMEQERQRLVASMEEARRRQHEAE

EGVRRKQEELQQLEQQRRQQEELLAEENQRLREQLQLLEEQHRAALAHSEEVTASQVAAT

KTLPNGRDALDGPAAEAEPEHSFDGLRRKVSAQRLQEAGILSAEELQRLAQGHTTVDELA

RREDVRHYLQGRSSIAGLLLKATNEKLSVYAALQRQLLSPGTALILLEAQAASGFLLDPV

RNRRLTVNEAVKEGVVGPELHHKLLSAERAVTGYKDPYTGQQISLFQAMQKGLIVREHGI

RLLEAQIATGGVIDPVHSHRVPVDVAYRRGYFDEEMNRVLADPSDDTKGFFDPNTHENLT

YLQLLERCVEDPETGLCLLPLTDKAAKGGELVYTDSEARDVFEKATVSAPFGKFQGKTVT

IWEIINSEYFTAEQRRDLLRQFRTGRITVEKIIKIIITVVEEQEQKGRLCFEGLRSLVPA

AELLESRVIDRELYQQLQRGERSVRDVAEVDTVRRALRGANVIAGVWLEEAGQKLSIYNA

LKKDLLPSDMAVALLEAQAGTGHIIDPATSARLTVDEAVRAGLVGPEFHEKLLSAEKAVT

GYRDPYTGQSVSLFQALKKGLIPREQGLRLLDAQLSTGGIVDPSKSHRVPLDVACARGCL

DEETSRALSAPRADAKAYSDPSTGEPATYGELQQRCRPDQLTGLSLLPLSEKAARARQEE

LYSELQARETFEKTPVEVPVGGFKGRTVTVWELISSEYFTAEQRQELLRQFRTGKVTVEK

VIKILITIVEEVETLRQERLSFSGLRAPVPASELLASGVLSRAQFEQLKDGKTTVKDLSE

LGSVRTLLQGSGCLAGIYLEDTKEKVSIYEAMRRGLLRATTAALLLEAQAATGFLVDPVR

NQRLYVHEAVKAGVVGPELHEQLLSAEKAVTGYRDPYSGSTISLFQAMQKGLVLRQHGIR

LLEAQIATGGIIDPVHSHRVPVDVAYQRGYFSEEMNRVLADPSDDTKGFFDPNTHENLTY

RQLLERCVEDPETGLRLLPLKGAEKAEVVETTQVYTEEETRRAFEETQIDIPGGGSHGGS

TMSLWEVMQSDLIPEEQRAQLMADFQAGRVTKERMIIIIIEIIEKTEIIRQQGLASYDYV

RRRLTAEDLFEARIISLETYNLLREGTRSLREALEAESAWCYLYGTGSVAGVYLPGSRQT

LSIYQALKKGLLSAEVARLLLEAQAATGFLLDPVKGERLTVDEAVRKGLVGPELHDRLLS

AERAVTGYRDPYTEQTISLFQAMKKELIPTEEALRLLDAQLATGGIVDPRLGFHLPLEVA

YQRGYLNKDTHDQLSEPSEVRSYVDPSTDERLSYTQLLRRCRRDDGTGQLLLPLSDARKL

TFRGLRKQITMEELVRSQVMDEATALQLREGLTSIEEVTKNLQKFLEGTSCIAGVFVDAT

KERLSVYQAMKKGIIRPGTAFELLEAQAATGYVIDPIKGLKLTVEEAVRMGIVGPEFKDK

LLSAERAVTGYKDPYSGKLISLFQAMKKGLILKDHGIRLLEAQIATGGIIDPEESHRLPV

EVAYKRGLFDEEMNEILTDPSDDTKGFFDPNTEENLTYLQLMERCITDPQTGLCLLPLKE

KKRERKTSSKSSVRKRRVVIVDPETGKEMSVYEAYRKGLIDHQTYLELSEQECEWEEITI

SSSDGVVKSMIIDRRSGRQYDIDDAIAKNLIDRSALDQYRAGTLSITEFADMLSGNAGGF

RSRSSSVGSSSSYPISPAVSRTQLASWSDPTEETGPVAGILDTETLEKVSITEAMHRNLV

DNITGQRLLEAQACTGGIIDPSTGERFPVTDAVNKGLVDKIMVDRINLAQKAFCGFEDPR

TKTKMSAAQALKKGWLYYEAGQRFLEVQYLTGGLIEPDTPGRVPLDEALQRGTVDARTAQ

KLRDVGAYSKYLTCPKTKLKISYKDALDRSMVEEGTGLRLLEAAAQSTKGYYSPYSVSGS

GSTAGSRTGSRTGSRAGSRRGSFDATGSGFSMTFSSSSYSSSGYGRRYASGSSASLGGPE

SAVA

>sp|Q15149|PLEC1_HUMAN 732 GRLDLQYAKL

MVAGMLMPRDQLRAIYEVLFREGVMVAKKDRRPRSLHPHVPGVTNLQVMRAMASLRARGL

VRETFAWCHFFWYLTNEGIAHLRQYLHLPPEIVAASLQRVRRPVAMVMPARRTPHVQAVQ

GPLGSPPKRGPLPTEEQRLYRRKELEEVSPETPVVPATTQRTLARPGPEPAPATDERDRV

QKKTFTKWVNKHLIKAQRHISDLYEDLRDGHNLISLLEVLSGDSLPREKGRMRFHKLQNV

QIALDYLRHRQVKLVNIRNDDIADGNPKLTLGLIWTIILHFQISDIQVSGQSEDMTAKEK

LLLWSQRMVEGYQGLRCDNFTSSWRDGRLFNAIIHRHKPLLIDMNKVYRQTNLENLDQAF

SVAERDLGVTRLLDPEDVDVPQPDEKSIITYVSSLYDAMPRVPDVQDGVRANELQLRWQE

YRELVLLLLQWMRHHTAAFEERRFPSSFEEIEILWSQFLKFKEMELPAKEADKNRSKGIY

QSLEGAVQAGQLKVPPGYHPLDVEKEWGKLHVAILEREKQLRSEFERLECLQRIVTKLQM

EAGLCEEQLNQADALLQSDVRLLAAGKVPQRAGEVERDLDKADSMIRLLFNDVQTLKDGR

HPQGEQMYRRVYRLHERLVAIRTEYNLRLKAGVAAPATQVAQVTLQSVQRRPELEDSTLR

YLQDLLAWVEENQHRVDGAEWGVDLPSVEAQLGSHRGLHQSIEEFRAKIERARSDEGQLS

PATRGAYRDCLGRLDLQYAKLLNSSKARLRSLESLHSFVAAATKELMWLNEKEEEEVGFD

WSDRNTNMTAKKESYSALMRELELKEKKIKELQNAGDRLLREDHPARPTVESFQAALQTQ

WSWMLQLCCCIEAHLKENAAYFQFFSDVREAEGQLQKLQEALRRKYSCDRSATVTRLEDL

LQDAQDEKEQLNEYKGHLSGLAKRAKAVVQLKPRHPAHPMRGRLPLLAVCDYKQVEVTVH

KGDECQLVGPAQPSHWKVLSSSGSEAAVPSVCFLVPPPNQEAQEAVTRLEAQHQALVTLW

HQLHVDMKSLLAWQSLRRDVQLIRSWSLATFRTLKPEEQRQALHSLELHYQAFLRDSQDA

GGFGPEDRLMAEREYGSCSHHYQQLLQSLEQGAQEESRCQRCISELKDIRLQLEACETRT

VHRLRLPLDKEPARECAQRIAEQQKAQAEVEGLGKGVARLSAEAEKVLALPEPSPAAPTL

RSELELTLGKLEQVRSLSAIYLEKLKTISLVIRGTQGAEEVLRAHEEQLKEAQAVPATLP

ELEATKASLKKLRAQAEAQQPTFDALRDELRGAQEVGERLQQRHGERDVEVERWRERVAQ

LLERWQAVLAQTDVRQRELEQLGRQLRYYRESADPLGAWLQDARRRQEQIQAMPLADSQA

VREQLRQEQALLEEIERHGEKVEECQRFAKQYINAIKDYELQLVTYKAQLEPVASPAKKP

KVQSGSESVIQEYVDLRTHYSELTTLTSQYIKFISETLRRMEEEERLAEQQRAEERERLA

EVEAALEKQRQLAEAHAQAKAQAEREAKELQQRMQEEVVRREEAAVDAQQQKRSIQEELQ

QLRQSSEAEIQAKARQAEAAERSRLRIEEEIRVVRLQLEATERQRGGAEGELQALRARAE

EAEAQKRQAQEEAERLRRQVQDESQRKRQAEVELASRVKAEAEAAREKQRALQALEELRL

QAEEAERRLRQAEVERARQVQVALETAQRSAEAELQSKRASFAEKTAQLERSLQEEHVAV

AQLREEAERRAQQQAEAERAREEAERELERWQLKANEALRLRLQAEEVAQQKSLAQAEAE

KQKEEAEREARRRGKAEEQAVRQRELAEQELEKQRQLAEGTAQQRLAAEQELIRLRAETE

QGEQQRQLLEEELARLQREAAAATQKRQELEAELAKVRAEMEVLLASKARAEEESRSTSE

KSKQRLEAEAGRFRELAEEAARLRALAEEAKRQRQLAEEDAARQRAEAERVLAEKLAAIG

EATRLKTEAEIALKEKEAENERLRRLAEDEAFQRRRLEEQAAQHKADIEERLAQLRKASD

SELERQKGLVEDTLRQRRQVEEEILALKASFEKAAAGKAELELELGRIRSNAEDTLRSKE

QAELEAARQRQLAAEEERRRREAEERVQKSLAAEEEAARQRKAALEEVERLKANVEEARR

LRERAEQESARQLQLAQEAAQKRLQAEEKAHAFAVQQKEQELQQTLQQEQSVLDQLRGEA

EAARRAAEEAEEARVQAEREAAQARRQVEEAERLKQSAEEQAQARAQAQAAAEKLRKEAE

QEAARRAQAEQAALRQKQAADAEMEKHKKFAEQTLRQKAQVEQELTTLRLQLEETDHQKN

LLDEELQRLKAEATEAARQRSQVEEELFSVRVQMEELSKLKARIEAENRALILRDKDNTQ

RFLQEEAEKMKQVAEEAARLSVAAQEAARLRQLAEEDLAQQRALAEKMLKEKMQAVQEAT

RLKAEAELLQQQKELAQEQARRLQEDKEQMAQQLAEETQGFQRTLEAERQRQLEMSAEAE

RLKLRVAEMSRAQARAEEDAQRFRKQAEEIGEKLHRTELATQEKVTLVQTLEIQRQQSDH

DAERLREAIAELEREKEKLQQEAKLLQLKSEEMQTVQQEQLLQETQALQQSFLSEKDSLL

QRERFIEQEKAKLEQLFQDEVAKAQQLREEQQRQQQQMEQERQRLVASMEEARRRQHEAE

EGVRRKQEELQQLEQQRRQQEELLAEENQRLREQLQLLEEQHRAALAHSEEVTASQVAAT

KTLPNGRDALDGPAAEAEPEHSFDGLRRKVSAQRLQEAGILSAEELQRLAQGHTTVDELA

RREDVRHYLQGRSSIAGLLLKATNEKLSVYAALQRQLLSPGTALILLEAQAASGFLLDPV

RNRRLTVNEAVKEGVVGPELHHKLLSAERAVTGYKDPYTGQQISLFQAMQKGLIVREHGI

RLLEAQIATGGVIDPVHSHRVPVDVAYRRGYFDEEMNRVLADPSDDTKGFFDPNTHENLT

YLQLLERCVEDPETGLCLLPLTDKAAKGGELVYTDSEARDVFEKATVSAPFGKFQGKTVT

IWEIINSEYFTAEQRRDLLRQFRTGRITVEKIIKIIITVVEEQEQKGRLCFEGLRSLVPA

AELLESRVIDRELYQQLQRGERSVRDVAEVDTVRRALRGANVIAGVWLEEAGQKLSIYNA

LKKDLLPSDMAVALLEAQAGTGHIIDPATSARLTVDEAVRAGLVGPEFHEKLLSAEKAVT

GYRDPYTGQSVSLFQALKKGLIPREQGLRLLDAQLSTGGIVDPSKSHRVPLDVACARGCL

DEETSRALSAPRADAKAYSDPSTGEPATYGELQQRCRPDQLTGLSLLPLSEKAARARQEE

LYSELQARETFEKTPVEVPVGGFKGRTVTVWELISSEYFTAEQRQELLRQFRTGKVTVEK

VIKILITIVEEVETLRQERLSFSGLRAPVPASELLASGVLSRAQFEQLKDGKTTVKDLSE

LGSVRTLLQGSGCLAGIYLEDTKEKVSIYEAMRRGLLRATTAALLLEAQAATGFLVDPVR

NQRLYVHEAVKAGVVGPELHEQLLSAEKAVTGYRDPYSGSTISLFQAMQKGLVLRQHGIR

LLEAQIATGGIIDPVHSHRVPVDVAYQRGYFSEEMNRVLADPSDDTKGFFDPNTHENLTY

RQLLERCVEDPETGLRLLPLKGAEKAEVVETTQVYTEEETRRAFEETQIDIPGGGSHGGS

TMSLWEVMQSDLIPEEQRAQLMADFQAGRVTKERMIIIIIEIIEKTEIIRQQGLASYDYV

RRRLTAEDLFEARIISLETYNLLREGTRSLREALEAESAWCYLYGTGSVAGVYLPGSRQT

LSIYQALKKGLLSAEVARLLLEAQAATGFLLDPVKGERLTVDEAVRKGLVGPELHDRLLS

AERAVTGYRDPYTEQTISLFQAMKKELIPTEEALRLLDAQLATGGIVDPRLGFHLPLEVA

YQRGYLNKDTHDQLSEPSEVRSYVDPSTDERLSYTQLLRRCRRDDGTGQLLLPLSDARKL

TFRGLRKQITMEELVRSQVMDEATALQLREGLTSIEEVTKNLQKFLEGTSCIAGVFVDAT

KERLSVYQAMKKGIIRPGTAFELLEAQAATGYVIDPIKGLKLTVEEAVRMGIVGPEFKDK

LLSAERAVTGYKDPYSGKLISLFQAMKKGLILKDHGIRLLEAQIATGGIIDPEESHRLPV

EVAYKRGLFDEEMNEILTDPSDDTKGFFDPNTEENLTYLQLMERCITDPQTGLCLLPLKE

KKRERKTSSKSSVRKRRVVIVDPETGKEMSVYEAYRKGLIDHQTYLELSEQECEWEEITI

SSSDGVVKSMIIDRRSGRQYDIDDAIAKNLIDRSALDQYRAGTLSITEFADMLSGNAGGF

RSRSSSVGSSSSYPISPAVSRTQLASWSDPTEETGPVAGILDTETLEKVSITEAMHRNLV

DNITGQRLLEAQACTGGIIDPSTGERFPVTDAVNKGLVDKIMVDRINLAQKAFCGFEDPR

TKTKMSAAQALKKGWLYYEAGQRFLEVQYLTGGLIEPDTPGRVPLDEALQRGTVDARTAQ

KLRDVGAYSKYLTCPKTKLKISYKDALDRSMVEEGTGLRLLEAAAQSTKGYYSPYSVSGS

GSTAGSRTGSRTGSRAGSRRGSFDATGSGFSMTFSSSSYSSSGYGRRYASGSSASLGGPE

SAVA

>sp|Q96DT5|DYH11_HUMAN 1433 TLADLLALR

MAAQVAAREARDFREAPTLRLTSGAGLEAVGAVELEEEEENEEEAAARRARSFAQDARVR

FLGGRLAMMLGFTEEKWSQYLESEDNRQVLGEFLESTSPACLVFSFAASGRLAASQEIPR

DANHKLVFISKKITESIGVNDFSQVVLFGELPALSLGHVSAFLDEILVPVLSNKNNHKSW

SCFTSQDMEYHIEVMKKKMYIFRGKMSRRTLLPIPTVAGKMDLDQNCSENKPPSNERIIL

HAIESVVIEWSHQIQEIIERDSVQRLLNGLHLSPQAELDFWMMRRENLSCIYDQLQAPVV

LKMVKILTTKQSSYFPTLKDIFLAVENALLEAQDVELYLRPLRRHIQCLQETEFPQTRIL

IAPLFHTICLIWSHSKFYNTPARVIVLLQEFCNLFINQATAYLSPEDLLRGEIEESLEKV

QVAVNILKTFKNSFFNYRKKLASYFMGRKLRPWDFQSHLVFCRFDKFLDRLIKIEDIFAT

TLEFEKLERLEFGGTKGAILNGQVHEMSEELMELCKLFKQSTYDPSDCTNMEFESDYVAF

KSKTLEFDRRLGTIICEAFFNCNGLEAAFKLLTIFGNFLEKPVVMEIFSLHYSTLVHMFN

TELDVCKQLYNEHMKQIECGHVVLNKNMPFTSGNMKWAQQVLQRLQMFWSNFASLRYLFL

GNPDHALVYQKYVEMTTLLDQFESRIYNEWKSNVDEICEFNLNQPLVKFSAINGLLCVNF

DPKLVAVLREVKYLLMLKKQDIPDSALAIFKKRNTILKYIGNLDLLVQGYNKLKQTLLEV

EYPLIEDELRAIDEQLTAATTWLTWQDDCWGYIERVRAATSELEHRVERTQKNVKVIQQT

MRGWARCVLPPRREHRREAAFTLEDKGDLFTKKYKLIQGDGCKIHNLVEENRKLFKANPS

LDTWKIYVEFIDDIVVEGFFQAIMHDLDFFLKNTEKQLKPAPFFQAQMILLPPEIVFKPS

LDREAGDGFYDLVEEMLCNSFRMSAQMNRIATHLEIKNYQNDMDNMLGLAEVRQEIMNRV

VNVINKVLDFRNTLETHAYLWVDDRAEFMKHFLLYGHAVSSDEMDAHANEEIPEQPPTLE

QFKEQIDIYEALYVQMSKFEDFRVFDSWFKVDMKPFKVSLLTIIKKWSWMFQEHLLRFVI

DSLNELQEFIKETDSGLQRELNEGDHDGLVDIMVHLLAVRSRQRATDELFEPLKETITLL

ESYGQKMPEQVYIQLEELPERWETTKKIAATVRHEVSPLHNAEVTLIRKKCILFDAKQAE

FRERFRHYAPLGFNAENPYTALDKANEELEALEEEMLQMQESTRLFEVALPEYKQMKQCR

KEIKLLKGLWDVIIYVRRSIDNWTKTQWRQIHVEQMDVELRRFAKASSITEIWSLNKEVR

VWDAYTGLEGTVKDMTASLRAITELQSPALRDRHWHQLMKAIGVKFLINEATTLADLLAL

RLHRVEDDVRRIVDKAVKELGTEKVITEISQTWATMKFSYEVHYRTGIPLLKSDEQLFET

LEHNQVQLQTLLQSKYVEYFIEQVLSWQNKLNIADLVIFTWMEVQRTWSHLESIFVCSED

IRIQLVKDARRFDGVDAEFKELMFKTAKVENVLEATCRPNLYEKLKDLQSRLSLCEKALA

EYLETKRIAFPRFYFVSSADLLDILSKGAQPKQVTCHLAKLFDSIADLQFEDNQDVSAHR

AVGMYSKEKEYVPFQAECECVGHVETWLLQLEQTMQETVRHSITEAIVAYEEKPRELWIF

DFPAQVALTSSQIWWTTDVGIAFSRLEEGYETALKDFHKKQISQLNTLITLLLGELPPGD

RQKIMTICTIDVHARDVVAKLISQKQVVVSPQAFTWLSQLRHRWEDTQKHCFVNICDAQF

QYFYEYLGNSPRLVITPLTDRCYITLTQSLHLTMSGAPAGPAGTGKTETTKDLGRALGMM

VYVFNCSEQMDYKSIGNIYKGLVQTGAWGCFDEFNRISVEVLSVVAVQVKMIHDAIRNRK

KRFVFLGEAITLKPSVGIFITMNPGYAGRTELPENLKALFRPCAMVAPDIELICEILLVA

EGFVDARALARKFITLYTLCKELLSKQDHYDWGLRAIKSVLVVAGSLKRGDKNRPEDQVL

MRALRDFNMPKIVTDDIPVFLGLVGDLFPALDVPRRRKLHFEQMVRQSTLELRLQPEESF

ILKVVQLEELLAVRHSVFVVGNAGTGKSKILRTLNRTYVNMKQKPVWNDLNPKAVTTDEL

FGFIHHATREWKDGKIVYSYFIGLFSSILREQANLKHDGPKWIVLDGDIDPMWIESLNTV

MDDNKVLTLASNERIALTPFMRLLFEIHHLRSATPATVSRAGILYVNPQDLGWNPYVASW

IDRRRHQSEKANLTILFDKYVPACLDKLRTSFKTITSIPESSLVQTLCVLLECLLTPENV

PSDSPKEVYEVYFVFACIWAFGGTLLQDQISDYQADFSRWWQKEMKAVKFPSQGTIFDYY

VDHKTKKLLPWADKIAQFTMDPDVPLQTVLVHTTETARLRYFMELLLEKGKPLMLVGNAG

VGKTVFVGDTLASLSEDYIVSRVPFNYYTTSTALQKILEKPLEKKAGHNYGPGGNKKLIY

FIDDMNMPEVDLYGTVQPHTLIRQHIDYGHWYDRQKVMLKEIHNCQYVACMNPMVGSFTI

SPRLQRHFTVFAFNFPSLDALNTIYGQIFSFHFQQQAFAPSILRSGPTLIQATIAFHQTM

MCNFLPTAIKFHYIFNLRDLSNVFQGILFASPECLKGPLDLIHLWLHESARVYGDKLIDK

KDCDLFQRRMLETAYKYFEGIDSHMLLQQPLIYCHFADRGKDPHYMPVKDWEVLKTILTE

TLDNYNELNAAMHLVLFEDAMQHVCRISRILRTPQGCALLVGVGGSGKQSLSRLAAYLRG

LEVFQITLTEGYGIQELRVDLANLYIRTGAKNMPTVFLLTDAQVLDESFLVLINDLLASG

EIPDLFSDEDVDKIISGIHNEVHALGMVDSRENCWKFFMARVRLQLKIILCFSPVGRTLR

VRARKFPAIVNCTAIDWFHAWPQEALVSVSRRFIEETKGIEPVHKDSISLFMAHVHTTVN

EMSTRYYQNERRHNYTTPKSFLEQISLFKNLLKKKQNEVSEKKERLVNGIQKLKTTASQV

GDLKARLASQEAELQLRNHDAEALITKIGLQTEKVSREKTIADAEERKVTAIQTEVFQKQ

RECEADLLKAEPALVAATAALNTLNRVNLSELKAFPNPPIAVTNVTAAVMVLLAPRGRVP

KDRSWKAAKVFMGKVDDFLQALINYDKEHIPENCLKVVNEHYLKDPEFNPNLIRTKSFAA

AGLCAWVINIIKFYEVYCDVEPKRQALAQANLELAAATEKLEAIRKKLVDLDRNLSRLTA

SFEKATAEKVRCQEEVNQTNKTIKLANRLVKELEAKKIRWGQSIKSFEAQEKTLCGDVLL

TAAFVSYVGPFTRQYRQELVHCKWVPFLQQKVSIPLTEGLDLISMLTDDATIAAWNNEGL

PSDRMSTENAAILTHCERWPLVIDPQQQGIKWIKNKYGMDLKVTHLGQKGFLNAIETALA

FGDVILIENLEETIDPVLDPLLGRNTIKKGKYIRIGDKECEFNKNFRLILHTKLANPHYK

PELQAQTTLLNFTVTEDGLEAQLLAEVVSIERPDLEKLKLVLTKHQNDFKIELKYLEDDL

LLRLSAAEGSFLDDTKLVERLEATKTTVAEIEHKVIEAKENERKINEARECYRPLAARAS

LLYFVINDLQKINPLYQFSLKAFNVLFHRAIEQADKVEDMQGRISILMESITHAVFLYTS

QALFEKDKLTFLSQMAFQILLRKKEIDPLELDFLLRFTVEHTHLSPVDFLTSQSWSAIKA

IAVMEEFRGIDRDVEGSAKQWRKWVESECPEKEKLPQEWKKKSLIQKLILLRAMRPDRMT

YALRNFVEEKLGAKYVERTRLDLVKAFEESSPATPIFFILSPGVDALKDLEILGKRLGFT

IDSGKFHNVSLGQGQETVAEVALEKASKGGHWVILQNVHLVAKWLGTLEKLLERFSQGSH

RDYRVFMSAESAPTPDEHIIPQGLLENSIKITNEPPTGMLANLHAALYNFDQDTLEICSK

EQEFKSILFSLCYFHACVAGRLRFGPQGWSRSYPFNPGDLTICASVLYNYLEANSKVPWE

DLRYLFGEIMYGGHITDDWDRKLCRVYLEEFMNPSLTEDELMLAPGFAAPPYLDYAGYHQ

YIEEMLPPESPALYGLHPNAEIEFLTVTSNTLFRTLLEMQPRNALSGDELGQSTEEKVKN

VLDDILEKLPEEFNMAEIMQKNSNRSPYVLVCFQECERMNILIREIRISLEQLDLSLKGE

LALSPAVEAQQFALSYDTVPDTWSKLAYPSTYGLAQWFNDLLLRCRELDTWTQDLTLPAV

VWLSGFFNPQSFLTAIMQTMARKNEWPLDKTRLTADVTKKTKEDYGHPPREGAYLHGLFM

EGARWDTQAGTIVEARLKELACPMPVIFAKATPVDRQETKQTYECPVYRTKLRGPSYIWT

FRLKSEEKTAKWVLAGVALLLEA

>sp|Q96JB1|DYH8_HUMAN 1729 FLDILNTLI

MMKLYIDNAAPDKLKGLCIFFVRCRNDVAINVKTIQEEALFTVLDASKGLLNGIRDMLAN

IFLPAVLATNNWGALNQSKQGESEKHIFTETINRYLSFLDGARISIEGTVKLKTIDNVNF

SKLHTFEEVTAAASNSETVHQLEEVLMVWYKQIEQVLIESEQMRKEAGDSGPLTELEHWK

RMSAKFNYIIEQIKGPSCKAVINVLNVAHSKLLKNWRDLDARITDTANESKDNVRYLYTL

EKVCQPLYNHDLVSMAHGIQNLINAIRMIHGVSRYYNTSERMTSLFIKVTNQMVTACKAY

ITDGGLNHVWDQETPVVLKKIQDCIFLFKEYQASFHKTRKLISESSGEKSFEVSEMYIFG

KFEAFCKRLEKITEMITVVQTYSTLSNSTIEGIDIMAIKFRNIYQGVKKKQYDILDPRRT

EFDTDFLDFMTKINGLEVQIQAFMNSSFGKILSSQQALQLLQRFQKLNIPCLGLEINHTI

ERILQYYVAELDATKKLYHSQKDDPPLARNMPPIAGKILWVRQLYRRISEPINYFFKNSD

ILSSPDGKAVIRQYNKISYVLVEFEVVYHTAWIREISQLHYALQATLFVRHPETGKLLVN

FDPKILEVVRETKCMIKMKLDVPEQAKRLLKLESKLKADKLYLQGLLQYYDELCQEVPSV

FVNLMTPKMKKVESVLRQGLTVLTWSSLTLESFFQEVELVLDMFNQLLKKISDLCEMHID

TVLKEIAKTVLISLPESGATKVEDMLTLNETYTKEWADILNHKSKHVEEAVRELISIFEQ

IYEVKYTGKVGKQSEQRKHVVFGSETGEGENNDYEANIVNEFDTHDKEDEFKKECKEVFA

FFSHQLLDSLQKATRLSLDTMKRRIFVASLYGRKQSEDIISFIKSEVHLAIPNVVMIPSL

DDIQQAINRMIQLTLEVSRGVAHWGQQQIRPIKSVIPSPTTTDVTHQNTGKLLKKEERSF

EEAIPARKLKNFYPGVAEHKDISKLVLLLSSSVNSLRKAAHEALQDFQKYKTLWTEDRDV

KVKEFLANNPSLTEIRSEILHYATFEQEIDELKPIIVVGALELHTEPMKLALSIEAKAWK

MLLCRYLNEEYKKKMSYMIAFINEYLKKLSRPIRDLDDVRFAMEALSCIRDNEIQMDMTL

GPIEEAYAILNRFEVEVTKEESEAVDTLRYSFNKLQSKAVSVQEDLVQVQPKFKSNLLES

VEVFREDVINFAEAYELEGPMVPNIPPQEASNRLQIFQASFDDLWRKFVTYSSGEQLFGL

PVTDYEVLHKTRKELNLLQKLYGLYDTVMSSISGYYEILWGDVDIEKINAELLEFQNRCR

KLPKGLKDWQAFLDLKKRIDDFSESCPLLEMMTNKAMKQRHWDRISELTGTPFDVESDSF

CLRNIMEAPLLKHKDDIEDICISAIKEKDIEAKLTQVIENWTNQNLSFAAFKGKGELLLK

GTESGEIITLMEDSLMVLGSLLSNRYNAPFKKNIQNWVYKLSTSSDIIEEWLVVQNLWVY

LEAVFVGGDIAKQLPQEAKRFQNIDKSWIKIMQRAHENPNVINCCVGDETMGQLLPHLHE

QLEVCQKSLTGYLEKKRLLFPRFFFVSDPVLLEILGQASDSHTIQPHLPAVSDNINEVTF

HAKDYDRIMAVISREGEKIVLDNSVMAKGPVEIWLLDLLKMQMSSLHNIIRSAFYQISDS

GFQLLPFLSHFPAQVGLLGIQMLWTHDSEEALRNAKDDRKIMQVTNQKFLDILNTLISQT

THDLSKFDRVKFETLITIHVHQRDIFDDLVKMHIKSPTDFEWLKQSRFYFKEDLDQTVVS

ITDVDFIYQNEFLGCTDRLVITPLTDRCYITLAQALGMNMGGAPAGPAGTGKTETTKDMG

RCLGKYVVVFNCSDQMDFRGLGRIFKGLAQSGSWGCFDEFNRIELPVLSVAAQQIYIVLT

ARKERKKQFIFSDGDCVDLNPEFGIFLTMNPGYAGRQELPENLKIQFRTVAMMVPDRQII

MRVKLASCGFLENVILAQKFYVLYKLCEEQLTKQVHYDFGLRNILSVLRTLGSQKRARPE

DSELSIVMRGLRDMNLSKLVDEDEPLFLSLINDLFPGLQLDSNTYAELQNAVAHQVQIEG

LINHPPWNLKLVQLYETSLVRHGLMTLGPSGSGKTTVITILMKAQTECGRPHREMRMNPK

AITAPQMFGRLDTATNDWTDGIFSTLWRKTLKAKKGENIFLILDGPVDAIWIENLNSVLD

DNKTLTLANGDRIPMAPSCKLLFEVHNIENASPATVSRMGMVYISSSALSWRPILQAWLK

KRTAQEAAVFLTLYEKVFEDTYTYMKLNLNPKMQLLECNYIVQSLNLLEGLIPSKEEGGV

SCVEHLHKLFVFGLMWSLGALLELESREKLEAFLRQHESKLDLPEIPKGSNQTMYEFYVT

DYGDWEHWNKKLQPYYYPTDSIPEYSSILVPNVDNIRTNFLIDTIAKQHKAVLLTGEQGT

AKTVMVKAYLKKYDPEVQLSKSLNFSSATEPMMFQRTIESYVDKRIGSTYGPPGGRKMTV

FIDDINMPVINEWGDQITNEIVRQMMEMEGMYSLDKPGDFTTIVDVQLIAAMIHPGGGRN

DIPQRLKRQFTVFNCTLPSNASIDKIFGIIGCGYFDPCRSFKPQICEMIVNLVSVGRVLW

QWTKVKMLPTPSKFHYIFNLRDLSRIWQGMLTIKAEECASIPTLLSLFKHECSRVIADRF

ITPEDEQWFNAHLTRAVEENIGSDAASCILPEPYFVDFLREMPEPTGDEPEDSVFEVPKI

YELMPSFDFLAEKLQFYQRQFNEIIRGTSLDLVFFKDAMTHLIKISRIIRTSCGNALLVG

VGGSGKQSLSRLASFIAGYQIFQITLTRSYNVTNLTDDLKALYKVAGADGKGITFIFTDS

EIKDEAFLEYLNNLLSSGEISNLFARDEMDEITQGLISVMKRELPRHPPTFDNLYEYFIS

RSRKNLHVVLCFSPVGEKFRARSLKFPGLISGCTMDWFSRWPREALIAVASYFLSDYNIV

CSSEIKRQVVETMGLFHDMVSESCESYFQRYRRRAHVTPKSYLSFINGYKNIYAEKVKFI

NEQAERMNIGLDKLMEASESVAKLSQDLAVKEKELAVASIKADEVLAEVTVSAQASAKIK

NEVQEVKDKAQKIVDEIDSEKVKAESKLEAAKPALEEAEAALNTIKPNDIATVRKLAKPP

HLIMRIMDCVLLLFQKKIDPVTMDPEKSCCKPSWGESLKLMSATGFLWSLQQFPKDTINE

ETVELLQPYFNMDDYTFESAKKVCGNVAGLLSWTLAMAIFYGINREVLPLKANLAKQEGR

LAVANAELGKAQALLDEKQAELDKVQAKFDAAMNEKMDLLNDADTCRKKMQAASTLIDGL

SGEKIRWTQQSKEFKAQINRLVGDILLCTGFLSYLGPFNQIFRNYLLKDQWEMELRARKI

PFTENLNLISMLVDPPTIGEWGLQGLPGDDLSIQNGIIVTKATRYPLLIDPQTQGKTWIK

SKEKENDLQVTSLNHKYFRTHLEDSLSLGRPLLIEDIHEELDPALDNVLEKNFIKSGTTF

KVKVGDKECDIMDTFKLYITTKLPNPAFTPEINAKTSVIDFTVTMKGLENQLLRRVILTE

KQELEAERVKLLEDVTFNKRKMKELEDNLLYKLSATKGSLVDDESLIGVLRTTKQTAAEV

SEKLHVAAETEIKINAAQEEFRPAATRGSILYFLITEMSMVNIMYQTSLAQFLKLFDQSM

ARSEKSPLPQKRITNIIEYLTYEVFTYSVRGLYENHKFLFVLLMTLKIDLQRGTVKHREF

QALIKGGAALDLKACPPKPYRWILDMTWLNLVELSKLPQFAEIMNQISRNEKGWKSWFDK

DAPEEEIIPDGYNDSLDTCHKLLLIRSWCPDRTVFQARKYIADSLEEKYTEPVILNLEKT

WEESDTRTPLICFLSMGSDPTNQIDALAKKLKLECRTISMGQGQEVHARKLIQMSMQQGG

WVLLQNCHLGLEFMEELLETLITTEASDDSFRVWITTEPHDRFPITLLQTSLKFTNEPPQ

GVRAGLKRTFAGINQDLLDISNLPMWKPMLYTVAFLHSTVQERRKFGPLGWNIPYEFNSA

DFSASVQFIQNHLDECDIKKGVSWNTVRYMIGEVQYGGRVTDDFDKRLLNCFARVWFSEK

MFEPSFCFYTGYKIPLCKTLDQYFEYIQSLPSLDNPEVFGLHPNADITYQSNTASAVLET

ITNIQPKESGGGVGETREAIVYRLSEDMLSKLPPDYIPHEVKSRLIKMGHLNSMNIFLRQ

EIDRMQRVISILRSSLSDLKLAIEGTIIMSENLRDALDNMYDARIPQLWKRVSWDSSTLG

FWFTELLERNAQFSTWIFEGRPNVFWMTGFFNPQGFLTAMRQEVTRAHKGWALDTVTIHN

EVLRQTKEEITSPPGEGVYIYGLYMDGAAWDRRNGKLMESTPKVLFTQLPVLHIFAINST

APKDPKLYVCPIYKKPRRTDLTFITVVYLRTVLSPDHWILRGVALLCDIK

>sp|Q9NYC9|DYH9_HUMAN 4234 DEFNIPELM

MRLAEERAALAAENADGEPGADRRLRLLGTYVAMSLRPAAGAWERCAGSAEAEQLLQAFL

GRDAAEGPRPLLVVRPGPRGLAIRPGLEVGPESGLAGAKALFFLRTGPEPPGPDSFRGAV

VCGDLPAAPLEHLAALFSEVVLPVLANEKNRLNWPHMICEDVRRHAHSLQCDLSVILEQV

KGKTLLPLPAGSEKMEFADSKSETVLDSIDKSVIYAIESAVIKWSYQVQVVLKRESSQPL

LQGENPTPKVELEFWKSRYEDLKYIYNQLRTITVRGMAKLLDKLQSSYFPAFKAMYRDVV

AALAEAQDIHVHLIPLQRHLEALENAEFPEVKPQLRPLLHVVCLIWATCKSYRSPGRLTV

LLQEICNLLIQQASNYLSPEDLLRSEVEESQRKLQVVSDTLSFFKQEFQDRRENLHTYFK

ENQEVKEWDFQSSLVFVRLDGFLGRLHVVEGLLKTALDFHKLGKVEFSGVRGNALSQQVQ

QMHEEFQEMYRLLSGSSSDCLYLQSTDFENDVSEFNQKVEDLDRRLGTIFIQAFDDAPGL

EHAFKLLDIAGNLLERPLVARDTSDKYLVLIQMFNKDLDAVRMIYSQHVQEEAELGFSPV

HKNMPTVAGGLRWAQELRQRIQGPFSNFGRITHPCMESAEGKRMQQKYEDMLSLLEKYET

RLYEDWCRTVSEKSQYNLSQPLLKRDPETKEITINFNPQLISVLKEMSYLEPREMKHMPE

TAAAMFSSRDFYRQLVANLELMANWYNKVMKTLLEVEFPLVEEELQNIDLRLRAAEETLN

WKTEGICDYVTEITSSIHDLEQRIQKTKDNVEEIQNIMKTWVTPIFKTKDGKRESLLSLD

DRHDRMEKYYNLIKESGLKIHALVQENLGLFSADPTSNIWKTYVNSIDNLLLNGFFLAIE

CSLKYLLENTECKAGLTPIFEAQLSLAIPELVFYPSLESGVKGGFCDIVEGLITSIFRIP

SLVPRLSPQNGSPHYQVDLDGIPDLANMRRTLMERVQRMMGLCCGYQSTFSQYSYLYVED

RKEVLGQFLLYGHILTPEEIEDHVEDGIPENPPLLSQFKVQIDSYETLYEEVCRLEPIKV

FDGWMKIDIRPFKASLLNIIKRWSLLFKQHLVDHVTHSLANLDAFIKKSESGLLKKVEKG

DFQGLVEIMGHLMAVKERQSNTDEMFEPLKQTIELLKTYEQELPETVFKQLEELPEKWNN

IKKVAITVKQQVAPLQANEVTLLRQRCTAFDAEQQQFWEQFHKEAPFRFDSIHPHQMLDA

RHIEIQQMESTMASISESASLFEVNVPDYKQLRQCRKEVCQLKELWDTIGMVTSSIHAWE

TTPWRNINVEAMELECKQFARHIRNLDKEVRAWDAFTGLESTVWNTLSSLRAVAELQNPA

IRERHWRQLMQATGVSFTMDQDTTLAHLLQLQLHHYEDEVRGIVDKAAKEMGMEKTLKEL

QTTWAGMEFQYEPHPRTNVPLLCSDEDLIEVLEDNQVQLQNLVMSKYVAFFLEEVSGWQK

KLSTVDAVISIWFEVQRTWTHLESIFTGSEDIRAQLPQDSKRFEGIDIDFKELAYDAQKI

PNVVQTTNKPGLYEKLEDIQGRLCLCEKALAEYLDTKRLAFPRFYFLSSSDLLDILSNGT

APQQVQRHLSKLFDNMAKMRFQLDASGEPTKTSLGMYSKEEEYVAFSEPCDCSGQVEIWL

NHVLGHMKATVRHEMTEGVTAYEEKPREQWLFDHPAQVALTCTQIWWTTEVGMAFARLEE

GYESAMKDYYKKQVAQLKTLITMLIGQLSKGDRQKIMTICTIDVHARDVVAKMIAQKVDN

AQAFLWLSQLRHRWDDEVKHCFANICDAQFLYSYEYLGNTPRLVITPLTDRCYITLTQSL

HLTMSGAPAGPAGTGKTETTKDLGRALGILVYVFNCSEQMDYKSCGNIYKGLAQTGAWGC

FDEFNRISVEVLSVVAVQVKSIQDAIRDKKQWFSFLGEEISLNPSVGIFITMNPGYAGRT

ELPENLKSLFRPCAMVVPDFELICEIMLVAEGFIEAQSLARKFITLYQLCKELLSKQDHY

DWGLRAIKSVLVVAGSLKRGDPDRPEDQVLMRSLRDFNIPKIVTDDMPIFMGLIGDLFPA

LDVPRRRDPNFEALVRKAIVDLKLQAEDNFVLKVVQLEELLAVRHSVFVVGGAGTGKSQV

LRSLHKTYQIMKRRPVWTDLNPKAVTNDELFGIINPATGEWKDGLFSSIMRELANITHDG

PKWILLDGDIDPMWIESLNTVMDDNKVLTLASNERIPLNPTMKLLFEISHLRTATPATVS

RAGILYINPADLGWNPPVSSWIEKREIQTERANLTILFDKYLPTCLDTLRTRFKKIIPIP

EQSMVQMVCHLLECLLTTEDIPADCPKEIYEHYFVFAAIWAFGGAMVQDQLVDYRAEFSK

WWLTEFKTVKFPSQGTIFDYYIDPETKKFEPWSKLVPQFEFDPEMPLQACLVHTSETIRV

CYFMERLMARQRPVMLVGTAGTGKSVLVGAKLASLDPEAYLVKNLPFNYYTTSAMLQAVL

EKPLEKKAGRNYGPPGNKKLIYFIDDMNMPEVDAYGTVQPHTIIRQHLDYGHWYDRSKLS

LKEITNVQYVSCMNPTAGSFTINPRLQRHFSVFVLSFPGADALSSIYSIILTQHLKLGNF

PASLQKSIPPLIDLALAFHQKIATTFLPTGIKFHYIFNLRDFANIFQGILFSSVECVKST

WDLIRLYLHESNRVYRDKMVEEKDFDLFDKIQTEVLKKTFDDIEDPVEQTQSPNLYCHFA

NGIGEPKYMPVQSWELLTQTLVEALENHNEVNTVMDLVLFEDAMRHVCHINRILESPRGN

ALLVGVGGSGKQSLTRLAAFISSMDVFQITLRKGYQIQDFKMDLASLCLKAGVKNLNTVF

LMTDAQVADERFLVLINDLLASGEIPDLYSDDEVENIISNVRNEVKSQGLVDNRENCWKF

FIDRIRRQLKVTLCFSPVGNKLRVRSRKFPAIVNCTAIHWFHEWPQQALESVSLRFLQNT

EGIEPTVKQSISKFMAFVHTSVNQTSQSYLSNEQRYNYTTPKSFLEFIRLYQSLLHRHRK

ELKCKTERLENGLLKLHSTSAQVDDLKAKLAAQEVELKQKNEDADKLIQVVGVETDKVSR

EKAMADEEEQKVAVIMLEVKQKQKDCEEDLAKAEPALTAAQAALNTLNKTNLTELKSFGS

PPLAVSNVSAAVMVLMAPRGRVPKDRSWKAAKVTMAKVDGFLDSLINFNKENIHENCLKA

IRPYLQDPEFNPEFVATKSYAAAGLCSWVINIVRFYEVFCDVEPKRQALNKATADLTAAQ

EKLAAIKAKIAHLNENLAKLTARFEKATADKLKCQQEAEVTAVTISLANRLVGGLASENV

RWADAVQNFKQQERTLCGDILLITAFISYLGFFTKKYRQSLLDRTWRPYLSQLKTPIPVT

PALDPLRMLMDDADVAAWQNEGLPADRMSVENATILINCERWPLMVDPQLQGIKWIKNKY

GEDLRVTQIGQKGYLQIIEQALEAGAVVLIENLEESIDPVLGPLLGREVIKKGRFIKIGD

KECEYNPKFRLILHTKLANPHYQPELQAQATLINFTVTRDGLEDQLLAAVVSMERPDLEQ

LKSDLTKQQNGFKITLKTLEDSLLSRLSSASGNFLGETVLVENLEITKQTAAEVEKKVQE

AKVTEVKINEAREHYRPTAARASLLYFIMNDLSKIHPMYQFSLKAFSIVFQKAVERAAPD

ESLRERVANLIDSITFSVYQYTIRGLFECDKLTYLAQLTFQILLMNREVNAVELDFLLRS

PVQTGTASPVEFLSHQAWGAVKVLSSMEEFSNLDRDIEGSAKSWKKFVESECPEKEKLPQ

EWKNKTALQRLCMLRAMRPDRMTYALRDFVEEKLGSKYVVGRALDFATSFEESGPATPMF

FILSPGVDPLKDVESQGRKLGYTFNNQNFHNVSLGQGQEVVAEAALDLAAKKGHWVILQN

IHLVAKWLSTLEKKLEEHSENSHPEFRVFMSAEPAPSPEGHIIPQGILENSIKITNEPPT

GMHANLHKALDNFTQDTLEMCSRETEFKSILFALCYFHAVVAERRKFGPQGWNRSYPFNT

GDLTISVNVLYNFLEANAKVPYDDLRYLFGEIMYGGHITDDWDRRLCRTYLGEFIRPEML

EGELSLAPGFPLPGNMDYNGYHQYIDAELPPESPYLYGLHPNAEIGFLTQTSEKLFRTVL

ELQPRDSQARDGAGATREEKVKALLEEILERVTDEFNIPELMAKVEERTPYIVVAFQECG

RMNILTREIQRSLRELELGLKGELTMTSHMENLQNALYFDMVPESWARRAYPSTAGLAAW

FPDLLNRIKELEAWTGDFTMPSTVWLTGFFNPQSFLTAIMQSTARKNEWPLDQIALQCDM

TKKNREEFRSPPREGAYIHGLFMEGACWDTQAGIITEAKLKDLTPPMPVMFIKAIPADKQ

DCRSVYSCPVYKTSQRGPTYVWTFNLKTKENPSKWVLAGVALLLQI

>sp|Q5THJ4|VP13D_HUMAN 1985 EVVAFIQHF

MLEGLVAWVLNTYLGKYVNNLNTDQLSVALLKGAVELENLPLKKDALKELELPFEVKAGF

IGKVTLQIPFYRPHVDPWVISISSLHLIGAPEKIQDFNDEKEKLLERERKKALLQALEEK

WKNDRQQKGESYWYSVTASVVTRIVENIELKIQDVHLRFEDGVTNPSHPFAFGICIKNVS

MQNAVNEPVQKLMRKKQLDVAEFSIYWDVDCTLLGDLPQMELQEAMARSMESRSHHYVLE

PVFASALLKRNCSKKPLRSRHSPRIDCDIQLETIPLKLSQLQYRQIMEFLKELERKERQV

KFRRWKPKVAISKNCREWWYFALNANLYEIREQRKRCTWDFMLHRARDAVSYTDKYFNKL

KGGLLSTDDKEEMCRIEEEQSFEELKILRELVHDRFHKQEELAESLREPQFDSPGACPGA

PEPGGGSGMLQYLQSWFPGWGGWYGQQTPEGNVVEGLSAEQQEQWIPEEILGTEEFFDPT

ADASCMNTYTKRDHVFAKLNLQLQRGTVTLLHKEQGTPQMNESAFMQLEFSDVKLLAESL

PRRNSSLLSVRLGGLFLRDLATEGTMFPLLVFPNPQKEVGRVSQSFGLQTTSADRSDHYP

AADPDGPVFEMLYERNPAHSHFERRLNVSTRPLNIIYNPQAIKKVADFFYKGKVHTSGFG

YQSELELRVAEAARRQYNKLKMQTKAEIRQTLDRLLVGDFIEESKRWTVRLDISAPQVIF

PDDFKFKNPVLVVVDLGRMLLTNTQDNSRRKSRDGSASEETQFSDDEYKTPLATPPNTPP

PESSSSNGEKTPPFSGVEFSEEQLQAHLMSTKMYERYSLSFMDLQIMVGRVKDNWKHVQD

IDVGPTHVVEKFNVHLQLERRLIYTSDPKYPGAVLSGNLPDLKIHINEDKISALKNCFAL

LTTPEMKTSDTQIKEKIFPQEEQRGSLQDSVMNLTQSIVLLEQHTREVLVESQLLLAEFK

VNCMQLGVESNGRYISVLKVFGTNAHFVKRPYDAEVSLTVHGLLLVDTMQTYGADFDLLM

ASHKNLSFDIPTGSLRDSRAQSPVSGPNVAHLTDGATLNDRSATSVSLDKILTKEQESLI

KLEYQFVSSECPSMNLDSTLQVISLQVNNLDIILNPETIVELIGFLQKSFPKEKDDLSPQ

PLMTDFERSFREQGTYQSTYEQNTEVAVEIHRLNLLLLRTVGMANREKYGRKIATASIGG

TKVNVSMGSTFDMNGSLGCLQLMDLTQDNVKNQYVVSIGNSVGYENIISDIGYFESVFVR

MEDAALTEALSFTFVERSKQECFLNLKMASLHYNHSAKFLKELTLSMDELEENFRGMLKS

AATKVTTVLATKTAEYSEMVSLFETPRKTREPFILEENEIYGFDLASSHLDTVKLILNIN

IESPVVSIPRKPGSPELLVGHLGQIFIQNFVAGDDESRSDRLQVEIKDIKLYSLNCTQLA

GREAVGSEGSRMFCPPSGSGSANSQEEAHFTRHDFFESLHRGQAFHILNNTTIQFKLEKI

PIERESELTFSLSPDDLGTSSIMKIEGKFVNPVQVVLAKHVYEQVLQTLDNLVYSEDLNK

YPASATSSPCPDSPLPPLSTCGESSVERKENGLFSHSSLSNTSQKSLSVKEVKSFTQIQA

TFCISELQVQLSGDLTLGAQGLVSLKFQDFEVEFSKDHPQTLSIQIALHSLLMEDLLEKN

PDSKYKNLMVSRGAPKPSSLAQKEYLSQSCPSVSNVEYPDMPRSLPSHMEEAPNVFQLYQ

RPTSASRKKQKEVQDKDYPLTPPPSPTVDEPKILVGKSKFDDSLVHINIFLVDKKHPEFS

SSYNRVNRSIDVDFNCLDVLITLQTWVVILDFFGIGSTADNHAMRLPPEGILHNVKLEPH

ASMESGLQDPVNTKLDLKVHSLSLVLNKTTSELAKANVSKLVAHLEMIEGDLALQGSIGS

LSLSDLTCHGEFYRERFTTSGEEALIFQTFKYGRPDPLLRREHDIRVSLRMASVQYVHTQ

RFQAEVVAFIQHFTQLQDVLGRQRAAIEGQTVRDQAQRCSRVLLDIEAGAPVLLIPESSR

SNNLIVANLGKLKVKNKFLFAGFPGTFSLQDKESVPSASPTGIPKHSLRKTTSTEEPRGT

HSQGQFTMPLAGMSLGSLKSEFVPSTSTKQQGPQPTLSVGQESSSPEDHVCLLDCVVVDL

QDMDIFAAERHPREYSKAPEDSSGDLIFPSYFVRQTGGSLLTEPCRLKLQVERNLDKEIS

HTVPDISIHGNLSSVHCSLDLYKYKLIRGLLENNLGEPIEEFMRPYDLQDPRIHTVLSGE

VYTCMCFLIDMVNVSLELKDPKRKEGAGSLARFDFKKCKLLYESFSNQTKSINLVSHSMM

AFDTRYAGQKTSPGMTNVFSCIFQPAKNSSTTQGSIQIELHFRSTKDSSCFTVVLNNLRV

FLIFDWLLLVHDFLHTPSDIKKQNHVTPSRHRNSSSESAIVPKTVKSGVVTKRSSLPVSN

ERHLEVKVNVTGTEFVVIEDVSCFDTNAIILKGTTVLTYKPRFVDRPFSGSLFGIEVFSC

RLGNEHDTALSIVDPVQIQMELVGNSSYQNSSGLMDAFNSEDFPPVLEIQLQALDIRLSY

NDVQLFLAIAKSIPEQANAAVPDSVALESDSVGTYLPGASRVGEEIREGTRHTLDPVLEL

QLARLQELGFSMDDCRKALLACQGQLKKAASWLFKNAEPLKSLSLASTSRDSPGAVAAPL

ISGVEIKAESVCICFIDDCMDCDVPLAELTFSRLNFLQRVRTSPEGYAHFTLSGDYYNRA

LSGWEPFIEPWPCSVSWQQQAASRLHPPRLKLEAKAKPRLDINITSVLIDQYVSTKESWM

ADYCKDDKDIESAKSEDWMGSSVDPPCFGQSLPLVYLRTRSTASLTNLEHQIYAREVKTP

KRRQPFVPFALRNHTGCTLWFATLTTTPTRAALSHSGSPGVVPEGNGTFLDDTHNVSEWR

EVLTGEEIPFEFEARGKLRHRHTHDLRIHQLQVRVNGWEQVSPVSVDKVGTFFRYAAPDK

NSSSSTIGSPSSRTNIIHPQVYFSSLPPVRVVFAVTMEGSARKVITVRSALIVRNRLETP

MELRLDSPSAPDKPVVLPAIMPGDSFAVPLHLTSWRLQARPKGLGVFFCKAPIHWTNVVK

TAEISSSKRECHSMDTEKSRFFRFCVAIKKENYPDYMPSNIFSDSAKQIFRQPGHTIYLL

PTVVICNLLPCELDFYVKGMPINGTLKPGKEAALHTADTSQNIELGVSLENFPLCKELLI

PPGTQNYMVRMRLYDVNRRQLNLTIRIVCRAEGSLKIFISAPYWLINKTGLPLIFRQDNA

KTDAAGQFEEHELARSLSPLLFCYADKEQPNLCTMRIGRGIHPEGMPGWCQGFSLDGGSG

VRALKVIQQGNRPGLIYNIGIDVKKGRGRYIDTCMVIFAPRYLLDNKSSHKLAFAQREFA

RGQGTANPEGYISTLPGSSVVFHWPRNDYDQLLCVRLMDVPNCIWSGGFEVNKNNSFHIN

MRDTLGKCFFLRVEITLRGATYRISFSDTDQLPPPFRIDNFSKVPVVFTQHGVAEPRLRT

EVKPMTSLDYAWDEPTLPPFITLTVKGAGSSEINCNMNDFQDNRQLYYENFIYIAATYTF

SGLQEGTGRPVASNKAITCAELVLDVSPKTQRVILKKKEPGKRSQLWRMTGTGMLAHEGS

SVPHNPNKPSAARSTEGSAILDIAGLAAVTDNRYEPLMLRKPDRRRSTTQTWSFREGKLT

CGLHGLVVQAKGGLSGLFDGAEVVLGPDTSMELLGPVPPEQQFINQKMRPGSGMLSIRVI

PDGPTRALQITDFCHRKSSRSYEVDELPVTEQELQKLKNPDTEQELEVLVRLEGGIGLSL

INKVPEELVFASLTGINVHYTQLATSHMLELSIQDVQVDNQLIGTTQPFMLYVTPLSNEN

EVIETGPAVQVNAVKFPSKSALTNIYKHLMITAQRFTVQIEEKLLLKLLSFFGYDQAESE

VEKYDENLHEKTAEQGGTPIRYYFENLKISIPQIKLSVFTSNKLPLDLKALKSTLGFPLI

RFEDAVINLDPFTRVHPYETKEFIINDILKHFQEELLSQAARILGSVDFLGNPMGLLNDV

SEGVTGLIKYGNVGGLIRNVTHGVSNSAAKFAGTLSDGLGKTMDNRHQSEREYIRYHAAT

SGEHLVAGIHGLAHGIIGGLTSVITSTVEGVKTEGGVSGFISGLGKGLVGTVTKPVAGAL

DFASETAQAVRDTATLSGPRTQAQRVRKPRCCTGPQGLLPRYSESQAEGQEQLFKLTDNI

QDEFFIAVENIDSYCVLISSKAVYFLKSGDYVDREAIFLEVKYDDLYHCLVSKDHGKVYV

QVTKKAVSTSSGVSIPGPSHQKPMVHVKSEVLAVKLSQEINYAKSLYYEQQLMLRLSENR

EQLELDS

>sp|P78527|PRKDC_HUMAN 2320 AEVLGLILRY

MAGSGAGVRCSLLRLQETLSAADRCGAALAGHQLIRGLGQECVLSSSPAVLALQTSLVFS

RDFGLLVFVRKSLNSIEFRECREEILKFLCIFLEKMGQKIAPYSVEIKNTCTSVYTKDRA

AKCKIPALDLLIKLLQTFRSSRLMDEFKIGELFSKFYGELALKKKIPDTVLEKVYELLGL

LGEVHPSEMINNAENLFRAFLGELKTQMTSAVREPKLPVLAGCLKGLSSLLCNFTKSMEE

DPQTSREIFNFVLKAIRPQIDLKRYAVPSAGLRLFALHASQFSTCLLDNYVSLFEVLLKW

CAHTNVELKKAALSALESFLKQVSNMVAKNAEMHKNKLQYFMEQFYGIIRNVDSNNKELS

IAIRGYGLFAGPCKVINAKDVDFMYVELIQRCKQMFLTQTDTGDDRVYQMPSFLQSVASV

LLYLDTVPEVYTPVLEHLVVMQIDSFPQYSPKMQLVCCRAIVKVFLALAAKGPVLRNCIS

TVVHQGLIRICSKPVVLPKGPESESEDHRASGEVRTGKWKVPTYKDYVDLFRHLLSSDQM

MDSILADEAFFSVNSSSESLNHLLYDEFVKSVLKIVEKLDLTLEIQTVGEQENGDEAPGV

WMIPTSDPAANLHPAKPKDFSAFINLVEFCREILPEKQAEFFEPWVYSFSYELILQSTRL

PLISGFYKLLSITVRNAKKIKYFEGVSPKSLKHSPEDPEKYSCFALFVKFGKEVAVKMKQ

YKDELLASCLTFLLSLPHNIIELDVRAYVPALQMAFKLGLSYTPLAEVGLNALEEWSIYI

DRHVMQPYYKDILPCLDGYLKTSALSDETKNNWEVSALSRAAQKGFNKVVLKHLKKTKNL

SSNEAISLEEIRIRVVQMLGSLGGQINKNLLTVTSSDEMMKSYVAWDREKRLSFAVPFRE

MKPVIFLDVFLPRVTELALTASDRQTKVAACELLHSMVMFMLGKATQMPEGGQGAPPMYQ

LYKRTFPVLLRLACDVDQVTRQLYEPLVMQLIHWFTNNKKFESQDTVALLEAILDGIVDP

VDSTLRDFCGRCIREFLKWSIKQITPQQQEKSPVNTKSLFKRLYSLALHPNAFKRLGASL

AFNNIYREFREEESLVEQFVFEALVIYMESLALAHADEKSLGTIQQCCDAIDHLCRIIEK

KHVSLNKAKKRRLPRGFPPSASLCLLDLVKWLLAHCGRPQTECRHKSIELFYKFVPLLPG

NRSPNLWLKDVLKEEGVSFLINTFEGGGCGQPSGILAQPTLLYLRGPFSLQATLCWLDLL

LAALECYNTFIGERTVGALQVLGTEAQSSLLKAVAFFLESIAMHDIIAAEKCFGTGAAGN

RTSPQEGERYNYSKCTVVVRIMEFTTTLLNTSPEGWKLLKKDLCNTHLMRVLVQTLCEPA

SIGFNIGDVQVMAHLPDVCVNLMKALKMSPYKDILETHLREKITAQSIEELCAVNLYGPD

AQVDRSRLAAVVSACKQLHRAGLLHNILPSQSTDLHHSVGTELLSLVYKGIAPGDERQCL

PSLDLSCKQLASGLLELAFAFGGLCERLVSLLLNPAVLSTASLGSSQGSVIHFSHGEYFY

SLFSETINTELLKNLDLAVLELMQSSVDNTKMVSAVLNGMLDQSFRERANQKHQGLKLAT

TILQHWKKCDSWWAKDSPLETKMAVLALLAKILQIDSSVSFNTSHGSFPEVFTTYISLLA

DTKLDLHLKGQAVTLLPFFTSLTGGSLEELRRVLEQLIVAHFPMQSREFPPGTPRFNNYV

DCMKKFLDALELSQSPMLLELMTEVLCREQQHVMEELFQSSFRRIARRGSCVTQVGLLES

VYEMFRKDDPRLSFTRQSFVDRSLLTLLWHCSLDALREFFSTIVVDAIDVLKSRFTKLNE

STFDTQITKKMGYYKILDVMYSRLPKDDVHAKESKINQVFHGSCITEGNELTKTLIKLCY

DAFTENMAGENQLLERRRLYHCAAYNCAISVICCVFNELKFYQGFLFSEKPEKNLLIFEN

LIDLKRRYNFPVEVEVPMERKKKYIEIRKEAREAANGDSDGPSYMSSLSYLADSTLSEEM

SQFDFSTGVQSYSYSSQDPRPATGRFRRREQRDPTVHDDVLELEMDELNRHECMAPLTAL

VKHMHRSLGPPQGEEDSVPRDLPSWMKFLHGKLGNPIVPLNIRLFLAKLVINTEEVFRPY

AKHWLSPLLQLAASENNGGEGIHYMVVEIVATILSWTGLATPTGVPKDEVLANRLLNFLM

KHVFHPKRAVFRHNLEIIKTLVECWKDCLSIPYRLIFEKFSGKDPNSKDNSVGIQLLGIV

MANDLPPYDPQCGIQSSEYFQALVNNMSFVRYKEVYAAAAEVLGLILRYVMERKNILEES

LCELVAKQLKQHQNTMEDKFIVCLNKVTKSFPPLADRFMNAVFFLLPKFHGVLKTLCLEV

VLCRVEGMTELYFQLKSKDFVQVMRHRDDERQKVCLDIIYKMMPKLKPVELRELLNPVVE

FVSHPSTTCREQMYNILMWIHDNYRDPESETDNDSQEIFKLAKDVLIQGLIDENPGLQLI

IRNFWSHETRLPSNTLDRLLALNSLYSPKIEVHFLSLATNFLLEMTSMSPDYPNPMFEHP

LSECEFQEYTIDSDWRFRSTVLTPMFVETQASQGTLQTRTQEGSLSARWPVAGQIRATQQ

QHDFTLTQTADGRSSFDWLTGSSTDPLVDHTSPSSDSLLFAHKRSERLQRAPLKSVGPDF

GKKRLGLPGDEVDNKVKGAAGRTDLLRLRRRFMRDQEKLSLMYARKGVAEQKREKEIKSE

LKMKQDAQVVLYRSYRHGDLPDIQIKHSSLITPLQAVAQRDPIIAKQLFSSLFSGILKEM

DKFKTLSEKNNITQKLLQDFNRFLNTTFSFFPPFVSCIQDISCQHAALLSLDPAAVSAGC

LASLQQPVGIRLLEEALLRLLPAELPAKRVRGKARLPPDVLRWVELAKLYRSIGEYDVLR

GIFTSEIGTKQITQSALLAEARSDYSEAAKQYDEALNKQDWVDGEPTEAEKDFWELASLD

CYNHLAEWKSLEYCSTASIDSENPPDLNKIWSEPFYQETYLPYMIRSKLKLLLQGEADQS

LLTFIDKAMHGELQKAILELHYSQELSLLYLLQDDVDRAKYYIQNGIQSFMQNYSSIDVL

LHQSRLTKLQSVQALTEIQEFISFISKQGNLSSQVPLKRLLNTWTNRYPDAKMDPMNIWD

DIITNRCFFLSKIEEKLTPLPEDNSMNVDQDGDPSDRMEVQEQEEDISSLIRSCKFSMKM

KMIDSARKQNNFSLAMKLLKELHKESKTRDDWLVSWVQSYCRLSHCRSRSQGCSEQVLTV

LKTVSLLDENNVSSYLSKNILAFRDQNILLGTTYRIIANALSSEPACLAEIEEDKARRIL

ELSGSSSEDSEKVIAGLYQRAFQHLSEAVQAAEEEAQPPSWSCGPAAGVIDAYMTLADFC

DQQLRKEEENASVIDSAELQAYPALVVEKMLKALKLNSNEARLKFPRLLQIIERYPEETL

SLMTKEISSVPCWQFISWISHMVALLDKDQAVAVQHSVEEITDNYPQAIVYPFIISSESY

SFKDTSTGHKNKEFVARIKSKLDQGGVIQDFINALDQLSNPELLFKDWSNDVRAELAKTP

VNKKNIEKMYERMYAALGDPKAPGLGAFRRKFIQTFGKEFDKHFGKGGSKLLRMKLSDFN

DITNMLLLKMNKDSKPPGNLKECSPWMSDFKVEFLRNELEIPGQYDGRGKPLPEYHVRIA

GFDERVTVMASLRRPKRIIIRGHDEREHPFLVKGGEDLRQDQRVEQLFQVMNGILAQDSA

CSQRALQLRTYSVVPMTSRLGLIEWLENTVTLKDLLLNTMSQEEKAAYLSDPRAPPCEYK

DWLTKMSGKHDVGAYMLMYKGANRTETVTSFRKRESKVPADLLKRAFVRMSTSPEAFLAL

RSHFASSHALICISHWILGIGDRHLNNFMVAMETGGVIGIDFGHAFGSATQFLPVPELMP

FRLTRQFINLMLPMKETGLMYSIMVHALRAFRSDPGLLTNTMDVFVKEPSFDWKNFEQKM

LKKGGSWIQEINVAEKNWYPRQKICYAKRKLAGANPAVITCDELLLGHEKAPAFRDYVAV

ARGSKDHNIRAQEPESGLSEETQVKCLMDQATDPNILGRTWEGWEPWM

>sp|P78527|PRKDC_HUMAN 4111 ATDPNILGR

MAGSGAGVRCSLLRLQETLSAADRCGAALAGHQLIRGLGQECVLSSSPAVLALQTSLVFS

RDFGLLVFVRKSLNSIEFRECREEILKFLCIFLEKMGQKIAPYSVEIKNTCTSVYTKDRA

AKCKIPALDLLIKLLQTFRSSRLMDEFKIGELFSKFYGELALKKKIPDTVLEKVYELLGL

LGEVHPSEMINNAENLFRAFLGELKTQMTSAVREPKLPVLAGCLKGLSSLLCNFTKSMEE

DPQTSREIFNFVLKAIRPQIDLKRYAVPSAGLRLFALHASQFSTCLLDNYVSLFEVLLKW

CAHTNVELKKAALSALESFLKQVSNMVAKNAEMHKNKLQYFMEQFYGIIRNVDSNNKELS

IAIRGYGLFAGPCKVINAKDVDFMYVELIQRCKQMFLTQTDTGDDRVYQMPSFLQSVASV

LLYLDTVPEVYTPVLEHLVVMQIDSFPQYSPKMQLVCCRAIVKVFLALAAKGPVLRNCIS

TVVHQGLIRICSKPVVLPKGPESESEDHRASGEVRTGKWKVPTYKDYVDLFRHLLSSDQM

MDSILADEAFFSVNSSSESLNHLLYDEFVKSVLKIVEKLDLTLEIQTVGEQENGDEAPGV

WMIPTSDPAANLHPAKPKDFSAFINLVEFCREILPEKQAEFFEPWVYSFSYELILQSTRL

PLISGFYKLLSITVRNAKKIKYFEGVSPKSLKHSPEDPEKYSCFALFVKFGKEVAVKMKQ

YKDELLASCLTFLLSLPHNIIELDVRAYVPALQMAFKLGLSYTPLAEVGLNALEEWSIYI

DRHVMQPYYKDILPCLDGYLKTSALSDETKNNWEVSALSRAAQKGFNKVVLKHLKKTKNL

SSNEAISLEEIRIRVVQMLGSLGGQINKNLLTVTSSDEMMKSYVAWDREKRLSFAVPFRE

MKPVIFLDVFLPRVTELALTASDRQTKVAACELLHSMVMFMLGKATQMPEGGQGAPPMYQ

LYKRTFPVLLRLACDVDQVTRQLYEPLVMQLIHWFTNNKKFESQDTVALLEAILDGIVDP

VDSTLRDFCGRCIREFLKWSIKQITPQQQEKSPVNTKSLFKRLYSLALHPNAFKRLGASL

AFNNIYREFREEESLVEQFVFEALVIYMESLALAHADEKSLGTIQQCCDAIDHLCRIIEK

KHVSLNKAKKRRLPRGFPPSASLCLLDLVKWLLAHCGRPQTECRHKSIELFYKFVPLLPG

NRSPNLWLKDVLKEEGVSFLINTFEGGGCGQPSGILAQPTLLYLRGPFSLQATLCWLDLL

LAALECYNTFIGERTVGALQVLGTEAQSSLLKAVAFFLESIAMHDIIAAEKCFGTGAAGN

RTSPQEGERYNYSKCTVVVRIMEFTTTLLNTSPEGWKLLKKDLCNTHLMRVLVQTLCEPA

SIGFNIGDVQVMAHLPDVCVNLMKALKMSPYKDILETHLREKITAQSIEELCAVNLYGPD

AQVDRSRLAAVVSACKQLHRAGLLHNILPSQSTDLHHSVGTELLSLVYKGIAPGDERQCL

PSLDLSCKQLASGLLELAFAFGGLCERLVSLLLNPAVLSTASLGSSQGSVIHFSHGEYFY

SLFSETINTELLKNLDLAVLELMQSSVDNTKMVSAVLNGMLDQSFRERANQKHQGLKLAT

TILQHWKKCDSWWAKDSPLETKMAVLALLAKILQIDSSVSFNTSHGSFPEVFTTYISLLA

DTKLDLHLKGQAVTLLPFFTSLTGGSLEELRRVLEQLIVAHFPMQSREFPPGTPRFNNYV

DCMKKFLDALELSQSPMLLELMTEVLCREQQHVMEELFQSSFRRIARRGSCVTQVGLLES

VYEMFRKDDPRLSFTRQSFVDRSLLTLLWHCSLDALREFFSTIVVDAIDVLKSRFTKLNE

STFDTQITKKMGYYKILDVMYSRLPKDDVHAKESKINQVFHGSCITEGNELTKTLIKLCY

DAFTENMAGENQLLERRRLYHCAAYNCAISVICCVFNELKFYQGFLFSEKPEKNLLIFEN

LIDLKRRYNFPVEVEVPMERKKKYIEIRKEAREAANGDSDGPSYMSSLSYLADSTLSEEM

SQFDFSTGVQSYSYSSQDPRPATGRFRRREQRDPTVHDDVLELEMDELNRHECMAPLTAL

VKHMHRSLGPPQGEEDSVPRDLPSWMKFLHGKLGNPIVPLNIRLFLAKLVINTEEVFRPY

AKHWLSPLLQLAASENNGGEGIHYMVVEIVATILSWTGLATPTGVPKDEVLANRLLNFLM

KHVFHPKRAVFRHNLEIIKTLVECWKDCLSIPYRLIFEKFSGKDPNSKDNSVGIQLLGIV

MANDLPPYDPQCGIQSSEYFQALVNNMSFVRYKEVYAAAAEVLGLILRYVMERKNILEES

LCELVAKQLKQHQNTMEDKFIVCLNKVTKSFPPLADRFMNAVFFLLPKFHGVLKTLCLEV

VLCRVEGMTELYFQLKSKDFVQVMRHRDDERQKVCLDIIYKMMPKLKPVELRELLNPVVE

FVSHPSTTCREQMYNILMWIHDNYRDPESETDNDSQEIFKLAKDVLIQGLIDENPGLQLI

IRNFWSHETRLPSNTLDRLLALNSLYSPKIEVHFLSLATNFLLEMTSMSPDYPNPMFEHP

LSECEFQEYTIDSDWRFRSTVLTPMFVETQASQGTLQTRTQEGSLSARWPVAGQIRATQQ

QHDFTLTQTADGRSSFDWLTGSSTDPLVDHTSPSSDSLLFAHKRSERLQRAPLKSVGPDF

GKKRLGLPGDEVDNKVKGAAGRTDLLRLRRRFMRDQEKLSLMYARKGVAEQKREKEIKSE

LKMKQDAQVVLYRSYRHGDLPDIQIKHSSLITPLQAVAQRDPIIAKQLFSSLFSGILKEM

DKFKTLSEKNNITQKLLQDFNRFLNTTFSFFPPFVSCIQDISCQHAALLSLDPAAVSAGC

LASLQQPVGIRLLEEALLRLLPAELPAKRVRGKARLPPDVLRWVELAKLYRSIGEYDVLR

GIFTSEIGTKQITQSALLAEARSDYSEAAKQYDEALNKQDWVDGEPTEAEKDFWELASLD

CYNHLAEWKSLEYCSTASIDSENPPDLNKIWSEPFYQETYLPYMIRSKLKLLLQGEADQS

LLTFIDKAMHGELQKAILELHYSQELSLLYLLQDDVDRAKYYIQNGIQSFMQNYSSIDVL

LHQSRLTKLQSVQALTEIQEFISFISKQGNLSSQVPLKRLLNTWTNRYPDAKMDPMNIWD

DIITNRCFFLSKIEEKLTPLPEDNSMNVDQDGDPSDRMEVQEQEEDISSLIRSCKFSMKM

KMIDSARKQNNFSLAMKLLKELHKESKTRDDWLVSWVQSYCRLSHCRSRSQGCSEQVLTV

LKTVSLLDENNVSSYLSKNILAFRDQNILLGTTYRIIANALSSEPACLAEIEEDKARRIL

ELSGSSSEDSEKVIAGLYQRAFQHLSEAVQAAEEEAQPPSWSCGPAAGVIDAYMTLADFC

DQQLRKEEENASVIDSAELQAYPALVVEKMLKALKLNSNEARLKFPRLLQIIERYPEETL

SLMTKEISSVPCWQFISWISHMVALLDKDQAVAVQHSVEEITDNYPQAIVYPFIISSESY

SFKDTSTGHKNKEFVARIKSKLDQGGVIQDFINALDQLSNPELLFKDWSNDVRAELAKTP

VNKKNIEKMYERMYAALGDPKAPGLGAFRRKFIQTFGKEFDKHFGKGGSKLLRMKLSDFN

DITNMLLLKMNKDSKPPGNLKECSPWMSDFKVEFLRNELEIPGQYDGRGKPLPEYHVRIA

GFDERVTVMASLRRPKRIIIRGHDEREHPFLVKGGEDLRQDQRVEQLFQVMNGILAQDSA

CSQRALQLRTYSVVPMTSRLGLIEWLENTVTLKDLLLNTMSQEEKAAYLSDPRAPPCEYK

DWLTKMSGKHDVGAYMLMYKGANRTETVTSFRKRESKVPADLLKRAFVRMSTSPEAFLAL

RSHFASSHALICISHWILGIGDRHLNNFMVAMETGGVIGIDFGHAFGSATQFLPVPELMP

FRLTRQFINLMLPMKETGLMYSIMVHALRAFRSDPGLLTNTMDVFVKEPSFDWKNFEQKM

LKKGGSWIQEINVAEKNWYPRQKICYAKRKLAGANPAVITCDELLLGHEKAPAFRDYVAV

ARGSKDHNIRAQEPESGLSEETQVKCLMDQATDPNILGRTWEGWEPWM

>sp|P78527|PRKDC_HUMAN 145 DEFKIGELF

MAGSGAGVRCSLLRLQETLSAADRCGAALAGHQLIRGLGQECVLSSSPAVLALQTSLVFS

RDFGLLVFVRKSLNSIEFRECREEILKFLCIFLEKMGQKIAPYSVEIKNTCTSVYTKDRA

AKCKIPALDLLIKLLQTFRSSRLMDEFKIGELFSKFYGELALKKKIPDTVLEKVYELLGL

LGEVHPSEMINNAENLFRAFLGELKTQMTSAVREPKLPVLAGCLKGLSSLLCNFTKSMEE

DPQTSREIFNFVLKAIRPQIDLKRYAVPSAGLRLFALHASQFSTCLLDNYVSLFEVLLKW

CAHTNVELKKAALSALESFLKQVSNMVAKNAEMHKNKLQYFMEQFYGIIRNVDSNNKELS

IAIRGYGLFAGPCKVINAKDVDFMYVELIQRCKQMFLTQTDTGDDRVYQMPSFLQSVASV

LLYLDTVPEVYTPVLEHLVVMQIDSFPQYSPKMQLVCCRAIVKVFLALAAKGPVLRNCIS

TVVHQGLIRICSKPVVLPKGPESESEDHRASGEVRTGKWKVPTYKDYVDLFRHLLSSDQM

MDSILADEAFFSVNSSSESLNHLLYDEFVKSVLKIVEKLDLTLEIQTVGEQENGDEAPGV

WMIPTSDPAANLHPAKPKDFSAFINLVEFCREILPEKQAEFFEPWVYSFSYELILQSTRL

PLISGFYKLLSITVRNAKKIKYFEGVSPKSLKHSPEDPEKYSCFALFVKFGKEVAVKMKQ

YKDELLASCLTFLLSLPHNIIELDVRAYVPALQMAFKLGLSYTPLAEVGLNALEEWSIYI

DRHVMQPYYKDILPCLDGYLKTSALSDETKNNWEVSALSRAAQKGFNKVVLKHLKKTKNL

SSNEAISLEEIRIRVVQMLGSLGGQINKNLLTVTSSDEMMKSYVAWDREKRLSFAVPFRE

MKPVIFLDVFLPRVTELALTASDRQTKVAACELLHSMVMFMLGKATQMPEGGQGAPPMYQ

LYKRTFPVLLRLACDVDQVTRQLYEPLVMQLIHWFTNNKKFESQDTVALLEAILDGIVDP

VDSTLRDFCGRCIREFLKWSIKQITPQQQEKSPVNTKSLFKRLYSLALHPNAFKRLGASL

AFNNIYREFREEESLVEQFVFEALVIYMESLALAHADEKSLGTIQQCCDAIDHLCRIIEK

KHVSLNKAKKRRLPRGFPPSASLCLLDLVKWLLAHCGRPQTECRHKSIELFYKFVPLLPG

NRSPNLWLKDVLKEEGVSFLINTFEGGGCGQPSGILAQPTLLYLRGPFSLQATLCWLDLL

LAALECYNTFIGERTVGALQVLGTEAQSSLLKAVAFFLESIAMHDIIAAEKCFGTGAAGN

RTSPQEGERYNYSKCTVVVRIMEFTTTLLNTSPEGWKLLKKDLCNTHLMRVLVQTLCEPA

SIGFNIGDVQVMAHLPDVCVNLMKALKMSPYKDILETHLREKITAQSIEELCAVNLYGPD

AQVDRSRLAAVVSACKQLHRAGLLHNILPSQSTDLHHSVGTELLSLVYKGIAPGDERQCL

PSLDLSCKQLASGLLELAFAFGGLCERLVSLLLNPAVLSTASLGSSQGSVIHFSHGEYFY

SLFSETINTELLKNLDLAVLELMQSSVDNTKMVSAVLNGMLDQSFRERANQKHQGLKLAT

TILQHWKKCDSWWAKDSPLETKMAVLALLAKILQIDSSVSFNTSHGSFPEVFTTYISLLA

DTKLDLHLKGQAVTLLPFFTSLTGGSLEELRRVLEQLIVAHFPMQSREFPPGTPRFNNYV

DCMKKFLDALELSQSPMLLELMTEVLCREQQHVMEELFQSSFRRIARRGSCVTQVGLLES

VYEMFRKDDPRLSFTRQSFVDRSLLTLLWHCSLDALREFFSTIVVDAIDVLKSRFTKLNE

STFDTQITKKMGYYKILDVMYSRLPKDDVHAKESKINQVFHGSCITEGNELTKTLIKLCY

DAFTENMAGENQLLERRRLYHCAAYNCAISVICCVFNELKFYQGFLFSEKPEKNLLIFEN

LIDLKRRYNFPVEVEVPMERKKKYIEIRKEAREAANGDSDGPSYMSSLSYLADSTLSEEM

SQFDFSTGVQSYSYSSQDPRPATGRFRRREQRDPTVHDDVLELEMDELNRHECMAPLTAL

VKHMHRSLGPPQGEEDSVPRDLPSWMKFLHGKLGNPIVPLNIRLFLAKLVINTEEVFRPY

AKHWLSPLLQLAASENNGGEGIHYMVVEIVATILSWTGLATPTGVPKDEVLANRLLNFLM

KHVFHPKRAVFRHNLEIIKTLVECWKDCLSIPYRLIFEKFSGKDPNSKDNSVGIQLLGIV

MANDLPPYDPQCGIQSSEYFQALVNNMSFVRYKEVYAAAAEVLGLILRYVMERKNILEES

LCELVAKQLKQHQNTMEDKFIVCLNKVTKSFPPLADRFMNAVFFLLPKFHGVLKTLCLEV

VLCRVEGMTELYFQLKSKDFVQVMRHRDDERQKVCLDIIYKMMPKLKPVELRELLNPVVE

FVSHPSTTCREQMYNILMWIHDNYRDPESETDNDSQEIFKLAKDVLIQGLIDENPGLQLI

IRNFWSHETRLPSNTLDRLLALNSLYSPKIEVHFLSLATNFLLEMTSMSPDYPNPMFEHP

LSECEFQEYTIDSDWRFRSTVLTPMFVETQASQGTLQTRTQEGSLSARWPVAGQIRATQQ

QHDFTLTQTADGRSSFDWLTGSSTDPLVDHTSPSSDSLLFAHKRSERLQRAPLKSVGPDF

GKKRLGLPGDEVDNKVKGAAGRTDLLRLRRRFMRDQEKLSLMYARKGVAEQKREKEIKSE

LKMKQDAQVVLYRSYRHGDLPDIQIKHSSLITPLQAVAQRDPIIAKQLFSSLFSGILKEM

DKFKTLSEKNNITQKLLQDFNRFLNTTFSFFPPFVSCIQDISCQHAALLSLDPAAVSAGC

LASLQQPVGIRLLEEALLRLLPAELPAKRVRGKARLPPDVLRWVELAKLYRSIGEYDVLR

GIFTSEIGTKQITQSALLAEARSDYSEAAKQYDEALNKQDWVDGEPTEAEKDFWELASLD

CYNHLAEWKSLEYCSTASIDSENPPDLNKIWSEPFYQETYLPYMIRSKLKLLLQGEADQS

LLTFIDKAMHGELQKAILELHYSQELSLLYLLQDDVDRAKYYIQNGIQSFMQNYSSIDVL

LHQSRLTKLQSVQALTEIQEFISFISKQGNLSSQVPLKRLLNTWTNRYPDAKMDPMNIWD

DIITNRCFFLSKIEEKLTPLPEDNSMNVDQDGDPSDRMEVQEQEEDISSLIRSCKFSMKM

KMIDSARKQNNFSLAMKLLKELHKESKTRDDWLVSWVQSYCRLSHCRSRSQGCSEQVLTV

LKTVSLLDENNVSSYLSKNILAFRDQNILLGTTYRIIANALSSEPACLAEIEEDKARRIL

ELSGSSSEDSEKVIAGLYQRAFQHLSEAVQAAEEEAQPPSWSCGPAAGVIDAYMTLADFC

DQQLRKEEENASVIDSAELQAYPALVVEKMLKALKLNSNEARLKFPRLLQIIERYPEETL

SLMTKEISSVPCWQFISWISHMVALLDKDQAVAVQHSVEEITDNYPQAIVYPFIISSESY

SFKDTSTGHKNKEFVARIKSKLDQGGVIQDFINALDQLSNPELLFKDWSNDVRAELAKTP

VNKKNIEKMYERMYAALGDPKAPGLGAFRRKFIQTFGKEFDKHFGKGGSKLLRMKLSDFN

DITNMLLLKMNKDSKPPGNLKECSPWMSDFKVEFLRNELEIPGQYDGRGKPLPEYHVRIA

GFDERVTVMASLRRPKRIIIRGHDEREHPFLVKGGEDLRQDQRVEQLFQVMNGILAQDSA

CSQRALQLRTYSVVPMTSRLGLIEWLENTVTLKDLLLNTMSQEEKAAYLSDPRAPPCEYK

DWLTKMSGKHDVGAYMLMYKGANRTETVTSFRKRESKVPADLLKRAFVRMSTSPEAFLAL

RSHFASSHALICISHWILGIGDRHLNNFMVAMETGGVIGIDFGHAFGSATQFLPVPELMP

FRLTRQFINLMLPMKETGLMYSIMVHALRAFRSDPGLLTNTMDVFVKEPSFDWKNFEQKM

LKKGGSWIQEINVAEKNWYPRQKICYAKRKLAGANPAVITCDELLLGHEKAPAFRDYVAV

ARGSKDHNIRAQEPESGLSEETQVKCLMDQATDPNILGRTWEGWEPWM

>sp|P78527|PRKDC_HUMAN 263 KRYAVPSAGL

MAGSGAGVRCSLLRLQETLSAADRCGAALAGHQLIRGLGQECVLSSSPAVLALQTSLVFS

RDFGLLVFVRKSLNSIEFRECREEILKFLCIFLEKMGQKIAPYSVEIKNTCTSVYTKDRA

AKCKIPALDLLIKLLQTFRSSRLMDEFKIGELFSKFYGELALKKKIPDTVLEKVYELLGL

LGEVHPSEMINNAENLFRAFLGELKTQMTSAVREPKLPVLAGCLKGLSSLLCNFTKSMEE

DPQTSREIFNFVLKAIRPQIDLKRYAVPSAGLRLFALHASQFSTCLLDNYVSLFEVLLKW

CAHTNVELKKAALSALESFLKQVSNMVAKNAEMHKNKLQYFMEQFYGIIRNVDSNNKELS

IAIRGYGLFAGPCKVINAKDVDFMYVELIQRCKQMFLTQTDTGDDRVYQMPSFLQSVASV

LLYLDTVPEVYTPVLEHLVVMQIDSFPQYSPKMQLVCCRAIVKVFLALAAKGPVLRNCIS

TVVHQGLIRICSKPVVLPKGPESESEDHRASGEVRTGKWKVPTYKDYVDLFRHLLSSDQM

MDSILADEAFFSVNSSSESLNHLLYDEFVKSVLKIVEKLDLTLEIQTVGEQENGDEAPGV

WMIPTSDPAANLHPAKPKDFSAFINLVEFCREILPEKQAEFFEPWVYSFSYELILQSTRL

PLISGFYKLLSITVRNAKKIKYFEGVSPKSLKHSPEDPEKYSCFALFVKFGKEVAVKMKQ

YKDELLASCLTFLLSLPHNIIELDVRAYVPALQMAFKLGLSYTPLAEVGLNALEEWSIYI

DRHVMQPYYKDILPCLDGYLKTSALSDETKNNWEVSALSRAAQKGFNKVVLKHLKKTKNL

SSNEAISLEEIRIRVVQMLGSLGGQINKNLLTVTSSDEMMKSYVAWDREKRLSFAVPFRE

MKPVIFLDVFLPRVTELALTASDRQTKVAACELLHSMVMFMLGKATQMPEGGQGAPPMYQ

LYKRTFPVLLRLACDVDQVTRQLYEPLVMQLIHWFTNNKKFESQDTVALLEAILDGIVDP

VDSTLRDFCGRCIREFLKWSIKQITPQQQEKSPVNTKSLFKRLYSLALHPNAFKRLGASL

AFNNIYREFREEESLVEQFVFEALVIYMESLALAHADEKSLGTIQQCCDAIDHLCRIIEK

KHVSLNKAKKRRLPRGFPPSASLCLLDLVKWLLAHCGRPQTECRHKSIELFYKFVPLLPG

NRSPNLWLKDVLKEEGVSFLINTFEGGGCGQPSGILAQPTLLYLRGPFSLQATLCWLDLL

LAALECYNTFIGERTVGALQVLGTEAQSSLLKAVAFFLESIAMHDIIAAEKCFGTGAAGN

RTSPQEGERYNYSKCTVVVRIMEFTTTLLNTSPEGWKLLKKDLCNTHLMRVLVQTLCEPA

SIGFNIGDVQVMAHLPDVCVNLMKALKMSPYKDILETHLREKITAQSIEELCAVNLYGPD

AQVDRSRLAAVVSACKQLHRAGLLHNILPSQSTDLHHSVGTELLSLVYKGIAPGDERQCL

PSLDLSCKQLASGLLELAFAFGGLCERLVSLLLNPAVLSTASLGSSQGSVIHFSHGEYFY

SLFSETINTELLKNLDLAVLELMQSSVDNTKMVSAVLNGMLDQSFRERANQKHQGLKLAT

TILQHWKKCDSWWAKDSPLETKMAVLALLAKILQIDSSVSFNTSHGSFPEVFTTYISLLA

DTKLDLHLKGQAVTLLPFFTSLTGGSLEELRRVLEQLIVAHFPMQSREFPPGTPRFNNYV

DCMKKFLDALELSQSPMLLELMTEVLCREQQHVMEELFQSSFRRIARRGSCVTQVGLLES

VYEMFRKDDPRLSFTRQSFVDRSLLTLLWHCSLDALREFFSTIVVDAIDVLKSRFTKLNE

STFDTQITKKMGYYKILDVMYSRLPKDDVHAKESKINQVFHGSCITEGNELTKTLIKLCY

DAFTENMAGENQLLERRRLYHCAAYNCAISVICCVFNELKFYQGFLFSEKPEKNLLIFEN

LIDLKRRYNFPVEVEVPMERKKKYIEIRKEAREAANGDSDGPSYMSSLSYLADSTLSEEM

SQFDFSTGVQSYSYSSQDPRPATGRFRRREQRDPTVHDDVLELEMDELNRHECMAPLTAL

VKHMHRSLGPPQGEEDSVPRDLPSWMKFLHGKLGNPIVPLNIRLFLAKLVINTEEVFRPY

AKHWLSPLLQLAASENNGGEGIHYMVVEIVATILSWTGLATPTGVPKDEVLANRLLNFLM

KHVFHPKRAVFRHNLEIIKTLVECWKDCLSIPYRLIFEKFSGKDPNSKDNSVGIQLLGIV

MANDLPPYDPQCGIQSSEYFQALVNNMSFVRYKEVYAAAAEVLGLILRYVMERKNILEES

LCELVAKQLKQHQNTMEDKFIVCLNKVTKSFPPLADRFMNAVFFLLPKFHGVLKTLCLEV

VLCRVEGMTELYFQLKSKDFVQVMRHRDDERQKVCLDIIYKMMPKLKPVELRELLNPVVE

FVSHPSTTCREQMYNILMWIHDNYRDPESETDNDSQEIFKLAKDVLIQGLIDENPGLQLI

IRNFWSHETRLPSNTLDRLLALNSLYSPKIEVHFLSLATNFLLEMTSMSPDYPNPMFEHP

LSECEFQEYTIDSDWRFRSTVLTPMFVETQASQGTLQTRTQEGSLSARWPVAGQIRATQQ

QHDFTLTQTADGRSSFDWLTGSSTDPLVDHTSPSSDSLLFAHKRSERLQRAPLKSVGPDF

GKKRLGLPGDEVDNKVKGAAGRTDLLRLRRRFMRDQEKLSLMYARKGVAEQKREKEIKSE

LKMKQDAQVVLYRSYRHGDLPDIQIKHSSLITPLQAVAQRDPIIAKQLFSSLFSGILKEM

DKFKTLSEKNNITQKLLQDFNRFLNTTFSFFPPFVSCIQDISCQHAALLSLDPAAVSAGC

LASLQQPVGIRLLEEALLRLLPAELPAKRVRGKARLPPDVLRWVELAKLYRSIGEYDVLR

GIFTSEIGTKQITQSALLAEARSDYSEAAKQYDEALNKQDWVDGEPTEAEKDFWELASLD

CYNHLAEWKSLEYCSTASIDSENPPDLNKIWSEPFYQETYLPYMIRSKLKLLLQGEADQS

LLTFIDKAMHGELQKAILELHYSQELSLLYLLQDDVDRAKYYIQNGIQSFMQNYSSIDVL

LHQSRLTKLQSVQALTEIQEFISFISKQGNLSSQVPLKRLLNTWTNRYPDAKMDPMNIWD

DIITNRCFFLSKIEEKLTPLPEDNSMNVDQDGDPSDRMEVQEQEEDISSLIRSCKFSMKM

KMIDSARKQNNFSLAMKLLKELHKESKTRDDWLVSWVQSYCRLSHCRSRSQGCSEQVLTV

LKTVSLLDENNVSSYLSKNILAFRDQNILLGTTYRIIANALSSEPACLAEIEEDKARRIL

ELSGSSSEDSEKVIAGLYQRAFQHLSEAVQAAEEEAQPPSWSCGPAAGVIDAYMTLADFC

DQQLRKEEENASVIDSAELQAYPALVVEKMLKALKLNSNEARLKFPRLLQIIERYPEETL

SLMTKEISSVPCWQFISWISHMVALLDKDQAVAVQHSVEEITDNYPQAIVYPFIISSESY

SFKDTSTGHKNKEFVARIKSKLDQGGVIQDFINALDQLSNPELLFKDWSNDVRAELAKTP

VNKKNIEKMYERMYAALGDPKAPGLGAFRRKFIQTFGKEFDKHFGKGGSKLLRMKLSDFN

DITNMLLLKMNKDSKPPGNLKECSPWMSDFKVEFLRNELEIPGQYDGRGKPLPEYHVRIA

GFDERVTVMASLRRPKRIIIRGHDEREHPFLVKGGEDLRQDQRVEQLFQVMNGILAQDSA

CSQRALQLRTYSVVPMTSRLGLIEWLENTVTLKDLLLNTMSQEEKAAYLSDPRAPPCEYK

DWLTKMSGKHDVGAYMLMYKGANRTETVTSFRKRESKVPADLLKRAFVRMSTSPEAFLAL

RSHFASSHALICISHWILGIGDRHLNNFMVAMETGGVIGIDFGHAFGSATQFLPVPELMP

FRLTRQFINLMLPMKETGLMYSIMVHALRAFRSDPGLLTNTMDVFVKEPSFDWKNFEQKM

LKKGGSWIQEINVAEKNWYPRQKICYAKRKLAGANPAVITCDELLLGHEKAPAFRDYVAV

ARGSKDHNIRAQEPESGLSEETQVKCLMDQATDPNILGRTWEGWEPWM

>sp|P78527|PRKDC_HUMAN 1957 NELKFYQGF

MAGSGAGVRCSLLRLQETLSAADRCGAALAGHQLIRGLGQECVLSSSPAVLALQTSLVFS

RDFGLLVFVRKSLNSIEFRECREEILKFLCIFLEKMGQKIAPYSVEIKNTCTSVYTKDRA

AKCKIPALDLLIKLLQTFRSSRLMDEFKIGELFSKFYGELALKKKIPDTVLEKVYELLGL

LGEVHPSEMINNAENLFRAFLGELKTQMTSAVREPKLPVLAGCLKGLSSLLCNFTKSMEE

DPQTSREIFNFVLKAIRPQIDLKRYAVPSAGLRLFALHASQFSTCLLDNYVSLFEVLLKW

CAHTNVELKKAALSALESFLKQVSNMVAKNAEMHKNKLQYFMEQFYGIIRNVDSNNKELS

IAIRGYGLFAGPCKVINAKDVDFMYVELIQRCKQMFLTQTDTGDDRVYQMPSFLQSVASV

LLYLDTVPEVYTPVLEHLVVMQIDSFPQYSPKMQLVCCRAIVKVFLALAAKGPVLRNCIS

TVVHQGLIRICSKPVVLPKGPESESEDHRASGEVRTGKWKVPTYKDYVDLFRHLLSSDQM

MDSILADEAFFSVNSSSESLNHLLYDEFVKSVLKIVEKLDLTLEIQTVGEQENGDEAPGV

WMIPTSDPAANLHPAKPKDFSAFINLVEFCREILPEKQAEFFEPWVYSFSYELILQSTRL

PLISGFYKLLSITVRNAKKIKYFEGVSPKSLKHSPEDPEKYSCFALFVKFGKEVAVKMKQ

YKDELLASCLTFLLSLPHNIIELDVRAYVPALQMAFKLGLSYTPLAEVGLNALEEWSIYI

DRHVMQPYYKDILPCLDGYLKTSALSDETKNNWEVSALSRAAQKGFNKVVLKHLKKTKNL

SSNEAISLEEIRIRVVQMLGSLGGQINKNLLTVTSSDEMMKSYVAWDREKRLSFAVPFRE

MKPVIFLDVFLPRVTELALTASDRQTKVAACELLHSMVMFMLGKATQMPEGGQGAPPMYQ

LYKRTFPVLLRLACDVDQVTRQLYEPLVMQLIHWFTNNKKFESQDTVALLEAILDGIVDP

VDSTLRDFCGRCIREFLKWSIKQITPQQQEKSPVNTKSLFKRLYSLALHPNAFKRLGASL

AFNNIYREFREEESLVEQFVFEALVIYMESLALAHADEKSLGTIQQCCDAIDHLCRIIEK

KHVSLNKAKKRRLPRGFPPSASLCLLDLVKWLLAHCGRPQTECRHKSIELFYKFVPLLPG

NRSPNLWLKDVLKEEGVSFLINTFEGGGCGQPSGILAQPTLLYLRGPFSLQATLCWLDLL

LAALECYNTFIGERTVGALQVLGTEAQSSLLKAVAFFLESIAMHDIIAAEKCFGTGAAGN

RTSPQEGERYNYSKCTVVVRIMEFTTTLLNTSPEGWKLLKKDLCNTHLMRVLVQTLCEPA

SIGFNIGDVQVMAHLPDVCVNLMKALKMSPYKDILETHLREKITAQSIEELCAVNLYGPD

AQVDRSRLAAVVSACKQLHRAGLLHNILPSQSTDLHHSVGTELLSLVYKGIAPGDERQCL

PSLDLSCKQLASGLLELAFAFGGLCERLVSLLLNPAVLSTASLGSSQGSVIHFSHGEYFY

SLFSETINTELLKNLDLAVLELMQSSVDNTKMVSAVLNGMLDQSFRERANQKHQGLKLAT

TILQHWKKCDSWWAKDSPLETKMAVLALLAKILQIDSSVSFNTSHGSFPEVFTTYISLLA

DTKLDLHLKGQAVTLLPFFTSLTGGSLEELRRVLEQLIVAHFPMQSREFPPGTPRFNNYV

DCMKKFLDALELSQSPMLLELMTEVLCREQQHVMEELFQSSFRRIARRGSCVTQVGLLES

VYEMFRKDDPRLSFTRQSFVDRSLLTLLWHCSLDALREFFSTIVVDAIDVLKSRFTKLNE

STFDTQITKKMGYYKILDVMYSRLPKDDVHAKESKINQVFHGSCITEGNELTKTLIKLCY

DAFTENMAGENQLLERRRLYHCAAYNCAISVICCVFNELKFYQGFLFSEKPEKNLLIFEN

LIDLKRRYNFPVEVEVPMERKKKYIEIRKEAREAANGDSDGPSYMSSLSYLADSTLSEEM

SQFDFSTGVQSYSYSSQDPRPATGRFRRREQRDPTVHDDVLELEMDELNRHECMAPLTAL

VKHMHRSLGPPQGEEDSVPRDLPSWMKFLHGKLGNPIVPLNIRLFLAKLVINTEEVFRPY

AKHWLSPLLQLAASENNGGEGIHYMVVEIVATILSWTGLATPTGVPKDEVLANRLLNFLM

KHVFHPKRAVFRHNLEIIKTLVECWKDCLSIPYRLIFEKFSGKDPNSKDNSVGIQLLGIV

MANDLPPYDPQCGIQSSEYFQALVNNMSFVRYKEVYAAAAEVLGLILRYVMERKNILEES

LCELVAKQLKQHQNTMEDKFIVCLNKVTKSFPPLADRFMNAVFFLLPKFHGVLKTLCLEV

VLCRVEGMTELYFQLKSKDFVQVMRHRDDERQKVCLDIIYKMMPKLKPVELRELLNPVVE

FVSHPSTTCREQMYNILMWIHDNYRDPESETDNDSQEIFKLAKDVLIQGLIDENPGLQLI

IRNFWSHETRLPSNTLDRLLALNSLYSPKIEVHFLSLATNFLLEMTSMSPDYPNPMFEHP

LSECEFQEYTIDSDWRFRSTVLTPMFVETQASQGTLQTRTQEGSLSARWPVAGQIRATQQ

QHDFTLTQTADGRSSFDWLTGSSTDPLVDHTSPSSDSLLFAHKRSERLQRAPLKSVGPDF

GKKRLGLPGDEVDNKVKGAAGRTDLLRLRRRFMRDQEKLSLMYARKGVAEQKREKEIKSE

LKMKQDAQVVLYRSYRHGDLPDIQIKHSSLITPLQAVAQRDPIIAKQLFSSLFSGILKEM

DKFKTLSEKNNITQKLLQDFNRFLNTTFSFFPPFVSCIQDISCQHAALLSLDPAAVSAGC

LASLQQPVGIRLLEEALLRLLPAELPAKRVRGKARLPPDVLRWVELAKLYRSIGEYDVLR

GIFTSEIGTKQITQSALLAEARSDYSEAAKQYDEALNKQDWVDGEPTEAEKDFWELASLD

CYNHLAEWKSLEYCSTASIDSENPPDLNKIWSEPFYQETYLPYMIRSKLKLLLQGEADQS

LLTFIDKAMHGELQKAILELHYSQELSLLYLLQDDVDRAKYYIQNGIQSFMQNYSSIDVL

LHQSRLTKLQSVQALTEIQEFISFISKQGNLSSQVPLKRLLNTWTNRYPDAKMDPMNIWD

DIITNRCFFLSKIEEKLTPLPEDNSMNVDQDGDPSDRMEVQEQEEDISSLIRSCKFSMKM

KMIDSARKQNNFSLAMKLLKELHKESKTRDDWLVSWVQSYCRLSHCRSRSQGCSEQVLTV

LKTVSLLDENNVSSYLSKNILAFRDQNILLGTTYRIIANALSSEPACLAEIEEDKARRIL

ELSGSSSEDSEKVIAGLYQRAFQHLSEAVQAAEEEAQPPSWSCGPAAGVIDAYMTLADFC

DQQLRKEEENASVIDSAELQAYPALVVEKMLKALKLNSNEARLKFPRLLQIIERYPEETL

SLMTKEISSVPCWQFISWISHMVALLDKDQAVAVQHSVEEITDNYPQAIVYPFIISSESY

SFKDTSTGHKNKEFVARIKSKLDQGGVIQDFINALDQLSNPELLFKDWSNDVRAELAKTP

VNKKNIEKMYERMYAALGDPKAPGLGAFRRKFIQTFGKEFDKHFGKGGSKLLRMKLSDFN

DITNMLLLKMNKDSKPPGNLKECSPWMSDFKVEFLRNELEIPGQYDGRGKPLPEYHVRIA

GFDERVTVMASLRRPKRIIIRGHDEREHPFLVKGGEDLRQDQRVEQLFQVMNGILAQDSA

CSQRALQLRTYSVVPMTSRLGLIEWLENTVTLKDLLLNTMSQEEKAAYLSDPRAPPCEYK

DWLTKMSGKHDVGAYMLMYKGANRTETVTSFRKRESKVPADLLKRAFVRMSTSPEAFLAL

RSHFASSHALICISHWILGIGDRHLNNFMVAMETGGVIGIDFGHAFGSATQFLPVPELMP

FRLTRQFINLMLPMKETGLMYSIMVHALRAFRSDPGLLTNTMDVFVKEPSFDWKNFEQKM

LKKGGSWIQEINVAEKNWYPRQKICYAKRKLAGANPAVITCDELLLGHEKAPAFRDYVAV

ARGSKDHNIRAQEPESGLSEETQVKCLMDQATDPNILGRTWEGWEPWM

>sp|P78527|PRKDC_HUMAN 3432 SVIDSAELQAY

MAGSGAGVRCSLLRLQETLSAADRCGAALAGHQLIRGLGQECVLSSSPAVLALQTSLVFS

RDFGLLVFVRKSLNSIEFRECREEILKFLCIFLEKMGQKIAPYSVEIKNTCTSVYTKDRA

AKCKIPALDLLIKLLQTFRSSRLMDEFKIGELFSKFYGELALKKKIPDTVLEKVYELLGL

LGEVHPSEMINNAENLFRAFLGELKTQMTSAVREPKLPVLAGCLKGLSSLLCNFTKSMEE

DPQTSREIFNFVLKAIRPQIDLKRYAVPSAGLRLFALHASQFSTCLLDNYVSLFEVLLKW

CAHTNVELKKAALSALESFLKQVSNMVAKNAEMHKNKLQYFMEQFYGIIRNVDSNNKELS

IAIRGYGLFAGPCKVINAKDVDFMYVELIQRCKQMFLTQTDTGDDRVYQMPSFLQSVASV

LLYLDTVPEVYTPVLEHLVVMQIDSFPQYSPKMQLVCCRAIVKVFLALAAKGPVLRNCIS

TVVHQGLIRICSKPVVLPKGPESESEDHRASGEVRTGKWKVPTYKDYVDLFRHLLSSDQM

MDSILADEAFFSVNSSSESLNHLLYDEFVKSVLKIVEKLDLTLEIQTVGEQENGDEAPGV

WMIPTSDPAANLHPAKPKDFSAFINLVEFCREILPEKQAEFFEPWVYSFSYELILQSTRL

PLISGFYKLLSITVRNAKKIKYFEGVSPKSLKHSPEDPEKYSCFALFVKFGKEVAVKMKQ

YKDELLASCLTFLLSLPHNIIELDVRAYVPALQMAFKLGLSYTPLAEVGLNALEEWSIYI

DRHVMQPYYKDILPCLDGYLKTSALSDETKNNWEVSALSRAAQKGFNKVVLKHLKKTKNL

SSNEAISLEEIRIRVVQMLGSLGGQINKNLLTVTSSDEMMKSYVAWDREKRLSFAVPFRE

MKPVIFLDVFLPRVTELALTASDRQTKVAACELLHSMVMFMLGKATQMPEGGQGAPPMYQ

LYKRTFPVLLRLACDVDQVTRQLYEPLVMQLIHWFTNNKKFESQDTVALLEAILDGIVDP

VDSTLRDFCGRCIREFLKWSIKQITPQQQEKSPVNTKSLFKRLYSLALHPNAFKRLGASL

AFNNIYREFREEESLVEQFVFEALVIYMESLALAHADEKSLGTIQQCCDAIDHLCRIIEK

KHVSLNKAKKRRLPRGFPPSASLCLLDLVKWLLAHCGRPQTECRHKSIELFYKFVPLLPG

NRSPNLWLKDVLKEEGVSFLINTFEGGGCGQPSGILAQPTLLYLRGPFSLQATLCWLDLL

LAALECYNTFIGERTVGALQVLGTEAQSSLLKAVAFFLESIAMHDIIAAEKCFGTGAAGN

RTSPQEGERYNYSKCTVVVRIMEFTTTLLNTSPEGWKLLKKDLCNTHLMRVLVQTLCEPA

SIGFNIGDVQVMAHLPDVCVNLMKALKMSPYKDILETHLREKITAQSIEELCAVNLYGPD

AQVDRSRLAAVVSACKQLHRAGLLHNILPSQSTDLHHSVGTELLSLVYKGIAPGDERQCL

PSLDLSCKQLASGLLELAFAFGGLCERLVSLLLNPAVLSTASLGSSQGSVIHFSHGEYFY

SLFSETINTELLKNLDLAVLELMQSSVDNTKMVSAVLNGMLDQSFRERANQKHQGLKLAT

TILQHWKKCDSWWAKDSPLETKMAVLALLAKILQIDSSVSFNTSHGSFPEVFTTYISLLA

DTKLDLHLKGQAVTLLPFFTSLTGGSLEELRRVLEQLIVAHFPMQSREFPPGTPRFNNYV

DCMKKFLDALELSQSPMLLELMTEVLCREQQHVMEELFQSSFRRIARRGSCVTQVGLLES

VYEMFRKDDPRLSFTRQSFVDRSLLTLLWHCSLDALREFFSTIVVDAIDVLKSRFTKLNE

STFDTQITKKMGYYKILDVMYSRLPKDDVHAKESKINQVFHGSCITEGNELTKTLIKLCY

DAFTENMAGENQLLERRRLYHCAAYNCAISVICCVFNELKFYQGFLFSEKPEKNLLIFEN

LIDLKRRYNFPVEVEVPMERKKKYIEIRKEAREAANGDSDGPSYMSSLSYLADSTLSEEM

SQFDFSTGVQSYSYSSQDPRPATGRFRRREQRDPTVHDDVLELEMDELNRHECMAPLTAL

VKHMHRSLGPPQGEEDSVPRDLPSWMKFLHGKLGNPIVPLNIRLFLAKLVINTEEVFRPY

AKHWLSPLLQLAASENNGGEGIHYMVVEIVATILSWTGLATPTGVPKDEVLANRLLNFLM

KHVFHPKRAVFRHNLEIIKTLVECWKDCLSIPYRLIFEKFSGKDPNSKDNSVGIQLLGIV

MANDLPPYDPQCGIQSSEYFQALVNNMSFVRYKEVYAAAAEVLGLILRYVMERKNILEES

LCELVAKQLKQHQNTMEDKFIVCLNKVTKSFPPLADRFMNAVFFLLPKFHGVLKTLCLEV

VLCRVEGMTELYFQLKSKDFVQVMRHRDDERQKVCLDIIYKMMPKLKPVELRELLNPVVE

FVSHPSTTCREQMYNILMWIHDNYRDPESETDNDSQEIFKLAKDVLIQGLIDENPGLQLI

IRNFWSHETRLPSNTLDRLLALNSLYSPKIEVHFLSLATNFLLEMTSMSPDYPNPMFEHP

LSECEFQEYTIDSDWRFRSTVLTPMFVETQASQGTLQTRTQEGSLSARWPVAGQIRATQQ

QHDFTLTQTADGRSSFDWLTGSSTDPLVDHTSPSSDSLLFAHKRSERLQRAPLKSVGPDF

GKKRLGLPGDEVDNKVKGAAGRTDLLRLRRRFMRDQEKLSLMYARKGVAEQKREKEIKSE

LKMKQDAQVVLYRSYRHGDLPDIQIKHSSLITPLQAVAQRDPIIAKQLFSSLFSGILKEM

DKFKTLSEKNNITQKLLQDFNRFLNTTFSFFPPFVSCIQDISCQHAALLSLDPAAVSAGC

LASLQQPVGIRLLEEALLRLLPAELPAKRVRGKARLPPDVLRWVELAKLYRSIGEYDVLR

GIFTSEIGTKQITQSALLAEARSDYSEAAKQYDEALNKQDWVDGEPTEAEKDFWELASLD

CYNHLAEWKSLEYCSTASIDSENPPDLNKIWSEPFYQETYLPYMIRSKLKLLLQGEADQS

LLTFIDKAMHGELQKAILELHYSQELSLLYLLQDDVDRAKYYIQNGIQSFMQNYSSIDVL

LHQSRLTKLQSVQALTEIQEFISFISKQGNLSSQVPLKRLLNTWTNRYPDAKMDPMNIWD

DIITNRCFFLSKIEEKLTPLPEDNSMNVDQDGDPSDRMEVQEQEEDISSLIRSCKFSMKM

KMIDSARKQNNFSLAMKLLKELHKESKTRDDWLVSWVQSYCRLSHCRSRSQGCSEQVLTV

LKTVSLLDENNVSSYLSKNILAFRDQNILLGTTYRIIANALSSEPACLAEIEEDKARRIL

ELSGSSSEDSEKVIAGLYQRAFQHLSEAVQAAEEEAQPPSWSCGPAAGVIDAYMTLADFC

DQQLRKEEENASVIDSAELQAYPALVVEKMLKALKLNSNEARLKFPRLLQIIERYPEETL

SLMTKEISSVPCWQFISWISHMVALLDKDQAVAVQHSVEEITDNYPQAIVYPFIISSESY

SFKDTSTGHKNKEFVARIKSKLDQGGVIQDFINALDQLSNPELLFKDWSNDVRAELAKTP

VNKKNIEKMYERMYAALGDPKAPGLGAFRRKFIQTFGKEFDKHFGKGGSKLLRMKLSDFN

DITNMLLLKMNKDSKPPGNLKECSPWMSDFKVEFLRNELEIPGQYDGRGKPLPEYHVRIA

GFDERVTVMASLRRPKRIIIRGHDEREHPFLVKGGEDLRQDQRVEQLFQVMNGILAQDSA

CSQRALQLRTYSVVPMTSRLGLIEWLENTVTLKDLLLNTMSQEEKAAYLSDPRAPPCEYK

DWLTKMSGKHDVGAYMLMYKGANRTETVTSFRKRESKVPADLLKRAFVRMSTSPEAFLAL

RSHFASSHALICISHWILGIGDRHLNNFMVAMETGGVIGIDFGHAFGSATQFLPVPELMP

FRLTRQFINLMLPMKETGLMYSIMVHALRAFRSDPGLLTNTMDVFVKEPSFDWKNFEQKM

LKKGGSWIQEINVAEKNWYPRQKICYAKRKLAGANPAVITCDELLLGHEKAPAFRDYVAV

ARGSKDHNIRAQEPESGLSEETQVKCLMDQATDPNILGRTWEGWEPWM

>sp|Q8TD57|DYH3_HUMAN 1336 TLGALTVIDV

MGATGRLELTLAAPPHPGPAFQRSKARETQGEEEGSEMQIAKSDSIHHMSHSQGQPELPP

LPASANEEPSGLYQTVMSHSFYPPLMQRTSWTLAAPFKEQHHHRGPSDSIANNYSLMAQD

LKLKDLLKVYQPATISVPRDRTGQGLPSSGNRSSSEPMRKKTKFSSRNKEDSTRIKLAFK

TSIFSPMKKEVKTSLTFPGSRPMSPEQQLDVMLQQEMEMESKEKKPSESDLERYYYYLTN

GIRKDMIAPEEGEVMVRISKLISNTLLTSPFLEPLMVVLVQEKENDYYCSLMKSIVDYIL

MDPMERKRLFIESIPRLFPQRVIRAPVPWHSVYRSAKKWNEEHLHTVNPMMLRLKELWFA

EFRDLRFVRTAEILAGKLPLQPQEFWDVIQKHCLEAHQTLLNKWIPTCAQLFTSRKEHWI

HFAPKSNYDSSRNIEEYFASVASFMSLQLRELVIKSLEDLVSLFMIHKDGNDFKEPYQEM

KFFIPQLIMIKLEVSEPIIVFNPSFDGCWELIRDSFLEIIKNSNGIPKLKYIPLKFSFTA

AAADRQCVKAAEPGEPSMHAAATAMAELKGYNLLLGTVNAEEKLVSDFLIQTFKVFQKNQ

VGPCKYLNVYKKYVDLLDNTAEQNIAAFLKENHDIDDFVTKINAIKKRRNEIASMNITVP

LAMFCLDATALNHDLCERAQNLKDHLIQFQVDVNRDTNTSICNQYSHIADKVSEVPANTK

ELVSLIEFLKKSSAVTVFKLRRQLRDASERLEFLMDYADLPYQIEDIFDNSRNLLLHKRD

QAEMDLIKRCSEFELRLEGYHRELESFRKREVMTTEEMKHNVEKLNELSKNLNRAFAEFE

LINKEEELLEKEKSTYPLLQAMLKNKVPYEQLWSTAYEFSIKSEEWMNGPLFLLNAEQIA

EEIGNMWRTTYKLIKTLSDVPAPRRLAENVKIKIDKFKQYIPILSISCNPGMKDRHWQQI

SEIVGYEIKPTETTCLSNMLEFGFGKFVEKLEPIGAAASKEYSLEKNLDRMKLDWVNVTF

SFVKYRDTDTNILCAIDDIQMLLDDHVIKTQTMCGSPFIKPIEAECRKWEEKLIRIQDNL

DAWLKCQATWLYLEPIFSSEDIIAQMPEEGRKFGIVDSYWKSLMSQAVKDNRILVAADQP

RMAEKLQEANFLLEDIQKGLNDYLEKKRLFFPRFFFLSNDELLEILSETKDPLRVQPHLK

KCFEGIAKLEFTDNLEIVGMISSEKETVPFIQKIYPANAKGMVEKWLQQVEQMMLASMRE

VIGLGIEAYVKVPRNHWVLQWPGQVVICVSSIFWTQEVSQALAENTLLDFLKKSNDQIAQ

IVQLVRGKLSSGARLTLGALTVIDVHARDVVAKLSEDRVSDLNDFQWISQLRYYWVAKDV

QVQIITTEALYGYEYLGNSPRLVITPLTDRCYRTLMGALKLNLGGAPEGPAGTGKTETTK

DLAKALAKQCVVFNCSDGLDYKAMGKFFKGLAQAGAWACFDEFNRIEVEVLSVVAQQILS

IQQAIIRKLKTFIFEGTELSLNPTCAVFITMNPGYAGRAELPDNLKALFRTVAMMVPDYA

LIGEISLYSMGFLDSRSLAQKIVATYRLCSEQLSSQHHYDYGMRAVKSVLTAAGNLKLKY

PEENESVLLLRALLDVNLAKFLAQDVPLFQGIISDLFPGVVLPKPDYEVFLKVLNDNIKK

MKLQPVPWFIGKIIQIYEMMLVRHGYMIVGDPMGGKTSAYKVLAAALGDLHAANQMEEFA

VEYKIINPKAITMGQLYGCFDQVSHEWMDGVLANAFREQASSLSDDRKWIIFDGPVDAIW

IENMNTVLDDNKKLCLMSGEIIQMNSKMSLIFEPADLEQASPATVSRCGMIYMEPHQLGW

KPLKDSYMDTLPSSLTKEHKELVNDMFMWLVQPCLEFGRLHCKFVVQTSPIHLAFSMMRL

YSSLLDEIRAVEEEEMELGEGLSSQQIFLWLQGLFLFSLVWTVAGTINADSRKKFDVFFR

NLIMGMDDNHPRPKSVKLTKNNIFPERGSIYDFYFIKQASGHWETWTQYITKEEEKVPAG

AKVSELIIPTMETARQSFFLKTYLDHEIPMLFVGPTGTGKSAITNNFLLHLPKNTYLPNC

INFSARTSANQTQDIIMSKLDRRRKGLFGPPIGKKAVVFVDDLNMPAKEVYGAQPPIELL

RQWIDHGYWFDKKDTTRLDIVDMLLVTAMGPPGGGRNDITGRFTRHLNIISINAFEDDIL

TKIFSSIVDWHFGKGFDVMFLRYGKMLVQATKTIYRDAVENFLPTPSKSHYVFNLRDFSR

VIQGVLLCPHTHLQDVEKCIRLWIHEVYRVFYDRLIDKEDRQVFFNMVKETTSNCFKQTI

EKVLIHLSPTGKIVDDNIRSLFFGDYFKPESDQKIYDEITDLKQLTVVMEHYLEEFNNIS

KAPMSLVMFRFAIEHISRICRVLKQDKGHLLLVGIGGSGRQSAAKLSTFMNAYELYQIEI

TKNYAGNDWREDLKKIILQVGVATKSTVFLFADNQIKDESFVEDINMLLNTGDVPNIFPA

DEKADIVEKMQTAARTQGEKVEVTPLSMYNFFIERVINKISFSLAMSPIGDAFRNRLRMF

PSLINCCTIDWFQSWPTDALELVANKFLEDVELDDNIRVEVVSMCKYFQESVKKLSLDYY

NKLRRHNYVTPTSYLELILTFKTLLNSKRQEVAMMRNRYLTGLQKLDFAASQVAVMQREL

TALQPQLILTSEETAKMMVKIEAETREADGKKLLVQADEKEANVAAAIAQGIKNECEGDL

AEAMPALEAALAALDTLNPADISLVKSMQNPPGPVKLVMESICIMKGMKPERKPDPSGSG

KMIEDYWGVSKKILGDLKFLESLKTYDKDNIPPLTMKRIRERFINHPEFQPAVIKNVSSA

CEGLCKWVRAMEVYDRVAKVVAPKRERLREAEGKLAAQMQKLNQKRAELKLVVDRLQALN

DDFEEMNTKKKDLEENIEICSQKLVRAEKLISGLGGEKDRWTEAARQLGIRYTNLTGDVL

LSSGTVAYLGAFTVDYRVQCQNQWLAECKDKVIPGFSDFSLSHTLGDPIKIRAWQIAGLP

VDSFSIDNGIIVSNSRRWALMIDPHGQANKWIKNMEKANKLAVIKFSDSNYMRMLENALQ

LGTPVLIENIGEELDASIEPILLKATFKQQGVEYMRLGENIIEYSRDFKLYITTRLRNPH

YLPEVAVKVCLLNFMITPLGLQDQLLGIVAAKEKPELEEKKNQLIVESAKNKKHLKEIED

KILEVLSMSKGNILEDETAIKVLSSSKVLSEEISEKQKVASMTETQIDETRMGYKPVAVH

SATIFFCISDLANIEPMYQYSLTWFINLYMHSLTHSTKSEELNLRIKYIIDHFTLSIYNN

VCRSLFEKDKLLFSLLLTIGIMKQKKEITEEVWYFLLTGGIALDNPYPNPAPQWLSEKAW

AEIVRASALPKLHGLMEHLEQNLGEWKLIYDSAWPHEEQLPGSWKFSQGLEKMVILRCLR

PDKMVPAVREFIAEHMGKLYIEAPTFDLQGSYNDSSCCAPLIFVLSPSADPMAGLLKFAD

DLGMGGTRTQTISLGQGQGPIAAKMINNAIKDGTWVVLQNCHLAASWMPTLEKICEEVIV

PESTNARFRLWLTSYPSEKFPVSILQNGIKMTNEPPKGLRANLLRSYLNDPISDPVFFQS

CAKAVMWQKMLFGLCFFHAVVQERRNFGPLGWNIPYEFNESDLRISMWQIQMFLNDYKEV

PFDALTYLTGECNYGGRVTDDKDRRLLLSLLSMFYCKEIEEDYYSLAPGDTYYIPPHGSY

QSYIDYLRNLPITAHPEVFGLHENADITKDNQETNQLFEGVLLTLPRQSGGSGKSPQEVV

EELAQDILSKLPRDFDLEEVMKLYPVVYEESMNTVLRQELIRFNRLTKVVRRSLINLGRA

IKGQVLMSSELEEVFNSMLVGKVPAMWAAKSYPSLKPLGGYVADLLARLTFFQEWIDKGP

PVVFWISGFYFTQSFLTGVSQNYARKYTIPIDHIGFEFEVTPQETVMENNPEDGAYIKGL

FLEGARWDRKTMQIGESLPKILYDPLPIIWLKPGESAMFLHQDIYVCPVYKTSARRGTLS

TTGHSTNYVLSIELPTDMPQKHWINRGVASLCQLDN

>sp|Q03164|HRX_HUMAN 1764 RVFPWFSVK

MAHSCRWRFPARPGTTGGGGGGGRRGLGGAPRQRVPALLLPPGPPVGGGGPGAPPSPPAV

AAAAAAAGSSGAGVPGGAAAASAASSSSASSSSSSSSSASSGPALLRVGPGFDAALQVSA

AIGTNLRRFRAVFGESGGGGGSGEDEQFLGFGSDEEVRVRSPTRSPSVKTSPRKPRGRPR

SGSDRNSAILSDPSVFSPLNKSETKSGDKIKKKDSKSIEKKRGRPPTFPGVKIKITHGKD

ISELPKGNKEDSLKKIKRTPSATFQQATKIKKLRAGKLSPLKSKFKTGKLQIGRKGVQIV

RRRGRPPSTERIKTPSGLLINSELEKPQKVRKDKEGTPPLTKEDKTVVRQSPRRIKPVRI

IPSSKRTDATIAKQLLQRAKKGAQKKIEKEAAQLQGRKVKTQVKNIRQFIMPVVSAISSR

IIKTPRRFIEDEDYDPPIKIARLESTPNSRFSAPSCGSSEKSSAASQHSSQMSSDSSRSS

SPSVDTSTDSQASEEIQVLPEERSDTPEVHPPLPISQSPENESNDRRSRRYSVSERSFGS

RTTKKLSTLQSAPQQQTSSSPPPPLLTPPPPLQPASSISDHTPWLMPPTIPLASPFLPAS

TAPMQGKRKSILREPTFRWTSLKHSRSEPQYFSSAKYAKEGLIRKPIFDNFRPPPLTPED

VGFASGFSASGTAASARLFSPLHSGTRFDMHKRSPLLRAPRFTPSEAHSRIFESVTLPSN

RTSAGTSSSGVSNRKRKRKVFSPIRSEPRSPSHSMRTRSGRLSSSELSPLTPPSSVSSSL

SISVSPLATSALNPTFTFPSHSLTQSGESAEKNQRPRKQTSAPAEPFSSSSPTPLFPWFT

PGSQTERGRNKDKAPEELSKDRDADKSVEKDKSRERDREREKENKRESRKEKRKKGSEIQ

SSSALYPVGRVSKEKVVGEDVATSSSAKKATGRKKSSSHDSGTDITSVTLGDTTAVKTKI

LIKKGRGNLEKTNLDLGPTAPSLEKEKTLCLSTPSSSTVKHSTSSIGSMLAQADKLPMTD

KRVASLLKKAKAQLCKIEKSKSLKQTDQPKAQGQESDSSETSVRGPRIKHVCRRAAVALG

RKRAVFPDDMPTLSALPWEEREKILSSMGNDDKSSIAGSEDAEPLAPPIKPIKPVTRNKA

PQEPPVKKGRRSRRCGQCPGCQVPEDCGVCTNCLDKPKFGGRNIKKQCCKMRKCQNLQWM

PSKAYLQKQAKAVKKKEKKSKTSEKKDSKESSVVKNVVDSSQKPTPSAREDPAPKKSSSE

PPPRKPVEEKSEEGNVSAPGPESKQATTPASRKSSKQVSQPALVIPPQPPTTGPPRKEVP

KTTPSEPKKKQPPPPESGPEQSKQKKVAPRPSIPVKQKPKEKEKPPPVNKQENAGTLNIL

STLSNGNSSKQKIPADGVHRIRVDFKEDCEAENVWEMGGLGILTSVPITPRVVCFLCASS

GHVEFVYCQVCCEPFHKFCLEENERPLEDQLENWCCRRCKFCHVCGRQHQATKQLLECNK

CRNSYHPECLGPNYPTKPTKKKKVWICTKCVRCKSCGSTTPGKGWDAQWSHDFSLCHDCA

KLFAKGNFCPLCDKCYDDDDYESKMMQCGKCDRWVHSKCENLSDEMYEILSNLPESVAYT

CVNCTERHPAEWRLALEKELQISLKQVLTALLNSRTTSHLLRYRQAAKPPDLNPETEESI

PSRSSPEGPDPPVLTEVSKQDDQQPLDLEGVKRKMDQGNYTSVLEFSDDIVKIIQAAINS

DGGQPEIKKANSMVKSFFIRQMERVFPWFSVKKSRFWEPNKVSSNSGMLPNAVLPPSLDH

NYAQWQEREENSHTEQPPLMKKIIPAPKPKGPGEPDSPTPLHPPTPPILSTDRSREDSPE

LNPPPGIEDNRQCALCLTYGDDSANDAGRLLYIGQNEWTHVNCALWSAEVFEDDDGSLKN

VHMAVIRGKQLRCEFCQKPGATVGCCLTSCTSNYHFMCSRAKNCVFLDDKKVYCQRHRDL

IKGEVVPENGFEVFRRVFVDFEGISLRRKFLNGLEPENIHMMIGSMTIDCLGILNDLSDC

EDKLFPIGYQCSRVYWSTTDARKRCVYTCKIVECRPPVVEPDINSTVEHDENRTIAHSPT

SFTESSSKESQNTAEIISPPSPDRPPHSQTSGSCYYHVISKVPRIRTPSYSPTQRSPGCR

PLPSAGSPTPTTHEIVTVGDPLLSSGLRSIGSRRHSTSSLSPQRSKLRIMSPMRTGNTYS

RNNVSSVSTTGTATDLESSAKVVDHVLGPLNSSTSLGQNTSTSSNLQRTVVTVGNKNSHL

DGSSSSEMKQSSASDLVSKSSSLKGEKTKVLSSKSSEGSAHNVAYPGIPKLAPQVHNTTS

RELNVSKIGSFAEPSSVSFSSKEALSFPHLHLRGQRNDRDQHTDSTQSANSSPDEDTEVK

TLKLSGMSNRSSIINEHMGSSSRDRRQKGKKSCKETFKEKHSSKSFLEPGQVTTGEEGNL

KPEFMDEVLTPEYMGQRPCNNVSSDKIGDKGLSMPGVPKAPPMQVEGSAKELQAPRKRTV

KVTLTPLKMENESQSKNALKESSPASPLQIESTSPTEPISASENPGDGPVAQPSPNNTSC

QDSQSNNYQNLPVQDRNLMLPDGPKPQEDGSFKRRYPRRSARARSNMFFGLTPLYGVRSY

GEEDIPFYSSSTGKKRGKRSAEGQVDGADDLSTSDEDDLYYYNFTRTVISSGGEERLASH

NLFREEEQCDLPKISQLDGVDDGTESDTSVTATTRKSSQIPKRNGKENGTENLKIDRPED

AGEKEHVTKSSVGHKNEPKMDNCHSVSRVKTQGQDSLEAQLSSLESSRRVHTSTPSDKNL

LDTYNTELLKSDSDNNNSDDCGNILPSDIMDFVLKNTPSMQALGESPESSSSELLNLGEG

LGLDSNREKDMGLFEVFSQQLPTTEPVDSSVSSSISAEEQFELPLELPSDLSVLTTRSPT

VPSQNPSRLAVISDSGEKRVTITEKSVASSESDPALLSPGVDPTPEGHMTPDHFIQGHMD

ADHISSPPCGSVEQGHGNNQDLTRNSSTPGLQVPVSPTVPIQNQKYVPNSTDSPGPSQIS

NAAVQTTPPHLKPATEKLIVVNQNMQPLYVLQTLPNGVTQKIQLTSSVSSTPSVMETNTS

VLGPMGGGLTLTTGLNPSLPTSQSLFPSASKGLLPMSHHQHLHSFPAATQSSFPPNISNP

PSGLLIGVQPPPDPQLLVSESSQRTDLSTTVATPSSGLKKRPISRLQTRKNKKLAPSSTP

SNIAPSDVVSNMTLINFTPSQLPNHPSLLDLGSLNTSSHRTVPNIIKRSKSSIMYFEPAP

LLPQSVGGTAATAAGTSTISQDTSHLTSGSVSGLASSSSVLNVVSMQTTTTPTSSASVPG

HVTLTNPRLLGTPDIGSISNLLIKASQQSLGIQDQPVALPPSSGMFPQLGTSQTPSTAAI

TAASSICVLPSTQTTGITAASPSGEADEHYQLQHVNQLLASKTGIHSSQRDLDSASGPQV

SNFTQTVDAPNSMGLEQNKALSSAVQASPTSPGGSPSSPSSGQRSASPSVPGPTKPKPKT

KRFQLPLDKGNGKKHKVSHLRTSSSEAHIPDQETTSLTSGTGTPGAEAEQQDTASVEQSS

QKECGQPAGQVAVLPEVQVTQNPANEQESAEPKTVEEEESNFSSPLMLWLQQEQKRKESI

TEKKPKKGLVFEISSDDGFQICAESIEDAWKSLTDKVQEARSNARLKQLSFAGVNGLRML

GILHDAVVFLIEQLSGAKHCRNYKFRFHKPEEANEPPLNPHGSARAEVHLRKSAFDMFNF

LASKHRQPPEYNPNDEEEEEVQLKSARRATSMDLPMPMRFRHLKKTSKEAVGVYRSPIHG

RGLFCKRNIDAGEMVIEYAGNVIRSIQTDKREKYYDSKGIGCYMFRIDDSEVVDATMHGN

AARFINHSCEPNCYSRVINIDGQKHIVIFAMRKIYRGEELTYDYKFPIEDASNKLPCNCG

AKKCRKFLN

>sp|Q01484|ANK2_HUMAN 1363 GQHHIFSFF

MMNEDAAQKSDSGEKFNGSSQRRKRPKKSDSNASFLRAARAGNLDKVVEYLKGGIDINTC

NQNGLNALHLAAKEGHVGLVQELLGRGSSVDSATKKGNTALHIASLAGQAEVVKVLVKEG

ANINAQSQNGFTPLYMAAQENHIDVVKYLLENGANQSTATEDGFTPLAVALQQGHNQAVA

ILLENDTKGKVRLPALHIAARKDDTKSAALLLQNDHNADVQSKMMVNRTTESGFTPLHIA

AHYGNVNVATLLLNRGAAVDFTARNGITPLHVASKRGNTNMVKLLLDRGGQIDAKTRDGL

TPLHCAARSGHDQVVELLLERGAPLLARTKNGLSPLHMAAQGDHVECVKHLLQHKAPVDD

VTLDYLTALHVAAHCGHYRVTKLLLDKRANPNARALNGFTPLHIACKKNRIKVMELLVKY

GASIQAITESGLTPIHVAAFMGHLNIVLLLLQNGASPDVTNIRGETALHMAARAGQVEVV

RCLLRNGALVDARAREEQTPLHIASRLGKTEIVQLLLQHMAHPDAATTNGYTPLHISARE

GQVDVASVLLEAGAAHSLATKKGFTPLHVAAKYGSLDVAKLLLQRRAAADSAGKNGLTPL

HVAAHYDNQKVALLLLEKGASPHATAKNGYTPLHIAAKKNQMQIASTLLNYGAETNIVTK

QGVTPLHLASQEGHTDMVTLLLDKGANIHMSTKSGLTSLHLAAQEDKVNVADILTKHGAD

QDAHTKLGYTPLIVACHYGNVKMVNFLLKQGANVNAKTKNGYTPLHQAAQQGHTHIINVL

LQHGAKPNATTANGNTALAIAKRLGYISVVDTLKVVTEEVTTTTTTITEKHKLNVPETMT

EVLDVSDEEGDDTMTGDGGEYLRPEDLKELGDDSLPSSQFLDGMNYLRYSLEGGRSDSLR

SFSSDRSHTLSHASYLRDSAVMDDSVVIPSHQVSTLAKEAERNSYRLSWGTENLDNVALS

SSPIHSGFLVIFMVDARGGAMRGCRHNGLRIIIPPRKCTAPTRVTCRLVKRHRLATMPPM

VEGEGLASRLIEVGPSGAQFLGPVIVEIPHFAALRGKERELVVLRSENGDSWKEHFCDYT

EDELNEILNGMDEVLDSPEDLEKKRICRIITRDFPQYFAVVSRIKQDSNLIGPEGGVLSS

TVVPQVQAVFPEGALTKRIRVGLQAQPMHSELVKKILGNKATFSPIVTLEPRRRKFHKPI

TMTIPVPKASSDVMLNGFGGDAPTLRLLCSITGGTTPAQWEDITGTTPLTFVNECVSFTT

NVSARFWLIDCRQIQESVTFASQVYREIICVPYMAKFVVFAKSHDPIEARLRCFCMTDDK

VDKTLEQQENFAEVARSRDVEVLEGKPIYVDCFGNLVPLTKSGQHHIFSFFAFKENRLPL

FVKVRDTTQEPCGRLSFMKEPKSTRGLVHQAICNLNITLPIYTKESESDQEQEEEIDMTS

EKNDETESTETSVLKSHLVNEVPVLASPDLLSEVSEMKQDLIKMTAILTTDVSDKAGSIK

VKELVKAAEEEPGEPFEIVERVKEDLEKVNEILRSGTCTRDESSVQSSRSERGLVEEEWV

IVSDEEIEEARQKAPLEITEYPCVEVRIDKEIKGKVEKDSTGLVNYLTDDLNTCVPLPKE

QLQTVQDKAGKKCEALAVGRSSEKEGKDIPPDETQSTQKQHKPSLGIKKPVRRKLKEKQK

QKEEGLQASAEKAELKKGSSEESLGEDPGLAPEPLPTVKATSPLIEETPIGSIKDKVKAL

QKRVEDEQKGRSKLPIRVKGKEDVPKKTTHRPHPAASPSLKSERHAPGSPSPKTERHSTL

SSSAKTERHPPVSPSSKTEKHSPVSPSAKTERHSPASSSSKTEKHSPVSPSTKTERHSPV

SSTKTERHPPVSPSGKTDKRPPVSPSGRTEKHPPVSPGRTEKRLPVSPSGRTDKHQPVST

AGKTEKHLPVSPSGKTEKQPPVSPTSKTERIEETMSVRELMKAFQSGQDPSKHKTGLFEH

KSAKQKQPQEKGKVRVEKEKGPILTQREAQKTENQTIKRGQRLPVTGTAESKRGVRVSSI

GVKKEDAAGGKEKVLSHKIPEPVQSVPEEESHRESEVPKEKMADEQGDMDLQISPDRKTS

TDFSEVIKQELEDNDKYQQFRLSEETEKAQLHLDQVLTSPFNTTFPLDYMKDEFLPALSL

QSGALDGSSESLKNEGVAGSPCGSLMEGTPQISSEESYKHEGLAETPETSPESLSFSPKK

SEEQTGETKESTKTETTTEIRSEKEHPTTKDITGGSEERGATVTEDSETSTESFQKEATL

GSPKDTSPKRQDDCTGSCSVALAKETPTGLTEEAACDEGQRTFGSSAHKTQTDSEAQEST

ATSDETKALPLPEASVKTDTGTESKPQGVIRSPQGLELALPSRDSEVLSAVADDSLAVSH

KDSLEASPVLEDNSSHKTPDSLEPSPLKESPCRDSLESSPVEPKMKAGIFPSHFPLPAAV

AKTELLTEVASVRSRLLRDPDGSAEDDSLEQTSLMESSGKSPLSPDTPSSEEVSYEVTPK

TTDVSTPKPAVIHECAEEDDSENGEKKRFTPEEEMFKMVTKIKMFDELEQEAKQKRDYKK

EPKQEESSSSSDPDADCSVDVDEPKHTGSGEDESGVPVLVTSESRKVSSSSESEPELAQL

KKGADSGLLPEPVIRVQPPSPLPSSMDSNSSPEEVQFQPVVSKQYTFKMNEDTQEEPGKS

EEEKDSESHLAEDRHAVSTEAEDRSYDKLNRDTDQPKICDGHGCEAMSPSSSARPVSSGL

QSPTGDDVDEQPVIYKESLALQGTHEKDTEGEELDVSRAESPQADCPSESFSSSSSLPHC

LVSEGKELDEDISATSSIQKTEVTKTDETFENLPKDCPSQDSSITTQTDRFSMDVPVSDL

AENDEIYDPQITSPYENVPSQSFFSSEESKTQTDANHTTSFHSSEVYSVTITSPVEDVVV

ASSSSGTVLSKESNFEGQDIKMESQLESTLWEMQSDSVSSSFEPTMSATTTVVGEQISKV

IITKTDVDSDSWSEIREDDEAFEARVKEEEQKIFGLMVDRQSQGTTPDTTPARTPTEEGT

PTSEQNPFLFQEGKLFEMTRSGAIDMTKRSYADESFHFFQIGQESREETLSEDVKEGATG

ADPLPLETSAESLALSESKETVDDEADLLPDSVSEEVEEIPASDAQLNSQMGISASTETP

TKEAVSVGTKDLPTVQTGDIPPLSGVKQISCPDSSEPAVQVQLDFSTLTRSVYSDRGDDS

PDSSPEEQKSVIEIPTAPMENVPFTESKSKIPVRTMPTSTPAPPSAEYESSVSEDFLSSV

DEENKADEAKPKSKLPVKVPLQRVEQQLSDLDTSVQKTVAPQGQDMASIAPDNRSKSESD

ASSLDSKTKCPVKTRSYTETETESRERAEELELESEEGATRPKILTSRLPVKSRSTTSSC

RGGTSPTKESKEHFFDLYRNSIEFFEEISDEASKLVDRLTQSEREQEIVSDDESSSALEV

SVIENLPPVETEHSVPEDIFDTRPIWDESIETLIERIPDENGHDHAEDPQDEQERIEERL

AYIADHLGFSWTELARELDFTEEQIHQIRIENPNSLQDQSQYLLKIWLERDGKHATDTNL

VECLTKINRMDIVHLMETNTEPLQERISHSYAEIEQTITLDHSEGFSVLQEELCTAQHKQ

KEEQAVSKESETCDHPPIVSEEDISVGYSTFQDGVPKTEGDSSSTALFPQTHKEQVQQDF

SGKMQDLPEESSLEYQQEYFVTTPGTETSETQKAMIVPSSPSKTPEEVSTPAEEEKLYLQ

TPTSSERGGSPIIQEPEEPSEHREESSPRKTSLVIVESADNQPETCERLDEDAAFEKGDD

MPEIPPETVTEEEYIDEHGHTVVKKVTRKIIRRYVSSEGTEKEEIMVQGMPQEPVNIEEG

DGYSKVIKRVVLKSDTEQSEDNNE

>sp|Q01484|ANK2_HUMAN 1272 RQIQESVTF

MMNEDAAQKSDSGEKFNGSSQRRKRPKKSDSNASFLRAARAGNLDKVVEYLKGGIDINTC

NQNGLNALHLAAKEGHVGLVQELLGRGSSVDSATKKGNTALHIASLAGQAEVVKVLVKEG

ANINAQSQNGFTPLYMAAQENHIDVVKYLLENGANQSTATEDGFTPLAVALQQGHNQAVA

ILLENDTKGKVRLPALHIAARKDDTKSAALLLQNDHNADVQSKMMVNRTTESGFTPLHIA

AHYGNVNVATLLLNRGAAVDFTARNGITPLHVASKRGNTNMVKLLLDRGGQIDAKTRDGL

TPLHCAARSGHDQVVELLLERGAPLLARTKNGLSPLHMAAQGDHVECVKHLLQHKAPVDD

VTLDYLTALHVAAHCGHYRVTKLLLDKRANPNARALNGFTPLHIACKKNRIKVMELLVKY

GASIQAITESGLTPIHVAAFMGHLNIVLLLLQNGASPDVTNIRGETALHMAARAGQVEVV

RCLLRNGALVDARAREEQTPLHIASRLGKTEIVQLLLQHMAHPDAATTNGYTPLHISARE

GQVDVASVLLEAGAAHSLATKKGFTPLHVAAKYGSLDVAKLLLQRRAAADSAGKNGLTPL

HVAAHYDNQKVALLLLEKGASPHATAKNGYTPLHIAAKKNQMQIASTLLNYGAETNIVTK

QGVTPLHLASQEGHTDMVTLLLDKGANIHMSTKSGLTSLHLAAQEDKVNVADILTKHGAD

QDAHTKLGYTPLIVACHYGNVKMVNFLLKQGANVNAKTKNGYTPLHQAAQQGHTHIINVL

LQHGAKPNATTANGNTALAIAKRLGYISVVDTLKVVTEEVTTTTTTITEKHKLNVPETMT

EVLDVSDEEGDDTMTGDGGEYLRPEDLKELGDDSLPSSQFLDGMNYLRYSLEGGRSDSLR

SFSSDRSHTLSHASYLRDSAVMDDSVVIPSHQVSTLAKEAERNSYRLSWGTENLDNVALS

SSPIHSGFLVIFMVDARGGAMRGCRHNGLRIIIPPRKCTAPTRVTCRLVKRHRLATMPPM

VEGEGLASRLIEVGPSGAQFLGPVIVEIPHFAALRGKERELVVLRSENGDSWKEHFCDYT

EDELNEILNGMDEVLDSPEDLEKKRICRIITRDFPQYFAVVSRIKQDSNLIGPEGGVLSS

TVVPQVQAVFPEGALTKRIRVGLQAQPMHSELVKKILGNKATFSPIVTLEPRRRKFHKPI

TMTIPVPKASSDVMLNGFGGDAPTLRLLCSITGGTTPAQWEDITGTTPLTFVNECVSFTT

NVSARFWLIDCRQIQESVTFASQVYREIICVPYMAKFVVFAKSHDPIEARLRCFCMTDDK

VDKTLEQQENFAEVARSRDVEVLEGKPIYVDCFGNLVPLTKSGQHHIFSFFAFKENRLPL

FVKVRDTTQEPCGRLSFMKEPKSTRGLVHQAICNLNITLPIYTKESESDQEQEEEIDMTS

EKNDETESTETSVLKSHLVNEVPVLASPDLLSEVSEMKQDLIKMTAILTTDVSDKAGSIK

VKELVKAAEEEPGEPFEIVERVKEDLEKVNEILRSGTCTRDESSVQSSRSERGLVEEEWV

IVSDEEIEEARQKAPLEITEYPCVEVRIDKEIKGKVEKDSTGLVNYLTDDLNTCVPLPKE

QLQTVQDKAGKKCEALAVGRSSEKEGKDIPPDETQSTQKQHKPSLGIKKPVRRKLKEKQK

QKEEGLQASAEKAELKKGSSEESLGEDPGLAPEPLPTVKATSPLIEETPIGSIKDKVKAL

QKRVEDEQKGRSKLPIRVKGKEDVPKKTTHRPHPAASPSLKSERHAPGSPSPKTERHSTL

SSSAKTERHPPVSPSSKTEKHSPVSPSAKTERHSPASSSSKTEKHSPVSPSTKTERHSPV

SSTKTERHPPVSPSGKTDKRPPVSPSGRTEKHPPVSPGRTEKRLPVSPSGRTDKHQPVST

AGKTEKHLPVSPSGKTEKQPPVSPTSKTERIEETMSVRELMKAFQSGQDPSKHKTGLFEH

KSAKQKQPQEKGKVRVEKEKGPILTQREAQKTENQTIKRGQRLPVTGTAESKRGVRVSSI

GVKKEDAAGGKEKVLSHKIPEPVQSVPEEESHRESEVPKEKMADEQGDMDLQISPDRKTS

TDFSEVIKQELEDNDKYQQFRLSEETEKAQLHLDQVLTSPFNTTFPLDYMKDEFLPALSL

QSGALDGSSESLKNEGVAGSPCGSLMEGTPQISSEESYKHEGLAETPETSPESLSFSPKK

SEEQTGETKESTKTETTTEIRSEKEHPTTKDITGGSEERGATVTEDSETSTESFQKEATL

GSPKDTSPKRQDDCTGSCSVALAKETPTGLTEEAACDEGQRTFGSSAHKTQTDSEAQEST

ATSDETKALPLPEASVKTDTGTESKPQGVIRSPQGLELALPSRDSEVLSAVADDSLAVSH

KDSLEASPVLEDNSSHKTPDSLEPSPLKESPCRDSLESSPVEPKMKAGIFPSHFPLPAAV

AKTELLTEVASVRSRLLRDPDGSAEDDSLEQTSLMESSGKSPLSPDTPSSEEVSYEVTPK

TTDVSTPKPAVIHECAEEDDSENGEKKRFTPEEEMFKMVTKIKMFDELEQEAKQKRDYKK

EPKQEESSSSSDPDADCSVDVDEPKHTGSGEDESGVPVLVTSESRKVSSSSESEPELAQL

KKGADSGLLPEPVIRVQPPSPLPSSMDSNSSPEEVQFQPVVSKQYTFKMNEDTQEEPGKS

EEEKDSESHLAEDRHAVSTEAEDRSYDKLNRDTDQPKICDGHGCEAMSPSSSARPVSSGL

QSPTGDDVDEQPVIYKESLALQGTHEKDTEGEELDVSRAESPQADCPSESFSSSSSLPHC

LVSEGKELDEDISATSSIQKTEVTKTDETFENLPKDCPSQDSSITTQTDRFSMDVPVSDL

AENDEIYDPQITSPYENVPSQSFFSSEESKTQTDANHTTSFHSSEVYSVTITSPVEDVVV

ASSSSGTVLSKESNFEGQDIKMESQLESTLWEMQSDSVSSSFEPTMSATTTVVGEQISKV

IITKTDVDSDSWSEIREDDEAFEARVKEEEQKIFGLMVDRQSQGTTPDTTPARTPTEEGT

PTSEQNPFLFQEGKLFEMTRSGAIDMTKRSYADESFHFFQIGQESREETLSEDVKEGATG

ADPLPLETSAESLALSESKETVDDEADLLPDSVSEEVEEIPASDAQLNSQMGISASTETP

TKEAVSVGTKDLPTVQTGDIPPLSGVKQISCPDSSEPAVQVQLDFSTLTRSVYSDRGDDS

PDSSPEEQKSVIEIPTAPMENVPFTESKSKIPVRTMPTSTPAPPSAEYESSVSEDFLSSV

DEENKADEAKPKSKLPVKVPLQRVEQQLSDLDTSVQKTVAPQGQDMASIAPDNRSKSESD

ASSLDSKTKCPVKTRSYTETETESRERAEELELESEEGATRPKILTSRLPVKSRSTTSSC

RGGTSPTKESKEHFFDLYRNSIEFFEEISDEASKLVDRLTQSEREQEIVSDDESSSALEV

SVIENLPPVETEHSVPEDIFDTRPIWDESIETLIERIPDENGHDHAEDPQDEQERIEERL

AYIADHLGFSWTELARELDFTEEQIHQIRIENPNSLQDQSQYLLKIWLERDGKHATDTNL

VECLTKINRMDIVHLMETNTEPLQERISHSYAEIEQTITLDHSEGFSVLQEELCTAQHKQ

KEEQAVSKESETCDHPPIVSEEDISVGYSTFQDGVPKTEGDSSSTALFPQTHKEQVQQDF

SGKMQDLPEESSLEYQQEYFVTTPGTETSETQKAMIVPSSPSKTPEEVSTPAEEEKLYLQ

TPTSSERGGSPIIQEPEEPSEHREESSPRKTSLVIVESADNQPETCERLDEDAAFEKGDD

MPEIPPETVTEEEYIDEHGHTVVKKVTRKIIRRYVSSEGTEKEEIMVQGMPQEPVNIEEG

DGYSKVIKRVVLKSDTEQSEDNNE

>sp|Q9Y4A5|TRRAP_HUMAN 1806 SITSVFITK

MAFVATQGATVVDQTTLMKKYLQFVAALTDVNTPDETKLKMMQEVSENFENVTSSPQYST

FLEHIIPRFLTFLQDGEVQFLQEKPAQQLRKLVLEIIHRIPTNEHLRPHTKNVLSVMFRF

LETENEENVLICLRIIIELHKQFRPPITQEIHHFLDFVKQIYKELPKVVNRYFENPQVIP

ENTVPPPEMVGMITTIAVKVNPEREDSETRTHSIIPRGSLSLKVLAELPIIVVLMYQLYK

LNIHNVVAEFVPLIMNTIAIQVSAQARQHKLYNKELYADFIAAQIKTLSFLAYIIRIYQE

LVTKYSQQMVKGMLQLLSNCPAETAHLRKELLIAAKHILTTELRNQFIPCMDKLFDESIL

IGSGYTARETLRPLAYSTLADLVHHVRQHLPLSDLSLAVQLFAKNIDDESLPSSIQTMSC

KLLLNLVDCIRSKSEQESGNGRDVLMRMLEVFVLKFHTIARYQLSAIFKKCKPQSELGAV

EAALPGVPTAPAAPGPAPSPAPVPAPPPPPPPPPPATPVTPAPVPPFEKQGEKDKEDKQT

FQVTDCRSLVKTLVCGVKTITWGITSCKAPGEAQFIPNKQLQPKETQIYIKLVKYAMQAL

DIYQVQIAGNGQTYIRVANCQTVRMKEEKEVLEHFAGVFTMMNPLTFKEIFQTTVPYMVE

RISKNYALQIVANSFLANPTTSALFATILVEYLLDRLPEMGSNVELSNLYLKLFKLVFGS

VSLFAAENEQMLKPHLHKIVNSSMELAQTAKEPYNYFLLLRALFRSIGGGSHDLLYQEFL

PLLPNLLQGLNMLQSGLHKQHMKDLFVELCLTVPVRLSSLLPYLPMLMDPLVSALNGSQT

LVSQGLRTLELCVDNLQPDFLYDHIQPVRAELMQALWRTLRNPADSISHVAYRVLGKFGG

SNRKMLKESQKLHYVVTEVQGPSITVEFSDCKASLQLPMEKAIETALDCLKSANTEPYYR

RQAWEVIKCFLVAMMSLEDNKHALYQLLAHPNFTEKTIPNVIISHRYKAQDTPARKTFEQ

ALTGAFMSAVIKDLRPSALPFVASLIRHYTMVAVAQQCGPFLLPCYQVGSQPSTAMFHSE

ENGSKGMDPLVLIDAIAICMAYEEKELCKIGEVALAVIFDVASIILGSKERACQLPLFSY

IVERLCACCYEQAWYAKLGGVVSIKFLMERLPLTWVLQNQQTFLKALLFVMMDLTGEVSN

GAVAMAKTTLEQLLMRCATPLKDEERAEEIVAAQEKSFHHVTHDLVREVTSPNSTVRKQA

MHSLQVLAQVTGKSVTVIMEPHKEVLQDMVPPKKHLLRHQPANAQIGLMEGNTFCTTLQP

RLFTMDLNVVEHKVFYTELLNLCEAEDSALTKLPCYKSLPSLVPLRIAALNALAACNYLP

QSREKIIAALFKALNSTNSELQEAGEACMRKFLEGATIEVDQIHTHMRPLLMMLGDYRSL

TLNVVNRLTSVTRLFPNSFNDKFCDQMMQHLRKWMEVVVITHKGGQRSDGNESISECGRC

PLSPFCQFEEMKICSAIINLFHLIPAAPQTLVKPLLEVVMKTERAMLIEAGSPFREPLIK

FLTRHPSQTVELFMMEATLNDPQWSRMFMSFLKHKDARPLRDVLAANPNRFITLLLPGGA

QTAVRPGSPSTSTMRLDLQFQAIKIISIIVKNDDSWLASQHSLVSQLRRVWVSENFQERH

RKENMAATNWKEPKLLAYCLLNYCKRNYGDIELLFQLLRAFTGRFLCNMTFLKEYMEEEI

PKNYSIAQKRALFFRFVDFNDPNFGDELKAKVLQHILNPAFLYSFEKGEGEQLLGPPNPE

GDNPESITSVFITKVLDPEKQADMLDSLRIYLLQYATLLVEHAPHHIHDNNKNRNSKLRR

LMTFAWPCLLSKACVDPACKYSGHLLLAHIIAKFAIHKKIVLQVFHSLLKAHAMEARAIV

RQAMAILTPAVPARMEDGHQMLTHWTRKIIVEEGHTVPQLVHILHLIVQHFKVYYPVRHH

LVQHMVSAMQRLGFTPSVTIEQRRLAVDLSEVVIKWELQRIKDQQPDSDMDPNSSGEGVN

SVSSSIKRGLSVDSAQEVKRFRTATGAISAVFGRSQSLPGADSLLAKPIDKQHTDTVVNF

LIRVACQVNDNTNTAGSPGEVLSRRCVNLLKTALRPDMWPKSELKLQWFDKLLMTVEQPN

QVNYGNICTGLEVLSFLLTVLQSPAILSSFKPLQRGIAACMTCGNTKVLRAVHSLLSRLM

SIFPTEPSTSSVASKYEELECLYAAVGKVIYEGLTNYEKATNANPSQLFGTLMILKSACS

NNPSYIDRLISVFMRSLQKMVREHLNPQAASGSTEATSGTSELVMLSLELVKTRLAVMSM

EMRKNFIQAILTSLIEKSPDAKILRAVVKIVEEWVKNNSPMAANQTPTLREKSILLVKMM

TYIEKRFPEDLELNAQFLDLVNYVYRDETLSGSELTAKLEPAFLSGLRCAQPLIRAKFFE

VFDNSMKRRVYERLLYVTCSQNWEAMGNHFWIKQCIELLLAVCEKSTPIGTSCQGAMLPS

ITNVINLADSHDRAAFAMVTHVKQEPRERENSESKEEDVEIDIELAPGDQTSTPKTKELS

EKDIGNQLHMLTNRHDKFLDTLREVKTGALLSAFVQLCHISTTLAEKTWVQLFPRLWKIL

SDRQQHALAGEISPFLCSGSHQVQRDCQPSALNCFVEAMSQCVPPIPIRPCVLKYLGKTH

NLWFRSTLMLEHQAFEKGLSLQIKPKQTTEFYEQESITPPQQEILDSLAELYSLLQEEDM

WAGLWQKRCKYSETATAIAYEQHGFFEQAQESYEKAMDKAKKEHERSNASPAIFPEYQLW

EDHWIRCSKELNQWEALTEYGQSKGHINPYLVLECAWRVSNWTAMKEALVQVEVSCPKEM

AWKVNMYRGYLAICHPEEQQLSFIERLVEMASSLAIREWRRLPHVVSHVHTPLLQAAQQI

IELQEAAQINAGLQPTNLGRNNSLHDMKTVVKTWRNRLPIVSDDLSHWSSIFMWRQHHYQ

GKPTWSGMHSSSIVTAYENSSQHDPSSNNAMLGVHASASAIIQYGKIARKQGLVNVALDI

LSRIHTIPTVPIVDCFQKIRQQVKCYLQLAGVMGKNECMQGLEVIESTNLKYFTKEMTAE

FYALKGMFLAQINKSEEANKAFSAAVQMHDVLVKAWAMWGDYLENIFVKERQLHLGVSAI

TCYLHACRHQNESKSRKYLAKVLWLLSFDDDKNTLADAVDKYCIGVPPIQWLAWIPQLLT

CLVGSEGKLLLNLISQVGRVYPQAVYFPIRTLYLTLKIEQRERYKSDPGPIRATAPMWRC

SRIMHMQRELHPTLLSSLEGIVDQMVWFRENWHEEVLRQLQQGLAKCYSVAFEKSGAVSD

AKITPHTLNFVKKLVSTFGVGLENVSNVSTMFSSAASESLARRAQATAQDPVFQKLKGQF

TTDFDFSVPGSMKLHNLISKLKKWIKILEAKTKQLPKFFLIEEKCRFLSNFSAQTAEVEI

PGEFLMPKPTHYYIKIARFMPRVEIVQKHNTAARRLYIRGHNGKIYPYLVMNDACLTESR

REERVLQLLRLLNPCLEKRKETTKRHLFFTVPRVVAVSPQMRLVEDNPSSLSLVEIYKQR

CAKKGIEHDNPISRYYDRLATVQARGTQASHQVLRDILKEVQSNMVPRSMLKEWALHTFP

NATDYWTFRKMFTIQLALIGFAEFVLHLNRLNPEMLQIAQDTGKLNVAYFRFDINDATGD

LDANRPVPFRLTPNISEFLTTIGVSGPLTASMIAVARCFAQPNFKVDGILKTVLRDEIIA

WHKKTQEDTSSPLSAAGQPENMDSQQLVSLVQKAVTAIMTRLHNLAQFEGGESKVNTLVA

AANSLDNLCRMDPAWHPWL

>sp|Q9Y4A5|TRRAP_HUMAN 378 TLADLVHHV

MAFVATQGATVVDQTTLMKKYLQFVAALTDVNTPDETKLKMMQEVSENFENVTSSPQYST

FLEHIIPRFLTFLQDGEVQFLQEKPAQQLRKLVLEIIHRIPTNEHLRPHTKNVLSVMFRF

LETENEENVLICLRIIIELHKQFRPPITQEIHHFLDFVKQIYKELPKVVNRYFENPQVIP

ENTVPPPEMVGMITTIAVKVNPEREDSETRTHSIIPRGSLSLKVLAELPIIVVLMYQLYK

LNIHNVVAEFVPLIMNTIAIQVSAQARQHKLYNKELYADFIAAQIKTLSFLAYIIRIYQE

LVTKYSQQMVKGMLQLLSNCPAETAHLRKELLIAAKHILTTELRNQFIPCMDKLFDESIL

IGSGYTARETLRPLAYSTLADLVHHVRQHLPLSDLSLAVQLFAKNIDDESLPSSIQTMSC

KLLLNLVDCIRSKSEQESGNGRDVLMRMLEVFVLKFHTIARYQLSAIFKKCKPQSELGAV

EAALPGVPTAPAAPGPAPSPAPVPAPPPPPPPPPPATPVTPAPVPPFEKQGEKDKEDKQT

FQVTDCRSLVKTLVCGVKTITWGITSCKAPGEAQFIPNKQLQPKETQIYIKLVKYAMQAL

DIYQVQIAGNGQTYIRVANCQTVRMKEEKEVLEHFAGVFTMMNPLTFKEIFQTTVPYMVE

RISKNYALQIVANSFLANPTTSALFATILVEYLLDRLPEMGSNVELSNLYLKLFKLVFGS

VSLFAAENEQMLKPHLHKIVNSSMELAQTAKEPYNYFLLLRALFRSIGGGSHDLLYQEFL

PLLPNLLQGLNMLQSGLHKQHMKDLFVELCLTVPVRLSSLLPYLPMLMDPLVSALNGSQT

LVSQGLRTLELCVDNLQPDFLYDHIQPVRAELMQALWRTLRNPADSISHVAYRVLGKFGG

SNRKMLKESQKLHYVVTEVQGPSITVEFSDCKASLQLPMEKAIETALDCLKSANTEPYYR

RQAWEVIKCFLVAMMSLEDNKHALYQLLAHPNFTEKTIPNVIISHRYKAQDTPARKTFEQ

ALTGAFMSAVIKDLRPSALPFVASLIRHYTMVAVAQQCGPFLLPCYQVGSQPSTAMFHSE

ENGSKGMDPLVLIDAIAICMAYEEKELCKIGEVALAVIFDVASIILGSKERACQLPLFSY

IVERLCACCYEQAWYAKLGGVVSIKFLMERLPLTWVLQNQQTFLKALLFVMMDLTGEVSN

GAVAMAKTTLEQLLMRCATPLKDEERAEEIVAAQEKSFHHVTHDLVREVTSPNSTVRKQA

MHSLQVLAQVTGKSVTVIMEPHKEVLQDMVPPKKHLLRHQPANAQIGLMEGNTFCTTLQP

RLFTMDLNVVEHKVFYTELLNLCEAEDSALTKLPCYKSLPSLVPLRIAALNALAACNYLP

QSREKIIAALFKALNSTNSELQEAGEACMRKFLEGATIEVDQIHTHMRPLLMMLGDYRSL

TLNVVNRLTSVTRLFPNSFNDKFCDQMMQHLRKWMEVVVITHKGGQRSDGNESISECGRC

PLSPFCQFEEMKICSAIINLFHLIPAAPQTLVKPLLEVVMKTERAMLIEAGSPFREPLIK

FLTRHPSQTVELFMMEATLNDPQWSRMFMSFLKHKDARPLRDVLAANPNRFITLLLPGGA

QTAVRPGSPSTSTMRLDLQFQAIKIISIIVKNDDSWLASQHSLVSQLRRVWVSENFQERH

RKENMAATNWKEPKLLAYCLLNYCKRNYGDIELLFQLLRAFTGRFLCNMTFLKEYMEEEI

PKNYSIAQKRALFFRFVDFNDPNFGDELKAKVLQHILNPAFLYSFEKGEGEQLLGPPNPE

GDNPESITSVFITKVLDPEKQADMLDSLRIYLLQYATLLVEHAPHHIHDNNKNRNSKLRR

LMTFAWPCLLSKACVDPACKYSGHLLLAHIIAKFAIHKKIVLQVFHSLLKAHAMEARAIV

RQAMAILTPAVPARMEDGHQMLTHWTRKIIVEEGHTVPQLVHILHLIVQHFKVYYPVRHH

LVQHMVSAMQRLGFTPSVTIEQRRLAVDLSEVVIKWELQRIKDQQPDSDMDPNSSGEGVN

SVSSSIKRGLSVDSAQEVKRFRTATGAISAVFGRSQSLPGADSLLAKPIDKQHTDTVVNF

LIRVACQVNDNTNTAGSPGEVLSRRCVNLLKTALRPDMWPKSELKLQWFDKLLMTVEQPN

QVNYGNICTGLEVLSFLLTVLQSPAILSSFKPLQRGIAACMTCGNTKVLRAVHSLLSRLM

SIFPTEPSTSSVASKYEELECLYAAVGKVIYEGLTNYEKATNANPSQLFGTLMILKSACS

NNPSYIDRLISVFMRSLQKMVREHLNPQAASGSTEATSGTSELVMLSLELVKTRLAVMSM

EMRKNFIQAILTSLIEKSPDAKILRAVVKIVEEWVKNNSPMAANQTPTLREKSILLVKMM

TYIEKRFPEDLELNAQFLDLVNYVYRDETLSGSELTAKLEPAFLSGLRCAQPLIRAKFFE

VFDNSMKRRVYERLLYVTCSQNWEAMGNHFWIKQCIELLLAVCEKSTPIGTSCQGAMLPS

ITNVINLADSHDRAAFAMVTHVKQEPRERENSESKEEDVEIDIELAPGDQTSTPKTKELS

EKDIGNQLHMLTNRHDKFLDTLREVKTGALLSAFVQLCHISTTLAEKTWVQLFPRLWKIL

SDRQQHALAGEISPFLCSGSHQVQRDCQPSALNCFVEAMSQCVPPIPIRPCVLKYLGKTH

NLWFRSTLMLEHQAFEKGLSLQIKPKQTTEFYEQESITPPQQEILDSLAELYSLLQEEDM

WAGLWQKRCKYSETATAIAYEQHGFFEQAQESYEKAMDKAKKEHERSNASPAIFPEYQLW

EDHWIRCSKELNQWEALTEYGQSKGHINPYLVLECAWRVSNWTAMKEALVQVEVSCPKEM

AWKVNMYRGYLAICHPEEQQLSFIERLVEMASSLAIREWRRLPHVVSHVHTPLLQAAQQI

IELQEAAQINAGLQPTNLGRNNSLHDMKTVVKTWRNRLPIVSDDLSHWSSIFMWRQHHYQ

GKPTWSGMHSSSIVTAYENSSQHDPSSNNAMLGVHASASAIIQYGKIARKQGLVNVALDI

LSRIHTIPTVPIVDCFQKIRQQVKCYLQLAGVMGKNECMQGLEVIESTNLKYFTKEMTAE

FYALKGMFLAQINKSEEANKAFSAAVQMHDVLVKAWAMWGDYLENIFVKERQLHLGVSAI

TCYLHACRHQNESKSRKYLAKVLWLLSFDDDKNTLADAVDKYCIGVPPIQWLAWIPQLLT

CLVGSEGKLLLNLISQVGRVYPQAVYFPIRTLYLTLKIEQRERYKSDPGPIRATAPMWRC

SRIMHMQRELHPTLLSSLEGIVDQMVWFRENWHEEVLRQLQQGLAKCYSVAFEKSGAVSD

AKITPHTLNFVKKLVSTFGVGLENVSNVSTMFSSAASESLARRAQATAQDPVFQKLKGQF

TTDFDFSVPGSMKLHNLISKLKKWIKILEAKTKQLPKFFLIEEKCRFLSNFSAQTAEVEI

PGEFLMPKPTHYYIKIARFMPRVEIVQKHNTAARRLYIRGHNGKIYPYLVMNDACLTESR

REERVLQLLRLLNPCLEKRKETTKRHLFFTVPRVVAVSPQMRLVEDNPSSLSLVEIYKQR

CAKKGIEHDNPISRYYDRLATVQARGTQASHQVLRDILKEVQSNMVPRSMLKEWALHTFP

NATDYWTFRKMFTIQLALIGFAEFVLHLNRLNPEMLQIAQDTGKLNVAYFRFDINDATGD

LDANRPVPFRLTPNISEFLTTIGVSGPLTASMIAVARCFAQPNFKVDGILKTVLRDEIIA

WHKKTQEDTSSPLSAAGQPENMDSQQLVSLVQKAVTAIMTRLHNLAQFEGGESKVNTLVA

AANSLDNLCRMDPAWHPWL

>sp|Q709C8|VP13C_HUMAN 916 EIKEVILEF

MVLESVVADLLNRFLGDYVENLNKSQLKLGIWGGNVALDNLQIKENALSELDVPFKVKAG

QIDKLTLKIPWKNLYGEAVVATLEGLYLLVVPGASIKYDAVKEEKSLQDVKQKELSRIEE

ALQKAAEKGTHSGEFIYGLENFVYKDIKPGRKRKKHKKHFKKPFKGLDRSKDKPKEAKKD

TFVEKLATQVIKNVQVKITDIHIKYEDDVTDPKRPLSFGVTLGELSLLTANEHWTPCILN

EADKIIYKLIRLDSLSAYWNVNCSMSYQRSREQILDQLKNEILTSGNIPPNYQYIFQPIS

ASAKLYMNPYAESELKTPKLDCNIEIQNIAIELTKPQYLSMIDLLESVDYMVRNAPYRKY

KPYLPLHTNGRRWWKYAIDSVLEVHIRRYTQMWSWSNIKKHRQLLKSYKIAYKNKLTQSK

VSEEIQKEIQDLEKTLDVFNIILARQQAQVEVIRSGQKLRKKSADTGEKRGGWFSGLWGK

KESKKKDEESLIPETIDDLMTPEEKDKLFTAIGYSESTHNLTLPKQYVAHIMTLKLVSTS

VTIRENKNIPEILKIQIIGLGTQVSQRPGAQALKVEAKLEHWYITGLRQQDIVPSLVASI

GDTTSSLLKIKFETNPEDSPADQTLIVQSQPVEVIYDAKTVNAVVEFFQSNKGLDLEQIT

SATLMKLEEIKERTATGLTHIIETRKVLDLRINLKPSYLVVPQTGFHHEKSDLLILDFGT

FQLNSKDQGLQKTTNSSLEEIMDKAYDKFDVEIKNVQLLFARAEETWKKCRFQHPSTMHI

LQPMDIHVELAKAMVEKDIRMARFKVSGGLPLMHVRISDQKMKDVLYLMNSIPLPQKSSA

QSPERQVSSIPIISGGTKGLLGTSLLLDTVESESDDEYFDAEDGEPQTCKSMKGSELKKA

AEVPNEELINLLLKFEIKEVILEFTKQQKEEDTILVFNVTQLGTEATMRTFDLTVVSYLK

KISLDYHEIEGSKRKPLHLISSSDKPGLDLLKVEYIKADKNGPSFQTAFGKTEQTVKVAF

SSLNLLLQTQALVASINYLTTIIPSDDQSISVAKEVQISTEKQQKNSTLPKAIVSSRDSD

IIDFRLFAKLNAFCVIVCNEKNNIAEIKIQGLDSSLSLQSRKQSLFARLENIIVTDVDPK

TVHKKAVSIMGNEVFRFNLDLYPDATEGDLYTDMSKVDGVLSLNVGCIQIVYLHKFLMSL

LNFLNNFQTAKESLSAATAQAAERAATSVKDLAQRSFRVSINIDLKAPVIVIPQSSISTN

AVVVDLGLIRVHNQFSLVSDEDYLNPPVIDRMDVQLTKLTLYRTVIQPGIYHPDIQLLHP

INLEFLVNRNLAASWYHKVPVVEIKGHLDSMNVSLNQEDLNLLFRILTENLCEGTEDLDK

VKPRVQETGEIKEPLEISISQDVHDSKNTLTTGVEEIRSVDIINMLLNFEIKEVVVTLMK

KSEKKGRPLHELNVLQLGMEAKVKTYDMTAKAYLKKISMQCFDFTDSKGEPLHIINSSNV

TDEPLLKMLLTKADSDGPEFKTIHDSTKQRLKVSFASLDLVLHLEALLSFMDFLSSAAPF

SEPSSSEKESELKPLVGESRSIAVKAVSSNISQKDVFDLKITAELNAFNVFVCDQKCNIA

DIKIHGMDASISVKPKQTDVFARLKDIIVMNVDLQSIHKKAVSILGDEVFRFQLTLYPDA

TEGEAYADMSKVDGKLSFKVGCIQIVYVHKFFMSLLNFLNNFQTAKEALSTATVQAAERA

ASSMKDLAQKSFRLLMDINLKAPVIIIPQSSVSPNAVIADLGLIRVENKFSLVPMEHYSL

PPVIDKMNIELTQLKLSRTILQASLPQNDIEILKPVNMLLSIQRNLAAAWYVQIPGMEIK

GKLKPMQVALSEDDLTVLMKILLENLGEASSQPSPTQSVQETVRVRKVDVSSVPDHLKEQ

EDWTDSKLSMNQIVSLQFDFHFESLSIILYNNDINQESGVAFHNDSFQLGELRLHLMASS

GKMFKDGSMNVSVKLKTCTLDDLREGIERATSRMIDRKNDQDNNSSMIDISYKQDKNGSQ

IDAVLDKLYVCASVEFLMTVADFFIKAVPQSPENVAKETQILPRQTATGKVKIEKDDSVR

PNMTLKAMITDPEVVFVASLTKADAPALTASFQCNLSLSTSKLEQMMEASVRDLKVLACP

FLREKRGKNITTVLQPCSLFMEKCTWASGKQNINIMVKEFIIKISPIILNTVLTIMAALS

PKTKEDGSKDTSKEMENLWGIKSINDYNTWFLGVDTATEITESFKGIEHSLIEENCGVVV

ESIQVTLECGLGHRTVPLLLAESKFSGNIKNWTSLMAAVADVTLQVHYYNEIHAVWEPLI

ERVEGKRQWNLRLDVKKNPVQDKSLLPGDDFIPEPQMAIHISSGNTMNITISKSCLNVFN

NLAKGFSEGTASTFDYSLKDRAPFTVKNAVGVPIKVKPNCNLRVMGFPEKSDIFDVDAGQ

NLELEYASMVPSSQGNLSILSRQESSFFTLTIVPHGYTEVANIPVARPGRRLYNVRNPNA

SHSDSVLVQIDATEGNKVITLRSPLQIKNHFSIAFIIYKFVKNVKLLERIGIARPEEEFH

VPLDSYRCQLFIQPAGILEHQYKESTTYISWKEELHRSREVRCMLQCPSVEVSFLPLIVN

TVALPDELSYICTHGEDWDVAYIIHLYPSLTLRNLLPYSLRYLLEGTAETHELAEGSTAD

VLHSRISGEIMELVLVKYQGKNWNGHFRIRDTLPEFFPVCFSSDSTEVTTVDLSVHVRRI

GSRMVLSVFSPYWLINKTTRVLQYRSEDIHVKHPADFRDIILFSFKKKNIFTKNKVQLKI

STSAWSSSFSLDTVGSYGCVKCPANNMEYLVGVSIKMSSFNLSRIVTLTPFCTIANKSSL

ELEVGEIASDGSMPTNKWNYIASSECLPFWPESLSGKLCVRVVGCEGSSKPFFYNRQDNG

TLLSLEDLNGGILVDVNTAEHSTVITFSDYHEGSAPALIMNHTPWDILTYKQSGSPEEMV

LLPRQARLFAWADPTGTRKLTWTYAANVGEHDLLKDGCGQFPYDANIQIHWVSFLDGRQR

VLLFTDDVALVSKALQAEEMEQADYEITLSLHSLGLSLVNNESKQEVSYIGITSSGVVWE

VKPKQKWKPFSQKQIILLEQSYQKHQISRDHGWIKLDNNFEVNFDKDPMEMRLPIRSPIK

RDFLSGIQIEFKQSSHQRSLRARLYWLQVDNQLPGAMFPVVFHPVAPPKSIALDSEPKPF

IDVSVITRFNEYSKVLQFKYFMVLIQEMALKIDQGFLGAIIALFTPTTDPEAERRRTKLI

QQDIDALNAELMETSMTDMSILSFFEHFHISPVKLHLSLSLGSGGEESDKEKQEMFAVHS

VNLLLKSIGATLTDVDDLIFKLAYYEIRYQFYKRDQLIWSVVRHYSEQFLKQMYVLVLGL

DVLGNPFGLIRGLSEGVEALFYEPFQGAVQGPEEFAEGLVIGVRSLFGHTVGGAAGVVSR

ITGSVGKGLAAITMDKEYQQKRREELSRQPRDFGDSLARGGKGFLRGVVGGVTGIITKPV

EGAKKEGAAGFFKGIGKGLVGAVARPTGGIVDMASSTFQGIQRAAESTEEVSSLRPPRLI

HEDGIIRPYDRQESEGSDLLENHIKKLEGETYRYHCAIPGSKKTILMVTNRRVLCIKEVE

ILGLMCVDWQCPFEDFVFPPSVSENVLKISVKEQGLFHKKDSANQGCVRKVYLKDTATAE

RACNAIEDAQSTRQQQKLMKQSSVRLLRPQLPS

>sp|Q9NRC6|SPTN5_HUMAN 739 ARLQTALL

MAGQPHSPRELLGAAGHRSRRPSTELRVPPSPSLTMDSQYETGHIRKLQARHMQMQEKTF

TKWINNVFQCGQAGIKIRNLYTELADGIHLLRLLELISGEALPPPSRGRLRVHFLENSSR

ALAFLRAKVPVPLIGPENIVDGDQTLILGLIWVIILRFQISHISLDKEEFGASAALLSTK

EALLVWCQRKTASYTNVNITDFSRSWSDGLGFNALIHAHRPDLLDYGSLRPDRPLHNLAF

AFLVAEQELGIAQLLDPEDVAAAQPDERSIMTYVSLYYHYCSRLHQGQTVQRRLTKILLQ

LQETELLQTQYEQLVADLLRWIAEKQMQLEARDFPDSLPAMRQLLAAFTIFRTQEKPPRL

QQRGAAEALLFRLQTALQAQNRRPFLPHEGLGLAELSQCWAGLEWAEAARSQALQQRLLQ

LQRLETLARRFQRKAALRESFLKDAEQVLDQARAPPASLATVEAAVQRLGMLEAGILPQE

GRFQALAEIADILRQEQYHSWADVARRQEEVTVRWQRLLQHLQGQRKQVADMQAVLSLLQ

EVEAASHQLEELQEPARSTACGQQLAEVVELLQRHDLLEAQVSAHGAHVSHLAQQTAELD

SSLGTSVEVLQAKARTLAQLQQSLVALVRARRALLEQTLQRAEFLRNCEEEEAWLKECGQ

RVGNAALGRDLSQIAGALQKHKALEAEVHRHQAVCVDLVRRGRDLSARRPPTQPDPGERA

EAVQGGWQLLQTRVVGRGARLQTALLVLQYFADAAEAASWLRERRSSLERASCGQDQAAA

ETLLRRHVRLERVLRAFAAELRRLEEQGRAASARASLFTVNSALSPPGESLRNPGPWSEA

SCHPGPGDAWKMALPAEPDPDFDPNTILQTQDHLSQDYESLRALAQLRRARLEEAMALFG

FCSSCGELQLWLEKQTVLLQRVQPQADTLEVMQLKYENFLTALAVGKGLWAEVSSSAEQL

RQRYPGNSTQIQRQQEELSQRWGQLEALKREKAVQLAHSVEVCSFLQECGPTQVQLRDVL

LQLEALQPGSSEDTRHALQLAQKKTLVLERRVYFLQSVVVKVEEPGYAESQPLQGQVETL

QGLLKQVQEQVAQRARRQAETQARQSFLQESQQLLLWAESVQAQLRSKEVSVDVASAQRL

LREHQDLLEEIHLWQERLQQLDAQSQPMAALDCPDSQEVPNTLRVLGQQGQELKVLWEQR

QQWLQEGLELQKFGREVDGFTATCANHQAWLHLDNLGEDVREALSLLQQHREFGRLLSTL

GPRAEALRAHGEKLVQSQHPAAHTVREQLQSIQAQWTRLQGRSEQRRRQLLASLQLQEWK

QDVAELMQWMEEKGLMAAHEPSGARRNILQTLKRHEAAESELLATRRHVEALQQVGRELL

SRRPCGQEDIQTRLQGLRSKWEALNRKMTERGDELQQAGQQEQLLRQLQDAKEQLEQLEG

ALQSSETGQDLRSSQRLQKRHQQLESESRTLAAKMAALASMAHGMAASPAILEETQKHLR

RLELLQGHLAIRGLQLQASVELHQFCHLSNMELSWVAEHMPHGSPTSYTECLNGAQSLHR

KHKELQVEVKAHQGQVQRVLSSGRSLAASGHPQAQHIVEQCQELEGHWAELERACEARAQ

CLQQAVTFQQYFLDVSELEGWVEEKRPLVSSRDYGRDEAATLRLINKHQALQEELAIYWS

SMEELDQTAQTLTGPEVPEQQRVVQERLREQLRALQELAATRDRELEGTLRLHEFLREAE

DLQGWLASQKQAAKGGESLGEDPEHALHLCTKFAKFQHQVEMGSQRVAACRLLAESLLER

GHSAGPMVRQRQQDLQTAWSELWELTQARGHALRDTETTLRVHRDLLEVLTQVQEKATSL

PNNVARDLCGLEAQLRSHQGLERELVGTERQLQELLETAGRVQKLCPGPQAHAVQQRQQA

VTQAWAVLQRRMEQRRAQLERARLLARFRTAVRDYASWAARVRQDLQVEESSQEPSSGPL

KLSAHQWLRAELEAREKLWQQATQLGQQALLAAGTPTKEVQEELRALQDQRDQVYQTWAR

KQERLQAEQQEQLFLRECGRLEEILAAQEVSLKTSALGSSVEEVEQLIRKHEVFLKVLTA

QDKKEAALRERLKTLRRPRVRDRLPILLQRRMRVKELAESRGHALHASLLMASFTQAATQ

AEDWIQAWAQQLKEPVPPGDLRDKLKPLLKHQAFEAEVQAHEEVMTSVAKKGEALLAQSH

PRAGEVSQRLQGLRKHWEDLRQAMALRGQELEDRRNFLEFLQRVDLAEAWIQEKEVKMNV

GDLGQDLEHCLQLRRRLREFRGNSAGDTVGDACIRSISDLSLQLKNRDPEEVKIICQRRS

QLNNRWASFHGNLLRYQQQLEGALEIHVLSRELDNVTKRIQEKEALIQALDCGKDLESVQ

RLLRKHEELEREVHPIQAQVESLEREVGRLCQRSPEAAHGLRHRQQEVAESWWQLRSRAQ

KRREALDALHQAQKLQAMLQELLVSAQRLRAQMDTSPAPRSPVEARRMLEEHQECKAELD

SWTDSISLARSTGQQLLTAGHPFSSDIRQVLAGLEQELSSLEGAWQEHQLQLQQALELQL

FLSSVEKMERWLCSKEDSLASEGLWDPLAPMEPLLWKHKMLEWDLEVQAGKISALEATAR

GLHQGGHPEAQSALGRCQAMLLRKEALFRQAGTRRHRLEELRQLQAFLQDSQEVAAWLRE

KNLVALEEGLLDTAMLPAQLQKQQNFQAELDASMHQQQELQREGQRLLQGGHPASEAIQE

RLEELGALWGELQDNSQKKVAKLQKACEALRLRRSMEELENWLEPIEVELRAPTVGQALP

GVGELLGTQRELEAAVDKKARQAEALLGQAQAFVREGHCLAQDVEEQARRLLQRFKSLRE

PLQERRTALEARSLLLKFFRDADEEMAWVQEKLPLAAAQDYGQSLSAVRHLQEQHQNLES

EMSSHEALTRVVLGTGYKLVQAGHFAAHEVAARVQQLEKAMAHLRAEAARRRLLLQQAQE

AQQFLTELLEAGSWLAERGHVLDSEDMGHSAEATQALLRRLEATKRDLEAFSPRIERLQQ

TAALLESRKNPESPKVLAQLQAVREAHAELLRRAEARGHGLQEQLQLHQLERETLLLDAW

LTTKAATAESQDYGQDLEGVKVLEEKFDAFRKEVQSLGQAKVYALRKLAGTLERGAPRRY

PHIQAQRSRIEAAWERLDQAIKARTENLAAAHEVHSFQQAAAELQGRMQEKTALMKGEDG

GHSLSSVRTLQQQHRRLERELEAMEKEVARLQTEACRLGQLHPAAPGGLAKVQEAWATLQ

AKAQERGQWLAQAAQGHAFLGRCQELLAWAQERQELASSEELAEDVAGAEQLLGQHEELG

QEIRECRLQAQDLRQEGQQLVDNSHFMSAEVTECLQELEGRLQELEEAWALRWQRCAESW

GLQKLRQRLEQAEAWLACWEGLLLKPDYGHSVSDVELLLHRHQDLEKLLAAQEEKFAQMQ

KTEMEQELLLQPQELKPGRAGSSLTSFQWRPSGHQGLGAQLAETRDPQDAKGTPTMEGSL

EFKQHLLPGGRQPSSSSWDSCRGTLQGSSLSLFLDERMAAEKVASIALLDLTGARCERLR

GRHGRKHTFSLRLTSGAEILFAAPSEEQAESWWRALGSTAAQSLSPKLKAKPVSSLNECT

TKDARPGCLLRSDP

>sp|Q9NRC6|SPTN5_HUMAN 739 ARLQTALLV

MAGQPHSPRELLGAAGHRSRRPSTELRVPPSPSLTMDSQYETGHIRKLQARHMQMQEKTF

TKWINNVFQCGQAGIKIRNLYTELADGIHLLRLLELISGEALPPPSRGRLRVHFLENSSR

ALAFLRAKVPVPLIGPENIVDGDQTLILGLIWVIILRFQISHISLDKEEFGASAALLSTK

EALLVWCQRKTASYTNVNITDFSRSWSDGLGFNALIHAHRPDLLDYGSLRPDRPLHNLAF

AFLVAEQELGIAQLLDPEDVAAAQPDERSIMTYVSLYYHYCSRLHQGQTVQRRLTKILLQ

LQETELLQTQYEQLVADLLRWIAEKQMQLEARDFPDSLPAMRQLLAAFTIFRTQEKPPRL

QQRGAAEALLFRLQTALQAQNRRPFLPHEGLGLAELSQCWAGLEWAEAARSQALQQRLLQ

LQRLETLARRFQRKAALRESFLKDAEQVLDQARAPPASLATVEAAVQRLGMLEAGILPQE

GRFQALAEIADILRQEQYHSWADVARRQEEVTVRWQRLLQHLQGQRKQVADMQAVLSLLQ

EVEAASHQLEELQEPARSTACGQQLAEVVELLQRHDLLEAQVSAHGAHVSHLAQQTAELD

SSLGTSVEVLQAKARTLAQLQQSLVALVRARRALLEQTLQRAEFLRNCEEEEAWLKECGQ

RVGNAALGRDLSQIAGALQKHKALEAEVHRHQAVCVDLVRRGRDLSARRPPTQPDPGERA

EAVQGGWQLLQTRVVGRGARLQTALLVLQYFADAAEAASWLRERRSSLERASCGQDQAAA

ETLLRRHVRLERVLRAFAAELRRLEEQGRAASARASLFTVNSALSPPGESLRNPGPWSEA

SCHPGPGDAWKMALPAEPDPDFDPNTILQTQDHLSQDYESLRALAQLRRARLEEAMALFG

FCSSCGELQLWLEKQTVLLQRVQPQADTLEVMQLKYENFLTALAVGKGLWAEVSSSAEQL

RQRYPGNSTQIQRQQEELSQRWGQLEALKREKAVQLAHSVEVCSFLQECGPTQVQLRDVL

LQLEALQPGSSEDTRHALQLAQKKTLVLERRVYFLQSVVVKVEEPGYAESQPLQGQVETL

QGLLKQVQEQVAQRARRQAETQARQSFLQESQQLLLWAESVQAQLRSKEVSVDVASAQRL

LREHQDLLEEIHLWQERLQQLDAQSQPMAALDCPDSQEVPNTLRVLGQQGQELKVLWEQR

QQWLQEGLELQKFGREVDGFTATCANHQAWLHLDNLGEDVREALSLLQQHREFGRLLSTL

GPRAEALRAHGEKLVQSQHPAAHTVREQLQSIQAQWTRLQGRSEQRRRQLLASLQLQEWK

QDVAELMQWMEEKGLMAAHEPSGARRNILQTLKRHEAAESELLATRRHVEALQQVGRELL

SRRPCGQEDIQTRLQGLRSKWEALNRKMTERGDELQQAGQQEQLLRQLQDAKEQLEQLEG

ALQSSETGQDLRSSQRLQKRHQQLESESRTLAAKMAALASMAHGMAASPAILEETQKHLR

RLELLQGHLAIRGLQLQASVELHQFCHLSNMELSWVAEHMPHGSPTSYTECLNGAQSLHR

KHKELQVEVKAHQGQVQRVLSSGRSLAASGHPQAQHIVEQCQELEGHWAELERACEARAQ

CLQQAVTFQQYFLDVSELEGWVEEKRPLVSSRDYGRDEAATLRLINKHQALQEELAIYWS

SMEELDQTAQTLTGPEVPEQQRVVQERLREQLRALQELAATRDRELEGTLRLHEFLREAE

DLQGWLASQKQAAKGGESLGEDPEHALHLCTKFAKFQHQVEMGSQRVAACRLLAESLLER

GHSAGPMVRQRQQDLQTAWSELWELTQARGHALRDTETTLRVHRDLLEVLTQVQEKATSL

PNNVARDLCGLEAQLRSHQGLERELVGTERQLQELLETAGRVQKLCPGPQAHAVQQRQQA

VTQAWAVLQRRMEQRRAQLERARLLARFRTAVRDYASWAARVRQDLQVEESSQEPSSGPL

KLSAHQWLRAELEAREKLWQQATQLGQQALLAAGTPTKEVQEELRALQDQRDQVYQTWAR

KQERLQAEQQEQLFLRECGRLEEILAAQEVSLKTSALGSSVEEVEQLIRKHEVFLKVLTA

QDKKEAALRERLKTLRRPRVRDRLPILLQRRMRVKELAESRGHALHASLLMASFTQAATQ

AEDWIQAWAQQLKEPVPPGDLRDKLKPLLKHQAFEAEVQAHEEVMTSVAKKGEALLAQSH

PRAGEVSQRLQGLRKHWEDLRQAMALRGQELEDRRNFLEFLQRVDLAEAWIQEKEVKMNV

GDLGQDLEHCLQLRRRLREFRGNSAGDTVGDACIRSISDLSLQLKNRDPEEVKIICQRRS

QLNNRWASFHGNLLRYQQQLEGALEIHVLSRELDNVTKRIQEKEALIQALDCGKDLESVQ

RLLRKHEELEREVHPIQAQVESLEREVGRLCQRSPEAAHGLRHRQQEVAESWWQLRSRAQ

KRREALDALHQAQKLQAMLQELLVSAQRLRAQMDTSPAPRSPVEARRMLEEHQECKAELD

SWTDSISLARSTGQQLLTAGHPFSSDIRQVLAGLEQELSSLEGAWQEHQLQLQQALELQL

FLSSVEKMERWLCSKEDSLASEGLWDPLAPMEPLLWKHKMLEWDLEVQAGKISALEATAR

GLHQGGHPEAQSALGRCQAMLLRKEALFRQAGTRRHRLEELRQLQAFLQDSQEVAAWLRE

KNLVALEEGLLDTAMLPAQLQKQQNFQAELDASMHQQQELQREGQRLLQGGHPASEAIQE

RLEELGALWGELQDNSQKKVAKLQKACEALRLRRSMEELENWLEPIEVELRAPTVGQALP

GVGELLGTQRELEAAVDKKARQAEALLGQAQAFVREGHCLAQDVEEQARRLLQRFKSLRE

PLQERRTALEARSLLLKFFRDADEEMAWVQEKLPLAAAQDYGQSLSAVRHLQEQHQNLES

EMSSHEALTRVVLGTGYKLVQAGHFAAHEVAARVQQLEKAMAHLRAEAARRRLLLQQAQE

AQQFLTELLEAGSWLAERGHVLDSEDMGHSAEATQALLRRLEATKRDLEAFSPRIERLQQ

TAALLESRKNPESPKVLAQLQAVREAHAELLRRAEARGHGLQEQLQLHQLERETLLLDAW

LTTKAATAESQDYGQDLEGVKVLEEKFDAFRKEVQSLGQAKVYALRKLAGTLERGAPRRY

PHIQAQRSRIEAAWERLDQAIKARTENLAAAHEVHSFQQAAAELQGRMQEKTALMKGEDG

GHSLSSVRTLQQQHRRLERELEAMEKEVARLQTEACRLGQLHPAAPGGLAKVQEAWATLQ

AKAQERGQWLAQAAQGHAFLGRCQELLAWAQERQELASSEELAEDVAGAEQLLGQHEELG

QEIRECRLQAQDLRQEGQQLVDNSHFMSAEVTECLQELEGRLQELEEAWALRWQRCAESW

GLQKLRQRLEQAEAWLACWEGLLLKPDYGHSVSDVELLLHRHQDLEKLLAAQEEKFAQMQ

KTEMEQELLLQPQELKPGRAGSSLTSFQWRPSGHQGLGAQLAETRDPQDAKGTPTMEGSL

EFKQHLLPGGRQPSSSSWDSCRGTLQGSSLSLFLDERMAAEKVASIALLDLTGARCERLR

GRHGRKHTFSLRLTSGAEILFAAPSEEQAESWWRALGSTAAQSLSPKLKAKPVSSLNECT

TKDARPGCLLRSDP

>sp|Q96T58|MINT_HUMAN 3541 SEGGPPLRI

MVRETRHLWVGNLPENVREEKIIEHFKRYGRVESVKILPKRGSEGGVAAFVDFVDIKSAQ

KAHNSVNKMGDRDLRTDYNEPGTIPSAARGLDDTVSIASRSREVSGFRGGGGGPAYGPPP

SLHAREGRYERRLDGASDNRERAYEHSAYGHHERGTGGFDRTRHYDQDYYRDPRERTLQH

GLYYASRSRSPNRFDAHDPRYEPRAREQFTLPSVVHRDIYRDDITREVRGRRPERNYQHS

RSRSPHSSQSRNQSPQRLASQASRPTRSPSGSGSRSRSSSSDSISSSSSTSSDSSDSSSS

SSDDSPARSVQSAAVPAPTSQLLSSLEKDEPRKSFGIKVQNLPVRSTDTSLKDGLFHEFK

KFGKVTSVQIHGTSEERYGLVFFRQQEDQEKALTASKGKLFFGMQIEVTAWIGPETESEN

EFRPLDERIDEFHPKATRTLFIGNLEKTTTYHDLRNIFQRFGEIVDIDIKKVNGVPQYAF

LQYCDIASVCKAIKKMDGEYLGNNRLKLGFGKSMPTNCVWLDGLSSNVSDQYLTRHFCRY

GPVVKVVFDRLKGMALVLYNEIEYAQAAVKETKGRKIGGNKIKVDFANRESQLAFYHCME

KSGQDIRDFYEMLAERREERRASYDYNQDRTYYESVRTPGTYPEDSRRDYPARGREFYSE

WETYQGDYYESRYYDDPREYRDYRNDPYEQDIREYSYRQRERERERERFESDRDRDHERR

PIERSQSPVHLRRPQSPGASPSQAERLPSDSERRLYSRSSDRSGSCSSLSPPRYEKLDKS

RLERYTKNEKTDKERTFDPERVERERRLIRKEKVEKDKTDKQKRKGKVHSPSSQSSETDQ

ENEREQSPEKPRSCNKLSREKADKEGIAKNRLELMPCVVLTRVKEKEGKVIDHTPVEKLK

AKLDNDTVKSSALDQKLQVSQTEPAKSDLSKLESVRMKVPKEKGLSSHVEVVEKEGRLKA

RKHLKPEQPADGVSAVDLEKLEARKRRFADSNLKAEKQKPEVKKSSPEMEDARVLSKKQP

DVSSREVILLREGEAERKPVRKEILKRESKKIKLDRLNTVASPKDCQELASISVGSGSRP

SSDLQARLGELAGESVENQEVQSKKPIPSKPQLKQLQVLDDQGPEREDVRKNYCSLRDET

PERKSGQEKSHSVNTEEKIGIDIDHTQSYRKQMEQSRRKQQMEMEIAKSEKFGSPKKDVD

EYERRSLVHEVGKPPQDVTDDSPPSKKKRMDHVDFDICTKRERNYRSSRQISEDSERTGG

SPSVRHGSFHEDEDPIGSPRLLSVKGSPKVDEKVLPYSNITVREESLKFNPYDSSRREQM

ADMAKIKLSVLNSEDELNRWDSQMKQDAGRFDVSFPNSIIKRDSLRKRSVRDLEPGEVPS

DSDEDGEHKSHSPRASALYESSRLSFLLRDREDKLRERDERLSSSLERNKFYSFALDKTI

TPDTKALLERAKSLSSSREENWSFLDWDSRFANFRNNKDKEKVDSAPRPIPSWYMKKKKI

RTDSEGKMDDKKEDHKEEEQERQELFASRFLHSSIFEQDSKRLQHLERKEEDSDFISGRI

YGKQTSEGANSTTDSIQEPVVLFHSRFMELTRMQQKEKEKDQKPKEVEKQEDTENHPKTP

ESAPENKDSELKTPPSVGPPSVTVVTLESAPSALEKTTGDKTVEAPLVTEEKTVEPATVS

EEAKPASEPAPAPVEQLEQVDLPPGADPDKEAAMMPAGVEEGSSGDQPPYLDAKPPTPGA

SFSQAESNVDPEPDSTQPLSKPAQKSEEANEPKAEKPDATADAEPDANQKAEAAPESQPP

ASEDLEVDPPVAAKDKKPNKSKRSKTPVQAAAVSIVEKPVTRKSERIDREKLKRSNSPRG

EAQKLLELKMEAEKITRTASKNSAADLEHPEPSLPLSRTRRRNVRSVYATMGDHENRSPV

KEPVEQPRVTRKRLERELQEAAAVPTTPRRGRPPKTRRRADEEEENEAKEPAETLKPPEG

WRSPRSQKTAAGGGPQGKKGKNEPKVDATRPEATTEVGPQIGVKESSMEPKAAEEEAGSE

QKRDRKDAGTDKNPPETAPVEVVEKKPAPEKNSKSKRGRSRNSRLAVDKSASLKNVDAAV

SPRGAAAQAGERESGVVAVSPEKSESPQKEDGLSSQLKSDPVDPDKEPEKEDVSASGPSP

EATQLAKQMELEQAVEHIAKLAEASASAAYKADAPEGLAPEDRDKPAHQASETELAAAIG

SIINDISGEPENFPAPPPYPGESQTDLQPPAGAQALQPSEEGMETDEAVSGILETEAATE

SSRPPVNAPDPSAGPTDTKEARGNSSETSHSVPEAKGSKEVEVTLVRKDKGRQKTTRSRR

KRNTNKKVVAPVESHVPESNQAQGESPAANEGTTVQHPEAPQEEKQSEKPHSTPPQSCTS

DLSKIPSTENSSQEISVEERTPTKASVPPDLPPPPQPAPVDEEPQARFRVHSIIESDPVT

PPSDPSIPIPTLPSVTAAKLSPPVASGGIPHQSPPTKVTEWITRQEEPRAQSTPSPALPP

DTKASDVDTSSSTLRKILMDPKYVSATSVTSTSVTTAIAEPVSAAPCLHEAPPPPVDSKK

PLEEKTAPPVTNNSEIQASEVLVAADKEKVAPVIAPKITSVISRMPVSIDLENSQKITLA

KPAPQTLTGLVSALTGLVNVSLVPVNALKGPVKGSVTTLKSLVSTPAGPVNVLKGPVNVL

TGPVNVLTTPVNATVGTVNAAPGTVNAAASAVNATASAVTVTAGAVTAASGGVTATTGTV

TMAGAVIAPSTKCKQRASANENSRFHPGSMPVIDDRPADAGSGAGLRVNTSEGVVLLSYS

GQKTEGPQRISAKISQIPPASAMDIEFQQSVSKSQVKPDSVTASQPPSKGPQAPAGYANV

ATHSTLVLTAQTYNASPVISSVKADRPSLEKPEPIHLSVSTPVTQGGTVKVLTQGINTPP

VLVHNQLVLTPSIVTTNKKLADPVTLKIETKVLQPANLGSTLTPHHPPALPSKLPTEVNH

VPSGPSIPADRTVSHLAAAKLDAHSPRPSGPGPSSFPRASHPSSTASTALSTNATVMLAA

GIPVPQFISSIHPEQSVIMPPHSITQTVSLSHLSQGEVRMNTPTLPSITYSIRPEALHSP

RAPLQPQQIEVRAPQRASTPQPAPAGVPALASQHPPEEEVHYHLPVARATAPVQSEVLVM

QSEYRLHPYTVPRDVRIMVHPHVTAVSEQPRAADGVVKVPPASKAPQQPGKEAAKTPDAK

AAPTPTPAPVPVPVPLPAPAPAPHGEARILTVTPSNQLQGLPLTPPVVVTHGVQIVHSSG

ELFQEYRYGDIRTYHPPAQLTHTQFPAASSVGLPSRTKTAAQGPPPEGEPLQPPQPVQST

QPAQPAPPCPPSQLGQPGQPPSSKMPQVSQEAKGTQTGVEQPRLPAGPANRPPEPHTQVQ

RAQAETGPTSFPSPVSVSMKPDLPVSLPTQTAPKQPLFVPTTSGPSTPPGLVLPHTEFQP

APKQDSSPHLTSQRPVDMVQLLKKYPIVWQGLLALKNDTAAVQLHFVSGNNVLAHRSLPL

SEGGPPLRIAQRMRLEATQLEGVARRMTVETDYCLLLALPCGRDQEDVVSQTESLKAAFI

TYLQAKQAAGIINVPNPGSNQPAYVLQIFPPCEFSESHLSRLAPDLLASISNISPHLMIV

IASV

>sp|P13611|CSPG2_HUMAN 1115 KTDEVVTL

MFINIKSILWMCSTLIVTHALHKVKVGKSPPVRGSLSGKVSLPCHFSTMPTLPPSYNTSE

FLRIKWSKIEVDKNGKDLKETTVLVAQNGNIKIGQDYKGRVSVPTHPEAVGDASLTVVKL

LASDAGLYRCDVMYGIEDTQDTVSLTVDGVVFHYRAATSRYTLNFEAAQKACLDVGAVIA

TPEQLFAAYEDGFEQCDAGWLADQTVRYPIRAPRVGCYGDKMGKAGVRTYGFRSPQETYD

VYCYVDHLDGDVFHLTVPSKFTFEEAAKECENQDARLATVGELQAAWRNGFDQCDYGWLS

DASVRHPVTVARAQCGGGLLGVRTLYRFENQTGFPPPDSRFDAYCFKPKEATTIDLSILA

ETASPSLSKEPQMVSDRTTPIIPLVDELPVIPTEFPPVGNIVSFEQKATVQPQAITDSLA

TKLPTPTGSTKKPWDMDDYSPSASGPLGKLDISEIKEEVLQSTTGVSHYATDSWDGVVED

KQTQESVTQIEQIEVGPLVTSMEILKHIPSKEFPVTETPLVTARMILESKTEKKMVSTVS

ELVTTGHYGFTLGEEDDEDRTLTVGSDESTLIFDQIPEVITVSKTSEDTIHTHLEDLESV

SASTTVSPLIMPDNNGSSMDDWEERQTSGRITEEFLGKYLSTTPFPSQHRTEIELFPYSG

DKILVEGISTVIYPSLQTEMTHRRERTETLIPEMRTDTYTDEIQEEITKSPFMGKTEEEV

FSGMKLSTSLSEPIHVTESSVEMTKSFDFPTLITKLSAEPTEVRDMEEDFTATPGTTKYD

ENITTVLLAHGTLSVEAATVSKWSWDEDNTTSKPLESTEPSASSKLPPALLTTVGMNGKD

KDIPSFTEDGADEFTLIPDSTQKQLEEVTDEDIAAHGKFTIRFQPTTSTGIAEKSTLRDS

TTEEKVPPITSTEGQVYATMEGSALGEVEDVDLSKPVSTVPQFAHTSEVEGLAFVSYSST

QEPTTYVDSSHTIPLSVIPKTDWGVLVPSVPSEDEVLGEPSQDILVIDQTRLEATISPET

MRTTKITEGTTQEEFPWKEQTAEKPVPALSSTAWTPKEAVTPLDEQEGDGSAYTVSEDEL

LTGSERVPVLETTPVGKIDHSVSYPPGAVTEHKVKTDEVVTLTPRIGPKVSLSPGPEQKY

ETEGSSTTGFTSSLSPFSTHITQLMEETTTEKTSLEDIDLGSGLFEKPKATELIEFSTIK

VTVPSDITTAFSSVDRLHTTSAFKPSSAITKKPPLIDREPGEETTSDMVIIGESTSHVPP

TTLEDIVAKETETDIDREYFTTSSPPATQPTRPPTVEDKEAFGPQALSTPQPPASTKFHP

DINVYIIEVRENKTGRMSDLSVIGHPIDSESKEDEPCSEETDPVHDLMAEILPEFPDIIE

IDLYHSEENEEEEEECANATDVTTTPSVQYINGKHLVTTVPKDPEAAEARRGQFESVAPS

QNFSDSSESDTHPFVIAKTELSTAVQPNESTETTESLEVTWKPETYPETSEHFSGGEPDV

FPTVPFHEEFESGTAKKGAESVTERDTEVGHQAHEHTEPVSLFPEESSGEIAIDQESQKI

AFARATEVTFGEEVEKSTSVTYTPTIVPSSASAYVSEEEAVTLIGNPWPDDLLSTKESWV

EATPRQVVELSGSSSIPITEGSGEAEEDEDTMFTMVTDLSQRNTTDTLITLDTSRIITES

FFEVPATTIYPVSEQPSAKVVPTKFVSETDTSEWISSTTVEEKKRKEEEGTTGTASTFEV

YSSTQRSDQLILPFELESPNVATSSDSGTRKSFMSLTTPTQSEREMTDSTPVFTETNTLE

NLGAQTTEHSSIHQPGVQEGLTTLPRSPASVFMEQGSGEAAADPETTTVSSFSLNVEYAI

QAEKEVAGTLSPHVETTFSTEPTGLVLSTVMDRVVAENITQTSREIVISERLGEPNYGAE

IRGFSTGFPLEEDFSGDFREYSTVSHPIAKEETVMMEGSGDAAFRDTQTSPSTVPTSVHI

SHISDSEGPSSTMVSTSAFPWEEFTSSAEGSGEQLVTVSSSVVPVLPSAVQKFSGTASSI

IDEGLGEVGTVNEIDRRSTILPTAEVEGTKAPVEKEEVKVSGTVSTNFPQTIEPAKLWSR

QEVNPVRQEIESETTSEEQIQEEKSFESPQNSPATEQTIFDSQTFTETELKTTDYSVLTT

KKTYSDDKEMKEEDTSLVNMSTPDPDANGLESYTTLPEATEKSHFFLATALVTESIPAEH

VVTDSPIKKEESTKHFPKGMRPTIQESDTELLFSGLGSGEEVLPTLPTESVNFTEVEQIN

NTLYPHTSQVESTSSDKIEDFNRMENVAKEVGPLVSQTDIFEGSGSVTSTTLIEILSDTG

AEGPTVAPLPFSTDIGHPQNQTVRWAEEIQTSRPQTITEQDSNKNSSTAEINETTTSSTD

FLARAYGFEMAKEFVTSAPKPSDLYYEPSGEGSGEVDIVDSFHTSATTQATRQESSTTFV

SDGSLEKHPEVPSAKAVTADGFPTVSVMLPLHSEQNKSSPDPTSTLSNTVSYERSTDGSF

QDRFREFEDSTLKPNRKKPTENIIIDLDKEDKDLILTITESTILEILPELTSDKNTIIDI

DHTKPVYEDILGMQTDIDTEVPSEPHDSNDESNDDSTQVQEIYEAAVNLSLTEETFEGSA

DVLASYTQATHDESMTYEDRSQLDHMGFHFTTGIPAPSTETELDVLLPTATSLPIPRKSA

TVIPEIEGIKAEAKALDDMFESSTLSDGQAIADQSEIIPTLGQFERTQEEYEDKKHAGPS

FQPEFSSGAEEALVDHTPYLSIATTHLMDQSVTEVPDVMEGSNPPYYTDTTLAVSTFAKL

SSQTPSSPLTIYSGSEASGHTEIPQPSALPGIDVGSSVMSPQDSFKEIHVNIEATFKPSS

EEYLHITEPPSLSPDTKLEPSEDDGKPELLEEMEASPTELIAVEGTEILQDFQNKTDGQV

SGEAIKMFPTIKTPEAGTVITTADEIELEGATQWPHSTSASATYGVEAGVVPWLSPQTSE

RPTLSSSPEINPETQAALIRGQDSTIAASEQQVAARILDSNDQATVNPVEFNTEVATPPF

SLLETSNETDFLIGINEESVEGTAIYLPGPDRCKMNPCLNGGTCYPTETSYVCTCVPGYS

GDQCELDFDECHSNPCRNGATCVDGFNTFRCLCLPSYVGALCEQDTETCDYGWHKFQGQC

YKYFAHRRTWDAAERECRLQGAHLTSILSHEEQMFVNRVGHDYQWIGLNDKMFEHDFRWT

DGSTLQYENWRPNQPDSFFSAGEDCVVIIWHENGQWNDVPCNYHLTYTCKKGTVACGQPP

VVENAKTFGKMKPRYEINSLIRYHCKDGFIQRHLPTIRCLGNGRWAIPKITCMNPSAYQR

TYSMKYFKNSSSAKDNSINTSKHDHRWSRRWQESRR

>sp|Q63HN8|RN213_HUMAN 2218 DVKDYIQEY

MVCDGDWEHCYLPSAFSQHKVFVTPQAPLEAIQAYLAGHYRVPKQTLSAAAVFNDRLCVG

IVASERAGVGKSLYVKRLHDKMKMQLNVKNVPLKTIRLIDPQVDESRVLGALLPFLDAQY

QKVPVLFHLDVTSSVQTGIWVFLFKLLILQYLMDINGKMWLRNPCHLYIVEILERRTSVP

SRSSSALRTRVPQFSFLDIFPKVTCRPPKEVIDMELSALRSDTEPGMDLWEFCSETFQRP

YQYLRRFNQNQDLDTFQYQEGSVEGTPEECLQHFLFHCGVINPSWSELRNFARFLNYQLR

DCEASLFCNPSFIGDTLRGFKKFVVTFMIFMARDFATPSLHTSDQSPGKHMVTMDGVREE

DLAPFSLRKRWESEPHPYVFFNDDHTTMTFIGFHLQPNINGSVDAISHLTGKVIKRDVMT

RDLYQGLLLQRVPFNVDFDKLPRHKKLERLCLTLGIPQATDPDKTYELTTDNMLKILAIE

MRFRCGIPVIIMGETGCGKTRLIKFLSDLRRGGTNADTIKLVKVHGGTTADMIYSRVREA

ENVAFANKDQHQLDTILFFDEANTTEAISCIKEVLCDHMVDGQPLAEDSGLHIIAACNPY

RKHSEEMICRLESAGLGYRVSMEETADRLGSIPLRQLVYRVHALPPSLIPLVWDFGQLSD

VAEKLYIQQIVQRLVESISLDENGTRVITEVLCASQGFMRKTEDECSFVSLRDVERCVKV

FRWFHEHSAMLLAQLNAFLSKSSVSKNHTERDPVLWSLMLAIGVCYHASLEKKDSYRKAI

ARFFPKPYDDSRLLLDEITRAQDLFLDGVPLRKTIAKNLALKENVFMMVVCIELKIPLFL

VGKPGSSKSLAKTIVADAMQGPAAYSDLFRSLKQVHLVSFQCSPHSTPQGIISTFRQCAR

FQQGKDLQQYVSVVVLDEVGLAEDSPKMPLKTLHPLLEDGCIEDDPAPHKKVGFVGISNW

ALDPAKMNRGIFVSRGSPNETELIESAKGICSSDILVQDRVQGYFASFAKAYETVCKRQD

KEFFGLRDYYSLIKMVFAAAKASNRKPSPQDIAQAVLRNFSGKDDIQALDIFLANLPEAK

CSEEVSPMQLIKQNIFGPSQKVPGGEQEDAESRYLLVLTKNYVALQILQQTFFEGDQQPE

IIFGSGFPKDQEYTQLCRNINRVKICMETGKMVLLLNLQNLYESLYDALNQYYVHLGGQK

YVDLGLGTHRVKCRVHPNFRLIVIEEKDVVYKHFPIPLINRLEKHYLDINTVLEKWQKSI

VEELCAWVEKFINVKAHHFQKRHKYSPSDVFIGYHSDACASVVLQVIERQGPRALTEELH

QKVSEEAKSILLNCATPDAVVRLSAYSLGGFAAEWLSQEYFHRQRHNSFADFLQAHLHTA

DLERHAIFTEITTFSRLLTSHDCEILESEVTGRAPKPTLLWLQQFDTEYSFLKEVRNCLT

NTAKCKILIFQTDFEDGIRSAQLIASAKYSVINEINKIRENEDRIFVYFITKLSRVGRGT

AYVGFHGGLWQSVHIDDLRRSTLMVSDVTRLQHVTISQLFAPGDLPELGLEHRAEDGHEE

AMETEASTSGEVAEVAEEAMETESSEKVGKETSELGGSDVSILDTTRLLRSCVQSAVGML

RDQNESCTRNMRRVVLLLGLLNEDDACHASFLRVSKMRLSVFLKKQEESQFHPLEWLARE

ACNQDALQEAGTFRHTLWKRVQGAVTPLLASMISFIDRDGNLELLTRPDTPPWARDLWMF

IFSDTMLLNIPLVMNNERHKGEMAYIVVQNHMNLSENASNNVPFSWKIKDYLEELWVQAQ

YITDAEGLPKKFVDIFQQTPLGRFLAQLHGEPQQELLQCYLKDFILLTMRVSTEEELKFL

QMALWSCTRKLKAASEAPEEEVSLPWVHLAYQRFRSRLQNFSRILTIYPQVLHSLMEARW

NHELAGCEMTLDAFAAMACTEMLTRNTLKPSPQAWLQLVKNLSMPLELICSDEHMQGSGS

LAQAVIREVRAQWSRIFSTALFVEHVLLGTESRVPELQGLVTEHVFLLDKCLRENSDVKT

HGPFEAVMRTLCECKETASKTLSRFGIQPCSICLGDAKDPVCLPCDHVHCLRCLRAWFAS

EQMICPYCLTALPDEFSPAVSQAHREAIEKHARFRQMCNSFFVDLVSTICFKDNAPPEKE

VIESLLSLLFVQKGRLRDAAQRHCEHTKSLSPFNDVVDKTPVIRSVILKLLLKYSFHDVK

DYIQEYLTLLKKKAFITEDKTELYMLFINCLEDSILEKTSAYSRNDELNHLEEEGRFLKA

YSPASRGREPANEASVEYLQEVARIRLCLDRAADFLSEPEGGPEMAKEKQCYLQQVKQFC

IRVENDWHRVYLVRKLSSQRGMEFVQGLSKPGRPHQWVFPKDVVKQQGLRQDHPGQMDRY

LVYGDEYKALRDAVAKAVLECKPLGIKTALKACKTPQSQQSAYFLLTLFREVAILYRSHN

ASLHPTPEQCEAVSKFIGECKILSPPDISRFATSLVDNSVPLLRAGPSDSNLDGTVTEMA

IHAAAVLLCGQNELLEPLKNLAFSPATMAHAFLPTMPEDLLAQARRWKGLERVHWYTCPN

GHPCSVGECGRPMEQSICIDCHAPIGGIDHKPRDGFHLVKDKADRTQTGHVLGNPQRRDV

VTCDRGLPPVVFLLIRLLTHLALLLGASQSSQALINIIKPPVRDPKGFLQQHILKDLEQL

AKMLGHSADETIGVVHLVLRRLLQEQHQLSSRRLLNFDTELSTKEMRNNWEKEIAAVISP

ELEHLDKTLPTMNNLISQDKRISSNPVAKIIYGDPVTFLPHLPRKSVVHCSKIWSCRKRI

TVEYLQHIVEQKNGKERVPILWHFLQKEAELRLVKFLPEILALQRDLVKQFQNVQQVEYS

SIRGFLSKHSSDGLRQLLHNRITVFLSTWNKLRRSLETNGEINLPKDYCSTDLDLDTEFE

ILLPRRRGLGLCATALVSYLIRLHNEIVYAVEKLSKENNSYSVDAAEVTELHVISYEVER

DLTPLILSNCQYQVEEGRETVQEFDLEKIQRQIVSRFLQGKPRLSLKGIPTLVYRHDWNY

EHLFMDIKNKMAQDSLPSSVISAISGQLQSYSDACEVLSVVEVTLGFLSTAGGDPNMQLN

VYTQDILQMGDQTIHVLKALNRCQLKHTIALWQFLSAHKSEQLLRLHKEPFGEISSRYKA

DLSPENAKLLSTFLNQTGLDAFLLELHEMIILKLKNPQTQTEERFRPQWSLRDTLVSYMQ

TKESEILPEMASQFPEEILLASCVSVWKTAAVLKWNREMR

>sp|Q63HN8|RN213_HUMAN 3207 HEMIILKL

MVCDGDWEHCYLPSAFSQHKVFVTPQAPLEAIQAYLAGHYRVPKQTLSAAAVFNDRLCVG

IVASERAGVGKSLYVKRLHDKMKMQLNVKNVPLKTIRLIDPQVDESRVLGALLPFLDAQY

QKVPVLFHLDVTSSVQTGIWVFLFKLLILQYLMDINGKMWLRNPCHLYIVEILERRTSVP

SRSSSALRTRVPQFSFLDIFPKVTCRPPKEVIDMELSALRSDTEPGMDLWEFCSETFQRP

YQYLRRFNQNQDLDTFQYQEGSVEGTPEECLQHFLFHCGVINPSWSELRNFARFLNYQLR

DCEASLFCNPSFIGDTLRGFKKFVVTFMIFMARDFATPSLHTSDQSPGKHMVTMDGVREE

DLAPFSLRKRWESEPHPYVFFNDDHTTMTFIGFHLQPNINGSVDAISHLTGKVIKRDVMT

RDLYQGLLLQRVPFNVDFDKLPRHKKLERLCLTLGIPQATDPDKTYELTTDNMLKILAIE

MRFRCGIPVIIMGETGCGKTRLIKFLSDLRRGGTNADTIKLVKVHGGTTADMIYSRVREA

ENVAFANKDQHQLDTILFFDEANTTEAISCIKEVLCDHMVDGQPLAEDSGLHIIAACNPY

RKHSEEMICRLESAGLGYRVSMEETADRLGSIPLRQLVYRVHALPPSLIPLVWDFGQLSD

VAEKLYIQQIVQRLVESISLDENGTRVITEVLCASQGFMRKTEDECSFVSLRDVERCVKV

FRWFHEHSAMLLAQLNAFLSKSSVSKNHTERDPVLWSLMLAIGVCYHASLEKKDSYRKAI

ARFFPKPYDDSRLLLDEITRAQDLFLDGVPLRKTIAKNLALKENVFMMVVCIELKIPLFL

VGKPGSSKSLAKTIVADAMQGPAAYSDLFRSLKQVHLVSFQCSPHSTPQGIISTFRQCAR

FQQGKDLQQYVSVVVLDEVGLAEDSPKMPLKTLHPLLEDGCIEDDPAPHKKVGFVGISNW

ALDPAKMNRGIFVSRGSPNETELIESAKGICSSDILVQDRVQGYFASFAKAYETVCKRQD

KEFFGLRDYYSLIKMVFAAAKASNRKPSPQDIAQAVLRNFSGKDDIQALDIFLANLPEAK

CSEEVSPMQLIKQNIFGPSQKVPGGEQEDAESRYLLVLTKNYVALQILQQTFFEGDQQPE

IIFGSGFPKDQEYTQLCRNINRVKICMETGKMVLLLNLQNLYESLYDALNQYYVHLGGQK

YVDLGLGTHRVKCRVHPNFRLIVIEEKDVVYKHFPIPLINRLEKHYLDINTVLEKWQKSI

VEELCAWVEKFINVKAHHFQKRHKYSPSDVFIGYHSDACASVVLQVIERQGPRALTEELH

QKVSEEAKSILLNCATPDAVVRLSAYSLGGFAAEWLSQEYFHRQRHNSFADFLQAHLHTA

DLERHAIFTEITTFSRLLTSHDCEILESEVTGRAPKPTLLWLQQFDTEYSFLKEVRNCLT

NTAKCKILIFQTDFEDGIRSAQLIASAKYSVINEINKIRENEDRIFVYFITKLSRVGRGT

AYVGFHGGLWQSVHIDDLRRSTLMVSDVTRLQHVTISQLFAPGDLPELGLEHRAEDGHEE

AMETEASTSGEVAEVAEEAMETESSEKVGKETSELGGSDVSILDTTRLLRSCVQSAVGML

RDQNESCTRNMRRVVLLLGLLNEDDACHASFLRVSKMRLSVFLKKQEESQFHPLEWLARE

ACNQDALQEAGTFRHTLWKRVQGAVTPLLASMISFIDRDGNLELLTRPDTPPWARDLWMF

IFSDTMLLNIPLVMNNERHKGEMAYIVVQNHMNLSENASNNVPFSWKIKDYLEELWVQAQ

YITDAEGLPKKFVDIFQQTPLGRFLAQLHGEPQQELLQCYLKDFILLTMRVSTEEELKFL

QMALWSCTRKLKAASEAPEEEVSLPWVHLAYQRFRSRLQNFSRILTIYPQVLHSLMEARW

NHELAGCEMTLDAFAAMACTEMLTRNTLKPSPQAWLQLVKNLSMPLELICSDEHMQGSGS

LAQAVIREVRAQWSRIFSTALFVEHVLLGTESRVPELQGLVTEHVFLLDKCLRENSDVKT

HGPFEAVMRTLCECKETASKTLSRFGIQPCSICLGDAKDPVCLPCDHVHCLRCLRAWFAS

EQMICPYCLTALPDEFSPAVSQAHREAIEKHARFRQMCNSFFVDLVSTICFKDNAPPEKE

VIESLLSLLFVQKGRLRDAAQRHCEHTKSLSPFNDVVDKTPVIRSVILKLLLKYSFHDVK

DYIQEYLTLLKKKAFITEDKTELYMLFINCLEDSILEKTSAYSRNDELNHLEEEGRFLKA

YSPASRGREPANEASVEYLQEVARIRLCLDRAADFLSEPEGGPEMAKEKQCYLQQVKQFC

IRVENDWHRVYLVRKLSSQRGMEFVQGLSKPGRPHQWVFPKDVVKQQGLRQDHPGQMDRY

LVYGDEYKALRDAVAKAVLECKPLGIKTALKACKTPQSQQSAYFLLTLFREVAILYRSHN

ASLHPTPEQCEAVSKFIGECKILSPPDISRFATSLVDNSVPLLRAGPSDSNLDGTVTEMA

IHAAAVLLCGQNELLEPLKNLAFSPATMAHAFLPTMPEDLLAQARRWKGLERVHWYTCPN

GHPCSVGECGRPMEQSICIDCHAPIGGIDHKPRDGFHLVKDKADRTQTGHVLGNPQRRDV

VTCDRGLPPVVFLLIRLLTHLALLLGASQSSQALINIIKPPVRDPKGFLQQHILKDLEQL

AKMLGHSADETIGVVHLVLRRLLQEQHQLSSRRLLNFDTELSTKEMRNNWEKEIAAVISP

ELEHLDKTLPTMNNLISQDKRISSNPVAKIIYGDPVTFLPHLPRKSVVHCSKIWSCRKRI

TVEYLQHIVEQKNGKERVPILWHFLQKEAELRLVKFLPEILALQRDLVKQFQNVQQVEYS

SIRGFLSKHSSDGLRQLLHNRITVFLSTWNKLRRSLETNGEINLPKDYCSTDLDLDTEFE

ILLPRRRGLGLCATALVSYLIRLHNEIVYAVEKLSKENNSYSVDAAEVTELHVISYEVER

DLTPLILSNCQYQVEEGRETVQEFDLEKIQRQIVSRFLQGKPRLSLKGIPTLVYRHDWNY

EHLFMDIKNKMAQDSLPSSVISAISGQLQSYSDACEVLSVVEVTLGFLSTAGGDPNMQLN

VYTQDILQMGDQTIHVLKALNRCQLKHTIALWQFLSAHKSEQLLRLHKEPFGEISSRYKA

DLSPENAKLLSTFLNQTGLDAFLLELHEMIILKLKNPQTQTEERFRPQWSLRDTLVSYMQ

TKESEILPEMASQFPEEILLASCVSVWKTAAVLKWNREMR

>sp|P46013|KI67_HUMAN 780 SENLLGKQF

MWPTRRLVTIKRSGVDGPHFPLSLSTCLFGRGIECDIRIQLPVVSKQHCKIEIHEQEAIL

HNFSSTNPTQVNGSVIDEPVRLKHGDVITIIDRSFRYENESLQNGRKSTEFPRKIREQEP

ARRVSRSSFSSDPDEKAQDSKAYSKITEGKVSGNPQVHIKNVKEDSTADDSKDSVAQGTT

NVHSSEHAGRNGRNAADPISGDFKEISSVKLVSRYGELKSVPTTQCLDNSKKNESPFWKL

YESVKKELDVKSQKENVLQYCRKSGLQTDYATEKESADGLQGETQLLVSRKSRPKSGGSG

HAVAEPASPEQELDQNKGKGRDVESVQTPSKAVGASFPLYEPAKMKTPVQYSQQQNSPQK

HKNKDLYTTGRRESVNLGKSEGFKAGDKTLTPRKLSTRNRTPAKVEDAADSATKPENLSS

KTRGSIPTDVEVLPTETEIHNEPFLTLWLTQVERKIQKDSLSKPEKLGTTAGQMCSGLPG

LSSVDINNFGDSINESEGIPLKRRRVSFGGHLRPELFDENLPPNTPLKRGEAPTKRKSLV

MHTPPVLKKIIKEQPQPSGKQESGSEIHVEVKAQSLVISPPAPSPRKTPVASDQRRRSCK

TAPASSSKSQTEVPKRGGRKSGNLPSKRVSISRSQHDILQMICSKRRSGASEANLIVAKS

WADVVKLGAKQTQTKVIKHGPQRSMNKRQRRPATPKKPVGEVHSQFSTGHANSPCTIIIG

KAHTEKVHVPARPYRVLNNFISNQKMDFKEDLSGIAEMFKTPVKEQPQLTSTCHIAISNS

ENLLGKQFQGTDSGEEPLLPTSESFGGNVFFSAQNAAKQPSDKCSASPPLRRQCIRENGN

VAKTPRNTYKMTSLETKTSDTETEPSKTVSTANRSGRSTEFRNIQKLPVESKSEETNTEI

VECILKRGQKATLLQQRREGEMKEIERPFETYKENIELKENDEKMKAMKRSRTWGQKCAP

MSDLTDLKSLPDTELMKDTARGQNLLQTQDHAKAPKSEKGKITKMPCQSLQPEPINTPTH

TKQQLKASLGKVGVKEELLAVGKFTRTSGETTHTHREPAGDGKSIRTFKESPKQILDPAA

RVTGMKKWPRTPKEEAQSLEDLAGFKELFQTPGPSEESMTDEKTTKIACKSPPPESVDTP

TSTKQWPKRSLRKADVEEEFLALRKLTPSAGKAMLTPKPAGGDEKDIKAFMGTPVQKLDL

AGTLPGSKRQLQTPKEKAQALEDLAGFKELFQTPGHTEELVAAGKTTKIPCDSPQSDPVD

TPTSTKQRPKRSIRKADVEGELLACRNLMPSAGKAMHTPKPSVGEEKDIIIFVGTPVQKL

DLTENLTGSKRRPQTPKEEAQALEDLTGFKELFQTPGHTEEAVAAGKTTKMPCESSPPES

ADTPTSTRRQPKTPLEKRDVQKELSALKKLTQTSGETTHTDKVPGGEDKSINAFRETAKQ

KLDPAASVTGSKRHPKTKEKAQPLEDLAGLKELFQTPVCTDKPTTHEKTTKIACRSQPDP

VDTPTSSKPQSKRSLRKVDVEEEFFALRKRTPSAGKAMHTPKPAVSGEKNIYAFMGTPVQ

KLDLTENLTGSKRRLQTPKEKAQALEDLAGFKELFQTRGHTEESMTNDKTAKVACKSSQP

DPDKNPASSKRRLKTSLGKVGVKEELLAVGKLTQTSGETTHTHTEPTGDGKSMKAFMESP

KQILDSAASLTGSKRQLRTPKGKSEVPEDLAGFIELFQTPSHTKESMTNEKTTKVSYRAS

QPDLVDTPTSSKPQPKRSLRKADTEEEFLAFRKQTPSAGKAMHTPKPAVGEEKDINTFLG

TPVQKLDQPGNLPGSNRRLQTRKEKAQALEELTGFRELFQTPCTDNPTTDEKTTKKILCK

SPQSDPADTPTNTKQRPKRSLKKADVEEEFLAFRKLTPSAGKAMHTPKAAVGEEKDINTF

VGTPVEKLDLLGNLPGSKRRPQTPKEKAKALEDLAGFKELFQTPGHTEESMTDDKITEVS

CKSPQPDPVKTPTSSKQRLKISLGKVGVKEEVLPVGKLTQTSGKTTQTHRETAGDGKSIK

AFKESAKQMLDPANYGTGMERWPRTPKEEAQSLEDLAGFKELFQTPDHTEESTTDDKTTK

IACKSPPPESMDTPTSTRRRPKTPLGKRDIVEELSALKQLTQTTHTDKVPGDEDKGINVF

RETAKQKLDPAASVTGSKRQPRTPKGKAQPLEDLAGLKELFQTPICTDKPTTHEKTTKIA

CRSPQPDPVGTPTIFKPQSKRSLRKADVEEESLALRKRTPSVGKAMDTPKPAGGDEKDMK

AFMGTPVQKLDLPGNLPGSKRWPQTPKEKAQALEDLAGFKELFQTPGTDKPTTDEKTTKI

ACKSPQPDPVDTPASTKQRPKRNLRKADVEEEFLALRKRTPSAGKAMDTPKPAVSDEKNI

NTFVETPVQKLDLLGNLPGSKRQPQTPKEKAEALEDLVGFKELFQTPGHTEESMTDDKIT

EVSCKSPQPESFKTSRSSKQRLKIPLVKVDMKEEPLAVSKLTRTSGETTQTHTEPTGDSK

SIKAFKESPKQILDPAASVTGSRRQLRTRKEKARALEDLVDFKELFSAPGHTEESMTIDK

NTKIPCKSPPPELTDTATSTKRCPKTRPRKEVKEELSAVERLTQTSGQSTHTHKEPASGD

EGIKVLKQRAKKKPNPVEEEPSRRRPRAPKEKAQPLEDLAGFTELSETSGHTQESLTAGK

ATKIPCESPPLEVVDTTASTKRHLRTRVQKVQVKEEPSAVKFTQTSGETTDADKEPAGED

KGIKALKESAKQTPAPAASVTGSRRRPRAPRESAQAIEDLAGFKDPAAGHTEESMTDDKT

TKIPCKSSPELEDTATSSKRRPRTRAQKVEVKEELLAVGKLTQTSGETTHTDKEPVGEGK

GTKAFKQPAKRKLDAEDVIGSRRQPRAPKEKAQPLEDLASFQELSQTPGHTEELANGAAD

SFTSAPKQTPDSGKPLKISRRVLRAPKVEPVGDVVSTRDPVKSQSKSNTSLPPLPFKRGG

GKDGSVTGTKRLRCMPAPEEIVEELPASKKQRVAPRARGKSSEPVVIMKRSLRTSAKRIE

PAEELNSNDMKTNKEEHKLQDSVPENKGISLRSRRQNKTEAEQQITEVFVLAERIEINRN

EKKPMKTSPEMDIQNPDDGARKPIPRDKVTENKRCLRSARQNESSQPKVAEESGGQKSAK

VLMQNQKGKGEAGNSDSMCLRSRKTKSQPAASTLESKSVQRVTRSVKRCAENPKKAEDNV

CVKKIRTRSHRDSEDI

>sp|P49792|RBP2_HUMAN 3197 DTVKKIESF

MRRSKADVERYIASVQGSTPSPRQKSMKGFYFAKLYYEAKEYDLAKKYICTYINVQERDP

KAHRFLGLLYELEENTDKAVECYRRSVELNPTQKDLVLKIAELLCKNDVTDGRAKYWLER

AAKLFPGSPAIYKLKEQLLDCEGEDGWNKLFDLIQSELYVRPDDVHVNIRLVEVYRSTKR

LKDAVAHCHEAERNIALRSSLEWNSCVVQTLKEYLESLQCLESDKSDWRATNTDLLLAYA

NLMLLTLSTRDVQESRELLQSFDSALQSVKSLGGNDELSATFLEMKGHFYMHAGSLLLKM

GQHSSNVQWRALSELAALCYLIAFQVPRPKIKLIKGEAGQNLLEMMACDRLSQSGHMLLN

LSRGKQDFLKEIVETFANKSGQSALYDALFSSQSPKDTSFLGSDDIGNIDVREPELEDLT

RYDVGAIRAHNGSLQHLTWLGLQWNSLPALPGIRKWLKQLFHHLPHETSRLETNAPESIC

ILDLEVFLLGVVYTSHLQLKEKCNSHHSSYQPLCLPLPVCKQLCTERQKSWWDAVCTLIH

RKAVPGNVAKLRLLVQHEINTLRAQEKHGLQPALLVHWAECLQKTGSGLNSFYDQREYIG

RSVHYWKKVLPLLKIIKKKNSIPEPIDPLFKHFHSVDIQASEIVEYEEDAHITFAILDAV

NGNIEDAVTAFESIKSVVSYWNLALIFHRKAEDIENDALSPEEQEECKNYLRKTRDYLIK

IIDDSDSNLSVVKKLPVPLESVKEMLNSVMQELEDYSEGGPLYKNGSLRNADSEIKHSTP

SPTRYSLSPSKSYKYSPKTPPRWAEDQNSLLKMICQQVEAIKKEMQELKLNSSNSASPHR

WPTENYGPDSVPDGYQGSQTFHGAPLTVATTGPSVYYSQSPAYNSQYLLRPAANVTPTKG

PVYGMNRLPPQQHIYAYPQQMHTPPVQSSSACMFSQEMYGPPALRFESPATGILSPRGDD

YFNYNVQQTSTNPPLPEPGYFTKPPIAAHASRSAESKTIEFGKTNFVQPMPGEGLRPSLP

TQAHTTQPTPFKFNSNFKSNDGDFTFSSPQVVTQPPPAAYSNSESLLGLLTSDKPLQGDG

YSGAKPIPGGQTIGPRNTFNFGSKNVSGISFTENMGSSQQKNSGFRRSDDMFTFHGPGKS

VFGTPTLETANKNHETDGGSAHGDDDDDGPHFEPVVPLPDKIEVKTGEEDEEEFFCNRAK

LFRFDVESKEWKERGIGNVKILRHKTSGKIRLLMRREQVLKICANHYISPDMKLTPNAGS

DRSFVWHALDYADELPKPEQLAIRFKTPEEAALFKCKFEEAQSILKAPGTNVAMASNQAV

RIVKEPTSHDNKDICKSDAGNLNFEFQVAKKEGSWWHCNSCSLKNASTAKKCVSCQNLNP

SNKELVGPPLAETVFTPKTSPENVQDRFALVTPKKEGHWDCSICLVRNEPTVSRCIACQN

TKSANKSGSSFVHQASFKFGQGDLPKPINSDFRSVFSTKEGQWDCSACLVQNEGSSTKCA

ACQNPRKQSLPATSIPTPASFKFGTSETSKTLKSGFEDMFAKKEGQWDCSSCLVRNEANA

TRCVACQNPDKPSPSTSVPAPASFKFGTSETSKAPKSGFEGMFTKKEGQWDCSVCLVRNE

ASATKCIACQNPGKQNQTTSAVSTPASSETSKAPKSGFEGMFTKKEGQWDCSVCLVRNEA

SATKCIACQNPGKQNQTTSAVSTPASSETSKAPKSGFEGMFTKKEGQWDCSVCLVRNEAS

ATKCIACQCPSKQNQTTAISTPASSEISKAPKSGFEGMFIRKGQWDCSVCCVQNESSSLK

CVACDASKPTHKPIAEAPSAFTLGSEMKLHDSSGSQVGTGFKSNFSEKASKFGNTEQGFK

FGHVDQENSPSFMFQGSSNTEFKSTKEGFSIPVSADGFKFGISEPGNQEKKSEKPLENGT

GFQAQDISGQKNGRGVIFGQTSSTFTFADLAKSTSGEGFQFGKKDPNFKGFSGAGEKLFS

SQYGKMANKANTSGDFEKDDDAYKTEDSDDIHFEPVVQMPEKVELVTGEEDEKVLYSQRV

KLFRFDAEVSQWKERGLGNLKILKNEVNGKLRMLMRREQVLKVCANHWITTTMNLKPLSG

SDRAWMWLASDFSDGDAKLEQLAAKFKTPELAEEFKQKFEECQRLLLDIPLQTPHKLVDT

GRAAKLIQRAEEMKSGLKDFKTFLTNDQTKVTEEENKGSGTGAAGASDTTIKPNPENTGP

TLEWDNYDLREDALDDSVSSSSVHASPLASSPVRKNLFRFGESTTGFNFSFKSALSPSKS

PAKLNQSGTSVGTDEESDVTQEEERDGQYFEPVVPLPDLVEVSSGEENEQVVFSHRAKLY

RYDKDVGQWKERGIGDIKILQNYDNKQVRIVMRRDQVLKLCANHRITPDMTLQNMKGTER

VWLWTACDFADGERKVEHLAVRFKLQDVADSFKKIFDEAKTAQEKDSLITPHVSRSSTPR

ESPCGKIAVAVLEETTRERTDVIQGDDVADATSEVEVSSTSETTPKAVVSPPKFVFGSES

VKSIFSSEKSKPFAFGNSSATGSLFGFSFNAPLKSNNSETSSVAQSGSESKVEPKKCELS

KNSDIEQSSDSKVKNLFASFPTEESSINYTFKTPEKAKEKKKPEDSPSDDDVLIVYELTP

TAEQKALATKLKLPPTFFCYKNRPDYVSEEEEDDEDFETAVKKLNGKLYLDGSEKCRPLE

ENTADNEKECIIVWEKKPTVEEKAKADTLKLPPTFFCGVCSDTDEDNGNGEDFQSELQKV

QEAQKSQTEEITSTTDSVYTGGTEVMVPSFCKSEEPDSITKSISSPSVSSETMDKPVDLS

TRKEIDTDSTSQGESKIVSFGFGSSTGLSFADLASSNSGDFAFGSKDKNFQWANTGAAVF

GTQSVGTQSAGKVGEDEDGSDEEVVHNEDIHFEPIVSLPEVEVKSGEEDEEILFKERAKL

YRWDRDVSQWKERGVGDIKILWHTMKNYYRILMRRDQVFKVCANHVITKTMELKPLNVSN

NALVWTASDYADGEAKVEQLAVRFKTKEVADCFKKTFEECQQNLMKLQKGHVSLAAELSK

ETNPVVFFDVCADGEPLGRITMELFSNIVPRTAENFRALCTGEKGFGFKNSIFHRVIPDF

VCQGGDITKHDGTGGQSIYGDKFEDENFDVKHTGPGLLSMANQGQNTNNSQFVITLKKAE

HLDFKHVVFGFVKDGMDTVKKIESFGSPKGSVCRRITITECGQI

>sp|P49792|RBP2_HUMAN 261 SFDSALQSV

MRRSKADVERYIASVQGSTPSPRQKSMKGFYFAKLYYEAKEYDLAKKYICTYINVQERDP

KAHRFLGLLYELEENTDKAVECYRRSVELNPTQKDLVLKIAELLCKNDVTDGRAKYWLER

AAKLFPGSPAIYKLKEQLLDCEGEDGWNKLFDLIQSELYVRPDDVHVNIRLVEVYRSTKR

LKDAVAHCHEAERNIALRSSLEWNSCVVQTLKEYLESLQCLESDKSDWRATNTDLLLAYA

NLMLLTLSTRDVQESRELLQSFDSALQSVKSLGGNDELSATFLEMKGHFYMHAGSLLLKM

GQHSSNVQWRALSELAALCYLIAFQVPRPKIKLIKGEAGQNLLEMMACDRLSQSGHMLLN

LSRGKQDFLKEIVETFANKSGQSALYDALFSSQSPKDTSFLGSDDIGNIDVREPELEDLT

RYDVGAIRAHNGSLQHLTWLGLQWNSLPALPGIRKWLKQLFHHLPHETSRLETNAPESIC

ILDLEVFLLGVVYTSHLQLKEKCNSHHSSYQPLCLPLPVCKQLCTERQKSWWDAVCTLIH

RKAVPGNVAKLRLLVQHEINTLRAQEKHGLQPALLVHWAECLQKTGSGLNSFYDQREYIG

RSVHYWKKVLPLLKIIKKKNSIPEPIDPLFKHFHSVDIQASEIVEYEEDAHITFAILDAV

NGNIEDAVTAFESIKSVVSYWNLALIFHRKAEDIENDALSPEEQEECKNYLRKTRDYLIK

IIDDSDSNLSVVKKLPVPLESVKEMLNSVMQELEDYSEGGPLYKNGSLRNADSEIKHSTP

SPTRYSLSPSKSYKYSPKTPPRWAEDQNSLLKMICQQVEAIKKEMQELKLNSSNSASPHR

WPTENYGPDSVPDGYQGSQTFHGAPLTVATTGPSVYYSQSPAYNSQYLLRPAANVTPTKG

PVYGMNRLPPQQHIYAYPQQMHTPPVQSSSACMFSQEMYGPPALRFESPATGILSPRGDD

YFNYNVQQTSTNPPLPEPGYFTKPPIAAHASRSAESKTIEFGKTNFVQPMPGEGLRPSLP

TQAHTTQPTPFKFNSNFKSNDGDFTFSSPQVVTQPPPAAYSNSESLLGLLTSDKPLQGDG

YSGAKPIPGGQTIGPRNTFNFGSKNVSGISFTENMGSSQQKNSGFRRSDDMFTFHGPGKS

VFGTPTLETANKNHETDGGSAHGDDDDDGPHFEPVVPLPDKIEVKTGEEDEEEFFCNRAK

LFRFDVESKEWKERGIGNVKILRHKTSGKIRLLMRREQVLKICANHYISPDMKLTPNAGS

DRSFVWHALDYADELPKPEQLAIRFKTPEEAALFKCKFEEAQSILKAPGTNVAMASNQAV

RIVKEPTSHDNKDICKSDAGNLNFEFQVAKKEGSWWHCNSCSLKNASTAKKCVSCQNLNP

SNKELVGPPLAETVFTPKTSPENVQDRFALVTPKKEGHWDCSICLVRNEPTVSRCIACQN

TKSANKSGSSFVHQASFKFGQGDLPKPINSDFRSVFSTKEGQWDCSACLVQNEGSSTKCA

ACQNPRKQSLPATSIPTPASFKFGTSETSKTLKSGFEDMFAKKEGQWDCSSCLVRNEANA

TRCVACQNPDKPSPSTSVPAPASFKFGTSETSKAPKSGFEGMFTKKEGQWDCSVCLVRNE

ASATKCIACQNPGKQNQTTSAVSTPASSETSKAPKSGFEGMFTKKEGQWDCSVCLVRNEA

SATKCIACQNPGKQNQTTSAVSTPASSETSKAPKSGFEGMFTKKEGQWDCSVCLVRNEAS

ATKCIACQCPSKQNQTTAISTPASSEISKAPKSGFEGMFIRKGQWDCSVCCVQNESSSLK

CVACDASKPTHKPIAEAPSAFTLGSEMKLHDSSGSQVGTGFKSNFSEKASKFGNTEQGFK

FGHVDQENSPSFMFQGSSNTEFKSTKEGFSIPVSADGFKFGISEPGNQEKKSEKPLENGT

GFQAQDISGQKNGRGVIFGQTSSTFTFADLAKSTSGEGFQFGKKDPNFKGFSGAGEKLFS

SQYGKMANKANTSGDFEKDDDAYKTEDSDDIHFEPVVQMPEKVELVTGEEDEKVLYSQRV

KLFRFDAEVSQWKERGLGNLKILKNEVNGKLRMLMRREQVLKVCANHWITTTMNLKPLSG

SDRAWMWLASDFSDGDAKLEQLAAKFKTPELAEEFKQKFEECQRLLLDIPLQTPHKLVDT

GRAAKLIQRAEEMKSGLKDFKTFLTNDQTKVTEEENKGSGTGAAGASDTTIKPNPENTGP

TLEWDNYDLREDALDDSVSSSSVHASPLASSPVRKNLFRFGESTTGFNFSFKSALSPSKS

PAKLNQSGTSVGTDEESDVTQEEERDGQYFEPVVPLPDLVEVSSGEENEQVVFSHRAKLY

RYDKDVGQWKERGIGDIKILQNYDNKQVRIVMRRDQVLKLCANHRITPDMTLQNMKGTER

VWLWTACDFADGERKVEHLAVRFKLQDVADSFKKIFDEAKTAQEKDSLITPHVSRSSTPR

ESPCGKIAVAVLEETTRERTDVIQGDDVADATSEVEVSSTSETTPKAVVSPPKFVFGSES

VKSIFSSEKSKPFAFGNSSATGSLFGFSFNAPLKSNNSETSSVAQSGSESKVEPKKCELS

KNSDIEQSSDSKVKNLFASFPTEESSINYTFKTPEKAKEKKKPEDSPSDDDVLIVYELTP

TAEQKALATKLKLPPTFFCYKNRPDYVSEEEEDDEDFETAVKKLNGKLYLDGSEKCRPLE

ENTADNEKECIIVWEKKPTVEEKAKADTLKLPPTFFCGVCSDTDEDNGNGEDFQSELQKV

QEAQKSQTEEITSTTDSVYTGGTEVMVPSFCKSEEPDSITKSISSPSVSSETMDKPVDLS

TRKEIDTDSTSQGESKIVSFGFGSSTGLSFADLASSNSGDFAFGSKDKNFQWANTGAAVF

GTQSVGTQSAGKVGEDEDGSDEEVVHNEDIHFEPIVSLPEVEVKSGEEDEEILFKERAKL

YRWDRDVSQWKERGVGDIKILWHTMKNYYRILMRRDQVFKVCANHVITKTMELKPLNVSN

NALVWTASDYADGEAKVEQLAVRFKTKEVADCFKKTFEECQQNLMKLQKGHVSLAAELSK

ETNPVVFFDVCADGEPLGRITMELFSNIVPRTAENFRALCTGEKGFGFKNSIFHRVIPDF

VCQGGDITKHDGTGGQSIYGDKFEDENFDVKHTGPGLLSMANQGQNTNNSQFVITLKKAE

HLDFKHVVFGFVKDGMDTVKKIESFGSPKGSVCRRITITECGQI

>sp|P12111|CO6A3_HUMAN 2452 NEVTTEIRF

MRKHRHLPLVAVFCLFLSGFPTTHAQQQQADVKNGAAADIIFLVDSSWTIGEEHFQLVRE

FLYDVVKSLAVGENDFHFALVQFNGNPHTEFLLNTYRTKQEVLSHISNMSYIGGTNQTGK

GLEYIMQSHLTKAAGSRAGDGVPQVIVVLTDGHSKDGLALPSAELKSADVNVFAIGVEDA

DEGALKEIASEPLNMHMFNLENFTSLHDIVGNLVSCVHSSVSPERAGDTETLKDITAQDS

ADIIFLIDGSNNTGSVNFAVILDFLVNLLEKLPIGTQQIRVGVVQFSDEPRTMFSLDTYS

TKAQVLGAVKALGFAGGELANIGLALDFVVENHFTRAGGSRVEEGVPQVLVLISAGPSSD

EIRYGVVALKQASVFSFGLGAQAASRAELQHIATDDNLVFTVPEFRSFGDLQEKLLPYIV

GVAQRHIVLKPPTIVTQVIEVNKRDIVFLVDGSSALGLANFNAIRDFIAKVIQRLEIGQD

LIQVAVAQYADTVRPEFYFNTHPTKREVITAVRKMKPLDGSALYTGSALDFVRNNLFTSS

AGYRAAEGIPKLLVLITGGKSLDEISQPAQELKRSSIMAFAIGNKGADQAELEEIAFDSS

LVFIPAEFRAAPLQGMLPGLLAPLRTLSGTPEVHSNKRDIIFLLDGSANVGKTNFPYVRD

FVMNLVNSLDIGNDNIRVGLVQFSDTPVTEFSLNTYQTKSDILGHLRQLQLQGGSGLNTG

SALSYVYANHFTEAGGSRIREHVPQLLLLLTAGQSEDSYLQAANALTRAGILTFCVGASQ

ANKAELEQIAFNPSLVYLMDDFSSLPALPQQLIQPLTTYVSGGVEEVPLAQPESKRDILF

LFDGSANLVGQFPVVRDFLYKIIDELNVKPEGTRIAVAQYSDDVKVESRFDEHQSKPEIL

NLVKRMKIKTGKALNLGYALDYAQRYIFVKSAGSRIEDGVLQFLVLLVAGRSSDRVDGPA

SNLKQSGVVPFIFQAKNADPAELEQIVLSPAFILAAESLPKIGDLHPQIVNLLKSVHNGA

PAPVSGEKDVVFLLDGSEGVRSGFPLLKEFVQRVVESLDVGQDRVRVAVVQYSDRTRPEF

YLNSYMNKQDVVNAVRQLTLLGGPTPNTGAALEFVLRNILVSSAGSRITEGVPQLLIVLT

ADRSGDDVRNPSVVVKRGGAVPIGIGIGNADITEMQTISFIPDFAVAIPTFRQLGTVQQV

ISERVTQLTREELSRLQPVLQPLPSPGVGGKRDVVFLIDGSQSAGPEFQYVRTLIERLVD

YLDVGFDTTRVAVIQFSDDPKAEFLLNAHSSKDEVQNAVQRLRPKGGRQINVGNALEYVS

RNIFKRPLGSRIEEGVPQFLVLISSGKSDDEVVVPAVELKQFGVAPFTIARNADQEELVK

ISLSPEYVFSVSTFRELPSLEQKLLTPITTLTSEQIQKLLASTRYPPPAVESDAADIVFL

IDSSEGVRPDGFAHIRDFVSRIVRRLNIGPSKVRVGVVQFSNDVFPEFYLKTYRSQAPVL

DAIRRLRLRGGSPLNTGKALEFVARNLFVKSAGSRIEDGVPQHLVLVLGGKSQDDVSRFA

QVIRSSGIVSLGVGDRNIDRTELQTITNDPRLVFTVREFRELPNIEERIMNSFGPSAATP

APPGVDTPPPSRPEKKKADIVFLLDGSINFRRDSFQEVLRFVSEIVDTVYEDGDSIQVGL

VQYNSDPTDEFFLKDFSTKRQIIDAINKVVYKGGRHANTKVGLEHLRVNHFVPEAGSRLD

QRVPQIAFVITGGKSVEDAQDVSLALTQRGVKVFAVGVRNIDSEEVGKIASNSATAFRVG

NVQELSELSEQVLETLHDAMHETLCPGVTDAAKACNLDVILGFDGSRDQNVFVAQKGFES

KVDAILNRISQMHRVSCSGGRSPTVRVSVVANTPSGPVEAFDFDEYQPEMLEKFRNMRSQ

HPYVLTEDTLKVYLNKFRQSSPDSVKVVIHFTDGADGDLADLHRASENLRQEGVRALILV

GLERVVNLERLMHLEFGRGFMYDRPLRLNLLDLDYELAEQLDNIAEKACCGVPCKCSGQR

GDRGPIGSIGPKGIPGEDGYRGYPGDEGGPGERGPPGVNGTQGFQGCPGQRGVKGSRGFP

GEKGEVGEIGLDGLDGEDGDKGLPGSSGEKGNPGRRGDKGPRGEKGERGDVGIRGDPGNP

GQDSQERGPKGETGDLGPMGVPGRDGVPGGPGETGKNGGFGRRGPPGAKGNKGGPGQPGF

EGEQGTRGAQGPAGPAGPPGLIGEQGISGPRGSGGARGAPGERGRTGPLGRKGEPGEPGP

KGGIGNPGPRGETGDDGRDGVGSEGRRGKKGERGFPGYPGPKGNPGEPGLNGTTGPKGIR

GRRGNSGPPGIVGQKGRPGYPGPAGPRGNRGDSIDQCALIQSIKDKCPCCYGPLECPVFP

TELAFALDTSEGVNQDTFGRMRDVVLSIVNVLTIAESNCPTGARVAVVTYNNEVTTEIRF

ADSKRKSVLLDKIKNLQVALTSKQQSLETAMSFVARNTFKRVRNGFLMRKVAVFFSNTPT

RASPQLREAVLKLSDAGITPLFLTRQEDRQLINALQINNTAVGHALVLPAGRDLTDFLEN

VLTCHVCLDICNIDPSCGFGSWRPSFRDRRAAGSDVDIDMAFILDSAETTTLFQFNEMKK

YIAYLVRQLDMSPDPKASQHFARVAVVQHAPSESVDNASMPPVKVEFSLTDYGSKEKLVD

FLSRGMTQLQGTRALGSAIEYTIENVFESAPNPRDLKIVVLMLTGEVPEQQLEEAQRVIL

QAKCKGYFFVVLGIGRKVNIKEVYTFASEPNDVFFKLVDKSTELNEEPLMRFGRLLPSFV

SSENAFYLSPDIRKQCDWFQGDQPTKNLVKFGHKQVNVPNNVTSSPTSNPVTTTKPVTTT

KPVTTTTKPVTTTTKPVTIINQPSVKPAAAKPAPAKPVAAKPVATKTATVRPPVAVKPAT

AAKPVAAKPAAVRPPAAAAKPVATKPEVPRPQAAKPAATKPATTKPVVKMLREVQVFEIT

ENSAKLHWERPEPPGPYFYDLTVTSAHDQSLVLKQNLTVTDRVIGGLLAGQTYHVAVVCY

LRSQVRATYHGSFSTKKSQPPPPQPARSASSSTINLMVSTEPLALTETDICKLPKDEGTC

RDFILKWYYDPNTKSCARFWYGGCGGNENKFGSQKECEKVCAPVLAKPGVISVMGT

>sp|O94915|FRYL_HUMAN 2225 EIIKIIGKY

MSNITIDPDVKPGEYVIKSLFAEFAVQAEKKIEVVMAEPLEKLLSRSLQRGEDLQFDQLI

SSMSSVAEHCLPSLLRTLFDWYRRQNGTEDESYEYRPRSSTKSKGDEQQRERDYLLERRD

LAVDFIFCLVLVEVLKQIPVHPVPDPLVHEVLNLAFKHFKHKEGYSGTNTGNVHIIADLY

AEVIGVLAQSKFQAVRKKFVTELKELRQKEQSPHVVQSVISLIMGMKFFRVKMYPVEDFE

ASFQFMQECAQYFLEVKDKDIKHALAGLFVEILIPVAAAVKNEVNVPCLKNFVEMLYQTT

FELSSRKKHSLALYPLITCLLCVSQKQFFLNNWHIFLQNCLSHLKNKDPKMSRVALESLY

RLLWVYVIRIKCESNTVTQSRLMSIVSALFPKGSRSVVPRDTPLNIFVKIIQFIAQERLD

FAMKEIIFDLLSVGKSTKTFTINPERMNIGLRVFLVIADSLQQKDGEPPMPTTGVILPSG

NTLRVKKIFLNKTLTDEEAKVIGMSVYYPQVRKALDSILRHLDKEVGRPMCMTSVQMSNK

EPEDMITGERKPKIDLFRTCIAAIPRLIPDGMSRTDLIELLARLTIHMDEELRALAFNTL

QALMLDFPDWREDVLSGFVYFIVREVTDVHPTLLDNAVKMLVQLINQWKQAAQMHNKNQD

TQHGVANGASHPPPLERSPYSNVFHVVEGFALVILCSSRPATRRLAVSVLREIRALFALL

EIPKGDDELAIDVMDRLSPSILESFIHLTGADQTTLLYCPSSIDLQTLAEWNSSPISHQF

DVISPSHIWIFAHVTQGQDPWIISLSSFLKQENLPKHCSTAVSYAWMFAYTRLQLLSPQV

DINSPINAKKVNTTTSSDSYIGLWRNYLILCCSAATSSSSTSAGSVRCSPPETLASTPDS

GYSIDSKIIGIPSPSSLFKHIVPMMRSESMEITESLVLGLGRTNPGAFRELIEELHPIIK

EALERRPENMKRRRRRDILRVQLVRIFELLADAGVISHSASGGLDNETHFLNNTLLEYVD

LTRQLLEAENEKDSDTLKDIRCHFSALVANIIQNVPVHQRRSIFPQQSLRHSLFMLFSHW

AGPFSIMFTPLDRYSDRNMQINRHQYCALKAMSAVLCCGPVADNVGLSSDGYLYKWLDNI

LDSLDKKVHQLGCEAVTLLLELNPDQSNLMYWAVDRCYTGSGRVAAGCFKAIANVFQNRD

YQCDTVMLLNLILFKAADSSRSIYEVAMQLLQILEPKMFRYAHKLEVQRTDGVLSQLSPL

PHLYSVSYYQLSEELARAYPELTLAIFSEISQRIQTAHPAGRQVMLHYLLPWMNNIELVD

LKPLPTARRHDEDEDDSLKDRELMVTSRRWLRGEGWGSPQATAMVLNNLMYMTAKYGDEL

AWSEVENVWTTLADGWPKNLKIILHFLISICGVNSEPSLLPYVKKVIVYLGRDKTMQLLE

ELVSELQLTDPVSSGVTHMDNPPYYRITSSYKIPSVTSGTTSSSNTMVAPTDGNPDNKPI

KENIEESYVHLDIYSGLNSHLNRQHHRLESRYSSSSGGSYEEEKSDSMPLYSNWRLKVME

HNQGEPLPFPPAGGCWSPLVDYVPETSSPGLPLHRCNIAVILLTDLIIDHSVKVEWGSYL

HLLLHAIFIGFDHCHPEVYEHCKRLLLHLLIVMGPNSNIRTVASVLLRNKEFNEPRVLTV

KQVAHLDYNFTAGINDFIPDYQPSPMTDSGLSSSSTSSSISLGNNSAAISHLHTTILNEV

DISVEQDGKVKTLMEFITSRKRGPLWNHEDVSAKNPSIKSAEQLTTFLKHVVSVFKQSSS

EGIHLEHHLSEVALQTALSCSSRHYAGRSFQIFRALKQPLTATTLSDVLSRLVETVGDPG

EDAQGFVIELLLTLESAIDTLAETMKHYDLLSALSQTSYHDPIMGNKYAANRKSTGQLNL

STSPINSSSYLGYNSNARSNSLRLSLIGDRRGDRRRSNTLDIMDGRINHSSSLARTRSLS

SLREKGMYDVQSTTEPTNLMATIFWIAASLLESDYEYEYLLALRLLNKLLIHLPLDKSES

REKIENVQSKLKWTNFPGLQQLFLKGFTSASTQEMTVHLLSKLISVSKHTLVDPSQLSGF

PLNILCLLPHLIQHFDSPTQFCKETASRIAKVCAEEKCPTLVNLAHMMSLYSTHTYSRDC

SNWINVVCRYLHDSFSDTTFNLVTYLAELLEKGLSSMQQSLLQIIYSLLSHIDLSAAPAK

QFNLEIIKIIGKYVQSPYWKEALNILKLVVSRSASLVVPSDIPKTYGGDTGSPEISFTKI

FNNVSKELPGKTLDFHFDISETPIIGNKYGDQHSAAGRNGKPKVIAVTRSTSSTSSGSNS

NALVPVSWKRPQLSQRRTREKLMNVLSLCGPESGLPKNPSVVFSSNEDLEVGDQQTSLIS

TTEDINQEEEVAVEDNSSEQQFGVFKDFDFLDVELEDAEGESMDNFNWGVRRRSLDSIDK

GDTPSLQEYQCSSSTPSLNLTNQEDTDESSEEEAALTASQILSRTQMLNSDSATDETIPD

HPDLLLQSEDSTGSITTEEVLQIRDETPTLEASLDNANSRLPEDTTSVLKEEHVTTFEDE

GSYIIQEQQESLVCQGILDLEETEMPEPLAPESYPESVCEEDVTLALKELDERCEEEEAD

FSGLSSQDEEEQDGFPEVQTSPLPSPFLSAIIAAFQPVAYDDEEEAWRCHVNQMLSDTDG

SSAVFTFHVFSRLFQTIQRKFGEITNEAVSFLGDSLQRIGTKFKSSLEVMMLCSECPTVF

VDAETLMSCGLLETLKFGVLELQEHLDTYNVKREAAEQWLDDCKRTFGAKEDMYRINTDA

QQMEILAELELCRRLYKLHFQLLLLFQAYCKLINQVNTIKNEAEVINMSEELAQLESILK

EAESASENEEIDISKAAQTTIETAIHSLIETLKNKEFISAVAQVKAFRSLWPSDIFGSCE

DDPVQTLLHIYFHHQTLGQTGSFAVIGSNLDMSEANYKLMELNLEIRESLRMVQSYQLLA

QAKPMGNMVSTGF

>sp|O60229|KALRN_HUMAN 363 NAYVNINRI

MTDRFWDQWYLWYLRLLRLLDRGSFRNDGLKASDVLPILKEKVAFVSGGRDKRGGPILTF

PARSNHDRIRQEDLRKLVTYLASVPSEDVCKRGFTVIIDMRGSKWDLIKPLLKTLQEAFP

AEIHVALIIKPDNFWQKQKTNFGSSKFIFETSMVSVEGLTKLVDPSQLTEEFDGSLDYNH

EEWIELRLSLEEFFNSAVHLLSRLEDLQEMLARKEFPVDVEGSRRLIDEHTQLKKKVLKA

PVEELDREGQRLLQCIRCSDGFSGRNCIPGSADFQSLVPKITSLLDKLHSTRQHLHQMWH

VRKLKLDQCFQLRLFEQDAEKMFDWISHNKELFLQSHTEIGVSYQYALDLQTQHNHFAMN

SMNAYVNINRIMSVASRLSEAGHYASQQIKQISTQLDQEWKSFAAALDERSTILAMSAVF

HQKAEQFLSGVDAWCKMCSEGGLPSEMQDLELAIHHHQTLYEQVTQAYTEVSQDGKALLD

VLQRPLSPGNSESLTATANYSKAVHQVLDVVHEVLHHQRRLESIWQHRKVRLHQRLQLCV

FQQDVQQVLDWIENHGEAFLSKHTGVGKSLHRARALQKRHDDFEEVAQNTYTNADKLLEA

AEQLAQTGECDPEEIYKAARHLEVRIQDFVRRVEQRKLLLDMSVSFHTHTKELWTWMEDL

QKEMLEDVCADSVDAVQELIKQFQQQQTATLDATLNVIKEGEDLIQQLRSAPPSLGEPSE

ARDSAVSNNKTPHSSSISHIESVLQQLDDAQVQMEELFHERKIKLDIFLQLRIFEQYTIE

VTAELDAWNEDLLRQMNDFNTEDLTLAEQRLQRHTERKLAMNNMTFEVIQQGQDLHQYIT

EVQASGIELICEKDIDLAAQVQELLEFLHEKQHELELNAEQTHKRLEQCLQLRHLQAEVK

QVLGWIRNGESMLNASLVNASSLSEAEQLQREHEQFQLAIESLFHATSLQKTHQSALQVQ

QKAEVLLQAGHYDADAIRECAEKVALHWQQLMLKMEDRLKLVNASVAFYKTSEQVCSVLE

SLEQEYRRDEDWCGGRDKLGPAAEIDHVIPLISKHLEQKEAFLKACTLARRNAEVFLKYI

HRNNVSMPSVASHTRGPEQQVKAILSELLQRENRVLHFWTLKKRRLDQCQQYVVFERSAK

QALDWIQETGEFYLSTHTSTGETTEETQELLKEYGEFRVPAKQTKEKVKLLIQLADSFVE

KGHIHATEIRKWVTTVDKHYRDFSLRMGKYRYSLEKALGVNTEDNKDLELDIIPASLSDR

EVKLRDANHEVNEEKRKSARKKEFIMAELLQTEKAYVRDLHECLETYLWEMTSGVEEIPP

GILNKEHIIFGNIQEIYDFHNNIFLKELEKYEQLPEDVGHCFVTWADKFQMYVTYCKNKP

DSNQLILEHAGTFFDEIQQRHGLANSISSYLIKPVQRITKYQLLLKELLTCCEEGKGELK

DGLEVMLSVPKKANDAMHVSMLEGFDENLDVQGELILQDAFQVWDPKSLIRKGRERHLFL

FEISLVFSKEIKDSSGHTKYVYKNKLLTSELGVTEHVEGDPCKFALWSGRTPSSDNKTVL

KASNIETKQEWIKNIREVIQERIIHLKGALKEPLQLPKTPAKQRNNSKRDGVEDIDSQGD

GSSQPDTISIASRTSQNTVDSDKLSGGCELTVVLQDFSAGHSSELTIQVGQTVELLERPS

ERPGWCLVRTTERSPPLEGLVPSSALCISHSRSSVEMDCFFPLVKDAYSHSSSENGGKSE

SVANLQAQPSLNSIHSSPGPKRSTNTLKKWLTSPVRRLNSGKADGNIKKQKKVRDGRKSF

DLGSPKPGDETTPQGDSADEKSKKGWGEDEPDEESHTPLPPPMKIFDNDPTQDEMSSLLA

ARQASTEVPTAADLVNAIEKLVKNKLSLEGSSYRGSLKDPAGCLNEGMAPPTPPKNPEEE

QKAKALRGRMFVLNELVQTEKDYVKDLGIVVEGFMKRIEEKGVPEDMRGKDKIVFGNIHQ

IYDWHKDFFLAELEKCIQEQDRLAQLFIKHERKLHIYVWYCQNKPRSEYIVAEYDAYFEE

VKQEINQRLTLSDFLIKPIQRITKYQLLLKDFLRYSEKAGLECSDIEKAVELMCLVPKRC

NDMMNLGRLQGFEGTLTAQGKLLQQDTFYVIELDAGMQSRTKERRVFLFEQIVIFSELLR

KGSLTPGYMFKRSIKMNYLVLEENVDNDPCKFALMNRETSERVVLQAANADIQQAWVQDI

NQVLETQRDFLNALQSPIEYQRKERSTAVMRSQPARLPQASPRPYSSVPAGSEKPPKGSS

YNPPLPPLKISTSNGSPGFEYHQPGDKFEASKQNDLGGCNGTSSMAVIKDYYALKENEIC

VSQGEVVQVLAVNQQNMCLVYQPASDHSPAAEGWVPGSILAPLTKATAAESSDGSIKKSC

SWHTLRMRKRAEVENTGKNEATGPRKPKDILGNKVSVKETNSSEESECDDLDPNTSMEIL

NPNFIQEVAPEFLVPLVDVTCLLGDTVILQCKVCGRPKPTITWKGPDQNILDTDNSSATY

TVSSCDSGEITLKICNLMPQDSGIYTCIATNDHGTTSTSATVKVQGVPAAPNRPIAQERS

CTSVILRWLPPSSTGNCTISGYTVEYREEGSQIWQQSVASTLDTYLVIEDLSPGCPYQFR

VSASNPWGISLPSEPSEFVRLPEYDAAADGATISWKENFDSAYTELNEIGRGRFSIVKKC

IHKATRKDVAVKFVSKKMKKKEQAAHEAALLQHLQHPQYITLHDTYESPTSYILILELMD

DGRLLDYLMNHDELMEEKVAFYIRDIMEALQYLHNCRVAHLDIKPENLLIDLRIPVPRVK

LIDLEDAVQISGHFHIHHLLGNPEFAAPEVIQGIPVSLGTDIWSIGVLTYVMLSGVSPFL

DESKEETCINVCRVDFSFPHEYFCGVSNAARDFINVILQEDFRRRPTAATCLQHPWLQPH

NGSYSKIPLDTSRLACFIERRKHQNDVRPIPNVKSYIVNRVNQGT

>sp|O15417|TNC18_HUMAN 1676 NRKALAKGLGL

MDGRDFGPQRSVHGPPPPLLSGLAMDSHRVGAATAGRLPASGLPGPLPPGKYMAGLNLHP

HPGEAFLGSFVASGMGPSASSHGSPVPLPSDLSFRSPTPSNLPMVQLWAAHAHEGFSHLP

SGLYPSYLHLNHLEPPSSGSPLLSQLGQPSIFDTQKGQGPGGDGFYLPTAGAPGSLHSHA

PSARTPGGGHSSGAPAKGSSSRDGPAKERAGRGGEPPPLFGKKDPRARGEEASGPRGVVD

LTQEARAEGRQDRGPPRLAERLSPFLAESKTKNAALQPSVLTMCNGGAGDVGLPALVAEA

GRGGAKEAARQDEGARLLRRTETLLPGPRPCPSPLPPPPAPPKGPPAPPAATPAGVYTVF

REQGREHRVVAPTFVPSVEAFDERPGPIQIASQARDARAREREAGRPGVLQAPPGSPRPL

DRPEGLREKNSVIRSLKRPPPADAPTVRATRASPDPRAYVPAKELLKPEADPRPCERAPR

GPAGPAAQQAAKLFGLEPGRPPPTGPEHKWKPFELGNFAATQMAVLAAQHHHSRAEEEAA

VVAASSSKKAYLDPGAVLPRSAATCGRPVADMHSAAHGSGEASAMQSLIKYSGSFARDAV

AVRPGGCGKKSPFGGLGTMKPEPAPTSAGASRAQARLPHSGGPAAGGGRQLKRDPERPES

AKAFGREGSGAQGEAEVRHPPVGIAVAVARQKDSGGSGRLGPGLVDQERSLSLSNVKGHG

RADEDCVDDRARHREERLLGARLDRDQEKLLRESKELADLARLHPTSCAPNGLNPNLMVT

GGPALAGSGRWSADPAAHLATHPWLPRSGNASMWLAGHPYGLGPPSLHQGMAPAFPPGLG

GSLPSAYQFVRDPQSGQLVVIPSDHLPHFAELMERATVPPLWPALYPPGRSPLHHAQQLQ

LFSQQHFLRQQEFLYLQQQAAQALELQRSAQLVQERLKAQEHRAEMEEKGSKRGLEAAGK

AGLATAGPGLLPRKPPGLAAGPAGTYGKAVSPPPSPRASPVAALKAKVIQKLEDVSKPPA

YAYPATPSSHPTSPPPASPPPTPGITRKEEAPENVVEKKDLELEKEAPSPFQALFSDIPP

RYPFQALPPHYGRPYPFLLQPTAAADADGLAPDVPLPADGPERLALSPEDKPIRLSPSKI

TEPLREGPEEEPLAEREVKAEVEDMDEGPTELPPLESPLPLPAAEAMATPSPAGGCGGGL

LEAQALSATGQSCAEPSECPDFVEGPEPRVDSPGRTEPCTAALDLGVQLTPETLVEAKEE

PVEVPVAVPVVEAVPEEGLAQVAPSESQPTLEMSDCDVPAGEGQCPSLEPQEAVPVLGST

CFLEEASSDQFLPSLEDPLAGMNALAAAAELPQARPLPSPGAAGAQALEKLEAAESLVLE

QSFLHGITLLSEIAELELERRSQEMGGAERALVARPSLESLLAAGSHMLREVLDGPVVDP

LKNLRLPRELKPNKKYSWMRKKEERMYAMKSSLEDMDALELDFRMRLAEVQRQYKEKQRE

LVKLQRRRDSEDRREEPHRSLARRGPGRPRKRTHAPSALSPPRKRGKSGHSSGKLSSKSL

LTSDDYELGAGIRKRHKGSEEEHDALIGMGKARGRNQTWDEHEASSDFISQLKIKKKKMA

SDQEQLASKLDKALSLTKQDKLKSPFKFSDSAGGKSKTSGGCGRYLTPYDSLLGKNRKAL

AKGLGLSLKSSREGKHKRAAKTRKMEVGFKARGQPKSAHSPFASEVSSYSYNTDSEEDEE

FLKDEWPAQGPSSSKLTPSLLCSMVAKNSKAAGGPKLTKRGLAAPRTLKPKPATSRKQPF

CLLLREAEARSSFSDSSEESFDQDESSEEEDEEEELEEEDEASGGGYRLGARERALSPGL

EESGLGLLARFAASALPSPTVGPSLSVVQLEAKQKARKKEERQSLLGTEFEYTDSESEVK

VRKRSPAGLLRPKKGLGEPGPSLAAPTPGARGPDPSSPDKAKLAVEKGRKARKLRGPKEP

GFEAGPEASDDDLWTRRRSERIFLHDASAAAPAPVSTAPATKTSRCAKGGPLSPRKDAGR

AKDRKDPRKKKKGKEAGPGAGLPPPRAPALPSEARAPHASSLTAAKRSKAKAKGKEVKKE

NRGKGGAVSKLMESMAAEEDFEPNQDSSFSEDEHLPRGGAVERPLTPAPRSCIIDKDELK

DGLRVLIPMDDKLLYAGHVQTVHSPDIYRVVVEGERGNRPHIYCLEQLLQEAIIDVRPAS

TRFLPQGTRIAAYWSQQYRCLYPGTVVRGLLDLEDDGDLITVEFDDGDTGRIPLSHIRLL

PPDYKIQCAEPSPALLVPSAKRRSRKTSKDTGEGKDGGTAGSEEPGAKARGRGRKPSAKA

KGDRAATLEEGNPTDEVPSTPLALEPSSTPGSKKSPPEPVDKRAKAPKARPAPPQPSPAP

PAFTSCPAPEPFAELPAPATSLAPAPLITMPATRPKPKKARAAEESGAKGPRRPGEEAEL

LVKLDHEGVTSPKSKKAKEALLLREDPGAGGWQEPKSLLSLGSYPPAAGSSEPKAPWPKA

TDGDLAQEPGPGLTFEDSGNPKSPDKAQAEQDGAEESESSSSSSSGSSSSSSSSSSSGSE

TEGEEEGDKNGDGGCGTGGRNCSAASSRAASPASSSSSSSSSSSSSSSSSSSSSSSSSSS

SSSSSSSSSSSSSSSSSSSSSSSSSSSSSSSTTDEDSSCSSDDEAAPAPTAGPSAQAALP

TKATKQAGKARPSAHSPGKKTPAPQPQAPPPQPTQPLQPKAQAGAKSRPKKREGVHLPTT

KELAKRQRLPSVENRPKIAAFLPARQLWKWFGKPTQRRGMKGKARKLFYKAIVRGKEMIR

IGDCAVFLSAGRPNLPYIGRIQSMWESWGNNMVVRVKWFYHPEETSPGKQFHQGQHWDQK

SSRSLPAALRVSSQRKDFMERALYQSSHVDENDVQTVSHKCLVVGLEQYEQMLKTKKYQD

SEGLYYLAGTYEPTTGMIFSTDGVPVLC

>sp|O95359|TACC2_HUMAN 2929 KEIEELTKI

MGNENSTSDNQRTLSAQTPRSAQPPGNSQNIKRKQQDTPGSPDHRDASSIGSVGLGGFCT

ASESSASLDPCLVSPEVTEPRKDPQGARGPEGSLLPSPPPSQEREHPSSSMPFAECPPEG

CLASPAAAPEDGPQTQSPRREPAPNAPGDIAAAFPAERDSSTPYQEIAAVPSAGRERQPK

EEGQKSSFSFSSGIDQSPGMSPVPLREPMKAPLCGEGDQPGGFESQEKEAAGGFPPAESR

QGVASVQVTPEAPAAAQQGTESSAVLEKSPLKPMAPIPQDPAPRASDRERGQGEAPPQYL

TDDLEFLRACHLPRSNSGAAPEAEVNAASQESCQQPVGAYLPHAELPWGLPSPALVPEAG

GSGKEALDTIDVQGHPQTGMRGTKPNQVVCVAAGGQPEGGLPVSPEPSLLTPTEEAHPAS

SLASFPAAQIPIAVEEPGSSSRESVSKAGMPVSADAAKEVVDAGLVGLERQVSDLGSKGE

HPEGDPGEVPAPSPQERGEHLNTEQSHEVQPGVPPPPLPKEQSHEVQPGAPPPPLPKAPS

ESARGPPGPTDGAKVHEDSTSPAVAKEGSRSPGDSPGGKEEAPEPPDGGDPGNLQGEDSQ

AFSSKRDPEVGKDELSKPSSDAESRDHPSSHSAQPPRKGGAGHTDGPHSQTAEADASGLP

HKLGEEDPVLPPVPDGAGEPTVPEGAIWEGSGLQPKCPDTLQSREGLGRMESFLTLESEK

SDFPPTPVAEVAPKAQEGESTLEIRKMGSCDGEGLLTSPDQPRGPACDASRQEFHAGVPH

PPQGENLAADLGLTALILDQDQQGIPSCPGEGWIRGAASEWPLLSSEKHLQPSQAQPETS

IFDVLKEQAQPPENGKETSPSHPGFKDQGADSSQIHVPVEPQEDNNLPTHGGQEQALGSE

LQSQLPKGTLSDTPTSSPTDMVWESSLTEESELSAPTRQKLPALGEKRPEGACGDGQSSR

VSPPAADVLKDFSLAGNFSRKETCCTGQGPNKSQQALADALEEGSQHEEACQRHPGASEA

ADGCSPLWGLSKREMASGNTGEAPPCQPDSVALLDAVPCLPALAPASPGVTPTQDAPETE

ACDETQEGRQQPVPAPQQKMECWATSDAESPKLLASFPSAGEQGGEAGAAETGGSAGAGD

PGKQQAPEKPGEATLSCGLLQTEHCLTSGEEASTSALRESCQAEHPMASCQDALLPAREL

GGIPRSTMDFSTHQAVPDPKELLLSGPPEVAAPDTPYLHVDSAAQRGAEDSGVKAVSSAD

PRAPGESPCPVGEPPLALENAASLKLFAGSLAPLLQPGAAGGEIPAVQASSGSPKARTTE

GPVDSMPCLDRMPLLAKGKQATGEEKAATAPGAGAKASGEGMAGDAAGETEGSMERMGEP

SQDPKQGTSGGVDTSSEQIATLTGFPDFREHIAKIFEKPVLGALATPGEKAGAGRSAVGK

DLTRPLGPEKLLDGPPGVDVTLLPAPPARLQVEKKQQLAGEAEISHLALQDPASDKLLGP

AGLTWERNLPGAGVGKEMAGVPPTLREDERPEGPGAAWPGLEGQAYSQLERSRQELASGL

PSPAATQELPVERAAAFQVAPHSHGEEAVAQDRIPSGKQHQETSACDSPHGEDGPGDFAH

TGVPGHVPRSTCAPSPQREVLTVPEANSEPWTLDTLGGERRPGVTAGILEMRNALGNQST

PAPPTGEVADTPLEPGKVAGAAGEAEGDITLSTAETQACASGDLPEAGTTRTFSVVAGDL

VLPGSCQDPACSDKAPGMEGTAALHGDSPARPQQAKEQPGPERPIPAGDGKVCVSSPPEP

DETHDPKLQHLAPEELHTDRESPRPGPSMLPSVPKKDAPRVMDKVTSDETRGAEGTESSP

VADDIIQPAAPADLESPTLAASSYHGDVVGQVSTDLIAQSISPAAAHAGLPPSAAEHIVS

PSAPAGDRVEASTPSCPDPAKDLSRSSDSEEAFETPESTTPVKAPPAPPPPPPEVIPEPE

VSTQPPPEEPGCGSETVPVPDGPRSDSVEGSPFRPPSHSFSAVFDEDKPIASSGTYNLDF

DNIELVDTFQTLEPRASDAKNQEGKVNTRRKSTDSVPISKSTLSRSLSLQASDFDGASSS

GNPEAVALAPDAYSTGSSSASSTLKRTKKPRPPSLKKKQTTKKPTETPPVKETQQEPDEE

SLVPSGENLASETKTESAKTEGPSPALLEETPLEPAVGPKAACPLDSESAEGVVPPASGG

GRVQNSPPVGRKTLPLTTAPEAGEVTPSDSGGQEDSPAKGLSVRLEFDYSEDKSSWDNQQ

ENPPPTKKIGKKPVAKMPLRRPKMKKTPEKLDNTPASPPRSPAEPNDIPIAKGTYTFDID

KWDDPNFNPFSSTSKMQESPKLPQQSYNFDPDTCDESVDPFKTSSKTPSSPSKSPASFEI

PASAMEANGVDGDGLNKPAKKKKTPLKTDTFRVKKSPKRSPLSDPPSQDPTPAATPETPP

VISAVVHATDEEKLAVTNQKWTCMTVDLEADKQDYPQPSDLSTFVNETKFSSPTEELDYR

NSYEIEYMEKIGSSLPQDDDAPKKQALYLMFDTSQESPVKSSPVRMSESPTPCSGSSFEE

TEALVNTAAKNQHPVPRGLAPNQESHLQVPEKSSQKELEAMGLGTPSEAIEITAPEGSFA

SADALLSRLAHPVSLCGALDYLEPDLAEKNPPLFAQKLQEELEFAIMRIEALKLARQIAL

ASRSHQDAKREAAHPTDVSISKTALYSRIGTAEVEKPAGLLFQQPDLDSALQIARAEIIT

KEREVSEWKDKYEESRREVMEMRKIVAEYEKTIAQMIEDEQREKSVSHQTVQQLVLEKEQ

ALADLNSVEKSLADLFRRYEKMKEVLEGFRKNEEVLKRCAQEYLSRVKKEEQRYQALKVH

AEEKLDRANAEIAQVRGKAQQEQAAHQASLRKEQLRVDALERTLEQKNKEIEELTKICDE

LIAKMGKS

>sp|Q12802|AKP13_HUMAN 1683 SPLTKSISL

MKLNPQQAPLYGDCVVTVLLAEEDKAEDDVVFYLVFLGSTLRHCTSTRKVSSDTLETIAP

GHDCCETVKVQLCASKEGLPVFVVAEEDFHFVQDEAYDAAQFLATSAGNQQALNFTRFLD

QSGPPSGDVNSLDKKLVLAFRHLKLPTEWNVLGTDQSLHDAGPRETLMHFAVRLGLLRLT

WFLLQKPGGRGALSIHNQEGATPVSLALERGYHKLHQLLTEENAGEPDSWSSLSYEIPYG

DCSVRHHRELDIYTLTSESDSHHEHPFPGDGCTGPIFKLMNIQQQLMKTNLKQMDSLMPL

MMTAQDPSSAPETDGQFLPCAPEPTDPQRLSSSEETESTQCCPGSPVAQTESPCDLSSIV

EEENTDRSCRKKNKGVERKGEEVEPAPIVDSGTVSDQDSCLQSLPDCGVKGTEGLSSCGN

RNEETGTKSSGMPTDQESLSSGDAVLQRDLVMEPGTAQYSSGGELGGISTTNVSTPDTAG

EMEHGLMNPDATVWKNVLQGGESTKERFENSNIGTAGASDVHVTSKPVDKISVPNCAPAA

SSLDGNKPAESSLAFSNEETSTEKTAETETSRSREESADAPVDQNSVVIPAAAKDKISDG

LEPYTLLAAGIGEAMSPSDLALLGLEEDVMPHQNSETNSSHAQSQKGKSSPICSTTGDDK

LCADSACQQNTVTSSGDLVAKLCDNIVSESESTTARQPSSQDPPDASHCEDPQAHTVTSD

PVRDTQERADFCPFKVVDNKGQRKDVKLDKPLTNMLEVVSHPHPVVPKMEKELVPDQAVI

SDSTFSLANSPGSESVTKDDALSFVPSQKEKGTATPELHTATDYRDGPDGNSNEPDTRPL

EDRAVGLSTSSTAAELQHGMGNTSLTGLGGEHEGPAPPAIPEALNIKGNTDSSLQSVGKA

TLALDSVLTEEGKLLVVSESSAAQEQDKDKAVTCSSIKENALSSGTLQEEQRTPPPGQDT

QQFHEKSISADCAKDKALQLSNSPGASSAFLKAETEHNKEVAPQVSLLTQGGAAQSLVPP

GASLATESRQEALGAEHNSSALLPCLLPDGSDGSDALNCSQPSPLDVGVKNTQSQGKTSA

CEVSGDVTVDVTGVNALQGMAEPRRENISHNTQDILIPNVLLSQEKNAVLGLPVALQDKA

VTDPQGVGTPEMIPLDWEKGKLEGADHSCTMGDAEEAQIDDEAHPVLLQPVAKELPTDME

LSAHDDGAPAGVREVMRAPPSGRERSTPSLPCMVSAQDAPLPKGADLIEEAASRIVDAVI

EQVKAAGALLTEGEACHMSLSSPELGPLTKGLESAFTEKVSTFPPGESLPMGSTPEEATG

SLAGCFAGREEPEKIILPVQGPEPAAEMPDVKAEDEVDFRASSISEEVAVGSIAATLKMK

QGPMTQAINRENWCTIEPCPDAASLLASKQSPECENFLDVGLGRECTSKQGVLKRESGSD

SDLFHSPSDDMDSIIFPKPEEEHLACDITGSSSSTDDTASLDRHSSHGSDVSLSQILKPN

RSRDRQSLDGFYSHGMGAEGRESESEPADPGDVEEEEMDSITEVPANCSVLRSSMRSLSP

FRRHSWGPGKNAASDAEMNHRSSMRVLGDVVRRPPIHRRSFSLEGLTGGAGVGNKPSSSL

EVSSANAEELRHPFSGEERVDSLVSLSEEDLESDQREHRMFDQQICHRSKQQGFNYCTSA

ISSPLTKSISLMTISHPGLDNSRPFHSTFHNTSANLTESITEENYNFLPHSPSKKDSEWK

SGTKVSRTFSYIKNKMSSSKKSKEKEKEKDKIKEKEKDSKDKEKDKKTVNGHTFSSIPVV

GPISCSQCMKPFTNKDAYTCANCSAFVHKGCRESLASCAKVKMKQPKGSLQAHDTSSLPT

VIMRNKPSQPKERPRSAVLLVDETATTPIFANRRSQQSVSLSKSVSIQNITGVGNDENMS

NTWKFLSHSTDSLNKISKVNESTESLTDEGVGTDMNEGQLLGDFEIESKQLEAESWSRII

DSKFLKQQKKDVVKRQEVIYELMQTEFHHVRTLKIMSGVYSQGMMADLLFEQQMVEKLFP

CLDELISIHSQFFQRILERKKESLVDKSEKNFLIKRIGDVLVNQFSGENAERLKKTYGKF

CGQHNQSVNYFKDLYAKDKRFQAFVKKKMSSSVVRRLGIPECILLVTQRITKYPVLFQRI

LQCTKDNEVEQEDLAQSLSLVKDVIGAVDSKVASYEKKVRLNEIYTKTDSKSIMRMKSGQ

MFAKEDLKRKKLVRDGSVFLKNAAGRLKEVQAVLLTDILVFLQEKDQKYIFASLDQKSTV

ISLKKLIVREVAHEEKGLFLISMGMTDPEMVEVHASSKEERNSWIQIIQDTINTLNRDED

EGIPSENEEEKKMLDTRARELKEQLHQKDQKILLLLEEKEMIFRDMAECSTPLPEDCSPT

HSPRVLFRSNTEEALKGGPLMKSAINEVEILQGLVSGNLGGTLGPTVSSPIEQDVVGPVS

LPRRAETFGGFDSHQMNASKGGEKEEGDDGQDLRRTESDSGLKKGGNANLVFMLKRNSEQ

VVQSVVHLYELLSALQGVVLQQDSYIEDQKLVLSERALTRSLSRPSSLIEQEKQRSLEKQ

RQDLANLQKQQAQYLEEKRRREREWEARERELREREALLAQREEEVQQGQQDLEKEREEL

QQKKGTYQYDLERLRAAQKQLEREQEQLRREAERLSQRQTERDLCQVSHPHTKLMRIPSF

FPSPEEPPSPSAPSIAKSGSLDSELSVSPKRNSISRTHKDKGPFHILSSTSQTNKGPEGQ

SQAPASTSASTRLFGLTKPKEKKEKKKKNKTSRSQPGDGPASEVSAEGEEIFC

>sp|P78559|MAP1A_HUMAN 1398 NEAVKQQDKAL

MDGVAEFSEYVSETVDVPSPFDLLEPPTSGGFLKLSKPCCYIFPGGRGDSALFAVNGFNI

LVDGGSDRKSCFWKLVRHLDRIDSVLLTHIGADNLPGINGLLQRKVAELEEEQSQGSSSY

SDWVKNLISPELGVVFFNVPEKLRLPDASRKAKRSIEEACLTLQHLNRLGIQAEPLYRVV

SNTIEPLTLFHKMGVGRLDMYVLNPVKDSKEMQFLMQKWAGNSKAKTGIVLPNGKEAEIS

VPYLTSITALVVWLPANPTEKIVRVLFPGNAPQNKILEGLEKLRHLDFLRYPVATQKDLA

SGAVPTNLKPSKIKQRADSKESLKATTKTAVSKLAKREEVVEEGAKEARSELAKELAKTE

KKAKESSEKPPEKPAKPERVKTESSEALKAEKRKLIKDKVGKKHLKEKISKLEEKKDKEK

KEIKKERKELKKDEGRKEEKKDAKKEEKRKDTKPELKKISKPDLKPFTPEVRKTLYKAKV

PGRVKIDRSRAIRGEKELSSEPQTPPAQKGTVPLPTISGHRELVLSSPEDLTQDFEEMKR

EERALLAEQRDTGLGDKPFPLDTAEEGPPSTAIQGTPPSVPGLGQEEHVMKEKELVPEVP

EEQGSKDRGLDSGAETEEEKDTWEEKKQREAERLPDRTEAREESEPEVKEDVIEKAELEE

MEEVHPSDEEEEDATKAEGFYQKHMQEPLKVTPRSREAFGGRELGLQGKAPEKETSLFLS

SLTTPAGATEHVSYIQDETIPGYSETEQTISDEEIHDEPEERPAPPRFHTSTYDLPGPEG

AGPFEASQPADSAVPATSGKVYGTPETELTYPTNIVAAPLAEEEHVSSATSITECDKLSS

FATSVAEDQSVASLTAPQTEETGKSSLLLDTVTSIPSSRTEATQGLDYVPSAGTISPTSS

LEEDKGFKSPPCEDFSVTGESEKRGEIIGKGLSGERAVEEEEEETANVEMSEKLCSQYGT

PVFSAPGHALHPGEPALGEAEERCLSPDDSTVKMASPPPSGPPSATHTPFHQSPVEEKSE

PQDFQEADSWGDTKRTPGVGKEDAAEETVKPGPEEGTLEKEEKVPPPRSPQAQEAPVNID

EGLTGCTIQLLPAQDKAIVFEIMEAGEPTGPILGAEALPGGLRTLPQEPGKPQKDEVLRY

PDRSLSPEDAESLSVLSVPSPDTANQEPTPKSPCGLTEQYLHKDRWPEVSPEDTQSLSLS

EESPSKETSLDVSSKQLSPESLGTLQFGELNLGKEEMGHLMQAEDTSHHTAPMSVPEPHA

ATASPPTDGTTRYSAQTDITDDSLDRKSPASSFSHSTPSGNGKYLPGAITSPDEHILTPD

SSFSKSPESLPGPALEDIAIKWEDKVPGLKDRTSEQKKEPEPKDEVLQQKDKTLEHKEVV

EPKDTAIYQKDEALHVKNEAVKQQDKALEQKGRDLEQKDTALEQKDKALEPKDKDLEEKD

KALEQKDKIPEEKDKALEQKDTALEQKDKALEPKDKDLEQKDRVLEQKEKIPEEKDKALD

QKVRSVEHKAPEDTVAEMKDRDLEQTDKAPEQKHQAQEQKDKVSEKKDQALEQKYWALGQ

KDEALEQNIQALEENHQTQEQESLVQEDKTRKPKMLEEKSPEKVKAMEEKLEALLEKTKA

LGLEESLVQEGRAREQEEKYWRGQDVVQEWQETSPTREEPAGEQKELAPAWEDTSPEQDN

RYWRGREDVALEQDTYWRELSCERKVWFPHELDGQGARPHYTEERESTFLDEGPDDEQEV

PLREHATRSPWASDFKDFQESSPQKGLEVERWLAESPVGLPPEEEDKLTRSPFEIISPPA

SPPEMVGQRVPSAPGQESPIPDPKLMPHMKNEPTTPSWLADIPPWVPKDRPLPPAPLSPA

PGPPTPAPESHTPAPFSWGTAEYDSVVAAVQEGAAELEGGPYSPLGKDYRKAEGEREEEG

RAEAPDKSSHSSKVPEASKSHATTEPEQTEPEQREPTPYPDERSFQYADIYEQMMLTGLG

PACPTREPPLGAAGDWPPCLSTKEAAAGRNTSAEKELSSPISPKSLQSDTPTFSYAALAG

PTVPPRPEPGPSMEPSLTPPAVPPRAPILSKGPSPPLNGNILSCSPDRRSPSPKESGRSH

WDDSTSDSELEKGAREQPEKEAQSPSPPHPIPMGSPTLWPETEAHVSPPLDSHLGPARPS

LDFPASAFGFSSLQPAPPQLPSPAEPRSAPCGSLAFSGDRALALAPGPPTRTRHDEYLEV

TKAPSLDSSLPQLPSPSSPGAPLLSNLPRPASPALSEGSSSEATTPVISSVAERFSPSLE

AAEQESGELDPGMEPAAHSLWDLTPLSPAPPASLDLALAPAPSLPGDMGDGILPCHLECS

EAATEKPSPFQVPSEDCAANGPTETSPNPPGPAPAKAENEEAAACPAWERGAWPEGAERS

SRPDTLLSPEQPVCPAGGSGGPPSSASPEVEAGPQGCATEPRPHRGELSPSFLNPPLPPS

IDDRDLSTEEVRLVGRGGRRRVGGPGTTGGPCPVTDETPPTSASDSGSSQSDSDVPPETE

ECPSITAEAALDSDEDGDFLPVDKAGGVSGTHHPRPGHDPPPLPQPDPRPSPPRPDVCMA

DPEGLSSESGRVERLREKEKVQGRVGRRAPGKAKPASPARRLDLRGKRSPTPGKGPADRA

SRAPPRPRSTTSQVTPAEEKDGHSPMSKGLVNGLKAGPSALSSKGSSGAPVYVDLAYIPN

HCSGKTADLDFFRRVRASYYVVSGNDPANGEPSRAVLDALLEGKAQWGENLQVSVTLIPT

HDTEVTREWYQQTHEQQQQLNVLVLASSSTVVMQDESFPACKIEF

>sp|Q6KC79|NIPBL_HUMAN 1331 DVIERVIQY

MNGDMPHVPITTLAGIASLTDLLNQLPLPSPLPATTTKSLLFNARIAEEVNCLLACRDDN

LVSQLVHSLNQVSTDHIELKDNLGSDDPEGDIPVLLQAVLARSPNVFREKSMQNRYVQSG

MMMSQYKLSQNSMHSSPASSNYQQTTISHSPSSRFVPPQTSSGNRFMPQQNSPVPSPYAP

QSPAGYMPYSHPSSYTTHPQMQQASVSSPIVAGGLRNIHDNKVSGPLSGNSANHHADNPR

HGSSEDYLHMVHRLSSDDGDSSTMRNAASFPLRSPQPVCSPAGSEGTPKGSRPPLILQSQ

SLPCSSPRDVPPDILLDSPERKQKKQKKMKLGKDEKEQSEKAAMYDIISSPSKDSTKLTL

RLSRVRSSDMDQQEDMISGVENSNVSENDIPFNVQYPGQTSKTPITPQDINRPLNAAQCL

SQQEQTAFLPANQVPVLQQNTSVAAKQPQTSVVQNQQQISQQGPIYDEVELDALAEIERI

ERESAIERERFSKEVQDKDKPLKKRKQDSYPQEAGGATGGNRPASQETGSTGNGSRPALM

VSIDLHQAGRVDSQASITQDSDSIKKPEEIKQCNDAPVSVLQEDIVGSLKSTPENHPETP

KKKSDPELSKSEMKQSESRLAESKPNENRLVETKSSENKLETKVETQTEELKQNESRTTE

CKQNESTIVEPKQNENRLSDTKPNDNKQNNGRSETTKSRPETPKQKGESRPETPKQKSDG

HPETPKQKGDGRPETPKQKGESRPETPKQKNEGRPETPKHRHDNRRDSGKPSTEKKPEVS

KHKQDTKSDSPRLKSERAEALKQRPDGRSVSESLRRDHDNKQKSDDRGESERHRGDQSRV

RRPETLRSSSRNEHGIKSDSSKTDKLERKHRHESGDSRERPSSGEQKSRPDSPRVKQGDS

NKSRSDKLGFKSPTSKDDKRTEGNKSKVDTNKAHPDNKAEFPSYLLGGRSGALKNFVIPK

IKRDKDGNVTQETKKMEMKGEPKDKVEKIGLVEDLNKGAKPVVVLQKLSLDDVQKLIKDR

EDKSRSSLKPIKNKPSKSNKGSIDQSVLKELPPELLAEIESTMPLCERVKMNKRKRSTVN

EKPKYAEISSDEDNDSDEAFESSRKRHKKDDDKAWEYEERDRRSSGDHRRSGHSHEGRRS

SGGGRYRNRSPSDSDMEDYSPPPSLSEVARKMKKKEKQKKRKAYEPKLTPEEMMDSSTFK

RFTASIENILDNLEDMDFTAFGDDDEIPQELLLGKHQLNELGSESAKIKAMGIMDKLSTD

KTVKVLNILEKNIQDGSKLSTLLNHNNDTEEEERLWRDLIMERVTKSADACLTTINIMTS

PNMPKAVYIEDVIERVIQYTKFHLQNTLYPQYDPVYRLDPHGGGLLSSKAKRAKCSTHKQ

RVIVMLYNKVCDIVSSLSELLEIQLLTDTTILQVSSMGITPFFVENVSELQLCAIKLVTA

VFSRYEKHRQLILEEIFTSLARLPTSKRSLRNFRLNSSDMDGEPMYIQMVTALVLQLIQC

VVHLPSSEKDSNAEEDSNKKIDQDVVITNSYETAMRTAQNFLSIFLKKCGSKQGEEDYRP

LFENFVQDLLSTVNKPEWPAAELLLSLLGRLLVHQFSNKSTEMALRVASLDYLGTVAARL

RKDAVTSKMDQGSIERILKQVSGGEDEIQQLQKALLDYLDENTETDPSLVFSRKFYIAQW

FRDTTLETEKAMKSQKDEESSEGTHHAKEIETTGQIMHRAENRKKFLRSIIKTTPSQFST

LKMNSDTVDYDDACLIVRYLASMRPFAQSFDIYLTQILRVLGENAIAVRTKAMKCLSEVV

AVDPSILARLDMQRGVHGRLMDNSTSVREAAVELLGRFVLCRPQLAEQYYDMLIERILDT

GISVRKRVIKILRDICIEQPTFPKITEMCVKMIRRVNDEEGIKKLVNETFQKLWFTPTPH

NDKEAMTRKILNITDVVAACRDTGYDWFEQLLQNLLKSEEDSSYKPVKKACTQLVDNLVE

HILKYEESLADSDNKGVNSGRLVACITTLFLFSKIRPQLMVKHAMTMQPYLTTKCSTQND

FMVICNVAKILELVVPLMEHPSETFLATIEEDLMKLIIKYGMTVVQHCVSCLGAVVNKVT

QNFKFVWACFNRYYGAISKLKSQHQEDPNNTSLLTNKPALLRSLFTVGALCRHFDFDLED

FKGNSKVNIKDKVLELLMYFTKHSDEEVQTKAIIGLGFAFIQHPSLMFEQEVKNLYNNIL

SDKNSSVNLKIQVLKNLQTYLQEEDTRMQQADRDWKKVAKQEDLKEMGDVSSGMSSSIMQ

LYLKQVLEAFFHTQSSVRHFALNVIALTLNQGLIHPVQCVPYLIAMGTDPEPAMRNKADQ

QLVEIDKKYAGFIHMKAVAGMKMSYQVQQAINTCLKDPVRGFRQDESSSALCSHLYSMIR

GNRQHRRAFLISLLNLFDDTAKTDVTMLLYIADNLACFPYQTQEEPLFIMHHIDITLSVS

GSNLLQSFKESMVKDKRKERKSSPSKENESSDSEEEVSRPRKSRKRVDSDSDSDSEDDIN

SVMKCLPENSAPLIEFANVSQGILLLLMLKQHLKNLCGFSDSKIQKYSPSESAKVYDKAI

NRKTGVHFHPKQTLDFLRSDMANSKITEEVKRSIVKQYLDFKLLMEHLDPDEEEEEGEVS

ASTNARNKAITSLLGGGSPKNNTAAETEDDESDGEDRGGGTSGSLRRSKRNSDSTELAAQ

MNESVDVMDVIAICCPKYKDRPQIARVVQKTSSGFSVQWMAGSYSGSWTEAKRRDGRKLV

PWVDTIKESDIIYKKIALTSANKLTNKVVQTLRSLYAAKDGTSS

>sp|O75691|UTP20_HUMAN 626 ALMELFPKL

MKTKPVSHKTENTYRFLTFAERLGNVNIDIIHRIDRTASYEEEVETYFFEGLLKWRELNL

TEHFGKFYKEVIDKCQSFNQLVYHQNEIVQSLKTHLQVKNSFAYQPLLDLVVQLARDLQM

DFYPHFPEFFLTITSILETQDTELLEWAFTSLSYLYKYLWRLMVKDMSSIYSMYSTLLAH

KKLHIRNFAAESFTFLMRKVSDKNALFNLMFLDLDKHPEKVEGVGQLLFEMCKGVRNMFH

SCTGQAVKLILRKLGPVTETETQLPWMLIGETLKNMVKSTVSYISKEHFGTFFECLQESL

LDLHTKVTKTNCCESSEQIKRLLETYLILVKHGSGTKIPTPADVCKVLSQTLQVASLSTS

CWETLLDVISALILGENVSLPETLIKETIEKIFESRFEKRLIFSFSEVMFAMKQFEQLFL

PSFLSYIVNCFLIDDAVVKDEALAILAKLILNKAAPPTAGSMAIEKYPLVFSPQMVGFYI

KQKKTRSKGRNEQFPVLDHLLSIIKLPPNKDTTYLSQSWAALVVLPHIRPLEKEKVIPLV

TGFIEALFMTVDKGSFGKGNLFVLCQAVNTLLSLEESSELLHLVPVERVKNLVLTFPLEP

SVLLLTDLYYQRLALCGCKGPLSQEALMELFPKLQANISTGVSKIRLLTIRILNHFDVQL

PESMEDDGLSERQSVFAILRQAELVPATVNDYREKLLHLRKLRHDVVQTAVPDGPLQEVP

LRYLLGMLYINFSALWDPVIELISSHAHEMENKQFWKVYYEHLEKAATHAEKELQNDMTD

EKSVGDESWEQTQEGDVGALYHEQLALKTDCQERLDHTNFRFLLWRALTKFPERVEPRSR

ELSPLFLRFINNEYYPADLQVAPTQDLRRKGKGMVAEEIEEEPAAGDDEELEEEAVPQDE

SSQKKKTRRAAAKQLIAHLQVFSKFSNPRALYLESKLYELYLQLLLHQDQMVQKITLDCI

MTYKHPHVLPYRENLQRLLEDRSFKEEIVHFSISEDNAVVKTAHRADLFPILMRILYGRM

KNKTGSKTQGKSASGTRMAIVLRFLAGTQPEEIQIFLDLLFEPVRHFKNGECHSAVIQAV

EDLDLSKVLPLGRQHGILNSLEIVLKNISHLISAYLPKILQILLCMTATVSHILDQREKI

QLRFINPLKNLRRLGIKMVTDIFLDWESYQFRTEEIDAVFHGAVWPQISRLGSESQYSPT

PLLKLISIWSRNARYFPLLAKQKPGHPECDILTNVFAILSAKNLSDATASIVMDIVDDLL

NLPDFEPTETVLNLLVTGCVYPGIAENIGESITIGGRLILPHVPAILQYLSKTTISAEKV

KKKKNRAQVSKELGILSKISKFMKDKEQSSVLITLLLPFLHRGNIAEDTEVDILVTVQNL

LKHCVDPTSFLKPIAKLFSVIKNKLSRKLLCTVFETLSDFESGLKYITDVVKLNAFDQRH

LDDINFDVRFETFQTITSYIKEMQIVDVNYLIPVMHNCFYNLELGDMSLSDNASMCLMSI

IKKLPALNVTEKDYREIIHRSLLEKLRKGLKSQTESIQQDYTTILSCLIQTFPNQLEFKD

LVQLTHYHDPEMDFFENMKHIQIHRRARALKKLAKQLMEGKVVLSSKSLQNYIMPYAMTP

IFDEKMLKHENITTAATEIIGAICKHLSWSAYMYYLKHFIHVLQTGQINQKLGVSLLVIV

LEAFHFDHKTLEEQMGKIENEENAIEAIELPEPEAMELERVDEEEKEYTCKSLSDNGQPG

TPDPADSGGTSAKESECITKPVSFLPQNKEEIERTIKNIQGTITGDILPRLHKCLASTTK

REEEHKLVKSKVVNDEEVVRVPLAFAMVKLMQSLPQEVMEANLPSILLKVCALLKNRAQE

IRDIARSTLAKIIEDLGVHFLQYVLKELQTTLVRGYQVHVLTFTVHMLLQGLTNKLQVGD

LDSCLDIMIEIFNHELFGAVAEEKEVKQILSKVMEARRSKSYDSYEILGKFVGKDQVTKL

ILPLKEILQNTTSLKLARKVHETLRRITVGLIVNQEMTAESILLLSYGLISENLPLLTEK

EKNPVAPAPDPRLPPQSCLLLPPTPVRGGQKAVVSRKTNMHIFIESGLRLLHLSLKTSKI

KSSGECVLEMLDPFVSLLIDCLGSMDVKVITGALQCLIWVLRFPLPSIETKAEQLTKHLF

LLLKDYAKLGAARGQNFHLVVNCFKCVTILVKKVKSYQITEKQLQVLLAYAEEDIYDTSR

QATAFGLLKAILSRKLLVPEIDEVMRKVSKLAVSAQSEPARVQCRQVFLKYILDYPLGDK

LRPNLEFMLAQLNYEHETGRESTLEMIAYLFDTFPQGLLHENCGMFFIPLCLMTINDDSA

TCKKMASMTIKSLLGKISLEKKDWLFDMVTTWFGAKKRLNRQLAALICGLFVESEGVDFE

KRLGTVLPVIEKEIDPENFKDIMEETEEKAADRLLFSFLTLITKLIKECNIIQFTKPAET

LSKIWSHVHSHLRHPHNWVWLTAAQIFGLLFASCQPEELIQKWNTKKTKKHLPEPVAIKF

LASDLDQKMKSISLASCHQLHSKFLDQSLGEQVVKNLLFAAKVLYLLELYCEDKQSKIKE

DLEEQEALEDGVACADEKAESDGEEKEEVKEELGRPATLLWLIQKLSRIAKLEAAYSPRN

PLKRTCIFKFLGAVAMDLGIDKVKPYLPMIIAPLFRELNSTYSEQDPLLKNLSQEIIELL

KKLVGLESFSLAFASVQKQANEKRALRKKRKALEFVTNPDIAAKKKMKKHKNKSEAKKRK

IEFLRPGYKAKRQKSHSLKDLAMVE

>sp|Q14643|ITPR1_HUMAN 2434 TLLNVIKSV

MSDKMSSFLHIGDICSLYAEGSTNGFISTLGLVDDRCVVQPETGDLNNPPKKFRDCLFKL

CPMNRYSAQKQFWKAAKPGANSTTDAVLLNKLHHAADLEKKQNETENRKLLGTVIQYGNV

IQLLHLKSNKYLTVNKRLPALLEKNAMRVTLDEAGNEGSWFYIQPFYKLRSIGDSVVIGD

KVVLNPVNAGQPLHASSHQLVDNPGCNEVNSVNCNTSWKIVLFMKWSDNKDDILKGGDVV

RLFHAEQEKFLTCDEHRKKQHVFLRTTGRQSATSATSSKALWEVEVVQHDPCRGGAGYWN

SLFRFKHLATGHYLAAEVDPDFEEECLEFQPSVDPDQDASRSRLRNAQEKMVYSLVSVPE

GNDISSIFELDPTTLRGGDSLVPRNSYVRLRHLCTNTWVHSTNIPIDKEEEKPVMLKIGT

SPVKEDKEAFAIVPVSPAEVRDLDFANDASKVLGSIAGKLEKGTITQNERRSVTKLLEDL

VYFVTGGTNSGQDVLEVVFSKPNRERQKLMREQNILKQIFKLLQAPFTDCGDGPMLRLEE

LGDQRHAPFRHICRLCYRVLRHSQQDYRKNQEYIAKQFGFMQKQIGYDVLAEDTITALLH

NNRKLLEKHITAAEIDTFVSLVRKNREPRFLDYLSDLCVSMNKSIPVTQELICKAVLNPT

NADILIETKLVLSRFEFEGVSSTGENALEAGEDEEEVWLFWRDSNKEIRSKSVRELAQDA

KEGQKEDRDVLSYYRYQLNLFARMCLDRQYLAINEISGQLDVDLILRCMSDENLPYDLRA

SFCRLMLHMHVDRDPQEQVTPVKYARLWSEIPSEIAIDDYDSSGASKDEIKERFAQTMEF

VEEYLRDVVCQRFPFSDKEKNKLTFEVVNLARNLIYFGFYNFSDLLRLTKILLAILDCVH

VTTIFPISKMAKGEENKGNNDVEKLKSSNVMRSIHGVGELMTQVVLRGGGFLPMTPMAAA

PEGNVKQAEPEKEDIMVMDTKLKIIEILQFILNVRLDYRISCLLCIFKREFDESNSQTSE

TSSGNSSQEGPSNVPGALDFEHIEEQAEGIFGGSEENTPLDLDDHGGRTFLRVLLHLTMH

DYPPLVSGALQLLFRHFSQRQEVLQAFKQVQLLVTSQDVDNYKQIKQDLDQLRSIVEKSE

LWVYKGQGPDETMDGASGENEHKKTEEGNNKPQKHESTSSYNYRVVKEILIRLSKLCVQE

SASVRKSRKQQQRLLRNMGAHAVVLELLQIPYEKAEDTKMQEIMRLAHEFLQNFCAGNQQ

NQALLHKHINLFLNPGILEAVTMQHIFMNNFQLCSEINERVVQHFVHCIETHGRNVQYIK

FLQTIVKAEGKFIKKCQDMVMAELVNSGEDVLVFYNDRASFQTLIQMMRSERDRMDENSP

LMYHIHLVELLAVCTEGKNVYTEIKCNSLLPLDDIVRVVTHEDCIPEVKIAYINFLNHCY

VDTEVEMKEIYTSNHMWKLFENFLVDICRACNNTSDRKHADSILEKYVTEIVMSIVTTFF

SSPFSDQSTTLQTRQPVFVQLLQGVFRVYHCNWLMPSQKASVESCIRVLSDVAKSRAIAI

PVDLDSQVNNLFLKSHSIVQKTAMNWRLSARNAARRDSVLAASRDYRNIIERLQDIVSAL

EDRLRPLVQAELSVLVDVLHRPELLFPENTDARRKCESGGFICKLIKHTKQLLEENEEKL

CIKVLQTLREMMTKDRGYGEKLISIDELDNAELPPAPDSENSTEELEPSPPLRQLEDHKR

GEALRQVLVNRYYGNVRPSGRRESLTSFGNGPLSAGGPGKPGGGGGGSGSSSMSRGEMSL

AEVQCHLDKEGASNLVIDLIMNASSDRVFHESILLAIALLEGGNTTIQHSFFCRLTEDKK

SEKFFKVFYDRMKVAQQEIKATVTVNTSDLGNKKKDDEVDRDAPSRKKAKEPTTQITEEV

RDQLLEASAATRKAFTTFRREADPDDHYQPGEGTQATADKAKDDLEMSAVITIMQPILRF

LQLLCENHNRDLQNFLRCQNNKTNYNLVCETLQFLDCICGSTTGGLGLLGLYINEKNVAL

INQTLESLTEYCQGPCHENQNCIATHESNGIDIITALILNDINPLGKKRMDLVLELKNNA

SKLLLAIMESRHDSENAERILYNMRPKELVEVIKKAYMQGEVEFEDGENGEDGAASPRNV

GHNIYILAHQLARHNKELQSMLKPGGQVDGDEALEFYAKHTAQIEIVRLDRTMEQIVFPV

PSICEFLTKESKLRIYYTTERDEQGSKINDFFLRSEDLFNEMNWQKKLRAQPVLYWCARN

MSFWSSISFNLAVLMNLLVAFFYPFKGVRGGTLEPHWSGLLWTAMLISLAIVIALPKPHG

IRALIASTILRLIFSVGLQPTLFLLGAFNVCNKIIFLMSFVGNCGTFTRGYRAMVLDVEF

LYHLLYLVICAMGLFVHEFFYSLLLFDLVYREETLLNVIKSVTRNGRSIILTAVLALILV

YLFSIVGYLFFKDDFILEVDRLPNETAVPETGESLASEFLFSDVCRVESGENCSSPAPRE

ELVPAEETEQDKEHTCETLLMCIVTVLSHGLRSGGGVGDVLRKPSKEEPLFAARVIYDLL

FFFMVIIIVLNLIFGVIIDTFADLRSEKQKKEEILKTTCFICGLERDKFDNKTVTFEEHI

KEEHNMWHYLCFIVLVKVKDSTEYTGPESYVAEMIKERNLDWFPRMRAMSLVSSDSEGEQ

NELRNLQEKLESTMKLVTNLSGQLSELKDQMTEQRKQKQRIGLLGHPPHMNVNPQQPA

>sp|Q14315|FLNC_HUMAN 1796 GELTGEVRM

MMNNSGYSDAGLGLGDETDEMPSTEKDLAEDAPWKKIQQNTFTRWCNEHLKCVGKRLTDL

QRDLSDGLRLIALLEVLSQKRMYRKFHPRPNFRQMKLENVSVALEFLEREHIKLVSIDSK

AIVDGNLKLILGLIWTLILHYSISMPMWEDEDDEDARKQTPKQRLLGWIQNKVPQLPITN

FNRDWQDGKALGALVDNCAPGLCPDWEAWDPNQPVENSREAMQQADDWLGVPQVIAPEEI

VDPNVDEHSVMTYLSQFPKAKLKPGAPVRSKQLNPKKAIAYGPGIEPQGNTVLQPAHFTV

QTVDAGVGEVLVYIEDPEGHTEEAKVVPNNDKDRTYAVSYVPKVAGLHKVTVLFAGQNIE

RSPFEVNVGMALGDANKVSARGPGLEPVGNVANKPTYFDIYTAGAGTGDVAVVIVDPQGR

RDTVEVALEDKGDSTFRCTYRPAMEGPHTVHVAFAGAPITRSPFPVHVSEACNPNACRAS

GRGLQPKGVRVKEVADFKVFTKGAGSGELKVTVKGPKGTEEPVKVREAGDGVFECEYYPV

VPGKYVVTITWGGYAIPRSPFEVQVSPEAGVQKVRAWGPGLETGQVGKSADFVVEAIGTE

VGTLGFSIEGPSQAKIECDDKGDGSCDVRYWPTEPGEYAVHVICDDEDIRDSPFIAHILP

APPDCFPDKVKAFGPGLEPTGCIVDKPAEFTIDARAAGKGDLKLYAQDADGCPIDIKVIP

NGNGTFRCSYVPTKPIKHTIIISWGGVNVPKSPFRVNVGEGSHPERVKVYGPGVEKTGLK

ANEPTYFTVDCSEAGQGDVSIGIKCAPGVVGPAEADIDFDIIKNDNDTFTVKYTPPGAGR

YTIMVLFANQEIPASPFHIKVDPSHDASKVKAEGPGLNRTGVEVGKPTHFTVLTKGAGKA

KLDVQFAGTAKGEVVRDFEIIDNHDYSYTVKYTAVQQGNMAVTVTYGGDPVPKSPFVVNV

APPLDLSKIKVQGLNSKVAVGQEQAFSVNTRGAGGQGQLDVRMTSPSRRPIPCKLEPGGG

AEAQAVRYMPPEEGPYKVDITYDGHPVPGSPFAVEGVLPPDPSKVCAYGPGLKGGLVGTP

APFSIDTKGAGTGGLGLTVEGPCEAKIECQDNGDGSCAVSYLPTEPGEYTINILFAEAHI

PGSPFKATIRPVFDPSKVRASGPGLERGKVGEAATFTVDCSEAGEAELTIEILSDAGVKA

EVLIHNNADGTYHITYSPAFPGTYTITIKYGGHPVPKFPTRVHVQPAVDTSGVKVSGPGV

EPHGVLREVTTEFTVDARSLTATGGNHVTARVLNPSGAKTDTYVTDNGDGTYRVQYTAYE

EGVHLVEVLYDEVAVPKSPFRVGVTEGCDPTRVRAFGPGLEGGLVNKANRFTVETRGAGT

GGLGLAIEGPSEAKMSCKDNKDGSCTVEYIPFTPGDYDVNITFGGRPIPGSPFRVPVKDV

VDPGKVKCSGPGLGAGVRARVPQTFTVDCSQAGRAPLQVAVLGPTGVAEPVEVRDNGDGT

HTVHYTPATDGPYTVAVKYADQEVPRSPFKIKVLPAHDASKVRASGPGLNASGIPASLPV

EFTIDARDAGEGLLTVQILGPEGKPKKANIRDNGDGTYAVSYLPDMSGRYTITIKYGGDE

IPYSPFRIHALPTGDASKCLVTVSIGGHGLGACLGPRIQIGQETVITVDAKAAGEGKVTC

TVSTPDGAELDVDVVENHDGTFDIYYTAPEPGKYVITIRFGGEHIPNSPFHVLACDPLPH

EEEPSEVPQLRQPYAPPRPGARPTHWATEEPVVPVEPMESMLRPFNLVIPFAVQKGELTG

EVRMPSGKTARPNITDNKDGTITVRYAPTEKGLHQMGIKYDGNHIPGSPLQFYVDAINSR

HVSAYGPGLSHGMVNKPATFTIVTKDAGEGGLSLAVEGPSKAEITCKDNKDGTCTVSYLP

TAPGDYSIIVRFDDKHIPGSPFTAKITGDDSMRTSQLNVGTSTDVSLKITESDLSQLTAS

IRAPSGNEEPCLLKRLPNRHIGISFTPKEVGEHVVSVRKSGKHVTNSPFKILVGPSEIGD

ASKVRVWGKGLSEGHTFQVAEFIVDTRNAGYGGLGLSIEGPSKVDINCEDMEDGTCKVTY

CPTEPGTYIINIKFADKHVPGSPFTVKVTGEGRMKESITRRRQAPSIATIGSTCDLNLKI

PGNWFQMVSAQERLTRTFTRSSHTYTRTERTEISKTRGGETKPEVRVEESTQVGGDPFPA

VFGDFLGRERLGSFGSITRQQEGEASSQDMTAQVTSPSGKVEAAEIVEGEDSAYSVRFVP

QEMGPHTVAVKYRGQHVPGSPFQFTVGPLGEGGAHKVRAGRAGLERGVAGVPAEFSIWTR

EAGAGGLSIAVEGPSKAEIAFEDRKDGSCGVSYVVQEPGDYEVSIKFNDEHIPDSPFVVP

VASLSDDARRLTVTSLQETGLKVNQPASFAVQLNGARGVIDARVHTPSGAVEECYVSELD

SDKHTIRFIPHENGVHSIDVKFNGAHIPGSPFKIRVGEQSQAGDPGLVSAYGPGLEGGTT

GVSSEFIVNTLNAGSGALSVTIDGPSKVQLDCRECPEGHVVTYTPMAPGNYLIAIKYGGP

QHIVGSPFKAKVTGPRLSGGHSLHETSTVLVETVTKSSSSRGSSYSSIPKFSSDASKVVT

RGPGLSQAFVGQKNSFTVDCSKAGTNMMMVGVHGPKTPCEEVYVKHMGNRVYNVTYTVKE

KGDYILIVKWGDESVPGSPFKVKVP

>sp|Q14315|FLNC_HUMAN 1649 GLGACLGPRI

MMNNSGYSDAGLGLGDETDEMPSTEKDLAEDAPWKKIQQNTFTRWCNEHLKCVGKRLTDL

QRDLSDGLRLIALLEVLSQKRMYRKFHPRPNFRQMKLENVSVALEFLEREHIKLVSIDSK

AIVDGNLKLILGLIWTLILHYSISMPMWEDEDDEDARKQTPKQRLLGWIQNKVPQLPITN

FNRDWQDGKALGALVDNCAPGLCPDWEAWDPNQPVENSREAMQQADDWLGVPQVIAPEEI

VDPNVDEHSVMTYLSQFPKAKLKPGAPVRSKQLNPKKAIAYGPGIEPQGNTVLQPAHFTV

QTVDAGVGEVLVYIEDPEGHTEEAKVVPNNDKDRTYAVSYVPKVAGLHKVTVLFAGQNIE

RSPFEVNVGMALGDANKVSARGPGLEPVGNVANKPTYFDIYTAGAGTGDVAVVIVDPQGR

RDTVEVALEDKGDSTFRCTYRPAMEGPHTVHVAFAGAPITRSPFPVHVSEACNPNACRAS

GRGLQPKGVRVKEVADFKVFTKGAGSGELKVTVKGPKGTEEPVKVREAGDGVFECEYYPV

VPGKYVVTITWGGYAIPRSPFEVQVSPEAGVQKVRAWGPGLETGQVGKSADFVVEAIGTE

VGTLGFSIEGPSQAKIECDDKGDGSCDVRYWPTEPGEYAVHVICDDEDIRDSPFIAHILP

APPDCFPDKVKAFGPGLEPTGCIVDKPAEFTIDARAAGKGDLKLYAQDADGCPIDIKVIP

NGNGTFRCSYVPTKPIKHTIIISWGGVNVPKSPFRVNVGEGSHPERVKVYGPGVEKTGLK

ANEPTYFTVDCSEAGQGDVSIGIKCAPGVVGPAEADIDFDIIKNDNDTFTVKYTPPGAGR

YTIMVLFANQEIPASPFHIKVDPSHDASKVKAEGPGLNRTGVEVGKPTHFTVLTKGAGKA

KLDVQFAGTAKGEVVRDFEIIDNHDYSYTVKYTAVQQGNMAVTVTYGGDPVPKSPFVVNV

APPLDLSKIKVQGLNSKVAVGQEQAFSVNTRGAGGQGQLDVRMTSPSRRPIPCKLEPGGG

AEAQAVRYMPPEEGPYKVDITYDGHPVPGSPFAVEGVLPPDPSKVCAYGPGLKGGLVGTP

APFSIDTKGAGTGGLGLTVEGPCEAKIECQDNGDGSCAVSYLPTEPGEYTINILFAEAHI

PGSPFKATIRPVFDPSKVRASGPGLERGKVGEAATFTVDCSEAGEAELTIEILSDAGVKA

EVLIHNNADGTYHITYSPAFPGTYTITIKYGGHPVPKFPTRVHVQPAVDTSGVKVSGPGV

EPHGVLREVTTEFTVDARSLTATGGNHVTARVLNPSGAKTDTYVTDNGDGTYRVQYTAYE

EGVHLVEVLYDEVAVPKSPFRVGVTEGCDPTRVRAFGPGLEGGLVNKANRFTVETRGAGT

GGLGLAIEGPSEAKMSCKDNKDGSCTVEYIPFTPGDYDVNITFGGRPIPGSPFRVPVKDV

VDPGKVKCSGPGLGAGVRARVPQTFTVDCSQAGRAPLQVAVLGPTGVAEPVEVRDNGDGT

HTVHYTPATDGPYTVAVKYADQEVPRSPFKIKVLPAHDASKVRASGPGLNASGIPASLPV

EFTIDARDAGEGLLTVQILGPEGKPKKANIRDNGDGTYAVSYLPDMSGRYTITIKYGGDE

IPYSPFRIHALPTGDASKCLVTVSIGGHGLGACLGPRIQIGQETVITVDAKAAGEGKVTC

TVSTPDGAELDVDVVENHDGTFDIYYTAPEPGKYVITIRFGGEHIPNSPFHVLACDPLPH

EEEPSEVPQLRQPYAPPRPGARPTHWATEEPVVPVEPMESMLRPFNLVIPFAVQKGELTG

EVRMPSGKTARPNITDNKDGTITVRYAPTEKGLHQMGIKYDGNHIPGSPLQFYVDAINSR

HVSAYGPGLSHGMVNKPATFTIVTKDAGEGGLSLAVEGPSKAEITCKDNKDGTCTVSYLP

TAPGDYSIIVRFDDKHIPGSPFTAKITGDDSMRTSQLNVGTSTDVSLKITESDLSQLTAS

IRAPSGNEEPCLLKRLPNRHIGISFTPKEVGEHVVSVRKSGKHVTNSPFKILVGPSEIGD

ASKVRVWGKGLSEGHTFQVAEFIVDTRNAGYGGLGLSIEGPSKVDINCEDMEDGTCKVTY

CPTEPGTYIINIKFADKHVPGSPFTVKVTGEGRMKESITRRRQAPSIATIGSTCDLNLKI

PGNWFQMVSAQERLTRTFTRSSHTYTRTERTEISKTRGGETKPEVRVEESTQVGGDPFPA

VFGDFLGRERLGSFGSITRQQEGEASSQDMTAQVTSPSGKVEAAEIVEGEDSAYSVRFVP

QEMGPHTVAVKYRGQHVPGSPFQFTVGPLGEGGAHKVRAGRAGLERGVAGVPAEFSIWTR

EAGAGGLSIAVEGPSKAEIAFEDRKDGSCGVSYVVQEPGDYEVSIKFNDEHIPDSPFVVP

VASLSDDARRLTVTSLQETGLKVNQPASFAVQLNGARGVIDARVHTPSGAVEECYVSELD

SDKHTIRFIPHENGVHSIDVKFNGAHIPGSPFKIRVGEQSQAGDPGLVSAYGPGLEGGTT

GVSSEFIVNTLNAGSGALSVTIDGPSKVQLDCRECPEGHVVTYTPMAPGNYLIAIKYGGP

QHIVGSPFKAKVTGPRLSGGHSLHETSTVLVETVTKSSSSRGSSYSSIPKFSSDASKVVT

RGPGLSQAFVGQKNSFTVDCSKAGTNMMMVGVHGPKTPCEEVYVKHMGNRVYNVTYTVKE

KGDYILIVKWGDESVPGSPFKVKVP

>sp|Q58A63|BMCC1_HUMAN 1906 ALFDGDPHL

MVSNSRTSSTEAVAGSAPLSQGSSGIMELYGSDIEPQPSSVNFIENPPDLNDSNQAQVDA

NVDLVSPDSGLATIRSSRSSKESSVFLSDDSPVGEGAGPHHTLLPGLDSYSPIPEGAVAE

EHAWSGEHGEHFDLFNFDPAPMASGQSQQSSHSADYSPADDFFPNSDLSEGQLPAGPEGL

DGMGTNMSNYSSSSLLSGAGKDSLVEHDEEFVQRQDSPRDNSERNLSLTDFVGDESPSPE

RLKNTGKRIPPTPMNSLVESSPSTEEPASLYTEDMTQKATDTGHMGPPQTHARCSSWWGG

LEIDSKNIADAWSSSEQESVFQSPESWKEHKPSSIDRRASDSVFQPKSLEFTKSGPWESE

FGQPELGSNDIQDKNEESLPFQNLPMEKSPLPNTSPQGTNHLIEDFASLWHSGRSPTAMP

EPWGNPTDDGEPAAVAPFPAWSAFGKEDHDEALKNTWNLHPTSSKTPSVRDPNEWAMAKS

GFAFSSSELLDNSPSEINNEAAPEIWGKKNNDSRDHIFAPGNPSSDLDHTWTNSKPPKED

QNGLVDPKTRGKVYEKVDSWNLFEENMKKGGSDVLVPWEDSFLSYKCSDYSASNLGEDSV

PSPLDTNYSTSDSYTSPTFAGDEKETEHKPFAKEEGFESKDGNSTAEETDIPPQSLQQSS

RNRISSGPGNLDMWASPHTDNSSEINTTHNLDENELKTEHTDGKNISMEDDVGESSQSSY

DDPSMMQLYNETNRQLTLLHSSTNSRQTAPDSLDLWNRVILEDTQSTATISDMDNDLDWD

DCSGGAAIPSDGQTEGYMAEGSEPETRFTVRQLEPWGLEYQEANQVDWELPASDEHTKDS

APSEHHTLNEKSGQLIANSIWDSVMRDKDMSSFMLPGSSHITDSEQRELPPEIPSHSANV

KDTHSPDAPAASGTSESEALISHLDKQDTERETLQSDAASLATRLENPGYFPHPDPWKGH

GDGQSESEKEAQGATDRGHLDEEEVIASGVENASGISEKGQSDQELSSLVASEHQEICIK

SGKISSLAVTFSPQTEEPEEVLEYEEGSYNLDSRDVQTGMSADNLQPKDTHEKHLMSQRN

SGETTETSDGMNFTKYVSVPEKDLEKTEECNFLEPENVGGGPPHRVPRSLDFGDVPIDSD

VHVSSTCSEITKNLDVKGSENSLPGAGSSGNFDRDTISSEYTHSSASSPELNDSSVALSS

WGQQPSSGYQEENQGNWSEQNHQESELITTDGQVEIVTKVKDLEKNRINEFEKSFDRKTP

TFLEIWNDSVDGDSFSSLSSPETGKYSEHSGTHQESNLIASYQEKNEHDISATVQPEDAR

VISTSSGSDDDSVGGEESIEEEIQVANCHVAEDESRAWDSLNESNKFLVTADPKSENIYD

YLDSSEPAENENKSNPFCDNQQSSPDPWTFSPLTETEMQITAVEKEKRSSPETGTTGDVA

WQISPKASFPKNEDNSQLEMLGFSADSTEWWKASPQEGRLIESPFERELSDSSGVLEINS

SVHQNASPWGVPVQGDIEPVETHYTNPFSDNHQSPFLEGNGKNSHEQLWNIQPRQPDPDA

DKFSQLVKLDQIKEKDSREQTFVSAAGDELTPETPTQEQCQDTMLPVCDHPDTAFTHAEE

NSCVTSNVSTNEGQETNQWEQEKSYLGEMTNSSIATENFPAVSSPTQLIMKPGSEWDGST

PSEDSRGTFVPDILHGNFQEGGQLASAAPDLWIDAKKPFSLKADGENPDILTHCEHDSNS

QASDSPDICHDSEAKQETEKHLSACMGPEVESSELCLTEPEIDEEPIYEPGREFVPSNAE

LDSENATVLPPIGYQADIKGSSQPASHKGSPEPSEINGDNSTGLQVSEKGASPDMAPILE

PVDRRIPRIENVATSIFVTHQEPTPEGDGSWISDSFSPESQPGARALFDGDPHLSTENPA

LVPDALLASDTCLDISEAAFDHSFSDASGLNTSTGTIDDMSKLTLSEGHPETPVDGDLGK

QDICSSEASWGDFEYDVMGQNIDEDLLREPEHFLYGGDPPLEEDSLKQSLAPYTPPFDLS

YLTEPAQSAETIEEAGSPEDESLGCRAAEIVLSALPDRRSEGNQAETKNRLPGSQLAVLH

IREDPESVYLPVGAGSNILSPSNVDWEVETDNSDLPAGGDIGPPNGASKEISELEEEKTI

PTKEPEQIKSEYKEERCTEKNEDRHALHMDYILVNREENSHSKPETCEERESIAELELYV

GSKETGLQGTQLASFPDTCQPASLNERKGLSAEKMSSKSDTRSSFESPAQDQSWMFLGHS

EVGDPSLDARDSGPGWSGKTVEPFSELGLGEGPQLQILEEMKPLESLALEEASGPVSQSQ

KSKSRGRAGPDAVTHDNEWEMLSPQPVQKNMIPDTEMEEETEFLELGTRISRPNGLLSED

VGMDIPFEEGVLSPSAADMRPEPPNSLDLNDTHPRRIKLTAPNINLSLDQSEGSILSDDN

LDSPDEIDINVDELDTPDEADSFEYTGHDPTANKDSGQESESIPEYTAEEEREDNRLWRT

VVIGEQEQRIDMKVIEPYRRVISHGGYYGDGLNAIIVFAACFLPDSSRADYHYVMENLFL

YVISTLELMVAEDYMIVYLNGATPRRRMPGLGWMKKCYQMIDRRLRKNLKSFIIVHPSWF

IRTILAVTRPFISSKFSSKIKYVNSLSELSGLIPMDCIHIPESIIKLDEELREASEAAKT

SCLYNDPEMSSMEKDIDLKLKEKP

>sp|Q7Z333|SETX_HUMAN 98 LFDITGQDF

MSTCCWCTPGGASTIDFLKRYASNTPSGEFQTADEDLCYCLECVAEYHKARDELPFLHEV

LWELETLRLINHFEKSMKAEIGDDDELYIVDNNGEMPLFDITGQDFENKLRVPLLEILKY

PYLLLHERVNELCVEALCRMEQANCSFQVFDKHPGIYLFLVHPNEMVRRWAILTARNLGK

VDRDDYYDLQEVLLCLFKVIELGLLESPDIYTSSVLEKGKLILLPSHMYDTTNYKSYWLG

ICMLLTILEEQAMDSLLLGSDKQNDFMQSILHTMEREADDDSVDPFWPALHCFMVILDRL

GSKVWGQLMDPIVAFQTIINNASYNREIRHIRNSSVRTKLEPESYLDDMVTCSQIVYNYN

PEKTKKDSGWRTAICPDYCPNMYEEMETLASVLQSDIGQDMRVHNSTFLWFIPFVQSLMD

LKDLGVAYIAQVVNHLYSEVKEVLNQTDAVCDKVTEFFLLILVSVIELHRNKKCLHLLWV

SSQQWVEAVVKCAKLPTTAFTRSSEKSSGNCSKGTAMISSLSLHSMPSNSVQLAYVQLIR

SLLKEGYQLGQQSLCKRFWDKLNLFLRGNLSLGWQLTSQETHELQSCLKQIIRNIKFKAP

PCNTFVDLTSACKISPASYNKEESEQMGKTSRKDMHCLEASSPTFSKEPMKVQDSVLIKA

DNTIEGDNNEQNYIKDVKLEDHLLAGSCLKQSSKNIFTERAEDQIKISTRKQKSVKEISS

YTPKDCTSRNGPERGCDRGIIVSTRLLTDSSTDALEKVSTSNEDFSLKDDALAKTSKRKT

KVQKDEICAKLSHVIKKQHRKSTLVDNTINLDENLTVSNIESFYSRKDTGVQKGDGFIHN

LSLDPSGVLDDKNGEQKSQNNVLPKEKQLKNEELVIFSFHENNCKIQEFHVDGKELIPFT

EMTNASEKKSSPFKDLMTVPESRDEEMSNSTSVIYSNLTREQAPDISPKSDTLTDSQIDR

DLHKLSLLAQASVITFPSDSPQNSSQLQRKVKEDKRCFTANQNNVGDTSRGQVIIISDSD

DDDDERILSLEKLTKQDKICLEREHPEQHVSTVNSKEEKNPVKEEKTETLFQFEESDSQC

FEFESSSEVFSVWQDHPDDNNSVQDGEKKCLAPIANTTNGQGCTDYVSEVVKKGAEGIEE

HTRPRSISVEEFCEIEVKKPKRKRSEKPMAEDPVRPSSSVRNEGQSDTNKRELVGNDFKS

IDRRTSTPNSRIQRATTVSQKKSSKLCTCTEPIRKVPVSKTPKKTHSDAKKRQNRSSNYL

SCRTTPAIVPPKKFRQCPEPTSTAEKLGLKKGPRKAYELSQRSLDYVAQLRDHGKTVGVV

DTRKKTKLISPQNLSVRNNKKLLTSQELQMQRQIRPKSQKNRRRLSDCESTDVKRAGSHT

AQNSDVFVPESDRSDYNCTGGTEVLANSNRKQLIKCMPSEPETIKAKHGSPATDDACPLN

QCDSVVLNGTVPTNEVIVSTSEDPLGGGDPTARHIEMAALKEGEPDSSSDAEEDNLFLTQ

NDPEDMDLCSQMENDNYKLIELIHGKDTVEVEEDSVSRPQLESLSGTKCKYKDCLETTKN

QGEYCPKHSEVKAADEDVFRKPGLPPPASKPLRPTTKIFSSKSTSRIAGLSKSLETSSAL

SPSLKNKSKGIQSILKVPQPVPLIAQKPVGEMKNSCNVLHPQSPNNSNRQGCKVPFGESK

YFPSSSPVNILLSSQSVSDTFVKEVLKWKYEMFLNFGQCGPPASLCQSISRPVPVRFHNY

GDYFNVFFPLMVLNTFETVAQEWLNSPNRENFYQLQVRKFPADYIKYWEFAVYLEECELA

KQLYPKENDLVFLAPERINEEKKDTERNDIQDLHEYHSGYVHKFRRTSVMRNGKTECYLS

IQTQENFPANLNELVNCIVISSLVTTQRKLKAMSLLGSRNQLARAVLNPNPMDFCTKDLL

TTTSERIIAYLRDFNEDQKKAIETAYAMVKHSPSVAKICLIHGPPGTGKSKTIVGLLYRL

LTENQRKGHSDENSNAKIKQNRVLVCAPSNAAVDELMKKIILEFKEKCKDKKNPLGNCGD

INLVRLGPEKSINSEVLKFSLDSQVNHRMKKELPSHVQAMHKRKEFLDYQLDELSRQRAL

CRGGREIQRQELDENISKVSKERQELASKIKEVQGRPQKTQSIIILESHIICCTLSTSGG

LLLESAFRGQGGVPFSCVIVDEAGQSCEIETLTPLIHRCNKLILVGDPKQLPPTVISMKA

QEYGYDQSMMARFCRLLEENVEHNMISRLPILQLTVQYRMHPDICLFPSNYVYNRNLKTN

RQTEAIRCSSDWPFQPYLVFDVGDGSERRDNDSYINVQEIKLVMEIIKLIKDKRKDVSFR

NIGIITHYKAQKTMIQKDLDKEFDRKGPAEVDTVDAFQGRQKDCVIVTCVRANSIQGSIG

FLASLQRLNVTITRAKYSLFILGHLRTLMENQHWNQLIQDAQKRGAIIKTCDKNYRHDAV

KILKLKPVLQRSLTHPPTIAPEGSRPQGGLPSSKLDSGFAKTSVAASLYHTPSDSKEITL

TVTSKDPERPPVHDQLQDPRLLKRMGIEVKGGIFLWDPQPSSPQHPGATPPTGEPGFPVV

HQDLSHIQQPAAVVAALSSHKPPVRGEPPAASPEASTCQSKCDDPEEELCHRREARAFSE

GEQEKCGSETHHTRRNSRWDKRTLEQEDSSSKKRKLL

>sp|P21333|FLNA_HUMAN 856 ATPTSPIRVK

MSSSHSRAGQSAAGAAPGGGVDTRDAEMPATEKDLAEDAPWKKIQQNTFTRWCNEHLKCV

SKRIANLQTDLSDGLRLIALLEVLSQKKMHRKHNQRPTFRQMQLENVSVALEFLDRESIK

LVSIDSKAIVDGNLKLILGLIWTLILHYSISMPMWDEEEDEEAKKQTPKQRLLGWIQNKL

PQLPITNFSRDWQSGRALGALVDSCAPGLCPDWDSWDASKPVTNAREAMQQADDWLGIPQ

VITPEEIVDPNVDEHSVMTYLSQFPKAKLKPGAPLRPKLNPKKARAYGPGIEPTGNMVKK

RAEFTVETRSAGQGEVLVYVEDPAGHQEEAKVTANNDKNRTFSVWYVPEVTGTHKVTVLF

AGQHIAKSPFEVYVDKSQGDASKVTAQGPGLEPSGNIANKTTYFEIFTAGAGTGEVEVVI

QDPMGQKGTVEPQLEARGDSTYRCSYQPTMEGVHTVHVTFAGVPIPRSPYTVTVGQACNP

SACRAVGRGLQPKGVRVKETADFKVYTKGAGSGELKVTVKGPKGEERVKQKDLGDGVYGF

EYYPMVPGTYIVTITWGGQNIGRSPFEVKVGTECGNQKVRAWGPGLEGGVVGKSADFVVE

AIGDDVGTLGFSVEGPSQAKIECDDKGDGSCDVRYWPQEAGEYAVHVLCNSEDIRLSPFM

ADIRDAPQDFHPDRVKARGPGLEKTGVAVNKPAEFTVDAKHGGKAPLRVQVQDNEGCPVE

ALVKDNGNGTYSCSYVPRKPVKHTAMVSWGGVSIPNSPFRVNVGAGSHPNKVKVYGPGVA

KTGLKAHEPTYFTVDCAEAGQGDVSIGIKCAPGVVGPAEADIDFDIIRNDNDTFTVKYTP

RGAGSYTIMVLFADQATPTSPIRVKVEPSHDASKVKAEGPGLSRTGVELGKPTHFTVNAK

AAGKGKLDVQFSGLTKGDAVRDVDIIDHHDNTYTVKYTPVQQGPVGVNVTYGGDPIPKSP

FSVAVSPSLDLSKIKVSGLGEKVDVGKDQEFTVKSKGAGGQGKVASKIVGPSGAAVPCKV

EPGLGADNSVVRFLPREEGPYEVEVTYDGVPVPGSPFPLEAVAPTKPSKVKAFGPGLQGG

SAGSPARFTIDTKGAGTGGLGLTVEGPCEAQLECLDNGDGTCSVSYVPTEPGDYNINILF

ADTHIPGSPFKAHVVPCFDASKVKCSGPGLERATAGEVGQFQVDCSSAGSAELTIEICSE

AGLPAEVYIQDHGDGTHTITYIPLCPGAYTVTIKYGGQPVPNFPSKLQVEPAVDTSGVQC

YGPGIEGQGVFREATTEFSVDARALTQTGGPHVKARVANPSGNLTETYVQDRGDGMYKVE

YTPYEEGLHSVDVTYDGSPVPSSPFQVPVTEGCDPSRVRVHGPGIQSGTTNKPNKFTVET

RGAGTGGLGLAVEGPSEAKMSCMDNKDGSCSVEYIPYEAGTYSLNVTYGGHQVPGSPFKV

PVHDVTDASKVKCSGPGLSPGMVRANLPQSFQVDTSKAGVAPLQVKVQGPKGLVEPVDVV

DNADGTQTVNYVPSREGPYSISVLYGDEEVPRSPFKVKVLPTHDASKVKASGPGLNTTGV

PASLPVEFTIDAKDAGEGLLAVQITDPEGKPKKTHIQDNHDGTYTVAYVPDVTGRYTILI

KYGGDEIPFSPYRVRAVPTGDASKCTVTVSIGGHGLGAGIGPTIQIGEETVITVDTKAAG

KGKVTCTVCTPDGSEVDVDVVENEDGTFDIFYTAPQPGKYVICVRFGGEHVPNSPFQVTA

LAGDQPSVQPPLRSQQLAPQYTYAQGGQQTWAPERPLVGVNGLDVTSLRPFDLVIPFTIK

KGEITGEVRMPSGKVAQPTITDNKDGTVTVRYAPSEAGLHEMDIRYDNMHIPGSPLQFYV

DYVNCGHVTAYGPGLTHGVVNKPATFTVNTKDAGEGGLSLAIEGPSKAEISCTDNQDGTC

SVSYLPVLPGDYSILVKYNEQHVPGSPFTARVTGDDSMRMSHLKVGSAADIPINISETDL

SLLTATVVPPSGREEPCLLKRLRNGHVGISFVPKETGEHLVHVKKNGQHVASSPIPVVIS

QSEIGDASRVRVSGQGLHEGHTFEPAEFIIDTRDAGYGGLSLSIEGPSKVDINTEDLEDG

TCRVTYCPTEPGNYIINIKFADQHVPGSPFSVKVTGEGRVKESITRRRRAPSVANVGSHC

DLSLKIPEISIQDMTAQVTSPSGKTHEAEIVEGENHTYCIRFVPAEMGTHTVSVKYKGQH

VPGSPFQFTVGPLGEGGAHKVRAGGPGLERAEAGVPAEFSIWTREAGAGGLAIAVEGPSK

AEISFEDRKDGSCGVAYVVQEPGDYEVSVKFNEEHIPDSPFVVPVASPSGDARRLTVSSL

QESGLKVNQPASFAVSLNGAKGAIDAKVHSPSGALEECYVTEIDQDKYAVRFIPRENGVY

LIDVKFNGTHIPGSPFKIRVGEPGHGGDPGLVSAYGAGLEGGVTGNPAEFVVNTSNAGAG

ALSVTIDGPSKVKMDCQECPEGYRVTYTPMAPGSYLISIKYGGPYHIGGSPFKAKVTGPR

LVSNHSLHETSSVFVDSLTKATCAPQHGAPGPGPADASKVVAKGLGLSKAYVGQKSSFTV

DCSKAGNNMLLVGVHGPRTPCEEILVKHVGSRLYSVSYLLKDKGEYTLVVKWGDEHIPGS

PYRVVVP

>sp|P21333|FLNA_HUMAN 1802 GEITGEVRM

MSSSHSRAGQSAAGAAPGGGVDTRDAEMPATEKDLAEDAPWKKIQQNTFTRWCNEHLKCV

SKRIANLQTDLSDGLRLIALLEVLSQKKMHRKHNQRPTFRQMQLENVSVALEFLDRESIK

LVSIDSKAIVDGNLKLILGLIWTLILHYSISMPMWDEEEDEEAKKQTPKQRLLGWIQNKL

PQLPITNFSRDWQSGRALGALVDSCAPGLCPDWDSWDASKPVTNAREAMQQADDWLGIPQ

VITPEEIVDPNVDEHSVMTYLSQFPKAKLKPGAPLRPKLNPKKARAYGPGIEPTGNMVKK

RAEFTVETRSAGQGEVLVYVEDPAGHQEEAKVTANNDKNRTFSVWYVPEVTGTHKVTVLF

AGQHIAKSPFEVYVDKSQGDASKVTAQGPGLEPSGNIANKTTYFEIFTAGAGTGEVEVVI

QDPMGQKGTVEPQLEARGDSTYRCSYQPTMEGVHTVHVTFAGVPIPRSPYTVTVGQACNP

SACRAVGRGLQPKGVRVKETADFKVYTKGAGSGELKVTVKGPKGEERVKQKDLGDGVYGF

EYYPMVPGTYIVTITWGGQNIGRSPFEVKVGTECGNQKVRAWGPGLEGGVVGKSADFVVE

AIGDDVGTLGFSVEGPSQAKIECDDKGDGSCDVRYWPQEAGEYAVHVLCNSEDIRLSPFM

ADIRDAPQDFHPDRVKARGPGLEKTGVAVNKPAEFTVDAKHGGKAPLRVQVQDNEGCPVE

ALVKDNGNGTYSCSYVPRKPVKHTAMVSWGGVSIPNSPFRVNVGAGSHPNKVKVYGPGVA

KTGLKAHEPTYFTVDCAEAGQGDVSIGIKCAPGVVGPAEADIDFDIIRNDNDTFTVKYTP

RGAGSYTIMVLFADQATPTSPIRVKVEPSHDASKVKAEGPGLSRTGVELGKPTHFTVNAK

AAGKGKLDVQFSGLTKGDAVRDVDIIDHHDNTYTVKYTPVQQGPVGVNVTYGGDPIPKSP

FSVAVSPSLDLSKIKVSGLGEKVDVGKDQEFTVKSKGAGGQGKVASKIVGPSGAAVPCKV

EPGLGADNSVVRFLPREEGPYEVEVTYDGVPVPGSPFPLEAVAPTKPSKVKAFGPGLQGG

SAGSPARFTIDTKGAGTGGLGLTVEGPCEAQLECLDNGDGTCSVSYVPTEPGDYNINILF

ADTHIPGSPFKAHVVPCFDASKVKCSGPGLERATAGEVGQFQVDCSSAGSAELTIEICSE

AGLPAEVYIQDHGDGTHTITYIPLCPGAYTVTIKYGGQPVPNFPSKLQVEPAVDTSGVQC

YGPGIEGQGVFREATTEFSVDARALTQTGGPHVKARVANPSGNLTETYVQDRGDGMYKVE

YTPYEEGLHSVDVTYDGSPVPSSPFQVPVTEGCDPSRVRVHGPGIQSGTTNKPNKFTVET

RGAGTGGLGLAVEGPSEAKMSCMDNKDGSCSVEYIPYEAGTYSLNVTYGGHQVPGSPFKV

PVHDVTDASKVKCSGPGLSPGMVRANLPQSFQVDTSKAGVAPLQVKVQGPKGLVEPVDVV

DNADGTQTVNYVPSREGPYSISVLYGDEEVPRSPFKVKVLPTHDASKVKASGPGLNTTGV

PASLPVEFTIDAKDAGEGLLAVQITDPEGKPKKTHIQDNHDGTYTVAYVPDVTGRYTILI

KYGGDEIPFSPYRVRAVPTGDASKCTVTVSIGGHGLGAGIGPTIQIGEETVITVDTKAAG

KGKVTCTVCTPDGSEVDVDVVENEDGTFDIFYTAPQPGKYVICVRFGGEHVPNSPFQVTA

LAGDQPSVQPPLRSQQLAPQYTYAQGGQQTWAPERPLVGVNGLDVTSLRPFDLVIPFTIK

KGEITGEVRMPSGKVAQPTITDNKDGTVTVRYAPSEAGLHEMDIRYDNMHIPGSPLQFYV

DYVNCGHVTAYGPGLTHGVVNKPATFTVNTKDAGEGGLSLAIEGPSKAEISCTDNQDGTC

SVSYLPVLPGDYSILVKYNEQHVPGSPFTARVTGDDSMRMSHLKVGSAADIPINISETDL

SLLTATVVPPSGREEPCLLKRLRNGHVGISFVPKETGEHLVHVKKNGQHVASSPIPVVIS

QSEIGDASRVRVSGQGLHEGHTFEPAEFIIDTRDAGYGGLSLSIEGPSKVDINTEDLEDG

TCRVTYCPTEPGNYIINIKFADQHVPGSPFSVKVTGEGRVKESITRRRRAPSVANVGSHC

DLSLKIPEISIQDMTAQVTSPSGKTHEAEIVEGENHTYCIRFVPAEMGTHTVSVKYKGQH

VPGSPFQFTVGPLGEGGAHKVRAGGPGLERAEAGVPAEFSIWTREAGAGGLAIAVEGPSK

AEISFEDRKDGSCGVAYVVQEPGDYEVSVKFNEEHIPDSPFVVPVASPSGDARRLTVSSL

QESGLKVNQPASFAVSLNGAKGAIDAKVHSPSGALEECYVTEIDQDKYAVRFIPRENGVY

LIDVKFNGTHIPGSPFKIRVGEPGHGGDPGLVSAYGAGLEGGVTGNPAEFVVNTSNAGAG

ALSVTIDGPSKVKMDCQECPEGYRVTYTPMAPGSYLISIKYGGPYHIGGSPFKAKVTGPR

LVSNHSLHETSSVFVDSLTKATCAPQHGAPGPGPADASKVVAKGLGLSKAYVGQKSSFTV

DCSKAGNNMLLVGVHGPRTPCEEILVKHVGSRLYSVSYLLKDKGEYTLVVKWGDEHIPGS

PYRVVVP

>sp|P21333|FLNA_HUMAN 613 VEGPSQAKI

MSSSHSRAGQSAAGAAPGGGVDTRDAEMPATEKDLAEDAPWKKIQQNTFTRWCNEHLKCV

SKRIANLQTDLSDGLRLIALLEVLSQKKMHRKHNQRPTFRQMQLENVSVALEFLDRESIK

LVSIDSKAIVDGNLKLILGLIWTLILHYSISMPMWDEEEDEEAKKQTPKQRLLGWIQNKL

PQLPITNFSRDWQSGRALGALVDSCAPGLCPDWDSWDASKPVTNAREAMQQADDWLGIPQ

VITPEEIVDPNVDEHSVMTYLSQFPKAKLKPGAPLRPKLNPKKARAYGPGIEPTGNMVKK

RAEFTVETRSAGQGEVLVYVEDPAGHQEEAKVTANNDKNRTFSVWYVPEVTGTHKVTVLF

AGQHIAKSPFEVYVDKSQGDASKVTAQGPGLEPSGNIANKTTYFEIFTAGAGTGEVEVVI

QDPMGQKGTVEPQLEARGDSTYRCSYQPTMEGVHTVHVTFAGVPIPRSPYTVTVGQACNP

SACRAVGRGLQPKGVRVKETADFKVYTKGAGSGELKVTVKGPKGEERVKQKDLGDGVYGF

EYYPMVPGTYIVTITWGGQNIGRSPFEVKVGTECGNQKVRAWGPGLEGGVVGKSADFVVE

AIGDDVGTLGFSVEGPSQAKIECDDKGDGSCDVRYWPQEAGEYAVHVLCNSEDIRLSPFM

ADIRDAPQDFHPDRVKARGPGLEKTGVAVNKPAEFTVDAKHGGKAPLRVQVQDNEGCPVE

ALVKDNGNGTYSCSYVPRKPVKHTAMVSWGGVSIPNSPFRVNVGAGSHPNKVKVYGPGVA

KTGLKAHEPTYFTVDCAEAGQGDVSIGIKCAPGVVGPAEADIDFDIIRNDNDTFTVKYTP

RGAGSYTIMVLFADQATPTSPIRVKVEPSHDASKVKAEGPGLSRTGVELGKPTHFTVNAK

AAGKGKLDVQFSGLTKGDAVRDVDIIDHHDNTYTVKYTPVQQGPVGVNVTYGGDPIPKSP

FSVAVSPSLDLSKIKVSGLGEKVDVGKDQEFTVKSKGAGGQGKVASKIVGPSGAAVPCKV

EPGLGADNSVVRFLPREEGPYEVEVTYDGVPVPGSPFPLEAVAPTKPSKVKAFGPGLQGG

SAGSPARFTIDTKGAGTGGLGLTVEGPCEAQLECLDNGDGTCSVSYVPTEPGDYNINILF

ADTHIPGSPFKAHVVPCFDASKVKCSGPGLERATAGEVGQFQVDCSSAGSAELTIEICSE

AGLPAEVYIQDHGDGTHTITYIPLCPGAYTVTIKYGGQPVPNFPSKLQVEPAVDTSGVQC

YGPGIEGQGVFREATTEFSVDARALTQTGGPHVKARVANPSGNLTETYVQDRGDGMYKVE

YTPYEEGLHSVDVTYDGSPVPSSPFQVPVTEGCDPSRVRVHGPGIQSGTTNKPNKFTVET

RGAGTGGLGLAVEGPSEAKMSCMDNKDGSCSVEYIPYEAGTYSLNVTYGGHQVPGSPFKV

PVHDVTDASKVKCSGPGLSPGMVRANLPQSFQVDTSKAGVAPLQVKVQGPKGLVEPVDVV

DNADGTQTVNYVPSREGPYSISVLYGDEEVPRSPFKVKVLPTHDASKVKASGPGLNTTGV

PASLPVEFTIDAKDAGEGLLAVQITDPEGKPKKTHIQDNHDGTYTVAYVPDVTGRYTILI

KYGGDEIPFSPYRVRAVPTGDASKCTVTVSIGGHGLGAGIGPTIQIGEETVITVDTKAAG

KGKVTCTVCTPDGSEVDVDVVENEDGTFDIFYTAPQPGKYVICVRFGGEHVPNSPFQVTA

LAGDQPSVQPPLRSQQLAPQYTYAQGGQQTWAPERPLVGVNGLDVTSLRPFDLVIPFTIK

KGEITGEVRMPSGKVAQPTITDNKDGTVTVRYAPSEAGLHEMDIRYDNMHIPGSPLQFYV

DYVNCGHVTAYGPGLTHGVVNKPATFTVNTKDAGEGGLSLAIEGPSKAEISCTDNQDGTC

SVSYLPVLPGDYSILVKYNEQHVPGSPFTARVTGDDSMRMSHLKVGSAADIPINISETDL

SLLTATVVPPSGREEPCLLKRLRNGHVGISFVPKETGEHLVHVKKNGQHVASSPIPVVIS

QSEIGDASRVRVSGQGLHEGHTFEPAEFIIDTRDAGYGGLSLSIEGPSKVDINTEDLEDG

TCRVTYCPTEPGNYIINIKFADQHVPGSPFSVKVTGEGRVKESITRRRRAPSVANVGSHC

DLSLKIPEISIQDMTAQVTSPSGKTHEAEIVEGENHTYCIRFVPAEMGTHTVSVKYKGQH

VPGSPFQFTVGPLGEGGAHKVRAGGPGLERAEAGVPAEFSIWTREAGAGGLAIAVEGPSK

AEISFEDRKDGSCGVAYVVQEPGDYEVSVKFNEEHIPDSPFVVPVASPSGDARRLTVSSL

QESGLKVNQPASFAVSLNGAKGAIDAKVHSPSGALEECYVTEIDQDKYAVRFIPRENGVY

LIDVKFNGTHIPGSPFKIRVGEPGHGGDPGLVSAYGAGLEGGVTGNPAEFVVNTSNAGAG

ALSVTIDGPSKVKMDCQECPEGYRVTYTPMAPGSYLISIKYGGPYHIGGSPFKAKVTGPR

LVSNHSLHETSSVFVDSLTKATCAPQHGAPGPGPADASKVVAKGLGLSKAYVGQKSSFTV

DCSKAGNNMLLVGVHGPRTPCEEILVKHVGSRLYSVSYLLKDKGEYTLVVKWGDEHIPGS

PYRVVVP

>sp|P21333|FLNA_HUMAN 2380 VTEIDQDKY

MSSSHSRAGQSAAGAAPGGGVDTRDAEMPATEKDLAEDAPWKKIQQNTFTRWCNEHLKCV

SKRIANLQTDLSDGLRLIALLEVLSQKKMHRKHNQRPTFRQMQLENVSVALEFLDRESIK

LVSIDSKAIVDGNLKLILGLIWTLILHYSISMPMWDEEEDEEAKKQTPKQRLLGWIQNKL

PQLPITNFSRDWQSGRALGALVDSCAPGLCPDWDSWDASKPVTNAREAMQQADDWLGIPQ

VITPEEIVDPNVDEHSVMTYLSQFPKAKLKPGAPLRPKLNPKKARAYGPGIEPTGNMVKK

RAEFTVETRSAGQGEVLVYVEDPAGHQEEAKVTANNDKNRTFSVWYVPEVTGTHKVTVLF

AGQHIAKSPFEVYVDKSQGDASKVTAQGPGLEPSGNIANKTTYFEIFTAGAGTGEVEVVI

QDPMGQKGTVEPQLEARGDSTYRCSYQPTMEGVHTVHVTFAGVPIPRSPYTVTVGQACNP

SACRAVGRGLQPKGVRVKETADFKVYTKGAGSGELKVTVKGPKGEERVKQKDLGDGVYGF

EYYPMVPGTYIVTITWGGQNIGRSPFEVKVGTECGNQKVRAWGPGLEGGVVGKSADFVVE

AIGDDVGTLGFSVEGPSQAKIECDDKGDGSCDVRYWPQEAGEYAVHVLCNSEDIRLSPFM

ADIRDAPQDFHPDRVKARGPGLEKTGVAVNKPAEFTVDAKHGGKAPLRVQVQDNEGCPVE

ALVKDNGNGTYSCSYVPRKPVKHTAMVSWGGVSIPNSPFRVNVGAGSHPNKVKVYGPGVA

KTGLKAHEPTYFTVDCAEAGQGDVSIGIKCAPGVVGPAEADIDFDIIRNDNDTFTVKYTP

RGAGSYTIMVLFADQATPTSPIRVKVEPSHDASKVKAEGPGLSRTGVELGKPTHFTVNAK

AAGKGKLDVQFSGLTKGDAVRDVDIIDHHDNTYTVKYTPVQQGPVGVNVTYGGDPIPKSP

FSVAVSPSLDLSKIKVSGLGEKVDVGKDQEFTVKSKGAGGQGKVASKIVGPSGAAVPCKV

EPGLGADNSVVRFLPREEGPYEVEVTYDGVPVPGSPFPLEAVAPTKPSKVKAFGPGLQGG

SAGSPARFTIDTKGAGTGGLGLTVEGPCEAQLECLDNGDGTCSVSYVPTEPGDYNINILF

ADTHIPGSPFKAHVVPCFDASKVKCSGPGLERATAGEVGQFQVDCSSAGSAELTIEICSE

AGLPAEVYIQDHGDGTHTITYIPLCPGAYTVTIKYGGQPVPNFPSKLQVEPAVDTSGVQC

YGPGIEGQGVFREATTEFSVDARALTQTGGPHVKARVANPSGNLTETYVQDRGDGMYKVE

YTPYEEGLHSVDVTYDGSPVPSSPFQVPVTEGCDPSRVRVHGPGIQSGTTNKPNKFTVET

RGAGTGGLGLAVEGPSEAKMSCMDNKDGSCSVEYIPYEAGTYSLNVTYGGHQVPGSPFKV

PVHDVTDASKVKCSGPGLSPGMVRANLPQSFQVDTSKAGVAPLQVKVQGPKGLVEPVDVV

DNADGTQTVNYVPSREGPYSISVLYGDEEVPRSPFKVKVLPTHDASKVKASGPGLNTTGV

PASLPVEFTIDAKDAGEGLLAVQITDPEGKPKKTHIQDNHDGTYTVAYVPDVTGRYTILI

KYGGDEIPFSPYRVRAVPTGDASKCTVTVSIGGHGLGAGIGPTIQIGEETVITVDTKAAG

KGKVTCTVCTPDGSEVDVDVVENEDGTFDIFYTAPQPGKYVICVRFGGEHVPNSPFQVTA

LAGDQPSVQPPLRSQQLAPQYTYAQGGQQTWAPERPLVGVNGLDVTSLRPFDLVIPFTIK

KGEITGEVRMPSGKVAQPTITDNKDGTVTVRYAPSEAGLHEMDIRYDNMHIPGSPLQFYV

DYVNCGHVTAYGPGLTHGVVNKPATFTVNTKDAGEGGLSLAIEGPSKAEISCTDNQDGTC

SVSYLPVLPGDYSILVKYNEQHVPGSPFTARVTGDDSMRMSHLKVGSAADIPINISETDL

SLLTATVVPPSGREEPCLLKRLRNGHVGISFVPKETGEHLVHVKKNGQHVASSPIPVVIS

QSEIGDASRVRVSGQGLHEGHTFEPAEFIIDTRDAGYGGLSLSIEGPSKVDINTEDLEDG

TCRVTYCPTEPGNYIINIKFADQHVPGSPFSVKVTGEGRVKESITRRRRAPSVANVGSHC

DLSLKIPEISIQDMTAQVTSPSGKTHEAEIVEGENHTYCIRFVPAEMGTHTVSVKYKGQH

VPGSPFQFTVGPLGEGGAHKVRAGGPGLERAEAGVPAEFSIWTREAGAGGLAIAVEGPSK

AEISFEDRKDGSCGVAYVVQEPGDYEVSVKFNEEHIPDSPFVVPVASPSGDARRLTVSSL

QESGLKVNQPASFAVSLNGAKGAIDAKVHSPSGALEECYVTEIDQDKYAVRFIPRENGVY

LIDVKFNGTHIPGSPFKIRVGEPGHGGDPGLVSAYGAGLEGGVTGNPAEFVVNTSNAGAG

ALSVTIDGPSKVKMDCQECPEGYRVTYTPMAPGSYLISIKYGGPYHIGGSPFKAKVTGPR

LVSNHSLHETSSVFVDSLTKATCAPQHGAPGPGPADASKVVAKGLGLSKAYVGQKSSFTV

DCSKAGNNMLLVGVHGPRTPCEEILVKHVGSRLYSVSYLLKDKGEYTLVVKWGDEHIPGS

PYRVVVP

>sp|Q9ULT8|HECD1_HUMAN 616 ARLGVISKV

MADVDPDTLLEWLQMGQGDERDMQLIALEQLCMLLLMSDNVDRCFETCPPRTFLPALCKI

FLDESAPDNVLEVTARAITYYLDVSAECTRRIVGVDGAIKALCNRLVVVELNNRTSRDLA

EQCVKVLELICTRESGAVFEAGGLNCVLTFIRDSGHLVHKDTLHSAMAVVSRLCGKMEPQ

DSSLEICVESLSSLLKHEDHQVSDGALRCFASLADRFTRRGVDPAPLAKHGLTEELLSRM

AAAGGTVSGPSSACKPGRSTTGAPSTTADSKLSNQVSTIVSLLSTLCRGSPVVTHDLLRS

ELPDSIESALQGDERCVLDTMRLVDLLLVLLFEGRKALPKSSAGSTGRIPGLRRLDSSGE

RSHRQLIDCIRSKDTDALIDAIDTGAFEVNFMDDVGQTLLNWASAFGTQEMVEFLCERGA

DVNRGQRSSSLHYAACFGRPQVAKTLLRHGANPDLRDEDGKTPLDKARERGHSEVVAILQ

SPGDWMCPVNKGDDKKKKDTNKDEEECNEPKGDPEMAPIYLKRLLPVFAQTFQQTMLPSI

RKASLALIRKMIHFCSEALLQEVCDSDVGHNLPTILVEITATVLDQEDDDDGHLLALQII

RDIVDKGGDIYKHQLARLGVISKVSTLAGPSSDDENEEESKPEKEDEPQEDAQELQQGKP

YHWRDWSIIRGRDCLYIWSDAAALELSNGSNGWFRFILDGKLATMYSSGSPEGGSDSSES

RSEFLEKLQRARGQVKPSTSSQPILSAPGPTKLTVGNWSLTCLKEGEIAIHNSDGQQATI

LKEDLPGFVFESNRGTKHSFTAETSLGSEFVTGWTGKRGRKLKSKLEKTKQKVRTMARDL

YDDHFKAVESMPRGVVVTLRNIATQLESSWELHTNRQCIESENTWRDLMKTALKNLIVLL

KDENTISPYEMCSSGLVQALLTVLNNVSIFRATKQKQNEVPKVILSVFKTAFTENEDDES

RPAVALIRKLIAVLESIERLPLHLYDTPGSTYNLQILTRRLRFRLERAPGETALIDRTGR

MLKMEPLATVESLEQYLLKMVAKQWYDFDRSSFVFVRKLREGQNFIFRHQHDFDENGIIY

WIGTNAKTAYEWVNPAAYGLVVVTSSEGRNLPYGRLEDILSRDNSALNCHSNDDKNAWFA

IDLGLWVIPSAYTLRHARGYGRSALRNWVFQVSKDGQNWTSLYTHVDDCSLNEPGSTATW

PLDPPKDEKQGWRHVRIKQMGKNASGQTHYLSLSGFELYGTVNGVCEDQLGKAAKEAEAN

LRRQRRLVRSQVLKYMVPGARVIRGLDWKWRDQDGSPQGEGTVTGELHNGWIDVTWDAGG

SNSYRMGAEGKFDLKLAPGYDPDTVASPKPVSSTVSGTTQSWSSLVKNNCPDKTSAAAGS

SSRKGSSSSVCSVASSSDISLGSTKTERRSEIVMEHSIVSGADVHEPIVVLSSAENVPQT

EVGSSSSASTSTLTAETGSENAERKLGPDSSVRTPGESSAISMGIVSVSSPDVSSVSELT

NKEAASQRPLSSSASNRLSVSSLLAAGAPMSSSASVPNLSSRETSSLESFVRRVANIART

NATNNMNLSRSSSDNNTNTLGRNVMSTATSPLMGAQSFPNLTTPGTTSTVTMSTSSVTSS

SNVATATTVLSVGQSLSNTLTTSLTSTSSESDTGQEAEYSLYDFLDSCRASTLLAELDDD

EDLPEPDEEDDENEDDNQEDQEYEEVMILRRPSLQRRAGSRSDVTHHAVTSQLPQVPAGA

GSRPIGEQEEEEYETKGGRRRTWDDDYVLKRQFSALVPAFDPRPGRTNVQQTTDLEIPPP

GTPHSELLEEVECTPSPRLALTLKVTGLGTTREVELPLTNFRSTIFYYVQKLLQLSCNGN

VKSDKLRRIWEPTYTIMYREMKDSDKEKENGKMGCWSIEHVEQYLGTDELPKNDLITYLQ

KNADAAFLRHWKLTGTNKSIRKNRNCSQLIAAYKDFCEHGTKSGLNQGAISTLQSSDILN

LTKEQPQAKAGNGQNSCGVEDVLQLLRILYIVASDPYSRISQEDGDEQPQFTFPPDEFTS

KKITTKILQQIEEPLALASGALPDWCEQLTSKCPFLIPFETRQLYFTCTAFGASRAIVWL

QNRREATVERTRTTSSVRRDDPGEFRVGRLKHERVKVPRGESLMEWAENVMQIHADRKSV

LEVEFLGEEGTGLGPTLEFYALVAAEFQRTDLGAWLCDDNFPDDESRHVDLGGGLKPPGY

YVQRSCGLFTAPFPQDSDELERITKLFHFLGIFLAKCIQDNRLVDLPISKPFFKLMCMGD

IKSNMSKLIYESRGDRDLHCTESQSEASTEEGHDSLSVGSFEEDSKSEFILDPPKPKPPA

WFNGILTWEDFELVNPHRARFLKEIKDLAIKRRQILSNKGLSEDEKNTKLQELVLKNPSG

SGPPLSIEDLGLNFQFCPSSRIYGFTAVDLKPSGEDEMITMDNAEEYVDLMFDFCMHTGI

QKQMEAFRDGFNKVFPMEKLSSFSHEEVQMILCGNQSPSWAAEDIINYTEPKLGYTRDSP

GFLRFVRVLCGMSSDERKAFLQFTTGCSTLPPGGLANLHPRLTVVRKVDATDASYPSVNT

CVHYLKLPEYSSEEIMRERLLAATMEKGFHLN

>sp|Q9ULT8|HECD1_HUMAN 851 MPRGVVVTL

MADVDPDTLLEWLQMGQGDERDMQLIALEQLCMLLLMSDNVDRCFETCPPRTFLPALCKI

FLDESAPDNVLEVTARAITYYLDVSAECTRRIVGVDGAIKALCNRLVVVELNNRTSRDLA

EQCVKVLELICTRESGAVFEAGGLNCVLTFIRDSGHLVHKDTLHSAMAVVSRLCGKMEPQ

DSSLEICVESLSSLLKHEDHQVSDGALRCFASLADRFTRRGVDPAPLAKHGLTEELLSRM

AAAGGTVSGPSSACKPGRSTTGAPSTTADSKLSNQVSTIVSLLSTLCRGSPVVTHDLLRS

ELPDSIESALQGDERCVLDTMRLVDLLLVLLFEGRKALPKSSAGSTGRIPGLRRLDSSGE

RSHRQLIDCIRSKDTDALIDAIDTGAFEVNFMDDVGQTLLNWASAFGTQEMVEFLCERGA

DVNRGQRSSSLHYAACFGRPQVAKTLLRHGANPDLRDEDGKTPLDKARERGHSEVVAILQ

SPGDWMCPVNKGDDKKKKDTNKDEEECNEPKGDPEMAPIYLKRLLPVFAQTFQQTMLPSI

RKASLALIRKMIHFCSEALLQEVCDSDVGHNLPTILVEITATVLDQEDDDDGHLLALQII

RDIVDKGGDIYKHQLARLGVISKVSTLAGPSSDDENEEESKPEKEDEPQEDAQELQQGKP

YHWRDWSIIRGRDCLYIWSDAAALELSNGSNGWFRFILDGKLATMYSSGSPEGGSDSSES

RSEFLEKLQRARGQVKPSTSSQPILSAPGPTKLTVGNWSLTCLKEGEIAIHNSDGQQATI

LKEDLPGFVFESNRGTKHSFTAETSLGSEFVTGWTGKRGRKLKSKLEKTKQKVRTMARDL

YDDHFKAVESMPRGVVVTLRNIATQLESSWELHTNRQCIESENTWRDLMKTALKNLIVLL

KDENTISPYEMCSSGLVQALLTVLNNVSIFRATKQKQNEVPKVILSVFKTAFTENEDDES

RPAVALIRKLIAVLESIERLPLHLYDTPGSTYNLQILTRRLRFRLERAPGETALIDRTGR

MLKMEPLATVESLEQYLLKMVAKQWYDFDRSSFVFVRKLREGQNFIFRHQHDFDENGIIY

WIGTNAKTAYEWVNPAAYGLVVVTSSEGRNLPYGRLEDILSRDNSALNCHSNDDKNAWFA

IDLGLWVIPSAYTLRHARGYGRSALRNWVFQVSKDGQNWTSLYTHVDDCSLNEPGSTATW

PLDPPKDEKQGWRHVRIKQMGKNASGQTHYLSLSGFELYGTVNGVCEDQLGKAAKEAEAN

LRRQRRLVRSQVLKYMVPGARVIRGLDWKWRDQDGSPQGEGTVTGELHNGWIDVTWDAGG

SNSYRMGAEGKFDLKLAPGYDPDTVASPKPVSSTVSGTTQSWSSLVKNNCPDKTSAAAGS

SSRKGSSSSVCSVASSSDISLGSTKTERRSEIVMEHSIVSGADVHEPIVVLSSAENVPQT

EVGSSSSASTSTLTAETGSENAERKLGPDSSVRTPGESSAISMGIVSVSSPDVSSVSELT

NKEAASQRPLSSSASNRLSVSSLLAAGAPMSSSASVPNLSSRETSSLESFVRRVANIART

NATNNMNLSRSSSDNNTNTLGRNVMSTATSPLMGAQSFPNLTTPGTTSTVTMSTSSVTSS

SNVATATTVLSVGQSLSNTLTTSLTSTSSESDTGQEAEYSLYDFLDSCRASTLLAELDDD

EDLPEPDEEDDENEDDNQEDQEYEEVMILRRPSLQRRAGSRSDVTHHAVTSQLPQVPAGA

GSRPIGEQEEEEYETKGGRRRTWDDDYVLKRQFSALVPAFDPRPGRTNVQQTTDLEIPPP

GTPHSELLEEVECTPSPRLALTLKVTGLGTTREVELPLTNFRSTIFYYVQKLLQLSCNGN

VKSDKLRRIWEPTYTIMYREMKDSDKEKENGKMGCWSIEHVEQYLGTDELPKNDLITYLQ

KNADAAFLRHWKLTGTNKSIRKNRNCSQLIAAYKDFCEHGTKSGLNQGAISTLQSSDILN

LTKEQPQAKAGNGQNSCGVEDVLQLLRILYIVASDPYSRISQEDGDEQPQFTFPPDEFTS

KKITTKILQQIEEPLALASGALPDWCEQLTSKCPFLIPFETRQLYFTCTAFGASRAIVWL

QNRREATVERTRTTSSVRRDDPGEFRVGRLKHERVKVPRGESLMEWAENVMQIHADRKSV

LEVEFLGEEGTGLGPTLEFYALVAAEFQRTDLGAWLCDDNFPDDESRHVDLGGGLKPPGY

YVQRSCGLFTAPFPQDSDELERITKLFHFLGIFLAKCIQDNRLVDLPISKPFFKLMCMGD

IKSNMSKLIYESRGDRDLHCTESQSEASTEEGHDSLSVGSFEEDSKSEFILDPPKPKPPA

WFNGILTWEDFELVNPHRARFLKEIKDLAIKRRQILSNKGLSEDEKNTKLQELVLKNPSG

SGPPLSIEDLGLNFQFCPSSRIYGFTAVDLKPSGEDEMITMDNAEEYVDLMFDFCMHTGI

QKQMEAFRDGFNKVFPMEKLSSFSHEEVQMILCGNQSPSWAAEDIINYTEPKLGYTRDSP

GFLRFVRVLCGMSSDERKAFLQFTTGCSTLPPGGLANLHPRLTVVRKVDATDASYPSVNT

CVHYLKLPEYSSEEIMRERLLAATMEKGFHLN

>sp|O75179|ANR17_HUMAN 744 APRVPVQAL

MEKATVPVAAATAAEGEGSPPAVAAVAGPPAAAEVGGGVGGSSRARSASSPRGMVRVCDL

LLKKKPPQQQHHKAKRNRTCRPPSSSESSSDSDNSGGGGGGGGGGGGGGGTSSNNSEEEE

DDDDEEEEVSEVESFILDQDDLENPMLETASKLLLSGTADGADLRTVDPETQARLEALLE

AAGIGKLSTADGKAFADPEVLRRLTSSVSCALDEAAAALTRMRAESTANAGQSDNRSLAE

ACSEGDVNAVRKLLIEGRSVNEHTEEGESLLCLACSAGYYELAQVLLAMHANVEDRGIKG

DITPLMAAANGGHVKIVKLLLAHKADVNAQSSTGNTALTYACAGGYVDVVKVLLESGASI

EDHNENGHTPLMEAGSAGHVEVARLLLENGAGINTHSNEFKESALTLACYKGHLEMVRFL

LEAGADQEHKTDEMHTALMEACMDGHVEVARLLLDSGAQVNMPADSFESPLTLAACGGHV

ELAALLIERGASLEEVNDEGYTPLMEAAREGHEEMVALLLGQGANINAQTEETQETALTL

ACCGGFLEVADFLIKAGADIELGCSTPLMEAAQEGHLELVKYLLAAGANVHATTATGDTA

LTYACENGHTDVADVLLQAGADLEHESEGGRTPLMKAARAGHVCTVQFLISKGANVNRTT

ANNDHTVLSLACAGGHLAVVELLLAHGADPTHRLKDGSTMLIEAAKGGHTSVVCYLLDYP

NNLLSAPPPDVTQLTPPSHDLNRAPRVPVQALPMVVPPQEPDKPPANVATTLPIRNKAAS

KQKSSSHLPANSQDVQGYITNQSPESIVEEAQGKLTELEQRIKEAIEKNAQLQSLELAHA

DQLTKEKIEELNKTREEQIQKKQKILEELQKVERELQLKTQQQLKKQYLEVKAQRIQLQQ

QQQQSCQHLGLLTPVGVGEQLSEGDYARLQQVDPVLLKDEPQQTAAQMGFAPIQPLAMPQ

ALPLAAGPLPPGSIANLTELQGVIVGQPVLGQAQLAGLGQGILTETQQGLMVASPAQTLN

DTLDDIMAAVSGRASAMSNTPTHSIAASISQPQTPTPSPIISPSAMLPIYPAIDIDAQTE

SNHDTALTLACAGGHEELVQTLLERGASIEHRDKKGFTPLILAATAGHVGVVEILLDNGA

DIEAQSERTKDTPLSLACSGGRQEVVELLLARGANKEHRNVSDYTPLSLAASGGYVNIIK

ILLNAGAEINSRTGSKLGISPLMLAAMNGHTAAVKLLLDMGSDINAQIETNRNTALTLAC

FQGRTEVVSLLLDRKANVEHRAKTGLTPLMEAASGGYAEVGRVLLDKGADVNAPPVPSSR

DTALTIAADKGHYKFCELLIGRGAHIDVRNKKGNTPLWLAANGGHLDVVQLLVQAGADVD

AADNRKITPLMAAFRKGHVKVVRYLVKEVNQFPSDSECMRYIATITDKEMLKKCHLCMES

IVQAKDRQAAEANKNASILLEELDLEKLREESRRLALAAKREKRKEKRRKKKEEQRRKLE

EIEAKNKENFELQAAQEKEKLKVEDEPEVLTEPPSATTTTTIGISATWTTLAGSHGKRNN

TITTTSSKRKNRKNKITPENVQIIFDDPLPISYSQPEKVNGESKSSSTSESGDSDNMRIS

SCSDESSNSNSSRKSDNHSPAVVTTTVSSKKQPSVLVTFPKEERKSVSGKASIKLSETIS

EGTSNSLSTCTKSGPSPLSSPNGKLTVASPKRGQKREEGWKEVVRRSKKVSVPSTVISRV

IGRGGCNINAIREFTGAHIDIDKQKDKTGDRIITIRGGTESTRQATQLINALIKDPDKEI

DELIPKNRLKSSSANSKIGSSAPTTTAANTSLMGIKMTTVALSSTSQTATALTVPAISSA

STHKTIKNPVNNVRPGFPVSLPLAYPPPQFAHALLAAQTFQQIRPPRLPMTHFGGTFPPA

QSTWGPFPVRPLSPARATNSPKPHMVPRHSNQNSSGSQVNSAGSLTSSPTTTTSSSASTV

PGTSTNGSPSSPSVRRQLFVTVVKTSNATTTTVTTTASNNNTAPTNATYPMPTAKEHYPV

SSPSSPSPPAQPGGVSRNSPLDCGSASPNKVASSSEQEAGSPPVVETTNTRPPNSSSSSG

SSSAHSNQQQPPGSVSQEPRPPLQQSQVPPPEVRMTVPPLATSSAPVAVPSTAPVTYPMP

QTPMGCPQPTPKMETPAIRPPPHGTTAPHKNSASVQNSSVAVLSVNHIKRPHSVPSSVQL

PSTLSTQSACQNSVHPANKPIAPNFSAPLPFGPFSTLFENSPTSAHAFWGGSVVSSQSTP

ESMLSGKSSYLPNSDPLHQSDTSKAPGFRPPLQRPAPSPSGIVNMDSPYGSVTPSSTHLG

NFASNISGGQMYGPGAPLGGAPAAANFNRQHFSPLSLLTPCSSASNDSSAQSVSSGVRAP

SPAPSSVPLGSEKPSNVSQDRKVPVPIGTERSARIRQTGTSAPSVIGSNLSTSVGHSGIW

SFEGIGGNQDKVDWCNPGMGNPMIHRPMSDPGVFSQHQAMERDSTGIVTPSGTFHQHVPA

GYMDFPKVGGMPFSVYGNAMIPPVAPIPDGAGGPIFNGPHAADPSWNSLIKMVSSSTENN

GPQTVWTGPWAPHMNSVHMNQLG

>sp|O75179|ANR17_HUMAN 1297 YAEVGRVL

MEKATVPVAAATAAEGEGSPPAVAAVAGPPAAAEVGGGVGGSSRARSASSPRGMVRVCDL

LLKKKPPQQQHHKAKRNRTCRPPSSSESSSDSDNSGGGGGGGGGGGGGGGTSSNNSEEEE

DDDDEEEEVSEVESFILDQDDLENPMLETASKLLLSGTADGADLRTVDPETQARLEALLE

AAGIGKLSTADGKAFADPEVLRRLTSSVSCALDEAAAALTRMRAESTANAGQSDNRSLAE

ACSEGDVNAVRKLLIEGRSVNEHTEEGESLLCLACSAGYYELAQVLLAMHANVEDRGIKG

DITPLMAAANGGHVKIVKLLLAHKADVNAQSSTGNTALTYACAGGYVDVVKVLLESGASI

EDHNENGHTPLMEAGSAGHVEVARLLLENGAGINTHSNEFKESALTLACYKGHLEMVRFL

LEAGADQEHKTDEMHTALMEACMDGHVEVARLLLDSGAQVNMPADSFESPLTLAACGGHV

ELAALLIERGASLEEVNDEGYTPLMEAAREGHEEMVALLLGQGANINAQTEETQETALTL

ACCGGFLEVADFLIKAGADIELGCSTPLMEAAQEGHLELVKYLLAAGANVHATTATGDTA

LTYACENGHTDVADVLLQAGADLEHESEGGRTPLMKAARAGHVCTVQFLISKGANVNRTT

ANNDHTVLSLACAGGHLAVVELLLAHGADPTHRLKDGSTMLIEAAKGGHTSVVCYLLDYP

NNLLSAPPPDVTQLTPPSHDLNRAPRVPVQALPMVVPPQEPDKPPANVATTLPIRNKAAS

KQKSSSHLPANSQDVQGYITNQSPESIVEEAQGKLTELEQRIKEAIEKNAQLQSLELAHA

DQLTKEKIEELNKTREEQIQKKQKILEELQKVERELQLKTQQQLKKQYLEVKAQRIQLQQ

QQQQSCQHLGLLTPVGVGEQLSEGDYARLQQVDPVLLKDEPQQTAAQMGFAPIQPLAMPQ

ALPLAAGPLPPGSIANLTELQGVIVGQPVLGQAQLAGLGQGILTETQQGLMVASPAQTLN

DTLDDIMAAVSGRASAMSNTPTHSIAASISQPQTPTPSPIISPSAMLPIYPAIDIDAQTE

SNHDTALTLACAGGHEELVQTLLERGASIEHRDKKGFTPLILAATAGHVGVVEILLDNGA

DIEAQSERTKDTPLSLACSGGRQEVVELLLARGANKEHRNVSDYTPLSLAASGGYVNIIK

ILLNAGAEINSRTGSKLGISPLMLAAMNGHTAAVKLLLDMGSDINAQIETNRNTALTLAC

FQGRTEVVSLLLDRKANVEHRAKTGLTPLMEAASGGYAEVGRVLLDKGADVNAPPVPSSR

DTALTIAADKGHYKFCELLIGRGAHIDVRNKKGNTPLWLAANGGHLDVVQLLVQAGADVD

AADNRKITPLMAAFRKGHVKVVRYLVKEVNQFPSDSECMRYIATITDKEMLKKCHLCMES

IVQAKDRQAAEANKNASILLEELDLEKLREESRRLALAAKREKRKEKRRKKKEEQRRKLE

EIEAKNKENFELQAAQEKEKLKVEDEPEVLTEPPSATTTTTIGISATWTTLAGSHGKRNN

TITTTSSKRKNRKNKITPENVQIIFDDPLPISYSQPEKVNGESKSSSTSESGDSDNMRIS

SCSDESSNSNSSRKSDNHSPAVVTTTVSSKKQPSVLVTFPKEERKSVSGKASIKLSETIS

EGTSNSLSTCTKSGPSPLSSPNGKLTVASPKRGQKREEGWKEVVRRSKKVSVPSTVISRV

IGRGGCNINAIREFTGAHIDIDKQKDKTGDRIITIRGGTESTRQATQLINALIKDPDKEI

DELIPKNRLKSSSANSKIGSSAPTTTAANTSLMGIKMTTVALSSTSQTATALTVPAISSA

STHKTIKNPVNNVRPGFPVSLPLAYPPPQFAHALLAAQTFQQIRPPRLPMTHFGGTFPPA

QSTWGPFPVRPLSPARATNSPKPHMVPRHSNQNSSGSQVNSAGSLTSSPTTTTSSSASTV

PGTSTNGSPSSPSVRRQLFVTVVKTSNATTTTVTTTASNNNTAPTNATYPMPTAKEHYPV

SSPSSPSPPAQPGGVSRNSPLDCGSASPNKVASSSEQEAGSPPVVETTNTRPPNSSSSSG

SSSAHSNQQQPPGSVSQEPRPPLQQSQVPPPEVRMTVPPLATSSAPVAVPSTAPVTYPMP

QTPMGCPQPTPKMETPAIRPPPHGTTAPHKNSASVQNSSVAVLSVNHIKRPHSVPSSVQL

PSTLSTQSACQNSVHPANKPIAPNFSAPLPFGPFSTLFENSPTSAHAFWGGSVVSSQSTP

ESMLSGKSSYLPNSDPLHQSDTSKAPGFRPPLQRPAPSPSGIVNMDSPYGSVTPSSTHLG

NFASNISGGQMYGPGAPLGGAPAAANFNRQHFSPLSLLTPCSSASNDSSAQSVSSGVRAP

SPAPSSVPLGSEKPSNVSQDRKVPVPIGTERSARIRQTGTSAPSVIGSNLSTSVGHSGIW

SFEGIGGNQDKVDWCNPGMGNPMIHRPMSDPGVFSQHQAMERDSTGIVTPSGTFHQHVPA

GYMDFPKVGGMPFSVYGNAMIPPVAPIPDGAGGPIFNGPHAADPSWNSLIKMVSSSTENN

GPQTVWTGPWAPHMNSVHMNQLG

>sp|O75369|FLNB_HUMAN 1758 GEITGEVHM

MPVTEKDLAEDAPWKKIQQNTFTRWCNEHLKCVNKRIGNLQTDLSDGLRLIALLEVLSQK

RMYRKYHQRPTFRQMQLENVSVALEFLDRESIKLVSIDSKAIVDGNLKLILGLVWTLILH

YSISMPVWEDEGDDDAKKQTPKQRLLGWIQNKIPYLPITNFNQNWQDGKALGALVDSCAP

GLCPDWESWDPQKPVDNAREAMQQADDWLGVPQVITPEEIIHPDVDEHSVMTYLSQFPKA

KLKPGAPLKPKLNPKKARAYGRGIEPTGNMVKQPAKFTVDTISAGQGDVMVFVEDPEGNK

EEAQVTPDSDKNKTYSVEYLPKVTGLHKVTVLFAGQHISKSPFEVSVDKAQGDASKVTAK

GPGLEAVGNIANKPTYFDIYTAGAGVGDIGVEVEDPQGKNTVELLVEDKGNQVYRCVYKP

MQPGPHVVKIFFAGDTIPKSPFVVQVGEACNPNACRASGRGLQPKGVRIRETTDFKVDTK

AAGSGELGVTMKGPKGLEELVKQKDFLDGVYAFEYYPSTPGRYSIAITWGGHHIPKSPFE

VQVGPEAGMQKVRAWGPGLHGGIVGRSADFVVESIGSEVGSLGFAIEGPSQAKIEYNDQN

DGSCDVKYWPKEPGEYAVHIMCDDEDIKDSPYMAFIHPATGGYNPDLVRAYGPGLEKSGC

IVNNLAEFTVDPKDAGKAPLKIFAQDGEGQRIDIQMKNRMDGTYACSYTPVKAIKHTIAV

VWGGVNIPHSPYRVNIGQGSHPQKVKVFGPGVERSGLKANEPTHFTVDCTEAGEGDVSVG

IKCDARVLSEDEEDVDFDIIHNANDTFTVKYVPPAAGRYTIKVLFASQEIPASPFRVKVD

PSHDASKVKAEGPGLSKAGVENGKPTHFTVYTKGAGKAPLNVQFNSPLPGDAVKDLDIID

NYDYSHTVKYTPTQQGNMQVLVTYGGDPIPKSPFTVGVAAPLDLSKIKLNGLENRVEVGK

DQEFTVDTRGAGGQGKLDVTILSPSRKVVPCLVTPVTGRENSTAKFIPREEGLYAVDVTY

DGHPVPGSPYTVEASLPPDPSKVKAHGPGLEGGLVGKPAEFTIDTKGAGTGGLGLTVEGP

CEAKIECSDNGDGTCSVSYLPTKPGEYFVNILFEEVHIPGSPFKADIEMPFDPSKVVASG

PGLEHGKVGEAGLLSVNCSEAGPGALGLEAVSDSGTKAEVSIQNNKDGTYAVTYVPLTAG

MYTLTMKYGGELVPHFPARVKVEPAVDTSRIKVFGPGIEGKDVFREATTDFTVDSRPLTQ

VGGDHIKAHIANPSGASTECFVTDNADGTYQVEYTPFEKGLHVVEVTYDDVPIPNSPFKV

AVTEGCQPSRVQAQGPGLKEAFTNKPNVFTVVTRGAGIGGLGITVEGPSESKINCRDNKD

GSCSAEYIPFAPGDYDVNITYGGAHIPGSPFRVPVKDVVDPSKVKIAGPGLGSGVRARVL

QSFTVDSSKAGLAPLEVRVLGPRGLVEPVNMVDNGDGTHTVTYTPSQEGPYMVSVKYADE

EIPRSPFKVKVLPTYDASKVTASGPGLSSYGVPASLPVDFAIDARDAGEGLLAVQITDQE

GKPKRAIVHDNKDGTYAVTYIPDKTGRYMIGVTYGGDDIPLSPYRIRATQTGDASKCLAT

GPGIASTVKTGEEVGFVVDAKTAGKGKVTCTVLTPDGTEAEADVIENEDGTYDIFYTAAK

PGTYVIYVRFGGVDIPNSPFTVMATDGEVTAVEEAPVNACPPGFRPWVTEEAYVPVSDMN

GLGFKPFDLVIPFAVRKGEITGEVHMPSGKTATPEIVDNKDGTVTVRYAPTEVGLHEMHI

KYMGSHIPESPLQFYVNYPNSGSVSAYGPGLVYGVANKTATFTIVTEDAGEGGLDLAIEG

PSKAEISCIDNKDGTCTVTYLPTLPGDYSILVKYNDKHIPGSPFTAKITDDSRRCSQVKL

GSAADFLLDISETDLSSLTASIKAPSGRDEPCLLKRLPNNHIGISFIPREVGEHLVSIKK

NGNHVANSPVSIMVVQSEIGDARRAKVYGRGLSEGRTFEMSDFIVDTRDAGYGGISLAVE

GPSKVDIQTEDLEDGTCKVSYFPTVPGVYIVSTKFADEHVPGSPFTVKISGEGRVKESIT

RTSRAPSVATVGSICDLNLKIPEINSSDMSAHVTSPSGRVTEAEIVPMGKNSHCVRFVPQ

EMGVHTVSVKYRGQHVTGSPFQFTVGPLGEGGAHKVRAGGPGLERGEAGVPAEFSIWTRE

AGAGGLSIAVEGPSKAEITFDDHKNGSCGVSYIAQEPGNYEVSIKFNDEHIPESPYLVPV

IAPSDDARRLTVMSLQESGLKVNQPASFAIRLNGAKGKIDAKVHSPSGAVEECHVSELEP

DKYAVRFIPHENGVHTIDVKFNGSHVVGSPFKVRVGEPGQAGNPALVSAYGTGLEGGTTG

IQSEFFINTTRAGPGTLSVTIEGPSKVKMDCQETPEGYKVMYTPMAPGNYLISVKYGGPN

HIVGSPFKAKVTGQRLVSPGSANETSSILVESVTRSSTETCYSAIPKASSDASKVTSKGA

GLSKAFVGQKSSFLVDCSKAGSNMLLIGVHGPTTPCEEVSMKHVGNQQYNVTYVVKERGD

YVLAVKWGEEHIPGSPFHVTVP

>sp|O00507|USP9Y_HUMAN 1521 IVDCLTEMY

MTAITHGSPVGGNDSQGQVLDGQSQHLFQQNQTSSPDSSNENSVATPPPEEQGQGDAPPQ

HEDEEPAFPHTELANLDDMINRPRWVVPVLPKGELEVLLEAAIDLSVKGLDVKSEACQRF

FRDGLTISFTKILMDEAVSGWKFEIHRCIINNTHRLVELCVAKLSQDWFPLLELLAMALN

PHCKFHIYNGTRPCELISSNAQLPEEELFARSSDPRSPKGWLVDLINKFGTLNGFQILHD

RFFNGSALNIQIIAALIKPFGQCYEFLSQHTLKKYFIPVIEIVPHLLENLTDEELKKEAK

NEAKNDALSMIIKSLKNLASRISGQDETIKNLEIFRLKMILRLLQISSFNGKMNALNEIN

KVISSVSYYTHRHSNPEEEEWLTAERMAEWIQQNNILSIVLQDSLHQPQYVEKLEKILRF

VIKEKALTLQDLDNIWAAQAGKHEAIVKNVHDLLAKLAWDFSPGQLDHLFDCFKASWTNA

SKKQREKLLELIRRLAEDDKDGVMAHKVLNLLWNLAQSDDVPVDIMDLALSAHIKILDYS

CSQDRDAQKIQWIDHFIEELRTNDKWVIPALKQIREICSLFGEASQNLSQTQRSPHIFYR

HDLINQLQQNHALVTLVAENLATYMNSIRLYAGDHEDYDPQTVRLGSRYSHVQEVQERLN

FLRFLLKDGQLWLCAPQAKQIWKCLAENAVYLCDREACFKWYSKLMGDEPDLDPDINKDF

FESNVLQLDPSLLTENGMKCFERFFKAVNCRERKLIAKRRSYMMDDLELIGLDYLWRVVI

QSSDEIANRAIDLLKEIYTNLGPRLKANQVVIHEDFIQSCFDRLKASYDTLCVFDGDKNS

INCARQEAIRMVRVLTVIKEYINECDSDYHKERMILPMSRAFCGKHLSLIVRFPNQGRQV

DELDIWFHTNDTIGSVRRCIVNRIKANVAHKKIELFVGGELIDSENDRKLIGQLNLKDKS

LITAKLTQINFNMPSSPDSSSDSSTASPGNHRNHYNDGPNLKVESCLPGVIMSVHPKYIS

FLWQFANLGSNLNMPPLKNGARVLMKLMPPDRTAVEKLRTVCLDHANLGEGKLSPPLDSL

FFGPSASQVLYLTEVVYALLMPAGVPLTDGSSDFQVHFLKSGGLPLVLSMLIRNNFLPNT

DMETRRGAYLNALKIAKLLLTAIGYGHVRAVAEACQPVVDGTDPITQINQVTHDQAVVLQ

SALQSIPNPSSECVLRNESILLAQEISNEASRYMPDICVIRAIQKIIWASACGALGLFFS

PNEEITKIYQMTTNGSNKLEVEDEQVCCEALEVMTLCFALLPTALDALSKEKAWQTFIID

LLLHCPSKTVRQLAQEQFFLMCTRCCMGHRPLLFFITLLFTILGSTAREKGKYSGDYFTL

LRHLLNYAYNGNINIPNAEVLLVSEIDWLKRIRDNVKNTGETGVEEPILEGHLGVTKELL

AFQTSEKKYHFGCEKGGANLIKELIDDFIFPASKVYLQYLRSGELPAEQAIPVCSSPVTI

NAGFELLVALAIGCVRNLKQIVDCLTEMYYMGTAITTCEALTEWEYLPPVGPRPPKGFVG

LKNAGATCYMNSVIQQLYMIPSIRNSILAIEGTGSDLHDDMFGDEKQDSESNVDPRDDVF

GYPHQFEDKPALSKTEDRKEYNIGVLRHLQVIFGHLAASQLQYYVPRGFWKQFRLWGEPV

NLREQHDALEFFNSLVDSLDEALKALGHPAILSKVLGGSFADQKICQGCPHRFECEESFT

TLNVDIRNHQNLLDSLEQYIKGDLLEGANAYHCEKCDKKVDTVKRLLIKKLPRVLAIQLK

RFDYDWERECAIKFNDYFEFPRELDMGPYTVAGVANLERDNVNSENELIEQKEQSDNETA

GGTKYRLVGVLVHSGQASGGHYYSYIIQRNGKDDQTDHWYKFDDGDVTECKMDDDEEMKN

QCFGGEYMGEVFDHMMKRMSYRRQKRWWNAYIPFYEQMDMIDEDDEMIRYISELTIARPH

QIIMSPAIERSVRKQNVKFMHNRLQYSLEYFQFVKKLLTCNGVYLNPAPGQDYLLPEAEE

ITMISIQLAARFLFTTGFHTKKIVRGPASDWYDALCVLLRHSKNVRFWFTHNVLFNVSNR

FSEYLLECPSAEVRGAFAKLIVFIAHFSLQDGSCPSPFASPGPSSQACDNLSLSDHLLRA

TLNLLRREVSEHGHHLQQYFNLFVMYANLGVAEKTQLLKLNVPATFMLVSLDEGPGPPIK

YQYAELGKLYSVVSQLIRCCNVSSTMQSSINGNPPLPNPFGDLNLSQPIMPIQQNVLDIL

FVRTSYVKKIIEDCSNSEDTIKLLRFCSWENPQFSSTVLSELLWQVAYSYTYELRPYLDL

LFQILLIEDSWQTHRIHNALKGIPDDRDGLFDTIQRSKNHYQKRAYQCIKCMVALFSSCP

VAYQILQGNGDLKRKWTWAVEWLGDELERRPYTGNPQYSYNNWSPPVQSNETANGYFLER

SHSARMTLAKACELCPEEEPDDQDAPDEHEPSPSEDAPLYPHSPASQYQQNNHVHGQPYT

GPAAHHLNNPQKTGQRTQENYEGNEEVSSPQMKDQ

>sp|O00507|USP9Y_HUMAN 1674 RLWGEPVNL

MTAITHGSPVGGNDSQGQVLDGQSQHLFQQNQTSSPDSSNENSVATPPPEEQGQGDAPPQ

HEDEEPAFPHTELANLDDMINRPRWVVPVLPKGELEVLLEAAIDLSVKGLDVKSEACQRF

FRDGLTISFTKILMDEAVSGWKFEIHRCIINNTHRLVELCVAKLSQDWFPLLELLAMALN

PHCKFHIYNGTRPCELISSNAQLPEEELFARSSDPRSPKGWLVDLINKFGTLNGFQILHD

RFFNGSALNIQIIAALIKPFGQCYEFLSQHTLKKYFIPVIEIVPHLLENLTDEELKKEAK

NEAKNDALSMIIKSLKNLASRISGQDETIKNLEIFRLKMILRLLQISSFNGKMNALNEIN

KVISSVSYYTHRHSNPEEEEWLTAERMAEWIQQNNILSIVLQDSLHQPQYVEKLEKILRF

VIKEKALTLQDLDNIWAAQAGKHEAIVKNVHDLLAKLAWDFSPGQLDHLFDCFKASWTNA

SKKQREKLLELIRRLAEDDKDGVMAHKVLNLLWNLAQSDDVPVDIMDLALSAHIKILDYS

CSQDRDAQKIQWIDHFIEELRTNDKWVIPALKQIREICSLFGEASQNLSQTQRSPHIFYR

HDLINQLQQNHALVTLVAENLATYMNSIRLYAGDHEDYDPQTVRLGSRYSHVQEVQERLN

FLRFLLKDGQLWLCAPQAKQIWKCLAENAVYLCDREACFKWYSKLMGDEPDLDPDINKDF

FESNVLQLDPSLLTENGMKCFERFFKAVNCRERKLIAKRRSYMMDDLELIGLDYLWRVVI

QSSDEIANRAIDLLKEIYTNLGPRLKANQVVIHEDFIQSCFDRLKASYDTLCVFDGDKNS

INCARQEAIRMVRVLTVIKEYINECDSDYHKERMILPMSRAFCGKHLSLIVRFPNQGRQV

DELDIWFHTNDTIGSVRRCIVNRIKANVAHKKIELFVGGELIDSENDRKLIGQLNLKDKS

LITAKLTQINFNMPSSPDSSSDSSTASPGNHRNHYNDGPNLKVESCLPGVIMSVHPKYIS

FLWQFANLGSNLNMPPLKNGARVLMKLMPPDRTAVEKLRTVCLDHANLGEGKLSPPLDSL

FFGPSASQVLYLTEVVYALLMPAGVPLTDGSSDFQVHFLKSGGLPLVLSMLIRNNFLPNT

DMETRRGAYLNALKIAKLLLTAIGYGHVRAVAEACQPVVDGTDPITQINQVTHDQAVVLQ

SALQSIPNPSSECVLRNESILLAQEISNEASRYMPDICVIRAIQKIIWASACGALGLFFS

PNEEITKIYQMTTNGSNKLEVEDEQVCCEALEVMTLCFALLPTALDALSKEKAWQTFIID

LLLHCPSKTVRQLAQEQFFLMCTRCCMGHRPLLFFITLLFTILGSTAREKGKYSGDYFTL

LRHLLNYAYNGNINIPNAEVLLVSEIDWLKRIRDNVKNTGETGVEEPILEGHLGVTKELL

AFQTSEKKYHFGCEKGGANLIKELIDDFIFPASKVYLQYLRSGELPAEQAIPVCSSPVTI

NAGFELLVALAIGCVRNLKQIVDCLTEMYYMGTAITTCEALTEWEYLPPVGPRPPKGFVG

LKNAGATCYMNSVIQQLYMIPSIRNSILAIEGTGSDLHDDMFGDEKQDSESNVDPRDDVF

GYPHQFEDKPALSKTEDRKEYNIGVLRHLQVIFGHLAASQLQYYVPRGFWKQFRLWGEPV

NLREQHDALEFFNSLVDSLDEALKALGHPAILSKVLGGSFADQKICQGCPHRFECEESFT

TLNVDIRNHQNLLDSLEQYIKGDLLEGANAYHCEKCDKKVDTVKRLLIKKLPRVLAIQLK

RFDYDWERECAIKFNDYFEFPRELDMGPYTVAGVANLERDNVNSENELIEQKEQSDNETA

GGTKYRLVGVLVHSGQASGGHYYSYIIQRNGKDDQTDHWYKFDDGDVTECKMDDDEEMKN

QCFGGEYMGEVFDHMMKRMSYRRQKRWWNAYIPFYEQMDMIDEDDEMIRYISELTIARPH

QIIMSPAIERSVRKQNVKFMHNRLQYSLEYFQFVKKLLTCNGVYLNPAPGQDYLLPEAEE

ITMISIQLAARFLFTTGFHTKKIVRGPASDWYDALCVLLRHSKNVRFWFTHNVLFNVSNR

FSEYLLECPSAEVRGAFAKLIVFIAHFSLQDGSCPSPFASPGPSSQACDNLSLSDHLLRA

TLNLLRREVSEHGHHLQQYFNLFVMYANLGVAEKTQLLKLNVPATFMLVSLDEGPGPPIK

YQYAELGKLYSVVSQLIRCCNVSSTMQSSINGNPPLPNPFGDLNLSQPIMPIQQNVLDIL

FVRTSYVKKIIEDCSNSEDTIKLLRFCSWENPQFSSTVLSELLWQVAYSYTYELRPYLDL

LFQILLIEDSWQTHRIHNALKGIPDDRDGLFDTIQRSKNHYQKRAYQCIKCMVALFSSCP

VAYQILQGNGDLKRKWTWAVEWLGDELERRPYTGNPQYSYNNWSPPVQSNETANGYFLER

SHSARMTLAKACELCPEEEPDDQDAPDEHEPSPSEDAPLYPHSPASQYQQNNHVHGQPYT

GPAAHHLNNPQKTGQRTQENYEGNEEVSSPQMKDQ

>sp|Q8IWZ3|ANKH1_HUMAN 995 TLDDLIAAV

MLTDSGGGGTSFEEDLDSVAPRSAPAGASEPPPPGGVGLGIRTVRLFGEAGPASGVGSSG

GGGSGSGTGGGDAALDFKLAAAVLRTGGGGGASGSDEDEVSEVESFILDQEDLDNPVLKT

TSEIFLSSTAEGADLRTVDPETQARLEALLEAAGIGKLSTADGKAFADPEVLRRLTSSVS

CALDEAAAALTRMKAENSHNAGQVDTRSLAEACSDGDVNAVRKLLDEGRSVNEHTEEGES

LLCLACSAGYYELAQVLLAMHANVEDRGNKGDITPLMAASSGGYLDIVKLLLLHDADVNS

QSATGNTALTYACAGGFVDIVKVLLNEGANIEDHNENGHTPLMEAASAGHVEVARVLLDH

GAGINTHSNEFKESALTLACYKGHLDMVRFLLEAGADQEHKTDEMHTALMEACMDGHVEV

ARLLLDSGAQVNMPADSFESPLTLAACGGHVELAALLIERGANLEEVNDEGYTPLMEAAR

EGHEEMVALLLAQGANINAQTEETQETALTLACCGGFSEVADFLIKAGADIELGCSTPLM

EASQEGHLELVKYLLASGANVHATTATGDTALTYACENGHTDVADVLLQAGADLEHESEG

GRTPLMKAARAGHLCTVQFLISKGANVNRATANNDHTVVSLACAGGHLAVVELLLAHGAD

PTHRLKDGSTMLIEAAKGGHTNVVSYLLDYPNNVLSVPTTDVSQLPPPSQDQSQVPRVPT

HTLAMVVPPQEPDRTSQENSPALLGVQKGTSKQKSSSLQVADQDLLPSFHPYQPLECIVE

ETEGKLNELGQRISAIEKAQLKSLELIQGEPLNKDKIEELKKNREEQVQKKKKILKELQK

VERQLQMKTQQQFTKEYLETKGQKDTVSLHQQCSHRGVFPEGEGDGSLPEDHFSELPQVD

TILFKDNDVDDEQQSPPSAEQIDFVPVQPLSSPQCNFSSDLGSNGTNSLELQKVSGNQQI

VGQPQIAITGHDQGLLVQEPDGLMVATPAQTLTDTLDDLIAAVSTRVPTGSNSSSQTTEC

LTPESCSQTTSNVASQSMPPVYPSVDIDAHTESNHDTALTLACAGGHEELVSVLIARDAK

IEHRDKKGFTPLILAATAGHVGVVEILLDKGGDIEAQSERTKDTPLSLACSGGRQEVVDL

LLARGANKEHRNVSDYTPLSLAASGGYVNIIKILLNAGAEINSRTGSKLGISPLMLAAMN

GHVPAVKLLLDMGSDINAQIETNRNTALTLACFQGRAEVVSLLLDRKANVEHRAKTGLTP

LMEAASGGYAEVGRVLLDKGADVNAPPVPSSRDTALTIAADKGHYKFCELLIHRGAHIDV

RNKKGNTPLWLASNGGHFDVVQLLVQAGADVDAADNRKITPLMSAFRKGHVKVVQYLVKE

VNQFPSDIECMRYIATITDKELLKKCHQCVETIVKAKDQQAAEANKNASILLKELDLEKS

REESRKQALAAKREKRKEKRKKKKEEQKRKQEEDEENKPKENSELPEDEDEEENDEDVEQ

EVPIEPPSATTTTTIGISATSATFTNVFGKKRANVVTTPSTNRKNKKNKTKETPPTAHLI

LPEQHMSLAQQKADKNKINGEPRGGGAGGNSDSDNLDSTDCNSESSSGGKSQELNFVMDV

NSSKYPSLLLHSQEEKTSTATSKTQTRLEGEVTPNSLSTSYKTVSLPLSSPNIKLNLTSP

KRGQKREEGWKEVVRRSKKLSVPASVVSRIMGRGGCNITAIQDVTGAHIDVDKQKDKNGE

RMITIRGGTESTRYAVQLINALIQDPAKELEDLIPKNHIRTPASTKSIHANFSSGVGTTA

ASSKNAFPLGAPTLVTSQATTLSTFQPANKLNKNVPTNVRSSFPVSLPLAYPHPHFALLA

AQTMQQIRHPRLPMAQFGGTFSPSPNTWGPFPVRPVNPGNTNSSPKHNNTSRLPNQNGTV

LPSESAGLATASCPITVSSVVAASQQLCVTNTRTPSSVRKQLFACVPKTSPPATVISSVT

STCSSLPSVSSAPITSGQAPTTFLPASTSQAQLSSQKMESFSAVPPTKEKVSTQDQPMAN

LCTPSSTANSCSSSASNTPGAPETHPSSSPTPTSSNTQEEAQPSSVSDLSPMSMPFASNS

EPAPLTLTSPRMVAADNQDTSNLPQLAVPAPRVSHRMQPRGSFYSMVPNATIHQDPQSIF

VTNPVTLTPPQGPPAAVQLSSAVNIMNGSQMHINPANKSLPPTFGPATLFNHFSSLFDSS

QVPANQGWGDGPLSSRVATDASFTVQSAFLGNSVLGHLENMHPDNSKAPGFRPPSQRVST

SPVGLPSIDPSGSSPSSSSAPLASFSGIPGTRVFLQGPAPVGTPSFNRQHFSPHPWTSAS

NSSTSAPPTLGQPKGVSASQDRKIPPPIGTERLARIRQGGSVAQAPAGTSFVAPVGHSGI

WSFGVNAVSEGLSGWSQSVMGNHPMHQQLSDPSTFSQHQPMERDDSGMVAPSNIFHQPMA

SGFVDFSKGLPISMYGGTIIPSHPQLADVPGGPLFNGLHNPDPAWNPMIKVIQNSTECTD

AQQIWPGTWAPHIGNMHLKYVN

>sp|Q9Y490|TLN1_HUMAN 777 ALNELLQHV

MVALSLKISIGNVVKTMQFEPSTMVYDACRIIRERIPEAPAGPPSDFGLFLSDDDPKKGI

WLEAGKALDYYMLRNGDTMEYRKKQRPLKIRMLDGTVKTIMVDDSKTVTDMLMTICARIG

ITNHDEYSLVRELMEEKKEEITGTLRKDKTLLRDEKKMEKLKQKLHTDDELNWLDHGRTL

REQGVEEHETLLLRRKFFYSDQNVDSRDPVQLNLLYVQARDDILNGSHPVSFDKACEFAG

FQCQIQFGPHNEQKHKAGFLDLKDFLPKEYVKQKGERKIFQAHKNCGQMSEIEAKVRYVK

LARSLKTYGVSFFLVKEKMKGKNKLVPRLLGITKECVMRVDEKTKEVIQEWNLTNIKRWA

ASPKSFTLDFGDYQDGYYSVQTTEGEQIAQLIAGYIDIILKKKKSKDHFGLEGDEESTML

EDSVSPKKSTVLQQQYNRVGKVEHGSVALPAIMRSGASGPENFQVGSMPPAQQQITSGQM

HRGHMPPLTSAQQALTGTINSSMQAVQAAQATLDDFDTLPPLGQDAASKAWRKNKMDESK

HEIHSQVDAITAGTASVVNLTAGDPAETDYTAVGCAVTTISSNLTEMSRGVKLLAALLED

EGGSGRPLLQAAKGLAGAVSELLRSAQPASAEPRQNLLQAAGNVGQASGELLQQIGESDT

DPHFQDALMQLAKAVASAAAALVLKAKSVAQRTEDSGLQTQVIAAATQCALSTSQLVACT

KVVAPTISSPVCQEQLVEAGRLVAKAVEGCVSASQAATEDGQLLRGVGAAATAVTQALNE

LLQHVKAHATGAGPAGRYDQATDTILTVTENIFSSMGDAGEMVRQARILAQATSDLVNAI

KADAEGESDLENSRKLLSAAKILADATAKMVEAAKGAAAHPDSEEQQQRLREAAEGLRMA

TNAAAQNAIKKKLVQRLEHAAKQAAASATQTIAAAQHAASTPKASAGPQPLLVQSCKAVA

EQIPLLVQGVRGSQAQPDSPSAQLALIAASQSFLQPGGKMVAAAKASVPTIQDQASAMQL

SQCAKNLGTALAELRTAAQKAQEACGPLEMDSALSVVQNLEKDLQEVKAAARDGKLKPLP

GETMEKCTQDLGNSTKAVSSAIAQLLGEVAQGNENYAGIAARDVAGGLRSLAQAARGVAA

LTSDPAVQAIVLDTASDVLDKASSLIEEAKKAAGHPGDPESQQRLAQVAKAVTQALNRCV

SCLPGQRDVDNALRAVGDASKRLLSDSLPPSTGTFQEAQSRLNEAAAGLNQAATELVQAS

RGTPQDLARASGRFGQDFSTFLEAGVEMAGQAPSQEDRAQVVSNLKGISMSSSKLLLAAK

ALSTDPAAPNLKSQLAAAARAVTDSINQLITMCTQQAPGQKECDNALRELETVRELLENP

VQPINDMSYFGCLDSVMENSKVLGEAMTGISQNAKNGNLPEFGDAISTASKALCGFTEAA

AQAAYLVGVSDPNSQAGQQGLVEPTQFARANQAIQMACQSLGEPGCTQAQVLSAATIVAK

HTSALCNSCRLASARTTNPTAKRQFVQSAKEVANSTANLVKTIKALDGAFTEENRAQCRA

ATAPLLEAVDNLSAFASNPEFSSIPAQISPEGRAAMEPIVISAKTMLESAGGLIQTARAL

AVNPRDPPSWSVLAGHSRTVSDSIKKLITSMRDKAPGQLECETAIAALNSCLRDLDQASL

AAVSQQLAPREGISQEALHTQMLTAVQEISHLIEPLANAARAEASQLGHKVSQMAQYFEP

LTLAAVGAASKTLSHPQQMALLDQTKTLAESALQLLYTAKEAGGNPKQAAHTQEALEEAV

QMMTEAVEDLTTTLNEAASAAGVVGGMVDSITQAINQLDEGPMGEPEGSFVDYQTTMVRT

AKAIAVTVQEMVTKSNTSPEELGPLANQLTSDYGRLASEAKPAAVAAENEEIGSHIKHRV

QELGHGCAALVTKAGALQCSPSDAYTKKELIECARRVSEKVSHVLAALQAGNRGTQACIT

AASAVSGIIADLDTTIMFATAGTLNREGTETFADHREGILKTAKVLVEDTKVLVQNAAGS

QEKLAQAAQSSVATITRLADVVKLGAASLGAEDPETQVVLINAVKDVAKALGDLISATKA

AAGKVGDDPAVWQLKNSAKVMVTNVTSLLKTVKAVEDEATKGTRALEATTEHIRQELAVF

CSPEPPAKTSTPEDFIRMTKGITMATAKAVAAGNSCRQEDVIATANLSRRAIADMLRACK

EAAYHPEVAPDVRLRALHYGRECANGYLELLDHVLLTLQKPSPELKQQLTGHSKRVAGSV

TELIQAAEAMKGTEWVDPEDPTVIAENELLGAAAAIEAAAKKLEQLKPRAKPKEADESLN

FEEQILEAAKSIAAATSALVKAASAAQRELVAQGKVGAIPANALDDGQWSQGLISAARMV

AAATNNLCEAANAAVQGHASQEKLISSAKQVAASTAQLLVACKVKADQDSEAMKRLQAAG

NAVKRASDNLVKAAQKAAAFEEQENETVVVKEKMVGGIAQIIAAQEEMLRKERELEEARK

KLAQIRQQQYKFLPSELRDEH

>sp|Q9Y490|TLN1_HUMAN 125 DEYSLVREL

MVALSLKISIGNVVKTMQFEPSTMVYDACRIIRERIPEAPAGPPSDFGLFLSDDDPKKGI

WLEAGKALDYYMLRNGDTMEYRKKQRPLKIRMLDGTVKTIMVDDSKTVTDMLMTICARIG

ITNHDEYSLVRELMEEKKEEITGTLRKDKTLLRDEKKMEKLKQKLHTDDELNWLDHGRTL

REQGVEEHETLLLRRKFFYSDQNVDSRDPVQLNLLYVQARDDILNGSHPVSFDKACEFAG

FQCQIQFGPHNEQKHKAGFLDLKDFLPKEYVKQKGERKIFQAHKNCGQMSEIEAKVRYVK

LARSLKTYGVSFFLVKEKMKGKNKLVPRLLGITKECVMRVDEKTKEVIQEWNLTNIKRWA

ASPKSFTLDFGDYQDGYYSVQTTEGEQIAQLIAGYIDIILKKKKSKDHFGLEGDEESTML

EDSVSPKKSTVLQQQYNRVGKVEHGSVALPAIMRSGASGPENFQVGSMPPAQQQITSGQM

HRGHMPPLTSAQQALTGTINSSMQAVQAAQATLDDFDTLPPLGQDAASKAWRKNKMDESK

HEIHSQVDAITAGTASVVNLTAGDPAETDYTAVGCAVTTISSNLTEMSRGVKLLAALLED

EGGSGRPLLQAAKGLAGAVSELLRSAQPASAEPRQNLLQAAGNVGQASGELLQQIGESDT

DPHFQDALMQLAKAVASAAAALVLKAKSVAQRTEDSGLQTQVIAAATQCALSTSQLVACT

KVVAPTISSPVCQEQLVEAGRLVAKAVEGCVSASQAATEDGQLLRGVGAAATAVTQALNE

LLQHVKAHATGAGPAGRYDQATDTILTVTENIFSSMGDAGEMVRQARILAQATSDLVNAI

KADAEGESDLENSRKLLSAAKILADATAKMVEAAKGAAAHPDSEEQQQRLREAAEGLRMA

TNAAAQNAIKKKLVQRLEHAAKQAAASATQTIAAAQHAASTPKASAGPQPLLVQSCKAVA

EQIPLLVQGVRGSQAQPDSPSAQLALIAASQSFLQPGGKMVAAAKASVPTIQDQASAMQL

SQCAKNLGTALAELRTAAQKAQEACGPLEMDSALSVVQNLEKDLQEVKAAARDGKLKPLP

GETMEKCTQDLGNSTKAVSSAIAQLLGEVAQGNENYAGIAARDVAGGLRSLAQAARGVAA

LTSDPAVQAIVLDTASDVLDKASSLIEEAKKAAGHPGDPESQQRLAQVAKAVTQALNRCV

SCLPGQRDVDNALRAVGDASKRLLSDSLPPSTGTFQEAQSRLNEAAAGLNQAATELVQAS

RGTPQDLARASGRFGQDFSTFLEAGVEMAGQAPSQEDRAQVVSNLKGISMSSSKLLLAAK

ALSTDPAAPNLKSQLAAAARAVTDSINQLITMCTQQAPGQKECDNALRELETVRELLENP

VQPINDMSYFGCLDSVMENSKVLGEAMTGISQNAKNGNLPEFGDAISTASKALCGFTEAA

AQAAYLVGVSDPNSQAGQQGLVEPTQFARANQAIQMACQSLGEPGCTQAQVLSAATIVAK

HTSALCNSCRLASARTTNPTAKRQFVQSAKEVANSTANLVKTIKALDGAFTEENRAQCRA

ATAPLLEAVDNLSAFASNPEFSSIPAQISPEGRAAMEPIVISAKTMLESAGGLIQTARAL

AVNPRDPPSWSVLAGHSRTVSDSIKKLITSMRDKAPGQLECETAIAALNSCLRDLDQASL

AAVSQQLAPREGISQEALHTQMLTAVQEISHLIEPLANAARAEASQLGHKVSQMAQYFEP

LTLAAVGAASKTLSHPQQMALLDQTKTLAESALQLLYTAKEAGGNPKQAAHTQEALEEAV

QMMTEAVEDLTTTLNEAASAAGVVGGMVDSITQAINQLDEGPMGEPEGSFVDYQTTMVRT

AKAIAVTVQEMVTKSNTSPEELGPLANQLTSDYGRLASEAKPAAVAAENEEIGSHIKHRV

QELGHGCAALVTKAGALQCSPSDAYTKKELIECARRVSEKVSHVLAALQAGNRGTQACIT

AASAVSGIIADLDTTIMFATAGTLNREGTETFADHREGILKTAKVLVEDTKVLVQNAAGS

QEKLAQAAQSSVATITRLADVVKLGAASLGAEDPETQVVLINAVKDVAKALGDLISATKA

AAGKVGDDPAVWQLKNSAKVMVTNVTSLLKTVKAVEDEATKGTRALEATTEHIRQELAVF

CSPEPPAKTSTPEDFIRMTKGITMATAKAVAAGNSCRQEDVIATANLSRRAIADMLRACK

EAAYHPEVAPDVRLRALHYGRECANGYLELLDHVLLTLQKPSPELKQQLTGHSKRVAGSV

TELIQAAEAMKGTEWVDPEDPTVIAENELLGAAAAIEAAAKKLEQLKPRAKPKEADESLN

FEEQILEAAKSIAAATSALVKAASAAQRELVAQGKVGAIPANALDDGQWSQGLISAARMV

AAATNNLCEAANAAVQGHASQEKLISSAKQVAASTAQLLVACKVKADQDSEAMKRLQAAG

NAVKRASDNLVKAAQKAAAFEEQENETVVVKEKMVGGIAQIIAAQEEMLRKERELEEARK

KLAQIRQQQYKFLPSELRDEH

>sp|Q9Y490|TLN1_HUMAN 343 KTKEVIQEW

MVALSLKISIGNVVKTMQFEPSTMVYDACRIIRERIPEAPAGPPSDFGLFLSDDDPKKGI

WLEAGKALDYYMLRNGDTMEYRKKQRPLKIRMLDGTVKTIMVDDSKTVTDMLMTICARIG

ITNHDEYSLVRELMEEKKEEITGTLRKDKTLLRDEKKMEKLKQKLHTDDELNWLDHGRTL

REQGVEEHETLLLRRKFFYSDQNVDSRDPVQLNLLYVQARDDILNGSHPVSFDKACEFAG

FQCQIQFGPHNEQKHKAGFLDLKDFLPKEYVKQKGERKIFQAHKNCGQMSEIEAKVRYVK

LARSLKTYGVSFFLVKEKMKGKNKLVPRLLGITKECVMRVDEKTKEVIQEWNLTNIKRWA

ASPKSFTLDFGDYQDGYYSVQTTEGEQIAQLIAGYIDIILKKKKSKDHFGLEGDEESTML

EDSVSPKKSTVLQQQYNRVGKVEHGSVALPAIMRSGASGPENFQVGSMPPAQQQITSGQM

HRGHMPPLTSAQQALTGTINSSMQAVQAAQATLDDFDTLPPLGQDAASKAWRKNKMDESK

HEIHSQVDAITAGTASVVNLTAGDPAETDYTAVGCAVTTISSNLTEMSRGVKLLAALLED

EGGSGRPLLQAAKGLAGAVSELLRSAQPASAEPRQNLLQAAGNVGQASGELLQQIGESDT

DPHFQDALMQLAKAVASAAAALVLKAKSVAQRTEDSGLQTQVIAAATQCALSTSQLVACT

KVVAPTISSPVCQEQLVEAGRLVAKAVEGCVSASQAATEDGQLLRGVGAAATAVTQALNE

LLQHVKAHATGAGPAGRYDQATDTILTVTENIFSSMGDAGEMVRQARILAQATSDLVNAI

KADAEGESDLENSRKLLSAAKILADATAKMVEAAKGAAAHPDSEEQQQRLREAAEGLRMA

TNAAAQNAIKKKLVQRLEHAAKQAAASATQTIAAAQHAASTPKASAGPQPLLVQSCKAVA

EQIPLLVQGVRGSQAQPDSPSAQLALIAASQSFLQPGGKMVAAAKASVPTIQDQASAMQL

SQCAKNLGTALAELRTAAQKAQEACGPLEMDSALSVVQNLEKDLQEVKAAARDGKLKPLP

GETMEKCTQDLGNSTKAVSSAIAQLLGEVAQGNENYAGIAARDVAGGLRSLAQAARGVAA

LTSDPAVQAIVLDTASDVLDKASSLIEEAKKAAGHPGDPESQQRLAQVAKAVTQALNRCV

SCLPGQRDVDNALRAVGDASKRLLSDSLPPSTGTFQEAQSRLNEAAAGLNQAATELVQAS

RGTPQDLARASGRFGQDFSTFLEAGVEMAGQAPSQEDRAQVVSNLKGISMSSSKLLLAAK

ALSTDPAAPNLKSQLAAAARAVTDSINQLITMCTQQAPGQKECDNALRELETVRELLENP

VQPINDMSYFGCLDSVMENSKVLGEAMTGISQNAKNGNLPEFGDAISTASKALCGFTEAA

AQAAYLVGVSDPNSQAGQQGLVEPTQFARANQAIQMACQSLGEPGCTQAQVLSAATIVAK

HTSALCNSCRLASARTTNPTAKRQFVQSAKEVANSTANLVKTIKALDGAFTEENRAQCRA

ATAPLLEAVDNLSAFASNPEFSSIPAQISPEGRAAMEPIVISAKTMLESAGGLIQTARAL

AVNPRDPPSWSVLAGHSRTVSDSIKKLITSMRDKAPGQLECETAIAALNSCLRDLDQASL

AAVSQQLAPREGISQEALHTQMLTAVQEISHLIEPLANAARAEASQLGHKVSQMAQYFEP

LTLAAVGAASKTLSHPQQMALLDQTKTLAESALQLLYTAKEAGGNPKQAAHTQEALEEAV

QMMTEAVEDLTTTLNEAASAAGVVGGMVDSITQAINQLDEGPMGEPEGSFVDYQTTMVRT

AKAIAVTVQEMVTKSNTSPEELGPLANQLTSDYGRLASEAKPAAVAAENEEIGSHIKHRV

QELGHGCAALVTKAGALQCSPSDAYTKKELIECARRVSEKVSHVLAALQAGNRGTQACIT

AASAVSGIIADLDTTIMFATAGTLNREGTETFADHREGILKTAKVLVEDTKVLVQNAAGS

QEKLAQAAQSSVATITRLADVVKLGAASLGAEDPETQVVLINAVKDVAKALGDLISATKA

AAGKVGDDPAVWQLKNSAKVMVTNVTSLLKTVKAVEDEATKGTRALEATTEHIRQELAVF

CSPEPPAKTSTPEDFIRMTKGITMATAKAVAAGNSCRQEDVIATANLSRRAIADMLRACK

EAAYHPEVAPDVRLRALHYGRECANGYLELLDHVLLTLQKPSPELKQQLTGHSKRVAGSV

TELIQAAEAMKGTEWVDPEDPTVIAENELLGAAAAIEAAAKKLEQLKPRAKPKEADESLN

FEEQILEAAKSIAAATSALVKAASAAQRELVAQGKVGAIPANALDDGQWSQGLISAARMV

AAATNNLCEAANAAVQGHASQEKLISSAKQVAASTAQLLVACKVKADQDSEAMKRLQAAG

NAVKRASDNLVKAAQKAAAFEEQENETVVVKEKMVGGIAQIIAAQEEMLRKERELEEARK

KLAQIRQQQYKFLPSELRDEH

>sp|Q9Y490|TLN1_HUMAN 290 SEIEAKVRY

MVALSLKISIGNVVKTMQFEPSTMVYDACRIIRERIPEAPAGPPSDFGLFLSDDDPKKGI

WLEAGKALDYYMLRNGDTMEYRKKQRPLKIRMLDGTVKTIMVDDSKTVTDMLMTICARIG

ITNHDEYSLVRELMEEKKEEITGTLRKDKTLLRDEKKMEKLKQKLHTDDELNWLDHGRTL

REQGVEEHETLLLRRKFFYSDQNVDSRDPVQLNLLYVQARDDILNGSHPVSFDKACEFAG

FQCQIQFGPHNEQKHKAGFLDLKDFLPKEYVKQKGERKIFQAHKNCGQMSEIEAKVRYVK

LARSLKTYGVSFFLVKEKMKGKNKLVPRLLGITKECVMRVDEKTKEVIQEWNLTNIKRWA

ASPKSFTLDFGDYQDGYYSVQTTEGEQIAQLIAGYIDIILKKKKSKDHFGLEGDEESTML

EDSVSPKKSTVLQQQYNRVGKVEHGSVALPAIMRSGASGPENFQVGSMPPAQQQITSGQM

HRGHMPPLTSAQQALTGTINSSMQAVQAAQATLDDFDTLPPLGQDAASKAWRKNKMDESK

HEIHSQVDAITAGTASVVNLTAGDPAETDYTAVGCAVTTISSNLTEMSRGVKLLAALLED

EGGSGRPLLQAAKGLAGAVSELLRSAQPASAEPRQNLLQAAGNVGQASGELLQQIGESDT

DPHFQDALMQLAKAVASAAAALVLKAKSVAQRTEDSGLQTQVIAAATQCALSTSQLVACT

KVVAPTISSPVCQEQLVEAGRLVAKAVEGCVSASQAATEDGQLLRGVGAAATAVTQALNE

LLQHVKAHATGAGPAGRYDQATDTILTVTENIFSSMGDAGEMVRQARILAQATSDLVNAI

KADAEGESDLENSRKLLSAAKILADATAKMVEAAKGAAAHPDSEEQQQRLREAAEGLRMA

TNAAAQNAIKKKLVQRLEHAAKQAAASATQTIAAAQHAASTPKASAGPQPLLVQSCKAVA

EQIPLLVQGVRGSQAQPDSPSAQLALIAASQSFLQPGGKMVAAAKASVPTIQDQASAMQL

SQCAKNLGTALAELRTAAQKAQEACGPLEMDSALSVVQNLEKDLQEVKAAARDGKLKPLP

GETMEKCTQDLGNSTKAVSSAIAQLLGEVAQGNENYAGIAARDVAGGLRSLAQAARGVAA

LTSDPAVQAIVLDTASDVLDKASSLIEEAKKAAGHPGDPESQQRLAQVAKAVTQALNRCV

SCLPGQRDVDNALRAVGDASKRLLSDSLPPSTGTFQEAQSRLNEAAAGLNQAATELVQAS

RGTPQDLARASGRFGQDFSTFLEAGVEMAGQAPSQEDRAQVVSNLKGISMSSSKLLLAAK

ALSTDPAAPNLKSQLAAAARAVTDSINQLITMCTQQAPGQKECDNALRELETVRELLENP

VQPINDMSYFGCLDSVMENSKVLGEAMTGISQNAKNGNLPEFGDAISTASKALCGFTEAA

AQAAYLVGVSDPNSQAGQQGLVEPTQFARANQAIQMACQSLGEPGCTQAQVLSAATIVAK

HTSALCNSCRLASARTTNPTAKRQFVQSAKEVANSTANLVKTIKALDGAFTEENRAQCRA

ATAPLLEAVDNLSAFASNPEFSSIPAQISPEGRAAMEPIVISAKTMLESAGGLIQTARAL

AVNPRDPPSWSVLAGHSRTVSDSIKKLITSMRDKAPGQLECETAIAALNSCLRDLDQASL

AAVSQQLAPREGISQEALHTQMLTAVQEISHLIEPLANAARAEASQLGHKVSQMAQYFEP

LTLAAVGAASKTLSHPQQMALLDQTKTLAESALQLLYTAKEAGGNPKQAAHTQEALEEAV

QMMTEAVEDLTTTLNEAASAAGVVGGMVDSITQAINQLDEGPMGEPEGSFVDYQTTMVRT

AKAIAVTVQEMVTKSNTSPEELGPLANQLTSDYGRLASEAKPAAVAAENEEIGSHIKHRV

QELGHGCAALVTKAGALQCSPSDAYTKKELIECARRVSEKVSHVLAALQAGNRGTQACIT

AASAVSGIIADLDTTIMFATAGTLNREGTETFADHREGILKTAKVLVEDTKVLVQNAAGS

QEKLAQAAQSSVATITRLADVVKLGAASLGAEDPETQVVLINAVKDVAKALGDLISATKA

AAGKVGDDPAVWQLKNSAKVMVTNVTSLLKTVKAVEDEATKGTRALEATTEHIRQELAVF

CSPEPPAKTSTPEDFIRMTKGITMATAKAVAAGNSCRQEDVIATANLSRRAIADMLRACK

EAAYHPEVAPDVRLRALHYGRECANGYLELLDHVLLTLQKPSPELKQQLTGHSKRVAGSV

TELIQAAEAMKGTEWVDPEDPTVIAENELLGAAAAIEAAAKKLEQLKPRAKPKEADESLN

FEEQILEAAKSIAAATSALVKAASAAQRELVAQGKVGAIPANALDDGQWSQGLISAARMV

AAATNNLCEAANAAVQGHASQEKLISSAKQVAASTAQLLVACKVKADQDSEAMKRLQAAG

NAVKRASDNLVKAAQKAAAFEEQENETVVVKEKMVGGIAQIIAAQEEMLRKERELEEARK

KLAQIRQQQYKFLPSELRDEH

>sp|P49327|FAS_HUMAN 2335 FLFDGSPTYV

MEEVVIAGMSGKLPESENLQEFWDNLIGGVDMVTDDDRRWKAGLYGLPRRSGKLKDLSRF

DASFFGVHPKQAHTMDPQLRLLLEVTYEAIVDGGINPDSLRGTHTGVWVGVSGSETSEAL

SRDPETLVGYSMVGCQRAMMANRLSFFFDFRGPSIALDTACSSSLMALQNAYQAIHSGQC

PAAIVGGINVLLKPNTSVQFLRLGMLSPEGTCKAFDTAGNGYCRSEGVVAVLLTKKSLAR

RVYATILNAGTNTDGFKEQGVTFPSGDIQEQLIRSLYQSAGVAPESFEYIEAHGTGTKVG

DPQELNGITRALCATRQEPLLIGSTKSNMGHPEPASGLAALAKVLLSLEHGLWAPNLHFH

SPNPEIPALLDGRLQVVDQPLPVRGGNVGINSFGFGGSNVHIILRPNTQPPPAPAPHATL

PRLLRASGRTPEAVQKLLEQGLRHSQDLAFLSMLNDIAAVPATAMPFRGYAVLGGERGGP

EVQQVPAGERPLWFICSGMGTQWRGMGLSLMRLDRFRDSILRSDEAVKPFGLKVSQLLLS

TDESTFDDIVHSFVSLTAIQIGLIDLLSCMGLRPDGIVGHSLGEVACGYADGCLSQEEAV

LAAYWRGQCIKEAHLPPGAMAAVGLSWEECKQRCPPGVVPACHNSKDTVTISGPQAPVFE

FVEQLRKEGVFAKEVRTGGMAFHSYFMEAIAPPLLQELKKVIREPKPRSARWLSTSIPEA

QWHSSLARTSSAEYNVNNLVSPVLFQEALWHVPEHAVVLEIAPHALLQAVLKRGLKPSCT

IIPLMKKDHRDNLEFFLAGIGRLHLSGIDANPNALFPPVEFPAPRGTPLISPLIKWDHSL

AWDVPAAEDFPNGSGSPSAAIYNIDTSSESPDHYLVDHTLDGRVLFPATGYLSIVWKTLA

RALGLGVEQLPVVFEDVVLHQATILPKTGTVSLEVRLLEASRAFEVSENGNLVVSGKVYQ

WDDPDPRLFDHPESPTPNPTEPLFLAQAEVYKELRLRGYDYGPHFQGILEASLEGDSGRL

LWKDNWVSFMDTMLQMSILGSAKHGLYLPTRVTAIHIDPATHRQKLYTLQDKAQVADVVV

SRWLRVTVAGGVHISGLHTESAPRRQQEQQVPILEKFCFTPHTEEGCLSERAALQEELQL

CKGLVQALQTTVTQQGLKMVVPGLDGAQIPRDPSQQELPRLLSAACRLQLNGNLQLELAQ

VLAQERPKLPEDPLLSGLLDSPALKACLDTAVENMPSLKMKVVEVLAGHGHLYSRIPGLL

SPHPLLQLSYTATDRHPQALEAAQAELQQHDVAQGQWDPADPAPSALGSADLLVCNCAVA

ALGDPASALSNMVAALREGGFLLLHTLLRGHPLGDIVAFLTSTEPQYGQGILSQDAWESL

FSRVSLRLVGLKKSFYGSTLFLCRRPTPQDSPIFLPVDDTSFRWVESLKGILADEDSSRP

VWLKAINCATSGVVGLVNCLRREPGGNRLRCVLLSNLSSTSHVPEVDPGSAELQKVLQGD

LVMNVYRDGAWGAFRHFLLEEDKPEEPTAHAFVSTLTRGDLSSIRWVCSSLRHAQPTCPG

AQLCTVYYASLNFRDIMLATGKLSPDAIPGKWTSQDSLLGMEFSGRDASGKRVMGLVPAK

GLATSVLLSPDFLWDVPSNWTLEEAASVPVVYSTAYYALVVRGRVRPGETLLIHSGSGGV

GQAAIAIALSLGCRVFTTVGSAEKRAYLQARFPQLDSTSFANSRDTSFEQHVLWHTGGKG

VDLVLNSLAEEKLQASVRCLATHGRFLEIGKFDLSQNHPLGMAIFLKNVTFHGVLLDAFF

NESSADWREVWALVQAGIRDGVVRPLKCTVFHGAQVEDAFRYMAQGKHIGKVVVQVLAEE

PEAVLKGAKPKLMSAISKTFCPAHKSYIIAGGLGGFGLELAQWLIQRGVQKLVLTSRSGI

RTGYQAKQVRRWRRQGVQVQVSTSNISSLEGARGLIAEAAQLGPVGGVFNLAVVLRDGLL

ENQTPEFFQDVCKPKYSGTLNLDRVTREACPELDYFVVFSSVSCGRGNAGQSNYGFANSA

MERICEKRRHEGLPGLAVQWGAIGDVGILVETMSTNDTIVSGTLPQRMASCLEVLDLFLN

QPHMVLSSFVLAEKAAAYRDRDSQRDLVEAVAHILGIRDLAAVNLDSSLADLGLDSLMSV

EVRQTLERELNLVLSVREVRQLTLRKLQELSSKADEASELACPTPKEDGLAQQQTQLNLR

SLLVNPEGPTLMRLNSVQSSERPLFLVHPIEGSTTVFHSLASRLSIPTYGLQCTRAAPLD

SIHSLAAYYIDCIRQVQPEGPYRVAGYSYGACVAFEMCSQLQAQQSPAPTHNSLFLFDGS

PTYVLAYTQSYRAKLTPGCEAEAETEAICFFVQQFTDMEHNRVLEALLPLKGLEERVAAA

VDLIIKSHQGLDRQELSFAARSFYYKLRAAEQYTPKAKYHGNVMLLRAKTGGAYGEDLGA

DYNLSQVCDGKVSVHVIEGDHRTLLEGSGLESIISIIHSSLAEPRVSVREG

>sp|P49327|FAS_HUMAN 2335 FLFDGSPTYVL

MEEVVIAGMSGKLPESENLQEFWDNLIGGVDMVTDDDRRWKAGLYGLPRRSGKLKDLSRF

DASFFGVHPKQAHTMDPQLRLLLEVTYEAIVDGGINPDSLRGTHTGVWVGVSGSETSEAL

SRDPETLVGYSMVGCQRAMMANRLSFFFDFRGPSIALDTACSSSLMALQNAYQAIHSGQC

PAAIVGGINVLLKPNTSVQFLRLGMLSPEGTCKAFDTAGNGYCRSEGVVAVLLTKKSLAR

RVYATILNAGTNTDGFKEQGVTFPSGDIQEQLIRSLYQSAGVAPESFEYIEAHGTGTKVG

DPQELNGITRALCATRQEPLLIGSTKSNMGHPEPASGLAALAKVLLSLEHGLWAPNLHFH

SPNPEIPALLDGRLQVVDQPLPVRGGNVGINSFGFGGSNVHIILRPNTQPPPAPAPHATL

PRLLRASGRTPEAVQKLLEQGLRHSQDLAFLSMLNDIAAVPATAMPFRGYAVLGGERGGP

EVQQVPAGERPLWFICSGMGTQWRGMGLSLMRLDRFRDSILRSDEAVKPFGLKVSQLLLS

TDESTFDDIVHSFVSLTAIQIGLIDLLSCMGLRPDGIVGHSLGEVACGYADGCLSQEEAV

LAAYWRGQCIKEAHLPPGAMAAVGLSWEECKQRCPPGVVPACHNSKDTVTISGPQAPVFE

FVEQLRKEGVFAKEVRTGGMAFHSYFMEAIAPPLLQELKKVIREPKPRSARWLSTSIPEA

QWHSSLARTSSAEYNVNNLVSPVLFQEALWHVPEHAVVLEIAPHALLQAVLKRGLKPSCT

IIPLMKKDHRDNLEFFLAGIGRLHLSGIDANPNALFPPVEFPAPRGTPLISPLIKWDHSL

AWDVPAAEDFPNGSGSPSAAIYNIDTSSESPDHYLVDHTLDGRVLFPATGYLSIVWKTLA

RALGLGVEQLPVVFEDVVLHQATILPKTGTVSLEVRLLEASRAFEVSENGNLVVSGKVYQ

WDDPDPRLFDHPESPTPNPTEPLFLAQAEVYKELRLRGYDYGPHFQGILEASLEGDSGRL

LWKDNWVSFMDTMLQMSILGSAKHGLYLPTRVTAIHIDPATHRQKLYTLQDKAQVADVVV

SRWLRVTVAGGVHISGLHTESAPRRQQEQQVPILEKFCFTPHTEEGCLSERAALQEELQL

CKGLVQALQTTVTQQGLKMVVPGLDGAQIPRDPSQQELPRLLSAACRLQLNGNLQLELAQ

VLAQERPKLPEDPLLSGLLDSPALKACLDTAVENMPSLKMKVVEVLAGHGHLYSRIPGLL

SPHPLLQLSYTATDRHPQALEAAQAELQQHDVAQGQWDPADPAPSALGSADLLVCNCAVA

ALGDPASALSNMVAALREGGFLLLHTLLRGHPLGDIVAFLTSTEPQYGQGILSQDAWESL

FSRVSLRLVGLKKSFYGSTLFLCRRPTPQDSPIFLPVDDTSFRWVESLKGILADEDSSRP

VWLKAINCATSGVVGLVNCLRREPGGNRLRCVLLSNLSSTSHVPEVDPGSAELQKVLQGD

LVMNVYRDGAWGAFRHFLLEEDKPEEPTAHAFVSTLTRGDLSSIRWVCSSLRHAQPTCPG

AQLCTVYYASLNFRDIMLATGKLSPDAIPGKWTSQDSLLGMEFSGRDASGKRVMGLVPAK

GLATSVLLSPDFLWDVPSNWTLEEAASVPVVYSTAYYALVVRGRVRPGETLLIHSGSGGV

GQAAIAIALSLGCRVFTTVGSAEKRAYLQARFPQLDSTSFANSRDTSFEQHVLWHTGGKG

VDLVLNSLAEEKLQASVRCLATHGRFLEIGKFDLSQNHPLGMAIFLKNVTFHGVLLDAFF

NESSADWREVWALVQAGIRDGVVRPLKCTVFHGAQVEDAFRYMAQGKHIGKVVVQVLAEE

PEAVLKGAKPKLMSAISKTFCPAHKSYIIAGGLGGFGLELAQWLIQRGVQKLVLTSRSGI

RTGYQAKQVRRWRRQGVQVQVSTSNISSLEGARGLIAEAAQLGPVGGVFNLAVVLRDGLL

ENQTPEFFQDVCKPKYSGTLNLDRVTREACPELDYFVVFSSVSCGRGNAGQSNYGFANSA

MERICEKRRHEGLPGLAVQWGAIGDVGILVETMSTNDTIVSGTLPQRMASCLEVLDLFLN

QPHMVLSSFVLAEKAAAYRDRDSQRDLVEAVAHILGIRDLAAVNLDSSLADLGLDSLMSV

EVRQTLERELNLVLSVREVRQLTLRKLQELSSKADEASELACPTPKEDGLAQQQTQLNLR

SLLVNPEGPTLMRLNSVQSSERPLFLVHPIEGSTTVFHSLASRLSIPTYGLQCTRAAPLD

SIHSLAAYYIDCIRQVQPEGPYRVAGYSYGACVAFEMCSQLQAQQSPAPTHNSLFLFDGS

PTYVLAYTQSYRAKLTPGCEAEAETEAICFFVQQFTDMEHNRVLEALLPLKGLEERVAAA

VDLIIKSHQGLDRQELSFAARSFYYKLRAAEQYTPKAKYHGNVMLLRAKTGGAYGEDLGA

DYNLSQVCDGKVSVHVIEGDHRTLLEGSGLESIISIIHSSLAEPRVSVREG

>sp|P49327|FAS_HUMAN 1663 GRVRPGETL

MEEVVIAGMSGKLPESENLQEFWDNLIGGVDMVTDDDRRWKAGLYGLPRRSGKLKDLSRF

DASFFGVHPKQAHTMDPQLRLLLEVTYEAIVDGGINPDSLRGTHTGVWVGVSGSETSEAL

SRDPETLVGYSMVGCQRAMMANRLSFFFDFRGPSIALDTACSSSLMALQNAYQAIHSGQC

PAAIVGGINVLLKPNTSVQFLRLGMLSPEGTCKAFDTAGNGYCRSEGVVAVLLTKKSLAR

RVYATILNAGTNTDGFKEQGVTFPSGDIQEQLIRSLYQSAGVAPESFEYIEAHGTGTKVG

DPQELNGITRALCATRQEPLLIGSTKSNMGHPEPASGLAALAKVLLSLEHGLWAPNLHFH

SPNPEIPALLDGRLQVVDQPLPVRGGNVGINSFGFGGSNVHIILRPNTQPPPAPAPHATL

PRLLRASGRTPEAVQKLLEQGLRHSQDLAFLSMLNDIAAVPATAMPFRGYAVLGGERGGP

EVQQVPAGERPLWFICSGMGTQWRGMGLSLMRLDRFRDSILRSDEAVKPFGLKVSQLLLS

TDESTFDDIVHSFVSLTAIQIGLIDLLSCMGLRPDGIVGHSLGEVACGYADGCLSQEEAV

LAAYWRGQCIKEAHLPPGAMAAVGLSWEECKQRCPPGVVPACHNSKDTVTISGPQAPVFE

FVEQLRKEGVFAKEVRTGGMAFHSYFMEAIAPPLLQELKKVIREPKPRSARWLSTSIPEA

QWHSSLARTSSAEYNVNNLVSPVLFQEALWHVPEHAVVLEIAPHALLQAVLKRGLKPSCT

IIPLMKKDHRDNLEFFLAGIGRLHLSGIDANPNALFPPVEFPAPRGTPLISPLIKWDHSL

AWDVPAAEDFPNGSGSPSAAIYNIDTSSESPDHYLVDHTLDGRVLFPATGYLSIVWKTLA

RALGLGVEQLPVVFEDVVLHQATILPKTGTVSLEVRLLEASRAFEVSENGNLVVSGKVYQ

WDDPDPRLFDHPESPTPNPTEPLFLAQAEVYKELRLRGYDYGPHFQGILEASLEGDSGRL

LWKDNWVSFMDTMLQMSILGSAKHGLYLPTRVTAIHIDPATHRQKLYTLQDKAQVADVVV

SRWLRVTVAGGVHISGLHTESAPRRQQEQQVPILEKFCFTPHTEEGCLSERAALQEELQL

CKGLVQALQTTVTQQGLKMVVPGLDGAQIPRDPSQQELPRLLSAACRLQLNGNLQLELAQ

VLAQERPKLPEDPLLSGLLDSPALKACLDTAVENMPSLKMKVVEVLAGHGHLYSRIPGLL

SPHPLLQLSYTATDRHPQALEAAQAELQQHDVAQGQWDPADPAPSALGSADLLVCNCAVA

ALGDPASALSNMVAALREGGFLLLHTLLRGHPLGDIVAFLTSTEPQYGQGILSQDAWESL

FSRVSLRLVGLKKSFYGSTLFLCRRPTPQDSPIFLPVDDTSFRWVESLKGILADEDSSRP

VWLKAINCATSGVVGLVNCLRREPGGNRLRCVLLSNLSSTSHVPEVDPGSAELQKVLQGD

LVMNVYRDGAWGAFRHFLLEEDKPEEPTAHAFVSTLTRGDLSSIRWVCSSLRHAQPTCPG

AQLCTVYYASLNFRDIMLATGKLSPDAIPGKWTSQDSLLGMEFSGRDASGKRVMGLVPAK

GLATSVLLSPDFLWDVPSNWTLEEAASVPVVYSTAYYALVVRGRVRPGETLLIHSGSGGV

GQAAIAIALSLGCRVFTTVGSAEKRAYLQARFPQLDSTSFANSRDTSFEQHVLWHTGGKG

VDLVLNSLAEEKLQASVRCLATHGRFLEIGKFDLSQNHPLGMAIFLKNVTFHGVLLDAFF

NESSADWREVWALVQAGIRDGVVRPLKCTVFHGAQVEDAFRYMAQGKHIGKVVVQVLAEE

PEAVLKGAKPKLMSAISKTFCPAHKSYIIAGGLGGFGLELAQWLIQRGVQKLVLTSRSGI

RTGYQAKQVRRWRRQGVQVQVSTSNISSLEGARGLIAEAAQLGPVGGVFNLAVVLRDGLL

ENQTPEFFQDVCKPKYSGTLNLDRVTREACPELDYFVVFSSVSCGRGNAGQSNYGFANSA

MERICEKRRHEGLPGLAVQWGAIGDVGILVETMSTNDTIVSGTLPQRMASCLEVLDLFLN

QPHMVLSSFVLAEKAAAYRDRDSQRDLVEAVAHILGIRDLAAVNLDSSLADLGLDSLMSV

EVRQTLERELNLVLSVREVRQLTLRKLQELSSKADEASELACPTPKEDGLAQQQTQLNLR

SLLVNPEGPTLMRLNSVQSSERPLFLVHPIEGSTTVFHSLASRLSIPTYGLQCTRAAPLD

SIHSLAAYYIDCIRQVQPEGPYRVAGYSYGACVAFEMCSQLQAQQSPAPTHNSLFLFDGS

PTYVLAYTQSYRAKLTPGCEAEAETEAICFFVQQFTDMEHNRVLEALLPLKGLEERVAAA

VDLIIKSHQGLDRQELSFAARSFYYKLRAAEQYTPKAKYHGNVMLLRAKTGGAYGEDLGA

DYNLSQVCDGKVSVHVIEGDHRTLLEGSGLESIISIIHSSLAEPRVSVREG

>sp|P49327|FAS_HUMAN 545 TFDDIVHSF

MEEVVIAGMSGKLPESENLQEFWDNLIGGVDMVTDDDRRWKAGLYGLPRRSGKLKDLSRF

DASFFGVHPKQAHTMDPQLRLLLEVTYEAIVDGGINPDSLRGTHTGVWVGVSGSETSEAL

SRDPETLVGYSMVGCQRAMMANRLSFFFDFRGPSIALDTACSSSLMALQNAYQAIHSGQC

PAAIVGGINVLLKPNTSVQFLRLGMLSPEGTCKAFDTAGNGYCRSEGVVAVLLTKKSLAR

RVYATILNAGTNTDGFKEQGVTFPSGDIQEQLIRSLYQSAGVAPESFEYIEAHGTGTKVG

DPQELNGITRALCATRQEPLLIGSTKSNMGHPEPASGLAALAKVLLSLEHGLWAPNLHFH

SPNPEIPALLDGRLQVVDQPLPVRGGNVGINSFGFGGSNVHIILRPNTQPPPAPAPHATL

PRLLRASGRTPEAVQKLLEQGLRHSQDLAFLSMLNDIAAVPATAMPFRGYAVLGGERGGP

EVQQVPAGERPLWFICSGMGTQWRGMGLSLMRLDRFRDSILRSDEAVKPFGLKVSQLLLS

TDESTFDDIVHSFVSLTAIQIGLIDLLSCMGLRPDGIVGHSLGEVACGYADGCLSQEEAV

LAAYWRGQCIKEAHLPPGAMAAVGLSWEECKQRCPPGVVPACHNSKDTVTISGPQAPVFE

FVEQLRKEGVFAKEVRTGGMAFHSYFMEAIAPPLLQELKKVIREPKPRSARWLSTSIPEA

QWHSSLARTSSAEYNVNNLVSPVLFQEALWHVPEHAVVLEIAPHALLQAVLKRGLKPSCT

IIPLMKKDHRDNLEFFLAGIGRLHLSGIDANPNALFPPVEFPAPRGTPLISPLIKWDHSL

AWDVPAAEDFPNGSGSPSAAIYNIDTSSESPDHYLVDHTLDGRVLFPATGYLSIVWKTLA

RALGLGVEQLPVVFEDVVLHQATILPKTGTVSLEVRLLEASRAFEVSENGNLVVSGKVYQ

WDDPDPRLFDHPESPTPNPTEPLFLAQAEVYKELRLRGYDYGPHFQGILEASLEGDSGRL

LWKDNWVSFMDTMLQMSILGSAKHGLYLPTRVTAIHIDPATHRQKLYTLQDKAQVADVVV

SRWLRVTVAGGVHISGLHTESAPRRQQEQQVPILEKFCFTPHTEEGCLSERAALQEELQL

CKGLVQALQTTVTQQGLKMVVPGLDGAQIPRDPSQQELPRLLSAACRLQLNGNLQLELAQ

VLAQERPKLPEDPLLSGLLDSPALKACLDTAVENMPSLKMKVVEVLAGHGHLYSRIPGLL

SPHPLLQLSYTATDRHPQALEAAQAELQQHDVAQGQWDPADPAPSALGSADLLVCNCAVA

ALGDPASALSNMVAALREGGFLLLHTLLRGHPLGDIVAFLTSTEPQYGQGILSQDAWESL

FSRVSLRLVGLKKSFYGSTLFLCRRPTPQDSPIFLPVDDTSFRWVESLKGILADEDSSRP

VWLKAINCATSGVVGLVNCLRREPGGNRLRCVLLSNLSSTSHVPEVDPGSAELQKVLQGD

LVMNVYRDGAWGAFRHFLLEEDKPEEPTAHAFVSTLTRGDLSSIRWVCSSLRHAQPTCPG

AQLCTVYYASLNFRDIMLATGKLSPDAIPGKWTSQDSLLGMEFSGRDASGKRVMGLVPAK

GLATSVLLSPDFLWDVPSNWTLEEAASVPVVYSTAYYALVVRGRVRPGETLLIHSGSGGV

GQAAIAIALSLGCRVFTTVGSAEKRAYLQARFPQLDSTSFANSRDTSFEQHVLWHTGGKG

VDLVLNSLAEEKLQASVRCLATHGRFLEIGKFDLSQNHPLGMAIFLKNVTFHGVLLDAFF

NESSADWREVWALVQAGIRDGVVRPLKCTVFHGAQVEDAFRYMAQGKHIGKVVVQVLAEE

PEAVLKGAKPKLMSAISKTFCPAHKSYIIAGGLGGFGLELAQWLIQRGVQKLVLTSRSGI

RTGYQAKQVRRWRRQGVQVQVSTSNISSLEGARGLIAEAAQLGPVGGVFNLAVVLRDGLL

ENQTPEFFQDVCKPKYSGTLNLDRVTREACPELDYFVVFSSVSCGRGNAGQSNYGFANSA

MERICEKRRHEGLPGLAVQWGAIGDVGILVETMSTNDTIVSGTLPQRMASCLEVLDLFLN

QPHMVLSSFVLAEKAAAYRDRDSQRDLVEAVAHILGIRDLAAVNLDSSLADLGLDSLMSV

EVRQTLERELNLVLSVREVRQLTLRKLQELSSKADEASELACPTPKEDGLAQQQTQLNLR

SLLVNPEGPTLMRLNSVQSSERPLFLVHPIEGSTTVFHSLASRLSIPTYGLQCTRAAPLD

SIHSLAAYYIDCIRQVQPEGPYRVAGYSYGACVAFEMCSQLQAQQSPAPTHNSLFLFDGS

PTYVLAYTQSYRAKLTPGCEAEAETEAICFFVQQFTDMEHNRVLEALLPLKGLEERVAAA

VDLIIKSHQGLDRQELSFAARSFYYKLRAAEQYTPKAKYHGNVMLLRAKTGGAYGEDLGA

DYNLSQVCDGKVSVHVIEGDHRTLLEGSGLESIISIIHSSLAEPRVSVREG

>sp|Q13813|SPTA2_HUMAN 781 ADSLRLQQL

MDPSGVKVLETAEDIQERRQQVLDRYHRFKELSTLRRQKLEDSYRFQFFQRDAEELEKWI

QEKLQIASDENYKDPTNLQGKLQKHQAFEAEVQANSGAIVKLDETGNLMISEGHFASETI

RTRLMELHRQWELLLEKMREKGIKLLQAQKLVQYLRECEDVMDWINDKEAIVTSEELGQD

LEHVEVLQKKFEEFQTDMAAHEERVNEVNQFAAKLIQEQHPEEELIKTKQDEVNAAWQRL

KGLALQRQGKLFGAAEVQRFNRDVDETISWIKEKEQLMASDDFGRDLASVQALLRKHEGL

ERDLAALEDKVKALCAEADRLQQSHPLSATQIQVKREELITNWEQIRTLAAERHARLNDS

YRLQRFLADFRDLTSWVTEMKALINADELASDVAGAEALLDRHQEHKGEIDAHEDSFKSA

DESGQALLAAGHYASDEVREKLTVLSEERAALLELWELRRQQYEQCMDLQLFYRDTEQVD

NWMSKQEAFLLNEDLGDSLDSVEALLKKHEDFEKSLSAQEEKITALDEFATKLIQNNHYA

MEDVATRRDALLSRRNALHERAMRRRAQLADSFHLQQFFRDSDELKSWVNEKMKTATDEA

YKDPSNLQGKVQKHQAFEAELSANQSRIDALEKAGQKLIDVNHYAKDEVAARMNEVISLW

KKLLEATELKGIKLREANQQQQFNRNVEDIELWLYEVEGHLASDDYGKDLTNVQNLQKKH

ALLEADVAAHQDRIDGITIQARQFQDAGHFDAENIKKKQEALVARYEALKEPMVARKQKL

ADSLRLQQLFRDVEDEETWIREKEPIAASTNRGKDLIGVQNLLKKHQALQAEIAGHEPRI

KAVTQKGNAMVEEGHFAAEDVKAKLHELNQKWEALKAKASQRRQDLEDSLQAQQYFADAN

EAESWMREKEPIVGSTDYGKDEDSAEALLKKHEALMSDLSAYGSSIQALREQAQSCRQQV

APTDDETGKELVLALYDYQEKSPREVTMKKGDILTLLNSTNKDWWKVEVNDRQGFVPAAY

VKKLDPAQSASRENLLEEQGSIALRQEQIDNQTRITKEAGSVSLRMKQVEELYHSLLELG

EKRKGMLEKSCKKFMLFREANELQQWINEKEAALTSEEVGADLEQVEVLQKKFDDFQKDL

KANESRLKDINKVAEDLESEGLMAEEVQAVQQQEVYGMMPRDETDSKTASPWKSARLMVH

TVATFNSIKELNERWRSLQQLAEERSQLLGSAHEVQRFHRDADETKEWIEEKNQALNTDN

YGHDLASVQALQRKHEGFERDLAALGDKVNSLGETAERLIQSHPESAEDLQEKCTELNQA

WSSLGKRADQRKAKLGDSHDLQRFLSDFRDLMSWINGIRGLVSSDELAKDVTGAEALLER

HQEHRTEIDARAGTFQAFEQFGQQLLAHGHYASPEIKQKLDILDQERADLEKAWVQRRMM

LDQCLELQLFHRDCEQAENWMAAREAFLNTEDKGDSLDSVEALIKKHEDFDKAINVQEEK

IAALQAFADQLIAAGHYAKGDISSRRNEVLDRWRRLKAQMIEKRSKLGESQTLQQFSRDV

DEIEAWISEKLQTASDESYKDPTNIQSKHQKHQAFEAELHANADRIRGVIDMGNSLIERG

ACAGSEDAVKARLAALADQWQFLVQKSAEKSQKLKEANKQQNFNTGIKDFDFWLSEVEAL

LASEDYGKDLASVNNLLKKHQLLEADISAHEDRLKDLNSQADSLMTSSAFDTSQVKDKRD

TINGRFQKIKSMAASRRAKLNESHRLHQFFRDMDDEESWIKEKKLLVGSEDYGRDLTGVQ

NLRKKHKRLEAELAAHEPAIQGVLDTGKKLSDDNTIGKEEIQQRLAQFVEHWKELKQLAA

ARGQRLEESLEYQQFVANVEEEEAWINEKMTLVASEDYGDTLAAIQGLLKKHEAFETDFT

VHKDRVNDVCTNGQDLIKKNNHHEENISSKMKGLNGKVSDLEKAAAQRKAKLDENSAFLQ

FNWKADVVESWIGEKENSLKTDDYGRDLSSVQTLLTKQETFDAGLQAFQQEGIANITALK

DQLLAAKHVQSKAIEARHASLMKRWSQLLANSAARKKKLLEAQSHFRKVEDLFLTFAKKA

SAFNSWFENAEEDLTDPVRCNSLEEIKALREAHDAFRSSLSSAQADFNQLAELDRQIKSF

RVASNPYTWFTMEALEETWRNLQKIIKERELELQKEQRRQEENDKLRQEFAQHANAFHQW

IQETRTYLLDGSCMVEESGTLESQLEATKRKHQEIRAMRSQLKKIEDLGAAMEEALILDN

KYTEHSTVGLAQQWDQLDQLGMRMQHNLEQQIQARNTTGVTEEALKEFSMMFKHFDKDKS

GRLNHQEFKSCLRSLGYDLPMVEEGEPDPEFEAILDTVDPNRDGHVSLQEYMAFMISRET

ENVKSSEEIESAFRALSSEGKPYVTKEELYQNLTREQADYCVSHMKPYVDGKGRELPTAF

DYVEFTRSLFVN

>sp|Q13813|SPTA2_HUMAN 2019 ETFDAGLQAF

MDPSGVKVLETAEDIQERRQQVLDRYHRFKELSTLRRQKLEDSYRFQFFQRDAEELEKWI

QEKLQIASDENYKDPTNLQGKLQKHQAFEAEVQANSGAIVKLDETGNLMISEGHFASETI

RTRLMELHRQWELLLEKMREKGIKLLQAQKLVQYLRECEDVMDWINDKEAIVTSEELGQD

LEHVEVLQKKFEEFQTDMAAHEERVNEVNQFAAKLIQEQHPEEELIKTKQDEVNAAWQRL

KGLALQRQGKLFGAAEVQRFNRDVDETISWIKEKEQLMASDDFGRDLASVQALLRKHEGL

ERDLAALEDKVKALCAEADRLQQSHPLSATQIQVKREELITNWEQIRTLAAERHARLNDS

YRLQRFLADFRDLTSWVTEMKALINADELASDVAGAEALLDRHQEHKGEIDAHEDSFKSA

DESGQALLAAGHYASDEVREKLTVLSEERAALLELWELRRQQYEQCMDLQLFYRDTEQVD

NWMSKQEAFLLNEDLGDSLDSVEALLKKHEDFEKSLSAQEEKITALDEFATKLIQNNHYA

MEDVATRRDALLSRRNALHERAMRRRAQLADSFHLQQFFRDSDELKSWVNEKMKTATDEA

YKDPSNLQGKVQKHQAFEAELSANQSRIDALEKAGQKLIDVNHYAKDEVAARMNEVISLW

KKLLEATELKGIKLREANQQQQFNRNVEDIELWLYEVEGHLASDDYGKDLTNVQNLQKKH

ALLEADVAAHQDRIDGITIQARQFQDAGHFDAENIKKKQEALVARYEALKEPMVARKQKL

ADSLRLQQLFRDVEDEETWIREKEPIAASTNRGKDLIGVQNLLKKHQALQAEIAGHEPRI

KAVTQKGNAMVEEGHFAAEDVKAKLHELNQKWEALKAKASQRRQDLEDSLQAQQYFADAN

EAESWMREKEPIVGSTDYGKDEDSAEALLKKHEALMSDLSAYGSSIQALREQAQSCRQQV

APTDDETGKELVLALYDYQEKSPREVTMKKGDILTLLNSTNKDWWKVEVNDRQGFVPAAY

VKKLDPAQSASRENLLEEQGSIALRQEQIDNQTRITKEAGSVSLRMKQVEELYHSLLELG

EKRKGMLEKSCKKFMLFREANELQQWINEKEAALTSEEVGADLEQVEVLQKKFDDFQKDL

KANESRLKDINKVAEDLESEGLMAEEVQAVQQQEVYGMMPRDETDSKTASPWKSARLMVH

TVATFNSIKELNERWRSLQQLAEERSQLLGSAHEVQRFHRDADETKEWIEEKNQALNTDN

YGHDLASVQALQRKHEGFERDLAALGDKVNSLGETAERLIQSHPESAEDLQEKCTELNQA

WSSLGKRADQRKAKLGDSHDLQRFLSDFRDLMSWINGIRGLVSSDELAKDVTGAEALLER

HQEHRTEIDARAGTFQAFEQFGQQLLAHGHYASPEIKQKLDILDQERADLEKAWVQRRMM

LDQCLELQLFHRDCEQAENWMAAREAFLNTEDKGDSLDSVEALIKKHEDFDKAINVQEEK

IAALQAFADQLIAAGHYAKGDISSRRNEVLDRWRRLKAQMIEKRSKLGESQTLQQFSRDV

DEIEAWISEKLQTASDESYKDPTNIQSKHQKHQAFEAELHANADRIRGVIDMGNSLIERG

ACAGSEDAVKARLAALADQWQFLVQKSAEKSQKLKEANKQQNFNTGIKDFDFWLSEVEAL

LASEDYGKDLASVNNLLKKHQLLEADISAHEDRLKDLNSQADSLMTSSAFDTSQVKDKRD

TINGRFQKIKSMAASRRAKLNESHRLHQFFRDMDDEESWIKEKKLLVGSEDYGRDLTGVQ

NLRKKHKRLEAELAAHEPAIQGVLDTGKKLSDDNTIGKEEIQQRLAQFVEHWKELKQLAA

ARGQRLEESLEYQQFVANVEEEEAWINEKMTLVASEDYGDTLAAIQGLLKKHEAFETDFT

VHKDRVNDVCTNGQDLIKKNNHHEENISSKMKGLNGKVSDLEKAAAQRKAKLDENSAFLQ

FNWKADVVESWIGEKENSLKTDDYGRDLSSVQTLLTKQETFDAGLQAFQQEGIANITALK

DQLLAAKHVQSKAIEARHASLMKRWSQLLANSAARKKKLLEAQSHFRKVEDLFLTFAKKA

SAFNSWFENAEEDLTDPVRCNSLEEIKALREAHDAFRSSLSSAQADFNQLAELDRQIKSF

RVASNPYTWFTMEALEETWRNLQKIIKERELELQKEQRRQEENDKLRQEFAQHANAFHQW

IQETRTYLLDGSCMVEESGTLESQLEATKRKHQEIRAMRSQLKKIEDLGAAMEEALILDN

KYTEHSTVGLAQQWDQLDQLGMRMQHNLEQQIQARNTTGVTEEALKEFSMMFKHFDKDKS

GRLNHQEFKSCLRSLGYDLPMVEEGEPDPEFEAILDTVDPNRDGHVSLQEYMAFMISRET

ENVKSSEEIESAFRALSSEGKPYVTKEELYQNLTREQADYCVSHMKPYVDGKGRELPTAF

DYVEFTRSLFVN

>sp|Q13813|SPTA2_HUMAN 1012 RQGFVPAAY

MDPSGVKVLETAEDIQERRQQVLDRYHRFKELSTLRRQKLEDSYRFQFFQRDAEELEKWI

QEKLQIASDENYKDPTNLQGKLQKHQAFEAEVQANSGAIVKLDETGNLMISEGHFASETI

RTRLMELHRQWELLLEKMREKGIKLLQAQKLVQYLRECEDVMDWINDKEAIVTSEELGQD

LEHVEVLQKKFEEFQTDMAAHEERVNEVNQFAAKLIQEQHPEEELIKTKQDEVNAAWQRL

KGLALQRQGKLFGAAEVQRFNRDVDETISWIKEKEQLMASDDFGRDLASVQALLRKHEGL

ERDLAALEDKVKALCAEADRLQQSHPLSATQIQVKREELITNWEQIRTLAAERHARLNDS

YRLQRFLADFRDLTSWVTEMKALINADELASDVAGAEALLDRHQEHKGEIDAHEDSFKSA

DESGQALLAAGHYASDEVREKLTVLSEERAALLELWELRRQQYEQCMDLQLFYRDTEQVD

NWMSKQEAFLLNEDLGDSLDSVEALLKKHEDFEKSLSAQEEKITALDEFATKLIQNNHYA

MEDVATRRDALLSRRNALHERAMRRRAQLADSFHLQQFFRDSDELKSWVNEKMKTATDEA

YKDPSNLQGKVQKHQAFEAELSANQSRIDALEKAGQKLIDVNHYAKDEVAARMNEVISLW

KKLLEATELKGIKLREANQQQQFNRNVEDIELWLYEVEGHLASDDYGKDLTNVQNLQKKH

ALLEADVAAHQDRIDGITIQARQFQDAGHFDAENIKKKQEALVARYEALKEPMVARKQKL

ADSLRLQQLFRDVEDEETWIREKEPIAASTNRGKDLIGVQNLLKKHQALQAEIAGHEPRI

KAVTQKGNAMVEEGHFAAEDVKAKLHELNQKWEALKAKASQRRQDLEDSLQAQQYFADAN

EAESWMREKEPIVGSTDYGKDEDSAEALLKKHEALMSDLSAYGSSIQALREQAQSCRQQV

APTDDETGKELVLALYDYQEKSPREVTMKKGDILTLLNSTNKDWWKVEVNDRQGFVPAAY

VKKLDPAQSASRENLLEEQGSIALRQEQIDNQTRITKEAGSVSLRMKQVEELYHSLLELG

EKRKGMLEKSCKKFMLFREANELQQWINEKEAALTSEEVGADLEQVEVLQKKFDDFQKDL

KANESRLKDINKVAEDLESEGLMAEEVQAVQQQEVYGMMPRDETDSKTASPWKSARLMVH

TVATFNSIKELNERWRSLQQLAEERSQLLGSAHEVQRFHRDADETKEWIEEKNQALNTDN

YGHDLASVQALQRKHEGFERDLAALGDKVNSLGETAERLIQSHPESAEDLQEKCTELNQA

WSSLGKRADQRKAKLGDSHDLQRFLSDFRDLMSWINGIRGLVSSDELAKDVTGAEALLER

HQEHRTEIDARAGTFQAFEQFGQQLLAHGHYASPEIKQKLDILDQERADLEKAWVQRRMM

LDQCLELQLFHRDCEQAENWMAAREAFLNTEDKGDSLDSVEALIKKHEDFDKAINVQEEK

IAALQAFADQLIAAGHYAKGDISSRRNEVLDRWRRLKAQMIEKRSKLGESQTLQQFSRDV

DEIEAWISEKLQTASDESYKDPTNIQSKHQKHQAFEAELHANADRIRGVIDMGNSLIERG

ACAGSEDAVKARLAALADQWQFLVQKSAEKSQKLKEANKQQNFNTGIKDFDFWLSEVEAL

LASEDYGKDLASVNNLLKKHQLLEADISAHEDRLKDLNSQADSLMTSSAFDTSQVKDKRD

TINGRFQKIKSMAASRRAKLNESHRLHQFFRDMDDEESWIKEKKLLVGSEDYGRDLTGVQ

NLRKKHKRLEAELAAHEPAIQGVLDTGKKLSDDNTIGKEEIQQRLAQFVEHWKELKQLAA

ARGQRLEESLEYQQFVANVEEEEAWINEKMTLVASEDYGDTLAAIQGLLKKHEAFETDFT

VHKDRVNDVCTNGQDLIKKNNHHEENISSKMKGLNGKVSDLEKAAAQRKAKLDENSAFLQ

FNWKADVVESWIGEKENSLKTDDYGRDLSSVQTLLTKQETFDAGLQAFQQEGIANITALK

DQLLAAKHVQSKAIEARHASLMKRWSQLLANSAARKKKLLEAQSHFRKVEDLFLTFAKKA

SAFNSWFENAEEDLTDPVRCNSLEEIKALREAHDAFRSSLSSAQADFNQLAELDRQIKSF

RVASNPYTWFTMEALEETWRNLQKIIKERELELQKEQRRQEENDKLRQEFAQHANAFHQW

IQETRTYLLDGSCMVEESGTLESQLEATKRKHQEIRAMRSQLKKIEDLGAAMEEALILDN

KYTEHSTVGLAQQWDQLDQLGMRMQHNLEQQIQARNTTGVTEEALKEFSMMFKHFDKDKS

GRLNHQEFKSCLRSLGYDLPMVEEGEPDPEFEAILDTVDPNRDGHVSLQEYMAFMISRET

ENVKSSEEIESAFRALSSEGKPYVTKEELYQNLTREQADYCVSHMKPYVDGKGRELPTAF

DYVEFTRSLFVN

>sp|Q5UIP0|RIF1_HUMAN 2454 SVIKNLQSR

MTARGQSPLAPLLETLEDPSASHGGQTDAYLTLTSRMTGEEGKEVITEIEKKLPRLYKVL

KTHISSQNSELSSAALQALGFCLYNPKITSELSEANALELLSKLNDTIKNSDKNVRTRAL

WVISKQTFPSEVVGKMVSSIIDSLEILFNKGETHSAVVDFEALNVIVRLIEQAPIQMGEE

AVRWAKLVIPLVVHSAQKVHLRGATALEMGMPLLLQKQQEIASITEQLMTTKLISELQKL

FMSKNETYVLKLWPLFVKLLGRTLHRSGSFINSLLQLEELGFRSGAPMIKKIAFIAWKSL

IDNFALNPDILCSAKRLKLLMQPLSSIHVRTETLALTKLEVWWYLLMRLGPHLPANFEQV

CVPLIQSTISIDSNASPQGNSCHVATSPGLNPMTPVHKGASSPYGAPGTPRMNLSSNLGG

MATIPSIQLLGLEMLLHFLLGPEALSFAKQNKLVLSLEPLEHPLISSPSFFSKHANTLIT

AVHDSFVAVGKDAPDVVVSAIWKELISLVKSVTESGNKKEKPGSEVLTLLLKSLESIVKS

EVFPVSKTLVLMEITIKGLPQKVLGSPAYQVANMDILNGTPALFLIQLIFNNFLECGVSD

ERFFLSLESLVGCVLSGPTSPLAFSDSVLNVINQNAKQLENKEHLWKMWSVIVTPLTELI

NQTNEVNQGDALEHNFSAIYGALTLPVNHIFSEQRFPVATMKTLLRTWSELYRAFARCAA

LVATAEENLCCEELSSKIMSSLEDEGFSNLLFVDRIIYIITVMVDCIDFSPYNIKYQPKV

KSPQRPSDWSKKKNEPLGKLTSLFKLIVKVIYSFHTLSFKEAHSDTLFTIGNSITGIISS

VLGHISLPSMIRKIFATLTRPLALFYENSKLDEVPKVYSCLNNKLEKLLGEIIACLQFSY

TGTYDSELLEQLSPLLCIIFLHKNKQIRKQSAQFWNATFAKVMMLVYPEELKPVLTQAKQ

KFLLLLPGLETVEMMEESSGPYSDGTENSQLNVKISGMERKSNGKRDSFLAQTKNKKENM

KPAAKLKLESSSLKVKGEILLEEEKSTDFVFIPPEGKDAKERILTDHQKEVLKTKRCDIP

AMYNNLDVSQDTLFTQYSQEEPMEIPTLTRKPKEDSKMMITEEQMDSDIVIPQDVTEDCG

MAEHLEKSSLSNNECGSLDKTSPEMSNSNNDERKKALISSRKTSTECASSTENSFVVSSS

SVSNTTVAGTPPYPTSRRQTFITLEKFDGSENRPFSPSPLNNISSTVTVKNNQETMIKTD

FLPKAKQREGTFSKSDSEKIVNGTKRSSRRAGKAEQTGNKRSKPLMRSEPEKNTEESVEG

IVVLENNPPGLLNQTECVSDNQVHLSESTMEHDNTKLKAATVENAVLLETNTVEEKNVEI

NLESKENTPPVVISADQMVNEDSQVQITPNQKTLRRSSRRRSEVVESTTESQDKENSHQK

KERRKEEEKPLQKSPLHIKDDVLPKQKLIAEQTLQENLIEKGSNLHEKTLGETSANAETE

QNKKKADPENIKSEGDGTQDIVDKSSEKLVRGRTRYQTRRASQGLLSSIENSESDSSEAK

EEGSRKKRSGKWKNKSNESVDIQDQEEKVVKQECIKAENQSHDYKATSEEDVSIKSPICE

KQDESNTVICQDSTVTSDLLQVPDDLPNVCEEKNETSKYAEYSFTSLPVPESNLRTRNAI

KRLHKRDSFDNCSLGESSKIGISDISSLSEKTFQTLECQHKRSRRVRRSKGCDCCGEKSQ

PQEKSLIGLKNTENNDVEISETKKADVQAPVSPSETSQANPYSEGQFLDEHHSVNFHLGL

KEDNDTINDSLIVSETKSKENTMQESLPSGIVNFREEICDMDSSEAMSLESQESPNENFK

TVGPCLGDSKNVSQESLETKEEKPEETPKMELSLENVTVEGNACKVTESNLEKAKTMELN

VGNEASFHGQERTKTGISEEAAIEENKRNDDSEADTAKLNAKEVATEEFNSDISLSDNTT

PVKLNAQTEISEQTAAGELDGGNDVSDLHSSEETNTKMKNNEEMMIGEAMAETGHDGETE

NEGITTKTSKPDEAETNMLTAEMDNFVCDTVEMSTEEGIIDANKTETNTEYSKSEEKLDN

NQMVMESDILQEDHHTSQKVEEPSQCLASGTAISELIIEDNNASPQKLRELDPSLVSAND

SPSGMQTRCVWSPLASPSTSILKRGLKRSQEDEISSPVNKVRRVSFADPIYQAGLADDID

RRCSIVRSHSSNSSPIGKSVKTSPTTQSKHNTTSAKGFLSPGSRSPKFKSSKKCLISEMA

KESIPCPTESVYPPLVNCVAPVDIILPQITSNMWARGLGQLIRAKNIKTIGDLSTLTASE

IKTLPIRSPKVSNVKKALRIYHEQQVKTRGLEEIPVFDISEKTVNGIENKSLSPDEERLV

SDIIDPVALEIPLSKNLLAQISALALQLDSEDLHNYSGSQLFEMHEKLSCMANSVIKNLQ

SRWRSPSHENSI

>sp|P46821|MAP1B_HUMAN 225 GLSEFTEYL

MATVVVEATEPEPSGSIANPAASTSPSLSHRFLDSKFYLLVVVGEIVTEEHLRRAIGNIE

LGIRSWDTNLIECNLDQELKLFVSRHSARFSPEVPGQKILHHRSDVLETVVLINPSDEAV

STEVRLMITDAARHKLLVLTGQCFENTGELILQSGSFSFQNFIEIFTDQEIGELLSTTHP

ANKASLTLFCPEEGDWKNSNLDRHNLQDFINIKLNSASILPEMEGLSEFTEYLSESVEVP

SPFDILEPPTSGGFLKLSKPCCYIFPGGRGDSALFAVNGFNMLINGGSERKSCFWKLIRH

LDRVDSILLTHIGDDNLPGINSMLQRKIAELEEEQSQGSTTNSDWMKNLISPDLGVVFLN

VPENLKNPEPNIKMKRSIEEACFTLQYLNKLSMKPEPLFRSVGNTIDPVILFQKMGVGKL

EMYVLNPVKSSKEMQYFMQQWTGTNKDKAEFILPNGQEVDLPISYLTSVSSLIVWHPANP

AEKIIRVLFPGNSTQYNILEGLEKLKHLDFLKQPLATQKDLTGQVPTPVVKQTKLKQRAD

SRESLKPAAKPLPSKSVRKESKEETPEVTKVNHVEKPPKVESKEKVMVKKDKPVKTETKP

SVTEKEVPSKEEPSPVKAEVAEKQATDVKPKAAKEKTVKKETKVKPEDKKEEKEKPKKEV

AKKEDKTPIKKEEKPKKEEVKKEVKKEIKKEEKKEPKKEVKKETPPKEVKKEVKKEEKKE

VKKEEKEPKKEIKKLPKDAKKSSTPLSEAKKPAALKPKVPKKEESVKKDSVAAGKPKEKG

KIKVIKKEGKAAEAVAAAVGTGATTAAVMAAAGIAAIGPAKELEAERSLMSSPEDLTKDF

EELKAEEVDVTKDIKPQLELIEDEEKLKETEPVEAYVIQKEREVTKGPAESPDEGITTTE

GEGECEQTPEELEPVEKQGVDDIEKFEDEGAGFEESSETGDYEEKAETEEAEEPEEDGEE

HVCVSASKHSPTEDEESAKAEADAYIREKRESVASGDDRAEEDMDEAIEKGEAEQSEEEA

DEEDKAEDAREEEYEPEKMEAEDYVMAVVDKAAEAGGAEEQYGFLTTPTKQLGAQSPGRE

PASSIHDETLPGGSESEATASDEENREDQPEEFTATSGYTQSTIEISSEPTPMDEMSTPR

DVMSDETNNEETESPSQEFVNITKYESSLYSQEYSKPADVTPLNGFSEGSKTDATDGKDY

NASASTISPPSSMEEDKFSRSALRDAYCSEVKASTTLDIKDSISAVSSEKVSPSKSPSLS

PSPPSPLEKTPLGERSVNFSLTPNEIKVSAEAEVAPVSPEVTQEVVEEHCASPEDKTLEV

VSPSQSVTGSAGHTPYYQSPTDEKSSHLPTEVIEKPPAVPVSFEFSDAKDENERASVSPM

DEPVPDSESPIEKVLSPLRSPPLIGSESAYESFLSADDKASGRGAESPFEEKSGKQGSPD

QVSPVSEMTSTSLYQDKQEGKSTDFAPIKEDFGQEKKTDDVEAMSSQPALALDERKLGDV

SPTQIDVSQFGSFKEDTKMSISEGTVSDKSATPVDEGVAEDTYSHMEGVASVSTASVATS

SFPEPTTDDVSPSLHAEVGSPHSTEVDDSLSVSVVQTPTTFQETEMSPSKEECPRPMSIS

PPDFSPKTAKSRTPVQDHRSEQSSMSIEFGQESPEQSLAMDFSRQSPDHPTVGAGVLHIT

ENGPTEVDYSPSDMQDSSLSHKIPPMEEPSYTQDNDLSELISVSQVEASPSTSSAHTPSQ

IASPLQEDTLSDVAPPRDMSLYASLTSEKVQSLEGEKLSPKSDISPLTPRESSPLYSPTF

SDSTSAVKEKTATCHSSSSPPIDAASAEPYGFRASVLFDTMQHHLALNRDLSTPGLEKDS

GGKTPGDFSYAYQKPEETTRSPDEEDYDYESYEKTTRTSDVGGYYYEKIERTTKSPSDSG

YSYETIGKTTKTPEDGDYSYEIIEKTTRTPEEGGYSYDISEKTTSPPEVSGYSYEKTERS

RRLLDDISNGYDDSEDGGHTLGDPSYSYETTEKITSFPESEGYSYETSTKTTRTPDTSTY

CYETAEKITRTPQASTYSYETSDLCYTAEKKSPSEARQDVDLCLVSSCEYKHPKTELSPS

FINPNPLEWFASEEPTEESEKPLTQSGGAPPPPGGKQQGRQCDETPPTSVSESAPSQTDS

DVPPETEECPSITADANIDSEDESETIPTDKTVTYKHMDPPPAPVQDRSPSPRHPDVSMV

DPEALAIEQNLGKALKKDLKEKTKTKKPGTKTKSSSPVKKSDGKSKPLAASPKPAGLKES

SDKVSRVASPKKKESVEKAAKPTTTPEVKAARGEEKDKETKNAANASASKSAKTATAGPG

TTKTTKSSAVPPGLPVYLDLCYIPNHSNSKNVDVEFFKRVRSSYYVVSGNDPAAEEPSRA

VLDALLEGKAQWGSNMQVTLIPTHDSEVMREWYQETHEKQQDLNIMVLASSSTVVMQDES

FPACKIEL

>sp|P31629|ZEP2_HUMAN 2115 RPVSPGKDI

MDTGDTALGQKATSRSGETDKASGRWRQEQSAVIKMSTFGSHEGQRQPQIEPEQIGNTAS

AQLFGSGKLASPSEVVQQVAEKQYPPHRPSPYSCQHSLSFPQHSLPQGVMHSTKPHQSLE

GPPWLFPGPLPSVASEDLFPFPIHGHSGGYPRKKISSLNPAYSQYSQKSIEQAEEAHKKE

HKPKKPGKYICPYCSRACAKPSVLKKHIRSHTGERPYPCIPCGFSFKTKSNLYKHRKSHA

HAIKAGLVPFTESAVSKLDLEAGFIDVEAEIHSDGEQSTDTDEESSLFAEASDKMSPGPP

IPLDIASRGGYHGSLEESLGGPMKVPILIIPKSGIPLPNESSQYIGPDMLPNPSLNTKAD

DSHTVKQKLALRLSEKKGQDSEPSLNLLSPHSKGSTDSGYFSRSESAEQQISPPNTNAKS

YEEIIFGKYCRLSPRNALSVTTTSQERAAMGRKGIMEPLPHVNTRLDVKMFEDPVSQLIP

SKGDVDPSQTSMLKSTKFNSESRQPQIIPSSIRNEGKLYPANFQGSNPVLLEAPVDSSPL

IRSNSVPTSSATNLTIPPSLRGSHSFDERMTGSDDVFYPGTVGIPPQRMLRRQAAFELPS

VQEGHVEVEHHGRMLKGISSSSLKEKKLSPGDRVGYDYDVCRKPYKKWEDSETPKQNYRD

ISCLSSLKHGGEYFMDPVVPLQGVPSMFGTTCENRKRRKEKSVGDEEDTPMICSSIVSTP

VGIMASDYDPKLQMQEGVRSGFAMAGHENLSHGHTERFDPCRPQLQPGSPSLVSEESPSA

IDSDKMSDLGGRKPPGNVISVIQHTNSLSRPNSFERSESAELVACTQDKAPSPSETCDSE

ISEAPVSPEWAPPGDGAESGGKPSPSQQVQQQSYHTQPRLVRQHNIQVPEIRVTEEPDKP

EKEKEAQSKEPEKPVEEFQWPQRSETLSQLPAEKLPPKKKRLRLADMEHSSGESSFESTG

TGLSRSPSQESNLSHSSSFSMSFEREETSKLSALPKQDEFGKHSEFLTVPAGSYSLSVPG

HHHQKEMRRCSSEQMPCPHPAEVPEVRSKSFDYGNLSHAPVSGAAASTVSPSRERKKCFL

VRQASFSGSPEISQGEVGMDQSVKQEQLEHLHAGLRSGWHHGPPAVLPPLQQEDPGKQVA

GPCPPLSSGPLHLAQPQIMHMDSQESLRNPLIQPTSYMTSKHLPEQPHLFPHQETIPFSP

IQNALFQFQYPTVCMVHLPAQQPPWWQAHFPHPFAQHPQKSYGKPSFQTEIHSSYPLEHV

AEHTGKKPAEYAHTKEQTYPCYSGASGLHPKNLLPKFPSDQSSKSTETPSEQVLQEDFAS

ANAGSLQSLPGTVVPVRIQTHVPSYGSVMYTSISQILGQNSPAIVICKVDENMTQRTLVT

NAAMQGIGFNIAQVLGQHAGLEKYPIWKAPQTLPLGLESSIPLCLPSTSDSVATLGGSKR

MLSPASSLELFMETKQQKRVKEEKMYGQIVEELSAVELTNSDIKKDLSRPQKPQLVRQGC

ASEPKDGLQSGSSSFSSLSPSSSQDYPSVSPSSREPFLPSKEMLSGSRAPLPGQKSSGPS

ESKESSDELDIDETASDMSMSPQSSSLPAGDGQLEEEGKGHKRPVGMLVRMASAPSGNVA

DSTLLLTDMADFQQILQFPSLRTTTTVSWCFLNYTKPNYVQQATFKSSVYASWCISSCNP

NPSGLNTKTTLALLRSKQKITAEIYTLAAMHRPGTGKLTSSSAWKQFTQMKPDASFLFGS

KLERKLVGNILKERGKGDIHGDKDIGSKQTEPIRIKIFEGGYKSNEDYVYVRGRGRGKYI

CEECGIRCKKPSMLKKHIRTHTDVRPYVCKLCNFAFKTKGNLTKHMKSKAHMKKCLELGV

SMTSVDDTETEEAENLEDLHKAAEKHSMSSISTDHQFSDAEESDGEDGDDNDDDDEDEDD

FDDQGDLTPKTRSRSTSPQPPRFSSLPVNVGAVPHGVPSDSSLGHSSLISYLVTLPSIRV

TQLMTPSDSCEDTQMTEYQRLFQSKSTDSEPDKDRLDIPSCMDEECMLPSEPSSSPRDFS

PSSHHSSPGYDSSPCRDNSPKRYLIPKGDLSPRRHLSPRRDLSPMRHLSPRKEAALRREM

SQRDVSPRRHLSPRRPVSPGKDITARRDLSPRRERRYMTTIRAPSPRRALYHNPPLSMGQ

YLQAEPIVLGPPNLRRGLPQVPYFSLYGDQEGAYEHPGSSLFPEGPNDYVFSHLPLHSQQ

QVRAPIPMVPVGGIQMVHSMPPALSSLHPSPTLPLPMEGFEEKKGASGESFSKDPYVLSK

QHEKRGPHALQSSGPPSTPSSPRLLMKQSTSEDSLNATEREQEENIQTCTKAIASLRIAT

EEAALLGPDQPARVQEPHQNPLGSAHVSIRHFSRPEPGQPCTSATHPDLHDGEKDNFGTS

QTPLAHSTFYSKSCVDDKQLDFHSSKELSSSTEESKDPSSEKSQLH

>sp|P18583|SON_HUMAN 2026 RRFSRSPIR

MATNIEQIFRSFVVSKFREIQQELSSGRNEGQLNGETNTPIEGNQAGDAAASARSLPNEE

IVQKIEEVLSGVLDTELRYKPDLKEGSRKSRCVSVQTDPTDEIPTKKSKKHKKHKNKKKK

KKKEKEKKYKRQPEESESKTKSHDDGNIDLESDSFLKFDSEPSAVALELPTRAFGPSETN

ESPAVVLEPPVVSMEVSEPHILETLKPATKTAELSVVSTSVISEQSEQSVAVMPEPSMTK

ILDSFAAAPVPTTTLVLKSSEPVVTMSVEYQMKSVLKSVESTSPEPSKIMLVEPPVAKVL

EPSETLVVSSETPTEVYPEPSTSTTMDFPESSAIEALRLPEQPVDVPSEIADSSMTRPQE

LPELPKTTALELQESSVASAMELPGPPATSMPELQGPPVTPVLELPGPSATPVPELPGPL

STPVPELPGPPATAVPELPGPSVTPVPQLSQELPGLPAPSMGLEPPQEVPEPSVMAQELP

GLPLVTAAVELPEQPAVTVAMELTEQPVTTTELEQPVGMTTVEHPGHPEVTTATGLLGQP

EATMVLELPGQPVATTALELPGQPSVTGVPELPGLPSATRALELSGQPVATGALELPGPL

MAAGALEFSGQSGAAGALELLGQPLATGVLELPGQPGAPELPGQPVATVALEISVQSVVT

TSELSTMTVSQSLEVPSTTALESYNTVAQELPTTLVGETSVTVGVDPLMAPESHILASNT

METHILASNTMDSQMLASNTMDSQMLASNTMDSQMLASSTMDSQMLATSSMDSQMLATSS

MDSQMLATSTMDSQMLATSSMDSQMLATSSMDSQMLATSSMDSQMLATSSMDSQMLATST

MDSQMLATSTMDSQMLATSSMDSQMLASGTMDSQMLASGTMDAQMLASGTMDAQMLASST

QDSAMLGSKSPDPYRLAQDPYRLAQDPYRLGHDPYRLGHDAYRLGQDPYRLGHDPYRLTP

DPYRMSPRPYRIAPRSYRIAPRPYRLAPRPLMLASRRSMMMSYAAERSMMSSYERSMMSY

ERSMMSPMAERSMMSAYERSMMSAYERSMMSPMAERSMMSAYERSMMSAYERSMMSPMAD

RSMMSMGADRSMMSSYSAADRSMMSSYSAADRSMMSSYTADRSMMSMAADSYTDSYTDTY

TEAYMVPPLPPEEPPTMPPLPPEEPPMTPPLPPEEPPEGPALPTEQSALTAENTWPTEVP

SLPSEESVSQPEPPVSQSEISEPSAVPTDYSVSASDPSVLVSEAAVTVPEPPPEPESSIT

LTPVESAVVAEEHEVVPERPVTCMVSETPAMSAEPTVLASEPPVMSETAETFDSMRASGH

VASEVSTSLLVPAVTTPVLAESILEPPAMAAPESSAMAVLESSAVTVLESSTVTVLESST

VTVLEPSVVTVPEPPVVAEPDYVTIPVPVVSALEPSVPVLEPAVSVLQPSMIVSEPSVSV

QESTVTVSEPAVTVSEQTQVIPTEVAIESTPMILESSIMSSHVMKGINLSSGDQNLAPEI

GMQEIALHSGEEPHAEEHLKGDFYESEHGINIDLNINNHLIAKEMEHNTVCAAGTSPVGE

IGEEKILPTSETKQRTVLDTYPGVSEADAGETLSSTGPFALEPDATGTSKGIEFTTASTL

SLVNKYDVDLSLTTQDTEHDMVISTSPSGGSEADIEGPLPAKDIHLDLPSNNNLVSKDTE

EPLPVKESDQTLAALLSPKESSGGEKEVPPPPKETLPDSGFSANIEDINEADLVRPLLPK

DMERLTSLRAGIEGPLLASDVGRDRSAASPVVSSMPERASESSSEEKDDYEIFVKVKDTH

EKSKKNKNRDKGEKEKKRDSSLRSRSKRSKSSEHKSRKRTSESRSRARKRSSKSKSHRSQ

TRSRSRSRRRRRSSRSRSKSRGRRSVSKEKRKRSPKHRSKSRERKRKRSSSRDNRKTVRA

RSRTPSRRSRSHTPSRRRRSRSVGRRRSFSISPSRRSRTPSRRSRTPSRRSRTPSRRSRT

PSRRSRTPSRRSRTPSRRRRSRSVVRRRSFSISPVRLRRSRTPLRRRFSRSPIRRKRSRS

SERGRSPKRLTDLDKAQLLEIAKANAAAMCAKAGVPLPPNLKPAPPPTIEEKVAKKSGGA

TIEELTEKCKQIAQSKEDDDVIVNKPHVSDEEEEEPPFYHHPFKLSEPKPIFFNLNIAAA

KPTPPKSQVTLTKEFPVSSGSQHRKKEADSVYGEWVPVEKNGEENKDDDNVFSSNLPSEP

VDISTAMSERALAQKRLSENAFDLEAMSMLNRAQERIDAWAQLNSIPGQFTGSTGVQVLT

QEQLANTGAQAWIKKDQFLRAAPVTGGMGAVLMRKMGWREGEGLGKNKEGNKEPILVDFK

TDRKGLVAVGERAQKRSGNFSAAMKDLSGKHPVSALMEICNKRRWQPPEFLLVHDSGPDH

RKHFLFRVLRNGALTRPNCMFFLNRY

>sp|P18583|SON_HUMAN 2026 RRFSRSPIRR

MATNIEQIFRSFVVSKFREIQQELSSGRNEGQLNGETNTPIEGNQAGDAAASARSLPNEE

IVQKIEEVLSGVLDTELRYKPDLKEGSRKSRCVSVQTDPTDEIPTKKSKKHKKHKNKKKK

KKKEKEKKYKRQPEESESKTKSHDDGNIDLESDSFLKFDSEPSAVALELPTRAFGPSETN

ESPAVVLEPPVVSMEVSEPHILETLKPATKTAELSVVSTSVISEQSEQSVAVMPEPSMTK

ILDSFAAAPVPTTTLVLKSSEPVVTMSVEYQMKSVLKSVESTSPEPSKIMLVEPPVAKVL

EPSETLVVSSETPTEVYPEPSTSTTMDFPESSAIEALRLPEQPVDVPSEIADSSMTRPQE

LPELPKTTALELQESSVASAMELPGPPATSMPELQGPPVTPVLELPGPSATPVPELPGPL

STPVPELPGPPATAVPELPGPSVTPVPQLSQELPGLPAPSMGLEPPQEVPEPSVMAQELP

GLPLVTAAVELPEQPAVTVAMELTEQPVTTTELEQPVGMTTVEHPGHPEVTTATGLLGQP

EATMVLELPGQPVATTALELPGQPSVTGVPELPGLPSATRALELSGQPVATGALELPGPL

MAAGALEFSGQSGAAGALELLGQPLATGVLELPGQPGAPELPGQPVATVALEISVQSVVT

TSELSTMTVSQSLEVPSTTALESYNTVAQELPTTLVGETSVTVGVDPLMAPESHILASNT

METHILASNTMDSQMLASNTMDSQMLASNTMDSQMLASSTMDSQMLATSSMDSQMLATSS

MDSQMLATSTMDSQMLATSSMDSQMLATSSMDSQMLATSSMDSQMLATSSMDSQMLATST

MDSQMLATSTMDSQMLATSSMDSQMLASGTMDSQMLASGTMDAQMLASGTMDAQMLASST

QDSAMLGSKSPDPYRLAQDPYRLAQDPYRLGHDPYRLGHDAYRLGQDPYRLGHDPYRLTP

DPYRMSPRPYRIAPRSYRIAPRPYRLAPRPLMLASRRSMMMSYAAERSMMSSYERSMMSY

ERSMMSPMAERSMMSAYERSMMSAYERSMMSPMAERSMMSAYERSMMSAYERSMMSPMAD

RSMMSMGADRSMMSSYSAADRSMMSSYSAADRSMMSSYTADRSMMSMAADSYTDSYTDTY

TEAYMVPPLPPEEPPTMPPLPPEEPPMTPPLPPEEPPEGPALPTEQSALTAENTWPTEVP

SLPSEESVSQPEPPVSQSEISEPSAVPTDYSVSASDPSVLVSEAAVTVPEPPPEPESSIT

LTPVESAVVAEEHEVVPERPVTCMVSETPAMSAEPTVLASEPPVMSETAETFDSMRASGH

VASEVSTSLLVPAVTTPVLAESILEPPAMAAPESSAMAVLESSAVTVLESSTVTVLESST

VTVLEPSVVTVPEPPVVAEPDYVTIPVPVVSALEPSVPVLEPAVSVLQPSMIVSEPSVSV

QESTVTVSEPAVTVSEQTQVIPTEVAIESTPMILESSIMSSHVMKGINLSSGDQNLAPEI

GMQEIALHSGEEPHAEEHLKGDFYESEHGINIDLNINNHLIAKEMEHNTVCAAGTSPVGE

IGEEKILPTSETKQRTVLDTYPGVSEADAGETLSSTGPFALEPDATGTSKGIEFTTASTL

SLVNKYDVDLSLTTQDTEHDMVISTSPSGGSEADIEGPLPAKDIHLDLPSNNNLVSKDTE

EPLPVKESDQTLAALLSPKESSGGEKEVPPPPKETLPDSGFSANIEDINEADLVRPLLPK

DMERLTSLRAGIEGPLLASDVGRDRSAASPVVSSMPERASESSSEEKDDYEIFVKVKDTH

EKSKKNKNRDKGEKEKKRDSSLRSRSKRSKSSEHKSRKRTSESRSRARKRSSKSKSHRSQ

TRSRSRSRRRRRSSRSRSKSRGRRSVSKEKRKRSPKHRSKSRERKRKRSSSRDNRKTVRA

RSRTPSRRSRSHTPSRRRRSRSVGRRRSFSISPSRRSRTPSRRSRTPSRRSRTPSRRSRT

PSRRSRTPSRRSRTPSRRRRSRSVVRRRSFSISPVRLRRSRTPLRRRFSRSPIRRKRSRS

SERGRSPKRLTDLDKAQLLEIAKANAAAMCAKAGVPLPPNLKPAPPPTIEEKVAKKSGGA

TIEELTEKCKQIAQSKEDDDVIVNKPHVSDEEEEEPPFYHHPFKLSEPKPIFFNLNIAAA

KPTPPKSQVTLTKEFPVSSGSQHRKKEADSVYGEWVPVEKNGEENKDDDNVFSSNLPSEP

VDISTAMSERALAQKRLSENAFDLEAMSMLNRAQERIDAWAQLNSIPGQFTGSTGVQVLT

QEQLANTGAQAWIKKDQFLRAAPVTGGMGAVLMRKMGWREGEGLGKNKEGNKEPILVDFK

TDRKGLVAVGERAQKRSGNFSAAMKDLSGKHPVSALMEICNKRRWQPPEFLLVHDSGPDH

RKHFLFRVLRNGALTRPNCMFFLNRY

>sp|Q01082|SPTB2_HUMAN 2260 AVCEVALDY

MTTTVATDYDNIEIQQQYSDVNNRWDVDDWDNENSSARLFERSRIKALADEREAVQKKTF

TKWVNSHLARVSCRITDLYTDLRDGRMLIKLLEVLSGERLPKPTKGRMRIHCLENVDKAL

QFLKEQRVHLENMGSHDIVDGNHRLTLGLIWTIILRFQIQDISVETEDNKEKKSAKDALL

LWCQMKTAGYPNVNIHNFTTSWRDGMAFNALIHKHRPDLIDFDKLKKSNAHYNLQNAFNL

AEQHLGLTKLLDPEDISVDHPDEKSIITYVVTYYHYFSKMKALAVEGKRIGKVLDNAIET

EKMIEKYESLASDLLEWIEQTIIILNNRKFANSLVGVQQQLQAFNTYRTVEKPPKFTEKG

NLEVLLFTIQSKMRANNQKVYMPREGKLISDINKAWERLEKAEHERELALRNELIRQEKL

EQLARRFDRKAAMRETWLSENQRLVSQDNFGFDLPAVEAATKKHEAIETDIAAYEERVQA

VVAVARELEAENYHDIKRITARKDNVIRLWEYLLELLRARRQRLEMNLGLQKIFQEMLYI

MDWMDEMKVLVLSQDYGKHLLGVEDLLQKHTLVEADIGIQAERVRGVNASAQKFATDGEG

YKPCDPQVIRDRVAHMEFCYQELCQLAAERRARLEESRRLWKFFWEMAEEEGWIREKEKI

LSSDDYGKDLTSVMRLLSKHRAFEDEMSGRSGHFEQAIKEGEDMIAEEHFGSEKIRERII

YIREQWANLEQLSAIRKKRLEEASLLHQFQADADDIDAWMLDILKIVSSSDVGHDEYSTQ

SLVKKHKDVAEEIANYRPTLDTLHEQASALPQEHAESPDVRGRLSGIEERYKEVAELTRL

RKQALQDTLALYKMFSEADACELWIDEKEQWLNNMQIPEKLEDLEVIQHRFESLEPEMNN

QASRVAVVNQIARQLMHSGHPSEKEIKAQQDKLNTRWSQFRELVDRKKDALLSALSIQNY

HLECNETKSWIREKTKVIESTQDLGNDLAGVMALQRKLTGMERDLVAIEAKLSDLQKEAE

KLESEHPDQAQAILSRLAEISDVWEEMKTTLKNREASLGEASKLQQFLRDLDDFQSWLSR

TQTAIASEDMPNTLTEAEKLLTQHENIKNEIDNYEEDYQKMRDMGEMVTQGQTDAQYMFL

RQRLQALDTGWNELHKMWENRQNLLSQSHAYQQFLRDTKQAEAFLNNQEYVLAHTEMPTT

LEGAEAAIKKQEDFMTTMDANEEKINAVVETGRRLVSDGNINSDRIQEKVDSIDDRHRKN

RETASELLMRLKDNRDLQKFLQDCQELSLWINEKMLTAQDMSYDEARNLHSKWLKHQAFM

AELASNKEWLDKIEKEGMQLISEKPETEAVVKEKLTGLHKMWEVLESTTQTKAQRLFDAN

KAELFTQSCADLDKWLHGLESQIQSDDYGKDLTSVNILLKKQQMLENQMEVRKKEIEELQ

SQAQALSQEGKSTDEVDSKRLTVQTKFMELLEPLNERKHNLLASKEIHQFNRDVEDEILW

VGERMPLATSTDHGHNLQTVQLLIKKNQTLQKEIQGHQPRIDDIFERSQNIVTDSSSLSA

EAIRQRLADLKQLWGLLIEETEKRHRRLEEAHRAQQYYFDAAEAEAWMSEQELYMMSEEK

AKDEQSAVSMLKKHQILEQAVEDYAETVHQLSKTSRALVADSHPESERISMRQSKVDKLY

AGLKDLAEERRGKLDERHRLFQLNREVDDLEQWIAEREVVAGSHELGQDYEHVTMLQERF

REFARDTGNIGQERVDTVNHLADELINSGHSDAATIAEWKDGLNEAWADLLELIDTRTQI

LAASYELHKFYHDAKEIFGRIQDKHKKLPEELGRDQNTVETLQRMHTTFEHDIQALGTQV

RQLQEDAARLQAAYAGDKADDIQKRENEVLEAWKSLLDACESRRVRLVDTGDKFRFFSMV

RDLMLWMEDVIRQIEAQEKPRDVSSVELLMNNHQGIKAEIDARNDSFTTCIELGKSLLAR

KHYASEEIKEKLLQLTEKRKEMIDKWEDRWEWLRLILEVHQFSRDASVAEAWLLGQEPYL

SSREIGQSVDEVEKLIKRHEAFEKSAATWDERFSALERLTTLELLEVRRQQEEEERKRRP

PSPEPSTKVSEEAESQQQWDTSKGEQVSQNGLPAEQGSPRMAETVDTSEMVNGATEQRTS

SKESSPIPSPTSDRKAKTALPAQSAATLPARTQETPSAQMEGFLNRKHEWEAHNKKASSR

SWHNVYCVINNQEMGFYKDAKTAASGIPYHSEVPVSLKEAVCEVALDYKKKKHVFKLRLN

DGNEYLFQAKDDEEMNTWIQAISSAISSDKHEVSASTQSTPASSRAQTLPTSVVTITSES

SPGKREKDKEKDKEKRFSLFGKKK

>sp|Q01082|SPTB2_HUMAN 545 DEMKVLVL

MTTTVATDYDNIEIQQQYSDVNNRWDVDDWDNENSSARLFERSRIKALADEREAVQKKTF

TKWVNSHLARVSCRITDLYTDLRDGRMLIKLLEVLSGERLPKPTKGRMRIHCLENVDKAL

QFLKEQRVHLENMGSHDIVDGNHRLTLGLIWTIILRFQIQDISVETEDNKEKKSAKDALL

LWCQMKTAGYPNVNIHNFTTSWRDGMAFNALIHKHRPDLIDFDKLKKSNAHYNLQNAFNL

AEQHLGLTKLLDPEDISVDHPDEKSIITYVVTYYHYFSKMKALAVEGKRIGKVLDNAIET

EKMIEKYESLASDLLEWIEQTIIILNNRKFANSLVGVQQQLQAFNTYRTVEKPPKFTEKG

NLEVLLFTIQSKMRANNQKVYMPREGKLISDINKAWERLEKAEHERELALRNELIRQEKL

EQLARRFDRKAAMRETWLSENQRLVSQDNFGFDLPAVEAATKKHEAIETDIAAYEERVQA

VVAVARELEAENYHDIKRITARKDNVIRLWEYLLELLRARRQRLEMNLGLQKIFQEMLYI

MDWMDEMKVLVLSQDYGKHLLGVEDLLQKHTLVEADIGIQAERVRGVNASAQKFATDGEG

YKPCDPQVIRDRVAHMEFCYQELCQLAAERRARLEESRRLWKFFWEMAEEEGWIREKEKI

LSSDDYGKDLTSVMRLLSKHRAFEDEMSGRSGHFEQAIKEGEDMIAEEHFGSEKIRERII

YIREQWANLEQLSAIRKKRLEEASLLHQFQADADDIDAWMLDILKIVSSSDVGHDEYSTQ

SLVKKHKDVAEEIANYRPTLDTLHEQASALPQEHAESPDVRGRLSGIEERYKEVAELTRL

RKQALQDTLALYKMFSEADACELWIDEKEQWLNNMQIPEKLEDLEVIQHRFESLEPEMNN

QASRVAVVNQIARQLMHSGHPSEKEIKAQQDKLNTRWSQFRELVDRKKDALLSALSIQNY

HLECNETKSWIREKTKVIESTQDLGNDLAGVMALQRKLTGMERDLVAIEAKLSDLQKEAE

KLESEHPDQAQAILSRLAEISDVWEEMKTTLKNREASLGEASKLQQFLRDLDDFQSWLSR

TQTAIASEDMPNTLTEAEKLLTQHENIKNEIDNYEEDYQKMRDMGEMVTQGQTDAQYMFL

RQRLQALDTGWNELHKMWENRQNLLSQSHAYQQFLRDTKQAEAFLNNQEYVLAHTEMPTT

LEGAEAAIKKQEDFMTTMDANEEKINAVVETGRRLVSDGNINSDRIQEKVDSIDDRHRKN

RETASELLMRLKDNRDLQKFLQDCQELSLWINEKMLTAQDMSYDEARNLHSKWLKHQAFM

AELASNKEWLDKIEKEGMQLISEKPETEAVVKEKLTGLHKMWEVLESTTQTKAQRLFDAN

KAELFTQSCADLDKWLHGLESQIQSDDYGKDLTSVNILLKKQQMLENQMEVRKKEIEELQ

SQAQALSQEGKSTDEVDSKRLTVQTKFMELLEPLNERKHNLLASKEIHQFNRDVEDEILW

VGERMPLATSTDHGHNLQTVQLLIKKNQTLQKEIQGHQPRIDDIFERSQNIVTDSSSLSA

EAIRQRLADLKQLWGLLIEETEKRHRRLEEAHRAQQYYFDAAEAEAWMSEQELYMMSEEK

AKDEQSAVSMLKKHQILEQAVEDYAETVHQLSKTSRALVADSHPESERISMRQSKVDKLY

AGLKDLAEERRGKLDERHRLFQLNREVDDLEQWIAEREVVAGSHELGQDYEHVTMLQERF

REFARDTGNIGQERVDTVNHLADELINSGHSDAATIAEWKDGLNEAWADLLELIDTRTQI

LAASYELHKFYHDAKEIFGRIQDKHKKLPEELGRDQNTVETLQRMHTTFEHDIQALGTQV

RQLQEDAARLQAAYAGDKADDIQKRENEVLEAWKSLLDACESRRVRLVDTGDKFRFFSMV

RDLMLWMEDVIRQIEAQEKPRDVSSVELLMNNHQGIKAEIDARNDSFTTCIELGKSLLAR

KHYASEEIKEKLLQLTEKRKEMIDKWEDRWEWLRLILEVHQFSRDASVAEAWLLGQEPYL

SSREIGQSVDEVEKLIKRHEAFEKSAATWDERFSALERLTTLELLEVRRQQEEEERKRRP

PSPEPSTKVSEEAESQQQWDTSKGEQVSQNGLPAEQGSPRMAETVDTSEMVNGATEQRTS

SKESSPIPSPTSDRKAKTALPAQSAATLPARTQETPSAQMEGFLNRKHEWEAHNKKASSR

SWHNVYCVINNQEMGFYKDAKTAASGIPYHSEVPVSLKEAVCEVALDYKKKKHVFKLRLN

DGNEYLFQAKDDEEMNTWIQAISSAISSDKHEVSASTQSTPASSRAQTLPTSVVTITSES

SPGKREKDKEKDKEKRFSLFGKKK

>sp|Q01082|SPTB2_HUMAN 741 EEASLLHQF

MTTTVATDYDNIEIQQQYSDVNNRWDVDDWDNENSSARLFERSRIKALADEREAVQKKTF

TKWVNSHLARVSCRITDLYTDLRDGRMLIKLLEVLSGERLPKPTKGRMRIHCLENVDKAL

QFLKEQRVHLENMGSHDIVDGNHRLTLGLIWTIILRFQIQDISVETEDNKEKKSAKDALL

LWCQMKTAGYPNVNIHNFTTSWRDGMAFNALIHKHRPDLIDFDKLKKSNAHYNLQNAFNL

AEQHLGLTKLLDPEDISVDHPDEKSIITYVVTYYHYFSKMKALAVEGKRIGKVLDNAIET

EKMIEKYESLASDLLEWIEQTIIILNNRKFANSLVGVQQQLQAFNTYRTVEKPPKFTEKG

NLEVLLFTIQSKMRANNQKVYMPREGKLISDINKAWERLEKAEHERELALRNELIRQEKL

EQLARRFDRKAAMRETWLSENQRLVSQDNFGFDLPAVEAATKKHEAIETDIAAYEERVQA

VVAVARELEAENYHDIKRITARKDNVIRLWEYLLELLRARRQRLEMNLGLQKIFQEMLYI

MDWMDEMKVLVLSQDYGKHLLGVEDLLQKHTLVEADIGIQAERVRGVNASAQKFATDGEG

YKPCDPQVIRDRVAHMEFCYQELCQLAAERRARLEESRRLWKFFWEMAEEEGWIREKEKI

LSSDDYGKDLTSVMRLLSKHRAFEDEMSGRSGHFEQAIKEGEDMIAEEHFGSEKIRERII

YIREQWANLEQLSAIRKKRLEEASLLHQFQADADDIDAWMLDILKIVSSSDVGHDEYSTQ

SLVKKHKDVAEEIANYRPTLDTLHEQASALPQEHAESPDVRGRLSGIEERYKEVAELTRL

RKQALQDTLALYKMFSEADACELWIDEKEQWLNNMQIPEKLEDLEVIQHRFESLEPEMNN

QASRVAVVNQIARQLMHSGHPSEKEIKAQQDKLNTRWSQFRELVDRKKDALLSALSIQNY

HLECNETKSWIREKTKVIESTQDLGNDLAGVMALQRKLTGMERDLVAIEAKLSDLQKEAE

KLESEHPDQAQAILSRLAEISDVWEEMKTTLKNREASLGEASKLQQFLRDLDDFQSWLSR

TQTAIASEDMPNTLTEAEKLLTQHENIKNEIDNYEEDYQKMRDMGEMVTQGQTDAQYMFL

RQRLQALDTGWNELHKMWENRQNLLSQSHAYQQFLRDTKQAEAFLNNQEYVLAHTEMPTT

LEGAEAAIKKQEDFMTTMDANEEKINAVVETGRRLVSDGNINSDRIQEKVDSIDDRHRKN

RETASELLMRLKDNRDLQKFLQDCQELSLWINEKMLTAQDMSYDEARNLHSKWLKHQAFM

AELASNKEWLDKIEKEGMQLISEKPETEAVVKEKLTGLHKMWEVLESTTQTKAQRLFDAN

KAELFTQSCADLDKWLHGLESQIQSDDYGKDLTSVNILLKKQQMLENQMEVRKKEIEELQ

SQAQALSQEGKSTDEVDSKRLTVQTKFMELLEPLNERKHNLLASKEIHQFNRDVEDEILW

VGERMPLATSTDHGHNLQTVQLLIKKNQTLQKEIQGHQPRIDDIFERSQNIVTDSSSLSA

EAIRQRLADLKQLWGLLIEETEKRHRRLEEAHRAQQYYFDAAEAEAWMSEQELYMMSEEK

AKDEQSAVSMLKKHQILEQAVEDYAETVHQLSKTSRALVADSHPESERISMRQSKVDKLY

AGLKDLAEERRGKLDERHRLFQLNREVDDLEQWIAEREVVAGSHELGQDYEHVTMLQERF

REFARDTGNIGQERVDTVNHLADELINSGHSDAATIAEWKDGLNEAWADLLELIDTRTQI

LAASYELHKFYHDAKEIFGRIQDKHKKLPEELGRDQNTVETLQRMHTTFEHDIQALGTQV

RQLQEDAARLQAAYAGDKADDIQKRENEVLEAWKSLLDACESRRVRLVDTGDKFRFFSMV

RDLMLWMEDVIRQIEAQEKPRDVSSVELLMNNHQGIKAEIDARNDSFTTCIELGKSLLAR

KHYASEEIKEKLLQLTEKRKEMIDKWEDRWEWLRLILEVHQFSRDASVAEAWLLGQEPYL

SSREIGQSVDEVEKLIKRHEAFEKSAATWDERFSALERLTTLELLEVRRQQEEEERKRRP

PSPEPSTKVSEEAESQQQWDTSKGEQVSQNGLPAEQGSPRMAETVDTSEMVNGATEQRTS

SKESSPIPSPTSDRKAKTALPAQSAATLPARTQETPSAQMEGFLNRKHEWEAHNKKASSR

SWHNVYCVINNQEMGFYKDAKTAASGIPYHSEVPVSLKEAVCEVALDYKKKKHVFKLRLN

DGNEYLFQAKDDEEMNTWIQAISSAISSDKHEVSASTQSTPASSRAQTLPTSVVTITSES

SPGKREKDKEKDKEKRFSLFGKKK

>sp|Q01082|SPTB2_HUMAN 668 KDLTSVMRL

MTTTVATDYDNIEIQQQYSDVNNRWDVDDWDNENSSARLFERSRIKALADEREAVQKKTF

TKWVNSHLARVSCRITDLYTDLRDGRMLIKLLEVLSGERLPKPTKGRMRIHCLENVDKAL

QFLKEQRVHLENMGSHDIVDGNHRLTLGLIWTIILRFQIQDISVETEDNKEKKSAKDALL

LWCQMKTAGYPNVNIHNFTTSWRDGMAFNALIHKHRPDLIDFDKLKKSNAHYNLQNAFNL

AEQHLGLTKLLDPEDISVDHPDEKSIITYVVTYYHYFSKMKALAVEGKRIGKVLDNAIET

EKMIEKYESLASDLLEWIEQTIIILNNRKFANSLVGVQQQLQAFNTYRTVEKPPKFTEKG

NLEVLLFTIQSKMRANNQKVYMPREGKLISDINKAWERLEKAEHERELALRNELIRQEKL

EQLARRFDRKAAMRETWLSENQRLVSQDNFGFDLPAVEAATKKHEAIETDIAAYEERVQA

VVAVARELEAENYHDIKRITARKDNVIRLWEYLLELLRARRQRLEMNLGLQKIFQEMLYI

MDWMDEMKVLVLSQDYGKHLLGVEDLLQKHTLVEADIGIQAERVRGVNASAQKFATDGEG

YKPCDPQVIRDRVAHMEFCYQELCQLAAERRARLEESRRLWKFFWEMAEEEGWIREKEKI

LSSDDYGKDLTSVMRLLSKHRAFEDEMSGRSGHFEQAIKEGEDMIAEEHFGSEKIRERII

YIREQWANLEQLSAIRKKRLEEASLLHQFQADADDIDAWMLDILKIVSSSDVGHDEYSTQ

SLVKKHKDVAEEIANYRPTLDTLHEQASALPQEHAESPDVRGRLSGIEERYKEVAELTRL

RKQALQDTLALYKMFSEADACELWIDEKEQWLNNMQIPEKLEDLEVIQHRFESLEPEMNN

QASRVAVVNQIARQLMHSGHPSEKEIKAQQDKLNTRWSQFRELVDRKKDALLSALSIQNY

HLECNETKSWIREKTKVIESTQDLGNDLAGVMALQRKLTGMERDLVAIEAKLSDLQKEAE

KLESEHPDQAQAILSRLAEISDVWEEMKTTLKNREASLGEASKLQQFLRDLDDFQSWLSR

TQTAIASEDMPNTLTEAEKLLTQHENIKNEIDNYEEDYQKMRDMGEMVTQGQTDAQYMFL

RQRLQALDTGWNELHKMWENRQNLLSQSHAYQQFLRDTKQAEAFLNNQEYVLAHTEMPTT

LEGAEAAIKKQEDFMTTMDANEEKINAVVETGRRLVSDGNINSDRIQEKVDSIDDRHRKN

RETASELLMRLKDNRDLQKFLQDCQELSLWINEKMLTAQDMSYDEARNLHSKWLKHQAFM

AELASNKEWLDKIEKEGMQLISEKPETEAVVKEKLTGLHKMWEVLESTTQTKAQRLFDAN

KAELFTQSCADLDKWLHGLESQIQSDDYGKDLTSVNILLKKQQMLENQMEVRKKEIEELQ

SQAQALSQEGKSTDEVDSKRLTVQTKFMELLEPLNERKHNLLASKEIHQFNRDVEDEILW

VGERMPLATSTDHGHNLQTVQLLIKKNQTLQKEIQGHQPRIDDIFERSQNIVTDSSSLSA

EAIRQRLADLKQLWGLLIEETEKRHRRLEEAHRAQQYYFDAAEAEAWMSEQELYMMSEEK

AKDEQSAVSMLKKHQILEQAVEDYAETVHQLSKTSRALVADSHPESERISMRQSKVDKLY

AGLKDLAEERRGKLDERHRLFQLNREVDDLEQWIAEREVVAGSHELGQDYEHVTMLQERF

REFARDTGNIGQERVDTVNHLADELINSGHSDAATIAEWKDGLNEAWADLLELIDTRTQI

LAASYELHKFYHDAKEIFGRIQDKHKKLPEELGRDQNTVETLQRMHTTFEHDIQALGTQV

RQLQEDAARLQAAYAGDKADDIQKRENEVLEAWKSLLDACESRRVRLVDTGDKFRFFSMV

RDLMLWMEDVIRQIEAQEKPRDVSSVELLMNNHQGIKAEIDARNDSFTTCIELGKSLLAR

KHYASEEIKEKLLQLTEKRKEMIDKWEDRWEWLRLILEVHQFSRDASVAEAWLLGQEPYL

SSREIGQSVDEVEKLIKRHEAFEKSAATWDERFSALERLTTLELLEVRRQQEEEERKRRP

PSPEPSTKVSEEAESQQQWDTSKGEQVSQNGLPAEQGSPRMAETVDTSEMVNGATEQRTS

SKESSPIPSPTSDRKAKTALPAQSAATLPARTQETPSAQMEGFLNRKHEWEAHNKKASSR

SWHNVYCVINNQEMGFYKDAKTAASGIPYHSEVPVSLKEAVCEVALDYKKKKHVFKLRLN

DGNEYLFQAKDDEEMNTWIQAISSAISSDKHEVSASTQSTPASSRAQTLPTSVVTITSES

SPGKREKDKEKDKEKRFSLFGKKK

>sp|Q01082|SPTB2_HUMAN 1939 KPRDVSSVEL

MTTTVATDYDNIEIQQQYSDVNNRWDVDDWDNENSSARLFERSRIKALADEREAVQKKTF

TKWVNSHLARVSCRITDLYTDLRDGRMLIKLLEVLSGERLPKPTKGRMRIHCLENVDKAL

QFLKEQRVHLENMGSHDIVDGNHRLTLGLIWTIILRFQIQDISVETEDNKEKKSAKDALL

LWCQMKTAGYPNVNIHNFTTSWRDGMAFNALIHKHRPDLIDFDKLKKSNAHYNLQNAFNL

AEQHLGLTKLLDPEDISVDHPDEKSIITYVVTYYHYFSKMKALAVEGKRIGKVLDNAIET

EKMIEKYESLASDLLEWIEQTIIILNNRKFANSLVGVQQQLQAFNTYRTVEKPPKFTEKG

NLEVLLFTIQSKMRANNQKVYMPREGKLISDINKAWERLEKAEHERELALRNELIRQEKL

EQLARRFDRKAAMRETWLSENQRLVSQDNFGFDLPAVEAATKKHEAIETDIAAYEERVQA

VVAVARELEAENYHDIKRITARKDNVIRLWEYLLELLRARRQRLEMNLGLQKIFQEMLYI

MDWMDEMKVLVLSQDYGKHLLGVEDLLQKHTLVEADIGIQAERVRGVNASAQKFATDGEG

YKPCDPQVIRDRVAHMEFCYQELCQLAAERRARLEESRRLWKFFWEMAEEEGWIREKEKI

LSSDDYGKDLTSVMRLLSKHRAFEDEMSGRSGHFEQAIKEGEDMIAEEHFGSEKIRERII

YIREQWANLEQLSAIRKKRLEEASLLHQFQADADDIDAWMLDILKIVSSSDVGHDEYSTQ

SLVKKHKDVAEEIANYRPTLDTLHEQASALPQEHAESPDVRGRLSGIEERYKEVAELTRL

RKQALQDTLALYKMFSEADACELWIDEKEQWLNNMQIPEKLEDLEVIQHRFESLEPEMNN

QASRVAVVNQIARQLMHSGHPSEKEIKAQQDKLNTRWSQFRELVDRKKDALLSALSIQNY

HLECNETKSWIREKTKVIESTQDLGNDLAGVMALQRKLTGMERDLVAIEAKLSDLQKEAE

KLESEHPDQAQAILSRLAEISDVWEEMKTTLKNREASLGEASKLQQFLRDLDDFQSWLSR

TQTAIASEDMPNTLTEAEKLLTQHENIKNEIDNYEEDYQKMRDMGEMVTQGQTDAQYMFL

RQRLQALDTGWNELHKMWENRQNLLSQSHAYQQFLRDTKQAEAFLNNQEYVLAHTEMPTT

LEGAEAAIKKQEDFMTTMDANEEKINAVVETGRRLVSDGNINSDRIQEKVDSIDDRHRKN

RETASELLMRLKDNRDLQKFLQDCQELSLWINEKMLTAQDMSYDEARNLHSKWLKHQAFM

AELASNKEWLDKIEKEGMQLISEKPETEAVVKEKLTGLHKMWEVLESTTQTKAQRLFDAN

KAELFTQSCADLDKWLHGLESQIQSDDYGKDLTSVNILLKKQQMLENQMEVRKKEIEELQ

SQAQALSQEGKSTDEVDSKRLTVQTKFMELLEPLNERKHNLLASKEIHQFNRDVEDEILW

VGERMPLATSTDHGHNLQTVQLLIKKNQTLQKEIQGHQPRIDDIFERSQNIVTDSSSLSA

EAIRQRLADLKQLWGLLIEETEKRHRRLEEAHRAQQYYFDAAEAEAWMSEQELYMMSEEK

AKDEQSAVSMLKKHQILEQAVEDYAETVHQLSKTSRALVADSHPESERISMRQSKVDKLY

AGLKDLAEERRGKLDERHRLFQLNREVDDLEQWIAEREVVAGSHELGQDYEHVTMLQERF

REFARDTGNIGQERVDTVNHLADELINSGHSDAATIAEWKDGLNEAWADLLELIDTRTQI

LAASYELHKFYHDAKEIFGRIQDKHKKLPEELGRDQNTVETLQRMHTTFEHDIQALGTQV

RQLQEDAARLQAAYAGDKADDIQKRENEVLEAWKSLLDACESRRVRLVDTGDKFRFFSMV

RDLMLWMEDVIRQIEAQEKPRDVSSVELLMNNHQGIKAEIDARNDSFTTCIELGKSLLAR

KHYASEEIKEKLLQLTEKRKEMIDKWEDRWEWLRLILEVHQFSRDASVAEAWLLGQEPYL

SSREIGQSVDEVEKLIKRHEAFEKSAATWDERFSALERLTTLELLEVRRQQEEEERKRRP

PSPEPSTKVSEEAESQQQWDTSKGEQVSQNGLPAEQGSPRMAETVDTSEMVNGATEQRTS

SKESSPIPSPTSDRKAKTALPAQSAATLPARTQETPSAQMEGFLNRKHEWEAHNKKASSR

SWHNVYCVINNQEMGFYKDAKTAASGIPYHSEVPVSLKEAVCEVALDYKKKKHVFKLRLN

DGNEYLFQAKDDEEMNTWIQAISSAISSDKHEVSASTQSTPASSRAQTLPTSVVTITSES

SPGKREKDKEKDKEKRFSLFGKKK

>sp|Q01082|SPTB2_HUMAN 474 YEERVQAVV

MTTTVATDYDNIEIQQQYSDVNNRWDVDDWDNENSSARLFERSRIKALADEREAVQKKTF

TKWVNSHLARVSCRITDLYTDLRDGRMLIKLLEVLSGERLPKPTKGRMRIHCLENVDKAL

QFLKEQRVHLENMGSHDIVDGNHRLTLGLIWTIILRFQIQDISVETEDNKEKKSAKDALL

LWCQMKTAGYPNVNIHNFTTSWRDGMAFNALIHKHRPDLIDFDKLKKSNAHYNLQNAFNL

AEQHLGLTKLLDPEDISVDHPDEKSIITYVVTYYHYFSKMKALAVEGKRIGKVLDNAIET

EKMIEKYESLASDLLEWIEQTIIILNNRKFANSLVGVQQQLQAFNTYRTVEKPPKFTEKG

NLEVLLFTIQSKMRANNQKVYMPREGKLISDINKAWERLEKAEHERELALRNELIRQEKL

EQLARRFDRKAAMRETWLSENQRLVSQDNFGFDLPAVEAATKKHEAIETDIAAYEERVQA

VVAVARELEAENYHDIKRITARKDNVIRLWEYLLELLRARRQRLEMNLGLQKIFQEMLYI

MDWMDEMKVLVLSQDYGKHLLGVEDLLQKHTLVEADIGIQAERVRGVNASAQKFATDGEG

YKPCDPQVIRDRVAHMEFCYQELCQLAAERRARLEESRRLWKFFWEMAEEEGWIREKEKI

LSSDDYGKDLTSVMRLLSKHRAFEDEMSGRSGHFEQAIKEGEDMIAEEHFGSEKIRERII

YIREQWANLEQLSAIRKKRLEEASLLHQFQADADDIDAWMLDILKIVSSSDVGHDEYSTQ

SLVKKHKDVAEEIANYRPTLDTLHEQASALPQEHAESPDVRGRLSGIEERYKEVAELTRL

RKQALQDTLALYKMFSEADACELWIDEKEQWLNNMQIPEKLEDLEVIQHRFESLEPEMNN

QASRVAVVNQIARQLMHSGHPSEKEIKAQQDKLNTRWSQFRELVDRKKDALLSALSIQNY

HLECNETKSWIREKTKVIESTQDLGNDLAGVMALQRKLTGMERDLVAIEAKLSDLQKEAE

KLESEHPDQAQAILSRLAEISDVWEEMKTTLKNREASLGEASKLQQFLRDLDDFQSWLSR

TQTAIASEDMPNTLTEAEKLLTQHENIKNEIDNYEEDYQKMRDMGEMVTQGQTDAQYMFL

RQRLQALDTGWNELHKMWENRQNLLSQSHAYQQFLRDTKQAEAFLNNQEYVLAHTEMPTT

LEGAEAAIKKQEDFMTTMDANEEKINAVVETGRRLVSDGNINSDRIQEKVDSIDDRHRKN

RETASELLMRLKDNRDLQKFLQDCQELSLWINEKMLTAQDMSYDEARNLHSKWLKHQAFM

AELASNKEWLDKIEKEGMQLISEKPETEAVVKEKLTGLHKMWEVLESTTQTKAQRLFDAN

KAELFTQSCADLDKWLHGLESQIQSDDYGKDLTSVNILLKKQQMLENQMEVRKKEIEELQ

SQAQALSQEGKSTDEVDSKRLTVQTKFMELLEPLNERKHNLLASKEIHQFNRDVEDEILW

VGERMPLATSTDHGHNLQTVQLLIKKNQTLQKEIQGHQPRIDDIFERSQNIVTDSSSLSA

EAIRQRLADLKQLWGLLIEETEKRHRRLEEAHRAQQYYFDAAEAEAWMSEQELYMMSEEK

AKDEQSAVSMLKKHQILEQAVEDYAETVHQLSKTSRALVADSHPESERISMRQSKVDKLY

AGLKDLAEERRGKLDERHRLFQLNREVDDLEQWIAEREVVAGSHELGQDYEHVTMLQERF

REFARDTGNIGQERVDTVNHLADELINSGHSDAATIAEWKDGLNEAWADLLELIDTRTQI

LAASYELHKFYHDAKEIFGRIQDKHKKLPEELGRDQNTVETLQRMHTTFEHDIQALGTQV

RQLQEDAARLQAAYAGDKADDIQKRENEVLEAWKSLLDACESRRVRLVDTGDKFRFFSMV

RDLMLWMEDVIRQIEAQEKPRDVSSVELLMNNHQGIKAEIDARNDSFTTCIELGKSLLAR

KHYASEEIKEKLLQLTEKRKEMIDKWEDRWEWLRLILEVHQFSRDASVAEAWLLGQEPYL

SSREIGQSVDEVEKLIKRHEAFEKSAATWDERFSALERLTTLELLEVRRQQEEEERKRRP

PSPEPSTKVSEEAESQQQWDTSKGEQVSQNGLPAEQGSPRMAETVDTSEMVNGATEQRTS

SKESSPIPSPTSDRKAKTALPAQSAATLPARTQETPSAQMEGFLNRKHEWEAHNKKASSR

SWHNVYCVINNQEMGFYKDAKTAASGIPYHSEVPVSLKEAVCEVALDYKKKKHVFKLRLN

DGNEYLFQAKDDEEMNTWIQAISSAISSDKHEVSASTQSTPASSRAQTLPTSVVTITSES

SPGKREKDKEKDKEKRFSLFGKKK

>sp|Q01082|SPTB2_HUMAN 2249 YHSEVPVSL

MTTTVATDYDNIEIQQQYSDVNNRWDVDDWDNENSSARLFERSRIKALADEREAVQKKTF

TKWVNSHLARVSCRITDLYTDLRDGRMLIKLLEVLSGERLPKPTKGRMRIHCLENVDKAL

QFLKEQRVHLENMGSHDIVDGNHRLTLGLIWTIILRFQIQDISVETEDNKEKKSAKDALL

LWCQMKTAGYPNVNIHNFTTSWRDGMAFNALIHKHRPDLIDFDKLKKSNAHYNLQNAFNL

AEQHLGLTKLLDPEDISVDHPDEKSIITYVVTYYHYFSKMKALAVEGKRIGKVLDNAIET

EKMIEKYESLASDLLEWIEQTIIILNNRKFANSLVGVQQQLQAFNTYRTVEKPPKFTEKG

NLEVLLFTIQSKMRANNQKVYMPREGKLISDINKAWERLEKAEHERELALRNELIRQEKL

EQLARRFDRKAAMRETWLSENQRLVSQDNFGFDLPAVEAATKKHEAIETDIAAYEERVQA

VVAVARELEAENYHDIKRITARKDNVIRLWEYLLELLRARRQRLEMNLGLQKIFQEMLYI

MDWMDEMKVLVLSQDYGKHLLGVEDLLQKHTLVEADIGIQAERVRGVNASAQKFATDGEG

YKPCDPQVIRDRVAHMEFCYQELCQLAAERRARLEESRRLWKFFWEMAEEEGWIREKEKI

LSSDDYGKDLTSVMRLLSKHRAFEDEMSGRSGHFEQAIKEGEDMIAEEHFGSEKIRERII

YIREQWANLEQLSAIRKKRLEEASLLHQFQADADDIDAWMLDILKIVSSSDVGHDEYSTQ

SLVKKHKDVAEEIANYRPTLDTLHEQASALPQEHAESPDVRGRLSGIEERYKEVAELTRL

RKQALQDTLALYKMFSEADACELWIDEKEQWLNNMQIPEKLEDLEVIQHRFESLEPEMNN

QASRVAVVNQIARQLMHSGHPSEKEIKAQQDKLNTRWSQFRELVDRKKDALLSALSIQNY

HLECNETKSWIREKTKVIESTQDLGNDLAGVMALQRKLTGMERDLVAIEAKLSDLQKEAE

KLESEHPDQAQAILSRLAEISDVWEEMKTTLKNREASLGEASKLQQFLRDLDDFQSWLSR

TQTAIASEDMPNTLTEAEKLLTQHENIKNEIDNYEEDYQKMRDMGEMVTQGQTDAQYMFL

RQRLQALDTGWNELHKMWENRQNLLSQSHAYQQFLRDTKQAEAFLNNQEYVLAHTEMPTT

LEGAEAAIKKQEDFMTTMDANEEKINAVVETGRRLVSDGNINSDRIQEKVDSIDDRHRKN

RETASELLMRLKDNRDLQKFLQDCQELSLWINEKMLTAQDMSYDEARNLHSKWLKHQAFM

AELASNKEWLDKIEKEGMQLISEKPETEAVVKEKLTGLHKMWEVLESTTQTKAQRLFDAN

KAELFTQSCADLDKWLHGLESQIQSDDYGKDLTSVNILLKKQQMLENQMEVRKKEIEELQ

SQAQALSQEGKSTDEVDSKRLTVQTKFMELLEPLNERKHNLLASKEIHQFNRDVEDEILW

VGERMPLATSTDHGHNLQTVQLLIKKNQTLQKEIQGHQPRIDDIFERSQNIVTDSSSLSA

EAIRQRLADLKQLWGLLIEETEKRHRRLEEAHRAQQYYFDAAEAEAWMSEQELYMMSEEK

AKDEQSAVSMLKKHQILEQAVEDYAETVHQLSKTSRALVADSHPESERISMRQSKVDKLY

AGLKDLAEERRGKLDERHRLFQLNREVDDLEQWIAEREVVAGSHELGQDYEHVTMLQERF

REFARDTGNIGQERVDTVNHLADELINSGHSDAATIAEWKDGLNEAWADLLELIDTRTQI

LAASYELHKFYHDAKEIFGRIQDKHKKLPEELGRDQNTVETLQRMHTTFEHDIQALGTQV

RQLQEDAARLQAAYAGDKADDIQKRENEVLEAWKSLLDACESRRVRLVDTGDKFRFFSMV

RDLMLWMEDVIRQIEAQEKPRDVSSVELLMNNHQGIKAEIDARNDSFTTCIELGKSLLAR

KHYASEEIKEKLLQLTEKRKEMIDKWEDRWEWLRLILEVHQFSRDASVAEAWLLGQEPYL

SSREIGQSVDEVEKLIKRHEAFEKSAATWDERFSALERLTTLELLEVRRQQEEEERKRRP

PSPEPSTKVSEEAESQQQWDTSKGEQVSQNGLPAEQGSPRMAETVDTSEMVNGATEQRTS

SKESSPIPSPTSDRKAKTALPAQSAATLPARTQETPSAQMEGFLNRKHEWEAHNKKASSR

SWHNVYCVINNQEMGFYKDAKTAASGIPYHSEVPVSLKEAVCEVALDYKKKKHVFKLRLN

DGNEYLFQAKDDEEMNTWIQAISSAISSDKHEVSASTQSTPASSRAQTLPTSVVTITSES

SPGKREKDKEKDKEKRFSLFGKKK

>sp|Q6P2Q9|PRP8_HUMAN 1377 SEFIDSQRVW

MAGVFPYRGPGNPVPGPLAPLPDYMSEEKLQEKARKWQQLQAKRYAEKRKFGFVDAQKED

MPPEHVRKIIRDHGDMTNRKFRHDKRVYLGALKYMPHAVLKLLENMPMPWEQIRDVPVLY

HITGAISFVNEIPWVIEPVYISQWGSMWIMMRREKRDRRHFKRMRFPPFDDEEPPLDYAD

NILDVEPLEAIQLELDPEEDAPVLDWFYDHQPLRDSRKYVNGSTYQRWQFTLPMMSTLYR

LANQLLTDLVDDNYFYLFDLKAFFTSKALNMAIPGGPKFEPLVRDINLQDEDWNEFNDIN

KIIIRQPIRTEYKIAFPYLYNNLPHHVHLTWYHTPNVVFIKTEDPDLPAFYFDPLINPIS

HRHSVKSQEPLPDDDEEFELPEFVEPFLKDTPLYTDNTANGIALLWAPRPFNLRSGRTRR

ALDIPLVKNWYREHCPAGQPVKVRVSYQKLLKYYVLNALKHRPPKAQKKRYLFRSFKATK

FFQSTKLDWVEVGLQVCRQGYNMLNLLIHRKNLNYLHLDYNFNLKPVKTLTTKERKKSRF

GNAFHLCREVLRLTKLVVDSHVQYRLGNVDAFQLADGLQYIFAHVGQLTGMYRYKYKLMR

QIRMCKDLKHLIYYRFNTGPVGKGPGCGFWAAGWRVWLFFMRGITPLLERWLGNLLARQF

EGRHSKGVAKTVTKQRVESHFDLELRAAVMHDILDMMPEGIKQNKARTILQHLSEAWRCW

KANIPWKVPGLPTPIENMILRYVKAKADWWTNTAHYNRERIRRGATVDKTVCKKNLGRLT

RLYLKAEQERQHNYLKDGPYITAEEAVAVYTTTVHWLESRRFSPIPFPPLSYKHDTKLLI

LALERLKEAYSVKSRLNQSQREELGLIEQAYDNPHEALSRIKRHLLTQRAFKEVGIEFMD

LYSHLVPVYDVEPLEKITDAYLDQYLWYEADKRRLFPPWIKPADTEPPPLLVYKWCQGIN

NLQDVWETSEGECNVMLESRFEKMYEKIDLTLLNRLLRLIVDHNIADYMTAKNNVVINYK

DMNHTNSYGIIRGLQFASFIVQYYGLVMDLLVLGLHRASEMAGPPQMPNDFLSFQDIATE

AAHPIRLFCRYIDRIHIFFRFTADEARDLIQRYLTEHPDPNNENIVGYNNKKCWPRDARM

RLMKHDVNLGRAVFWDIKNRLPRSVTTVQWENSFVSVYSKDNPNLLFNMCGFECRILPKC

RTSYEEFTHKDGVWNLQNEVTKERTAQCFLRVDDESMQRFHNRVRQILMASGSTTFTKIV

NKWNTALIGLMTYFREAVVNTQELLDLLVKCENKIQTRIKIGLNSKMPSRFPPVVFYTPK

ELGGLGMLSMGHVLIPQSDLRWSKQTDVGITHFRSGMSHEEDQLIPNLYRYIQPWESEFI

DSQRVWAEYALKRQEAIAQNRRLTLEDLEDSWDRGIPRINTLFQKDRHTLAYDKGWRVRT

DFKQYQVLKQNPFWWTHQRHDGKLWNLNNYRTDMIQALGGVEGILEHTLFKGTYFPTWEG

LFWEKASGFEESMKWKKLTNAQRSGLNQIPNRRFTLWWSPTINRANVYVGFQVQLDLTGI

FMHGKIPTLKISLIQIFRAHLWQKIHESIVMDLCQVFDQELDALEIETVQKETIHPRKSY

KMNSSCADILLFASYKWNVSRPSLLADSKDVMDSTTTQKYWIDIQLRWGDYDSHDIERYA

RAKFLDYTTDNMSIYPSPTGVLIAIDLAYNLHSAYGNWFPGSKPLIQQAMAKIMKANPAL

YVLRERIRKGLQLYSSEPTEPYLSSQNYGELFSNQIIWFVDDTNVYRVTIHKTFEGNLTT

KPINGAIFIFNPRTGQLFLKIIHTSVWAGQKRLGQLAKWKTAEEVAALIRSLPVEEQPKQ

IIVTRKGMLDPLEVHLLDFPNIVIKGSELQLPFQACLKVEKFGDLILKATEPQMVLFNLY

DDWLKTISSYTAFSRLILILRALHVNNDRAKVILKPDKTTITEPHHIWPTLTDEEWIKVE

VQLKDLILADYGKKNNVNVASLTQSEIRDIILGMEISAPSQQRQQIAEIEKQTKEQSQLT

ATQTRTVNKHGDEIITSTTSNYETQTFSSKTEWRVRAISAANLHLRTNHIYVSSDDIKET

GYTYILPKNVLKKFICISDLRAQIAGYLYGVSPPDNPQVKEIRCIVMVPQWGTHQTVHLP

GQLPQHEYLKEMEPLGWIHTQPNESPQLSPQDVTTHAKIMADNPSWDGEKTIIITCSFTP

GSCTLTAYKLTPSGYEWGRQNTDKGNNPKGYLPSHYERVQMLLSDRFLGFFMVPAQSSWN

YNFMGVRHDPNMKYELQLANPKEFYHEVHRPSHFLNFALLQEGEVYSADREDLYA

>sp|Q6P2Q9|PRP8_HUMAN 538 SRFGNAFHL

MAGVFPYRGPGNPVPGPLAPLPDYMSEEKLQEKARKWQQLQAKRYAEKRKFGFVDAQKED

MPPEHVRKIIRDHGDMTNRKFRHDKRVYLGALKYMPHAVLKLLENMPMPWEQIRDVPVLY

HITGAISFVNEIPWVIEPVYISQWGSMWIMMRREKRDRRHFKRMRFPPFDDEEPPLDYAD

NILDVEPLEAIQLELDPEEDAPVLDWFYDHQPLRDSRKYVNGSTYQRWQFTLPMMSTLYR

LANQLLTDLVDDNYFYLFDLKAFFTSKALNMAIPGGPKFEPLVRDINLQDEDWNEFNDIN

KIIIRQPIRTEYKIAFPYLYNNLPHHVHLTWYHTPNVVFIKTEDPDLPAFYFDPLINPIS

HRHSVKSQEPLPDDDEEFELPEFVEPFLKDTPLYTDNTANGIALLWAPRPFNLRSGRTRR

ALDIPLVKNWYREHCPAGQPVKVRVSYQKLLKYYVLNALKHRPPKAQKKRYLFRSFKATK

FFQSTKLDWVEVGLQVCRQGYNMLNLLIHRKNLNYLHLDYNFNLKPVKTLTTKERKKSRF

GNAFHLCREVLRLTKLVVDSHVQYRLGNVDAFQLADGLQYIFAHVGQLTGMYRYKYKLMR

QIRMCKDLKHLIYYRFNTGPVGKGPGCGFWAAGWRVWLFFMRGITPLLERWLGNLLARQF

EGRHSKGVAKTVTKQRVESHFDLELRAAVMHDILDMMPEGIKQNKARTILQHLSEAWRCW

KANIPWKVPGLPTPIENMILRYVKAKADWWTNTAHYNRERIRRGATVDKTVCKKNLGRLT

RLYLKAEQERQHNYLKDGPYITAEEAVAVYTTTVHWLESRRFSPIPFPPLSYKHDTKLLI

LALERLKEAYSVKSRLNQSQREELGLIEQAYDNPHEALSRIKRHLLTQRAFKEVGIEFMD

LYSHLVPVYDVEPLEKITDAYLDQYLWYEADKRRLFPPWIKPADTEPPPLLVYKWCQGIN

NLQDVWETSEGECNVMLESRFEKMYEKIDLTLLNRLLRLIVDHNIADYMTAKNNVVINYK

DMNHTNSYGIIRGLQFASFIVQYYGLVMDLLVLGLHRASEMAGPPQMPNDFLSFQDIATE

AAHPIRLFCRYIDRIHIFFRFTADEARDLIQRYLTEHPDPNNENIVGYNNKKCWPRDARM

RLMKHDVNLGRAVFWDIKNRLPRSVTTVQWENSFVSVYSKDNPNLLFNMCGFECRILPKC

RTSYEEFTHKDGVWNLQNEVTKERTAQCFLRVDDESMQRFHNRVRQILMASGSTTFTKIV

NKWNTALIGLMTYFREAVVNTQELLDLLVKCENKIQTRIKIGLNSKMPSRFPPVVFYTPK

ELGGLGMLSMGHVLIPQSDLRWSKQTDVGITHFRSGMSHEEDQLIPNLYRYIQPWESEFI

DSQRVWAEYALKRQEAIAQNRRLTLEDLEDSWDRGIPRINTLFQKDRHTLAYDKGWRVRT

DFKQYQVLKQNPFWWTHQRHDGKLWNLNNYRTDMIQALGGVEGILEHTLFKGTYFPTWEG

LFWEKASGFEESMKWKKLTNAQRSGLNQIPNRRFTLWWSPTINRANVYVGFQVQLDLTGI

FMHGKIPTLKISLIQIFRAHLWQKIHESIVMDLCQVFDQELDALEIETVQKETIHPRKSY

KMNSSCADILLFASYKWNVSRPSLLADSKDVMDSTTTQKYWIDIQLRWGDYDSHDIERYA

RAKFLDYTTDNMSIYPSPTGVLIAIDLAYNLHSAYGNWFPGSKPLIQQAMAKIMKANPAL

YVLRERIRKGLQLYSSEPTEPYLSSQNYGELFSNQIIWFVDDTNVYRVTIHKTFEGNLTT

KPINGAIFIFNPRTGQLFLKIIHTSVWAGQKRLGQLAKWKTAEEVAALIRSLPVEEQPKQ

IIVTRKGMLDPLEVHLLDFPNIVIKGSELQLPFQACLKVEKFGDLILKATEPQMVLFNLY

DDWLKTISSYTAFSRLILILRALHVNNDRAKVILKPDKTTITEPHHIWPTLTDEEWIKVE

VQLKDLILADYGKKNNVNVASLTQSEIRDIILGMEISAPSQQRQQIAEIEKQTKEQSQLT

ATQTRTVNKHGDEIITSTTSNYETQTFSSKTEWRVRAISAANLHLRTNHIYVSSDDIKET

GYTYILPKNVLKKFICISDLRAQIAGYLYGVSPPDNPQVKEIRCIVMVPQWGTHQTVHLP

GQLPQHEYLKEMEPLGWIHTQPNESPQLSPQDVTTHAKIMADNPSWDGEKTIIITCSFTP

GSCTLTAYKLTPSGYEWGRQNTDKGNNPKGYLPSHYERVQMLLSDRFLGFFMVPAQSSWN

YNFMGVRHDPNMKYELQLANPKEFYHEVHRPSHFLNFALLQEGEVYSADREDLYA

>sp|Q6P2Q9|PRP8_HUMAN 1079 TEAAHPIRLF

MAGVFPYRGPGNPVPGPLAPLPDYMSEEKLQEKARKWQQLQAKRYAEKRKFGFVDAQKED

MPPEHVRKIIRDHGDMTNRKFRHDKRVYLGALKYMPHAVLKLLENMPMPWEQIRDVPVLY

HITGAISFVNEIPWVIEPVYISQWGSMWIMMRREKRDRRHFKRMRFPPFDDEEPPLDYAD

NILDVEPLEAIQLELDPEEDAPVLDWFYDHQPLRDSRKYVNGSTYQRWQFTLPMMSTLYR

LANQLLTDLVDDNYFYLFDLKAFFTSKALNMAIPGGPKFEPLVRDINLQDEDWNEFNDIN

KIIIRQPIRTEYKIAFPYLYNNLPHHVHLTWYHTPNVVFIKTEDPDLPAFYFDPLINPIS

HRHSVKSQEPLPDDDEEFELPEFVEPFLKDTPLYTDNTANGIALLWAPRPFNLRSGRTRR

ALDIPLVKNWYREHCPAGQPVKVRVSYQKLLKYYVLNALKHRPPKAQKKRYLFRSFKATK

FFQSTKLDWVEVGLQVCRQGYNMLNLLIHRKNLNYLHLDYNFNLKPVKTLTTKERKKSRF

GNAFHLCREVLRLTKLVVDSHVQYRLGNVDAFQLADGLQYIFAHVGQLTGMYRYKYKLMR

QIRMCKDLKHLIYYRFNTGPVGKGPGCGFWAAGWRVWLFFMRGITPLLERWLGNLLARQF

EGRHSKGVAKTVTKQRVESHFDLELRAAVMHDILDMMPEGIKQNKARTILQHLSEAWRCW

KANIPWKVPGLPTPIENMILRYVKAKADWWTNTAHYNRERIRRGATVDKTVCKKNLGRLT

RLYLKAEQERQHNYLKDGPYITAEEAVAVYTTTVHWLESRRFSPIPFPPLSYKHDTKLLI

LALERLKEAYSVKSRLNQSQREELGLIEQAYDNPHEALSRIKRHLLTQRAFKEVGIEFMD

LYSHLVPVYDVEPLEKITDAYLDQYLWYEADKRRLFPPWIKPADTEPPPLLVYKWCQGIN

NLQDVWETSEGECNVMLESRFEKMYEKIDLTLLNRLLRLIVDHNIADYMTAKNNVVINYK

DMNHTNSYGIIRGLQFASFIVQYYGLVMDLLVLGLHRASEMAGPPQMPNDFLSFQDIATE

AAHPIRLFCRYIDRIHIFFRFTADEARDLIQRYLTEHPDPNNENIVGYNNKKCWPRDARM

RLMKHDVNLGRAVFWDIKNRLPRSVTTVQWENSFVSVYSKDNPNLLFNMCGFECRILPKC

RTSYEEFTHKDGVWNLQNEVTKERTAQCFLRVDDESMQRFHNRVRQILMASGSTTFTKIV

NKWNTALIGLMTYFREAVVNTQELLDLLVKCENKIQTRIKIGLNSKMPSRFPPVVFYTPK

ELGGLGMLSMGHVLIPQSDLRWSKQTDVGITHFRSGMSHEEDQLIPNLYRYIQPWESEFI

DSQRVWAEYALKRQEAIAQNRRLTLEDLEDSWDRGIPRINTLFQKDRHTLAYDKGWRVRT

DFKQYQVLKQNPFWWTHQRHDGKLWNLNNYRTDMIQALGGVEGILEHTLFKGTYFPTWEG

LFWEKASGFEESMKWKKLTNAQRSGLNQIPNRRFTLWWSPTINRANVYVGFQVQLDLTGI

FMHGKIPTLKISLIQIFRAHLWQKIHESIVMDLCQVFDQELDALEIETVQKETIHPRKSY

KMNSSCADILLFASYKWNVSRPSLLADSKDVMDSTTTQKYWIDIQLRWGDYDSHDIERYA

RAKFLDYTTDNMSIYPSPTGVLIAIDLAYNLHSAYGNWFPGSKPLIQQAMAKIMKANPAL

YVLRERIRKGLQLYSSEPTEPYLSSQNYGELFSNQIIWFVDDTNVYRVTIHKTFEGNLTT

KPINGAIFIFNPRTGQLFLKIIHTSVWAGQKRLGQLAKWKTAEEVAALIRSLPVEEQPKQ

IIVTRKGMLDPLEVHLLDFPNIVIKGSELQLPFQACLKVEKFGDLILKATEPQMVLFNLY

DDWLKTISSYTAFSRLILILRALHVNNDRAKVILKPDKTTITEPHHIWPTLTDEEWIKVE

VQLKDLILADYGKKNNVNVASLTQSEIRDIILGMEISAPSQQRQQIAEIEKQTKEQSQLT

ATQTRTVNKHGDEIITSTTSNYETQTFSSKTEWRVRAISAANLHLRTNHIYVSSDDIKET

GYTYILPKNVLKKFICISDLRAQIAGYLYGVSPPDNPQVKEIRCIVMVPQWGTHQTVHLP

GQLPQHEYLKEMEPLGWIHTQPNESPQLSPQDVTTHAKIMADNPSWDGEKTIIITCSFTP

GSCTLTAYKLTPSGYEWGRQNTDKGNNPKGYLPSHYERVQMLLSDRFLGFFMVPAQSSWN

YNFMGVRHDPNMKYELQLANPKEFYHEVHRPSHFLNFALLQEGEVYSADREDLYA

>sp|Q9NTG1|PKDRE_HUMAN 701 KDFLPAGYLL

MRPGPALLLLGVGLSLSVGRLPLPPVPRGAQAAVSGAPGGLLRGAPGLGVRGGRALLSLR

PSAVRAGGAVLSGRGSLCFPHGGTGRRWYCLDLRVLLSAQRLPWPAAPALALVDLQLSAR

GGRLSLTWSVRLPRSPGRLAWAFRLRLLGPGAARPASPAARVSPRSAAPGPRPQQGFVAR

TECPTDGPARVMLQAVNSSSHRAVESSVSCQINACVIQRVRINTDQKGAPVRLSMQAEAT

INASVQLDCPAARAIAQYWQVFSVPAVGQAPDWTQPLDLPQLEIRNSPLFIHIPNNSLQW

GVYVFNFTVSITTGNPKMPEVKDSDAVYVWIVRSSLQAVMLGDANITANFTEQLILDGST

SSDPDADSPLQGLQFFWYCTTDPRNYGGDRIILGSKEVCHPEQANLKWPWASGPVLTLLP

ETLKGDHVYFFRMVIRKDSRTAFSDKRVHVLQGPKAIAHITCIENCERNFIVSDRFSLFL

NCTNCASRDFYKWSILSSSGGEMLFDWMGETVTGRNGAYLSIKAFAFRHFLEAEFSISLY

LACWSGVTSVFRHSFIINHGPQIGECKINPAKGIALITKFVVQCSNFRDKHVPLTYKIIV

SDLHSVGEISSVKENTLGTILYLGPQSTVPPSFLPVGMLASQYGLKIYAQVYDSLGAFSQ

VTLHATAQAPTDKNSSKTVLNQLLSFTVGPSSLLSTLIQKKDFLPAGYLLYIVASVLNNM

KTELPLRDDRVNLRKHLIDQSFLLPVSTLVEIGQVVMTITKLTQKPSEFTWDAQKRATMR

VWQANQALQEYQQKDKRFRSEQIEIVSTGILMSLSNILKMTSPHQVVKDPFYVIESLSDT

ILANKVPGNKTTSMRTPNFNMYVKKVEKWGINQLFRNEKHCRNCFYPTLNVSSVPGLSAN

GPISTMFCDFTNDLFPWLNDQENTSVEVSGFRMTGVADNGSVLEITPDVAEVYLVRKNLT

FAAFNLTVGPNSEVDGSLKKTTGGFSFQVDSTVLREVLVHIVTEVMVLFTVLVYTGSQIT

PTALVATFLVPHDIPPFASQSALFDPACTVKKARVVCLPVSLLQLIAQHSHSPHCTVSIV

LQAPRFVMKLNDKLVRISIFSVQCLDMYGIQSEWREGYCILGEKTSWYEVHCICKNVVRA

RRQLGTIGLTGIHLHTHYVMAKVIVIPNPVDLRLNIIKSLHQNPVTLFTVLFIILLYVGL

AFWALYRDEMDQHLRGHVIVLPDNDPYDNLCYLVTIFTGSRWGSGTRANVFVQLRGTVST

SDVHCLSHPHFTTLYRGSINTFLLTTKSDLGDIHSIRVWHNNEGRSPSWYLSRIKVENLF

SRHIWLFICQKWLSVDTTLDRTFHVTHPDERLTRKDFFFIDVSSNLRKNHMWFSIFASVV

AKTFNRLQRLSCCLAMLLSSLLCNIMFFNLNRQEQTESRERKYMRSMMIGIESVLITIPV

QLLITFLFTCSQRKPQADLKEVSPQKHPLMSEASEHWEEYLRKWHAYETAKVHPREVAKP

ASKGKPRLPKASPKATSKPKHRHRKAQIKTPETLGPNTNSNNNIEDDQDVHSEQHPSQKD

LQQLKKKPRIVLPWWCVYVAWFLVFATSSISSFFIVFYGLTYGYDKSIEWLFASFCSFCQ

SVLLVQPSKIILLSGFRTNKPKYCKNLSWSTKYKYTEIRLDGMRMHPEEMQRIHDQIVRI

RGTRMYQPLTEDEIRIFKRKKRIKRRALLFLSYILTHFIFLALLLILIVLLRHTDCFYYN

QFIRDRFSMDLATVTKLEDIYRWLNSVLLPLLHNDLNPTFLPESSSKILGLPLMRQVRAK

SSEKMCLPAEKFVQNSIRREIHCHPKYGIDPEDTKNYSGFWNEVDKQAIDESTNGFTYKP

QGTQWLYYSYGLLHTYGSGGYALYFFPEQQRFNSTLRLKELQESNWLDEKTWAVVLELTT

FNPDINLFCSISVIFEVSQLGVVNTSISLHSFSLADFDRKASAEIYLYVAILIFFLAYVV

DEGCIIMQERASYVRSVYNLLNFALKCIFTVLIVLFLRKHFLATGIIRFYLSNPEDFIPF

HAVSQVDHIMRIILGFLLFLTILKTLRYSRFFYDVRLAQRAIQAALPGICHMAFVVSVYF

FVYMAFGYLVFGQHEWNYSNLIHSTQTVFSYCVSAFQNTEFSNNRILGVLFLSSFMLVMI

CVLINLFQAVILSAYEEMKQPVYEEPSDEVEAMTYLCRKLRTMFSFLTSQSKAKDEPEFF

IDMLYGQPEKNSHRYLGLKTRNINGKKMVYLVV

>sp|Q9C0F0|ASXL3_HUMAN 2030 ALPPPPPPP

MKDKRKKKDRTWAEAARLALEKHPNSPMTAKQILEVIQKEGLKETSGTSPLACLNAMLHT

NTRIGDGTFFKIPGKSGLYALKKEESSCPADGTLDLVCESELDGTDMAEANAHGEENGVC

SKQVTDEASSTRDSSLTNTAVQSKLVSSFQQHTKKALKQALRQQQKRRNGVSMMVNKTVP

RVVLTPLKVSDEQSDSPSGSESKNGEADSSDKEMKHGQKSPTGKQTSQHLKRLKKSGLGH

LKWTKAEDIDIETPGSILVNTNLRALINKHTFASLPQHFQQYLLLLLPEVDRQMGSDGIL

RLSTSALNNEFFAYAAQGWKQRLAEGEFTPEMQLRIRQEIEKEKKTEPWKEKFFERFYGE

KLGMSREESVKLTTGPNNAGAQSSSSCGTSGLPVSAQTALAEQQPKSMKSPASPEPGFCA

TLCPMVEIPPKDIMAELESEDILIPEESVIQEEIAEEVETSICECQDENHKTIPEFSEEA

ESLTNSHEEPQIAPPEDNLESCVMMNDVLETLPHIEVKIEGKSESPQEEMTVVIDQLEVC

DSLIPSTSSMTHVSDTEHKESETAVETSTPKIKTGSSSLEGQFPNEGIAIDMELQSDPEE

QLSENACISETSFSSESPEGACTSLPSPGGETQSTSEESCTPASLETTFCSEVSSTENTD

KYNQRNSTDENFHASLMSEISPISTSPEISEASLMSNLPLTSEASPVSNLPLTSETSPMS

DLPLTSETSSVSSMLLTSETTFVSSLPLPSETSPISNSSINERMAHQQRKSPSVSEEPLS

PQKDESSATAKPLGENLTSQQKNLSNTPEPIIMSSSSIAPEAFPSEDLHNKTLSQQTCKS

HVDTEKPYPASIPELASTEMIKVKNHSVLQRTEKKVLPSPLELSVFSEGTDNKGNELPSA

KLQDKQYISSVDKAPFSEGSRNKTHKQGSTQSRLETSHTSKSSEPSKSPDGIRNESRDSE

ISKRKTAEQHSFGICKEKRARIEDDQSTRNISSSSPPEKEQPPREEPRVPPLKIQLSKIG

PPFIIKSQPVSKPESRASTSTSVSGGRNTGARTLADIKARAQQARAQREAAAAAAVAAAA

SIVSGAMGSPGEGGKTRTLAHIKEQTKAKLFAKHQARAHLFQTSKETRLPPPLSSKEGPP

NLEVSSTPETKMEGSTGVIIVNPNCRSPSNKSAHLRETTTVLQQSLNPSKLPETATDLSV

HSSDENIPVSHLSEKIVSSTSSENSSVPMLFNKNSVPVSVCSTAISGAIKEHPFVSSVDK

SSVLMSVDSANTTISACNISMLKTIQGTDTPCIAIIPKCIESTPISATTEGSSISSSMDD

KQLLISSSSASNLVSTQYTSVPTPSIGNNLPNLSTSSVLIPPMGINNRFPSEKIAIPGSE

EQATVSMGTTVRAALSCSDSVAVTDSLVAHPTVAMFTGNMLTINSYDSPPKLSAESLDKN

SGPRNRADNSGKPQQPPGGFAPAAINRSIPCKVIVDHSTTLTSSLSLTVSVESSEASLDL

QGRPVRTEASVQPVACPQVSVISRPEPVANEGIDHSSTFIAASAAKQDSKTLPATCTSLR

ELPLVPDKLNEPTAPSHNFAEQARGPAPFKSEADTTCSNQYNPSNRICWNDDGMRSTGQP

LVTHSGSSKQKEYLEQSCPKAIKTEHANYLNVSELHPRNLVTNVALPVKSELHEADKGFR

MDTEDFPGPELPPPAAEGASSVQQTQNMKASTSSPMEEAISLATDALKRVPGAGSSGCRL

SSVEANNPLVTQLLQGNLPLEKVLPQPRLGAKLEINRLPLPLQTTSVGKTAPERNVEIPP

SSPNPDGKGYLAGTLAPLQMRKRENHPKKRVARTVGEHTQVKCEPGKLLVEPDVKGVPCV

ISSGISQLGHSQPFKQEWLNKHSMQNRIVHSPEVKQQKRLLPSCSFQQNLFHVDKNGGFH

TDAGTSHRQQFYQMPVAARGPIPTAALLQASSKTPVGCNAFAFNRHLEQKGLGEVSLSSA

PHQLRLANMLSPNMPMKEGDEVGGTAHTMPNKALVHPPPPPPPPPPPPLALPPPPPPPPP

LPPPLPNAEVPSDQKQPPVTMETTKRLSWPQSTGICSNIKSEPLSFEEGLSSSCELGMKQ

VSYDQNEMKEQLKAFALKSADFSSYLLSEPQKPFTQLAAQKMQVQQQQQLCGNYPTIHFG

STSFKRAASAIEKSIGILGSGSNPATGLSGQNAQMPVQNFADSSNADELELKCSCRLKAM

IVCKGCGAFCHDDCIGPSKLCVACLVVR

>sp|P27708|PYR1_HUMAN 445 GLADKVYFL

MAALVLEDGSVLRGQPFGAAVSTAGEVVFQTGMVGYPEALTDPSYKAQILVLTYPLIGNY

GIPPDEMDEFGLCKWFESSGIHVAALVVGECCPTPSHWSATRTLHEWLQQHGIPGLQGVD

TRELTKKLREQGSLLGKLVQNGTEPSSLPFLDPNARPLVPEVSIKTPRVFNTGGAPRILA

LDCGLKYNQIRCLCQRGAEVTVVPWDHALDSQEYEGLFLSNGPGDPASYPSVVSTLSRVL

SEPNPRPVFGICLGHQLLALAIGAKTYKMRYGNRGHNQPCLLVGSGRCFLTSQNHGFAVE

TDSLPADWAPLFTNANDGSNEGIVHNSLPFFSVQFHPEHQAGPSDMELLFDIFLETVKEA

TAGNPGGQTVRERLTERLCPPGIPTPGSGLPPPRKVLILGSGGLSIGQAGEFDYSGSQAI

KALKEENIQTLLINPNIATVQTSQGLADKVYFLPITPHYVTQVIRNERPDGVLLTFGGQT

ALNCGVELTKAGVLARYGVRVLGTPVETIELTEDRRAFAARMAEIGEHVAPSEAANSLEQ

AQAAAERLGYPVLVRAAFALGGLGSGFASNREELSALVAPAFAHTSQVLVDKSLKGWKEI

EYEVVRDAYGNCVTVCNMENLDPLGIHTGESIVVAPSQTLNDREYQLLRQTAIKVTQHLG

IVGECNVQYALNPESEQYYIIEVNARLSRSSALASKATGYPLAYVAAKLALGIPLPELRN

SVTGGTAAFEPSVDYCVVKIPRWDLSKFLRVSTKIGSCMKSVGEVMGIGRSFEEAFQKAL

RMVDENCVGFDHTVKPVSDMELETPTDKRIFVVAAALWAGYSVDRLYELTRIDRWFLHRM

KRIIAHAQLLEQHRGQPLPPDLLQQAKCLGFSDKQIALAVLSTELAVRKLRQELGICPAV

KQIDTVAAEWPAQTNYLYLTYWGTTHDLTFRTPHVLVLGSGVYRIGSSVEFDWCAVGCIQ

QLRKMGYKTIMVNYNPETVSTDYDMCDRLYFDEISFEVVMDIYELENPEGVILSMGGQLP

NNMAMALHRQQCRVLGTSPEAIDSAENRFKFSRLLDTIGISQPQWRELSDLESARQFCQT

VGYPCVVRPSYVLSGAAMNVAYTDGDLERFLSSAAAVSKEHPVVISKFIQEAKEIDVDAV

ASDGVVAAIAISEHVENAGVHSGDATLVTPPQDITAKTLERIKAIVHAVGQELQVTGPFN

LQLIAKDDQLKVIECNVRVSRSFPFVSKTLGVDLVALATRVIMGEEVEPVGLMTGSGVVG

VKVPQFSFSRLAGADVVLGVEMTSTGEVAGFGESRCEAYLKAMLSTGFKIPKKNILLTIG

SYKNKSELLPTVRLLESLGYSLYASLGTADFYTEHGVKVTAVDWHFEEAVDGECPPQRSI

LEQLAEKNFELVINLSMRGAGGRRLSSFVTKGYRTRRLAADFSVPLIIDIKCTKLFVEAL

GQIGPAPPLKVHVDCMTSQKLVRLPGLIDVHVHLREPGGTHKEDFASGTAAALAGGITMV

CAMPNTRPPIIDAPALALAQKLAEAGARCDFALFLGASSENAGTLGTVAGSAAGLKLYLN

ETFSELRLDSVVQWMEHFETWPSHLPIVAHAEQQTVAAVLMVAQLTQRSVHICHVARKEE

ILLIKAAKARGLPVTCEVAPHHLFLSHDDLERLGPGKGEVRPELGSRQDVEALWENMAVI

DCFASDHAPHTLEEKCGSRPPPGFPGLETMLPLLLTAVSEGRLSLDDLLQRLHHNPRRIF

HLPPQEDTYVEVDLEHEWTIPSHMPFSKAHWTPFEGQKVKGTVRRVVLRGEVAYIDGQVL

VPPGYGQDVRKWPQGAVPQLPPSAPATSEMTTTPERPRRGIPGLPDGRFHLPPRIHRASD

PGLPAEEPKEKSSRKVAEPELMGTPDGTCYPPPPVPRQASPQNLGTPGLLHPQTSPLLHS

LVGQHILSVQQFTKDQMSHLFNVAHTLRMMVQKERSLDILKGKVMASMFYEVSTRTSSSF

AAAMARLGGAVLSFSEATSSVQKGESLADSVQTMSCYADVVVLRHPQPGAVELAAKHCRR

PVINAGDGVGEHPTQALLDIFTIREELGTVNGMTITMVGDLKHGRTVHSLACLLTQYRVS

LRYVAPPSLRMPPTVRAFVASRGTKQEEFESIEEALPDTDVLYMTRIQKERFGSTQEYEA

CFGQFILTPHIMTRAKKKMVVMHPMPRVNEISVEVDSDPRAAYFRQAENGMYIRMALLAT

VLGRF

>sp|Q92673|SORL_HUMAN 245 QEHVKSFSW

MATRSSRRESRLPFLFTLVALLPPGALCEVWTQRLHGGSAPLPQDRGFLVVQGDPRELRL

WARGDARGASRADEKPLRRKRSAALQPEPIKVYGQVSLNDSHNQMVVHWAGEKSNVIVAL

ARDSLALARPKSSDVYVSYDYGKSFKKISDKLNFGLGNRSEAVIAQFYHSPADNKRYIFA

DAYAQYLWITFDFCNTLQGFSIPFRAADLLLHSKASNLLLGFDRSHPNKQLWKSDDFGQT

WIMIQEHVKSFSWGIDPYDKPNTIYIERHEPSGYSTVFRSTDFFQSRENQEVILEEVRDF

QLRDKYMFATKVVHLLGSEQQSSVQLWVSFGRKPMRAAQFVTRHPINEYYIADASEDQVF

VCVSHSNNRTNLYISEAEGLKFSLSLENVLYYSPGGAGSDTLVRYFANEPFADFHRVEGL

QGVYIATLINGSMNEENMRSVITFDKGGTWEFLQAPAFTGYGEKINCELSQGCSLHLAQR

LSQLLNLQLRRMPILSKESAPGLIIATGSVGKNLASKTNVYISSSAGARWREALPGPHYY

TWGDHGGIITAIAQGMETNELKYSTNEGETWKTFIFSEKPVFVYGLLTEPGEKSTVFTIF

GSNKENVHSWLILQVNATDALGVPCTENDYKLWSPSDERGNECLLGHKTVFKRRTPHATC

FNGEDFDRPVVVSNCSCTREDYECDFGFKMSEDLSLEVCVPDPEFSGKSYSPPVPCPVGS

TYRRTRGYRKISGDTCSGGDVEARLEGELVPCPLAEENEFILYAVRKSIYRYDLASGATE

QLPLTGLRAAVALDFDYEHNCLYWSDLALDVIQRLCLNGSTGQEVIINSGLETVEALAFE

PLSQLLYWVDAGFKKIEVANPDGDFRLTIVNSSVLDRPRALVLVPQEGVMFWTDWGDLKP

GIYRSNMDGSAAYHLVSEDVKWPNGISVDDQWIYWTDAYLECIERITFSGQQRSVILDNL

PHPYAIAVFKNEIYWDDWSQLSIFRASKYSGSQMEILANQLTGLMDMKIFYKGKNTGSNA

CVPRPCSLLCLPKANNSRSCRCPEDVSSSVLPSGDLMCDCPQGYQLKNNTCVKEENTCLR

NQYRCSNGNCINSIWWCDFDNDCGDMSDERNCPTTICDLDTQFRCQESGTCIPLSYKCDL

EDDCGDNSDESHCEMHQCRSDEYNCSSGMCIRSSWVCDGDNDCRDWSDEANCTAIYHTCE

ASNFQCRNGHCIPQRWACDGDTDCQDGSDEDPVNCEKKCNGFRCPNGTCIPSSKHCDGLR

DCSDGSDEQHCEPLCTHFMDFVCKNRQQCLFHSMVCDGIIQCRDGSDEDAAFAGCSQDPE

FHKVCDEFGFQCQNGVCISLIWKCDGMDDCGDYSDEANCENPTEAPNCSRYFQFRCENGH

CIPNRWKCDRENDCGDWSDEKDCGDSHILPFSTPGPSTCLPNYYRCSSGTCVMDTWVCDG

YRDCADGSDEEACPLLANVTAASTPTQLGRCDRFEFECHQPKTCIPNWKRCDGHQDCQDG

RDEANCPTHSTLTCMSREFQCEDGEACIVLSERCDGFLDCSDESDEKACSDELTVYKVQN

LQWTADFSGDVTLTWMRPKKMPSASCVYNVYYRVVGESIWKTLETHSNKTNTVLKVLKPD

TTYQVKVQVQCLSKAHNTNDFVTLRTPEGLPDAPRNLQLSLPREAEGVIVGHWAPPIHTH

GLIREYIVEYSRSGSKMWASQRAASNFTEIKNLLVNTLYTVRVAAVTSRGIGNWSDSKSI

TTIKGKVIPPPDIHIDSYGENYLSFTLTMESDIKVNGYVVNLFWAFDTHKQERRTLNFRG

SILSHKVGNLTAHTSYEISAWAKTDLGDSPLAFEHVMTRGVRPPAPSLKAKAINQTAVEC

TWTGPRNVVYGIFYATSFLDLYRNPKSLTTSLHNKTVIVSKDEQYLFLVRVVVPYQGPSS

DYVVVKMIPDSRLPPRHLHVVHTGKTSVVIKWESPYDSPDQDLLYAIAVKDLIRKTDRSY

KVKSRNSTVEYTLNKLEPGGKYHIIVQLGNMSKDSSIKITTVSLSAPDALKIITENDHVL

LFWKSLALKEKHFNESRGYEIHMFDSAMNITAYLGNTTDNFFKISNLKMGHNYTFTVQAR

CLFGNQICGEPAILLYDELGSGADASATQAARSTDVAAVVVPILFLILLSLGVGFAILYT

KHRRLQSSFTAFANSHYSSRLGSAIFSSGDDLGEDDEDAPMITGFSDDVPMVIA

>sp|P24821|TENA_HUMAN 1823 IATVDSYVI

MGAMTQLLAGVFLAFLALATEGGVLKKVIRHKRQSGVNATLPEENQPVVFNHVYNIKLPV

GSQCSVDLESASGEKDLAPPSEPSESFQEHTVDGENQIVFTHRINIPRRACGCAAAPDVK

ELLSRLEELENLVSSLREQCTAGAGCCLQPATGRLDTRPFCSGRGNFSTEGCGCVCEPGW

KGPNCSEPECPGNCHLRGRCIDGQCICDDGFTGEDCSQLACPSDCNDQGKCVNGVCICFE

GYAGADCSREICPVPCSEEHGTCVDGLCVCHDGFAGDDCNKPLCLNNCYNRGRCVENECV

CDEGFTGEDCSELICPNDCFDRGRCINGTCYCEEGFTGEDCGKPTCPHACHTQGRCEEGQ

CVCDEGFAGLDCSEKRCPADCHNRGRCVDGRCECDDGFTGADCGELKCPNGCSGHGRCVN

GQCVCDEGYTGEDCSQLRCPNDCHSRGRCVEGKCVCEQGFKGYDCSDMSCPNDCHQHGRC

VNGMCVCDDGYTGEDCRDRQCPRDCSNRGLCVDGQCVCEDGFTGPDCAELSCPNDCHGQG

RCVNGQCVCHEGFMGKDCKEQRCPSDCHGQGRCVDGQCICHEGFTGLDCGQHSCPSDCNN

LGQCVSGRCICNEGYSGEDCSEVSPPKDLVVTEVTEETVNLAWDNEMRVTEYLVVYTPTH

EGGLEMQFRVPGDQTSTIIQELEPGVEYFIRVFAILENKKSIPVSARVATYLPAPEGLKF

KSIKETSVEVEWDPLDIAFETWEIIFRNMNKEDEGEITKSLRRPETSYRQTGLAPGQEYE

ISLHIVKNNTRGPGLKRVTTTRLDAPSQIEVKDVTDTTALITWFKPLAEIDGIELTYGIK

DVPGDRTTIDLTEDENQYSIGNLKPDTEYEVSLISRRGDMSSNPAKETFTTGLDAPRNLR

RVSQTDNSITLEWRNGKAAIDSYRIKYAPISGGDHAEVDVPKSQQATTKTTLTGLRPGTE

YGIGVSAVKEDKESNPATINAATELDTPKDLQVSETAETSLTLLWKTPLAKFDRYRLNYS

LPTGQWVGVQLPRNTTSYVLRGLEPGQEYNVLLTAEKGRHKSKPARVKASTEQAPELENL

TVTEVGWDGLRLNWTAADQAYEHFIIQVQEANKVEAARNLTVPGSLRAVDIPGLKAATPY

TVSIYGVIQGYRTPVLSAEASTGETPNLGEVVVAEVGWDALKLNWTAPEGAYEYFFIQVQ

EADTVEAAQNLTVPGGLRSTDLPGLKAATHYTITIRGVTQDFSTTPLSVEVLTEEVPDMG

NLTVTEVSWDALRLNWTTPDGTYDQFTIQVQEADQVEEAHNLTVPGSLRSMEIPGLRAGT

PYTVTLHGEVRGHSTRPLAVEVVTEDLPQLGDLAVSEVGWDGLRLNWTAADNAYEHFVIQ

VQEVNKVEAAQNLTLPGSLRAVDIPGLEAATPYRVSIYGVIRGYRTPVLSAEASTAKEPE

IGNLNVSDITPESFNLSWMATDGIFETFTIEIIDSNRLLETVEYNISGAERTAHISGLPP

STDFIVYLSGLAPSIRTKTISATATTEALPLLENLTISDINPYGFTVSWMASENAFDSFL

VTVVDSGKLLDPQEFTLSGTQRKLELRGLITGIGYEVMVSGFTQGHQTKPLRAEIVTEAE

PEVDNLLVSDATPDGFRLSWTADEGVFDNFVLKIRDTKKQSEPLEITLLAPERTRDLTGL

REATEYEIELYGISKGRRSQTVSAIATTAMGSPKEVIFSDITENSATVSWRAPTAQVESF

RITYVPITGGTPSMVTVDGTKTQTRLVKLIPGVEYLVSIIAMKGFEESEPVSGSFTTALD

GPSGLVTANITDSEALARWQPAIATVDSYVISYTGEKVPEITRTVSGNTVEYALTDLEPA

TEYTLRIFAEKGPQKSSTITAKFTTDLDSPRDLTATEVQSETALLTWRPPRASVTGYLLV

YESVDGTVKEVIVGPDTTSYSLADLSPSTHYTAKIQALNGPLRSNMIQTIFTTIGLLYPF

PKDCSQAMLNGDTTSGLYTIYLNGDKAQALEVFCDMTSDGGGWIVFLRRKNGRENFYQNW

KAYAAGFGDRREEFWLGLDNLNKITAQGQYELRVDLRDHGETAFAVYDKFSVGDAKTRYK

LKVEGYSGTAGDSMAYHNGRSFSTFDKDTDSAITNCALSYKGAFWYRNCHRVNLMGRYGD

NNHSQGVNWFHWKGHEHSIQFAEMKLRPSNFRNLEGRRKRA

>sp|Q93074|MED12_HUMAN 1143 LLLEDLIRC

MAAFGILSYEHRPLKRPRPRLGPPDVYPQDPKQKEDELTALNVKQGFNNQPAVSGDEHGS

AKNVSFNPAKISSNFSSIIAEKLRCNTLPDTGRRKPQVNQKDNFWLVTARSQSAINTWFT

DLAGTKPLTQLAKKVPIFSKKEEVFGYLAKYTVPVMRAAWLIKMTCAYYAAISETKVKKR

HVDPFMEWTQIITKYLWEQLQKMAEYYRPGPAGSGGCGSTIGPLPHDVEVAIRQWDYTEK

LAMFMFQDGMLDRHEFLTWVLECFEKIRPGEDELLKLLLPLLLRYSGEFVQSAYLSRRLA

YFCTRRLALQLDGVSSHSSHVISAQSTSTLPTTPAPQPPTSSTPSTPFSDLLMCPQHRPL

VFGLSCILQTILLCCPSALVWHYSLTDSRIKTGSPLDHLPIAPSNLPMPEGNSAFTQQVR

AKLREIEQQIKERGQAVEVRWSFDKCQEATAGFTIGRVLHTLEVLDSHSFERSDFSNSLD

SLCNRIFGLGPSKDGHEISSDDDAVVSLLCEWAVSCKRSGRHRAMVVAKLLEKRQAEIEA

ERCGESEAADEKGSIASGSLSAPSAPIFQDVLLQFLDTQAPMLTDPRSESERVEFFNLVL

LFCELIRHDVFSHNMYTCTLISRGDLAFGAPGPRPPSPFDDPADDPEHKEAEGSSSSKLE

DPGLSESMDIDPSSSVLFEDMEKPDFSLFSPTMPCEGKGSPSPEKPDVEKEVKPPPKEKI

EGTLGVLYDQPRHVQYATHFPIPQEESCSHECNQRLVVLFGVGKQRDDARHAIKKITKDI

LKVLNRKGTAETDQLAPIVPLNPGDLTFLGGEDGQKRRRNRPEAFPTAEDIFAKFQHLSH

YDQHQVTAQVSRNVLEQITSFALGMSYHLPLVQHVQFIFDLMEYSLSISGLIDFAIQLLN

ELSVVEAELLLKSSDLVGSYTTSLCLCIVAVLRHYHACLILNQDQMAQVFEGLCGVVKHG

MNRSDGSSAERCILAYLYDLYTSCSHLKNKFGELFSDFCSKVKNTIYCNVEPSESNMRWA

PEFMIDTLENPAAHTFTYTGLGKSLSENPANRYSFVCNALMHVCVGHHDPDRVNDIAILC

AELTGYCKSLSAEWLGVLKALCCSSNNGTCGFNDLLCNVDVSDLSFHDSLATFVAILIAR

QCLLLEDLIRCAAIPSLLNAACSEQDSEPGARLTCRILLHLFKTPQLNPCQSDGNKPTVG

IRSSCDRHLLAASQNRIVDGAVFAVLKAVFVLGDAELKGSGFTVTGGTEELPEEEGGGGS

GGRRQGGRNISVETASLDVYAKYVLRSICQQEWVGERCLKSLCEDSNDLQDPVLSSAQAQ

RLMQLICYPHRLLDNEDGENPQRQRIKRILQNLDQWTMRQSSLELQLMIKQTPNNEMNSL

LENIAKATIEVFQRSAETGSSSGSTASNMPSSSKTKPVLSSLERSGVWLVAPLIAKLPTS

VQGHVLKAAGEELEKGQHLGSSSRKERDRQKQKSMSLLSQQPFLSLVLTCLKGQDEQREG

LLTSLYSQVHQIVNNWRDDQYLDDCKPKQLMHEALKLRLNLVGGMFDTVQRSTQQTTEWA

MLLLEIIISGTVDMQSNNELFTTVLDMLSVLINGTLAADMSSISQGSMEENKRAYMNLAK

KLQKELGERQSDSLEKVRQLLPLPKQTRDVITCEPQGSLIDTKGNKIAGFDSIFKKEGLQ

VSTKQKISPWDLFEGLKPSAPLSWGWFGTVRVDRRVARGEEQQRLLLYHTHLRPRPRAYY

LEPLPLPPEDEEPPAPTLLEPEKKAPEPPKTDKPGAAPPSTEERKKKSTKGKKRSQPATK

TEDYGMGPGRSGPYGVTVPPDLLHHPNPGSITHLNYRQGSIGLYTQNQPLPAGGPRVDPY

RPVRLPMQKLPTRPTYPGVLPTTMTGVMGLEPSSYKTSVYRQQQPAVPQGQRLRQQLQQS

QGMLGQSSVHQMTPSSSYGLQTSQGYTPYVSHVGLQQHTGPAGTMVPPSYSSQPYQSTHP

STNPTLVDPTRHLQQRPSGYVHQQAPTYGHGLTSTQRFSHQTLQQTPMISTMTPMSAQGV

QAGVRSTAILPEQQQQQQQQQQQQQQQQQQQQQQQQQQYHIRQQQQQQILRQQQQQQQQQ

QQQQQQQQQQQQQQQQQHQQQQQQQAAPPQPQPQSQPQFQRQGLQQTQQQQQTAALVRQL

QQQLSNTQPQPSTNIFGRY

>sp|P48634|BAT2_HUMAN 167 REEFPTLQA

MSDRSGPTAKGKDGKKYSSLNLFDTYKGKSLEIQKPAVAPRHGLQSLGKVAIARRMPPPA

NLPSLKAENKGNDPNVSLVPKDGTGWASKQEQSDPKSSDASTAQPPESQPLPASQTPASN

QPKRPPAAPENTPLVPSGVKSWAQASVTHGAHGDGGRASSLLSRFSREEFPTLQAAGDQD

KAAKERESAEQSSGPGPSLRPQNSTTWRDGGGRGPDELEGPDSKLHHGHDPRGGLQPSGP

PQFPPYRGMMPPFMYPPYLPFPPPYGPQGPYRYPTPDGPSRFPRVAGPRGSGPPMRLVEP

VGRPSILKEDNLKEFDQLDQENDDGWAGAHEEVDYTEKLKFSDEEDGRDSDEEGAEGHRD

SQSASGEERPPEADGKKGNSPNSEPPTPKTAWAETSRPPETEPGPPAPKPPLPPPHRGPA

GNWGPPGDYPDRGGPPCKPPAPEDEDEAWRQRRKQSSSEISLAVERARRRREEEERRMQE

ERRAACAEKLKRLDEKFGAPDKRLKAEPAAPPAAPSTPAPPPAVPKELPAPPAPPPASAP

TPEKEPEEPAQAPPAQSTPTPGVAAAPTLVSGGGSTSSTSSGSFEASPVEPQLPSKEGPE

PPEEVPPPTTPPVPKVEPKGDGIGPTRQPPSQGLGYPKYQKSLPPRFQRQQQEQLLKQQQ

QHQWQQHQQGSAPPTPVPPSPPQPVTLGAVPAPQAPPPPPKALYPGALGRPPPMPPMNFD

PRWMMIPPYVDPRLLQGRPPLDFYPPGVHPSGLVPRERSDSGGSSSEPFDRHAPAMLRER

GTPPVDPKLAWVGDVFTATPAEPRPLTSPLRQAADEDDKGMRSETPPVPPPPPYLASYPG

FPENGAPGPPISRFPLEEPGPRPLPWPPGSDEVAKIQTPPPKKEPPKEETAQLTGPEAGR

KPARGVGSGGQGPPPPRRESRTETRWGPRPGSSRRGIPPEEPGAPPRRAGPIKKPPPPTK

VEELPPKPLEQGDETPKPPKPDPLKITKGKLGGPKETPPNGNLSPAPRLRRDYSYERVGP

TSCRGRGRGEYFARGRGFRGTYGGRGRGARSREFRSYREFRGDDGRGGGTGGPNHPPAPR

GRTASETRSEGSEYEEIPKRRRQRGSETGSETHESDLAPSDKEAPTPKEGTLTQVPLAPP

PPGAPPSPAPARFTARGGRVFTPRGVPSRRGRGGGRPPPQVCPGWSPPAKSLAPKKPPTG

PLPPSKEPLKEKLIPGPLSPVARGGSNGGSNVGMEDGERPRRRRHGRAQQQDKPPRFRRL

KQERENAARGSEGKPSLTLPASAPGPEEALTTVTVAPAPRRAAAKSPDLSNQNSDQANEE

WETASESSDFTSERRGDKEAPPPVLLTPKAVGTPGGGGGGAVPGISAMSRGDLSQRAKDL

SKRSFSSQRPGMERQNRRPGPGGKAGSSGSSSGGGGGGPGGRTGPGRGDKRSWPSPKNRS

RPPEERPPGLPLPPPPPSSSAVFRLDQVIHSNPAGIQQALAQLSSRQGSVTAPGGHPRHK

PGPPQAPQGPSPRPPTRYEPQRVNSGLSSDPHFEEPGPMVRGVGGTPRDSAGVSPFPPKR

RERPPRKPELLQEESLPPPHSSGFLGSKPEGPGPQAESRDTGTEALTPHIWNRLHTATSR

KSYRPSSMEPWMEPLSPFEDVAGTEMSQSDSGVDLSGDSQVSSGPCSQRSSPDGGLKGAA

EGPPKRPGGSSPLNAVPCEGPPGSEPPRRPPPAPHDGDRKELPREQPLPPGPIGTERSQR

TDRGTEPGPIRPSHRPGPPVQFGTSDKDSDLRLVVGDSLKAEKELTASVTEAIPVSRDWE

LLPSAAASAEPQSKNLDSGHCVPEPSSSGQRLYPEVFYGSAGPSSSQISGGAMDSQLHPN

SGGFRPGTPSLHPYRSQPLYLPPGPAPPSALLSGVALKGQFLDFSTMQATELGKLPAGGV

LYPPPSFLYSPAFCPSPLPDTSLLQVRQDLPSPSDFYSTPLQPGGQSGFLPSGAPAQQML

LPMVDSQLPVVNFGSLPPAPPPAPPPLSLLPVGPALQPPSLAVRPPPAPATRVLPSPARP

FPASLGRAELHPVELKPFQDYQKLSSNLGGPGSSRTPPTGRSFSGLNSRLKATPSTYSGV

FRTQRVDLYQQASPPDALRWIPKPWERTGPPPREGPSRRAEEPGSRGDKEPGLPPPR

>sp|O75643|U520_HUMAN 1248 DEHLITFF

MADVTARSLQYEYKANSNLVLQADRSLIDRTRRDEPTGEVLSLVGKLEGTRMGDKAQRTK

PQMQEERRAKRRKRDEDRHDINKMKGYTLLSEGIDEMVGIIYKPKTKETRETYEVLLSFI

QAALGDQPRDILCGAADEVLAVLKNEKLRDKERRKEIDLLLGQTDDTRYHVLVNLGKKIT

DYGGDKEIQNMDDNIDETYGVNVQFESDEEEGDEDVYGEVREEASDDDMEGDEAVVRCTL

SANLVASGELMSSKKKDLHPRDIDAFWLQRQLSRFYDDAIVSQKKADEVLEILKTASDDR

ECENQLVLLLGFNTFDFIKVLRQHRMMILYCTLLASAQSEAEKERIMGKMEADPELSKFL

YQLHETEKEDLIREERSRRERVRQSRMDTDLETMDLDQGGEALAPRQVLDLEDLVFTQGS

HFMANKRCQLPDGSFRRQRKGYEEVHVPALKPKPFGSEEQLLPVEKLPKYAQAGFEGFKT

LNRIQSKLYRAALETDENLLLCAPTGAGKTNVALMCMLREIGKHINMDGTINVDDFKIIY

IAPMRSLVQEMVGSFGKRLATYGITVAELTGDHQLCKEEISATQIIVCTPEKWDIITRKG

GERTYTQLVRLIILDEIHLLHDDRGPVLEALVARAIRNIEMTQEDVRLIGLSATLPNYED

VATFLRVDPAKGLFYFDNSFRPVPLEQTYVGITEKKAIKRFQIMNEIVYEKIMEHAGKNQ

VLVFVHSRKETGKTARAIRDMCLEKDTLGLFLREGSASTEVLRTEAEQCKNLELKDLLPY

GFAIHHAGMTRVDRTLVEDLFADKHIQVLVSTATLAWGVNLPAHTVIIKGTQVYSPEKGR

WTELGALDILQMLGRAGRPQYDTKGEGILITSHGELQYYLSLLNQQLPIESQMVSKLPDM

LNAEIVLGNVQNAKDAVNWLGYAYLYIRMLRSPTLYGISHDDLKGDPLLDQRRLDLVHTA

ALMLDKNNLVKYDKKTGNFQVTELGRIASHYYITNDTVQTYNQLLKPTLSEIELFRVFSL

SSEFKNITVREEEKLELQKLLERVPIPVKESIEEPSAKINVLLQAFISQLKLEGFALMAD

MVYVTQSAGRLMRAIFEIVLNRGWAQLTDKTLNLCKMIDKRMWQSMCPLRQFRKLPEEVV

KKIEKKNFPFERLYDLNHNEIGELIRMPKMGKTIHKYVHLFPKLELSVHLQPITRSTLKV

ELTITPDFQWDEKVHGSSEAFWILVEDVDSEVILHHEYFLLKAKYAQDEHLITFFVPVFE

PLPPQYFIRVVSDRWLSCETQLPVSFRHLILPEKYPPPTELLDLQPLPVSALRNSAFESL

YQDKFPFFNPIQTQVFNTVYNSDDNVFVGAPTGSGKTICAEFAILRMLLQSSEGRCVYIT

PMEALAEQVYMDWYEKFQDRLNKKVVLLTGETSTDLKLLGKGNIIISTPEKWDILSRRWK

QRKNVQNINLFVVDEVHLIGGENGPVLEVICSRMRYISSQIERPIRIVALSSSLSNAKDV

AHWLGCSATSTFNFHPNVRPVPLELHIQGFNISHTQTRLLSMAKPVYHAITKHSPKKPVI

VFVPSRKQTRLTAIDILTTCAADIQRQRFLHCTEKDLIPYLEKLSDSTLKETLLNGVGYL

HEGLSPMERRLVEQLFSSGAIQVVVASRSLCWGMNVAAHLVIIMDTQYYNGKIHAYVDYP

IYDVLQMVGHANRPLQDDEGRCVIMCQGSKKDFFKKFLYEPLPVESHLDHCMHDHFNAEI

VTKTIENKQDAVDYLTWTFLYRRMTQNPNYYNLQGISHRHLSDHLSELVEQTLSDLEQSK

CISIEDEMDVAPLNLGMIAAYYYINYTTIELFSMSLNAKTKVRGLIEIISNAAEYENIPI

RHHEDNLLRQLAQKVPHKLNNPKFNDPHVKTNLLLQAHLSRMQLSAELQSDTEEILSKAI

RLIQACVDVLSSNGWLSPALAAMELAQMVTQAMWSKDSYLKQLPHFTSEHIKRCTDKGVE

SVFDIMEMEDEERNALLQLTDSQIADVARFCNRYPNIELSYEVVDKDSIRSGGPVVVLVQ

LEREEEVTGPVIAPLFPQKREEGWWVVIGDAKSNSLISIKRLTLQQKAKVKLDFVAPATG

AHNYTLYFMSDAYMGCDQEYKFSVDVKEAETDSDSD

>sp|O75643|U520_HUMAN 687 QTYVGITEK

MADVTARSLQYEYKANSNLVLQADRSLIDRTRRDEPTGEVLSLVGKLEGTRMGDKAQRTK

PQMQEERRAKRRKRDEDRHDINKMKGYTLLSEGIDEMVGIIYKPKTKETRETYEVLLSFI

QAALGDQPRDILCGAADEVLAVLKNEKLRDKERRKEIDLLLGQTDDTRYHVLVNLGKKIT

DYGGDKEIQNMDDNIDETYGVNVQFESDEEEGDEDVYGEVREEASDDDMEGDEAVVRCTL

SANLVASGELMSSKKKDLHPRDIDAFWLQRQLSRFYDDAIVSQKKADEVLEILKTASDDR

ECENQLVLLLGFNTFDFIKVLRQHRMMILYCTLLASAQSEAEKERIMGKMEADPELSKFL

YQLHETEKEDLIREERSRRERVRQSRMDTDLETMDLDQGGEALAPRQVLDLEDLVFTQGS

HFMANKRCQLPDGSFRRQRKGYEEVHVPALKPKPFGSEEQLLPVEKLPKYAQAGFEGFKT

LNRIQSKLYRAALETDENLLLCAPTGAGKTNVALMCMLREIGKHINMDGTINVDDFKIIY

IAPMRSLVQEMVGSFGKRLATYGITVAELTGDHQLCKEEISATQIIVCTPEKWDIITRKG

GERTYTQLVRLIILDEIHLLHDDRGPVLEALVARAIRNIEMTQEDVRLIGLSATLPNYED

VATFLRVDPAKGLFYFDNSFRPVPLEQTYVGITEKKAIKRFQIMNEIVYEKIMEHAGKNQ

VLVFVHSRKETGKTARAIRDMCLEKDTLGLFLREGSASTEVLRTEAEQCKNLELKDLLPY

GFAIHHAGMTRVDRTLVEDLFADKHIQVLVSTATLAWGVNLPAHTVIIKGTQVYSPEKGR

WTELGALDILQMLGRAGRPQYDTKGEGILITSHGELQYYLSLLNQQLPIESQMVSKLPDM

LNAEIVLGNVQNAKDAVNWLGYAYLYIRMLRSPTLYGISHDDLKGDPLLDQRRLDLVHTA

ALMLDKNNLVKYDKKTGNFQVTELGRIASHYYITNDTVQTYNQLLKPTLSEIELFRVFSL

SSEFKNITVREEEKLELQKLLERVPIPVKESIEEPSAKINVLLQAFISQLKLEGFALMAD

MVYVTQSAGRLMRAIFEIVLNRGWAQLTDKTLNLCKMIDKRMWQSMCPLRQFRKLPEEVV

KKIEKKNFPFERLYDLNHNEIGELIRMPKMGKTIHKYVHLFPKLELSVHLQPITRSTLKV

ELTITPDFQWDEKVHGSSEAFWILVEDVDSEVILHHEYFLLKAKYAQDEHLITFFVPVFE

PLPPQYFIRVVSDRWLSCETQLPVSFRHLILPEKYPPPTELLDLQPLPVSALRNSAFESL

YQDKFPFFNPIQTQVFNTVYNSDDNVFVGAPTGSGKTICAEFAILRMLLQSSEGRCVYIT

PMEALAEQVYMDWYEKFQDRLNKKVVLLTGETSTDLKLLGKGNIIISTPEKWDILSRRWK

QRKNVQNINLFVVDEVHLIGGENGPVLEVICSRMRYISSQIERPIRIVALSSSLSNAKDV

AHWLGCSATSTFNFHPNVRPVPLELHIQGFNISHTQTRLLSMAKPVYHAITKHSPKKPVI

VFVPSRKQTRLTAIDILTTCAADIQRQRFLHCTEKDLIPYLEKLSDSTLKETLLNGVGYL

HEGLSPMERRLVEQLFSSGAIQVVVASRSLCWGMNVAAHLVIIMDTQYYNGKIHAYVDYP

IYDVLQMVGHANRPLQDDEGRCVIMCQGSKKDFFKKFLYEPLPVESHLDHCMHDHFNAEI

VTKTIENKQDAVDYLTWTFLYRRMTQNPNYYNLQGISHRHLSDHLSELVEQTLSDLEQSK

CISIEDEMDVAPLNLGMIAAYYYINYTTIELFSMSLNAKTKVRGLIEIISNAAEYENIPI

RHHEDNLLRQLAQKVPHKLNNPKFNDPHVKTNLLLQAHLSRMQLSAELQSDTEEILSKAI

RLIQACVDVLSSNGWLSPALAAMELAQMVTQAMWSKDSYLKQLPHFTSEHIKRCTDKGVE

SVFDIMEMEDEERNALLQLTDSQIADVARFCNRYPNIELSYEVVDKDSIRSGGPVVVLVQ

LEREEEVTGPVIAPLFPQKREEGWWVVIGDAKSNSLISIKRLTLQQKAKVKLDFVAPATG

AHNYTLYFMSDAYMGCDQEYKFSVDVKEAETDSDSD

>sp|O75643|U520_HUMAN 1010 SEIELFRVF

MADVTARSLQYEYKANSNLVLQADRSLIDRTRRDEPTGEVLSLVGKLEGTRMGDKAQRTK

PQMQEERRAKRRKRDEDRHDINKMKGYTLLSEGIDEMVGIIYKPKTKETRETYEVLLSFI

QAALGDQPRDILCGAADEVLAVLKNEKLRDKERRKEIDLLLGQTDDTRYHVLVNLGKKIT

DYGGDKEIQNMDDNIDETYGVNVQFESDEEEGDEDVYGEVREEASDDDMEGDEAVVRCTL

SANLVASGELMSSKKKDLHPRDIDAFWLQRQLSRFYDDAIVSQKKADEVLEILKTASDDR

ECENQLVLLLGFNTFDFIKVLRQHRMMILYCTLLASAQSEAEKERIMGKMEADPELSKFL

YQLHETEKEDLIREERSRRERVRQSRMDTDLETMDLDQGGEALAPRQVLDLEDLVFTQGS

HFMANKRCQLPDGSFRRQRKGYEEVHVPALKPKPFGSEEQLLPVEKLPKYAQAGFEGFKT

LNRIQSKLYRAALETDENLLLCAPTGAGKTNVALMCMLREIGKHINMDGTINVDDFKIIY

IAPMRSLVQEMVGSFGKRLATYGITVAELTGDHQLCKEEISATQIIVCTPEKWDIITRKG

GERTYTQLVRLIILDEIHLLHDDRGPVLEALVARAIRNIEMTQEDVRLIGLSATLPNYED

VATFLRVDPAKGLFYFDNSFRPVPLEQTYVGITEKKAIKRFQIMNEIVYEKIMEHAGKNQ

VLVFVHSRKETGKTARAIRDMCLEKDTLGLFLREGSASTEVLRTEAEQCKNLELKDLLPY

GFAIHHAGMTRVDRTLVEDLFADKHIQVLVSTATLAWGVNLPAHTVIIKGTQVYSPEKGR

WTELGALDILQMLGRAGRPQYDTKGEGILITSHGELQYYLSLLNQQLPIESQMVSKLPDM

LNAEIVLGNVQNAKDAVNWLGYAYLYIRMLRSPTLYGISHDDLKGDPLLDQRRLDLVHTA

ALMLDKNNLVKYDKKTGNFQVTELGRIASHYYITNDTVQTYNQLLKPTLSEIELFRVFSL

SSEFKNITVREEEKLELQKLLERVPIPVKESIEEPSAKINVLLQAFISQLKLEGFALMAD

MVYVTQSAGRLMRAIFEIVLNRGWAQLTDKTLNLCKMIDKRMWQSMCPLRQFRKLPEEVV

KKIEKKNFPFERLYDLNHNEIGELIRMPKMGKTIHKYVHLFPKLELSVHLQPITRSTLKV

ELTITPDFQWDEKVHGSSEAFWILVEDVDSEVILHHEYFLLKAKYAQDEHLITFFVPVFE

PLPPQYFIRVVSDRWLSCETQLPVSFRHLILPEKYPPPTELLDLQPLPVSALRNSAFESL

YQDKFPFFNPIQTQVFNTVYNSDDNVFVGAPTGSGKTICAEFAILRMLLQSSEGRCVYIT

PMEALAEQVYMDWYEKFQDRLNKKVVLLTGETSTDLKLLGKGNIIISTPEKWDILSRRWK

QRKNVQNINLFVVDEVHLIGGENGPVLEVICSRMRYISSQIERPIRIVALSSSLSNAKDV

AHWLGCSATSTFNFHPNVRPVPLELHIQGFNISHTQTRLLSMAKPVYHAITKHSPKKPVI

VFVPSRKQTRLTAIDILTTCAADIQRQRFLHCTEKDLIPYLEKLSDSTLKETLLNGVGYL

HEGLSPMERRLVEQLFSSGAIQVVVASRSLCWGMNVAAHLVIIMDTQYYNGKIHAYVDYP

IYDVLQMVGHANRPLQDDEGRCVIMCQGSKKDFFKKFLYEPLPVESHLDHCMHDHFNAEI

VTKTIENKQDAVDYLTWTFLYRRMTQNPNYYNLQGISHRHLSDHLSELVEQTLSDLEQSK

CISIEDEMDVAPLNLGMIAAYYYINYTTIELFSMSLNAKTKVRGLIEIISNAAEYENIPI

RHHEDNLLRQLAQKVPHKLNNPKFNDPHVKTNLLLQAHLSRMQLSAELQSDTEEILSKAI

RLIQACVDVLSSNGWLSPALAAMELAQMVTQAMWSKDSYLKQLPHFTSEHIKRCTDKGVE

SVFDIMEMEDEERNALLQLTDSQIADVARFCNRYPNIELSYEVVDKDSIRSGGPVVVLVQ

LEREEEVTGPVIAPLFPQKREEGWWVVIGDAKSNSLISIKRLTLQQKAKVKLDFVAPATG

AHNYTLYFMSDAYMGCDQEYKFSVDVKEAETDSDSD

>sp|O75643|U520_HUMAN 1230 SEVILHHEY

MADVTARSLQYEYKANSNLVLQADRSLIDRTRRDEPTGEVLSLVGKLEGTRMGDKAQRTK

PQMQEERRAKRRKRDEDRHDINKMKGYTLLSEGIDEMVGIIYKPKTKETRETYEVLLSFI

QAALGDQPRDILCGAADEVLAVLKNEKLRDKERRKEIDLLLGQTDDTRYHVLVNLGKKIT

DYGGDKEIQNMDDNIDETYGVNVQFESDEEEGDEDVYGEVREEASDDDMEGDEAVVRCTL

SANLVASGELMSSKKKDLHPRDIDAFWLQRQLSRFYDDAIVSQKKADEVLEILKTASDDR

ECENQLVLLLGFNTFDFIKVLRQHRMMILYCTLLASAQSEAEKERIMGKMEADPELSKFL

YQLHETEKEDLIREERSRRERVRQSRMDTDLETMDLDQGGEALAPRQVLDLEDLVFTQGS

HFMANKRCQLPDGSFRRQRKGYEEVHVPALKPKPFGSEEQLLPVEKLPKYAQAGFEGFKT

LNRIQSKLYRAALETDENLLLCAPTGAGKTNVALMCMLREIGKHINMDGTINVDDFKIIY

IAPMRSLVQEMVGSFGKRLATYGITVAELTGDHQLCKEEISATQIIVCTPEKWDIITRKG

GERTYTQLVRLIILDEIHLLHDDRGPVLEALVARAIRNIEMTQEDVRLIGLSATLPNYED

VATFLRVDPAKGLFYFDNSFRPVPLEQTYVGITEKKAIKRFQIMNEIVYEKIMEHAGKNQ

VLVFVHSRKETGKTARAIRDMCLEKDTLGLFLREGSASTEVLRTEAEQCKNLELKDLLPY

GFAIHHAGMTRVDRTLVEDLFADKHIQVLVSTATLAWGVNLPAHTVIIKGTQVYSPEKGR

WTELGALDILQMLGRAGRPQYDTKGEGILITSHGELQYYLSLLNQQLPIESQMVSKLPDM

LNAEIVLGNVQNAKDAVNWLGYAYLYIRMLRSPTLYGISHDDLKGDPLLDQRRLDLVHTA

ALMLDKNNLVKYDKKTGNFQVTELGRIASHYYITNDTVQTYNQLLKPTLSEIELFRVFSL

SSEFKNITVREEEKLELQKLLERVPIPVKESIEEPSAKINVLLQAFISQLKLEGFALMAD

MVYVTQSAGRLMRAIFEIVLNRGWAQLTDKTLNLCKMIDKRMWQSMCPLRQFRKLPEEVV

KKIEKKNFPFERLYDLNHNEIGELIRMPKMGKTIHKYVHLFPKLELSVHLQPITRSTLKV

ELTITPDFQWDEKVHGSSEAFWILVEDVDSEVILHHEYFLLKAKYAQDEHLITFFVPVFE

PLPPQYFIRVVSDRWLSCETQLPVSFRHLILPEKYPPPTELLDLQPLPVSALRNSAFESL

YQDKFPFFNPIQTQVFNTVYNSDDNVFVGAPTGSGKTICAEFAILRMLLQSSEGRCVYIT

PMEALAEQVYMDWYEKFQDRLNKKVVLLTGETSTDLKLLGKGNIIISTPEKWDILSRRWK

QRKNVQNINLFVVDEVHLIGGENGPVLEVICSRMRYISSQIERPIRIVALSSSLSNAKDV

AHWLGCSATSTFNFHPNVRPVPLELHIQGFNISHTQTRLLSMAKPVYHAITKHSPKKPVI

VFVPSRKQTRLTAIDILTTCAADIQRQRFLHCTEKDLIPYLEKLSDSTLKETLLNGVGYL

HEGLSPMERRLVEQLFSSGAIQVVVASRSLCWGMNVAAHLVIIMDTQYYNGKIHAYVDYP

IYDVLQMVGHANRPLQDDEGRCVIMCQGSKKDFFKKFLYEPLPVESHLDHCMHDHFNAEI

VTKTIENKQDAVDYLTWTFLYRRMTQNPNYYNLQGISHRHLSDHLSELVEQTLSDLEQSK

CISIEDEMDVAPLNLGMIAAYYYINYTTIELFSMSLNAKTKVRGLIEIISNAAEYENIPI

RHHEDNLLRQLAQKVPHKLNNPKFNDPHVKTNLLLQAHLSRMQLSAELQSDTEEILSKAI

RLIQACVDVLSSNGWLSPALAAMELAQMVTQAMWSKDSYLKQLPHFTSEHIKRCTDKGVE

SVFDIMEMEDEERNALLQLTDSQIADVARFCNRYPNIELSYEVVDKDSIRSGGPVVVLVQ

LEREEEVTGPVIAPLFPQKREEGWWVVIGDAKSNSLISIKRLTLQQKAKVKLDFVAPATG

AHNYTLYFMSDAYMGCDQEYKFSVDVKEAETDSDSD

>sp|O75643|U520_HUMAN 1510 STFNFHPNVR

MADVTARSLQYEYKANSNLVLQADRSLIDRTRRDEPTGEVLSLVGKLEGTRMGDKAQRTK

PQMQEERRAKRRKRDEDRHDINKMKGYTLLSEGIDEMVGIIYKPKTKETRETYEVLLSFI

QAALGDQPRDILCGAADEVLAVLKNEKLRDKERRKEIDLLLGQTDDTRYHVLVNLGKKIT

DYGGDKEIQNMDDNIDETYGVNVQFESDEEEGDEDVYGEVREEASDDDMEGDEAVVRCTL

SANLVASGELMSSKKKDLHPRDIDAFWLQRQLSRFYDDAIVSQKKADEVLEILKTASDDR

ECENQLVLLLGFNTFDFIKVLRQHRMMILYCTLLASAQSEAEKERIMGKMEADPELSKFL

YQLHETEKEDLIREERSRRERVRQSRMDTDLETMDLDQGGEALAPRQVLDLEDLVFTQGS

HFMANKRCQLPDGSFRRQRKGYEEVHVPALKPKPFGSEEQLLPVEKLPKYAQAGFEGFKT

LNRIQSKLYRAALETDENLLLCAPTGAGKTNVALMCMLREIGKHINMDGTINVDDFKIIY

IAPMRSLVQEMVGSFGKRLATYGITVAELTGDHQLCKEEISATQIIVCTPEKWDIITRKG

GERTYTQLVRLIILDEIHLLHDDRGPVLEALVARAIRNIEMTQEDVRLIGLSATLPNYED

VATFLRVDPAKGLFYFDNSFRPVPLEQTYVGITEKKAIKRFQIMNEIVYEKIMEHAGKNQ

VLVFVHSRKETGKTARAIRDMCLEKDTLGLFLREGSASTEVLRTEAEQCKNLELKDLLPY

GFAIHHAGMTRVDRTLVEDLFADKHIQVLVSTATLAWGVNLPAHTVIIKGTQVYSPEKGR

WTELGALDILQMLGRAGRPQYDTKGEGILITSHGELQYYLSLLNQQLPIESQMVSKLPDM

LNAEIVLGNVQNAKDAVNWLGYAYLYIRMLRSPTLYGISHDDLKGDPLLDQRRLDLVHTA

ALMLDKNNLVKYDKKTGNFQVTELGRIASHYYITNDTVQTYNQLLKPTLSEIELFRVFSL

SSEFKNITVREEEKLELQKLLERVPIPVKESIEEPSAKINVLLQAFISQLKLEGFALMAD

MVYVTQSAGRLMRAIFEIVLNRGWAQLTDKTLNLCKMIDKRMWQSMCPLRQFRKLPEEVV

KKIEKKNFPFERLYDLNHNEIGELIRMPKMGKTIHKYVHLFPKLELSVHLQPITRSTLKV

ELTITPDFQWDEKVHGSSEAFWILVEDVDSEVILHHEYFLLKAKYAQDEHLITFFVPVFE

PLPPQYFIRVVSDRWLSCETQLPVSFRHLILPEKYPPPTELLDLQPLPVSALRNSAFESL

YQDKFPFFNPIQTQVFNTVYNSDDNVFVGAPTGSGKTICAEFAILRMLLQSSEGRCVYIT

PMEALAEQVYMDWYEKFQDRLNKKVVLLTGETSTDLKLLGKGNIIISTPEKWDILSRRWK

QRKNVQNINLFVVDEVHLIGGENGPVLEVICSRMRYISSQIERPIRIVALSSSLSNAKDV

AHWLGCSATSTFNFHPNVRPVPLELHIQGFNISHTQTRLLSMAKPVYHAITKHSPKKPVI

VFVPSRKQTRLTAIDILTTCAADIQRQRFLHCTEKDLIPYLEKLSDSTLKETLLNGVGYL

HEGLSPMERRLVEQLFSSGAIQVVVASRSLCWGMNVAAHLVIIMDTQYYNGKIHAYVDYP

IYDVLQMVGHANRPLQDDEGRCVIMCQGSKKDFFKKFLYEPLPVESHLDHCMHDHFNAEI

VTKTIENKQDAVDYLTWTFLYRRMTQNPNYYNLQGISHRHLSDHLSELVEQTLSDLEQSK

CISIEDEMDVAPLNLGMIAAYYYINYTTIELFSMSLNAKTKVRGLIEIISNAAEYENIPI

RHHEDNLLRQLAQKVPHKLNNPKFNDPHVKTNLLLQAHLSRMQLSAELQSDTEEILSKAI

RLIQACVDVLSSNGWLSPALAAMELAQMVTQAMWSKDSYLKQLPHFTSEHIKRCTDKGVE

SVFDIMEMEDEERNALLQLTDSQIADVARFCNRYPNIELSYEVVDKDSIRSGGPVVVLVQ

LEREEEVTGPVIAPLFPQKREEGWWVVIGDAKSNSLISIKRLTLQQKAKVKLDFVAPATG

AHNYTLYFMSDAYMGCDQEYKFSVDVKEAETDSDSD

>sp|Q12789|TF3C1_HUMAN 434 AEESDLSRQY

MDALESLLDEVALEGLDGLCLPALWSRLETRVPPFPLPLEPCTQEFLWRALATHPGISFY

EEPRERPDLQLQDRYEEIDLETGILESRRDPVALEDVYPIHMILENKDGIQGSCRYFKER

KNITNDIRTKSLQPRCTMVEPFDRWGKKLIIGSLPAHAVQALDSPGGGSRPEAARLLLLH

PGTARPVQVQGELQRDLHTTAFKVDAGKLHYHRKILNKNGLITMQSHVIRLPTGAQQHSI

LLLLNRFHVDRRSKYDILMEKLSVMLSTRTNHIETLGKLREELGLCERTFKRLYQYMLNA

GLAKVVSLRLQEIHPECGPCKTKKGTDVMVRCLKLLKEFKRNDHDDDEDEEVISKTVPPV

DIVFERDMLTQTYDLIERRGTKGISQAEIRVAMNVGKLEARMLCRLLQRFKVVKGFMEDE

GRQRTTKYISCVFAEESDLSRQYQREKARSELLTTVSLASMQEESLLPEGEDTFLSESDS

EEERSSSKRRGRGSQKDTRASANLRPKTQPHHSTPTKGGWKVVNLHPLKKQPPSFPGAAE

ERACQSLASRDSLLDTSSVSEPNVSFVSHCADSNSGDIAVIEEVRMENPKESSSSLKTGR

HSSGQDKPHETYRLLKRRNLIIEAVTNLRLIESLFTIQKMIMDQEKQEGVSTKCCKKSIV

RLVRNLSEEGLLRLYRTTVIQDGIKKKVDLVVHPSMDQNDPLVRSAIEQVRFRISNSSTA

NRVKTSQPPVPQGEAEEDSQGKEGPSGSGDSQLSASSRSESGRMKKSDNKMGITPLRNYH

PIVVPGLGRSLGFLPKMPRLRVVHMFLWYLIYGHPASNTVEKPSFISERRTIKQESGRAG

VRPSSSGSAWEACSEAPSKGSQDGVTWEAEVELATETVYVDDASWMRYIPPIPVHRDFGF

GWALVSDILLCLPLSIFIQIVQVSYKVDNLEEFLNDPLKKHTLIRFLPRPIRQQLLYKRR

YIFSVVENLQRLCYMGVLQFGPTEKFQDKDQVFIFLKKNAVIVDTTICDPHYNLARSSRP

FERRLYVLNSMQDVENYWFDLQCVCLNTPLGVVRCPRVRKNSSTDQGSDEEGSLQKEQES

AMDKHNLERKCAMLEYTTGSREVVDEGLIPGDGLGAAGLDSSFYGHLKRNWIWTSYIINQ

AKKENTAAENGLTVRLQTFLSKRPMPLSARGNSRLNIWGEARVGSELCAGWEEQFEVDRE

PSLDRNRRVRGGKSQKRKRLKKDPGKKIKRKKKGEFPGEKSKRLRYHDEADQSALQRMTR

LRVTWSMQEDGLLVLCRIASNVLNTKVKGPFVTWQVVRDILHATFEESLDKTSHSVGRRA

RYIVKNPQAYLNYKVCLAEVYQDKALVGDFMNRRGDYDDPKVCANEFKEFVEKLKEKFSS

ALRNSNLEIPDTLQELFARYRVLAIGDEKDQTRKEDELNSVDDIHFLVLQNLIQSTLALS

DSQMKSYQSFQTFRLYREYKDHVLVKAFMECQKRSLVNRRRVNHTLGPKKNRALPFVPMS

YQLSQTYYRIFTWRFPSTICTESFQFLDRMRAAGKLDQPDRFSFKDQDNNEPTNDMVAFS

LDGPGGNCVAVLTLFSLGLISVDVRIPEQIIVVDSSMVENEVIKSLGKDGSLEDDEDEED

DLDEGVGGKRRSMEVKPAQASHTNYLLMRGYYSPGIVSTRNLNPNDSIVVNSCQMKFQLR

CTPVPARLRPAAAPLEELTMGTSCLPDTFTKLINPQENTCSLEEFVLQLELSGYSPEDLT

AALEILEAIIATGCFGIDKEELRRRFSALEKAGGGRTRTFADCIQALLEQHQVLEVGGNT

ARLVAMGSAWPWLLHSVRLKDREDADIQREDPQARPLEGSSSEDSPPEGQAPPSHSPRGT

KRRASWASENGETDAEGTQMTPAKRPALQDSNLAPSLGPGAEDGAEAQAPSPPPALEDTA

AAGAAQEDQEGVGEFSSPGQEQLSGQAQPPEGSEDPRGFTESFGAANISQAARERDCESV

CFIGRPWRVVDGHLNLPVCKGMMEAMLYHIMTRPGIPESSLLRHYQGVLQPVAVLELLQG

LESLGCIRKRWLRKPRPVSLFSTPVVEEVEVPSSLDESPMAFYEPTLDCTLRLGRVFPHE

VNWNKWIHL

>sp|Q8NF50|DOCK8_HUMAN 1998 AEIPADPKLY

MATLPSAERRAFALKINRYSSAEIRKQFTLPPNLGQYHRQSISTSGFPSLQLPQFYDPVE

PVDFEGLLMTHLNSLDVQLAQELGDFTDDDLDVVFTPKECRTLQPSLPEEGVELDPHVRD

CVQTYIREWLIVNRKNQGSPEICGFKKTGSRKDFHKTLPKQTFESETLECSEPAAQAGPR

HLNVLCDVSGKGPVTACDFDLRSLQPDKRLENLLQQVSAEDFEKQNEEARRTNRQAELFA

LYPSVDEEDAVEIRPVPECPKEHLGNRILVKLLTLKFEIEIEPLFASIALYDVKERKKIS

ENFHCDLNSDQFKGFLRAHTPSVAASSQARSAVFSVTYPSSDIYLVVKIEKVLQQGEIGD

CAEPYTVIKESDGGKSKEKIEKLKLQAESFCQRLGKYRMPFAWAPISLSSFFNVSTLERE

VTDVDSVVGRSSVGERRTLAQSRRLSERALSLEENGVGSNFKTSTLSVSSFFKQEGDRLS

DEDLFKFLADYKRSSSLQRRVKSIPGLLRLEISTAPEIINCCLTPEMLPVKPFPENRTRP

HKEILEFPTREVYVPHTVYRNLLYVYPQRLNFVNKLASARNITIKIQFMCGEDASNAMPV

IFGKSSGPEFLQEVYTAVTYHNKSPDFYEEVKIKLPAKLTVNHHLLFTFYHISCQQKQGA

SVETLLGYSWLPILLNERLQTGSYCLPVALEKLPPNYSMHSAEKVPLQNPPIKWAEGHKG

VFNIEVQAVSSVHTQDNHLEKFFTLCHSLESQVTFPIRVLDQKISEMALEHELKLSIICL

NSSRLEPLVLFLHLVLDKLFQLSVQPMVIAGQTANFSQFAFESVVAIANSLHNSKDLSKD

QHGRNCLLASYVHYVFRLPEVQRDVPKSGAPTALLDPRSYHTYGRTSAAAVSSKLLQARV

MSSSNPDLAGTHSAADEEVKNIMSSKIADRNCSRMSYYCSGSSDAPSSPAAPRPASKKHF

HEELALQMVVSTGMVRETVFKYAWFFFELLVKSMAQHVHNMDKRDSFRRTRFSDRFMDDI

TTIVNVVTSEIAALLVKPQKENEQAEKMNISLAFFLYDLLSLMDRGFVFNLIRHYCSQLS

AKLSNLPTLISMRLEFLRILCSHEHYLNLNLFFMNADTAPTSPCPSISSQNSSSCSSFQD

QKIASMFDLTSEYRQQHFLTGLLFTELAAALDAEGEGISKVQRKAVSAIHSLLSSHDLDP

RCVKPEVKVKIAALYLPLVGIILDALPQLCDFTVADTRRYRTSGSDEEQEGAGAINQNVA

LAIAGNNFNLKTSGIVLSSLPYKQYNMLNADTTRNLMICFLWIMKNADQSLIRKWIADLP

STQLNRILDLLFICVLCFEYKGKQSSDKVSTQVLQKSRDVKARLEEALLRGEGARGEMMR

RRAPGNDRFPGLNENLRWKKEQTHWRQANEKLDKTKAELDQEALISGNLATEAHLIILDM

QENIIQASSALDCKDSLLGGVLRVLVNSLNCDQSTTYLTHCFATLRALIAKFGDLLFEEE

VEQCFDLCHQVLHHCSSSMDVTRSQACATLYLLMRFSFGATSNFARVKMQVTMSLASLVG

RAPDFNEEHLRRSLRTILAYSEEDTAMQMTPFPTQVEELLCNLNSILYDTVKMREFQEDP

EMLMDLMYRIAKSYQASPDLRLTWLQNMAEKHTKKKCYTEAAMCLVHAAALVAEYLSMLE

DHSYLPVGSVSFQNISSNVLEESVVSEDTLSPDEDGVCAGQYFTESGLVGLLEQAAELFS

TGGLYETVNEVYKLVIPILEAHREFRKLTLTHSKLQRAFDSIVNKDHKRMFGTYFRVGFF

GSKFGDLDEQEFVYKEPAITKLPEISHRLEAFYGQCFGAEFVEVIKDSTPVDKTKLDPNK

AYIQITFVEPYFDEYEMKDRVTYFEKNFNLRRFMYTTPFTLEGRPRGELHEQYRRNTVLT

TMHAFPYIKTRISVIQKEEFVLTPIEVAIEDMKKKTLQLAVAINQEPPDAKMLQMVLQGS

VGATVNQGPLEVAQVFLAEIPADPKLYRHHNKLRLCFKEFIMRCGEAVEKNKRLITADQR

EYQQELKKNYNKLKENLRPMIERKIPELYKPIFRVESQKRDSFHRSSFRKCETQLSQGS

>sp|Q6GYQ0|GRIPE_HUMAN 411 VTEIFRQAF

MFSKKPHGDVKKSTQKVLDTKKDALTRLKHLRIVIENAESIDLKQFFDQHFSHIYYVFFE

NFVTIEASLKQKGHKSQREELDAILFIFEKILQLLPERIHQRWQFHSIGLILKKLLHTGN

SLKIRREGVRLFLLWLQALQNNCSKEQLWMFSCLIPGFSAPQSEHGPRTLDNLINPPLNL

QETQVTIEEITPLVPPQSGDKGQEDLTSYFLEALLKYIVIQVKSLEWKNKENQERGFSFL

FSHFKKYYLPYIFPNICKENSLYHPILDIPQMRPKPHYVVIKKDAETNEAIYCTKEPFIK

ARVIVIRWLVSFWLEPKPHTGPHIPGMEGEVLPKNIQRAAASLVSREESKNDNADKTDRT

TEPEQSHSNTSTLTEREPSSSSLCSIDEEHLTDIEIVRRVFSSKRSNVNFVTEIFRQAFL

LPICEAAAMRKVVKVYQEWIQQEEKPLFMQEPEEIVITSSDLPCIENVTDHDISMEEGEK

REEENGTNTADHVRNSSWAKNGSYQGALHNASEEATEQNIRAGTQAVLQVFIINSSNIFL

LEPANEIKNLLDEHTDMCKRILNIYRYMVVQVSMDKKTWEQMLLVLLRVTESVLKMPSQA

FLQFQGKKNMTLAGRLAGPLFQTLIVAWIKANLNVYISRELWDDLLSVLSSLTYWEELAT

EWSLTMETLTKVLARNLYSLDLSDLPLDKLSEQKQKKHKGKGVGHEFQKVSVDKSFSRGW

SRDQPGQAPMRQRSATTTGSPGTEKARSIVRQKTVDIDDAQILPRSTRVRHFSQSEETGN

EVFGALNEEQPLPRSSSTSDILEPFTVERAKVNKEDMSQKLPPLNSDIGGSSANVPDLMD

EFIAERLRSGNASTMTRRGSSPGSLEIPKDLPDILNKQNQMRPIDDPGVPSEWTSPASAG

SSDLISSDSHSDSFSAFQYDGRKFDNFGFGTDTGVTSSADVDSGSGHHQSAEEQEVASLT

TLHIDSETSSLNQQAFSAEVATITGSESASPVHSPLGSRSQTPSPSTLNIDHMEQKDLQL

DEKLHHSVLQTPDDLEISEFPSECCSVMAGGTLTGWHADVATVMWRRMLGILGDVNSIMD

PEIHAQVFDYLCELWQNLAKIRDNLGISTDNLTSPSPPVLIPPLRILTPWLFKATMLTDK

YKQGKLHAYKLICNTMKRRQDVSPNRDFLTHFYNIMHCGLLHIDQDIVNTIIKHCSPQFF

SLGLPGATMLIMDFIVAAGRVASSAFLNAPRVEAQVLLGSLVCFPNLYCELPSLHPNIPD

VAVSQFTDVKELIIKTVLSSARDEPSGPARCVALCSLGIWICEELVHESHHPQIKEALNV

ICVSLKFTNKTVAHVACNMLHMLVHYVPRLQIYQPDSPLKIIQILIATITHLLPSTEASS

YEMDKRLVVSLLLCLLDWIMALPLKTLLQPFHATGAESDKTEKSVLNCIYKVLHGCVYGA

QCFSNPRYFPMSLSDLASVDYDPFMHLESLKEPEPLHSPDSERSSKLQPVTEVKTQMQHG

LISIAARTVITHLVNHLGHYPMSGGPAMLTSQVCENHDNHYSESTELSPELFESPNIQFF

VLNNTTLVSCIQIRSEENMPGGGLSAGLASANSNVRIIVRDLSGKYSWDSAILYGPPPVS

GLSEPTSFMLSLSHQEKPEEPPTSNECLEDITVKDGLSLQFKRFRETVPTWDTIRDEEDV

LDELLQYLGVTSPECLQRTGISLNIPAPQPVCISEKQENDVINAILKQHTEEKEFVEKHF

NDLNMKAVEQDEPIPQKPQSAFYYCRLLLSILGMNSWDKRRSFHLLKKNEKLLRELRNLD

SRQCRETHKIAVFYVAEGQEDKHSILTNTGGSQAYEDFVAGLGWEVNLTNHCGFMGGLQK

NKSTGLTTPYFATSTVEVIFHVSTRMPSDSDDSLTKKLRHLGNDEVHIVWSEHTRDYRRG

IIPTEFGDVLIVIYPMKNHMFSIQIMKKPEVPFFGPLFDGAIVNGKVLPIMVRATAINAS

RALKSLIPLYQNFYEERARYLQTIVQHHLEPTTFEDFAAQVFSPAPYHHLPSDADH

>sp|Q92621|NU205_HUMAN 1574 LRSGVIVRL

MATPLAVNSAASLWGPYKDIWHKVGNALWRRQPEAVHLLDKILKKHKPDFISLFKNPPKN

VQQHEKVQKASTEGVAIQGQQGTRLLPEQLIKEAFILSDLFDIGELAAVELLLAGEHQQP

HFPGLTRGLVAVLLYWDGKRCIANSLKALIQSRRGKTWTLELSPELASMTTRFTDELMEQ

GLTYKVLTLVSQIDVNNEFEKLQRERGLGSEKHRKEVSDLIKECRQSLAESLFAWACQSP

LGKEDTLLLIGHLERVTVEANGSLDAVNLALLMALLYCFDISFIEQSTEERDDMIHQLPL

LTEKQYIATIHSRLQDSQLWKLPGLQATVRLAWALALRGISQLPDVTALAEFTEADEAMA

ELAIADNVFLFLMESVVVSEYFYQEEFYIRRVHNLITDFLALMPMKVKQLRNRADEDARM

IHMSMQMGNEPPISLRRDLEHLMLLIGELYKKNPFHLELALEYWCPTEPLQTPTIMGSYL

GVAHQRPPQRQVVLSKFVRQMGDLLPPTIYIPYLKMLQGLANGPQCAHYCFSLLKVNGSS

HVENIQGAGGSPVSWEHFFHSLMLYHEHLRKDLPSADSVQYRHLPSRGITQKEQDGLIAF

LQLTSTIITWSENARLALCEHPQWTPVVVILGLLQCSIPPVLKAELLKTLAAFGKSPEIA

ASLWQSLEYTQILQTVRIPSQRQAIGIEVELNEIESRCEEYPLTRAFCQLISTLVESSFP

SNLGAGLRPPGFDPYLQFLRDSVFLRFRTRAYRRAAEKWEVAEVVLEVFYKLLRDYEPQL

EDFVDQFVELQGEEIIAYKPPGFSLMYHLLNESPMLELALSLLEEGVKQLDTYAPFPGKK

HLEKAVQHCLALLNLTLQKENLFMDLLRESQLALIVCPLEQLLQGINPRTKKADNVVNIA

RYLYHGNTNPELAFESAKILCCISCNSNIQIKLVGDFTHDQSISQKLMAGFVECLDCEDA

EEFVRLEEGSELEKKLVAIRHETRIHILNLLITSLECNPPNLALYLLGFELKKPVSTTNL

QDPGVLGCPRTCLHAILNILEKGTEGRTGPVAVRESPQLAELCYQVIYQLCACSDTSGPT

MRYLRTSQDFLFSQLQYLPFSNKEYEISMLNQMSWLMKTASIELRVTSLNRQRSHTQRLL

HLLLDDMPVKPYSDGEGGIEDENRSVSGFLHFDTATKVRRKILNILDSIDFSQEIPEPLQ

LDFFDRAQIEQVIANCEHKNLRGQTVCNVKLLHRVLVAEVNALQGMAAIGQRPLLMEEIS

TVLQYVVGRNKLLQCLHAKRHALESWRQLVEIILTACPQDLIQAEDRQLIIRDILQDVHD

KILDDEAAQELMPVVAGAVFTLTAHLSQAVLTEQKQTSVLGPAEAHYAFMLDSCFTSPPP

EENPLVGFASIGDSSLYIILKKLLDFILKTGGGFQRVRTHLYGSLLYYLQIAQRPDEPDT

LEAAKKTMWERLTAPEDVFSKLQRENIAIIESYGAALMEVVCRDACDGHEIGRMLALALL

DRIVSVDKQQQWLLYLSNSGYLKVLVDSLVEDDRTLQSLLTPQPPLLKALYTYESKMAFL

TRVAKIQQGALELLRSGVIVRLAQCQVYDMRPETDPQSMFGMRDPPMFIPTPVDRYRQIL

LPALQLCQVILTSSMAQHLQAAGQVLQFLISHSDTIQAILRCQDVSAGSLQELALLTGII

SKAALPGILSELDVDVNEGSLMELQGHIGRFQRQCLGLLSRFGGSDRLRQFKFQDDNVEG

DKVSKKDEIELAMQQICANVMEYCQSLMLQSSPTFQHAVCLFTPSLSETVNRDGPRQDTQ

APVVPYWRLPGLGIIIYLLKQSANDFFSYYDSHRQSVSKLQNVEQLPPDEIKELCQSVMP

AGVDKISTAQKYVLARRRLVKVINNRAKLLSLCSFIIETCLFILWRHLEYYLLHCMPTDS

QDSLFASRTLFKSRRLQDSFASETNLDFRSGLAIVSQHDLDQLQADAINAFGESLQKKLL

DIEGLYSKVRSRYSFIQALVRRIRGLLRISRN

>sp|Q92621|NU205_HUMAN 1720 SRFGGSDRL

MATPLAVNSAASLWGPYKDIWHKVGNALWRRQPEAVHLLDKILKKHKPDFISLFKNPPKN

VQQHEKVQKASTEGVAIQGQQGTRLLPEQLIKEAFILSDLFDIGELAAVELLLAGEHQQP

HFPGLTRGLVAVLLYWDGKRCIANSLKALIQSRRGKTWTLELSPELASMTTRFTDELMEQ

GLTYKVLTLVSQIDVNNEFEKLQRERGLGSEKHRKEVSDLIKECRQSLAESLFAWACQSP

LGKEDTLLLIGHLERVTVEANGSLDAVNLALLMALLYCFDISFIEQSTEERDDMIHQLPL

LTEKQYIATIHSRLQDSQLWKLPGLQATVRLAWALALRGISQLPDVTALAEFTEADEAMA

ELAIADNVFLFLMESVVVSEYFYQEEFYIRRVHNLITDFLALMPMKVKQLRNRADEDARM

IHMSMQMGNEPPISLRRDLEHLMLLIGELYKKNPFHLELALEYWCPTEPLQTPTIMGSYL

GVAHQRPPQRQVVLSKFVRQMGDLLPPTIYIPYLKMLQGLANGPQCAHYCFSLLKVNGSS

HVENIQGAGGSPVSWEHFFHSLMLYHEHLRKDLPSADSVQYRHLPSRGITQKEQDGLIAF

LQLTSTIITWSENARLALCEHPQWTPVVVILGLLQCSIPPVLKAELLKTLAAFGKSPEIA

ASLWQSLEYTQILQTVRIPSQRQAIGIEVELNEIESRCEEYPLTRAFCQLISTLVESSFP

SNLGAGLRPPGFDPYLQFLRDSVFLRFRTRAYRRAAEKWEVAEVVLEVFYKLLRDYEPQL

EDFVDQFVELQGEEIIAYKPPGFSLMYHLLNESPMLELALSLLEEGVKQLDTYAPFPGKK

HLEKAVQHCLALLNLTLQKENLFMDLLRESQLALIVCPLEQLLQGINPRTKKADNVVNIA

RYLYHGNTNPELAFESAKILCCISCNSNIQIKLVGDFTHDQSISQKLMAGFVECLDCEDA

EEFVRLEEGSELEKKLVAIRHETRIHILNLLITSLECNPPNLALYLLGFELKKPVSTTNL

QDPGVLGCPRTCLHAILNILEKGTEGRTGPVAVRESPQLAELCYQVIYQLCACSDTSGPT

MRYLRTSQDFLFSQLQYLPFSNKEYEISMLNQMSWLMKTASIELRVTSLNRQRSHTQRLL

HLLLDDMPVKPYSDGEGGIEDENRSVSGFLHFDTATKVRRKILNILDSIDFSQEIPEPLQ

LDFFDRAQIEQVIANCEHKNLRGQTVCNVKLLHRVLVAEVNALQGMAAIGQRPLLMEEIS

TVLQYVVGRNKLLQCLHAKRHALESWRQLVEIILTACPQDLIQAEDRQLIIRDILQDVHD

KILDDEAAQELMPVVAGAVFTLTAHLSQAVLTEQKQTSVLGPAEAHYAFMLDSCFTSPPP

EENPLVGFASIGDSSLYIILKKLLDFILKTGGGFQRVRTHLYGSLLYYLQIAQRPDEPDT

LEAAKKTMWERLTAPEDVFSKLQRENIAIIESYGAALMEVVCRDACDGHEIGRMLALALL

DRIVSVDKQQQWLLYLSNSGYLKVLVDSLVEDDRTLQSLLTPQPPLLKALYTYESKMAFL

TRVAKIQQGALELLRSGVIVRLAQCQVYDMRPETDPQSMFGMRDPPMFIPTPVDRYRQIL

LPALQLCQVILTSSMAQHLQAAGQVLQFLISHSDTIQAILRCQDVSAGSLQELALLTGII

SKAALPGILSELDVDVNEGSLMELQGHIGRFQRQCLGLLSRFGGSDRLRQFKFQDDNVEG

DKVSKKDEIELAMQQICANVMEYCQSLMLQSSPTFQHAVCLFTPSLSETVNRDGPRQDTQ

APVVPYWRLPGLGIIIYLLKQSANDFFSYYDSHRQSVSKLQNVEQLPPDEIKELCQSVMP

AGVDKISTAQKYVLARRRLVKVINNRAKLLSLCSFIIETCLFILWRHLEYYLLHCMPTDS

QDSLFASRTLFKSRRLQDSFASETNLDFRSGLAIVSQHDLDQLQADAINAFGESLQKKLL

DIEGLYSKVRSRYSFIQALVRRIRGLLRISRN

>sp|Q92621|NU205_HUMAN 302 TEKQYIATI

MATPLAVNSAASLWGPYKDIWHKVGNALWRRQPEAVHLLDKILKKHKPDFISLFKNPPKN

VQQHEKVQKASTEGVAIQGQQGTRLLPEQLIKEAFILSDLFDIGELAAVELLLAGEHQQP

HFPGLTRGLVAVLLYWDGKRCIANSLKALIQSRRGKTWTLELSPELASMTTRFTDELMEQ

GLTYKVLTLVSQIDVNNEFEKLQRERGLGSEKHRKEVSDLIKECRQSLAESLFAWACQSP

LGKEDTLLLIGHLERVTVEANGSLDAVNLALLMALLYCFDISFIEQSTEERDDMIHQLPL

LTEKQYIATIHSRLQDSQLWKLPGLQATVRLAWALALRGISQLPDVTALAEFTEADEAMA

ELAIADNVFLFLMESVVVSEYFYQEEFYIRRVHNLITDFLALMPMKVKQLRNRADEDARM

IHMSMQMGNEPPISLRRDLEHLMLLIGELYKKNPFHLELALEYWCPTEPLQTPTIMGSYL

GVAHQRPPQRQVVLSKFVRQMGDLLPPTIYIPYLKMLQGLANGPQCAHYCFSLLKVNGSS

HVENIQGAGGSPVSWEHFFHSLMLYHEHLRKDLPSADSVQYRHLPSRGITQKEQDGLIAF

LQLTSTIITWSENARLALCEHPQWTPVVVILGLLQCSIPPVLKAELLKTLAAFGKSPEIA

ASLWQSLEYTQILQTVRIPSQRQAIGIEVELNEIESRCEEYPLTRAFCQLISTLVESSFP

SNLGAGLRPPGFDPYLQFLRDSVFLRFRTRAYRRAAEKWEVAEVVLEVFYKLLRDYEPQL

EDFVDQFVELQGEEIIAYKPPGFSLMYHLLNESPMLELALSLLEEGVKQLDTYAPFPGKK

HLEKAVQHCLALLNLTLQKENLFMDLLRESQLALIVCPLEQLLQGINPRTKKADNVVNIA

RYLYHGNTNPELAFESAKILCCISCNSNIQIKLVGDFTHDQSISQKLMAGFVECLDCEDA

EEFVRLEEGSELEKKLVAIRHETRIHILNLLITSLECNPPNLALYLLGFELKKPVSTTNL

QDPGVLGCPRTCLHAILNILEKGTEGRTGPVAVRESPQLAELCYQVIYQLCACSDTSGPT

MRYLRTSQDFLFSQLQYLPFSNKEYEISMLNQMSWLMKTASIELRVTSLNRQRSHTQRLL

HLLLDDMPVKPYSDGEGGIEDENRSVSGFLHFDTATKVRRKILNILDSIDFSQEIPEPLQ

LDFFDRAQIEQVIANCEHKNLRGQTVCNVKLLHRVLVAEVNALQGMAAIGQRPLLMEEIS

TVLQYVVGRNKLLQCLHAKRHALESWRQLVEIILTACPQDLIQAEDRQLIIRDILQDVHD

KILDDEAAQELMPVVAGAVFTLTAHLSQAVLTEQKQTSVLGPAEAHYAFMLDSCFTSPPP

EENPLVGFASIGDSSLYIILKKLLDFILKTGGGFQRVRTHLYGSLLYYLQIAQRPDEPDT

LEAAKKTMWERLTAPEDVFSKLQRENIAIIESYGAALMEVVCRDACDGHEIGRMLALALL

DRIVSVDKQQQWLLYLSNSGYLKVLVDSLVEDDRTLQSLLTPQPPLLKALYTYESKMAFL

TRVAKIQQGALELLRSGVIVRLAQCQVYDMRPETDPQSMFGMRDPPMFIPTPVDRYRQIL

LPALQLCQVILTSSMAQHLQAAGQVLQFLISHSDTIQAILRCQDVSAGSLQELALLTGII

SKAALPGILSELDVDVNEGSLMELQGHIGRFQRQCLGLLSRFGGSDRLRQFKFQDDNVEG

DKVSKKDEIELAMQQICANVMEYCQSLMLQSSPTFQHAVCLFTPSLSETVNRDGPRQDTQ

APVVPYWRLPGLGIIIYLLKQSANDFFSYYDSHRQSVSKLQNVEQLPPDEIKELCQSVMP

AGVDKISTAQKYVLARRRLVKVINNRAKLLSLCSFIIETCLFILWRHLEYYLLHCMPTDS

QDSLFASRTLFKSRRLQDSFASETNLDFRSGLAIVSQHDLDQLQADAINAFGESLQKKLL

DIEGLYSKVRSRYSFIQALVRRIRGLLRISRN

>sp|Q7Z406|MYH14_HUMAN 855 FTKVKPLL

MAAVTMSVPGRKAPPRPGPVPEAAQPFLFTPRGPSAGGGPGSGTSPQVEWTARRLVWVPS

ELHGFEAAALRDEGEEEAEVELAESGRRLRLPRDQIQRMNPPKFSKAEDMAELTCLNEAS

VLHNLRERYYSGLIYTYSGLFCVVINPYKQLPIYTEAIVEMYRGKKRHEVPPHVYAVTEG

AYRSMLQDREDQSILCTGESGAGKTENTKKVIQYLAHVASSPKGRKEPGVPGELERQLLQ

ANPILEAFGNAKTVKNDNSSRFGKFIRINFDVAGYIVGANIETYLLEKSRAIRQAKDECS

FHIFYQLLGGAGEQLKADLLLEPCSHYRFLTNGPSSSPGQERELFQETLESLRVLGFSHE

EIISMLRMVSAVLQFGNIALKRERNTDQATMPDNTAAQKLCRLLGLGVTDFSRALLTPRI

KVGRDYVQKAQTKEQADFALEALAKATYERLFRWLVLRLNRALDRSPRQGASFLGILDIA

GFEIFQLNSFEQLCINYTNEKLQQLFNHTMFVLEQEEYQREGIPWTFLDFGLDLQPCIDL

IERPANPPGLLALLDEECWFPKATDKSFVEKVAQEQGGHPKFQRPRHLRDQADFSVLHYA

GKVDYKANEWLMKNMDPLNDNVAALLHQSTDRLTAEIWKDVEGIVGLEQVSSLGDGPPGG

RPRRGMFRTVGQLYKESLSRLMATLSNTNPSFVRCIVPNHEKRAGKLEPRLVLDQLRCNG

VLEGIRICRQGFPNRILFQEFRQRYEILTPNAIPKGFMDGKQACEKMIQALELDPNLYRV

GQSKIFFRAGVLAQLEEERDLKVTDIIVSFQAAARGYLARRAFQKRQQQQSALRVMQRNC

AAYLKLRHWQWWRLFTKVKPLLQVTRQDEVLQARAQELQKVQELQQQSAREVGELQGRVA

QLEEERARLAEQLRAEAELCAEAEETRGRLAARKQELELVVSELEARVGEEEECSRQMQT

EKKRLQQHIQELEAHLEAEEGARQKLQLEKVTTEAKMKKFEEDLLLLEDQNSKLSKSGSC

WKIVWPSSHPRQLRRRRRSRASISYGSNMRPQSQTWRDRLRKEEKGRQELEKLKRRLDGE

SSELQEQMVEQQQRAEELRAQLGRKEEELQAALARAEDEGGARAQLLKSLREAQAALAEA

QEDLESERVARTKAEKQRRDLGEELEALRGELEDTLDSTNAQQELRSKREQEVTELKKTL

EEETRIHEAAVQELRQRHGQALGELAEQLEQARRGKGAWEKTRLALEAEVSELRAELSSL

QTARQEGEQRRRRLELQLQEVQGRAGDGERARAEAAEKLQRAQAELENVSGALNEAESKT

IRLSKELSSTEAQLHDAQELLQEETRAKLALGSRVRAMEAEAAGLREQLEEEAAARERAG

RELQTAQAQLSEWRRRQEEEAGALEAGEEARRRAAREAEALTQRLAEKTETVDRLERGRR

RLQQELDDATMDLEQQRQLVSTLEKKQRKFDQLLAEEKAAVLRAVEERERAEAEGREREA

RALSLTRALEEEQEAREELERQNRALRAELEALLSSKDDVGKSVHELERACRVAEQAAND

LRAQVTELEDELTAAEDAKLRLEVTVQALKTQHERDLQGRDEAGEERRRQLAKQLRDAEV

ERDEERKQRTLAVAARKKLEGELEELKAQMASAGQGKEEAVKQLRKMQAQMKELWREVEE

TRTSREEIFSQNRESEKRLKGLEAEVLRLQEELAASDRARRQAQQDRDEMADEVANGNLS

KAAILEEKRQLEGRLGQLEEELEEEQSNSELLNDRYRKLLLQVESLTTELSAERSFSAKA

ESGRQQLERQIQELRGRLGEEDAGARARHKMTIAALESKLAQAEEQLEQETRERILSGKL

VRRAEKRLKEVVLQVEEERRVADQLRDQLEKGNLRVKQLKRQLEEAEEEASRAQAGRRRL

QRELEDVTESAESMNREVTTLRNRLRRGPLTFTTRTVRQVFRLEEGVASDEEAEEAQPGS

GPSPEPEGSPPAHPQ

>sp|Q7Z406|MYH14_HUMAN 284 YLLEKSRAI

MAAVTMSVPGRKAPPRPGPVPEAAQPFLFTPRGPSAGGGPGSGTSPQVEWTARRLVWVPS

ELHGFEAAALRDEGEEEAEVELAESGRRLRLPRDQIQRMNPPKFSKAEDMAELTCLNEAS

VLHNLRERYYSGLIYTYSGLFCVVINPYKQLPIYTEAIVEMYRGKKRHEVPPHVYAVTEG

AYRSMLQDREDQSILCTGESGAGKTENTKKVIQYLAHVASSPKGRKEPGVPGELERQLLQ

ANPILEAFGNAKTVKNDNSSRFGKFIRINFDVAGYIVGANIETYLLEKSRAIRQAKDECS

FHIFYQLLGGAGEQLKADLLLEPCSHYRFLTNGPSSSPGQERELFQETLESLRVLGFSHE

EIISMLRMVSAVLQFGNIALKRERNTDQATMPDNTAAQKLCRLLGLGVTDFSRALLTPRI

KVGRDYVQKAQTKEQADFALEALAKATYERLFRWLVLRLNRALDRSPRQGASFLGILDIA

GFEIFQLNSFEQLCINYTNEKLQQLFNHTMFVLEQEEYQREGIPWTFLDFGLDLQPCIDL

IERPANPPGLLALLDEECWFPKATDKSFVEKVAQEQGGHPKFQRPRHLRDQADFSVLHYA

GKVDYKANEWLMKNMDPLNDNVAALLHQSTDRLTAEIWKDVEGIVGLEQVSSLGDGPPGG

RPRRGMFRTVGQLYKESLSRLMATLSNTNPSFVRCIVPNHEKRAGKLEPRLVLDQLRCNG

VLEGIRICRQGFPNRILFQEFRQRYEILTPNAIPKGFMDGKQACEKMIQALELDPNLYRV

GQSKIFFRAGVLAQLEEERDLKVTDIIVSFQAAARGYLARRAFQKRQQQQSALRVMQRNC

AAYLKLRHWQWWRLFTKVKPLLQVTRQDEVLQARAQELQKVQELQQQSAREVGELQGRVA

QLEEERARLAEQLRAEAELCAEAEETRGRLAARKQELELVVSELEARVGEEEECSRQMQT

EKKRLQQHIQELEAHLEAEEGARQKLQLEKVTTEAKMKKFEEDLLLLEDQNSKLSKSGSC

WKIVWPSSHPRQLRRRRRSRASISYGSNMRPQSQTWRDRLRKEEKGRQELEKLKRRLDGE

SSELQEQMVEQQQRAEELRAQLGRKEEELQAALARAEDEGGARAQLLKSLREAQAALAEA

QEDLESERVARTKAEKQRRDLGEELEALRGELEDTLDSTNAQQELRSKREQEVTELKKTL

EEETRIHEAAVQELRQRHGQALGELAEQLEQARRGKGAWEKTRLALEAEVSELRAELSSL

QTARQEGEQRRRRLELQLQEVQGRAGDGERARAEAAEKLQRAQAELENVSGALNEAESKT

IRLSKELSSTEAQLHDAQELLQEETRAKLALGSRVRAMEAEAAGLREQLEEEAAARERAG

RELQTAQAQLSEWRRRQEEEAGALEAGEEARRRAAREAEALTQRLAEKTETVDRLERGRR

RLQQELDDATMDLEQQRQLVSTLEKKQRKFDQLLAEEKAAVLRAVEERERAEAEGREREA

RALSLTRALEEEQEAREELERQNRALRAELEALLSSKDDVGKSVHELERACRVAEQAAND

LRAQVTELEDELTAAEDAKLRLEVTVQALKTQHERDLQGRDEAGEERRRQLAKQLRDAEV

ERDEERKQRTLAVAARKKLEGELEELKAQMASAGQGKEEAVKQLRKMQAQMKELWREVEE

TRTSREEIFSQNRESEKRLKGLEAEVLRLQEELAASDRARRQAQQDRDEMADEVANGNLS

KAAILEEKRQLEGRLGQLEEELEEEQSNSELLNDRYRKLLLQVESLTTELSAERSFSAKA

ESGRQQLERQIQELRGRLGEEDAGARARHKMTIAALESKLAQAEEQLEQETRERILSGKL

VRRAEKRLKEVVLQVEEERRVADQLRDQLEKGNLRVKQLKRQLEEAEEEASRAQAGRRRL

QRELEDVTESAESMNREVTTLRNRLRRGPLTFTTRTVRQVFRLEEGVASDEEAEEAQPGS

GPSPEPEGSPPAHPQ

>sp|Q14669|TRIPC_HUMAN 1751 HLEDIVRQK

MSNRPNNNPGGSLRRSQRNTAGAQPQDDSIGGRSCSSSSAVIVPQPEDPDRANTSERQKT

GQVPKKDNSRGVKRSASPDYNRTNSPSSAKKPKALQHTESPSETNKPHSKSKKRHLDQEQ

QLKSAQSPSTSKAHTRKSGATGGSRSQKRKRTESSCVKSGSGSESTGAEERSAKPTKLAS

KSATSAKAGCSTITDSSSAASTSSSSSAVASASSTVPPGARVKQGKDQNKARRSRSASSP

SPRRSSREKEQSKTGGSSKFDWAARFSPKVSLPKTKLSLPGSSKSETSKPGPSGLQAKLA

SLRKSTKKRSESPPAELPSLRRSTRQKTTGSCASTSRRGSGLGKRGAAEARRQEKMADPE

SNQEAVNSSAARTDEAPQGAAGAVGMTTSGESESDDSEMGRLQALLEARGLPPHLFGPLG

PRMSQLFHRTIGSGASSKAQQLLQGLQASDESQQLQAVIEMCQLLVMGNEETLGGFPVKS

VVPALITLLQMEHNFDIMNHACRALTYMMEALPRSSAVVVDAIPVFLEKLQVIQCIDVAE

QALTALEMLSRRHSKAILQAGGLADCLLYLEFFSINAQRNALAIAANCCQSITPDEFHFV

ADSLPLLTQRLTHQDKKSVESTCLCFARLVDNFQHEENLLQQVASKDLLTNVQQLLVVTP

PILSSGMFIMVVRMFSLMCSNCPTLAVQLMKQNIAETLHFLLCGASNGSCQEQIDLVPRS

PQELYELTSLICELMPCLPKEGIFAVDTMLKKGNAQNTDGAIWQWRDDRGLWHPYNRIDS

RIIEQINEDTGTARAIQRKPNPLANSNTSGYSESKKDDARAQLMKEDPELAKSFIKTLFG

VLYEVYSSSAGPAVRHKCLRAILRIIYFADAELLKDVLKNHAVSSHIASMLSSQDLKIVV

GALQMAEILMQKLPDIFSVYFRREGVMHQVKHLAESESLLTSPPKACTNGSGSMGSTTSV

SSGTATAATHAAADLGSPSLQHSRDDSLDLSPQGRLSDVLKRKRLPKRGPRRPKYSPPRD

DDKVDNQAKSPTTTQSPKSSFLASLNPKTWGRLSTQSNSNNIEPARTAGGSGLARAASKD

TISNNREKIKGWIKEQAHKFVERYFSSENMDGSNPALNVLQRLCAATEQLNLQVDGGAEC

LVEIRSIVSESDVSSFEIQHSGFVKQLLLYLTSKSEKDAVSREIRLKRFLHVFFSSPLPG

EEPIGRVEPVGNAPLLALVHKMNNCLSQMEQFPVKVHDFPSGNGTGGSFSLNRGSQALKF

FNTHQLKCQLQRHPDCANVKQWKGGPVKIDPLALVQAIERYLVVRGYGRVREDDEDSDDD

GSDEEIDESLAAQFLNSGNVRHRLQFYIGEHLLPYNMTVYQAVRQFSIQAEDERESTDDE

SNPLGRAGIWTKTHTIWYKPVREDEESNKDCVGGKRGRAQTAPTKTSPRNAKKHDELWHD

GVCPSVSNPLEVYLIPTPPENITFEDPSLDVILLLRVLHAISRYWYYLYDNAMCKEIIPT

SEFINSKLTAKANRQLQDPLVIMTGNIPTWLTELGKTCPFFFPFDTRQMLFYVTAFDRDR

AMQRLLDTNPEINQSDSQDSRVAPRLDRKKRTVNREELLKQAESVMQDLGSSRAMLEIQY

ENEVGTGLGPTLEFYALVSQELQRADLGLWRGEEVTLSNPKGSQEGTKYIQNLQGLFALP

FGRTAKPAHIAKVKMKFRFLGKLMAKAIMDFRLVDLPLGLPFYKWMLRQETSLTSHDLFD

IDPVVARSVYHLEDIVRQKKRLEQDKSQTKESLQYALETLTMNGCSVEDLGLDFTLPGFP

NIELKKGGKDIPVTIHNLEEYLRLVIFWALNEGVSRQFDSFRDGFESVFPLSHLQYFYPE

ELDQLLCGSKADTWDAKTLMECCRPDHGYTHDSRAVKFLFEILSSFDNEQQRLFLQFVTG

SPRLPVGGFRSLNPPLTIVRKTFESTENPDDFLPSVMTCVNYLKLPDYSSIEIMREKLLI

AAREGQQSFHLS

>sp|Q14669|TRIPC_HUMAN 1921 SPRLPVGGF

MSNRPNNNPGGSLRRSQRNTAGAQPQDDSIGGRSCSSSSAVIVPQPEDPDRANTSERQKT

GQVPKKDNSRGVKRSASPDYNRTNSPSSAKKPKALQHTESPSETNKPHSKSKKRHLDQEQ

QLKSAQSPSTSKAHTRKSGATGGSRSQKRKRTESSCVKSGSGSESTGAEERSAKPTKLAS

KSATSAKAGCSTITDSSSAASTSSSSSAVASASSTVPPGARVKQGKDQNKARRSRSASSP

SPRRSSREKEQSKTGGSSKFDWAARFSPKVSLPKTKLSLPGSSKSETSKPGPSGLQAKLA

SLRKSTKKRSESPPAELPSLRRSTRQKTTGSCASTSRRGSGLGKRGAAEARRQEKMADPE

SNQEAVNSSAARTDEAPQGAAGAVGMTTSGESESDDSEMGRLQALLEARGLPPHLFGPLG

PRMSQLFHRTIGSGASSKAQQLLQGLQASDESQQLQAVIEMCQLLVMGNEETLGGFPVKS

VVPALITLLQMEHNFDIMNHACRALTYMMEALPRSSAVVVDAIPVFLEKLQVIQCIDVAE

QALTALEMLSRRHSKAILQAGGLADCLLYLEFFSINAQRNALAIAANCCQSITPDEFHFV

ADSLPLLTQRLTHQDKKSVESTCLCFARLVDNFQHEENLLQQVASKDLLTNVQQLLVVTP

PILSSGMFIMVVRMFSLMCSNCPTLAVQLMKQNIAETLHFLLCGASNGSCQEQIDLVPRS

PQELYELTSLICELMPCLPKEGIFAVDTMLKKGNAQNTDGAIWQWRDDRGLWHPYNRIDS

RIIEQINEDTGTARAIQRKPNPLANSNTSGYSESKKDDARAQLMKEDPELAKSFIKTLFG

VLYEVYSSSAGPAVRHKCLRAILRIIYFADAELLKDVLKNHAVSSHIASMLSSQDLKIVV

GALQMAEILMQKLPDIFSVYFRREGVMHQVKHLAESESLLTSPPKACTNGSGSMGSTTSV

SSGTATAATHAAADLGSPSLQHSRDDSLDLSPQGRLSDVLKRKRLPKRGPRRPKYSPPRD

DDKVDNQAKSPTTTQSPKSSFLASLNPKTWGRLSTQSNSNNIEPARTAGGSGLARAASKD

TISNNREKIKGWIKEQAHKFVERYFSSENMDGSNPALNVLQRLCAATEQLNLQVDGGAEC

LVEIRSIVSESDVSSFEIQHSGFVKQLLLYLTSKSEKDAVSREIRLKRFLHVFFSSPLPG

EEPIGRVEPVGNAPLLALVHKMNNCLSQMEQFPVKVHDFPSGNGTGGSFSLNRGSQALKF

FNTHQLKCQLQRHPDCANVKQWKGGPVKIDPLALVQAIERYLVVRGYGRVREDDEDSDDD

GSDEEIDESLAAQFLNSGNVRHRLQFYIGEHLLPYNMTVYQAVRQFSIQAEDERESTDDE

SNPLGRAGIWTKTHTIWYKPVREDEESNKDCVGGKRGRAQTAPTKTSPRNAKKHDELWHD

GVCPSVSNPLEVYLIPTPPENITFEDPSLDVILLLRVLHAISRYWYYLYDNAMCKEIIPT

SEFINSKLTAKANRQLQDPLVIMTGNIPTWLTELGKTCPFFFPFDTRQMLFYVTAFDRDR

AMQRLLDTNPEINQSDSQDSRVAPRLDRKKRTVNREELLKQAESVMQDLGSSRAMLEIQY

ENEVGTGLGPTLEFYALVSQELQRADLGLWRGEEVTLSNPKGSQEGTKYIQNLQGLFALP

FGRTAKPAHIAKVKMKFRFLGKLMAKAIMDFRLVDLPLGLPFYKWMLRQETSLTSHDLFD

IDPVVARSVYHLEDIVRQKKRLEQDKSQTKESLQYALETLTMNGCSVEDLGLDFTLPGFP

NIELKKGGKDIPVTIHNLEEYLRLVIFWALNEGVSRQFDSFRDGFESVFPLSHLQYFYPE

ELDQLLCGSKADTWDAKTLMECCRPDHGYTHDSRAVKFLFEILSSFDNEQQRLFLQFVTG

SPRLPVGGFRSLNPPLTIVRKTFESTENPDDFLPSVMTCVNYLKLPDYSSIEIMREKLLI

AAREGQQSFHLS

>sp|P35580|MYH10_HUMAN 277 DERTFHIFY

MAQRTGLEDPERYLFVDRAVIYNPATQADWTAKKLVWIPSERHGFEAASIKEERGDEVMV

ELAENGKKAMVNKDDIQKMNPPKFSKVEDMAELTCLNEASVLHNLKDRYYSGLIYTYSGL

FCVVINPYKNLPIYSENIIEMYRGKKRHEMPPHIYAISESAYRCMLQDREDQSILCTGES

GAGKTENTKKVIQYLAHVASSHKGRKDHNIPGELERQLLQANPILESFGNAKTVKNDNSS

RFGKFIRINFDVTGYIVGANIETYLLEKSRAVRQAKDERTFHIFYQLLSGAGEHLKSDLL

LEGFNNYRFLSNGYIPIPGQQDKDNFQETMEAMHIMGFSHEEILSMLKVVSSVLQFGNIS

FKKERNTDQASMPENTVAQKLCHLLGMNVMEFTRAILTPRIKVGRDYVQKAQTKEQADFA

VEALAKATYERLFRWLVHRINKALDRTKRQGASFIGILDIAGFEIFELNSFEQLCINYTN

EKLQQLFNHTMFILEQEEYQREGIEWNFIDFGLDLQPCIDLIERPANPPGVLALLDEECW

FPKATDKTFVEKLVQEQGSHSKFQKPRQLKDKADFCIIHYAGKVDYKADEWLMKNMDPLN

DNVATLLHQSSDRFVAELWKDVDRIVGLDQVTGMTETAFGSAYKTKKGMFRTVGQLYKES

LTKLMATLRNTNPNFVRCIIPNHEKRAGKLDPHLVLDQLRCNGVLEGIRICRQGFPNRIV

FQEFRQRYEILTPNAIPKGFMDGKQACERMIRALELDPNLYRIGQSKIFFRAGVLAHLEE

ERDLKITDIIIFFQAVCRGCLARKAFAKKQQQLSALKVLQRNCAAYLKLRHWQWWRVFTK

VKPLLQVTRQEEELQAKDEELLKVKEKQTKVEGELEEMERKHQQLLEEKNILAEQLQAET

ELFAEAEEMRARLAAKKQELEEILHDLESRVEEEEERNQILQNEKKKMQAHIQDLEEQLD

EEEGARQKLQLEKVTAEAKIKKMEEEILLLEDQNSKFIKEKKLMEDRIAECSSQLAEEEE

KAKNLAKIRNKQEVMISDLEERLKKEEKTRQELEKAKRKLDGETTDLQDQIAELQAQIDE

LKLQLAKKEEELQGALARGDDETLHKNNALKVVRELQAQIAELQEDFESEKASRNKAEKQ

KRDLSEELEALKTELEDTLDTTAAQQELRTKREQEVAELKKALEEETKNHEAQIQDMRQR

HATALEELSEQLEQAKRFKANLEKNKQGLETDNKELACEVKVLQQVKAESEHKRKKLDAQ

VQELHAKVSEGDRLRVELAEKASKLQNELDNVSTLLEEAEKKGIKFAKDAASLESQLQDT

QELLQEETRQKLNLSSRIRQLEEEKNSLQEQQEEEEEARKNLEKQVLALQSQLADTKKKV

DDDLGTIESLEEAKKKLLKDAEALSQRLEEKALAYDKLEKTKNRLQQELDDLTVDLDHQR

QVASNLEKKQKKFDQLLAEEKSISARYAEERDRAEAEAREKETKALSLARALEEALEAKE

EFERQNKQLRADMEDLMSSKDDVGKNVHELEKSKRALEQQVEEMRTQLEELEDELQATED

AKLRLEVNMQAMKAQFERDLQTRDEQNEEKKRLLIKQVRELEAELEDERKQRALAVASKK

KMEIDLKDLEAQIEAANKARDEVIKQLRKLQAQMKDYQRELEEARASRDEIFAQSKESEK

KLKSLEAEILQLQEELASSERARRHAEQERDELADEITNSASGKSALLDEKRRLEARIAQ

LEEELEEEQSNMELLNDRFRKTTLQVDTLNAELAAERSAAQKSDNARQQLERQNKELKAK

LQELEGAVKSKFKATISALEAKIGQLEEQLEQEAKERAAANKLVRRTEKKLKEIFMQVED

ERRHADQYKEQMEKANARMKQLKRQLEEAEEEATRANASRRKLQRELDDATEANEGLSRE

VSTLKNRLRRGGPISFSSSRSGRRQLHLEGASLELSDDDTESKTSDVNETQPPQSE

>sp|P35749|MYH11_HUMAN 1860 DERKMAEQY

MAQKGQLSDDEKFLFVDKNFINSPVAQADWAAKRLVWVPSEKQGFEAASIKEEKGDEVVV

ELVENGKKVTVGKDDIQKMNPPKFSKVEDMAELTCLNEASVLHNLRERYFSGLIYTYSGL

FCVVVNPYKHLPIYSEKIVDMYKGKKRHEMPPHIYAIADTAYRSMLQDREDQSILCTGES

GAGKTENTKKVIQYLAVVASSHKGKKDTSITGELEKQLLQANPILEAFGNAKTVKNDNSS

RFGKFIRINFDVTGYIVGANIETYLLEKSRAIRQARDERTFHIFYYMIAGAKEKMRSDLL

LEGFNNYTFLSNGFVPIPAAQDDEMFQETVEAMAIMGFSEEEQLSILKVVSSVLQLGNIV

FKKERNTDQASMPDNTAAQKVCHLMGINVTDFTRSILTPRIKVGRDVVQKAQTKEQADFA

VEALAKATYERLFRWILTRVNKALDKTHRQGASFLGILDIAGFEIFEVNSFEQLCINYTN

EKLQQLFNHTMFILEQEEYQREGIEWNFIDFGLDLQPCIELIERPNNPPGVLALLDEECW

FPKATDKSFVEKLCTEQGSHPKFQKPKQLKDKTEFSIIHYAGKVDYNASAWLTKNMDPLN

DNVTSLLNASSDKFVADLWKDVDRIVGLDQMAKMTESSLPSASKTKKGMFRTVGQLYKEQ

LGKLMTTLRNTTPNFVRCIIPNHEKRSGKLDAFLVLEQLRCNGVLEGIRICRQGFPNRIV

FQEFRQRYEILAANAIPKGFMDGKQACILMIKALELDPNLYRIGQSKIFFRTGVLAHLEE

ERDLKITDVIMAFQAMCRGYLARKAFAKRQQQLTAMKVIQRNCAAYLKLRNWQWWRLFTK

VKPLLQVTRQEEEMQAKEDELQKTKERQQKAENELKELEQKHSQLTEEKNLLQEQLQAET

ELYAEAEEMRVRLAAKKQELEEILHEMEARLEEEEDRGQQLQAERKKMAQQMLDLEEQLE

EEEAARQKLQLEKVTAEAKIKKLEDEILVMDDQNNKLSKERKLLEERISDLTTNLAEEEE

KAKNLTKLKNKHESMISELEVRLKKEEKSRQELEKLKRKLEGDASDFHEQIADLQAQIAE

LKMQLAKKEEELQAALARLDDEIAQKNNALKKIRELEGHISDLQEDLDSERAARNKAEKQ

KRDLGEELEALKTELEDTLDSTATQQELRAKREQEVTVLKKALDEETRSHEAQVQEMRQK

HAQAVEELTEQLEQFKRAKANLDKNKQTLEKENADLAGELRVLGQAKQEVEHKKKKLEAQ

VQELQSKCSDGERARAELNDKVHKLQNEVESVTGMLNEAEGKAIKLAKDVASLSSQLQDT

QELLQEETRQKLNVSTKLRQLEEERNSLQDQLDEEMEAKQNLERHISTLNIQLSDSKKKL

QDFASTVEALEEGKKRFQKEIENLTQQYEEKAAAYDKLEKTKNRLQQELDDLVVDLDNQR

QLVSNLEKKQRKFDQLLAEEKNISSKYADERDRAEAEAREKETKALSLARALEEALEAKE

ELERTNKMLKAEMEDLVSSKDDVGKNVHELEKSKRALETQMEEMKTQLEELEDELQATED

AKLRLEVNMQALKGQFERDLQARDEQNEEKRRQLQRQLHEYETELEDERKQRALAAAAKK

KLEGDLKDLELQADSAIKGREEAIKQLRKLQAQMKDFQRELEDARASRDEIFATAKENEK

KAKSLEADLMQLQEDLAAAERARKQADLEKEELAEELASSLSGRNALQDEKRRLEARIAQ

LEEELEEEQGNMEAMSDRVRKATQQAEQLSNELATERSTAQKNESARQQLERQNKELRSK

LHEMEGAVKSKFKSTIAALEAKIAQLEEQVEQEAREKQAATKSLKQKDKKLKEILLQVED

ERKMAEQYKEQAEKGNARVKQLKRQLEEAEEESQRINANRRKLQRELDEATESNEAMGRE

VNALKSKLRRGNETSFVPSRRSGGRRVIENADGSEEETDTRDADFNGTKASE

>sp|P35749|MYH11_HUMAN 134 YSEKIVDMY

MAQKGQLSDDEKFLFVDKNFINSPVAQADWAAKRLVWVPSEKQGFEAASIKEEKGDEVVV

ELVENGKKVTVGKDDIQKMNPPKFSKVEDMAELTCLNEASVLHNLRERYFSGLIYTYSGL

FCVVVNPYKHLPIYSEKIVDMYKGKKRHEMPPHIYAIADTAYRSMLQDREDQSILCTGES

GAGKTENTKKVIQYLAVVASSHKGKKDTSITGELEKQLLQANPILEAFGNAKTVKNDNSS

RFGKFIRINFDVTGYIVGANIETYLLEKSRAIRQARDERTFHIFYYMIAGAKEKMRSDLL

LEGFNNYTFLSNGFVPIPAAQDDEMFQETVEAMAIMGFSEEEQLSILKVVSSVLQLGNIV

FKKERNTDQASMPDNTAAQKVCHLMGINVTDFTRSILTPRIKVGRDVVQKAQTKEQADFA

VEALAKATYERLFRWILTRVNKALDKTHRQGASFLGILDIAGFEIFEVNSFEQLCINYTN

EKLQQLFNHTMFILEQEEYQREGIEWNFIDFGLDLQPCIELIERPNNPPGVLALLDEECW

FPKATDKSFVEKLCTEQGSHPKFQKPKQLKDKTEFSIIHYAGKVDYNASAWLTKNMDPLN

DNVTSLLNASSDKFVADLWKDVDRIVGLDQMAKMTESSLPSASKTKKGMFRTVGQLYKEQ

LGKLMTTLRNTTPNFVRCIIPNHEKRSGKLDAFLVLEQLRCNGVLEGIRICRQGFPNRIV

FQEFRQRYEILAANAIPKGFMDGKQACILMIKALELDPNLYRIGQSKIFFRTGVLAHLEE

ERDLKITDVIMAFQAMCRGYLARKAFAKRQQQLTAMKVIQRNCAAYLKLRNWQWWRLFTK

VKPLLQVTRQEEEMQAKEDELQKTKERQQKAENELKELEQKHSQLTEEKNLLQEQLQAET

ELYAEAEEMRVRLAAKKQELEEILHEMEARLEEEEDRGQQLQAERKKMAQQMLDLEEQLE

EEEAARQKLQLEKVTAEAKIKKLEDEILVMDDQNNKLSKERKLLEERISDLTTNLAEEEE

KAKNLTKLKNKHESMISELEVRLKKEEKSRQELEKLKRKLEGDASDFHEQIADLQAQIAE

LKMQLAKKEEELQAALARLDDEIAQKNNALKKIRELEGHISDLQEDLDSERAARNKAEKQ

KRDLGEELEALKTELEDTLDSTATQQELRAKREQEVTVLKKALDEETRSHEAQVQEMRQK

HAQAVEELTEQLEQFKRAKANLDKNKQTLEKENADLAGELRVLGQAKQEVEHKKKKLEAQ

VQELQSKCSDGERARAELNDKVHKLQNEVESVTGMLNEAEGKAIKLAKDVASLSSQLQDT

QELLQEETRQKLNVSTKLRQLEEERNSLQDQLDEEMEAKQNLERHISTLNIQLSDSKKKL

QDFASTVEALEEGKKRFQKEIENLTQQYEEKAAAYDKLEKTKNRLQQELDDLVVDLDNQR

QLVSNLEKKQRKFDQLLAEEKNISSKYADERDRAEAEAREKETKALSLARALEEALEAKE

ELERTNKMLKAEMEDLVSSKDDVGKNVHELEKSKRALETQMEEMKTQLEELEDELQATED

AKLRLEVNMQALKGQFERDLQARDEQNEEKRRQLQRQLHEYETELEDERKQRALAAAAKK

KLEGDLKDLELQADSAIKGREEAIKQLRKLQAQMKDFQRELEDARASRDEIFATAKENEK

KAKSLEADLMQLQEDLAAAERARKQADLEKEELAEELASSLSGRNALQDEKRRLEARIAQ

LEEELEEEQGNMEAMSDRVRKATQQAEQLSNELATERSTAQKNESARQQLERQNKELRSK

LHEMEGAVKSKFKSTIAALEAKIAQLEEQVEQEAREKQAATKSLKQKDKKLKEILLQVED

ERKMAEQYKEQAEKGNARVKQLKRQLEEAEEESQRINANRRKLQRELDEATESNEAMGRE

VNALKSKLRRGNETSFVPSRRSGGRRVIENADGSEEETDTRDADFNGTKASE

>sp|P24928|RPB1_HUMAN 1380 RELYHVISF

MHGGGPPSGDSACPLRTIKRVQFGVLSPDELKRMSVTEGGIKYPETTEGGRPKLGGLMDP

RQGVIERTGRCQTCAGNMTECPGHFGHIELAKPVFHVGFLVKTMKVLRCVCFFCSKLLVD

SNNPKIKDILAKSKGQPKKRLTHVYDLCKGKNICEGGEEMDNKFGVEQPEGDEDLTKEKG

HGGCGRYQPRIRRSGLELYAEWKHVNEDSQEKKILLSPERVHEIFKRISDEECFVLGMEP

RYARPEWMIVTVLPVPPLSVRPAVVMQGSARNQDDLTHKLADIVKINNQLRRNEQNGAAA

HVIAEDVKLLQFHVATMVDNELPGLPRAMQKSGRPLKSLKQRLKGKEGRVRGNLMGKRVD

FSARTVITPDPNLSIDQVGVPRSIAANMTFAEIVTPFNIDRLQELVRRGNSQYPGAKYII

RDNGDRIDLRFHPKPSDLHLQTGYKVERHMCDGDIVIFNRQPTLHKMSMMGHRVRILPWS

TFRLNLSVTTPYNADFDGDEMNLHLPQSLETRAEIQELAMVPRMIVTPQSNRPVMGIVQD

TLTAVRKFTKRDVFLERGEVMNLLMFLSTWDGKVPQPAILKPRPLWTGKQIFSLIIPGHI

NCIRTHSTHPDDEDSGPYKHISPGDTKVVVENGELIMGILCKKSLGTSAGSLVHISYLEM

GHDITRLFYSNIQTVINNWLLIEGHTIGIGDSIADSKTYQDIQNTIKKAKQDVIEVIEKA

HNNELEPTPGNTLRQTFENQVNRILNDARDKTGSSAQKSLSEYNNFKSMVVSGAKGSKIN

ISQVIAVVGQQNVEGKRIPFGFKHRTLPHFIKDDYGPESRGFVENSYLAGLTPTEFFFHA

MGGREGLIDTAVKTAETGYIQRRLIKSMESVMVKYDATVRNSINQVVQLRYGEDGLAGES

VEFQNLATLKPSNKAFEKKFRFDYTNERALRRTLQEDLVKDVLSNAHIQNELEREFERMR

EDREVLRVIFPTGDSKVVLPCNLLRMIWNAQKIFHINPRLPSDLHPIKVVEGVKELSKKL

VIVNGDDPLSRQAQENATLLFNIHLRSTLCSRRMAEEFRLSGEAFDWLLGEIESKFNQAI

AHPGEMVGALAAQSLGEPATQMTLNTFHYAGVSAKNVTLGVPRLKELINISKKPKTPSLT

VFLLGQSARDAERAKDILCRLEHTTLRKVTANTAIYYDPNPQSTVVAEDQEWVNVYYEMP

DFDVARISPWLLRVELDRKHMTDRKLTMEQIAEKINAGFGDDLNCIFNDDNAEKLVLRIR

IMNSDENKMQEEEEVVDKMDDDVFLRCIESNMLTDMTLQGIEQISKVYMHLPQTDNKKKI

IITEDGEFKALQEWILETDGVSLMRVLSEKDVDPVRTTSNDIVEIFTVLGIEAVRKALER

ELYHVISFDGSYVNYRHLALLCDTMTCRGHLMAITRHGVNRQDTGPLMKCSFEETVDVLM

EAAAHGESDPMKGVSENIMLGQLAPAGTGCFDLLLDAEKCKYGMEIPTNIPGLGAAGPTG

MFFGSAPSPMGGISPAMTPWNQGATPAYGAWSPSVGSGMTPGAAGFSPSAASDASGFSPG

YSPAWSPTPGSPGSPGPSSPYIPSPGGAMSPSYSPTSPAYEPRSPGGYTPQSPSYSPTSP

SYSPTSPSYSPTSPNYSPTSPSYSPTSPSYSPTSPSYSPTSPSYSPTSPSYSPTSPSYSP

TSPSYSPTSPSYSPTSPSYSPTSPSYSPTSPSYSPTSPSYSPTSPSYSPTSPSYSPTSPS

YSPTSPNYSPTSPNYTPTSPSYSPTSPSYSPTSPNYTPTSPNYSPTSPSYSPTSPSYSPT

SPSYSPSSPRYTPQSPTYTPSSPSYSPSSPSYSPTSPKYTPTSPSYSPSSPEYTPTSPKY

SPTSPKYSPTSPKYSPTSPTYSPTTPKYSPTSPTYSPTSPVYTPTSPKYSPTSPTYSPTS

PKYSPTSPTYSPTSPKGSTYSPTSPGYSPTSPTYSLTSPAISPDDSDEEN

>sp|P24928|RPB1_HUMAN 527 TPQSNRPVM

MHGGGPPSGDSACPLRTIKRVQFGVLSPDELKRMSVTEGGIKYPETTEGGRPKLGGLMDP

RQGVIERTGRCQTCAGNMTECPGHFGHIELAKPVFHVGFLVKTMKVLRCVCFFCSKLLVD

SNNPKIKDILAKSKGQPKKRLTHVYDLCKGKNICEGGEEMDNKFGVEQPEGDEDLTKEKG

HGGCGRYQPRIRRSGLELYAEWKHVNEDSQEKKILLSPERVHEIFKRISDEECFVLGMEP

RYARPEWMIVTVLPVPPLSVRPAVVMQGSARNQDDLTHKLADIVKINNQLRRNEQNGAAA

HVIAEDVKLLQFHVATMVDNELPGLPRAMQKSGRPLKSLKQRLKGKEGRVRGNLMGKRVD

FSARTVITPDPNLSIDQVGVPRSIAANMTFAEIVTPFNIDRLQELVRRGNSQYPGAKYII

RDNGDRIDLRFHPKPSDLHLQTGYKVERHMCDGDIVIFNRQPTLHKMSMMGHRVRILPWS

TFRLNLSVTTPYNADFDGDEMNLHLPQSLETRAEIQELAMVPRMIVTPQSNRPVMGIVQD

TLTAVRKFTKRDVFLERGEVMNLLMFLSTWDGKVPQPAILKPRPLWTGKQIFSLIIPGHI

NCIRTHSTHPDDEDSGPYKHISPGDTKVVVENGELIMGILCKKSLGTSAGSLVHISYLEM

GHDITRLFYSNIQTVINNWLLIEGHTIGIGDSIADSKTYQDIQNTIKKAKQDVIEVIEKA

HNNELEPTPGNTLRQTFENQVNRILNDARDKTGSSAQKSLSEYNNFKSMVVSGAKGSKIN

ISQVIAVVGQQNVEGKRIPFGFKHRTLPHFIKDDYGPESRGFVENSYLAGLTPTEFFFHA

MGGREGLIDTAVKTAETGYIQRRLIKSMESVMVKYDATVRNSINQVVQLRYGEDGLAGES

VEFQNLATLKPSNKAFEKKFRFDYTNERALRRTLQEDLVKDVLSNAHIQNELEREFERMR

EDREVLRVIFPTGDSKVVLPCNLLRMIWNAQKIFHINPRLPSDLHPIKVVEGVKELSKKL

VIVNGDDPLSRQAQENATLLFNIHLRSTLCSRRMAEEFRLSGEAFDWLLGEIESKFNQAI

AHPGEMVGALAAQSLGEPATQMTLNTFHYAGVSAKNVTLGVPRLKELINISKKPKTPSLT

VFLLGQSARDAERAKDILCRLEHTTLRKVTANTAIYYDPNPQSTVVAEDQEWVNVYYEMP

DFDVARISPWLLRVELDRKHMTDRKLTMEQIAEKINAGFGDDLNCIFNDDNAEKLVLRIR

IMNSDENKMQEEEEVVDKMDDDVFLRCIESNMLTDMTLQGIEQISKVYMHLPQTDNKKKI

IITEDGEFKALQEWILETDGVSLMRVLSEKDVDPVRTTSNDIVEIFTVLGIEAVRKALER

ELYHVISFDGSYVNYRHLALLCDTMTCRGHLMAITRHGVNRQDTGPLMKCSFEETVDVLM

EAAAHGESDPMKGVSENIMLGQLAPAGTGCFDLLLDAEKCKYGMEIPTNIPGLGAAGPTG

MFFGSAPSPMGGISPAMTPWNQGATPAYGAWSPSVGSGMTPGAAGFSPSAASDASGFSPG

YSPAWSPTPGSPGSPGPSSPYIPSPGGAMSPSYSPTSPAYEPRSPGGYTPQSPSYSPTSP

SYSPTSPSYSPTSPNYSPTSPSYSPTSPSYSPTSPSYSPTSPSYSPTSPSYSPTSPSYSP

TSPSYSPTSPSYSPTSPSYSPTSPSYSPTSPSYSPTSPSYSPTSPSYSPTSPSYSPTSPS

YSPTSPNYSPTSPNYTPTSPSYSPTSPSYSPTSPNYTPTSPNYSPTSPSYSPTSPSYSPT

SPSYSPSSPRYTPQSPTYTPSSPSYSPSSPSYSPTSPKYTPTSPSYSPSSPEYTPTSPKY

SPTSPKYSPTSPKYSPTSPTYSPTTPKYSPTSPTYSPTSPVYTPTSPKYSPTSPTYSPTS

PKYSPTSPTYSPTSPKGSTYSPTSPGYSPTSPTYSLTSPAISPDDSDEEN

>sp|P35579|MYH9_HUMAN 345 GVLQLGNIVFK

MAQQAADKYLYVDKNFINNPLAQADWAAKKLVWVPSDKSGFEPASLKEEVGEEAIVELVE

NGKKVKVNKDDIQKMNPPKFSKVEDMAELTCLNEASVLHNLKERYYSGLIYTYSGLFCVV

INPYKNLPIYSEEIVEMYKGKKRHEMPPHIYAITDTAYRSMMQDREDQSILCTGESGAGK

TENTKKVIQYLAYVASSHKSKKDQGELERQLLQANPILEAFGNAKTVKNDNSSRFGKFIR

INFDVNGYIVGANIETYLLEKSRAIRQAKEERTFHIFYYLLSGAGEHLKTDLLLEPYNKY

RFLSNGHVTIPGQQDKDMFQETMEAMRIMGIPEEEQMGLLRVISGVLQLGNIVFKKERNT

DQASMPDNTAAQKVSHLLGINVTDFTRGILTPRIKVGRDYVQKAQTKEQADFAIEALAKA

TYERMFRWLVLRINKALDKTKRQGASFIGILDIAGFEIFDLNSFEQLCINYTNEKLQQLF

NHTMFILEQEEYQREGIEWNFIDFGLDLQPCIDLIEKPAGPPGILALLDEECWFPKATDK

SFVEKVMQEQGTHPKFQKPKQLKDKADFCIIHYAGKVDYKADEWLMKNMDPLNDNIATLL

HQSSDKFVSELWKDVDRIIGLDQVAGMSETALPGAFKTRKGMFRTVGQLYKEQLAKLMAT

LRNTNPNFVRCIIPNHEKKAGKLDPHLVLDQLRCNGVLEGIRICRQGFPNRVVFQEFRQR

YEILTPNSIPKGFMDGKQACVLMIKALELDSNLYRIGQSKVFFRAGVLAHLEEERDLKIT

DVIIGFQACCRGYLARKAFAKRQQQLTAMKVLQRNCAAYLKLRNWQWWRLFTKVKPLLQV

SRQEEEMMAKEEELVKVREKQLAAENRLTEMETLQSQLMAEKLQLQEQLQAETELCAEAE

ELRARLTAKKQELEEICHDLEARVEEEEERCQHLQAEKKKMQQNIQELEEQLEEEESARQ

KLQLEKVTTEAKLKKLEEEQIILEDQNCKLAKEKKLLEDRIAEFTTNLTEEEEKSKSLAK

LKNKHEAMITDLEERLRREEKQRQELEKTRRKLEGDSTDLSDQIAELQAQIAELKMQLAK

KEEELQAALARVEEEAAQKNMALKKIRELESQISELQEDLESERASRNKAEKQKRDLGEE

LEALKTELEDTLDSTAAQQELRSKREQEVNILKKTLEEEAKTHEAQIQEMRQKHSQAVEE

LAEQLEQTKRVKANLEKAKQTLENERGELANEVKVLLQGKGDSEHKRKKVEAQLQELQVK

FNEGERVRTELADKVTKLQVELDNVTGLLSQSDSKSSKLTKDFSALESQLQDTQELLQEE

NRQKLSLSTKLKQVEDEKNSFREQLEEEEEAKHNLEKQIATLHAQVADMKKKMEDSVGCL

ETAEEVKRKLQKDLEGLSQRHEEKVAAYDKLEKTKTRLQQELDDLLVDLDHQRQSACNLE

KKQKKFDQLLAEEKTISAKYAEERDRAEAEAREKETKALSLARALEEAMEQKAELERLNK

QFRTEMEDLMSSKDDVGKSVHELEKSKRALEQQVEEMKTQLEELEDELQATEDAKLRLEV

NLQAMKAQFERDLQGRDEQSEEKKKQLVRQVREMEAELEDERKQRSMAVAARKKLEMDLK

DLEAHIDSANKNRDEAIKQLRKLQAQMKDCMRELDDTRASREEILAQAKENEKKLKSMEA

EMIQLQEELAAAERAKRQAQQERDELADEIANSSGKGALALEEKRRLEARIAQLEEELEE

EQGNTELINDRLKKANLQIDQINTDLNLERSHAQKNENARQQLERQNKELKVKLQEMEGT

VKSKYKASITALEAKIAQLEEQLDNETKERQAACKQVRRTEKKLKDVLLQVDDERRNAEQ

YKDQADKASTRLKQLKRQLEEAEEEAQRANASRRKLQRELEDATETADAMNREVSSLKNK

LRRGDLPFVVPRRMARKGAGDGSDEEVDGKADGAEAKPAE

>sp|Q9P2E3|ZNFX1_HUMAN 1009 AEVLEAHTI

MEERRPHLDARPRNSHTNHRGPVDGELPPRARNQANNPPANALRGGASHPGRHPRANNHP

AAYWQREERFRAMGRNPHQGRRNQEGHASDEARDQRHDQENDTRWRNGNQDCRNRRPPWS

NDNFQQWRTPHQKPTEQPQQAKKLGYKFLESLLQKDPSEVVITLATSLGLKELLSHSSMK

SNFLELICQVLRKACSSKMDRQSVLHVLGILKNSKFLKVCLPAYVVGMITEPIPDIRNQY

PEHISNIISLLQDLVSVFPASSVQETSMLVSLLPTSLNALRASGVDIEEETEKNLEKVQT

IIEHLQEKRREGTLRVDTYTLVQPEAEDHVESYRTMPIYPTYNEVHLDERPFLRPNIISG

KYDSTAIYLDTHFRLLREDFVRPLREGILELLQSFEDQGLRKRKFDDIRIYFDTRIITPM

CSSSGIVYKVQFDTKPLKFVRWQNSKRLLYGSLVCMSKDNFETFLFATVSNREQEDLCRG

IVQLCFNEQSQQLLAEVQPSDSFLMVETTAYFEAYRHVLEGLQEVQEEDVPFQRNIVECN

SHVKEPRYLLMGGRYDFTPLIENPSATGEFLRNVEGLRHPRINVLDPGQWPSKEALKLDD

SQMEALQFALTRELAIIQGPPGTGKTYVGLKIVQALLTNESVWQISLQKFPILVVCYTNH

ALDQFLEGIYNCQKTSIVRVGGRSNSEILKQFTLRELRNKREFRRNLPMHLRRAYMSIMT

QMKESEQELHEGAKTLECTMRGVLREQYLQKYISPQHWESLMNGPVQDSEWICFQHWKHS

MMLEWLGLGVGSFTQSVSPAGPENTAQAEGDEEEEGEEESSLIEIAEEADLIQADRVIEE

EEVVRPQRRKKEESGADQELAKMLLAMRLDHCGTGTAAGQEQATGEWQTQRNQKKKMKKR

VKDELRKLNTMTAAEANEIEDVWQLDLSSRWQLYRLWLQLYQADTRRKILSYERQYRTSA

ERMAELRLQEDLHILKDAQVVGMTTTGAAKYRQILQKVEPRIVIVEEAAEVLEAHTIATL

SKACQHLILIGDHQQLRPSANVYDLAKNFNLEVSLFERLVKVNIPFVRLNYQHRMCPEIA

RLLTPHIYQDLENHPSVLKYEKIKGVSSNLFFVEHNFPEQEIQEGKSHQNQHEAHFVVEL

CKYFLCQEYLPSQITILTTYTGQLFCLRKLMPAKTFAGVRVHVVDKYQGEENDIILLSLV

RSNQEGKVGFLQISNRICVALSRAKKGMYCIGNMQMLAKVPLWSKIIHTLRENNQIGPML

RLCCQNHPETHTLVSKASDFQKVPEGGCSLPCEFRLGCGHVCTRACHPYDSSHKEFQCMK

PCQKVICQEGHRCPLVCFQECQPCQVKVPKTIPRCGHEQMVPCSVPESDFCCQEPCSKSL

RCGHRCSHPCGEDCVQLCSEMVTIKLKCGHSQPVKCGHVEGLLYGGLLVKCTTKCGTILD

CGHPCPGSCHSCFEGRFHERCQQPCKRLLICSHKCQEPCIGECPPCQRTCQNRCVHSQCK

KKCGELCSPCVEPCVWRCQHYQCTKLCSEPCNRPPCYVPCTKLLVCGHPCIGLCGEPCPK

KCRICHMDEVTQIFFGFEDEPDARFVQLEDCSHIFEVQALDRYMNEQKDDEVAIRLKVCP

ICQVPIRKNLRYGTSIKQRLEEIEIIKEKIQGSAGEIATSQERLKALLERKSLLHQLLPE

DFLMLKEKLAQKNLSVKDLGLVENYISFYDHLASLWDSLKKMHVLEEKRVRTRLEQVHEW

LAKKRLSFTSQELSDLRSEIQRLTYLVNLLTRYKIAEKKVKDSIAVEVYSVQNILEKTCK

FTQEDEQLVQEKMEALKATLPCSGLGISEEERVQIVSAIGYPRGHWFKCRNGHIYVIGDC

GGAMERGTCPDCKEVIGGTNHTLERSNQLASEMDGAQHAAWSDTANNLMNFEEIQGMM

>sp|Q9P2P1|K1305_HUMAN 1487 TLADIIARL

MLLSGGDPPAQEWFMVQTKSKPRVQRQRLQVQRIFRVKLNAFQSRPDTPYFWLQLEGPRE

NMGKAKEYLKGLCSPELWKEVRYPPILHCAFLGAQGLFLDCLCWSTLAYLVPGPPGSLMV

GGLTESFIMTQNWLEELVGRLRWGPAPLLTPRGIWEAEVTRAFGALVWIRGDQHAGDLLQ

LPPAVQELLLSLVRDAAGKEDIIEWLSRFGISDSHSDPEVLICPPQQQKEAPAMVSVGES

PGPFVDMGTLQNRGPENSKRLSSLGATGSLITAQSTPQEAANQLVRVGSNNQDGMDSAQE

EGTVQATSSQDSTNHTQALLKQRQVQKIEDKLLFQPPVSALGVCPPWKAWTPGPAFGPLW

PGAIAATFWRINELHSLHLAWLLSQACFNFPFWQRPLGPIQLKLPGQNPLPLNLEWKQKE

LAPLPSAESPAGRPDGGLGGEAALQNCPRPEISPKVTSLLVVPGSSDVKDKVSSDLPQIG

PPLTSTPQLQAGGEPGDQGSMQLDFKGLEEGPAPVLPTGQGKPVAQGGLTDQSVPGAQTV

PETLKVPMAAAVPKAENPSRTQVPSAAPKLPTSRMMLAVHTEPAAPEVPLAPTKPTAQLM

ATAQKTVVNQPVLVAQVEPTTPKTPQAQKMPVAKTSPAGPKTPKAQAGPAATVSKAPAAS

KAPAAPKVPVTPRVSRAPKTPAAQKVPTDAGPTLDVARLLSEVQPTSRASVSLLKGQGQA

GRQGPQSSGTLALSSKHQFQMEGLLGAWEGAPRQPPRHLQANSTVTSFQRYHEALNTPFE

LNLSGEPGNQGLRRVVIDGSSVAMVHGLQHFFSCRGIAMAVQFFWNRGHREVTVFVPTWQ

LKKNRRVRESHFLTKLHSLKMLSITPSQLENGKKITTYDYRFMVKLAEETDGIIVTNEQI

HILMNSSKKLMVKDRLLPFTFAGNLFMVPDDPLGRDGPTLDEFLKKPNRLDTDIGNFLKV

WKTLPPSSASVTELSDDADSGPLESLPNMEEVREEKEERQDEEQRQGQGTQKAAEEDDLD

SSLASVFRVECPSLSEEILRCLSLHDPPDGALDIDLLPGAASPYLGIPWDGKAPCQQVLA

HLAQLTIPSNFTALSFFMGFMDSHRDAIPDYEALVGPLHSLLKQKPDWQWDQEHEEAFLA

LKRALVSALCLMAPNSQLPFRLEVTVSHVALTAILHQEHSGRKHPIAYTSKPLLPDEESQ

GPQSGGDSPYAVAWALKHFSRCIGDTPVVLDLSYASRTTADPEVREGRRVSKAWLIRWSL

LVQDKGKRALELALLQGLLGENRLLTPAASMPRFFQVLPPFSDLSTFVCIHMSGYCFYRE

DEWCAGFGLYVLSPTSPPVSLSFSCSPYTPTYAHLAAVACGLERFGQSPLPVVFLTHCNW

IFSLLWELLPLWRARGFLSSDGAPLPHPSLLSYIISLTSGLSSLPFIYRTSYRGSLFAVT

VDTLAKQGAQGGGQWWSLPKDVPAPTVSPHAMGKRPNLLALQLSDSTLADIIARLQAGQK

LSGSSPFSSAFNSLSLDKESGLLMFKGDKKPRVWVVPTQLRRDLIFSVHDIPLGAHQRPE

ETYKKLRLLGWWPGMQEHVKDYCRSCLFCIPRNLIGSELKVIESPWPLRSTAPWSNLQIE

VVGPVTISEEGHKHVLIVADPNTRWVEAFPLKPYTHTAVAQVLLQHVFARWGVPVRLEAA

QGPQFARHVLVSCGLALGAQVASLSRDLQFPCLTSSGAYWEFKRALKEFIFLHGKKWAAS

LPLLHLAFRASSTDATPFKVLTGGESRLTEPLWWEMSSANIEGLKMDVFLLQLVGELLEL

HWRVADKASEKAENRRFKRESQEKEWNVGDQVLLLSLPRNGSSAKWVGPFYIGDRLSLSL

YRIWGFPTPEKLGCIYPSSLMKAFAKSGTPLSFKVLEQ

>sp|Q6ZT12|UBR3_HUMAN 1651 LPFLRITSL

MAAAAAAAVGGQQPSQPELPAPGLALDKAATAAHLKAALSRPDNRAGAEELQALLERVLS

AERPLAAAAGGEDAAAAGGGGGPGAAEEEALEWCKCLLAGGGGYDEFCAAVRAYDPAALC

GLVWTANFVAYRCRTCGISPCMSLCAECFHQGDHTGHDFNMFRSQAGGACDCGDSNVMRE

SGFCKRHQIKSSSNIPCVPKDLLMMSEFVLPRFIFCLIQYLREGYNEPAADGPSEKDLNK

VLQLLEPQISFLEDLTKMGGAMRSVLTQVLTNQQNYKDLTSGLGENACVKKSHEKYLIAL

KSSGLTYPEDKLVYGVQEPSAGTSSLAVQGFIGATGTLGQVDSSDEDDQDGSQGLGKRKR

VKLSSGTKDQSIMDVLKHKSFLEELLFWTIKYEFPQKMVTFLLNMLPDQEYKVAFTKTFV

QHYAFIMKTLKKSHESDTMSNRIVHISVQLFSNEELARQVTEECQLLDIMVTVLLYMMES

CLIKSELQDEENSLHVVVNCGEALLKNNTYWPLVSDFINILSHQSVAKRFLEDHGLLVTW

MNFVSFFQGMNLNKRELNEHVEFESQTYYAAFAAELEACAQPMWGLLSHCKVRETQEYTR

NVVRYCLEALQDWFDAINFVDEPAPNQVTFHLPLHRYYAMFLSKAVKCQELDLDSVLPDQ

EMLMKLMIHPLQIQASLAEIHSNMWVRNGLQIKGQAMTYVQSHFCNSMIDPDIYLLQVCA

SRLDPDYFISSVFERFKVVDLLTMASQHQNTVLDAEHERSMLEGALTFLVILLSLRLHLG

MSDDEILRAEMVAQLCMNDRTHSSLLDLIPENPNPKSGIIPGSYSFESVLSAVADFKAPV

FEPGGSMQQGMYTPKAEVWDQEFDPVMVILRTVYRRDVQSAMDRYTAFLKQSGKFPGNPW

PPYKKRTSLHPSYKGLMRLLHCKTLHIVLFTLLYKILMDHQNLSEHVLCMVLYLIELGLE

NSAEEESDEEASVGGPERCHDSWFPGSNLVSNMRHFINYVRVRVPETAPEVKRDSPASTS

SDNLGSLQNSGTAQVFSLVAERRKKFQEIINRSSSEANQVVRPKTSSKWSAPGSAPQLTT

AILEIKESILSLLIKLHHKLSGKQNSYYPPWLDDIEILIQPEIPKYSHGDGITAVERILL

KAASQSRMNKRIIEEICRKVTPPVPPKKVTAAEKKTLDKEERRQKARERQQKLLAEFASR

QKSFMETAMDVDSPENDIPMEITTAEPQVSEAVYDCVICGQSGPSSEDRPTGLVVLLQAS

SVLGQCRDNVEPKKLPISEEEQIYPWDTCAAVHDVRLSLLQRYFKDSSCLLAVSIGWEGG

VYVQTCGHTLHIDCHKSYMESLRNDQVLQGFSVDKGEFTCPLCRQFANSVLPCYPGSNVE

NNPWQRPSNKSIQDLIKEVEELQGRPGAFPSETNLSKEMESVMKDIKNTTQKKYRDYSKT

PGSPDNDFLFMYSVARTNLELELIHRGGNLCSGGASTAGKRSCLNQLFHVLALHMRLYSI

DSEYNPWRKLTQLEEMNPQLGYEEQQPEVPILYHDVTSLLLIQILMMPQPLRKDHFTCIV

KVLFTLLYTQALAALSVKCSEEDRSAWKHAGALKKSTCDAEKSYEVLLSFVISELFKGKL

YHEEGTQECAMVNPIAWSPESMEKCLQDFCLPFLRITSLLQHHLFGEDLPSCQEEEEFSV

LASCLGLLPTFYQTEHPFISASCLDWPVPAFDIITQWCFEIKSFTERHAEQGKALLIQES

KWKLPHLLQLPENYNTIFQYYHRKTCSVCTKVPKDPAVCLVCGTFVCLKGLCCKQQSYCE

CVLHSQNCGAGTGIFLLINASVIIIIRGHRFCLWGSVYLDAHGEEDRDLRRGKPLYICKE

RYKVLEQQWISHTFDHINKRWGPHYNGL

>sp|Q8TEM1|PO210_HUMAN 547 ARVGQALEL

MAARGRGLLLLTLSVLLAAGPSAAAAKLNIPKVLLPFTRATRVNFTLEASEGCYRWLSTR

PEVASIEPLGLDEQQCSQKAVVQARLTQPARLTSIIFAEDITTGQVLRCDAIVDLIHDIQ

IVSTTRELYLEDSPLELKIQALDSEGNTFSTLAGLVFEWTIVKDSEADRFSDSHNALRIL

TFLESTYIPPSYISEMEKAAKQGDTILVSGMKTGSSKLKARIQEAVYKNVRPAEVRLLIL

ENILLNPAYDVYLMVGTSIHYKVQKIRQGKITELSMPSDQYELQLQNSIPGPEGDPARPV

AVLAQDTSMVTALQLGQSSLVLGHRSIRMQGASRLPNSTIYVVEPGYLGFTVHPGDRWVL

ETGRLYEITIEVFDKFSNKVYVSDNIRIETVLPAEFFEVLSSSQNGSYHRIRALKRGQTA

IDAALTSVVDQDGGVHILQVPVWNQQEVEIHIPITLYPSILTFPWQPKTGAYQYTIRAHG

GSGNFSWSSSSHLVATVTVKGVMTTGSDIGFSVIQAHDVQNPLHFGEMKVYVIEPHSMEF

APCQVEARVGQALELPLRISGLMPGGASEVVTLSDCSHFDLAVEVENQGVFQPLPGRLPP

GSEHCSGIRVKAEAQGSTTLLVSYRHGHVHLSAKITIAAYLPLKAVDPSSVALVTLGSSK

EMLFEGGPRPWILEPSKFFQNVTAEDTDSIGLALFAPHSSRNYQQHWILVTCQALGEQVI

ALSVGNKPSLTNPFPAVEPAVVKFVCAPPSRLTLAPVYTSPQLDMSCPLLQQNKQVVPVS

SHRNPRLDLAAYDQEGRRFDNFSSLSIQWESTRPVLASIEPELPMQLVSQDDESGQKKLH

GLQAILVHEASGTTAITATATGYQESHLSSARTKQPHDPLVPLSASIELILVEDVRVSPE

EVTIYNHPGIQAELRIREGSGYFFLNTSTADVVKVAYQEARGVAMVHPLLPGSSTIMIHD

LCLVFPAPAKAVVYVSDIQELYIRVVDKVEIGKTVKAYVRVLDLHKKPFLAKYFPFMDLK

LRAASPIITLVALDEALDNYTITFLIRGVAIGQTSLTASVTNKAGQRINSAPQQIEVFPP

FRLMPRKVTLLIGATMQVTSEGGPQPQSNILFSISNESVALVSAAGLVQGLAIGNGTVSG

LVQAVDAETGKVVIISQDLVQVEVLLLRAVRIRAPIMRMRTGTQMPIYVTGITNHQNPFS

FGNAVPGLTFHWSVTKRDVLDLRGRHHEASIRLPSQYNFAMNVLGRVKGRTGLRVVVKAV

DPTSGQLYGLARELSDEIQVQVFEKLQLLNPEIEAEQILMSPNSYIKLQTNRDGAASLSY

RVLDGPEKVPVVHVDEKGFLASGSMIGTSTIEVIAQEPFGANQTIIVAVKVSPVSYLRVS

MSPVLHTQNKEALVAVPLGMTVTFTVHFHDNSGDVFHAHSSVLNFATNRDDFVQIGKGPT

NNTCVVRTVSVGLTLLRVWDAEHPGLSDFMPLPVLQAISPELSGAMVVGDVLCLATVLTS

LEGLSGTWSSSANSILHIDPKTGVAVARAVGSVTVYYEVAGHLRTYKEVVVSVPQRIMAR

HLHPIQTSFQEATASKVIVAVGDRSSNLRGECTPTQREVIQALHPETLISCQSQFKPAVF

DFPSQDVFTVEPQFDTALGQYFCSITMHRLTDKQRKHLSMKKTALVVSASLSSSHFSTEQ

VGAEVPFSPGLFADQAEILLSNHYTSSEIRVFGAPEVLENLEVKSGSPAVLAFAKEKSFG

WPSFITYTVGVLDPAAGSQGPLSTTLTFSSPVTNQAIAIPVTVAFVVDRRGPGPYGASLF

QHFLDSYQVMFFTLFALLAGTAVMIIAYHTVCTPRDLAVPAALTPRASPGHSPHYFAASS

PTSPNALPPARKASPPSGLWSPAYASH

>sp|Q8TEM1|PO210_HUMAN 1245 GRVKGRTGL

MAARGRGLLLLTLSVLLAAGPSAAAAKLNIPKVLLPFTRATRVNFTLEASEGCYRWLSTR

PEVASIEPLGLDEQQCSQKAVVQARLTQPARLTSIIFAEDITTGQVLRCDAIVDLIHDIQ

IVSTTRELYLEDSPLELKIQALDSEGNTFSTLAGLVFEWTIVKDSEADRFSDSHNALRIL

TFLESTYIPPSYISEMEKAAKQGDTILVSGMKTGSSKLKARIQEAVYKNVRPAEVRLLIL

ENILLNPAYDVYLMVGTSIHYKVQKIRQGKITELSMPSDQYELQLQNSIPGPEGDPARPV

AVLAQDTSMVTALQLGQSSLVLGHRSIRMQGASRLPNSTIYVVEPGYLGFTVHPGDRWVL

ETGRLYEITIEVFDKFSNKVYVSDNIRIETVLPAEFFEVLSSSQNGSYHRIRALKRGQTA

IDAALTSVVDQDGGVHILQVPVWNQQEVEIHIPITLYPSILTFPWQPKTGAYQYTIRAHG

GSGNFSWSSSSHLVATVTVKGVMTTGSDIGFSVIQAHDVQNPLHFGEMKVYVIEPHSMEF

APCQVEARVGQALELPLRISGLMPGGASEVVTLSDCSHFDLAVEVENQGVFQPLPGRLPP

GSEHCSGIRVKAEAQGSTTLLVSYRHGHVHLSAKITIAAYLPLKAVDPSSVALVTLGSSK

EMLFEGGPRPWILEPSKFFQNVTAEDTDSIGLALFAPHSSRNYQQHWILVTCQALGEQVI

ALSVGNKPSLTNPFPAVEPAVVKFVCAPPSRLTLAPVYTSPQLDMSCPLLQQNKQVVPVS

SHRNPRLDLAAYDQEGRRFDNFSSLSIQWESTRPVLASIEPELPMQLVSQDDESGQKKLH

GLQAILVHEASGTTAITATATGYQESHLSSARTKQPHDPLVPLSASIELILVEDVRVSPE

EVTIYNHPGIQAELRIREGSGYFFLNTSTADVVKVAYQEARGVAMVHPLLPGSSTIMIHD

LCLVFPAPAKAVVYVSDIQELYIRVVDKVEIGKTVKAYVRVLDLHKKPFLAKYFPFMDLK

LRAASPIITLVALDEALDNYTITFLIRGVAIGQTSLTASVTNKAGQRINSAPQQIEVFPP

FRLMPRKVTLLIGATMQVTSEGGPQPQSNILFSISNESVALVSAAGLVQGLAIGNGTVSG

LVQAVDAETGKVVIISQDLVQVEVLLLRAVRIRAPIMRMRTGTQMPIYVTGITNHQNPFS

FGNAVPGLTFHWSVTKRDVLDLRGRHHEASIRLPSQYNFAMNVLGRVKGRTGLRVVVKAV

DPTSGQLYGLARELSDEIQVQVFEKLQLLNPEIEAEQILMSPNSYIKLQTNRDGAASLSY

RVLDGPEKVPVVHVDEKGFLASGSMIGTSTIEVIAQEPFGANQTIIVAVKVSPVSYLRVS

MSPVLHTQNKEALVAVPLGMTVTFTVHFHDNSGDVFHAHSSVLNFATNRDDFVQIGKGPT

NNTCVVRTVSVGLTLLRVWDAEHPGLSDFMPLPVLQAISPELSGAMVVGDVLCLATVLTS

LEGLSGTWSSSANSILHIDPKTGVAVARAVGSVTVYYEVAGHLRTYKEVVVSVPQRIMAR

HLHPIQTSFQEATASKVIVAVGDRSSNLRGECTPTQREVIQALHPETLISCQSQFKPAVF

DFPSQDVFTVEPQFDTALGQYFCSITMHRLTDKQRKHLSMKKTALVVSASLSSSHFSTEQ

VGAEVPFSPGLFADQAEILLSNHYTSSEIRVFGAPEVLENLEVKSGSPAVLAFAKEKSFG

WPSFITYTVGVLDPAAGSQGPLSTTLTFSSPVTNQAIAIPVTVAFVVDRRGPGPYGASLF

QHFLDSYQVMFFTLFALLAGTAVMIIAYHTVCTPRDLAVPAALTPRASPGHSPHYFAASS

PTSPNALPPARKASPPSGLWSPAYASH

>sp|Q8TEM1|PO210_HUMAN 1231 IRLPSQYNF

MAARGRGLLLLTLSVLLAAGPSAAAAKLNIPKVLLPFTRATRVNFTLEASEGCYRWLSTR

PEVASIEPLGLDEQQCSQKAVVQARLTQPARLTSIIFAEDITTGQVLRCDAIVDLIHDIQ

IVSTTRELYLEDSPLELKIQALDSEGNTFSTLAGLVFEWTIVKDSEADRFSDSHNALRIL

TFLESTYIPPSYISEMEKAAKQGDTILVSGMKTGSSKLKARIQEAVYKNVRPAEVRLLIL

ENILLNPAYDVYLMVGTSIHYKVQKIRQGKITELSMPSDQYELQLQNSIPGPEGDPARPV

AVLAQDTSMVTALQLGQSSLVLGHRSIRMQGASRLPNSTIYVVEPGYLGFTVHPGDRWVL

ETGRLYEITIEVFDKFSNKVYVSDNIRIETVLPAEFFEVLSSSQNGSYHRIRALKRGQTA

IDAALTSVVDQDGGVHILQVPVWNQQEVEIHIPITLYPSILTFPWQPKTGAYQYTIRAHG

GSGNFSWSSSSHLVATVTVKGVMTTGSDIGFSVIQAHDVQNPLHFGEMKVYVIEPHSMEF

APCQVEARVGQALELPLRISGLMPGGASEVVTLSDCSHFDLAVEVENQGVFQPLPGRLPP

GSEHCSGIRVKAEAQGSTTLLVSYRHGHVHLSAKITIAAYLPLKAVDPSSVALVTLGSSK

EMLFEGGPRPWILEPSKFFQNVTAEDTDSIGLALFAPHSSRNYQQHWILVTCQALGEQVI

ALSVGNKPSLTNPFPAVEPAVVKFVCAPPSRLTLAPVYTSPQLDMSCPLLQQNKQVVPVS

SHRNPRLDLAAYDQEGRRFDNFSSLSIQWESTRPVLASIEPELPMQLVSQDDESGQKKLH

GLQAILVHEASGTTAITATATGYQESHLSSARTKQPHDPLVPLSASIELILVEDVRVSPE

EVTIYNHPGIQAELRIREGSGYFFLNTSTADVVKVAYQEARGVAMVHPLLPGSSTIMIHD

LCLVFPAPAKAVVYVSDIQELYIRVVDKVEIGKTVKAYVRVLDLHKKPFLAKYFPFMDLK

LRAASPIITLVALDEALDNYTITFLIRGVAIGQTSLTASVTNKAGQRINSAPQQIEVFPP

FRLMPRKVTLLIGATMQVTSEGGPQPQSNILFSISNESVALVSAAGLVQGLAIGNGTVSG

LVQAVDAETGKVVIISQDLVQVEVLLLRAVRIRAPIMRMRTGTQMPIYVTGITNHQNPFS

FGNAVPGLTFHWSVTKRDVLDLRGRHHEASIRLPSQYNFAMNVLGRVKGRTGLRVVVKAV

DPTSGQLYGLARELSDEIQVQVFEKLQLLNPEIEAEQILMSPNSYIKLQTNRDGAASLSY

RVLDGPEKVPVVHVDEKGFLASGSMIGTSTIEVIAQEPFGANQTIIVAVKVSPVSYLRVS

MSPVLHTQNKEALVAVPLGMTVTFTVHFHDNSGDVFHAHSSVLNFATNRDDFVQIGKGPT

NNTCVVRTVSVGLTLLRVWDAEHPGLSDFMPLPVLQAISPELSGAMVVGDVLCLATVLTS

LEGLSGTWSSSANSILHIDPKTGVAVARAVGSVTVYYEVAGHLRTYKEVVVSVPQRIMAR

HLHPIQTSFQEATASKVIVAVGDRSSNLRGECTPTQREVIQALHPETLISCQSQFKPAVF

DFPSQDVFTVEPQFDTALGQYFCSITMHRLTDKQRKHLSMKKTALVVSASLSSSHFSTEQ

VGAEVPFSPGLFADQAEILLSNHYTSSEIRVFGAPEVLENLEVKSGSPAVLAFAKEKSFG

WPSFITYTVGVLDPAAGSQGPLSTTLTFSSPVTNQAIAIPVTVAFVVDRRGPGPYGASLF

QHFLDSYQVMFFTLFALLAGTAVMIIAYHTVCTPRDLAVPAALTPRASPGHSPHYFAASS

PTSPNALPPARKASPPSGLWSPAYASH

>sp|Q8TEM1|PO210_HUMAN 491 SHLVATVTV

MAARGRGLLLLTLSVLLAAGPSAAAAKLNIPKVLLPFTRATRVNFTLEASEGCYRWLSTR

PEVASIEPLGLDEQQCSQKAVVQARLTQPARLTSIIFAEDITTGQVLRCDAIVDLIHDIQ

IVSTTRELYLEDSPLELKIQALDSEGNTFSTLAGLVFEWTIVKDSEADRFSDSHNALRIL

TFLESTYIPPSYISEMEKAAKQGDTILVSGMKTGSSKLKARIQEAVYKNVRPAEVRLLIL

ENILLNPAYDVYLMVGTSIHYKVQKIRQGKITELSMPSDQYELQLQNSIPGPEGDPARPV

AVLAQDTSMVTALQLGQSSLVLGHRSIRMQGASRLPNSTIYVVEPGYLGFTVHPGDRWVL

ETGRLYEITIEVFDKFSNKVYVSDNIRIETVLPAEFFEVLSSSQNGSYHRIRALKRGQTA

IDAALTSVVDQDGGVHILQVPVWNQQEVEIHIPITLYPSILTFPWQPKTGAYQYTIRAHG

GSGNFSWSSSSHLVATVTVKGVMTTGSDIGFSVIQAHDVQNPLHFGEMKVYVIEPHSMEF

APCQVEARVGQALELPLRISGLMPGGASEVVTLSDCSHFDLAVEVENQGVFQPLPGRLPP

GSEHCSGIRVKAEAQGSTTLLVSYRHGHVHLSAKITIAAYLPLKAVDPSSVALVTLGSSK

EMLFEGGPRPWILEPSKFFQNVTAEDTDSIGLALFAPHSSRNYQQHWILVTCQALGEQVI

ALSVGNKPSLTNPFPAVEPAVVKFVCAPPSRLTLAPVYTSPQLDMSCPLLQQNKQVVPVS

SHRNPRLDLAAYDQEGRRFDNFSSLSIQWESTRPVLASIEPELPMQLVSQDDESGQKKLH

GLQAILVHEASGTTAITATATGYQESHLSSARTKQPHDPLVPLSASIELILVEDVRVSPE

EVTIYNHPGIQAELRIREGSGYFFLNTSTADVVKVAYQEARGVAMVHPLLPGSSTIMIHD

LCLVFPAPAKAVVYVSDIQELYIRVVDKVEIGKTVKAYVRVLDLHKKPFLAKYFPFMDLK

LRAASPIITLVALDEALDNYTITFLIRGVAIGQTSLTASVTNKAGQRINSAPQQIEVFPP

FRLMPRKVTLLIGATMQVTSEGGPQPQSNILFSISNESVALVSAAGLVQGLAIGNGTVSG

LVQAVDAETGKVVIISQDLVQVEVLLLRAVRIRAPIMRMRTGTQMPIYVTGITNHQNPFS

FGNAVPGLTFHWSVTKRDVLDLRGRHHEASIRLPSQYNFAMNVLGRVKGRTGLRVVVKAV

DPTSGQLYGLARELSDEIQVQVFEKLQLLNPEIEAEQILMSPNSYIKLQTNRDGAASLSY

RVLDGPEKVPVVHVDEKGFLASGSMIGTSTIEVIAQEPFGANQTIIVAVKVSPVSYLRVS

MSPVLHTQNKEALVAVPLGMTVTFTVHFHDNSGDVFHAHSSVLNFATNRDDFVQIGKGPT

NNTCVVRTVSVGLTLLRVWDAEHPGLSDFMPLPVLQAISPELSGAMVVGDVLCLATVLTS

LEGLSGTWSSSANSILHIDPKTGVAVARAVGSVTVYYEVAGHLRTYKEVVVSVPQRIMAR

HLHPIQTSFQEATASKVIVAVGDRSSNLRGECTPTQREVIQALHPETLISCQSQFKPAVF

DFPSQDVFTVEPQFDTALGQYFCSITMHRLTDKQRKHLSMKKTALVVSASLSSSHFSTEQ

VGAEVPFSPGLFADQAEILLSNHYTSSEIRVFGAPEVLENLEVKSGSPAVLAFAKEKSFG

WPSFITYTVGVLDPAAGSQGPLSTTLTFSSPVTNQAIAIPVTVAFVVDRRGPGPYGASLF

QHFLDSYQVMFFTLFALLAGTAVMIIAYHTVCTPRDLAVPAALTPRASPGHSPHYFAASS

PTSPNALPPARKASPPSGLWSPAYASH

>sp|O95248|MTMR5_HUMAN 1581 RPYSNVSNL

MARLADYFVLVAFGPHPRGSGEGQGQILQRFPEKDWEDNPFPQGIELFCQPSGWQLCPER

NPPTFFVAVLTDINSERHYCACLTFWEPAEPSQETTRVEDATEREEEGDEGGQTHLSPTA

PAPSAQLFAPKTLVLVSRLDHTEVFRNSLGLIYAIHVEGLNVCLENVIGNLLTCTVPLAG

GSQRTISLGAGDRQVIQTPLADSLPVSRCSVALLFRQLGITNVLSLFCAALTEHKVLFLS

RSYQRLADACRGLLALLFPLRYSFTYVPILPAQLLEVLSTPTPFIIGVNAAFQAETQELL

DVIVADLDGGTVTIPECVHIPPLPEPLQSQTHSVLSMVLDPELELADLAFPPPTTSTSSL

KMQDKELRAVFLRLFAQLLQGYRWCLHVVRIHPEPVIRFHKAAFLGQRGLVEDDFLMKVL

EGMAFAGFVSERGVPYRPTDLFDELVAHEVARMRADENHPQRVLRHVQELAEQLYKNENP

YPAVAMHKVQRPGESSHLRRVPRPFPRLDEGTVQWIVDQAAAKMQGAPPAVKAERRTTVP

SGPPMTAILERCSGLHVNSARRLEVVRNCISYVFEGKMLEAKKLLPAVLRALKGRAARRC

LAQELHLHVQQNRAVLDHQQFDFVVRMMNCCLQDCTSLDEHGIAAALLPLVTAFCRKLSP

GVTQFAYSCVQEHVVWSTPQFWEAMFYGDVQTHIRALYLEPTEDLAPAQEVGEAPSQEDE

RSALDVASEQRRLWPTLSREKQQELVQKEESTVFSQAIHYANRMSYLLLPLDSSKSRLLR

ERAGLGDLESASNSLVTNSMAGSVAESYDTESGFEDAETCDVAGAVVRFINRFVDKVCTE

SGVTSDHLKGLHVMVPDIVQMHIETLEAVQRESRRLPPIQKPKLLRPRLLPGEECVLDGL

RVYLLPDGREEGAGGSAGGPALLPAEGAVFLTTYRVIFTGMPTDPLVGEQVVVRSFPVAA

LTKEKRISVQTPVDQLLQDGLQLRSCTFQLLKMAFDEEVGSDSAELFRKQLHKLRYPPDI

RATFAFTLGSAHTPGRPPRVTKDKGPSLRTLSRNLVKNAKKTIGRQHVTRKKYNPPSWEH

RGQPPPEDQEDEISVSEELEPSTLTPSSALKPSDRMTMSSLVERACCRDYQRLGLGTLSS

SLSRAKSEPFRISPVNRMYAICRSYPGLLIVPQSVQDNALQRVSRCYRQNRFPVVCWRSG

RSKAVLLRSGGLHGKGVVGLFKAQNAPSPGQSQADSSSLEQEKYLQAVVSSMPRYADASG

RNTLSGFSSAHMGSHGKWGSVRTSGRSSGLGTDVGSRLAGRDALAPPQANGGPPDPGFLR

PQRAALYILGDKAQLKGVRSDPLQQWELVPIEVFEARQVKASFKKLLKACVPGCPAAEPS

PASFLRSLEDSEWLIQIHKLLQVSVLVVELLDSGSSVLVGLEDGWDITTQVVSLVQLLSD

PFYRTLEGFRLLVEKEWLSFGHRFSHRGAHTLAGQSSGFTPVFLQFLDCVHQVHLQFPME

FEFSQFYLKFLGYHHVSRRFRTFLLDSDYERIELGLLYEEKGERRGQVPCRSVWEYVDRL

SKRTPVFHNYMYAPEDAEVLRPYSNVSNLKVWDFYTEETLAEGPPYDWELAQGPPEPPEE

ERSDGGAPQSRRRVVWPCYDSCPRAQPDAISRLLEELQRLETELGQPAERWKDTWDRVKA

AQRLEGRPDGRGTPSSLLVSTAPHHRRSLGVYLQEGPVGSTLSLSLDSDQSSGSTTSGSR

QAARRSTSTLYSQFQTAESENRSYEGTLYKKGAFMKPWKARWFVLDKTKHQLRYYDHRVD

TECKGVIDLAEVEAVAPGTPTMGAPKTVDEKAFFDVKTTRRVYNFCAQDVPSAQQWVDRI

QSCLSDA

>sp|Q9Y6D6|BIG1_HUMAN 1533 TTIPHALLTW

MYEGKKTKNMFLTRALEKILADKEVKKAHHSQLRKACEVALEEIKAETEKQSPPHGEAKA

GSSTLPPVKSKTNFIEADKYFLPFELACQSKCPRIVSTSLDCLQKLIAYGHLTGNAPDST

TPGKKLIDRIIETICGCFQGPQTDEGVQLQIIKALLTAVTSQHIEIHEGTVLQAVRTCYN

IYLASKNLINQTTAKATLTQMLNVIFARMENQALQEAKQMEKERHRQHHHLLQSPVSHHE

PESPQLRYLPPQTVDHISQEHEGDLDLHTNDVDKSLQDDTEPENGSDISSAENEQTEADQ

ATAAETLSKNEVLYDGENHDCEEKPQDIVQNIVEEMVNIVVGDMGEGTTINASADGNIGT

IEDGSDSENIQANGIPGTPISVAYTPSLPDDRLSVSSNDTQESGNSSGPSPGAKFSHILQ

KDAFLVFRSLCKLSMKPLSDGPPDPKSHELRSKILSLQLLLSILQNAGPIFRTNEMFINA

IKQYLCVALSKNGVSSVPEVFELSLSIFLTLLSNFKTHLKMQIEVFFKEIFLYILETSTS

SFDHKWMVIQTLTRICADAQSVVDIYVNYDCDLNAANIFERLVNDLSKIAQGRGSQELGM

SNVQELSLRKKGLECLVSILKCMVEWSKDQYVNPNSQTTLGQEKPSEQEMSEIKHPETIN

RYGSLNSLESTSSSGIGSYSTQMSGTDNPEQFEVLKQQKEIIEQGIDLFNKKPKRGIQYL

QEQGMLGTTPEDIAQFLHQEERLDSTQVGEFLGDNDKFNKEVMYAYVDQHDFSGKDFVSA

LRMFLEGFRLPGEAQKIDRLMEKFAARYLECNQGQTLFASADTAYVLAYSIIMLTTDLHS

PQVKNKMTKEQYIKMNRGINDSKDLPEEYLSAIYNEIAGKKISMKETKELTIPTKSSKQN

VASEKQRRLLYNLEMEQMAKTAKALMEAVSHVQAPFTSATHLEHVRPMFKLAWTPFLAAF

SVGLQDCDDTEVASLCLEGIRCAIRIACIFSIQLERDAYVQALARFTLLTVSSGITEMKQ

KNIDTIKTLITVAHTDGNYLGNSWHEILKCISQLELAQLIGTGVKPRYISGTVRGREGSL

TGTKDQAPDEFVGLGLVGGNVDWKQIASIQESIGETSSQSVVVAVDRIFTGSTRLDGNAI

VDFVRWLCAVSMDELLSTTHPRMFSLQKIVEISYYNMGRIRLQWSRIWEVIGDHFNKVGC

NPNEDVAIFAVDSLRQLSMKFLEKGELANFRFQKDFLRPFEHIMKRNRSPTIRDMVVRCI

AQMVNSQAANIRSGWKNIFSVFHLAASDQDESIVELAFQTTGHIVTLVFEKHFPATIDSF

QDAVKCLSEFACNAAFPDTSMEAIRLIRHCAKYVSDRPQAFKEYTSDDMNVAPEDRVWVR

GWFPILFELSCIINRCKLDVRTRGLTVMFEIMKTYGHTYEKHWWQDLFRIVFRIFDNMKL

PEQQTEKAEWMTTTCNHALYAICDVFTQYLEVLSDVLLDDIFAQLYWCVQQDNEQLARSG

TNCLENVVILNGEKFTLEIWDKTCNCTLDIFKTTIPHALLTWRPNSGETAPPPPSPVSEK

PLDTISQKSVDIHDSIQPRSVDNRPQAPLVSASAVNEEVSKIKSTAKFPEQKLFAALLIK

CVVQLELIQTIDNIVFFPATSKKEDAENLAAAQRDAVDFDVRVDTQDQGMYRFLTSQQLF

KLLDCLLESHRFAKAFNSNNEQRTALWKAGFKGKSKPNLLKQETSSLACGLRILFRMYMD

ESRVSAWEEVQQRLLNVCSEALSYFLTLTSESHREAWTNLLLLFLTKVLKISDNRFKAHA

SFYYPLLCEIMQFDLIPELRAVLRRFFLRIGVVFQISQPPEQELGINKQ

>sp|O15031|PLXB2_HUMAN 1395 TVVERMLSNW

MALQLWALTLLGLLGAGASLRPRKLDFFRSEKELNHLAVDEASGVVYLGAVNALYQLDAK

LQLEQQVATGPALDNKKCTPPIEASQCHEAEMTDNVNQLLLLDPPRKRLVECGSLFKGIC

ALRALSNISLRLFYEDGSGEKSFVASNDEGVATVGLVSSTGPGGDRVLFVGKGNGPHDNG

IIVSTRLLDRTDSREAFEAYTDHATYKAGYLSTNTQQFVAAFEDGPYVFFVFNQQDKHPA

RNRTLLARMCREDPNYYSYLEMDLQCRDPDIHAAAFGTCLAASVAAPGSGRVLYAVFSRD

SRSSGGPGAGLCLFPLDKVHAKMEANRNACYTGTREARDIFYKPFHGDIQCGGHAPGSSK

SFPCGSEHLPYPLGSRDGLRGTAVLQRGGLNLTAVTVAAENNHTVAFLGTSDGRILKVYL

TPDGTSSEYDSILVEINKRVKRDLVLSGDLGSLYAMTQDKVFRLPVQECLSYPTCTQCRD

SQDPYCGWCVVEGRCTRKAECPRAEEASHWLWSRSKSCVAVTSAQPQNMSRRAQGEVQLT

VSPLPALSEEDELLCLFGESPPHPARVEGEAVICNSPSSIPVTPPGQDHVAVTIQLLLRR

GNIFLTSYQYPFYDCRQAMSLEENLPCISCVSNRWTCQWDLRYHECREASPNPEDGIVRA

HMEDSCPQFLGPSPLVIPMNHETDVNFQGKNLDTVKGSSLHVGSDLLKFMEPVTMQESGT

FAFRTPKLSHDANETLPLHLYVKSYGKNIDSKLHVTLYNCSFGRSDCSLCRAANPDYRCA

WCGGQSRCVYEALCNTTSECPPPVITRIQPETGPLGGGIRITILGSNLGVQAGDIQRISV

AGRNCSFQPERYSVSTRIVCVIEAAETPFTGGVEVDVFGKLGRSPPNVQFTFQQPKPLSV

EPQQGPQAGGTTLTIHGTHLDTGSQEDVRVTLNGVPCKVTKFGAQLQCVTGPQATRGQML

LEVSYGGSPVPNPGIFFTYRENPVLRAFEPLRSFASGGRSINVTGQGFSLIQRFAMVVIA

EPLQSWQPPREAESLQPMTVVGTDYVFHNDTKVVFLSPAVPEEPEAYNLTVLIEMDGHRA

LLRTEAGAFEYVPDPTFENFTGGVKKQVNKLIHARGTNLNKAMTLQEAEAFVGAERCTMK

TLTETDLYCEPPEVQPPPKRRQKRDTTHNLPEFIVKFGSREWVLGRVEYDTRVSDVPLSL

ILPLVIVPMVVVIAVSVYCYWRKSQQAEREYEKIKSQLEGLEESVRDRCKKEFTDLMIEM

EDQTNDVHEAGIPVLDYKTYTDRVFFLPSKDGDKDVMITGKLDIPEPRRPVVEQALYQFS

NLLNSKSFLINFIHTLENQREFSARAKVYFASLLTVALHGKLEYYTDIMHTLFLELLEQY

VVAKNPKLMLRRSETVVERMLSNWMSICLYQYLKDSAGEPLYKLFKAIKHQVEKGPVDAV

QKKAKYTLNDTGLLGDDVEYAPLTVSVIVQDEGVDAIPVKVLNCDTISQVKEKIIDQVYR

GQPCSCWPRPDSVVLEWRPGSTAQILSDLDLTSQREGRWKRVNTLMHYNVRDGATLILSK

VGVSQQPEDSQQDLPGERHALLEEENRVWHLVRPTDEVDEGKSKRGSVKEKERTKAITEI

YLTRLLSVKGTLQQFVDNFFQSVLAPGHAVPPAVKYFFDFLDEQAEKHNIQDEDTIHIWK

TNSLPLRFWVNILKNPHFIFDVHVHEVVDASLSVIAQTFMDACTRTEHKLSRDSPSNKLL

YAKEISTYKKMVEDYYKGIRQMVQVSDQDMNTHLAEISRAHTDSLNTLVALHQLYQYTQK

YYDEIINALEEDPAAQKMQLAFRLQQIAAALENKVTDL

>sp|O15031|PLXB2_HUMAN 1279 TYTDRVFFL

MALQLWALTLLGLLGAGASLRPRKLDFFRSEKELNHLAVDEASGVVYLGAVNALYQLDAK

LQLEQQVATGPALDNKKCTPPIEASQCHEAEMTDNVNQLLLLDPPRKRLVECGSLFKGIC

ALRALSNISLRLFYEDGSGEKSFVASNDEGVATVGLVSSTGPGGDRVLFVGKGNGPHDNG

IIVSTRLLDRTDSREAFEAYTDHATYKAGYLSTNTQQFVAAFEDGPYVFFVFNQQDKHPA

RNRTLLARMCREDPNYYSYLEMDLQCRDPDIHAAAFGTCLAASVAAPGSGRVLYAVFSRD

SRSSGGPGAGLCLFPLDKVHAKMEANRNACYTGTREARDIFYKPFHGDIQCGGHAPGSSK

SFPCGSEHLPYPLGSRDGLRGTAVLQRGGLNLTAVTVAAENNHTVAFLGTSDGRILKVYL

TPDGTSSEYDSILVEINKRVKRDLVLSGDLGSLYAMTQDKVFRLPVQECLSYPTCTQCRD

SQDPYCGWCVVEGRCTRKAECPRAEEASHWLWSRSKSCVAVTSAQPQNMSRRAQGEVQLT

VSPLPALSEEDELLCLFGESPPHPARVEGEAVICNSPSSIPVTPPGQDHVAVTIQLLLRR

GNIFLTSYQYPFYDCRQAMSLEENLPCISCVSNRWTCQWDLRYHECREASPNPEDGIVRA

HMEDSCPQFLGPSPLVIPMNHETDVNFQGKNLDTVKGSSLHVGSDLLKFMEPVTMQESGT

FAFRTPKLSHDANETLPLHLYVKSYGKNIDSKLHVTLYNCSFGRSDCSLCRAANPDYRCA

WCGGQSRCVYEALCNTTSECPPPVITRIQPETGPLGGGIRITILGSNLGVQAGDIQRISV

AGRNCSFQPERYSVSTRIVCVIEAAETPFTGGVEVDVFGKLGRSPPNVQFTFQQPKPLSV

EPQQGPQAGGTTLTIHGTHLDTGSQEDVRVTLNGVPCKVTKFGAQLQCVTGPQATRGQML

LEVSYGGSPVPNPGIFFTYRENPVLRAFEPLRSFASGGRSINVTGQGFSLIQRFAMVVIA

EPLQSWQPPREAESLQPMTVVGTDYVFHNDTKVVFLSPAVPEEPEAYNLTVLIEMDGHRA

LLRTEAGAFEYVPDPTFENFTGGVKKQVNKLIHARGTNLNKAMTLQEAEAFVGAERCTMK

TLTETDLYCEPPEVQPPPKRRQKRDTTHNLPEFIVKFGSREWVLGRVEYDTRVSDVPLSL

ILPLVIVPMVVVIAVSVYCYWRKSQQAEREYEKIKSQLEGLEESVRDRCKKEFTDLMIEM

EDQTNDVHEAGIPVLDYKTYTDRVFFLPSKDGDKDVMITGKLDIPEPRRPVVEQALYQFS

NLLNSKSFLINFIHTLENQREFSARAKVYFASLLTVALHGKLEYYTDIMHTLFLELLEQY

VVAKNPKLMLRRSETVVERMLSNWMSICLYQYLKDSAGEPLYKLFKAIKHQVEKGPVDAV

QKKAKYTLNDTGLLGDDVEYAPLTVSVIVQDEGVDAIPVKVLNCDTISQVKEKIIDQVYR

GQPCSCWPRPDSVVLEWRPGSTAQILSDLDLTSQREGRWKRVNTLMHYNVRDGATLILSK

VGVSQQPEDSQQDLPGERHALLEEENRVWHLVRPTDEVDEGKSKRGSVKEKERTKAITEI

YLTRLLSVKGTLQQFVDNFFQSVLAPGHAVPPAVKYFFDFLDEQAEKHNIQDEDTIHIWK

TNSLPLRFWVNILKNPHFIFDVHVHEVVDASLSVIAQTFMDACTRTEHKLSRDSPSNKLL

YAKEISTYKKMVEDYYKGIRQMVQVSDQDMNTHLAEISRAHTDSLNTLVALHQLYQYTQK

YYDEIINALEEDPAAQKMQLAFRLQQIAAALENKVTDL

>sp|P49815|TSC2_HUMAN 526 SLLDIIEKV

MAKPTSKDSGLKEKFKILLGLGTPRPNPRSAEGKQTEFIITAEILRELSMECGLNNRIRM

IGQICEVAKTKKFEEHAVEALWKAVADLLQPERTLEARHAVLALLKAIVQGQGERLGVLR

ALFFKVIKDYPSNEDLHERLEVFKALTDNGRHITYLEEELADFVLQWMDVGLSSEFLLVL

VNLVKFNSCYLDEYIARMVQMICLLCVRTASSVDIEVSLQVLDAVVCYNCLPAESLPLFI

VTLCRTINVKELCEPCWKLMRNLLGTHLGHSAIYNMCHLMEDRAYMEDAPLLRGAVFFVG

MALWGAHRLYSLRNSPTSVFPSFYQAMACPNEVVSYEIVLSITRLIKKYRKELQVVAWDI

LLNIIERLLQQLQTLDSPELRTIVHDLLTTVEELCDQNEFHGSQERYFELVERCADQRPE

SSLLNLISYRAQSIHPAKDGWIQNLQALMERFFRSESRGAVRIKVLDVLSFVLLINRQFY

EEELINSVVISQLSHIPEDKDHQVRKLATQLLVDLAEGCHTHHFNSLLDIIEKVMARSLS

PPPELEERDVAAYSASLEDVKTAVLGLLVILQTKLYTLPASHATRVYEMLVSHIQLHYKH

SYTLPIASSIRLQAFDFLFLLRADSLHRLGLPNKDGVVRFSPYCVCDYMEPERGSEKKTS

GPLSPPTGPPGPAPAGPAVRLGSVPYSLLFRVLLQCLKQESDWKVLKLVLGRLPESLRYK

VLIFTSPCSVDQLCSALCSMLSGPKTLERLRGAPEGFSRTDLHLAVVPVLTALISYHNYL

DKTKQREMVYCLEQGLIHRCARQCVVALSICSVEMPDIIIKALPVLVVKLTHISATASMA

VPLLEFLSTLARLPHLYRNFAAEQYASVFAISLPYTNPSKFNQYIVCLAHHVIAMWFIRC

RLPFRKDFVPFITKGLRSNVLLSFDDTPEKDSFRARSTSLNERPKSLRIARPPKQGLNNS

PPVKEFKESSAAEAFRCRSISVSEHVVRSRIQTSLTSASLGSADENSVAQADDSLKNLHL

ELTETCLDMMARYVFSNFTAVPKRSPVGEFLLAGGRTKTWLVGNKLVTVTTSVGTGTRSL

LGLDSGELQSGPESSSSPGVHVRQTKEAPAKLESQAGQQVSRGARDRVRSMSGGHGLRVG

ALDVPASQFLGSATSPGPRTAPAAKPEKASAGTRVPVQEKTNLAAYVPLLTQGWAEILVR

RPTGNTSWLMSLENPLSPFSSDINNMPLQELSNALMAAERFKEHRDTALYKSLSVPAAST

AKPPPLPRSNTVASFSSLYQSSCQGQLHRSVSWADSAVVMEEGSPGEVPVLVEPPGLEDV

EAALGMDRRTDAYSRSSSVSSQEEKSLHAEELVGRGIPIERVVSSEGGRPSVDLSFQPSQ

PLSKSSSSPELQTLQDILGDPGDKADVGRLSPEVKARSQSGTLDGESAAWSASGEDSRGQ

PEGPLPSSSPRSPSGLRPRGYTISDSAPSRRGKRVERDALKSRATASNAEKVPGINPSFV

FLQLYHSPFFGDESNKPILLPNESQSFERSVQLLDQIPSYDTHKIAVLYVGEGQSNSELA

ILSNEHGSYRYTEFLTGLGRLIELKDCQPDKVYLGGLDVCGEDGQFTYCWHDDIMQAVFH

IATLMPTKDVDKHRCDKKRHLGNDFVSIVYNDSGEDFKLGTIKGQFNFVHVIVTPLDYEC

NLVSLQCRKDMEGLVDTSVAKIVSDRNLPFVARQMALHANMASQVHHSRSNPTDIYPSKW

IARLRHIKRLRQRICEEAAYSNPSLPLVHPPSHSKAPAQTPAEPTPGYEVGQRKRLISSV

EDFTEFV

>sp|P12107|COBA1_HUMAN 1106 VQGPVGL

MEPWSSRWKTKRWLWDFTVTTLALTFLFQAREVRGAAPVDVLKALDFHNSPEGISKTTGF

CTNRKNSKGSDTAYRVSKQAQLSAPTKQLFPGGTFPEDFSILFTVKPKKGIQSFLLSIYN

EHGIQQIGVEVGRSPVFLFEDHTGKPAPEDYPLFRTVNIADGKWHRVAISVEKKTVTMIV

DCKKKTTKPLDRSERAIVDTNGITVFGTRILDEEVFEGDIQQFLITGDPKAAYDYCEHYS

PDCDSSAPKAAQAQEPQIDEYAPEDIIEYDYEYGEAEYKEAESVTEGPTVTEETIAQTEA

NIVDDFQEYNYGTMESYQTEAPRHVSGTNEPNPVEEIFTEEYLTGEDYDSQRKNSEDTLY

ENKEIDGRDSDLLVDGDLGEYDFYEYKEYEDKPTSPPNEEFGPGVPAETDITETSINGHG

AYGEKGQKGEPAVVEPGMLVEGPPGPAGPAGIMGPPGLQGPTGPPGDPGDRGPPGRPGLP

GADGLPGPPGTMLMLPFRYGGDGSKGPTISAQEAQAQAILQQARIALRGPPGPMGLTGRP

GPVGGPGSSGAKGESGDPGPQGPRGVQGPPGPTGKPGKRGRPGADGGRGMPGEPGAKGDR

GFDGLPGLPGDKGHRGERGPQGPPGPPGDDGMRGEDGEIGPRGLPGEAGPRGLLGPRGTP

GAPGQPGMAGVDGPPGPKGNMGPQGEPGPPGQQGNPGPQGLPGPQGPIGPPGEKGPQGKP

GLAGLPGADGPPGHPGKEGQSGEKGALGPPGPQGPIGYPGPRGVKGADGVRGLKGSKGEK

GEDGFPGFKGDMGLKGDRGEVGQIGPRGEDGPEGPKGRAGPTGDPGPSGQAGEKGKLGVP

GLPGYPGRQGPKGSTGFPGFPGANGEKGARGVAGKPGPRGQRGPTGPRGSRGARGPTGKP

GPKGTSGGDGPPGPPGERGPQGPQGPVGFPGPKGPPGPPGKDGLPGHPGQRGETGFQGKT

GPPGPGGVVGPQGPTGETGPIGERGYPGPPGPPGEQGLPGAAGKEGAKGDPGPQGISGKD

GPAGLRGFPGERGLPGAQGAPGLKGGEGPQGPPGPVGSPGERGSAGTAGPIGLRGRPGPQ

GPPGPAGEKGAPGEKGPQGPAGRDGVQGPVGLPGPAGPAGSPGEDGDKGEIGEPGQKGSK

GGKGENGPPGPPGLQGPVGAPGIAGGDGEPGPRGQQGMFGQKGDEGARGFPGPPGPIGLQ

GLPGPPGEKGENGDVGPMGPPGPPGPRGPQGPNGADGPQGPPGSVGSVGGVGEKGEPGEA

GNPGPPGEAGVGGPKGERGEKGEAGPPGAAGPPGAKGPPGDDGPKGNPGPVGFPGDPGPP

GELGPAGQDGVGGDKGEDGDPGQPGPPGPSGEAGPPGPPGKRGPPGAAGAEGRQGEKGAK

GEAGAEGPPGKTGPVGPQGPAGKPGPEGLRGIPGPVGEQGLPGAAGQDGPPGPMGPPGLP

GLKGDPGSKGEKGHPGLIGLIGPPGEQGEKGDRGLPGTQGSPGAKGDGGIPGPAGPLGPP

GPPGLPGPQGPKGNKGSTGPAGQKGDSGLPGPPGPPGPPGEVIQPLPILSSKKTRRHTEG

MQADADDNILDYSDGMEEIFGSLNSLKQDIEHMKFPMGTQTNPARTCKDLQLSHPDFPDG

EYWIDPNQGCSGDSFKVYCNFTSGGETCIYPDKKSEGVRISSWPKEKPGSWFSEFKRGKL

LSYLDVEGNSINMVQMTFLKLLTASARQNFTYHCHQSAAWYDVSSGSYDKALRFLGSNDE

EMSYDNNPFIKTLYDGCTSRKGYEKTVIEINTPKIDQVPIVDVMISDFGDQNQKFGFEVG

PVCFLG

>sp|Q8NI35|INADL_HUMAN 106 GLFPWTPKL

MPENPATDKLQVLQVLDRLKMKLQEKGDTSQNEKLSMFYETLKSPLFNQILTLQQSIKQL

KGQLNHIPSDCSANFDFSRKGLLVFTDGSITNGNVHRPSNNSTVSGLFPWTPKLGNEDFN

SVIQQMAQGRQIEYIDIERPSTGGLGFSVVALRSQNLGKVDIFVKDVQPGSVADRDQRLK

ENDQILAINHTPLDQNISHQQAIALLQQTTGSLRLIVAREPVHTKSSTSSSLNDTTLPET

VCWGHVEEVELINDGSGLGFGIVGGKTSGVVVRTIVPGGLADRDGRLQTGDHILKIGGTN

VQGMTSEQVAQVLRNCGNSVRMLVARDPAGDISVTPPAPAALPVALPTVASKGPGSDSSL

FETYNVELVRKDGQSLGIRIVGYVGTSHTGEASGIYVKSIIPGSAAYHNGHIQVNDKIVA

VDGVNIQGFANHDVVEVLRNAGQVVHLTLVRRKTSSSTSPLEPPSDRGTVVEPLKPPALF

LTGAVETETNVDGEDEEIKERIDTLKNDNIQALEKLEKVPDSPENELKSRWENLLGPDYE

VMVATLDTQIADDAELQKYSKLLPIHTLRLGVEVDSFDGHHYISSIVSGGPVDTLGLLQP

EDELLEVNGMQLYGKSRREAVSFLKEVPPPFTLVCCRRLFDDEASVDEPRRTETSLPETE

VDHNMDVNTEEDDDGELALWSPEVKIVELVKDCKGLGFSILDYQDPLDPTRSVIVIRSLV

ADGVAERSGGLLPGDRLVSVNEYCLDNTSLAEAVEILKAVPPGLVHLGICKPLVEDNEEE

SCYILHSSSNEDKTEFSGTIHDINSSLILEAPKGFRDEPYFKEELVDEPFLDLGKSFHSQ
[truncated: 1,123,390 more chars]
