# Supplementary material for: Systematic Characterisation of Cellular Localisation and Expression Profiles of Proteins Containing MHC Ligands
Source: PLoS One. 2009 Oct 14;4(10):e7448. doi: 10.1371/journal.pone.0007448 (PMC2758592; doi:10.1371/journal.pone.0007448)
Supplement: Data set S2 — The MHCII data set. The amino acid sequences of the proteins in the MHCII data set in fasta format. The first column of the heading lists the UniProtKB Accession number and ID of the protein, the second column lists the starting position of the MHC class II ligand in the protein, the third column lists the amino acid sequence of the MHC class II ligand. (0.56 MB DOC) [file pone.0007448.s002.doc]

>sp|P21817|RYR1_HUMAN 2615 RPSMLQHLLR

MGDAEGEDEVQFLRTDDEVVLQCSATVLKEQLKLCLAAEGFGNRLCFLEPTSNAQNVPPD

LAICCFVLEQSLSVRALQEMLANTVEAGVESSQGGGHRTLLYGHAILLRHAHSRMYLSCL

TTSRSMTDKLAFDVGLQEDATGEACWWTMHPASKQRSEGEKVRVGDDIILVSVSSERYLH

LSTASGELQVDASFMQTLWNMNPICSRCEEGFVTGGHVLRLFHGHMDECLTISPADSDDQ

RRLVYYEGGAVCTHARSLWRLEPLRISWSGSHLRWGQPLRVRHVTTGQYLALTEDQGLVV

VDASKAHTKATSFCFRISKEKLDVAPKRDVEGMGPPEIKYGESLCFVQHVASGLWLTYAA

PDPKALRLGVLKKKAMLHQEGHMDDALSLTRCQQEESQAARMIHSTNGLYNQFIKSLDSF

SGKPRGSGPPAGTALPIEGVILSLQDLIIYFEPPSEDLQHEEKQSKLRSLRNRQSLFQEE

GMLSMVLNCIDRLNVYTTAAHFAEFAGEEAAESWKEIVNLLYELLASLIRGNRSNCALFS

TNLDWLVSKLDRLEASSGILEVLYCVLIESPEVLNIIQENHIKSIISLLDKHGRNHKVLD

VLCSLCVCNGVAVRSNQDLITENLLPGRELLLQTNLINYVTSIRPNIFVGRAEGTTQYSK

WYFEVMVDEVTPFLTAQATHLRVGWALTEGYTPYPGAGEGWGGNGVGDDLYSYGFDGLHL

WTGHVARPVTSPGQHLLAPEDVISCCLDLSVPSISFRINGCPVQGVFESFNLDGLFFPVV

SFSAGVKVRFLLGGRHGEFKFLPPPGYAPCHEAVLPRERLHLEPIKEYRREGPRGPHLVG

PSRCLSHTDFVPCPVDTVQIVLPPHLERIREKLAENIHELWALTRIEQGWTYGPVRDDNK

RLHPCLVDFHSLPEPERNYNLQMSGETLKTLLALGCHVGMADEKAEDNLKKTKLPKTYMM

SNGYKPAPLDLSHVRLTPAQTTLVDRLAENGHNVWARDRVGQGWSYSAVQDIPARRNPRL

VPYRLLDEATKRSNRDSLCQAVRTLLGYGYNIEPPDQEPSQVENQSRCDRVRIFRAEKSY

TVQSGRWYFEFEAVTTGEMRVGWARPELRPDVELGADELAYVFNGHRGQRWHLGSEPFGR

PWQPGDVVGCMIDLTENTIIFTLNGEVLMSDSGSETAFREIEIGDGFLPVCSLGPGQVGH

LNLGQDVSSLRFFAICGLQEGFEPFAINMQRPVTTWFSKGLPQFEPVPLEHPHYEVSRVD

GTVDTPPCLRLTHRTWGSQNSLVEMLFLRLSLPVQFHQHFRCTAGATPLAPPGLQPPAED

EARAAEPDPDYENLRRSAGGWSEAENGKEGTAKEGAPGGTPQAGGEAQPARAENEKDATT

EKNKKRGFLFKAKKVAMMTQPPATPTLPRLPHDVVPADNRDDPEIILNTTTYYYSVRVFA

GQEPSCVWAGWVTPDYHQHDMSFDLSKVRVVTVTMGDEQGNVHSSLKCSNCYMVWGGDFV

SPGQQGRISHTDLVIGCLVDLATGLMTFTANGKESNTFFQVEPNTKLFPAVFVLPTHQNV

IQFELGKQKNIMPLSAAMFQSERKNPAPQCPPRLEMQMLMPVSWSRMPNHFLQVETRRAG

ERLGWAVQCQEPLTMMALHIPEENRCMDILELSERLDLQRFHSHTLRLYRAVCALGNNRV

AHALCSHVDQAQLLHALEDAHLPGPLRAGYYDLLISIHLESACRSRRSMLSEYIVPLTPE

TRAITLFPPGRSTENGHPRHGLPGVGVTTSLRPPHHFSPPCFVAALPAAGAAEAPARLSP

AIPLEALRDKALRMLGEAVRDGGQHARDPVGGSVEFQFVPVLKLVSTLLVMGIFGDEDVK

QILKMIEPEVFTEEEEEEDEEEEGEEEDEEEKEEDEEETAQEKEDEEKEEEEAAEGEKEE

GLEEGLLQMKLPESVKLQMCHLLEYFCDQELQHRVESLAAFAERYVDKLQANQRSRYGLL

IKAFSMTAAETARRTREFRSPPQEQINMLLQFKDGTDEEDCPLPEEIRQDLLDFHQDLLA

HCGIQLDGEEEEPEEETTLGSRLMSLLEKVRLVKKKEEKPEEERSAEESKPRSLQELVSH

MVVRWAQEDFVQSPELVRAMFSLLHRQYDGLGELLRALPRAYTISPSSVEDTMSLLECLG

QIRSLLIVQMGPQEENLMIQSIGNIMNNKVFYQHPNLMRALGMHETVMEVMVNVLGGGES

KEIRFPKMVTSCCRFLCYFCRISRQNQRSMFDHLSYLLENSGIGLGMQGSTPLDVAAASV

IDNNELALALQEQDLEKVVSYLAGCGLQSCPMLVAKGYPDIGWNPCGGERYLDFLRFAVF

VNGESVEENANVVVRLLIRKPECFGPALRGEGGSGLLAAIEEAIRISEDPARDGPGIRRD

RRREHFGEEPPEENRVHLGHAIMSFYAALIDLLGRCAPEMHLIQAGKGEALRIRAILRSL

VPLEDLVGIISLPLQIPTLGKDGALVQPKMSASFVPDHKASMVLFLDRVYGIENQDFLLH

VLDVGFLPDMRAAASLDTATFSTTEMALALNRYLCLAVLPLITKCAPLFAGTEHRAIMVD

SMLHTVYRLSRGRSLTKAQRDVIEDCLMSLCRYIRPSMLQHLLRRLVFDVPILNEFAKMP

LKLLTNHYERCWKYYCLPTGWANFGVTSEEELHLTRKLFWGIFDSLAHKKYDPELYRMAM

PCLCAIAGALPPDYVDASYSSKAEKKATVDAEGNFDPRPVETLNVIIPEKLDSFINKFAE

YTHEKWAFDKIQNNWSYGENIDEELKTHPMLRPYKTFSEKDKEIYRWPIKESLKAMIAWE

WTIEKAREGEEEKTEKKKTRKISQSAQTYDPREGYNPQPPDLSAVTLSRELQAMAEQLAE

NYHNTWGRKKKQELEAKGGGTHPLLVPYDTLTAKEKARDREKAQELLKFLQMNGYAVTRG

LKDMELDSSSIEKRFAFGFLQQLLRWMDISQEFIAHLEAVVSSGRVEKSPHEQEIKFFAK

ILLPLINQYFTNHCLYFLSTPAKVLGSGGHASNKEKEMITSLFCKLAALVRHRVSLFGTD

APAVVNCLHILARSLDARTVMKSGPEIVKAGLRSFFESASEDIEKMVENLRLGKVSQART

QVKGVGQNLTYTTVALLPVLTTLFQHIAQHQFGDDVILDDVQVSCYRTLCSIYSLGTTKN

TYVEKLRPALGECLARLAAAMPVAFLEPQLNEYNACSVYTTKSPRERAILGLPNSVEEMC

PDIPVLERLMADIGGLAESGARYTEMPHVIEITLPMLCSYLPRWWERGPEAPPSALPAGA

PPPCTAVTSDHLNSLLGNILRIIVNNLGIDEASWMKRLAVFAQPIVSRARPELLQSHFIP

TIGRLRKRAGKVVSEEEQLRLEAKAEAQEGELLVRDEFSVLCRDLYALYPLLIRYVDNNR

AQWLTEPNPSAEELFRMVGEIFIYWSKSHNFKREEQNFVVQNEINNMSFLTADNKSKMAK

AGDIQSGGSDQERTKKKRRGDRYSVQTSLIVATLKKMLPIGLNMCAPTDQDLITLAKTRY

ALKDTDEEVREFLHNNLHLQGKVEGSPSLRWQMALYRGVPGREEDADDPEKIVRRVQEVS

AVLYYLDQTEHPYKSKKAVWHKLLSKQRRRAVVACFRMTPLYNLPTHRACNMFLESYKAA

WILTEDHSFEDRMIDDLSKAGEQEEEEEEVEEKKPDPLHQLVLHFSRTALTEKSKLDEDY

LYMAYADIMAKSCHLEEGGENGEAEEEVEVSFEEKQMEKQRLLYQQARLHTRGAAEMVLQ

MISACKGETGAMVSSTLKLGISILNGGNAEVQQKMLDYLKDKKEVGFFQSIQALMQTCSV

LDLNAFERQNKAEGLGMVNEDGTVINRQNGEKVMADDEFTQDLFRFLQLLCEGHNNDFQN

YLRTQTGNTTTINIIICTVDYLLRLQESISDFYWYYSGKDVIEEQGKRNFSKAMSVAKQV

FNSLTEYIQGPCTGNQQSLAHSRLWDAVVGFLHVFAHMMMKLAQDSSQIELLKELLDLQK

DMVVMLLSLLEGNVVNGMIARQMVDMLVESSSNVEMILKFFDMFLKLKDIVGSEAFQDYV

TDPRGLISKKDFQKAMDSQKQFSGPEIQFLLSCSEADENEMINCEEFANRFQEPARDIGF

NVAVLLTNLSEHVPHDPRLHNFLELAESILEYFRPYLGRIEIMGASRRIERIYFEISETN

RAQWEMPQVKESKRQFIFDVVNEGGEAEKMELFVSFCEDTIFEMQIAAQISEPEGEPETD

EDEGAGAAEAGAEGAEEGAAGLEGTAATAAAGATARVVAAAGRALRGLSYRSLRRRVRRL

RRLTAREAATAVAALLWAAVTRAGAAGAGAAAGALGLLWGSLFGGGLVEGAKKVTVTELL

AGMPDPTSDEVHGEQPAGPGGDADGEGASEGAGDAAEGAGDEEEAVHEAGPGGADGAVAV

TDGGPFRPEGAGGLGDMGDTTPAEPPTPEGSPILKRKLGVDGVEEELPPEPEPEPEPELE

PEKADAENGEKEEVPEPTPEPPKKQAPPSPPPKKEEAGGEFWGELEVQRVKFLNYLSRNF

YTLRFLALFLAFAINFILLFYKVSDSPPGEDDMEGSAAGDVSGAGSGGSSGWGLGAGEEA

EGDEDENMVYYFLEESTGYMEPALRCLSLLHTLVAFLCIIGYNCLKVPLVIFKREKELAR

KLEFDGLYITEQPEDDDVKGQWDRLVLNTPSFPSNYWDKFVKRKVLDKHGDIYGRERIAE

LLGMDLATLEITAHNERKPNPPPGLLTWLMSIDVKYQIWKFGVIFTDNSFLYLGWYMVMS

LLGHYNNFFFAAHLLDIAMGVKTLRTILSSVTHNGKQLVMTVGLLAVVVYLYTVVAFNFF

RKFYNKSEDEDEPDMKCDDMMTCYLFHMYVGVRAGGGIGDEIEDPAGDEYELYRVVFDIT

FFFFVIVILLAIIQGLIIDAFGELRDQQEQVKEDMETKCFICGIGSDYFDTTPHGFETHT

LEEHNLANYMFFLMYLINKDETEHTGQESYVWKMYQERCWDFFPAGDCFRKQYEDQLS

>sp|P04114|APOB_HUMAN 2617 FIVPLTDLRIPS

MDPPRPALLALLALPALLLLLLAGARAEEEMLENVSLVCPKDATRFKHLRKYTYNYEAES

SSGVPGTADSRSATRINCKVELEVPQLCSFILKTSQCTLKEVYGFNPEGKALLKKTKNSE

EFAAAMSRYELKLAIPEGKQVFLYPEKDEPTYILNIKRGIISALLVPPETEEAKQVLFLD

TVYGNCSTHFTVKTRKGNVATEISTERDLGQCDRFKPIRTGISPLALIKGMTRPLSTLIS

SSQSCQYTLDAKRKHVAEAICKEQHLFLPFSYNNKYGMVAQVTQTLKLEDTPKINSRFFG

EGTKKMGLAFESTKSTSPPKQAEAVLKTLQELKKLTISEQNIQRANLFNKLVTELRGLSD

EAVTSLLPQLIEVSSPITLQALVQCGQPQCSTHILQWLKRVHANPLLIDVVTYLVALIPE

PSAQQLREIFNMARDQRSRATLYALSHAVNNYHKTNPTGTQELLDIANYLMEQIQDDCTG

DEDYTYLILRVIGNMGQTMEQLTPELKSSILKCVQSTKPSLMIQKAAIQALRKMEPKDKD

QEVLLQTFLDDASPGDKRLAAYLMLMRSPSQADINKIVQILPWEQNEQVKNFVASHIANI

LNSEELDIQDLKKLVKEALKESQLPTVMDFRKFSRNYQLYKSVSLPSLDPASAKIEGNLI

FDPNNYLPKESMLKTTLTAFGFASADLIEIGLEGKGFEPTLEALFGKQGFFPDSVNKALY

WVNGQVPDGVSKVLVDHFGYTKDDKHEQDMVNGIMLSVEKLIKDLKSKEVPEARAYLRIL

GEELGFASLHDLQLLGKLLLMGARTLQGIPQMIGEVIRKGSKNDFFLHYIFMENAFELPT

GAGLQLQISSSGVIAPGAKAGVKLEVANMQAELVAKPSVSVEFVTNMGIIIPDFARSGVQ

MNTNFFHESGLEAHVALKAGKLKFIIPSPKRPVKLLSGGNTLHLVSTTKTEVIPPLIENR

QSWSVCKQVFPGLNYCTSGAYSNASSTDSASYYPLTGDTRLELELRPTGEIEQYSVSATY

ELQREDRALVDTLKFVTQAEGAKQTEATMTFKYNRQSMTLSSEVQIPDFDVDLGTILRVN

DESTEGKTSYRLTLDIQNKKITEVALMGHLSCDTKEERKIKGVISIPRLQAEARSEILAH

WSPAKLLLQMDSSATAYGSTVSKRVAWHYDEEKIEFEWNTGTNVDTKKMTSNFPVDLSDY

PKSLHMYANRLLDHRVPETDMTFRHVGSKLIVAMSSWLQKASGSLPYTQTLQDHLNSLKE

FNLQNMGLPDFHIPENLFLKSDGRVKYTLNKNSLKIEIPLPFGGKSSRDLKMLETVRTPA

LHFKSVGFHLPSREFQVPTFTIPKLYQLQVPLLGVLDLSTNVYSNLYNWSASYSGGNTST

DHFSLRARYHMKADSVVDLLSYNVQGSGETTYDHKNTFTLSCDGSLRHKFLDSNIKFSHV

EKLGNNPVSKGLLIFDASSSWGPQMSASVHLDSKKKQHLFVKEVKIDGQFRVSSFYAKGT

YGLSCQRDPNTGRLNGESNLRFNSSYLQGTNQITGRYEDGTLSLTSTSDLQSGIIKNTAS

LKYENYELTLKSDTNGKYKNFATSNKMDMTFSKQNALLRSEYQADYESLRFFSLLSGSLN

SHGLELNADILGTDKINSGAHKATLRIGQDGISTSATTNLKCSLLVLENELNAELGLSGA

SMKLTTNGRFREHNAKFSLDGKAALTELSLGSAYQAMILGVDSKNIFNFKVSQEGLKLSN

DMMGSYAEMKFDHTNSLNIAGLSLDFSSKLDNIYSSDKFYKQTVNLQLQPYSLVTTLNSD

LKYNALDLTNNGKLRLEPLKLHVAGNLKGAYQNNEIKHIYAISSAALSASYKADTVAKVQ

GVEFSHRLNTDIAGLASAIDMSTNYNSDSLHFSNVFRSVMAPFTMTIDAHTNGNGKLALW

GEHTGQLYSKFLLKAEPLAFTFSHDYKGSTSHHLVSRKSISAALEHKVSALLTPAEQTGT

WKLKTQFNNNEYSQDLDAYNTKDKIGVELTGRTLADLTLLDSPIKVPLLLSEPINIIDAL

EMRDAVEKPQEFTIVAFVKYDKNQDVHSINLPFFETLQEYFERNRQTIIVVVENVQRNLK

HINIDQFVRKYRAALGKLPQQANDYLNSFNWERQVSHAKEKLTALTKKYRITENDIQIAL

DDAKINFNEKLSQLQTYMIQFDQYIKDSYDLHDLKIAIANIIDEIIEKLKSLDEHYHIRV

NLVKTIHDLHLFIENIDFNKSGSSTASWIQNVDTKYQIRIQIQEKLQQLKRHIQNIDIQH

LAGKLKQHIEAIDVRVLLDQLGTTISFERINDVLEHVKHFVINLIGDFEVAEKINAFRAK

VHELIERYEVDQQIQVLMDKLVELTHQYKLKETIQKLSNVLQQVKIKDYFEKLVGFIDDA

VKKLNELSFKTFIEDVNKFLDMLIKKLKSFDYHQFVDETNDKIREVTQRLNGEIQALELP

QKAEALKLFLEETKATVAVYLESLQDTKITLIINWLQEALSSASLAHMKAKFRETLEDTR

DRMYQMDIQQELQRYLSLVGQVYSTLVTYISDWWTLAAKNLTDFAEQYSIQDWAKRMKAL

VEQGFTVPEIKTILGTMPAFEVSLQALQKATFQTPDFIVPLTDLRIPSVQINFKDLKNIK

IPSRFSTPEFTILNTFHIPSFTIDFVEMKVKIIRTIDQMQNSELQWPVPDIYLRDLKVED

IPLARITLPDFRLPEIAIPEFIIPTLNLNDFQVPDLHIPEFQLPHISHTIEVPTFGKLYS

ILKIQSPLFTLDANADIGNGTTSANEAGIAASITAKGESKLEVLNFDFQANAQLSNPKIN

PLALKESVKFSSKYLRTEHGSEMLFFGNAIEGKSNTVASLHTEKNTLELSNGVIVKINNQ

LTLDSNTKYFHKLNIPKLDFSSQADLRNEIKTLLKAGHIAWTSSGKGSWKWACPRFSDEG

THESQISFTIEGPLTSFGLSNKINSKHLRVNQNLVYESGSLNFSKLEIQSQVDSQHVGHS

VLTAKGMALFGEGKAEFTGRHDAHLNGKVIGTLKNSLFFSAQPFEITASTNNEGNLKVRF

PLRLTGKIDFLNNYALFLSPSAQQASWQVSARFNQYKYNQNFSAGNNENIMEAHVGINGE

ANLDFLNIPLTIPEMRLPYTIITTPPLKDFSLWEKTGLKEFLKTTKQSFDLSVKAQYKKN

KHRHSITNPLAVLCEFISQSIKSFDRHFEKNRNNALDFVTKSYNETKIKFDKYKAEKSHD

ELPRTFQIPGYTVPVVNVEVSPFTIEMSAFGYVFPKAVSMPSFSILGSDVRVPSYTLILP

SLELPVLHVPRNLKLSLPHFKELCTISHIFIPAMGNITYDFSFKSSVITLNTNAELFNQS

DIVAHLLSSSSSVIDALQYKLEGTTRLTRKRGLKLATALSLSNKFVEGSHNSTVSLTTKN

MEVSVAKTTKAEIPILRMNFKQELNGNTKSKPTVSSSMEFKYDFNSSMLYSTAKGAVDHK

LSLESLTSYFSIESSTKGDVKGSVLSREYSGTIASEANTYLNSKSTRSSVKLQGTSKIDD

IWNLEVKENFAGEATLQRIYSLWEHSTKNHLQLEGLFFTNGEHTSKATLELSPWQMSALV

QVHASQPSSFHDFPDLGQEVALNANTKNQKIRWKNEVRIHSGSFQSQVELSNDQEKAHLD

IAGSLEGHLRFLKNIILPVYDKSLWDFLKLDVTTSIGRRQHLRVSTAFVYTKNPNGYSFS

IPVKVLADKFITPGLKLNDLNSVLVMPTFHVPFTDLQVPSCKLDFREIQIYKKLRTSSFA

LNLPTLPEVKFPEVDVLTKYSQPEDSLIPFFEITVPESQLTVSQFTLPKSVSDGIAALDL

NAVANKIADFELPTIIVPEQTIEIPSIKFSVPAGIVIPSFQALTARFEVDSPVYNATWSA

SLKNKADYVETVLDSTCSSTVQFLEYELNVLGTHKIEDGTLASKTKGTLAHRDFSAEYEE

DGKFEGLQEWEGKAHLNIKSPAFTDLHLRYQKDKKGISTSAASPAVGTVGMDMDEDDDFS

KWNFYYSPQSSPDKKLTIFKTELRVRESDEETQIKVNWEEEAASGLLTSLKDNVPKATGV

LYDYVNKYHWEHTGLTLREVSSKLRRNLQNNAEWVYQGAIRQIDDIDVRFQKAASGTTGT

YQEWKDKAQNLYQELLTQEGQASFQGLKDNVFDGLVRVTQKFHMKVKHLIDSLIDFLNFP

RFQFPGKPGIYTREELCTMFIREVGTVLSQVYSKVHNGSEILFSYFQDLVITLPFELRKH

KLIDVISMYRELLKDLSKEAQEVFKAIQSLKTTEVLRNLQDLLQFIFQLIEDNIKQLKEM

KFTYLINYIQDEINTIFNDYIPYVFKLLKENLCLNLHKFNEFIQNELQEASQELQQIHQY

IMALREEYFDPSIVGWTVKYYELEEKIVSLIKNLLVALKDFHSEYIVSASNFTSQLSSQV

EQFLHRNIQEYLSILTDPDGKGKEKIAELSATAQEIIKSQAIATKKIISDYHQQFRYKLQ

DFSDQLSDYYEKFIAESKRLIDLSIQNYHTFLIYITELLKKLQSTTVMNPYMKLAPGELT

IIL

>sp|P04114|APOB_HUMAN 2612 FQTPDFIVPLTDLRIPSVQI

MDPPRPALLALLALPALLLLLLAGARAEEEMLENVSLVCPKDATRFKHLRKYTYNYEAES

SSGVPGTADSRSATRINCKVELEVPQLCSFILKTSQCTLKEVYGFNPEGKALLKKTKNSE

EFAAAMSRYELKLAIPEGKQVFLYPEKDEPTYILNIKRGIISALLVPPETEEAKQVLFLD

TVYGNCSTHFTVKTRKGNVATEISTERDLGQCDRFKPIRTGISPLALIKGMTRPLSTLIS

SSQSCQYTLDAKRKHVAEAICKEQHLFLPFSYNNKYGMVAQVTQTLKLEDTPKINSRFFG

EGTKKMGLAFESTKSTSPPKQAEAVLKTLQELKKLTISEQNIQRANLFNKLVTELRGLSD

EAVTSLLPQLIEVSSPITLQALVQCGQPQCSTHILQWLKRVHANPLLIDVVTYLVALIPE

PSAQQLREIFNMARDQRSRATLYALSHAVNNYHKTNPTGTQELLDIANYLMEQIQDDCTG

DEDYTYLILRVIGNMGQTMEQLTPELKSSILKCVQSTKPSLMIQKAAIQALRKMEPKDKD

QEVLLQTFLDDASPGDKRLAAYLMLMRSPSQADINKIVQILPWEQNEQVKNFVASHIANI

LNSEELDIQDLKKLVKEALKESQLPTVMDFRKFSRNYQLYKSVSLPSLDPASAKIEGNLI

FDPNNYLPKESMLKTTLTAFGFASADLIEIGLEGKGFEPTLEALFGKQGFFPDSVNKALY

WVNGQVPDGVSKVLVDHFGYTKDDKHEQDMVNGIMLSVEKLIKDLKSKEVPEARAYLRIL

GEELGFASLHDLQLLGKLLLMGARTLQGIPQMIGEVIRKGSKNDFFLHYIFMENAFELPT

GAGLQLQISSSGVIAPGAKAGVKLEVANMQAELVAKPSVSVEFVTNMGIIIPDFARSGVQ

MNTNFFHESGLEAHVALKAGKLKFIIPSPKRPVKLLSGGNTLHLVSTTKTEVIPPLIENR

QSWSVCKQVFPGLNYCTSGAYSNASSTDSASYYPLTGDTRLELELRPTGEIEQYSVSATY

ELQREDRALVDTLKFVTQAEGAKQTEATMTFKYNRQSMTLSSEVQIPDFDVDLGTILRVN

DESTEGKTSYRLTLDIQNKKITEVALMGHLSCDTKEERKIKGVISIPRLQAEARSEILAH

WSPAKLLLQMDSSATAYGSTVSKRVAWHYDEEKIEFEWNTGTNVDTKKMTSNFPVDLSDY

PKSLHMYANRLLDHRVPETDMTFRHVGSKLIVAMSSWLQKASGSLPYTQTLQDHLNSLKE

FNLQNMGLPDFHIPENLFLKSDGRVKYTLNKNSLKIEIPLPFGGKSSRDLKMLETVRTPA

LHFKSVGFHLPSREFQVPTFTIPKLYQLQVPLLGVLDLSTNVYSNLYNWSASYSGGNTST

DHFSLRARYHMKADSVVDLLSYNVQGSGETTYDHKNTFTLSCDGSLRHKFLDSNIKFSHV

EKLGNNPVSKGLLIFDASSSWGPQMSASVHLDSKKKQHLFVKEVKIDGQFRVSSFYAKGT

YGLSCQRDPNTGRLNGESNLRFNSSYLQGTNQITGRYEDGTLSLTSTSDLQSGIIKNTAS

LKYENYELTLKSDTNGKYKNFATSNKMDMTFSKQNALLRSEYQADYESLRFFSLLSGSLN

SHGLELNADILGTDKINSGAHKATLRIGQDGISTSATTNLKCSLLVLENELNAELGLSGA

SMKLTTNGRFREHNAKFSLDGKAALTELSLGSAYQAMILGVDSKNIFNFKVSQEGLKLSN

DMMGSYAEMKFDHTNSLNIAGLSLDFSSKLDNIYSSDKFYKQTVNLQLQPYSLVTTLNSD

LKYNALDLTNNGKLRLEPLKLHVAGNLKGAYQNNEIKHIYAISSAALSASYKADTVAKVQ

GVEFSHRLNTDIAGLASAIDMSTNYNSDSLHFSNVFRSVMAPFTMTIDAHTNGNGKLALW

GEHTGQLYSKFLLKAEPLAFTFSHDYKGSTSHHLVSRKSISAALEHKVSALLTPAEQTGT

WKLKTQFNNNEYSQDLDAYNTKDKIGVELTGRTLADLTLLDSPIKVPLLLSEPINIIDAL

EMRDAVEKPQEFTIVAFVKYDKNQDVHSINLPFFETLQEYFERNRQTIIVVVENVQRNLK

HINIDQFVRKYRAALGKLPQQANDYLNSFNWERQVSHAKEKLTALTKKYRITENDIQIAL

DDAKINFNEKLSQLQTYMIQFDQYIKDSYDLHDLKIAIANIIDEIIEKLKSLDEHYHIRV

NLVKTIHDLHLFIENIDFNKSGSSTASWIQNVDTKYQIRIQIQEKLQQLKRHIQNIDIQH

LAGKLKQHIEAIDVRVLLDQLGTTISFERINDVLEHVKHFVINLIGDFEVAEKINAFRAK

VHELIERYEVDQQIQVLMDKLVELTHQYKLKETIQKLSNVLQQVKIKDYFEKLVGFIDDA

VKKLNELSFKTFIEDVNKFLDMLIKKLKSFDYHQFVDETNDKIREVTQRLNGEIQALELP

QKAEALKLFLEETKATVAVYLESLQDTKITLIINWLQEALSSASLAHMKAKFRETLEDTR

DRMYQMDIQQELQRYLSLVGQVYSTLVTYISDWWTLAAKNLTDFAEQYSIQDWAKRMKAL

VEQGFTVPEIKTILGTMPAFEVSLQALQKATFQTPDFIVPLTDLRIPSVQINFKDLKNIK

IPSRFSTPEFTILNTFHIPSFTIDFVEMKVKIIRTIDQMQNSELQWPVPDIYLRDLKVED

IPLARITLPDFRLPEIAIPEFIIPTLNLNDFQVPDLHIPEFQLPHISHTIEVPTFGKLYS

ILKIQSPLFTLDANADIGNGTTSANEAGIAASITAKGESKLEVLNFDFQANAQLSNPKIN

PLALKESVKFSSKYLRTEHGSEMLFFGNAIEGKSNTVASLHTEKNTLELSNGVIVKINNQ

LTLDSNTKYFHKLNIPKLDFSSQADLRNEIKTLLKAGHIAWTSSGKGSWKWACPRFSDEG

THESQISFTIEGPLTSFGLSNKINSKHLRVNQNLVYESGSLNFSKLEIQSQVDSQHVGHS

VLTAKGMALFGEGKAEFTGRHDAHLNGKVIGTLKNSLFFSAQPFEITASTNNEGNLKVRF

PLRLTGKIDFLNNYALFLSPSAQQASWQVSARFNQYKYNQNFSAGNNENIMEAHVGINGE

ANLDFLNIPLTIPEMRLPYTIITTPPLKDFSLWEKTGLKEFLKTTKQSFDLSVKAQYKKN

KHRHSITNPLAVLCEFISQSIKSFDRHFEKNRNNALDFVTKSYNETKIKFDKYKAEKSHD

ELPRTFQIPGYTVPVVNVEVSPFTIEMSAFGYVFPKAVSMPSFSILGSDVRVPSYTLILP

SLELPVLHVPRNLKLSLPHFKELCTISHIFIPAMGNITYDFSFKSSVITLNTNAELFNQS

DIVAHLLSSSSSVIDALQYKLEGTTRLTRKRGLKLATALSLSNKFVEGSHNSTVSLTTKN

MEVSVAKTTKAEIPILRMNFKQELNGNTKSKPTVSSSMEFKYDFNSSMLYSTAKGAVDHK

LSLESLTSYFSIESSTKGDVKGSVLSREYSGTIASEANTYLNSKSTRSSVKLQGTSKIDD

IWNLEVKENFAGEATLQRIYSLWEHSTKNHLQLEGLFFTNGEHTSKATLELSPWQMSALV

QVHASQPSSFHDFPDLGQEVALNANTKNQKIRWKNEVRIHSGSFQSQVELSNDQEKAHLD

IAGSLEGHLRFLKNIILPVYDKSLWDFLKLDVTTSIGRRQHLRVSTAFVYTKNPNGYSFS

IPVKVLADKFITPGLKLNDLNSVLVMPTFHVPFTDLQVPSCKLDFREIQIYKKLRTSSFA

LNLPTLPEVKFPEVDVLTKYSQPEDSLIPFFEITVPESQLTVSQFTLPKSVSDGIAALDL

NAVANKIADFELPTIIVPEQTIEIPSIKFSVPAGIVIPSFQALTARFEVDSPVYNATWSA

SLKNKADYVETVLDSTCSSTVQFLEYELNVLGTHKIEDGTLASKTKGTLAHRDFSAEYEE

DGKFEGLQEWEGKAHLNIKSPAFTDLHLRYQKDKKGISTSAASPAVGTVGMDMDEDDDFS

KWNFYYSPQSSPDKKLTIFKTELRVRESDEETQIKVNWEEEAASGLLTSLKDNVPKATGV

LYDYVNKYHWEHTGLTLREVSSKLRRNLQNNAEWVYQGAIRQIDDIDVRFQKAASGTTGT

YQEWKDKAQNLYQELLTQEGQASFQGLKDNVFDGLVRVTQKFHMKVKHLIDSLIDFLNFP

RFQFPGKPGIYTREELCTMFIREVGTVLSQVYSKVHNGSEILFSYFQDLVITLPFELRKH

KLIDVISMYRELLKDLSKEAQEVFKAIQSLKTTEVLRNLQDLLQFIFQLIEDNIKQLKEM

KFTYLINYIQDEINTIFNDYIPYVFKLLKENLCLNLHKFNEFIQNELQEASQELQQIHQY

IMALREEYFDPSIVGWTVKYYELEEKIVSLIKNLLVALKDFHSEYIVSASNFTSQLSSQV

EQFLHRNIQEYLSILTDPDGKGKEKIAELSATAQEIIKSQAIATKKIISDYHQQFRYKLQ

DFSDQLSDYYEKFIAESKRLIDLSIQNYHTFLIYITELLKKLQSTTVMNPYMKLAPGELT

IIL

>sp|P04114|APOB_HUMAN 1273 IPENLFLKSDGRVKYT

MDPPRPALLALLALPALLLLLLAGARAEEEMLENVSLVCPKDATRFKHLRKYTYNYEAES

SSGVPGTADSRSATRINCKVELEVPQLCSFILKTSQCTLKEVYGFNPEGKALLKKTKNSE

EFAAAMSRYELKLAIPEGKQVFLYPEKDEPTYILNIKRGIISALLVPPETEEAKQVLFLD

TVYGNCSTHFTVKTRKGNVATEISTERDLGQCDRFKPIRTGISPLALIKGMTRPLSTLIS

SSQSCQYTLDAKRKHVAEAICKEQHLFLPFSYNNKYGMVAQVTQTLKLEDTPKINSRFFG

EGTKKMGLAFESTKSTSPPKQAEAVLKTLQELKKLTISEQNIQRANLFNKLVTELRGLSD

EAVTSLLPQLIEVSSPITLQALVQCGQPQCSTHILQWLKRVHANPLLIDVVTYLVALIPE

PSAQQLREIFNMARDQRSRATLYALSHAVNNYHKTNPTGTQELLDIANYLMEQIQDDCTG

DEDYTYLILRVIGNMGQTMEQLTPELKSSILKCVQSTKPSLMIQKAAIQALRKMEPKDKD

QEVLLQTFLDDASPGDKRLAAYLMLMRSPSQADINKIVQILPWEQNEQVKNFVASHIANI

LNSEELDIQDLKKLVKEALKESQLPTVMDFRKFSRNYQLYKSVSLPSLDPASAKIEGNLI

FDPNNYLPKESMLKTTLTAFGFASADLIEIGLEGKGFEPTLEALFGKQGFFPDSVNKALY

WVNGQVPDGVSKVLVDHFGYTKDDKHEQDMVNGIMLSVEKLIKDLKSKEVPEARAYLRIL

GEELGFASLHDLQLLGKLLLMGARTLQGIPQMIGEVIRKGSKNDFFLHYIFMENAFELPT

GAGLQLQISSSGVIAPGAKAGVKLEVANMQAELVAKPSVSVEFVTNMGIIIPDFARSGVQ

MNTNFFHESGLEAHVALKAGKLKFIIPSPKRPVKLLSGGNTLHLVSTTKTEVIPPLIENR

QSWSVCKQVFPGLNYCTSGAYSNASSTDSASYYPLTGDTRLELELRPTGEIEQYSVSATY

ELQREDRALVDTLKFVTQAEGAKQTEATMTFKYNRQSMTLSSEVQIPDFDVDLGTILRVN

DESTEGKTSYRLTLDIQNKKITEVALMGHLSCDTKEERKIKGVISIPRLQAEARSEILAH

WSPAKLLLQMDSSATAYGSTVSKRVAWHYDEEKIEFEWNTGTNVDTKKMTSNFPVDLSDY

PKSLHMYANRLLDHRVPETDMTFRHVGSKLIVAMSSWLQKASGSLPYTQTLQDHLNSLKE

FNLQNMGLPDFHIPENLFLKSDGRVKYTLNKNSLKIEIPLPFGGKSSRDLKMLETVRTPA

LHFKSVGFHLPSREFQVPTFTIPKLYQLQVPLLGVLDLSTNVYSNLYNWSASYSGGNTST

DHFSLRARYHMKADSVVDLLSYNVQGSGETTYDHKNTFTLSCDGSLRHKFLDSNIKFSHV

EKLGNNPVSKGLLIFDASSSWGPQMSASVHLDSKKKQHLFVKEVKIDGQFRVSSFYAKGT

YGLSCQRDPNTGRLNGESNLRFNSSYLQGTNQITGRYEDGTLSLTSTSDLQSGIIKNTAS

LKYENYELTLKSDTNGKYKNFATSNKMDMTFSKQNALLRSEYQADYESLRFFSLLSGSLN

SHGLELNADILGTDKINSGAHKATLRIGQDGISTSATTNLKCSLLVLENELNAELGLSGA

SMKLTTNGRFREHNAKFSLDGKAALTELSLGSAYQAMILGVDSKNIFNFKVSQEGLKLSN

DMMGSYAEMKFDHTNSLNIAGLSLDFSSKLDNIYSSDKFYKQTVNLQLQPYSLVTTLNSD

LKYNALDLTNNGKLRLEPLKLHVAGNLKGAYQNNEIKHIYAISSAALSASYKADTVAKVQ

GVEFSHRLNTDIAGLASAIDMSTNYNSDSLHFSNVFRSVMAPFTMTIDAHTNGNGKLALW

GEHTGQLYSKFLLKAEPLAFTFSHDYKGSTSHHLVSRKSISAALEHKVSALLTPAEQTGT

WKLKTQFNNNEYSQDLDAYNTKDKIGVELTGRTLADLTLLDSPIKVPLLLSEPINIIDAL

EMRDAVEKPQEFTIVAFVKYDKNQDVHSINLPFFETLQEYFERNRQTIIVVVENVQRNLK

HINIDQFVRKYRAALGKLPQQANDYLNSFNWERQVSHAKEKLTALTKKYRITENDIQIAL

DDAKINFNEKLSQLQTYMIQFDQYIKDSYDLHDLKIAIANIIDEIIEKLKSLDEHYHIRV

NLVKTIHDLHLFIENIDFNKSGSSTASWIQNVDTKYQIRIQIQEKLQQLKRHIQNIDIQH

LAGKLKQHIEAIDVRVLLDQLGTTISFERINDVLEHVKHFVINLIGDFEVAEKINAFRAK

VHELIERYEVDQQIQVLMDKLVELTHQYKLKETIQKLSNVLQQVKIKDYFEKLVGFIDDA

VKKLNELSFKTFIEDVNKFLDMLIKKLKSFDYHQFVDETNDKIREVTQRLNGEIQALELP

QKAEALKLFLEETKATVAVYLESLQDTKITLIINWLQEALSSASLAHMKAKFRETLEDTR

DRMYQMDIQQELQRYLSLVGQVYSTLVTYISDWWTLAAKNLTDFAEQYSIQDWAKRMKAL

VEQGFTVPEIKTILGTMPAFEVSLQALQKATFQTPDFIVPLTDLRIPSVQINFKDLKNIK

IPSRFSTPEFTILNTFHIPSFTIDFVEMKVKIIRTIDQMQNSELQWPVPDIYLRDLKVED

IPLARITLPDFRLPEIAIPEFIIPTLNLNDFQVPDLHIPEFQLPHISHTIEVPTFGKLYS

ILKIQSPLFTLDANADIGNGTTSANEAGIAASITAKGESKLEVLNFDFQANAQLSNPKIN

PLALKESVKFSSKYLRTEHGSEMLFFGNAIEGKSNTVASLHTEKNTLELSNGVIVKINNQ

LTLDSNTKYFHKLNIPKLDFSSQADLRNEIKTLLKAGHIAWTSSGKGSWKWACPRFSDEG

THESQISFTIEGPLTSFGLSNKINSKHLRVNQNLVYESGSLNFSKLEIQSQVDSQHVGHS

VLTAKGMALFGEGKAEFTGRHDAHLNGKVIGTLKNSLFFSAQPFEITASTNNEGNLKVRF

PLRLTGKIDFLNNYALFLSPSAQQASWQVSARFNQYKYNQNFSAGNNENIMEAHVGINGE

ANLDFLNIPLTIPEMRLPYTIITTPPLKDFSLWEKTGLKEFLKTTKQSFDLSVKAQYKKN

KHRHSITNPLAVLCEFISQSIKSFDRHFEKNRNNALDFVTKSYNETKIKFDKYKAEKSHD

ELPRTFQIPGYTVPVVNVEVSPFTIEMSAFGYVFPKAVSMPSFSILGSDVRVPSYTLILP

SLELPVLHVPRNLKLSLPHFKELCTISHIFIPAMGNITYDFSFKSSVITLNTNAELFNQS

DIVAHLLSSSSSVIDALQYKLEGTTRLTRKRGLKLATALSLSNKFVEGSHNSTVSLTTKN

MEVSVAKTTKAEIPILRMNFKQELNGNTKSKPTVSSSMEFKYDFNSSMLYSTAKGAVDHK

LSLESLTSYFSIESSTKGDVKGSVLSREYSGTIASEANTYLNSKSTRSSVKLQGTSKIDD

IWNLEVKENFAGEATLQRIYSLWEHSTKNHLQLEGLFFTNGEHTSKATLELSPWQMSALV

QVHASQPSSFHDFPDLGQEVALNANTKNQKIRWKNEVRIHSGSFQSQVELSNDQEKAHLD

IAGSLEGHLRFLKNIILPVYDKSLWDFLKLDVTTSIGRRQHLRVSTAFVYTKNPNGYSFS

IPVKVLADKFITPGLKLNDLNSVLVMPTFHVPFTDLQVPSCKLDFREIQIYKKLRTSSFA

LNLPTLPEVKFPEVDVLTKYSQPEDSLIPFFEITVPESQLTVSQFTLPKSVSDGIAALDL

NAVANKIADFELPTIIVPEQTIEIPSIKFSVPAGIVIPSFQALTARFEVDSPVYNATWSA

SLKNKADYVETVLDSTCSSTVQFLEYELNVLGTHKIEDGTLASKTKGTLAHRDFSAEYEE

DGKFEGLQEWEGKAHLNIKSPAFTDLHLRYQKDKKGISTSAASPAVGTVGMDMDEDDDFS

KWNFYYSPQSSPDKKLTIFKTELRVRESDEETQIKVNWEEEAASGLLTSLKDNVPKATGV

LYDYVNKYHWEHTGLTLREVSSKLRRNLQNNAEWVYQGAIRQIDDIDVRFQKAASGTTGT

YQEWKDKAQNLYQELLTQEGQASFQGLKDNVFDGLVRVTQKFHMKVKHLIDSLIDFLNFP

RFQFPGKPGIYTREELCTMFIREVGTVLSQVYSKVHNGSEILFSYFQDLVITLPFELRKH

KLIDVISMYRELLKDLSKEAQEVFKAIQSLKTTEVLRNLQDLLQFIFQLIEDNIKQLKEM

KFTYLINYIQDEINTIFNDYIPYVFKLLKENLCLNLHKFNEFIQNELQEASQELQQIHQY

IMALREEYFDPSIVGWTVKYYELEEKIVSLIKNLLVALKDFHSEYIVSASNFTSQLSSQV

EQFLHRNIQEYLSILTDPDGKGKEKIAELSATAQEIIKSQAIATKKIISDYHQQFRYKLQ

DFSDQLSDYYEKFIAESKRLIDLSIQNYHTFLIYITELLKKLQSTTVMNPYMKLAPGELT

IIL

>sp|P04114|APOB_HUMAN 2615 PDFIVPLTDLRIP

MDPPRPALLALLALPALLLLLLAGARAEEEMLENVSLVCPKDATRFKHLRKYTYNYEAES

SSGVPGTADSRSATRINCKVELEVPQLCSFILKTSQCTLKEVYGFNPEGKALLKKTKNSE

EFAAAMSRYELKLAIPEGKQVFLYPEKDEPTYILNIKRGIISALLVPPETEEAKQVLFLD

TVYGNCSTHFTVKTRKGNVATEISTERDLGQCDRFKPIRTGISPLALIKGMTRPLSTLIS

SSQSCQYTLDAKRKHVAEAICKEQHLFLPFSYNNKYGMVAQVTQTLKLEDTPKINSRFFG

EGTKKMGLAFESTKSTSPPKQAEAVLKTLQELKKLTISEQNIQRANLFNKLVTELRGLSD

EAVTSLLPQLIEVSSPITLQALVQCGQPQCSTHILQWLKRVHANPLLIDVVTYLVALIPE

PSAQQLREIFNMARDQRSRATLYALSHAVNNYHKTNPTGTQELLDIANYLMEQIQDDCTG

DEDYTYLILRVIGNMGQTMEQLTPELKSSILKCVQSTKPSLMIQKAAIQALRKMEPKDKD

QEVLLQTFLDDASPGDKRLAAYLMLMRSPSQADINKIVQILPWEQNEQVKNFVASHIANI

LNSEELDIQDLKKLVKEALKESQLPTVMDFRKFSRNYQLYKSVSLPSLDPASAKIEGNLI

FDPNNYLPKESMLKTTLTAFGFASADLIEIGLEGKGFEPTLEALFGKQGFFPDSVNKALY

WVNGQVPDGVSKVLVDHFGYTKDDKHEQDMVNGIMLSVEKLIKDLKSKEVPEARAYLRIL

GEELGFASLHDLQLLGKLLLMGARTLQGIPQMIGEVIRKGSKNDFFLHYIFMENAFELPT

GAGLQLQISSSGVIAPGAKAGVKLEVANMQAELVAKPSVSVEFVTNMGIIIPDFARSGVQ

MNTNFFHESGLEAHVALKAGKLKFIIPSPKRPVKLLSGGNTLHLVSTTKTEVIPPLIENR

QSWSVCKQVFPGLNYCTSGAYSNASSTDSASYYPLTGDTRLELELRPTGEIEQYSVSATY

ELQREDRALVDTLKFVTQAEGAKQTEATMTFKYNRQSMTLSSEVQIPDFDVDLGTILRVN

DESTEGKTSYRLTLDIQNKKITEVALMGHLSCDTKEERKIKGVISIPRLQAEARSEILAH

WSPAKLLLQMDSSATAYGSTVSKRVAWHYDEEKIEFEWNTGTNVDTKKMTSNFPVDLSDY

PKSLHMYANRLLDHRVPETDMTFRHVGSKLIVAMSSWLQKASGSLPYTQTLQDHLNSLKE

FNLQNMGLPDFHIPENLFLKSDGRVKYTLNKNSLKIEIPLPFGGKSSRDLKMLETVRTPA

LHFKSVGFHLPSREFQVPTFTIPKLYQLQVPLLGVLDLSTNVYSNLYNWSASYSGGNTST

DHFSLRARYHMKADSVVDLLSYNVQGSGETTYDHKNTFTLSCDGSLRHKFLDSNIKFSHV

EKLGNNPVSKGLLIFDASSSWGPQMSASVHLDSKKKQHLFVKEVKIDGQFRVSSFYAKGT

YGLSCQRDPNTGRLNGESNLRFNSSYLQGTNQITGRYEDGTLSLTSTSDLQSGIIKNTAS

LKYENYELTLKSDTNGKYKNFATSNKMDMTFSKQNALLRSEYQADYESLRFFSLLSGSLN

SHGLELNADILGTDKINSGAHKATLRIGQDGISTSATTNLKCSLLVLENELNAELGLSGA

SMKLTTNGRFREHNAKFSLDGKAALTELSLGSAYQAMILGVDSKNIFNFKVSQEGLKLSN

DMMGSYAEMKFDHTNSLNIAGLSLDFSSKLDNIYSSDKFYKQTVNLQLQPYSLVTTLNSD

LKYNALDLTNNGKLRLEPLKLHVAGNLKGAYQNNEIKHIYAISSAALSASYKADTVAKVQ

GVEFSHRLNTDIAGLASAIDMSTNYNSDSLHFSNVFRSVMAPFTMTIDAHTNGNGKLALW

GEHTGQLYSKFLLKAEPLAFTFSHDYKGSTSHHLVSRKSISAALEHKVSALLTPAEQTGT

WKLKTQFNNNEYSQDLDAYNTKDKIGVELTGRTLADLTLLDSPIKVPLLLSEPINIIDAL

EMRDAVEKPQEFTIVAFVKYDKNQDVHSINLPFFETLQEYFERNRQTIIVVVENVQRNLK

HINIDQFVRKYRAALGKLPQQANDYLNSFNWERQVSHAKEKLTALTKKYRITENDIQIAL

DDAKINFNEKLSQLQTYMIQFDQYIKDSYDLHDLKIAIANIIDEIIEKLKSLDEHYHIRV

NLVKTIHDLHLFIENIDFNKSGSSTASWIQNVDTKYQIRIQIQEKLQQLKRHIQNIDIQH

LAGKLKQHIEAIDVRVLLDQLGTTISFERINDVLEHVKHFVINLIGDFEVAEKINAFRAK

VHELIERYEVDQQIQVLMDKLVELTHQYKLKETIQKLSNVLQQVKIKDYFEKLVGFIDDA

VKKLNELSFKTFIEDVNKFLDMLIKKLKSFDYHQFVDETNDKIREVTQRLNGEIQALELP

QKAEALKLFLEETKATVAVYLESLQDTKITLIINWLQEALSSASLAHMKAKFRETLEDTR

DRMYQMDIQQELQRYLSLVGQVYSTLVTYISDWWTLAAKNLTDFAEQYSIQDWAKRMKAL

VEQGFTVPEIKTILGTMPAFEVSLQALQKATFQTPDFIVPLTDLRIPSVQINFKDLKNIK

IPSRFSTPEFTILNTFHIPSFTIDFVEMKVKIIRTIDQMQNSELQWPVPDIYLRDLKVED

IPLARITLPDFRLPEIAIPEFIIPTLNLNDFQVPDLHIPEFQLPHISHTIEVPTFGKLYS

ILKIQSPLFTLDANADIGNGTTSANEAGIAASITAKGESKLEVLNFDFQANAQLSNPKIN

PLALKESVKFSSKYLRTEHGSEMLFFGNAIEGKSNTVASLHTEKNTLELSNGVIVKINNQ

LTLDSNTKYFHKLNIPKLDFSSQADLRNEIKTLLKAGHIAWTSSGKGSWKWACPRFSDEG

THESQISFTIEGPLTSFGLSNKINSKHLRVNQNLVYESGSLNFSKLEIQSQVDSQHVGHS

VLTAKGMALFGEGKAEFTGRHDAHLNGKVIGTLKNSLFFSAQPFEITASTNNEGNLKVRF

PLRLTGKIDFLNNYALFLSPSAQQASWQVSARFNQYKYNQNFSAGNNENIMEAHVGINGE

ANLDFLNIPLTIPEMRLPYTIITTPPLKDFSLWEKTGLKEFLKTTKQSFDLSVKAQYKKN

KHRHSITNPLAVLCEFISQSIKSFDRHFEKNRNNALDFVTKSYNETKIKFDKYKAEKSHD

ELPRTFQIPGYTVPVVNVEVSPFTIEMSAFGYVFPKAVSMPSFSILGSDVRVPSYTLILP

SLELPVLHVPRNLKLSLPHFKELCTISHIFIPAMGNITYDFSFKSSVITLNTNAELFNQS

DIVAHLLSSSSSVIDALQYKLEGTTRLTRKRGLKLATALSLSNKFVEGSHNSTVSLTTKN

MEVSVAKTTKAEIPILRMNFKQELNGNTKSKPTVSSSMEFKYDFNSSMLYSTAKGAVDHK

LSLESLTSYFSIESSTKGDVKGSVLSREYSGTIASEANTYLNSKSTRSSVKLQGTSKIDD

IWNLEVKENFAGEATLQRIYSLWEHSTKNHLQLEGLFFTNGEHTSKATLELSPWQMSALV

QVHASQPSSFHDFPDLGQEVALNANTKNQKIRWKNEVRIHSGSFQSQVELSNDQEKAHLD

IAGSLEGHLRFLKNIILPVYDKSLWDFLKLDVTTSIGRRQHLRVSTAFVYTKNPNGYSFS

IPVKVLADKFITPGLKLNDLNSVLVMPTFHVPFTDLQVPSCKLDFREIQIYKKLRTSSFA

LNLPTLPEVKFPEVDVLTKYSQPEDSLIPFFEITVPESQLTVSQFTLPKSVSDGIAALDL

NAVANKIADFELPTIIVPEQTIEIPSIKFSVPAGIVIPSFQALTARFEVDSPVYNATWSA

SLKNKADYVETVLDSTCSSTVQFLEYELNVLGTHKIEDGTLASKTKGTLAHRDFSAEYEE

DGKFEGLQEWEGKAHLNIKSPAFTDLHLRYQKDKKGISTSAASPAVGTVGMDMDEDDDFS

KWNFYYSPQSSPDKKLTIFKTELRVRESDEETQIKVNWEEEAASGLLTSLKDNVPKATGV

LYDYVNKYHWEHTGLTLREVSSKLRRNLQNNAEWVYQGAIRQIDDIDVRFQKAASGTTGT

YQEWKDKAQNLYQELLTQEGQASFQGLKDNVFDGLVRVTQKFHMKVKHLIDSLIDFLNFP

RFQFPGKPGIYTREELCTMFIREVGTVLSQVYSKVHNGSEILFSYFQDLVITLPFELRKH

KLIDVISMYRELLKDLSKEAQEVFKAIQSLKTTEVLRNLQDLLQFIFQLIEDNIKQLKEM

KFTYLINYIQDEINTIFNDYIPYVFKLLKENLCLNLHKFNEFIQNELQEASQELQQIHQY

IMALREEYFDPSIVGWTVKYYELEEKIVSLIKNLLVALKDFHSEYIVSASNFTSQLSSQV

EQFLHRNIQEYLSILTDPDGKGKEKIAELSATAQEIIKSQAIATKKIISDYHQQFRYKLQ

DFSDQLSDYYEKFIAESKRLIDLSIQNYHTFLIYITELLKKLQSTTVMNPYMKLAPGELT

IIL

>sp|P04114|APOB_HUMAN 2615 PDFIVPLTDLRIPSVQ

MDPPRPALLALLALPALLLLLLAGARAEEEMLENVSLVCPKDATRFKHLRKYTYNYEAES

SSGVPGTADSRSATRINCKVELEVPQLCSFILKTSQCTLKEVYGFNPEGKALLKKTKNSE

EFAAAMSRYELKLAIPEGKQVFLYPEKDEPTYILNIKRGIISALLVPPETEEAKQVLFLD

TVYGNCSTHFTVKTRKGNVATEISTERDLGQCDRFKPIRTGISPLALIKGMTRPLSTLIS

SSQSCQYTLDAKRKHVAEAICKEQHLFLPFSYNNKYGMVAQVTQTLKLEDTPKINSRFFG

EGTKKMGLAFESTKSTSPPKQAEAVLKTLQELKKLTISEQNIQRANLFNKLVTELRGLSD

EAVTSLLPQLIEVSSPITLQALVQCGQPQCSTHILQWLKRVHANPLLIDVVTYLVALIPE

PSAQQLREIFNMARDQRSRATLYALSHAVNNYHKTNPTGTQELLDIANYLMEQIQDDCTG

DEDYTYLILRVIGNMGQTMEQLTPELKSSILKCVQSTKPSLMIQKAAIQALRKMEPKDKD

QEVLLQTFLDDASPGDKRLAAYLMLMRSPSQADINKIVQILPWEQNEQVKNFVASHIANI

LNSEELDIQDLKKLVKEALKESQLPTVMDFRKFSRNYQLYKSVSLPSLDPASAKIEGNLI

FDPNNYLPKESMLKTTLTAFGFASADLIEIGLEGKGFEPTLEALFGKQGFFPDSVNKALY

WVNGQVPDGVSKVLVDHFGYTKDDKHEQDMVNGIMLSVEKLIKDLKSKEVPEARAYLRIL

GEELGFASLHDLQLLGKLLLMGARTLQGIPQMIGEVIRKGSKNDFFLHYIFMENAFELPT

GAGLQLQISSSGVIAPGAKAGVKLEVANMQAELVAKPSVSVEFVTNMGIIIPDFARSGVQ

MNTNFFHESGLEAHVALKAGKLKFIIPSPKRPVKLLSGGNTLHLVSTTKTEVIPPLIENR

QSWSVCKQVFPGLNYCTSGAYSNASSTDSASYYPLTGDTRLELELRPTGEIEQYSVSATY

ELQREDRALVDTLKFVTQAEGAKQTEATMTFKYNRQSMTLSSEVQIPDFDVDLGTILRVN

DESTEGKTSYRLTLDIQNKKITEVALMGHLSCDTKEERKIKGVISIPRLQAEARSEILAH

WSPAKLLLQMDSSATAYGSTVSKRVAWHYDEEKIEFEWNTGTNVDTKKMTSNFPVDLSDY

PKSLHMYANRLLDHRVPETDMTFRHVGSKLIVAMSSWLQKASGSLPYTQTLQDHLNSLKE

FNLQNMGLPDFHIPENLFLKSDGRVKYTLNKNSLKIEIPLPFGGKSSRDLKMLETVRTPA

LHFKSVGFHLPSREFQVPTFTIPKLYQLQVPLLGVLDLSTNVYSNLYNWSASYSGGNTST

DHFSLRARYHMKADSVVDLLSYNVQGSGETTYDHKNTFTLSCDGSLRHKFLDSNIKFSHV

EKLGNNPVSKGLLIFDASSSWGPQMSASVHLDSKKKQHLFVKEVKIDGQFRVSSFYAKGT

YGLSCQRDPNTGRLNGESNLRFNSSYLQGTNQITGRYEDGTLSLTSTSDLQSGIIKNTAS

LKYENYELTLKSDTNGKYKNFATSNKMDMTFSKQNALLRSEYQADYESLRFFSLLSGSLN

SHGLELNADILGTDKINSGAHKATLRIGQDGISTSATTNLKCSLLVLENELNAELGLSGA

SMKLTTNGRFREHNAKFSLDGKAALTELSLGSAYQAMILGVDSKNIFNFKVSQEGLKLSN

DMMGSYAEMKFDHTNSLNIAGLSLDFSSKLDNIYSSDKFYKQTVNLQLQPYSLVTTLNSD

LKYNALDLTNNGKLRLEPLKLHVAGNLKGAYQNNEIKHIYAISSAALSASYKADTVAKVQ

GVEFSHRLNTDIAGLASAIDMSTNYNSDSLHFSNVFRSVMAPFTMTIDAHTNGNGKLALW

GEHTGQLYSKFLLKAEPLAFTFSHDYKGSTSHHLVSRKSISAALEHKVSALLTPAEQTGT

WKLKTQFNNNEYSQDLDAYNTKDKIGVELTGRTLADLTLLDSPIKVPLLLSEPINIIDAL

EMRDAVEKPQEFTIVAFVKYDKNQDVHSINLPFFETLQEYFERNRQTIIVVVENVQRNLK

HINIDQFVRKYRAALGKLPQQANDYLNSFNWERQVSHAKEKLTALTKKYRITENDIQIAL

DDAKINFNEKLSQLQTYMIQFDQYIKDSYDLHDLKIAIANIIDEIIEKLKSLDEHYHIRV

NLVKTIHDLHLFIENIDFNKSGSSTASWIQNVDTKYQIRIQIQEKLQQLKRHIQNIDIQH

LAGKLKQHIEAIDVRVLLDQLGTTISFERINDVLEHVKHFVINLIGDFEVAEKINAFRAK

VHELIERYEVDQQIQVLMDKLVELTHQYKLKETIQKLSNVLQQVKIKDYFEKLVGFIDDA

VKKLNELSFKTFIEDVNKFLDMLIKKLKSFDYHQFVDETNDKIREVTQRLNGEIQALELP

QKAEALKLFLEETKATVAVYLESLQDTKITLIINWLQEALSSASLAHMKAKFRETLEDTR

DRMYQMDIQQELQRYLSLVGQVYSTLVTYISDWWTLAAKNLTDFAEQYSIQDWAKRMKAL

VEQGFTVPEIKTILGTMPAFEVSLQALQKATFQTPDFIVPLTDLRIPSVQINFKDLKNIK

IPSRFSTPEFTILNTFHIPSFTIDFVEMKVKIIRTIDQMQNSELQWPVPDIYLRDLKVED

IPLARITLPDFRLPEIAIPEFIIPTLNLNDFQVPDLHIPEFQLPHISHTIEVPTFGKLYS

ILKIQSPLFTLDANADIGNGTTSANEAGIAASITAKGESKLEVLNFDFQANAQLSNPKIN

PLALKESVKFSSKYLRTEHGSEMLFFGNAIEGKSNTVASLHTEKNTLELSNGVIVKINNQ

LTLDSNTKYFHKLNIPKLDFSSQADLRNEIKTLLKAGHIAWTSSGKGSWKWACPRFSDEG

THESQISFTIEGPLTSFGLSNKINSKHLRVNQNLVYESGSLNFSKLEIQSQVDSQHVGHS

VLTAKGMALFGEGKAEFTGRHDAHLNGKVIGTLKNSLFFSAQPFEITASTNNEGNLKVRF

PLRLTGKIDFLNNYALFLSPSAQQASWQVSARFNQYKYNQNFSAGNNENIMEAHVGINGE

ANLDFLNIPLTIPEMRLPYTIITTPPLKDFSLWEKTGLKEFLKTTKQSFDLSVKAQYKKN

KHRHSITNPLAVLCEFISQSIKSFDRHFEKNRNNALDFVTKSYNETKIKFDKYKAEKSHD

ELPRTFQIPGYTVPVVNVEVSPFTIEMSAFGYVFPKAVSMPSFSILGSDVRVPSYTLILP

SLELPVLHVPRNLKLSLPHFKELCTISHIFIPAMGNITYDFSFKSSVITLNTNAELFNQS

DIVAHLLSSSSSVIDALQYKLEGTTRLTRKRGLKLATALSLSNKFVEGSHNSTVSLTTKN

MEVSVAKTTKAEIPILRMNFKQELNGNTKSKPTVSSSMEFKYDFNSSMLYSTAKGAVDHK

LSLESLTSYFSIESSTKGDVKGSVLSREYSGTIASEANTYLNSKSTRSSVKLQGTSKIDD

IWNLEVKENFAGEATLQRIYSLWEHSTKNHLQLEGLFFTNGEHTSKATLELSPWQMSALV

QVHASQPSSFHDFPDLGQEVALNANTKNQKIRWKNEVRIHSGSFQSQVELSNDQEKAHLD

IAGSLEGHLRFLKNIILPVYDKSLWDFLKLDVTTSIGRRQHLRVSTAFVYTKNPNGYSFS

IPVKVLADKFITPGLKLNDLNSVLVMPTFHVPFTDLQVPSCKLDFREIQIYKKLRTSSFA

LNLPTLPEVKFPEVDVLTKYSQPEDSLIPFFEITVPESQLTVSQFTLPKSVSDGIAALDL

NAVANKIADFELPTIIVPEQTIEIPSIKFSVPAGIVIPSFQALTARFEVDSPVYNATWSA

SLKNKADYVETVLDSTCSSTVQFLEYELNVLGTHKIEDGTLASKTKGTLAHRDFSAEYEE

DGKFEGLQEWEGKAHLNIKSPAFTDLHLRYQKDKKGISTSAASPAVGTVGMDMDEDDDFS

KWNFYYSPQSSPDKKLTIFKTELRVRESDEETQIKVNWEEEAASGLLTSLKDNVPKATGV

LYDYVNKYHWEHTGLTLREVSSKLRRNLQNNAEWVYQGAIRQIDDIDVRFQKAASGTTGT

YQEWKDKAQNLYQELLTQEGQASFQGLKDNVFDGLVRVTQKFHMKVKHLIDSLIDFLNFP

RFQFPGKPGIYTREELCTMFIREVGTVLSQVYSKVHNGSEILFSYFQDLVITLPFELRKH

KLIDVISMYRELLKDLSKEAQEVFKAIQSLKTTEVLRNLQDLLQFIFQLIEDNIKQLKEM

KFTYLINYIQDEINTIFNDYIPYVFKLLKENLCLNLHKFNEFIQNELQEASQELQQIHQY

IMALREEYFDPSIVGWTVKYYELEEKIVSLIKNLLVALKDFHSEYIVSASNFTSQLSSQV

EQFLHRNIQEYLSILTDPDGKGKEKIAELSATAQEIIKSQAIATKKIISDYHQQFRYKLQ

DFSDQLSDYYEKFIAESKRLIDLSIQNYHTFLIYITELLKKLQSTTVMNPYMKLAPGELT

IIL

>sp|P04114|APOB_HUMAN 223 SPLALIKGMTRPLSTLIS

MDPPRPALLALLALPALLLLLLAGARAEEEMLENVSLVCPKDATRFKHLRKYTYNYEAES

SSGVPGTADSRSATRINCKVELEVPQLCSFILKTSQCTLKEVYGFNPEGKALLKKTKNSE

EFAAAMSRYELKLAIPEGKQVFLYPEKDEPTYILNIKRGIISALLVPPETEEAKQVLFLD

TVYGNCSTHFTVKTRKGNVATEISTERDLGQCDRFKPIRTGISPLALIKGMTRPLSTLIS

SSQSCQYTLDAKRKHVAEAICKEQHLFLPFSYNNKYGMVAQVTQTLKLEDTPKINSRFFG

EGTKKMGLAFESTKSTSPPKQAEAVLKTLQELKKLTISEQNIQRANLFNKLVTELRGLSD

EAVTSLLPQLIEVSSPITLQALVQCGQPQCSTHILQWLKRVHANPLLIDVVTYLVALIPE

PSAQQLREIFNMARDQRSRATLYALSHAVNNYHKTNPTGTQELLDIANYLMEQIQDDCTG

DEDYTYLILRVIGNMGQTMEQLTPELKSSILKCVQSTKPSLMIQKAAIQALRKMEPKDKD

QEVLLQTFLDDASPGDKRLAAYLMLMRSPSQADINKIVQILPWEQNEQVKNFVASHIANI

LNSEELDIQDLKKLVKEALKESQLPTVMDFRKFSRNYQLYKSVSLPSLDPASAKIEGNLI

FDPNNYLPKESMLKTTLTAFGFASADLIEIGLEGKGFEPTLEALFGKQGFFPDSVNKALY

WVNGQVPDGVSKVLVDHFGYTKDDKHEQDMVNGIMLSVEKLIKDLKSKEVPEARAYLRIL

GEELGFASLHDLQLLGKLLLMGARTLQGIPQMIGEVIRKGSKNDFFLHYIFMENAFELPT

GAGLQLQISSSGVIAPGAKAGVKLEVANMQAELVAKPSVSVEFVTNMGIIIPDFARSGVQ

MNTNFFHESGLEAHVALKAGKLKFIIPSPKRPVKLLSGGNTLHLVSTTKTEVIPPLIENR

QSWSVCKQVFPGLNYCTSGAYSNASSTDSASYYPLTGDTRLELELRPTGEIEQYSVSATY

ELQREDRALVDTLKFVTQAEGAKQTEATMTFKYNRQSMTLSSEVQIPDFDVDLGTILRVN

DESTEGKTSYRLTLDIQNKKITEVALMGHLSCDTKEERKIKGVISIPRLQAEARSEILAH

WSPAKLLLQMDSSATAYGSTVSKRVAWHYDEEKIEFEWNTGTNVDTKKMTSNFPVDLSDY

PKSLHMYANRLLDHRVPETDMTFRHVGSKLIVAMSSWLQKASGSLPYTQTLQDHLNSLKE

FNLQNMGLPDFHIPENLFLKSDGRVKYTLNKNSLKIEIPLPFGGKSSRDLKMLETVRTPA

LHFKSVGFHLPSREFQVPTFTIPKLYQLQVPLLGVLDLSTNVYSNLYNWSASYSGGNTST

DHFSLRARYHMKADSVVDLLSYNVQGSGETTYDHKNTFTLSCDGSLRHKFLDSNIKFSHV

EKLGNNPVSKGLLIFDASSSWGPQMSASVHLDSKKKQHLFVKEVKIDGQFRVSSFYAKGT

YGLSCQRDPNTGRLNGESNLRFNSSYLQGTNQITGRYEDGTLSLTSTSDLQSGIIKNTAS

LKYENYELTLKSDTNGKYKNFATSNKMDMTFSKQNALLRSEYQADYESLRFFSLLSGSLN

SHGLELNADILGTDKINSGAHKATLRIGQDGISTSATTNLKCSLLVLENELNAELGLSGA

SMKLTTNGRFREHNAKFSLDGKAALTELSLGSAYQAMILGVDSKNIFNFKVSQEGLKLSN

DMMGSYAEMKFDHTNSLNIAGLSLDFSSKLDNIYSSDKFYKQTVNLQLQPYSLVTTLNSD

LKYNALDLTNNGKLRLEPLKLHVAGNLKGAYQNNEIKHIYAISSAALSASYKADTVAKVQ

GVEFSHRLNTDIAGLASAIDMSTNYNSDSLHFSNVFRSVMAPFTMTIDAHTNGNGKLALW

GEHTGQLYSKFLLKAEPLAFTFSHDYKGSTSHHLVSRKSISAALEHKVSALLTPAEQTGT

WKLKTQFNNNEYSQDLDAYNTKDKIGVELTGRTLADLTLLDSPIKVPLLLSEPINIIDAL

EMRDAVEKPQEFTIVAFVKYDKNQDVHSINLPFFETLQEYFERNRQTIIVVVENVQRNLK

HINIDQFVRKYRAALGKLPQQANDYLNSFNWERQVSHAKEKLTALTKKYRITENDIQIAL

DDAKINFNEKLSQLQTYMIQFDQYIKDSYDLHDLKIAIANIIDEIIEKLKSLDEHYHIRV

NLVKTIHDLHLFIENIDFNKSGSSTASWIQNVDTKYQIRIQIQEKLQQLKRHIQNIDIQH

LAGKLKQHIEAIDVRVLLDQLGTTISFERINDVLEHVKHFVINLIGDFEVAEKINAFRAK

VHELIERYEVDQQIQVLMDKLVELTHQYKLKETIQKLSNVLQQVKIKDYFEKLVGFIDDA

VKKLNELSFKTFIEDVNKFLDMLIKKLKSFDYHQFVDETNDKIREVTQRLNGEIQALELP

QKAEALKLFLEETKATVAVYLESLQDTKITLIINWLQEALSSASLAHMKAKFRETLEDTR

DRMYQMDIQQELQRYLSLVGQVYSTLVTYISDWWTLAAKNLTDFAEQYSIQDWAKRMKAL

VEQGFTVPEIKTILGTMPAFEVSLQALQKATFQTPDFIVPLTDLRIPSVQINFKDLKNIK

IPSRFSTPEFTILNTFHIPSFTIDFVEMKVKIIRTIDQMQNSELQWPVPDIYLRDLKVED

IPLARITLPDFRLPEIAIPEFIIPTLNLNDFQVPDLHIPEFQLPHISHTIEVPTFGKLYS

ILKIQSPLFTLDANADIGNGTTSANEAGIAASITAKGESKLEVLNFDFQANAQLSNPKIN

PLALKESVKFSSKYLRTEHGSEMLFFGNAIEGKSNTVASLHTEKNTLELSNGVIVKINNQ

LTLDSNTKYFHKLNIPKLDFSSQADLRNEIKTLLKAGHIAWTSSGKGSWKWACPRFSDEG

THESQISFTIEGPLTSFGLSNKINSKHLRVNQNLVYESGSLNFSKLEIQSQVDSQHVGHS

VLTAKGMALFGEGKAEFTGRHDAHLNGKVIGTLKNSLFFSAQPFEITASTNNEGNLKVRF

PLRLTGKIDFLNNYALFLSPSAQQASWQVSARFNQYKYNQNFSAGNNENIMEAHVGINGE

ANLDFLNIPLTIPEMRLPYTIITTPPLKDFSLWEKTGLKEFLKTTKQSFDLSVKAQYKKN

KHRHSITNPLAVLCEFISQSIKSFDRHFEKNRNNALDFVTKSYNETKIKFDKYKAEKSHD

ELPRTFQIPGYTVPVVNVEVSPFTIEMSAFGYVFPKAVSMPSFSILGSDVRVPSYTLILP

SLELPVLHVPRNLKLSLPHFKELCTISHIFIPAMGNITYDFSFKSSVITLNTNAELFNQS

DIVAHLLSSSSSVIDALQYKLEGTTRLTRKRGLKLATALSLSNKFVEGSHNSTVSLTTKN

MEVSVAKTTKAEIPILRMNFKQELNGNTKSKPTVSSSMEFKYDFNSSMLYSTAKGAVDHK

LSLESLTSYFSIESSTKGDVKGSVLSREYSGTIASEANTYLNSKSTRSSVKLQGTSKIDD

IWNLEVKENFAGEATLQRIYSLWEHSTKNHLQLEGLFFTNGEHTSKATLELSPWQMSALV

QVHASQPSSFHDFPDLGQEVALNANTKNQKIRWKNEVRIHSGSFQSQVELSNDQEKAHLD

IAGSLEGHLRFLKNIILPVYDKSLWDFLKLDVTTSIGRRQHLRVSTAFVYTKNPNGYSFS

IPVKVLADKFITPGLKLNDLNSVLVMPTFHVPFTDLQVPSCKLDFREIQIYKKLRTSSFA

LNLPTLPEVKFPEVDVLTKYSQPEDSLIPFFEITVPESQLTVSQFTLPKSVSDGIAALDL

NAVANKIADFELPTIIVPEQTIEIPSIKFSVPAGIVIPSFQALTARFEVDSPVYNATWSA

SLKNKADYVETVLDSTCSSTVQFLEYELNVLGTHKIEDGTLASKTKGTLAHRDFSAEYEE

DGKFEGLQEWEGKAHLNIKSPAFTDLHLRYQKDKKGISTSAASPAVGTVGMDMDEDDDFS

KWNFYYSPQSSPDKKLTIFKTELRVRESDEETQIKVNWEEEAASGLLTSLKDNVPKATGV

LYDYVNKYHWEHTGLTLREVSSKLRRNLQNNAEWVYQGAIRQIDDIDVRFQKAASGTTGT

YQEWKDKAQNLYQELLTQEGQASFQGLKDNVFDGLVRVTQKFHMKVKHLIDSLIDFLNFP

RFQFPGKPGIYTREELCTMFIREVGTVLSQVYSKVHNGSEILFSYFQDLVITLPFELRKH

KLIDVISMYRELLKDLSKEAQEVFKAIQSLKTTEVLRNLQDLLQFIFQLIEDNIKQLKEM

KFTYLINYIQDEINTIFNDYIPYVFKLLKENLCLNLHKFNEFIQNELQEASQELQQIHQY

IMALREEYFDPSIVGWTVKYYELEEKIVSLIKNLLVALKDFHSEYIVSASNFTSQLSSQV

EQFLHRNIQEYLSILTDPDGKGKEKIAELSATAQEIIKSQAIATKKIISDYHQQFRYKLQ

DFSDQLSDYYEKFIAESKRLIDLSIQNYHTFLIYITELLKKLQSTTVMNPYMKLAPGELT

IIL

>sp|P04114|APOB_HUMAN 2646 STPEFTILNTFHIPSFTI

MDPPRPALLALLALPALLLLLLAGARAEEEMLENVSLVCPKDATRFKHLRKYTYNYEAES

SSGVPGTADSRSATRINCKVELEVPQLCSFILKTSQCTLKEVYGFNPEGKALLKKTKNSE

EFAAAMSRYELKLAIPEGKQVFLYPEKDEPTYILNIKRGIISALLVPPETEEAKQVLFLD

TVYGNCSTHFTVKTRKGNVATEISTERDLGQCDRFKPIRTGISPLALIKGMTRPLSTLIS

SSQSCQYTLDAKRKHVAEAICKEQHLFLPFSYNNKYGMVAQVTQTLKLEDTPKINSRFFG

EGTKKMGLAFESTKSTSPPKQAEAVLKTLQELKKLTISEQNIQRANLFNKLVTELRGLSD

EAVTSLLPQLIEVSSPITLQALVQCGQPQCSTHILQWLKRVHANPLLIDVVTYLVALIPE

PSAQQLREIFNMARDQRSRATLYALSHAVNNYHKTNPTGTQELLDIANYLMEQIQDDCTG

DEDYTYLILRVIGNMGQTMEQLTPELKSSILKCVQSTKPSLMIQKAAIQALRKMEPKDKD

QEVLLQTFLDDASPGDKRLAAYLMLMRSPSQADINKIVQILPWEQNEQVKNFVASHIANI

LNSEELDIQDLKKLVKEALKESQLPTVMDFRKFSRNYQLYKSVSLPSLDPASAKIEGNLI

FDPNNYLPKESMLKTTLTAFGFASADLIEIGLEGKGFEPTLEALFGKQGFFPDSVNKALY

WVNGQVPDGVSKVLVDHFGYTKDDKHEQDMVNGIMLSVEKLIKDLKSKEVPEARAYLRIL

GEELGFASLHDLQLLGKLLLMGARTLQGIPQMIGEVIRKGSKNDFFLHYIFMENAFELPT

GAGLQLQISSSGVIAPGAKAGVKLEVANMQAELVAKPSVSVEFVTNMGIIIPDFARSGVQ

MNTNFFHESGLEAHVALKAGKLKFIIPSPKRPVKLLSGGNTLHLVSTTKTEVIPPLIENR

QSWSVCKQVFPGLNYCTSGAYSNASSTDSASYYPLTGDTRLELELRPTGEIEQYSVSATY

ELQREDRALVDTLKFVTQAEGAKQTEATMTFKYNRQSMTLSSEVQIPDFDVDLGTILRVN

DESTEGKTSYRLTLDIQNKKITEVALMGHLSCDTKEERKIKGVISIPRLQAEARSEILAH

WSPAKLLLQMDSSATAYGSTVSKRVAWHYDEEKIEFEWNTGTNVDTKKMTSNFPVDLSDY

PKSLHMYANRLLDHRVPETDMTFRHVGSKLIVAMSSWLQKASGSLPYTQTLQDHLNSLKE

FNLQNMGLPDFHIPENLFLKSDGRVKYTLNKNSLKIEIPLPFGGKSSRDLKMLETVRTPA

LHFKSVGFHLPSREFQVPTFTIPKLYQLQVPLLGVLDLSTNVYSNLYNWSASYSGGNTST

DHFSLRARYHMKADSVVDLLSYNVQGSGETTYDHKNTFTLSCDGSLRHKFLDSNIKFSHV

EKLGNNPVSKGLLIFDASSSWGPQMSASVHLDSKKKQHLFVKEVKIDGQFRVSSFYAKGT

YGLSCQRDPNTGRLNGESNLRFNSSYLQGTNQITGRYEDGTLSLTSTSDLQSGIIKNTAS

LKYENYELTLKSDTNGKYKNFATSNKMDMTFSKQNALLRSEYQADYESLRFFSLLSGSLN

SHGLELNADILGTDKINSGAHKATLRIGQDGISTSATTNLKCSLLVLENELNAELGLSGA

SMKLTTNGRFREHNAKFSLDGKAALTELSLGSAYQAMILGVDSKNIFNFKVSQEGLKLSN

DMMGSYAEMKFDHTNSLNIAGLSLDFSSKLDNIYSSDKFYKQTVNLQLQPYSLVTTLNSD

LKYNALDLTNNGKLRLEPLKLHVAGNLKGAYQNNEIKHIYAISSAALSASYKADTVAKVQ

GVEFSHRLNTDIAGLASAIDMSTNYNSDSLHFSNVFRSVMAPFTMTIDAHTNGNGKLALW

GEHTGQLYSKFLLKAEPLAFTFSHDYKGSTSHHLVSRKSISAALEHKVSALLTPAEQTGT

WKLKTQFNNNEYSQDLDAYNTKDKIGVELTGRTLADLTLLDSPIKVPLLLSEPINIIDAL

EMRDAVEKPQEFTIVAFVKYDKNQDVHSINLPFFETLQEYFERNRQTIIVVVENVQRNLK

HINIDQFVRKYRAALGKLPQQANDYLNSFNWERQVSHAKEKLTALTKKYRITENDIQIAL

DDAKINFNEKLSQLQTYMIQFDQYIKDSYDLHDLKIAIANIIDEIIEKLKSLDEHYHIRV

NLVKTIHDLHLFIENIDFNKSGSSTASWIQNVDTKYQIRIQIQEKLQQLKRHIQNIDIQH

LAGKLKQHIEAIDVRVLLDQLGTTISFERINDVLEHVKHFVINLIGDFEVAEKINAFRAK

VHELIERYEVDQQIQVLMDKLVELTHQYKLKETIQKLSNVLQQVKIKDYFEKLVGFIDDA

VKKLNELSFKTFIEDVNKFLDMLIKKLKSFDYHQFVDETNDKIREVTQRLNGEIQALELP

QKAEALKLFLEETKATVAVYLESLQDTKITLIINWLQEALSSASLAHMKAKFRETLEDTR

DRMYQMDIQQELQRYLSLVGQVYSTLVTYISDWWTLAAKNLTDFAEQYSIQDWAKRMKAL

VEQGFTVPEIKTILGTMPAFEVSLQALQKATFQTPDFIVPLTDLRIPSVQINFKDLKNIK

IPSRFSTPEFTILNTFHIPSFTIDFVEMKVKIIRTIDQMQNSELQWPVPDIYLRDLKVED

IPLARITLPDFRLPEIAIPEFIIPTLNLNDFQVPDLHIPEFQLPHISHTIEVPTFGKLYS

ILKIQSPLFTLDANADIGNGTTSANEAGIAASITAKGESKLEVLNFDFQANAQLSNPKIN

PLALKESVKFSSKYLRTEHGSEMLFFGNAIEGKSNTVASLHTEKNTLELSNGVIVKINNQ

LTLDSNTKYFHKLNIPKLDFSSQADLRNEIKTLLKAGHIAWTSSGKGSWKWACPRFSDEG

THESQISFTIEGPLTSFGLSNKINSKHLRVNQNLVYESGSLNFSKLEIQSQVDSQHVGHS

VLTAKGMALFGEGKAEFTGRHDAHLNGKVIGTLKNSLFFSAQPFEITASTNNEGNLKVRF

PLRLTGKIDFLNNYALFLSPSAQQASWQVSARFNQYKYNQNFSAGNNENIMEAHVGINGE

ANLDFLNIPLTIPEMRLPYTIITTPPLKDFSLWEKTGLKEFLKTTKQSFDLSVKAQYKKN

KHRHSITNPLAVLCEFISQSIKSFDRHFEKNRNNALDFVTKSYNETKIKFDKYKAEKSHD

ELPRTFQIPGYTVPVVNVEVSPFTIEMSAFGYVFPKAVSMPSFSILGSDVRVPSYTLILP

SLELPVLHVPRNLKLSLPHFKELCTISHIFIPAMGNITYDFSFKSSVITLNTNAELFNQS

DIVAHLLSSSSSVIDALQYKLEGTTRLTRKRGLKLATALSLSNKFVEGSHNSTVSLTTKN

MEVSVAKTTKAEIPILRMNFKQELNGNTKSKPTVSSSMEFKYDFNSSMLYSTAKGAVDHK

LSLESLTSYFSIESSTKGDVKGSVLSREYSGTIASEANTYLNSKSTRSSVKLQGTSKIDD

IWNLEVKENFAGEATLQRIYSLWEHSTKNHLQLEGLFFTNGEHTSKATLELSPWQMSALV

QVHASQPSSFHDFPDLGQEVALNANTKNQKIRWKNEVRIHSGSFQSQVELSNDQEKAHLD

IAGSLEGHLRFLKNIILPVYDKSLWDFLKLDVTTSIGRRQHLRVSTAFVYTKNPNGYSFS

IPVKVLADKFITPGLKLNDLNSVLVMPTFHVPFTDLQVPSCKLDFREIQIYKKLRTSSFA

LNLPTLPEVKFPEVDVLTKYSQPEDSLIPFFEITVPESQLTVSQFTLPKSVSDGIAALDL

NAVANKIADFELPTIIVPEQTIEIPSIKFSVPAGIVIPSFQALTARFEVDSPVYNATWSA

SLKNKADYVETVLDSTCSSTVQFLEYELNVLGTHKIEDGTLASKTKGTLAHRDFSAEYEE

DGKFEGLQEWEGKAHLNIKSPAFTDLHLRYQKDKKGISTSAASPAVGTVGMDMDEDDDFS

KWNFYYSPQSSPDKKLTIFKTELRVRESDEETQIKVNWEEEAASGLLTSLKDNVPKATGV

LYDYVNKYHWEHTGLTLREVSSKLRRNLQNNAEWVYQGAIRQIDDIDVRFQKAASGTTGT

YQEWKDKAQNLYQELLTQEGQASFQGLKDNVFDGLVRVTQKFHMKVKHLIDSLIDFLNFP

RFQFPGKPGIYTREELCTMFIREVGTVLSQVYSKVHNGSEILFSYFQDLVITLPFELRKH

KLIDVISMYRELLKDLSKEAQEVFKAIQSLKTTEVLRNLQDLLQFIFQLIEDNIKQLKEM

KFTYLINYIQDEINTIFNDYIPYVFKLLKENLCLNLHKFNEFIQNELQEASQELQQIHQY

IMALREEYFDPSIVGWTVKYYELEEKIVSLIKNLLVALKDFHSEYIVSASNFTSQLSSQV

EQFLHRNIQEYLSILTDPDGKGKEKIAELSATAQEIIKSQAIATKKIISDYHQQFRYKLQ

DFSDQLSDYYEKFIAESKRLIDLSIQNYHTFLIYITELLKKLQSTTVMNPYMKLAPGELT

IIL

>sp|P04114|APOB_HUMAN 2614 TPDFIVPLTDLRIP

MDPPRPALLALLALPALLLLLLAGARAEEEMLENVSLVCPKDATRFKHLRKYTYNYEAES

SSGVPGTADSRSATRINCKVELEVPQLCSFILKTSQCTLKEVYGFNPEGKALLKKTKNSE

EFAAAMSRYELKLAIPEGKQVFLYPEKDEPTYILNIKRGIISALLVPPETEEAKQVLFLD

TVYGNCSTHFTVKTRKGNVATEISTERDLGQCDRFKPIRTGISPLALIKGMTRPLSTLIS

SSQSCQYTLDAKRKHVAEAICKEQHLFLPFSYNNKYGMVAQVTQTLKLEDTPKINSRFFG

EGTKKMGLAFESTKSTSPPKQAEAVLKTLQELKKLTISEQNIQRANLFNKLVTELRGLSD

EAVTSLLPQLIEVSSPITLQALVQCGQPQCSTHILQWLKRVHANPLLIDVVTYLVALIPE

PSAQQLREIFNMARDQRSRATLYALSHAVNNYHKTNPTGTQELLDIANYLMEQIQDDCTG

DEDYTYLILRVIGNMGQTMEQLTPELKSSILKCVQSTKPSLMIQKAAIQALRKMEPKDKD

QEVLLQTFLDDASPGDKRLAAYLMLMRSPSQADINKIVQILPWEQNEQVKNFVASHIANI

LNSEELDIQDLKKLVKEALKESQLPTVMDFRKFSRNYQLYKSVSLPSLDPASAKIEGNLI

FDPNNYLPKESMLKTTLTAFGFASADLIEIGLEGKGFEPTLEALFGKQGFFPDSVNKALY

WVNGQVPDGVSKVLVDHFGYTKDDKHEQDMVNGIMLSVEKLIKDLKSKEVPEARAYLRIL

GEELGFASLHDLQLLGKLLLMGARTLQGIPQMIGEVIRKGSKNDFFLHYIFMENAFELPT

GAGLQLQISSSGVIAPGAKAGVKLEVANMQAELVAKPSVSVEFVTNMGIIIPDFARSGVQ

MNTNFFHESGLEAHVALKAGKLKFIIPSPKRPVKLLSGGNTLHLVSTTKTEVIPPLIENR

QSWSVCKQVFPGLNYCTSGAYSNASSTDSASYYPLTGDTRLELELRPTGEIEQYSVSATY

ELQREDRALVDTLKFVTQAEGAKQTEATMTFKYNRQSMTLSSEVQIPDFDVDLGTILRVN

DESTEGKTSYRLTLDIQNKKITEVALMGHLSCDTKEERKIKGVISIPRLQAEARSEILAH

WSPAKLLLQMDSSATAYGSTVSKRVAWHYDEEKIEFEWNTGTNVDTKKMTSNFPVDLSDY

PKSLHMYANRLLDHRVPETDMTFRHVGSKLIVAMSSWLQKASGSLPYTQTLQDHLNSLKE

FNLQNMGLPDFHIPENLFLKSDGRVKYTLNKNSLKIEIPLPFGGKSSRDLKMLETVRTPA

LHFKSVGFHLPSREFQVPTFTIPKLYQLQVPLLGVLDLSTNVYSNLYNWSASYSGGNTST

DHFSLRARYHMKADSVVDLLSYNVQGSGETTYDHKNTFTLSCDGSLRHKFLDSNIKFSHV

EKLGNNPVSKGLLIFDASSSWGPQMSASVHLDSKKKQHLFVKEVKIDGQFRVSSFYAKGT

YGLSCQRDPNTGRLNGESNLRFNSSYLQGTNQITGRYEDGTLSLTSTSDLQSGIIKNTAS

LKYENYELTLKSDTNGKYKNFATSNKMDMTFSKQNALLRSEYQADYESLRFFSLLSGSLN

SHGLELNADILGTDKINSGAHKATLRIGQDGISTSATTNLKCSLLVLENELNAELGLSGA

SMKLTTNGRFREHNAKFSLDGKAALTELSLGSAYQAMILGVDSKNIFNFKVSQEGLKLSN

DMMGSYAEMKFDHTNSLNIAGLSLDFSSKLDNIYSSDKFYKQTVNLQLQPYSLVTTLNSD

LKYNALDLTNNGKLRLEPLKLHVAGNLKGAYQNNEIKHIYAISSAALSASYKADTVAKVQ

GVEFSHRLNTDIAGLASAIDMSTNYNSDSLHFSNVFRSVMAPFTMTIDAHTNGNGKLALW

GEHTGQLYSKFLLKAEPLAFTFSHDYKGSTSHHLVSRKSISAALEHKVSALLTPAEQTGT

WKLKTQFNNNEYSQDLDAYNTKDKIGVELTGRTLADLTLLDSPIKVPLLLSEPINIIDAL

EMRDAVEKPQEFTIVAFVKYDKNQDVHSINLPFFETLQEYFERNRQTIIVVVENVQRNLK

HINIDQFVRKYRAALGKLPQQANDYLNSFNWERQVSHAKEKLTALTKKYRITENDIQIAL

DDAKINFNEKLSQLQTYMIQFDQYIKDSYDLHDLKIAIANIIDEIIEKLKSLDEHYHIRV

NLVKTIHDLHLFIENIDFNKSGSSTASWIQNVDTKYQIRIQIQEKLQQLKRHIQNIDIQH

LAGKLKQHIEAIDVRVLLDQLGTTISFERINDVLEHVKHFVINLIGDFEVAEKINAFRAK

VHELIERYEVDQQIQVLMDKLVELTHQYKLKETIQKLSNVLQQVKIKDYFEKLVGFIDDA

VKKLNELSFKTFIEDVNKFLDMLIKKLKSFDYHQFVDETNDKIREVTQRLNGEIQALELP

QKAEALKLFLEETKATVAVYLESLQDTKITLIINWLQEALSSASLAHMKAKFRETLEDTR

DRMYQMDIQQELQRYLSLVGQVYSTLVTYISDWWTLAAKNLTDFAEQYSIQDWAKRMKAL

VEQGFTVPEIKTILGTMPAFEVSLQALQKATFQTPDFIVPLTDLRIPSVQINFKDLKNIK

IPSRFSTPEFTILNTFHIPSFTIDFVEMKVKIIRTIDQMQNSELQWPVPDIYLRDLKVED

IPLARITLPDFRLPEIAIPEFIIPTLNLNDFQVPDLHIPEFQLPHISHTIEVPTFGKLYS

ILKIQSPLFTLDANADIGNGTTSANEAGIAASITAKGESKLEVLNFDFQANAQLSNPKIN

PLALKESVKFSSKYLRTEHGSEMLFFGNAIEGKSNTVASLHTEKNTLELSNGVIVKINNQ

LTLDSNTKYFHKLNIPKLDFSSQADLRNEIKTLLKAGHIAWTSSGKGSWKWACPRFSDEG

THESQISFTIEGPLTSFGLSNKINSKHLRVNQNLVYESGSLNFSKLEIQSQVDSQHVGHS

VLTAKGMALFGEGKAEFTGRHDAHLNGKVIGTLKNSLFFSAQPFEITASTNNEGNLKVRF

PLRLTGKIDFLNNYALFLSPSAQQASWQVSARFNQYKYNQNFSAGNNENIMEAHVGINGE

ANLDFLNIPLTIPEMRLPYTIITTPPLKDFSLWEKTGLKEFLKTTKQSFDLSVKAQYKKN

KHRHSITNPLAVLCEFISQSIKSFDRHFEKNRNNALDFVTKSYNETKIKFDKYKAEKSHD

ELPRTFQIPGYTVPVVNVEVSPFTIEMSAFGYVFPKAVSMPSFSILGSDVRVPSYTLILP

SLELPVLHVPRNLKLSLPHFKELCTISHIFIPAMGNITYDFSFKSSVITLNTNAELFNQS

DIVAHLLSSSSSVIDALQYKLEGTTRLTRKRGLKLATALSLSNKFVEGSHNSTVSLTTKN

MEVSVAKTTKAEIPILRMNFKQELNGNTKSKPTVSSSMEFKYDFNSSMLYSTAKGAVDHK

LSLESLTSYFSIESSTKGDVKGSVLSREYSGTIASEANTYLNSKSTRSSVKLQGTSKIDD

IWNLEVKENFAGEATLQRIYSLWEHSTKNHLQLEGLFFTNGEHTSKATLELSPWQMSALV

QVHASQPSSFHDFPDLGQEVALNANTKNQKIRWKNEVRIHSGSFQSQVELSNDQEKAHLD

IAGSLEGHLRFLKNIILPVYDKSLWDFLKLDVTTSIGRRQHLRVSTAFVYTKNPNGYSFS

IPVKVLADKFITPGLKLNDLNSVLVMPTFHVPFTDLQVPSCKLDFREIQIYKKLRTSSFA

LNLPTLPEVKFPEVDVLTKYSQPEDSLIPFFEITVPESQLTVSQFTLPKSVSDGIAALDL

NAVANKIADFELPTIIVPEQTIEIPSIKFSVPAGIVIPSFQALTARFEVDSPVYNATWSA

SLKNKADYVETVLDSTCSSTVQFLEYELNVLGTHKIEDGTLASKTKGTLAHRDFSAEYEE

DGKFEGLQEWEGKAHLNIKSPAFTDLHLRYQKDKKGISTSAASPAVGTVGMDMDEDDDFS

KWNFYYSPQSSPDKKLTIFKTELRVRESDEETQIKVNWEEEAASGLLTSLKDNVPKATGV

LYDYVNKYHWEHTGLTLREVSSKLRRNLQNNAEWVYQGAIRQIDDIDVRFQKAASGTTGT

YQEWKDKAQNLYQELLTQEGQASFQGLKDNVFDGLVRVTQKFHMKVKHLIDSLIDFLNFP

RFQFPGKPGIYTREELCTMFIREVGTVLSQVYSKVHNGSEILFSYFQDLVITLPFELRKH

KLIDVISMYRELLKDLSKEAQEVFKAIQSLKTTEVLRNLQDLLQFIFQLIEDNIKQLKEM

KFTYLINYIQDEINTIFNDYIPYVFKLLKENLCLNLHKFNEFIQNELQEASQELQQIHQY

IMALREEYFDPSIVGWTVKYYELEEKIVSLIKNLLVALKDFHSEYIVSASNFTSQLSSQV

EQFLHRNIQEYLSILTDPDGKGKEKIAELSATAQEIIKSQAIATKKIISDYHQQFRYKLQ

DFSDQLSDYYEKFIAESKRLIDLSIQNYHTFLIYITELLKKLQSTTVMNPYMKLAPGELT

IIL

>sp|P04114|APOB_HUMAN 2614 TPDFIVPLTDLRIPS

MDPPRPALLALLALPALLLLLLAGARAEEEMLENVSLVCPKDATRFKHLRKYTYNYEAES

SSGVPGTADSRSATRINCKVELEVPQLCSFILKTSQCTLKEVYGFNPEGKALLKKTKNSE

EFAAAMSRYELKLAIPEGKQVFLYPEKDEPTYILNIKRGIISALLVPPETEEAKQVLFLD

TVYGNCSTHFTVKTRKGNVATEISTERDLGQCDRFKPIRTGISPLALIKGMTRPLSTLIS

SSQSCQYTLDAKRKHVAEAICKEQHLFLPFSYNNKYGMVAQVTQTLKLEDTPKINSRFFG

EGTKKMGLAFESTKSTSPPKQAEAVLKTLQELKKLTISEQNIQRANLFNKLVTELRGLSD

EAVTSLLPQLIEVSSPITLQALVQCGQPQCSTHILQWLKRVHANPLLIDVVTYLVALIPE

PSAQQLREIFNMARDQRSRATLYALSHAVNNYHKTNPTGTQELLDIANYLMEQIQDDCTG

DEDYTYLILRVIGNMGQTMEQLTPELKSSILKCVQSTKPSLMIQKAAIQALRKMEPKDKD

QEVLLQTFLDDASPGDKRLAAYLMLMRSPSQADINKIVQILPWEQNEQVKNFVASHIANI

LNSEELDIQDLKKLVKEALKESQLPTVMDFRKFSRNYQLYKSVSLPSLDPASAKIEGNLI

FDPNNYLPKESMLKTTLTAFGFASADLIEIGLEGKGFEPTLEALFGKQGFFPDSVNKALY

WVNGQVPDGVSKVLVDHFGYTKDDKHEQDMVNGIMLSVEKLIKDLKSKEVPEARAYLRIL

GEELGFASLHDLQLLGKLLLMGARTLQGIPQMIGEVIRKGSKNDFFLHYIFMENAFELPT

GAGLQLQISSSGVIAPGAKAGVKLEVANMQAELVAKPSVSVEFVTNMGIIIPDFARSGVQ

MNTNFFHESGLEAHVALKAGKLKFIIPSPKRPVKLLSGGNTLHLVSTTKTEVIPPLIENR

QSWSVCKQVFPGLNYCTSGAYSNASSTDSASYYPLTGDTRLELELRPTGEIEQYSVSATY

ELQREDRALVDTLKFVTQAEGAKQTEATMTFKYNRQSMTLSSEVQIPDFDVDLGTILRVN

DESTEGKTSYRLTLDIQNKKITEVALMGHLSCDTKEERKIKGVISIPRLQAEARSEILAH

WSPAKLLLQMDSSATAYGSTVSKRVAWHYDEEKIEFEWNTGTNVDTKKMTSNFPVDLSDY

PKSLHMYANRLLDHRVPETDMTFRHVGSKLIVAMSSWLQKASGSLPYTQTLQDHLNSLKE

FNLQNMGLPDFHIPENLFLKSDGRVKYTLNKNSLKIEIPLPFGGKSSRDLKMLETVRTPA

LHFKSVGFHLPSREFQVPTFTIPKLYQLQVPLLGVLDLSTNVYSNLYNWSASYSGGNTST

DHFSLRARYHMKADSVVDLLSYNVQGSGETTYDHKNTFTLSCDGSLRHKFLDSNIKFSHV

EKLGNNPVSKGLLIFDASSSWGPQMSASVHLDSKKKQHLFVKEVKIDGQFRVSSFYAKGT

YGLSCQRDPNTGRLNGESNLRFNSSYLQGTNQITGRYEDGTLSLTSTSDLQSGIIKNTAS

LKYENYELTLKSDTNGKYKNFATSNKMDMTFSKQNALLRSEYQADYESLRFFSLLSGSLN

SHGLELNADILGTDKINSGAHKATLRIGQDGISTSATTNLKCSLLVLENELNAELGLSGA

SMKLTTNGRFREHNAKFSLDGKAALTELSLGSAYQAMILGVDSKNIFNFKVSQEGLKLSN

DMMGSYAEMKFDHTNSLNIAGLSLDFSSKLDNIYSSDKFYKQTVNLQLQPYSLVTTLNSD

LKYNALDLTNNGKLRLEPLKLHVAGNLKGAYQNNEIKHIYAISSAALSASYKADTVAKVQ

GVEFSHRLNTDIAGLASAIDMSTNYNSDSLHFSNVFRSVMAPFTMTIDAHTNGNGKLALW

GEHTGQLYSKFLLKAEPLAFTFSHDYKGSTSHHLVSRKSISAALEHKVSALLTPAEQTGT

WKLKTQFNNNEYSQDLDAYNTKDKIGVELTGRTLADLTLLDSPIKVPLLLSEPINIIDAL

EMRDAVEKPQEFTIVAFVKYDKNQDVHSINLPFFETLQEYFERNRQTIIVVVENVQRNLK

HINIDQFVRKYRAALGKLPQQANDYLNSFNWERQVSHAKEKLTALTKKYRITENDIQIAL

DDAKINFNEKLSQLQTYMIQFDQYIKDSYDLHDLKIAIANIIDEIIEKLKSLDEHYHIRV

NLVKTIHDLHLFIENIDFNKSGSSTASWIQNVDTKYQIRIQIQEKLQQLKRHIQNIDIQH

LAGKLKQHIEAIDVRVLLDQLGTTISFERINDVLEHVKHFVINLIGDFEVAEKINAFRAK

VHELIERYEVDQQIQVLMDKLVELTHQYKLKETIQKLSNVLQQVKIKDYFEKLVGFIDDA

VKKLNELSFKTFIEDVNKFLDMLIKKLKSFDYHQFVDETNDKIREVTQRLNGEIQALELP

QKAEALKLFLEETKATVAVYLESLQDTKITLIINWLQEALSSASLAHMKAKFRETLEDTR

DRMYQMDIQQELQRYLSLVGQVYSTLVTYISDWWTLAAKNLTDFAEQYSIQDWAKRMKAL

VEQGFTVPEIKTILGTMPAFEVSLQALQKATFQTPDFIVPLTDLRIPSVQINFKDLKNIK

IPSRFSTPEFTILNTFHIPSFTIDFVEMKVKIIRTIDQMQNSELQWPVPDIYLRDLKVED

IPLARITLPDFRLPEIAIPEFIIPTLNLNDFQVPDLHIPEFQLPHISHTIEVPTFGKLYS

ILKIQSPLFTLDANADIGNGTTSANEAGIAASITAKGESKLEVLNFDFQANAQLSNPKIN

PLALKESVKFSSKYLRTEHGSEMLFFGNAIEGKSNTVASLHTEKNTLELSNGVIVKINNQ

LTLDSNTKYFHKLNIPKLDFSSQADLRNEIKTLLKAGHIAWTSSGKGSWKWACPRFSDEG

THESQISFTIEGPLTSFGLSNKINSKHLRVNQNLVYESGSLNFSKLEIQSQVDSQHVGHS

VLTAKGMALFGEGKAEFTGRHDAHLNGKVIGTLKNSLFFSAQPFEITASTNNEGNLKVRF

PLRLTGKIDFLNNYALFLSPSAQQASWQVSARFNQYKYNQNFSAGNNENIMEAHVGINGE

ANLDFLNIPLTIPEMRLPYTIITTPPLKDFSLWEKTGLKEFLKTTKQSFDLSVKAQYKKN

KHRHSITNPLAVLCEFISQSIKSFDRHFEKNRNNALDFVTKSYNETKIKFDKYKAEKSHD

ELPRTFQIPGYTVPVVNVEVSPFTIEMSAFGYVFPKAVSMPSFSILGSDVRVPSYTLILP

SLELPVLHVPRNLKLSLPHFKELCTISHIFIPAMGNITYDFSFKSSVITLNTNAELFNQS

DIVAHLLSSSSSVIDALQYKLEGTTRLTRKRGLKLATALSLSNKFVEGSHNSTVSLTTKN

MEVSVAKTTKAEIPILRMNFKQELNGNTKSKPTVSSSMEFKYDFNSSMLYSTAKGAVDHK

LSLESLTSYFSIESSTKGDVKGSVLSREYSGTIASEANTYLNSKSTRSSVKLQGTSKIDD

IWNLEVKENFAGEATLQRIYSLWEHSTKNHLQLEGLFFTNGEHTSKATLELSPWQMSALV

QVHASQPSSFHDFPDLGQEVALNANTKNQKIRWKNEVRIHSGSFQSQVELSNDQEKAHLD

IAGSLEGHLRFLKNIILPVYDKSLWDFLKLDVTTSIGRRQHLRVSTAFVYTKNPNGYSFS

IPVKVLADKFITPGLKLNDLNSVLVMPTFHVPFTDLQVPSCKLDFREIQIYKKLRTSSFA

LNLPTLPEVKFPEVDVLTKYSQPEDSLIPFFEITVPESQLTVSQFTLPKSVSDGIAALDL

NAVANKIADFELPTIIVPEQTIEIPSIKFSVPAGIVIPSFQALTARFEVDSPVYNATWSA

SLKNKADYVETVLDSTCSSTVQFLEYELNVLGTHKIEDGTLASKTKGTLAHRDFSAEYEE

DGKFEGLQEWEGKAHLNIKSPAFTDLHLRYQKDKKGISTSAASPAVGTVGMDMDEDDDFS

KWNFYYSPQSSPDKKLTIFKTELRVRESDEETQIKVNWEEEAASGLLTSLKDNVPKATGV

LYDYVNKYHWEHTGLTLREVSSKLRRNLQNNAEWVYQGAIRQIDDIDVRFQKAASGTTGT

YQEWKDKAQNLYQELLTQEGQASFQGLKDNVFDGLVRVTQKFHMKVKHLIDSLIDFLNFP

RFQFPGKPGIYTREELCTMFIREVGTVLSQVYSKVHNGSEILFSYFQDLVITLPFELRKH

KLIDVISMYRELLKDLSKEAQEVFKAIQSLKTTEVLRNLQDLLQFIFQLIEDNIKQLKEM

KFTYLINYIQDEINTIFNDYIPYVFKLLKENLCLNLHKFNEFIQNELQEASQELQQIHQY

IMALREEYFDPSIVGWTVKYYELEEKIVSLIKNLLVALKDFHSEYIVSASNFTSQLSSQV

EQFLHRNIQEYLSILTDPDGKGKEKIAELSATAQEIIKSQAIATKKIISDYHQQFRYKLQ

DFSDQLSDYYEKFIAESKRLIDLSIQNYHTFLIYITELLKKLQSTTVMNPYMKLAPGELT

IIL

>sp|P04114|APOB_HUMAN 1794 VTTLNSDLKYNALDLTN

MDPPRPALLALLALPALLLLLLAGARAEEEMLENVSLVCPKDATRFKHLRKYTYNYEAES

SSGVPGTADSRSATRINCKVELEVPQLCSFILKTSQCTLKEVYGFNPEGKALLKKTKNSE

EFAAAMSRYELKLAIPEGKQVFLYPEKDEPTYILNIKRGIISALLVPPETEEAKQVLFLD

TVYGNCSTHFTVKTRKGNVATEISTERDLGQCDRFKPIRTGISPLALIKGMTRPLSTLIS

SSQSCQYTLDAKRKHVAEAICKEQHLFLPFSYNNKYGMVAQVTQTLKLEDTPKINSRFFG

EGTKKMGLAFESTKSTSPPKQAEAVLKTLQELKKLTISEQNIQRANLFNKLVTELRGLSD

EAVTSLLPQLIEVSSPITLQALVQCGQPQCSTHILQWLKRVHANPLLIDVVTYLVALIPE

PSAQQLREIFNMARDQRSRATLYALSHAVNNYHKTNPTGTQELLDIANYLMEQIQDDCTG

DEDYTYLILRVIGNMGQTMEQLTPELKSSILKCVQSTKPSLMIQKAAIQALRKMEPKDKD

QEVLLQTFLDDASPGDKRLAAYLMLMRSPSQADINKIVQILPWEQNEQVKNFVASHIANI

LNSEELDIQDLKKLVKEALKESQLPTVMDFRKFSRNYQLYKSVSLPSLDPASAKIEGNLI

FDPNNYLPKESMLKTTLTAFGFASADLIEIGLEGKGFEPTLEALFGKQGFFPDSVNKALY

WVNGQVPDGVSKVLVDHFGYTKDDKHEQDMVNGIMLSVEKLIKDLKSKEVPEARAYLRIL

GEELGFASLHDLQLLGKLLLMGARTLQGIPQMIGEVIRKGSKNDFFLHYIFMENAFELPT

GAGLQLQISSSGVIAPGAKAGVKLEVANMQAELVAKPSVSVEFVTNMGIIIPDFARSGVQ

MNTNFFHESGLEAHVALKAGKLKFIIPSPKRPVKLLSGGNTLHLVSTTKTEVIPPLIENR

QSWSVCKQVFPGLNYCTSGAYSNASSTDSASYYPLTGDTRLELELRPTGEIEQYSVSATY

ELQREDRALVDTLKFVTQAEGAKQTEATMTFKYNRQSMTLSSEVQIPDFDVDLGTILRVN

DESTEGKTSYRLTLDIQNKKITEVALMGHLSCDTKEERKIKGVISIPRLQAEARSEILAH

WSPAKLLLQMDSSATAYGSTVSKRVAWHYDEEKIEFEWNTGTNVDTKKMTSNFPVDLSDY

PKSLHMYANRLLDHRVPETDMTFRHVGSKLIVAMSSWLQKASGSLPYTQTLQDHLNSLKE

FNLQNMGLPDFHIPENLFLKSDGRVKYTLNKNSLKIEIPLPFGGKSSRDLKMLETVRTPA

LHFKSVGFHLPSREFQVPTFTIPKLYQLQVPLLGVLDLSTNVYSNLYNWSASYSGGNTST

DHFSLRARYHMKADSVVDLLSYNVQGSGETTYDHKNTFTLSCDGSLRHKFLDSNIKFSHV

EKLGNNPVSKGLLIFDASSSWGPQMSASVHLDSKKKQHLFVKEVKIDGQFRVSSFYAKGT

YGLSCQRDPNTGRLNGESNLRFNSSYLQGTNQITGRYEDGTLSLTSTSDLQSGIIKNTAS

LKYENYELTLKSDTNGKYKNFATSNKMDMTFSKQNALLRSEYQADYESLRFFSLLSGSLN

SHGLELNADILGTDKINSGAHKATLRIGQDGISTSATTNLKCSLLVLENELNAELGLSGA

SMKLTTNGRFREHNAKFSLDGKAALTELSLGSAYQAMILGVDSKNIFNFKVSQEGLKLSN

DMMGSYAEMKFDHTNSLNIAGLSLDFSSKLDNIYSSDKFYKQTVNLQLQPYSLVTTLNSD

LKYNALDLTNNGKLRLEPLKLHVAGNLKGAYQNNEIKHIYAISSAALSASYKADTVAKVQ

GVEFSHRLNTDIAGLASAIDMSTNYNSDSLHFSNVFRSVMAPFTMTIDAHTNGNGKLALW

GEHTGQLYSKFLLKAEPLAFTFSHDYKGSTSHHLVSRKSISAALEHKVSALLTPAEQTGT

WKLKTQFNNNEYSQDLDAYNTKDKIGVELTGRTLADLTLLDSPIKVPLLLSEPINIIDAL

EMRDAVEKPQEFTIVAFVKYDKNQDVHSINLPFFETLQEYFERNRQTIIVVVENVQRNLK

HINIDQFVRKYRAALGKLPQQANDYLNSFNWERQVSHAKEKLTALTKKYRITENDIQIAL

DDAKINFNEKLSQLQTYMIQFDQYIKDSYDLHDLKIAIANIIDEIIEKLKSLDEHYHIRV

NLVKTIHDLHLFIENIDFNKSGSSTASWIQNVDTKYQIRIQIQEKLQQLKRHIQNIDIQH

LAGKLKQHIEAIDVRVLLDQLGTTISFERINDVLEHVKHFVINLIGDFEVAEKINAFRAK

VHELIERYEVDQQIQVLMDKLVELTHQYKLKETIQKLSNVLQQVKIKDYFEKLVGFIDDA

VKKLNELSFKTFIEDVNKFLDMLIKKLKSFDYHQFVDETNDKIREVTQRLNGEIQALELP

QKAEALKLFLEETKATVAVYLESLQDTKITLIINWLQEALSSASLAHMKAKFRETLEDTR

DRMYQMDIQQELQRYLSLVGQVYSTLVTYISDWWTLAAKNLTDFAEQYSIQDWAKRMKAL

VEQGFTVPEIKTILGTMPAFEVSLQALQKATFQTPDFIVPLTDLRIPSVQINFKDLKNIK

IPSRFSTPEFTILNTFHIPSFTIDFVEMKVKIIRTIDQMQNSELQWPVPDIYLRDLKVED

IPLARITLPDFRLPEIAIPEFIIPTLNLNDFQVPDLHIPEFQLPHISHTIEVPTFGKLYS

ILKIQSPLFTLDANADIGNGTTSANEAGIAASITAKGESKLEVLNFDFQANAQLSNPKIN

PLALKESVKFSSKYLRTEHGSEMLFFGNAIEGKSNTVASLHTEKNTLELSNGVIVKINNQ

LTLDSNTKYFHKLNIPKLDFSSQADLRNEIKTLLKAGHIAWTSSGKGSWKWACPRFSDEG

THESQISFTIEGPLTSFGLSNKINSKHLRVNQNLVYESGSLNFSKLEIQSQVDSQHVGHS

VLTAKGMALFGEGKAEFTGRHDAHLNGKVIGTLKNSLFFSAQPFEITASTNNEGNLKVRF

PLRLTGKIDFLNNYALFLSPSAQQASWQVSARFNQYKYNQNFSAGNNENIMEAHVGINGE

ANLDFLNIPLTIPEMRLPYTIITTPPLKDFSLWEKTGLKEFLKTTKQSFDLSVKAQYKKN

KHRHSITNPLAVLCEFISQSIKSFDRHFEKNRNNALDFVTKSYNETKIKFDKYKAEKSHD

ELPRTFQIPGYTVPVVNVEVSPFTIEMSAFGYVFPKAVSMPSFSILGSDVRVPSYTLILP

SLELPVLHVPRNLKLSLPHFKELCTISHIFIPAMGNITYDFSFKSSVITLNTNAELFNQS

DIVAHLLSSSSSVIDALQYKLEGTTRLTRKRGLKLATALSLSNKFVEGSHNSTVSLTTKN

MEVSVAKTTKAEIPILRMNFKQELNGNTKSKPTVSSSMEFKYDFNSSMLYSTAKGAVDHK

LSLESLTSYFSIESSTKGDVKGSVLSREYSGTIASEANTYLNSKSTRSSVKLQGTSKIDD

IWNLEVKENFAGEATLQRIYSLWEHSTKNHLQLEGLFFTNGEHTSKATLELSPWQMSALV

QVHASQPSSFHDFPDLGQEVALNANTKNQKIRWKNEVRIHSGSFQSQVELSNDQEKAHLD

IAGSLEGHLRFLKNIILPVYDKSLWDFLKLDVTTSIGRRQHLRVSTAFVYTKNPNGYSFS

IPVKVLADKFITPGLKLNDLNSVLVMPTFHVPFTDLQVPSCKLDFREIQIYKKLRTSSFA

LNLPTLPEVKFPEVDVLTKYSQPEDSLIPFFEITVPESQLTVSQFTLPKSVSDGIAALDL

NAVANKIADFELPTIIVPEQTIEIPSIKFSVPAGIVIPSFQALTARFEVDSPVYNATWSA

SLKNKADYVETVLDSTCSSTVQFLEYELNVLGTHKIEDGTLASKTKGTLAHRDFSAEYEE

DGKFEGLQEWEGKAHLNIKSPAFTDLHLRYQKDKKGISTSAASPAVGTVGMDMDEDDDFS

KWNFYYSPQSSPDKKLTIFKTELRVRESDEETQIKVNWEEEAASGLLTSLKDNVPKATGV

LYDYVNKYHWEHTGLTLREVSSKLRRNLQNNAEWVYQGAIRQIDDIDVRFQKAASGTTGT

YQEWKDKAQNLYQELLTQEGQASFQGLKDNVFDGLVRVTQKFHMKVKHLIDSLIDFLNFP

RFQFPGKPGIYTREELCTMFIREVGTVLSQVYSKVHNGSEILFSYFQDLVITLPFELRKH

KLIDVISMYRELLKDLSKEAQEVFKAIQSLKTTEVLRNLQDLLQFIFQLIEDNIKQLKEM

KFTYLINYIQDEINTIFNDYIPYVFKLLKENLCLNLHKFNEFIQNELQEASQELQQIHQY

IMALREEYFDPSIVGWTVKYYELEEKIVSLIKNLLVALKDFHSEYIVSASNFTSQLSSQV

EQFLHRNIQEYLSILTDPDGKGKEKIAELSATAQEIIKSQAIATKKIISDYHQQFRYKLQ

DFSDQLSDYYEKFIAESKRLIDLSIQNYHTFLIYITELLKKLQSTTVMNPYMKLAPGELT

IIL

>sp|P04114|APOB_HUMAN 4022 WNFYYSPQSSPDKKL

MDPPRPALLALLALPALLLLLLAGARAEEEMLENVSLVCPKDATRFKHLRKYTYNYEAES

SSGVPGTADSRSATRINCKVELEVPQLCSFILKTSQCTLKEVYGFNPEGKALLKKTKNSE

EFAAAMSRYELKLAIPEGKQVFLYPEKDEPTYILNIKRGIISALLVPPETEEAKQVLFLD

TVYGNCSTHFTVKTRKGNVATEISTERDLGQCDRFKPIRTGISPLALIKGMTRPLSTLIS

SSQSCQYTLDAKRKHVAEAICKEQHLFLPFSYNNKYGMVAQVTQTLKLEDTPKINSRFFG

EGTKKMGLAFESTKSTSPPKQAEAVLKTLQELKKLTISEQNIQRANLFNKLVTELRGLSD

EAVTSLLPQLIEVSSPITLQALVQCGQPQCSTHILQWLKRVHANPLLIDVVTYLVALIPE

PSAQQLREIFNMARDQRSRATLYALSHAVNNYHKTNPTGTQELLDIANYLMEQIQDDCTG

DEDYTYLILRVIGNMGQTMEQLTPELKSSILKCVQSTKPSLMIQKAAIQALRKMEPKDKD

QEVLLQTFLDDASPGDKRLAAYLMLMRSPSQADINKIVQILPWEQNEQVKNFVASHIANI

LNSEELDIQDLKKLVKEALKESQLPTVMDFRKFSRNYQLYKSVSLPSLDPASAKIEGNLI

FDPNNYLPKESMLKTTLTAFGFASADLIEIGLEGKGFEPTLEALFGKQGFFPDSVNKALY

WVNGQVPDGVSKVLVDHFGYTKDDKHEQDMVNGIMLSVEKLIKDLKSKEVPEARAYLRIL

GEELGFASLHDLQLLGKLLLMGARTLQGIPQMIGEVIRKGSKNDFFLHYIFMENAFELPT

GAGLQLQISSSGVIAPGAKAGVKLEVANMQAELVAKPSVSVEFVTNMGIIIPDFARSGVQ

MNTNFFHESGLEAHVALKAGKLKFIIPSPKRPVKLLSGGNTLHLVSTTKTEVIPPLIENR

QSWSVCKQVFPGLNYCTSGAYSNASSTDSASYYPLTGDTRLELELRPTGEIEQYSVSATY

ELQREDRALVDTLKFVTQAEGAKQTEATMTFKYNRQSMTLSSEVQIPDFDVDLGTILRVN

DESTEGKTSYRLTLDIQNKKITEVALMGHLSCDTKEERKIKGVISIPRLQAEARSEILAH

WSPAKLLLQMDSSATAYGSTVSKRVAWHYDEEKIEFEWNTGTNVDTKKMTSNFPVDLSDY

PKSLHMYANRLLDHRVPETDMTFRHVGSKLIVAMSSWLQKASGSLPYTQTLQDHLNSLKE

FNLQNMGLPDFHIPENLFLKSDGRVKYTLNKNSLKIEIPLPFGGKSSRDLKMLETVRTPA

LHFKSVGFHLPSREFQVPTFTIPKLYQLQVPLLGVLDLSTNVYSNLYNWSASYSGGNTST

DHFSLRARYHMKADSVVDLLSYNVQGSGETTYDHKNTFTLSCDGSLRHKFLDSNIKFSHV

EKLGNNPVSKGLLIFDASSSWGPQMSASVHLDSKKKQHLFVKEVKIDGQFRVSSFYAKGT

YGLSCQRDPNTGRLNGESNLRFNSSYLQGTNQITGRYEDGTLSLTSTSDLQSGIIKNTAS

LKYENYELTLKSDTNGKYKNFATSNKMDMTFSKQNALLRSEYQADYESLRFFSLLSGSLN

SHGLELNADILGTDKINSGAHKATLRIGQDGISTSATTNLKCSLLVLENELNAELGLSGA

SMKLTTNGRFREHNAKFSLDGKAALTELSLGSAYQAMILGVDSKNIFNFKVSQEGLKLSN

DMMGSYAEMKFDHTNSLNIAGLSLDFSSKLDNIYSSDKFYKQTVNLQLQPYSLVTTLNSD

LKYNALDLTNNGKLRLEPLKLHVAGNLKGAYQNNEIKHIYAISSAALSASYKADTVAKVQ

GVEFSHRLNTDIAGLASAIDMSTNYNSDSLHFSNVFRSVMAPFTMTIDAHTNGNGKLALW

GEHTGQLYSKFLLKAEPLAFTFSHDYKGSTSHHLVSRKSISAALEHKVSALLTPAEQTGT

WKLKTQFNNNEYSQDLDAYNTKDKIGVELTGRTLADLTLLDSPIKVPLLLSEPINIIDAL

EMRDAVEKPQEFTIVAFVKYDKNQDVHSINLPFFETLQEYFERNRQTIIVVVENVQRNLK

HINIDQFVRKYRAALGKLPQQANDYLNSFNWERQVSHAKEKLTALTKKYRITENDIQIAL

DDAKINFNEKLSQLQTYMIQFDQYIKDSYDLHDLKIAIANIIDEIIEKLKSLDEHYHIRV

NLVKTIHDLHLFIENIDFNKSGSSTASWIQNVDTKYQIRIQIQEKLQQLKRHIQNIDIQH

LAGKLKQHIEAIDVRVLLDQLGTTISFERINDVLEHVKHFVINLIGDFEVAEKINAFRAK

VHELIERYEVDQQIQVLMDKLVELTHQYKLKETIQKLSNVLQQVKIKDYFEKLVGFIDDA

VKKLNELSFKTFIEDVNKFLDMLIKKLKSFDYHQFVDETNDKIREVTQRLNGEIQALELP

QKAEALKLFLEETKATVAVYLESLQDTKITLIINWLQEALSSASLAHMKAKFRETLEDTR

DRMYQMDIQQELQRYLSLVGQVYSTLVTYISDWWTLAAKNLTDFAEQYSIQDWAKRMKAL

VEQGFTVPEIKTILGTMPAFEVSLQALQKATFQTPDFIVPLTDLRIPSVQINFKDLKNIK

IPSRFSTPEFTILNTFHIPSFTIDFVEMKVKIIRTIDQMQNSELQWPVPDIYLRDLKVED

IPLARITLPDFRLPEIAIPEFIIPTLNLNDFQVPDLHIPEFQLPHISHTIEVPTFGKLYS

ILKIQSPLFTLDANADIGNGTTSANEAGIAASITAKGESKLEVLNFDFQANAQLSNPKIN

PLALKESVKFSSKYLRTEHGSEMLFFGNAIEGKSNTVASLHTEKNTLELSNGVIVKINNQ

LTLDSNTKYFHKLNIPKLDFSSQADLRNEIKTLLKAGHIAWTSSGKGSWKWACPRFSDEG

THESQISFTIEGPLTSFGLSNKINSKHLRVNQNLVYESGSLNFSKLEIQSQVDSQHVGHS

VLTAKGMALFGEGKAEFTGRHDAHLNGKVIGTLKNSLFFSAQPFEITASTNNEGNLKVRF

PLRLTGKIDFLNNYALFLSPSAQQASWQVSARFNQYKYNQNFSAGNNENIMEAHVGINGE

ANLDFLNIPLTIPEMRLPYTIITTPPLKDFSLWEKTGLKEFLKTTKQSFDLSVKAQYKKN

KHRHSITNPLAVLCEFISQSIKSFDRHFEKNRNNALDFVTKSYNETKIKFDKYKAEKSHD

ELPRTFQIPGYTVPVVNVEVSPFTIEMSAFGYVFPKAVSMPSFSILGSDVRVPSYTLILP

SLELPVLHVPRNLKLSLPHFKELCTISHIFIPAMGNITYDFSFKSSVITLNTNAELFNQS

DIVAHLLSSSSSVIDALQYKLEGTTRLTRKRGLKLATALSLSNKFVEGSHNSTVSLTTKN

MEVSVAKTTKAEIPILRMNFKQELNGNTKSKPTVSSSMEFKYDFNSSMLYSTAKGAVDHK

LSLESLTSYFSIESSTKGDVKGSVLSREYSGTIASEANTYLNSKSTRSSVKLQGTSKIDD

IWNLEVKENFAGEATLQRIYSLWEHSTKNHLQLEGLFFTNGEHTSKATLELSPWQMSALV

QVHASQPSSFHDFPDLGQEVALNANTKNQKIRWKNEVRIHSGSFQSQVELSNDQEKAHLD

IAGSLEGHLRFLKNIILPVYDKSLWDFLKLDVTTSIGRRQHLRVSTAFVYTKNPNGYSFS

IPVKVLADKFITPGLKLNDLNSVLVMPTFHVPFTDLQVPSCKLDFREIQIYKKLRTSSFA

LNLPTLPEVKFPEVDVLTKYSQPEDSLIPFFEITVPESQLTVSQFTLPKSVSDGIAALDL

NAVANKIADFELPTIIVPEQTIEIPSIKFSVPAGIVIPSFQALTARFEVDSPVYNATWSA

SLKNKADYVETVLDSTCSSTVQFLEYELNVLGTHKIEDGTLASKTKGTLAHRDFSAEYEE

DGKFEGLQEWEGKAHLNIKSPAFTDLHLRYQKDKKGISTSAASPAVGTVGMDMDEDDDFS

KWNFYYSPQSSPDKKLTIFKTELRVRESDEETQIKVNWEEEAASGLLTSLKDNVPKATGV

LYDYVNKYHWEHTGLTLREVSSKLRRNLQNNAEWVYQGAIRQIDDIDVRFQKAASGTTGT

YQEWKDKAQNLYQELLTQEGQASFQGLKDNVFDGLVRVTQKFHMKVKHLIDSLIDFLNFP

RFQFPGKPGIYTREELCTMFIREVGTVLSQVYSKVHNGSEILFSYFQDLVITLPFELRKH

KLIDVISMYRELLKDLSKEAQEVFKAIQSLKTTEVLRNLQDLLQFIFQLIEDNIKQLKEM

KFTYLINYIQDEINTIFNDYIPYVFKLLKENLCLNLHKFNEFIQNELQEASQELQQIHQY

IMALREEYFDPSIVGWTVKYYELEEKIVSLIKNLLVALKDFHSEYIVSASNFTSQLSSQV

EQFLHRNIQEYLSILTDPDGKGKEKIAELSATAQEIIKSQAIATKKIISDYHQQFRYKLQ

DFSDQLSDYYEKFIAESKRLIDLSIQNYHTFLIYITELLKKLQSTTVMNPYMKLAPGELT

IIL

>sp|P04275|VWF_HUMAN 1380 IALLLMASQEPQRM

MIPARFAGVLLALALILPGTLCAEGTRGRSSTARCSLFGSDFVNTFDGSMYSFAGYCSYL

LAGGCQKRSFSIIGDFQNGKRVSLSVYLGEFFDIHLFVNGTVTQGDQRVSMPYASKGLYL

ETEAGYYKLSGEAYGFVARIDGSGNFQVLLSDRYFNKTCGLCGNFNIFAEDDFMTQEGTL

TSDPYDFANSWALSSGEQWCERASPPSSSCNISSGEMQKGLWEQCQLLKSTSVFARCHPL

VDPEPFVALCEKTLCECAGGLECACPALLEYARTCAQEGMVLYGWTDHSACSPVCPAGME

YRQCVSPCARTCQSLHINEMCQERCVDGCSCPEGQLLDEGLCVESTECPCVHSGKRYPPG

TSLSRDCNTCICRNSQWICSNEECPGECLVTGQSHFKSFDNRYFTFSGICQYLLARDCQD

HSFSIVIETVQCADDRDAVCTRSVTVRLPGLHNSLVKLKHGAGVAMDGQDIQLPLLKGDL

RIQHTVTASVRLSYGEDLQMDWDGRGRLLVKLSPVYAGKTCGLCGNYNGNQGDDFLTPSG

LAEPRVEDFGNAWKLHGDCQDLQKQHSDPCALNPRMTRFSEEACAVLTSPTFEACHRAVS

PLPYLRNCRYDVCSCSDGRECLCGALASYAAACAGRGVRVAWREPGRCELNCPKGQVYLQ

CGTPCNLTCRSLSYPDEECNEACLEGCFCPPGLYMDERGDCVPKAQCPCYYDGEIFQPED

IFSDHHTMCYCEDGFMHCTMSGVPGSLLPDAVLSSPLSHRSKRSLSCRPPMVKLVCPADN

LRAEGLECTKTCQNYDLECMSMGCVSGCLCPPGMVRHENRCVALERCPCFHQGKEYAPGE

TVKIGCNTCVCRDRKWNCTDHVCDATCSTIGMAHYLTFDGLKYLFPGECQYVLVQDYCGS

NPGTFRILVGNKGCSHPSVKCKKRVTILVEGGEIELFDGEVNVKRPMKDETHFEVVESGR

YIILLLGKALSVVWDRHLSISVVLKQTYQEKVCGLCGNFDGIQNNDLTSSNLQVEEDPVD

FGNSWKVSSQCADTRKVPLDSSPATCHNNIMKQTMVDSSCRILTSDVFQDCNKLVDPEPY

LDVCIYDTCSCESIGDCACFCDTIAAYAHVCAQHGKVVTWRTATLCPQSCEERNLRENGY

ECEWRYNSCAPACQVTCQHPEPLACPVQCVEGCHAHCPPGKILDELLQTCVDPEDCPVCE

VAGRRFASGKKVTLNPSDPEHCQICHCDVVNLTCEACQEPGGLVVPPTDAPVSPTTLYVE

DISEPPLHDFYCSRLLDLVFLLDGSSRLSEAEFEVLKAFVVDMMERLRISQKWVRVAVVE

YHDGSHAYIGLKDRKRPSELRRIASQVKYAGSQVASTSEVLKYTLFQIFSKIDRPEASRI

ALLLMASQEPQRMSRNFVRYVQGLKKKKVIVIPVGIGPHANLKQIRLIEKQAPENKAFVL

SSVDELEQQRDEIVSYLCDLAPEAPPPTLPPHMAQVTVGPGLLGVSTLGPKRNSMVLDVA

FVLEGSDKIGEADFNRSKEFMEEVIQRMDVGQDSIHVTVLQYSYMVTVEYPFSEAQSKGD

ILQRVREIRYQGGNRTNTGLALRYLSDHSFLVSQGDREQAPNLVYMVTGNPASDEIKRLP

GDIQVVPIGVGPNANVQELERIGWPNAPILIQDFETLPREAPDLVLQRCCSGEGLQIPTL

SPAPDCSQPLDVILLLDGSSSFPASYFDEMKSFAKAFISKANIGPRLTQVSVLQYGSITT

IDVPWNVVPEKAHLLSLVDVMQREGGPSQIGDALGFAVRYLTSEMHGARPGASKAVVILV

TDVSVDSVDAAADAARSNRVTVFPIGIGDRYDAAQLRILAGPAGDSNVVKLQRIEDLPTM

VTLGNSFLHKLCSGFVRICMDEDGNEKRPGDVWTLPDQCHTVTCQPDGQTLLKSHRVNCD

RGLRPSCPNSQSPVKVEETCGCRWTCPCVCTGSSTRHIVTFDGQNFKLTGSCSYVLFQNK

EQDLEVILHNGACSPGARQGCMKSIEVKHSALSVELHSDMEVTVNGRLVSVPYVGGNMEV

NVYGAIMHEVRFNHLGHIFTFTPQNNEFQLQLSPKTFASKTYGLCGICDENGANDFMLRD

GTVTTDWKTLVQEWTVQRPGQTCQPILEEQCLVPDSSHCQVLLLPLFAECHKVLAPATFY

AICQQDSCHQEQVCEVIASYAHLCRTNGVCVDWRTPDFCAMSCPPSLVYNHCEHGCPRHC

DGNVSSCGDHPSEGCFCPPDKVMLEGSCVPEEACTQCIGEDGVQHQFLEAWVPDHQPCQI

CTCLSGRKVNCTTQPCPTAKAPTCGLCEVARLRQNADQCCPEYECVCDPVSCDLPPVPHC

ERGLQPTLTNPGECRPNFTCACRKEECKRVSPPSCPPHRLPTLRKTQCCDEYECACNCVN

STVSCPLGYLASTATNDCGCTTTTCLPDKVCVHRSTIYPVGQFWEEGCDVCTCTDMEDAV

MGLRVAQCSQKPCEDSCRSGFTYVLHEGECCGRCLPSACEVVTGSPRGDSQSSWKSVGSQ

WASPENPCLINECVRVKEEVFIQQRNVSCPQLEVPVCPSGFQLSCKTSACCPSCRCERME

ACMLNGTVIGPGKTVMIDVCTTCRCMVQVGVISGFKLECRKTTCNPCPLGYKEENNTGEC

CGRCLPTACTIQLRGGQIMTLKRDETLQDGCDTHFCKVNERGEYFWEKRVTGCPPFDEHK

CLAEGGKIMKIPGTCCDTCEEPECNDITARLQYVKVGSCKSEVEVDIHYCQGKCASKAMY

SIDINDVQDQCSCCSPTRTEPMQVALHCTNGSVVYHEVLNAMECKCSPRKCSK

>sp|P04275|VWF_HUMAN 1380 IALLLMASQEPQRMSRNFVR

MIPARFAGVLLALALILPGTLCAEGTRGRSSTARCSLFGSDFVNTFDGSMYSFAGYCSYL

LAGGCQKRSFSIIGDFQNGKRVSLSVYLGEFFDIHLFVNGTVTQGDQRVSMPYASKGLYL

ETEAGYYKLSGEAYGFVARIDGSGNFQVLLSDRYFNKTCGLCGNFNIFAEDDFMTQEGTL

TSDPYDFANSWALSSGEQWCERASPPSSSCNISSGEMQKGLWEQCQLLKSTSVFARCHPL

VDPEPFVALCEKTLCECAGGLECACPALLEYARTCAQEGMVLYGWTDHSACSPVCPAGME

YRQCVSPCARTCQSLHINEMCQERCVDGCSCPEGQLLDEGLCVESTECPCVHSGKRYPPG

TSLSRDCNTCICRNSQWICSNEECPGECLVTGQSHFKSFDNRYFTFSGICQYLLARDCQD

HSFSIVIETVQCADDRDAVCTRSVTVRLPGLHNSLVKLKHGAGVAMDGQDIQLPLLKGDL

RIQHTVTASVRLSYGEDLQMDWDGRGRLLVKLSPVYAGKTCGLCGNYNGNQGDDFLTPSG

LAEPRVEDFGNAWKLHGDCQDLQKQHSDPCALNPRMTRFSEEACAVLTSPTFEACHRAVS

PLPYLRNCRYDVCSCSDGRECLCGALASYAAACAGRGVRVAWREPGRCELNCPKGQVYLQ

CGTPCNLTCRSLSYPDEECNEACLEGCFCPPGLYMDERGDCVPKAQCPCYYDGEIFQPED

IFSDHHTMCYCEDGFMHCTMSGVPGSLLPDAVLSSPLSHRSKRSLSCRPPMVKLVCPADN

LRAEGLECTKTCQNYDLECMSMGCVSGCLCPPGMVRHENRCVALERCPCFHQGKEYAPGE

TVKIGCNTCVCRDRKWNCTDHVCDATCSTIGMAHYLTFDGLKYLFPGECQYVLVQDYCGS

NPGTFRILVGNKGCSHPSVKCKKRVTILVEGGEIELFDGEVNVKRPMKDETHFEVVESGR

YIILLLGKALSVVWDRHLSISVVLKQTYQEKVCGLCGNFDGIQNNDLTSSNLQVEEDPVD

FGNSWKVSSQCADTRKVPLDSSPATCHNNIMKQTMVDSSCRILTSDVFQDCNKLVDPEPY

LDVCIYDTCSCESIGDCACFCDTIAAYAHVCAQHGKVVTWRTATLCPQSCEERNLRENGY

ECEWRYNSCAPACQVTCQHPEPLACPVQCVEGCHAHCPPGKILDELLQTCVDPEDCPVCE

VAGRRFASGKKVTLNPSDPEHCQICHCDVVNLTCEACQEPGGLVVPPTDAPVSPTTLYVE

DISEPPLHDFYCSRLLDLVFLLDGSSRLSEAEFEVLKAFVVDMMERLRISQKWVRVAVVE

YHDGSHAYIGLKDRKRPSELRRIASQVKYAGSQVASTSEVLKYTLFQIFSKIDRPEASRI

ALLLMASQEPQRMSRNFVRYVQGLKKKKVIVIPVGIGPHANLKQIRLIEKQAPENKAFVL

SSVDELEQQRDEIVSYLCDLAPEAPPPTLPPHMAQVTVGPGLLGVSTLGPKRNSMVLDVA

FVLEGSDKIGEADFNRSKEFMEEVIQRMDVGQDSIHVTVLQYSYMVTVEYPFSEAQSKGD

ILQRVREIRYQGGNRTNTGLALRYLSDHSFLVSQGDREQAPNLVYMVTGNPASDEIKRLP

GDIQVVPIGVGPNANVQELERIGWPNAPILIQDFETLPREAPDLVLQRCCSGEGLQIPTL

SPAPDCSQPLDVILLLDGSSSFPASYFDEMKSFAKAFISKANIGPRLTQVSVLQYGSITT

IDVPWNVVPEKAHLLSLVDVMQREGGPSQIGDALGFAVRYLTSEMHGARPGASKAVVILV

TDVSVDSVDAAADAARSNRVTVFPIGIGDRYDAAQLRILAGPAGDSNVVKLQRIEDLPTM

VTLGNSFLHKLCSGFVRICMDEDGNEKRPGDVWTLPDQCHTVTCQPDGQTLLKSHRVNCD

RGLRPSCPNSQSPVKVEETCGCRWTCPCVCTGSSTRHIVTFDGQNFKLTGSCSYVLFQNK

EQDLEVILHNGACSPGARQGCMKSIEVKHSALSVELHSDMEVTVNGRLVSVPYVGGNMEV

NVYGAIMHEVRFNHLGHIFTFTPQNNEFQLQLSPKTFASKTYGLCGICDENGANDFMLRD

GTVTTDWKTLVQEWTVQRPGQTCQPILEEQCLVPDSSHCQVLLLPLFAECHKVLAPATFY

AICQQDSCHQEQVCEVIASYAHLCRTNGVCVDWRTPDFCAMSCPPSLVYNHCEHGCPRHC

DGNVSSCGDHPSEGCFCPPDKVMLEGSCVPEEACTQCIGEDGVQHQFLEAWVPDHQPCQI

CTCLSGRKVNCTTQPCPTAKAPTCGLCEVARLRQNADQCCPEYECVCDPVSCDLPPVPHC

ERGLQPTLTNPGECRPNFTCACRKEECKRVSPPSCPPHRLPTLRKTQCCDEYECACNCVN

STVSCPLGYLASTATNDCGCTTTTCLPDKVCVHRSTIYPVGQFWEEGCDVCTCTDMEDAV

MGLRVAQCSQKPCEDSCRSGFTYVLHEGECCGRCLPSACEVVTGSPRGDSQSSWKSVGSQ

WASPENPCLINECVRVKEEVFIQQRNVSCPQLEVPVCPSGFQLSCKTSACCPSCRCERME

ACMLNGTVIGPGKTVMIDVCTTCRCMVQVGVISGFKLECRKTTCNPCPLGYKEENNTGEC

CGRCLPTACTIQLRGGQIMTLKRDETLQDGCDTHFCKVNERGEYFWEKRVTGCPPFDEHK

CLAEGGKIMKIPGTCCDTCEEPECNDITARLQYVKVGSCKSEVEVDIHYCQGKCASKAMY

SIDINDVQDQCSCCSPTRTEPMQVALHCTNGSVVYHEVLNAMECKCSPRKCSK

>sp|P11717|MPRI_HUMAN 185 DLNPLIKLSGAYLVDDSD

MGAAAGRSPHLGPAPARRPQRSLLLLQLLLLVAAPGSTQAQAAPFPELCSYTWEAVDTKN

NVLYKINICGSVDIVQCGPSSAVCMHDLKTRTYHSVGDSVLRSATRSLLEFNTTVSCDQQ

GTNHRVQSSIAFLCGKTLGTPEFVTATECVHYFEWRTTAACKKDIFKANKEVPCYVFDEE

LRKHDLNPLIKLSGAYLVDDSDPDTSLFINVCRDIDTLRDPGSQLRACPPGTAACLVRGH

QAFDVGQPRDGLKLVRKDRLVLSYVREEAGKLDFCDGHSPAVTITFVCPSERREGTIPKL

TAKSNCRYEIEWITEYACHRDYLESKTCSLSGEQQDVSIDLTPLAQSGGSSYISDGKEYL

FYLNVCGETEIQFCNKKQAAVCQVKKSDTSQVKAAGRYHNQTLRYSDGDLTLIYFGGDEC

SSGFQRMSVINFECNKTAGNDGKGTPVFTGEVDCTYFFTWDTEYACVKEKEDLLCGATDG

KKRYDLSALVRHAEPEQNWEAVDGSQTETEKKHFFINICHRVLQEGKARGCPEDAAVCAV

DKNGSKNLGKFISSPMKEKGNIQLSYSDGDDCGHGKKIKTNITLVCKPGDLESAPVLRTS

GEGGCFYEFEWHTAAACVLSKTEGENCTVFDSQAGFSFDLSPLTKKNGAYKVETKKYDFY

INVCGPVSVSPCQPDSGACQVAKSDEKTWNLGLSNAKLSYYDGMIQLNYRGGTPYNNERH

TPRATLITFLCDRDAGVGFPEYQEEDNSTYNFRWYTSYACPEEPLECVVTDPSTLEQYDL

SSLAKSEGGLGGNWYAMDNSGEHVTWRKYYINVCRPLNPVPGCNRYASACQMKYEKDQGS

FTEVVSISNLGMAKTGPVVEDSGSLLLEYVNGSACTTSDGRQTTYTTRIHLVCSRGRLNS

HPIFSLNWECVVSFLWNTEAACPIQTTTDTDQACSIRDPNSGFVFNLNPLNSSQGYNVSG

IGKIFMFNVCGTMPVCGTILGKPASGCEAETQTEELKNWKPARPVGIEKSLQLSTEGFIT

LTYKGPLSAKGTADAFIVRFVCNDDVYSGPLKFLHQDIDSGQGIRNTYFEFETALACVPS

PVDCQVTDLAGNEYDLTGLSTVRKPWTAVDTSVDGRKRTFYLSVCNPLPYIPGCQGSAVG

SCLVSEGNSWNLGVVQMSPQAAANGSLSIMYVNGDKCGNQRFSTRITFECAQISGSPAFQ

LQDGCEYVFIWRTVEACPVVRVEGDNCEVKDPRHGNLYDLKPLGLNDTIVSAGEYTYYFR

VCGKLSSDVCPTSDKSKVVSSCQEKREPQGFHKVAGLLTQKLTYENGLLKMNFTGGDTCH

KVYQRSTAIFFYCDRGTQRPVFLKETSDCSYLFEWRTQYACPPFDLTECSFKDGAGNSFD

LSSLSRYSDNWEAITGTGDPEHYLINVCKSLAPQAGTEPCPPEAAACLLGGSKPVNLGRV

RDGPQWRDGIIVLKYVDGDLCPDGIRKKSTTIRFTCSESQVNSRPMFISAVEDCEYTFAW

PTATACPMKSNEHDDCQVTNPSTGHLFDLSSLSGRAGFTAAYSEKGLVYMSICGENENCP

PGVGACFGQTRISVGKANKRLRYVDQVLQLVYKDGSPCPSKSGLSYKSVISFVCRPEAGP

TNRPMLISLDKQTCTLFFSWHTPLACEQATECSVRNGSSIVDLSPLIHRTGGYEAYDESE

DDASDTNPDFYINICQPLNPMHGVPCPAGAAVCKVPIDGPPIDIGRVAGPPILNPIANEI

YLNFESSTPCLADKHFNYTSLIAFHCKRGVSMGTPKLLRTSECDFVFEWETPVVCPDEVR

MDGCTLTDEQLLYSFNLSSLSTSTFKVTRDSRTYSVGVCTFAVGPEQGGCKDGGVCLLSG

TKGASFGRLQSMKLDYRHQDEAVVLSYVNGDRCPPETDDGVPCVFPFIFNGKSYEECIIE

SRAKLWCSTTADYDRDHEWGFCRHSNSYRTSSIIFKCDEDEDIGRPQVFSEVRGCDVTFE

WKTKVVCPPKKLECKFVQKHKTYDLRLLSSLTGSWSLVHNGVSYYINLCQKIYKGPLGCS

ERASICRRTTTGDVQVLGLVHTQKLGVIGDKVVVTYSKGYPCGGNKTASSVIELTCTKTV

GRPAFKRFDIDSCTYYFSWDSRAACAVKPQEVQMVNGTITNPINGKSFSLGDIYFKLFRA

SGDMRTNGDNYLYEIQLSSITSSRNPACSGANICQVKPNDQHFSRKVGTSDKTKYYLQDG

DLDVVFASSSKCGKDKTKSVSSTIFFHCDPLVEDGIPEFSHETADCQYLFSWYTSAVCPL

GVGFDSENPGDDGQMHKGLSERSQAVGAVLSLLLVALTCCLLALLLYKKERRETVISKLT

TCCRRSSNVSYKYSKVNKEEETDENETEWLMEEIQLPPPRQGKEGQENGHITTKSVKALS

SLHGDDQDSEDEVLTIPEVKVHSGRGAGAESSHPVRNAQSNALQEREDDRVGLVRGEKAR

KGKSSSAQQKTVSSTKLVSFHDDSDEDLLHI

>sp|P11717|MPRI_HUMAN 726 LITFLCDRDAGVGFPE

MGAAAGRSPHLGPAPARRPQRSLLLLQLLLLVAAPGSTQAQAAPFPELCSYTWEAVDTKN

NVLYKINICGSVDIVQCGPSSAVCMHDLKTRTYHSVGDSVLRSATRSLLEFNTTVSCDQQ

GTNHRVQSSIAFLCGKTLGTPEFVTATECVHYFEWRTTAACKKDIFKANKEVPCYVFDEE

LRKHDLNPLIKLSGAYLVDDSDPDTSLFINVCRDIDTLRDPGSQLRACPPGTAACLVRGH

QAFDVGQPRDGLKLVRKDRLVLSYVREEAGKLDFCDGHSPAVTITFVCPSERREGTIPKL

TAKSNCRYEIEWITEYACHRDYLESKTCSLSGEQQDVSIDLTPLAQSGGSSYISDGKEYL

FYLNVCGETEIQFCNKKQAAVCQVKKSDTSQVKAAGRYHNQTLRYSDGDLTLIYFGGDEC

SSGFQRMSVINFECNKTAGNDGKGTPVFTGEVDCTYFFTWDTEYACVKEKEDLLCGATDG

KKRYDLSALVRHAEPEQNWEAVDGSQTETEKKHFFINICHRVLQEGKARGCPEDAAVCAV

DKNGSKNLGKFISSPMKEKGNIQLSYSDGDDCGHGKKIKTNITLVCKPGDLESAPVLRTS

GEGGCFYEFEWHTAAACVLSKTEGENCTVFDSQAGFSFDLSPLTKKNGAYKVETKKYDFY

INVCGPVSVSPCQPDSGACQVAKSDEKTWNLGLSNAKLSYYDGMIQLNYRGGTPYNNERH

TPRATLITFLCDRDAGVGFPEYQEEDNSTYNFRWYTSYACPEEPLECVVTDPSTLEQYDL

SSLAKSEGGLGGNWYAMDNSGEHVTWRKYYINVCRPLNPVPGCNRYASACQMKYEKDQGS

FTEVVSISNLGMAKTGPVVEDSGSLLLEYVNGSACTTSDGRQTTYTTRIHLVCSRGRLNS

HPIFSLNWECVVSFLWNTEAACPIQTTTDTDQACSIRDPNSGFVFNLNPLNSSQGYNVSG

IGKIFMFNVCGTMPVCGTILGKPASGCEAETQTEELKNWKPARPVGIEKSLQLSTEGFIT

LTYKGPLSAKGTADAFIVRFVCNDDVYSGPLKFLHQDIDSGQGIRNTYFEFETALACVPS

PVDCQVTDLAGNEYDLTGLSTVRKPWTAVDTSVDGRKRTFYLSVCNPLPYIPGCQGSAVG

SCLVSEGNSWNLGVVQMSPQAAANGSLSIMYVNGDKCGNQRFSTRITFECAQISGSPAFQ

LQDGCEYVFIWRTVEACPVVRVEGDNCEVKDPRHGNLYDLKPLGLNDTIVSAGEYTYYFR

VCGKLSSDVCPTSDKSKVVSSCQEKREPQGFHKVAGLLTQKLTYENGLLKMNFTGGDTCH

KVYQRSTAIFFYCDRGTQRPVFLKETSDCSYLFEWRTQYACPPFDLTECSFKDGAGNSFD

LSSLSRYSDNWEAITGTGDPEHYLINVCKSLAPQAGTEPCPPEAAACLLGGSKPVNLGRV

RDGPQWRDGIIVLKYVDGDLCPDGIRKKSTTIRFTCSESQVNSRPMFISAVEDCEYTFAW

PTATACPMKSNEHDDCQVTNPSTGHLFDLSSLSGRAGFTAAYSEKGLVYMSICGENENCP

PGVGACFGQTRISVGKANKRLRYVDQVLQLVYKDGSPCPSKSGLSYKSVISFVCRPEAGP

TNRPMLISLDKQTCTLFFSWHTPLACEQATECSVRNGSSIVDLSPLIHRTGGYEAYDESE

DDASDTNPDFYINICQPLNPMHGVPCPAGAAVCKVPIDGPPIDIGRVAGPPILNPIANEI

YLNFESSTPCLADKHFNYTSLIAFHCKRGVSMGTPKLLRTSECDFVFEWETPVVCPDEVR

MDGCTLTDEQLLYSFNLSSLSTSTFKVTRDSRTYSVGVCTFAVGPEQGGCKDGGVCLLSG

TKGASFGRLQSMKLDYRHQDEAVVLSYVNGDRCPPETDDGVPCVFPFIFNGKSYEECIIE

SRAKLWCSTTADYDRDHEWGFCRHSNSYRTSSIIFKCDEDEDIGRPQVFSEVRGCDVTFE

WKTKVVCPPKKLECKFVQKHKTYDLRLLSSLTGSWSLVHNGVSYYINLCQKIYKGPLGCS

ERASICRRTTTGDVQVLGLVHTQKLGVIGDKVVVTYSKGYPCGGNKTASSVIELTCTKTV

GRPAFKRFDIDSCTYYFSWDSRAACAVKPQEVQMVNGTITNPINGKSFSLGDIYFKLFRA

SGDMRTNGDNYLYEIQLSSITSSRNPACSGANICQVKPNDQHFSRKVGTSDKTKYYLQDG

DLDVVFASSSKCGKDKTKSVSSTIFFHCDPLVEDGIPEFSHETADCQYLFSWYTSAVCPL

GVGFDSENPGDDGQMHKGLSERSQAVGAVLSLLLVALTCCLLALLLYKKERRETVISKLT

TCCRRSSNVSYKYSKVNKEEETDENETEWLMEEIQLPPPRQGKEGQENGHITTKSVKALS

SLHGDDQDSEDEVLTIPEVKVHSGRGAGAESSHPVRNAQSNALQEREDDRVGLVRGEKAR

KGKSSSAQQKTVSSTKLVSFHDDSDEDLLHI

>sp|P00451|FA8_HUMAN 1725 LWDYGMSSSPHVLRNR

MQIELSTCFFLCLLRFCFSATRRYYLGAVELSWDYMQSDLGELPVDARFPPRVPKSFPFN

TSVVYKKTLFVEFTDHLFNIAKPRPPWMGLLGPTIQAEVYDTVVITLKNMASHPVSLHAV

GVSYWKASEGAEYDDQTSQREKEDDKVFPGGSHTYVWQVLKENGPMASDPLCLTYSYLSH

VDLVKDLNSGLIGALLVCREGSLAKEKTQTLHKFILLFAVFDEGKSWHSETKNSLMQDRD

AASARAWPKMHTVNGYVNRSLPGLIGCHRKSVYWHVIGMGTTPEVHSIFLEGHTFLVRNH

RQASLEISPITFLTAQTLLMDLGQFLLFCHISSHQHDGMEAYVKVDSCPEEPQLRMKNNE

EAEDYDDDLTDSEMDVVRFDDDNSPSFIQIRSVAKKHPKTWVHYIAAEEEDWDYAPLVLA

PDDRSYKSQYLNNGPQRIGRKYKKVRFMAYTDETFKTREAIQHESGILGPLLYGEVGDTL

LIIFKNQASRPYNIYPHGITDVRPLYSRRLPKGVKHLKDFPILPGEIFKYKWTVTVEDGP

TKSDPRCLTRYYSSFVNMERDLASGLIGPLLICYKESVDQRGNQIMSDKRNVILFSVFDE

NRSWYLTENIQRFLPNPAGVQLEDPEFQASNIMHSINGYVFDSLQLSVCLHEVAYWYILS

IGAQTDFLSVFFSGYTFKHKMVYEDTLTLFPFSGETVFMSMENPGLWILGCHNSDFRNRG

MTALLKVSSCDKNTGDYYEDSYEDISAYLLSKNNAIEPRSFSQNSRHPSTRQKQFNATTI

PENDIEKTDPWFAHRTPMPKIQNVSSSDLLMLLRQSPTPHGLSLSDLQEAKYETFSDDPS

PGAIDSNNSLSEMTHFRPQLHHSGDMVFTPESGLQLRLNEKLGTTAATELKKLDFKVSST

SNNLISTIPSDNLAAGTDNTSSLGPPSMPVHYDSQLDTTLFGKKSSPLTESGGPLSLSEE

NNDSKLLESGLMNSQESSWGKNVSSTESGRLFKGKRAHGPALLTKDNALFKVSISLLKTN

KTSNNSATNRKTHIDGPSLLIENSPSVWQNILESDTEFKKVTPLIHDRMLMDKNATALRL

NHMSNKTTSSKNMEMVQQKKEGPIPPDAQNPDMSFFKMLFLPESARWIQRTHGKNSLNSG

QGPSPKQLVSLGPEKSVEGQNFLSEKNKVVVGKGEFTKDVGLKEMVFPSSRNLFLTNLDN

LHENNTHNQEKKIQEEIEKKETLIQENVVLPQIHTVTGTKNFMKNLFLLSTRQNVEGSYD

GAYAPVLQDFRSLNDSTNRTKKHTAHFSKKGEEENLEGLGNQTKQIVEKYACTTRISPNT

SQQNFVTQRSKRALKQFRLPLEETELEKRIIVDDTSTQWSKNMKHLTPSTLTQIDYNEKE

KGAITQSPLSDCLTRSHSIPQANRSPLPIAKVSSFPSIRPIYLTRVLFQDNSSHLPAASY

RKKDSGVQESSHFLQGAKKNNLSLAILTLEMTGDQREVGSLGTSATNSVTYKKVENTVLP

KPDLPKTSGKVELLPKVHIYQKDLFPTETSNGSPGHLDLVEGSLLQGTEGAIKWNEANRP

GKVPFLRVATESSAKTPSKLLDPLAWDNHYGTQIPKEEWKSQEKSPEKTAFKKKDTILSL

NACESNHAIAAINEGQNKPEIEVTWAKQGRTERLCSQNPPVLKRHQREITRTTLQSDQEE

IDYDDTISVEMKKEDFDIYDEDENQSPRSFQKKTRHYFIAAVERLWDYGMSSSPHVLRNR

AQSGSVPQFKKVVFQEFTDGSFTQPLYRGELNEHLGLLGPYIRAEVEDNIMVTFRNQASR

PYSFYSSLISYEEDQRQGAEPRKNFVKPNETKTYFWKVQHHMAPTKDEFDCKAWAYFSDV

DLEKDVHSGLIGPLLVCHTNTLNPAHGRQVTVQEFALFFTIFDETKSWYFTENMERNCRA

PCNIQMEDPTFKENYRFHAINGYIMDTLPGLVMAQDQRIRWYLLSMGSNENIHSIHFSGH

VFTVRKKEEYKMALYNLYPGVFETVEMLPSKAGIWRVECLIGEHLHAGMSTLFLVYSNKC

QTPLGMASGHIRDFQITASGQYGQWAPKLARLHYSGSINAWSTKEPFSWIKVDLLAPMII

HGIKTQGARQKFSSLYISQFIIMYSLDGKKWQTYRGNSTGTLMVFFGNVDSSGIKHNIFN

PPIIARYIRLHPTHYSIRSTLRMELMGCDLNSCSMPLGMESKAISDAQITASSYFTNMFA

TWSPSKARLHLQGRSNAWRPQVNNPKEWLQVDFQKTMKVTGVTTQGVKSLLTSMYVKEFL

ISSSQDGHQWTLFFQNGKVKVFQGNQDSFTPVVNSLDPPLLTRYLRIHPQSWVHQIALRM

EVLGCEAQDLY

>sp|P12259|FA5_HUMAN 1613 TTYKKVVFRKYLDSTFTK

MFPGCPRLWVLVVLGTSWVGWGSQGTEAAQLRQFYVAAQGISWSYRPEPTNSSLNLSVTS

FKKIVYREYEPYFKKEKPQSTISGLLGPTLYAEVGDIIKVHFKNKADKPLSIHPQGIRYS

KLSEGASYLDHTFPAEKMDDAVAPGREYTYEWSISEDSGPTHDDPPCLTHIYYSHENLIE

DFNSGLIGPLLICKKGTLTEGGTQKTFDKQIVLLFAVFDESKSWSQSSSLMYTVNGYVNG

TMPDITVCAHDHISWHLLGMSSGPELFSIHFNGQVLEQNHHKVSAITLVSATSTTANMTV

GPEGKWIISSLTPKHLQAGMQAYIDIKNCPKKTRNLKKITREQRRHMKRWEYFIAAEEVI

WDYAPVIPANMDKKYRSQHLDNFSNQIGKHYKKVMYTQYEDESFTKHTVNPNMKEDGILG

PIIRAQVRDTLKIVFKNMASRPYSIYPHGVTFSPYEDEVNSSFTSGRNNTMIRAVQPGET

YTYKWNILEFDEPTENDAQCLTRPYYSDVDIMRDIASGLIGLLLICKSRSLDRRGIQRAA

DIEQQAVFAVFDENKSWYLEDNINKFCENPDEVKRDDPKFYESNIMSTINGYVPESITTL

GFCFDDTVQWHFCSVGTQNEILTIHFTGHSFIYGKRHEDTLTLFPMRGESVTVTMDNVGT

WMLTSMNSSPRSKKLRLKFRDVKCIPDDDEDSYEIFEPPESTVMATRKMHDRLEPEDEES

DADYDYQNRLAAALGIRSFRNSSLNQEEEEFNLTALALENGTEFVSSNTDIIVGSNYSSP

SNISKFTVNNLAEPQKAPSHQQATTAGSPLRHLIGKNSVLNSSTAEHSSPYSEDPIEDPL

QPDVTGIRLLSLGAGEFKSQEHAKHKGPKVERDQAAKHRFSWMKLLAHKVGRHLSQDTGS

PSGMRPWEDLPSQDTGSPSRMRPWKDPPSDLLLLKQSNSSKILVGRWHLASEKGSYEIIQ

DTDEDTAVNNWLISPQNASRAWGESTPLANKPGKQSGHPKFPRVRHKSLQVRQDGGKSRL

KKSQFLIKTRKKKKEKHTHHAPLSPRTFHPLRSEAYNTFSERRLKHSLVLHKSNETSLPT

DLNQTLPSMDFGWIASLPDHNQNSSNDTGQASCPPGLYQTVPPEEHYQTFPIQDPDQMHS

TSDPSHRSSSPELSEMLEYDRSHKSFPTDISQMSPSSEHEVWQTVISPDLSQVTLSPELS

QTNLSPDLSHTTLSPELIQRNLSPALGQMPISPDLSHTTLSPDLSHTTLSLDLSQTNLSP

ELSQTNLSPALGQMPLSPDLSHTTLSLDFSQTNLSPELSHMTLSPELSQTNLSPALGQMP

ISPDLSHTTLSLDFSQTNLSPELSQTNLSPALGQMPLSPDPSHTTLSLDLSQTNLSPELS

QTNLSPDLSEMPLFADLSQIPLTPDLDQMTLSPDLGETDLSPNFGQMSLSPDLSQVTLSP

DISDTTLLPDLSQISPPPDLDQIFYPSESSQSLLLQEFNESFPYPDLGQMPSPSSPTLND

TFLSKEFNPLVIVGLSKDGTDYIEIIPKEEVQSSEDDYAEIDYVPYDDPYKTDVRTNINS

SRDPDNIAAWYLRSNNGNRRNYYIAAEEISWDYSEFVQRETDIEDSDDIPEDTTYKKVVF

RKYLDSTFTKRDPRGEYEEHLGILGPIIRAEVDDVIQVRFKNLASRPYSLHAHGLSYEKS

SEGKTYEDDSPEWFKEDNAVQPNSSYTYVWHATERSGPESPGSACRAWAYYSAVNPEKDI

HSGLIGPLLICQKGILHKDSNMPVDMREFVLLFMTFDEKKSWYYEKKSRSSWRLTSSEMK

KSHEFHAINGMIYSLPGLKMYEQEWVRLHLLNIGGSQDIHVVHFHGQTLLENGNKQHQLG

VWPLLPGSFKTLEMKASKPGWWLLNTEVGENQRAGMQTPFLIMDRDCRMPMGLSTGIISD

SQIKASEFLGYWEPRLARLNNGGSYNAWSVEKLAAEFASKPWIQVDMQKEVIITGIQTQG

AKHYLKSCYTTEFYVAYSSNQINWQIFKGNSTRNVMYFNGNSDASTIKENQFDPPIVARY

IRISPTRAYNRPTLRLELQGCEVNGCSTPLGMENGKIENKQITASSFKKSWWGDYWEPFR

ARLNAQGRVNAWQAKANNNKQWLEIDLLKIKKITAIITQGCKSLSSEMYVKSYTIHYSEQ

GVEWKPYRLKSSMVDKIFEGNTNTKGHVKNFFNPPIISRFIRVIPKTWNQSIALRLELFG

CDIY

>sp|Q92673|SORL_HUMAN 899 KPGIYRSNMDGSAAY

MATRSSRRESRLPFLFTLVALLPPGALCEVWTQRLHGGSAPLPQDRGFLVVQGDPRELRL

WARGDARGASRADEKPLRRKRSAALQPEPIKVYGQVSLNDSHNQMVVHWAGEKSNVIVAL

ARDSLALARPKSSDVYVSYDYGKSFKKISDKLNFGLGNRSEAVIAQFYHSPADNKRYIFA

DAYAQYLWITFDFCNTLQGFSIPFRAADLLLHSKASNLLLGFDRSHPNKQLWKSDDFGQT

WIMIQEHVKSFSWGIDPYDKPNTIYIERHEPSGYSTVFRSTDFFQSRENQEVILEEVRDF

QLRDKYMFATKVVHLLGSEQQSSVQLWVSFGRKPMRAAQFVTRHPINEYYIADASEDQVF

VCVSHSNNRTNLYISEAEGLKFSLSLENVLYYSPGGAGSDTLVRYFANEPFADFHRVEGL

QGVYIATLINGSMNEENMRSVITFDKGGTWEFLQAPAFTGYGEKINCELSQGCSLHLAQR

LSQLLNLQLRRMPILSKESAPGLIIATGSVGKNLASKTNVYISSSAGARWREALPGPHYY

TWGDHGGIITAIAQGMETNELKYSTNEGETWKTFIFSEKPVFVYGLLTEPGEKSTVFTIF

GSNKENVHSWLILQVNATDALGVPCTENDYKLWSPSDERGNECLLGHKTVFKRRTPHATC

FNGEDFDRPVVVSNCSCTREDYECDFGFKMSEDLSLEVCVPDPEFSGKSYSPPVPCPVGS

TYRRTRGYRKISGDTCSGGDVEARLEGELVPCPLAEENEFILYAVRKSIYRYDLASGATE

QLPLTGLRAAVALDFDYEHNCLYWSDLALDVIQRLCLNGSTGQEVIINSGLETVEALAFE

PLSQLLYWVDAGFKKIEVANPDGDFRLTIVNSSVLDRPRALVLVPQEGVMFWTDWGDLKP

GIYRSNMDGSAAYHLVSEDVKWPNGISVDDQWIYWTDAYLECIERITFSGQQRSVILDNL

PHPYAIAVFKNEIYWDDWSQLSIFRASKYSGSQMEILANQLTGLMDMKIFYKGKNTGSNA

CVPRPCSLLCLPKANNSRSCRCPEDVSSSVLPSGDLMCDCPQGYQLKNNTCVKEENTCLR

NQYRCSNGNCINSIWWCDFDNDCGDMSDERNCPTTICDLDTQFRCQESGTCIPLSYKCDL

EDDCGDNSDESHCEMHQCRSDEYNCSSGMCIRSSWVCDGDNDCRDWSDEANCTAIYHTCE

ASNFQCRNGHCIPQRWACDGDTDCQDGSDEDPVNCEKKCNGFRCPNGTCIPSSKHCDGLR

DCSDGSDEQHCEPLCTHFMDFVCKNRQQCLFHSMVCDGIIQCRDGSDEDAAFAGCSQDPE

FHKVCDEFGFQCQNGVCISLIWKCDGMDDCGDYSDEANCENPTEAPNCSRYFQFRCENGH

CIPNRWKCDRENDCGDWSDEKDCGDSHILPFSTPGPSTCLPNYYRCSSGTCVMDTWVCDG

YRDCADGSDEEACPLLANVTAASTPTQLGRCDRFEFECHQPKTCIPNWKRCDGHQDCQDG

RDEANCPTHSTLTCMSREFQCEDGEACIVLSERCDGFLDCSDESDEKACSDELTVYKVQN

LQWTADFSGDVTLTWMRPKKMPSASCVYNVYYRVVGESIWKTLETHSNKTNTVLKVLKPD

TTYQVKVQVQCLSKAHNTNDFVTLRTPEGLPDAPRNLQLSLPREAEGVIVGHWAPPIHTH

GLIREYIVEYSRSGSKMWASQRAASNFTEIKNLLVNTLYTVRVAAVTSRGIGNWSDSKSI

TTIKGKVIPPPDIHIDSYGENYLSFTLTMESDIKVNGYVVNLFWAFDTHKQERRTLNFRG

SILSHKVGNLTAHTSYEISAWAKTDLGDSPLAFEHVMTRGVRPPAPSLKAKAINQTAVEC

TWTGPRNVVYGIFYATSFLDLYRNPKSLTTSLHNKTVIVSKDEQYLFLVRVVVPYQGPSS

DYVVVKMIPDSRLPPRHLHVVHTGKTSVVIKWESPYDSPDQDLLYAIAVKDLIRKTDRSY

KVKSRNSTVEYTLNKLEPGGKYHIIVQLGNMSKDSSIKITTVSLSAPDALKIITENDHVL

LFWKSLALKEKHFNESRGYEIHMFDSAMNITAYLGNTTDNFFKISNLKMGHNYTFTVQAR

CLFGNQICGEPAILLYDELGSGADASATQAARSTDVAAVVVPILFLILLSLGVGFAILYT

KHRRLQSSFTAFANSHYSSRLGSAIFSSGDDLGEDDEDAPMITGFSDDVPMVIA

>sp|Q92673|SORL_HUMAN 343 RHPINEYYIADASEDQVF

MATRSSRRESRLPFLFTLVALLPPGALCEVWTQRLHGGSAPLPQDRGFLVVQGDPRELRL

WARGDARGASRADEKPLRRKRSAALQPEPIKVYGQVSLNDSHNQMVVHWAGEKSNVIVAL

ARDSLALARPKSSDVYVSYDYGKSFKKISDKLNFGLGNRSEAVIAQFYHSPADNKRYIFA

DAYAQYLWITFDFCNTLQGFSIPFRAADLLLHSKASNLLLGFDRSHPNKQLWKSDDFGQT

WIMIQEHVKSFSWGIDPYDKPNTIYIERHEPSGYSTVFRSTDFFQSRENQEVILEEVRDF

QLRDKYMFATKVVHLLGSEQQSSVQLWVSFGRKPMRAAQFVTRHPINEYYIADASEDQVF

VCVSHSNNRTNLYISEAEGLKFSLSLENVLYYSPGGAGSDTLVRYFANEPFADFHRVEGL

QGVYIATLINGSMNEENMRSVITFDKGGTWEFLQAPAFTGYGEKINCELSQGCSLHLAQR

LSQLLNLQLRRMPILSKESAPGLIIATGSVGKNLASKTNVYISSSAGARWREALPGPHYY

TWGDHGGIITAIAQGMETNELKYSTNEGETWKTFIFSEKPVFVYGLLTEPGEKSTVFTIF

GSNKENVHSWLILQVNATDALGVPCTENDYKLWSPSDERGNECLLGHKTVFKRRTPHATC

FNGEDFDRPVVVSNCSCTREDYECDFGFKMSEDLSLEVCVPDPEFSGKSYSPPVPCPVGS

TYRRTRGYRKISGDTCSGGDVEARLEGELVPCPLAEENEFILYAVRKSIYRYDLASGATE

QLPLTGLRAAVALDFDYEHNCLYWSDLALDVIQRLCLNGSTGQEVIINSGLETVEALAFE

PLSQLLYWVDAGFKKIEVANPDGDFRLTIVNSSVLDRPRALVLVPQEGVMFWTDWGDLKP

GIYRSNMDGSAAYHLVSEDVKWPNGISVDDQWIYWTDAYLECIERITFSGQQRSVILDNL

PHPYAIAVFKNEIYWDDWSQLSIFRASKYSGSQMEILANQLTGLMDMKIFYKGKNTGSNA

CVPRPCSLLCLPKANNSRSCRCPEDVSSSVLPSGDLMCDCPQGYQLKNNTCVKEENTCLR

NQYRCSNGNCINSIWWCDFDNDCGDMSDERNCPTTICDLDTQFRCQESGTCIPLSYKCDL

EDDCGDNSDESHCEMHQCRSDEYNCSSGMCIRSSWVCDGDNDCRDWSDEANCTAIYHTCE

ASNFQCRNGHCIPQRWACDGDTDCQDGSDEDPVNCEKKCNGFRCPNGTCIPSSKHCDGLR

DCSDGSDEQHCEPLCTHFMDFVCKNRQQCLFHSMVCDGIIQCRDGSDEDAAFAGCSQDPE

FHKVCDEFGFQCQNGVCISLIWKCDGMDDCGDYSDEANCENPTEAPNCSRYFQFRCENGH

CIPNRWKCDRENDCGDWSDEKDCGDSHILPFSTPGPSTCLPNYYRCSSGTCVMDTWVCDG

YRDCADGSDEEACPLLANVTAASTPTQLGRCDRFEFECHQPKTCIPNWKRCDGHQDCQDG

RDEANCPTHSTLTCMSREFQCEDGEACIVLSERCDGFLDCSDESDEKACSDELTVYKVQN

LQWTADFSGDVTLTWMRPKKMPSASCVYNVYYRVVGESIWKTLETHSNKTNTVLKVLKPD

TTYQVKVQVQCLSKAHNTNDFVTLRTPEGLPDAPRNLQLSLPREAEGVIVGHWAPPIHTH

GLIREYIVEYSRSGSKMWASQRAASNFTEIKNLLVNTLYTVRVAAVTSRGIGNWSDSKSI

TTIKGKVIPPPDIHIDSYGENYLSFTLTMESDIKVNGYVVNLFWAFDTHKQERRTLNFRG

SILSHKVGNLTAHTSYEISAWAKTDLGDSPLAFEHVMTRGVRPPAPSLKAKAINQTAVEC

TWTGPRNVVYGIFYATSFLDLYRNPKSLTTSLHNKTVIVSKDEQYLFLVRVVVPYQGPSS

DYVVVKMIPDSRLPPRHLHVVHTGKTSVVIKWESPYDSPDQDLLYAIAVKDLIRKTDRSY

KVKSRNSTVEYTLNKLEPGGKYHIIVQLGNMSKDSSIKITTVSLSAPDALKIITENDHVL

LFWKSLALKEKHFNESRGYEIHMFDSAMNITAYLGNTTDNFFKISNLKMGHNYTFTVQAR

CLFGNQICGEPAILLYDELGSGADASATQAARSTDVAAVVVPILFLILLSLGVGFAILYT

KHRRLQSSFTAFANSHYSSRLGSAIFSSGDDLGEDDEDAPMITGFSDDVPMVIA

>sp|O00468|AGRIN_HUMAN 1372 APVPAFEGRSFLAFPTLRAYHTL

MAGRSHPGPLRPLLPLLVVAACVLPGAGGTCPERALERREEEANVVLTGTVEEILNVDPV

QHTYSCKVRVWRYLKGKDLVARESLLDGGNKVVISGFGDPLICDNQVSTGDTRIFFVNPA

PPYLWPAHKNELMLNSSLMRITLRNLEEVEFCVEDKPGTHFTPVPPTPPDACRGMLCGFG

AVCEPNAEGPGRASCVCKKSPCPSVVAPVCGSDASTYSNECELQRAQCSQQRRIRLLSRG

PCGSRDPCSNVTCSFGSTCARSADGLTASCLCPATCRGAPEGTVCGSDGADYPGECQLLR

RACARQENVFKKFDGPCDPCQGALPDPSRSCRVNPRTRRPEMLLRPESCPARQAPVCGDD

GVTYENDCVMGRSGAARGLLLQKVRSGQCQGRDQCPEPCRFNAVCLSRRGRPRCSCDRVT

CDGAYRPVCAQDGRTYDSDCWRQQAECRQQRAIPSKHQGPCDQAPSPCLGVQCAFGATCA

VKNGQAACECLQACSSLYDPVCGSDGVTYGSACELEATACTLGREIQVARKGPCDRCGQC

RFGALCEAETGRCVCPSECVALAQPVCGSDGHTYPSECMLHVHACTHQISLHVASAGPCE

TCGDAVCAFGAVCSAGQCVCPRCEHPPPGPVCGSDGVTYGSACELREAACLQQTQIEEAR

AGPCEQAECGSGGSGSGEDGDCEQELCRQRGGIWDEDSEDGPCVCDFSCQSVPGSPVCGS

DGVTYSTECELKKARCESQRGLYVAAQGACRGPTFAPLPPVAPLHCAQTPYGCCQDNITA

ARGVGLAGCPSACQCNPHGSYGGTCDPATGQCSCRPGVGGLRCDRCEPGFWNFRGIVTDG

RSGCTPCSCDPQGAVRDDCEQMTGLCSCKPGVAGPKCGQCPDGRALGPAGCEADASAPAT

CAEMRCEFGARCVEESGSAHCVCPMLTCPEANATKVCGSDGVTYGNECQLKTIACRQGLQ

ISIQSLGPCQEAVAPSTHPTSASVTVTTPGLLLSQALPAPPGALPLAPSSTAHSQTTPPP

SSRPRTTASVPRTTVWPVLTVPPTAPSPAPSLVASAFGESGSTDGSSDEELSGDQEASGG

GSGGLEPLEGSSVATPGPPVERASCYNSALGCCSDGKTPSLDAEGSNCPATKVFQGVLEL

EGVEGQELFYTPEMADPKSELFGETARSIESTLDDLFRNSDVKKDFRSVRLRDLGPGKSV

RAIVDVHFDPTTAFRAPDVARALLRQIQVSRRRSLGVRRPLQEHVRFMDFDWFPAFITGA

TSGAIAAGATARATTASRLPSSAVTPRAPHPSHTSQPVAKTTAAPTTRRPPTTAPSRVPG

RRPPAPQQPPKPCDSQPCFHGGTCQDWALGGGFTCSCPAGRGGAVCEKVLGAPVPAFEGR

SFLAFPTLRAYHTLRLALEFRALEPQGLLLYNGNARGKDFLALALLDGRVQLRFDTGSGP

AVLTSAVPVEPGQWHRLELSRHWRRGTLSVDGETPVLGESPSGTDGLNLDTDLFVGGVPE

DQAAVALERTFVGAGLRGCIRLLDVNNQRLELGIGPGAATRGSGVGECGDHPCLPNPCHG

GAPCQNLEAGRFHCQCPPGRVGPTCADEKSPCQPNPCHGAAPCRVLPEGGAQCECPLGRE

GTFCQTASGQDGSGPFLADFNGFSHLELRGLHTFARDLGEKMALEVVFLARGPSGLLLYN

GQKTDGKGDFVSLALRDRRLEFRYDLGKGAAVIRSREPVTLGAWTRVSLERNGRKGALRV

GDGPRVLGESPVPHTVLNLKEPLYVGGAPDFSKLARAAAVSSGFDGAIQLVSLGGRQLLT

PEHVLRQVDVTSFAGHPCTRASGHPCLNGASCVPREAAYVCLCPGGFSGPHCEKGLVEKS

AGDVDTLAFDGRTFVEYLNAVTESEKALQSNHFELSLRTEATQGLVLWSGKATERADYVA

LAIVDGHLQLSYNLGSQPVVLRSTVPVNTNRWLRVVAHREQREGSLQVGNEAPVTGSSPL

GATQLDTDGALWLGGLPELPVGPALPKAYGTGFVGCLRDVVVGRHPLHLLEDAVTKPELR

PCPTP

>sp|P17927|CR1_HUMAN 359 DDFMGQLLNGRVLFPVNLQLGA

MGASSPRSPEPVGPPAPGLPFCCGGSLLAVVVLLALPVAWGQCNAPEWLPFARPTNLTDE

FEFPIGTYLNYECRPGYSGRPFSIICLKNSVWTGAKDRCRRKSCRNPPDPVNGMVHVIKG

IQFGSQIKYSCTKGYRLIGSSSATCIISGDTVIWDNETPICDRIPCGLPPTITNGDFIST

NRENFHYGSVVTYRCNPGSGGRKVFELVGEPSIYCTSNDDQVGIWSGPAPQCIIPNKCTP

PNVENGILVSDNRSLFSLNEVVEFRCQPGFVMKGPRRVKCQALNKWEPELPSCSRVCQPP

PDVLHAERTQRDKDNFSPGQEVFYSCEPGYDLRGAASMRCTPQGDWSPAAPTCEVKSCDD

FMGQLLNGRVLFPVNLQLGAKVDFVCDEGFQLKGSSASYCVLAGMESLWNSSVPVCEQIF

CPSPPVIPNGRHTGKPLEVFPFGKAVNYTCDPHPDRGTSFDLIGESTIRCTSDPQGNGVW

SSPAPRCGILGHCQAPDHFLFAKLKTQTNASDFPIGTSLKYECRPEYYGRPFSITCLDNL

VWSSPKDVCKRKSCKTPPDPVNGMVHVITDIQVGSRINYSCTTGHRLIGHSSAECILSGN

AAHWSTKPPICQRIPCGLPPTIANGDFISTNRENFHYGSVVTYRCNPGSGGRKVFELVGE

PSIYCTSNDDQVGIWSGPAPQCIIPNKCTPPNVENGILVSDNRSLFSLNEVVEFRCQPGF

VMKGPRRVKCQALNKWEPELPSCSRVCQPPPDVLHAERTQRDKDNFSPGQEVFYSCEPGY

DLRGAASMRCTPQGDWSPAAPTCEVKSCDDFMGQLLNGRVLFPVNLQLGAKVDFVCDEGF

QLKGSSASYCVLAGMESLWNSSVPVCEQIFCPSPPVIPNGRHTGKPLEVFPFGKAVNYTC

DPHPDRGTSFDLIGESTIRCTSDPQGNGVWSSPAPRCGILGHCQAPDHFLFAKLKTQTNA

SDFPIGTSLKYECRPEYYGRPFSITCLDNLVWSSPKDVCKRKSCKTPPDPVNGMVHVITD

IQVGSRINYSCTTGHRLIGHSSAECILSGNTAHWSTKPPICQRIPCGLPPTIANGDFIST

NRENFHYGSVVTYRCNLGSRGRKVFELVGEPSIYCTSNDDQVGIWSGPAPQCIIPNKCTP

PNVENGILVSDNRSLFSLNEVVEFRCQPGFVMKGPRRVKCQALNKWEPELPSCSRVCQPP

PEILHGEHTPSHQDNFSPGQEVFYSCEPGYDLRGAASLHCTPQGDWSPEAPRCAVKSCDD

FLGQLPHGRVLFPLNLQLGAKVSFVCDEGFRLKGSSVSHCVLVGMRSLWNNSVPVCEHIF

CPNPPAILNGRHTGTPSGDIPYGKEISYTCDPHPDRGMTFNLIGESTIRCTSDPHGNGVW

SSPAPRCELSVRAGHCKTPEQFPFASPTIPINDFEFPVGTSLNYECRPGYFGKMFSISCL

ENLVWSSVEDNCRRKSCGPPPEPFNGMVHINTDTQFGSTVNYSCNEGFRLIGSPSTTCLV

SGNNVTWDKKAPICEIISCEPPPTISNGDFYSNNRTSFHNGTVVTYQCHTGPDGEQLFEL

VGERSIYCTSKDDQVGVWSSPPPRCISTNKCTAPEVENAIRVPGNRSFFSLTEIIRFRCQ

PGFVMVGSHTVQCQTNGRWGPKLPHCSRVCQPPPEILHGEHTLSHQDNFSPGQEVFYSCE

PSYDLRGAASLHCTPQGDWSPEAPRCTVKSCDDFLGQLPHGRVLLPLNLQLGAKVSFVCD

EGFRLKGRSASHCVLAGMKALWNSSVPVCEQIFCPNPPAILNGRHTGTPFGDIPYGKEIS

YACDTHPDRGMTFNLIGESSIRCTSDPQGNGVWSSPAPRCELSVPAACPHPPKIQNGHYI

GGHVSLYLPGMTISYTCDPGYLLVGKGFIFCTDQGIWSQLDHYCKEVNCSFPLFMNGISK

ELEMKKVYHYGDYVTLKCEDGYTLEGSPWSQCQADDRWDPPLAKCTSRAHDALIVGTLSG

TIFFILLIIFLSWIILKHRKGNNAHENPKEVAIHLHSQGGSSVHPRTLQTNEENSRVLP

>sp|P13535|MYH8_HUMAN 1527 HELEKIKKQVEQEKCEIQAAL

MSASSDAEMAVFGEAAPYLRKSEKERIEAQNKPFDAKTSVFVAEPKESYVKSTIQSKEGG

KVTVKTEGGATLTVREDQVFPMNPPKYDKIEDMAMMTHLHEPGVLYNLKERYAAWMIYTY

SGLFCVTVNPYKWLPVYKPEVVAAYRGKKRQEAPPHIFSISDNAYQFMLTDRENQSILIT

GESGAGKTVNTKRVIQYFATIAVTGEKKKDESGKMQGTLEDQIISANPLLEAFGNAKTVR

NDNSSRFGKFIRIHFGTTGKLASADIETYLLEKSRVTFQLKAERSYHIFYQITSNKKPDL

IEMLLITTNPYDYAFVSQGEITVPSIDDQEELMATDSAIDILGFTPEEKVSIYKLTGAVM

HYGNMKFKQKQREEQAEPDGTEVADKAAYLQSLNSADLLKALCYPRVKVGNEYVTKGQTV

QQVYNAVGALAKAVYEKMFLWMVTRINQQLDTKQPRQYFIGVLDIAGFEIFDFNSLEQLC

INFTNEKLQQFFNHHMFVLEQEEYKKEGIEWTFIDFGMDLAACIELIEKPLGIFSILEEE

CMFPKATDTSFKNKLYDQHLGKSANFQKPKVVKGKAEAHFSLIHYAGTVDYNITGWLDKN

KDPLNDTVVGLYQKSAMKTLASLFSTYASAEADSSAKKGAKKKGSSFQTVSALFRENLNK

LMTNLRSTHPHFVRCIIPNETKTPGAMEHELVLHQLRCNGVLEGIRICRKGFPSRILYGD

FKQRYKVLNASAIPEGQFIDSKKASEKLLASIDIDHTQYKFGHTKVFFKAGLLGLLEEMR

DEKLAQIITRTQAVCRGFLMRVEYQKMLQRREALFCIQYNVRAFMNVKHWPWMKLFFKIK

PLLKSAETEKEMATMKEEFQKTKDELAKSEAKRKELEEKMVTLLKEKNDLQLQVQSEADS

LADAEERCEQLIKNKIQLEAKIKEVTERAEEEEEINAELTAKKRKLEDECSELKKDIDDL

ELTLAKVEKEKHATENKVKNLTEEMAGLDETIAKLSKEKKALQETHQQTLDDLQAEEDKV

NILTKAKTKLEQQVDDLEGSLEQEKKLRMDLERAKRKLEGDLKLAQESTMDMENDKQQLD

EKLEKKEFEISNLISKIEDEQAVEIQLQKKIKELQARIEELGEEIEAERASRAKAEKQRS

DLSRELEEISERLEEAGGATSAQVELNKKREAEFQKLRRDLEEATLQHEAMVAALRKKHA

DSMAELGEQIDNLQRVKQKLEKEKSELKMETDDLSSNAEAISKAKGNLEKMCRSLEDQVS

ELKTKEEEQQRLINDLTAQRARLQTEAGEYSRQLDEKDALVSQLSRSKQASTQQIEELKH

QLEEETKAKNALAHALQSSRHDCDLLREQYEEEQEGKAELQRALSKANSEVAQWRTKYET

DAIQRTEELEEAKKKLAQRLQEAEEHVEAVNAKCASLEKTKQRLQNEVEDLMLDVERSNA

ACAALDKKQRNFDKVLSEWKQKYEETQAELEASQKESRSLSTELFKVKNVYEESLDQLET

LRRENKNLQQEISDLTEQIAEGGKQIHELEKIKKQVEQEKCEIQAALEEAEASLEHEEGK

ILRIQLELNQVKSEVDRKIAEKDEEIDQLKRNHTRVVETMQSTLDAEIRSRNDALRVKKK

MEGDLNEMEIQLNHANRLAAESLRNYRNTQGILKETQLHLDDALRGQEDLKEQLAIVERR

ANLLQAEIEELWATLEQTERSRKIAEQELLDASERVQLLHTQNTSLINTKKKLENDVSQL

QSEVEEVIQESRNAEEKAKKAITDAAMMAEELKKEQDTSAHLERMKKNLEQTVKDLQHRL

DEAEQLALKGGKKQIQKLEARVRELEGEVENEQKRNAEAVKGLRKHERRVKELTYQTEED

RKNVLRLQDLVDKLQAKVKSYKRQAEEAEEQSNANLSKFRKLQHELEEAEERADIAESQV

NKLRVKSREVHTKISAE

>sp|P10586|PTPRF_HUMAN 1302 DPVEMRRLNYQTPG

MVPLVPALVMLGLVAGAHGDSKPVFIKVPEDQTGLSGGVASFVCQATGEPKPRITWMKKG

KKVSSQRFEVIEFDDGAGSVLRIQPLRVQRDEAIYECTATNSLGEINTSAKLSVLEEEQL

PPGFPSIDMGPQLKVVEKARTATMLCAAGGNPDPEISWFKDFLPVDPATSNGRIKQLRSG

ALQIESSEESDQGKYECVATNSAGTRYSAPANLYVRVRRVAPRFSIPPSSQEVMPGGSVN

LTCVAVGAPMPYVKWMMGAEELTKEDEMPVGRNVLELSNVVRSANYTCVAISSLGMIEAT

AQVTVKALPKPPIDLVVTETTATSVTLTWDSGNSEPVTYYGIQYRAAGTEGPFQEVDGVA

TTRYSIGGLSPFSEYAFRVLAVNSIGRGPPSEAVRARTGEQAPSSPPRRVQARMLSASTM

LVQWEPPEEPNGLVRGYRVYYTPDSRRPPNAWHKHNTDAGLLTTVGSLLPGITYSLRVLA

FTAVGDGPPSPTIQVKTQQGVPAQPADFQAEVESDTRIQLSWLLPPQERIIMYELVYWAA

EDEDQQHKVTFDPTSSYTLEDLKPDTLYRFQLAARSDMGVGVFTPTIEARTAQSTPSAPP

QKVMCVSMGSTTVRVSWVPPPADSRNGVITQYSVAHEAVDGEDRGRHVVDGISREHSSWD

LVGLEKWTEYRVWVRAHTDVGPGPESSPVLVRTDEDVPSGPPRKVEVEPLNSTAVHVYWK

LPVPSKQHGQIRGYQVTYVRLENGEPRGLPIIQDVMLAEAQWRPEESEDYETTISGLTPE

TTYSVTVAAYTTKGDGARSKPKIVTTTGAVPGRPTMMISTTAMNTALLQWHPPKELPGEL

LGYRLQYCRADEARPNTIDFGKDDQHFTVTGLHKGTTYIFRLAAKNRAGLGEEFEKEIRT

PEDLPSGFPQNLHVTGLTTSTTELAWDPPVLAERNGRIISYTVVFRDINSQQELQNITTD

TRFTLTGLKPDTTYDIKVRAWTSKGSGPLSPSIQSRTMPVEQVFAKNFRVAAAMKTSVLL

SWEVPDSYKSAVPFKILYNGQSVEVDGHSMRKLIADLQPNTEYSFVLMNRGSSAGGLQHL

VSIRTAPDLLPHKPLPASAYIEDGRFDLSMPHVQDPSLVRWFYIVVVPIDRVGGSMLTPR

WSTPEELELDELLEAIEQGGEEQRRRRRQAERLKPYVAAQLDVLPETFTLGDKKNYRGFY

NRPLSPDLSYQCFVLASLKEPMDQKRYASSPYSDEIVVQVTPAQQQEEPEMLWVTGPVLA

VILIILIVIAILLFKRKRTHSPSSKDEQSIGLKDSLLAHSSDPVEMRRLNYQTPGMRDHP

PIPITDLADNIERLKANDGLKFSQEYESIDPGQQFTWENSNLEVNKPKNRYANVIAYDHS

RVILTSIDGVPGSDYINANYIDGYRKQNAYIATQGPLPETMGDFWRMVWEQRTATVVMMT

RLEEKSRVKCDQYWPARGTETCGLIQVTLLDTVELATYTVRTFALHKSGSSEKRELRQFQ

FMAWPDHGVPEYPTPILAFLRRVKACNPLDAGPMVVHCSAGVGRTGCFIVIDAMLERMKH

EKTVDIYGHVTCMRSQRNYMVQTEDQYVFIHEALLEAATCGHTEVPARNLYAHIQKLGQV

PPGESVTAMELEFKLLASSKAHTSRFISANLPCNKFKNRLVNIMPYELTRVCLQPIRGVE

GSDYINASFLDGYRQQKAYIATQGPLAESTEDFWRMLWEHNSTIIVMLTKLREMGREKCH

QYWPAERSARYQYFVVDPMAEYNMPQYILREFKVTDARDGQSRTIRQFQFTDWPEQGVPK

TGEGFIDFIGQVHKTKEQFGQDGPITVHCSAGVGRTGVFITLSIVLERMRYEGVVDMFQT

VKTLRTQRPAMVQTEDQYQLCYRAALEYLGSFDHYAT

>sp|Q00610|CLH1_HUMAN 1008 LEKIVLDNSVFSEHRN

MAQILPIRFQEHLQLQNLGINPANIGFSTLTMESDKFICIREKVGEQAQVVIIDMNDPSN

PIRRPISADSAIMNPASKVIALKAGKTLQIFNIEMKSKMKAHTMTDDVTFWKWISLNTVA

LVTDNAVYHWSMEGESQPVKMFDRHSSLAGCQIINYRTDAKQKWLLLTGISAQQNRVVGA

MQLYSVDRKVSQPIEGHAASFAQFKMEGNAEESTLFCFAVRGQAGGKLHIIEVGTPPTGN

QPFPKKAVDVFFPPEAQNDFPVAMQISEKHDVVFLITKYGYIHLYDLETGTCIYMNRISG

ETIFVTAPHEATAGIIGVNRKGQVLSVCVEEENIIPYITNVLQNPDLALRMAVRNNLAGA

EELFARKFNALFAQGNYSEAAKVAANAPKGILRTPDTIRRFQSVPAQPGQTSPLLQYFGI

LLDQGQLNKYESLELCRPVLQQGRKQLLEKWLKEDKLECSEELGDLVKSVDPTLALSVYL

RANVPNKVIQCFAETGQVQKIVLYAKKVGYTPDWIFLLRNVMRISPDQGQQFAQMLVQDE

EPLADITQIVDVFMEYNLIQQCTAFLLDALKNNRPSEGPLQTRLLEMNLMHAPQVADAIL

GNQMFTHYDRAHIAQLCEKAGLLQRALEHFTDLYDIKRAVVHTHLLNPEWLVNYFGSLSV

EDSLECLRAMLSANIRQNLQICVQVASKYHEQLSTQSLIELFESFKSFEGLFYFLGSIVN

FSQDPDVHFKYIQAACKTGQIKEVERICRESNCYDPERVKNFLKEAKLTDQLPLIIVCDR

FDFVHDLVLYLYRNNLQKYIEIYVQKVNPSRLPVVIGGLLDVDCSEDVIKNLILVVRGQF

STDELVAEVEKRNRLKLLLPWLEARIHEGCEEPATHNALAKIYIDSNNNPERFLRENPYY

DSRVVGKYCEKRDPHLACVAYERGQCDLELINVCNENSLFKSLSRYLVRRKDPELWGSVL

LESNPYRRPLIDQVVQTALSETQDPEEVSVTVKAFMTADLPNELIELLEKIVLDNSVFSE

HRNLQNLLILTAIKADRTRVMEYINRLDNYDAPDIANIAISNELFEEAFAIFRKFDVNTS

AVQVLIEHIGNLDRAYEFAERCNEPAVWSQLAKAQLQKGMVKEAIDSYIKADDPSSYMEV

VQAANTSGNWEELVKYLQMARKKARESYVETELIFALAKTNRLAELEEFINGPNNAHIQQ

VGDRCYDEKMYDAAKLLYNNVSNFGRLASTLVHLGEYQAAVDGARKANSTRTWKEVCFAC

VDGKEFRLAQMCGLHIVVHADELEELINYYQDRGYFEELITMLEAALGLERAHMGMFTEL

AILYSKFKPQKMREHLELFWSRVNIPKVLRAAEQAHLWAELVFLYDKYEEYDNAIITMMN

HPTDAWKEGQFKDIITKVANVELYYRAIQFYLEFKPLLLNDLLMVLSPRLDHTRAVNYFS

KVKQLPLVKPYLRSVQNHNNKSVNESLNNLFITEEDYQALRTSIDAYDNFDNISLAQRLE

KHELIEFRRIAAYLFKGNNRWKQSVELCKKDSLYKDAMQYASESKDTELAEELLQWFLQE

EKRECFGACLFTCYDLLRPDVVLETAWRHNIMDFAMPYFIQVMKEYLTKVDKLDASESLR

KEEEQATETQPIVYGQPQLMLTAGPSVAVPPQAPFGYGYTAPPYGQPQPGFGYSM

>sp|P08581|MET_HUMAN 59 EHHIFLGATNYIYVLNEEDLQKV

MKAPAVLAPGILVLLFTLVQRSNGECKEALAKSEMNVNMKYQLPNFTAETPIQNVILHEH

HIFLGATNYIYVLNEEDLQKVAEYKTGPVLEHPDCFPCQDCSSKANLSGGVWKDNINMAL

VVDTYYDDQLISCGSVNRGTCQRHVFPHNHTADIQSEVHCIFSPQIEEPSQCPDCVVSAL

GAKVLSSVKDRFINFFVGNTINSSYFPDHPLHSISVRRLKETKDGFMFLTDQSYIDVLPE

FRDSYPIKYVHAFESNNFIYFLTVQRETLDAQTFHTRIIRFCSINSGLHSYMEMPLECIL

TEKRKKRSTKKEVFNILQAAYVSKPGAQLARQIGASLNDDILFGVFAQSKPDSAEPMDRS

AMCAFPIKYVNDFFNKIVNKNNVRCLQHFYGPNHEHCFNRTLLRNSSGCEARRDEYRTEF

TTALQRVDLFMGQFSEVLLTSISTFIKGDLTIANLGTSEGRFMQVVVSRSGPSTPHVNFL

LDSHPVSPEVIVEHTLNQNGYTLVITGKKITKIPLNGLGCRHFQSCSQCLSAPPFVQCGW

CHDKCVRSEECLSGTWTQQICLPAIYKVFPNSAPLEGGTRLTICGWDFGFRRNNKFDLKK

TRVLLGNESCTLTLSESTMNTLKCTVGPAMNKHFNMSIIISNGHGTTQYSTFSYVDPVIT

SISPKYGPMAGGTLLTLTGNYLNSGNSRHISIGGKTCTLKSVSNSILECYTPAQTISTEF

AVKLKIDLANRETSIFSYREDPIVYEIHPTKSFISGGSTITGVGKNLNSVSVPRMVINVH

EAGRNFTVACQHRSNSEIICCTTPSLQQLNLQLPLKTKAFFMLDGILSKYFDLIYVHNPV

FKPFEKPVMISMGNENVLEIKGNDIDPEAVKGEVLKVGNKSCENIHLHSEAVLCTVPNDL

LKLNSELNIEWKQAISSTVLGKVIVQPDQNFTGLIAGVVSISTALLLLLGFFLWLKKRKQ

IKDLGSELVRYDARVHTPHLDRLVSARSVSPTTEMVSNESVDYRATFPEDQFPNSSQNGS

CRQVQYPLTDMSPILTSGDSDISSPLLQNTVHIDLSALNPELVQAVQHVVIGPSSLIVHF

NEVIGRGHFGCVYHGTLLDNDGKKIHCAVKSLNRITDIGEVSQFLTEGIIMKDFSHPNVL

SLLGICLRSEGSPLVVLPYMKHGDLRNFIRNETHNPTVKDLIGFGLQVAKGMKYLASKKF

VHRDLAARNCMLDEKFTVKVADFGLARDMYDKEYYSVHNKTGAKLPVKWMALESLQTQKF

TTKSDVWSFGVVLWELMTRGAPPYPDVNTFDITVYLLQGRRLLQPEYCPDPLYEVMLKCW

HPKAEMRPSFSELVSRISAIFSTFIGEHYVHVNATYVNVKCVAPYPSLLSSEDNADDEVD

TRPASFWETS

>sp|P08581|MET_HUMAN 724 LKIDLANRETSI

MKAPAVLAPGILVLLFTLVQRSNGECKEALAKSEMNVNMKYQLPNFTAETPIQNVILHEH

HIFLGATNYIYVLNEEDLQKVAEYKTGPVLEHPDCFPCQDCSSKANLSGGVWKDNINMAL

VVDTYYDDQLISCGSVNRGTCQRHVFPHNHTADIQSEVHCIFSPQIEEPSQCPDCVVSAL

GAKVLSSVKDRFINFFVGNTINSSYFPDHPLHSISVRRLKETKDGFMFLTDQSYIDVLPE

FRDSYPIKYVHAFESNNFIYFLTVQRETLDAQTFHTRIIRFCSINSGLHSYMEMPLECIL

TEKRKKRSTKKEVFNILQAAYVSKPGAQLARQIGASLNDDILFGVFAQSKPDSAEPMDRS

AMCAFPIKYVNDFFNKIVNKNNVRCLQHFYGPNHEHCFNRTLLRNSSGCEARRDEYRTEF

TTALQRVDLFMGQFSEVLLTSISTFIKGDLTIANLGTSEGRFMQVVVSRSGPSTPHVNFL

LDSHPVSPEVIVEHTLNQNGYTLVITGKKITKIPLNGLGCRHFQSCSQCLSAPPFVQCGW

CHDKCVRSEECLSGTWTQQICLPAIYKVFPNSAPLEGGTRLTICGWDFGFRRNNKFDLKK

TRVLLGNESCTLTLSESTMNTLKCTVGPAMNKHFNMSIIISNGHGTTQYSTFSYVDPVIT

SISPKYGPMAGGTLLTLTGNYLNSGNSRHISIGGKTCTLKSVSNSILECYTPAQTISTEF

AVKLKIDLANRETSIFSYREDPIVYEIHPTKSFISGGSTITGVGKNLNSVSVPRMVINVH

EAGRNFTVACQHRSNSEIICCTTPSLQQLNLQLPLKTKAFFMLDGILSKYFDLIYVHNPV

FKPFEKPVMISMGNENVLEIKGNDIDPEAVKGEVLKVGNKSCENIHLHSEAVLCTVPNDL

LKLNSELNIEWKQAISSTVLGKVIVQPDQNFTGLIAGVVSISTALLLLLGFFLWLKKRKQ

IKDLGSELVRYDARVHTPHLDRLVSARSVSPTTEMVSNESVDYRATFPEDQFPNSSQNGS

CRQVQYPLTDMSPILTSGDSDISSPLLQNTVHIDLSALNPELVQAVQHVVIGPSSLIVHF

NEVIGRGHFGCVYHGTLLDNDGKKIHCAVKSLNRITDIGEVSQFLTEGIIMKDFSHPNVL

SLLGICLRSEGSPLVVLPYMKHGDLRNFIRNETHNPTVKDLIGFGLQVAKGMKYLASKKF

VHRDLAARNCMLDEKFTVKVADFGLARDMYDKEYYSVHNKTGAKLPVKWMALESLQTQKF

TTKSDVWSFGVVLWELMTRGAPPYPDVNTFDITVYLLQGRRLLQPEYCPDPLYEVMLKCW

HPKAEMRPSFSELVSRISAIFSTFIGEHYVHVNATYVNVKCVAPYPSLLSSEDNADDEVD

TRPASFWETS

>sp|O75976|CBPD_HUMAN 836 VPGTYKITASARGYN

MASGRDERPPWRLGRLLLLMCLLLLGSSARAAHIKKAEATTTTTSAGAEAAEGQFDRYYH

EEELESALREAAAAGLPGLARLFSIGRSVEGRPLWVLRLTAGLGSLIPEGDAGPDAAGPD

AAGPLLPGRPQVKLVGNMHGDETVSRQVLIYLARELAAGYRRGDPRLVRLLNTTDVYLLP

SLNPDGFERAREGDCGFGDGGPSGASGRDNSRGRDLNRSFPDQFSTGEPPALDEVPEVRA

LIEWIRRNKFVLSGNLHGGSVVASYPFDDSPEHKATGIYSKTSDDEVFKYLAKAYASNHP

IMKTGEPHCPGDEDETFKDGITNGAHWYDVEGGMQDYNYVWANCFEITLELSCCKYPPAS

QLRQEWENNRESLITLIEKVHIGVKGFVKDSITGSGLENATISVAGINHNITTGRFGDFY

RLLVPGTYNLTVVLTGYMPLTVTNVVVKEGPATEVDFSLRPTVTSVIPDTTEAVSTASTV

AIPNILSGTSSSYQPIQPKDFHHHHFPDMEIFLRRFANEYPNITRLYSLGKSVESRELYV

MEISDNPGVHEPGEPEFKYIGNMHGNEVVGRELLLNLIEYLCKNFGTDPEVTDLVHNTRI

HLMPSMNPDGYEKSQEGDSISVIGRNNSNNFDLNRNFPDQFVQITDPTQPETIAVMSWMK

SYPFVLSANLHGGSLVVNYPFDDDEQGLATYSKSPDDAVFQQIALSYSKENSQMFQGRPC

KNMYPNEYFPHGITNGASWYNVPGGMQDWNYLQTNCFEVTIELGCVKYPLEKELPNFWEQ

NRRSLIQFMKQVHQGVRGFVLDATDGRGILNATISVAEINHPVTTYKTGDYWRLLVPGTY

KITASARGYNPVTKNVTVKSEGAIQVNFTLVRSSTDSNNESKKGKGASSSTNDASDPTTK

EFETLIKDLSAENGLESLMLRSSSNLALALYRYHSYKDLSEFLRGLVMNYPHITNLTNLG

QSTEYRHIWSLEISNKPNVSEPEEPKIRFVAGIHGNAPVGTELLLALAEFLCLNYKKNPA

VTQLVDRTRIVIVPSLNPDGRERAQEKDCTSKIGQTNARGKDLDTDFTNNASQPETKAII

ENLIQKQDFSLSVALDGGSMLVTYPYDKPVQTVENKETLKHLASLYANNHPSMHMGQPSC

PNKSDENIPGGVMRGAEWHSHLGSMKDYSVTYGHCPEITVYTSCCYFPSAARLPSLWADN

KRSLLSMLVEVHKGVHGFVKDKTGKPISKAVIVLNEGIKVQTKEGGYFHVLLAPGVHNII

AIADGYQQQHSQVFVHHDAASSVVIVFDTDNRIFGLPRELVVTVSGATMSALILTACIIW

CICSIKSNRHKDGFHRLRQHHDEYEDEIRMMSTGSKKSLLSHEFQDETDTEEETLYSSKH

>sp|O75976|CBPD_HUMAN 836 VPGTYKITASARGYNPV

MASGRDERPPWRLGRLLLLMCLLLLGSSARAAHIKKAEATTTTTSAGAEAAEGQFDRYYH

EEELESALREAAAAGLPGLARLFSIGRSVEGRPLWVLRLTAGLGSLIPEGDAGPDAAGPD

AAGPLLPGRPQVKLVGNMHGDETVSRQVLIYLARELAAGYRRGDPRLVRLLNTTDVYLLP

SLNPDGFERAREGDCGFGDGGPSGASGRDNSRGRDLNRSFPDQFSTGEPPALDEVPEVRA

LIEWIRRNKFVLSGNLHGGSVVASYPFDDSPEHKATGIYSKTSDDEVFKYLAKAYASNHP

IMKTGEPHCPGDEDETFKDGITNGAHWYDVEGGMQDYNYVWANCFEITLELSCCKYPPAS

QLRQEWENNRESLITLIEKVHIGVKGFVKDSITGSGLENATISVAGINHNITTGRFGDFY

RLLVPGTYNLTVVLTGYMPLTVTNVVVKEGPATEVDFSLRPTVTSVIPDTTEAVSTASTV

AIPNILSGTSSSYQPIQPKDFHHHHFPDMEIFLRRFANEYPNITRLYSLGKSVESRELYV

MEISDNPGVHEPGEPEFKYIGNMHGNEVVGRELLLNLIEYLCKNFGTDPEVTDLVHNTRI

HLMPSMNPDGYEKSQEGDSISVIGRNNSNNFDLNRNFPDQFVQITDPTQPETIAVMSWMK

SYPFVLSANLHGGSLVVNYPFDDDEQGLATYSKSPDDAVFQQIALSYSKENSQMFQGRPC

KNMYPNEYFPHGITNGASWYNVPGGMQDWNYLQTNCFEVTIELGCVKYPLEKELPNFWEQ

NRRSLIQFMKQVHQGVRGFVLDATDGRGILNATISVAEINHPVTTYKTGDYWRLLVPGTY

KITASARGYNPVTKNVTVKSEGAIQVNFTLVRSSTDSNNESKKGKGASSSTNDASDPTTK

EFETLIKDLSAENGLESLMLRSSSNLALALYRYHSYKDLSEFLRGLVMNYPHITNLTNLG

QSTEYRHIWSLEISNKPNVSEPEEPKIRFVAGIHGNAPVGTELLLALAEFLCLNYKKNPA

VTQLVDRTRIVIVPSLNPDGRERAQEKDCTSKIGQTNARGKDLDTDFTNNASQPETKAII

ENLIQKQDFSLSVALDGGSMLVTYPYDKPVQTVENKETLKHLASLYANNHPSMHMGQPSC

PNKSDENIPGGVMRGAEWHSHLGSMKDYSVTYGHCPEITVYTSCCYFPSAARLPSLWADN

KRSLLSMLVEVHKGVHGFVKDKTGKPISKAVIVLNEGIKVQTKEGGYFHVLLAPGVHNII

AIADGYQQQHSQVFVHHDAASSVVIVFDTDNRIFGLPRELVVTVSGATMSALILTACIIW

CICSIKSNRHKDGFHRLRQHHDEYEDEIRMMSTGSKKSLLSHEFQDETDTEEETLYSSKH

>sp|O75976|CBPD_HUMAN 836 VPGTYKITASARGYNPVT

MASGRDERPPWRLGRLLLLMCLLLLGSSARAAHIKKAEATTTTTSAGAEAAEGQFDRYYH

EEELESALREAAAAGLPGLARLFSIGRSVEGRPLWVLRLTAGLGSLIPEGDAGPDAAGPD

AAGPLLPGRPQVKLVGNMHGDETVSRQVLIYLARELAAGYRRGDPRLVRLLNTTDVYLLP

SLNPDGFERAREGDCGFGDGGPSGASGRDNSRGRDLNRSFPDQFSTGEPPALDEVPEVRA

LIEWIRRNKFVLSGNLHGGSVVASYPFDDSPEHKATGIYSKTSDDEVFKYLAKAYASNHP

IMKTGEPHCPGDEDETFKDGITNGAHWYDVEGGMQDYNYVWANCFEITLELSCCKYPPAS

QLRQEWENNRESLITLIEKVHIGVKGFVKDSITGSGLENATISVAGINHNITTGRFGDFY

RLLVPGTYNLTVVLTGYMPLTVTNVVVKEGPATEVDFSLRPTVTSVIPDTTEAVSTASTV

AIPNILSGTSSSYQPIQPKDFHHHHFPDMEIFLRRFANEYPNITRLYSLGKSVESRELYV

MEISDNPGVHEPGEPEFKYIGNMHGNEVVGRELLLNLIEYLCKNFGTDPEVTDLVHNTRI

HLMPSMNPDGYEKSQEGDSISVIGRNNSNNFDLNRNFPDQFVQITDPTQPETIAVMSWMK

SYPFVLSANLHGGSLVVNYPFDDDEQGLATYSKSPDDAVFQQIALSYSKENSQMFQGRPC

KNMYPNEYFPHGITNGASWYNVPGGMQDWNYLQTNCFEVTIELGCVKYPLEKELPNFWEQ

NRRSLIQFMKQVHQGVRGFVLDATDGRGILNATISVAEINHPVTTYKTGDYWRLLVPGTY

KITASARGYNPVTKNVTVKSEGAIQVNFTLVRSSTDSNNESKKGKGASSSTNDASDPTTK

EFETLIKDLSAENGLESLMLRSSSNLALALYRYHSYKDLSEFLRGLVMNYPHITNLTNLG

QSTEYRHIWSLEISNKPNVSEPEEPKIRFVAGIHGNAPVGTELLLALAEFLCLNYKKNPA

VTQLVDRTRIVIVPSLNPDGRERAQEKDCTSKIGQTNARGKDLDTDFTNNASQPETKAII

ENLIQKQDFSLSVALDGGSMLVTYPYDKPVQTVENKETLKHLASLYANNHPSMHMGQPSC

PNKSDENIPGGVMRGAEWHSHLGSMKDYSVTYGHCPEITVYTSCCYFPSAARLPSLWADN

KRSLLSMLVEVHKGVHGFVKDKTGKPISKAVIVLNEGIKVQTKEGGYFHVLLAPGVHNII

AIADGYQQQHSQVFVHHDAASSVVIVFDTDNRIFGLPRELVVTVSGATMSALILTACIIW

CICSIKSNRHKDGFHRLRQHHDEYEDEIRMMSTGSKKSLLSHEFQDETDTEEETLYSSKH

>sp|O15439|MRP4_HUMAN 808 APVLFFDRNPIGRIL

MLPVYQEVKPNPLQDANICSRVFFWWLNPLFKIGHKRRLEEDDMYSVLPEDRSQHLGEEL

QGFWDKEVLRAENDAQKPSLTRAIIKCYWKSYLVLGIFTLIEESAKVIQPIFLGKIINYF

ENYDPMDSVALNTAYAYATVLTFCTLILAILHHLYFYHVQCAGMRLRVAMCHMIYRKALR

LSNMAMGKTTTGQIVNLLSNDVNKFDQVTVFLHFLWAGPLQAIAVTALLWMEIGISCLAG

MAVLIILLPLQSCFGKLFSSLRSKTATFTDARIRTMNEVITGIRIIKMYAWEKSFSNLIT

NLRKKEISKILRSSCLRGMNLASFFSASKIIVFVTFTTYVLLGSVITASRVFVAVTLYGA

VRLTVTLFFPSAIERVSEAIVSIRRIQTFLLLDEISQRNRQLPSDGKKMVHVQDFTAFWD

KASETPTLQGLSFTVRPGELLAVVGPVGAGKSSLLSAVLGELAPSHGLVSVHGRIAYVSQ

QPWVFSGTLRSNILFGKKYEKERYEKVIKACALKKDLQLLEDGDLTVIGDRGTTLSGGQK

ARVNLARAVYQDADIYLLDDPLSAVDAEVSRHLFELCICQILHEKITILVTHQLQYLKAA

SQILILKDGKMVQKGTYTEFLKSGIDFGSLLKKDNEESEQPPVPGTPTLRNRTFSESSVW

SQQSSRPSLKDGALESQDTENVPVTLSEENRSEGKVGFQAYKNYFRAGAHWIVFIFLILL

NTAAQVAYVLQDWWLSYWANKQSMLNVTVNGGGNVTEKLDLNWYLGIYSGLTVATVLFGI

ARSLLVFYVLVNSSQTLHNKMFESILKAPVLFFDRNPIGRILNRFSKDIGHLDDLLPLTF

LDFIQTLLQVVGVVSVAVAVIPWIAIPLVPLGIIFIFLRRYFLETSRDVKRLESTTRSPV

FSHLSSSLQGLWTIRAYKAEERCQELFDAHQDLHSEAWFLFLTTSRWFAVRLDAICAMFV

IIVAFGSLILAKTLDAGQVGLALSYALTLMGMFQWCVRQSAEVENMMISVERVIEYTDLE

KEAPWEYQKRPPPAWPHEGVIIFDNVNFMYSPGGPLVLKHLTALIKSQEKVGIVGRTGAG

KSSLISALFRLSEPEGKIWIDKILTTEIGLHDLRKKMSIIPQEPVLFTGTMRKNLDPFKE

HTDEELWNALQEVQLKETIEDLPGKMDTELAESGSNFSVGQRQLVCLARAILRKNQILII

DEATANVDPRTDELIQKKIREKFAHCTVLTIAHRLNTIIDSDKIMVLDSGRLKEYDEPYV

LLQNKESLFYKMVQQLGKAEAAALTETAKQVYFKRNYPHIGHTDHMVTNTSNGQPSTLTI

FETAL

>sp|P08575|CD45_HUMAN 389 SPGEPQIIFCRSEAAHQGVI

MYLWLKLLAFGFAFLDTEVFVTGQSPTPSPTGLTTAKMPSVPLSSDPLPTHTTAFSPAST

FERENDFSETTTSLSPDNTSTQVSPDSLDNASAFNTTGVSSVQTPHLPTHADSQTPSAGT

DTQTFSGSAANAKLNPTPGSNAISDVPGERSTASTFPTDPVSPLTTTLSLAHHSSAALPA

RTSNTTITANTSDAYLNASETTTLSPSGSAVISTTTIATTPSKPTCDEKYANITVDYLYN

KETKLFTAKLNVNENVECGNNTCTNNEVHNLTECKNASVSISHNSCTAPDKTLILDVPPG

VEKFQLHDCTQVEKADTTICLKWKNIETFTCDTQNITYRFQCGNMIFDNKEIKLENLEPE

HEYKCDSEILYNNHKFTNASKIIKTDFGSPGEPQIIFCRSEAAHQGVITWNPPQRSFHNF

TLCYIKETEKDCLNLDKNLIKYDLQNLKPYTKYVLSLHAYIIAKVQRNGSAAMCHFTTKS

APPSQVWNMTVSMTSDNSMHVKCRPPRDRNGPHERYHLEVEAGNTLVRNESHKNCDFRVK

DLQYSTDYTFKAYFHNGDYPGEPFILHHSTSYNSKALIAFLAFLIIVTSIALLVVLYKIY

DLHKKRSCNLDEQQELVERDDEKQLMNVEPIHADILLETYKRKIADEGRLFLAEFQSIPR

VFSKFPIKEARKPFNQNKNRYVDILPYDYNRVELSEINGDAGSNYINASYIDGFKEPRKY

IAAQGPRDETVDDFWRMIWEQKATVIVMVTRCEEGNRNKCAEYWPSMEEGTRAFGDVVVK

INQHKRCPDYIIQKLNIVNKKEKATGREVTHIQFTSWPDHGVPEDPHLLLKLRRRVNAFS

NFFSGPIVVHCSAGVGRTGTYIGIDAMLEGLEAENKVDVYGYVVKLRRQRCLMVQVEAQY

ILIHQALVEYNQFGETEVNLSELHPYLHNMKKRDPPSEPSPLEAEFQRLPSYRSWRTQHI

GNQEENKSKNRNSNVIPYDYNRVPLKHELEMSKESEHDSDESSDDDSDSEEPSKYINASF

IMSYWKPEVMIAAQGPLKETIGDFWQMIFQRKVKVIVMLTELKHGDQEICAQYWGEGKQT

YGDIEVDLKDTDKSSTYTLRVFELRHSKRKDSRTVYQYQYTNWSVEQLPAEPKELISMIQ

VVKQKLPQKNSSEGNKHHKSTPLLIHCRDGSQQTGIFCALLNLLESAETEEVVDIFQVVK

ALRKARPGMVSTFEQYQFLYDVIASTYPAQNGQVKKNNHQEDKIEFDNEVDKVKQDANCV

NPLGAPEKLPEAKEQAEGSEPTSGTEGPEHSVNGPASPALNQGS

>sp|P14543|NID1_HUMAN 457 DLHSYVVMNHGRSYTAIS

MLASSSRIRAAWTRALLLPLLLAGPVGCLSRQELFPFGPGQGDLELEDGDDFVSPALELS

GALRFYDRSDIDAVYVTTNGIIATSEPPAKESHPGLFPPTFGAVAPFLADLDTTDGLGKV

YYREDLSPSITQRAAECVHRGFPEISFQPSSAVVVTWESVAPYQGPSRDPDQKGKRNTFQ

AVLASSDSSSYAIFLYPEDGLQFHTTFSKKENNQVPAVVAFSQGSVGFLWKSNGAYNIFA

NDRESIENLAKSSNSGQQGVWVFEIGSPATTNGVVPADVILGTEDGAEYDDEDEDYDLAT

TRLGLEDVGTTPFSYKALRRGGADTYSVPSVLSPRRAATERPLGPPTERTRSFQLAVETF

HQQHPQVIDVDEVEETGVVFSYNTDSRQTCANNRHQCSVHAECRDYATGFCCSCVAGYTG

NGRQCVAEGSPQRVNGKVKGRIFVGSSQVPIVFENTDLHSYVVMNHGRSYTAISTIPETV

GYSLLPLAPVGGIIGWMFAVEQDGFKNGFSITGGEFTRQAEVTFVGHPGNLVIKQRFSGI

DEHGHLTIDTELEGRVPQIPFGSSVHIEPYTELYHYSTSVITSSSTREYTVTEPERDGAS

PSRIYTYQWRQTITFQECVHDDSRPALPSTQQLSVDSVFVLYNQEEKILRYAFSNSIGPV

REGSPDALQNPCYIGTHGCDTNAACRPGPRTQFTCECSIGFRGDGRTCYDIDECSEQPSV

CGSHTICNNHPGTFRCECVEGYQFSDEGTCVAVVDQRPINYCETGLHNCDIPQRAQCIYT

GGSSYTCSCLPGFSGDGQACQDVDECQPSRCHPDAFCYNTPGSFTCQCKPGYQGDGFRCV

PGEVEKTRCQHEREHILGAAGATDPQRPIPPGLFVPECDAHGHYAPTQCHGSTGYCWCVD

RDGREVEGTRTRPGMTPPCLSTVAPPIHQGPAVPTAVIPLPPGTHLLFAQTGKIERLPLE

GNTMRKTEAKAFLHVPAKVIIGLAFDCVDKMVYWTDITEPSIGRASLHGGEPTTIIRQDL

GSPEGIAVDHLGRNIFWTDSNLDRIEVAKLDGTQRRVLFETDLVNPRGIVTDSVRGNLYW

TDWNRDNPKIETSYMDGTNRRILVQDDLGLPNGLTFDAFSSQLCWVDAGTNRAECLNPSQ

PSRRKALEGLQYPFAVTSYGKNLYFTDWKMNSVVALDLAISKETDAFQPHKQTRLYGITT

ALSQCPQGHNYCSVNNGGCTHLCLATPGSRTCRCPDNTLGVDCIERK

>sp|Q86VP6|CAND1_HUMAN 152 LEALDIMADMLSRQGG

MASASYHISNLLEKMTSSDKDFRFMATNDLMTELQKDSIKLDDDSERKVVKMILKLLEDK

NGEVQNLAVKCLGPLVSKVKEYQVETIVDTLCTNMLSDKEQLRDISSIGLKTVIGELPPA

SSGSALAANVCKKITGRLTSAIAKQEDVSVQLEALDIMADMLSRQGGLLVNFHPSILTCL

LPQLTSPRLAVRKRTIIALGHLVMSCGNIVFVDLIEHLLSELSKNDSMSTTRTYIQCIAA

ISRQAGHRIGEYLEKIIPLVVKFCNVDDDELREYCIQAFESFVRRCPKEVYPHVSTIINI

CLKYLTYDPNYNYDDEDEDENAMDADGGDDDDQGSDDEYSDDDDMSWKVRRAAAKCLDAV

VSTRHEMLPEFYKTVSPALISRFKEREENVKADVFHAYLSLLKQTRPVQSWLCDPDAMEQ

GETPLTMLQSQVPNIVKALHKQMKEKSVKTRQCCFNMLTELVNVLPGALTQHIPVLVPGI

IFSLNDKSSSSNLKIDALSCLYVILCNHSPQVFHPHVQALVPPVVACVGDPFYKITSEAL

LVTQQLVKVIRPLDQPSSFDATPYIKDLFTCTIKRLKAADIDQEVKERAISCMGQIICNL

GDNLGSDLPNTLQIFLERLKNEITRLTTVKALTLIAGSPLKIDLRPVLGEGVPILASFLR

KNQRALKLGTLSALDILIKNYSDSLTAAMIDAVLDELPPLISESDMHVSQMAISFLTTLA

KVYPSSLSKISGSILNELIGLVRSPLLQGGALSAMLDFFQALVVTGTNNLGYMDLLRMLT

GPVYSQSTALTHKQSYYSIAKCVAALTRACPKEGPAVVGQFIQDVKNSRSTDSIRLLALL

SLGEVGHHIDLSGQLELKSVILEAFSSPSEEVKSAASYALGSISVGNLPEYLPFVLQEIT

SQPKRQYLLLHSLKEIISSASVVGLKPYVENIWALLLKHCECAEEGTRNVVAECLGKLTL

IDPETLLPRLKGYLISGSSYARSSVVTAVKFTISDHPQPIDPLLKNCIGDFLKTLEDPDL

NVRRVALVTFNSAAHNKPSLIRDLLDTVLPHLYNETKVRKELIREVEMGPFKHTVDDGLD

IRKAAFECMYTLLDSCLDRLDIFEFLNHVEDGLKDHYDIKMLTFLMLVRLSTLCPSAVLQ

RLDRLVEPLRATCTTKVKANSVKQEFEKQDELKRSAMRAVAALLTIPEAEKSPLMSEFQS

QISSNPELAAIFESIQKDSSSTNLESMDTS

>sp|P00533|EGFR_HUMAN 271 NPTTYQMDVNPEGKYS

MRPSGTAGAALLALLAALCPASRALEEKKVCQGTSNKLTQLGTFEDHFLSLQRMFNNCEV

VLGNLEITYVQRNYDLSFLKTIQEVAGYVLIALNTVERIPLENLQIIRGNMYYENSYALA

VLSNYDANKTGLKELPMRNLQEILHGAVRFSNNPALCNVESIQWRDIVSSDFLSNMSMDF

QNHLGSCQKCDPSCPNGSCWGAGEENCQKLTKIICAQQCSGRCRGKSPSDCCHNQCAAGC

TGPRESDCLVCRKFRDEATCKDTCPPLMLYNPTTYQMDVNPEGKYSFGATCVKKCPRNYV

VTDHGSCVRACGADSYEMEEDGVRKCKKCEGPCRKVCNGIGIGEFKDSLSINATNIKHFK

NCTSISGDLHILPVAFRGDSFTHTPPLDPQELDILKTVKEITGFLLIQAWPENRTDLHAF

ENLEIIRGRTKQHGQFSLAVVSLNITSLGLRSLKEISDGDVIISGNKNLCYANTINWKKL

FGTSGQKTKIISNRGENSCKATGQVCHALCSPEGCWGPEPRDCVSCRNVSRGRECVDKCN

LLEGEPREFVENSECIQCHPECLPQAMNITCTGRGPDNCIQCAHYIDGPHCVKTCPAGVM

GENNTLVWKYADAGHVCHLCHPNCTYGCTGPGLEGCPTNGPKIPSIATGMVGALLLLLVV

ALGIGLFMRRRHIVRKRTLRRLLQERELVEPLTPSGEAPNQALLRILKETEFKKIKVLGS

GAFGTVYKGLWIPEGEKVKIPVAIKELREATSPKANKEILDEAYVMASVDNPHVCRLLGI

CLTSTVQLITQLMPFGCLLDYVREHKDNIGSQYLLNWCVQIAKGMNYLEDRRLVHRDLAA

RNVLVKTPQHVKITDFGLAKLLGAEEKEYHAEGGKVPIKWMALESILHRIYTHQSDVWSY

GVTVWELMTFGSKPYDGIPASEISSILEKGERLPQPPICTIDVYMIMVKCWMIDADSRPK

FRELIIEFSKMARDPQRYLVIQGDERMHLPSPTDSNFYRALMDEEDMDDVVDADEYLIPQ

QGFFSSPSTSRTPLLSSLSATSNNSTVACIDRNGLQSCPIKEDSFLQRYSSDPTGALTED

SIDDTFLPVPEYINQSVPKRPAGSVQNPVYHNQPLNPAPSRDPHYQDPHSTAVGNPEYLN

TVQPTCVNSTFDSPAHWAQKGSHQISLDNPDYQQDFFPKEAKPNGIFKGSTAENAEYLRV

APQSSEFIGA

>sp|P00533|EGFR_HUMAN 270 YNPTTYQMDVNPEGKYS

MRPSGTAGAALLALLAALCPASRALEEKKVCQGTSNKLTQLGTFEDHFLSLQRMFNNCEV

VLGNLEITYVQRNYDLSFLKTIQEVAGYVLIALNTVERIPLENLQIIRGNMYYENSYALA

VLSNYDANKTGLKELPMRNLQEILHGAVRFSNNPALCNVESIQWRDIVSSDFLSNMSMDF

QNHLGSCQKCDPSCPNGSCWGAGEENCQKLTKIICAQQCSGRCRGKSPSDCCHNQCAAGC

TGPRESDCLVCRKFRDEATCKDTCPPLMLYNPTTYQMDVNPEGKYSFGATCVKKCPRNYV

VTDHGSCVRACGADSYEMEEDGVRKCKKCEGPCRKVCNGIGIGEFKDSLSINATNIKHFK

NCTSISGDLHILPVAFRGDSFTHTPPLDPQELDILKTVKEITGFLLIQAWPENRTDLHAF

ENLEIIRGRTKQHGQFSLAVVSLNITSLGLRSLKEISDGDVIISGNKNLCYANTINWKKL

FGTSGQKTKIISNRGENSCKATGQVCHALCSPEGCWGPEPRDCVSCRNVSRGRECVDKCN

LLEGEPREFVENSECIQCHPECLPQAMNITCTGRGPDNCIQCAHYIDGPHCVKTCPAGVM

GENNTLVWKYADAGHVCHLCHPNCTYGCTGPGLEGCPTNGPKIPSIATGMVGALLLLLVV

ALGIGLFMRRRHIVRKRTLRRLLQERELVEPLTPSGEAPNQALLRILKETEFKKIKVLGS

GAFGTVYKGLWIPEGEKVKIPVAIKELREATSPKANKEILDEAYVMASVDNPHVCRLLGI

CLTSTVQLITQLMPFGCLLDYVREHKDNIGSQYLLNWCVQIAKGMNYLEDRRLVHRDLAA

RNVLVKTPQHVKITDFGLAKLLGAEEKEYHAEGGKVPIKWMALESILHRIYTHQSDVWSY

GVTVWELMTFGSKPYDGIPASEISSILEKGERLPQPPICTIDVYMIMVKCWMIDADSRPK

FRELIIEFSKMARDPQRYLVIQGDERMHLPSPTDSNFYRALMDEEDMDDVVDADEYLIPQ

QGFFSSPSTSRTPLLSSLSATSNNSTVACIDRNGLQSCPIKEDSFLQRYSSDPTGALTED

SIDDTFLPVPEYINQSVPKRPAGSVQNPVYHNQPLNPAPSRDPHYQDPHSTAVGNPEYLN

TVQPTCVNSTFDSPAHWAQKGSHQISLDNPDYQQDFFPKEAKPNGIFKGSTAENAEYLRV

APQSSEFIGA

>sp|Q92896|GSLG1_HUMAN 873 DPELDYTLMRVCKQMIK

MAACGRVRRMFRLSAALHLLLLFAAGAEKLPGHGVHSQGQGPGANFVSFVGQAGGGGPAG

QQLPQLLQSSQLQQQQQQQQQQQQLQPPQPPFPAGGPPARRGGAGAGGGWKLAEEESCRE

DVTRVCPKHTWSNNLAVLECLQDVREPENEISSDCNHLLWNYKLNLTTDPKFESVAREVC

KSTITEIKECADEPVGKGYMVSCLVDHRGNITEYQCHQYITKMTAIIFSDYRLICGFMDD

CKNDINILKCGSIRLGEKDAHSQGEVVSCLEKGLVKEAEEREPKIQVSELCKKAILRVAE

LSSDDFHLDRHLYFACRDDRERFCENTQAGEGRVYKCLFNHKFEESMSEKCREALTTRQK

LIAQDYKVSYSLAKSCKSDLKKYRCNVENLPRSREARLSYLLMCLESAVHRGRQVSSECQ

GEMLDYRRMLMEDFSLSPEIILSCRGEIEHHCSGLHRKGRTLHCLMKVVRGEKGNLGMNC

QQALQTLIQETDPGADYRIDRALNEACESVIQTACKHIRSGDPMILSCLMEHLYTEKMVE

DCEHRLLELQYFISRDWKLDPVLYRKCQGDASRLCHTHGWNETSEFMPQGAVFSCLYRHA

YRTEEQGRRLSRECRAEVQRILHQRAMDVKLDPALQDKCLIDLGKWCSEKTETGQELECL

QDHLDDLVVECRDIVGNLTELESEDIQIEALLMRACEPIIQNFCHDVADNQIDSGDLMEC

LIQNKHQKDMNEKCAIGVTHFQLVQMKDFRFSYKFKMACKEDVLKLCPNIKKKVDVVICL

STTVRNDTLQEAKEHRVSLKCRRQLRVEELEMTEDIRLEPDLYEACKSDIKNFCSAVQYG

NAQIIECLKENKKQLSTRCHQKVFKLQETEMMDPELDYTLMRVCKQMIKRFCPEADSKTM

LQCLKQNKNSELMDPKCKQMITKRQITQNTDYRLNPMLRKACKADIPKFCHGILTKAKDD

SELEGQVISCLKLRYADQRLSSDCEDQIRIIIQESALDYRLDPQLQLHCSDEISSLCAEE

AAAQEQTGQVEECLKVNLLKIKTELCKKEVLNMLKESKADIFVDPVLHTACALDIKHHCA

AITPGRGRQMSCLMEALEDKRVRLQPECKKRLNDRIEMWSYAAKVAPADGFSDLAMQVMT

SPSKNYILSVISGSICILFLIGLMCGRITKRVTRELKDR

>sp|Q92896|GSLG1_HUMAN 1035 KVNLLKIKTELCKKEV

MAACGRVRRMFRLSAALHLLLLFAAGAEKLPGHGVHSQGQGPGANFVSFVGQAGGGGPAG

QQLPQLLQSSQLQQQQQQQQQQQQLQPPQPPFPAGGPPARRGGAGAGGGWKLAEEESCRE

DVTRVCPKHTWSNNLAVLECLQDVREPENEISSDCNHLLWNYKLNLTTDPKFESVAREVC

KSTITEIKECADEPVGKGYMVSCLVDHRGNITEYQCHQYITKMTAIIFSDYRLICGFMDD

CKNDINILKCGSIRLGEKDAHSQGEVVSCLEKGLVKEAEEREPKIQVSELCKKAILRVAE

LSSDDFHLDRHLYFACRDDRERFCENTQAGEGRVYKCLFNHKFEESMSEKCREALTTRQK

LIAQDYKVSYSLAKSCKSDLKKYRCNVENLPRSREARLSYLLMCLESAVHRGRQVSSECQ

GEMLDYRRMLMEDFSLSPEIILSCRGEIEHHCSGLHRKGRTLHCLMKVVRGEKGNLGMNC

QQALQTLIQETDPGADYRIDRALNEACESVIQTACKHIRSGDPMILSCLMEHLYTEKMVE

DCEHRLLELQYFISRDWKLDPVLYRKCQGDASRLCHTHGWNETSEFMPQGAVFSCLYRHA

YRTEEQGRRLSRECRAEVQRILHQRAMDVKLDPALQDKCLIDLGKWCSEKTETGQELECL

QDHLDDLVVECRDIVGNLTELESEDIQIEALLMRACEPIIQNFCHDVADNQIDSGDLMEC

LIQNKHQKDMNEKCAIGVTHFQLVQMKDFRFSYKFKMACKEDVLKLCPNIKKKVDVVICL

STTVRNDTLQEAKEHRVSLKCRRQLRVEELEMTEDIRLEPDLYEACKSDIKNFCSAVQYG

NAQIIECLKENKKQLSTRCHQKVFKLQETEMMDPELDYTLMRVCKQMIKRFCPEADSKTM

LQCLKQNKNSELMDPKCKQMITKRQITQNTDYRLNPMLRKACKADIPKFCHGILTKAKDD

SELEGQVISCLKLRYADQRLSSDCEDQIRIIIQESALDYRLDPQLQLHCSDEISSLCAEE

AAAQEQTGQVEECLKVNLLKIKTELCKKEVLNMLKESKADIFVDPVLHTACALDIKHHCA

AITPGRGRQMSCLMEALEDKRVRLQPECKKRLNDRIEMWSYAAKVAPADGFSDLAMQVMT

SPSKNYILSVISGSICILFLIGLMCGRITKRVTRELKDR

>sp|Q92896|GSLG1_HUMAN 643 LGKWCSEKTETGQE

MAACGRVRRMFRLSAALHLLLLFAAGAEKLPGHGVHSQGQGPGANFVSFVGQAGGGGPAG

QQLPQLLQSSQLQQQQQQQQQQQQLQPPQPPFPAGGPPARRGGAGAGGGWKLAEEESCRE

DVTRVCPKHTWSNNLAVLECLQDVREPENEISSDCNHLLWNYKLNLTTDPKFESVAREVC

KSTITEIKECADEPVGKGYMVSCLVDHRGNITEYQCHQYITKMTAIIFSDYRLICGFMDD

CKNDINILKCGSIRLGEKDAHSQGEVVSCLEKGLVKEAEEREPKIQVSELCKKAILRVAE

LSSDDFHLDRHLYFACRDDRERFCENTQAGEGRVYKCLFNHKFEESMSEKCREALTTRQK

LIAQDYKVSYSLAKSCKSDLKKYRCNVENLPRSREARLSYLLMCLESAVHRGRQVSSECQ

GEMLDYRRMLMEDFSLSPEIILSCRGEIEHHCSGLHRKGRTLHCLMKVVRGEKGNLGMNC

QQALQTLIQETDPGADYRIDRALNEACESVIQTACKHIRSGDPMILSCLMEHLYTEKMVE

DCEHRLLELQYFISRDWKLDPVLYRKCQGDASRLCHTHGWNETSEFMPQGAVFSCLYRHA

YRTEEQGRRLSRECRAEVQRILHQRAMDVKLDPALQDKCLIDLGKWCSEKTETGQELECL

QDHLDDLVVECRDIVGNLTELESEDIQIEALLMRACEPIIQNFCHDVADNQIDSGDLMEC

LIQNKHQKDMNEKCAIGVTHFQLVQMKDFRFSYKFKMACKEDVLKLCPNIKKKVDVVICL

STTVRNDTLQEAKEHRVSLKCRRQLRVEELEMTEDIRLEPDLYEACKSDIKNFCSAVQYG

NAQIIECLKENKKQLSTRCHQKVFKLQETEMMDPELDYTLMRVCKQMIKRFCPEADSKTM

LQCLKQNKNSELMDPKCKQMITKRQITQNTDYRLNPMLRKACKADIPKFCHGILTKAKDD

SELEGQVISCLKLRYADQRLSSDCEDQIRIIIQESALDYRLDPQLQLHCSDEISSLCAEE

AAAQEQTGQVEECLKVNLLKIKTELCKKEVLNMLKESKADIFVDPVLHTACALDIKHHCA

AITPGRGRQMSCLMEALEDKRVRLQPECKKRLNDRIEMWSYAAKVAPADGFSDLAMQVMT

SPSKNYILSVISGSICILFLIGLMCGRITKRVTRELKDR

>sp|P53396|ACLY_HUMAN 247 YPEEAYIADLDAKSGAS

MSAKAISEQTGKELLYKFICTTSAIQNRFKYARVTPDTDWARLLQDHPWLLSQNLVVKPD

QLIKRRGKLGLVGVNLTLDGVKSWLKPRLGQEATVGKATGFLKNFLIEPFVPHSQAEEFY

VCIYATREGDYVLFHHEGGVDVGDVDAKAQKLLVGVDEKLNPEDIKKHLLVHAPEDKKEI

LASFISGLFNFYEDLYFTYLEINPLVVTKDGVYVLDLAAKVDATADYICKVKWGDIEFPP

PFGREAYPEEAYIADLDAKSGASLKLTLLNPKGRIWTMVAGGGASVVYSDTICDLGGVNE

LANYGEYSGAPSEQQTYDYAKTILSLMTREKHPDGKILIIGGSIANFTNVAATFKGIVRA

IRDYQGPLKEHEVTIFVRRGGPNYQEGLRVMGEVGKTTGIPIHVFGTETHMTAIVGMALG

HRPIPNQPPTAAHTANFLLNASGSTSTPAPSRTASFSESRADEVAPAKKAKPAMPQDSVP

SPRSLQGKSTTLFSRHTKAIVWGMQTRAVQGMLDFDYVCSRDEPSVAAMVYPFTGDHKQK

FYWGHKEILIPVFKNMADAMRKHPEVDVLINFASLRSAYDSTMETMNYAQIRTIAIIAEG

IPEALTRKLIKKADQKGVTIIGPATVGGIKPGCFKIGNTGGMLDNILASKLYRPGSVAYV

SRSGGMSNELNNIISRTTDGVYEGVAIGGDRYPGSTFMDHVLRYQDTPGVKMIVVLGEIG

GTEEYKICRGIKEGRLTKPIVCWCIGTCATMFSSEVQFGHAGACANQASETAVAKNQALK

EAGVFVPRSFDELGEIIQSVYEDLVANGVIVPAQEVPPPTVPMDYSWARELGLIRKPASF

MTSICDERGQELIYAGMPITEVFKEEMGIGGVLGLLWFQKRLPKYSCQFIEMCLMVTADH

GPAVSGAHNTIICARAGKDLVSSLTSGLLTIGDRFGGALDAAAKMFSKAFDSGIIPMEFV

NKMKKEGKLIMGIGHRVKSINNPDMRVQILKDYVRQHFPATPLLDYALEVEKITTSKKPN

LILNVDGLIGVAFVDMLRNCGSFTREEADEYIDIGALNGIFVLGRSMGFIGHYLDQKRLK

QGLYRHPWDDISYVLPEHMSM

>sp|P42702|LIFR_HUMAN 854 TSILCYRKREWIK

MMDIYVCLKRPSWMVDNKRMRTASNFQWLLSTFILLYLMNQVNSQKKGAPHDLKCVTNNL

QVWNCSWKAPSGTGRGTDYEVCIENRSRSCYQLEKTSIKIPALSHGDYEITINSLHDFGS

STSKFTLNEQNVSLIPDTPEILNLSADFSTSTLYLKWNDRGSVFPHRSNVIWEIKVLRKE

SMELVKLVTHNTTLNGKDTLHHWSWASDMPLECAIHFVEIRCYIDNLHFSGLEEWSDWSP

VKNISWIPDSQTKVFPQDKVILVGSDITFCCVSQEKVLSALIGHTNCPLIHLDGENVAIK

IRNISVSASSGTNVVFTTEDNIFGTVIFAGYPPDTPQQLNCETHDLKEIICSWNPGRVTA

LVGPRATSYTLVESFSGKYVRLKRAEAPTNESYQLLFQMLPNQEIYNFTLNAHNPLGRSQ

STILVNITEKVYPHTPTSFKVKDINSTAVKLSWHLPGNFAKINFLCEIEIKKSNSVQEQR

NVTIKGVENSSYLVALDKLNPYTLYTFRIRCSTETFWKWSKWSNKKQHLTTEASPSKGPD

TWREWSSDGKNLIIYWKPLPINEANGKILSYNVSCSSDEETQSLSEIPDPQHKAEIRLDK

NDYIISVVAKNSVGSSPPSKIASMEIPNDDLKIEQVVGMGKGILLTWHYDPNMTCDYVIK

WCNSSRSEPCLMDWRKVPSNSTETVIESDEFRPGIRYNFFLYGCRNQGYQLLRSMIGYIE

ELAPIVAPNFTVEDTSADSILVKWEDIPVEELRGFLRGYLFYFGKGERDTSKMRVLESGR

SDIKVKNITDISQKTLRIADLQGKTSYHLVLRAYTDGGVGPEKSMYVVTKENSVGLIIAI

LIPVAVAVIVGVVTSILCYRKREWIKETFYPDIPNPENCKALQFQKSVCEGSSALKTLEM

NPCTPNNVEVLETRSAFPKIEDTEIISPVAERPEDRSDAEPENHVVVSYCPPIIEEEIPN

PAADEAGGTAQVIYIDVQSMYQPQAKPEEEQENDPVGGAGYKPQMHLPINSTVEDIAAEE

DLDKTAGYRPQANVNTWNLVSPDSPRSIDSNSEIVSFGSPCSINSRQFLIPPKDEDSPKS

NGGGWSFTNFFQNKPND

>sp|P42702|LIFR_HUMAN 709 YQLLRSMIGYIEELAPIV

MMDIYVCLKRPSWMVDNKRMRTASNFQWLLSTFILLYLMNQVNSQKKGAPHDLKCVTNNL

QVWNCSWKAPSGTGRGTDYEVCIENRSRSCYQLEKTSIKIPALSHGDYEITINSLHDFGS

STSKFTLNEQNVSLIPDTPEILNLSADFSTSTLYLKWNDRGSVFPHRSNVIWEIKVLRKE

SMELVKLVTHNTTLNGKDTLHHWSWASDMPLECAIHFVEIRCYIDNLHFSGLEEWSDWSP

VKNISWIPDSQTKVFPQDKVILVGSDITFCCVSQEKVLSALIGHTNCPLIHLDGENVAIK

IRNISVSASSGTNVVFTTEDNIFGTVIFAGYPPDTPQQLNCETHDLKEIICSWNPGRVTA

LVGPRATSYTLVESFSGKYVRLKRAEAPTNESYQLLFQMLPNQEIYNFTLNAHNPLGRSQ

STILVNITEKVYPHTPTSFKVKDINSTAVKLSWHLPGNFAKINFLCEIEIKKSNSVQEQR

NVTIKGVENSSYLVALDKLNPYTLYTFRIRCSTETFWKWSKWSNKKQHLTTEASPSKGPD

TWREWSSDGKNLIIYWKPLPINEANGKILSYNVSCSSDEETQSLSEIPDPQHKAEIRLDK

NDYIISVVAKNSVGSSPPSKIASMEIPNDDLKIEQVVGMGKGILLTWHYDPNMTCDYVIK

WCNSSRSEPCLMDWRKVPSNSTETVIESDEFRPGIRYNFFLYGCRNQGYQLLRSMIGYIE

ELAPIVAPNFTVEDTSADSILVKWEDIPVEELRGFLRGYLFYFGKGERDTSKMRVLESGR

SDIKVKNITDISQKTLRIADLQGKTSYHLVLRAYTDGGVGPEKSMYVVTKENSVGLIIAI

LIPVAVAVIVGVVTSILCYRKREWIKETFYPDIPNPENCKALQFQKSVCEGSSALKTLEM

NPCTPNNVEVLETRSAFPKIEDTEIISPVAERPEDRSDAEPENHVVVSYCPPIIEEEIPN

PAADEAGGTAQVIYIDVQSMYQPQAKPEEEQENDPVGGAGYKPQMHLPINSTVEDIAAEE

DLDKTAGYRPQANVNTWNLVSPDSPRSIDSNSEIVSFGSPCSINSRQFLIPPKDEDSPKS

NGGGWSFTNFFQNKPND

>sp|Q6S8J3|A26CA_HUMAN 1056 WISKQEYDESGPSIVHRKCF

MVVEVDSMPAASSVKKPFGLRSKMGKWCCRCFPCYRESGKSNVGTSGDHDDSAMKTLRSK

MGKWCHHCFPCCRGSGKSNVGASGDHDDSAMKTLRNKMGKWCCHCFPCCRGSGKSKVGAW

GDYDDSAFMEPRYHVRGEDLDKLHRAAWWGKVPRKDLIVMLRDTDVNKKDKQKRTALHLA

SANGNSEVVKLLLDRRCQLNVLDNKKRTALIKAVQCQEDECALMLLEHGTDPNIPDEYGN

TTLHYAIYNEDKLMAKALLLYGADIESKNKHGLTPLLLGVHEQKQQVVKFLIKKKANLNA

LDRYGRTALILAVCCGSASIVSLLLEQNIDVSSQDLSGQTAREYAVSSHHHVICQLLSDY

KEKQMLKISSENSNPEQELKLTSEEESQRFKGSENSQPEKMSQELEINKDGDREVEEEMK

KHESNNVGLLENLTNGVTAGNGDNGLIPQRKSRTPENQQFPDNESEEYHRICELLSDYKE

KQMPKYSSENSNPEQDLKLTSEEESQRLKGSENGQPEKRSQEPEINKDGDRELENFMAIE

EMKKHGSTHVGFPENLTNGATAGNGDDGLIPPRKSRTPESQQFPDTENEEYHSDEQNDTQ

KQFCEEQNTGILHDEILIHEEKQIEVVEKMNSELSLSCKKEKDVLHENSTLREEIAMLRL

ELDTMKHQSQLREKKYLEDIESVKKKNDNLLKALQLNELTMDDDTAVLVIDNGSGMCKAG

FAGDDAPRAVFPSIVGRPRQQGMMGGMHQKESYVGKEAQSKRGILTLKYPMEHGIITNWD

DMEKIWHHTFYNELRVAPEEHPILLTEAPLNPKANREKMTQIMFETFNTPAMYVAIQAVP

SLYTSGRTTGIVMDSGDGVTHTVPIYEGNALPHATLRLDLAGRELPDYLMKILTERGYRF

TTMAEREIVRDIKEKLCYVALDFEQEMATAASSSSLEKSYELPDGQVITIGNERFRCPEA

LFQPCFLGMESCGIHETTFNSIMKSDVDIRKDLYTNTVLSGGTTMYPGMAHRMQKEIAAL

APSMMKIRIIAPPKRKYSVWVGGSILASLSTFQQMWISKQEYDESGPSIVHRKCF

>sp|P08648|ITA5_HUMAN 601 LSPIHIALNFSLDPQAPVDSHGLRPALHYQ

MGSRTPESPLHAVQLRWGPRRRPPLLPLLLLLLPPPPRVGGFNLDAEAPAVLSGPPGSFF

GFSVEFYRPGTDGVSVLVGAPKANTSQPGVLQGGAVYLCPWGASPTQCTPIEFDSKGSRL

LESSLSSSEGEEPVEYKSLQWFGATVRAHGSSILACAPLYSWRTEKEPLSDPVGTCYLST

DNFTRILEYAPCRSDFSWAAGQGYCQGGFSAEFTKTGRVVLGGPGSYFWQGQILSATQEQ

IAESYYPEYLINLVQGQLQTRQASSIYDDSYLGYSVAVGEFSGDDTEDFVAGVPKGNLTY

GYVTILNGSDIRSLYNFSGEQMASYFGYAVAATDVNGDGLDDLLVGAPLLMDRTPDGRPQ

EVGRVYVYLQHPAGIEPTPTLTLTGHDEFGRFGSSLTPLGDLDQDGYNDVAIGAPFGGET

QQGVVFVFPGGPGGLGSKPSQVLQPLWAASHTPDFFGSALRGGRDLDGNGYPDLIVGSFG

VDKAVVYRGRPIVSASASLTIFPAMFNPEERSCSLEGNPVACINLSFCLNASGKHVADSI

GFTVELQLDWQKQKGGVRRALFLASRQATLTQTLLIQNGAREDCREMKIYLRNESEFRDK

LSPIHIALNFSLDPQAPVDSHGLRPALHYQSKSRIEDKAQILLDCGEDNICVPDLQLEVF

GEQNHVYLGDKNALNLTFHAQNVGEGGAYEAELRVTAPPEAEYSGLVRHPGNFSSLSCDY

FAVNQSRLLVCDLGNPMKAGASLWGGLRFTVPHLRDTKKTIQFDFQILSKNLNNSQSDVV

SFRLSVEAQAQVTLNGVSKPEAVLFPVSDWHPRDQPQKEEDLGPAVHHVYELINQGPSSI

SQGVLELSCPQALEGQQLLYVTRVTGLNCTTNHPINPKGLELDPEGSLHHQQKREAPSRS

SASSGPQILKCPEAECFRLRCELGPLHQQESQSLQLHFRVWAKTFLQREHQPFSLQCEAV

YKALKMPYRILPRQLPQKERQVATAVQWTKAEGSYGVPLWIIILAILFGLLLLGLLIYIL

YKLGFFKRSLPYGTAMEKAQLKPPATSDA

>sp|P13612|ITA4_HUMAN 229 GSLFVYNITTNKYKAFLDKQ

MFPTESAWLGKRGANPGPEAAVRETVMLLLCLGVPTGRPYNVDTESALLYQGPHNTLFGY

SVVLHSHGANRWLLVGAPTANWLANASVINPGAIYRCRIGKNPGQTCEQLQLGSPNGEPC

GKTCLEERDNQWLGVTLSRQPGENGSIVTCGHRWKNIFYIKNENKLPTGGCYGVPPDLRT

ELSKRIAPCYQDYVKKFGENFASCQAGISSFYTKDLIVMGAPGSSYWTGSLFVYNITTNK

YKAFLDKQNQVKFGSYLGYSVGAGHFRSQHTTEVVGGAPQHEQIGKAYIFSIDEKELNIL

HEMKGKKLGSYFGASVCAVDLNADGFSDLLVGAPMQSTIREEGRVFVYINSGSGAVMNAM

ETNLVGSDKYAARFGESIVNLGDIDNDGFEDVAIGAPQEDDLQGAIYIYNGRADGISSTF

SQRIEGLQISKSLSMFGQSISGQIDADNNGYVDVAVGAFRSDSAVLLRTRPVVIVDASLS

HPESVNRTKFDCVENGWPSVCIDLTLCFSYKGKEVPGYIVLFYNMSLDVNRKAESPPRFY

FSSNGTSDVITGSIQVSSREANCRTHQAFMRKDVRDILTPIQIEAAYHLGPHVISKRSTE

EFPPLQPILQQKKEKDIMKKTINFARFCAHENCSADLQVSAKIGFLKPHENKTYLAVGSM

KTLMLNVSLFNAGDDAYETTLHVKLPVGLYFIKILELEEKQINCEVTDNSGVVQLDCSIG

YIYVDHLSRIDISFLLDVSSLSRAEEDLSITVHATCENEEEMDNLKHSRVTVAIPLKYEV

KLTVHGFVNPTSFVYGSNDENEPETCMVEKMNLTFHVINTGNSMAPNVSVEIMVPNSFSP

QTDKLFNILDVQTTTGECHFENYQRVCALEQQKSAMQTLKGIVRFLSKTDKRLLYCIKAD

PHCLNFLCNFGKMESGKEASVHIQLEGRPSILEMDETSALKFEIRATGFPEPNPRVIELN

KDENVAHVLLEGLHHQRPKRYFTIVIISSSLLLGLIVLLLISYVMWKAGFFKRQYKSILQ

EENRRDSWSYINSKSNDD

>sp|P13612|ITA4_HUMAN 730 IDISFLLDVSSLSRAEE

MFPTESAWLGKRGANPGPEAAVRETVMLLLCLGVPTGRPYNVDTESALLYQGPHNTLFGY

SVVLHSHGANRWLLVGAPTANWLANASVINPGAIYRCRIGKNPGQTCEQLQLGSPNGEPC

GKTCLEERDNQWLGVTLSRQPGENGSIVTCGHRWKNIFYIKNENKLPTGGCYGVPPDLRT

ELSKRIAPCYQDYVKKFGENFASCQAGISSFYTKDLIVMGAPGSSYWTGSLFVYNITTNK

YKAFLDKQNQVKFGSYLGYSVGAGHFRSQHTTEVVGGAPQHEQIGKAYIFSIDEKELNIL

HEMKGKKLGSYFGASVCAVDLNADGFSDLLVGAPMQSTIREEGRVFVYINSGSGAVMNAM

ETNLVGSDKYAARFGESIVNLGDIDNDGFEDVAIGAPQEDDLQGAIYIYNGRADGISSTF

SQRIEGLQISKSLSMFGQSISGQIDADNNGYVDVAVGAFRSDSAVLLRTRPVVIVDASLS

HPESVNRTKFDCVENGWPSVCIDLTLCFSYKGKEVPGYIVLFYNMSLDVNRKAESPPRFY

FSSNGTSDVITGSIQVSSREANCRTHQAFMRKDVRDILTPIQIEAAYHLGPHVISKRSTE

EFPPLQPILQQKKEKDIMKKTINFARFCAHENCSADLQVSAKIGFLKPHENKTYLAVGSM

KTLMLNVSLFNAGDDAYETTLHVKLPVGLYFIKILELEEKQINCEVTDNSGVVQLDCSIG

YIYVDHLSRIDISFLLDVSSLSRAEEDLSITVHATCENEEEMDNLKHSRVTVAIPLKYEV

KLTVHGFVNPTSFVYGSNDENEPETCMVEKMNLTFHVINTGNSMAPNVSVEIMVPNSFSP

QTDKLFNILDVQTTTGECHFENYQRVCALEQQKSAMQTLKGIVRFLSKTDKRLLYCIKAD

PHCLNFLCNFGKMESGKEASVHIQLEGRPSILEMDETSALKFEIRATGFPEPNPRVIELN

KDENVAHVLLEGLHHQRPKRYFTIVIISSSLLLGLIVLLLISYVMWKAGFFKRQYKSILQ

EENRRDSWSYINSKSNDD

>sp|Q96T76|MMS19_HUMAN 191 LVAFRIVHDLISRDYS

MAAAAAVEAAAPMGALWGLVHDFVVGQQEGPADQVAADVKSGNYTVLQVVEALGSSLENP

EPRTRARAIQLLSQVLLHCHTLLLEKEVVHLILFYENRLKDHHLVIPSVLQGLKALSLCV

ALPPGLAVSVLKAIFQEVHVQSLPQVDRHTVYNIITNFMRTREEELKSLGADFTFGFIQV

MDGEKDPRNLLVAFRIVHDLISRDYSLGPFVEELFEVTSCYFPIDFTPPPNDPHGIQRED

LILSLRAVLASTPRFAEFLLPLLIEKVDSEVLSAKLDSLQTLNACCAVYGQKELKDFLPS

LWASIRREVFQTASERVEAEGLAALHSLTACLSRSVLRADAEDLLDSFLSNILQDCRHHL

CEPDMKLVWPSAKLLQAAAGASARACDSVTSNVLPLLLEQFHKHSQSSQRRTILEMLLGF

LKLQQKWSYEDKDQRPLNGFKDQLCSLVFMALTDPSTQLQLVGIRTLTVLGAQPDLLSYE

DLELAVGHLYRLSFLKEDSQSCRVAALEASGTLAALYPVAFSSHLVPKLAEELRVGESNL

TNGDEPTQCSRHLCCLQALSAVSTHPSIVKETLPLLLQHLWQVNRGNMVAQSSDVIAVCQ

SLRQMAEKCQQDPESCWYFHQTAIPCLLALAVQASMPEKEPSVLRKVLLEDEVLAAMVSV

IGTATTHLSPELAAQSVTHIVPLFLDGNVSFLPENSFPSRFQPFQDGSSGQRRLIALLMA

FVCSLPRNVEIPQLNQLMRELLELSCCHSCPFSSTAAAKCFAGLLNKHPAGQQLDEFLQL

AVDKVEAGLGSGPCRSQAFTLLLWVTKALVLRYHPLSSCLTARLMGLLSDPELGPAAADG

FSLLMSDCTDVLTRAGHAEVRIMFRQRFFTDNVPALVQGFHAAPQDVKPNYLKGLSHVLN

RLPKPVLLPELPTLLSLLLEALSCPDCVVQLSTLSCLQPLLLEAPQVMSLHVDTLVTKFL

NLSSSPSMAVRIAALQCMHALTRLPTPVLLPYKPQVIRALAKPLDDKKRLVRKEAVSARG

EWFLLGSPGS

>sp|Q9UIQ6|LCAP_HUMAN 902 DVRKLYWLMKSSLNGDN

MEPFTNDRLQLPRNMIENSMFEEEPDVVDLAKEPCLHPLEPDEVEYEPRGSRLLVRGLGE

HEMEEDEEDYESSAKLLGMSFMNRSSGLRNSATGYRQSPDGACSVPSARTMVVCAFVIVV

AVSVIMVIYLLPRCTFTKEGCHKKNQSIGLIQPFATNGKLFPWAQIRLPTAVVPLRYELS

LHPNLTSMTFRGSVTISVQALQVTWNIILHSTGHNISRVTFMSAVSSQEKQAEILEYAYH

GQIAIVAPEALLAGHNYTLKIEYSANISSSYYGFYGFSYTDESNEKKYFAATQFEPLAAR

SAFPCFDEPAFKATFIIKIIRDEQYTALSNMPKKSSVVLDDGLVQDEFSESVKMSTYLVA

FIVGEMKNLSQDVNGTLVSIYAVPEKIGQVHYALETTVKLLEFFQNYFEIQYPLKKLDLV

AIPDFEAGAMENWGLLTFREETLLYDSNTSSMADRKLVTKIIAHELAHQWFGNLVTMKWW

NDLWLNEGFATFMEYFSLEKIFKELSSYEDFLDARFKTMKKDSLNSSHPISSSVQSSEQI

EEMFDSLSYFKGSSLLLMLKTYLSEDVFQHAVVLYLHNHSYASIQSDDLWDSFNEVTNQT

LDVKRMMKTWTLQKGFPLVTVQKKGKELFIQQERFFLNMKPEIQPSDTSYLWHIPLSYVT

EGRNYSKYQSVSLLDKKSGVINLTEEVLWVKVNINMNGYYIVHYADDDWEALIHQLKINP

YVLSDKDRANLINNIFELAGLGKVPLKRAFDLINYLGNENHTAPITEALFQTDLIYNLLE

KLGYMDLASRLVTRVFKLLQNQIQQQTWTDEGTPSMRELRSALLEFACTHNLGNCSTTAM

KLFDDWMASNGTQSLPTDVMTTVFKVGAKTDKGWSFLLGKYISIGSEAEKNKILEALASS

EDVRKLYWLMKSSLNGDNFRTQKLSFIIRTVGRHFPGHLLAWDFVKENWNKLVQKFPLGS

YTIQNIVAGSTYLFSTKTHLSEVQAFFENQSEATFRLRCVQEALEVIQLNIQWMEKNLKS

LTWWL

>sp|Q9UIQ6|LCAP_HUMAN 710 EALIHQLKINPYVLS

MEPFTNDRLQLPRNMIENSMFEEEPDVVDLAKEPCLHPLEPDEVEYEPRGSRLLVRGLGE

HEMEEDEEDYESSAKLLGMSFMNRSSGLRNSATGYRQSPDGACSVPSARTMVVCAFVIVV

AVSVIMVIYLLPRCTFTKEGCHKKNQSIGLIQPFATNGKLFPWAQIRLPTAVVPLRYELS

LHPNLTSMTFRGSVTISVQALQVTWNIILHSTGHNISRVTFMSAVSSQEKQAEILEYAYH

GQIAIVAPEALLAGHNYTLKIEYSANISSSYYGFYGFSYTDESNEKKYFAATQFEPLAAR

SAFPCFDEPAFKATFIIKIIRDEQYTALSNMPKKSSVVLDDGLVQDEFSESVKMSTYLVA

FIVGEMKNLSQDVNGTLVSIYAVPEKIGQVHYALETTVKLLEFFQNYFEIQYPLKKLDLV

AIPDFEAGAMENWGLLTFREETLLYDSNTSSMADRKLVTKIIAHELAHQWFGNLVTMKWW

NDLWLNEGFATFMEYFSLEKIFKELSSYEDFLDARFKTMKKDSLNSSHPISSSVQSSEQI

EEMFDSLSYFKGSSLLLMLKTYLSEDVFQHAVVLYLHNHSYASIQSDDLWDSFNEVTNQT

LDVKRMMKTWTLQKGFPLVTVQKKGKELFIQQERFFLNMKPEIQPSDTSYLWHIPLSYVT

EGRNYSKYQSVSLLDKKSGVINLTEEVLWVKVNINMNGYYIVHYADDDWEALIHQLKINP

YVLSDKDRANLINNIFELAGLGKVPLKRAFDLINYLGNENHTAPITEALFQTDLIYNLLE

KLGYMDLASRLVTRVFKLLQNQIQQQTWTDEGTPSMRELRSALLEFACTHNLGNCSTTAM

KLFDDWMASNGTQSLPTDVMTTVFKVGAKTDKGWSFLLGKYISIGSEAEKNKILEALASS

EDVRKLYWLMKSSLNGDNFRTQKLSFIIRTVGRHFPGHLLAWDFVKENWNKLVQKFPLGS

YTIQNIVAGSTYLFSTKTHLSEVQAFFENQSEATFRLRCVQEALEVIQLNIQWMEKNLKS

LTWWL

>sp|P05023|AT1A1_HUMAN 256 GIVVYTGDRTVMGRI

MGKGVGRDKYEPAAVSEQGDKKGKKGKKDRDMDELKKEVSMDDHKLSLDELHRKYGTDLS

RGLTSARAAEILARDGPNALTPPPTTPEWIKFCRQLFGGFSMLLWIGAILCFLAYSIQAA

TEEEPQNDNLYLGVVLSAVVIITGCFSYYQEAKSSKIMESFKNMVPQQALVIRNGEKMSI

NAEEVVVGDLVEVKGGDRIPADLRIISANGCKVDNSSLTGESEPQTRSPDFTNENPLETR

NIAFFSTNCVEGTARGIVVYTGDRTVMGRIATLASGLEGGQTPIAAEIEHFIHIITGVAV

FLGVSFFILSLILEYTWLEAVIFLIGIIVANVPEGLLATVTVCLTLTAKRMARKNCLVKN

LEAVETLGSTSTICSDKTGTLTQNRMTVAHMWFDNQIHEADTTENQSGVSFDKTSATWLA

LSRIAGLCNRAVFQANQENLPILKRAVAGDASESALLKCIELCCGSVKEMRERYAKIVEI

PFNSTNKYQLSIHKNPNTSEPQHLLVMKGAPERILDRCSSILLHGKEQPLDEELKDAFQN

AYLELGGLGERVLGFCHLFLPDEQFPEGFQFDTDDVNFPIDNLCFVGLISMIDPPRAAVP

DAVGKCRSAGIKVIMVTGDHPITAKAIAKGVGIISEGNETVEDIAARLNIPVSQVNPRDA

KACVVHGSDLKDMTSEQLDDILKYHTEIVFARTSPQQKLIIVEGCQRQGAIVAVTGDGVN

DSPALKKADIGVAMGIAGSDVSKQAADMILLDDNFASIVTGVEEGRLIFDNLKKSIAYTL

TSNIPEITPFLIFIIANIPLPLGTVTILCIDLGTDMVPAISLAYEQAESDIMKRQPRNPK

TDKLVNERLISMAYGQIGMIQALGGFFTYFVILAENGFLPIHLLGLRVDWDDRWINDVED

SYGQQWTYEQRKIVEFTCHTAFFVSIVVVQWADLVICKTRRNSVFQQGMKNKILIFGLFE

ETALAAFLSYCPGMGVALRMYPLKPTWWFCAFPYSLLIFVYDEVRKLIIRRRPGGWVEKE

TYY

>sp|P05023|AT1A1_HUMAN 256 GIVVYTGDRTVMGRIA

MGKGVGRDKYEPAAVSEQGDKKGKKGKKDRDMDELKKEVSMDDHKLSLDELHRKYGTDLS

RGLTSARAAEILARDGPNALTPPPTTPEWIKFCRQLFGGFSMLLWIGAILCFLAYSIQAA

TEEEPQNDNLYLGVVLSAVVIITGCFSYYQEAKSSKIMESFKNMVPQQALVIRNGEKMSI

NAEEVVVGDLVEVKGGDRIPADLRIISANGCKVDNSSLTGESEPQTRSPDFTNENPLETR

NIAFFSTNCVEGTARGIVVYTGDRTVMGRIATLASGLEGGQTPIAAEIEHFIHIITGVAV

FLGVSFFILSLILEYTWLEAVIFLIGIIVANVPEGLLATVTVCLTLTAKRMARKNCLVKN

LEAVETLGSTSTICSDKTGTLTQNRMTVAHMWFDNQIHEADTTENQSGVSFDKTSATWLA

LSRIAGLCNRAVFQANQENLPILKRAVAGDASESALLKCIELCCGSVKEMRERYAKIVEI

PFNSTNKYQLSIHKNPNTSEPQHLLVMKGAPERILDRCSSILLHGKEQPLDEELKDAFQN

AYLELGGLGERVLGFCHLFLPDEQFPEGFQFDTDDVNFPIDNLCFVGLISMIDPPRAAVP

DAVGKCRSAGIKVIMVTGDHPITAKAIAKGVGIISEGNETVEDIAARLNIPVSQVNPRDA

KACVVHGSDLKDMTSEQLDDILKYHTEIVFARTSPQQKLIIVEGCQRQGAIVAVTGDGVN

DSPALKKADIGVAMGIAGSDVSKQAADMILLDDNFASIVTGVEEGRLIFDNLKKSIAYTL

TSNIPEITPFLIFIIANIPLPLGTVTILCIDLGTDMVPAISLAYEQAESDIMKRQPRNPK

TDKLVNERLISMAYGQIGMIQALGGFFTYFVILAENGFLPIHLLGLRVDWDDRWINDVED

SYGQQWTYEQRKIVEFTCHTAFFVSIVVVQWADLVICKTRRNSVFQQGMKNKILIFGLFE

ETALAAFLSYCPGMGVALRMYPLKPTWWFCAFPYSLLIFVYDEVRKLIIRRRPGGWVEKE

TYY

>sp|P05023|AT1A1_HUMAN 199 IPADLRIISANGCK

MGKGVGRDKYEPAAVSEQGDKKGKKGKKDRDMDELKKEVSMDDHKLSLDELHRKYGTDLS

RGLTSARAAEILARDGPNALTPPPTTPEWIKFCRQLFGGFSMLLWIGAILCFLAYSIQAA

TEEEPQNDNLYLGVVLSAVVIITGCFSYYQEAKSSKIMESFKNMVPQQALVIRNGEKMSI

NAEEVVVGDLVEVKGGDRIPADLRIISANGCKVDNSSLTGESEPQTRSPDFTNENPLETR

NIAFFSTNCVEGTARGIVVYTGDRTVMGRIATLASGLEGGQTPIAAEIEHFIHIITGVAV

FLGVSFFILSLILEYTWLEAVIFLIGIIVANVPEGLLATVTVCLTLTAKRMARKNCLVKN

LEAVETLGSTSTICSDKTGTLTQNRMTVAHMWFDNQIHEADTTENQSGVSFDKTSATWLA

LSRIAGLCNRAVFQANQENLPILKRAVAGDASESALLKCIELCCGSVKEMRERYAKIVEI

PFNSTNKYQLSIHKNPNTSEPQHLLVMKGAPERILDRCSSILLHGKEQPLDEELKDAFQN

AYLELGGLGERVLGFCHLFLPDEQFPEGFQFDTDDVNFPIDNLCFVGLISMIDPPRAAVP

DAVGKCRSAGIKVIMVTGDHPITAKAIAKGVGIISEGNETVEDIAARLNIPVSQVNPRDA

KACVVHGSDLKDMTSEQLDDILKYHTEIVFARTSPQQKLIIVEGCQRQGAIVAVTGDGVN

DSPALKKADIGVAMGIAGSDVSKQAADMILLDDNFASIVTGVEEGRLIFDNLKKSIAYTL

TSNIPEITPFLIFIIANIPLPLGTVTILCIDLGTDMVPAISLAYEQAESDIMKRQPRNPK

TDKLVNERLISMAYGQIGMIQALGGFFTYFVILAENGFLPIHLLGLRVDWDDRWINDVED

SYGQQWTYEQRKIVEFTCHTAFFVSIVVVQWADLVICKTRRNSVFQQGMKNKILIFGLFE

ETALAAFLSYCPGMGVALRMYPLKPTWWFCAFPYSLLIFVYDEVRKLIIRRRPGGWVEKE

TYY

>sp|P05023|AT1A1_HUMAN 257 IVVYTGDRTVMGRIA

MGKGVGRDKYEPAAVSEQGDKKGKKGKKDRDMDELKKEVSMDDHKLSLDELHRKYGTDLS

RGLTSARAAEILARDGPNALTPPPTTPEWIKFCRQLFGGFSMLLWIGAILCFLAYSIQAA

TEEEPQNDNLYLGVVLSAVVIITGCFSYYQEAKSSKIMESFKNMVPQQALVIRNGEKMSI

NAEEVVVGDLVEVKGGDRIPADLRIISANGCKVDNSSLTGESEPQTRSPDFTNENPLETR

NIAFFSTNCVEGTARGIVVYTGDRTVMGRIATLASGLEGGQTPIAAEIEHFIHIITGVAV

FLGVSFFILSLILEYTWLEAVIFLIGIIVANVPEGLLATVTVCLTLTAKRMARKNCLVKN

LEAVETLGSTSTICSDKTGTLTQNRMTVAHMWFDNQIHEADTTENQSGVSFDKTSATWLA

LSRIAGLCNRAVFQANQENLPILKRAVAGDASESALLKCIELCCGSVKEMRERYAKIVEI

PFNSTNKYQLSIHKNPNTSEPQHLLVMKGAPERILDRCSSILLHGKEQPLDEELKDAFQN

AYLELGGLGERVLGFCHLFLPDEQFPEGFQFDTDDVNFPIDNLCFVGLISMIDPPRAAVP

DAVGKCRSAGIKVIMVTGDHPITAKAIAKGVGIISEGNETVEDIAARLNIPVSQVNPRDA

KACVVHGSDLKDMTSEQLDDILKYHTEIVFARTSPQQKLIIVEGCQRQGAIVAVTGDGVN

DSPALKKADIGVAMGIAGSDVSKQAADMILLDDNFASIVTGVEEGRLIFDNLKKSIAYTL

TSNIPEITPFLIFIIANIPLPLGTVTILCIDLGTDMVPAISLAYEQAESDIMKRQPRNPK

TDKLVNERLISMAYGQIGMIQALGGFFTYFVILAENGFLPIHLLGLRVDWDDRWINDVED

SYGQQWTYEQRKIVEFTCHTAFFVSIVVVQWADLVICKTRRNSVFQQGMKNKILIFGLFE

ETALAAFLSYCPGMGVALRMYPLKPTWWFCAFPYSLLIFVYDEVRKLIIRRRPGGWVEKE

TYY

>sp|P05023|AT1A1_HUMAN 257 IVVYTGDRTVMGRIAT

MGKGVGRDKYEPAAVSEQGDKKGKKGKKDRDMDELKKEVSMDDHKLSLDELHRKYGTDLS

RGLTSARAAEILARDGPNALTPPPTTPEWIKFCRQLFGGFSMLLWIGAILCFLAYSIQAA

TEEEPQNDNLYLGVVLSAVVIITGCFSYYQEAKSSKIMESFKNMVPQQALVIRNGEKMSI

NAEEVVVGDLVEVKGGDRIPADLRIISANGCKVDNSSLTGESEPQTRSPDFTNENPLETR

NIAFFSTNCVEGTARGIVVYTGDRTVMGRIATLASGLEGGQTPIAAEIEHFIHIITGVAV

FLGVSFFILSLILEYTWLEAVIFLIGIIVANVPEGLLATVTVCLTLTAKRMARKNCLVKN

LEAVETLGSTSTICSDKTGTLTQNRMTVAHMWFDNQIHEADTTENQSGVSFDKTSATWLA

LSRIAGLCNRAVFQANQENLPILKRAVAGDASESALLKCIELCCGSVKEMRERYAKIVEI

PFNSTNKYQLSIHKNPNTSEPQHLLVMKGAPERILDRCSSILLHGKEQPLDEELKDAFQN

AYLELGGLGERVLGFCHLFLPDEQFPEGFQFDTDDVNFPIDNLCFVGLISMIDPPRAAVP

DAVGKCRSAGIKVIMVTGDHPITAKAIAKGVGIISEGNETVEDIAARLNIPVSQVNPRDA

KACVVHGSDLKDMTSEQLDDILKYHTEIVFARTSPQQKLIIVEGCQRQGAIVAVTGDGVN

DSPALKKADIGVAMGIAGSDVSKQAADMILLDDNFASIVTGVEEGRLIFDNLKKSIAYTL

TSNIPEITPFLIFIIANIPLPLGTVTILCIDLGTDMVPAISLAYEQAESDIMKRQPRNPK

TDKLVNERLISMAYGQIGMIQALGGFFTYFVILAENGFLPIHLLGLRVDWDDRWINDVED

SYGQQWTYEQRKIVEFTCHTAFFVSIVVVQWADLVICKTRRNSVFQQGMKNKILIFGLFE

ETALAAFLSYCPGMGVALRMYPLKPTWWFCAFPYSLLIFVYDEVRKLIIRRRPGGWVEKE

TYY

>sp|P05023|AT1A1_HUMAN 656 NPRDAKACVVHGSDLK

MGKGVGRDKYEPAAVSEQGDKKGKKGKKDRDMDELKKEVSMDDHKLSLDELHRKYGTDLS

RGLTSARAAEILARDGPNALTPPPTTPEWIKFCRQLFGGFSMLLWIGAILCFLAYSIQAA

TEEEPQNDNLYLGVVLSAVVIITGCFSYYQEAKSSKIMESFKNMVPQQALVIRNGEKMSI

NAEEVVVGDLVEVKGGDRIPADLRIISANGCKVDNSSLTGESEPQTRSPDFTNENPLETR

NIAFFSTNCVEGTARGIVVYTGDRTVMGRIATLASGLEGGQTPIAAEIEHFIHIITGVAV

FLGVSFFILSLILEYTWLEAVIFLIGIIVANVPEGLLATVTVCLTLTAKRMARKNCLVKN

LEAVETLGSTSTICSDKTGTLTQNRMTVAHMWFDNQIHEADTTENQSGVSFDKTSATWLA

LSRIAGLCNRAVFQANQENLPILKRAVAGDASESALLKCIELCCGSVKEMRERYAKIVEI

PFNSTNKYQLSIHKNPNTSEPQHLLVMKGAPERILDRCSSILLHGKEQPLDEELKDAFQN

AYLELGGLGERVLGFCHLFLPDEQFPEGFQFDTDDVNFPIDNLCFVGLISMIDPPRAAVP

DAVGKCRSAGIKVIMVTGDHPITAKAIAKGVGIISEGNETVEDIAARLNIPVSQVNPRDA

KACVVHGSDLKDMTSEQLDDILKYHTEIVFARTSPQQKLIIVEGCQRQGAIVAVTGDGVN

DSPALKKADIGVAMGIAGSDVSKQAADMILLDDNFASIVTGVEEGRLIFDNLKKSIAYTL

TSNIPEITPFLIFIIANIPLPLGTVTILCIDLGTDMVPAISLAYEQAESDIMKRQPRNPK

TDKLVNERLISMAYGQIGMIQALGGFFTYFVILAENGFLPIHLLGLRVDWDDRWINDVED

SYGQQWTYEQRKIVEFTCHTAFFVSIVVVQWADLVICKTRRNSVFQQGMKNKILIFGLFE

ETALAAFLSYCPGMGVALRMYPLKPTWWFCAFPYSLLIFVYDEVRKLIIRRRPGGWVEKE

TYY

>sp|Q96M86|DNHD1_HUMAN 305 LLQKLILWRVL

MEKVLGCELLKGLNVLDLGLNMEILEEQMLHEILCREYPELETRWQDLKIRALDTCKAVE

AAEERLLTMLLFQNPKRQKPAKFLRNIVRAQGKLCQLRAHCEELEGQKLQEMVLWAPYRP

VVWHGMAMVKALSQLQNLLPLFCMSPENWLAVTKQALDSMKPREINHGEDLASHLLQLRA

HLTRQLLGSTVTALGLTQVPLVGALGALALLQATGKASELERLALWPGLAASPSTVHSKP

VSDVARPAWLGPKAWHECEMLELLPPFVGLCASLAGHSSAWQAYLSLSSTVLGPAPGPGP

EPLSLLQKLILWRVLRPECLAGALADFTTSLLGRPLDENTYAPTMPFKHSQATQPMLILL

PPPGHPSATLHPLTVIQKLAAKYQQGQKQLQVIALGSEAWDPVSVVVSTLSQAMYEGHWL

VLDNCHLMPHWPKELLQLLLELLGRAKVVADLESEQLLDQPESRNVSTVHRDFRLWLIVP

AESSASLPAVLTQHSMPVFWNQSLELGHVLIDSVELAQQVLYMQPPTQALPLLLLHGLLL

HRQLYGTRLQAHRGRWSQVTLTQVLQTQDQLWASLSNPRAAMQELAASVFYGGPLGDTED

REALISLTQACLSPSSGSWVQPHTPQSLLATLMPLPELRELDAMAECKAQMHLLPSPPEP

RLCGLSEGPQAWLLRRQSRALLSALQRSSPVWVPESRRGAQLAERRLRQRLVQVNRRLES

LQDLLTHVIRQDESDAPWSVLGPNARRPLEGVLETEALELSQLVGTLQRDLDCLLQQLKG

APPCPSRRCAAVAHALWTGRLPLPWRPHAPAGPQPPWHWLRQLSRRGQLLVRYLGVGADA

SSDVPERVFHLSAFRHPRRLLLALRGEAALDQNVPSSNFPGSRGSVSSQLQYKRLEMNSN

PLHFRVENGPNPTVPERGLLLIGLQVLHAEWDPIAGALQDSPSSQPSPLPPVSISTQAPG

TSDLPAPADLTVYSCPVYMGGPLGTAKLQSRNIVMHLPLPTKLTPNTCVQRRVHVCSPPL

S

>sp|O00754|MA2B1_HUMAN 560 HPPELLFSASLPALGFST

MGAYARASGVCARGCLDSAGPWTMSRALRPPLPPLCFFLLLLAAAGARAGGYETCPTVQP

NMLNVHLLPHTHDDVGWLKTVDQYFYGIKNDIQHAGVQYILDSVISALLADPTRRFIYVE

IAFFSRWWHQQTNATQEVVRDLVRQGRLEFANGGWVMNDEAATHYGAIVDQMTLGLRFLE

DTFGNDGRPRVAWHIDPFGHSREQASLFAQMGFDGFFFGRLDYQDKWVRMQKLEMEQVWR

ASTSLKPPTADLFTGVLPNGYNPPRNLCWDVLCVDQPLVEDPRSPEYNAKELVDYFLNVA

TAQGRYYRTNHTVMTMGSDFQYENANMWFKNLDKLIRLVNAQQAKGSSVHVLYSTPACYL

WELNKANLTWSVKHDDFFPYADGPHQFWTGYFSSRPALKRYERLSYNFLQVCNQLEALVG

LAANVGPYGSGDSAPLNEAMAVLQHHDAVSGTSRQHVANDYARQLAAGWGPCEVLLSNAL

ARLRGFKDHFTFCQQLNISICPLSQTAARFQVIVYNPLGRKVNWMVRLPVSEGVFVVKDP

NGRTVPSDVVIFPSSDSQAHPPELLFSASLPALGFSTYSVAQVPRWKPQARAPQPIPRRS

WSPALTIENEHIRATFDPDTGLLMEIMNMNQQLLLPVRQTFFWYNASIGDNESDQASGAY

IFRPNQQKPLPVSRWAQIHLVKTPLVQEVHQNFSAWCSQVVRLYPGQRHLELEWSVGPIP

VGDTWGKEVISRFDTPLETKGRFYTDSNGREILERRRDYRPTWKLNQTEPVAGNYYPVNT

RIYITDGNMQLTVLTDRSQGGSSLRDGSLELMVHRRLLKDDGRGVSEPLMENGSGAWVRG

RHLVLLDTAQAAAAGHRLLAEQEVLAPQVVLAPGGGAAYNLGAPPRTQFSGLRRDLPPSV

HLLTLASWGPEMVLLRLEHQFAVGEDSGRNLSAPVTLNLRDLFSTFTITRLQETTLVANQ

LREAASRLKWTTNTGPTPHQTPYQLDPANITLEPMEIRTFLASVQWKEVDG

>sp|O00754|MA2B1_HUMAN 293 VDYFLNVATAQGRYY

MGAYARASGVCARGCLDSAGPWTMSRALRPPLPPLCFFLLLLAAAGARAGGYETCPTVQP

NMLNVHLLPHTHDDVGWLKTVDQYFYGIKNDIQHAGVQYILDSVISALLADPTRRFIYVE

IAFFSRWWHQQTNATQEVVRDLVRQGRLEFANGGWVMNDEAATHYGAIVDQMTLGLRFLE

DTFGNDGRPRVAWHIDPFGHSREQASLFAQMGFDGFFFGRLDYQDKWVRMQKLEMEQVWR

ASTSLKPPTADLFTGVLPNGYNPPRNLCWDVLCVDQPLVEDPRSPEYNAKELVDYFLNVA

TAQGRYYRTNHTVMTMGSDFQYENANMWFKNLDKLIRLVNAQQAKGSSVHVLYSTPACYL

WELNKANLTWSVKHDDFFPYADGPHQFWTGYFSSRPALKRYERLSYNFLQVCNQLEALVG

LAANVGPYGSGDSAPLNEAMAVLQHHDAVSGTSRQHVANDYARQLAAGWGPCEVLLSNAL

ARLRGFKDHFTFCQQLNISICPLSQTAARFQVIVYNPLGRKVNWMVRLPVSEGVFVVKDP

NGRTVPSDVVIFPSSDSQAHPPELLFSASLPALGFSTYSVAQVPRWKPQARAPQPIPRRS

WSPALTIENEHIRATFDPDTGLLMEIMNMNQQLLLPVRQTFFWYNASIGDNESDQASGAY

IFRPNQQKPLPVSRWAQIHLVKTPLVQEVHQNFSAWCSQVVRLYPGQRHLELEWSVGPIP

VGDTWGKEVISRFDTPLETKGRFYTDSNGREILERRRDYRPTWKLNQTEPVAGNYYPVNT

RIYITDGNMQLTVLTDRSQGGSSLRDGSLELMVHRRLLKDDGRGVSEPLMENGSGAWVRG

RHLVLLDTAQAAAAGHRLLAEQEVLAPQVVLAPGGGAAYNLGAPPRTQFSGLRRDLPPSV

HLLTLASWGPEMVLLRLEHQFAVGEDSGRNLSAPVTLNLRDLFSTFTITRLQETTLVANQ

LREAASRLKWTTNTGPTPHQTPYQLDPANITLEPMEIRTFLASVQWKEVDG

>sp|Q86W92|LIPB1_HUMAN 886 MELPDYVLLTAT

MMSDASDMLAAALEQMDGIIAGSKALEYSNGIFDCQSPTSPFMGSLRALHLVEDLRGLLE

MMETDEKEGLRCQIPDSTAETLVEWLQSQMTNGHLPGNGDVYQERLARLENDKESLVLQV

SVLTDQVEAQGEKIRDLEFCLEEHREKVNATEEMLQQELLSRTSLETQKLDLMAEISNLK

LKLTAVEKDRLDYEDKFRDTEGLIQEINDLRLKVSEMDSERLQYEKKLKSTKSLMAKLSS

MKIKVGQMQYEKQRMEQKWESLKDELASLKEQLEEKESEVKRLQEKLVCKMKGEGVEIVD

RDIEVQKMKKAVESLMAANEEKDRKIEDLRQCLNRYKKMQDTVVLAQGKDGEYEELLNSS

SISSLLDAQGFSDLEKSPSPTPVMGSPSCDPFNTSVPEEFHTTILQVSIPSLLPATVSME

TSEKSKLTPKPETSFEENDGNIILGATVDTQLCDKLLTSSLQKSSSLGNLKKETSDGEKE

TIQKTSEDRAPAESRPFGTLPPRPPGQDTSMDDNPFGTRKVRSSFGRGFFKIKSNKRTAS

APNLAETEKETAEHLDLAGASSRPKDSQRNSPFQIPPPSPDSKKKSRGIMKLFGKLRRSQ

STTFNPDDMSEPEFKRGGTRATAGPRLGWSRDLGQSNSDLDMPFAKWTKEQVCNWLMEQG

LGSYLNSGKHWIASGQTLLQASQQDLEKELGIKHSLHRKKLQLALQALGSEEETNHGKLD

FNWVTRWLDDIGLPQYKTQFDEGRVDGRMLHYMTVDDLLSLKVVSVLHHLSIKRAIQVLR

INNFEPNCLRRRPSDENTIAPSEVQKWTNHRVMEWLRSVDLAEYAPNLRGSGVHGGLMVL

EPRFNVETMAQLLNIPPNKTLLRRHLATHFNLLIGAEAQHQKRDAMELPDYVLLTATAKV

KPKKLAFSNFGNLRKKKQEDGEEYVCPMELGQASGSASKKGFKPGLDMRLYEEDDLDRLE

QMEDSEGTVRQIGAFSEGINNLTHMLKEDDMFKDFAARSPSASITDEDSNV

>sp|P32926|DSG3_HUMAN 190 LNSKIAFKIVSQEPA

MMGLFPRTTGALAIFVVVILVHGELRIETKGQYDEEEMTMQQAKRRQKREWVKFAKPCRE

GEDNSKRNPIAKITSDYQATQKITYRISGVGIDQPPFGIFVVDKNTGDINITAIVDREET

PSFLITCRALNAQGLDVEKPLILTVKILDINDNPPVFSQQIFMGEIEENSASNSLVMILN

ATDADEPNHLNSKIAFKIVSQEPAGTPMFLLSRNTGEVRTLTNSLDREQASSYRLVVSGA

DKDGEGLSTQCECNIKVKDVNDNFPMFRDSQYSARIEENILSSELLRFQVTDLDEEYTDN

WLAVYFFTSGNEGNWFEIQTDPRTNEGILKVVKALDYEQLQSVKLSIAVKNKAEFHQSVI

SRYRVQSTPVTIQVINVREGIAFRPASKTFTVQKGISSKKLVDYILGTYQAIDEDTNKAA

SNVKYVMGRNDGGYLMIDSKTAEIKFVKNMNRDSTFIVNKTITAEVLAIDEYTGKTSTGT

VYVRVPDFNDNCPTAVLEKDAVCSSSPSVVVSARTLNNRYTGPYTFALEDQPVKLPAVWS

ITTLNATSALLRAQEQIPPGVYHISLVLTDSQNNRCEMPRSLTLEVCQCDNRGICGTSYP

TTSPGTRYGRPHSGRLGPAAIGLLLLGLLLLLLAPLLLLTCDCGAGSTGGVTGGFIPVPD

GSEGTIHQWGIEGAHPEDKEITNICVPPVTANGADFMESSEVCTNTYARGTAVEGTSGME

MTTKLGAATESGGAAGFATGTVSGAASGFGAATGVGICSSGQSGTMRTRHSTGGTNKDYA

DGAISMNFLDSYFSQKAFACAEEDDGQEANDCLLIYDNEGADATGSPVGSVGCCSFIADD

LDDSFLDSLGPKFKKLAEISLGVDGEGKEVQPPSKDSGYGIESCGHPIEVQQTGFVKCQT

LSGSQGASALSASGSVQPAVSIPDPLQHGNYLVTETYSASGSLVQPSTAGFDPLLTQNVI

VTERVICPISSVPGNLAGPTQLRGSHTMLCTEDPCSRLI

>sp|P38935|SMBP2_HUMAN 68 EPRRYGSAAALPS

MASAAVESFVTKQLDLLELERDAEVEERRSWQENISLKELQSRGVCLLKLQVSSQRTGLY

GRLLVTFEPRRYGSAAALPSNSFTSGDIVGLYDAANEGSQLATGILTRVTQKSVTVAFDE

SHDFQLSLDRENSYRLLKLANDVTYRRLKKALIALKKYHSGPASSLIEVLFGRSAPSPAS

EIHPLTFFNTCLDTSQKEAVSFALSQKELAIIHGPPGTGKTTTVVEIILQAVKQGLKVLC

CAPSNIAVDNLVERLALCKQRILRLGHPARLLESVQQHSLDAVLARSDSAQNVADIRKDI

DQVFVKNKKTQDKREKSNFRNEIKLLRKELKEREEAAMLESLTSANVVLATNTGASADGP

LKLLPESYFDVVVIDECAQALEASCWIPLLKARKCILAGDHKQLPPTTVSHKAALAGLSL

SLMERLAEEYGARVVRTLTVQYRMHQAIMRWASDTMYLGQVTAHSSVARHLLRDLPGVAA

TEETGVPLLLVDTAGCGLFELEEEDEQSKGNPGEVRLVSLHIQALVDAGVPARDIAVVSP

YNLQVDLLRQSLVHRHPELEIKSVDGFQGREKEAVILSFVRSNRKGEVGFLAEDRRINVA

VTRARRHVAVICDSRTVNNHAFLKTLVEYFTQHGEVRTAFEYLDDIVPENYSHENSQGSS

HAATKPQGPATSTRTGSQRQEGGQEAAAPARQGRKKPAGKSLASEAPSQPSLNGGSPEGV

ESQDGVDHFRAMIVEFMASKKMQLEFPPSLNSHDRLRVHQIAEEHGLRHDSSGEGKRRFI

TVSKRAPRPRAALGPPAGTGGPAPLQPVPPTPAQTEQPPREQRGPDQPDLRTLHLERLQR

VRSAQGQPASKEQQASGQQKLPEKKKKKAKGHPATDLPTEEDFEALVSAAVKADNTCGFA

KCTAGVTTLGQFCQLCSRRYCLSHHLPEIHGCGERARAHARQRISREGVLYAGSGTKNGS

LDPAKRAQLQRRLDKKLSELSNQRTSRRKERGT

>sp|Q96PU5|NED4L_HUMAN 561 DGRTFYIDHNSKITQ

MATGLGEPVYGLSEDEGESRILRVKVVSGIDLAKKDIFGASDPYVKLSLYVADENRELAL

VQTKTIKKTLNPKWNEEFYFRVNPSNHRLLFEVFDENRLTRDDFLGQVDVPLSHLPTEDP

TMERPYTFKDFLLRPRSHKSRVKGFLRLKMAYMPKNGGQDEENSDQRDDMEHGWEVVDSN

DSASQHQEELPPPPLPPGWEEKVDNLGRTYYVNHNNRTTQWHRPSLMDVSSESDNNIRQI

NQEAAHRRFRSRRHISEDLEPEPSEGGDVPEPWETISEEVNIAGDSLGLALPPPPASPGS

RTSPQELSEELSRRLQITPDSNGEQFSSLIQREPSSRLRSCSVTDAVAEQGHLPPPSAPA

GRARSSTVTGGEEPTPSVAYVHTTPGLPSGWEERKDAKGRTYYVNHNNRTTTWTRPIMQL

AEDGASGSATNSNNHLIEPQIRRPRSLSSPTVTLSAPLEGAKDSPVRRAVKDTLSNPQSP

QPSPYNSPKPQHKVTQSFLPPGWEMRIAPNGRPFFIDHNTKTTTWEDPRLKFPVHMRSKT

SLNPNDLGPLPPGWEERIHLDGRTFYIDHNSKITQWEDPRLQNPAITGPAVPYSREFKQK

YDYFRKKLKKPADIPNRFEMKLHRNNIFEESYRRIMSVKRPDVLKARLWIEFESEKGLDY

GGVAREWFFLLSKEMFNPYYGLFEYSATDNYTLQINPNSGLCNEDHLSYFTFIGRVAGLA

VFHGKLLDGFFIRPFYKMMLGKQITLNDMESVDSEYYNSLKWILENDPTELDLMFCIDEE

NFGQTYQVDLKPNGSEIMVTNENKREYIDLVIQWRFVNRVQKQMNAFLEGFTELLPIDLI

KIFDENELELLMCGLGDVDVNDWRQHSIYKNGYCPNHPVIQWFWKAVLLMDAEKRIRLLQ

FVTGTSRVPMNGFAELYGSNGPQLFTIEQWGSPEKLPRAHTCFNRLDLPPYETFEDLREK

LLMAVENAQGFEGVD

>sp|Q14596|NBR1_HUMAN 644 SGTQFVCETVIRSLTLD

MEPQVTLNVTFKNEIQSFLVSDPENTTWADIEAMVKVSFDLNTIQIKYLDEENEEVSINS

QGEYEEALKMAVKQGNQLQMQVHEGHHVVDEAPPPVVGAKRLAARAGKKPLAHYSSLVRV

LGSDMKTPEDPAVQSFPLVPCDTDQPQDKPPDWFTSYLETFREQVVNETVEKLEQKLHEK

LVLQNPSLGSCPSEVSMPTSEETLFLPENQFSWHIACNNCQRRIVGVRYQCSLCPSYNIC

EDCEAGPYGHDTNHVLLKLRRPVVGSSEPFCHSKYSTPRLPAALEQVRLQKQVDKNFLKA

EKQRLRAEKKQRKAEVKELKKQLKLHRKIHLWNSIHGLQSPKSPLGRPESLLQSNTLMLP

LQPCTSVMPMLSAAFVDENLPDGTHLQPGTKFIKHWRMKNTGNVKWSADTKLKFMWGNLT

LASTEKKDVLVPCLKAGHVGVVSVEFIAPALEGTYTSHWRLSHKGQQFGPRVWCSIIVDP

FPSEESPDNIEKGMISSSKTDDLTCQQEETFLLAKEERQLGEVTEQTEGTAACIPQKAKN

VASERELYIPSVDLLTAQDLLSFELLDINIVQELERVPHNTPVDVTPCMSPLPHDSPLIE

KPGLGQIEEENEGAGFKALPDSMVSVKRKAENIASVEEAEEDLSGTQFVCETVIRSLTLD

AAPDHNPPCRQKSLQMTFALPEGPLGNEKEEIIHIAEEEAVMEEEEDEEDEEEEDELKDE

VQSQSSASSEDYIIILPECFDTSRPLGDSMYSSALSQPGLERGAEGKPGVEAGQEPAEAG

ERLPGGENQPQEHSISDILTTSQTLETVPLIPEVVELPPSLPRSSPCVHHHGSPGVDLPV

TIPEVSSVPDQIRGEPRGSSGLVNSRQKSYDHSRHHHGSSIAGGLVKGALSVAASAYKAL

FAGPPVTAQPIISEDQTAALMARLFEMGFCDRQLNLRLLKKHNYNILQVVTELLQLNNND

WYSQRY

>sp|Q9BQT9|CSTN3_HUMAN 47 NPPLFALDKDAPLRY

MTLLLLPLLLASLLASCSCNKANKHKPWIEAEYQGIVMENDNTVLLNPPLFALDKDAPLR

YAGEICGFRLHGSGVPFEAVILDKATGEGLIRAKEPVDCEAQKEHTFTIQAYDCGEGPDG

ANTKKSHKATVHVRVNDVNEFAPVFVERLYRAAVTEGKLYDRILRVEAIDGDCSPQYSQI

CYYEILTPNTPFLIDNDGNIENTEKLQYSGERLYKFTVTAYDCGKKRAADDAEVEIQVKP

TCKPSWQGWNKRIEYAPGAGSLALFPGIRLETCDEPLWNIQATIELQTSHVAKGCDRDNY

SERALRKLCGAATGEVDLLPMPGPNANWTAGLSVHYSQDSSLIYWFNGTQAVQVPLGGPS

GLGSGPQDSLSDHFTLSFWMKHGVTPNKGKKEEETIVCNTVQNEDGFSHYSLTVHGCRIA

FLYWPLLESARPVKFLWKLEQVCDDEWHHYALNLEFPTVTLYTDGISFDPALIHDNGLIH

PPRREPALMIGACWTEEKNKEKEKGDNSTDTTQGDPLSIHHYFHGYLAGFSVRSGRLESR

EVIECLYACREGLDYRDFESLGKGMKVHVNPSQSLLTLEGDDVETFNHALQHVAYMNTLR

FATPGVRPLRLTTAVKCFSEESCVSIPEVEGYVVVLQPDAPQILLSGTAHFARPAVDFEG

TNGVPLFPDLQITCSISHQVEAKKDESWQGTVTDTRMSDEIVHNLDGCEISLVGDDLDPE

RESLLLDTTSLQQRGLELTNTSAYLTIAGVESITVYEEILRQARYRLRHGAALYTRKFRL

SCSEMNGRYSSNEFIVEVNVLHSMNRVAHPSHVLSSQQFLHRGHQPPPEMAGHSLASSHR

NSMIPSAATLIIVVCVGFLVLMVVLGLVRIHSLHRRVSGAGGPPGASSDPKDPDLFWDDS

ALTIIVNPMESYQNRQSCVTGAVGGQQEDEDSSDSEVADSPSSDERRIIETPPHRY

>sp|P07202|PERT_HUMAN 632 IDVWLGGLAE

MRALAVLSVTLVMACTEAFFPFISRGKELLWGKPEESRVSSVLEESKRLVDTAMYATMQR

NLKKRGILSPAQLLSFSKLPEPTSGVIARAAEIMETSIQAMKRKVNLKTQQSQHPTDALS

EDLLSIIANMSGCLPYMLPPKCPNTCLANKYRPITGACNNRDHPRWGASNTALARWLPPV

YEDGFSQPRGWNPGFLYNGFPLPPVREVTRHVIQVSNEVVTDDDRYSDLLMAWGQYIDHD

IAFTPQSTSKAAFGGGADCQMTCENQNPCFPIQLPEEARPAAGTACLPFYRSSAACGTGD

QGALFGNLSTANPRQQMNGLTSFLDASTVYGSSPALERQLRNWTSAEGLLRVHARLRDSG

RAYLPFVPPRAPAACAPEPGIPGETRGPCFLAGDGRASEVPSLTALHTLWLREHNRLAAA

LKALNAHWSADAVYQEARKVVGALHQIITLRDYIPRILGPEAFQQYVGPYEGYDSTANPT

VSNVFSTAAFRFGHATIHPLVRRLDASFQEHPDLPGLWLHQAFFSPWTLLRGGGLDPLIR

GLLARPAKLQVQDQLMNEELTERLFVLSNSSTLDLASINLQRGRDHGLPGYNEWREFCGL

PRLETPADLSTAIASRSVADKILDLYKHPDNIDVWLGGLAENFLPRARTGPLFACLIGKQ

MKALRDGDWFWWENSHVFTDAQRRELEKHSLSRVICDNTGLTRVPMDAFQVGKFPEDFES

CDSITGMNLEAWRETFPQDDKCGFPESVENGDFVHCEESGRRVLVYSCRHGYELQGREQL

TCTQEGWDFQPPLCKDVNECADGAHPPCHASARCRNTKGGFQCLCADPYELGDDGRTCVD

SGRLPRVTWISMSLAALLIGGFAGLTSTVICRWTRTGTKSTLPISETGGGTPELRCGKHQ

AVGTSPQRAAAQDSEQESAGMEGRDTHRLPRAL

>sp|P40189|IL6RB_HUMAN 194 IEVWVEAENALGKVT

MLTLQTWVVQALFIFLTTESTGELLDPCGYISPESPVVQLHSNFTAVCVLKEKCMDYFHV

NANYIVWKTNHFTIPKEQYTIINRTASSVTFTDIASLNIQLTCNILTFGQLEQNVYGITI

ISGLPPEKPKNLSCIVNEGKKMRCEWDGGRETHLETNFTLKSEWATHKFADCKAKRDTPT

SCTVDYSTVYFVNIEVWVEAENALGKVTSDHINFDPVYKVKPNPPHNLSVINSEELSSIL

KLTWTNPSIKSVIILKYNIQYRTKDASTWSQIPPEDTASTRSSFTVQDLKPFTEYVFRIR

CMKEDGKGYWSDWSEEASGITYEDRPSKAPSFWYKIDPSHTQGYRTVQLVWKTLPPFEAN

GKILDYEVTLTRWKSHLQNYTVNATKLTVNLTNDRYLATLTVRNLVGKSDAAVLTIPACD

FQATHPVMDLKAFPKDNMLWVEWTTPRESVKKYILEWCVLSDKAPCITDWQQEDGTVHRT

YLRGNLAESKCYLITVTPVYADGPGSPESIKAYLKQAPPSKGPTVRTKKVGKNEAVLEWD

QLPVDVQNGFIRNYTIFYRTIIGNETAVNVDSSHTEYTLSSLTSDTLYMVRMAAYTDEGG

KDGPEFTFTTPKFAQGEIEAIVVPVCLAFLLTTLLGVLFCFNKRDLIKKHIWPNVPDPSK

SHIAQWSPHTPPRHNFNSKDQMYSDGNFTDVSVVEIEANDKKPFPEDLKSLDLFKKEKIN

TEGHSSGIGGSSCMSSSRPSISSSDENESSQNTSSTVQYSTVVHSGYRHQVPSVQVFSRS

ESTQPLLDSEERPEDLQLVDHVDGGDGILPRQQYFKQNCSQHESSPDISHFERSKQVSSV

NEEDFVRLKQQISDHISQSCGSGQMKMFQEVSAADAFGPGTEGQVERFETVGMEAATDEG

MPKSYLPQTVRQGGYMPQ

>sp|Q9P2B2|FPRP_HUMAN 417 VPGFADDPTELACRV

MGRLASRPLLLALLSLALCRGRVVRVPTATLVRVVGTELVIPCNVSDYDGPSEQNFDWSF

SSLGSSFVELASTWEVGFPAQLYQERLQRGEILLRRTANDAVELHIKNVQPSDQGHYKCS

TPSTDATVQGNYEDTVQVKVLADSLHVGPSARPPPSLSLREGEPFELRCTAASASPLHTH

LALLWEVHRGPARRSVLALTHEGRFHPGLGYEQRYHSGDVRLDTVGSDAYRLSVSRALSA

DQGSYRCIVSEWIAEQGNWQEIQEKAVEVATVVIQPSVLRAAVPKNVSVAEGKELDLTCN

ITTDRADDVRPEVTWSFSRMPDSTLPGSRVLARLDRDSLVHSSPHVALSHVDARSYHLLV

RDVSKENSGYYYCHVSLWAPGHNRSWHKVAEAVSSPAGVGVTWLEPDYQVYLNASKVPGF

ADDPTELACRVVDTKSGEANVRFTVSWYYRMNRRSDNVVTSELLAVMDGDWTLKYGERSK

QRAQDGDFIFSKEHTDTFNFRIQRTTEEDRGNYYCVVSAWTKQRNNSWVKSKDVFSKPVN

IFWALEDSVLVVKARQPKPFFAAGNTFEMTCKVSSKNIKSPRYSVLIMAEKPVGDLSSPN

ETKYIISLDQDSVVKLENWTDASRVDGVVLEKVQEDEFRYRMYQTQVSDAGLYRCMVTAW

SPVRGSLWREAATSLSNPIEIDFQTSGPIFNASVHSDTPSVIRGDLIKLFCIITVEGAAL

DPDDMAFDVSWFAVHSFGLDKAPVLLSSLDRKGIVTTSRRDWKSDLSLERVSVLEFLLQV

HGSEDQDFGNYYCSVTPWVKSPTGSWQKEAEIHSKPVFITVKMDVLNAFKYPLLIGVGLS

TVIGLLSCLIGYCSSHWCCKKEVQETRRERRRLMSMEMD

>sp|P01130|LDLR_HUMAN 518 KPRAIVVDPVHGFMY

MGPWGWKLRWTVALLLAAAGTAVGDRCERNEFQCQDGKCISYKWVCDGSAECQDGSDESQ

ETCLSVTCKSGDFSCGGRVNRCIPQFWRCDGQVDCDNGSDEQGCPPKTCSQDEFRCHDGK

CISRQFVCDSDRDCLDGSDEASCPVLTCGPASFQCNSSTCIPQLWACDNDPDCEDGSDEW

PQRCRGLYVFQGDSSPCSAFEFHCLSGECIHSSWRCDGGPDCKDKSDEENCAVATCRPDE

FQCSDGNCIHGSRQCDREYDCKDMSDEVGCVNVTLCEGPNKFKCHSGECITLDKVCNMAR

DCRDWSDEPIKECGTNECLDNNGGCSHVCNDLKIGYECLCPDGFQLVAQRRCEDIDECQD

PDTCSQLCVNLEGGYKCQCEEGFQLDPHTKACKAVGSIAYLFFTNRHEVRKMTLDRSEYT

SLIPNLRNVVALDTEVASNRIYWSDLSQRMICSTQLDRAHGVSSYDTVISRDIQAPDGLA

VDWIHSNIYWTDSVLGTVSVADTKGVKRKTLFRENGSKPRAIVVDPVHGFMYWTDWGTPA

KIKKGGLNGVDIYSLVTENIQWPNGITLDLLSGRLYWVDSKLHSISSIDVNGGNRKTILE

DEKRLAHPFSLAVFEDKVFWTDIINEAIFSANRLTGSDVNLLAENLLSPEDMVLFHNLTQ

PRGVNWCERTTLSNGGCQYLCLPAPQINPHSPKFTCACPDGMLLARDMRSCLTEAEAAVA

TQETSTVRLKVSSTAVRTQHTTTRPVPDTSRLPGATPGLTTVEIVTMSHQALGDVAGRGN

EKKPSSVRALSIVLPIVLLVFLCLGVFLLWKNWRLKNINSINFDNPVYQKTTEDEVHICH

NQDGYSYPSRQMVSLEDDVA

>sp|P13591|NCA11_HUMAN 668 AEYEVYVVAENQQGKSKA

MLQTKDLIWTLFFLGTAVSLQVDIVPSQGEISVGESKFFLCQVAGDAKDKDISWFSPNGE

KLTPNQQRISVVWNDDSSSTLTIYNANIDDAGIYKCVVTGEDGSESEATVNVKIFQKLMF

KNAPTPQEFREGEDAVIVCDVVSSLPPTIIWKHKGRDVILKKDVRFIVLSNNYLQIRGIK

KTDEGTYRCEGRILARGEINFKDIQVIVNVPPTIQARQNIVNATANLGQSVTLVCDAEGF

PEPTMSWTKDGEQIEQEEDDEKYIFSDDSSQLTIKKVDKNDEAEYICIAENKAGEQDATI

HLKVFAKPKITYVENQTAMELEEQVTLTCEASGDPIPSITWRTSTRNISSEEKTLDGHMV

VRSHARVSSLTLKSIQYTDAGEYICTASNTIGQDSQSMYLEVQYAPKLQGPVAVYTWEGN

QVNITCEVFAYPSATISWFRDGQLLPSSNYSNIKIYNTPSASYLEVTPDSENDFGNYNCT

AVNRIGQESLEFILVQADTPSSPSIDQVEPYSSTAQVQFDEPEATGGVPILKYKAEWRAV

GEEVWHSKWYDAKEASMEGIVTIVGLKPETTYAVRLAALNGKGLGEISAASEFKTQPVQG

EPSAPKLEGQMGEDGNSIKVNLIKQDDGGSPIRHYLVRYRALSSEWKPEIRLPSGSDHVM

LKSLDWNAEYEVYVVAENQQGKSKAAHFVFRTSAQPTAIPANGSPTSGLSTGAIVGILIV

IFVLLLVVVDITCYFLNKCGLFMCIAVNLCGKAGPGAKGKDMEEGKAAFSKDESKEPIVE

VRTEEERTPNHDGGKHTEPNETTPLTEPEKGPVEAKPECQETETKPAPAEVKTVPNDATQ

TKENESKA

>sp|P13591|NCA11_HUMAN 667 NAEYEVYVVAENQQGKSKA

MLQTKDLIWTLFFLGTAVSLQVDIVPSQGEISVGESKFFLCQVAGDAKDKDISWFSPNGE

KLTPNQQRISVVWNDDSSSTLTIYNANIDDAGIYKCVVTGEDGSESEATVNVKIFQKLMF

KNAPTPQEFREGEDAVIVCDVVSSLPPTIIWKHKGRDVILKKDVRFIVLSNNYLQIRGIK

KTDEGTYRCEGRILARGEINFKDIQVIVNVPPTIQARQNIVNATANLGQSVTLVCDAEGF

PEPTMSWTKDGEQIEQEEDDEKYIFSDDSSQLTIKKVDKNDEAEYICIAENKAGEQDATI

HLKVFAKPKITYVENQTAMELEEQVTLTCEASGDPIPSITWRTSTRNISSEEKTLDGHMV

VRSHARVSSLTLKSIQYTDAGEYICTASNTIGQDSQSMYLEVQYAPKLQGPVAVYTWEGN

QVNITCEVFAYPSATISWFRDGQLLPSSNYSNIKIYNTPSASYLEVTPDSENDFGNYNCT

AVNRIGQESLEFILVQADTPSSPSIDQVEPYSSTAQVQFDEPEATGGVPILKYKAEWRAV

GEEVWHSKWYDAKEASMEGIVTIVGLKPETTYAVRLAALNGKGLGEISAASEFKTQPVQG

EPSAPKLEGQMGEDGNSIKVNLIKQDDGGSPIRHYLVRYRALSSEWKPEIRLPSGSDHVM

LKSLDWNAEYEVYVVAENQQGKSKAAHFVFRTSAQPTAIPANGSPTSGLSTGAIVGILIV

IFVLLLVVVDITCYFLNKCGLFMCIAVNLCGKAGPGAKGKDMEEGKAAFSKDESKEPIVE

VRTEEERTPNHDGGKHTEPNETTPLTEPEKGPVEAKPECQETETKPAPAEVKTVPNDATQ

TKENESKA

>sp|P55072|TERA_HUMAN 641 QLIYIPLPDEKSRVA

MASGADSKGDDLSTAILKQKNRPNRLIVDEAINEDNSVVSLSQPKMDELQLFRGDTVLLK

GKKRREAVCIVLSDDTCSDEKIRMNRVVRNNLRVRLGDVISIQPCPDVKYGKRIHVLPID

DTVEGITGNLFEVYLKPYFLEAYRPIRKGDIFLVRGGMRAVEFKVVETDPSPYCIVAPDT

VIHCEGEPIKREDEEESLNEVGYDDIGGCRKQLAQIKEMVELPLRHPALFKAIGVKPPRG

ILLYGPPGTGKTLIARAVANETGAFFFLINGPEIMSKLAGESESNLRKAFEEAEKNAPAI

IFIDELDAIAPKREKTHGEVERRIVSQLLTLMDGLKQRAHVIVMAATNRPNSIDPALRRF

GRFDREVDIGIPDATGRLEILQIHTKNMKLADDVDLEQVANETHGHVGADLAALCSEAAL

QAIRKKMDLIDLEDETIDAEVMNSLAVTMDDFRWALSQSNPSALRETVVEVPQVTWEDIG

GLEDVKRELQELVQYPVEHPDKFLKFGMTPSKGVLFYGPPGCGKTLLAKAIANECQANFI

SIKGPELLTMWFGESEANVREIFDKARQAAPCVLFFDELDSIAKARGGNIGDGGGAADRV

INQILTEMDGMSTKKNVFIIGATNRPDIIDPAILRPGRLDQLIYIPLPDEKSRVAILKAN

LRKSPVAKDVDLEFLAKMTNGFSGADLTEICQRACKLAIRESIESEIRRERERQTNPSAM

EVEEDDPVPEIRRDHFEEAMRFARRSVSDNDIRKYEMFAQTLQQSRGFGSFRFPSGNQGG

AGPSQGSGGGTGGSVYTEDNDDDLYG

>sp|P55072|TERA_HUMAN 78 SDEKIRMNRVVRNNLR

MASGADSKGDDLSTAILKQKNRPNRLIVDEAINEDNSVVSLSQPKMDELQLFRGDTVLLK

GKKRREAVCIVLSDDTCSDEKIRMNRVVRNNLRVRLGDVISIQPCPDVKYGKRIHVLPID

DTVEGITGNLFEVYLKPYFLEAYRPIRKGDIFLVRGGMRAVEFKVVETDPSPYCIVAPDT

VIHCEGEPIKREDEEESLNEVGYDDIGGCRKQLAQIKEMVELPLRHPALFKAIGVKPPRG

ILLYGPPGTGKTLIARAVANETGAFFFLINGPEIMSKLAGESESNLRKAFEEAEKNAPAI

IFIDELDAIAPKREKTHGEVERRIVSQLLTLMDGLKQRAHVIVMAATNRPNSIDPALRRF

GRFDREVDIGIPDATGRLEILQIHTKNMKLADDVDLEQVANETHGHVGADLAALCSEAAL

QAIRKKMDLIDLEDETIDAEVMNSLAVTMDDFRWALSQSNPSALRETVVEVPQVTWEDIG

GLEDVKRELQELVQYPVEHPDKFLKFGMTPSKGVLFYGPPGCGKTLLAKAIANECQANFI

SIKGPELLTMWFGESEANVREIFDKARQAAPCVLFFDELDSIAKARGGNIGDGGGAADRV

INQILTEMDGMSTKKNVFIIGATNRPDIIDPAILRPGRLDQLIYIPLPDEKSRVAILKAN

LRKSPVAKDVDLEFLAKMTNGFSGADLTEICQRACKLAIRESIESEIRRERERQTNPSAM

EVEEDDPVPEIRRDHFEEAMRFARRSVSDNDIRKYEMFAQTLQQSRGFGSFRFPSGNQGG

AGPSQGSGGGTGGSVYTEDNDDDLYG

>sp|P26010|ITB7_HUMAN 697 LFFFLVEDDARGTV

MVALPMVLVLLLVLSRGESELDAKIPSTGDATEWRNPHLSMLGSCQPAPSCQKCILSHPS

CAWCKQLNFTASGEAEARRCARREELLARGCPLEELEEPRGQQEVLQDQPLSQGARGEGA

TQLAPQRVRVTLRPGEPQQLQVRFLRAEGYPVDLYYLMDLSYSMKDDLERVRQLGHALLV

RLQEVTHSVRIGFGSFVDKTVLPFVSTVPSKLRHPCPTRLERCQSPFSFHHVLSLTGDAQ

AFEREVGRQSVSGNLDSPEGGFDAILQAALCQEQIGWRNVSRLLVFTSDDTFHTAGDGKL

GGIFMPSDGHCHLDSNGLYSRSTEFDYPSVGQVAQALSAANIQPIFAVTSAALPVYQELS

KLIPKSAVGELSEDSSNVVQLIMDAYNSLSSTVTLEHSSLPPGVHISYESQCEGPEKREG

KAEDRGQCNHVRINQTVTFWVSLQATHCLPEPHLLRLRALGFSEELIVELHTLCDCNCSD

TQPQAPHCSDGQGHLQCGVCSCAPGRLGRLCECSVAELSSPDLESGCRAPNGTGPLCSGK

GHCQCGRCSCSGQSSGHLCECDDASCERHEGILCGGFGRCQCGVCHCHANRTGRACECSG

DMDSCISPEGGLCSGHGRCKCNRCQCLDGYYGALCDQCPGCKTPCERHRDCAECGAFRTG

PLATNCSTACAHTNVTLALAPILDDGWCKERTLDNQLFFFLVEDDARGTVVLRVRPQEKG

ADHTQAIVLGCVGGIVAVGLGLVLAYRLSVEIYDRREYSRFEKEQQQLNWKQDSNPLYKS

AITTTINPRFQEADSPTL

>sp|Q96QK1|VPS35_HUMAN 478 DPDPEDFADEQSLVGRFI

MPTTQQSPQDEQEKLLDEAIQAVKVQSFQMKRCLDKNKLMDALKHASNMLGELRTSMLSP

KSYYELYMAISDELHYLEVYLTDEFAKGRKVADLYELVQYAGNIIPRLYLLITVGVVYVK

SFPQSRKDILKDLVEMCRGVQHPLRGLFLRNYLLQCTRNILPDEGEPTDEETTGDISDSM

DFVLLNFAEMNKLWVRMQHQGHSRDREKRERERQELRILVGTNLVRLSQLEGVNVERYKQ

IVLTGILEQVVNCRDALAQEYLMECIIQVFPDEFHLQTLNPFLRACAELHQNVNVKNIII

ALIDRLALFAHREDGPGIPADIKLFDIFSQQVATVIQSRQDMPSEDVVSLQVSLINLAMK

CYPDRVDYVDKVLETTVEIFNKLNLEHIATSSAVSKELTRLLKIPVDTYNNILTVLKLKH

FHPLFEYFDYESRKSMSCYVLSNVLDYNTEIVSQDQVDSIMNLVSTLIQDQPDQPVEDPD

PEDFADEQSLVGRFIHLLRSEDPDQQYLILNTARKHFGAGGNQRIRFTLPPLVFAAYQLA

FRYKENSKVDDKWEKKCQKIFSFAHQTISALIKAELAELPLRLFLQGALAAGEIGFENHE

TVAYEFMSQAFSLYEDEISDSKAQLAAITLIIGTFERMKCFSEENHEPLRTQCALAASKL

LKKPDQGRAVSTCAHLFWSGRNTDKNGEELHGGKRVMECLKKALKIANQCMDPSLQVQLF

IEILNRYIYFYEKENDAVTIQVLNQLIQKIREDLPNLESSEETEQINKHFHNTLEHLRLR

RESPESEGPIYEGLIL

>sp|P21580|TNAP3_HUMAN 62 EIIHKALIDRNIQ

MAEQVLPQALYLSNMRKAVKIRERTPEDIFKPTNGIIHHFKTMHRYTLEMFRTCQFCPQF

REIIHKALIDRNIQATLESQKKLNWCREVRKLVALKTNGDGNCLMHATSQYMWGVQDTDL

VLRKALFSTLKETDTRNFKFRWQLESLKSQEFVETGLCYDTRNWNDEWDNLIKMASTDTP

MARSGLQYNSLEEIHIFVLCNILRRPIIVISDKMLRSLESGSNFAPLKVGGIYLPLHWPA

QECYRYPIVLGYDSHHFVPLVTLKDSGPEIRAVPLVNRDRGRFEDLKVHFLTDPENEMKE

KLLKEYLMVIEIPVQGWDHGTTHLINAAKLDEANLPKEINLVDDYFELVQHEYKKWQENS

EQGRREGHAQNPMEPSVPQLSLMDVKCETPNCPFFMSVNTQPLCHECSERRQKNQNKLPK

LNSKPGPEGLPGMALGASRGEAYEPLAWNPEESTGGPHSAPPTAPSPFLFSETTAMKCRS

PGCPFTLNVQHNGFCERCHNARQLHASHAPDHTRHLDPGKCQACLQDVTRTFNGICSTCF

KRTTAEASSSLSTSLPPSCHQRSKSDPSRLVRSPSPHSCHRAGNDAPAGCLSQAARTPGD

RTGTSKCRKAGCVYFGTPENKGFCTLCFIEYRENKHFAAASGKVSPTASRFQNTIPCLGR

ECGTLGSTMFEGYCQKCFIEAQNQRFHEAKRTEEQLRSSQRRDVPRTTQSTSRPKCARAS

CKNILACRSEELCMECQHPNQRMGPGAHRGEPAPEDPPKQRCRAPACDHFGNAKCNGYCN

ECFQFKQMYG

>sp|Q86VP1|TAXB1_HUMAN 107 HKGEIRGASTPFQFR

MTSFQEVPLQTSNFAHVIFQNVAKSYLPNAHLECHYTLTPYIHPHPKDWVGIFKVGWSTA

RDYYTFLWSPMPEHYVEGSTVNCVLAFQGYYLPNDDGEFYQFCYVTHKGEIRGASTPFQF

RASSPVEELLTMEDEGNSDMLVVTTKAGLLELKIEKTMKEKEELLKLIAVLEKETAQLRE

QVGRMERELNHEKERCDQLQAEQKGLTEVTQSLKMENEEFKKRFSDATSKAHQLEEDIVS

VTHKAIEKETELDSLKDKLKKAQHEREQLECQLKTEKDEKELYKVHLKNTEIENTKLMSE

VQTLKNLDGNKESVITHFKEEIGRLQLCLAEKENLQRTFLLTTSSKEDTCFLKEQLRKAE

EQVQATRQEVVFLAKELSDAVNVRDRTMADLHTARLENEKVKKQLADAVAELKLNAMKKD

QDKTDTLEHELRREVEDLKLRLQMAADHYKEKFKECQRLQKQINKLSDQSANNNNVFTKK

TGNQQKVNDASVNTDPATSASTVDVKPSPSAAEADFDIVTKGQVCEMTKEIADKTEKYNK

CKQLLQDEKAKCNKYADELAKMELKWKEQVKIAENVKLELAEVQDNYKELKRSLENPAER

KMEGQNSQSPQCFKTCSEQNGYVLTLSNAQPVLQYGNPYASQETRDGADGAFYPDEIQRP

PVRVPSWGLEDNVVCSQPARNFSRPDGLEDSEDSKEDENVPTAPDPPSQHLRGHGTGFCF

DSSFDVHKKCPLCELMFPPNYDQSKFEEHVESHWKVCPMCSEQFPPDYDQQVFERHVQTH

FDQNVLNFD

>sp|P02786|TFR1_HUMAN 353 CPSDWKTDSTCRMVTSE

MMDQARSAFSNLFGGEPLSYTRFSLARQVDGDNSHVEMKLAVDEEENADNNTKANVTKPK

RCSGSICYGTIAVIVFFLIGFMIGYLGYCKGVEPKTECERLAGTESPVREEPGEDFPAAR

RLYWDDLKRKLSEKLDSTDFTGTIKLLNENSYVPREAGSQKDENLALYVENQFREFKLSK

VWRDQHFVKIQVKDSAQNSVIIVDKNGRLVYLVENPGGYVAYSKAATVTGKLVHANFGTK

KDFEDLYTPVNGSIVIVRAGKITFAEKVANAESLNAIGVLIYMDQTKFPIVNAELSFFGH

AHLGTGDPYTPGFPSFNHTQFPPSRSSGLPNIPVQTISRAAAEKLFGNMEGDCPSDWKTD

STCRMVTSESKNVKLTVSNVLKEIKILNIFGVIKGFVEPDHYVVVGAQRDAWGPGAAKSG

VGTALLLKLAQMFSDMVLKDGFQPSRSIIFASWSAGDFGSVGATEWLEGYLSSLHLKAFT

YINLDKAVLGTSNFKVSASPLLYTLIEKTMQNVKHPVTGQFLYQDSNWASKVEKLTLDNA

AFPFLAYSGIPAVSFCFCEDTDYPYLGTTMDTYKELIERIPELNKVARAAAEVAGQFVIK

LTHDVELNLDYERYNSQLLSFVRDLNQYRADIKEMGLSLQWLYSARGDFFRATSRLTTDF

GNAEKTDRFVMKKLNDRVMRVEYHFLSPYVSPKESPFRHVFWGSGSHTLPALLENLKLRK

QNNGAFNETLFRNQLALATWTIQGAANALSGDVWDIDNEF

>sp|P02786|TFR1_HUMAN 139 DFTGTIKLLNENSYVPR

MMDQARSAFSNLFGGEPLSYTRFSLARQVDGDNSHVEMKLAVDEEENADNNTKANVTKPK

RCSGSICYGTIAVIVFFLIGFMIGYLGYCKGVEPKTECERLAGTESPVREEPGEDFPAAR

RLYWDDLKRKLSEKLDSTDFTGTIKLLNENSYVPREAGSQKDENLALYVENQFREFKLSK

VWRDQHFVKIQVKDSAQNSVIIVDKNGRLVYLVENPGGYVAYSKAATVTGKLVHANFGTK

KDFEDLYTPVNGSIVIVRAGKITFAEKVANAESLNAIGVLIYMDQTKFPIVNAELSFFGH

AHLGTGDPYTPGFPSFNHTQFPPSRSSGLPNIPVQTISRAAAEKLFGNMEGDCPSDWKTD

STCRMVTSESKNVKLTVSNVLKEIKILNIFGVIKGFVEPDHYVVVGAQRDAWGPGAAKSG

VGTALLLKLAQMFSDMVLKDGFQPSRSIIFASWSAGDFGSVGATEWLEGYLSSLHLKAFT

YINLDKAVLGTSNFKVSASPLLYTLIEKTMQNVKHPVTGQFLYQDSNWASKVEKLTLDNA

AFPFLAYSGIPAVSFCFCEDTDYPYLGTTMDTYKELIERIPELNKVARAAAEVAGQFVIK

LTHDVELNLDYERYNSQLLSFVRDLNQYRADIKEMGLSLQWLYSARGDFFRATSRLTTDF

GNAEKTDRFVMKKLNDRVMRVEYHFLSPYVSPKESPFRHVFWGSGSHTLPALLENLKLRK

QNNGAFNETLFRNQLALATWTIQGAANALSGDVWDIDNEF

>sp|P02786|TFR1_HUMAN 383 EIKILNIFGVIKGFVE

MMDQARSAFSNLFGGEPLSYTRFSLARQVDGDNSHVEMKLAVDEEENADNNTKANVTKPK

RCSGSICYGTIAVIVFFLIGFMIGYLGYCKGVEPKTECERLAGTESPVREEPGEDFPAAR

RLYWDDLKRKLSEKLDSTDFTGTIKLLNENSYVPREAGSQKDENLALYVENQFREFKLSK

VWRDQHFVKIQVKDSAQNSVIIVDKNGRLVYLVENPGGYVAYSKAATVTGKLVHANFGTK

KDFEDLYTPVNGSIVIVRAGKITFAEKVANAESLNAIGVLIYMDQTKFPIVNAELSFFGH

AHLGTGDPYTPGFPSFNHTQFPPSRSSGLPNIPVQTISRAAAEKLFGNMEGDCPSDWKTD

STCRMVTSESKNVKLTVSNVLKEIKILNIFGVIKGFVEPDHYVVVGAQRDAWGPGAAKSG

VGTALLLKLAQMFSDMVLKDGFQPSRSIIFASWSAGDFGSVGATEWLEGYLSSLHLKAFT

YINLDKAVLGTSNFKVSASPLLYTLIEKTMQNVKHPVTGQFLYQDSNWASKVEKLTLDNA

AFPFLAYSGIPAVSFCFCEDTDYPYLGTTMDTYKELIERIPELNKVARAAAEVAGQFVIK

LTHDVELNLDYERYNSQLLSFVRDLNQYRADIKEMGLSLQWLYSARGDFFRATSRLTTDF

GNAEKTDRFVMKKLNDRVMRVEYHFLSPYVSPKESPFRHVFWGSGSHTLPALLENLKLRK

QNNGAFNETLFRNQLALATWTIQGAANALSGDVWDIDNEF

>sp|P02786|TFR1_HUMAN 383 EIKILNIFGVIKGFVEP

MMDQARSAFSNLFGGEPLSYTRFSLARQVDGDNSHVEMKLAVDEEENADNNTKANVTKPK

RCSGSICYGTIAVIVFFLIGFMIGYLGYCKGVEPKTECERLAGTESPVREEPGEDFPAAR

RLYWDDLKRKLSEKLDSTDFTGTIKLLNENSYVPREAGSQKDENLALYVENQFREFKLSK

VWRDQHFVKIQVKDSAQNSVIIVDKNGRLVYLVENPGGYVAYSKAATVTGKLVHANFGTK

KDFEDLYTPVNGSIVIVRAGKITFAEKVANAESLNAIGVLIYMDQTKFPIVNAELSFFGH

AHLGTGDPYTPGFPSFNHTQFPPSRSSGLPNIPVQTISRAAAEKLFGNMEGDCPSDWKTD

STCRMVTSESKNVKLTVSNVLKEIKILNIFGVIKGFVEPDHYVVVGAQRDAWGPGAAKSG

VGTALLLKLAQMFSDMVLKDGFQPSRSIIFASWSAGDFGSVGATEWLEGYLSSLHLKAFT

YINLDKAVLGTSNFKVSASPLLYTLIEKTMQNVKHPVTGQFLYQDSNWASKVEKLTLDNA

AFPFLAYSGIPAVSFCFCEDTDYPYLGTTMDTYKELIERIPELNKVARAAAEVAGQFVIK

LTHDVELNLDYERYNSQLLSFVRDLNQYRADIKEMGLSLQWLYSARGDFFRATSRLTTDF

GNAEKTDRFVMKKLNDRVMRVEYHFLSPYVSPKESPFRHVFWGSGSHTLPALLENLKLRK

QNNGAFNETLFRNQLALATWTIQGAANALSGDVWDIDNEF

>sp|P02786|TFR1_HUMAN 398 EPDHYVVVGAQRDA

MMDQARSAFSNLFGGEPLSYTRFSLARQVDGDNSHVEMKLAVDEEENADNNTKANVTKPK

RCSGSICYGTIAVIVFFLIGFMIGYLGYCKGVEPKTECERLAGTESPVREEPGEDFPAAR

RLYWDDLKRKLSEKLDSTDFTGTIKLLNENSYVPREAGSQKDENLALYVENQFREFKLSK

VWRDQHFVKIQVKDSAQNSVIIVDKNGRLVYLVENPGGYVAYSKAATVTGKLVHANFGTK

KDFEDLYTPVNGSIVIVRAGKITFAEKVANAESLNAIGVLIYMDQTKFPIVNAELSFFGH

AHLGTGDPYTPGFPSFNHTQFPPSRSSGLPNIPVQTISRAAAEKLFGNMEGDCPSDWKTD

STCRMVTSESKNVKLTVSNVLKEIKILNIFGVIKGFVEPDHYVVVGAQRDAWGPGAAKSG

VGTALLLKLAQMFSDMVLKDGFQPSRSIIFASWSAGDFGSVGATEWLEGYLSSLHLKAFT

YINLDKAVLGTSNFKVSASPLLYTLIEKTMQNVKHPVTGQFLYQDSNWASKVEKLTLDNA

AFPFLAYSGIPAVSFCFCEDTDYPYLGTTMDTYKELIERIPELNKVARAAAEVAGQFVIK

LTHDVELNLDYERYNSQLLSFVRDLNQYRADIKEMGLSLQWLYSARGDFFRATSRLTTDF

GNAEKTDRFVMKKLNDRVMRVEYHFLSPYVSPKESPFRHVFWGSGSHTLPALLENLKLRK

QNNGAFNETLFRNQLALATWTIQGAANALSGDVWDIDNEF

>sp|P02786|TFR1_HUMAN 173 FREFKLSKVWRDQ

MMDQARSAFSNLFGGEPLSYTRFSLARQVDGDNSHVEMKLAVDEEENADNNTKANVTKPK

RCSGSICYGTIAVIVFFLIGFMIGYLGYCKGVEPKTECERLAGTESPVREEPGEDFPAAR

RLYWDDLKRKLSEKLDSTDFTGTIKLLNENSYVPREAGSQKDENLALYVENQFREFKLSK

VWRDQHFVKIQVKDSAQNSVIIVDKNGRLVYLVENPGGYVAYSKAATVTGKLVHANFGTK

KDFEDLYTPVNGSIVIVRAGKITFAEKVANAESLNAIGVLIYMDQTKFPIVNAELSFFGH

AHLGTGDPYTPGFPSFNHTQFPPSRSSGLPNIPVQTISRAAAEKLFGNMEGDCPSDWKTD

STCRMVTSESKNVKLTVSNVLKEIKILNIFGVIKGFVEPDHYVVVGAQRDAWGPGAAKSG

VGTALLLKLAQMFSDMVLKDGFQPSRSIIFASWSAGDFGSVGATEWLEGYLSSLHLKAFT

YINLDKAVLGTSNFKVSASPLLYTLIEKTMQNVKHPVTGQFLYQDSNWASKVEKLTLDNA

AFPFLAYSGIPAVSFCFCEDTDYPYLGTTMDTYKELIERIPELNKVARAAAEVAGQFVIK

LTHDVELNLDYERYNSQLLSFVRDLNQYRADIKEMGLSLQWLYSARGDFFRATSRLTTDF

GNAEKTDRFVMKKLNDRVMRVEYHFLSPYVSPKESPFRHVFWGSGSHTLPALLENLKLRK

QNNGAFNETLFRNQLALATWTIQGAANALSGDVWDIDNEF

>sp|P02786|TFR1_HUMAN 173 FREFKLSKVWRDQH

MMDQARSAFSNLFGGEPLSYTRFSLARQVDGDNSHVEMKLAVDEEENADNNTKANVTKPK

RCSGSICYGTIAVIVFFLIGFMIGYLGYCKGVEPKTECERLAGTESPVREEPGEDFPAAR

RLYWDDLKRKLSEKLDSTDFTGTIKLLNENSYVPREAGSQKDENLALYVENQFREFKLSK

VWRDQHFVKIQVKDSAQNSVIIVDKNGRLVYLVENPGGYVAYSKAATVTGKLVHANFGTK

KDFEDLYTPVNGSIVIVRAGKITFAEKVANAESLNAIGVLIYMDQTKFPIVNAELSFFGH

AHLGTGDPYTPGFPSFNHTQFPPSRSSGLPNIPVQTISRAAAEKLFGNMEGDCPSDWKTD

STCRMVTSESKNVKLTVSNVLKEIKILNIFGVIKGFVEPDHYVVVGAQRDAWGPGAAKSG

VGTALLLKLAQMFSDMVLKDGFQPSRSIIFASWSAGDFGSVGATEWLEGYLSSLHLKAFT

YINLDKAVLGTSNFKVSASPLLYTLIEKTMQNVKHPVTGQFLYQDSNWASKVEKLTLDNA

AFPFLAYSGIPAVSFCFCEDTDYPYLGTTMDTYKELIERIPELNKVARAAAEVAGQFVIK

LTHDVELNLDYERYNSQLLSFVRDLNQYRADIKEMGLSLQWLYSARGDFFRATSRLTTDF

GNAEKTDRFVMKKLNDRVMRVEYHFLSPYVSPKESPFRHVFWGSGSHTLPALLENLKLRK

QNNGAFNETLFRNQLALATWTIQGAANALSGDVWDIDNEF

>sp|P02786|TFR1_HUMAN 140 FTGTIKLLNENSYVPR

MMDQARSAFSNLFGGEPLSYTRFSLARQVDGDNSHVEMKLAVDEEENADNNTKANVTKPK

RCSGSICYGTIAVIVFFLIGFMIGYLGYCKGVEPKTECERLAGTESPVREEPGEDFPAAR

RLYWDDLKRKLSEKLDSTDFTGTIKLLNENSYVPREAGSQKDENLALYVENQFREFKLSK

VWRDQHFVKIQVKDSAQNSVIIVDKNGRLVYLVENPGGYVAYSKAATVTGKLVHANFGTK

KDFEDLYTPVNGSIVIVRAGKITFAEKVANAESLNAIGVLIYMDQTKFPIVNAELSFFGH

AHLGTGDPYTPGFPSFNHTQFPPSRSSGLPNIPVQTISRAAAEKLFGNMEGDCPSDWKTD

STCRMVTSESKNVKLTVSNVLKEIKILNIFGVIKGFVEPDHYVVVGAQRDAWGPGAAKSG

VGTALLLKLAQMFSDMVLKDGFQPSRSIIFASWSAGDFGSVGATEWLEGYLSSLHLKAFT

YINLDKAVLGTSNFKVSASPLLYTLIEKTMQNVKHPVTGQFLYQDSNWASKVEKLTLDNA

AFPFLAYSGIPAVSFCFCEDTDYPYLGTTMDTYKELIERIPELNKVARAAAEVAGQFVIK

LTHDVELNLDYERYNSQLLSFVRDLNQYRADIKEMGLSLQWLYSARGDFFRATSRLTTDF

GNAEKTDRFVMKKLNDRVMRVEYHFLSPYVSPKESPFRHVFWGSGSHTLPALLENLKLRK

QNNGAFNETLFRNQLALATWTIQGAANALSGDVWDIDNEF

>sp|P02786|TFR1_HUMAN 479 FTYINLDKAVLGTSN

MMDQARSAFSNLFGGEPLSYTRFSLARQVDGDNSHVEMKLAVDEEENADNNTKANVTKPK

RCSGSICYGTIAVIVFFLIGFMIGYLGYCKGVEPKTECERLAGTESPVREEPGEDFPAAR

RLYWDDLKRKLSEKLDSTDFTGTIKLLNENSYVPREAGSQKDENLALYVENQFREFKLSK

VWRDQHFVKIQVKDSAQNSVIIVDKNGRLVYLVENPGGYVAYSKAATVTGKLVHANFGTK

KDFEDLYTPVNGSIVIVRAGKITFAEKVANAESLNAIGVLIYMDQTKFPIVNAELSFFGH

AHLGTGDPYTPGFPSFNHTQFPPSRSSGLPNIPVQTISRAAAEKLFGNMEGDCPSDWKTD

STCRMVTSESKNVKLTVSNVLKEIKILNIFGVIKGFVEPDHYVVVGAQRDAWGPGAAKSG

VGTALLLKLAQMFSDMVLKDGFQPSRSIIFASWSAGDFGSVGATEWLEGYLSSLHLKAFT

YINLDKAVLGTSNFKVSASPLLYTLIEKTMQNVKHPVTGQFLYQDSNWASKVEKLTLDNA

AFPFLAYSGIPAVSFCFCEDTDYPYLGTTMDTYKELIERIPELNKVARAAAEVAGQFVIK

LTHDVELNLDYERYNSQLLSFVRDLNQYRADIKEMGLSLQWLYSARGDFFRATSRLTTDF

GNAEKTDRFVMKKLNDRVMRVEYHFLSPYVSPKESPFRHVFWGSGSHTLPALLENLKLRK

QNNGAFNETLFRNQLALATWTIQGAANALSGDVWDIDNEF

>sp|P02786|TFR1_HUMAN 15 GEPLSYTRFSLARQVDG

MMDQARSAFSNLFGGEPLSYTRFSLARQVDGDNSHVEMKLAVDEEENADNNTKANVTKPK

RCSGSICYGTIAVIVFFLIGFMIGYLGYCKGVEPKTECERLAGTESPVREEPGEDFPAAR

RLYWDDLKRKLSEKLDSTDFTGTIKLLNENSYVPREAGSQKDENLALYVENQFREFKLSK

VWRDQHFVKIQVKDSAQNSVIIVDKNGRLVYLVENPGGYVAYSKAATVTGKLVHANFGTK

KDFEDLYTPVNGSIVIVRAGKITFAEKVANAESLNAIGVLIYMDQTKFPIVNAELSFFGH

AHLGTGDPYTPGFPSFNHTQFPPSRSSGLPNIPVQTISRAAAEKLFGNMEGDCPSDWKTD

STCRMVTSESKNVKLTVSNVLKEIKILNIFGVIKGFVEPDHYVVVGAQRDAWGPGAAKSG

VGTALLLKLAQMFSDMVLKDGFQPSRSIIFASWSAGDFGSVGATEWLEGYLSSLHLKAFT

YINLDKAVLGTSNFKVSASPLLYTLIEKTMQNVKHPVTGQFLYQDSNWASKVEKLTLDNA

AFPFLAYSGIPAVSFCFCEDTDYPYLGTTMDTYKELIERIPELNKVARAAAEVAGQFVIK

LTHDVELNLDYERYNSQLLSFVRDLNQYRADIKEMGLSLQWLYSARGDFFRATSRLTTDF

GNAEKTDRFVMKKLNDRVMRVEYHFLSPYVSPKESPFRHVFWGSGSHTLPALLENLKLRK

QNNGAFNETLFRNQLALATWTIQGAANALSGDVWDIDNEF

>sp|P02786|TFR1_HUMAN 515 HPVTGQFLYQDSNWASKVE

MMDQARSAFSNLFGGEPLSYTRFSLARQVDGDNSHVEMKLAVDEEENADNNTKANVTKPK

RCSGSICYGTIAVIVFFLIGFMIGYLGYCKGVEPKTECERLAGTESPVREEPGEDFPAAR

RLYWDDLKRKLSEKLDSTDFTGTIKLLNENSYVPREAGSQKDENLALYVENQFREFKLSK

VWRDQHFVKIQVKDSAQNSVIIVDKNGRLVYLVENPGGYVAYSKAATVTGKLVHANFGTK

KDFEDLYTPVNGSIVIVRAGKITFAEKVANAESLNAIGVLIYMDQTKFPIVNAELSFFGH

AHLGTGDPYTPGFPSFNHTQFPPSRSSGLPNIPVQTISRAAAEKLFGNMEGDCPSDWKTD

STCRMVTSESKNVKLTVSNVLKEIKILNIFGVIKGFVEPDHYVVVGAQRDAWGPGAAKSG

VGTALLLKLAQMFSDMVLKDGFQPSRSIIFASWSAGDFGSVGATEWLEGYLSSLHLKAFT

YINLDKAVLGTSNFKVSASPLLYTLIEKTMQNVKHPVTGQFLYQDSNWASKVEKLTLDNA

AFPFLAYSGIPAVSFCFCEDTDYPYLGTTMDTYKELIERIPELNKVARAAAEVAGQFVIK

LTHDVELNLDYERYNSQLLSFVRDLNQYRADIKEMGLSLQWLYSARGDFFRATSRLTTDF

GNAEKTDRFVMKKLNDRVMRVEYHFLSPYVSPKESPFRHVFWGSGSHTLPALLENLKLRK

QNNGAFNETLFRNQLALATWTIQGAANALSGDVWDIDNEF

>sp|P02786|TFR1_HUMAN 384 IKILNIFGVIKGFVE

MMDQARSAFSNLFGGEPLSYTRFSLARQVDGDNSHVEMKLAVDEEENADNNTKANVTKPK

RCSGSICYGTIAVIVFFLIGFMIGYLGYCKGVEPKTECERLAGTESPVREEPGEDFPAAR

RLYWDDLKRKLSEKLDSTDFTGTIKLLNENSYVPREAGSQKDENLALYVENQFREFKLSK

VWRDQHFVKIQVKDSAQNSVIIVDKNGRLVYLVENPGGYVAYSKAATVTGKLVHANFGTK

KDFEDLYTPVNGSIVIVRAGKITFAEKVANAESLNAIGVLIYMDQTKFPIVNAELSFFGH

AHLGTGDPYTPGFPSFNHTQFPPSRSSGLPNIPVQTISRAAAEKLFGNMEGDCPSDWKTD

STCRMVTSESKNVKLTVSNVLKEIKILNIFGVIKGFVEPDHYVVVGAQRDAWGPGAAKSG

VGTALLLKLAQMFSDMVLKDGFQPSRSIIFASWSAGDFGSVGATEWLEGYLSSLHLKAFT

YINLDKAVLGTSNFKVSASPLLYTLIEKTMQNVKHPVTGQFLYQDSNWASKVEKLTLDNA

AFPFLAYSGIPAVSFCFCEDTDYPYLGTTMDTYKELIERIPELNKVARAAAEVAGQFVIK

LTHDVELNLDYERYNSQLLSFVRDLNQYRADIKEMGLSLQWLYSARGDFFRATSRLTTDF

GNAEKTDRFVMKKLNDRVMRVEYHFLSPYVSPKESPFRHVFWGSGSHTLPALLENLKLRK

QNNGAFNETLFRNQLALATWTIQGAANALSGDVWDIDNEF

>sp|P02786|TFR1_HUMAN 384 IKILNIFGVIKGFVEP

MMDQARSAFSNLFGGEPLSYTRFSLARQVDGDNSHVEMKLAVDEEENADNNTKANVTKPK

RCSGSICYGTIAVIVFFLIGFMIGYLGYCKGVEPKTECERLAGTESPVREEPGEDFPAAR

RLYWDDLKRKLSEKLDSTDFTGTIKLLNENSYVPREAGSQKDENLALYVENQFREFKLSK

VWRDQHFVKIQVKDSAQNSVIIVDKNGRLVYLVENPGGYVAYSKAATVTGKLVHANFGTK

KDFEDLYTPVNGSIVIVRAGKITFAEKVANAESLNAIGVLIYMDQTKFPIVNAELSFFGH

AHLGTGDPYTPGFPSFNHTQFPPSRSSGLPNIPVQTISRAAAEKLFGNMEGDCPSDWKTD

STCRMVTSESKNVKLTVSNVLKEIKILNIFGVIKGFVEPDHYVVVGAQRDAWGPGAAKSG

VGTALLLKLAQMFSDMVLKDGFQPSRSIIFASWSAGDFGSVGATEWLEGYLSSLHLKAFT

YINLDKAVLGTSNFKVSASPLLYTLIEKTMQNVKHPVTGQFLYQDSNWASKVEKLTLDNA

AFPFLAYSGIPAVSFCFCEDTDYPYLGTTMDTYKELIERIPELNKVARAAAEVAGQFVIK

LTHDVELNLDYERYNSQLLSFVRDLNQYRADIKEMGLSLQWLYSARGDFFRATSRLTTDF

GNAEKTDRFVMKKLNDRVMRVEYHFLSPYVSPKESPFRHVFWGSGSHTLPALLENLKLRK

QNNGAFNETLFRNQLALATWTIQGAANALSGDVWDIDNEF

>sp|P02786|TFR1_HUMAN 384 IKILNIFGVIKGFVEPD

MMDQARSAFSNLFGGEPLSYTRFSLARQVDGDNSHVEMKLAVDEEENADNNTKANVTKPK

RCSGSICYGTIAVIVFFLIGFMIGYLGYCKGVEPKTECERLAGTESPVREEPGEDFPAAR

RLYWDDLKRKLSEKLDSTDFTGTIKLLNENSYVPREAGSQKDENLALYVENQFREFKLSK

VWRDQHFVKIQVKDSAQNSVIIVDKNGRLVYLVENPGGYVAYSKAATVTGKLVHANFGTK

KDFEDLYTPVNGSIVIVRAGKITFAEKVANAESLNAIGVLIYMDQTKFPIVNAELSFFGH

AHLGTGDPYTPGFPSFNHTQFPPSRSSGLPNIPVQTISRAAAEKLFGNMEGDCPSDWKTD

STCRMVTSESKNVKLTVSNVLKEIKILNIFGVIKGFVEPDHYVVVGAQRDAWGPGAAKSG

VGTALLLKLAQMFSDMVLKDGFQPSRSIIFASWSAGDFGSVGATEWLEGYLSSLHLKAFT

YINLDKAVLGTSNFKVSASPLLYTLIEKTMQNVKHPVTGQFLYQDSNWASKVEKLTLDNA

AFPFLAYSGIPAVSFCFCEDTDYPYLGTTMDTYKELIERIPELNKVARAAAEVAGQFVIK

LTHDVELNLDYERYNSQLLSFVRDLNQYRADIKEMGLSLQWLYSARGDFFRATSRLTTDF

GNAEKTDRFVMKKLNDRVMRVEYHFLSPYVSPKESPFRHVFWGSGSHTLPALLENLKLRK

QNNGAFNETLFRNQLALATWTIQGAANALSGDVWDIDNEF

>sp|P02786|TFR1_HUMAN 580 IPELNKVARAAA

MMDQARSAFSNLFGGEPLSYTRFSLARQVDGDNSHVEMKLAVDEEENADNNTKANVTKPK

RCSGSICYGTIAVIVFFLIGFMIGYLGYCKGVEPKTECERLAGTESPVREEPGEDFPAAR

RLYWDDLKRKLSEKLDSTDFTGTIKLLNENSYVPREAGSQKDENLALYVENQFREFKLSK

VWRDQHFVKIQVKDSAQNSVIIVDKNGRLVYLVENPGGYVAYSKAATVTGKLVHANFGTK

KDFEDLYTPVNGSIVIVRAGKITFAEKVANAESLNAIGVLIYMDQTKFPIVNAELSFFGH

AHLGTGDPYTPGFPSFNHTQFPPSRSSGLPNIPVQTISRAAAEKLFGNMEGDCPSDWKTD

STCRMVTSESKNVKLTVSNVLKEIKILNIFGVIKGFVEPDHYVVVGAQRDAWGPGAAKSG

VGTALLLKLAQMFSDMVLKDGFQPSRSIIFASWSAGDFGSVGATEWLEGYLSSLHLKAFT

YINLDKAVLGTSNFKVSASPLLYTLIEKTMQNVKHPVTGQFLYQDSNWASKVEKLTLDNA

AFPFLAYSGIPAVSFCFCEDTDYPYLGTTMDTYKELIERIPELNKVARAAAEVAGQFVIK

LTHDVELNLDYERYNSQLLSFVRDLNQYRADIKEMGLSLQWLYSARGDFFRATSRLTTDF

GNAEKTDRFVMKKLNDRVMRVEYHFLSPYVSPKESPFRHVFWGSGSHTLPALLENLKLRK

QNNGAFNETLFRNQLALATWTIQGAANALSGDVWDIDNEF

>sp|P02786|TFR1_HUMAN 580 IPELNKVARAAAEVAGQF

MMDQARSAFSNLFGGEPLSYTRFSLARQVDGDNSHVEMKLAVDEEENADNNTKANVTKPK

RCSGSICYGTIAVIVFFLIGFMIGYLGYCKGVEPKTECERLAGTESPVREEPGEDFPAAR

RLYWDDLKRKLSEKLDSTDFTGTIKLLNENSYVPREAGSQKDENLALYVENQFREFKLSK

VWRDQHFVKIQVKDSAQNSVIIVDKNGRLVYLVENPGGYVAYSKAATVTGKLVHANFGTK

KDFEDLYTPVNGSIVIVRAGKITFAEKVANAESLNAIGVLIYMDQTKFPIVNAELSFFGH

AHLGTGDPYTPGFPSFNHTQFPPSRSSGLPNIPVQTISRAAAEKLFGNMEGDCPSDWKTD

STCRMVTSESKNVKLTVSNVLKEIKILNIFGVIKGFVEPDHYVVVGAQRDAWGPGAAKSG

VGTALLLKLAQMFSDMVLKDGFQPSRSIIFASWSAGDFGSVGATEWLEGYLSSLHLKAFT

YINLDKAVLGTSNFKVSASPLLYTLIEKTMQNVKHPVTGQFLYQDSNWASKVEKLTLDNA

AFPFLAYSGIPAVSFCFCEDTDYPYLGTTMDTYKELIERIPELNKVARAAAEVAGQFVIK

LTHDVELNLDYERYNSQLLSFVRDLNQYRADIKEMGLSLQWLYSARGDFFRATSRLTTDF

GNAEKTDRFVMKKLNDRVMRVEYHFLSPYVSPKESPFRHVFWGSGSHTLPALLENLKLRK

QNNGAFNETLFRNQLALATWTIQGAANALSGDVWDIDNEF

>sp|P02786|TFR1_HUMAN 332 IPVQTISRAAAEKLFG

MMDQARSAFSNLFGGEPLSYTRFSLARQVDGDNSHVEMKLAVDEEENADNNTKANVTKPK

RCSGSICYGTIAVIVFFLIGFMIGYLGYCKGVEPKTECERLAGTESPVREEPGEDFPAAR

RLYWDDLKRKLSEKLDSTDFTGTIKLLNENSYVPREAGSQKDENLALYVENQFREFKLSK

VWRDQHFVKIQVKDSAQNSVIIVDKNGRLVYLVENPGGYVAYSKAATVTGKLVHANFGTK

KDFEDLYTPVNGSIVIVRAGKITFAEKVANAESLNAIGVLIYMDQTKFPIVNAELSFFGH

AHLGTGDPYTPGFPSFNHTQFPPSRSSGLPNIPVQTISRAAAEKLFGNMEGDCPSDWKTD

STCRMVTSESKNVKLTVSNVLKEIKILNIFGVIKGFVEPDHYVVVGAQRDAWGPGAAKSG

VGTALLLKLAQMFSDMVLKDGFQPSRSIIFASWSAGDFGSVGATEWLEGYLSSLHLKAFT

YINLDKAVLGTSNFKVSASPLLYTLIEKTMQNVKHPVTGQFLYQDSNWASKVEKLTLDNA

AFPFLAYSGIPAVSFCFCEDTDYPYLGTTMDTYKELIERIPELNKVARAAAEVAGQFVIK

LTHDVELNLDYERYNSQLLSFVRDLNQYRADIKEMGLSLQWLYSARGDFFRATSRLTTDF

GNAEKTDRFVMKKLNDRVMRVEYHFLSPYVSPKESPFRHVFWGSGSHTLPALLENLKLRK

QNNGAFNETLFRNQLALATWTIQGAANALSGDVWDIDNEF

>sp|P02786|TFR1_HUMAN 737 LATWTIQGAANALSGDVW

MMDQARSAFSNLFGGEPLSYTRFSLARQVDGDNSHVEMKLAVDEEENADNNTKANVTKPK

RCSGSICYGTIAVIVFFLIGFMIGYLGYCKGVEPKTECERLAGTESPVREEPGEDFPAAR

RLYWDDLKRKLSEKLDSTDFTGTIKLLNENSYVPREAGSQKDENLALYVENQFREFKLSK

VWRDQHFVKIQVKDSAQNSVIIVDKNGRLVYLVENPGGYVAYSKAATVTGKLVHANFGTK

KDFEDLYTPVNGSIVIVRAGKITFAEKVANAESLNAIGVLIYMDQTKFPIVNAELSFFGH

AHLGTGDPYTPGFPSFNHTQFPPSRSSGLPNIPVQTISRAAAEKLFGNMEGDCPSDWKTD

STCRMVTSESKNVKLTVSNVLKEIKILNIFGVIKGFVEPDHYVVVGAQRDAWGPGAAKSG

VGTALLLKLAQMFSDMVLKDGFQPSRSIIFASWSAGDFGSVGATEWLEGYLSSLHLKAFT

YINLDKAVLGTSNFKVSASPLLYTLIEKTMQNVKHPVTGQFLYQDSNWASKVEKLTLDNA

AFPFLAYSGIPAVSFCFCEDTDYPYLGTTMDTYKELIERIPELNKVARAAAEVAGQFVIK

LTHDVELNLDYERYNSQLLSFVRDLNQYRADIKEMGLSLQWLYSARGDFFRATSRLTTDF

GNAEKTDRFVMKKLNDRVMRVEYHFLSPYVSPKESPFRHVFWGSGSHTLPALLENLKLRK

QNNGAFNETLFRNQLALATWTIQGAANALSGDVWDIDNEF

>sp|P02786|TFR1_HUMAN 618 LLSFVRDLNQYRADI

MMDQARSAFSNLFGGEPLSYTRFSLARQVDGDNSHVEMKLAVDEEENADNNTKANVTKPK

RCSGSICYGTIAVIVFFLIGFMIGYLGYCKGVEPKTECERLAGTESPVREEPGEDFPAAR

RLYWDDLKRKLSEKLDSTDFTGTIKLLNENSYVPREAGSQKDENLALYVENQFREFKLSK

VWRDQHFVKIQVKDSAQNSVIIVDKNGRLVYLVENPGGYVAYSKAATVTGKLVHANFGTK

KDFEDLYTPVNGSIVIVRAGKITFAEKVANAESLNAIGVLIYMDQTKFPIVNAELSFFGH

AHLGTGDPYTPGFPSFNHTQFPPSRSSGLPNIPVQTISRAAAEKLFGNMEGDCPSDWKTD

STCRMVTSESKNVKLTVSNVLKEIKILNIFGVIKGFVEPDHYVVVGAQRDAWGPGAAKSG

VGTALLLKLAQMFSDMVLKDGFQPSRSIIFASWSAGDFGSVGATEWLEGYLSSLHLKAFT

YINLDKAVLGTSNFKVSASPLLYTLIEKTMQNVKHPVTGQFLYQDSNWASKVEKLTLDNA

AFPFLAYSGIPAVSFCFCEDTDYPYLGTTMDTYKELIERIPELNKVARAAAEVAGQFVIK

LTHDVELNLDYERYNSQLLSFVRDLNQYRADIKEMGLSLQWLYSARGDFFRATSRLTTDF

GNAEKTDRFVMKKLNDRVMRVEYHFLSPYVSPKESPFRHVFWGSGSHTLPALLENLKLRK

QNNGAFNETLFRNQLALATWTIQGAANALSGDVWDIDNEF

>sp|P02786|TFR1_HUMAN 329 LPNIPVQTISRAAAEKLF

MMDQARSAFSNLFGGEPLSYTRFSLARQVDGDNSHVEMKLAVDEEENADNNTKANVTKPK

RCSGSICYGTIAVIVFFLIGFMIGYLGYCKGVEPKTECERLAGTESPVREEPGEDFPAAR

RLYWDDLKRKLSEKLDSTDFTGTIKLLNENSYVPREAGSQKDENLALYVENQFREFKLSK

VWRDQHFVKIQVKDSAQNSVIIVDKNGRLVYLVENPGGYVAYSKAATVTGKLVHANFGTK

KDFEDLYTPVNGSIVIVRAGKITFAEKVANAESLNAIGVLIYMDQTKFPIVNAELSFFGH

AHLGTGDPYTPGFPSFNHTQFPPSRSSGLPNIPVQTISRAAAEKLFGNMEGDCPSDWKTD

STCRMVTSESKNVKLTVSNVLKEIKILNIFGVIKGFVEPDHYVVVGAQRDAWGPGAAKSG

VGTALLLKLAQMFSDMVLKDGFQPSRSIIFASWSAGDFGSVGATEWLEGYLSSLHLKAFT

YINLDKAVLGTSNFKVSASPLLYTLIEKTMQNVKHPVTGQFLYQDSNWASKVEKLTLDNA

AFPFLAYSGIPAVSFCFCEDTDYPYLGTTMDTYKELIERIPELNKVARAAAEVAGQFVIK

LTHDVELNLDYERYNSQLLSFVRDLNQYRADIKEMGLSLQWLYSARGDFFRATSRLTTDF

GNAEKTDRFVMKKLNDRVMRVEYHFLSPYVSPKESPFRHVFWGSGSHTLPALLENLKLRK

QNNGAFNETLFRNQLALATWTIQGAANALSGDVWDIDNEF

>sp|P02786|TFR1_HUMAN 215 NPGGYVAYSKAATVTG

MMDQARSAFSNLFGGEPLSYTRFSLARQVDGDNSHVEMKLAVDEEENADNNTKANVTKPK

RCSGSICYGTIAVIVFFLIGFMIGYLGYCKGVEPKTECERLAGTESPVREEPGEDFPAAR

RLYWDDLKRKLSEKLDSTDFTGTIKLLNENSYVPREAGSQKDENLALYVENQFREFKLSK

VWRDQHFVKIQVKDSAQNSVIIVDKNGRLVYLVENPGGYVAYSKAATVTGKLVHANFGTK

KDFEDLYTPVNGSIVIVRAGKITFAEKVANAESLNAIGVLIYMDQTKFPIVNAELSFFGH

AHLGTGDPYTPGFPSFNHTQFPPSRSSGLPNIPVQTISRAAAEKLFGNMEGDCPSDWKTD

STCRMVTSESKNVKLTVSNVLKEIKILNIFGVIKGFVEPDHYVVVGAQRDAWGPGAAKSG

VGTALLLKLAQMFSDMVLKDGFQPSRSIIFASWSAGDFGSVGATEWLEGYLSSLHLKAFT

YINLDKAVLGTSNFKVSASPLLYTLIEKTMQNVKHPVTGQFLYQDSNWASKVEKLTLDNA

AFPFLAYSGIPAVSFCFCEDTDYPYLGTTMDTYKELIERIPELNKVARAAAEVAGQFVIK

LTHDVELNLDYERYNSQLLSFVRDLNQYRADIKEMGLSLQWLYSARGDFFRATSRLTTDF

GNAEKTDRFVMKKLNDRVMRVEYHFLSPYVSPKESPFRHVFWGSGSHTLPALLENLKLRK

QNNGAFNETLFRNQLALATWTIQGAANALSGDVWDIDNEF

>sp|P02786|TFR1_HUMAN 215 NPGGYVAYSKAATVTGKL

MMDQARSAFSNLFGGEPLSYTRFSLARQVDGDNSHVEMKLAVDEEENADNNTKANVTKPK

RCSGSICYGTIAVIVFFLIGFMIGYLGYCKGVEPKTECERLAGTESPVREEPGEDFPAAR

RLYWDDLKRKLSEKLDSTDFTGTIKLLNENSYVPREAGSQKDENLALYVENQFREFKLSK

VWRDQHFVKIQVKDSAQNSVIIVDKNGRLVYLVENPGGYVAYSKAATVTGKLVHANFGTK

KDFEDLYTPVNGSIVIVRAGKITFAEKVANAESLNAIGVLIYMDQTKFPIVNAELSFFGH

AHLGTGDPYTPGFPSFNHTQFPPSRSSGLPNIPVQTISRAAAEKLFGNMEGDCPSDWKTD

STCRMVTSESKNVKLTVSNVLKEIKILNIFGVIKGFVEPDHYVVVGAQRDAWGPGAAKSG

VGTALLLKLAQMFSDMVLKDGFQPSRSIIFASWSAGDFGSVGATEWLEGYLSSLHLKAFT

YINLDKAVLGTSNFKVSASPLLYTLIEKTMQNVKHPVTGQFLYQDSNWASKVEKLTLDNA

AFPFLAYSGIPAVSFCFCEDTDYPYLGTTMDTYKELIERIPELNKVARAAAEVAGQFVIK

LTHDVELNLDYERYNSQLLSFVRDLNQYRADIKEMGLSLQWLYSARGDFFRATSRLTTDF

GNAEKTDRFVMKKLNDRVMRVEYHFLSPYVSPKESPFRHVFWGSGSHTLPALLENLKLRK

QNNGAFNETLFRNQLALATWTIQGAANALSGDVWDIDNEF

>sp|P02786|TFR1_HUMAN 615 NSQLLSFVRDLNQYRADI

MMDQARSAFSNLFGGEPLSYTRFSLARQVDGDNSHVEMKLAVDEEENADNNTKANVTKPK

RCSGSICYGTIAVIVFFLIGFMIGYLGYCKGVEPKTECERLAGTESPVREEPGEDFPAAR

RLYWDDLKRKLSEKLDSTDFTGTIKLLNENSYVPREAGSQKDENLALYVENQFREFKLSK

VWRDQHFVKIQVKDSAQNSVIIVDKNGRLVYLVENPGGYVAYSKAATVTGKLVHANFGTK

KDFEDLYTPVNGSIVIVRAGKITFAEKVANAESLNAIGVLIYMDQTKFPIVNAELSFFGH

AHLGTGDPYTPGFPSFNHTQFPPSRSSGLPNIPVQTISRAAAEKLFGNMEGDCPSDWKTD

STCRMVTSESKNVKLTVSNVLKEIKILNIFGVIKGFVEPDHYVVVGAQRDAWGPGAAKSG

VGTALLLKLAQMFSDMVLKDGFQPSRSIIFASWSAGDFGSVGATEWLEGYLSSLHLKAFT

YINLDKAVLGTSNFKVSASPLLYTLIEKTMQNVKHPVTGQFLYQDSNWASKVEKLTLDNA

AFPFLAYSGIPAVSFCFCEDTDYPYLGTTMDTYKELIERIPELNKVARAAAEVAGQFVIK

LTHDVELNLDYERYNSQLLSFVRDLNQYRADIKEMGLSLQWLYSARGDFFRATSRLTTDF

GNAEKTDRFVMKKLNDRVMRVEYHFLSPYVSPKESPFRHVFWGSGSHTLPALLENLKLRK

QNNGAFNETLFRNQLALATWTIQGAANALSGDVWDIDNEF

>sp|P02786|TFR1_HUMAN 174 REFKLSKVWRDQ

MMDQARSAFSNLFGGEPLSYTRFSLARQVDGDNSHVEMKLAVDEEENADNNTKANVTKPK

RCSGSICYGTIAVIVFFLIGFMIGYLGYCKGVEPKTECERLAGTESPVREEPGEDFPAAR

RLYWDDLKRKLSEKLDSTDFTGTIKLLNENSYVPREAGSQKDENLALYVENQFREFKLSK

VWRDQHFVKIQVKDSAQNSVIIVDKNGRLVYLVENPGGYVAYSKAATVTGKLVHANFGTK

KDFEDLYTPVNGSIVIVRAGKITFAEKVANAESLNAIGVLIYMDQTKFPIVNAELSFFGH

AHLGTGDPYTPGFPSFNHTQFPPSRSSGLPNIPVQTISRAAAEKLFGNMEGDCPSDWKTD

STCRMVTSESKNVKLTVSNVLKEIKILNIFGVIKGFVEPDHYVVVGAQRDAWGPGAAKSG

VGTALLLKLAQMFSDMVLKDGFQPSRSIIFASWSAGDFGSVGATEWLEGYLSSLHLKAFT

YINLDKAVLGTSNFKVSASPLLYTLIEKTMQNVKHPVTGQFLYQDSNWASKVEKLTLDNA

AFPFLAYSGIPAVSFCFCEDTDYPYLGTTMDTYKELIERIPELNKVARAAAEVAGQFVIK

LTHDVELNLDYERYNSQLLSFVRDLNQYRADIKEMGLSLQWLYSARGDFFRATSRLTTDF

GNAEKTDRFVMKKLNDRVMRVEYHFLSPYVSPKESPFRHVFWGSGSHTLPALLENLKLRK

QNNGAFNETLFRNQLALATWTIQGAANALSGDVWDIDNEF

>sp|P02786|TFR1_HUMAN 174 REFKLSKVWRDQH

MMDQARSAFSNLFGGEPLSYTRFSLARQVDGDNSHVEMKLAVDEEENADNNTKANVTKPK

RCSGSICYGTIAVIVFFLIGFMIGYLGYCKGVEPKTECERLAGTESPVREEPGEDFPAAR

RLYWDDLKRKLSEKLDSTDFTGTIKLLNENSYVPREAGSQKDENLALYVENQFREFKLSK

VWRDQHFVKIQVKDSAQNSVIIVDKNGRLVYLVENPGGYVAYSKAATVTGKLVHANFGTK

KDFEDLYTPVNGSIVIVRAGKITFAEKVANAESLNAIGVLIYMDQTKFPIVNAELSFFGH

AHLGTGDPYTPGFPSFNHTQFPPSRSSGLPNIPVQTISRAAAEKLFGNMEGDCPSDWKTD

STCRMVTSESKNVKLTVSNVLKEIKILNIFGVIKGFVEPDHYVVVGAQRDAWGPGAAKSG

VGTALLLKLAQMFSDMVLKDGFQPSRSIIFASWSAGDFGSVGATEWLEGYLSSLHLKAFT

YINLDKAVLGTSNFKVSASPLLYTLIEKTMQNVKHPVTGQFLYQDSNWASKVEKLTLDNA

AFPFLAYSGIPAVSFCFCEDTDYPYLGTTMDTYKELIERIPELNKVARAAAEVAGQFVIK

LTHDVELNLDYERYNSQLLSFVRDLNQYRADIKEMGLSLQWLYSARGDFFRATSRLTTDF

GNAEKTDRFVMKKLNDRVMRVEYHFLSPYVSPKESPFRHVFWGSGSHTLPALLENLKLRK

QNNGAFNETLFRNQLALATWTIQGAANALSGDVWDIDNEF

>sp|P02786|TFR1_HUMAN 680 RVEYHFLSPYVSPKESP

MMDQARSAFSNLFGGEPLSYTRFSLARQVDGDNSHVEMKLAVDEEENADNNTKANVTKPK

RCSGSICYGTIAVIVFFLIGFMIGYLGYCKGVEPKTECERLAGTESPVREEPGEDFPAAR

RLYWDDLKRKLSEKLDSTDFTGTIKLLNENSYVPREAGSQKDENLALYVENQFREFKLSK

VWRDQHFVKIQVKDSAQNSVIIVDKNGRLVYLVENPGGYVAYSKAATVTGKLVHANFGTK

KDFEDLYTPVNGSIVIVRAGKITFAEKVANAESLNAIGVLIYMDQTKFPIVNAELSFFGH

AHLGTGDPYTPGFPSFNHTQFPPSRSSGLPNIPVQTISRAAAEKLFGNMEGDCPSDWKTD

STCRMVTSESKNVKLTVSNVLKEIKILNIFGVIKGFVEPDHYVVVGAQRDAWGPGAAKSG

VGTALLLKLAQMFSDMVLKDGFQPSRSIIFASWSAGDFGSVGATEWLEGYLSSLHLKAFT

YINLDKAVLGTSNFKVSASPLLYTLIEKTMQNVKHPVTGQFLYQDSNWASKVEKLTLDNA

AFPFLAYSGIPAVSFCFCEDTDYPYLGTTMDTYKELIERIPELNKVARAAAEVAGQFVIK

LTHDVELNLDYERYNSQLLSFVRDLNQYRADIKEMGLSLQWLYSARGDFFRATSRLTTDF

GNAEKTDRFVMKKLNDRVMRVEYHFLSPYVSPKESPFRHVFWGSGSHTLPALLENLKLRK

QNNGAFNETLFRNQLALATWTIQGAANALSGDVWDIDNEF

>sp|P02786|TFR1_HUMAN 141 TGTIKLLNENSYVP

MMDQARSAFSNLFGGEPLSYTRFSLARQVDGDNSHVEMKLAVDEEENADNNTKANVTKPK

RCSGSICYGTIAVIVFFLIGFMIGYLGYCKGVEPKTECERLAGTESPVREEPGEDFPAAR

RLYWDDLKRKLSEKLDSTDFTGTIKLLNENSYVPREAGSQKDENLALYVENQFREFKLSK

VWRDQHFVKIQVKDSAQNSVIIVDKNGRLVYLVENPGGYVAYSKAATVTGKLVHANFGTK

KDFEDLYTPVNGSIVIVRAGKITFAEKVANAESLNAIGVLIYMDQTKFPIVNAELSFFGH

AHLGTGDPYTPGFPSFNHTQFPPSRSSGLPNIPVQTISRAAAEKLFGNMEGDCPSDWKTD

STCRMVTSESKNVKLTVSNVLKEIKILNIFGVIKGFVEPDHYVVVGAQRDAWGPGAAKSG

VGTALLLKLAQMFSDMVLKDGFQPSRSIIFASWSAGDFGSVGATEWLEGYLSSLHLKAFT

YINLDKAVLGTSNFKVSASPLLYTLIEKTMQNVKHPVTGQFLYQDSNWASKVEKLTLDNA

AFPFLAYSGIPAVSFCFCEDTDYPYLGTTMDTYKELIERIPELNKVARAAAEVAGQFVIK

LTHDVELNLDYERYNSQLLSFVRDLNQYRADIKEMGLSLQWLYSARGDFFRATSRLTTDF

GNAEKTDRFVMKKLNDRVMRVEYHFLSPYVSPKESPFRHVFWGSGSHTLPALLENLKLRK

QNNGAFNETLFRNQLALATWTIQGAANALSGDVWDIDNEF

>sp|P02786|TFR1_HUMAN 143 TIKLLNENSYVPR

MMDQARSAFSNLFGGEPLSYTRFSLARQVDGDNSHVEMKLAVDEEENADNNTKANVTKPK

RCSGSICYGTIAVIVFFLIGFMIGYLGYCKGVEPKTECERLAGTESPVREEPGEDFPAAR

RLYWDDLKRKLSEKLDSTDFTGTIKLLNENSYVPREAGSQKDENLALYVENQFREFKLSK

VWRDQHFVKIQVKDSAQNSVIIVDKNGRLVYLVENPGGYVAYSKAATVTGKLVHANFGTK

KDFEDLYTPVNGSIVIVRAGKITFAEKVANAESLNAIGVLIYMDQTKFPIVNAELSFFGH

AHLGTGDPYTPGFPSFNHTQFPPSRSSGLPNIPVQTISRAAAEKLFGNMEGDCPSDWKTD

STCRMVTSESKNVKLTVSNVLKEIKILNIFGVIKGFVEPDHYVVVGAQRDAWGPGAAKSG

VGTALLLKLAQMFSDMVLKDGFQPSRSIIFASWSAGDFGSVGATEWLEGYLSSLHLKAFT

YINLDKAVLGTSNFKVSASPLLYTLIEKTMQNVKHPVTGQFLYQDSNWASKVEKLTLDNA

AFPFLAYSGIPAVSFCFCEDTDYPYLGTTMDTYKELIERIPELNKVARAAAEVAGQFVIK

LTHDVELNLDYERYNSQLLSFVRDLNQYRADIKEMGLSLQWLYSARGDFFRATSRLTTDF

GNAEKTDRFVMKKLNDRVMRVEYHFLSPYVSPKESPFRHVFWGSGSHTLPALLENLKLRK

QNNGAFNETLFRNQLALATWTIQGAANALSGDVWDIDNEF

>sp|P02786|TFR1_HUMAN 397 VEPDHYVVVGAQRDA

MMDQARSAFSNLFGGEPLSYTRFSLARQVDGDNSHVEMKLAVDEEENADNNTKANVTKPK

RCSGSICYGTIAVIVFFLIGFMIGYLGYCKGVEPKTECERLAGTESPVREEPGEDFPAAR

RLYWDDLKRKLSEKLDSTDFTGTIKLLNENSYVPREAGSQKDENLALYVENQFREFKLSK

VWRDQHFVKIQVKDSAQNSVIIVDKNGRLVYLVENPGGYVAYSKAATVTGKLVHANFGTK

KDFEDLYTPVNGSIVIVRAGKITFAEKVANAESLNAIGVLIYMDQTKFPIVNAELSFFGH

AHLGTGDPYTPGFPSFNHTQFPPSRSSGLPNIPVQTISRAAAEKLFGNMEGDCPSDWKTD

STCRMVTSESKNVKLTVSNVLKEIKILNIFGVIKGFVEPDHYVVVGAQRDAWGPGAAKSG

VGTALLLKLAQMFSDMVLKDGFQPSRSIIFASWSAGDFGSVGATEWLEGYLSSLHLKAFT

YINLDKAVLGTSNFKVSASPLLYTLIEKTMQNVKHPVTGQFLYQDSNWASKVEKLTLDNA

AFPFLAYSGIPAVSFCFCEDTDYPYLGTTMDTYKELIERIPELNKVARAAAEVAGQFVIK

LTHDVELNLDYERYNSQLLSFVRDLNQYRADIKEMGLSLQWLYSARGDFFRATSRLTTDF

GNAEKTDRFVMKKLNDRVMRVEYHFLSPYVSPKESPFRHVFWGSGSHTLPALLENLKLRK

QNNGAFNETLFRNQLALATWTIQGAANALSGDVWDIDNEF

>sp|P02786|TFR1_HUMAN 219 YVAYSKAATVTGKL

MMDQARSAFSNLFGGEPLSYTRFSLARQVDGDNSHVEMKLAVDEEENADNNTKANVTKPK

RCSGSICYGTIAVIVFFLIGFMIGYLGYCKGVEPKTECERLAGTESPVREEPGEDFPAAR

RLYWDDLKRKLSEKLDSTDFTGTIKLLNENSYVPREAGSQKDENLALYVENQFREFKLSK

VWRDQHFVKIQVKDSAQNSVIIVDKNGRLVYLVENPGGYVAYSKAATVTGKLVHANFGTK

KDFEDLYTPVNGSIVIVRAGKITFAEKVANAESLNAIGVLIYMDQTKFPIVNAELSFFGH

AHLGTGDPYTPGFPSFNHTQFPPSRSSGLPNIPVQTISRAAAEKLFGNMEGDCPSDWKTD

STCRMVTSESKNVKLTVSNVLKEIKILNIFGVIKGFVEPDHYVVVGAQRDAWGPGAAKSG

VGTALLLKLAQMFSDMVLKDGFQPSRSIIFASWSAGDFGSVGATEWLEGYLSSLHLKAFT

YINLDKAVLGTSNFKVSASPLLYTLIEKTMQNVKHPVTGQFLYQDSNWASKVEKLTLDNA

AFPFLAYSGIPAVSFCFCEDTDYPYLGTTMDTYKELIERIPELNKVARAAAEVAGQFVIK

LTHDVELNLDYERYNSQLLSFVRDLNQYRADIKEMGLSLQWLYSARGDFFRATSRLTTDF

GNAEKTDRFVMKKLNDRVMRVEYHFLSPYVSPKESPFRHVFWGSGSHTLPALLENLKLRK

QNNGAFNETLFRNQLALATWTIQGAANALSGDVWDIDNEF

>sp|Q14003|KCNC3_HUMAN 507 GDMYPKTWSGMLVGALCALAGVLTI

MLSSVCVSSFRGRQGASKQQPAPPPQPPESPPPPPLPPQQQQPAQPGPAASPAGPPAPRG

PGGRRAEPCPGLPAAAMGRHGGGGGDSGKIVINVGGVRHETYRSTLRTLPGTRLAGLTEP

EAAARFDYDPGADEFFFDRHPGVFAYVLNYYRTGKLHCPADVCGPLFEEELGFWGIDETD

VEACCWMTYRQHRDAEEALDSFEAPDPAGAANAANAAGAHDGGLDDEAGAGGGGLDGAGG

ELKRLCFQDAGGGAGGPPGGAGGAGGTWWRRWQPRVWALFEDPYSSRAARYVAFASLFFI

LISITTFCLETHEGFIHISNKTVTQASPIPGAPPENITNVEVETEPFLTYVEGVCVVWFT

FEFLMRITFCPDKVEFLKSSLNIIDCVAILPFYLEVGLSGLSSKAAKDVLGFLRVVRFVR

ILRIFKLTRHFVGLRVLGHTLRASTNEFLLLIIFLALGVLIFATMIYYAERIGADPDDIL

GSNHTYFKNIPIGFWWAVVTMTTLGYGDMYPKTWSGMLVGALCALAGVLTIAMPVPVIVN

NFGMYYSLAMAKQKLPKKKNKHIPRPPQPGSPNYCKPDPPPPPPPHPHHGSGGISPPPPI

TPPSMGVTVAGAYPAGPHTHPGLLRGGAGGLGIMGLPPLPAPGEPCPLAQEEVIEINRAD

PRPNGDPAAAALAHEDCPAIDQPAMSPEDKSPITPGSRGRYSRDRACFLLTDYAPSPDGS

IRKATGAPPLPPQDWRKPGPPSFLPDLNANAAAWISP

>sp|O95461|LARGE_HUMAN 166 NPLHFHLIADSIAEQIL

MLGICRGRRKFLAASLSLLCIPAITWIYLFSGSFEDGKPVSLSPLESQAHSPRYTASSQR

ERESLEVRMREVEEENRALRRQLSLAQGRAPSHRRGNHSKTYSMEEGTGDSENLRAGIVA

GNSSECGQQPVVEKCETIHVAIVCAGYNASRDVVTLVKSVLFHRRNPLHFHLIADSIAEQ

ILATLFQTWMVPAVRVDFYNADELKSEVSWIPNKHYSGIYGLMKLVLTKTLPANLERVIV

LDTDITFATDIAELWAVFHKFKGQQVLGLVENQSDWYLGNLWKNHRPWPALGRGYNTGVI

LLLLDKLRKMKWEQMWRLTAERELMGMLSTSLADQDIFNAVIKQNPFLVYQLPCFWNVQL

SDHTRSEQCYRDVSDLKVIHWNSPKKLRVKNKHVEFFRNLYLTFLEYDGNLLRRELFGCP

SEADVNSENLQKQLSELDEDDLCYEFRRERFTVHRTHLYFLHYEYEPAADSTDVTLVAQL

SMDRLQMLEAICKHWEGPISLALYLSDAEAQQFLRYAQGSEVLMSRHNVGYHIVYKEGQF

YPVNLLRNVAMKHISTPYMFLSDIDFLPMYGLYEYLRKSVIQLDLANTKKAMIVPAFETL

RYRLSFPKSKAELLSMLDMGTLFTFRYHVWTKGHAPTNFAKWRTATTPYRVEWEADFEPY

VVVRRDCPEYDRRFVGFGWNKVAHIMELDVQEYEFIVLPNAYMIHMPHAPSFDITKFRSN

KQYRICLKTLKEEFQQDMSRRYGFAALKYLTAENNS

>sp|Q92499|DDX1_HUMAN 730 GYLPNQLFRTF

MAAFSEMGVMPEIAQAVEEMDWLLPTDIQAESIPLILGGGDVLMAAETGSGKTGAFSIPV

IQIVYETLKDQQEGKKGKTTIKTGASVLNKWQMNPYDRGSAFAIGSDGLCCQSREVKEWH

GCRATKGLMKGKHYYEVSCHDQGLCRVGWSTMQASLDLGTDKFGFGFGGTGKKSHNKQFD

NYGEEFTMHDTIGCYLDIDKGHVKFSKNGKDLGLAFEIPPHMKNQALFPACVLKNAELKF

NFGEEEFKFPPKDGFVALSKAPDGYIVKSQHSGNAQVTQTKFLPNAPKALIVEPSRELAE

QTLNNIKQFKKYIDNPKLRELLIIGGVAARDQLSVLENGVDIVVGTPGRLDDLVSTGKLN

LSQVRFLVLDEADGLLSQGYSDFINRMHNQIPQVTSDGKRLQVIVCSATLHSFDVKKLSE

KIMHFPTWVDLKGEDSVPDTVHHVVVPVNPKTDRLWERLGKSHIRTDDVHAKDNTRPGAN

SPEMWSEAIKILKGEYAVRAIKEHKMDQAIIFCRTKIDCDNLEQYFIQQGGGPDKKGHQF

SCVCLHGDRKPHERKQNLERFKKGDVRFLICTDVAARGIDIHGVPYVINVTLPDEKQNYV

HRIGRVGRAERMGLAISLVATEKEKVWYHVCSSRGKGCYNTRLKEDGGCTIWYNEMQLLS

EIEEHLNCTISQVEPDIKVPVDEFDGKVTYGQKRAAGGGSYKGHVDILAPTVQELAALEK

EAQTSFLHLGYLPNQLFRTF

>sp|P08238|HS90B_HUMAN 69 KELKIDIIPNPQER

MPEEVHHGEEEVETFAFQAEIAQLMSLIINTFYSNKEIFLRELISNASDALDKIRYESLT

DPSKLDSGKELKIDIIPNPQERTLTLVDTGIGMTKADLINNLGTIAKSGTKAFMEALQAG

ADISMIGQFGVGFYSAYLVAEKVVVITKHNDDEQYAWESSAGGSFTVRADHGEPIGRGTK

VILHLKEDQTEYLEERRVKEVVKKHSQFIGYPITLYLEKEREKEISDDEAEEEKGEKEEE

DKDDEEKPKIEDVGSDEEDDSGKDKKKKTKKIKEKYIDQEELNKTKPIWTRNPDDITQEE

YGEFYKSLTNDWEDHLAVKHFSVEGQLEFRALLFIPRRAPFDLFENKKKKNNIKLYVRRV

FIMDSCDELIPEYLNFIRGVVDSEDLPLNISREMLQQSKILKVIRKNIVKKCLELFSELA

EDKENYKKFYEAFSKNLKLGIHEDSTNRRRLSELLRYHTSQSGDEMTSLSEYVSRMKETQ

KSIYYITGESKEQVANSAFVERVRKRGFEVVYMTEPIDEYCVQQLKEFDGKSLVSVTKEG

LELPEDEEEKKKMEESKAKFENLCKLMKEILDKKVEKVTISNRLVSSPCCIVTSTYGWTA

NMERIMKAQALRDNSTMGYMMAKKHLEINPDHPIVETLRQKAEADKNDKAVKDLVVLLFE

TALLSSGFSLEDPQTHSNRIYRMIKLGLGIDEDEVAAEEPNAAVPDEIPPLEGDEDASRM

EEVD

>sp|O75326|SEM7A_HUMAN 519 ISIYSSERSVLQ

MTPPPPGRAAPSAPRARVPGPPARLGLPLRLRLLLLLWAAAASAQGHLRSGPRIFAVWKG

HVGQDRVDFGQTEPHTVLFHEPGSSSVWVGGRGKVYLFDFPEGKNASVRTVNIGSTKGSC

LDKRDCENYITLLERRSEGLLACGTNARHPSCWNLVNGTVVPLGEMRGYAPFSPDENSLV

LFEGDEVYSTIRKQEYNGKIPRFRRIRGESELYTSDTVMQNPQFIKATIVHQDQAYDDKI

YYFFREDNPDKNPEAPLNVSRVAQLCRGDQGGESSLSVSKWNTFLKAMLVCSDAATNKNF

NRLQDVFLLPDPSGQWRDTRVYGVFSNPWNYSAVCVYSLGDIDKVFRTSSLKGYHSSLPN

PRPGKCLPDQQPIPTETFQVADRHPEVAQRVEPMGPLKTPLFHSKYHYQKVAVHRMQASH

GETFHVLYLTTDRGTIHKVVEPGEQEHSFAFNIMEIQPFRRAAAIQTMSLDAERRKLYVS

SQWEVSQVPLDLCEVYGGGCHGCLMSRDPYCGWDQGRCISIYSSERSVLQSINPAEPHKE

CPNPKPDKAPLQKVSLAPNSRYYLSCPMESRHATYSWRHKENVEQSCEPGHQSPNCILFI

ENLTAQQYGHYFCEAQEGSYFREAQHWQLLPEDGIMAEHLLGHACALAASLWLGVLPTLT

LGLLVH

>sp|P40967|PME17_HUMAN 44 WNRQLYPEWTEAQRLD

MDLVLKRCLLHLAVIGALLAVGATKVPRNQDWLGVSRQLRTKAWNRQLYPEWTEAQRLDC

WRGGQVSLKVSNDGPTLIGANASFSIALNFPGSQKVLPDGQVIWVNNTIINGSQVWGGQP

VYPQETDDACIFPDGGPCPSGSWSQKRSFVYVWKTWGQYWQVLGGPVSGLSIGTGRAMLG

THTMEVTVYHRRGSRSYVPLAHSSSAFTITDQVPFSVSVSQLRALDGGNKHFLRNQPLTF

ALQLHDPSGYLAEADLSYTWDFGDSSGTLISRALVVTHTYLEPGPVTAQVVLQAAIPLTS

CGSSPVPGTTDGHRPTAEAPNTTAGQVPTTEVVGTTPGQAPTAEPSGTTSVQVPTTEVIS

TAPVQMPTAESTGMTPEKVPVSEVMGTTLAEMSTPEATGMTPAEVSIVVLSGTTAAQVTT

TEWVETTARELPIPEPEGPDASSIMSTESITGSLGPLLDGTATLRLVKRQVPLDCVLYRY

GSFSVTLDIVQGIESAEILQAVPSGEGDAFELTVSCQGGLPKEACMEISSPGCQPPAQRL

CQPVLPSPACQLVLHQILKGGSGTYCLNVSLADTNSLAVVSTQLIMPGQEAGLGQVPLIV

GILLVLMAVVLASLIYRRRLMKQDFSVPQLPHSSSHWLRLPRIFCSCPIGENSPLLSGQQ

V

>sp|P11021|GRP78_HUMAN 195 VMRIINEPTAAAIAYG

MKLSLVAAMLLLLSAARAEEEDKKEDVGTVVGIDLGTTYSCVGVFKNGRVEIIANDQGNR

ITPSYVAFTPEGERLIGDAAKNQLTSNPENTVFDAKRLIGRTWNDPSVQQDIKFLPFKVV

EKKTKPYIQVDIGGGQTKTFAPEEISAMVLTKMKETAEAYLGKKVTHAVVTVPAYFNDAQ

RQATKDAGTIAGLNVMRIINEPTAAAIAYGLDKREGEKNILVFDLGGGTFDVSLLTIDNG

VFEVVATNGDTHLGGEDFDQRVMEHFIKLYKKKTGKDVRKDNRAVQKLRREVEKAKRALS

SQHQARIEIESFYEGEDFSETLTRAKFEELNMDLFRSTMKPVQKVLEDSDLKKSDIDEIV

LVGGSTRIPKIQQLVKEFFNGKEPSRGINPDEAVAYGAAVQAGVLSGDQDTGDLVLLDVC

PLTLGIETVGGVMTKLIPRNTVVPTKKSQIFSTASDNQPTVTIKVYEGERPLTKDNHLLG

TFDLTGIPPAPRGVPQIEVTFEIDVNGILRVTAEDKGTGNKNKITITNDQNRLTPEEIER

MVNDAEKFAEEDKKLKERIDTRNELESYAYSLKNQIGDKEKLGGKLSSEDKETMEKAVEE

KIEWLESHQDADIEDFKAKKKELEEIVQPIISKLYGSAGPPPTGEEDTAEKDEL

>sp|P11021|GRP78_HUMAN 443 VPTKKSQIFSTASDNQPTVT

MKLSLVAAMLLLLSAARAEEEDKKEDVGTVVGIDLGTTYSCVGVFKNGRVEIIANDQGNR

ITPSYVAFTPEGERLIGDAAKNQLTSNPENTVFDAKRLIGRTWNDPSVQQDIKFLPFKVV

EKKTKPYIQVDIGGGQTKTFAPEEISAMVLTKMKETAEAYLGKKVTHAVVTVPAYFNDAQ

RQATKDAGTIAGLNVMRIINEPTAAAIAYGLDKREGEKNILVFDLGGGTFDVSLLTIDNG

VFEVVATNGDTHLGGEDFDQRVMEHFIKLYKKKTGKDVRKDNRAVQKLRREVEKAKRALS

SQHQARIEIESFYEGEDFSETLTRAKFEELNMDLFRSTMKPVQKVLEDSDLKKSDIDEIV

LVGGSTRIPKIQQLVKEFFNGKEPSRGINPDEAVAYGAAVQAGVLSGDQDTGDLVLLDVC

PLTLGIETVGGVMTKLIPRNTVVPTKKSQIFSTASDNQPTVTIKVYEGERPLTKDNHLLG

TFDLTGIPPAPRGVPQIEVTFEIDVNGILRVTAEDKGTGNKNKITITNDQNRLTPEEIER

MVNDAEKFAEEDKKLKERIDTRNELESYAYSLKNQIGDKEKLGGKLSSEDKETMEKAVEE

KIEWLESHQDADIEDFKAKKKELEEIVQPIISKLYGSAGPPPTGEEDTAEKDEL

>sp|P11142|HSP7C_HUMAN 307 ADLFRGTLD

MSKGPAVGIDLGTTYSCVGVFQHGKVEIIANDQGNRTTPSYVAFTDTERLIGDAAKNQVA

MNPTNTVFDAKRLIGRRFDDAVVQSDMKHWPFMVVNDAGRPKVQVEYKGETKSFYPEEVS

SMVLTKMKEIAEAYLGKTVTNAVVTVPAYFNDSQRQATKDAGTIAGLNVLRIINEPTAAA

IAYGLDKKVGAERNVLIFDLGGGTFDVSILTIEDGIFEVKSTAGDTHLGGEDFDNRMVNH

FIAEFKRKHKKDISENKRAVRRLRTACERAKRTLSSSTQASIEIDSLYEGIDFYTSITRA

RFEELNADLFRGTLDPVEKALRDAKLDKSQIHDIVLVGGSTRIPKIQKLLQDFFNGKELN

KSINPDEAVAYGAAVQAAILSGDKSENVQDLLLLDVTPLSLGIETAGGVMTVLIKRNTTI

PTKQTQTFTTYSDNQPGVLIQVYEGERAMTKDNNLLGKFELTGIPPAPRGVPQIEVTFDI

DANGILNVSAVDKSTGKENKITITNDKGRLSKEDIERMVQEAEKYKAEDEKQRDKVSSKN

SLESYAFNMKATVEDEKLQGKINDEDKQKILDKCNEIINWLDKNQTAEKEEFEHQQKELE

KVCNPIITKLYQSAGGMPGGMPGGFPGGGAPPSGGASSGPTIEEVD

>sp|P11142|HSP7C_HUMAN 482 ANGILNVSAVDKSTGKE

MSKGPAVGIDLGTTYSCVGVFQHGKVEIIANDQGNRTTPSYVAFTDTERLIGDAAKNQVA

MNPTNTVFDAKRLIGRRFDDAVVQSDMKHWPFMVVNDAGRPKVQVEYKGETKSFYPEEVS

SMVLTKMKEIAEAYLGKTVTNAVVTVPAYFNDSQRQATKDAGTIAGLNVLRIINEPTAAA

IAYGLDKKVGAERNVLIFDLGGGTFDVSILTIEDGIFEVKSTAGDTHLGGEDFDNRMVNH

FIAEFKRKHKKDISENKRAVRRLRTACERAKRTLSSSTQASIEIDSLYEGIDFYTSITRA

RFEELNADLFRGTLDPVEKALRDAKLDKSQIHDIVLVGGSTRIPKIQKLLQDFFNGKELN

KSINPDEAVAYGAAVQAAILSGDKSENVQDLLLLDVTPLSLGIETAGGVMTVLIKRNTTI

PTKQTQTFTTYSDNQPGVLIQVYEGERAMTKDNNLLGKFELTGIPPAPRGVPQIEVTFDI

DANGILNVSAVDKSTGKENKITITNDKGRLSKEDIERMVQEAEKYKAEDEKQRDKVSSKN

SLESYAFNMKATVEDEKLQGKINDEDKQKILDKCNEIINWLDKNQTAEKEEFEHQQKELE

KVCNPIITKLYQSAGGMPGGMPGGFPGGGAPPSGGASSGPTIEEVD

>sp|P11142|HSP7C_HUMAN 574 CNEIINWLDKNQ

MSKGPAVGIDLGTTYSCVGVFQHGKVEIIANDQGNRTTPSYVAFTDTERLIGDAAKNQVA

MNPTNTVFDAKRLIGRRFDDAVVQSDMKHWPFMVVNDAGRPKVQVEYKGETKSFYPEEVS

SMVLTKMKEIAEAYLGKTVTNAVVTVPAYFNDSQRQATKDAGTIAGLNVLRIINEPTAAA

IAYGLDKKVGAERNVLIFDLGGGTFDVSILTIEDGIFEVKSTAGDTHLGGEDFDNRMVNH

FIAEFKRKHKKDISENKRAVRRLRTACERAKRTLSSSTQASIEIDSLYEGIDFYTSITRA

RFEELNADLFRGTLDPVEKALRDAKLDKSQIHDIVLVGGSTRIPKIQKLLQDFFNGKELN

KSINPDEAVAYGAAVQAAILSGDKSENVQDLLLLDVTPLSLGIETAGGVMTVLIKRNTTI

PTKQTQTFTTYSDNQPGVLIQVYEGERAMTKDNNLLGKFELTGIPPAPRGVPQIEVTFDI

DANGILNVSAVDKSTGKENKITITNDKGRLSKEDIERMVQEAEKYKAEDEKQRDKVSSKN

SLESYAFNMKATVEDEKLQGKINDEDKQKILDKCNEIINWLDKNQTAEKEEFEHQQKELE

KVCNPIITKLYQSAGGMPGGMPGGFPGGGAPPSGGASSGPTIEEVD

>sp|P11142|HSP7C_HUMAN 444 EGERAMTKDNNLLGKFEL

MSKGPAVGIDLGTTYSCVGVFQHGKVEIIANDQGNRTTPSYVAFTDTERLIGDAAKNQVA

MNPTNTVFDAKRLIGRRFDDAVVQSDMKHWPFMVVNDAGRPKVQVEYKGETKSFYPEEVS

SMVLTKMKEIAEAYLGKTVTNAVVTVPAYFNDSQRQATKDAGTIAGLNVLRIINEPTAAA

IAYGLDKKVGAERNVLIFDLGGGTFDVSILTIEDGIFEVKSTAGDTHLGGEDFDNRMVNH

FIAEFKRKHKKDISENKRAVRRLRTACERAKRTLSSSTQASIEIDSLYEGIDFYTSITRA

RFEELNADLFRGTLDPVEKALRDAKLDKSQIHDIVLVGGSTRIPKIQKLLQDFFNGKELN

KSINPDEAVAYGAAVQAAILSGDKSENVQDLLLLDVTPLSLGIETAGGVMTVLIKRNTTI

PTKQTQTFTTYSDNQPGVLIQVYEGERAMTKDNNLLGKFELTGIPPAPRGVPQIEVTFDI

DANGILNVSAVDKSTGKENKITITNDKGRLSKEDIERMVQEAEKYKAEDEKQRDKVSSKN

SLESYAFNMKATVEDEKLQGKINDEDKQKILDKCNEIINWLDKNQTAEKEEFEHQQKELE

KVCNPIITKLYQSAGGMPGGMPGGFPGGGAPPSGGASSGPTIEEVD

>sp|P11142|HSP7C_HUMAN 445 GERAMTKDNNLLG

MSKGPAVGIDLGTTYSCVGVFQHGKVEIIANDQGNRTTPSYVAFTDTERLIGDAAKNQVA

MNPTNTVFDAKRLIGRRFDDAVVQSDMKHWPFMVVNDAGRPKVQVEYKGETKSFYPEEVS

SMVLTKMKEIAEAYLGKTVTNAVVTVPAYFNDSQRQATKDAGTIAGLNVLRIINEPTAAA

IAYGLDKKVGAERNVLIFDLGGGTFDVSILTIEDGIFEVKSTAGDTHLGGEDFDNRMVNH

FIAEFKRKHKKDISENKRAVRRLRTACERAKRTLSSSTQASIEIDSLYEGIDFYTSITRA

RFEELNADLFRGTLDPVEKALRDAKLDKSQIHDIVLVGGSTRIPKIQKLLQDFFNGKELN

KSINPDEAVAYGAAVQAAILSGDKSENVQDLLLLDVTPLSLGIETAGGVMTVLIKRNTTI

PTKQTQTFTTYSDNQPGVLIQVYEGERAMTKDNNLLGKFELTGIPPAPRGVPQIEVTFDI

DANGILNVSAVDKSTGKENKITITNDKGRLSKEDIERMVQEAEKYKAEDEKQRDKVSSKN

SLESYAFNMKATVEDEKLQGKINDEDKQKILDKCNEIINWLDKNQTAEKEEFEHQQKELE

KVCNPIITKLYQSAGGMPGGMPGGFPGGGAPPSGGASSGPTIEEVD

>sp|P11142|HSP7C_HUMAN 484 GILNVSAVDKSTGK

MSKGPAVGIDLGTTYSCVGVFQHGKVEIIANDQGNRTTPSYVAFTDTERLIGDAAKNQVA

MNPTNTVFDAKRLIGRRFDDAVVQSDMKHWPFMVVNDAGRPKVQVEYKGETKSFYPEEVS

SMVLTKMKEIAEAYLGKTVTNAVVTVPAYFNDSQRQATKDAGTIAGLNVLRIINEPTAAA

IAYGLDKKVGAERNVLIFDLGGGTFDVSILTIEDGIFEVKSTAGDTHLGGEDFDNRMVNH

FIAEFKRKHKKDISENKRAVRRLRTACERAKRTLSSSTQASIEIDSLYEGIDFYTSITRA

RFEELNADLFRGTLDPVEKALRDAKLDKSQIHDIVLVGGSTRIPKIQKLLQDFFNGKELN

KSINPDEAVAYGAAVQAAILSGDKSENVQDLLLLDVTPLSLGIETAGGVMTVLIKRNTTI

PTKQTQTFTTYSDNQPGVLIQVYEGERAMTKDNNLLGKFELTGIPPAPRGVPQIEVTFDI

DANGILNVSAVDKSTGKENKITITNDKGRLSKEDIERMVQEAEKYKAEDEKQRDKVSSKN

SLESYAFNMKATVEDEKLQGKINDEDKQKILDKCNEIINWLDKNQTAEKEEFEHQQKELE

KVCNPIITKLYQSAGGMPGGMPGGFPGGGAPPSGGASSGPTIEEVD

>sp|P11142|HSP7C_HUMAN 484 GILNVSAVDKSTGKE

MSKGPAVGIDLGTTYSCVGVFQHGKVEIIANDQGNRTTPSYVAFTDTERLIGDAAKNQVA

MNPTNTVFDAKRLIGRRFDDAVVQSDMKHWPFMVVNDAGRPKVQVEYKGETKSFYPEEVS

SMVLTKMKEIAEAYLGKTVTNAVVTVPAYFNDSQRQATKDAGTIAGLNVLRIINEPTAAA

IAYGLDKKVGAERNVLIFDLGGGTFDVSILTIEDGIFEVKSTAGDTHLGGEDFDNRMVNH

FIAEFKRKHKKDISENKRAVRRLRTACERAKRTLSSSTQASIEIDSLYEGIDFYTSITRA

RFEELNADLFRGTLDPVEKALRDAKLDKSQIHDIVLVGGSTRIPKIQKLLQDFFNGKELN

KSINPDEAVAYGAAVQAAILSGDKSENVQDLLLLDVTPLSLGIETAGGVMTVLIKRNTTI

PTKQTQTFTTYSDNQPGVLIQVYEGERAMTKDNNLLGKFELTGIPPAPRGVPQIEVTFDI

DANGILNVSAVDKSTGKENKITITNDKGRLSKEDIERMVQEAEKYKAEDEKQRDKVSSKN

SLESYAFNMKATVEDEKLQGKINDEDKQKILDKCNEIINWLDKNQTAEKEEFEHQQKELE

KVCNPIITKLYQSAGGMPGGMPGGFPGGGAPPSGGASSGPTIEEVD

>sp|P11142|HSP7C_HUMAN 291 IDFYTSITRARFEE

MSKGPAVGIDLGTTYSCVGVFQHGKVEIIANDQGNRTTPSYVAFTDTERLIGDAAKNQVA

MNPTNTVFDAKRLIGRRFDDAVVQSDMKHWPFMVVNDAGRPKVQVEYKGETKSFYPEEVS

SMVLTKMKEIAEAYLGKTVTNAVVTVPAYFNDSQRQATKDAGTIAGLNVLRIINEPTAAA

IAYGLDKKVGAERNVLIFDLGGGTFDVSILTIEDGIFEVKSTAGDTHLGGEDFDNRMVNH

FIAEFKRKHKKDISENKRAVRRLRTACERAKRTLSSSTQASIEIDSLYEGIDFYTSITRA

RFEELNADLFRGTLDPVEKALRDAKLDKSQIHDIVLVGGSTRIPKIQKLLQDFFNGKELN

KSINPDEAVAYGAAVQAAILSGDKSENVQDLLLLDVTPLSLGIETAGGVMTVLIKRNTTI

PTKQTQTFTTYSDNQPGVLIQVYEGERAMTKDNNLLGKFELTGIPPAPRGVPQIEVTFDI

DANGILNVSAVDKSTGKENKITITNDKGRLSKEDIERMVQEAEKYKAEDEKQRDKVSSKN

SLESYAFNMKATVEDEKLQGKINDEDKQKILDKCNEIINWLDKNQTAEKEEFEHQQKELE

KVCNPIITKLYQSAGGMPGGMPGGFPGGGAPPSGGASSGPTIEEVD

>sp|P11142|HSP7C_HUMAN 62 NPTNTVFDAKRLIGRRFD

MSKGPAVGIDLGTTYSCVGVFQHGKVEIIANDQGNRTTPSYVAFTDTERLIGDAAKNQVA

MNPTNTVFDAKRLIGRRFDDAVVQSDMKHWPFMVVNDAGRPKVQVEYKGETKSFYPEEVS

SMVLTKMKEIAEAYLGKTVTNAVVTVPAYFNDSQRQATKDAGTIAGLNVLRIINEPTAAA

IAYGLDKKVGAERNVLIFDLGGGTFDVSILTIEDGIFEVKSTAGDTHLGGEDFDNRMVNH

FIAEFKRKHKKDISENKRAVRRLRTACERAKRTLSSSTQASIEIDSLYEGIDFYTSITRA

RFEELNADLFRGTLDPVEKALRDAKLDKSQIHDIVLVGGSTRIPKIQKLLQDFFNGKELN

KSINPDEAVAYGAAVQAAILSGDKSENVQDLLLLDVTPLSLGIETAGGVMTVLIKRNTTI

PTKQTQTFTTYSDNQPGVLIQVYEGERAMTKDNNLLGKFELTGIPPAPRGVPQIEVTFDI

DANGILNVSAVDKSTGKENKITITNDKGRLSKEDIERMVQEAEKYKAEDEKQRDKVSSKN

SLESYAFNMKATVEDEKLQGKINDEDKQKILDKCNEIINWLDKNQTAEKEEFEHQQKELE

KVCNPIITKLYQSAGGMPGGMPGGFPGGGAPPSGGASSGPTIEEVD

>sp|P11142|HSP7C_HUMAN 168 NVLRIINEPTAAAIAYG

MSKGPAVGIDLGTTYSCVGVFQHGKVEIIANDQGNRTTPSYVAFTDTERLIGDAAKNQVA

MNPTNTVFDAKRLIGRRFDDAVVQSDMKHWPFMVVNDAGRPKVQVEYKGETKSFYPEEVS

SMVLTKMKEIAEAYLGKTVTNAVVTVPAYFNDSQRQATKDAGTIAGLNVLRIINEPTAAA

IAYGLDKKVGAERNVLIFDLGGGTFDVSILTIEDGIFEVKSTAGDTHLGGEDFDNRMVNH

FIAEFKRKHKKDISENKRAVRRLRTACERAKRTLSSSTQASIEIDSLYEGIDFYTSITRA

RFEELNADLFRGTLDPVEKALRDAKLDKSQIHDIVLVGGSTRIPKIQKLLQDFFNGKELN

KSINPDEAVAYGAAVQAAILSGDKSENVQDLLLLDVTPLSLGIETAGGVMTVLIKRNTTI

PTKQTQTFTTYSDNQPGVLIQVYEGERAMTKDNNLLGKFELTGIPPAPRGVPQIEVTFDI

DANGILNVSAVDKSTGKENKITITNDKGRLSKEDIERMVQEAEKYKAEDEKQRDKVSSKN

SLESYAFNMKATVEDEKLQGKINDEDKQKILDKCNEIINWLDKNQTAEKEEFEHQQKELE

KVCNPIITKLYQSAGGMPGGMPGGFPGGGAPPSGGASSGPTIEEVD

>sp|P11142|HSP7C_HUMAN 38 TPSYVAFTDTERLIG

MSKGPAVGIDLGTTYSCVGVFQHGKVEIIANDQGNRTTPSYVAFTDTERLIGDAAKNQVA

MNPTNTVFDAKRLIGRRFDDAVVQSDMKHWPFMVVNDAGRPKVQVEYKGETKSFYPEEVS

SMVLTKMKEIAEAYLGKTVTNAVVTVPAYFNDSQRQATKDAGTIAGLNVLRIINEPTAAA

IAYGLDKKVGAERNVLIFDLGGGTFDVSILTIEDGIFEVKSTAGDTHLGGEDFDNRMVNH

FIAEFKRKHKKDISENKRAVRRLRTACERAKRTLSSSTQASIEIDSLYEGIDFYTSITRA

RFEELNADLFRGTLDPVEKALRDAKLDKSQIHDIVLVGGSTRIPKIQKLLQDFFNGKELN

KSINPDEAVAYGAAVQAAILSGDKSENVQDLLLLDVTPLSLGIETAGGVMTVLIKRNTTI

PTKQTQTFTTYSDNQPGVLIQVYEGERAMTKDNNLLGKFELTGIPPAPRGVPQIEVTFDI

DANGILNVSAVDKSTGKENKITITNDKGRLSKEDIERMVQEAEKYKAEDEKQRDKVSSKN

SLESYAFNMKATVEDEKLQGKINDEDKQKILDKCNEIINWLDKNQTAEKEEFEHQQKELE

KVCNPIITKLYQSAGGMPGGMPGGFPGGGAPPSGGASSGPTIEEVD

>sp|P11142|HSP7C_HUMAN 38 TPSYVAFTDTERLIGDA

MSKGPAVGIDLGTTYSCVGVFQHGKVEIIANDQGNRTTPSYVAFTDTERLIGDAAKNQVA

MNPTNTVFDAKRLIGRRFDDAVVQSDMKHWPFMVVNDAGRPKVQVEYKGETKSFYPEEVS

SMVLTKMKEIAEAYLGKTVTNAVVTVPAYFNDSQRQATKDAGTIAGLNVLRIINEPTAAA

IAYGLDKKVGAERNVLIFDLGGGTFDVSILTIEDGIFEVKSTAGDTHLGGEDFDNRMVNH

FIAEFKRKHKKDISENKRAVRRLRTACERAKRTLSSSTQASIEIDSLYEGIDFYTSITRA

RFEELNADLFRGTLDPVEKALRDAKLDKSQIHDIVLVGGSTRIPKIQKLLQDFFNGKELN

KSINPDEAVAYGAAVQAAILSGDKSENVQDLLLLDVTPLSLGIETAGGVMTVLIKRNTTI

PTKQTQTFTTYSDNQPGVLIQVYEGERAMTKDNNLLGKFELTGIPPAPRGVPQIEVTFDI

DANGILNVSAVDKSTGKENKITITNDKGRLSKEDIERMVQEAEKYKAEDEKQRDKVSSKN

SLESYAFNMKATVEDEKLQGKINDEDKQKILDKCNEIINWLDKNQTAEKEEFEHQQKELE

KVCNPIITKLYQSAGGMPGGMPGGFPGGGAPPSGGASSGPTIEEVD

>sp|P11142|HSP7C_HUMAN 238 VNHFIAEFKRKHK

MSKGPAVGIDLGTTYSCVGVFQHGKVEIIANDQGNRTTPSYVAFTDTERLIGDAAKNQVA

MNPTNTVFDAKRLIGRRFDDAVVQSDMKHWPFMVVNDAGRPKVQVEYKGETKSFYPEEVS

SMVLTKMKEIAEAYLGKTVTNAVVTVPAYFNDSQRQATKDAGTIAGLNVLRIINEPTAAA

IAYGLDKKVGAERNVLIFDLGGGTFDVSILTIEDGIFEVKSTAGDTHLGGEDFDNRMVNH

FIAEFKRKHKKDISENKRAVRRLRTACERAKRTLSSSTQASIEIDSLYEGIDFYTSITRA

RFEELNADLFRGTLDPVEKALRDAKLDKSQIHDIVLVGGSTRIPKIQKLLQDFFNGKELN

KSINPDEAVAYGAAVQAAILSGDKSENVQDLLLLDVTPLSLGIETAGGVMTVLIKRNTTI

PTKQTQTFTTYSDNQPGVLIQVYEGERAMTKDNNLLGKFELTGIPPAPRGVPQIEVTFDI

DANGILNVSAVDKSTGKENKITITNDKGRLSKEDIERMVQEAEKYKAEDEKQRDKVSSKN

SLESYAFNMKATVEDEKLQGKINDEDKQKILDKCNEIINWLDKNQTAEKEEFEHQQKELE

KVCNPIITKLYQSAGGMPGGMPGGFPGGGAPPSGGASSGPTIEEVD

>sp|P11142|HSP7C_HUMAN 238 VNHFIAEFKRKHKKD

MSKGPAVGIDLGTTYSCVGVFQHGKVEIIANDQGNRTTPSYVAFTDTERLIGDAAKNQVA

MNPTNTVFDAKRLIGRRFDDAVVQSDMKHWPFMVVNDAGRPKVQVEYKGETKSFYPEEVS

SMVLTKMKEIAEAYLGKTVTNAVVTVPAYFNDSQRQATKDAGTIAGLNVLRIINEPTAAA

IAYGLDKKVGAERNVLIFDLGGGTFDVSILTIEDGIFEVKSTAGDTHLGGEDFDNRMVNH

FIAEFKRKHKKDISENKRAVRRLRTACERAKRTLSSSTQASIEIDSLYEGIDFYTSITRA

RFEELNADLFRGTLDPVEKALRDAKLDKSQIHDIVLVGGSTRIPKIQKLLQDFFNGKELN

KSINPDEAVAYGAAVQAAILSGDKSENVQDLLLLDVTPLSLGIETAGGVMTVLIKRNTTI

PTKQTQTFTTYSDNQPGVLIQVYEGERAMTKDNNLLGKFELTGIPPAPRGVPQIEVTFDI

DANGILNVSAVDKSTGKENKITITNDKGRLSKEDIERMVQEAEKYKAEDEKQRDKVSSKN

SLESYAFNMKATVEDEKLQGKINDEDKQKILDKCNEIINWLDKNQTAEKEEFEHQQKELE

KVCNPIITKLYQSAGGMPGGMPGGFPGGGAPPSGGASSGPTIEEVD

>sp|P17066|HSP76_HUMAN 447 GERAMTKDNNLLGRFE

MQAPRELAVGIDLGTTYSCVGVFQQGRVEILANDQGNRTTPSYVAFTDTERLVGDAAKSQ

AALNPHNTVFDAKRLIGRKFADTTVQSDMKHWPFRVVSEGGKPKVRVCYRGEDKTFYPEE

ISSMVLSKMKETAEAYLGQPVKHAVITVPAYFNDSQRQATKDAGAIAGLNVLRIINEPTA

AAIAYGLDRRGAGERNVLIFDLGGGTFDVSVLSIDAGVFEVKATAGDTHLGGEDFDNRLV

NHFMEEFRRKHGKDLSGNKRALRRLRTACERAKRTLSSSTQATLEIDSLFEGVDFYTSIT

RARFEELCSDLFRSTLEPVEKALRDAKLDKAQIHDVVLVGGSTRIPKVQKLLQDFFNGKE

LNKSINPDEAVAYGAAVQAAVLMGDKCEKVQDLLLLDVAPLSLGLETAGGVMTTLIQRNA

TIPTKQTQTFTTYSDNQPGVFIQVYEGERAMTKDNNLLGRFELSGIPPAPRGVPQIEVTF

DIDANGILSVTATDRSTGKANKITITNDKGRLSKEEVERMVHEAEQYKAEDEAQRDRVAA

KNSLEAHVFHVKGSLQEESLRDKIPEEDRRKMQDKCREVLAWLEHNQLAEKEEYEHQKRE

LEQICRPIFSRLYGGPGVPGGSSCGTQARQGDPSTGPIIEEVD

>sp|P17066|HSP76_HUMAN 175 INEPTAAAIAYGLDR

MQAPRELAVGIDLGTTYSCVGVFQQGRVEILANDQGNRTTPSYVAFTDTERLVGDAAKSQ

AALNPHNTVFDAKRLIGRKFADTTVQSDMKHWPFRVVSEGGKPKVRVCYRGEDKTFYPEE

ISSMVLSKMKETAEAYLGQPVKHAVITVPAYFNDSQRQATKDAGAIAGLNVLRIINEPTA

AAIAYGLDRRGAGERNVLIFDLGGGTFDVSVLSIDAGVFEVKATAGDTHLGGEDFDNRLV

NHFMEEFRRKHGKDLSGNKRALRRLRTACERAKRTLSSSTQATLEIDSLFEGVDFYTSIT

RARFEELCSDLFRSTLEPVEKALRDAKLDKAQIHDVVLVGGSTRIPKVQKLLQDFFNGKE

LNKSINPDEAVAYGAAVQAAVLMGDKCEKVQDLLLLDVAPLSLGLETAGGVMTTLIQRNA

TIPTKQTQTFTTYSDNQPGVFIQVYEGERAMTKDNNLLGRFELSGIPPAPRGVPQIEVTF

DIDANGILSVTATDRSTGKANKITITNDKGRLSKEEVERMVHEAEQYKAEDEAQRDRVAA

KNSLEAHVFHVKGSLQEESLRDKIPEEDRRKMQDKCREVLAWLEHNQLAEKEEYEHQKRE

LEQICRPIFSRLYGGPGVPGGSSCGTQARQGDPSTGPIIEEVD

>sp|Q6UWB1|I27RA_HUMAN 385 VGVPYRITVTAVSASG

MRGGRGAPFWLWPLPKLALLPLLWVLFQRTRPQGSAGPLQCYGVGPLGDLNCSWEPLGDL

GAPSELHLQSQKYRSNKTQTVAVAAGRSWVAIPREQLTMSDKLLVWGTKAGQPLWPPVFV

NLETQMKPNAPRLGPDVDFSEDDPLEATVHWAPPTWPSHKVLICQFHYRRCQEAAWTLLE

PELKTIPLTPVEIQDLELATGYKVYGRCRMEKEEDLWGEWSPILSFQTPPSAPKDVWVSG

NLCGTPGGEEPLLLWKAPGPCVQVSYKVWFWVGGRELSPEGITCCCSLIPSGAEWARVSA

VNATSWEPLTNLSLVCLDSASAPRSVAVSSIAGSTELLVTWQPGPGEPLEHVVDWARDGD

PLEKLNWVRLPPGNLSALLPGNFTVGVPYRITVTAVSASGLASASSVWGFREELAPLVGP

TLWRLQDAPPGTPAIAWGEVPRHQLRGHLTHYTLCAQSGTSPSVCMNVSGNTQSVTLPDL

PWGPCELWVTASTIAGQGPPGPILRLHLPDNTLRWKVLPGILFLWGLFLLGCGLSLATSG

RCYHLRHKVLPRWVWEKVPDPANSSSGQPHMEQVPEAQPLGDLPILEVEEMEPPPVMESS

QPAQATAPLDSGYEKHFLPTPEELGLLGPPRPQVLA

>sp|P04844|RPN2_HUMAN 443 TGQEVVFVAEPDNKNVYK

MAPPGSSTVFLLALTIIASTWALTPTHYLTKHDVERLKASLDRPFTNLESAFYSIVGLSS

LGAQVPDAKKACTYIRSNLDPSNVDSLFYAAQASQALSGCEISISNETKDLLLAAVSEDS

SVTQIYHAVAALSGFGLPLASQEALSALTARLSKEETVLATVQALQTASHLSQQADLRSI

VEEIEDLVARLDELGGVYLQFEEGLETTALFVAATYKLMDHVGTEPSIKEDQVIQLMNAI

FSKKNFESLSEAFSVASAAAVLSHNRYHVPVVVVPEGSASDTHEQAILRLQVTNVLSQPL

TQATVKLEHAKSVASRATVLQKTSFTPVGDVFELNFMNVKFSSGYYDFLVEVEGDNRYIA

NTVELRVKISTEVGITNVDLSTVDKDQSIAPKTTRVTYPAKAKGTFIADSHQNFALFFQL

VDVNTGAELTPHQTFVRLHNQKTGQEVVFVAEPDNKNVYKFELDTSERKIEFDSASGTYT

LYLIIGDATLKNPILWNVADVVIKFPEEEAPSTVLSQNLFTPKQEIQHLFREPEKRPPTV

VSNTFTALILSPLLLLFALWIRIGANVSNFTFAPSTIIFHLGHAAMLGLMYVYWTQLNMF

QTLKYLAILGSVTFLAGNRMLAQQAVKRTAH

>sp|P13796|PLSL_HUMAN 581 NNAKYAISMARKIGA

MARGSVSDEEMMELREAFAKVDTDGNGYISFNELNDLFKAACLPLPGYRVREITENLMAT

GDLDQDGRISFDEFIKIFHGLKSTDVAKTFRKAINKKEGICAIGGTSEQSSVGTQHSYSE

EEKYAFVNWINKALENDPDCRHVIPMNPNTNDLFNAVGDGIVLCKMINLSVPDTIDERTI

NKKKLTPFTIQENLNLALNSASAIGCHVVNIGAEDLKEGKPYLVLGLLWQVIKIGLFADI

ELSRNEALIALLREGESLEDLMKLSPEELLLRWANYHLENAGCNKIGNFSTDIKDSKAYY

HLLEQVAPKGDEEGVPAVVIDMSGLREKDDIQRAECMLQQAERLGCRQFVTATDVVRGNP

KLNLAFIANLFNRYPALHKPENQDIDWGALEGETREERTFRNWMNSLGVNPRVNHLYSDL

SDALVIFQLYEKIKVPVDWNRVNKPPYPKLGGNMKKLENCNYAVELGKNQAKFSLVGIGG

QDLNEGNRTLTLALIWQLMRRYTLNILEEIGGGQKVNDDIIVNWVNETLREAEKSSSISS

FKDPKISTSLPVLDLIDAIQPGSINYDLLKTENLNDDEKLNNAKYAISMARKIGARVYAL

PEDLVEVNPKMVMTVFACLMGKGMKRV

>sp|Q9UHD9|UBQL2_HUMAN 439 NPDTLSAMSNPRAMQ

MAENGESSGPPRPSRGPAAAQGSAAAPAEPKIIKVTVKTPKEKEEFAVPENSSVQQFKEA

ISKRFKSQTDQLVLIFAGKILKDQDTLIQHGIHDGLTVHLVIKSQNRPQGQSTQPSNAAG

TNTTSASTPRSNSTPISTNSNPFGLGSLGGLAGLSSLGLSSTNFSELQSQMQQQLMASPE

MMIQIMENPFVQSMLSNPDLMRQLIMANPQMQQLIQRNPEISHLLNNPDIMRQTLEIARN

PAMMQEMMRNQDLALSNLESIPGGYNALRRMYTDIQEPMLNAAQEQFGGNPFASVGSSSS

SGEGTQPSRTENRDPLPNPWAPPPATQSSATTSTTTSTGSGSGNSSSNATGNTVAAANYV

ASIFSTPGMQSLLQQITENPQLIQNMLSAPYMRSMMQSLSQNPDLAAQMMLNSPLFTANP

QLQEQMRPQLPAFLQQMQNPDTLSAMSNPRAMQALMQIQQGLQTLATEAPGLIPSFTPGV

GVGVLGTAIGPVGPVTPIGPIGPIVPFTPIGPIGPIGPTGPAAPPGSTGSGGPTGPTVSS

AAPSETTSPTSESGPNQQFIQQMVQALAGANAPQLPNPEVRFQQQLEQLNAMGFLNREAN

LQALIATGGDINAAIERLLGSQPS

>sp|P22460|KCNA5_HUMAN 173 DGILYYYQSGGRLRRPVN

MEIALVPLENGGAMTVRGGDEARAGCGQATGGELQCPPTAGLSDGPKEPAPKGRGAQRDA

DSGVRPLPPLPDPGVRPLPPLPEELPRPRRPPPEDEEEEGDPGLGTVEDQALGTASLHHQ

RVHINISGLRFETQLGTLAQFPNTLLGDPAKRLRYFDPLRNEYFFDRNRPSFDGILYYYQ

SGGRLRRPVNVSLDVFADEIRFYQLGDEAMERFREDEGFIKEEEKPLPRNEFQRQVWLIF

EYPESSGSARAIAIVSVLVILISIITFCLETLPEFRDERELLRHPPAPHQPPAPAPGANG

SGVMAPPSGPTVAPLLPRTLADPFFIVETTCVIWFTFELLVRFFACPSKAGFSRNIMNII

DVVAIFPYFITLGTELAEQQPGGGGGGQNGQQAMSLAILRVIRLVRVFRIFKLSRHSKGL

QILGKTLQASMRELGLLIFFLFIGVILFSSAVYFAEADNQGTHFSSIPDAFWWAVVTMTT

VGYGDMRPITVGGKIVGSLCAIAGVLTIALPVPVIVSNFNYFYHRETDHEEPAVLKEEQG

TQSQGPGLDRGVQRKVSGSRGSFCKAGGTLENADSARRGSCPLEKCNVKAKSNVDLRRSL

YALCLDTSRETDL

>sp|P04843|RPN1_HUMAN 109 SGRFFTVKLPVALDPGAK

MEAPAAGLFLLLLLGTWAPAPGSASSEAPPLINEDVKRTVDLSSHLAKVTAEVVLAHLGG

GSTSRATSFLLALEPELEARLAHLGVQVKGEDEEENNLEVRETKIKGKSGRFFTVKLPVA

LDPGAKISVIVETVYTHVLHPYPTQITQSEKQFVVFEGNHYFYSPYPTKTQTMRVKLASR

NVESYTKLGNPTRSEDLLDYGPFRDVPAYSQDTFKVHYENNSPFLTITSMTRVIEVSHWG

NIAVEENVDLKHTGAVLKGPFSRYDYQRQPDSGISSIRSFKTILPAAAQDVYYRDEIGNV

STSHLLILDDSVEMEIRPRFPLFGGWKTHYIVGYNLPSYEYLYNLGDQYALKMRFVDHVF

DEQVIDSLTVKIILPEGAKNIEIDSPYEISRAPDELHYTYLDTFGRPVIVAYKKNLVEQH

IQDIVVHYTFNKVLMLQEPLLVVAAFYILFFTVIIYVRLDFSITKDPAAEARMKVACITE

QVLTLVNKRIGLYRHFDETVNRYKQSRDISTLNSGKKSLETEHKALTSEIALLQSRLKTE

GSDLCDRVSEMQKLDAQVKELVLKSAVEAERLVAGKLKKDTYIENEKLISGKRQELVTKI

DHILDAL

>sp|O75083|WDR1_HUMAN 67 APSGFYIASGDVSGKLR

MPYEIKKVFASLPQVERGVSKIIGGDPKGNNFLYTNGKCVILRNIDNPALADIYTEHAHQ

VVVAKYAPSGFYIASGDVSGKLRIWDTTQKEHLLKYEYQPFAGKIKDIAWTEDSKRIAVV

GEGREKFGAVFLWDSGSSVGEITGHNKVINSVDIKQSRPYRLATGSDDNCAAFFEGPPFK

FKFTIGDHSRFVNCVRFSPDGNRFATASADGQIYIYDGKTGEKVCALGGSKAHDGGIYAI

SWSPDSTHLLSASGDKTSKIWDVSVNSVVSTFPMGSTVLDQQLGCLWQKDHLLSVSLSGY

INYLDRNNPSKPLHVIKGHSKSIQCLTVHKNGGKSYIYSGSHDGHINYWDSETGENDSFA

GKGHTNQVSRMTVDESGQLISCSMDDTVRYTSLMLRDYSGQGVVKLDVQPKCVAVGPGGY

AVVVCIGQIVLLKDQRKCFSIDNPGYEPEVVAVHPGGDTVAIGGVDGNVRLYSILGTTLK

DEGKLLEAKGPVTDVAYSHDGAFLAVCDASKVVTVFSVADGYSENNVFYGHHAKIVCLAW

SPDNEHFASGGMDMMVYVWTLSDPETRVKIQDAHRLHHVSSLAWLDEHTLVTTSHDASVK

EWTITY

>sp|P28799|GRN_HUMAN 482 CPAGYTCNVKARSCEK

MWTLVSWVALTAGLVAGTRCPDGQFCPVACCLDPGGASYSCCRPLLDKWPTTLSRHLGGP

CQVDAHCSAGHSCIFTVSGTSSCCPFPEAVACGDGHHCCPRGFHCSADGRSCFQRSGNNS

VGAIQCPDSQFECPDFSTCCVMVDGSWGCCPMPQASCCEDRVHCCPHGAFCDLVHTRCIT

PTGTHPLAKKLPAQRTNRAVALSSSVMCPDARSRCPDGSTCCELPSGKYGCCPMPNATCC

SDHLHCCPQDTVCDLIQSKCLSKENATTDLLTKLPAHTVGDVKCDMEVSCPDGYTCCRLQ

SGAWGCCPFTQAVCCEDHIHCCPAGFTCDTQKGTCEQGPHQVPWMEKAPAHLSLPDPQAL

KRDVPCDNVSSCPSSDTCCQLTSGEWGCCPIPEAVCCSDHQHCCPQGYTCVAEGQCQRGS

EIVAGLEKMPARRASLSHPRDIGCDQHTSCPVGQTCCPSLGGSWACCQLPHAVCCEDRQH

CCPAGYTCNVKARSCEKEVVSAQPATFLARSPHVGVKDVECGEGHFCHDNQTCCRDNRQG

WACCPYRQGVCCADRRHCCPAGFRCAARGTKCLRREAPRWDAPLRDPALRQLL

>sp|P28799|GRN_HUMAN 482 CPAGYTCNVKARSCEKEV

MWTLVSWVALTAGLVAGTRCPDGQFCPVACCLDPGGASYSCCRPLLDKWPTTLSRHLGGP

CQVDAHCSAGHSCIFTVSGTSSCCPFPEAVACGDGHHCCPRGFHCSADGRSCFQRSGNNS

VGAIQCPDSQFECPDFSTCCVMVDGSWGCCPMPQASCCEDRVHCCPHGAFCDLVHTRCIT

PTGTHPLAKKLPAQRTNRAVALSSSVMCPDARSRCPDGSTCCELPSGKYGCCPMPNATCC

SDHLHCCPQDTVCDLIQSKCLSKENATTDLLTKLPAHTVGDVKCDMEVSCPDGYTCCRLQ

SGAWGCCPFTQAVCCEDHIHCCPAGFTCDTQKGTCEQGPHQVPWMEKAPAHLSLPDPQAL

KRDVPCDNVSSCPSSDTCCQLTSGEWGCCPIPEAVCCSDHQHCCPQGYTCVAEGQCQRGS

EIVAGLEKMPARRASLSHPRDIGCDQHTSCPVGQTCCPSLGGSWACCQLPHAVCCEDRQH

CCPAGYTCNVKARSCEKEVVSAQPATFLARSPHVGVKDVECGEGHFCHDNQTCCRDNRQG

WACCPYRQGVCCADRRHCCPAGFRCAARGTKCLRREAPRWDAPLRDPALRQLL

>sp|P31939|PUR9_HUMAN 17 LVEFARNLTALGLNLV

MAPGQLALFSVSDKTGLVEFARNLTALGLNLVASGGTAKALRDAGLAVRDVSELTGFPEM

LGGRVKTLHPAVHAGILARNIPEDNADMARLDFNLIRVVACNLYPFVKTVASPGVTVEEA

VEQIDIGGVTLLRAAAKNHARVTVVCEPEDYVVVSTEMQSSESKDTSLETRRQLALKAFT

HTAQYDEAISDYFRKQYSKGVSQMPLRYGMNPHQTPAQLYTLQPKLPITVLNGAPGFINL

CDALNAWQLVKELKEALGIPAAASFKHVSPAGAAVGIPLSEDEAKVCMVYDLYKTLTPIS

AAYARARGADRMSSFGDFVALSDVCDVPTAKIISREVSDGIIAPGYEEEALTILSKKKNG

NYCVLQMDQSYKPDENEVRTLFGLHLSQKRNNGVVDKSLFSNVVTKNKDLPESALRDLIV

ATIAVKYTQSNSVCYAKNGQVIGIGAGQQSRIHCTRLAGDKANYWWLRHHPQVLSMKFKT

GVKRAEISNAIDQYVTGTIGEDEDLIKWKALFEEVPELLTEAEKKEWVEKLTEVSISSDA

FFPFRDNVDRAKRSGVAYIAAPSGSAADKVVIEACDELGIILAHTNLRLFHH

>sp|Q6ZNA5|FRRS1_HUMAN 88 GPPIGSFTLIDSEVSQL

MAVSGFTLGTCILLLHISYVANYPNGKVTQSCHGMIPEHGHSPQSVPVHDIYVSQMTFRP

GDQIEVTLSGHPFKGFLLEARNAEDLNGPPIGSFTLIDSEVSQLLTCEDIQGSAVSHRSA

SKKTEIKVYWNAPSSAPNHTQFLVTVVEKYKIYWVKIPGPIISQPNAFPFTTPKATVVPL

PTLPPVSHLTKPFSASDCGNKKFCIRSPLNCDPEKEASCVFLSFTRDDQSVMVEMSGPSK

GYLSFALSHDQWMGDDDAYLCIHEDQTVYIQPSHLTGRSHPVMDSRDTLEDMAWRLADGV

MQCSFRRNITLPGVKNRFDLNTSYYIFLADGAANDGRIYKHSQQPLITYEKYDVTDSPKN

IGGSHSVLLLKVHGALMFVAWMTTVSIGVLVARFFKPVWSKAFLLGEAAWFQVHRMLMFT

TTVLTCIAFVMPFIYRGGWSRHAGYHPYLGCIVMTLAVLQPLLAVFRPPLHDPRRQMFNW

THWSMGTAARIIAVAAMFLGMDLPGLNLPDSWKTYAMTGFVAWHVGTEVVLEVHAYRLSR

KVEILDDDRIQILQSFTAVETEGHAFKKAVLAIYVCGNVTFLIIFLSAINHL

>sp|P32456|GBP2_HUMAN 439 QELKNKYYQVPRKGIQA

MAPEINLPGPMSLIDNTKGQLVVNPEALKILSAITQPVVVVAIVGLYRTGKSYLMNKLAG

KKNGFSLGSTVKSHTKGIWMWCVPHPKKPEHTLVLLDTEGLGDIEKGDNENDSWIFALAI

LLSSTFVYNSMGTINQQAMDQLHYVTELTDRIKANSSPGNNSVDDSADFVSFFPAFVWTL

RDFTLELEVDGEPITADDYLELSLKLRKGTDKKSKSFNDPRLCIRKFFPKRKCFVFDWPA

PKKYLAHLEQLKEEELNPDFIEQVAEFCSYILSHSNVKTLSGGIAVNGPRLESLVLTYVN

AISSGDLPCMENAVLALAQIENSAAVEKAIAHYEQQMGQKVQLPTETLQELLDLHRDSER

EAIEVFMKNSFKDVDQMFQRKLGAQLEARRDDFCKQNSKASSDCCMALLQDIFGPLEEDV

KQGTFSKPGGYRLFTQKLQELKNKYYQVPRKGIQAKEVLKKYLESKEDVADALLQTDQSL

SEKEKAIEVERIKAESAEAAKKMLEEIQKKNEEMMEQKEKSYQEHVKQLTEKMERDRAQL

MAEQEKTLALKLQEQERLLKEGFENESKRLQKDIWDIQMRSKSLEPICNIL

>sp|Q9UJ83|HACL1_HUMAN 146 AIPFVIEKAVRSSIYG

MPDSNFAERSEEQVSGAKVIAQALKTQDVEYIFGIVGIPVTEIAIAAQQLGIKYIGMRNE

QAACYAASAIGYLTSRPGVCLVVSGPGLIHALGGMANANMNCWPLLVIGGSSERNQETMG

AFQEFPQVEACRLYTKFSARPSSIEAIPFVIEKAVRSSIYGRPGACYVDIPADFVNLQVN

VNSIKYMERCMSPPISMAETSAVCTAASVIRNAKQPLLIIGKGAAYAHAEESIKKLVEQY

KLPFLPTPMGKGVVPDNHPYCVGAARSRALQFADVIVLFGARLNWILHFGLPPRYQPDVK

FIQVDICAEELGNNVKPAVTLLGNIHAVTKQLLEELDKTPWQYPPESKWWKTLREKMKSN

EAASKELASKKSLPMNYYTVFYHVQEQLPRDCFVVSEGANTMDIGRTVLQNYLPRHRLDA

GTFGTMGVGLGFAIAAAVVAKDRSPGQWIICVEGDSAFGFSGMEVETICRYNLPIILLVV

NNNGIYQGFDTDTWKEMLKFQDATAVVPPMCLLPNSHYEQVMTAFGGKGYFVQTPEELQK

SLRQSLADTTKPSLINIMIEPQATRKAQDFHWLTRSNM

>sp|Q14147|DHX34_HUMAN 367 IRFVVDSGKVKEM

MPPPRTREGRDRRDHHRAPSEEEALEKWDWNCPETRRLLEDAFFREEDYIRQGSEECQKF

WTFFERLQRFQNLKTSRKEEKDPGQPKHSIPALADLPRTYDPRYRINLSVLGPATRGSQG

LGRHLPAERVAEFRRALLHYLDFGQKQAFGRLAKLQRERAALPIAQYGNRILQTLKEHQV

VVVAGDTGCGKSTQVPQYLLAAGFSHVACTQPRRIACISLAKRVGFESLSQYGSQVGYQI

RFESTRSAATKIVFLTVGLLLRQIQREPSLPQYEVLIVDEVHERHLHNDFLLGVLQRLLP

TRPDLKVILMSATINISLFSSYFSNAPVVQVPGRLFPITVFDVAPPGVRKCILSTNIAET

SVTIDGIRFVVDSGKVKEMSYDPQAKLQRLQEFWISQASAEQRKGRAGRTGPGVCFRLYA

ESDYDAFAPYPVPEIRRVALDSLVLQMKSMSVGDPRTFPFIEPPPPASLETAILYLRDQG

ALDSSEALTPIGSLLAQLPVDVVIGKMLILGSMFSLVEPVLTIAAALSVQSPFTRSAQSS

PECCTPPASSLAAPRCCTHRSWRPATATEAETTRTR

>sp|P02748|CO9_HUMAN 465 APVLISQKLSPIYNLVPVK

MSACRSFAVAICILEISILTAQYTTSYDPELTESSGSASHIDCRMSPWSEWSQCDPCLRQ

MFRSRSIEVFGQFNGKRCTDAVGDRRQCVPTEPCEDAEDDCGNDFQCSTGRCIKMRLRCN

GDNDCGDFSDEDDCESEPRPPCRDRVVEESELARTAGYGINILGMDPLSTPFDNEFYNGL

CNRDRDGNTLTYYRRPWNVASLIYETKGEKNFRTEHYEEQIEAFKSIIQEKTSNFNAAIS

LKFTPTETNKAEQCCEETASSISLHGKGSFRFSYSKNETYQLFLSYSSKKEKMFLHVKGE

IHLGRFVMRNRDVVLTTTFVDDIKALPTTYEKGEYFAFLETYGTHYSSSGSLGGLYELIY

VLDKASMKRKGVELKDIKRCLGYHLDVSLAFSEISVGAEFNKDDCVKRGEGRAVNITSEN

LIDDVVSLIRGGTRKYAFELKEKLLRGTVIDVTDFVNWASSINDAPVLISQKLSPIYNLV

PVKMKNAHLKKQNLERAIEDYINEFSVRKCHTCQNGGTVILMDGKCLCACPFKFEGIACE

ISKQKISEGLPALEFPNEK

>sp|P17181|INAR1_HUMAN 271 GNHLYKWKQIPDCENVK

MMVVLLGATTLVLVAVAPWVLSAAAGGKNLKSPQKVEVDIIDDNFILRWNRSDESVGNVT

FSFDYQKTGMDNWIKLSGCQNITSTKCNFSSLKLNVYEEIKLRIRAEKENTSSWYEVDSF

TPFRKAQIGPPEVHLEAEDKAIVIHISPGTKDSVMWALDGLSFTYSLVIWKNSSGVEERI

ENIYSRHKIYKLSPETTYCLKVKAALLTSWKIGVYSPVHCIKTTVENELPPPENIEVSVQ

NQNYVLKWDYTYANMTFQVQWLHAFLKRNPGNHLYKWKQIPDCENVKTTQCVFPQNVFQK

GIYLLRVQASDGNNTSFWSEEIKFDTEIQAFLLPPVFNIRSLSDSFHIYIGAPKQSGNTP

VIQDYPLIYEIIFWENTSNAERKIIEKKTDVTVPNLKPLTVYCVKARAHTMDEKLNKSSV

FSDAVCEKTKPGNTSKIWLIVGICIALFALPFVIYAAKVFLRCINYVFFPSLKPSSSIDE

YFSEQPLKNLLLSTSEEQIEKCFIIENISTIATVEETNQTDEDHKKYSSQTSQDSGNYSN

EDESESKTSEELQQDFV

>sp|O75487|GPC4_HUMAN 211 VTRAFVAARTFAQGL

MARFGLPALLCTLAVLSAALLAAELKSKSCSEVRRLYVSKGFNKNDAPLHEINGDHLKIC

PQGSTCCSQEMEEKYSLQSKDDFKSVVSEQCNHLQAVFASRYKKFDEFFKELLENAEKSL

NDMFVKTYGHLYMQNSELFKDLFVELKRYYVVGNVNLEEMLNDFWARLLERMFRLVNSQY

HFTDEYLECVSKYTEQLKPFGDVPRKLKLQVTRAFVAARTFAQGLAVAGDVVSKVSVVNP

TAQCTHALLKMIYCSHCRGLVTVKPCYNYCSNIMRGCLANQGDLDFEWNNFIDAMLMVAE

RLEGPFNIESVMDPIDVKISDAIMNMQDNSVQVSQKVFQGCGPPKPLPAGRISRSISESA

FSARFRPHHPEERPTTAAGTSLDRLVTDVKEKLKQAKKFWSSLPSNVCNDERMAAGNGNE

DDCWNGKGKSRYLFAVTGNGLANQGNNPEVQVDTSKPDILILRQIMALRVMTSKMKNAYN

GNDVDFFDISDESSGEGSGSGCEYQQCPSEFDYNATDHAGKSANEKADSAGVRPGAQAYL

LTVFCILFLVMQREWR

>sp|O43405|COCH_HUMAN 176 IGQRRFNLQKNFVGKVAL

MSAAWIPALGLGVCLLLLPGPAGSEGAAPIAITCFTRGLDIRKEKADVLCPGGCPLEEFS

VYGNIVYASVSSICGAAVHRGVISNSGGPVRVYSLPGRENYSSVDANGIQSQMLSRWSAS

FTVTKGKSSTQEATGQAVSTAHPPTGKRLKKTPEKKTGNKDCKADIAFLIDGSFNIGQRR

FNLQKNFVGKVALMLGIGTEGPHVGLVQASEHPKIEFYLKNFTSAKDVLFAIKEVGFRGG

NSNTGKALKHTAQKFFTVDAGVRKGIPKVVVVFIDGWPSDDIEEAGIVAREFGVNVFIVS

VAKPIPEELGMVQDVTFVDKAVCRNNGFFSYHMPNWFGTTKYVKPLVQKLCTHEQMMCSK

TCYNSVNIAFLIDGSSSVGDSNFRLMLEFVSNIAKTFEISDIGAKIAAVQFTYDQRTEFS

FTDYSTKENVLAVIRNIRYMSGGTATGDAISFTVRNVFGPIRESPNKNFLVIVTDGQSYD

DVQGPAAAAHDAGITIFSVGVAWAPLDDLKDMASKPKESHAFFTREFTGLEPIVSDVIRG

ICRDFLESQQ

>sp|Q99832|TCPH_HUMAN 298 VATQYFADRDMFCAGRVP

MMPTPVILLKEGTDSSQGIPQLVSNISACQVIAEAVRTTLGPRGMDKLIVDGRGKATISN

DGATILKLLDVVHPAAKTLVDIAKSQDAEVGDGTTSVTLLAAEFLKQVKPYVEEGLHPQI

IIRAFRTATQLAVNKIKEIAVTVKKADKVEQRKLLEKCAMTALSSKLISQQKAFFAKMVV

DAVMMLDDLLQLKMIGIKKVQGGALEDSQLVAGVAFKKTFSYAGFEMQPKKYHNPKIALL

NVELELKAEKDNAEIRVHTVEDYQAIVDAEWNILYDKLEKIHHSGAKVVLSKLPIGDVAT

QYFADRDMFCAGRVPEEDLKRTMMACGGSIQTSVNALSADVLGRCQVFEETQIGGERYNF

FTGCPKAKTCTFILRGGAEQFMEETERSLHDAIMIVRRAIKNDSVVAGGGAIEMELSKYL

RDYSRTIPGKQQLLIGAYAKALEIIPRQLCDNAGFDATNILNKLRARHAQGGTWYGVDIN

NEDIADNFEAFVWEPAMVRINALTAASEAACLIVSVDETIKNPRSTVDAPTAAGRGRGRG

RPH

>sp|Q15758|AAAT_HUMAN 396 VAAVFIAQLSQQSLDFVK

MVADPPRDSKGLAAAEPTANGGLALASIEDQGAAAGGYCGSRDQVRRCLRANLLVLLTVV

AVVAGVALGLGVSGAGGALALGPERLSAFVFPGELLLRLLRMIILPLVVCSLIGGAASLD

PGALGRLGAWALLFFLVTTLLASALGVGLALALQPGAASAAINASVGAAGSAENAPSKEV

LDSFLDLARNIFPSNLVSAAFRSYSTTYEERNITGTRVKVPVGQEVEGMNILGLVVFAIV

FGVALRKLGPEGELLIRFFNSFNEATMVLVSWIMWYAPVGIMFLVAGKIVEMEDVGLLFA

RLGKYILCCLLGHAIHGLLVLPLIYFLFTRKNPYRFLWGIVTPLATAFGTSSSSATLPLM

MKCVEENNGVAKHISRFILPIGATVNMDGAALFQCVAAVFIAQLSQQSLDFVKIITILVT

ATASSVGAAGIPAGGVLTLAIILEAVNLPVDHISLILAVDWLVDRSCTVLNVEGDALGAG

LLQNYVDRTESRSTEPELIQVKSELPLDPLPVPTEEGNPLLKHYRGPAGDATVASEKESV

M

>sp|Q9HBE5|IL21R_HUMAN 143 EDPAFYMLKGKLQYEL

MPRGWAAPLLLLLLQGGWGCPDLVCYTDYLQTVICILEMWNLHPSTLTLTWQDQYEELKD

EATSCSLHRSAHNATHATYTCHMDVFHFMADDIFSVNITDQSGNYSQECGSFLLAESIKP

APPFNVTVTFSGQYNISWRSDYEDPAFYMLKGKLQYELQYRNRGDPWAVSPRRKLISVDS

RSVSLLPLEFRKDSSYELQVRAGPMPGSSYQGTWSEWSDPVIFQTQSEELKEGWNPHLLL

LLLLVIVFIPAFWSLKTHPLWRLWKKIWAVPSPERFFMPLYKGCSGDFKKWVGAPFTGSS

LELGPWSPEVPSTLEVYSCHPPRSPAKRLQLTELQEPAELVESDGVPKPSFWPTAQNSGG

SAYSEERDRPYGLVSIDTVTVLDAEGPCTWPCSCEDDGYPALDLDAGLEPSPGLEDPLLD

AGTTVLSCGCVSAGSPGLGGPLGSLLDRLKPPLADGEDWAGGLPWGGRSPGGVSESEAGS

PLAGLDMDTFDSGFVGSDCSSPVECDFTSPGDEGPPRSYLRQWVVIPPPLSSPGPQAS

>sp|O75131|CPNE3_HUMAN 404 VARFAAAATQQQTA

MAAQCVTKVALNVSCANLLDKDIGSKSDPLCVLFLNTSGQQWYEVERTERIKNCLNPQFS

KTFIIDYYFEVVQKLKFGVYDIDNKTIELSDDDFLGECECTLGQIVSSKKLTRPLVMKTG

RPAGKGSITISAEEIKDNRVVLFEMEARKLDNKDLFGKSDPYLEFHKQTSDGNWLMVHRT

EVVKNNLNPVWRPFKISLNSLCYGDMDKTIKVECYDYDNDGSHDLIGTFQTTMTKLKEAS

RSSPVEFECINEKKRQKKKSYKNSGVISVKQCEITVECTFLDYIMGGCQLNFTVGVDFTG

SNGDPRSPDSLHYISPNGVNEYLTALWSVGLVIQDYDADKMFPAFGFGAQIPPQWQVSHE

FPMNFNPSNPYCNGIQGIVEAYRSCLPQIKLYGPTNFSPIINHVARFAAAATQQQTASQY

FVLLIITDGVITDLDETRQAIVNASRLPMSIIIVGVGGADFSAMEFLDGDGGSLRSPLGE

VAIRDIVQFVPFRQFQNAPKEALAQCVLAEIPQQVVGYFNTYKLLPPKNPATKQQKQ

>sp|P78371|TCPB_HUMAN 60 SLMVTNDGATILKN

MASLSLAPVNIFKAGADEERAETARLTSFIGAIAIGDLVKSTLGPKGMDKILLSSGRDAS

LMVTNDGATILKNIGVDNPAAKVLVDMSRVQDDEVGDGTTSVTVLAAELLREAESLIAKK

IHPQTIIAGWREATKAAREALLSSAVDHGSDEVKFRQDLMNIAGTTLSSKLLTHHKDHFT

KLAVEAVLRLKGSGNLEAIHIIKKLGGSLADSYLDEGFLLDKKIGVNQPKRIENAKILIA

NTGMDTDKIKIFGSRVRVDSTAKVAEIEHAEKEKMKEKVERILKHGINCFINRQLIYNYP

EQLFGAAGVMAIEHADFAGVERLALVTGGEIASTFDHPELVKLGSCKLIEEVMIGEDKLI

HFSGVALGEACTIVLRGATQQILDEAERSLHDALCVLAQTVKDSRTVYGGGCSEMLMAHA

VTQLANRTPGKEAVAMESYAKALRMLPTIIADNAGYDSADLVAQLRAAHSEGNTTAGLDM

REGTIGDMAILGITESFQVKRQVLLSAAEAAEVILRVDNIIKAAPRKRVPDHHPC

>sp|P24557|THAS_HUMAN 406 PAFRFTREAAQDCEV

MEALGFLKLEVNGPMVTVALSVALLALLKWYSTSAFSRLEKLGLRHPKPSPFIGNLTFFR

QGFWESQMELRKLYGPLCGYYLGRRMFIVISEPDMIKQVLVENFSNFTNRMASGLEFKSV

ADSVLFLRDKRWEEVRGALMSAFSPEKLNEMVPLISQACDLLLAHLKRYAESGDAFDIQR

CYCNYTTDVVASVPFGTPVDSWQAPEDPFVKHCKRFFEFCIPRPILVLLLSFPSIMVPLA

RILPNKNRDELNGFFNKLIRNVIALRDQQAAEERRRDFLQMVLDARHSASPMGVQDFDIV

RDVFSSTGCKPNPSRQHQPSPMARPLTVDEIVGQAFIFLIAGYEIITNTLSFATYLLATN

PDCQEKLLREVDVFKEKHMAPEFCSLEEGLPYLDMVIAETLRMYPPAFRFTREAAQDCEV

LGQRIPAGAVLEMAVGALHHDPEHWPSPETFNPERFTAEARQQHRPFTYLPFGAGPRSCL

GVRLGLLEVKLTLLHVLHKFRFQACPETQVPLQLESKSALGPKNGVYIKIVSR

>sp|P14618|KPYM_HUMAN 101 DPILYRPVAVALDTKGP

MSKPHSEAGTAFIQTQQLHAAMADTFLEHMCRLDIDSPPITARNTGIICTIGPASRSVET

LKEMIKSGMNVARLNFSHGTHEYHAETIKNVRTATESFASDPILYRPVAVALDTKGPEIR

TGLIKGSGTAEVELKKGATLKITLDNAYMEKCDENILWLDYKNICKVVEVGSKIYVDDGL

ISLQVKQKGADFLVTEVENGGSLGSKKGVNLPGAAVDLPAVSEKDIQDLKFGVEQDVDMV

FASFIRKASDVHEVRKVLGEKGKNIKIISKIENHEGVRRFDEILEASDGIMVARGDLGIE

IPAEKVFLAQKMMIGRCNRAGKPVICATQMLESMIKKPRPTRAEGSDVANAVLDGADCIM

LSGETAKGDYPLEAVRMQHLIAREAEAAIYHLQLFEELRRLAPITSDPTEATAVGAVEAS

FKCCSGAIIVLTKSGRSAHQVARYRPRAPIIAVTRNPQTARQAHLYRGIFPVLCKDPVQE

AWAEDVDLRVNFAMNVGKARGFFKKGDVVIVLTGWRPGSGFTNTMRVVPVP

>sp|P14618|KPYM_HUMAN 101 DPILYRPVAVALDTKGPE

MSKPHSEAGTAFIQTQQLHAAMADTFLEHMCRLDIDSPPITARNTGIICTIGPASRSVET

LKEMIKSGMNVARLNFSHGTHEYHAETIKNVRTATESFASDPILYRPVAVALDTKGPEIR

TGLIKGSGTAEVELKKGATLKITLDNAYMEKCDENILWLDYKNICKVVEVGSKIYVDDGL

ISLQVKQKGADFLVTEVENGGSLGSKKGVNLPGAAVDLPAVSEKDIQDLKFGVEQDVDMV

FASFIRKASDVHEVRKVLGEKGKNIKIISKIENHEGVRRFDEILEASDGIMVARGDLGIE

IPAEKVFLAQKMMIGRCNRAGKPVICATQMLESMIKKPRPTRAEGSDVANAVLDGADCIM

LSGETAKGDYPLEAVRMQHLIAREAEAAIYHLQLFEELRRLAPITSDPTEATAVGAVEAS

FKCCSGAIIVLTKSGRSAHQVARYRPRAPIIAVTRNPQTARQAHLYRGIFPVLCKDPVQE

AWAEDVDLRVNFAMNVGKARGFFKKGDVVIVLTGWRPGSGFTNTMRVVPVP

>sp|P14618|KPYM_HUMAN 265 IKIISKIENHEGVR

MSKPHSEAGTAFIQTQQLHAAMADTFLEHMCRLDIDSPPITARNTGIICTIGPASRSVET

LKEMIKSGMNVARLNFSHGTHEYHAETIKNVRTATESFASDPILYRPVAVALDTKGPEIR

TGLIKGSGTAEVELKKGATLKITLDNAYMEKCDENILWLDYKNICKVVEVGSKIYVDDGL

ISLQVKQKGADFLVTEVENGGSLGSKKGVNLPGAAVDLPAVSEKDIQDLKFGVEQDVDMV

FASFIRKASDVHEVRKVLGEKGKNIKIISKIENHEGVRRFDEILEASDGIMVARGDLGIE

IPAEKVFLAQKMMIGRCNRAGKPVICATQMLESMIKKPRPTRAEGSDVANAVLDGADCIM

LSGETAKGDYPLEAVRMQHLIAREAEAAIYHLQLFEELRRLAPITSDPTEATAVGAVEAS

FKCCSGAIIVLTKSGRSAHQVARYRPRAPIIAVTRNPQTARQAHLYRGIFPVLCKDPVQE

AWAEDVDLRVNFAMNVGKARGFFKKGDVVIVLTGWRPGSGFTNTMRVVPVP

>sp|P14618|KPYM_HUMAN 265 IKIISKIENHEGVRR

MSKPHSEAGTAFIQTQQLHAAMADTFLEHMCRLDIDSPPITARNTGIICTIGPASRSVET

LKEMIKSGMNVARLNFSHGTHEYHAETIKNVRTATESFASDPILYRPVAVALDTKGPEIR

TGLIKGSGTAEVELKKGATLKITLDNAYMEKCDENILWLDYKNICKVVEVGSKIYVDDGL

ISLQVKQKGADFLVTEVENGGSLGSKKGVNLPGAAVDLPAVSEKDIQDLKFGVEQDVDMV

FASFIRKASDVHEVRKVLGEKGKNIKIISKIENHEGVRRFDEILEASDGIMVARGDLGIE

IPAEKVFLAQKMMIGRCNRAGKPVICATQMLESMIKKPRPTRAEGSDVANAVLDGADCIM

LSGETAKGDYPLEAVRMQHLIAREAEAAIYHLQLFEELRRLAPITSDPTEATAVGAVEAS

FKCCSGAIIVLTKSGRSAHQVARYRPRAPIIAVTRNPQTARQAHLYRGIFPVLCKDPVQE

AWAEDVDLRVNFAMNVGKARGFFKKGDVVIVLTGWRPGSGFTNTMRVVPVP

>sp|P14618|KPYM_HUMAN 100 SDPILYRPVAVALD

MSKPHSEAGTAFIQTQQLHAAMADTFLEHMCRLDIDSPPITARNTGIICTIGPASRSVET

LKEMIKSGMNVARLNFSHGTHEYHAETIKNVRTATESFASDPILYRPVAVALDTKGPEIR

TGLIKGSGTAEVELKKGATLKITLDNAYMEKCDENILWLDYKNICKVVEVGSKIYVDDGL

ISLQVKQKGADFLVTEVENGGSLGSKKGVNLPGAAVDLPAVSEKDIQDLKFGVEQDVDMV

FASFIRKASDVHEVRKVLGEKGKNIKIISKIENHEGVRRFDEILEASDGIMVARGDLGIE

IPAEKVFLAQKMMIGRCNRAGKPVICATQMLESMIKKPRPTRAEGSDVANAVLDGADCIM

LSGETAKGDYPLEAVRMQHLIAREAEAAIYHLQLFEELRRLAPITSDPTEATAVGAVEAS

FKCCSGAIIVLTKSGRSAHQVARYRPRAPIIAVTRNPQTARQAHLYRGIFPVLCKDPVQE

AWAEDVDLRVNFAMNVGKARGFFKKGDVVIVLTGWRPGSGFTNTMRVVPVP

>sp|P14679|TYRO_HUMAN 193 EIWRDIDFAHE

MLLAVLYCLLWSFQTSAGHFPRACVSSKNLMEKECCPPWSGDRSPCGQLSGRGSCQNILL

SNAPLGPQFPFTGVDDRESWPSVFYNRTCQCSGNFMGFNCGNCKFGFWGPNCTERRLLVR

RNIFDLSAPEKDKFFAYLTLAKHTISSDYVIPIGTYGQMKNGSTPMFNDINIYDLFVWMH

YYVSMDALLGGSEIWRDIDFAHEAPAFLPWHRLFLLRWEQEIQKLTGDENFTIPYWDWRD

AEKCDICTDEYMGGQHPTNPNLLSPASFFSSWQIVCSRLEEYNSHQSLCNGTPEGPLRRN

PGNHDKSRTPRLPSSADVEFCLSLTQYESGSMDKAANFSFRNTLEGFASPLTGIADASQS

SMHNALHIYMNGTMSQVQGSANDPIFLLHHAFVDSIFEQWLRRHRPLQEVYPEANAPIGH

NRESYMVPFIPLYRNGDFFISSKDLGYDYSYLQDSDPDSFQDYIKSYLEQASRIWSWLLG

AAMVGAVLTALLAGLVSLLCRHKRKQLPEEKQPLLMEKEDYHSLYQSHL

>sp|P08195|4F2_HUMAN 414 DPGSLLSLFRRLSDQRSK

MSQDTEVDMKEVELNELEPEKQPMNAASGAAMSLAGAEKNGLVKIKVAEDEAEAAAAAKF

TGLSKEELLKVAGSPGWVRTRWALLLLFWLGWLGMLAGAVVIIVRAPRCRELPAQKWWHT

GALYRIGDLQAFQGHGAGNLAGLKGRLDYLSSLKVKGLVLGPIHKNQKDDVAQTDLLQID

PNFGSKEDFDSLLQSAKKKSIRVILDLTPNYRGENSWFSTQVDTVATKVKDALEFWLQAG

VDGFQVRDIENLKDASSFLAEWQNITKGFSEDRLLIAGTNSSDLQQILSLLESNKDLLLT

SSYLSDSGSTGEHTKSLVTQYLNATGNRWCSWSLSQARLLTSFLPAQLLRLYQLMLFTLP

GTPVFSYGDEIGLDAAALPGQPMEAPVMLWDESSFPDIPGAVSANMTVKGQSEDPGSLLS

LFRRLSDQRSKERSLLHGDFHAFSAGPGLFSYIRHWDQNERFLVVLNFGDVGLSAGLQAS

DLPASASLPAKADLLLSTQPGREEGSPLELERLKLEPHEGLLLRFPYAA

>sp|P08195|4F2_HUMAN 120 TGALYRIGDLQAFQGHG

MSQDTEVDMKEVELNELEPEKQPMNAASGAAMSLAGAEKNGLVKIKVAEDEAEAAAAAKF

TGLSKEELLKVAGSPGWVRTRWALLLLFWLGWLGMLAGAVVIIVRAPRCRELPAQKWWHT

GALYRIGDLQAFQGHGAGNLAGLKGRLDYLSSLKVKGLVLGPIHKNQKDDVAQTDLLQID

PNFGSKEDFDSLLQSAKKKSIRVILDLTPNYRGENSWFSTQVDTVATKVKDALEFWLQAG

VDGFQVRDIENLKDASSFLAEWQNITKGFSEDRLLIAGTNSSDLQQILSLLESNKDLLLT

SSYLSDSGSTGEHTKSLVTQYLNATGNRWCSWSLSQARLLTSFLPAQLLRLYQLMLFTLP

GTPVFSYGDEIGLDAAALPGQPMEAPVMLWDESSFPDIPGAVSANMTVKGQSEDPGSLLS

LFRRLSDQRSKERSLLHGDFHAFSAGPGLFSYIRHWDQNERFLVVLNFGDVGLSAGLQAS

DLPASASLPAKADLLLSTQPGREEGSPLELERLKLEPHEGLLLRFPYAA

>sp|P08195|4F2_HUMAN 318 VTQYLNATGNRWCSWSL

MSQDTEVDMKEVELNELEPEKQPMNAASGAAMSLAGAEKNGLVKIKVAEDEAEAAAAAKF

TGLSKEELLKVAGSPGWVRTRWALLLLFWLGWLGMLAGAVVIIVRAPRCRELPAQKWWHT

GALYRIGDLQAFQGHGAGNLAGLKGRLDYLSSLKVKGLVLGPIHKNQKDDVAQTDLLQID

PNFGSKEDFDSLLQSAKKKSIRVILDLTPNYRGENSWFSTQVDTVATKVKDALEFWLQAG

VDGFQVRDIENLKDASSFLAEWQNITKGFSEDRLLIAGTNSSDLQQILSLLESNKDLLLT

SSYLSDSGSTGEHTKSLVTQYLNATGNRWCSWSLSQARLLTSFLPAQLLRLYQLMLFTLP

GTPVFSYGDEIGLDAAALPGQPMEAPVMLWDESSFPDIPGAVSANMTVKGQSEDPGSLLS

LFRRLSDQRSKERSLLHGDFHAFSAGPGLFSYIRHWDQNERFLVVLNFGDVGLSAGLQAS

DLPASASLPAKADLLLSTQPGREEGSPLELERLKLEPHEGLLLRFPYAA

>sp|P08195|4F2_HUMAN 318 VTQYLNATGNRWCSWSLSQAR

MSQDTEVDMKEVELNELEPEKQPMNAASGAAMSLAGAEKNGLVKIKVAEDEAEAAAAAKF

TGLSKEELLKVAGSPGWVRTRWALLLLFWLGWLGMLAGAVVIIVRAPRCRELPAQKWWHT

GALYRIGDLQAFQGHGAGNLAGLKGRLDYLSSLKVKGLVLGPIHKNQKDDVAQTDLLQID

PNFGSKEDFDSLLQSAKKKSIRVILDLTPNYRGENSWFSTQVDTVATKVKDALEFWLQAG

VDGFQVRDIENLKDASSFLAEWQNITKGFSEDRLLIAGTNSSDLQQILSLLESNKDLLLT

SSYLSDSGSTGEHTKSLVTQYLNATGNRWCSWSLSQARLLTSFLPAQLLRLYQLMLFTLP

GTPVFSYGDEIGLDAAALPGQPMEAPVMLWDESSFPDIPGAVSANMTVKGQSEDPGSLLS

LFRRLSDQRSKERSLLHGDFHAFSAGPGLFSYIRHWDQNERFLVVLNFGDVGLSAGLQAS

DLPASASLPAKADLLLSTQPGREEGSPLELERLKLEPHEGLLLRFPYAA

>sp|P07602|SAP_HUMAN 498 GPSYWCQNTETAAQ

MYALFLLASLLGAALAGPVLGLKECTRGSAVWCQNVKTASDCGAVKHCLQTVWNKPTVKS

LPCDICKDVVTAAGDMLKDNATEEEILVYLEKTCDWLPKPNMSASCKEIVDSYLPVILDI

IKGEMSRPGEVCSALNLCESLQKHLAELNHQKQLESNKIPELDMTEVVAPFMANIPLLLY

PQDGPRSKPQPKDNGDVCQDCIQMVTDIQTAVRTNSTFVQALVEHVKEECDRLGPGMADI

CKNYISQYSEIAIQMMMHMQPKEICALVGFCDEVKEMPMQTLVPAKVASKNVIPALELVE

PIKKHEVPAKSDVYCEVCEFLVKEVTKLIDNNKTEKEILDAFDKMCSKLPKSLSEECQEV

VDTYGSSILSILLEEVSPELVCSMLHLCSGTRLPALTVHVTQPKDGGFCEVCKKLVGYLD

RNLEKNSTKQEILAALEKGCSFLPDPYQKQCDQFVAEYEPVLIEILVEVMDPSFVCLKIG

ACPSAHKPLLGTEKCIWGPSYWCQNTETAAQCNAVEHCKRHVWN

>sp|Q9C0H2|TTYH3_HUMAN 450 GSSYGSETSIPAAAH

MAGVSYAAPWWVSLLHRLPHFDLSWEATSSQFRPEDTDYQQALLLLGAAALACLALDLLF

LLFYSFWLCCRRRKSEEHLDADCCCTAWCVIIATLVCSAGIAVGFYGNGETSDGIHRATY

SLRHANRTVAGVQDRVWDTAVGLNHTAEPSLQTLERQLAGRPEPLRAVQRLQGLLETLLG

YTAAIPFWRNTAVSLEVLAEQVDLYDWYRWLGYLGLLLLDVIICLLVLVGLIRSSKGILV

GVCLLGVLALVISWGALGLELAVSVGSSDFCVDPDAYVTKMVEEYSVLSGDILQYYLACS

PRAANPFQQKLSGSHKALVEMQDVVAELLRTVPWEQPATKDPLLRVQEVLNGTEVNLQHL

TALVDCRSLHLDYVQALTGFCYDGVEGLIYLALFSFVTALMFSSIVCSVPHTWQQKRGPD

EDGEEEAAPGPRQAHDSLYRVHMPSLYSCGSSYGSETSIPAAAHTVSNAPVTEYMSQNAN

FQNPRCENTPLIGRESPPPSYTSSMRAKYLATSQPRPDSSGSH

>sp|Q8TDB8|GTR14_HUMAN 162 VPMYIGEISPTALR

MEFHNGGHVSGIGGFLVSLTSRMKPHTLAVTPALIFAITVATIGSFQFGYNTGVINAPET

IIKEFINKTLTDKANAPPSEVLLTNLWSLSVAIFSVGGMIGSFSVGLFVNRFGRRNSMLI

VNLLAATGGCLMGLCKIAESVEMLILGRLVIGLFCGLCTGFVPMYIGEISPTALRGAFGT

LNQLGIVIGILVAQIFGLELILGSEELWPVLLGFTILPAILQSAALPCCPESPRFLLINR

KKEENATRILQRLWGTQDVSQDIQEMKDESARMSQEKQVTVLELFRVSSYRQPIIISIVL

QLSQQLSGINAVFYYSTGIFKDAGVQQPIYATISAGVVNTIFTLLSLFLVERAGRRTLHM

IGLGGMAFCSTLMTVSLLLKNHYNGMSFVCIGAILVFVACFEIGPGPIPWFIVAELFSQG

PRPAAMAVAGCSNWTSNFLVGLLFPSAAYYLGAYVFIIFTGFLITFLAFTFFKVPETRGR

TFEDITRAFEGQAHGADRSGKDGVMGMNSIEPAKETTTNV

>sp|P09622|DLDH_HUMAN 139 AHLFKQNKVVHVNG

MQSWSRVYCSLAKRGHFNRISHGLQGLSAVPLRTYADQPIDADVTVIGSGPGGYVAAIKA

AQLGFKTVCIEKNETLGGTCLNVGCIPSKALLNNSHYYHMAHGTDFASRGIEMSEVRLNL

DKMMEQKSTAVKALTGGIAHLFKQNKVVHVNGYGKITGKNQVTATKADGGTQVIDTKNIL

IATGSEVTPFPGITIDEDTIVSSTGALSLKKVPEKMVVIGAGVIGVELGSVWQRLGADVT

AVEFLGHVGGVGIDMEISKNFQRILQKQGFKFKLNTKVTGATKKSDGKIDVSIEAASGGK

AEVITCDVLLVCIGRRPFTKNLGLEELGIELDPRGRIPVNTRFQTKIPNIYAIGDVVAGP

MLAHKAEDEGIICVEGMAGGAVHIDYNCVPSVIYTHPEVAWVGKSEEQLKEEGIEYKVGK

FPFAANSRAKTNADTDGMVKILGQKSTDRVLGAHILGPGAGEMVNEAALALEYGASCEDI

ARVCHAHPTLSEAFREANLAASFGKSINF

>sp|P11230|ACHB_HUMAN 289 VFLLLLADKVPETSLS

MTPGALLMLLGALGAPLAPGVRGSEAEGRLREKLFSGYDSSVRPAREVGDRVRVSVGLIL

AQLISLNEKDEEMSTKVYLDLEWTDYRLSWDPAEHDGIDSLRITAESVWLPDVVLLNNND

GNFDVALDISVVVSSDGSVRWQPPGIYRSSCSIQVTYFPFDWQNCTMVFSSYSYDSSEVS

LQTGLGPDGQGHQEIHIHEGTFIENGQWEIIHKPSRLIQPPGDPRGGREGQRQEVIFYLI

IRRKPLFYLVNVIAPCILITLLAIFVFYLPPDAGEKMGLSIFALLTLTVFLLLLADKVPE

TSLSVPIIIKYLMFTMVLVTFSVILSVVVLNLHHRSPHTHQMPLWVRQIFIHKLPLYLRL

KRPKPERDLMPEPPHCSSPGSGWGRGTDEYFIRKPPSDFLFPKPNRFQPELSAPDLRRFI

DGPNRAVALLPELREVVSSISYIARQLQEQEDHDALKEDWQFVAMVVDRLFLWTFIIFTS

VGTLVIFLDATYHLPPPDPFP

>sp|O75071|K0494_HUMAN 411 FSQFLGDPVEKAAQ

MKKRKELNALIGLAGDSRRKKPKKGPSSHRLLRTEPPDSDSESSSEEEEEFGVVGNRSRF

AKGDYLRCCKICYPLCGFVILAACVVACVGLVWMQVALKEDLDALKEKFRTMESNQKSSF

QEIPKLNEELLSKQKQLEKIESGEMGLNKVWINITEMNKQISLLTSAVNHLKANVKSAAD

LISLPTTVEGLQKSVASIGNTLNSVHLAVEALQKTVDEHKKTMELLQSDMNQHFLKETPG

SNQIIPSPSATSELDNKTHSENLKQDILYLHNSLEEVNSALVGYQRQNDLKLEGMNETVS

NLTQRVNLIESDVVAMSKVEKKANLSFSMMGDRSATLKRQSLDQVTNRTDTVKIQSIKKE

DSSNSQVSKLREKLQLISALTNKPESNRPPETADEEQVESFTSKPSALPKFSQFLGDPVE

KAAQLRPISLPGVSSTEDLQDLFRKTGQDVDGKLTYQEIWTSLGSAMPEPESLRAFDSDG

DGRYSFLELRVALGI

>sp|Q9UHL4|DPP2_HUMAN 114 LPFGAQSTQRGHTE

MGSAPWAPVLLLALGLRGLQAGARRAPDPGFQERFFQQRLDHFNFERFGNKTFPQRFLVS

DRFWVRGEGPIFFYTGNEGDVWAFANNSGFVAELAAERGALLVFAEHRYYGKSLPFGAQS

TQRGHTELLTVEQALADFAELLRALRRDLGAQDAPAIAFGGSYGGMLSAYLRMKYPHLVA

GALAASAPVLAVAGLGDSNQFFRDVTADFEGQSPKCTQGVREAFRQIKDLFLQGAYDTVR

WEFGTCQPLSDEKDLTQLFMFARNAFTVLAMMDYPYPTDFLGPLPANPVKVGCDRLLSEA

QRITGLRALAGLVYNASGSEHCYDIYRLYHSCADPTGCGTGPDARAWDYQACTEINLTFA

SNNVTDMFPDLPFTDELRQRYCLDTWGVWPRPDWLLTSFWGGDLRAASNIIFSNGNLDPW

AGGGIRRNLSASVIAVTIQGGAHHLDLRASHPEDPASVVEARKLEATIIGEWVKAARREQ

QPALRGGPRLSL

>sp|P15260|INGR1_HUMAN 128 GPPKLDIRKEEKQIMIDIFH

MALLFLLPLVMQGVSRAEMGTADLGPSSVPTPTNVTIESYNMNPIVYWEYQIMPQVPVFT

VEVKNYGVKNSEWIDACINISHHYCNISDHVGDPSNSLWVRVKARVGQKESAYAKSEEFA

VCRDGKIGPPKLDIRKEEKQIMIDIFHPSVFVNGDEQEVDYDPETTCYIRVYNVYVRMNG

SEIQYKILTQKEDDCDEIQCQLAIPVSSLNSQYCVSAEGVLHVWGVTTEKSKEVCITIFN

SSIKGSLWIPVVAALLLFLVLSLVFICFYIKKINPLKEKSIILPKSLISVVRSATLETKP

ESKYVSLITSYQPFSLEKEVVCEEPLSPATVPGMHTEDNPGKVEHTEELSSITEVVTTEE

NIPDVVPGSHLTPIERESSSPLSSNQSEPGSIALNSYHSRNCSESDHSRNGFDTDSSCLE

SHSSLSDSEFPPNNKGEIKTEGQELITVIKAPTSFGYDKPHVLVDLLVDDSGKESLIGYR

PTEDSKEFS

>sp|P15260|INGR1_HUMAN 128 GPPKLDIRKEEKQIMIDIFHP

MALLFLLPLVMQGVSRAEMGTADLGPSSVPTPTNVTIESYNMNPIVYWEYQIMPQVPVFT

VEVKNYGVKNSEWIDACINISHHYCNISDHVGDPSNSLWVRVKARVGQKESAYAKSEEFA

VCRDGKIGPPKLDIRKEEKQIMIDIFHPSVFVNGDEQEVDYDPETTCYIRVYNVYVRMNG

SEIQYKILTQKEDDCDEIQCQLAIPVSSLNSQYCVSAEGVLHVWGVTTEKSKEVCITIFN

SSIKGSLWIPVVAALLLFLVLSLVFICFYIKKINPLKEKSIILPKSLISVVRSATLETKP

ESKYVSLITSYQPFSLEKEVVCEEPLSPATVPGMHTEDNPGKVEHTEELSSITEVVTTEE

NIPDVVPGSHLTPIERESSSPLSSNQSEPGSIALNSYHSRNCSESDHSRNGFDTDSSCLE

SHSSLSDSEFPPNNKGEIKTEGQELITVIKAPTSFGYDKPHVLVDLLVDDSGKESLIGYR

PTEDSKEFS

>sp|P10619|PPGB_HUMAN 38 LPGLAKQPSFRQYSG

MIRAAPPPLFLLLLLLLLLVSWASRGEAAPDQDEIQRLPGLAKQPSFRQYSGYLKGSGSK

HLHYWFVESQKDPENSPVVLWLNGGPGCSSLDGLLTEHGPFLVQPDGVTLEYNPYSWNLI

ANVLYLESPAGVGFSYSDDKFYATNDTEVAQSNFEALQDFFRLFPEYKNNKLFLTGESYA

GIYIPTLAVLVMQDPSMNLQGLAVGNGLSSYEQNDNSLVYFAYYHGLLGNRLWSSLQTHC

CSQNKCNFYDNKDLECVTNLQEVARIVGNSGLNIYNLYAPCAGGVPSHFRYEKDTVVVQD

LGNIFTRLPLKRMWHQALLRSGDKVRMDPPCTNTTAASTYLNNPYVRKALNIPEQLPQWD

MCNFLVNLQYRRLYRSMNSQYLKLLSSQKYQILLYNGDVDMACNFMGDEWFVDSLNQKME

VQRRPWLVKYGDSGEQIAGFVKEFSHIAFLTIKGAGHMVPTDKPLAAFTMFSRFLNKQPY

>sp|P16870|CBPE_HUMAN 101 EPGEPEFKYIGNMHGNEA

MAGRGGSALLALCGALAACGWLLGAEAQEPGAPAAGMRRRRRLQQEDGISFEYHRYPELR

EALVSVWLQCTAISRIYTVGRSFEGRELLVIELSDNPGVHEPGEPEFKYIGNMHGNEAVG

RELLIFLAQYLCNEYQKGNETIVNLIHSTRIHIMPSLNPDGFEKAASQPGELKDWFVGRS

NAQGIDLNRNFPDLDRIVYVNEKEGGPNNHLLKNMKKIVDQNTKLAPETKAVIHWIMDIP

FVLSANLHGGDLVANYPYDETRSGSAHEYSSSPDDAIFQSLARAYSSFNPAMSDPNRPPC

RKNDDDSSFVDGTTNGGAWYSVPGGMQDFNYLSSNCFEITVELSCEKFPPEETLKTYWED

NKNSLISYLEQIHRGVKGFVRDLQGNPIANATISVEGIDHDVTSAKDGDYWRLLIPGNYK

LTASAPGYLAITKKVAVPYSPAAGVDFELESFSERKEEEKEELMEWWKMMSETLNF

>sp|P16870|CBPE_HUMAN 103 GEPEFKYIGNMHGNEA

MAGRGGSALLALCGALAACGWLLGAEAQEPGAPAAGMRRRRRLQQEDGISFEYHRYPELR

EALVSVWLQCTAISRIYTVGRSFEGRELLVIELSDNPGVHEPGEPEFKYIGNMHGNEAVG

RELLIFLAQYLCNEYQKGNETIVNLIHSTRIHIMPSLNPDGFEKAASQPGELKDWFVGRS

NAQGIDLNRNFPDLDRIVYVNEKEGGPNNHLLKNMKKIVDQNTKLAPETKAVIHWIMDIP

FVLSANLHGGDLVANYPYDETRSGSAHEYSSSPDDAIFQSLARAYSSFNPAMSDPNRPPC

RKNDDDSSFVDGTTNGGAWYSVPGGMQDFNYLSSNCFEITVELSCEKFPPEETLKTYWED

NKNSLISYLEQIHRGVKGFVRDLQGNPIANATISVEGIDHDVTSAKDGDYWRLLIPGNYK

LTASAPGYLAITKKVAVPYSPAAGVDFELESFSERKEEEKEELMEWWKMMSETLNF

>sp|P16870|CBPE_HUMAN 184 GIDLNRNFPDLDRIVYV

MAGRGGSALLALCGALAACGWLLGAEAQEPGAPAAGMRRRRRLQQEDGISFEYHRYPELR

EALVSVWLQCTAISRIYTVGRSFEGRELLVIELSDNPGVHEPGEPEFKYIGNMHGNEAVG

RELLIFLAQYLCNEYQKGNETIVNLIHSTRIHIMPSLNPDGFEKAASQPGELKDWFVGRS

NAQGIDLNRNFPDLDRIVYVNEKEGGPNNHLLKNMKKIVDQNTKLAPETKAVIHWIMDIP

FVLSANLHGGDLVANYPYDETRSGSAHEYSSSPDDAIFQSLARAYSSFNPAMSDPNRPPC

RKNDDDSSFVDGTTNGGAWYSVPGGMQDFNYLSSNCFEITVELSCEKFPPEETLKTYWED

NKNSLISYLEQIHRGVKGFVRDLQGNPIANATISVEGIDHDVTSAKDGDYWRLLIPGNYK

LTASAPGYLAITKKVAVPYSPAAGVDFELESFSERKEEEKEELMEWWKMMSETLNF

>sp|P16870|CBPE_HUMAN 185 IDLNRNFPDLDRIVYV

MAGRGGSALLALCGALAACGWLLGAEAQEPGAPAAGMRRRRRLQQEDGISFEYHRYPELR

EALVSVWLQCTAISRIYTVGRSFEGRELLVIELSDNPGVHEPGEPEFKYIGNMHGNEAVG

RELLIFLAQYLCNEYQKGNETIVNLIHSTRIHIMPSLNPDGFEKAASQPGELKDWFVGRS

NAQGIDLNRNFPDLDRIVYVNEKEGGPNNHLLKNMKKIVDQNTKLAPETKAVIHWIMDIP

FVLSANLHGGDLVANYPYDETRSGSAHEYSSSPDDAIFQSLARAYSSFNPAMSDPNRPPC

RKNDDDSSFVDGTTNGGAWYSVPGGMQDFNYLSSNCFEITVELSCEKFPPEETLKTYWED

NKNSLISYLEQIHRGVKGFVRDLQGNPIANATISVEGIDHDVTSAKDGDYWRLLIPGNYK

LTASAPGYLAITKKVAVPYSPAAGVDFELESFSERKEEEKEELMEWWKMMSETLNF

>sp|Q9ULA0|DNPEP_HUMAN 462 FELFPSLSHNLLVD

MQVAMNGKARKEAVQTAAKELLKFVNRSPSPFHAVAECRNRLLQAGFSELKETEKWNIKP

ESKYFMTRNSSTIIAFAVGGQYVPGNGFSLIGAHTDSPCLRVKRRSRRSQVGFQQVGVET

YGGGIWSTWFDRDLTLAGRVIVKCPTSGRLEQQLVHVERPILRIPHLAIHLQRNINENFG

PNTEMHLVPILATAIQEELEKGTPEPGPLNAVDERHHSVLMSLLCAHLGLSPKDIVEMEL

CLADTQPAVLGGAYDEFIFAPRLDNLHSCFCALQALIDSCAGPGSLATEPHVRMVTLYDN

EEVGSESAQGAQSLLTELVLRRISASCQHPTAFEEAIPKSFMISADMAHAVHPNYLDKHE

ENHRPLFHKGPVIKVNSKQRYASNAVSEALIREVANKVKVPLQDLMVRNDTPCGTTIGPI

LASRLGLRVLDLGSPQLAMHSIREMACTTGVLQTLTLFKGFFELFPSLSHNLLVD

>sp|Q96KP4|CNDP2_HUMAN 22 LAKWVAIQSVSAWPE

MAALTTLFKYIDENQDRYIKKLAKWVAIQSVSAWPEKRGEIRRMMEVAAADVKQLGGSVE

LVDIGKQKLPDGSEIPLPPILLGRLGSDPQKKTVCIYGHLDVQPAALEDGWDSEPFTLVE

RDGKLYGRGSTDDKGPVAGWINALEAYQKTGQEIPVNVRFCLEGMEESGSEGLDELIFAR

KDTFFKDVDYVCISDNYWLGKKKPCITYGLRGICYFFIEVECSNKDLHSGVYGGSVHEAM

TDLILLMGSLVDKRGNILIPGINEAVAAVTEEEHKLYDDIDFDIEEFAKDVGAQILLHSH

KKDILMHRWRYPSLSLHGIEGAFSGSGAKTVIPRKVVGKFSIRLVPNMTPEVVGEQVTSY

LTKKFAELRSPNEFKVYMGHGGKPWVSDFSHPHYLAGRRAMKTVFGVEPDLTREGGSIPV

TLTFQEATGKNVMLLPVGSADDGAHSQNEKLNRYNYIEGTKMLAAYLYEVSQLKD

>sp|Q05639|EF1A2_HUMAN 39 IEKFEKEAAEMGKG

MGKEKTHINIVVIGHVDSGKSTTTGHLIYKCGGIDKRTIEKFEKEAAEMGKGSFKYAWVL

DKLKAERERGITIDISLWKFETTKYYITIIDAPGHRDFIKNMITGTSQADCAVLIVAAGV

GEFEAGISKNGQTREHALLAYTLGVKQLIVGVNKMDSTEPAYSEKRYDEIVKEVSAYIKK

IGYNPATVPFVPISGWHGDNMLEPSPNMPWFKGWKVERKEGNASGVSLLEALDTILPPTR

PTDKPLRLPLQDVYKIGGIGTVPVGRVETGILRPGMVVTFAPVNITTEVKSVEMHHEALS

EALPGDNVGFNVKNVSVKDIRRGNVCGDSKSDPPQEAAQFTSQVIILNHPGQISAGYSPV

IDCHTAHIACKFAELKEKIDRRSGKKLEDNPKSLKSGDAAIVEMVPGKPMCVESFSQYPP

LGRFAVRDMRQTVAVGVIKNVEKKSGGAGKVTKSAQKAQKAGK

>sp|Q05639|EF1A2_HUMAN 38 TIEKFEKEAAEMGKGSF

MGKEKTHINIVVIGHVDSGKSTTTGHLIYKCGGIDKRTIEKFEKEAAEMGKGSFKYAWVL

DKLKAERERGITIDISLWKFETTKYYITIIDAPGHRDFIKNMITGTSQADCAVLIVAAGV

GEFEAGISKNGQTREHALLAYTLGVKQLIVGVNKMDSTEPAYSEKRYDEIVKEVSAYIKK

IGYNPATVPFVPISGWHGDNMLEPSPNMPWFKGWKVERKEGNASGVSLLEALDTILPPTR

PTDKPLRLPLQDVYKIGGIGTVPVGRVETGILRPGMVVTFAPVNITTEVKSVEMHHEALS

EALPGDNVGFNVKNVSVKDIRRGNVCGDSKSDPPQEAAQFTSQVIILNHPGQISAGYSPV

IDCHTAHIACKFAELKEKIDRRSGKKLEDNPKSLKSGDAAIVEMVPGKPMCVESFSQYPP

LGRFAVRDMRQTVAVGVIKNVEKKSGGAGKVTKSAQKAQKAGK

>sp|P53634|CATC_HUMAN 58 GPQEKKVVVYLQKLDT

MGAGPSLLLAALLLLLSGDGAVRCDTPANCTYLDLLGTWVFQVGSSGSQRDVNCSVMGPQ

EKKVVVYLQKLDTAYDDLGNSGHFTIIYNQGFEIVLNDYKWFAFFKYKEEGSKVTTYCNE

TMTGWVHDVLGRNWACFTGKKVGTASENVYVNTAHLKNSQEKYSNRLYKYDHNFVKAINA

IQKSWTATTYMEYETLTLGDMIRRSGGHSRKIPRPKPAPLTAEIQQKILHLPTSWDWRNV

HGINFVSPVRNQASCGSCYSFASMGMLEARIRILTNNSQTPILSPQEVVSCSQYAQGCEG

GFPYLIAGKYAQDFGLVEEACFPYTGTDSPCKMKEDCFRYYSSEYHYVGGFYGGCNEALM

KLELVHHGPMAVAFEVYDDFLHYKKGIYHHTGLRDPFNPFELTNHAVLLVGYGTDSASGM

DYWIVKNSWGTGWGENGYFRIRRGTDECAIESIAVAATPIPKL

>sp|P53634|CATC_HUMAN 62 KKVVVYLQKLDTAYD

MGAGPSLLLAALLLLLSGDGAVRCDTPANCTYLDLLGTWVFQVGSSGSQRDVNCSVMGPQ

EKKVVVYLQKLDTAYDDLGNSGHFTIIYNQGFEIVLNDYKWFAFFKYKEEGSKVTTYCNE

TMTGWVHDVLGRNWACFTGKKVGTASENVYVNTAHLKNSQEKYSNRLYKYDHNFVKAINA

IQKSWTATTYMEYETLTLGDMIRRSGGHSRKIPRPKPAPLTAEIQQKILHLPTSWDWRNV

HGINFVSPVRNQASCGSCYSFASMGMLEARIRILTNNSQTPILSPQEVVSCSQYAQGCEG

GFPYLIAGKYAQDFGLVEEACFPYTGTDSPCKMKEDCFRYYSSEYHYVGGFYGGCNEALM

KLELVHHGPMAVAFEVYDDFLHYKKGIYHHTGLRDPFNPFELTNHAVLLVGYGTDSASGM

DYWIVKNSWGTGWGENGYFRIRRGTDECAIESIAVAATPIPKL

>sp|P53634|CATC_HUMAN 62 KKVVVYLQKLDTAYDDLG

MGAGPSLLLAALLLLLSGDGAVRCDTPANCTYLDLLGTWVFQVGSSGSQRDVNCSVMGPQ

EKKVVVYLQKLDTAYDDLGNSGHFTIIYNQGFEIVLNDYKWFAFFKYKEEGSKVTTYCNE

TMTGWVHDVLGRNWACFTGKKVGTASENVYVNTAHLKNSQEKYSNRLYKYDHNFVKAINA

IQKSWTATTYMEYETLTLGDMIRRSGGHSRKIPRPKPAPLTAEIQQKILHLPTSWDWRNV

HGINFVSPVRNQASCGSCYSFASMGMLEARIRILTNNSQTPILSPQEVVSCSQYAQGCEG

GFPYLIAGKYAQDFGLVEEACFPYTGTDSPCKMKEDCFRYYSSEYHYVGGFYGGCNEALM

KLELVHHGPMAVAFEVYDDFLHYKKGIYHHTGLRDPFNPFELTNHAVLLVGYGTDSASGM

DYWIVKNSWGTGWGENGYFRIRRGTDECAIESIAVAATPIPKL

>sp|P53634|CATC_HUMAN 63 KVVVYLQKLDTAYD

MGAGPSLLLAALLLLLSGDGAVRCDTPANCTYLDLLGTWVFQVGSSGSQRDVNCSVMGPQ

EKKVVVYLQKLDTAYDDLGNSGHFTIIYNQGFEIVLNDYKWFAFFKYKEEGSKVTTYCNE

TMTGWVHDVLGRNWACFTGKKVGTASENVYVNTAHLKNSQEKYSNRLYKYDHNFVKAINA

IQKSWTATTYMEYETLTLGDMIRRSGGHSRKIPRPKPAPLTAEIQQKILHLPTSWDWRNV

HGINFVSPVRNQASCGSCYSFASMGMLEARIRILTNNSQTPILSPQEVVSCSQYAQGCEG

GFPYLIAGKYAQDFGLVEEACFPYTGTDSPCKMKEDCFRYYSSEYHYVGGFYGGCNEALM

KLELVHHGPMAVAFEVYDDFLHYKKGIYHHTGLRDPFNPFELTNHAVLLVGYGTDSASGM

DYWIVKNSWGTGWGENGYFRIRRGTDECAIESIAVAATPIPKL

>sp|P53634|CATC_HUMAN 169 KYDHNFVKAINAIQKSWT

MGAGPSLLLAALLLLLSGDGAVRCDTPANCTYLDLLGTWVFQVGSSGSQRDVNCSVMGPQ

EKKVVVYLQKLDTAYDDLGNSGHFTIIYNQGFEIVLNDYKWFAFFKYKEEGSKVTTYCNE

TMTGWVHDVLGRNWACFTGKKVGTASENVYVNTAHLKNSQEKYSNRLYKYDHNFVKAINA

IQKSWTATTYMEYETLTLGDMIRRSGGHSRKIPRPKPAPLTAEIQQKILHLPTSWDWRNV

HGINFVSPVRNQASCGSCYSFASMGMLEARIRILTNNSQTPILSPQEVVSCSQYAQGCEG

GFPYLIAGKYAQDFGLVEEACFPYTGTDSPCKMKEDCFRYYSSEYHYVGGFYGGCNEALM

KLELVHHGPMAVAFEVYDDFLHYKKGIYHHTGLRDPFNPFELTNHAVLLVGYGTDSASGM

DYWIVKNSWGTGWGENGYFRIRRGTDECAIESIAVAATPIPKL

>sp|P53634|CATC_HUMAN 418 SGMDYWIVKNSWGTGWG

MGAGPSLLLAALLLLLSGDGAVRCDTPANCTYLDLLGTWVFQVGSSGSQRDVNCSVMGPQ

EKKVVVYLQKLDTAYDDLGNSGHFTIIYNQGFEIVLNDYKWFAFFKYKEEGSKVTTYCNE

TMTGWVHDVLGRNWACFTGKKVGTASENVYVNTAHLKNSQEKYSNRLYKYDHNFVKAINA

IQKSWTATTYMEYETLTLGDMIRRSGGHSRKIPRPKPAPLTAEIQQKILHLPTSWDWRNV

HGINFVSPVRNQASCGSCYSFASMGMLEARIRILTNNSQTPILSPQEVVSCSQYAQGCEG

GFPYLIAGKYAQDFGLVEEACFPYTGTDSPCKMKEDCFRYYSSEYHYVGGFYGGCNEALM

KLELVHHGPMAVAFEVYDDFLHYKKGIYHHTGLRDPFNPFELTNHAVLLVGYGTDSASGM

DYWIVKNSWGTGWGENGYFRIRRGTDECAIESIAVAATPIPKL

>sp|P53634|CATC_HUMAN 170 YDHNFVKAINAIQKSW

MGAGPSLLLAALLLLLSGDGAVRCDTPANCTYLDLLGTWVFQVGSSGSQRDVNCSVMGPQ

EKKVVVYLQKLDTAYDDLGNSGHFTIIYNQGFEIVLNDYKWFAFFKYKEEGSKVTTYCNE

TMTGWVHDVLGRNWACFTGKKVGTASENVYVNTAHLKNSQEKYSNRLYKYDHNFVKAINA

IQKSWTATTYMEYETLTLGDMIRRSGGHSRKIPRPKPAPLTAEIQQKILHLPTSWDWRNV

HGINFVSPVRNQASCGSCYSFASMGMLEARIRILTNNSQTPILSPQEVVSCSQYAQGCEG

GFPYLIAGKYAQDFGLVEEACFPYTGTDSPCKMKEDCFRYYSSEYHYVGGFYGGCNEALM

KLELVHHGPMAVAFEVYDDFLHYKKGIYHHTGLRDPFNPFELTNHAVLLVGYGTDSASGM

DYWIVKNSWGTGWGENGYFRIRRGTDECAIESIAVAATPIPKL

>sp|P68104|EF1A1_HUMAN 342 AQVIILNHPGQISAGYAP

MGKEKTHINIVVIGHVDSGKSTTTGHLIYKCGGIDKRTIEKFEKEAAEMGKGSFKYAWVL

DKLKAERERGITIDISLWKFETSKYYVTIIDAPGHRDFIKNMITGTSQADCAVLIVAAGV

GEFEAGISKNGQTREHALLAYTLGVKQLIVGVNKMDSTEPPYSQKRYEEIVKEVSTYIKK

IGYNPDTVAFVPISGWNGDNMLEPSANMPWFKGWKVTRKDGNASGTTLLEALDCILPPTR

PTDKPLRLPLQDVYKIGGIGTVPVGRVETGVLKPGMVVTFAPVNVTTEVKSVEMHHEALS

EALPGDNVGFNVKNVSVKDVRRGNVAGDSKNDPPMEAAGFTAQVIILNHPGQISAGYAPV

LDCHTAHIACKFAELKEKIDRRSGKKLEDGPKFLKSGDAAIVDMVPGKPMCVESFSDYPP

LGRFAVRDMRQTVAVGVIKAVDKKAAGAGKVTKSAQKAQKAK

>sp|P68104|EF1A1_HUMAN 83 SKYYVTIIDAPGHRD

MGKEKTHINIVVIGHVDSGKSTTTGHLIYKCGGIDKRTIEKFEKEAAEMGKGSFKYAWVL

DKLKAERERGITIDISLWKFETSKYYVTIIDAPGHRDFIKNMITGTSQADCAVLIVAAGV

GEFEAGISKNGQTREHALLAYTLGVKQLIVGVNKMDSTEPPYSQKRYEEIVKEVSTYIKK

IGYNPDTVAFVPISGWNGDNMLEPSANMPWFKGWKVTRKDGNASGTTLLEALDCILPPTR

PTDKPLRLPLQDVYKIGGIGTVPVGRVETGVLKPGMVVTFAPVNVTTEVKSVEMHHEALS

EALPGDNVGFNVKNVSVKDVRRGNVAGDSKNDPPMEAAGFTAQVIILNHPGQISAGYAPV

LDCHTAHIACKFAELKEKIDRRSGKKLEDGPKFLKSGDAAIVDMVPGKPMCVESFSDYPP

LGRFAVRDMRQTVAVGVIKAVDKKAAGAGKVTKSAQKAQKAK

>sp|Q16790|CAH9_HUMAN 249 EIHVVHLSTAFARVDEALGR

MAPLCPSPWLPLLIPAPAPGLTVQLLLSLLLLVPVHPQRLPRMQEDSPLGGGSSGEDDPL

GEEDLPSEEDSPREEDPPGEEDLPGEEDLPGEEDLPEVKPKSEEEGSLKLEDLPTVEAPG

DPQEPQNNAHRDKEGDDQSHWRYGGDPPWPRVSPACAGRFQSPVDIRPQLAAFCPALRPL

ELLGFQLPPLPELRLRNNGHSVQLTLPPGLEMALGPGREYRALQLHLHWGAAGRPGSEHT

VEGHRFPAEIHVVHLSTAFARVDEALGRPGGLAVLAAFLEEGPEENSAYEQLLSRLEEIA

EEGSETQVPGLDISALLPSDFSRYFQYEGSLTTPPCAQGVIWTVFNQTVMLSAKQLHTLS

DTLWGPGDSRLQLNFRATQPLNGRVIEASFPAGVDSSPRAAEPVQLNSCLAAGDILALVF

GLLFAVTSVAFLVQMRRQHRRGTKGGVSYRPAEVAETGA

>sp|Q05524|ENO1B_HUMAN 205 IKEKYGKDATNVGDEGG

MSILKIIHARDIFESRGNPTVEVDLYTNKGGLFGRAAVPSGASTGIYEALLELRDNDKTR

YMGGKGVSKAVEHIINKTIAPALISKNVNVVEQDKIDNLMLDMDGSENKSKFGANAILGV

SLAVCSNAGATAEKGVPLYRHIADLAGNNPEVILPVPAFNVINGGSHAGNKLAMQEFMIP

PCGADRFNDAIRIGAEVYHNLKNVIKEKYGKDATNVGDEGGFAPNILENKEALELLKTAI

GKAGYSDKVVIGMDVAASEFYRDGKYDLDFNSPDDPSRYISPDQLADLYKGFVLGHAVKN

YPVGVSIEDPPFDQDDWGAWKKLFTGSLVGIQVVGDDLTVTKPEARIAKAVEEVKACNCL

LLLKVNQIGSVTESLQACKLAQSNGWGVMPVSHRLSGETEDTFMADLVVGLCTGQIKTGP

TCRSERLAKYNQLLRIEEAEAGSKARFAGRNFRNPRIN

>sp|Q05524|ENO1B_HUMAN 204 VIKEKYGKDATNVGDEGG

MSILKIIHARDIFESRGNPTVEVDLYTNKGGLFGRAAVPSGASTGIYEALLELRDNDKTR

YMGGKGVSKAVEHIINKTIAPALISKNVNVVEQDKIDNLMLDMDGSENKSKFGANAILGV

SLAVCSNAGATAEKGVPLYRHIADLAGNNPEVILPVPAFNVINGGSHAGNKLAMQEFMIP

PCGADRFNDAIRIGAEVYHNLKNVIKEKYGKDATNVGDEGGFAPNILENKEALELLKTAI

GKAGYSDKVVIGMDVAASEFYRDGKYDLDFNSPDDPSRYISPDQLADLYKGFVLGHAVKN

YPVGVSIEDPPFDQDDWGAWKKLFTGSLVGIQVVGDDLTVTKPEARIAKAVEEVKACNCL

LLLKVNQIGSVTESLQACKLAQSNGWGVMPVSHRLSGETEDTFMADLVVGLCTGQIKTGP

TCRSERLAKYNQLLRIEEAEAGSKARFAGRNFRNPRIN

>sp|P01871|MUC_HUMAN 171 ESGPTTYKVTSTLTIKESDWL

GSASAPTLFPLVSCENSPSDTSSVAVGCLAQDFLPDSITFSWKYKNNSDISSTRGFPSVL

RGGKYAATSQVLLPSKDVMQGTDEHVVCKVQHPNGNKEKNVPLPVIAELPPKVSVFVPPR

DGFFGNPRSKSKLICQATGFSPRQIQVSWLREGKQVGSGVTTDQVQAEAKESGPTTYKVT

STLTIKESDWLSQSMFTCRVDHRGLTFQQNASSMCVPDQDTAIRVFAIPPSFASIFLTKS

TKLTCLVTDLTTYDSVTISWTRQNGEAVKTHTNISESHPNATFSAVGEASICEDDWNSGE

RFTCTVTHTDLPSPLKQTISRPKGVALHRPDVYLLPPAREQLNLRESATITCLVTGFSPA

DVFVQWMQRGQPLSPEKYVTSAPMPEPQAPGRYFAHSILTVSEEEWNTGETYTCVVAHEA

LPNRVTERTVDKSTGKPTLYNVSLVMSDTAGTCY

>sp|P68363|TBA1B_HUMAN 207 EAIYDICRRNLDI

MRECISIHVGQAGVQIGNACWELYCLEHGIQPDGQMPSDKTIGGGDDSFNTFFSETGAGK

HVPRAVFVDLEPTVIDEVRTGTYRQLFHPEQLITGKEDAANNYARGHYTIGKEIIDLVLD

RIRKLADQCTGLQGFLVFHSFGGGTGSGFTSLLMERLSVDYGKKSKLEFSIYPAPQVSTA

VVEPYNSILTTHTTLEHSDCAFMVDNEAIYDICRRNLDIERPTYTNLNRLISQIVSSITA

SLRFDGALNVDLTEFQTNLVPYPRIHFPLATYAPVISAEKAYHEQLSVAEITNACFEPAN

QMVKCDPRHGKYMACCLLYRGDVVPKDVNAAIATIKTKRSIQFVDWCPTGFKVGINYQPP

TVVPGGDLAKVQRAVCMLSNTTAIAEAWARLDHKFDLMYAKRAFVHWYVGEGMEEGEFSE

AREDMAALEKDYEEVGVDSVEGEGEEEGEEY

>sp|P68363|TBA1B_HUMAN 207 EAIYDICRRNLDIERPT

MRECISIHVGQAGVQIGNACWELYCLEHGIQPDGQMPSDKTIGGGDDSFNTFFSETGAGK

HVPRAVFVDLEPTVIDEVRTGTYRQLFHPEQLITGKEDAANNYARGHYTIGKEIIDLVLD

RIRKLADQCTGLQGFLVFHSFGGGTGSGFTSLLMERLSVDYGKKSKLEFSIYPAPQVSTA

VVEPYNSILTTHTTLEHSDCAFMVDNEAIYDICRRNLDIERPTYTNLNRLISQIVSSITA

SLRFDGALNVDLTEFQTNLVPYPRIHFPLATYAPVISAEKAYHEQLSVAEITNACFEPAN

QMVKCDPRHGKYMACCLLYRGDVVPKDVNAAIATIKTKRSIQFVDWCPTGFKVGINYQPP

TVVPGGDLAKVQRAVCMLSNTTAIAEAWARLDHKFDLMYAKRAFVHWYVGEGMEEGEFSE

AREDMAALEKDYEEVGVDSVEGEGEEEGEEY

>sp|P68363|TBA1B_HUMAN 391 LDHKFDLMYAKRAFVHWY

MRECISIHVGQAGVQIGNACWELYCLEHGIQPDGQMPSDKTIGGGDDSFNTFFSETGAGK

HVPRAVFVDLEPTVIDEVRTGTYRQLFHPEQLITGKEDAANNYARGHYTIGKEIIDLVLD

RIRKLADQCTGLQGFLVFHSFGGGTGSGFTSLLMERLSVDYGKKSKLEFSIYPAPQVSTA

VVEPYNSILTTHTTLEHSDCAFMVDNEAIYDICRRNLDIERPTYTNLNRLISQIVSSITA

SLRFDGALNVDLTEFQTNLVPYPRIHFPLATYAPVISAEKAYHEQLSVAEITNACFEPAN

QMVKCDPRHGKYMACCLLYRGDVVPKDVNAAIATIKTKRSIQFVDWCPTGFKVGINYQPP

TVVPGGDLAKVQRAVCMLSNTTAIAEAWARLDHKFDLMYAKRAFVHWYVGEGMEEGEFSE

AREDMAALEKDYEEVGVDSVEGEGEEEGEEY

>sp|Q71U36|TBA1A_HUMAN 220 ERPTYTNLNRLIGQIVSS

MRECISIHVGQAGVQIGNACWELYCLEHGIQPDGQMPSDKTIGGGDDSFNTFFSETGAGK

HVPRAVFVDLEPTVIDEVRTGTYRQLFHPEQLITGKEDAANNYARGHYTIGKEIIDLVLD

RIRKLADQCTGLQGFLVFHSFGGGTGSGFTSLLMERLSVDYGKKSKLEFSIYPAPQVSTA

VVEPYNSILTTHTTLEHSDCAFMVDNEAIYDICRRNLDIERPTYTNLNRLIGQIVSSITA

SLRFDGALNVDLTEFQTNLVPYPRIHFPLATYAPVISAEKAYHEQLSVAEITNACFEPAN

QMVKCDPRHGKYMACCLLYRGDVVPKDVNAAIATIKTKRTIQFVDWCPTGFKVGINYQPP

TVVPGGDLAKVQRAVCMLSNTTAIAEAWARLDHKFDLMYAKRAFVHWYVGEGMEEGEFSE

AREDMAALEKDYEEVGVDSVEGEGEEEGEEY

>sp|P17036|ZNF3_HUMAN 133 EVSLKRPLGNSPGER

METQADLVSQEPQALLDSALPSKVPAFSDKDSLGDEMLAAALLKAKSQELVTFEDVAVYF

IRKEWKRLEPAQRDLYRDVMLENYGNVFSLDRETRTENDQEISEDTRSHGVLLGRFQKDI

SQGLKFKEAYEREVSLKRPLGNSPGERLNRKMPDFGQVTVEEKLTPRGERSEKYNDFGNS

FTVNSNLISHQRLPVGDRPHKCDECSKSFNRTSDLIQHQRIHTGEKPYECNECGKAFSQS

SHLIQHQRIHTGEKPYECSDCGKTFSCSSALILHRRIHTGEKPYECNECGKTFSWSSTLT

HHQRIHTGEKPYACNECGKAFSRSSTLIHHQRIHTGEKPYECNECGKAFSQSSHLYQHQR

IHTGEKPYECMECGGKFTYSSGLIQHQRIHTGENPYECSECGKAFRYSSALVRHQRIHTG

EKPLNGIGMSKSSLRVTTELNIREST

>sp|P68371|TBB2C_HUMAN 18 AKFWEVISDEHGIDPT

MREIVHLQAGQCGNQIGAKFWEVISDEHGIDPTGTYHGDSDLQLERINVYYNEATGGKYV

PRAVLVDLEPGTMDSVRSGPFGQIFRPDNFVFGQSGAGNNWAKGHYTEGAELVDSVLDVV

RKEAESCDCLQGFQLTHSLGGGTGSGMGTLLISKIREEYPDRIMNTFSVVPSPKVSDTVV

EPYNATLSVHQLVENTDETYCIDNEALYDICFRTLKLTTPTYGDLNHLVSATMSGVTTCL

RFPGQLNADLRKLAVNMVPFPRLHFFMPGFAPLTSRGSQQYRALTVPELTQQMFDAKNMM

AACDPRHGRYLTVAAVFRGRMSMKEVDEQMLNVQNKNSSYFVEWIPNNVKTAVCDIPPRG

LKMSATFIGNSTAIQELFKRISEQFTAMFRRKAFLHWYTGEGMDEMEFTEAESNMNDLVS

EYQQYQDATAEEEGEFEEEAEEEVA

>sp|P50395|GDIB_HUMAN 374 EPIEQKFVSISDLLVPK

MNEEYDVIVLGTGLTECILSGIMSVNGKKVLHMDRNPYYGGESASITPLEDLYKRFKIPG

SPPESMGRGRDWNVDLIPKFLMANGQLVKMLLYTEVTRYLDFKVTEGSFVYKGGKIYKVP

STEAEALASSLMGLFEKRRFRKFLVYVANFDEKDPRTFEGIDPKKTTMRDVYKKFDLGQD

VIDFTGHALALYRTDDYLDQPCYETINRIKLYSESLARYGKSPYLYPLYGLGELPQGFAR

LSAIYGGTYMLNKPIEEIIVQNGKVIGVKSEGEIARCKQLICDPSYVKDRVEKVGQVIRV

ICILSHPIKNTNDANSCQIIIPQNQVNRKSDIYVCMISFAHNVAAQGKYIAIVSTTVETK

EPEKEIRPALELLEPIEQKFVSISDLLVPKDLGTESQIFISRTYDATTHFETTCDDIKNI

YKRMTGSEFDFEEMKRKKNDIYGED

>sp|Q9H993|CF211_HUMAN 285 IPWFVSDTTIHDFN

MAVVPASLSGQDVGSFAYLTIKDRIPQILTKVIDTLHRHKSEFFEKHGEEGVEAEKKAIS

LLSKLRNELQTDKPFIPLVEKFVDTDIWNQYLEYQQSLLNESDGKSRWFYSPWLLVECYM

YRRIHEAIIQSPPIDYFDVFKESKEQNFYGSQESIIALCTHLQQLIRTIEDLDENQLKDE

FFKLLQISLWGNKCDLSLSGGESSSQNTNVLNSLEDLKPFILLNDMEHLWSLLSNCKKTR

EKASATRVYIVLDNSGFELVTDLILADFLLSSELATEVHFYGKTIPWFVSDTTIHDFNWL

IEQVKHSNHKWMSKCGADWEEYIKMGKWVYHNHIFWTLPHEYCAMPQVAPDLYAELQKAH

LILFKGDLNYRKLTGDRKWEFSVPFHQALNGFHPAPLCTIRTLKAEIQVGLQPGQGEQLL

ASEPSWWTTGKYGIFQYDGPL

>sp|P06733|ENOA_HUMAN 127 GVPLYRHIADLAGNSEVI

MSILKIHAREIFDSRGNPTVEVDLFTSKGLFRAAVPSGASTGIYEALELRDNDKTRYMGK

GVSKAVEHINKTIAPALVSKKLNVTEQEKIDKLMIEMDGTENKSKFGANAILGVSLAVCK

AGAVEKGVPLYRHIADLAGNSEVILPVPAFNVINGGSHAGNKLAMQEFMILPVGAANFRE

AMRIGAEVYHNLKNVIKEKYGKDATNVGDEGGFAPNILENKEGLELLKTAIGKAGYTDKV

VIGMDVAASEFFRSGKYDLDFKSPDDPSRYISPDQLADLYKSFIKDYPVVSIEDPFDQDD

WGAWQKFTASAGIQVVGDDLTVTNPKRIAKAVNEKSCNCLLLKVNQIGSVTESLQACKLA

QANGWGVMVSHRSGETEDTFIADLVVGLCTGQIKTGAPCRSERLAKYNQLLRIEEELGSK

AKFAGRNFRNPLAK

>sp|Q99538|LGMN_HUMAN 98 GVPKDYTGEDVTPQN

MVWKVAVFLSVALGIGAVPIDDPEDGGKHWVVIVAGSNGWYNYRHQADACHAYQIIHRNG

IPDEQIVVMMYDDIAYSEDNPTPGIVINRPNGTDVYQGVPKDYTGEDVTPQNFLAVLRGD

AEAVKGIGSGKVLKSGPQDHVFIYFTDHGSTGILVFPNEDLHVKDLNETIHYMYKHKMYR

KMVFYIEACESGSMMNHLPDNINVYATTAANPRESSYACYYDEKRSTYLGDWYSVNWMED

SDVEDLTKETLHKQYHLVKSHTNTSHVMQYGNKTISTMKVMQFQGMKRKASSPVPLPPVT

HLDLTPSPDVPLTIMKRKLMNTNDLEESRQLTEEIQRHLDARHLIEKSVRKIVSLLAASE

AEVEQLLSERAPLTGHSCYPEALLHFRTHCFNWHSPTYEYALRHLYVLVNLCEKPYPLHR

IKLSMDHVCLGHY

>sp|P09110|THIK_HUMAN 260 LKPAFKKDGSTTAGN

MQRLQVVLGHLRGPADSGWMPQAAPCLSGAPQASAADVVVVHGRRTAICRAGRGGFKDTT

PDELLSAVMTAVLKDVNLRPEQLGDICVGNVLQPGAGAIMARIAQFLSDIPETVPLSTVN

RQCSSGLQAVASIAGGIRNGSYDIGMACGVESMSLADRGNPGNITSRLMEKEKARDCLIP

MGITSENVAERFGISREKQDTFALASQQKAARAQSKGCFQAEIVPVTTTVHDDKGTKRSI

TVTQDEGIRPSTTMEGLAKLKPAFKKDGSTTAGNSSQVSDGAAAILLARRSKAEELGLPI

LGVLRSYAVVGVPPDIMGIGPAYAIPVALQKAGLTVSDVDIFEINEAFASQAAYCVEKLR

LPPEKVNPLGGAVALGHPLGCTGARQVITLLNELKRRGKRAYGVVSMCIGTGMGAAAVFE

YPGN

>sp|P08908|5HT1A_HUMAN 359 LPFFIVALVLPFCESSCH

MDVLSPGQGNNTTSPPAPFETGGNTTGISDVTVSYQVITSLLLGTLIFCAVLGNACVVAA

IALERSLQNVANYLIGSLAVTDLMVSVLVLPMAALYQVLNKWTLGQVTCDLFIALDVLCC

TSSILHLCAIALDRYWAITDPIDYVNKRTPRRAAALISLTWLIGFLISIPPMLGWRTPED

RSDPDACTISKDHGYTIYSTFGAFYIPLLLMLVLYGRIFRAARFRIRKTVKKVEKTGADT

RHGASPAPQPKKSVNGESGSRNWRLGVESKAGGALCANGAVRQGDDGAALEVIEVHRVGN

SKEHLPLPSEAGPTPCAPASFERKNERNAEAKRKMALARERKTVKTLGIIMGTFILCWLP

FFIVALVLPFCESSCHMPTLLGAIINWLGYSNSLLNPVIYAYFNKDFQNAFKKIIKCKFC

RQ

>sp|P00558|PGK1_HUMAN 338 GPVGVFEWEAFARGT

MSLSNKLTLDKLDVKGKRVVMRVDFNVPMKNNQITNNQRIKAAVPSIKFCLDNGAKSVVL

MSHLGRPDGVPMPDKYSLEPVAVELKSLLGKDVLFLKDCVGPEVEKACANPAAGSVILLE

NLRFHVEEEGKGKDASGNKVKAEPAKIEAFRASLSKLGDVYVNDAFGTAHRAHSSMVGVN

LPQKAGGFLMKKELNYFAKALESPERPFLAILGGAKVADKIQLINNMLDKVNEMIIGGGM

AFTFLKVLNNMEIGTSLFDEEGAKIVKDLMSKAEKNGVKITLPVDFVTADKFDENAKTGQ

ATVASGIPAGWMGLDCGPESSKKYAEAVTRAKQIVWNGPVGVFEWEAFARGTKALMDEVV

KATSRGCITIIGGGDTATCCAKWNTEDKVSHVSTGGGASLELLEGKVLPGVDALSNI

>sp|P00558|PGK1_HUMAN 18 RVVMRVDFNVPMKN

MSLSNKLTLDKLDVKGKRVVMRVDFNVPMKNNQITNNQRIKAAVPSIKFCLDNGAKSVVL

MSHLGRPDGVPMPDKYSLEPVAVELKSLLGKDVLFLKDCVGPEVEKACANPAAGSVILLE

NLRFHVEEEGKGKDASGNKVKAEPAKIEAFRASLSKLGDVYVNDAFGTAHRAHSSMVGVN

LPQKAGGFLMKKELNYFAKALESPERPFLAILGGAKVADKIQLINNMLDKVNEMIIGGGM

AFTFLKVLNNMEIGTSLFDEEGAKIVKDLMSKAEKNGVKITLPVDFVTADKFDENAKTGQ

ATVASGIPAGWMGLDCGPESSKKYAEAVTRAKQIVWNGPVGVFEWEAFARGTKALMDEVV

KATSRGCITIIGGGDTATCCAKWNTEDKVSHVSTGGGASLELLEGKVLPGVDALSNI

>sp|P00558|PGK1_HUMAN 217 VADKIQLINNMLD

MSLSNKLTLDKLDVKGKRVVMRVDFNVPMKNNQITNNQRIKAAVPSIKFCLDNGAKSVVL

MSHLGRPDGVPMPDKYSLEPVAVELKSLLGKDVLFLKDCVGPEVEKACANPAAGSVILLE

NLRFHVEEEGKGKDASGNKVKAEPAKIEAFRASLSKLGDVYVNDAFGTAHRAHSSMVGVN

LPQKAGGFLMKKELNYFAKALESPERPFLAILGGAKVADKIQLINNMLDKVNEMIIGGGM

AFTFLKVLNNMEIGTSLFDEEGAKIVKDLMSKAEKNGVKITLPVDFVTADKFDENAKTGQ

ATVASGIPAGWMGLDCGPESSKKYAEAVTRAKQIVWNGPVGVFEWEAFARGTKALMDEVV

KATSRGCITIIGGGDTATCCAKWNTEDKVSHVSTGGGASLELLEGKVLPGVDALSNI

>sp|P07339|CATD_HUMAN 303 GPVDEVRELQKAIGAVPL

MQPSSLLPLALCLLAAPASALVRIPLHKFTSIRRTMSEVGGSVEDLIAKGPVSKYSQAVP

AVTEGPIPEVLKNYMDAQYYGEIGIGTPPQCFTVVFDTGSSNLWVPSIHCKLLDIACWIH

HKYNSDKSSTYVKNGTSFDIHYGSGSLSGYLSQDTVSVPCQSASSASALGGVKVERQVFG

EATKQPGITFIAAKFDGILGMAYPRISVNNVLPVFDNLMQQKLVDQNIFSFYLSRDPDAQ

PGGELMLGGTDSKYYKGSLSYLNVTRKAYWQVHLDQVEVASGLTLCKEGCEAIVDTGTSL

MVGPVDEVRELQKAIGAVPLIQGEYMIPCEKVSTLPAITLKLGGKGYKLSPEDYTLKVSQ

AGKTLCLSGFMGMDIPPPSGPLWILGDVFIGRYYTVFDRDNNRVGFAEAARL

>sp|P07339|CATD_HUMAN 228 IFSFYLSRDPDAQPG

MQPSSLLPLALCLLAAPASALVRIPLHKFTSIRRTMSEVGGSVEDLIAKGPVSKYSQAVP

AVTEGPIPEVLKNYMDAQYYGEIGIGTPPQCFTVVFDTGSSNLWVPSIHCKLLDIACWIH

HKYNSDKSSTYVKNGTSFDIHYGSGSLSGYLSQDTVSVPCQSASSASALGGVKVERQVFG

EATKQPGITFIAAKFDGILGMAYPRISVNNVLPVFDNLMQQKLVDQNIFSFYLSRDPDAQ

PGGELMLGGTDSKYYKGSLSYLNVTRKAYWQVHLDQVEVASGLTLCKEGCEAIVDTGTSL

MVGPVDEVRELQKAIGAVPLIQGEYMIPCEKVSTLPAITLKLGGKGYKLSPEDYTLKVSQ

AGKTLCLSGFMGMDIPPPSGPLWILGDVFIGRYYTVFDRDNNRVGFAEAARL

>sp|P07339|CATD_HUMAN 390 IGRYYTVFDRDNNRVGFA

MQPSSLLPLALCLLAAPASALVRIPLHKFTSIRRTMSEVGGSVEDLIAKGPVSKYSQAVP

AVTEGPIPEVLKNYMDAQYYGEIGIGTPPQCFTVVFDTGSSNLWVPSIHCKLLDIACWIH

HKYNSDKSSTYVKNGTSFDIHYGSGSLSGYLSQDTVSVPCQSASSASALGGVKVERQVFG

EATKQPGITFIAAKFDGILGMAYPRISVNNVLPVFDNLMQQKLVDQNIFSFYLSRDPDAQ

PGGELMLGGTDSKYYKGSLSYLNVTRKAYWQVHLDQVEVASGLTLCKEGCEAIVDTGTSL

MVGPVDEVRELQKAIGAVPLIQGEYMIPCEKVSTLPAITLKLGGKGYKLSPEDYTLKVSQ

AGKTLCLSGFMGMDIPPPSGPLWILGDVFIGRYYTVFDRDNNRVGFAEAARL

>sp|P07339|CATD_HUMAN 233 LSRDPDAQPGGE

MQPSSLLPLALCLLAAPASALVRIPLHKFTSIRRTMSEVGGSVEDLIAKGPVSKYSQAVP

AVTEGPIPEVLKNYMDAQYYGEIGIGTPPQCFTVVFDTGSSNLWVPSIHCKLLDIACWIH

HKYNSDKSSTYVKNGTSFDIHYGSGSLSGYLSQDTVSVPCQSASSASALGGVKVERQVFG

EATKQPGITFIAAKFDGILGMAYPRISVNNVLPVFDNLMQQKLVDQNIFSFYLSRDPDAQ

PGGELMLGGTDSKYYKGSLSYLNVTRKAYWQVHLDQVEVASGLTLCKEGCEAIVDTGTSL

MVGPVDEVRELQKAIGAVPLIQGEYMIPCEKVSTLPAITLKLGGKGYKLSPEDYTLKVSQ

AGKTLCLSGFMGMDIPPPSGPLWILGDVFIGRYYTVFDRDNNRVGFAEAARL

>sp|P54727|RD23B_HUMAN 313 LLQQISQHQEHF

MQVTLKTLQQQTFKIDIDPEETVKALKEKIESEKGKDAFPVAGQKLIYAGKILNDDTALK

EYKIDEKNFVVVMVTKPKAVSTPAPATTQQSAPASTTAVTSSTTTTVAQAPTPVPALAPT

STPASITPASATASSEPAPASAAKQEKPAEKPAETPVATSPTATDSTSGDSSRSNLFEDA

TSALVTGQSYENMVTEIMSMGYEREQVIAALRASFNNPDRAVEYLLMGIPGDRESQAVVD

PPQAASTGAPQSSAVAAAAATTTATTTTTSSGGHPLEFLRNQPQFQQMRQIIQQNPSLLP

ALLQQIGRENPQLLQQISQHQEHFIQMLNEPVQEAGGQGGGGGGGSGGIAEAGSGHMNYI

QVTPQEKEAIERLKALGFPEGLVIQAYFACEKNENLAANFLLQQNFDED

>sp|P15907|SIAT1_HUMAN 206 DAVLRFNGAPTANFQQDV

MIHTNLKKKFSCCVLVFLLFAVICVWKEKKKGSYYDSFKLQTKEFQVLKSLGKLAMGSDS

QSVSSSSTQDPHRGRQTLGSLRGLAKAKPEASFQVWNKDSSSKNLIPRLQKIWKNYLSMN

KYKVSYKGPGPGIKFSAEALRCHLRDHVNVSMVEVTDFPFNTSEWEGYLPKESIRTKAGP

WGRCAVVSSAGSLKSSQLGREIDDHDAVLRFNGAPTANFQQDVGTKTTIRLMNSQLVTTE

KRFLKDSLYNEGILIVWDPSVYHSDIPKWYQNPDYNFFNNYKTYRKLHPNQPFYILKPQM

PWELWDILQEISPEEIQPNPPSSGMLGIIIMMTLCDQVDIYEFLPSKRKTDVCYYYQKFF

DSACTMGAYHPLLYEKNLVKHLNQGTDEDIYLLGKATLPGFRTIHC

>sp|P15907|SIAT1_HUMAN 252 GILIVWDPSVYHSDIP

MIHTNLKKKFSCCVLVFLLFAVICVWKEKKKGSYYDSFKLQTKEFQVLKSLGKLAMGSDS

QSVSSSSTQDPHRGRQTLGSLRGLAKAKPEASFQVWNKDSSSKNLIPRLQKIWKNYLSMN

KYKVSYKGPGPGIKFSAEALRCHLRDHVNVSMVEVTDFPFNTSEWEGYLPKESIRTKAGP

WGRCAVVSSAGSLKSSQLGREIDDHDAVLRFNGAPTANFQQDVGTKTTIRLMNSQLVTTE

KRFLKDSLYNEGILIVWDPSVYHSDIPKWYQNPDYNFFNNYKTYRKLHPNQPFYILKPQM

PWELWDILQEISPEEIQPNPPSSGMLGIIIMMTLCDQVDIYEFLPSKRKTDVCYYYQKFF

DSACTMGAYHPLLYEKNLVKHLNQGTDEDIYLLGKATLPGFRTIHC

>sp|P15907|SIAT1_HUMAN 288 HPNQPFYILKPQMPWELW

MIHTNLKKKFSCCVLVFLLFAVICVWKEKKKGSYYDSFKLQTKEFQVLKSLGKLAMGSDS

QSVSSSSTQDPHRGRQTLGSLRGLAKAKPEASFQVWNKDSSSKNLIPRLQKIWKNYLSMN

KYKVSYKGPGPGIKFSAEALRCHLRDHVNVSMVEVTDFPFNTSEWEGYLPKESIRTKAGP

WGRCAVVSSAGSLKSSQLGREIDDHDAVLRFNGAPTANFQQDVGTKTTIRLMNSQLVTTE

KRFLKDSLYNEGILIVWDPSVYHSDIPKWYQNPDYNFFNNYKTYRKLHPNQPFYILKPQM

PWELWDILQEISPEEIQPNPPSSGMLGIIIMMTLCDQVDIYEFLPSKRKTDVCYYYQKFF

DSACTMGAYHPLLYEKNLVKHLNQGTDEDIYLLGKATLPGFRTIHC

>sp|P15907|SIAT1_HUMAN 106 IPRLQKIWKNYLSMNKY

MIHTNLKKKFSCCVLVFLLFAVICVWKEKKKGSYYDSFKLQTKEFQVLKSLGKLAMGSDS

QSVSSSSTQDPHRGRQTLGSLRGLAKAKPEASFQVWNKDSSSKNLIPRLQKIWKNYLSMN

KYKVSYKGPGPGIKFSAEALRCHLRDHVNVSMVEVTDFPFNTSEWEGYLPKESIRTKAGP

WGRCAVVSSAGSLKSSQLGREIDDHDAVLRFNGAPTANFQQDVGTKTTIRLMNSQLVTTE

KRFLKDSLYNEGILIVWDPSVYHSDIPKWYQNPDYNFFNNYKTYRKLHPNQPFYILKPQM

PWELWDILQEISPEEIQPNPPSSGMLGIIIMMTLCDQVDIYEFLPSKRKTDVCYYYQKFF

DSACTMGAYHPLLYEKNLVKHLNQGTDEDIYLLGKATLPGFRTIHC

>sp|P05121|PAI1_HUMAN 261 AAPYEKEVPLSALTNILSAQL

MQMSPALTCLVLGLALVFGEGSAVHHPPSYVAHLASDFGVRVFQQVAQASKDRNVVFSPY

GVASVLAMLQLTTGGETQQQIQAAMGFKIDDKGMAPALRHLYKELMGPWNKDEISTTDAI

FVQRDLKLVQGFMPHFFRLFRSTVKQVDFSEVERARFIINDWVKTHTKGMISNLLGKGAV

DQLTRLVLVNALYFNGQWKTPFPDSSTHRRLFHKSDGSTVSVPMMAQTNKFNYTEFTTPD

GHYYDILELPYHGDTLSMFIAAPYEKEVPLSALTNILSAQLISHWKGNMTRLPRLLVLPK

FSLETEVDLRKPLENLGMTDMFRQFQADFTSLSDQEPLHVAQALQKVKIEVNESGTVASS

STAVIVSARMAPEEIIMDRPFLFVVRHNPTGTVLFMGQVMEP

>sp|P05121|PAI1_HUMAN 378 DRPFLFVVRHNPTGTVLFM

MQMSPALTCLVLGLALVFGEGSAVHHPPSYVAHLASDFGVRVFQQVAQASKDRNVVFSPY

GVASVLAMLQLTTGGETQQQIQAAMGFKIDDKGMAPALRHLYKELMGPWNKDEISTTDAI

FVQRDLKLVQGFMPHFFRLFRSTVKQVDFSEVERARFIINDWVKTHTKGMISNLLGKGAV

DQLTRLVLVNALYFNGQWKTPFPDSSTHRRLFHKSDGSTVSVPMMAQTNKFNYTEFTTPD

GHYYDILELPYHGDTLSMFIAAPYEKEVPLSALTNILSAQLISHWKGNMTRLPRLLVLPK

FSLETEVDLRKPLENLGMTDMFRQFQADFTSLSDQEPLHVAQALQKVKIEVNESGTVASS

STAVIVSARMAPEEIIMDRPFLFVVRHNPTGTVLFMGQVMEP

>sp|P05121|PAI1_HUMAN 133 MPHFFRLFRSTVKQVD

MQMSPALTCLVLGLALVFGEGSAVHHPPSYVAHLASDFGVRVFQQVAQASKDRNVVFSPY

GVASVLAMLQLTTGGETQQQIQAAMGFKIDDKGMAPALRHLYKELMGPWNKDEISTTDAI

FVQRDLKLVQGFMPHFFRLFRSTVKQVDFSEVERARFIINDWVKTHTKGMISNLLGKGAV

DQLTRLVLVNALYFNGQWKTPFPDSSTHRRLFHKSDGSTVSVPMMAQTNKFNYTEFTTPD

GHYYDILELPYHGDTLSMFIAAPYEKEVPLSALTNILSAQLISHWKGNMTRLPRLLVLPK

FSLETEVDLRKPLENLGMTDMFRQFQADFTSLSDQEPLHVAQALQKVKIEVNESGTVASS

STAVIVSARMAPEEIIMDRPFLFVVRHNPTGTVLFMGQVMEP

>sp|P14091|CATE_HUMAN 89 QNFTVIFDTGSSNLWV

MKTLLLLLLVLLELGEAQGSLHRVPLRRHPSLKKKLRARSQLSEFWKSHNLDMIQFTESC

SMDQSAKEPLINYLDMEYFGTISIGSPPQNFTVIFDTGSSNLWVPSVYCTSPACKTHSRF

QPSQSSTYSQPGQSFSIQYGTGSLSGIIGADQVSAFATQVEGLTVVGQQFGESVTEPGQT

FVDAEFDGILGLGYPSLAVGGVTPVFDNMMAQNLVDLPMFSVYMSSNPEGGAGSELIFGG

YDHSHFSGSLNWVPVTKQAYWQIALDNIQVGGTVMFCSEGCQAIVDTGTSLITGPSDKIK

QLQNAIGAAPVDGEYAVECANLNVMPDVTFTINGVPYTLSPTAYTLLDFVDGMQFCSSGF

QGLDIHPPAGPLWILGDVFIRQFYSVFDRGNNRVGLAPAVP

>sp|P14091|CATE_HUMAN 89 QNFTVIFDTGSSNLWVPSVYCTSP

MKTLLLLLLVLLELGEAQGSLHRVPLRRHPSLKKKLRARSQLSEFWKSHNLDMIQFTESC

SMDQSAKEPLINYLDMEYFGTISIGSPPQNFTVIFDTGSSNLWVPSVYCTSPACKTHSRF

QPSQSSTYSQPGQSFSIQYGTGSLSGIIGADQVSAFATQVEGLTVVGQQFGESVTEPGQT

FVDAEFDGILGLGYPSLAVGGVTPVFDNMMAQNLVDLPMFSVYMSSNPEGGAGSELIFGG

YDHSHFSGSLNWVPVTKQAYWQIALDNIQVGGTVMFCSEGCQAIVDTGTSLITGPSDKIK

QLQNAIGAAPVDGEYAVECANLNVMPDVTFTINGVPYTLSPTAYTLLDFVDGMQFCSSGF

QGLDIHPPAGPLWILGDVFIRQFYSVFDRGNNRVGLAPAVP

>sp|P36222|CH3L1_HUMAN 106 FSKIASNTQ

MGVKASQTGFVVLVLLQCCSAYKLVCYYTSWSQYREGDGSCFPDALDRFLCTHIIYSFAN

ISNDHIDTWEWNDVTLYGMLNTLKNRNPNLKTLLSVGGWNFGSQRFSKIASNTQSRRTFI

KSVPPFLRTHGFDGLDLAWLYPGRRDKQHFTTLIKEMKAEFIKEAQPGKKQLLLSAALSA

GKVTIDSSYDIAKISQHLDFISIMTYDFHGAWRGTTGHHSPLFRGQEDASPDRFSNTDYA

VGYMLRLGAPASKLVMGIPTFGRSFTLASSETGVGAPISGPGIPGRFTKEAGTLAYYEIC

DFLRGATVHRILGQQVPYATKGNQWVGYDDQESVKSKVQYLKDRQLAGAMVWALDLDDFQ

GSFCGQDLRFPLTNAIKDALAAT

>sp|O43567|RNF13_HUMAN 145 IPSVFIGESSANSLKD

MLLSIGMLMLSATQVYTILTVQLFAFLNLLPVEADILAYNFENASQTFDDLPARFGYRLP

AEGLKGFLINSKPENACEPIVPPPVKDNSSGTFIVLIRRLDCNFDIKVLNAQRAGYKAAI

VHNVDSDDLISMGSNDIEVLKKIDIPSVFIGESSANSLKDEFTYEKGGHLILVPEFSLPL

EYYLIPFLIIVGICLILIVIFMITKFVQDRHRARRNRLRKDQLKKLPVHKFKKGDEYDVC

AICLDEYEDGDKLRILPCSHAYHCKCVDPWLTKTKKTCPVCKQKVVPSQGDSDSDTDSSQ

EENEVTEHTPLLRPLASVSAQSFGALSESRSHQNMTESSDYEEDDNEDTDSSDAENEINE

HDVVVQLQPNGERDYNIANTV

>sp|P32248|CCR7_HUMAN 305 NIAYDVTYSLACVR

MDLGKPMKSVLVVALLVIFQVCLCQDEVTDDYIGDNTTVDYTLFESLCSKKDVRNFKAWF

LPIMYSIICFVGLLGNGLVVLTYIYFKRLKTMTDTYLLNLAVADILFLLTLPFWAYSAAK

SWVFGVHFCKLIFAIYKMSFFSGMLLLLCISIDRYVAIVQAVSAHRHRARVLLISKLSCV

GIWILATVLSIPELLYSDLQRSSSEQAMRCSLITEHVEAFITIQVAQMVIGFLVPLLAMS

FCYLVIIRTLLQARNFERNKAIKVIIAVVVVFIVFQLPYNGVVLAQTVANFNITSSTCEL

SKQLNIAYDVTYSLACVRCCVNPFLYAFIGVKFRNDLFKLFKDLGCLSQEQLRQWSSCRH

IRRSSMSVEAETTTTFSP

>sp|Q14344|GNA13_HUMAN 269 LNIFETIVNNRVFS

MADFLPSRSVLSVCFPGCLLTSGEAEQQRKSKEIDKCLSREKTYVKRLVKILLLGAGESG

KSTFLKQMRIIHGQDFDQRAREEFRPTIYSNVIKGMRVLVDAREKLHIPWGDNSNQQHGD

KMMSFDTRAPMAAQGMVETRVFLQYLPAIRALWADSGIQNAYDRRREFQLGESVKYFLDN

LDKLGEPDYIPSQQDILLARRPTKGIHEYDFEIKNVPFKMVDVGGQRSERKRWFECFDSV

TSILFLVSSSEFDQVLMEDRLTNRLTESLNIFETIVNNRVFSNVSIILFLNKTDLLEEKV

QIVSIKDYFLEFEGDPHCLRDVQKFLVECFRNKRRDQQQKPLYHHFTTAINTENIRLVFR

DVKDTILHDNLKQLMLQ

>sp|P68133|ACTS_HUMAN 31 AVFPSIVGRPRHQGVMV

MCDEDETTALVCDNGSGLVKAGFAGDDAPRAVFPSIVGRPRHQGVMVGMGQKDSYVGDEA

QSKRGILTLKYPIEHGIITNWDDMEKIWHHTFYNELRVAPEEHPTLLTEAPLNPKANREK

MTQIMFETFNVPAMYVAIQAVLSLYASGRTTGIVLDSGDGVTHNVPIYEGYALPHAIMRL

DLAGRDLTDYLMKILTERGYSFVTTAEREIVRDIKEKLCYVALDFENEMATAASSSSLEK

SYELPDGQVITIGNERFRCPETLFQPSFIGMESAGIHETTYNSIMKCDIDIRKDLYANNV

MSGGTTMYPGIADRMQKEITALAPSTMKIKIIAPPERKYSVWIGGSILASLSTFQQMWIT

KQEYDEAGPSIVHRKCF

>sp|P68133|ACTS_HUMAN 49 MGQKDSYVGDEAQSKR

MCDEDETTALVCDNGSGLVKAGFAGDDAPRAVFPSIVGRPRHQGVMVGMGQKDSYVGDEA

QSKRGILTLKYPIEHGIITNWDDMEKIWHHTFYNELRVAPEEHPTLLTEAPLNPKANREK

MTQIMFETFNVPAMYVAIQAVLSLYASGRTTGIVLDSGDGVTHNVPIYEGYALPHAIMRL

DLAGRDLTDYLMKILTERGYSFVTTAEREIVRDIKEKLCYVALDFENEMATAASSSSLEK

SYELPDGQVITIGNERFRCPETLFQPSFIGMESAGIHETTYNSIMKCDIDIRKDLYANNV

MSGGTTMYPGIADRMQKEITALAPSTMKIKIIAPPERKYSVWIGGSILASLSTFQQMWIT

KQEYDEAGPSIVHRKCF

>sp|P68133|ACTS_HUMAN 205 TAEREIVRDIKEK

MCDEDETTALVCDNGSGLVKAGFAGDDAPRAVFPSIVGRPRHQGVMVGMGQKDSYVGDEA

QSKRGILTLKYPIEHGIITNWDDMEKIWHHTFYNELRVAPEEHPTLLTEAPLNPKANREK

MTQIMFETFNVPAMYVAIQAVLSLYASGRTTGIVLDSGDGVTHNVPIYEGYALPHAIMRL

DLAGRDLTDYLMKILTERGYSFVTTAEREIVRDIKEKLCYVALDFENEMATAASSSSLEK

SYELPDGQVITIGNERFRCPETLFQPSFIGMESAGIHETTYNSIMKCDIDIRKDLYANNV

MSGGTTMYPGIADRMQKEITALAPSTMKIKIIAPPERKYSVWIGGSILASLSTFQQMWIT

KQEYDEAGPSIVHRKCF

>sp|Q8NCH0|CHSTE_HUMAN 350 DVLPKYILDFSL

MFPRPLTPLAAPNGAEPLGRALRRAPLGRARAGLGGPPLLLPSMLMFAVIVASSGLLLMI

ERGILAEMKPLPLHPPGREGTAWRGKAPKPGGLSLRAGDADLQVRQDVRNRTLRAVCGQP

GMPRDPWDLPVGQRRTLLRHILVSDRYRFLYCYVPKVACSNWKRVMKVLAGVLDSVDVRL

KMDHRSDLVFLADLRPEEIRYRLQHYFKFLFVREPLERLLSAYRNKFGEIREYQQRYGAE

IVRRYRAGAGPSPAGDDVTFPEFLRYLVDEDPERMNEHWMPVYHLCQPCAVHYDFVGSYE

RLEADANQVLEWVRAPPHVRFPARQAWYRPASPESLHYHLCSAPRALLQDVLPKYILDFS

LFAYPLPNVTKEACQQ

>sp|Q8NCH0|CHSTE_HUMAN 352 LPKYILDFSL

MFPRPLTPLAAPNGAEPLGRALRRAPLGRARAGLGGPPLLLPSMLMFAVIVASSGLLLMI

ERGILAEMKPLPLHPPGREGTAWRGKAPKPGGLSLRAGDADLQVRQDVRNRTLRAVCGQP

GMPRDPWDLPVGQRRTLLRHILVSDRYRFLYCYVPKVACSNWKRVMKVLAGVLDSVDVRL

KMDHRSDLVFLADLRPEEIRYRLQHYFKFLFVREPLERLLSAYRNKFGEIREYQQRYGAE

IVRRYRAGAGPSPAGDDVTFPEFLRYLVDEDPERMNEHWMPVYHLCQPCAVHYDFVGSYE

RLEADANQVLEWVRAPPHVRFPARQAWYRPASPESLHYHLCSAPRALLQDVLPKYILDFS

LFAYPLPNVTKEACQQ

>sp|P63261|ACTG_HUMAN 182 GRDLTDYLMKILTERGYSFT

MEEEIAALVIDNGSGMCKAGFAGDDAPRAVFPSIVGRPRHQGVMVGMGQKDSYVGDEAQS

KRGILTLKYPIEHGIVTNWDDMEKIWHHTFYNELRVAPEEHPVLLTEAPLNPKANREKMT

QIMFETFNTPAMYVAIQAVLSLYASGRTTGIVMDSGDGVTHTVPIYEGYALPHAILRLDL

AGRDLTDYLMKILTERGYSFTTTAEREIVRDIKEKLCYVALDFEQEMATAASSSSLEKSY

ELPDGQVITIGNERFRCPEALFQPSFLGMESCGIHETTFNSIMKCDVDIRKDLYANTVLS

GGTTMYPGIADRMQKEITALAPSTMKIKIIAPPERKYSVWIGGSILASLSTFQQMWISKQ

EYDESGPSIVHRKCF

>sp|P63261|ACTG_HUMAN 66 TLKYPIEHGIVTNWDD

MEEEIAALVIDNGSGMCKAGFAGDDAPRAVFPSIVGRPRHQGVMVGMGQKDSYVGDEAQS

KRGILTLKYPIEHGIVTNWDDMEKIWHHTFYNELRVAPEEHPVLLTEAPLNPKANREKMT

QIMFETFNTPAMYVAIQAVLSLYASGRTTGIVMDSGDGVTHTVPIYEGYALPHAILRLDL

AGRDLTDYLMKILTERGYSFTTTAEREIVRDIKEKLCYVALDFEQEMATAASSSSLEKSY

ELPDGQVITIGNERFRCPEALFQPSFLGMESCGIHETTFNSIMKCDVDIRKDLYANTVLS

GGTTMYPGIADRMQKEITALAPSTMKIKIIAPPERKYSVWIGGSILASLSTFQQMWISKQ

EYDESGPSIVHRKCF

>sp|P63261|ACTG_HUMAN 96 VAPEEHPVLLTEAPLNPKA

MEEEIAALVIDNGSGMCKAGFAGDDAPRAVFPSIVGRPRHQGVMVGMGQKDSYVGDEAQS

KRGILTLKYPIEHGIVTNWDDMEKIWHHTFYNELRVAPEEHPVLLTEAPLNPKANREKMT

QIMFETFNTPAMYVAIQAVLSLYASGRTTGIVMDSGDGVTHTVPIYEGYALPHAILRLDL

AGRDLTDYLMKILTERGYSFTTTAEREIVRDIKEKLCYVALDFEQEMATAASSSSLEKSY

ELPDGQVITIGNERFRCPEALFQPSFLGMESCGIHETTFNSIMKCDVDIRKDLYANTVLS

GGTTMYPGIADRMQKEITALAPSTMKIKIIAPPERKYSVWIGGSILASLSTFQQMWISKQ

EYDESGPSIVHRKCF

>sp|P63261|ACTG_HUMAN 69 YPIEHGIVTNWDDM

MEEEIAALVIDNGSGMCKAGFAGDDAPRAVFPSIVGRPRHQGVMVGMGQKDSYVGDEAQS

KRGILTLKYPIEHGIVTNWDDMEKIWHHTFYNELRVAPEEHPVLLTEAPLNPKANREKMT

QIMFETFNTPAMYVAIQAVLSLYASGRTTGIVMDSGDGVTHTVPIYEGYALPHAILRLDL

AGRDLTDYLMKILTERGYSFTTTAEREIVRDIKEKLCYVALDFEQEMATAASSSSLEKSY

ELPDGQVITIGNERFRCPEALFQPSFLGMESCGIHETTFNSIMKCDVDIRKDLYANTVLS

GGTTMYPGIADRMQKEITALAPSTMKIKIIAPPERKYSVWIGGSILASLSTFQQMWISKQ

EYDESGPSIVHRKCF

>sp|Q95604|1C17_HUMAN 53 DDTQFVRFDSDAASP

MRVMAPQALLLLLSGALALIETWAGSHSMRYFYTAVSRPGRGEPRFIAVGYVDDTQFVRF

DSDAASPRGEPRAPWVEQEGPEYWDRETQKYKRQAQADRVNLRKLRGYYNQSEAGSHTIQ

RMYGCDLGPDGRLLRGYNQFAYDGKDYIALNEDLRSWTAADTAAQISQRKLEAAREAEQL

RAYLEGECVEWLRGYLENGKETLQRAERPKTHVTHHPVSDHEATLRCWALGFYPAEITLT

WQRDGEDQTQDTELVETRPAGDGTFQKWAAVVVPSGQEQRYTCHVQHEGLQEPCTLRWKP

SSQPTIPNLGIVSGPAVLAVLAVLAVLAVLGAVVAAVIHRRKSSGGKGGSCSQAASSNSA

QGSDESLIACKA

>sp|Q95604|1C17_HUMAN 53 DDTQFVRFDSDAASPR

MRVMAPQALLLLLSGALALIETWAGSHSMRYFYTAVSRPGRGEPRFIAVGYVDDTQFVRF

DSDAASPRGEPRAPWVEQEGPEYWDRETQKYKRQAQADRVNLRKLRGYYNQSEAGSHTIQ

RMYGCDLGPDGRLLRGYNQFAYDGKDYIALNEDLRSWTAADTAAQISQRKLEAAREAEQL

RAYLEGECVEWLRGYLENGKETLQRAERPKTHVTHHPVSDHEATLRCWALGFYPAEITLT

WQRDGEDQTQDTELVETRPAGDGTFQKWAAVVVPSGQEQRYTCHVQHEGLQEPCTLRWKP

SSQPTIPNLGIVSGPAVLAVLAVLAVLAVLGAVVAAVIHRRKSSGGKGGSCSQAASSNSA

QGSDESLIACKA

>sp|Q95604|1C17_HUMAN 53 DDTQFVRFDSDAASPRGE

MRVMAPQALLLLLSGALALIETWAGSHSMRYFYTAVSRPGRGEPRFIAVGYVDDTQFVRF

DSDAASPRGEPRAPWVEQEGPEYWDRETQKYKRQAQADRVNLRKLRGYYNQSEAGSHTIQ

RMYGCDLGPDGRLLRGYNQFAYDGKDYIALNEDLRSWTAADTAAQISQRKLEAAREAEQL

RAYLEGECVEWLRGYLENGKETLQRAERPKTHVTHHPVSDHEATLRCWALGFYPAEITLT

WQRDGEDQTQDTELVETRPAGDGTFQKWAAVVVPSGQEQRYTCHVQHEGLQEPCTLRWKP

SSQPTIPNLGIVSGPAVLAVLAVLAVLAVLGAVVAAVIHRRKSSGGKGGSCSQAASSNSA

QGSDESLIACKA

>sp|Q95604|1C17_HUMAN 53 DDTQFVRFDSDAASPRGEPR

MRVMAPQALLLLLSGALALIETWAGSHSMRYFYTAVSRPGRGEPRFIAVGYVDDTQFVRF

DSDAASPRGEPRAPWVEQEGPEYWDRETQKYKRQAQADRVNLRKLRGYYNQSEAGSHTIQ

RMYGCDLGPDGRLLRGYNQFAYDGKDYIALNEDLRSWTAADTAAQISQRKLEAAREAEQL

RAYLEGECVEWLRGYLENGKETLQRAERPKTHVTHHPVSDHEATLRCWALGFYPAEITLT

WQRDGEDQTQDTELVETRPAGDGTFQKWAAVVVPSGQEQRYTCHVQHEGLQEPCTLRWKP

SSQPTIPNLGIVSGPAVLAVLAVLAVLAVLGAVVAAVIHRRKSSGGKGGSCSQAASSNSA

QGSDESLIACKA

>sp|Q95604|1C17_HUMAN 143 DGKDYIALNEDLRSWTAA

MRVMAPQALLLLLSGALALIETWAGSHSMRYFYTAVSRPGRGEPRFIAVGYVDDTQFVRF

DSDAASPRGEPRAPWVEQEGPEYWDRETQKYKRQAQADRVNLRKLRGYYNQSEAGSHTIQ

RMYGCDLGPDGRLLRGYNQFAYDGKDYIALNEDLRSWTAADTAAQISQRKLEAAREAEQL

RAYLEGECVEWLRGYLENGKETLQRAERPKTHVTHHPVSDHEATLRCWALGFYPAEITLT

WQRDGEDQTQDTELVETRPAGDGTFQKWAAVVVPSGQEQRYTCHVQHEGLQEPCTLRWKP

SSQPTIPNLGIVSGPAVLAVLAVLAVLAVLGAVVAAVIHRRKSSGGKGGSCSQAASSNSA

QGSDESLIACKA

>sp|Q95604|1C17_HUMAN 54 DTQFVRFDSDAASP

MRVMAPQALLLLLSGALALIETWAGSHSMRYFYTAVSRPGRGEPRFIAVGYVDDTQFVRF

DSDAASPRGEPRAPWVEQEGPEYWDRETQKYKRQAQADRVNLRKLRGYYNQSEAGSHTIQ

RMYGCDLGPDGRLLRGYNQFAYDGKDYIALNEDLRSWTAADTAAQISQRKLEAAREAEQL

RAYLEGECVEWLRGYLENGKETLQRAERPKTHVTHHPVSDHEATLRCWALGFYPAEITLT

WQRDGEDQTQDTELVETRPAGDGTFQKWAAVVVPSGQEQRYTCHVQHEGLQEPCTLRWKP

SSQPTIPNLGIVSGPAVLAVLAVLAVLAVLGAVVAAVIHRRKSSGGKGGSCSQAASSNSA

QGSDESLIACKA

>sp|Q95604|1C17_HUMAN 54 DTQFVRFDSDAASPR

MRVMAPQALLLLLSGALALIETWAGSHSMRYFYTAVSRPGRGEPRFIAVGYVDDTQFVRF

DSDAASPRGEPRAPWVEQEGPEYWDRETQKYKRQAQADRVNLRKLRGYYNQSEAGSHTIQ

RMYGCDLGPDGRLLRGYNQFAYDGKDYIALNEDLRSWTAADTAAQISQRKLEAAREAEQL

RAYLEGECVEWLRGYLENGKETLQRAERPKTHVTHHPVSDHEATLRCWALGFYPAEITLT

WQRDGEDQTQDTELVETRPAGDGTFQKWAAVVVPSGQEQRYTCHVQHEGLQEPCTLRWKP

SSQPTIPNLGIVSGPAVLAVLAVLAVLAVLGAVVAAVIHRRKSSGGKGGSCSQAASSNSA

QGSDESLIACKA

>sp|Q95604|1C17_HUMAN 54 DTQFVRFDSDAASPRGE

MRVMAPQALLLLLSGALALIETWAGSHSMRYFYTAVSRPGRGEPRFIAVGYVDDTQFVRF

DSDAASPRGEPRAPWVEQEGPEYWDRETQKYKRQAQADRVNLRKLRGYYNQSEAGSHTIQ

RMYGCDLGPDGRLLRGYNQFAYDGKDYIALNEDLRSWTAADTAAQISQRKLEAAREAEQL

RAYLEGECVEWLRGYLENGKETLQRAERPKTHVTHHPVSDHEATLRCWALGFYPAEITLT

WQRDGEDQTQDTELVETRPAGDGTFQKWAAVVVPSGQEQRYTCHVQHEGLQEPCTLRWKP

SSQPTIPNLGIVSGPAVLAVLAVLAVLAVLGAVVAAVIHRRKSSGGKGGSCSQAASSNSA

QGSDESLIACKA

>sp|Q95604|1C17_HUMAN 54 DTQFVRFDSDAASPRGEP

MRVMAPQALLLLLSGALALIETWAGSHSMRYFYTAVSRPGRGEPRFIAVGYVDDTQFVRF

DSDAASPRGEPRAPWVEQEGPEYWDRETQKYKRQAQADRVNLRKLRGYYNQSEAGSHTIQ

RMYGCDLGPDGRLLRGYNQFAYDGKDYIALNEDLRSWTAADTAAQISQRKLEAAREAEQL

RAYLEGECVEWLRGYLENGKETLQRAERPKTHVTHHPVSDHEATLRCWALGFYPAEITLT

WQRDGEDQTQDTELVETRPAGDGTFQKWAAVVVPSGQEQRYTCHVQHEGLQEPCTLRWKP

SSQPTIPNLGIVSGPAVLAVLAVLAVLAVLGAVVAAVIHRRKSSGGKGGSCSQAASSNSA

QGSDESLIACKA

>sp|Q95604|1C17_HUMAN 54 DTQFVRFDSDAASPRGEPR

MRVMAPQALLLLLSGALALIETWAGSHSMRYFYTAVSRPGRGEPRFIAVGYVDDTQFVRF

DSDAASPRGEPRAPWVEQEGPEYWDRETQKYKRQAQADRVNLRKLRGYYNQSEAGSHTIQ

RMYGCDLGPDGRLLRGYNQFAYDGKDYIALNEDLRSWTAADTAAQISQRKLEAAREAEQL

RAYLEGECVEWLRGYLENGKETLQRAERPKTHVTHHPVSDHEATLRCWALGFYPAEITLT

WQRDGEDQTQDTELVETRPAGDGTFQKWAAVVVPSGQEQRYTCHVQHEGLQEPCTLRWKP

SSQPTIPNLGIVSGPAVLAVLAVLAVLAVLGAVVAAVIHRRKSSGGKGGSCSQAASSNSA

QGSDESLIACKA

>sp|Q95604|1C17_HUMAN 146 DYIALNEDLRSWT

MRVMAPQALLLLLSGALALIETWAGSHSMRYFYTAVSRPGRGEPRFIAVGYVDDTQFVRF

DSDAASPRGEPRAPWVEQEGPEYWDRETQKYKRQAQADRVNLRKLRGYYNQSEAGSHTIQ

RMYGCDLGPDGRLLRGYNQFAYDGKDYIALNEDLRSWTAADTAAQISQRKLEAAREAEQL

RAYLEGECVEWLRGYLENGKETLQRAERPKTHVTHHPVSDHEATLRCWALGFYPAEITLT

WQRDGEDQTQDTELVETRPAGDGTFQKWAAVVVPSGQEQRYTCHVQHEGLQEPCTLRWKP

SSQPTIPNLGIVSGPAVLAVLAVLAVLAVLGAVVAAVIHRRKSSGGKGGSCSQAASSNSA

QGSDESLIACKA

>sp|Q95604|1C17_HUMAN 146 DYIALNEDLRSWTAA

MRVMAPQALLLLLSGALALIETWAGSHSMRYFYTAVSRPGRGEPRFIAVGYVDDTQFVRF

DSDAASPRGEPRAPWVEQEGPEYWDRETQKYKRQAQADRVNLRKLRGYYNQSEAGSHTIQ

RMYGCDLGPDGRLLRGYNQFAYDGKDYIALNEDLRSWTAADTAAQISQRKLEAAREAEQL

RAYLEGECVEWLRGYLENGKETLQRAERPKTHVTHHPVSDHEATLRCWALGFYPAEITLT

WQRDGEDQTQDTELVETRPAGDGTFQKWAAVVVPSGQEQRYTCHVQHEGLQEPCTLRWKP

SSQPTIPNLGIVSGPAVLAVLAVLAVLAVLGAVVAAVIHRRKSSGGKGGSCSQAASSNSA

QGSDESLIACKA

>sp|Q95604|1C17_HUMAN 146 DYIALNEDLRSWTAAD

MRVMAPQALLLLLSGALALIETWAGSHSMRYFYTAVSRPGRGEPRFIAVGYVDDTQFVRF

DSDAASPRGEPRAPWVEQEGPEYWDRETQKYKRQAQADRVNLRKLRGYYNQSEAGSHTIQ

RMYGCDLGPDGRLLRGYNQFAYDGKDYIALNEDLRSWTAADTAAQISQRKLEAAREAEQL

RAYLEGECVEWLRGYLENGKETLQRAERPKTHVTHHPVSDHEATLRCWALGFYPAEITLT

WQRDGEDQTQDTELVETRPAGDGTFQKWAAVVVPSGQEQRYTCHVQHEGLQEPCTLRWKP

SSQPTIPNLGIVSGPAVLAVLAVLAVLAVLGAVVAAVIHRRKSSGGKGGSCSQAASSNSA

QGSDESLIACKA

>sp|Q95604|1C17_HUMAN 57 FVRFDSDAASP

MRVMAPQALLLLLSGALALIETWAGSHSMRYFYTAVSRPGRGEPRFIAVGYVDDTQFVRF

DSDAASPRGEPRAPWVEQEGPEYWDRETQKYKRQAQADRVNLRKLRGYYNQSEAGSHTIQ

RMYGCDLGPDGRLLRGYNQFAYDGKDYIALNEDLRSWTAADTAAQISQRKLEAAREAEQL

RAYLEGECVEWLRGYLENGKETLQRAERPKTHVTHHPVSDHEATLRCWALGFYPAEITLT

WQRDGEDQTQDTELVETRPAGDGTFQKWAAVVVPSGQEQRYTCHVQHEGLQEPCTLRWKP

SSQPTIPNLGIVSGPAVLAVLAVLAVLAVLGAVVAAVIHRRKSSGGKGGSCSQAASSNSA

QGSDESLIACKA

>sp|Q95604|1C17_HUMAN 57 FVRFDSDAASPR

MRVMAPQALLLLLSGALALIETWAGSHSMRYFYTAVSRPGRGEPRFIAVGYVDDTQFVRF

DSDAASPRGEPRAPWVEQEGPEYWDRETQKYKRQAQADRVNLRKLRGYYNQSEAGSHTIQ

RMYGCDLGPDGRLLRGYNQFAYDGKDYIALNEDLRSWTAADTAAQISQRKLEAAREAEQL

RAYLEGECVEWLRGYLENGKETLQRAERPKTHVTHHPVSDHEATLRCWALGFYPAEITLT

WQRDGEDQTQDTELVETRPAGDGTFQKWAAVVVPSGQEQRYTCHVQHEGLQEPCTLRWKP

SSQPTIPNLGIVSGPAVLAVLAVLAVLAVLGAVVAAVIHRRKSSGGKGGSCSQAASSNSA

QGSDESLIACKA

>sp|Q95604|1C17_HUMAN 80 GPEYWDRETQKYKRQA

MRVMAPQALLLLLSGALALIETWAGSHSMRYFYTAVSRPGRGEPRFIAVGYVDDTQFVRF

DSDAASPRGEPRAPWVEQEGPEYWDRETQKYKRQAQADRVNLRKLRGYYNQSEAGSHTIQ

RMYGCDLGPDGRLLRGYNQFAYDGKDYIALNEDLRSWTAADTAAQISQRKLEAAREAEQL

RAYLEGECVEWLRGYLENGKETLQRAERPKTHVTHHPVSDHEATLRCWALGFYPAEITLT

WQRDGEDQTQDTELVETRPAGDGTFQKWAAVVVPSGQEQRYTCHVQHEGLQEPCTLRWKP

SSQPTIPNLGIVSGPAVLAVLAVLAVLAVLGAVVAAVIHRRKSSGGKGGSCSQAASSNSA

QGSDESLIACKA

>sp|Q95604|1C17_HUMAN 131 GRLLRGYNQFAYDGK

MRVMAPQALLLLLSGALALIETWAGSHSMRYFYTAVSRPGRGEPRFIAVGYVDDTQFVRF

DSDAASPRGEPRAPWVEQEGPEYWDRETQKYKRQAQADRVNLRKLRGYYNQSEAGSHTIQ

RMYGCDLGPDGRLLRGYNQFAYDGKDYIALNEDLRSWTAADTAAQISQRKLEAAREAEQL

RAYLEGECVEWLRGYLENGKETLQRAERPKTHVTHHPVSDHEATLRCWALGFYPAEITLT

WQRDGEDQTQDTELVETRPAGDGTFQKWAAVVVPSGQEQRYTCHVQHEGLQEPCTLRWKP

SSQPTIPNLGIVSGPAVLAVLAVLAVLAVLGAVVAAVIHRRKSSGGKGGSCSQAASSNSA

QGSDESLIACKA

>sp|Q95604|1C17_HUMAN 25 GSHSMRYFYTAVSRPG

MRVMAPQALLLLLSGALALIETWAGSHSMRYFYTAVSRPGRGEPRFIAVGYVDDTQFVRF

DSDAASPRGEPRAPWVEQEGPEYWDRETQKYKRQAQADRVNLRKLRGYYNQSEAGSHTIQ

RMYGCDLGPDGRLLRGYNQFAYDGKDYIALNEDLRSWTAADTAAQISQRKLEAAREAEQL

RAYLEGECVEWLRGYLENGKETLQRAERPKTHVTHHPVSDHEATLRCWALGFYPAEITLT

WQRDGEDQTQDTELVETRPAGDGTFQKWAAVVVPSGQEQRYTCHVQHEGLQEPCTLRWKP

SSQPTIPNLGIVSGPAVLAVLAVLAVLAVLGAVVAAVIHRRKSSGGKGGSCSQAASSNSA

QGSDESLIACKA

>sp|Q95604|1C17_HUMAN 25 GSHSMRYFYTAVSRPGR

MRVMAPQALLLLLSGALALIETWAGSHSMRYFYTAVSRPGRGEPRFIAVGYVDDTQFVRF

DSDAASPRGEPRAPWVEQEGPEYWDRETQKYKRQAQADRVNLRKLRGYYNQSEAGSHTIQ

RMYGCDLGPDGRLLRGYNQFAYDGKDYIALNEDLRSWTAADTAAQISQRKLEAAREAEQL

RAYLEGECVEWLRGYLENGKETLQRAERPKTHVTHHPVSDHEATLRCWALGFYPAEITLT

WQRDGEDQTQDTELVETRPAGDGTFQKWAAVVVPSGQEQRYTCHVQHEGLQEPCTLRWKP

SSQPTIPNLGIVSGPAVLAVLAVLAVLAVLGAVVAAVIHRRKSSGGKGGSCSQAASSNSA

QGSDESLIACKA

>sp|Q95604|1C17_HUMAN 145 KDYIALNEDLRS

MRVMAPQALLLLLSGALALIETWAGSHSMRYFYTAVSRPGRGEPRFIAVGYVDDTQFVRF

DSDAASPRGEPRAPWVEQEGPEYWDRETQKYKRQAQADRVNLRKLRGYYNQSEAGSHTIQ

RMYGCDLGPDGRLLRGYNQFAYDGKDYIALNEDLRSWTAADTAAQISQRKLEAAREAEQL

RAYLEGECVEWLRGYLENGKETLQRAERPKTHVTHHPVSDHEATLRCWALGFYPAEITLT

WQRDGEDQTQDTELVETRPAGDGTFQKWAAVVVPSGQEQRYTCHVQHEGLQEPCTLRWKP

SSQPTIPNLGIVSGPAVLAVLAVLAVLAVLGAVVAAVIHRRKSSGGKGGSCSQAASSNSA

QGSDESLIACKA

>sp|Q95604|1C17_HUMAN 145 KDYIALNEDLRSWT

MRVMAPQALLLLLSGALALIETWAGSHSMRYFYTAVSRPGRGEPRFIAVGYVDDTQFVRF

DSDAASPRGEPRAPWVEQEGPEYWDRETQKYKRQAQADRVNLRKLRGYYNQSEAGSHTIQ

RMYGCDLGPDGRLLRGYNQFAYDGKDYIALNEDLRSWTAADTAAQISQRKLEAAREAEQL

RAYLEGECVEWLRGYLENGKETLQRAERPKTHVTHHPVSDHEATLRCWALGFYPAEITLT

WQRDGEDQTQDTELVETRPAGDGTFQKWAAVVVPSGQEQRYTCHVQHEGLQEPCTLRWKP

SSQPTIPNLGIVSGPAVLAVLAVLAVLAVLGAVVAAVIHRRKSSGGKGGSCSQAASSNSA

QGSDESLIACKA

>sp|Q95604|1C17_HUMAN 145 KDYIALNEDLRSWTA

MRVMAPQALLLLLSGALALIETWAGSHSMRYFYTAVSRPGRGEPRFIAVGYVDDTQFVRF

DSDAASPRGEPRAPWVEQEGPEYWDRETQKYKRQAQADRVNLRKLRGYYNQSEAGSHTIQ

RMYGCDLGPDGRLLRGYNQFAYDGKDYIALNEDLRSWTAADTAAQISQRKLEAAREAEQL

RAYLEGECVEWLRGYLENGKETLQRAERPKTHVTHHPVSDHEATLRCWALGFYPAEITLT

WQRDGEDQTQDTELVETRPAGDGTFQKWAAVVVPSGQEQRYTCHVQHEGLQEPCTLRWKP

SSQPTIPNLGIVSGPAVLAVLAVLAVLAVLGAVVAAVIHRRKSSGGKGGSCSQAASSNSA

QGSDESLIACKA

>sp|Q95604|1C17_HUMAN 145 KDYIALNEDLRSWTAADT

MRVMAPQALLLLLSGALALIETWAGSHSMRYFYTAVSRPGRGEPRFIAVGYVDDTQFVRF

DSDAASPRGEPRAPWVEQEGPEYWDRETQKYKRQAQADRVNLRKLRGYYNQSEAGSHTIQ

RMYGCDLGPDGRLLRGYNQFAYDGKDYIALNEDLRSWTAADTAAQISQRKLEAAREAEQL

RAYLEGECVEWLRGYLENGKETLQRAERPKTHVTHHPVSDHEATLRCWALGFYPAEITLT

WQRDGEDQTQDTELVETRPAGDGTFQKWAAVVVPSGQEQRYTCHVQHEGLQEPCTLRWKP

SSQPTIPNLGIVSGPAVLAVLAVLAVLAVLGAVVAAVIHRRKSSGGKGGSCSQAASSNSA

QGSDESLIACKA

>sp|Q95604|1C17_HUMAN 150 LNEDLRSWTAAD

MRVMAPQALLLLLSGALALIETWAGSHSMRYFYTAVSRPGRGEPRFIAVGYVDDTQFVRF

DSDAASPRGEPRAPWVEQEGPEYWDRETQKYKRQAQADRVNLRKLRGYYNQSEAGSHTIQ

RMYGCDLGPDGRLLRGYNQFAYDGKDYIALNEDLRSWTAADTAAQISQRKLEAAREAEQL

RAYLEGECVEWLRGYLENGKETLQRAERPKTHVTHHPVSDHEATLRCWALGFYPAEITLT

WQRDGEDQTQDTELVETRPAGDGTFQKWAAVVVPSGQEQRYTCHVQHEGLQEPCTLRWKP

SSQPTIPNLGIVSGPAVLAVLAVLAVLAVLGAVVAAVIHRRKSSGGKGGSCSQAASSNSA

QGSDESLIACKA

>sp|Q95604|1C17_HUMAN 56 QFVRFDSDAASPR

MRVMAPQALLLLLSGALALIETWAGSHSMRYFYTAVSRPGRGEPRFIAVGYVDDTQFVRF

DSDAASPRGEPRAPWVEQEGPEYWDRETQKYKRQAQADRVNLRKLRGYYNQSEAGSHTIQ

RMYGCDLGPDGRLLRGYNQFAYDGKDYIALNEDLRSWTAADTAAQISQRKLEAAREAEQL

RAYLEGECVEWLRGYLENGKETLQRAERPKTHVTHHPVSDHEATLRCWALGFYPAEITLT

WQRDGEDQTQDTELVETRPAGDGTFQKWAAVVVPSGQEQRYTCHVQHEGLQEPCTLRWKP

SSQPTIPNLGIVSGPAVLAVLAVLAVLAVLGAVVAAVIHRRKSSGGKGGSCSQAASSNSA

QGSDESLIACKA

>sp|Q95604|1C17_HUMAN 26 SHSMRYFYTAVSRP

MRVMAPQALLLLLSGALALIETWAGSHSMRYFYTAVSRPGRGEPRFIAVGYVDDTQFVRF

DSDAASPRGEPRAPWVEQEGPEYWDRETQKYKRQAQADRVNLRKLRGYYNQSEAGSHTIQ

RMYGCDLGPDGRLLRGYNQFAYDGKDYIALNEDLRSWTAADTAAQISQRKLEAAREAEQL

RAYLEGECVEWLRGYLENGKETLQRAERPKTHVTHHPVSDHEATLRCWALGFYPAEITLT

WQRDGEDQTQDTELVETRPAGDGTFQKWAAVVVPSGQEQRYTCHVQHEGLQEPCTLRWKP

SSQPTIPNLGIVSGPAVLAVLAVLAVLAVLGAVVAAVIHRRKSSGGKGGSCSQAASSNSA

QGSDESLIACKA

>sp|Q95604|1C17_HUMAN 26 SHSMRYFYTAVSRPG

MRVMAPQALLLLLSGALALIETWAGSHSMRYFYTAVSRPGRGEPRFIAVGYVDDTQFVRF

DSDAASPRGEPRAPWVEQEGPEYWDRETQKYKRQAQADRVNLRKLRGYYNQSEAGSHTIQ

RMYGCDLGPDGRLLRGYNQFAYDGKDYIALNEDLRSWTAADTAAQISQRKLEAAREAEQL

RAYLEGECVEWLRGYLENGKETLQRAERPKTHVTHHPVSDHEATLRCWALGFYPAEITLT

WQRDGEDQTQDTELVETRPAGDGTFQKWAAVVVPSGQEQRYTCHVQHEGLQEPCTLRWKP

SSQPTIPNLGIVSGPAVLAVLAVLAVLAVLGAVVAAVIHRRKSSGGKGGSCSQAASSNSA

QGSDESLIACKA

>sp|Q95604|1C17_HUMAN 26 SHSMRYFYTAVSRPGRG

MRVMAPQALLLLLSGALALIETWAGSHSMRYFYTAVSRPGRGEPRFIAVGYVDDTQFVRF

DSDAASPRGEPRAPWVEQEGPEYWDRETQKYKRQAQADRVNLRKLRGYYNQSEAGSHTIQ

RMYGCDLGPDGRLLRGYNQFAYDGKDYIALNEDLRSWTAADTAAQISQRKLEAAREAEQL

RAYLEGECVEWLRGYLENGKETLQRAERPKTHVTHHPVSDHEATLRCWALGFYPAEITLT

WQRDGEDQTQDTELVETRPAGDGTFQKWAAVVVPSGQEQRYTCHVQHEGLQEPCTLRWKP

SSQPTIPNLGIVSGPAVLAVLAVLAVLAVLGAVVAAVIHRRKSSGGKGGSCSQAASSNSA

QGSDESLIACKA

>sp|Q95604|1C17_HUMAN 55 TQFVRFDSDAASPR

MRVMAPQALLLLLSGALALIETWAGSHSMRYFYTAVSRPGRGEPRFIAVGYVDDTQFVRF

DSDAASPRGEPRAPWVEQEGPEYWDRETQKYKRQAQADRVNLRKLRGYYNQSEAGSHTIQ

RMYGCDLGPDGRLLRGYNQFAYDGKDYIALNEDLRSWTAADTAAQISQRKLEAAREAEQL

RAYLEGECVEWLRGYLENGKETLQRAERPKTHVTHHPVSDHEATLRCWALGFYPAEITLT

WQRDGEDQTQDTELVETRPAGDGTFQKWAAVVVPSGQEQRYTCHVQHEGLQEPCTLRWKP

SSQPTIPNLGIVSGPAVLAVLAVLAVLAVLGAVVAAVIHRRKSSGGKGGSCSQAASSNSA

QGSDESLIACKA

>sp|Q95604|1C17_HUMAN 52 VDDTQFVRFDSDAAS

MRVMAPQALLLLLSGALALIETWAGSHSMRYFYTAVSRPGRGEPRFIAVGYVDDTQFVRF

DSDAASPRGEPRAPWVEQEGPEYWDRETQKYKRQAQADRVNLRKLRGYYNQSEAGSHTIQ

RMYGCDLGPDGRLLRGYNQFAYDGKDYIALNEDLRSWTAADTAAQISQRKLEAAREAEQL

RAYLEGECVEWLRGYLENGKETLQRAERPKTHVTHHPVSDHEATLRCWALGFYPAEITLT

WQRDGEDQTQDTELVETRPAGDGTFQKWAAVVVPSGQEQRYTCHVQHEGLQEPCTLRWKP

SSQPTIPNLGIVSGPAVLAVLAVLAVLAVLGAVVAAVIHRRKSSGGKGGSCSQAASSNSA

QGSDESLIACKA

>sp|Q95604|1C17_HUMAN 52 VDDTQFVRFDSDAASP

MRVMAPQALLLLLSGALALIETWAGSHSMRYFYTAVSRPGRGEPRFIAVGYVDDTQFVRF

DSDAASPRGEPRAPWVEQEGPEYWDRETQKYKRQAQADRVNLRKLRGYYNQSEAGSHTIQ

RMYGCDLGPDGRLLRGYNQFAYDGKDYIALNEDLRSWTAADTAAQISQRKLEAAREAEQL

RAYLEGECVEWLRGYLENGKETLQRAERPKTHVTHHPVSDHEATLRCWALGFYPAEITLT

WQRDGEDQTQDTELVETRPAGDGTFQKWAAVVVPSGQEQRYTCHVQHEGLQEPCTLRWKP

SSQPTIPNLGIVSGPAVLAVLAVLAVLAVLGAVVAAVIHRRKSSGGKGGSCSQAASSNSA

QGSDESLIACKA

>sp|Q95604|1C17_HUMAN 52 VDDTQFVRFDSDAASPR

MRVMAPQALLLLLSGALALIETWAGSHSMRYFYTAVSRPGRGEPRFIAVGYVDDTQFVRF

DSDAASPRGEPRAPWVEQEGPEYWDRETQKYKRQAQADRVNLRKLRGYYNQSEAGSHTIQ

RMYGCDLGPDGRLLRGYNQFAYDGKDYIALNEDLRSWTAADTAAQISQRKLEAAREAEQL

RAYLEGECVEWLRGYLENGKETLQRAERPKTHVTHHPVSDHEATLRCWALGFYPAEITLT

WQRDGEDQTQDTELVETRPAGDGTFQKWAAVVVPSGQEQRYTCHVQHEGLQEPCTLRWKP

SSQPTIPNLGIVSGPAVLAVLAVLAVLAVLGAVVAAVIHRRKSSGGKGGSCSQAASSNSA

QGSDESLIACKA

>sp|Q95604|1C17_HUMAN 52 VDDTQFVRFDSDAASPRGEP

MRVMAPQALLLLLSGALALIETWAGSHSMRYFYTAVSRPGRGEPRFIAVGYVDDTQFVRF

DSDAASPRGEPRAPWVEQEGPEYWDRETQKYKRQAQADRVNLRKLRGYYNQSEAGSHTIQ

RMYGCDLGPDGRLLRGYNQFAYDGKDYIALNEDLRSWTAADTAAQISQRKLEAAREAEQL

RAYLEGECVEWLRGYLENGKETLQRAERPKTHVTHHPVSDHEATLRCWALGFYPAEITLT

WQRDGEDQTQDTELVETRPAGDGTFQKWAAVVVPSGQEQRYTCHVQHEGLQEPCTLRWKP

SSQPTIPNLGIVSGPAVLAVLAVLAVLAVLGAVVAAVIHRRKSSGGKGGSCSQAASSNSA

QGSDESLIACKA

>sp|Q95604|1C17_HUMAN 52 VDDTQFVRFDSDAASPRGEPR

MRVMAPQALLLLLSGALALIETWAGSHSMRYFYTAVSRPGRGEPRFIAVGYVDDTQFVRF

DSDAASPRGEPRAPWVEQEGPEYWDRETQKYKRQAQADRVNLRKLRGYYNQSEAGSHTIQ

RMYGCDLGPDGRLLRGYNQFAYDGKDYIALNEDLRSWTAADTAAQISQRKLEAAREAEQL

RAYLEGECVEWLRGYLENGKETLQRAERPKTHVTHHPVSDHEATLRCWALGFYPAEITLT

WQRDGEDQTQDTELVETRPAGDGTFQKWAAVVVPSGQEQRYTCHVQHEGLQEPCTLRWKP

SSQPTIPNLGIVSGPAVLAVLAVLAVLAVLGAVVAAVIHRRKSSGGKGGSCSQAASSNSA

QGSDESLIACKA

>sp|Q95604|1C17_HUMAN 52 VDDTQFVRFDSDAASPRGEPRAP

MRVMAPQALLLLLSGALALIETWAGSHSMRYFYTAVSRPGRGEPRFIAVGYVDDTQFVRF

DSDAASPRGEPRAPWVEQEGPEYWDRETQKYKRQAQADRVNLRKLRGYYNQSEAGSHTIQ

RMYGCDLGPDGRLLRGYNQFAYDGKDYIALNEDLRSWTAADTAAQISQRKLEAAREAEQL

RAYLEGECVEWLRGYLENGKETLQRAERPKTHVTHHPVSDHEATLRCWALGFYPAEITLT

WQRDGEDQTQDTELVETRPAGDGTFQKWAAVVVPSGQEQRYTCHVQHEGLQEPCTLRWKP

SSQPTIPNLGIVSGPAVLAVLAVLAVLAVLGAVVAAVIHRRKSSGGKGGSCSQAASSNSA

QGSDESLIACKA

>sp|Q95604|1C17_HUMAN 58 VRFDSDAASPR

MRVMAPQALLLLLSGALALIETWAGSHSMRYFYTAVSRPGRGEPRFIAVGYVDDTQFVRF

DSDAASPRGEPRAPWVEQEGPEYWDRETQKYKRQAQADRVNLRKLRGYYNQSEAGSHTIQ

RMYGCDLGPDGRLLRGYNQFAYDGKDYIALNEDLRSWTAADTAAQISQRKLEAAREAEQL

RAYLEGECVEWLRGYLENGKETLQRAERPKTHVTHHPVSDHEATLRCWALGFYPAEITLT

WQRDGEDQTQDTELVETRPAGDGTFQKWAAVVVPSGQEQRYTCHVQHEGLQEPCTLRWKP

SSQPTIPNLGIVSGPAVLAVLAVLAVLAVLGAVVAAVIHRRKSSGGKGGSCSQAASSNSA

QGSDESLIACKA

>sp|Q95604|1C17_HUMAN 51 YVDDTQFVRFDSDAASPR

MRVMAPQALLLLLSGALALIETWAGSHSMRYFYTAVSRPGRGEPRFIAVGYVDDTQFVRF

DSDAASPRGEPRAPWVEQEGPEYWDRETQKYKRQAQADRVNLRKLRGYYNQSEAGSHTIQ

RMYGCDLGPDGRLLRGYNQFAYDGKDYIALNEDLRSWTAADTAAQISQRKLEAAREAEQL

RAYLEGECVEWLRGYLENGKETLQRAERPKTHVTHHPVSDHEATLRCWALGFYPAEITLT

WQRDGEDQTQDTELVETRPAGDGTFQKWAAVVVPSGQEQRYTCHVQHEGLQEPCTLRWKP

SSQPTIPNLGIVSGPAVLAVLAVLAVLAVLGAVVAAVIHRRKSSGGKGGSCSQAASSNSA

QGSDESLIACKA

>sp|Q29865|1C18_HUMAN 153 DLRSWTAADTAAQITQ

MRVMAPRALLLLLSGGLALTETWACSHSMRYFDTAVSRPGRGEPRFISVGYVDDTQFVRF

DSDAASPRGEPRAPWVEQEGPEYWDRETQKYKRQAQADRVNLRKLRGYYNQSEDGSHTLQ

RMFGCDLGPDGRLLRGYNQFAYDGKDYIALNEDLRSWTAADTAAQITQRKWEAAREAEQR

RAYLEGTCVEWLRRYLENGKETLQRAEHPKTHVTHHPVSDHEATLRCWALGFYPAEITLT

WQWDGEDQTQDTELVETRPAGDGTFQKWAAVVVPSGEEQRYTCHVQHEGLPEPLTLRWKP

SSQPTIPIVGIVAGLAVLVVLAVLGAVVAVVMCRRKSSGGKGGSCSQAASSNSAQGSDES

LIACKA

>sp|Q29865|1C18_HUMAN 153 DLRSWTAADTAAQITQR

MRVMAPRALLLLLSGGLALTETWACSHSMRYFDTAVSRPGRGEPRFISVGYVDDTQFVRF

DSDAASPRGEPRAPWVEQEGPEYWDRETQKYKRQAQADRVNLRKLRGYYNQSEDGSHTLQ

RMFGCDLGPDGRLLRGYNQFAYDGKDYIALNEDLRSWTAADTAAQITQRKWEAAREAEQR

RAYLEGTCVEWLRRYLENGKETLQRAEHPKTHVTHHPVSDHEATLRCWALGFYPAEITLT

WQWDGEDQTQDTELVETRPAGDGTFQKWAAVVVPSGEEQRYTCHVQHEGLPEPLTLRWKP

SSQPTIPIVGIVAGLAVLVVLAVLGAVVAVVMCRRKSSGGKGGSCSQAASSNSAQGSDES

LIACKA

>sp|Q29865|1C18_HUMAN 152 EDLRSWTAADTAAQITQ

MRVMAPRALLLLLSGGLALTETWACSHSMRYFDTAVSRPGRGEPRFISVGYVDDTQFVRF

DSDAASPRGEPRAPWVEQEGPEYWDRETQKYKRQAQADRVNLRKLRGYYNQSEDGSHTLQ

RMFGCDLGPDGRLLRGYNQFAYDGKDYIALNEDLRSWTAADTAAQITQRKWEAAREAEQR

RAYLEGTCVEWLRRYLENGKETLQRAEHPKTHVTHHPVSDHEATLRCWALGFYPAEITLT

WQWDGEDQTQDTELVETRPAGDGTFQKWAAVVVPSGEEQRYTCHVQHEGLPEPLTLRWKP

SSQPTIPIVGIVAGLAVLVVLAVLGAVVAVVMCRRKSSGGKGGSCSQAASSNSAQGSDES

LIACKA

>sp|Q29865|1C18_HUMAN 152 EDLRSWTAADTAAQITQR

MRVMAPRALLLLLSGGLALTETWACSHSMRYFDTAVSRPGRGEPRFISVGYVDDTQFVRF

DSDAASPRGEPRAPWVEQEGPEYWDRETQKYKRQAQADRVNLRKLRGYYNQSEDGSHTLQ

RMFGCDLGPDGRLLRGYNQFAYDGKDYIALNEDLRSWTAADTAAQITQRKWEAAREAEQR

RAYLEGTCVEWLRRYLENGKETLQRAEHPKTHVTHHPVSDHEATLRCWALGFYPAEITLT

WQWDGEDQTQDTELVETRPAGDGTFQKWAAVVVPSGEEQRYTCHVQHEGLPEPLTLRWKP

SSQPTIPIVGIVAGLAVLVVLAVLGAVVAVVMCRRKSSGGKGGSCSQAASSNSAQGSDES

LIACKA

>sp|Q29865|1C18_HUMAN 150 LNEDLRSWTAADTAAQITQR

MRVMAPRALLLLLSGGLALTETWACSHSMRYFDTAVSRPGRGEPRFISVGYVDDTQFVRF

DSDAASPRGEPRAPWVEQEGPEYWDRETQKYKRQAQADRVNLRKLRGYYNQSEDGSHTLQ

RMFGCDLGPDGRLLRGYNQFAYDGKDYIALNEDLRSWTAADTAAQITQRKWEAAREAEQR

RAYLEGTCVEWLRRYLENGKETLQRAEHPKTHVTHHPVSDHEATLRCWALGFYPAEITLT

WQWDGEDQTQDTELVETRPAGDGTFQKWAAVVVPSGEEQRYTCHVQHEGLPEPLTLRWKP

SSQPTIPIVGIVAGLAVLVVLAVLGAVVAVVMCRRKSSGGKGGSCSQAASSNSAQGSDES

LIACKA

>sp|Q29865|1C18_HUMAN 154 LRSWTAADTAAQITQRKWEAA

MRVMAPRALLLLLSGGLALTETWACSHSMRYFDTAVSRPGRGEPRFISVGYVDDTQFVRF

DSDAASPRGEPRAPWVEQEGPEYWDRETQKYKRQAQADRVNLRKLRGYYNQSEDGSHTLQ

RMFGCDLGPDGRLLRGYNQFAYDGKDYIALNEDLRSWTAADTAAQITQRKWEAAREAEQR

RAYLEGTCVEWLRRYLENGKETLQRAEHPKTHVTHHPVSDHEATLRCWALGFYPAEITLT

WQWDGEDQTQDTELVETRPAGDGTFQKWAAVVVPSGEEQRYTCHVQHEGLPEPLTLRWKP

SSQPTIPIVGIVAGLAVLVVLAVLGAVVAVVMCRRKSSGGKGGSCSQAASSNSAQGSDES

LIACKA

>sp|Q29865|1C18_HUMAN 151 NEDLRSWTAADTAAQITQ

MRVMAPRALLLLLSGGLALTETWACSHSMRYFDTAVSRPGRGEPRFISVGYVDDTQFVRF

DSDAASPRGEPRAPWVEQEGPEYWDRETQKYKRQAQADRVNLRKLRGYYNQSEDGSHTLQ

RMFGCDLGPDGRLLRGYNQFAYDGKDYIALNEDLRSWTAADTAAQITQRKWEAAREAEQR

RAYLEGTCVEWLRRYLENGKETLQRAEHPKTHVTHHPVSDHEATLRCWALGFYPAEITLT

WQWDGEDQTQDTELVETRPAGDGTFQKWAAVVVPSGEEQRYTCHVQHEGLPEPLTLRWKP

SSQPTIPIVGIVAGLAVLVVLAVLGAVVAVVMCRRKSSGGKGGSCSQAASSNSAQGSDES

LIACKA

>sp|Q29865|1C18_HUMAN 158 TAADTAAQITQR

MRVMAPRALLLLLSGGLALTETWACSHSMRYFDTAVSRPGRGEPRFISVGYVDDTQFVRF

DSDAASPRGEPRAPWVEQEGPEYWDRETQKYKRQAQADRVNLRKLRGYYNQSEDGSHTLQ

RMFGCDLGPDGRLLRGYNQFAYDGKDYIALNEDLRSWTAADTAAQITQRKWEAAREAEQR

RAYLEGTCVEWLRRYLENGKETLQRAEHPKTHVTHHPVSDHEATLRCWALGFYPAEITLT

WQWDGEDQTQDTELVETRPAGDGTFQKWAAVVVPSGEEQRYTCHVQHEGLPEPLTLRWKP

SSQPTIPIVGIVAGLAVLVVLAVLGAVVAVVMCRRKSSGGKGGSCSQAASSNSAQGSDES

LIACKA

>sp|P30505|1C08_HUMAN 57 FVQFDSDAASPRGEP

MRVMAPRTLILLLSGALALTETWACSHSMRYFYTAVSRPGRGEPRFIAVGYVDDTQFVQF

DSDAASPRGEPRAPWVEQEGPEYWDRETQKYKRQAQTDRVSLRNLRGYYNQSEAGSHTLQ

RMYGCDLGPDGRLLRGYNQFAYDGKDYIALNEDLRSWTAADTAAQITQRKWEAARTAEQL

RAYLEGTCVEWLRRYLENGKKTLQRAEHPKTHVTHHPVSDHEATLRCWALGFYPAEITLT

WQRDGEDQTQDTELVETRPAGDGTFQKWAAVVVPSGEEQRYTCHVQHEGLPEPLTLRWGP

SSQPTIPIVGIVAGLAVLAVLAVLGAVMAVVMCRRKSSGGKGGSCSQAASSNSAQGSDES

LIACKA

>sp|P30505|1C08_HUMAN 52 VDDTQFVQFDSDAASPRGEPR

MRVMAPRTLILLLSGALALTETWACSHSMRYFYTAVSRPGRGEPRFIAVGYVDDTQFVQF

DSDAASPRGEPRAPWVEQEGPEYWDRETQKYKRQAQTDRVSLRNLRGYYNQSEAGSHTLQ

RMYGCDLGPDGRLLRGYNQFAYDGKDYIALNEDLRSWTAADTAAQITQRKWEAARTAEQL

RAYLEGTCVEWLRRYLENGKKTLQRAEHPKTHVTHHPVSDHEATLRCWALGFYPAEITLT

WQRDGEDQTQDTELVETRPAGDGTFQKWAAVVVPSGEEQRYTCHVQHEGLPEPLTLRWGP

SSQPTIPIVGIVAGLAVLAVLAVLGAVMAVVMCRRKSSGGKGGSCSQAASSNSAQGSDES

LIACKA

>sp|P30505|1C08_HUMAN 51 YVDDTQFVQFDSDAASPRGEPRAP

MRVMAPRTLILLLSGALALTETWACSHSMRYFYTAVSRPGRGEPRFIAVGYVDDTQFVQF

DSDAASPRGEPRAPWVEQEGPEYWDRETQKYKRQAQTDRVSLRNLRGYYNQSEAGSHTLQ

RMYGCDLGPDGRLLRGYNQFAYDGKDYIALNEDLRSWTAADTAAQITQRKWEAARTAEQL

RAYLEGTCVEWLRRYLENGKKTLQRAEHPKTHVTHHPVSDHEATLRCWALGFYPAEITLT

WQRDGEDQTQDTELVETRPAGDGTFQKWAAVVVPSGEEQRYTCHVQHEGLPEPLTLRWGP

SSQPTIPIVGIVAGLAVLAVLAVLGAVMAVVMCRRKSSGGKGGSCSQAASSNSAQGSDES

LIACKA

>sp|Q09160|1A80_HUMAN 158 TAADMAAQITKRKWEA

MAVMPPRTLLLLLSGALALTQTWAGSHSMRYFFTSVSRPGRGEPRFIAVGYVDDSQFVQF

DSDAASQRMEPRAPWIEQEEPEYWDEETRNVKAHSQTNRANLGTLRGYYNQSEDGSHTIQ

IMYGCDVGSDGRFLRGYRQDAYDGKDYIALNEDLRSWTAADMAAQITKRKWEAARRAEQL

RAYLEGECVDGLRRYLENGKETLQRTDPPKTHMTHHPISDHEATLRCWALSFYPAEITLT

WQRDGEDQTQDTELVETRPAGDGTFQKWAAVVVPSGKEKRYTCHVQHEGLPEPLTLRWEP

SSQPTIPIVGIIAGLVLLGAVIAGAVVAAVMWRKKSSVRKGGSYSQAASSDSAQGSDVSL

TACKV

>sp|P30459|1A74_HUMAN 323 AGAVVAAVRWRRKSSDRK

MAVMAPRTLLLLLLGALALTQTRAGSHSMRYFFTSVSRPGRGEPRFIAVGYVDDTQFVRF

DSDAASQRMEPRAPWIEQEGPEYWDQETRNVKAHSQTDRVDLGTLRGYYNQSEAGSHTIQ

MMYGCDVGPDGRLLRGYQQDAYDGKDYIALNEDLRSWTAADMAAQITQRKWEAARVAEQL

RAYLEGTCVEWLRRYLENGKETLQRTDAPKTHMTHHAVSDHEATLRCWALSFYPAEITLT

WQRDGEDQTQDTELVETRPAGDGTFQKWASVVVPSGQEQRYTCHVQHEGLPKPLTLRWEP

SSQPTIPIVGIIAGLVLFGAMFAGAVVAAVRWRRKSSDRKGGSYSQAASSDSAQGSDMSL

TACKV

>sp|P30459|1A74_HUMAN 53 DDTQFVRFDSDAASQ

MAVMAPRTLLLLLLGALALTQTRAGSHSMRYFFTSVSRPGRGEPRFIAVGYVDDTQFVRF

DSDAASQRMEPRAPWIEQEGPEYWDQETRNVKAHSQTDRVDLGTLRGYYNQSEAGSHTIQ

MMYGCDVGPDGRLLRGYQQDAYDGKDYIALNEDLRSWTAADMAAQITQRKWEAARVAEQL

RAYLEGTCVEWLRRYLENGKETLQRTDAPKTHMTHHAVSDHEATLRCWALSFYPAEITLT

WQRDGEDQTQDTELVETRPAGDGTFQKWASVVVPSGQEQRYTCHVQHEGLPKPLTLRWEP

SSQPTIPIVGIIAGLVLFGAMFAGAVVAAVRWRRKSSDRKGGSYSQAASSDSAQGSDMSL

TACKV

>sp|P30459|1A74_HUMAN 53 DDTQFVRFDSDAASQR

MAVMAPRTLLLLLLGALALTQTRAGSHSMRYFFTSVSRPGRGEPRFIAVGYVDDTQFVRF

DSDAASQRMEPRAPWIEQEGPEYWDQETRNVKAHSQTDRVDLGTLRGYYNQSEAGSHTIQ

MMYGCDVGPDGRLLRGYQQDAYDGKDYIALNEDLRSWTAADMAAQITQRKWEAARVAEQL

RAYLEGTCVEWLRRYLENGKETLQRTDAPKTHMTHHAVSDHEATLRCWALSFYPAEITLT

WQRDGEDQTQDTELVETRPAGDGTFQKWASVVVPSGQEQRYTCHVQHEGLPKPLTLRWEP

SSQPTIPIVGIIAGLVLFGAMFAGAVVAAVRWRRKSSDRKGGSYSQAASSDSAQGSDMSL

TACKV

>sp|P30459|1A74_HUMAN 53 DDTQFVRFDSDAASQRM

MAVMAPRTLLLLLLGALALTQTRAGSHSMRYFFTSVSRPGRGEPRFIAVGYVDDTQFVRF

DSDAASQRMEPRAPWIEQEGPEYWDQETRNVKAHSQTDRVDLGTLRGYYNQSEAGSHTIQ

MMYGCDVGPDGRLLRGYQQDAYDGKDYIALNEDLRSWTAADMAAQITQRKWEAARVAEQL

RAYLEGTCVEWLRRYLENGKETLQRTDAPKTHMTHHAVSDHEATLRCWALSFYPAEITLT

WQRDGEDQTQDTELVETRPAGDGTFQKWASVVVPSGQEQRYTCHVQHEGLPKPLTLRWEP

SSQPTIPIVGIIAGLVLFGAMFAGAVVAAVRWRRKSSDRKGGSYSQAASSDSAQGSDMSL

TACKV

>sp|P30459|1A74_HUMAN 53 DDTQFVRFDSDAASQRME

MAVMAPRTLLLLLLGALALTQTRAGSHSMRYFFTSVSRPGRGEPRFIAVGYVDDTQFVRF

DSDAASQRMEPRAPWIEQEGPEYWDQETRNVKAHSQTDRVDLGTLRGYYNQSEAGSHTIQ

MMYGCDVGPDGRLLRGYQQDAYDGKDYIALNEDLRSWTAADMAAQITQRKWEAARVAEQL

RAYLEGTCVEWLRRYLENGKETLQRTDAPKTHMTHHAVSDHEATLRCWALSFYPAEITLT

WQRDGEDQTQDTELVETRPAGDGTFQKWASVVVPSGQEQRYTCHVQHEGLPKPLTLRWEP

SSQPTIPIVGIIAGLVLFGAMFAGAVVAAVRWRRKSSDRKGGSYSQAASSDSAQGSDMSL

TACKV

>sp|P30459|1A74_HUMAN 53 DDTQFVRFDSDAASQRMEP

MAVMAPRTLLLLLLGALALTQTRAGSHSMRYFFTSVSRPGRGEPRFIAVGYVDDTQFVRF

DSDAASQRMEPRAPWIEQEGPEYWDQETRNVKAHSQTDRVDLGTLRGYYNQSEAGSHTIQ

MMYGCDVGPDGRLLRGYQQDAYDGKDYIALNEDLRSWTAADMAAQITQRKWEAARVAEQL

RAYLEGTCVEWLRRYLENGKETLQRTDAPKTHMTHHAVSDHEATLRCWALSFYPAEITLT

WQRDGEDQTQDTELVETRPAGDGTFQKWASVVVPSGQEQRYTCHVQHEGLPKPLTLRWEP

SSQPTIPIVGIIAGLVLFGAMFAGAVVAAVRWRRKSSDRKGGSYSQAASSDSAQGSDMSL

TACKV

>sp|P30459|1A74_HUMAN 53 DDTQFVRFDSDAASQRMEPR

MAVMAPRTLLLLLLGALALTQTRAGSHSMRYFFTSVSRPGRGEPRFIAVGYVDDTQFVRF

DSDAASQRMEPRAPWIEQEGPEYWDQETRNVKAHSQTDRVDLGTLRGYYNQSEAGSHTIQ

MMYGCDVGPDGRLLRGYQQDAYDGKDYIALNEDLRSWTAADMAAQITQRKWEAARVAEQL

RAYLEGTCVEWLRRYLENGKETLQRTDAPKTHMTHHAVSDHEATLRCWALSFYPAEITLT

WQRDGEDQTQDTELVETRPAGDGTFQKWASVVVPSGQEQRYTCHVQHEGLPKPLTLRWEP

SSQPTIPIVGIIAGLVLFGAMFAGAVVAAVRWRRKSSDRKGGSYSQAASSDSAQGSDMSL

TACKV

>sp|P30459|1A74_HUMAN 54 DTQFVRFDSDAASQ

MAVMAPRTLLLLLLGALALTQTRAGSHSMRYFFTSVSRPGRGEPRFIAVGYVDDTQFVRF

DSDAASQRMEPRAPWIEQEGPEYWDQETRNVKAHSQTDRVDLGTLRGYYNQSEAGSHTIQ

MMYGCDVGPDGRLLRGYQQDAYDGKDYIALNEDLRSWTAADMAAQITQRKWEAARVAEQL

RAYLEGTCVEWLRRYLENGKETLQRTDAPKTHMTHHAVSDHEATLRCWALSFYPAEITLT

WQRDGEDQTQDTELVETRPAGDGTFQKWASVVVPSGQEQRYTCHVQHEGLPKPLTLRWEP

SSQPTIPIVGIIAGLVLFGAMFAGAVVAAVRWRRKSSDRKGGSYSQAASSDSAQGSDMSL

TACKV

>sp|P30459|1A74_HUMAN 54 DTQFVRFDSDAASQR

MAVMAPRTLLLLLLGALALTQTRAGSHSMRYFFTSVSRPGRGEPRFIAVGYVDDTQFVRF

DSDAASQRMEPRAPWIEQEGPEYWDQETRNVKAHSQTDRVDLGTLRGYYNQSEAGSHTIQ

MMYGCDVGPDGRLLRGYQQDAYDGKDYIALNEDLRSWTAADMAAQITQRKWEAARVAEQL

RAYLEGTCVEWLRRYLENGKETLQRTDAPKTHMTHHAVSDHEATLRCWALSFYPAEITLT

WQRDGEDQTQDTELVETRPAGDGTFQKWASVVVPSGQEQRYTCHVQHEGLPKPLTLRWEP

SSQPTIPIVGIIAGLVLFGAMFAGAVVAAVRWRRKSSDRKGGSYSQAASSDSAQGSDMSL

TACKV

>sp|P30459|1A74_HUMAN 54 DTQFVRFDSDAASQRM

MAVMAPRTLLLLLLGALALTQTRAGSHSMRYFFTSVSRPGRGEPRFIAVGYVDDTQFVRF

DSDAASQRMEPRAPWIEQEGPEYWDQETRNVKAHSQTDRVDLGTLRGYYNQSEAGSHTIQ

MMYGCDVGPDGRLLRGYQQDAYDGKDYIALNEDLRSWTAADMAAQITQRKWEAARVAEQL

RAYLEGTCVEWLRRYLENGKETLQRTDAPKTHMTHHAVSDHEATLRCWALSFYPAEITLT

WQRDGEDQTQDTELVETRPAGDGTFQKWASVVVPSGQEQRYTCHVQHEGLPKPLTLRWEP

SSQPTIPIVGIIAGLVLFGAMFAGAVVAAVRWRRKSSDRKGGSYSQAASSDSAQGSDMSL

TACKV

>sp|P30459|1A74_HUMAN 54 DTQFVRFDSDAASQRME

MAVMAPRTLLLLLLGALALTQTRAGSHSMRYFFTSVSRPGRGEPRFIAVGYVDDTQFVRF

DSDAASQRMEPRAPWIEQEGPEYWDQETRNVKAHSQTDRVDLGTLRGYYNQSEAGSHTIQ

MMYGCDVGPDGRLLRGYQQDAYDGKDYIALNEDLRSWTAADMAAQITQRKWEAARVAEQL

RAYLEGTCVEWLRRYLENGKETLQRTDAPKTHMTHHAVSDHEATLRCWALSFYPAEITLT

WQRDGEDQTQDTELVETRPAGDGTFQKWASVVVPSGQEQRYTCHVQHEGLPKPLTLRWEP

SSQPTIPIVGIIAGLVLFGAMFAGAVVAAVRWRRKSSDRKGGSYSQAASSDSAQGSDMSL

TACKV

>sp|P30459|1A74_HUMAN 54 DTQFVRFDSDAASQRMEP

MAVMAPRTLLLLLLGALALTQTRAGSHSMRYFFTSVSRPGRGEPRFIAVGYVDDTQFVRF

DSDAASQRMEPRAPWIEQEGPEYWDQETRNVKAHSQTDRVDLGTLRGYYNQSEAGSHTIQ

MMYGCDVGPDGRLLRGYQQDAYDGKDYIALNEDLRSWTAADMAAQITQRKWEAARVAEQL

RAYLEGTCVEWLRRYLENGKETLQRTDAPKTHMTHHAVSDHEATLRCWALSFYPAEITLT

WQRDGEDQTQDTELVETRPAGDGTFQKWASVVVPSGQEQRYTCHVQHEGLPKPLTLRWEP

SSQPTIPIVGIIAGLVLFGAMFAGAVVAAVRWRRKSSDRKGGSYSQAASSDSAQGSDMSL

TACKV

>sp|P30459|1A74_HUMAN 54 DTQFVRFDSDAASQRMEPR

MAVMAPRTLLLLLLGALALTQTRAGSHSMRYFFTSVSRPGRGEPRFIAVGYVDDTQFVRF

DSDAASQRMEPRAPWIEQEGPEYWDQETRNVKAHSQTDRVDLGTLRGYYNQSEAGSHTIQ

MMYGCDVGPDGRLLRGYQQDAYDGKDYIALNEDLRSWTAADMAAQITQRKWEAARVAEQL

RAYLEGTCVEWLRRYLENGKETLQRTDAPKTHMTHHAVSDHEATLRCWALSFYPAEITLT

WQRDGEDQTQDTELVETRPAGDGTFQKWASVVVPSGQEQRYTCHVQHEGLPKPLTLRWEP

SSQPTIPIVGIIAGLVLFGAMFAGAVVAAVRWRRKSSDRKGGSYSQAASSDSAQGSDMSL

TACKV

>sp|P30459|1A74_HUMAN 70 EPRAPWIEQEGPE

MAVMAPRTLLLLLLGALALTQTRAGSHSMRYFFTSVSRPGRGEPRFIAVGYVDDTQFVRF

DSDAASQRMEPRAPWIEQEGPEYWDQETRNVKAHSQTDRVDLGTLRGYYNQSEAGSHTIQ

MMYGCDVGPDGRLLRGYQQDAYDGKDYIALNEDLRSWTAADMAAQITQRKWEAARVAEQL

RAYLEGTCVEWLRRYLENGKETLQRTDAPKTHMTHHAVSDHEATLRCWALSFYPAEITLT

WQRDGEDQTQDTELVETRPAGDGTFQKWASVVVPSGQEQRYTCHVQHEGLPKPLTLRWEP

SSQPTIPIVGIIAGLVLFGAMFAGAVVAAVRWRRKSSDRKGGSYSQAASSDSAQGSDMSL

TACKV

>sp|P30459|1A74_HUMAN 70 EPRAPWIEQEGPEY

MAVMAPRTLLLLLLGALALTQTRAGSHSMRYFFTSVSRPGRGEPRFIAVGYVDDTQFVRF

DSDAASQRMEPRAPWIEQEGPEYWDQETRNVKAHSQTDRVDLGTLRGYYNQSEAGSHTIQ

MMYGCDVGPDGRLLRGYQQDAYDGKDYIALNEDLRSWTAADMAAQITQRKWEAARVAEQL

RAYLEGTCVEWLRRYLENGKETLQRTDAPKTHMTHHAVSDHEATLRCWALSFYPAEITLT

WQRDGEDQTQDTELVETRPAGDGTFQKWASVVVPSGQEQRYTCHVQHEGLPKPLTLRWEP

SSQPTIPIVGIIAGLVLFGAMFAGAVVAAVRWRRKSSDRKGGSYSQAASSDSAQGSDMSL

TACKV

>sp|P30459|1A74_HUMAN 70 EPRAPWIEQEGPEYWD

MAVMAPRTLLLLLLGALALTQTRAGSHSMRYFFTSVSRPGRGEPRFIAVGYVDDTQFVRF

DSDAASQRMEPRAPWIEQEGPEYWDQETRNVKAHSQTDRVDLGTLRGYYNQSEAGSHTIQ

MMYGCDVGPDGRLLRGYQQDAYDGKDYIALNEDLRSWTAADMAAQITQRKWEAARVAEQL

RAYLEGTCVEWLRRYLENGKETLQRTDAPKTHMTHHAVSDHEATLRCWALSFYPAEITLT

WQRDGEDQTQDTELVETRPAGDGTFQKWASVVVPSGQEQRYTCHVQHEGLPKPLTLRWEP

SSQPTIPIVGIIAGLVLFGAMFAGAVVAAVRWRRKSSDRKGGSYSQAASSDSAQGSDMSL

TACKV

>sp|P30459|1A74_HUMAN 57 FVRFDSDAASQ

MAVMAPRTLLLLLLGALALTQTRAGSHSMRYFFTSVSRPGRGEPRFIAVGYVDDTQFVRF

DSDAASQRMEPRAPWIEQEGPEYWDQETRNVKAHSQTDRVDLGTLRGYYNQSEAGSHTIQ

MMYGCDVGPDGRLLRGYQQDAYDGKDYIALNEDLRSWTAADMAAQITQRKWEAARVAEQL

RAYLEGTCVEWLRRYLENGKETLQRTDAPKTHMTHHAVSDHEATLRCWALSFYPAEITLT

WQRDGEDQTQDTELVETRPAGDGTFQKWASVVVPSGQEQRYTCHVQHEGLPKPLTLRWEP

SSQPTIPIVGIIAGLVLFGAMFAGAVVAAVRWRRKSSDRKGGSYSQAASSDSAQGSDMSL

TACKV

>sp|P30459|1A74_HUMAN 57 FVRFDSDAASQR

MAVMAPRTLLLLLLGALALTQTRAGSHSMRYFFTSVSRPGRGEPRFIAVGYVDDTQFVRF

DSDAASQRMEPRAPWIEQEGPEYWDQETRNVKAHSQTDRVDLGTLRGYYNQSEAGSHTIQ

MMYGCDVGPDGRLLRGYQQDAYDGKDYIALNEDLRSWTAADMAAQITQRKWEAARVAEQL

RAYLEGTCVEWLRRYLENGKETLQRTDAPKTHMTHHAVSDHEATLRCWALSFYPAEITLT

WQRDGEDQTQDTELVETRPAGDGTFQKWASVVVPSGQEQRYTCHVQHEGLPKPLTLRWEP

SSQPTIPIVGIIAGLVLFGAMFAGAVVAAVRWRRKSSDRKGGSYSQAASSDSAQGSDMSL

TACKV

>sp|P30459|1A74_HUMAN 57 FVRFDSDAASQRM

MAVMAPRTLLLLLLGALALTQTRAGSHSMRYFFTSVSRPGRGEPRFIAVGYVDDTQFVRF

DSDAASQRMEPRAPWIEQEGPEYWDQETRNVKAHSQTDRVDLGTLRGYYNQSEAGSHTIQ

MMYGCDVGPDGRLLRGYQQDAYDGKDYIALNEDLRSWTAADMAAQITQRKWEAARVAEQL

RAYLEGTCVEWLRRYLENGKETLQRTDAPKTHMTHHAVSDHEATLRCWALSFYPAEITLT

WQRDGEDQTQDTELVETRPAGDGTFQKWASVVVPSGQEQRYTCHVQHEGLPKPLTLRWEP

SSQPTIPIVGIIAGLVLFGAMFAGAVVAAVRWRRKSSDRKGGSYSQAASSDSAQGSDMSL

TACKV

>sp|P30459|1A74_HUMAN 57 FVRFDSDAASQRME

MAVMAPRTLLLLLLGALALTQTRAGSHSMRYFFTSVSRPGRGEPRFIAVGYVDDTQFVRF

DSDAASQRMEPRAPWIEQEGPEYWDQETRNVKAHSQTDRVDLGTLRGYYNQSEAGSHTIQ

MMYGCDVGPDGRLLRGYQQDAYDGKDYIALNEDLRSWTAADMAAQITQRKWEAARVAEQL

RAYLEGTCVEWLRRYLENGKETLQRTDAPKTHMTHHAVSDHEATLRCWALSFYPAEITLT

WQRDGEDQTQDTELVETRPAGDGTFQKWASVVVPSGQEQRYTCHVQHEGLPKPLTLRWEP

SSQPTIPIVGIIAGLVLFGAMFAGAVVAAVRWRRKSSDRKGGSYSQAASSDSAQGSDMSL

TACKV

>sp|P30459|1A74_HUMAN 57 FVRFDSDAASQRMEP

MAVMAPRTLLLLLLGALALTQTRAGSHSMRYFFTSVSRPGRGEPRFIAVGYVDDTQFVRF

DSDAASQRMEPRAPWIEQEGPEYWDQETRNVKAHSQTDRVDLGTLRGYYNQSEAGSHTIQ

MMYGCDVGPDGRLLRGYQQDAYDGKDYIALNEDLRSWTAADMAAQITQRKWEAARVAEQL

RAYLEGTCVEWLRRYLENGKETLQRTDAPKTHMTHHAVSDHEATLRCWALSFYPAEITLT

WQRDGEDQTQDTELVETRPAGDGTFQKWASVVVPSGQEQRYTCHVQHEGLPKPLTLRWEP

SSQPTIPIVGIIAGLVLFGAMFAGAVVAAVRWRRKSSDRKGGSYSQAASSDSAQGSDMSL

TACKV

>sp|P30459|1A74_HUMAN 57 FVRFDSDAASQRMEPR

MAVMAPRTLLLLLLGALALTQTRAGSHSMRYFFTSVSRPGRGEPRFIAVGYVDDTQFVRF

DSDAASQRMEPRAPWIEQEGPEYWDQETRNVKAHSQTDRVDLGTLRGYYNQSEAGSHTIQ

MMYGCDVGPDGRLLRGYQQDAYDGKDYIALNEDLRSWTAADMAAQITQRKWEAARVAEQL

RAYLEGTCVEWLRRYLENGKETLQRTDAPKTHMTHHAVSDHEATLRCWALSFYPAEITLT

WQRDGEDQTQDTELVETRPAGDGTFQKWASVVVPSGQEQRYTCHVQHEGLPKPLTLRWEP

SSQPTIPIVGIIAGLVLFGAMFAGAVVAAVRWRRKSSDRKGGSYSQAASSDSAQGSDMSL

TACKV

>sp|P30459|1A74_HUMAN 261 GDGTFQKWASVVVPSGQE

MAVMAPRTLLLLLLGALALTQTRAGSHSMRYFFTSVSRPGRGEPRFIAVGYVDDTQFVRF

DSDAASQRMEPRAPWIEQEGPEYWDQETRNVKAHSQTDRVDLGTLRGYYNQSEAGSHTIQ

MMYGCDVGPDGRLLRGYQQDAYDGKDYIALNEDLRSWTAADMAAQITQRKWEAARVAEQL

RAYLEGTCVEWLRRYLENGKETLQRTDAPKTHMTHHAVSDHEATLRCWALSFYPAEITLT

WQRDGEDQTQDTELVETRPAGDGTFQKWASVVVPSGQEQRYTCHVQHEGLPKPLTLRWEP

SSQPTIPIVGIIAGLVLFGAMFAGAVVAAVRWRRKSSDRKGGSYSQAASSDSAQGSDMSL

TACKV

>sp|P30459|1A74_HUMAN 263 GTFQKWASVVVPSG

MAVMAPRTLLLLLLGALALTQTRAGSHSMRYFFTSVSRPGRGEPRFIAVGYVDDTQFVRF

DSDAASQRMEPRAPWIEQEGPEYWDQETRNVKAHSQTDRVDLGTLRGYYNQSEAGSHTIQ

MMYGCDVGPDGRLLRGYQQDAYDGKDYIALNEDLRSWTAADMAAQITQRKWEAARVAEQL

RAYLEGTCVEWLRRYLENGKETLQRTDAPKTHMTHHAVSDHEATLRCWALSFYPAEITLT

WQRDGEDQTQDTELVETRPAGDGTFQKWASVVVPSGQEQRYTCHVQHEGLPKPLTLRWEP

SSQPTIPIVGIIAGLVLFGAMFAGAVVAAVRWRRKSSDRKGGSYSQAASSDSAQGSDMSL

TACKV

>sp|P30459|1A74_HUMAN 263 GTFQKWASVVVPSGQ

MAVMAPRTLLLLLLGALALTQTRAGSHSMRYFFTSVSRPGRGEPRFIAVGYVDDTQFVRF

DSDAASQRMEPRAPWIEQEGPEYWDQETRNVKAHSQTDRVDLGTLRGYYNQSEAGSHTIQ

MMYGCDVGPDGRLLRGYQQDAYDGKDYIALNEDLRSWTAADMAAQITQRKWEAARVAEQL

RAYLEGTCVEWLRRYLENGKETLQRTDAPKTHMTHHAVSDHEATLRCWALSFYPAEITLT

WQRDGEDQTQDTELVETRPAGDGTFQKWASVVVPSGQEQRYTCHVQHEGLPKPLTLRWEP

SSQPTIPIVGIIAGLVLFGAMFAGAVVAAVRWRRKSSDRKGGSYSQAASSDSAQGSDMSL

TACKV

>sp|P30459|1A74_HUMAN 263 GTFQKWASVVVPSGQEQRYTCHV

MAVMAPRTLLLLLLGALALTQTRAGSHSMRYFFTSVSRPGRGEPRFIAVGYVDDTQFVRF

DSDAASQRMEPRAPWIEQEGPEYWDQETRNVKAHSQTDRVDLGTLRGYYNQSEAGSHTIQ

MMYGCDVGPDGRLLRGYQQDAYDGKDYIALNEDLRSWTAADMAAQITQRKWEAARVAEQL

RAYLEGTCVEWLRRYLENGKETLQRTDAPKTHMTHHAVSDHEATLRCWALSFYPAEITLT

WQRDGEDQTQDTELVETRPAGDGTFQKWASVVVPSGQEQRYTCHVQHEGLPKPLTLRWEP

SSQPTIPIVGIIAGLVLFGAMFAGAVVAAVRWRRKSSDRKGGSYSQAASSDSAQGSDMSL

TACKV

>sp|P30459|1A74_HUMAN 76 IEQEGPEYW

MAVMAPRTLLLLLLGALALTQTRAGSHSMRYFFTSVSRPGRGEPRFIAVGYVDDTQFVRF

DSDAASQRMEPRAPWIEQEGPEYWDQETRNVKAHSQTDRVDLGTLRGYYNQSEAGSHTIQ

MMYGCDVGPDGRLLRGYQQDAYDGKDYIALNEDLRSWTAADMAAQITQRKWEAARVAEQL

RAYLEGTCVEWLRRYLENGKETLQRTDAPKTHMTHHAVSDHEATLRCWALSFYPAEITLT

WQRDGEDQTQDTELVETRPAGDGTFQKWASVVVPSGQEQRYTCHVQHEGLPKPLTLRWEP

SSQPTIPIVGIIAGLVLFGAMFAGAVVAAVRWRRKSSDRKGGSYSQAASSDSAQGSDMSL

TACKV

>sp|P30459|1A74_HUMAN 170 KWEAARVAEQL

MAVMAPRTLLLLLLGALALTQTRAGSHSMRYFFTSVSRPGRGEPRFIAVGYVDDTQFVRF

DSDAASQRMEPRAPWIEQEGPEYWDQETRNVKAHSQTDRVDLGTLRGYYNQSEAGSHTIQ

MMYGCDVGPDGRLLRGYQQDAYDGKDYIALNEDLRSWTAADMAAQITQRKWEAARVAEQL

RAYLEGTCVEWLRRYLENGKETLQRTDAPKTHMTHHAVSDHEATLRCWALSFYPAEITLT

WQRDGEDQTQDTELVETRPAGDGTFQKWASVVVPSGQEQRYTCHVQHEGLPKPLTLRWEP

SSQPTIPIVGIIAGLVLFGAMFAGAVVAAVRWRRKSSDRKGGSYSQAASSDSAQGSDMSL

TACKV

>sp|P30459|1A74_HUMAN 170 KWEAARVAEQLR

MAVMAPRTLLLLLLGALALTQTRAGSHSMRYFFTSVSRPGRGEPRFIAVGYVDDTQFVRF

DSDAASQRMEPRAPWIEQEGPEYWDQETRNVKAHSQTDRVDLGTLRGYYNQSEAGSHTIQ

MMYGCDVGPDGRLLRGYQQDAYDGKDYIALNEDLRSWTAADMAAQITQRKWEAARVAEQL

RAYLEGTCVEWLRRYLENGKETLQRTDAPKTHMTHHAVSDHEATLRCWALSFYPAEITLT

WQRDGEDQTQDTELVETRPAGDGTFQKWASVVVPSGQEQRYTCHVQHEGLPKPLTLRWEP

SSQPTIPIVGIIAGLVLFGAMFAGAVVAAVRWRRKSSDRKGGSYSQAASSDSAQGSDMSL

TACKV

>sp|P30459|1A74_HUMAN 71 PRAPWIEQEGPEY

MAVMAPRTLLLLLLGALALTQTRAGSHSMRYFFTSVSRPGRGEPRFIAVGYVDDTQFVRF

DSDAASQRMEPRAPWIEQEGPEYWDQETRNVKAHSQTDRVDLGTLRGYYNQSEAGSHTIQ

MMYGCDVGPDGRLLRGYQQDAYDGKDYIALNEDLRSWTAADMAAQITQRKWEAARVAEQL

RAYLEGTCVEWLRRYLENGKETLQRTDAPKTHMTHHAVSDHEATLRCWALSFYPAEITLT

WQRDGEDQTQDTELVETRPAGDGTFQKWASVVVPSGQEQRYTCHVQHEGLPKPLTLRWEP

SSQPTIPIVGIIAGLVLFGAMFAGAVVAAVRWRRKSSDRKGGSYSQAASSDSAQGSDMSL

TACKV

>sp|P30459|1A74_HUMAN 56 QFVRFDSDAASQR

MAVMAPRTLLLLLLGALALTQTRAGSHSMRYFFTSVSRPGRGEPRFIAVGYVDDTQFVRF

DSDAASQRMEPRAPWIEQEGPEYWDQETRNVKAHSQTDRVDLGTLRGYYNQSEAGSHTIQ

MMYGCDVGPDGRLLRGYQQDAYDGKDYIALNEDLRSWTAADMAAQITQRKWEAARVAEQL

RAYLEGTCVEWLRRYLENGKETLQRTDAPKTHMTHHAVSDHEATLRCWALSFYPAEITLT

WQRDGEDQTQDTELVETRPAGDGTFQKWASVVVPSGQEQRYTCHVQHEGLPKPLTLRWEP

SSQPTIPIVGIIAGLVLFGAMFAGAVVAAVRWRRKSSDRKGGSYSQAASSDSAQGSDMSL

TACKV

>sp|P30459|1A74_HUMAN 168 QRKWEAARVA

MAVMAPRTLLLLLLGALALTQTRAGSHSMRYFFTSVSRPGRGEPRFIAVGYVDDTQFVRF

DSDAASQRMEPRAPWIEQEGPEYWDQETRNVKAHSQTDRVDLGTLRGYYNQSEAGSHTIQ

MMYGCDVGPDGRLLRGYQQDAYDGKDYIALNEDLRSWTAADMAAQITQRKWEAARVAEQL

RAYLEGTCVEWLRRYLENGKETLQRTDAPKTHMTHHAVSDHEATLRCWALSFYPAEITLT

WQRDGEDQTQDTELVETRPAGDGTFQKWASVVVPSGQEQRYTCHVQHEGLPKPLTLRWEP

SSQPTIPIVGIIAGLVLFGAMFAGAVVAAVRWRRKSSDRKGGSYSQAASSDSAQGSDMSL

TACKV

>sp|P30459|1A74_HUMAN 59 RFDSDAASQR

MAVMAPRTLLLLLLGALALTQTRAGSHSMRYFFTSVSRPGRGEPRFIAVGYVDDTQFVRF

DSDAASQRMEPRAPWIEQEGPEYWDQETRNVKAHSQTDRVDLGTLRGYYNQSEAGSHTIQ

MMYGCDVGPDGRLLRGYQQDAYDGKDYIALNEDLRSWTAADMAAQITQRKWEAARVAEQL

RAYLEGTCVEWLRRYLENGKETLQRTDAPKTHMTHHAVSDHEATLRCWALSFYPAEITLT

WQRDGEDQTQDTELVETRPAGDGTFQKWASVVVPSGQEQRYTCHVQHEGLPKPLTLRWEP

SSQPTIPIVGIIAGLVLFGAMFAGAVVAAVRWRRKSSDRKGGSYSQAASSDSAQGSDMSL

TACKV

>sp|P30459|1A74_HUMAN 169 RKWEAARVAE

MAVMAPRTLLLLLLGALALTQTRAGSHSMRYFFTSVSRPGRGEPRFIAVGYVDDTQFVRF

DSDAASQRMEPRAPWIEQEGPEYWDQETRNVKAHSQTDRVDLGTLRGYYNQSEAGSHTIQ

MMYGCDVGPDGRLLRGYQQDAYDGKDYIALNEDLRSWTAADMAAQITQRKWEAARVAEQL

RAYLEGTCVEWLRRYLENGKETLQRTDAPKTHMTHHAVSDHEATLRCWALSFYPAEITLT

WQRDGEDQTQDTELVETRPAGDGTFQKWASVVVPSGQEQRYTCHVQHEGLPKPLTLRWEP

SSQPTIPIVGIIAGLVLFGAMFAGAVVAAVRWRRKSSDRKGGSYSQAASSDSAQGSDMSL

TACKV

>sp|P30459|1A74_HUMAN 258 RPAGDGTFQKWASVVV

MAVMAPRTLLLLLLGALALTQTRAGSHSMRYFFTSVSRPGRGEPRFIAVGYVDDTQFVRF

DSDAASQRMEPRAPWIEQEGPEYWDQETRNVKAHSQTDRVDLGTLRGYYNQSEAGSHTIQ

MMYGCDVGPDGRLLRGYQQDAYDGKDYIALNEDLRSWTAADMAAQITQRKWEAARVAEQL

RAYLEGTCVEWLRRYLENGKETLQRTDAPKTHMTHHAVSDHEATLRCWALSFYPAEITLT

WQRDGEDQTQDTELVETRPAGDGTFQKWASVVVPSGQEQRYTCHVQHEGLPKPLTLRWEP

SSQPTIPIVGIIAGLVLFGAMFAGAVVAAVRWRRKSSDRKGGSYSQAASSDSAQGSDMSL

TACKV

>sp|P30459|1A74_HUMAN 258 RPAGDGTFQKWASVVVPSGQ

MAVMAPRTLLLLLLGALALTQTRAGSHSMRYFFTSVSRPGRGEPRFIAVGYVDDTQFVRF

DSDAASQRMEPRAPWIEQEGPEYWDQETRNVKAHSQTDRVDLGTLRGYYNQSEAGSHTIQ

MMYGCDVGPDGRLLRGYQQDAYDGKDYIALNEDLRSWTAADMAAQITQRKWEAARVAEQL

RAYLEGTCVEWLRRYLENGKETLQRTDAPKTHMTHHAVSDHEATLRCWALSFYPAEITLT

WQRDGEDQTQDTELVETRPAGDGTFQKWASVVVPSGQEQRYTCHVQHEGLPKPLTLRWEP

SSQPTIPIVGIIAGLVLFGAMFAGAVVAAVRWRRKSSDRKGGSYSQAASSDSAQGSDMSL

TACKV

>sp|P30459|1A74_HUMAN 55 TQFVRFDSDAASQ

MAVMAPRTLLLLLLGALALTQTRAGSHSMRYFFTSVSRPGRGEPRFIAVGYVDDTQFVRF

DSDAASQRMEPRAPWIEQEGPEYWDQETRNVKAHSQTDRVDLGTLRGYYNQSEAGSHTIQ

MMYGCDVGPDGRLLRGYQQDAYDGKDYIALNEDLRSWTAADMAAQITQRKWEAARVAEQL

RAYLEGTCVEWLRRYLENGKETLQRTDAPKTHMTHHAVSDHEATLRCWALSFYPAEITLT

WQRDGEDQTQDTELVETRPAGDGTFQKWASVVVPSGQEQRYTCHVQHEGLPKPLTLRWEP

SSQPTIPIVGIIAGLVLFGAMFAGAVVAAVRWRRKSSDRKGGSYSQAASSDSAQGSDMSL

TACKV

>sp|P30459|1A74_HUMAN 55 TQFVRFDSDAASQR

MAVMAPRTLLLLLLGALALTQTRAGSHSMRYFFTSVSRPGRGEPRFIAVGYVDDTQFVRF

DSDAASQRMEPRAPWIEQEGPEYWDQETRNVKAHSQTDRVDLGTLRGYYNQSEAGSHTIQ

MMYGCDVGPDGRLLRGYQQDAYDGKDYIALNEDLRSWTAADMAAQITQRKWEAARVAEQL

RAYLEGTCVEWLRRYLENGKETLQRTDAPKTHMTHHAVSDHEATLRCWALSFYPAEITLT

WQRDGEDQTQDTELVETRPAGDGTFQKWASVVVPSGQEQRYTCHVQHEGLPKPLTLRWEP

SSQPTIPIVGIIAGLVLFGAMFAGAVVAAVRWRRKSSDRKGGSYSQAASSDSAQGSDMSL

TACKV

>sp|P30459|1A74_HUMAN 55 TQFVRFDSDAASQRM

MAVMAPRTLLLLLLGALALTQTRAGSHSMRYFFTSVSRPGRGEPRFIAVGYVDDTQFVRF

DSDAASQRMEPRAPWIEQEGPEYWDQETRNVKAHSQTDRVDLGTLRGYYNQSEAGSHTIQ

MMYGCDVGPDGRLLRGYQQDAYDGKDYIALNEDLRSWTAADMAAQITQRKWEAARVAEQL

RAYLEGTCVEWLRRYLENGKETLQRTDAPKTHMTHHAVSDHEATLRCWALSFYPAEITLT

WQRDGEDQTQDTELVETRPAGDGTFQKWASVVVPSGQEQRYTCHVQHEGLPKPLTLRWEP

SSQPTIPIVGIIAGLVLFGAMFAGAVVAAVRWRRKSSDRKGGSYSQAASSDSAQGSDMSL

TACKV

>sp|P30459|1A74_HUMAN 55 TQFVRFDSDAASQRME

MAVMAPRTLLLLLLGALALTQTRAGSHSMRYFFTSVSRPGRGEPRFIAVGYVDDTQFVRF

DSDAASQRMEPRAPWIEQEGPEYWDQETRNVKAHSQTDRVDLGTLRGYYNQSEAGSHTIQ

MMYGCDVGPDGRLLRGYQQDAYDGKDYIALNEDLRSWTAADMAAQITQRKWEAARVAEQL

RAYLEGTCVEWLRRYLENGKETLQRTDAPKTHMTHHAVSDHEATLRCWALSFYPAEITLT

WQRDGEDQTQDTELVETRPAGDGTFQKWASVVVPSGQEQRYTCHVQHEGLPKPLTLRWEP

SSQPTIPIVGIIAGLVLFGAMFAGAVVAAVRWRRKSSDRKGGSYSQAASSDSAQGSDMSL

TACKV

>sp|P30459|1A74_HUMAN 55 TQFVRFDSDAASQRMEP

MAVMAPRTLLLLLLGALALTQTRAGSHSMRYFFTSVSRPGRGEPRFIAVGYVDDTQFVRF

DSDAASQRMEPRAPWIEQEGPEYWDQETRNVKAHSQTDRVDLGTLRGYYNQSEAGSHTIQ

MMYGCDVGPDGRLLRGYQQDAYDGKDYIALNEDLRSWTAADMAAQITQRKWEAARVAEQL

RAYLEGTCVEWLRRYLENGKETLQRTDAPKTHMTHHAVSDHEATLRCWALSFYPAEITLT

WQRDGEDQTQDTELVETRPAGDGTFQKWASVVVPSGQEQRYTCHVQHEGLPKPLTLRWEP

SSQPTIPIVGIIAGLVLFGAMFAGAVVAAVRWRRKSSDRKGGSYSQAASSDSAQGSDMSL

TACKV

>sp|P30459|1A74_HUMAN 52 VDDTQFVRFDSDAASQ

MAVMAPRTLLLLLLGALALTQTRAGSHSMRYFFTSVSRPGRGEPRFIAVGYVDDTQFVRF

DSDAASQRMEPRAPWIEQEGPEYWDQETRNVKAHSQTDRVDLGTLRGYYNQSEAGSHTIQ

MMYGCDVGPDGRLLRGYQQDAYDGKDYIALNEDLRSWTAADMAAQITQRKWEAARVAEQL

RAYLEGTCVEWLRRYLENGKETLQRTDAPKTHMTHHAVSDHEATLRCWALSFYPAEITLT

WQRDGEDQTQDTELVETRPAGDGTFQKWASVVVPSGQEQRYTCHVQHEGLPKPLTLRWEP

SSQPTIPIVGIIAGLVLFGAMFAGAVVAAVRWRRKSSDRKGGSYSQAASSDSAQGSDMSL

TACKV

>sp|P30459|1A74_HUMAN 52 VDDTQFVRFDSDAASQR

MAVMAPRTLLLLLLGALALTQTRAGSHSMRYFFTSVSRPGRGEPRFIAVGYVDDTQFVRF

DSDAASQRMEPRAPWIEQEGPEYWDQETRNVKAHSQTDRVDLGTLRGYYNQSEAGSHTIQ

MMYGCDVGPDGRLLRGYQQDAYDGKDYIALNEDLRSWTAADMAAQITQRKWEAARVAEQL

RAYLEGTCVEWLRRYLENGKETLQRTDAPKTHMTHHAVSDHEATLRCWALSFYPAEITLT

WQRDGEDQTQDTELVETRPAGDGTFQKWASVVVPSGQEQRYTCHVQHEGLPKPLTLRWEP

SSQPTIPIVGIIAGLVLFGAMFAGAVVAAVRWRRKSSDRKGGSYSQAASSDSAQGSDMSL

TACKV

>sp|P30459|1A74_HUMAN 52 VDDTQFVRFDSDAASQRM

MAVMAPRTLLLLLLGALALTQTRAGSHSMRYFFTSVSRPGRGEPRFIAVGYVDDTQFVRF

DSDAASQRMEPRAPWIEQEGPEYWDQETRNVKAHSQTDRVDLGTLRGYYNQSEAGSHTIQ

MMYGCDVGPDGRLLRGYQQDAYDGKDYIALNEDLRSWTAADMAAQITQRKWEAARVAEQL

RAYLEGTCVEWLRRYLENGKETLQRTDAPKTHMTHHAVSDHEATLRCWALSFYPAEITLT

WQRDGEDQTQDTELVETRPAGDGTFQKWASVVVPSGQEQRYTCHVQHEGLPKPLTLRWEP

SSQPTIPIVGIIAGLVLFGAMFAGAVVAAVRWRRKSSDRKGGSYSQAASSDSAQGSDMSL

TACKV

>sp|P30459|1A74_HUMAN 52 VDDTQFVRFDSDAASQRME

MAVMAPRTLLLLLLGALALTQTRAGSHSMRYFFTSVSRPGRGEPRFIAVGYVDDTQFVRF

DSDAASQRMEPRAPWIEQEGPEYWDQETRNVKAHSQTDRVDLGTLRGYYNQSEAGSHTIQ

MMYGCDVGPDGRLLRGYQQDAYDGKDYIALNEDLRSWTAADMAAQITQRKWEAARVAEQL

RAYLEGTCVEWLRRYLENGKETLQRTDAPKTHMTHHAVSDHEATLRCWALSFYPAEITLT

WQRDGEDQTQDTELVETRPAGDGTFQKWASVVVPSGQEQRYTCHVQHEGLPKPLTLRWEP

SSQPTIPIVGIIAGLVLFGAMFAGAVVAAVRWRRKSSDRKGGSYSQAASSDSAQGSDMSL

TACKV

>sp|P30459|1A74_HUMAN 52 VDDTQFVRFDSDAASQRMEP

MAVMAPRTLLLLLLGALALTQTRAGSHSMRYFFTSVSRPGRGEPRFIAVGYVDDTQFVRF

DSDAASQRMEPRAPWIEQEGPEYWDQETRNVKAHSQTDRVDLGTLRGYYNQSEAGSHTIQ

MMYGCDVGPDGRLLRGYQQDAYDGKDYIALNEDLRSWTAADMAAQITQRKWEAARVAEQL

RAYLEGTCVEWLRRYLENGKETLQRTDAPKTHMTHHAVSDHEATLRCWALSFYPAEITLT

WQRDGEDQTQDTELVETRPAGDGTFQKWASVVVPSGQEQRYTCHVQHEGLPKPLTLRWEP

SSQPTIPIVGIIAGLVLFGAMFAGAVVAAVRWRRKSSDRKGGSYSQAASSDSAQGSDMSL

TACKV

>sp|P30459|1A74_HUMAN 52 VDDTQFVRFDSDAASQRMEPRA

MAVMAPRTLLLLLLGALALTQTRAGSHSMRYFFTSVSRPGRGEPRFIAVGYVDDTQFVRF

DSDAASQRMEPRAPWIEQEGPEYWDQETRNVKAHSQTDRVDLGTLRGYYNQSEAGSHTIQ

MMYGCDVGPDGRLLRGYQQDAYDGKDYIALNEDLRSWTAADMAAQITQRKWEAARVAEQL

RAYLEGTCVEWLRRYLENGKETLQRTDAPKTHMTHHAVSDHEATLRCWALSFYPAEITLT

WQRDGEDQTQDTELVETRPAGDGTFQKWASVVVPSGQEQRYTCHVQHEGLPKPLTLRWEP

SSQPTIPIVGIIAGLVLFGAMFAGAVVAAVRWRRKSSDRKGGSYSQAASSDSAQGSDMSL

TACKV

>sp|P30459|1A74_HUMAN 52 VDDTQFVRFDSDAASQRMEPRAP

MAVMAPRTLLLLLLGALALTQTRAGSHSMRYFFTSVSRPGRGEPRFIAVGYVDDTQFVRF

DSDAASQRMEPRAPWIEQEGPEYWDQETRNVKAHSQTDRVDLGTLRGYYNQSEAGSHTIQ

MMYGCDVGPDGRLLRGYQQDAYDGKDYIALNEDLRSWTAADMAAQITQRKWEAARVAEQL

RAYLEGTCVEWLRRYLENGKETLQRTDAPKTHMTHHAVSDHEATLRCWALSFYPAEITLT

WQRDGEDQTQDTELVETRPAGDGTFQKWASVVVPSGQEQRYTCHVQHEGLPKPLTLRWEP

SSQPTIPIVGIIAGLVLFGAMFAGAVVAAVRWRRKSSDRKGGSYSQAASSDSAQGSDMSL

TACKV

>sp|P30459|1A74_HUMAN 49 VGYVDDTQFVRFDSDAASQR

MAVMAPRTLLLLLLGALALTQTRAGSHSMRYFFTSVSRPGRGEPRFIAVGYVDDTQFVRF

DSDAASQRMEPRAPWIEQEGPEYWDQETRNVKAHSQTDRVDLGTLRGYYNQSEAGSHTIQ

MMYGCDVGPDGRLLRGYQQDAYDGKDYIALNEDLRSWTAADMAAQITQRKWEAARVAEQL

RAYLEGTCVEWLRRYLENGKETLQRTDAPKTHMTHHAVSDHEATLRCWALSFYPAEITLT

WQRDGEDQTQDTELVETRPAGDGTFQKWASVVVPSGQEQRYTCHVQHEGLPKPLTLRWEP

SSQPTIPIVGIIAGLVLFGAMFAGAVVAAVRWRRKSSDRKGGSYSQAASSDSAQGSDMSL

TACKV

>sp|P30459|1A74_HUMAN 49 VGYVDDTQFVRFDSDAASQRMEPRAPWIE

MAVMAPRTLLLLLLGALALTQTRAGSHSMRYFFTSVSRPGRGEPRFIAVGYVDDTQFVRF

DSDAASQRMEPRAPWIEQEGPEYWDQETRNVKAHSQTDRVDLGTLRGYYNQSEAGSHTIQ

MMYGCDVGPDGRLLRGYQQDAYDGKDYIALNEDLRSWTAADMAAQITQRKWEAARVAEQL

RAYLEGTCVEWLRRYLENGKETLQRTDAPKTHMTHHAVSDHEATLRCWALSFYPAEITLT

WQRDGEDQTQDTELVETRPAGDGTFQKWASVVVPSGQEQRYTCHVQHEGLPKPLTLRWEP

SSQPTIPIVGIIAGLVLFGAMFAGAVVAAVRWRRKSSDRKGGSYSQAASSDSAQGSDMSL

TACKV

>sp|P30459|1A74_HUMAN 58 VRFDSDAASQR

MAVMAPRTLLLLLLGALALTQTRAGSHSMRYFFTSVSRPGRGEPRFIAVGYVDDTQFVRF

DSDAASQRMEPRAPWIEQEGPEYWDQETRNVKAHSQTDRVDLGTLRGYYNQSEAGSHTIQ

MMYGCDVGPDGRLLRGYQQDAYDGKDYIALNEDLRSWTAADMAAQITQRKWEAARVAEQL

RAYLEGTCVEWLRRYLENGKETLQRTDAPKTHMTHHAVSDHEATLRCWALSFYPAEITLT

WQRDGEDQTQDTELVETRPAGDGTFQKWASVVVPSGQEQRYTCHVQHEGLPKPLTLRWEP

SSQPTIPIVGIIAGLVLFGAMFAGAVVAAVRWRRKSSDRKGGSYSQAASSDSAQGSDMSL

TACKV

>sp|P30459|1A74_HUMAN 171 WEAARVAEQ

MAVMAPRTLLLLLLGALALTQTRAGSHSMRYFFTSVSRPGRGEPRFIAVGYVDDTQFVRF

DSDAASQRMEPRAPWIEQEGPEYWDQETRNVKAHSQTDRVDLGTLRGYYNQSEAGSHTIQ

MMYGCDVGPDGRLLRGYQQDAYDGKDYIALNEDLRSWTAADMAAQITQRKWEAARVAEQL

RAYLEGTCVEWLRRYLENGKETLQRTDAPKTHMTHHAVSDHEATLRCWALSFYPAEITLT

WQRDGEDQTQDTELVETRPAGDGTFQKWASVVVPSGQEQRYTCHVQHEGLPKPLTLRWEP

SSQPTIPIVGIIAGLVLFGAMFAGAVVAAVRWRRKSSDRKGGSYSQAASSDSAQGSDMSL

TACKV

>sp|P30459|1A74_HUMAN 171 WEAARVAEQLRAYLE

MAVMAPRTLLLLLLGALALTQTRAGSHSMRYFFTSVSRPGRGEPRFIAVGYVDDTQFVRF

DSDAASQRMEPRAPWIEQEGPEYWDQETRNVKAHSQTDRVDLGTLRGYYNQSEAGSHTIQ

MMYGCDVGPDGRLLRGYQQDAYDGKDYIALNEDLRSWTAADMAAQITQRKWEAARVAEQL

RAYLEGTCVEWLRRYLENGKETLQRTDAPKTHMTHHAVSDHEATLRCWALSFYPAEITLT

WQRDGEDQTQDTELVETRPAGDGTFQKWASVVVPSGQEQRYTCHVQHEGLPKPLTLRWEP

SSQPTIPIVGIIAGLVLFGAMFAGAVVAAVRWRRKSSDRKGGSYSQAASSDSAQGSDMSL

TACKV

>sp|P30459|1A74_HUMAN 75 WIEQEGPEYW

MAVMAPRTLLLLLLGALALTQTRAGSHSMRYFFTSVSRPGRGEPRFIAVGYVDDTQFVRF

DSDAASQRMEPRAPWIEQEGPEYWDQETRNVKAHSQTDRVDLGTLRGYYNQSEAGSHTIQ

MMYGCDVGPDGRLLRGYQQDAYDGKDYIALNEDLRSWTAADMAAQITQRKWEAARVAEQL

RAYLEGTCVEWLRRYLENGKETLQRTDAPKTHMTHHAVSDHEATLRCWALSFYPAEITLT

WQRDGEDQTQDTELVETRPAGDGTFQKWASVVVPSGQEQRYTCHVQHEGLPKPLTLRWEP

SSQPTIPIVGIIAGLVLFGAMFAGAVVAAVRWRRKSSDRKGGSYSQAASSDSAQGSDMSL

TACKV

>sp|P30459|1A74_HUMAN 51 YVDDTQFVRFDSDAASQRMEPRAP

MAVMAPRTLLLLLLGALALTQTRAGSHSMRYFFTSVSRPGRGEPRFIAVGYVDDTQFVRF

DSDAASQRMEPRAPWIEQEGPEYWDQETRNVKAHSQTDRVDLGTLRGYYNQSEAGSHTIQ

MMYGCDVGPDGRLLRGYQQDAYDGKDYIALNEDLRSWTAADMAAQITQRKWEAARVAEQL

RAYLEGTCVEWLRRYLENGKETLQRTDAPKTHMTHHAVSDHEATLRCWALSFYPAEITLT

WQRDGEDQTQDTELVETRPAGDGTFQKWASVVVPSGQEQRYTCHVQHEGLPKPLTLRWEP

SSQPTIPIVGIIAGLVLFGAMFAGAVVAAVRWRRKSSDRKGGSYSQAASSDSAQGSDMSL

TACKV

>sp|P10316|1A69_HUMAN 128 GSDWRFLRGYHQYA

MAVMAPRTLVLLLSGALALTQTWAGSHSMRYFYTSVSRPGRGEPRFIAVGYVDDTQFVRF

DSDAASQRMEPRAPWIEQEGPEYWDRNTRNVKAQSQTDRVDLGTLRGYYNQSEAGSHTVQ

RMYGCDVGSDWRFLRGYHQYAYDGKDYIALKEDLRSWTAADMAAQTTKHKWEAAHVAEQL

RAYLEGTCVEWLRRYLENGKETLQRTDAPKTHMTHHAVSDHEATLRCWALSFYPAEITLT

WQRDGEDQTQDTELVETRPAGDGTFQKWAAVVVPSGQEQRYTCHVQHEGLPKPLTLRWEP

SSQPTIPIVGIIAGLVLFGAVITGAVVAAVMWRRKSSDRKGGSYSQAASSDSAQGSDVSL

TACKV

>sp|P10316|1A69_HUMAN 166 TTKHKWEAAHVAEQLR

MAVMAPRTLVLLLSGALALTQTWAGSHSMRYFYTSVSRPGRGEPRFIAVGYVDDTQFVRF

DSDAASQRMEPRAPWIEQEGPEYWDRNTRNVKAQSQTDRVDLGTLRGYYNQSEAGSHTVQ

RMYGCDVGSDWRFLRGYHQYAYDGKDYIALKEDLRSWTAADMAAQTTKHKWEAAHVAEQL

RAYLEGTCVEWLRRYLENGKETLQRTDAPKTHMTHHAVSDHEATLRCWALSFYPAEITLT

WQRDGEDQTQDTELVETRPAGDGTFQKWAAVVVPSGQEQRYTCHVQHEGLPKPLTLRWEP

SSQPTIPIVGIIAGLVLFGAVITGAVVAAVMWRRKSSDRKGGSYSQAASSDSAQGSDVSL

TACKV

>sp|P10316|1A69_HUMAN 127 VGSDWRFLRGYHQY

MAVMAPRTLVLLLSGALALTQTWAGSHSMRYFYTSVSRPGRGEPRFIAVGYVDDTQFVRF

DSDAASQRMEPRAPWIEQEGPEYWDRNTRNVKAQSQTDRVDLGTLRGYYNQSEAGSHTVQ

RMYGCDVGSDWRFLRGYHQYAYDGKDYIALKEDLRSWTAADMAAQTTKHKWEAAHVAEQL

RAYLEGTCVEWLRRYLENGKETLQRTDAPKTHMTHHAVSDHEATLRCWALSFYPAEITLT

WQRDGEDQTQDTELVETRPAGDGTFQKWAAVVVPSGQEQRYTCHVQHEGLPKPLTLRWEP

SSQPTIPIVGIIAGLVLFGAVITGAVVAAVMWRRKSSDRKGGSYSQAASSDSAQGSDVSL

TACKV

>sp|P10316|1A69_HUMAN 127 VGSDWRFLRGYHQYA

MAVMAPRTLVLLLSGALALTQTWAGSHSMRYFYTSVSRPGRGEPRFIAVGYVDDTQFVRF

DSDAASQRMEPRAPWIEQEGPEYWDRNTRNVKAQSQTDRVDLGTLRGYYNQSEAGSHTVQ

RMYGCDVGSDWRFLRGYHQYAYDGKDYIALKEDLRSWTAADMAAQTTKHKWEAAHVAEQL

RAYLEGTCVEWLRRYLENGKETLQRTDAPKTHMTHHAVSDHEATLRCWALSFYPAEITLT

WQRDGEDQTQDTELVETRPAGDGTFQKWAAVVVPSGQEQRYTCHVQHEGLPKPLTLRWEP

SSQPTIPIVGIIAGLVLFGAVITGAVVAAVMWRRKSSDRKGGSYSQAASSDSAQGSDVSL

TACKV

>sp|P10316|1A69_HUMAN 127 VGSDWRFLRGYHQYAYDG

MAVMAPRTLVLLLSGALALTQTWAGSHSMRYFYTSVSRPGRGEPRFIAVGYVDDTQFVRF

DSDAASQRMEPRAPWIEQEGPEYWDRNTRNVKAQSQTDRVDLGTLRGYYNQSEAGSHTVQ

RMYGCDVGSDWRFLRGYHQYAYDGKDYIALKEDLRSWTAADMAAQTTKHKWEAAHVAEQL

RAYLEGTCVEWLRRYLENGKETLQRTDAPKTHMTHHAVSDHEATLRCWALSFYPAEITLT

WQRDGEDQTQDTELVETRPAGDGTFQKWAAVVVPSGQEQRYTCHVQHEGLPKPLTLRWEP

SSQPTIPIVGIIAGLVLFGAVITGAVVAAVMWRRKSSDRKGGSYSQAASSDSAQGSDVSL

TACKV

>sp|P05534|1A24_HUMAN 159 AADMAAQITKRKWEAAH

MAVMAPRTLVLLLSGALALTQTWAGSHSMRYFSTSVSRPGRGEPRFIAVGYVDDTQFVRF

DSDAASQRMEPRAPWIEQEGPEYWDEETGKVKAHSQTDRENLRIALRYYNQSEAGSHTLQ

MMFGCDVGSDGRFLRGYHQYAYDGKDYIALKEDLRSWTAADMAAQITKRKWEAAHVAEQQ

RAYLEGTCVDGLRRYLENGKETLQRTDPPKTHMTHHPISDHEATLRCWALGFYPAEITLT

WQRDGEDQTQDTELVETRPAGDGTFQKWAAVVVPSGEEQRYTCHVQHEGLPKPLTLRWEP

SSQPTVPIVGIIAGLVLLGAVITGAVVAAVMWRRNSSDRKGGSYSQAASSDSAQGSDVSL

TACKV

>sp|P13746|1A11_HUMAN 168 KRKWEAAHAAEQQR

MAVMAPRTLLLLLSGALALTQTWAGSHSMRYFYTSVSRPGRGEPRFIAVGYVDDTQFVRF

DSDAASQRMEPRAPWIEQEGPEYWDQETRNVKAQSQTDRVDLGTLRGYYNQSEDGSHTIQ

IMYGCDVGPDGRFLRGYRQDAYDGKDYIALNEDLRSWTAADMAAQITKRKWEAAHAAEQQ

RAYLEGRCVEWLRRYLENGKETLQRTDPPKTHMTHHPISDHEATLRCWALGFYPAEITLT

WQRDGEDQTQDTELVETRPAGDGTFQKWAAVVVPSGEEQRYTCHVQHEGLPKPLTLRWEL

SSQPTIPIVGIIAGLVLLGAVITGAVVAAVMWRRKSSDRKGGSYTQAASSDSAQGSDVSL

TACKV

>sp|P04075|ALDOA_HUMAN 25 APGKGILAADESTGSIA

MPYQYPALTPEQKKELSDIAHRIVAPGKGILAADESTGSIAKRLQSIGTENTEENRRFYR

QLLLTADDRVNPCIGGVILFHETLYQKADDGRPFPQVIKSKGGVVGIKVDKGVVPLAGTN

GETTTQGLDGLSERCAQYKKDGADFAKWRCVLKIGEHTPSALAIMENANVLARYASICQQ

NGIVPIVEPEILPDGDHDLKRCQYVTEKVLAAVYKALSDHHIYLEGTLLKPNMVTPGHAC

TQKFSHEEIAMATVTALRRTVPPAVTGITFLSGGQSEEEASINLNAINKCPLLKPWALTF

SYGRALQASALKAWGGKKENLKAAQEEYVKRALANSLACQGKYTPSGQAGAAASESLFVS

NHAY

>sp|Q31612|1B73_HUMAN 29 MRYFHTSVSRPGRGEP

MLVMAPRTVLLLLSAALALTETWAGSHSMRYFHTSVSRPGRGEPRFITVGYVDDTQFVRF

DSDAASPREEPRAPWIEQEGPEYWDRNTQICKAKAQTDRVGLRNLRGYYNQSEDGSHTWQ

TMYGCDMGPDGRLLRGYNQFAYDGKDYIALNEDLRSWTAADTAAQITQRKWEAARVAEQL

RAYLEGECVEWLRRHLENGKETLQRADPPKTHVTHHPISDHEATLRCWALGFYPAEITLT

WQRDGEDQTQDTELVETRPAGDGTFQKWAAVVVPSGQEQRYTCHVQHEGLQEPCTLRWKP

SSQSTIPIVGIVAGLAVLVVTVAVVAVVAAVMCRRKSSGGKGGSYSQAASSDSAQGSDVS

LTA

>sp|Q29718|1B82_HUMAN 143 DGKDYIALNEDLSS

MRVTAPRTLLLLLWGALALTETWAGSHSMRYFYTAMSRPGRGEPRFISVGYVDDTQFVRF

DSDAASPREEPRAPWIEQEGPEYWDRNTQIYKAQAQTDRESLRNLRGYYNQSEAGSHTLQ

RMFGCDLGPDGRLLRGHNQLAYDGKDYIALNEDLSSWTAADTAAQITQRKWEAARVAEQD

RAYLEDLCVESLRRYLENGKETLQRADPPKTHVTHHPISDHEATLRCWALGFYPAEITLT

WQRDGEDQTQDTELVETRPAGDRTFQKWAAVVVPSGEEQRYTCHVQHEGLPKPLTLRWEP

SSQSTIPIVGIVAGLAVLAVVVIGAVVATVMCRRKSSGGKGGSYSQAASSDSAQGSDVSL

TA

>sp|Q29718|1B82_HUMAN 153 DLSSWTAADTAAQ

MRVTAPRTLLLLLWGALALTETWAGSHSMRYFYTAMSRPGRGEPRFISVGYVDDTQFVRF

DSDAASPREEPRAPWIEQEGPEYWDRNTQIYKAQAQTDRESLRNLRGYYNQSEAGSHTLQ

RMFGCDLGPDGRLLRGHNQLAYDGKDYIALNEDLSSWTAADTAAQITQRKWEAARVAEQD

RAYLEDLCVESLRRYLENGKETLQRADPPKTHVTHHPISDHEATLRCWALGFYPAEITLT

WQRDGEDQTQDTELVETRPAGDRTFQKWAAVVVPSGEEQRYTCHVQHEGLPKPLTLRWEP

SSQSTIPIVGIVAGLAVLAVVVIGAVVATVMCRRKSSGGKGGSYSQAASSDSAQGSDVSL

TA

>sp|Q29718|1B82_HUMAN 153 DLSSWTAADTAAQIT

MRVTAPRTLLLLLWGALALTETWAGSHSMRYFYTAMSRPGRGEPRFISVGYVDDTQFVRF

DSDAASPREEPRAPWIEQEGPEYWDRNTQIYKAQAQTDRESLRNLRGYYNQSEAGSHTLQ

RMFGCDLGPDGRLLRGHNQLAYDGKDYIALNEDLSSWTAADTAAQITQRKWEAARVAEQD

RAYLEDLCVESLRRYLENGKETLQRADPPKTHVTHHPISDHEATLRCWALGFYPAEITLT

WQRDGEDQTQDTELVETRPAGDRTFQKWAAVVVPSGEEQRYTCHVQHEGLPKPLTLRWEP

SSQSTIPIVGIVAGLAVLAVVVIGAVVATVMCRRKSSGGKGGSYSQAASSDSAQGSDVSL

TA

>sp|Q29718|1B82_HUMAN 153 DLSSWTAADTAAQITQ

MRVTAPRTLLLLLWGALALTETWAGSHSMRYFYTAMSRPGRGEPRFISVGYVDDTQFVRF

DSDAASPREEPRAPWIEQEGPEYWDRNTQIYKAQAQTDRESLRNLRGYYNQSEAGSHTLQ

RMFGCDLGPDGRLLRGHNQLAYDGKDYIALNEDLSSWTAADTAAQITQRKWEAARVAEQD

RAYLEDLCVESLRRYLENGKETLQRADPPKTHVTHHPISDHEATLRCWALGFYPAEITLT

WQRDGEDQTQDTELVETRPAGDRTFQKWAAVVVPSGEEQRYTCHVQHEGLPKPLTLRWEP

SSQSTIPIVGIVAGLAVLAVVVIGAVVATVMCRRKSSGGKGGSYSQAASSDSAQGSDVSL

TA

>sp|Q29718|1B82_HUMAN 153 DLSSWTAADTAAQITQR

MRVTAPRTLLLLLWGALALTETWAGSHSMRYFYTAMSRPGRGEPRFISVGYVDDTQFVRF

DSDAASPREEPRAPWIEQEGPEYWDRNTQIYKAQAQTDRESLRNLRGYYNQSEAGSHTLQ

RMFGCDLGPDGRLLRGHNQLAYDGKDYIALNEDLSSWTAADTAAQITQRKWEAARVAEQD

RAYLEDLCVESLRRYLENGKETLQRADPPKTHVTHHPISDHEATLRCWALGFYPAEITLT

WQRDGEDQTQDTELVETRPAGDRTFQKWAAVVVPSGEEQRYTCHVQHEGLPKPLTLRWEP

SSQSTIPIVGIVAGLAVLAVVVIGAVVATVMCRRKSSGGKGGSYSQAASSDSAQGSDVSL

TA

>sp|Q29718|1B82_HUMAN 153 DLSSWTAADTAAQITQRK

MRVTAPRTLLLLLWGALALTETWAGSHSMRYFYTAMSRPGRGEPRFISVGYVDDTQFVRF

DSDAASPREEPRAPWIEQEGPEYWDRNTQIYKAQAQTDRESLRNLRGYYNQSEAGSHTLQ

RMFGCDLGPDGRLLRGHNQLAYDGKDYIALNEDLSSWTAADTAAQITQRKWEAARVAEQD

RAYLEDLCVESLRRYLENGKETLQRADPPKTHVTHHPISDHEATLRCWALGFYPAEITLT

WQRDGEDQTQDTELVETRPAGDRTFQKWAAVVVPSGEEQRYTCHVQHEGLPKPLTLRWEP

SSQSTIPIVGIVAGLAVLAVVVIGAVVATVMCRRKSSGGKGGSYSQAASSDSAQGSDVSL

TA

>sp|Q29718|1B82_HUMAN 153 DLSSWTAADTAAQITQRKWEAA

MRVTAPRTLLLLLWGALALTETWAGSHSMRYFYTAMSRPGRGEPRFISVGYVDDTQFVRF

DSDAASPREEPRAPWIEQEGPEYWDRNTQIYKAQAQTDRESLRNLRGYYNQSEAGSHTLQ

RMFGCDLGPDGRLLRGHNQLAYDGKDYIALNEDLSSWTAADTAAQITQRKWEAARVAEQD

RAYLEDLCVESLRRYLENGKETLQRADPPKTHVTHHPISDHEATLRCWALGFYPAEITLT

WQRDGEDQTQDTELVETRPAGDRTFQKWAAVVVPSGEEQRYTCHVQHEGLPKPLTLRWEP

SSQSTIPIVGIVAGLAVLAVVVIGAVVATVMCRRKSSGGKGGSYSQAASSDSAQGSDVSL

TA

>sp|Q29718|1B82_HUMAN 152 EDLSSWTAADTAAQ

MRVTAPRTLLLLLWGALALTETWAGSHSMRYFYTAMSRPGRGEPRFISVGYVDDTQFVRF

DSDAASPREEPRAPWIEQEGPEYWDRNTQIYKAQAQTDRESLRNLRGYYNQSEAGSHTLQ

RMFGCDLGPDGRLLRGHNQLAYDGKDYIALNEDLSSWTAADTAAQITQRKWEAARVAEQD

RAYLEDLCVESLRRYLENGKETLQRADPPKTHVTHHPISDHEATLRCWALGFYPAEITLT

WQRDGEDQTQDTELVETRPAGDRTFQKWAAVVVPSGEEQRYTCHVQHEGLPKPLTLRWEP

SSQSTIPIVGIVAGLAVLAVVVIGAVVATVMCRRKSSGGKGGSYSQAASSDSAQGSDVSL

TA

>sp|Q29718|1B82_HUMAN 152 EDLSSWTAADTAAQIT

MRVTAPRTLLLLLWGALALTETWAGSHSMRYFYTAMSRPGRGEPRFISVGYVDDTQFVRF

DSDAASPREEPRAPWIEQEGPEYWDRNTQIYKAQAQTDRESLRNLRGYYNQSEAGSHTLQ

RMFGCDLGPDGRLLRGHNQLAYDGKDYIALNEDLSSWTAADTAAQITQRKWEAARVAEQD

RAYLEDLCVESLRRYLENGKETLQRADPPKTHVTHHPISDHEATLRCWALGFYPAEITLT

WQRDGEDQTQDTELVETRPAGDRTFQKWAAVVVPSGEEQRYTCHVQHEGLPKPLTLRWEP

SSQSTIPIVGIVAGLAVLAVVVIGAVVATVMCRRKSSGGKGGSYSQAASSDSAQGSDVSL

TA

>sp|Q29718|1B82_HUMAN 152 EDLSSWTAADTAAQITQ

MRVTAPRTLLLLLWGALALTETWAGSHSMRYFYTAMSRPGRGEPRFISVGYVDDTQFVRF

DSDAASPREEPRAPWIEQEGPEYWDRNTQIYKAQAQTDRESLRNLRGYYNQSEAGSHTLQ

RMFGCDLGPDGRLLRGHNQLAYDGKDYIALNEDLSSWTAADTAAQITQRKWEAARVAEQD

RAYLEDLCVESLRRYLENGKETLQRADPPKTHVTHHPISDHEATLRCWALGFYPAEITLT

WQRDGEDQTQDTELVETRPAGDRTFQKWAAVVVPSGEEQRYTCHVQHEGLPKPLTLRWEP

SSQSTIPIVGIVAGLAVLAVVVIGAVVATVMCRRKSSGGKGGSYSQAASSDSAQGSDVSL

TA

>sp|Q29718|1B82_HUMAN 152 EDLSSWTAADTAAQITQR

MRVTAPRTLLLLLWGALALTETWAGSHSMRYFYTAMSRPGRGEPRFISVGYVDDTQFVRF

DSDAASPREEPRAPWIEQEGPEYWDRNTQIYKAQAQTDRESLRNLRGYYNQSEAGSHTLQ

RMFGCDLGPDGRLLRGHNQLAYDGKDYIALNEDLSSWTAADTAAQITQRKWEAARVAEQD

RAYLEDLCVESLRRYLENGKETLQRADPPKTHVTHHPISDHEATLRCWALGFYPAEITLT

WQRDGEDQTQDTELVETRPAGDRTFQKWAAVVVPSGEEQRYTCHVQHEGLPKPLTLRWEP

SSQSTIPIVGIVAGLAVLAVVVIGAVVATVMCRRKSSGGKGGSYSQAASSDSAQGSDVSL

TA

>sp|Q29718|1B82_HUMAN 152 EDLSSWTAADTAAQITQRK

MRVTAPRTLLLLLWGALALTETWAGSHSMRYFYTAMSRPGRGEPRFISVGYVDDTQFVRF

DSDAASPREEPRAPWIEQEGPEYWDRNTQIYKAQAQTDRESLRNLRGYYNQSEAGSHTLQ

RMFGCDLGPDGRLLRGHNQLAYDGKDYIALNEDLSSWTAADTAAQITQRKWEAARVAEQD

RAYLEDLCVESLRRYLENGKETLQRADPPKTHVTHHPISDHEATLRCWALGFYPAEITLT

WQRDGEDQTQDTELVETRPAGDRTFQKWAAVVVPSGEEQRYTCHVQHEGLPKPLTLRWEP

SSQSTIPIVGIVAGLAVLAVVVIGAVVATVMCRRKSSGGKGGSYSQAASSDSAQGSDVSL

TA

>sp|Q29718|1B82_HUMAN 145 KDYIALNEDLSSWTA

MRVTAPRTLLLLLWGALALTETWAGSHSMRYFYTAMSRPGRGEPRFISVGYVDDTQFVRF

DSDAASPREEPRAPWIEQEGPEYWDRNTQIYKAQAQTDRESLRNLRGYYNQSEAGSHTLQ

RMFGCDLGPDGRLLRGHNQLAYDGKDYIALNEDLSSWTAADTAAQITQRKWEAARVAEQD

RAYLEDLCVESLRRYLENGKETLQRADPPKTHVTHHPISDHEATLRCWALGFYPAEITLT

WQRDGEDQTQDTELVETRPAGDRTFQKWAAVVVPSGEEQRYTCHVQHEGLPKPLTLRWEP

SSQSTIPIVGIVAGLAVLAVVVIGAVVATVMCRRKSSGGKGGSYSQAASSDSAQGSDVSL

TA

>sp|Q29718|1B82_HUMAN 150 LNEDLSSWTAADTAAQITQ

MRVTAPRTLLLLLWGALALTETWAGSHSMRYFYTAMSRPGRGEPRFISVGYVDDTQFVRF

DSDAASPREEPRAPWIEQEGPEYWDRNTQIYKAQAQTDRESLRNLRGYYNQSEAGSHTLQ

RMFGCDLGPDGRLLRGHNQLAYDGKDYIALNEDLSSWTAADTAAQITQRKWEAARVAEQD

RAYLEDLCVESLRRYLENGKETLQRADPPKTHVTHHPISDHEATLRCWALGFYPAEITLT

WQRDGEDQTQDTELVETRPAGDRTFQKWAAVVVPSGEEQRYTCHVQHEGLPKPLTLRWEP

SSQSTIPIVGIVAGLAVLAVVVIGAVVATVMCRRKSSGGKGGSYSQAASSDSAQGSDVSL

TA

>sp|Q29718|1B82_HUMAN 150 LNEDLSSWTAADTAAQITQRKWEAARVA

MRVTAPRTLLLLLWGALALTETWAGSHSMRYFYTAMSRPGRGEPRFISVGYVDDTQFVRF

DSDAASPREEPRAPWIEQEGPEYWDRNTQIYKAQAQTDRESLRNLRGYYNQSEAGSHTLQ

RMFGCDLGPDGRLLRGHNQLAYDGKDYIALNEDLSSWTAADTAAQITQRKWEAARVAEQD

RAYLEDLCVESLRRYLENGKETLQRADPPKTHVTHHPISDHEATLRCWALGFYPAEITLT

WQRDGEDQTQDTELVETRPAGDRTFQKWAAVVVPSGEEQRYTCHVQHEGLPKPLTLRWEP

SSQSTIPIVGIVAGLAVLAVVVIGAVVATVMCRRKSSGGKGGSYSQAASSDSAQGSDVSL

TA

>sp|Q29718|1B82_HUMAN 154 LSSWTAADTAAQIT

MRVTAPRTLLLLLWGALALTETWAGSHSMRYFYTAMSRPGRGEPRFISVGYVDDTQFVRF

DSDAASPREEPRAPWIEQEGPEYWDRNTQIYKAQAQTDRESLRNLRGYYNQSEAGSHTLQ

RMFGCDLGPDGRLLRGHNQLAYDGKDYIALNEDLSSWTAADTAAQITQRKWEAARVAEQD

RAYLEDLCVESLRRYLENGKETLQRADPPKTHVTHHPISDHEATLRCWALGFYPAEITLT

WQRDGEDQTQDTELVETRPAGDRTFQKWAAVVVPSGEEQRYTCHVQHEGLPKPLTLRWEP

SSQSTIPIVGIVAGLAVLAVVVIGAVVATVMCRRKSSGGKGGSYSQAASSDSAQGSDVSL

TA

>sp|Q29718|1B82_HUMAN 154 LSSWTAADTAAQITQ

MRVTAPRTLLLLLWGALALTETWAGSHSMRYFYTAMSRPGRGEPRFISVGYVDDTQFVRF

DSDAASPREEPRAPWIEQEGPEYWDRNTQIYKAQAQTDRESLRNLRGYYNQSEAGSHTLQ

RMFGCDLGPDGRLLRGHNQLAYDGKDYIALNEDLSSWTAADTAAQITQRKWEAARVAEQD

RAYLEDLCVESLRRYLENGKETLQRADPPKTHVTHHPISDHEATLRCWALGFYPAEITLT

WQRDGEDQTQDTELVETRPAGDRTFQKWAAVVVPSGEEQRYTCHVQHEGLPKPLTLRWEP

SSQSTIPIVGIVAGLAVLAVVVIGAVVATVMCRRKSSGGKGGSYSQAASSDSAQGSDVSL

TA

>sp|Q29718|1B82_HUMAN 154 LSSWTAADTAAQITQR

MRVTAPRTLLLLLWGALALTETWAGSHSMRYFYTAMSRPGRGEPRFISVGYVDDTQFVRF

DSDAASPREEPRAPWIEQEGPEYWDRNTQIYKAQAQTDRESLRNLRGYYNQSEAGSHTLQ

RMFGCDLGPDGRLLRGHNQLAYDGKDYIALNEDLSSWTAADTAAQITQRKWEAARVAEQD

RAYLEDLCVESLRRYLENGKETLQRADPPKTHVTHHPISDHEATLRCWALGFYPAEITLT

WQRDGEDQTQDTELVETRPAGDRTFQKWAAVVVPSGEEQRYTCHVQHEGLPKPLTLRWEP

SSQSTIPIVGIVAGLAVLAVVVIGAVVATVMCRRKSSGGKGGSYSQAASSDSAQGSDVSL

TA

>sp|Q29718|1B82_HUMAN 151 NEDLSSWTAADTAAQITQ

MRVTAPRTLLLLLWGALALTETWAGSHSMRYFYTAMSRPGRGEPRFISVGYVDDTQFVRF

DSDAASPREEPRAPWIEQEGPEYWDRNTQIYKAQAQTDRESLRNLRGYYNQSEAGSHTLQ

RMFGCDLGPDGRLLRGHNQLAYDGKDYIALNEDLSSWTAADTAAQITQRKWEAARVAEQD

RAYLEDLCVESLRRYLENGKETLQRADPPKTHVTHHPISDHEATLRCWALGFYPAEITLT

WQRDGEDQTQDTELVETRPAGDRTFQKWAAVVVPSGEEQRYTCHVQHEGLPKPLTLRWEP

SSQSTIPIVGIVAGLAVLAVVVIGAVVATVMCRRKSSGGKGGSYSQAASSDSAQGSDVSL

TA

>sp|Q29718|1B82_HUMAN 30 RYFYTAMSRPGRGEPRFI

MRVTAPRTLLLLLWGALALTETWAGSHSMRYFYTAMSRPGRGEPRFISVGYVDDTQFVRF

DSDAASPREEPRAPWIEQEGPEYWDRNTQIYKAQAQTDRESLRNLRGYYNQSEAGSHTLQ

RMFGCDLGPDGRLLRGHNQLAYDGKDYIALNEDLSSWTAADTAAQITQRKWEAARVAEQD

RAYLEDLCVESLRRYLENGKETLQRADPPKTHVTHHPISDHEATLRCWALGFYPAEITLT

WQRDGEDQTQDTELVETRPAGDRTFQKWAAVVVPSGEEQRYTCHVQHEGLPKPLTLRWEP

SSQSTIPIVGIVAGLAVLAVVVIGAVVATVMCRRKSSGGKGGSYSQAASSDSAQGSDVSL

TA

>sp|Q29718|1B82_HUMAN 155 SSWTAADTAAQITQ

MRVTAPRTLLLLLWGALALTETWAGSHSMRYFYTAMSRPGRGEPRFISVGYVDDTQFVRF

DSDAASPREEPRAPWIEQEGPEYWDRNTQIYKAQAQTDRESLRNLRGYYNQSEAGSHTLQ

RMFGCDLGPDGRLLRGHNQLAYDGKDYIALNEDLSSWTAADTAAQITQRKWEAARVAEQD

RAYLEDLCVESLRRYLENGKETLQRADPPKTHVTHHPISDHEATLRCWALGFYPAEITLT

WQRDGEDQTQDTELVETRPAGDRTFQKWAAVVVPSGEEQRYTCHVQHEGLPKPLTLRWEP

SSQSTIPIVGIVAGLAVLAVVVIGAVVATVMCRRKSSGGKGGSYSQAASSDSAQGSDVSL

TA

>sp|Q31610|1B81_HUMAN 128 GPDGRLLRGHNQYAYD

MLVMAPRTVLLLLWGAVALTETWAGSHSMRYFYTSVSRPGRGEPRFISVGYVDDTQFVRF

DSDAASPREEPRAPWIEQEGPEYWDRNTQIYKAQAQTDRESLRNLRGYYNQSEAGSHTLQ

SMYGCDVGPDGRLLRGHNQYAYDGKDYIALNEDLRSWTAADTAAQISQRKLEAARVAEQL

RAYLEGECVEWLRRYLENGKDKLERADPPKTHVTHHPISDHEATLRCWALGFYPAEITLT

WQRDGEDQTQDTELVETRPAGDRTFQKWTAVVVPSGEEQRYTCHVQHEGLPKPLTLRWEP

SSQSTVPIVGIVAGLAVLAVVVIGAVVAAVMCRRKSSGGKGGSYSQAACSDSAQGSDVSL

TA

>sp|Q31610|1B81_HUMAN 296 LRWEPSSQSTVPIVGIVAG

MLVMAPRTVLLLLWGAVALTETWAGSHSMRYFYTSVSRPGRGEPRFISVGYVDDTQFVRF

DSDAASPREEPRAPWIEQEGPEYWDRNTQIYKAQAQTDRESLRNLRGYYNQSEAGSHTLQ

SMYGCDVGPDGRLLRGHNQYAYDGKDYIALNEDLRSWTAADTAAQISQRKLEAARVAEQL

RAYLEGECVEWLRRYLENGKDKLERADPPKTHVTHHPISDHEATLRCWALGFYPAEITLT

WQRDGEDQTQDTELVETRPAGDRTFQKWTAVVVPSGEEQRYTCHVQHEGLPKPLTLRWEP

SSQSTVPIVGIVAGLAVLAVVVIGAVVAAVMCRRKSSGGKGGSYSQAACSDSAQGSDVSL

TA

>sp|P30498|1B78_HUMAN 54 DTQFVRFDSDAASPRTEP

MRVTAPRTVLLLLWGAVALTETWAGSHSMRYFYTAMSRPGRGEPRFIAVGYVDDTQFVRF

DSDAASPRTEPRAPWIEQEGPEYWDRNTQIFKTNTQTDRESLRNLRGYYNQSEAGSHTWQ

TMYGCDVGPDGRLLRGHNQYAYDGKDYIALNEDLSSWTAADTAAQITQRKWEAAREAEQL

RAYLEGLCVEWLRRHLENGKETLQRADPPKTHVTHHPVSDHEATLRCWALGFYPAEITLT

WQRDGEDQTQDTELVETRPAGDRTFQKWAAVVVPSGEEQRYTCHVQHEGLPKPLTLRWEP

SSQSTIPIVGIVAGLAVLAVVVIGAVVATVMCRRKSSGGKGGSYSQAASSDSAQGSDVSL

TA

>sp|P30498|1B78_HUMAN 52 VDDTQFVRFDSDAASPRTEP

MRVTAPRTVLLLLWGAVALTETWAGSHSMRYFYTAMSRPGRGEPRFIAVGYVDDTQFVRF

DSDAASPRTEPRAPWIEQEGPEYWDRNTQIFKTNTQTDRESLRNLRGYYNQSEAGSHTWQ

TMYGCDVGPDGRLLRGHNQYAYDGKDYIALNEDLSSWTAADTAAQITQRKWEAAREAEQL

RAYLEGLCVEWLRRHLENGKETLQRADPPKTHVTHHPVSDHEATLRCWALGFYPAEITLT

WQRDGEDQTQDTELVETRPAGDRTFQKWAAVVVPSGEEQRYTCHVQHEGLPKPLTLRWEP

SSQSTIPIVGIVAGLAVLAVVVIGAVVATVMCRRKSSGGKGGSYSQAASSDSAQGSDVSL

TA

>sp|P30498|1B78_HUMAN 52 VDDTQFVRFDSDAASPRTEPR

MRVTAPRTVLLLLWGAVALTETWAGSHSMRYFYTAMSRPGRGEPRFIAVGYVDDTQFVRF

DSDAASPRTEPRAPWIEQEGPEYWDRNTQIFKTNTQTDRESLRNLRGYYNQSEAGSHTWQ

TMYGCDVGPDGRLLRGHNQYAYDGKDYIALNEDLSSWTAADTAAQITQRKWEAAREAEQL

RAYLEGLCVEWLRRHLENGKETLQRADPPKTHVTHHPVSDHEATLRCWALGFYPAEITLT

WQRDGEDQTQDTELVETRPAGDRTFQKWAAVVVPSGEEQRYTCHVQHEGLPKPLTLRWEP

SSQSTIPIVGIVAGLAVLAVVVIGAVVATVMCRRKSSGGKGGSYSQAASSDSAQGSDVSL

TA

>sp|Q29836|1B67_HUMAN 128 GPDGRLLRGHNQFAYD

MLVMAPRTVLLLLSAALALTETWAGSHSMRYFYTSVSRPGRGEPRFISVGYVDDTQFVRF

DSDAASPREEPRAPWIEQEGPEYWDRNTQIYKAQAQTDRESLRNLRGYYNQSEAGSHTLQ

RMYGCDVGPDGRLLRGHNQFAYDGKDYIALNEDLSSWTAADTAAQITQRKWEAARVAEQL

RTYLEGTCVEWLRRYLENGKETLQRADPPKTHVTHHPISDHEATLRCWALGFYPAEITLT

WQRDGEDQTQDTELVETRPAGDRTFQKWAAVVVPSGEEQRYTCHVQHEGLPKPLTLRWEP

SSQSTVPIVGIVAGLAVLAVVVIGAVVAAVMCRRKSSGGKGGSYSQAASSDSAQGSDVSL

TA

>sp|Q29836|1B67_HUMAN 128 GPDGRLLRGHNQFAYDG

MLVMAPRTVLLLLSAALALTETWAGSHSMRYFYTSVSRPGRGEPRFISVGYVDDTQFVRF

DSDAASPREEPRAPWIEQEGPEYWDRNTQIYKAQAQTDRESLRNLRGYYNQSEAGSHTLQ

RMYGCDVGPDGRLLRGHNQFAYDGKDYIALNEDLSSWTAADTAAQITQRKWEAARVAEQL

RTYLEGTCVEWLRRYLENGKETLQRADPPKTHVTHHPISDHEATLRCWALGFYPAEITLT

WQRDGEDQTQDTELVETRPAGDRTFQKWAAVVVPSGEEQRYTCHVQHEGLPKPLTLRWEP

SSQSTVPIVGIVAGLAVLAVVVIGAVVAAVMCRRKSSGGKGGSYSQAASSDSAQGSDVSL

TA

>sp|Q29836|1B67_HUMAN 128 GPDGRLLRGHNQFAYDGK

MLVMAPRTVLLLLSAALALTETWAGSHSMRYFYTSVSRPGRGEPRFISVGYVDDTQFVRF

DSDAASPREEPRAPWIEQEGPEYWDRNTQIYKAQAQTDRESLRNLRGYYNQSEAGSHTLQ

RMYGCDVGPDGRLLRGHNQFAYDGKDYIALNEDLSSWTAADTAAQITQRKWEAARVAEQL

RTYLEGTCVEWLRRYLENGKETLQRADPPKTHVTHHPISDHEATLRCWALGFYPAEITLT

WQRDGEDQTQDTELVETRPAGDRTFQKWAAVVVPSGEEQRYTCHVQHEGLPKPLTLRWEP

SSQSTVPIVGIVAGLAVLAVVVIGAVVAAVMCRRKSSGGKGGSYSQAASSDSAQGSDVSL

TA

>sp|Q29836|1B67_HUMAN 128 GPDGRLLRGHNQFAYDGKD

MLVMAPRTVLLLLSAALALTETWAGSHSMRYFYTSVSRPGRGEPRFISVGYVDDTQFVRF

DSDAASPREEPRAPWIEQEGPEYWDRNTQIYKAQAQTDRESLRNLRGYYNQSEAGSHTLQ

RMYGCDVGPDGRLLRGHNQFAYDGKDYIALNEDLSSWTAADTAAQITQRKWEAARVAEQL

RTYLEGTCVEWLRRYLENGKETLQRADPPKTHVTHHPISDHEATLRCWALGFYPAEITLT

WQRDGEDQTQDTELVETRPAGDRTFQKWAAVVVPSGEEQRYTCHVQHEGLPKPLTLRWEP

SSQSTVPIVGIVAGLAVLAVVVIGAVVAAVMCRRKSSGGKGGSYSQAASSDSAQGSDVSL

TA

>sp|Q29836|1B67_HUMAN 128 GPDGRLLRGHNQFAYDGKDY

MLVMAPRTVLLLLSAALALTETWAGSHSMRYFYTSVSRPGRGEPRFISVGYVDDTQFVRF

DSDAASPREEPRAPWIEQEGPEYWDRNTQIYKAQAQTDRESLRNLRGYYNQSEAGSHTLQ

RMYGCDVGPDGRLLRGHNQFAYDGKDYIALNEDLSSWTAADTAAQITQRKWEAARVAEQL

RTYLEGTCVEWLRRYLENGKETLQRADPPKTHVTHHPISDHEATLRCWALGFYPAEITLT

WQRDGEDQTQDTELVETRPAGDRTFQKWAAVVVPSGEEQRYTCHVQHEGLPKPLTLRWEP

SSQSTVPIVGIVAGLAVLAVVVIGAVVAAVMCRRKSSGGKGGSYSQAASSDSAQGSDVSL

TA

>sp|Q29836|1B67_HUMAN 131 GRLLRGHNQFAYDGK

MLVMAPRTVLLLLSAALALTETWAGSHSMRYFYTSVSRPGRGEPRFISVGYVDDTQFVRF

DSDAASPREEPRAPWIEQEGPEYWDRNTQIYKAQAQTDRESLRNLRGYYNQSEAGSHTLQ

RMYGCDVGPDGRLLRGHNQFAYDGKDYIALNEDLSSWTAADTAAQITQRKWEAARVAEQL

RTYLEGTCVEWLRRYLENGKETLQRADPPKTHVTHHPISDHEATLRCWALGFYPAEITLT

WQRDGEDQTQDTELVETRPAGDRTFQKWAAVVVPSGEEQRYTCHVQHEGLPKPLTLRWEP

SSQSTVPIVGIVAGLAVLAVVVIGAVVAAVMCRRKSSGGKGGSYSQAASSDSAQGSDVSL

TA

>sp|Q29836|1B67_HUMAN 127 VGPDGRLLRGHNQFAYD

MLVMAPRTVLLLLSAALALTETWAGSHSMRYFYTSVSRPGRGEPRFISVGYVDDTQFVRF

DSDAASPREEPRAPWIEQEGPEYWDRNTQIYKAQAQTDRESLRNLRGYYNQSEAGSHTLQ

RMYGCDVGPDGRLLRGHNQFAYDGKDYIALNEDLSSWTAADTAAQITQRKWEAARVAEQL

RTYLEGTCVEWLRRYLENGKETLQRADPPKTHVTHHPISDHEATLRCWALGFYPAEITLT

WQRDGEDQTQDTELVETRPAGDRTFQKWAAVVVPSGEEQRYTCHVQHEGLPKPLTLRWEP

SSQSTVPIVGIVAGLAVLAVVVIGAVVAAVMCRRKSSGGKGGSYSQAASSDSAQGSDVSL

TA

>sp|P18465|1B57_HUMAN 53 DDTQFVRFDSDAASPRM

MRVTAPRTVLLLLWGAVALTETWAGSHSMRYFYTAMSRPGRGEPRFIAVGYVDDTQFVRF

DSDAASPRMAPRAPWIEQEGPEYWDGETRNMKASAQTYRENLRIALRYYNQSEAGSHIIQ

VMYGCDVGPDGRLLRGHDQSAYDGKDYIALNEDLSSWTAADTAAQITQRKWEAARVAEQL

RAYLEGLCVEWLRRYLENGKETLQRADPPKTHVTHHPISDHEATLRCWALGFYPAEITLT

WQRDGEDQTQDTELVETRPAGDRTFQKWAAVVVPSGEEQRYTCHVQHEGLPKPLTLRWEP

SSQSTVPIVGIVAGLAVLAVVVIGAVVAAVMCRRKSSGGKGGSYSQAACSDSAQGSDVSL

TA

>sp|P18465|1B57_HUMAN 53 DDTQFVRFDSDAASPRMA

MRVTAPRTVLLLLWGAVALTETWAGSHSMRYFYTAMSRPGRGEPRFIAVGYVDDTQFVRF

DSDAASPRMAPRAPWIEQEGPEYWDGETRNMKASAQTYRENLRIALRYYNQSEAGSHIIQ

VMYGCDVGPDGRLLRGHDQSAYDGKDYIALNEDLSSWTAADTAAQITQRKWEAARVAEQL

RAYLEGLCVEWLRRYLENGKETLQRADPPKTHVTHHPISDHEATLRCWALGFYPAEITLT

WQRDGEDQTQDTELVETRPAGDRTFQKWAAVVVPSGEEQRYTCHVQHEGLPKPLTLRWEP

SSQSTVPIVGIVAGLAVLAVVVIGAVVAAVMCRRKSSGGKGGSYSQAACSDSAQGSDVSL

TA

>sp|P18465|1B57_HUMAN 53 DDTQFVRFDSDAASPRMAP

MRVTAPRTVLLLLWGAVALTETWAGSHSMRYFYTAMSRPGRGEPRFIAVGYVDDTQFVRF

DSDAASPRMAPRAPWIEQEGPEYWDGETRNMKASAQTYRENLRIALRYYNQSEAGSHIIQ

VMYGCDVGPDGRLLRGHDQSAYDGKDYIALNEDLSSWTAADTAAQITQRKWEAARVAEQL

RAYLEGLCVEWLRRYLENGKETLQRADPPKTHVTHHPISDHEATLRCWALGFYPAEITLT

WQRDGEDQTQDTELVETRPAGDRTFQKWAAVVVPSGEEQRYTCHVQHEGLPKPLTLRWEP

SSQSTVPIVGIVAGLAVLAVVVIGAVVAAVMCRRKSSGGKGGSYSQAACSDSAQGSDVSL

TA

>sp|P18465|1B57_HUMAN 53 DDTQFVRFDSDAASPRMAPR

MRVTAPRTVLLLLWGAVALTETWAGSHSMRYFYTAMSRPGRGEPRFIAVGYVDDTQFVRF

DSDAASPRMAPRAPWIEQEGPEYWDGETRNMKASAQTYRENLRIALRYYNQSEAGSHIIQ

VMYGCDVGPDGRLLRGHDQSAYDGKDYIALNEDLSSWTAADTAAQITQRKWEAARVAEQL

RAYLEGLCVEWLRRYLENGKETLQRADPPKTHVTHHPISDHEATLRCWALGFYPAEITLT

WQRDGEDQTQDTELVETRPAGDRTFQKWAAVVVPSGEEQRYTCHVQHEGLPKPLTLRWEP

SSQSTVPIVGIVAGLAVLAVVVIGAVVAAVMCRRKSSGGKGGSYSQAACSDSAQGSDVSL

TA

>sp|P18465|1B57_HUMAN 54 DTQFVRFDSDAASPRM

MRVTAPRTVLLLLWGAVALTETWAGSHSMRYFYTAMSRPGRGEPRFIAVGYVDDTQFVRF

DSDAASPRMAPRAPWIEQEGPEYWDGETRNMKASAQTYRENLRIALRYYNQSEAGSHIIQ

VMYGCDVGPDGRLLRGHDQSAYDGKDYIALNEDLSSWTAADTAAQITQRKWEAARVAEQL

RAYLEGLCVEWLRRYLENGKETLQRADPPKTHVTHHPISDHEATLRCWALGFYPAEITLT

WQRDGEDQTQDTELVETRPAGDRTFQKWAAVVVPSGEEQRYTCHVQHEGLPKPLTLRWEP

SSQSTVPIVGIVAGLAVLAVVVIGAVVAAVMCRRKSSGGKGGSYSQAACSDSAQGSDVSL

TA

>sp|P18465|1B57_HUMAN 54 DTQFVRFDSDAASPRMA

MRVTAPRTVLLLLWGAVALTETWAGSHSMRYFYTAMSRPGRGEPRFIAVGYVDDTQFVRF

DSDAASPRMAPRAPWIEQEGPEYWDGETRNMKASAQTYRENLRIALRYYNQSEAGSHIIQ

VMYGCDVGPDGRLLRGHDQSAYDGKDYIALNEDLSSWTAADTAAQITQRKWEAARVAEQL

RAYLEGLCVEWLRRYLENGKETLQRADPPKTHVTHHPISDHEATLRCWALGFYPAEITLT

WQRDGEDQTQDTELVETRPAGDRTFQKWAAVVVPSGEEQRYTCHVQHEGLPKPLTLRWEP

SSQSTVPIVGIVAGLAVLAVVVIGAVVAAVMCRRKSSGGKGGSYSQAACSDSAQGSDVSL

TA

>sp|P18465|1B57_HUMAN 54 DTQFVRFDSDAASPRMAP

MRVTAPRTVLLLLWGAVALTETWAGSHSMRYFYTAMSRPGRGEPRFIAVGYVDDTQFVRF

DSDAASPRMAPRAPWIEQEGPEYWDGETRNMKASAQTYRENLRIALRYYNQSEAGSHIIQ

VMYGCDVGPDGRLLRGHDQSAYDGKDYIALNEDLSSWTAADTAAQITQRKWEAARVAEQL

RAYLEGLCVEWLRRYLENGKETLQRADPPKTHVTHHPISDHEATLRCWALGFYPAEITLT

WQRDGEDQTQDTELVETRPAGDRTFQKWAAVVVPSGEEQRYTCHVQHEGLPKPLTLRWEP

SSQSTVPIVGIVAGLAVLAVVVIGAVVAAVMCRRKSSGGKGGSYSQAACSDSAQGSDVSL

TA

>sp|P18465|1B57_HUMAN 54 DTQFVRFDSDAASPRMAPR

MRVTAPRTVLLLLWGAVALTETWAGSHSMRYFYTAMSRPGRGEPRFIAVGYVDDTQFVRF

DSDAASPRMAPRAPWIEQEGPEYWDGETRNMKASAQTYRENLRIALRYYNQSEAGSHIIQ

VMYGCDVGPDGRLLRGHDQSAYDGKDYIALNEDLSSWTAADTAAQITQRKWEAARVAEQL

RAYLEGLCVEWLRRYLENGKETLQRADPPKTHVTHHPISDHEATLRCWALGFYPAEITLT

WQRDGEDQTQDTELVETRPAGDRTFQKWAAVVVPSGEEQRYTCHVQHEGLPKPLTLRWEP

SSQSTVPIVGIVAGLAVLAVVVIGAVVAAVMCRRKSSGGKGGSYSQAACSDSAQGSDVSL

TA

>sp|P18465|1B57_HUMAN 52 VDDTQFVRFDSDAASPRM

MRVTAPRTVLLLLWGAVALTETWAGSHSMRYFYTAMSRPGRGEPRFIAVGYVDDTQFVRF

DSDAASPRMAPRAPWIEQEGPEYWDGETRNMKASAQTYRENLRIALRYYNQSEAGSHIIQ

VMYGCDVGPDGRLLRGHDQSAYDGKDYIALNEDLSSWTAADTAAQITQRKWEAARVAEQL

RAYLEGLCVEWLRRYLENGKETLQRADPPKTHVTHHPISDHEATLRCWALGFYPAEITLT

WQRDGEDQTQDTELVETRPAGDRTFQKWAAVVVPSGEEQRYTCHVQHEGLPKPLTLRWEP

SSQSTVPIVGIVAGLAVLAVVVIGAVVAAVMCRRKSSGGKGGSYSQAACSDSAQGSDVSL

TA

>sp|P18465|1B57_HUMAN 52 VDDTQFVRFDSDAASPRMA

MRVTAPRTVLLLLWGAVALTETWAGSHSMRYFYTAMSRPGRGEPRFIAVGYVDDTQFVRF

DSDAASPRMAPRAPWIEQEGPEYWDGETRNMKASAQTYRENLRIALRYYNQSEAGSHIIQ

VMYGCDVGPDGRLLRGHDQSAYDGKDYIALNEDLSSWTAADTAAQITQRKWEAARVAEQL

RAYLEGLCVEWLRRYLENGKETLQRADPPKTHVTHHPISDHEATLRCWALGFYPAEITLT

WQRDGEDQTQDTELVETRPAGDRTFQKWAAVVVPSGEEQRYTCHVQHEGLPKPLTLRWEP

SSQSTVPIVGIVAGLAVLAVVVIGAVVAAVMCRRKSSGGKGGSYSQAACSDSAQGSDVSL

TA

>sp|P18465|1B57_HUMAN 52 VDDTQFVRFDSDAASPRMAP

MRVTAPRTVLLLLWGAVALTETWAGSHSMRYFYTAMSRPGRGEPRFIAVGYVDDTQFVRF

DSDAASPRMAPRAPWIEQEGPEYWDGETRNMKASAQTYRENLRIALRYYNQSEAGSHIIQ

VMYGCDVGPDGRLLRGHDQSAYDGKDYIALNEDLSSWTAADTAAQITQRKWEAARVAEQL

RAYLEGLCVEWLRRYLENGKETLQRADPPKTHVTHHPISDHEATLRCWALGFYPAEITLT

WQRDGEDQTQDTELVETRPAGDRTFQKWAAVVVPSGEEQRYTCHVQHEGLPKPLTLRWEP

SSQSTVPIVGIVAGLAVLAVVVIGAVVAAVMCRRKSSGGKGGSYSQAACSDSAQGSDVSL

TA

>sp|P18465|1B57_HUMAN 52 VDDTQFVRFDSDAASPRMAPR

MRVTAPRTVLLLLWGAVALTETWAGSHSMRYFYTAMSRPGRGEPRFIAVGYVDDTQFVRF

DSDAASPRMAPRAPWIEQEGPEYWDGETRNMKASAQTYRENLRIALRYYNQSEAGSHIIQ

VMYGCDVGPDGRLLRGHDQSAYDGKDYIALNEDLSSWTAADTAAQITQRKWEAARVAEQL

RAYLEGLCVEWLRRYLENGKETLQRADPPKTHVTHHPISDHEATLRCWALGFYPAEITLT

WQRDGEDQTQDTELVETRPAGDRTFQKWAAVVVPSGEEQRYTCHVQHEGLPKPLTLRWEP

SSQSTVPIVGIVAGLAVLAVVVIGAVVAAVMCRRKSSGGKGGSYSQAACSDSAQGSDVSL

TA

>sp|P30490|1B52_HUMAN 80 GPEYWDRETQISKTN

MRVTAPRTVLLLLWGAVALTETWAGSHSMRYFYTAMSRPGRGEPRFIAVGYVDDTQFVRF

DSDAASPRTEPRAPWIEQEGPEYWDRETQISKTNTQTYRENLRIALRYYNQSEAGSHTWQ

TMYGCDVGPDGRLLRGHNQYAYDGKDYIALNEDLSSWTAADTAAQITQRKWEAAREAEQL

RAYLEGLCVEWLRRHLENGKETLQRADPPKTHVTHHPVSDHEATLRCWALGFYPAEITLT

WQRDGEDQTQDTELVETRPAGDRTFQKWAAVVVPSGEEQRYTCHVQHEGLPKPLTLRWEP

SSQSTIPIVGIVAGLAVLAVVVIGAVVATVMCRRKSSGGKGGSYSQAASSDSAQGSDVSL

TA

>sp|P30490|1B52_HUMAN 86 RETQISKTNTQTYRE

MRVTAPRTVLLLLWGAVALTETWAGSHSMRYFYTAMSRPGRGEPRFIAVGYVDDTQFVRF

DSDAASPRTEPRAPWIEQEGPEYWDRETQISKTNTQTYRENLRIALRYYNQSEAGSHTWQ

TMYGCDVGPDGRLLRGHNQYAYDGKDYIALNEDLSSWTAADTAAQITQRKWEAAREAEQL

RAYLEGLCVEWLRRHLENGKETLQRADPPKTHVTHHPVSDHEATLRCWALGFYPAEITLT

WQRDGEDQTQDTELVETRPAGDRTFQKWAAVVVPSGEEQRYTCHVQHEGLPKPLTLRWEP

SSQSTIPIVGIVAGLAVLAVVVIGAVVATVMCRRKSSGGKGGSYSQAASSDSAQGSDVSL

TA

>sp|P30490|1B52_HUMAN 86 RETQISKTNTQTYREN

MRVTAPRTVLLLLWGAVALTETWAGSHSMRYFYTAMSRPGRGEPRFIAVGYVDDTQFVRF

DSDAASPRTEPRAPWIEQEGPEYWDRETQISKTNTQTYRENLRIALRYYNQSEAGSHTWQ

TMYGCDVGPDGRLLRGHNQYAYDGKDYIALNEDLSSWTAADTAAQITQRKWEAAREAEQL

RAYLEGLCVEWLRRHLENGKETLQRADPPKTHVTHHPVSDHEATLRCWALGFYPAEITLT

WQRDGEDQTQDTELVETRPAGDRTFQKWAAVVVPSGEEQRYTCHVQHEGLPKPLTLRWEP

SSQSTIPIVGIVAGLAVLAVVVIGAVVATVMCRRKSSGGKGGSYSQAASSDSAQGSDVSL

TA

>sp|P30490|1B52_HUMAN 86 RETQISKTNTQTYRENL

MRVTAPRTVLLLLWGAVALTETWAGSHSMRYFYTAMSRPGRGEPRFIAVGYVDDTQFVRF

DSDAASPRTEPRAPWIEQEGPEYWDRETQISKTNTQTYRENLRIALRYYNQSEAGSHTWQ

TMYGCDVGPDGRLLRGHNQYAYDGKDYIALNEDLSSWTAADTAAQITQRKWEAAREAEQL

RAYLEGLCVEWLRRHLENGKETLQRADPPKTHVTHHPVSDHEATLRCWALGFYPAEITLT

WQRDGEDQTQDTELVETRPAGDRTFQKWAAVVVPSGEEQRYTCHVQHEGLPKPLTLRWEP

SSQSTIPIVGIVAGLAVLAVVVIGAVVATVMCRRKSSGGKGGSYSQAASSDSAQGSDVSL

TA

>sp|P30488|1B50_HUMAN 25 GSHSMRYFHTAMSRPG

MRVTAPRTVLLLLSAALALTETWAGSHSMRYFHTAMSRPGRGEPRFITVGYVDDTLFVRF

DSDATSPRKEPRAPWIEQEGPEYWDRETQISKTNTQTYRESLRNLRGYYNQSEAGSHTWQ

RMYGCDLGPDGRLLRGYNQLAYDGKDYIALNEDLSSWTAADTAAQITQRKWEAAREAEQL

RAYLEGLCVEWLRRYLENGKETLQRADPPKTHVTHHPISDHEATLRCWALGFYPAEITLT

WQRDGEDQTQDTELVETRPAGDRTFQKWAAVVVPSGEEQRYTCHVQHEGLPKPLTLRWEP

SSQSTIPIVGIVAGLAVLAVVVIGAVVATVMCRRKSSGGKGGSYSQAASSDSAQGSDVSL

TA

>sp|P30488|1B50_HUMAN 25 GSHSMRYFHTAMSRPGRG

MRVTAPRTVLLLLSAALALTETWAGSHSMRYFHTAMSRPGRGEPRFITVGYVDDTLFVRF

DSDATSPRKEPRAPWIEQEGPEYWDRETQISKTNTQTYRESLRNLRGYYNQSEAGSHTWQ

RMYGCDLGPDGRLLRGYNQLAYDGKDYIALNEDLSSWTAADTAAQITQRKWEAAREAEQL

RAYLEGLCVEWLRRYLENGKETLQRADPPKTHVTHHPISDHEATLRCWALGFYPAEITLT

WQRDGEDQTQDTELVETRPAGDRTFQKWAAVVVPSGEEQRYTCHVQHEGLPKPLTLRWEP

SSQSTIPIVGIVAGLAVLAVVVIGAVVATVMCRRKSSGGKGGSYSQAASSDSAQGSDVSL

TA

>sp|P30488|1B50_HUMAN 25 GSHSMRYFHTAMSRPGRGE

MRVTAPRTVLLLLSAALALTETWAGSHSMRYFHTAMSRPGRGEPRFITVGYVDDTLFVRF

DSDATSPRKEPRAPWIEQEGPEYWDRETQISKTNTQTYRESLRNLRGYYNQSEAGSHTWQ

RMYGCDLGPDGRLLRGYNQLAYDGKDYIALNEDLSSWTAADTAAQITQRKWEAAREAEQL

RAYLEGLCVEWLRRYLENGKETLQRADPPKTHVTHHPISDHEATLRCWALGFYPAEITLT

WQRDGEDQTQDTELVETRPAGDRTFQKWAAVVVPSGEEQRYTCHVQHEGLPKPLTLRWEP

SSQSTIPIVGIVAGLAVLAVVVIGAVVATVMCRRKSSGGKGGSYSQAASSDSAQGSDVSL

TA

>sp|P30488|1B50_HUMAN 26 SHSMRYFHTAMSRP

MRVTAPRTVLLLLSAALALTETWAGSHSMRYFHTAMSRPGRGEPRFITVGYVDDTLFVRF

DSDATSPRKEPRAPWIEQEGPEYWDRETQISKTNTQTYRESLRNLRGYYNQSEAGSHTWQ

RMYGCDLGPDGRLLRGYNQLAYDGKDYIALNEDLSSWTAADTAAQITQRKWEAAREAEQL

RAYLEGLCVEWLRRYLENGKETLQRADPPKTHVTHHPISDHEATLRCWALGFYPAEITLT

WQRDGEDQTQDTELVETRPAGDRTFQKWAAVVVPSGEEQRYTCHVQHEGLPKPLTLRWEP

SSQSTIPIVGIVAGLAVLAVVVIGAVVATVMCRRKSSGGKGGSYSQAASSDSAQGSDVSL

TA

>sp|P30488|1B50_HUMAN 26 SHSMRYFHTAMSRPG

MRVTAPRTVLLLLSAALALTETWAGSHSMRYFHTAMSRPGRGEPRFITVGYVDDTLFVRF

DSDATSPRKEPRAPWIEQEGPEYWDRETQISKTNTQTYRESLRNLRGYYNQSEAGSHTWQ

RMYGCDLGPDGRLLRGYNQLAYDGKDYIALNEDLSSWTAADTAAQITQRKWEAAREAEQL

RAYLEGLCVEWLRRYLENGKETLQRADPPKTHVTHHPISDHEATLRCWALGFYPAEITLT

WQRDGEDQTQDTELVETRPAGDRTFQKWAAVVVPSGEEQRYTCHVQHEGLPKPLTLRWEP

SSQSTIPIVGIVAGLAVLAVVVIGAVVATVMCRRKSSGGKGGSYSQAASSDSAQGSDVSL

TA

>sp|P30488|1B50_HUMAN 26 SHSMRYFHTAMSRPGRGE

MRVTAPRTVLLLLSAALALTETWAGSHSMRYFHTAMSRPGRGEPRFITVGYVDDTLFVRF

DSDATSPRKEPRAPWIEQEGPEYWDRETQISKTNTQTYRESLRNLRGYYNQSEAGSHTWQ

RMYGCDLGPDGRLLRGYNQLAYDGKDYIALNEDLSSWTAADTAAQITQRKWEAAREAEQL

RAYLEGLCVEWLRRYLENGKETLQRADPPKTHVTHHPISDHEATLRCWALGFYPAEITLT

WQRDGEDQTQDTELVETRPAGDRTFQKWAAVVVPSGEEQRYTCHVQHEGLPKPLTLRWEP

SSQSTIPIVGIVAGLAVLAVVVIGAVVATVMCRRKSSGGKGGSYSQAASSDSAQGSDVSL

TA

>sp|P30488|1B50_HUMAN 52 VDDTLFVRFDSDATSPRKEPRAP

MRVTAPRTVLLLLSAALALTETWAGSHSMRYFHTAMSRPGRGEPRFITVGYVDDTLFVRF

DSDATSPRKEPRAPWIEQEGPEYWDRETQISKTNTQTYRESLRNLRGYYNQSEAGSHTWQ

RMYGCDLGPDGRLLRGYNQLAYDGKDYIALNEDLSSWTAADTAAQITQRKWEAAREAEQL

RAYLEGLCVEWLRRYLENGKETLQRADPPKTHVTHHPISDHEATLRCWALGFYPAEITLT

WQRDGEDQTQDTELVETRPAGDRTFQKWAAVVVPSGEEQRYTCHVQHEGLPKPLTLRWEP

SSQSTIPIVGIVAGLAVLAVVVIGAVVATVMCRRKSSGGKGGSYSQAASSDSAQGSDVSL

TA

>sp|P30485|1B47_HUMAN 128 GPDGRLLRGYHQDAYDGK

MRVTAPRTLLLLLWGAVALTETWAGSHSMRYFYTAMSRPGRGEPRFITVGYVDDTLFVRF

DSDATSPRKEPRAPWIEQEGPEYWDRETQISKTNTQTYREDLRTLLRYYNQSEAGSHTLQ

RMFGCDVGPDGRLLRGYHQDAYDGKDYIALNEDLSSWTAADTAAQITQRKWEAARVAEQL

RAYLEGECVEWLRRYLENGKETLQRADPPKTHVTHHPISDHEATLRCWALGFYPAEITLT

WQRDGEDQTQDTELVETRPAGDRTFQKWAAVVVPSGEEQRYTCHVQHEGLPKPLTLRWEP

SSQSTVPIVGIVAGLAVLAVVVIGAVVAAVVCRRKSSGGKGGSYSQAACSDSAQGSDVSL

TA

>sp|P30485|1B47_HUMAN 128 GPDGRLLRGYHQDAYDGKD

MRVTAPRTLLLLLWGAVALTETWAGSHSMRYFYTAMSRPGRGEPRFITVGYVDDTLFVRF

DSDATSPRKEPRAPWIEQEGPEYWDRETQISKTNTQTYREDLRTLLRYYNQSEAGSHTLQ

RMFGCDVGPDGRLLRGYHQDAYDGKDYIALNEDLSSWTAADTAAQITQRKWEAARVAEQL

RAYLEGECVEWLRRYLENGKETLQRADPPKTHVTHHPISDHEATLRCWALGFYPAEITLT

WQRDGEDQTQDTELVETRPAGDRTFQKWAAVVVPSGEEQRYTCHVQHEGLPKPLTLRWEP

SSQSTVPIVGIVAGLAVLAVVVIGAVVAAVVCRRKSSGGKGGSYSQAACSDSAQGSDVSL

TA

>sp|P30485|1B47_HUMAN 132 RLLRGYHQDAYDGK

MRVTAPRTLLLLLWGAVALTETWAGSHSMRYFYTAMSRPGRGEPRFITVGYVDDTLFVRF

DSDATSPRKEPRAPWIEQEGPEYWDRETQISKTNTQTYREDLRTLLRYYNQSEAGSHTLQ

RMFGCDVGPDGRLLRGYHQDAYDGKDYIALNEDLSSWTAADTAAQITQRKWEAARVAEQL

RAYLEGECVEWLRRYLENGKETLQRADPPKTHVTHHPISDHEATLRCWALGFYPAEITLT

WQRDGEDQTQDTELVETRPAGDRTFQKWAAVVVPSGEEQRYTCHVQHEGLPKPLTLRWEP

SSQSTVPIVGIVAGLAVLAVVVIGAVVAAVVCRRKSSGGKGGSYSQAACSDSAQGSDVSL

TA

>sp|P30485|1B47_HUMAN 132 RLLRGYHQDAYDGKD

MRVTAPRTLLLLLWGAVALTETWAGSHSMRYFYTAMSRPGRGEPRFITVGYVDDTLFVRF

DSDATSPRKEPRAPWIEQEGPEYWDRETQISKTNTQTYREDLRTLLRYYNQSEAGSHTLQ

RMFGCDVGPDGRLLRGYHQDAYDGKDYIALNEDLSSWTAADTAAQITQRKWEAARVAEQL

RAYLEGECVEWLRRYLENGKETLQRADPPKTHVTHHPISDHEATLRCWALGFYPAEITLT

WQRDGEDQTQDTELVETRPAGDRTFQKWAAVVVPSGEEQRYTCHVQHEGLPKPLTLRWEP

SSQSTVPIVGIVAGLAVLAVVVIGAVVAAVVCRRKSSGGKGGSYSQAACSDSAQGSDVSL

TA

>sp|Q31612|1B73_HUMAN 128 GPDGRLLRGHDQYAYDGKD

MLVMAPRTVLLLLSAALALTETWAGSHSMRYFYTSVSRPGRGEPRFISVGYVDDTQFVRF

DSDAASPREEPRAPWIEQEGPEYWDRNTQIYKAQAQTDRESLRNLRGYYNQSEAGSHTLQ

SMYGCDVGPDGRLLRGHDQYAYDGKDYIALNEDLRSWTAADTAAQITQRKWEAAREAEQR

RAYLEGECVEWLRRYLENGKDKLERADPPKTHVTHHPISDHEATLRCWALGFYPAEITLT

WQRDGEDQTQDTELVETRPAGDRTFQKWAAVVVPSGEEQRYTCHVQHEGLPKPLTLRWEP

SSQSTVPIVGIVAGLAVLAVVVIGAVVAAVMCRRKSSGGKGGSYSQAACSDSAQGSDVSL

TA

>sp|Q31612|1B73_HUMAN 128 GPDGRLLRGHDQYAYDGKDY

MLVMAPRTVLLLLSAALALTETWAGSHSMRYFYTSVSRPGRGEPRFISVGYVDDTQFVRF

DSDAASPREEPRAPWIEQEGPEYWDRNTQIYKAQAQTDRESLRNLRGYYNQSEAGSHTLQ

SMYGCDVGPDGRLLRGHDQYAYDGKDYIALNEDLRSWTAADTAAQITQRKWEAAREAEQR

RAYLEGECVEWLRRYLENGKDKLERADPPKTHVTHHPISDHEATLRCWALGFYPAEITLT

WQRDGEDQTQDTELVETRPAGDRTFQKWAAVVVPSGEEQRYTCHVQHEGLPKPLTLRWEP

SSQSTVPIVGIVAGLAVLAVVVIGAVVAAVMCRRKSSGGKGGSYSQAACSDSAQGSDVSL

TA

>sp|P13747|HLAE_HUMAN 150 DLRSWTAVDTAAQ

MVDGTLLLLLSEALALTQTWAGSHSLKYFHTSVSRPGRGEPRFISVGYVDDTQFVRFDND

AASPRMVPRAPWMEQEGSEYWDRETRSARDTAQIFRVNLRTLRGYYNQSEAGSHTLQWMH

GCELGPDRRFLRGYEQFAYDGKDYLTLNEDLRSWTAVDTAAQISEQKSNDASEAEHQRAY

LEDTCVEWLHKYLEKGKETLLHLEPPKTHVTHHPISDHEATLRCWALGFYPAEITLTWQQ

DGEGHTQDTELVETRPAGDGTFQKWAAVVVPSGEEQRYTCHVQHEGLPEPVTLRWKPASQ

PTIPIVGIIAGLVLLGSVVSGAVVAAVIWRKKSSGGKGGSYSKAEWSDSAQGSESHSL

>sp|P13747|HLAE_HUMAN 150 DLRSWTAVDTAAQIS

MVDGTLLLLLSEALALTQTWAGSHSLKYFHTSVSRPGRGEPRFISVGYVDDTQFVRFDND

AASPRMVPRAPWMEQEGSEYWDRETRSARDTAQIFRVNLRTLRGYYNQSEAGSHTLQWMH

GCELGPDRRFLRGYEQFAYDGKDYLTLNEDLRSWTAVDTAAQISEQKSNDASEAEHQRAY

LEDTCVEWLHKYLEKGKETLLHLEPPKTHVTHHPISDHEATLRCWALGFYPAEITLTWQQ

DGEGHTQDTELVETRPAGDGTFQKWAAVVVPSGEEQRYTCHVQHEGLPEPVTLRWKPASQ

PTIPIVGIIAGLVLLGSVVSGAVVAAVIWRKKSSGGKGGSYSKAEWSDSAQGSESHSL

>sp|P13747|HLAE_HUMAN 150 DLRSWTAVDTAAQISE

MVDGTLLLLLSEALALTQTWAGSHSLKYFHTSVSRPGRGEPRFISVGYVDDTQFVRFDND

AASPRMVPRAPWMEQEGSEYWDRETRSARDTAQIFRVNLRTLRGYYNQSEAGSHTLQWMH

GCELGPDRRFLRGYEQFAYDGKDYLTLNEDLRSWTAVDTAAQISEQKSNDASEAEHQRAY

LEDTCVEWLHKYLEKGKETLLHLEPPKTHVTHHPISDHEATLRCWALGFYPAEITLTWQQ

DGEGHTQDTELVETRPAGDGTFQKWAAVVVPSGEEQRYTCHVQHEGLPEPVTLRWKPASQ

PTIPIVGIIAGLVLLGSVVSGAVVAAVIWRKKSSGGKGGSYSKAEWSDSAQGSESHSL

>sp|P13747|HLAE_HUMAN 150 DLRSWTAVDTAAQISEQ

MVDGTLLLLLSEALALTQTWAGSHSLKYFHTSVSRPGRGEPRFISVGYVDDTQFVRFDND

AASPRMVPRAPWMEQEGSEYWDRETRSARDTAQIFRVNLRTLRGYYNQSEAGSHTLQWMH

GCELGPDRRFLRGYEQFAYDGKDYLTLNEDLRSWTAVDTAAQISEQKSNDASEAEHQRAY

LEDTCVEWLHKYLEKGKETLLHLEPPKTHVTHHPISDHEATLRCWALGFYPAEITLTWQQ

DGEGHTQDTELVETRPAGDGTFQKWAAVVVPSGEEQRYTCHVQHEGLPEPVTLRWKPASQ

PTIPIVGIIAGLVLLGSVVSGAVVAAVIWRKKSSGGKGGSYSKAEWSDSAQGSESHSL

>sp|P13747|HLAE_HUMAN 150 DLRSWTAVDTAAQISEQK

MVDGTLLLLLSEALALTQTWAGSHSLKYFHTSVSRPGRGEPRFISVGYVDDTQFVRFDND

AASPRMVPRAPWMEQEGSEYWDRETRSARDTAQIFRVNLRTLRGYYNQSEAGSHTLQWMH

GCELGPDRRFLRGYEQFAYDGKDYLTLNEDLRSWTAVDTAAQISEQKSNDASEAEHQRAY

LEDTCVEWLHKYLEKGKETLLHLEPPKTHVTHHPISDHEATLRCWALGFYPAEITLTWQQ

DGEGHTQDTELVETRPAGDGTFQKWAAVVVPSGEEQRYTCHVQHEGLPEPVTLRWKPASQ

PTIPIVGIIAGLVLLGSVVSGAVVAAVIWRKKSSGGKGGSYSKAEWSDSAQGSESHSL

>sp|P13747|HLAE_HUMAN 149 EDLRSWTAVDTAAQISE

MVDGTLLLLLSEALALTQTWAGSHSLKYFHTSVSRPGRGEPRFISVGYVDDTQFVRFDND

AASPRMVPRAPWMEQEGSEYWDRETRSARDTAQIFRVNLRTLRGYYNQSEAGSHTLQWMH

GCELGPDRRFLRGYEQFAYDGKDYLTLNEDLRSWTAVDTAAQISEQKSNDASEAEHQRAY

LEDTCVEWLHKYLEKGKETLLHLEPPKTHVTHHPISDHEATLRCWALGFYPAEITLTWQQ

DGEGHTQDTELVETRPAGDGTFQKWAAVVVPSGEEQRYTCHVQHEGLPEPVTLRWKPASQ

PTIPIVGIIAGLVLLGSVVSGAVVAAVIWRKKSSGGKGGSYSKAEWSDSAQGSESHSL

>sp|P13747|HLAE_HUMAN 149 EDLRSWTAVDTAAQISEQ

MVDGTLLLLLSEALALTQTWAGSHSLKYFHTSVSRPGRGEPRFISVGYVDDTQFVRFDND

AASPRMVPRAPWMEQEGSEYWDRETRSARDTAQIFRVNLRTLRGYYNQSEAGSHTLQWMH

GCELGPDRRFLRGYEQFAYDGKDYLTLNEDLRSWTAVDTAAQISEQKSNDASEAEHQRAY

LEDTCVEWLHKYLEKGKETLLHLEPPKTHVTHHPISDHEATLRCWALGFYPAEITLTWQQ

DGEGHTQDTELVETRPAGDGTFQKWAAVVVPSGEEQRYTCHVQHEGLPEPVTLRWKPASQ

PTIPIVGIIAGLVLLGSVVSGAVVAAVIWRKKSSGGKGGSYSKAEWSDSAQGSESHSL

>sp|P13747|HLAE_HUMAN 149 EDLRSWTAVDTAAQISEQK

MVDGTLLLLLSEALALTQTWAGSHSLKYFHTSVSRPGRGEPRFISVGYVDDTQFVRFDND

AASPRMVPRAPWMEQEGSEYWDRETRSARDTAQIFRVNLRTLRGYYNQSEAGSHTLQWMH

GCELGPDRRFLRGYEQFAYDGKDYLTLNEDLRSWTAVDTAAQISEQKSNDASEAEHQRAY

LEDTCVEWLHKYLEKGKETLLHLEPPKTHVTHHPISDHEATLRCWALGFYPAEITLTWQQ

DGEGHTQDTELVETRPAGDGTFQKWAAVVVPSGEEQRYTCHVQHEGLPEPVTLRWKPASQ

PTIPIVGIIAGLVLLGSVVSGAVVAAVIWRKKSSGGKGGSYSKAEWSDSAQGSESHSL

>sp|P13747|HLAE_HUMAN 151 LRSWTAVDTAAQISE

MVDGTLLLLLSEALALTQTWAGSHSLKYFHTSVSRPGRGEPRFISVGYVDDTQFVRFDND

AASPRMVPRAPWMEQEGSEYWDRETRSARDTAQIFRVNLRTLRGYYNQSEAGSHTLQWMH

GCELGPDRRFLRGYEQFAYDGKDYLTLNEDLRSWTAVDTAAQISEQKSNDASEAEHQRAY

LEDTCVEWLHKYLEKGKETLLHLEPPKTHVTHHPISDHEATLRCWALGFYPAEITLTWQQ

DGEGHTQDTELVETRPAGDGTFQKWAAVVVPSGEEQRYTCHVQHEGLPEPVTLRWKPASQ

PTIPIVGIIAGLVLLGSVVSGAVVAAVIWRKKSSGGKGGSYSKAEWSDSAQGSESHSL

>sp|P13747|HLAE_HUMAN 148 NEDLRSWTAVDTAAQISEQ

MVDGTLLLLLSEALALTQTWAGSHSLKYFHTSVSRPGRGEPRFISVGYVDDTQFVRFDND

AASPRMVPRAPWMEQEGSEYWDRETRSARDTAQIFRVNLRTLRGYYNQSEAGSHTLQWMH

GCELGPDRRFLRGYEQFAYDGKDYLTLNEDLRSWTAVDTAAQISEQKSNDASEAEHQRAY

LEDTCVEWLHKYLEKGKETLLHLEPPKTHVTHHPISDHEATLRCWALGFYPAEITLTWQQ

DGEGHTQDTELVETRPAGDGTFQKWAAVVVPSGEEQRYTCHVQHEGLPEPVTLRWKPASQ

PTIPIVGIIAGLVLLGSVVSGAVVAAVIWRKKSSGGKGGSYSKAEWSDSAQGSESHSL

>sp|P13747|HLAE_HUMAN 148 NEDLRSWTAVDTAAQISEQK

MVDGTLLLLLSEALALTQTWAGSHSLKYFHTSVSRPGRGEPRFISVGYVDDTQFVRFDND

AASPRMVPRAPWMEQEGSEYWDRETRSARDTAQIFRVNLRTLRGYYNQSEAGSHTLQWMH

GCELGPDRRFLRGYEQFAYDGKDYLTLNEDLRSWTAVDTAAQISEQKSNDASEAEHQRAY

LEDTCVEWLHKYLEKGKETLLHLEPPKTHVTHHPISDHEATLRCWALGFYPAEITLTWQQ

DGEGHTQDTELVETRPAGDGTFQKWAAVVVPSGEEQRYTCHVQHEGLPEPVTLRWKPASQ

PTIPIVGIIAGLVLLGSVVSGAVVAAVIWRKKSSGGKGGSYSKAEWSDSAQGSESHSL

>sp|O75503|CLN5_HUMAN 110 LGHLKIMHDAIGFR

MAQEVDTAQGAEMRRGAGAARGRASWCWALALLWLAVVPGWSRVSGIPSRRHWPVPYKRF

DFRPKPDPYCQAKYTFCPTGSPIPVMEGDDDIEVFRLQAPVWEFKYGDLLGHLKIMHDAI

GFRSTLTGKNYTMEWYELFQLGNCTFPHLRPEMDAPFWCNQGAACFFEGIDDVHWKENGT

LVQVATISGNMFNQMAKWVKQDNETGIYYETWNVKASPEKGAETWFDSYDCSKFVLRTFN

KLAEFGAEFKNIETNYTRIFLYSGEPTYLGNETSVFGPTGNKTLGLAIKRFYYPFKPHLP

TKEFLLSLLQIFDAVIVHKQFYLFYNFEYWFLPMKFPFIKITYEEIPLPIRNKTLSGL

>sp|Q15165|PON2_HUMAN 228 SPDDKYIYVADILAHEIH

MGAWVGCGLAGDRAGFLGERLLALRNRLKASREVESVDLPHCHLIKGIEAGSEDIDILPN

GLAFFSVGLKFPGLHSFAPDKPGGILMMDLKEEKPRARELRISRGFDLASFNPHGISTFI

DNDDTVYLFVVNHPEFKNTVEIFKFEEAENSLLHLKTVKHELLPSVNDITAVGPAHFYAT

NDHYFSDPFLKYLETYLNLHWANVVYYSPNEVKVVAEGFDSANGINISPDDKYIYVADIL

AHEIHVLEKHTNMNLTQLKVLELDTLVDNLSIDPSSGDIWVGCHPNGQKLFVYDPNNPPS

SEVLRIQNILCEKPTVTTVYANNGSVLQGSSVASVYDGKLLIGTLYHRALYCEL

>sp|P61073|CXCR4_HUMAN 306 GAKFKTSAQHAL

MEGISIYTSDNYTEEMGSGDYDSMKEPCFREENANFNKIFLPTIYSIIFLTGIVGNGLVI

LVMGYQKKLRSMTDKYRLHLSVADLLFVITLPFWAVDAVANWYFGNFLCKAVHVIYTVNL

YSSVLILAFISLDRYLAIVHATNSQRPRKLLAEKVVYVGVWIPALLLTIPDFIFANVSEA

DDRYICDRFYPNDLWVVVFQFQHIMVGLILPGIVILSCYCIIISKLSHSKGHQKRKALKT

TVILILAFFACWLPYYIGISIDSFILLEIIKQGCEFENTVHKWISITEALAFFHCCLNPI

LYAFLGAKFKTSAQHALTSVSRGSSLKILSKGKRGGHSSVSTESESSSFHSS

>sp|P08567|PLEK_HUMAN 161 VIDWLVSNQSVRNRQEGL

MEPKRIREGYLVKKGSVFNTWKPMWVVLLEDGIEFYKKKSDNSPKGMIPLKGSTLTSPCQ

DFGKRMFVFKITTTKQQDHFFQAAFLEERDAWVRDIKKAIKCIEGGQKFARKSTRRSIRL

PETIDLGALYLSMKDTEKGIKELNLEKDKKIFNHCFTGNCVIDWLVSNQSVRNRQEGLMI

ASSLLNEGYLQPAGDMSKSAVDGTAENPFLDNPDAFYYFPDSGFFCEENSSDDDVILKEE

FRGVIIKQGCLLKQGHRRKNWKVRKFILREDPAYLHYYDPAGAEDPLGAIHLRGCVVTSV

ESNSNGRKSEEENLFEIITADEVHYFLQAATPKERTEWIKAIQMASRTGK

>sp|P25024|CXCR1_HUMAN 169 LPFFLFRQAYHPNNSSPVCY

MSNITDPQMWDFDDLNFTGMPPADEDYSPCMLETETLNKYVVIIAYALVFLLSLLGNSLV

MLVILYSRVGRSVTDVYLLNLALADLLFALTLPIWAASKVNGWIFGTFLCKVVSLLKEVN

FYSGILLLACISVDRYLAIVHATRTLTQKRHLVKFVCLGCWGLSMNLSLPFFLFRQAYHP

NNSSPVCYEVLGNDTAKWRMVLRILPHTFGFIVPLFVMLFCYGFTLRTLFKAHMGQKHRA

MRVIFAVVLIFLLCWLPYNLVLLADTLMRTQVIQESCERRNNIGRALDATEILGFLHSCL

NPIIYAFIGQNFRHGFLKILAMHGLVSKEFLARHRVTSYTSSSVNVSSNL

>sp|O14828|SCAM3_HUMAN 83 YGSYSTQASAAAATAE

MAQSRDGGNPFAEPSELDNPFQDPAVIQHRPSRQYATRDVYNPFETREPPPAYEPPAPAP

LPPPSAPSLQPSRKLSPTEPKNYGSYSTQASAAAATAELLKKQEELNRKAEELDRREREL

QHAALGGTATRQNNWPPLPSFCPVQPCFFQDISMEIPQEFQKTVSTMYYLWMCSTLALLL

NFLACLASFCVETNNGAGFGLSILWVLLFTPCSFVCWYRPMYKAFRSDSSFNFFAFFFNF

FDQDVLFVLQAIGIPGWGFSGWISALVVPKGNTAVSVLMLLVALLFTGIAVLGIVMLKRI

HSLYRRTGASFQKAQQEFAAGVFSNPAVRTAAANAAAGAAENAFRAP

>sp|P02750|A2GL_HUMAN 235 QPDLRYLFLNGN

MSSWSRQRPKSPGGIQPHVSRTLFLLLLLAASAWGVTLSPKDCQVFRSDHGSSISCQPPA

EIPGYLPADTVHLAVEFFNLTHLPANLLQGASKLQELHLSSNGLESLSPEFLRPVPQLRV

LDLTRNALTGLPPGLFQASATLDTLVLKENQLEVLEVSWLHGLKALGHLDLSGNRLRKLP

PGLLANFTLLRTLDLGENQLETLPPDLLRGPLQLERLHLEGNKLQVLGKDLLLPQPDLRY

LFLNGNKLARVAAGAFQGLRQLDMLDLSNNSLASVPEGLWASLGQPNWDMRDGFDISGNP

WICDQNLSDLYRWLQAQKDKMFSQNDTRCAGPEAVKGQTLLAVAKSQ

>sp|P07355|ANXA2_HUMAN 209 DVPKWISIMTERSVPHL

MSTVHEILCKLSLEGDHSTPPSAYGSVKAYTNFDAERDALNIETAIKTKGVDEVTIVNIL

TNRSNAQRQDIAFAYQRRTKKELASALKSALSGHLETVILGLLKTPAQYDASELKASMKG

LGTDEDSLIEIICSRTNQELQEINRVYKEMYKTDLEKDIISDTSGDFRKLMVALAKGRRA

EDGSVIDYELIDQDARDLYDAGVKRKGTDVPKWISIMTERSVPHLQKVFDRYKSYSPYDM

LESIRKEVKGDLENAFLNLVQCIQNKPLYFADRLYDSMKGKGTRDKVLIRIMVSRSEVDM

LKIRSEFKRKYGKSLYYYIQQDTKGDYQKALLYLCGGDD

>sp|P07355|ANXA2_HUMAN 209 DVPKWISIMTERSVPHLQ

MSTVHEILCKLSLEGDHSTPPSAYGSVKAYTNFDAERDALNIETAIKTKGVDEVTIVNIL

TNRSNAQRQDIAFAYQRRTKKELASALKSALSGHLETVILGLLKTPAQYDASELKASMKG

LGTDEDSLIEIICSRTNQELQEINRVYKEMYKTDLEKDIISDTSGDFRKLMVALAKGRRA

EDGSVIDYELIDQDARDLYDAGVKRKGTDVPKWISIMTERSVPHLQKVFDRYKSYSPYDM

LESIRKEVKGDLENAFLNLVQCIQNKPLYFADRLYDSMKGKGTRDKVLIRIMVSRSEVDM

LKIRSEFKRKYGKSLYYYIQQDTKGDYQKALLYLCGGDD

>sp|P17693|HLAG_HUMAN 51 YVDDTQFVRFDSDSACPRMEPRAP

MVVMAPRTLFLLLSGALTLTETWAGSHSMRYFSAAVSRPGRGEPRFIAMGYVDDTQFVRF

DSDSACPRMEPRAPWVEQEGPEYWEEETRNTKAHAQTDRMNLQTLRGYYNQSEASSHTLQ

WMIGCDLGSDGRLLRGYEQYAYDGKDYLALNEDLRSWTAADTAAQISKRKCEAANVAEQR

RAYLEGTCVEWLHRYLENGKEMLQRADPPKTHVTHHPVFDYEATLRCWALGFYPAEIILT

WQRDGEDQTQDVELVETRPAGDGTFQKWAAVVVPSGEEQRYTCHVQHEGLPEPLMLRWKQ

SSLPTIPIMGIVAGLVVLAAVVTGAAVAAVLWRKKSSD

>sp|P04406|G3P_HUMAN 11 FGRIGRLVTRAAFN

MGKVKVGVNGFGRIGRLVTRAAFNSGKVDIVAINDPFIDLNYMVYMFQYDSTHGKFHGTV

KAENGKLVINGNPITIFQERDPSKIKWGDAGAEYVVESTGVFTTMEKAGAHLQGGAKRVI

ISAPSADAPMFVMGVNHEKYDNSLKIISNASCTTNCLAPLAKVIHDNFGIVEGLMTTVHA

ITATQKTVDGPSGKLWRDGRGALQNIIPASTGAAKAVGKVIPELNGKLTGMAFRVPTANV

SVVDLTCRLEKPAKYDDIKKVVKQASEGPLKGILGYTEHQVVSSDFNSDTHSSTFDAGAG

IALNDHFVKLISWYDNEFGYSNRVVDLMAHMASKE

>sp|P04406|G3P_HUMAN 11 FGRIGRLVTRAAFNS

MGKVKVGVNGFGRIGRLVTRAAFNSGKVDIVAINDPFIDLNYMVYMFQYDSTHGKFHGTV

KAENGKLVINGNPITIFQERDPSKIKWGDAGAEYVVESTGVFTTMEKAGAHLQGGAKRVI

ISAPSADAPMFVMGVNHEKYDNSLKIISNASCTTNCLAPLAKVIHDNFGIVEGLMTTVHA

ITATQKTVDGPSGKLWRDGRGALQNIIPASTGAAKAVGKVIPELNGKLTGMAFRVPTANV

SVVDLTCRLEKPAKYDDIKKVVKQASEGPLKGILGYTEHQVVSSDFNSDTHSSTFDAGAG

IALNDHFVKLISWYDNEFGYSNRVVDLMAHMASKE

>sp|P04406|G3P_HUMAN 10 GFGRIGRLVTRAAFN

MGKVKVGVNGFGRIGRLVTRAAFNSGKVDIVAINDPFIDLNYMVYMFQYDSTHGKFHGTV

KAENGKLVINGNPITIFQERDPSKIKWGDAGAEYVVESTGVFTTMEKAGAHLQGGAKRVI

ISAPSADAPMFVMGVNHEKYDNSLKIISNASCTTNCLAPLAKVIHDNFGIVEGLMTTVHA

ITATQKTVDGPSGKLWRDGRGALQNIIPASTGAAKAVGKVIPELNGKLTGMAFRVPTANV

SVVDLTCRLEKPAKYDDIKKVVKQASEGPLKGILGYTEHQVVSSDFNSDTHSSTFDAGAG

IALNDHFVKLISWYDNEFGYSNRVVDLMAHMASKE

>sp|P04406|G3P_HUMAN 10 GFGRIGRLVTRAAFNSG

MGKVKVGVNGFGRIGRLVTRAAFNSGKVDIVAINDPFIDLNYMVYMFQYDSTHGKFHGTV

KAENGKLVINGNPITIFQERDPSKIKWGDAGAEYVVESTGVFTTMEKAGAHLQGGAKRVI

ISAPSADAPMFVMGVNHEKYDNSLKIISNASCTTNCLAPLAKVIHDNFGIVEGLMTTVHA

ITATQKTVDGPSGKLWRDGRGALQNIIPASTGAAKAVGKVIPELNGKLTGMAFRVPTANV

SVVDLTCRLEKPAKYDDIKKVVKQASEGPLKGILGYTEHQVVSSDFNSDTHSSTFDAGAG

IALNDHFVKLISWYDNEFGYSNRVVDLMAHMASKE

>sp|P04406|G3P_HUMAN 203 LQNIIPASTGAAKAVG

MGKVKVGVNGFGRIGRLVTRAAFNSGKVDIVAINDPFIDLNYMVYMFQYDSTHGKFHGTV

KAENGKLVINGNPITIFQERDPSKIKWGDAGAEYVVESTGVFTTMEKAGAHLQGGAKRVI

ISAPSADAPMFVMGVNHEKYDNSLKIISNASCTTNCLAPLAKVIHDNFGIVEGLMTTVHA

ITATQKTVDGPSGKLWRDGRGALQNIIPASTGAAKAVGKVIPELNGKLTGMAFRVPTANV

SVVDLTCRLEKPAKYDDIKKVVKQASEGPLKGILGYTEHQVVSSDFNSDTHSSTFDAGAG

IALNDHFVKLISWYDNEFGYSNRVVDLMAHMASKE

>sp|P04406|G3P_HUMAN 293 STFDAGAGIALNDH

MGKVKVGVNGFGRIGRLVTRAAFNSGKVDIVAINDPFIDLNYMVYMFQYDSTHGKFHGTV

KAENGKLVINGNPITIFQERDPSKIKWGDAGAEYVVESTGVFTTMEKAGAHLQGGAKRVI

ISAPSADAPMFVMGVNHEKYDNSLKIISNASCTTNCLAPLAKVIHDNFGIVEGLMTTVHA

ITATQKTVDGPSGKLWRDGRGALQNIIPASTGAAKAVGKVIPELNGKLTGMAFRVPTANV

SVVDLTCRLEKPAKYDDIKKVVKQASEGPLKGILGYTEHQVVSSDFNSDTHSSTFDAGAG

IALNDHFVKLISWYDNEFGYSNRVVDLMAHMASKE

>sp|P04406|G3P_HUMAN 8 VNGFGRIGRLVTR

MGKVKVGVNGFGRIGRLVTRAAFNSGKVDIVAINDPFIDLNYMVYMFQYDSTHGKFHGTV

KAENGKLVINGNPITIFQERDPSKIKWGDAGAEYVVESTGVFTTMEKAGAHLQGGAKRVI

ISAPSADAPMFVMGVNHEKYDNSLKIISNASCTTNCLAPLAKVIHDNFGIVEGLMTTVHA

ITATQKTVDGPSGKLWRDGRGALQNIIPASTGAAKAVGKVIPELNGKLTGMAFRVPTANV

SVVDLTCRLEKPAKYDDIKKVVKQASEGPLKGILGYTEHQVVSSDFNSDTHSSTFDAGAG

IALNDHFVKLISWYDNEFGYSNRVVDLMAHMASKE

>sp|P04406|G3P_HUMAN 140 YDNSLKIISNASCTTN

MGKVKVGVNGFGRIGRLVTRAAFNSGKVDIVAINDPFIDLNYMVYMFQYDSTHGKFHGTV

KAENGKLVINGNPITIFQERDPSKIKWGDAGAEYVVESTGVFTTMEKAGAHLQGGAKRVI

ISAPSADAPMFVMGVNHEKYDNSLKIISNASCTTNCLAPLAKVIHDNFGIVEGLMTTVHA

ITATQKTVDGPSGKLWRDGRGALQNIIPASTGAAKAVGKVIPELNGKLTGMAFRVPTANV

SVVDLTCRLEKPAKYDDIKKVVKQASEGPLKGILGYTEHQVVSSDFNSDTHSSTFDAGAG

IALNDHFVKLISWYDNEFGYSNRVVDLMAHMASKE

>sp|P09668|CATH_HUMAN 185 LPSQAFEYILYNKG

MWATLPLLCAGAWLLGVPVCGAAELSVNSLEKFHFKSWMSKHRKTYSTEEYHHRLQTFAS

NWRKINAHNNGNHTFKMALNQFSDMSFAEIKHKYLWSEPQNCSATKSNYLRGTGPYPPSV

DWRKKGNFVSPVKNQGACGSCWTFSTTGALESAIAIATGKMLSLAEQQLVDCAQDFNNHG

CQGGLPSQAFEYILYNKGIMGEDTYPYQGKDGYCKFQPGKAIGFVKDVANITIYDEEAMV

EAVALYNPVSFAFEVTQDFMMYRTGIYSSTSCHKTPDKVNHAVLAVGYGEKNGIPYWIVK

NSWGPQWGMNGYFLIERGKNMCGLAACASYPIPLV

>sp|P52799|EFNB2_HUMAN 128 KNKDYYIISTSNGSLEG

MAVRRDSVWKYCWGVLMVLCRTAISKSIVLEPIYWNSSNSKFLPGQGLVLYPQIGDKLDI

ICPKVDSKTVGQYEYYKVYMVDKDQADRCTIKKENTPLLNCAKPDQDIKFTIKFQEFSPN

LWGLEFQKNKDYYIISTSNGSLEGLDNQEGGVCQTRAMKILMKVGQDASSAGSTRNKDPT

RRPELEAGTNGRSSTTSPFVKPNPGSSTDGNSAGHSGNNILGSEVALFAGIASGCIIFIV

IIITLVVLLLKYRRRHRKHSPQHTTTLSLSTLATPKRSGNNNGSEPSDIIIPLRTADSVF

CPHYEKVSGDYGHPVYIVQEMPPQSPANIYYKV

>sp|Q96DU3|SLAF6_HUMAN 103 DTGSYRAQISTKTSAK

MLWLFQSLLFVFCFGPGNVVSQSSLTPLMVNGILGESVTLPLEFPAGEKVNFITWLFNET

SLAFIVPHETKSPEIHVTNPKQGKRLNFTQSYSLQLSNLKMEDTGSYRAQISTKTSAKLS

SYTLRILRQLRNIQVTNHSQLFQNMTCELHLTCSVEDADDNVSFRWEALGNTLSSQPNLT

VSWDPRISSEQDYTCIAENAVSNLSFSVSAQKLCEDVKIQYTDTKMILFMVSGICIVFGF

IILLLLVLRKRRDSLSLSTQRTQGPESARNLEYVSVSPTNNTVYASVTHSNRETEIWTPR

ENDTITIYSTINHSKESKPTFSRATALDNVV

>sp|P25774|CATS_HUMAN 21 DPTLDHHWHLWKKTYGKQYK

MKRLVCVLLVCSSAVAQLHKDPTLDHHWHLWKKTYGKQYKEKNEEAVRRLIWEKNLKFVM

LHNLEHSMGMHSYDLGMNHLGDMTSEEVMSLMSSLRVPSQWQRNITYKSNPNRILPDSVD

WREKGCVTEVKYQGSCGACWAFSAVGALEAQLKLKTGKLVSLSAQNLVDCSTEKYGNKGC

NGGFMTTAFQYIIDNKGIDSDASYPYKAMDQKCQYDSKYRAATCSKYTELPYGREDVLKE

AVANKGPVSVGVDARHPSFFLYRSGVYYEPSCTQNVNHGVLVVGYGDLNGKEYWLVKNSW

GHNFGEEGYIRMARNKGNHCGIASFPSYPEI

>sp|P25774|CATS_HUMAN 21 DPTLDHHWHLWKKTYGKQYKE

MKRLVCVLLVCSSAVAQLHKDPTLDHHWHLWKKTYGKQYKEKNEEAVRRLIWEKNLKFVM

LHNLEHSMGMHSYDLGMNHLGDMTSEEVMSLMSSLRVPSQWQRNITYKSNPNRILPDSVD

WREKGCVTEVKYQGSCGACWAFSAVGALEAQLKLKTGKLVSLSAQNLVDCSTEKYGNKGC

NGGFMTTAFQYIIDNKGIDSDASYPYKAMDQKCQYDSKYRAATCSKYTELPYGREDVLKE

AVANKGPVSVGVDARHPSFFLYRSGVYYEPSCTQNVNHGVLVVGYGDLNGKEYWLVKNSW

GHNFGEEGYIRMARNKGNHCGIASFPSYPEI

>sp|P25774|CATS_HUMAN 290 GKEYWLVKNSWGHN

MKRLVCVLLVCSSAVAQLHKDPTLDHHWHLWKKTYGKQYKEKNEEAVRRLIWEKNLKFVM

LHNLEHSMGMHSYDLGMNHLGDMTSEEVMSLMSSLRVPSQWQRNITYKSNPNRILPDSVD

WREKGCVTEVKYQGSCGACWAFSAVGALEAQLKLKTGKLVSLSAQNLVDCSTEKYGNKGC

NGGFMTTAFQYIIDNKGIDSDASYPYKAMDQKCQYDSKYRAATCSKYTELPYGREDVLKE

AVANKGPVSVGVDARHPSFFLYRSGVYYEPSCTQNVNHGVLVVGYGDLNGKEYWLVKNSW

GHNFGEEGYIRMARNKGNHCGIASFPSYPEI

>sp|P25774|CATS_HUMAN 54 KNLKFVMLHNLEHSM

MKRLVCVLLVCSSAVAQLHKDPTLDHHWHLWKKTYGKQYKEKNEEAVRRLIWEKNLKFVM

LHNLEHSMGMHSYDLGMNHLGDMTSEEVMSLMSSLRVPSQWQRNITYKSNPNRILPDSVD

WREKGCVTEVKYQGSCGACWAFSAVGALEAQLKLKTGKLVSLSAQNLVDCSTEKYGNKGC

NGGFMTTAFQYIIDNKGIDSDASYPYKAMDQKCQYDSKYRAATCSKYTELPYGREDVLKE

AVANKGPVSVGVDARHPSFFLYRSGVYYEPSCTQNVNHGVLVVGYGDLNGKEYWLVKNSW

GHNFGEEGYIRMARNKGNHCGIASFPSYPEI

>sp|P25774|CATS_HUMAN 187 TAFQYIIDNKGIDSDAS

MKRLVCVLLVCSSAVAQLHKDPTLDHHWHLWKKTYGKQYKEKNEEAVRRLIWEKNLKFVM

LHNLEHSMGMHSYDLGMNHLGDMTSEEVMSLMSSLRVPSQWQRNITYKSNPNRILPDSVD

WREKGCVTEVKYQGSCGACWAFSAVGALEAQLKLKTGKLVSLSAQNLVDCSTEKYGNKGC

NGGFMTTAFQYIIDNKGIDSDASYPYKAMDQKCQYDSKYRAATCSKYTELPYGREDVLKE

AVANKGPVSVGVDARHPSFFLYRSGVYYEPSCTQNVNHGVLVVGYGDLNGKEYWLVKNSW

GHNFGEEGYIRMARNKGNHCGIASFPSYPEI

>sp|P25774|CATS_HUMAN 186 TTAFQYIIDNKGIDSD

MKRLVCVLLVCSSAVAQLHKDPTLDHHWHLWKKTYGKQYKEKNEEAVRRLIWEKNLKFVM

LHNLEHSMGMHSYDLGMNHLGDMTSEEVMSLMSSLRVPSQWQRNITYKSNPNRILPDSVD

WREKGCVTEVKYQGSCGACWAFSAVGALEAQLKLKTGKLVSLSAQNLVDCSTEKYGNKGC

NGGFMTTAFQYIIDNKGIDSDASYPYKAMDQKCQYDSKYRAATCSKYTELPYGREDVLKE

AVANKGPVSVGVDARHPSFFLYRSGVYYEPSCTQNVNHGVLVVGYGDLNGKEYWLVKNSW

GHNFGEEGYIRMARNKGNHCGIASFPSYPEI

>sp|O15127|SCAM2_HUMAN 310 SSRTFHRAASSAAQGAF

MSAFDTNPFADPVDVNPFQDPSVTQLTNAPQGGLAEFNPFSETNAATTVPVTQLPGSSQP

AVLQPSVEPTQPTPQAVVSAAQAGLLRQQEELDRKAAELERKERELQNTVANLHVRQNNW

PPLPSWCPVKPCFYQDFSTEIPADYQRICKMLYYLWMLHSVTLFLNLLACLAWFSGNSSK

GVDFGLSILWFLIFTPCAFLCWYRPIYKAFRSDNSFSFFVFFFVFFCQIGIYIIQLVGIP

GLGDSGWIAALSTLDNHSLAISVIMMVVAGFFTLCAVLSVFLLQRVHSLYRRTGASFQQA

QEEFSQGIFSSRTFHRAASSAAQGAFQGN

>sp|P06734|FCER2_HUMAN 163 CPEKWINFQRKCYYFGKG

MEEGQYSEIEELPRRRCCRRGTQIVLLGLVTAALWAGLLTLLLLWHWDTTQSLKQLEERA

ARNVSQVSKNLESHHGDQMAQKSQSTQISQELEELRAEQQRLKSQDLELSWNLNGLQADL

SSFKSQELNERNEASDLLERLREEVTKLRMELQVSSGFVCNTCPEKWINFQRKCYYFGKG

TKQWVHARYACDDMEGQLVSIHSPEEQDFLTKHASHTGSWIGLRNLDLKGEFIWVDGSHV

DYSNWAPGEPTSRSQGEDCVMMRGSGRWNDAFCDRKLGAWVCDRLATCTPPASEGSAESM

GPDSRPDPDGRLPTPSAPLHS

>sp|P06734|FCER2_HUMAN 206 EQDFLTKHASHTGSWIG

MEEGQYSEIEELPRRRCCRRGTQIVLLGLVTAALWAGLLTLLLLWHWDTTQSLKQLEERA

ARNVSQVSKNLESHHGDQMAQKSQSTQISQELEELRAEQQRLKSQDLELSWNLNGLQADL

SSFKSQELNERNEASDLLERLREEVTKLRMELQVSSGFVCNTCPEKWINFQRKCYYFGKG

TKQWVHARYACDDMEGQLVSIHSPEEQDFLTKHASHTGSWIGLRNLDLKGEFIWVDGSHV

DYSNWAPGEPTSRSQGEDCVMMRGSGRWNDAFCDRKLGAWVCDRLATCTPPASEGSAESM

GPDSRPDPDGRLPTPSAPLHS

>sp|P06734|FCER2_HUMAN 103 KSQDLELSWNLNGLQADLS

MEEGQYSEIEELPRRRCCRRGTQIVLLGLVTAALWAGLLTLLLLWHWDTTQSLKQLEERA

ARNVSQVSKNLESHHGDQMAQKSQSTQISQELEELRAEQQRLKSQDLELSWNLNGLQADL

SSFKSQELNERNEASDLLERLREEVTKLRMELQVSSGFVCNTCPEKWINFQRKCYYFGKG

TKQWVHARYACDDMEGQLVSIHSPEEQDFLTKHASHTGSWIGLRNLDLKGEFIWVDGSHV

DYSNWAPGEPTSRSQGEDCVMMRGSGRWNDAFCDRKLGAWVCDRLATCTPPASEGSAESM

GPDSRPDPDGRLPTPSAPLHS

>sp|P06734|FCER2_HUMAN 103 KSQDLELSWNLNGLQADLSS

MEEGQYSEIEELPRRRCCRRGTQIVLLGLVTAALWAGLLTLLLLWHWDTTQSLKQLEERA

ARNVSQVSKNLESHHGDQMAQKSQSTQISQELEELRAEQQRLKSQDLELSWNLNGLQADL

SSFKSQELNERNEASDLLERLREEVTKLRMELQVSSGFVCNTCPEKWINFQRKCYYFGKG

TKQWVHARYACDDMEGQLVSIHSPEEQDFLTKHASHTGSWIGLRNLDLKGEFIWVDGSHV

DYSNWAPGEPTSRSQGEDCVMMRGSGRWNDAFCDRKLGAWVCDRLATCTPPASEGSAESM

GPDSRPDPDGRLPTPSAPLHS

>sp|P06734|FCER2_HUMAN 102 LKSQDLELSWNLNGLQADLS

MEEGQYSEIEELPRRRCCRRGTQIVLLGLVTAALWAGLLTLLLLWHWDTTQSLKQLEERA

ARNVSQVSKNLESHHGDQMAQKSQSTQISQELEELRAEQQRLKSQDLELSWNLNGLQADL

SSFKSQELNERNEASDLLERLREEVTKLRMELQVSSGFVCNTCPEKWINFQRKCYYFGKG

TKQWVHARYACDDMEGQLVSIHSPEEQDFLTKHASHTGSWIGLRNLDLKGEFIWVDGSHV

DYSNWAPGEPTSRSQGEDCVMMRGSGRWNDAFCDRKLGAWVCDRLATCTPPASEGSAESM

GPDSRPDPDGRLPTPSAPLHS

>sp|P06734|FCER2_HUMAN 102 LKSQDLELSWNLNGLQADLSS

MEEGQYSEIEELPRRRCCRRGTQIVLLGLVTAALWAGLLTLLLLWHWDTTQSLKQLEERA

ARNVSQVSKNLESHHGDQMAQKSQSTQISQELEELRAEQQRLKSQDLELSWNLNGLQADL

SSFKSQELNERNEASDLLERLREEVTKLRMELQVSSGFVCNTCPEKWINFQRKCYYFGKG

TKQWVHARYACDDMEGQLVSIHSPEEQDFLTKHASHTGSWIGLRNLDLKGEFIWVDGSHV

DYSNWAPGEPTSRSQGEDCVMMRGSGRWNDAFCDRKLGAWVCDRLATCTPPASEGSAESM

GPDSRPDPDGRLPTPSAPLHS

>sp|P06734|FCER2_HUMAN 207 QDFLTKHASHTGSWIG

MEEGQYSEIEELPRRRCCRRGTQIVLLGLVTAALWAGLLTLLLLWHWDTTQSLKQLEERA

ARNVSQVSKNLESHHGDQMAQKSQSTQISQELEELRAEQQRLKSQDLELSWNLNGLQADL

SSFKSQELNERNEASDLLERLREEVTKLRMELQVSSGFVCNTCPEKWINFQRKCYYFGKG

TKQWVHARYACDDMEGQLVSIHSPEEQDFLTKHASHTGSWIGLRNLDLKGEFIWVDGSHV

DYSNWAPGEPTSRSQGEDCVMMRGSGRWNDAFCDRKLGAWVCDRLATCTPPASEGSAESM

GPDSRPDPDGRLPTPSAPLHS

>sp|P06734|FCER2_HUMAN 105 QDLELSWNLNGLQ

MEEGQYSEIEELPRRRCCRRGTQIVLLGLVTAALWAGLLTLLLLWHWDTTQSLKQLEERA

ARNVSQVSKNLESHHGDQMAQKSQSTQISQELEELRAEQQRLKSQDLELSWNLNGLQADL

SSFKSQELNERNEASDLLERLREEVTKLRMELQVSSGFVCNTCPEKWINFQRKCYYFGKG

TKQWVHARYACDDMEGQLVSIHSPEEQDFLTKHASHTGSWIGLRNLDLKGEFIWVDGSHV

DYSNWAPGEPTSRSQGEDCVMMRGSGRWNDAFCDRKLGAWVCDRLATCTPPASEGSAESM

GPDSRPDPDGRLPTPSAPLHS

>sp|P06734|FCER2_HUMAN 105 QDLELSWNLNGLQA

MEEGQYSEIEELPRRRCCRRGTQIVLLGLVTAALWAGLLTLLLLWHWDTTQSLKQLEERA

ARNVSQVSKNLESHHGDQMAQKSQSTQISQELEELRAEQQRLKSQDLELSWNLNGLQADL

SSFKSQELNERNEASDLLERLREEVTKLRMELQVSSGFVCNTCPEKWINFQRKCYYFGKG

TKQWVHARYACDDMEGQLVSIHSPEEQDFLTKHASHTGSWIGLRNLDLKGEFIWVDGSHV

DYSNWAPGEPTSRSQGEDCVMMRGSGRWNDAFCDRKLGAWVCDRLATCTPPASEGSAESM

GPDSRPDPDGRLPTPSAPLHS

>sp|P06734|FCER2_HUMAN 105 QDLELSWNLNGLQADL

MEEGQYSEIEELPRRRCCRRGTQIVLLGLVTAALWAGLLTLLLLWHWDTTQSLKQLEERA

ARNVSQVSKNLESHHGDQMAQKSQSTQISQELEELRAEQQRLKSQDLELSWNLNGLQADL

SSFKSQELNERNEASDLLERLREEVTKLRMELQVSSGFVCNTCPEKWINFQRKCYYFGKG

TKQWVHARYACDDMEGQLVSIHSPEEQDFLTKHASHTGSWIGLRNLDLKGEFIWVDGSHV

DYSNWAPGEPTSRSQGEDCVMMRGSGRWNDAFCDRKLGAWVCDRLATCTPPASEGSAESM

GPDSRPDPDGRLPTPSAPLHS

>sp|P06734|FCER2_HUMAN 203 SPEEQDFLTKHASHTGSWIG

MEEGQYSEIEELPRRRCCRRGTQIVLLGLVTAALWAGLLTLLLLWHWDTTQSLKQLEERA

ARNVSQVSKNLESHHGDQMAQKSQSTQISQELEELRAEQQRLKSQDLELSWNLNGLQADL

SSFKSQELNERNEASDLLERLREEVTKLRMELQVSSGFVCNTCPEKWINFQRKCYYFGKG

TKQWVHARYACDDMEGQLVSIHSPEEQDFLTKHASHTGSWIGLRNLDLKGEFIWVDGSHV

DYSNWAPGEPTSRSQGEDCVMMRGSGRWNDAFCDRKLGAWVCDRLATCTPPASEGSAESM

GPDSRPDPDGRLPTPSAPLHS

>sp|P06734|FCER2_HUMAN 104 SQDLELSWNLNGLQA

MEEGQYSEIEELPRRRCCRRGTQIVLLGLVTAALWAGLLTLLLLWHWDTTQSLKQLEERA

ARNVSQVSKNLESHHGDQMAQKSQSTQISQELEELRAEQQRLKSQDLELSWNLNGLQADL

SSFKSQELNERNEASDLLERLREEVTKLRMELQVSSGFVCNTCPEKWINFQRKCYYFGKG

TKQWVHARYACDDMEGQLVSIHSPEEQDFLTKHASHTGSWIGLRNLDLKGEFIWVDGSHV

DYSNWAPGEPTSRSQGEDCVMMRGSGRWNDAFCDRKLGAWVCDRLATCTPPASEGSAESM

GPDSRPDPDGRLPTPSAPLHS

>sp|P06734|FCER2_HUMAN 104 SQDLELSWNLNGLQADLS

MEEGQYSEIEELPRRRCCRRGTQIVLLGLVTAALWAGLLTLLLLWHWDTTQSLKQLEERA

ARNVSQVSKNLESHHGDQMAQKSQSTQISQELEELRAEQQRLKSQDLELSWNLNGLQADL

SSFKSQELNERNEASDLLERLREEVTKLRMELQVSSGFVCNTCPEKWINFQRKCYYFGKG

TKQWVHARYACDDMEGQLVSIHSPEEQDFLTKHASHTGSWIGLRNLDLKGEFIWVDGSHV

DYSNWAPGEPTSRSQGEDCVMMRGSGRWNDAFCDRKLGAWVCDRLATCTPPASEGSAESM

GPDSRPDPDGRLPTPSAPLHS

>sp|P06734|FCER2_HUMAN 104 SQDLELSWNLNGLQADLSS

MEEGQYSEIEELPRRRCCRRGTQIVLLGLVTAALWAGLLTLLLLWHWDTTQSLKQLEERA

ARNVSQVSKNLESHHGDQMAQKSQSTQISQELEELRAEQQRLKSQDLELSWNLNGLQADL

SSFKSQELNERNEASDLLERLREEVTKLRMELQVSSGFVCNTCPEKWINFQRKCYYFGKG

TKQWVHARYACDDMEGQLVSIHSPEEQDFLTKHASHTGSWIGLRNLDLKGEFIWVDGSHV

DYSNWAPGEPTSRSQGEDCVMMRGSGRWNDAFCDRKLGAWVCDRLATCTPPASEGSAESM

GPDSRPDPDGRLPTPSAPLHS

>sp|P06734|FCER2_HUMAN 104 SQDLELSWNLNGLQADLSSFK

MEEGQYSEIEELPRRRCCRRGTQIVLLGLVTAALWAGLLTLLLLWHWDTTQSLKQLEERA

ARNVSQVSKNLESHHGDQMAQKSQSTQISQELEELRAEQQRLKSQDLELSWNLNGLQADL

SSFKSQELNERNEASDLLERLREEVTKLRMELQVSSGFVCNTCPEKWINFQRKCYYFGKG

TKQWVHARYACDDMEGQLVSIHSPEEQDFLTKHASHTGSWIGLRNLDLKGEFIWVDGSHV

DYSNWAPGEPTSRSQGEDCVMMRGSGRWNDAFCDRKLGAWVCDRLATCTPPASEGSAESM

GPDSRPDPDGRLPTPSAPLHS

>sp|P43365|MAGAC_HUMAN 114 AELVHFLLLKYRAR

MPLEQRSQHCKPEEGLEAQGEALGLVGAQAPATEEQETASSSSTLVEVTLREVPAAESPS

PPHSPQGASTLPTTINYTLWSQSDEGSSNEEQEGPSTFPDLETSFQVALSRKMAELVHFL

LLKYRAREPFTKAEMLGSVIRNFQDFFPVIFSKASEYLQLVFGIEVVEVVRIGHLYILVT

CLGLSYDGLLGDNQIVPKTGLLIIVLAIIAKEGDCAPEEKIWEELSVLEASDGREDSVFA

HPRKLLTQDLVQENYLEYRQVPGSDPACYEFLWGPRALVETSYVKVLHHLLKISGGPHIS

YPPLHEWAFREGEE

>sp|P43360|MAGA6_HUMAN 121 LLKYRAREPVTKAE

MPLEQRSQHCKPEEGLEARGEALGLVGAQAPATEEQEAASSSSTLVEVTLGEVPAAESPD

PPQSPQGASSLPTTMNYPLWSQSYEDSSNQEEEGPSTFPDLESEFQAALSRKVAKLVHFL

LLKYRAREPVTKAEMLGSVVGNWQYFFPVIFSKASDSLQLVFGIELMEVDPIGHVYIFAT

CLGLSYDGLLGDNQIMPKTGFLIIILAIIAKEGDCAPEEKIWEELSVLEVFEGREDSIFG

DPKKLLTQYFVQENYLEYRQVPGSDPACYEFLWGPRALIETSYVKVLHHMVKISGGPRIS

YPLLHEWALREGEE

>sp|P02686|MBP_HUMAN 220 VVHFFKNIVTPRTPPPSQGK

MGNHAGKRELNAEKASTNSETNRGESEKKRNLGELSRTTSEDNEVFGEADANQNNGTSSQ

DTAVTDSKRTADPKNAWQDAHPADPGSRPHLIRLFSRDAPGREDNTFKDRPSESDELQTI

QEDSAATSESLDVMASQKRPSQRHGSKYLATASTMDHARHGFLPRHRDTGILDSIGRFFG

GDRGAPKRGSGKDSHHPARTAHYGSLPQKSHGRTQDENPVVHFFKNIVTPRTPPPSQGKG

RGLSLSRFSWGAEGQRPGFGYGGRASDYKSAHKGFKGVDAQGTLSKIFKLGGRDSRSGSP

MARR

>sp|Q9UBR2|CATZ_HUMAN 253 GTEYWIVRNSWGEPW

MARRGPGWRPLLLLVLLAGAAQGGLYFRRGQTCYRPLRGDGLAPLGRSTYPRPHEYLSPA

DLPKSWDWRNVDGVNYASITRNQHIPQYCGSCWAHASTSAMADRINIKRKGAWPSTLLSV

QNVIDCGNAGSCEGGNDLSVWDYAHQHGIPDETCNNYQAKDQECDKFNQCGTCNEFKECH

AIRNYTLWRVGDYGSLSGREKMMAEIYANGPISCGIMATERLANYTGGIYAEYQDTTYIN

HVVSVAGWGISDGTEYWIVRNSWGEPWGERGWLRIVTSTYKDGKGARYNLAIEEHCTFGD

PIV

>sp|P00387|NB5R3_HUMAN 155 GKFAIRPDKKSNPIIRTV

MGAQLSTLGHMVLFPVWFLYSLLMKLFQRSTPAITLESPDIKYPLRLIDREIISHDTRRF

RFALPSPQHILGLPVGQHIYLSARIDGNLVVRPYTPISSDDDKGFVDLVIKVYFKDTHPK

FPAGGKMSQYLESMQIGDTIEFRGPSGLLVYQGKGKFAIRPDKKSNPIIRTVKSVGMIAG

GTGITPMLQVIRAIMKDPDDHTVCHLLFANQTEKDILLRPELEELRNKHSARFKLWYTLD

RAPEAWDYGQGFVNEEMIRDHLPPPEEEPLVLMCGPPPMIQYACLPNLDHVGHPTERCFV

F

>sp|P28907|CD38_HUMAN 140 RDMFTLEDTL

MANCEFSPVSGDKPCCRLSRRAQLCLGVSILVLILVVVLAVVVPRWRQQWSGPGTTKRFP

ETVLARCVKYTEIHPEMRHVDCQSVWDAFKGAFISKHPCNITEEDYQPLMKLGTQTVPCN

KILLWSRIKDLAHQFTQVQRDMFTLEDTLLGYLADDLTWCGEFNTSKINYQSCPDWRKDC

SNNPVSVFWKTVSRRFAEAACDVVHVMLNGSRSKIFDKNSTFGSVEVHNLQPEKVQTLEA

WVIHGGREDSRDLCQDPTIKELESIISKRNIQFSCKNIYRPDKFLQCVKNPEDSSCTSEI

>sp|P28907|CD38_HUMAN 140 RDMFTLEDTLLG

MANCEFSPVSGDKPCCRLSRRAQLCLGVSILVLILVVVLAVVVPRWRQQWSGPGTTKRFP

ETVLARCVKYTEIHPEMRHVDCQSVWDAFKGAFISKHPCNITEEDYQPLMKLGTQTVPCN

KILLWSRIKDLAHQFTQVQRDMFTLEDTLLGYLADDLTWCGEFNTSKINYQSCPDWRKDC

SNNPVSVFWKTVSRRFAEAACDVVHVMLNGSRSKIFDKNSTFGSVEVHNLQPEKVQTLEA

WVIHGGREDSRDLCQDPTIKELESIISKRNIQFSCKNIYRPDKFLQCVKNPEDSSCTSEI

>sp|P28907|CD38_HUMAN 138 VQRDMFTLEDTLLGYLAD

MANCEFSPVSGDKPCCRLSRRAQLCLGVSILVLILVVVLAVVVPRWRQQWSGPGTTKRFP

ETVLARCVKYTEIHPEMRHVDCQSVWDAFKGAFISKHPCNITEEDYQPLMKLGTQTVPCN

KILLWSRIKDLAHQFTQVQRDMFTLEDTLLGYLADDLTWCGEFNTSKINYQSCPDWRKDC

SNNPVSVFWKTVSRRFAEAACDVVHVMLNGSRSKIFDKNSTFGSVEVHNLQPEKVQTLEA

WVIHGGREDSRDLCQDPTIKELESIISKRNIQFSCKNIYRPDKFLQCVKNPEDSSCTSEI

>sp|Q9H115|SNAB_HUMAN 149 ADYYKGEESNSSANK

MDNAGKEREAVQLMAEAEKRVKASHSFLRGLFGGNTRIEEACEMYTRAANMFKMAKNWSA

AGNAFCQAAKLHMQLQSKHDSATSFVDAGNAYKKADPQEAINCLNAAIDIYTDMGRFTIA

AKHHITIAEIYETELVDIEKAIAHYEQSADYYKGEESNSSANKCLLKVAAYAAQLEQYQK

AIEIYEQVGANTMDNPLLKYSAKDYFFKAALCHFIVDELNAKLALEKYEEMFPAFTDSRE

CKLLKKLLEAHEEQNSEAYTEAVKEFDSISRLDQWLTTMLLRIKKSIQGDGEGDGDLK

>sp|Q9H115|SNAB_HUMAN 150 DYYKGEESNSSAN

MDNAGKEREAVQLMAEAEKRVKASHSFLRGLFGGNTRIEEACEMYTRAANMFKMAKNWSA

AGNAFCQAAKLHMQLQSKHDSATSFVDAGNAYKKADPQEAINCLNAAIDIYTDMGRFTIA

AKHHITIAEIYETELVDIEKAIAHYEQSADYYKGEESNSSANKCLLKVAAYAAQLEQYQK

AIEIYEQVGANTMDNPLLKYSAKDYFFKAALCHFIVDELNAKLALEKYEEMFPAFTDSRE

CKLLKKLLEAHEEQNSEAYTEAVKEFDSISRLDQWLTTMLLRIKKSIQGDGEGDGDLK

>sp|Q9H115|SNAB_HUMAN 150 DYYKGEESNSSANK

MDNAGKEREAVQLMAEAEKRVKASHSFLRGLFGGNTRIEEACEMYTRAANMFKMAKNWSA

AGNAFCQAAKLHMQLQSKHDSATSFVDAGNAYKKADPQEAINCLNAAIDIYTDMGRFTIA

AKHHITIAEIYETELVDIEKAIAHYEQSADYYKGEESNSSANKCLLKVAAYAAQLEQYQK

AIEIYEQVGANTMDNPLLKYSAKDYFFKAALCHFIVDELNAKLALEKYEEMFPAFTDSRE

CKLLKKLLEAHEEQNSEAYTEAVKEFDSISRLDQWLTTMLLRIKKSIQGDGEGDGDLK

>sp|Q9H115|SNAB_HUMAN 148 SADYYKGEESNSSANK

MDNAGKEREAVQLMAEAEKRVKASHSFLRGLFGGNTRIEEACEMYTRAANMFKMAKNWSA

AGNAFCQAAKLHMQLQSKHDSATSFVDAGNAYKKADPQEAINCLNAAIDIYTDMGRFTIA

AKHHITIAEIYETELVDIEKAIAHYEQSADYYKGEESNSSANKCLLKVAAYAAQLEQYQK

AIEIYEQVGANTMDNPLLKYSAKDYFFKAALCHFIVDELNAKLALEKYEEMFPAFTDSRE

CKLLKKLLEAHEEQNSEAYTEAVKEFDSISRLDQWLTTMLLRIKKSIQGDGEGDGDLK

>sp|P11836|CD20_HUMAN 261 DIEIIPIQEE

MTTPRNSVNGTFPAEPMKGPIAMQSGPKPLFRRMSSLVGPTQSFFMRESKTLGAVQIMNG

LFHIALGGLLMIPAGIYAPICVTVWYPLWGGIMYIISGSLLAATEKNSRKCLVKGKMIMN

SLSLFAAISGMILSIMDILNIKISHFLKMESLNFIRAHTPYINIYNCEPANPSEKNSPST

QYCYSIQSLFLGILSVMLIFAFFQELVIAGIVENEWKRTCSRPKSNIVLLSAEEKKEQTI

EIKEEVVGLTETSSQPKNEEDIEIIPIQEEEEEETETNFPEPPQDQESSPIENDSSP

>sp|P11836|CD20_HUMAN 260 EDIEIIPIQEE

MTTPRNSVNGTFPAEPMKGPIAMQSGPKPLFRRMSSLVGPTQSFFMRESKTLGAVQIMNG

LFHIALGGLLMIPAGIYAPICVTVWYPLWGGIMYIISGSLLAATEKNSRKCLVKGKMIMN

SLSLFAAISGMILSIMDILNIKISHFLKMESLNFIRAHTPYINIYNCEPANPSEKNSPST

QYCYSIQSLFLGILSVMLIFAFFQELVIAGIVENEWKRTCSRPKSNIVLLSAEEKKEQTI

EIKEEVVGLTETSSQPKNEEDIEIIPIQEEEEEETETNFPEPPQDQESSPIENDSSP

>sp|P11836|CD20_HUMAN 25 SGPKPLFRRMSSLVGPTQSF

MTTPRNSVNGTFPAEPMKGPIAMQSGPKPLFRRMSSLVGPTQSFFMRESKTLGAVQIMNG

LFHIALGGLLMIPAGIYAPICVTVWYPLWGGIMYIISGSLLAATEKNSRKCLVKGKMIMN

SLSLFAAISGMILSIMDILNIKISHFLKMESLNFIRAHTPYINIYNCEPANPSEKNSPST

QYCYSIQSLFLGILSVMLIFAFFQELVIAGIVENEWKRTCSRPKSNIVLLSAEEKKEQTI

EIKEEVVGLTETSSQPKNEEDIEIIPIQEEEEEETETNFPEPPQDQESSPIENDSSP

>sp|P04233|HG2A_HUMAN 131 ATKYGNMTEDHVMHLLQNA

MHRRRSRSCREDQKPVMDDQRDLISNNEQLPMLGRRPGAPESKCSRGALYTGFSILVTLL

LAGQATTAYFLYQQQGRLDKLTVTSQNLQLENLRMKLPKPPKPVSKMRMATPLLMQALPM

GALPQGPMQNATKYGNMTEDHVMHLLQNADPLKVYPPLKGSFPENLRHLKNTMETIDWKV

FESWMHHWLLFEMSRHSLEQKPTDAPPKVLTKCQEEVSHIPAVHPGSFRPKCDENGNYLP

LQCYGSIGYCWCVFPNGTEVPNTRSRGHHNCSESLELEDPSSGLGVTKQDLGPVPM

>sp|P04233|HG2A_HUMAN 110 ATPLLMQALPMGALPQGP

MHRRRSRSCREDQKPVMDDQRDLISNNEQLPMLGRRPGAPESKCSRGALYTGFSILVTLL

LAGQATTAYFLYQQQGRLDKLTVTSQNLQLENLRMKLPKPPKPVSKMRMATPLLMQALPM

GALPQGPMQNATKYGNMTEDHVMHLLQNADPLKVYPPLKGSFPENLRHLKNTMETIDWKV

FESWMHHWLLFEMSRHSLEQKPTDAPPKVLTKCQEEVSHIPAVHPGSFRPKCDENGNYLP

LQCYGSIGYCWCVFPNGTEVPNTRSRGHHNCSESLELEDPSSGLGVTKQDLGPVPM

>sp|P04233|HG2A_HUMAN 186 HHWLLFEMSRHSLE

MHRRRSRSCREDQKPVMDDQRDLISNNEQLPMLGRRPGAPESKCSRGALYTGFSILVTLL

LAGQATTAYFLYQQQGRLDKLTVTSQNLQLENLRMKLPKPPKPVSKMRMATPLLMQALPM

GALPQGPMQNATKYGNMTEDHVMHLLQNADPLKVYPPLKGSFPENLRHLKNTMETIDWKV

FESWMHHWLLFEMSRHSLEQKPTDAPPKVLTKCQEEVSHIPAVHPGSFRPKCDENGNYLP

LQCYGSIGYCWCVFPNGTEVPNTRSRGHHNCSESLELEDPSSGLGVTKQDLGPVPM

>sp|P04233|HG2A_HUMAN 99 KPPKPVSKMRMATP

MHRRRSRSCREDQKPVMDDQRDLISNNEQLPMLGRRPGAPESKCSRGALYTGFSILVTLL

LAGQATTAYFLYQQQGRLDKLTVTSQNLQLENLRMKLPKPPKPVSKMRMATPLLMQALPM

GALPQGPMQNATKYGNMTEDHVMHLLQNADPLKVYPPLKGSFPENLRHLKNTMETIDWKV

FESWMHHWLLFEMSRHSLEQKPTDAPPKVLTKCQEEVSHIPAVHPGSFRPKCDENGNYLP

LQCYGSIGYCWCVFPNGTEVPNTRSRGHHNCSESLELEDPSSGLGVTKQDLGPVPM

>sp|P04233|HG2A_HUMAN 99 KPPKPVSKMRMATPLLMQ

MHRRRSRSCREDQKPVMDDQRDLISNNEQLPMLGRRPGAPESKCSRGALYTGFSILVTLL

LAGQATTAYFLYQQQGRLDKLTVTSQNLQLENLRMKLPKPPKPVSKMRMATPLLMQALPM

GALPQGPMQNATKYGNMTEDHVMHLLQNADPLKVYPPLKGSFPENLRHLKNTMETIDWKV

FESWMHHWLLFEMSRHSLEQKPTDAPPKVLTKCQEEVSHIPAVHPGSFRPKCDENGNYLP

LQCYGSIGYCWCVFPNGTEVPNTRSRGHHNCSESLELEDPSSGLGVTKQDLGPVPM

>sp|P04233|HG2A_HUMAN 99 KPPKPVSKMRMATPLLMQA

MHRRRSRSCREDQKPVMDDQRDLISNNEQLPMLGRRPGAPESKCSRGALYTGFSILVTLL

LAGQATTAYFLYQQQGRLDKLTVTSQNLQLENLRMKLPKPPKPVSKMRMATPLLMQALPM

GALPQGPMQNATKYGNMTEDHVMHLLQNADPLKVYPPLKGSFPENLRHLKNTMETIDWKV

FESWMHHWLLFEMSRHSLEQKPTDAPPKVLTKCQEEVSHIPAVHPGSFRPKCDENGNYLP

LQCYGSIGYCWCVFPNGTEVPNTRSRGHHNCSESLELEDPSSGLGVTKQDLGPVPM

>sp|P04233|HG2A_HUMAN 99 KPPKPVSKMRMATPLLMQALP

MHRRRSRSCREDQKPVMDDQRDLISNNEQLPMLGRRPGAPESKCSRGALYTGFSILVTLL

LAGQATTAYFLYQQQGRLDKLTVTSQNLQLENLRMKLPKPPKPVSKMRMATPLLMQALPM

GALPQGPMQNATKYGNMTEDHVMHLLQNADPLKVYPPLKGSFPENLRHLKNTMETIDWKV

FESWMHHWLLFEMSRHSLEQKPTDAPPKVLTKCQEEVSHIPAVHPGSFRPKCDENGNYLP

LQCYGSIGYCWCVFPNGTEVPNTRSRGHHNCSESLELEDPSSGLGVTKQDLGPVPM

>sp|P04233|HG2A_HUMAN 99 KPPKPVSKMRMATPLLMQALPM

MHRRRSRSCREDQKPVMDDQRDLISNNEQLPMLGRRPGAPESKCSRGALYTGFSILVTLL

LAGQATTAYFLYQQQGRLDKLTVTSQNLQLENLRMKLPKPPKPVSKMRMATPLLMQALPM

GALPQGPMQNATKYGNMTEDHVMHLLQNADPLKVYPPLKGSFPENLRHLKNTMETIDWKV

FESWMHHWLLFEMSRHSLEQKPTDAPPKVLTKCQEEVSHIPAVHPGSFRPKCDENGNYLP

LQCYGSIGYCWCVFPNGTEVPNTRSRGHHNCSESLELEDPSSGLGVTKQDLGPVPM

>sp|P04233|HG2A_HUMAN 97 LPKPPKPVSKMRMATPL

MHRRRSRSCREDQKPVMDDQRDLISNNEQLPMLGRRPGAPESKCSRGALYTGFSILVTLL

LAGQATTAYFLYQQQGRLDKLTVTSQNLQLENLRMKLPKPPKPVSKMRMATPLLMQALPM

GALPQGPMQNATKYGNMTEDHVMHLLQNADPLKVYPPLKGSFPENLRHLKNTMETIDWKV

FESWMHHWLLFEMSRHSLEQKPTDAPPKVLTKCQEEVSHIPAVHPGSFRPKCDENGNYLP

LQCYGSIGYCWCVFPNGTEVPNTRSRGHHNCSESLELEDPSSGLGVTKQDLGPVPM

>sp|P04233|HG2A_HUMAN 97 LPKPPKPVSKMRMATPLLM

MHRRRSRSCREDQKPVMDDQRDLISNNEQLPMLGRRPGAPESKCSRGALYTGFSILVTLL

LAGQATTAYFLYQQQGRLDKLTVTSQNLQLENLRMKLPKPPKPVSKMRMATPLLMQALPM

GALPQGPMQNATKYGNMTEDHVMHLLQNADPLKVYPPLKGSFPENLRHLKNTMETIDWKV

FESWMHHWLLFEMSRHSLEQKPTDAPPKVLTKCQEEVSHIPAVHPGSFRPKCDENGNYLP

LQCYGSIGYCWCVFPNGTEVPNTRSRGHHNCSESLELEDPSSGLGVTKQDLGPVPM

>sp|P04233|HG2A_HUMAN 97 LPKPPKPVSKMRMATPLLMQ

MHRRRSRSCREDQKPVMDDQRDLISNNEQLPMLGRRPGAPESKCSRGALYTGFSILVTLL

LAGQATTAYFLYQQQGRLDKLTVTSQNLQLENLRMKLPKPPKPVSKMRMATPLLMQALPM

GALPQGPMQNATKYGNMTEDHVMHLLQNADPLKVYPPLKGSFPENLRHLKNTMETIDWKV

FESWMHHWLLFEMSRHSLEQKPTDAPPKVLTKCQEEVSHIPAVHPGSFRPKCDENGNYLP

LQCYGSIGYCWCVFPNGTEVPNTRSRGHHNCSESLELEDPSSGLGVTKQDLGPVPM

>sp|P04233|HG2A_HUMAN 97 LPKPPKPVSKMRMATPLLMQAL

MHRRRSRSCREDQKPVMDDQRDLISNNEQLPMLGRRPGAPESKCSRGALYTGFSILVTLL

LAGQATTAYFLYQQQGRLDKLTVTSQNLQLENLRMKLPKPPKPVSKMRMATPLLMQALPM

GALPQGPMQNATKYGNMTEDHVMHLLQNADPLKVYPPLKGSFPENLRHLKNTMETIDWKV

FESWMHHWLLFEMSRHSLEQKPTDAPPKVLTKCQEEVSHIPAVHPGSFRPKCDENGNYLP

LQCYGSIGYCWCVFPNGTEVPNTRSRGHHNCSESLELEDPSSGLGVTKQDLGPVPM

>sp|P04233|HG2A_HUMAN 97 LPKPPKPVSKMRMATPLLMQALP

MHRRRSRSCREDQKPVMDDQRDLISNNEQLPMLGRRPGAPESKCSRGALYTGFSILVTLL

LAGQATTAYFLYQQQGRLDKLTVTSQNLQLENLRMKLPKPPKPVSKMRMATPLLMQALPM

GALPQGPMQNATKYGNMTEDHVMHLLQNADPLKVYPPLKGSFPENLRHLKNTMETIDWKV

FESWMHHWLLFEMSRHSLEQKPTDAPPKVLTKCQEEVSHIPAVHPGSFRPKCDENGNYLP

LQCYGSIGYCWCVFPNGTEVPNTRSRGHHNCSESLELEDPSSGLGVTKQDLGPVPM

>sp|P04233|HG2A_HUMAN 97 LPKPPKPVSKMRMATPLLMQALPM

MHRRRSRSCREDQKPVMDDQRDLISNNEQLPMLGRRPGAPESKCSRGALYTGFSILVTLL

LAGQATTAYFLYQQQGRLDKLTVTSQNLQLENLRMKLPKPPKPVSKMRMATPLLMQALPM

GALPQGPMQNATKYGNMTEDHVMHLLQNADPLKVYPPLKGSFPENLRHLKNTMETIDWKV

FESWMHHWLLFEMSRHSLEQKPTDAPPKVLTKCQEEVSHIPAVHPGSFRPKCDENGNYLP

LQCYGSIGYCWCVFPNGTEVPNTRSRGHHNCSESLELEDPSSGLGVTKQDLGPVPM

>sp|P04233|HG2A_HUMAN 109 MATPLLMQALPMGAL

MHRRRSRSCREDQKPVMDDQRDLISNNEQLPMLGRRPGAPESKCSRGALYTGFSILVTLL

LAGQATTAYFLYQQQGRLDKLTVTSQNLQLENLRMKLPKPPKPVSKMRMATPLLMQALPM

GALPQGPMQNATKYGNMTEDHVMHLLQNADPLKVYPPLKGSFPENLRHLKNTMETIDWKV

FESWMHHWLLFEMSRHSLEQKPTDAPPKVLTKCQEEVSHIPAVHPGSFRPKCDENGNYLP

LQCYGSIGYCWCVFPNGTEVPNTRSRGHHNCSESLELEDPSSGLGVTKQDLGPVPM

>sp|P04233|HG2A_HUMAN 109 MATPLLMQALPMGALPQ

MHRRRSRSCREDQKPVMDDQRDLISNNEQLPMLGRRPGAPESKCSRGALYTGFSILVTLL

LAGQATTAYFLYQQQGRLDKLTVTSQNLQLENLRMKLPKPPKPVSKMRMATPLLMQALPM

GALPQGPMQNATKYGNMTEDHVMHLLQNADPLKVYPPLKGSFPENLRHLKNTMETIDWKV

FESWMHHWLLFEMSRHSLEQKPTDAPPKVLTKCQEEVSHIPAVHPGSFRPKCDENGNYLP

LQCYGSIGYCWCVFPNGTEVPNTRSRGHHNCSESLELEDPSSGLGVTKQDLGPVPM

>sp|P04233|HG2A_HUMAN 185 MHHWLLFEMSRHSLE

MHRRRSRSCREDQKPVMDDQRDLISNNEQLPMLGRRPGAPESKCSRGALYTGFSILVTLL

LAGQATTAYFLYQQQGRLDKLTVTSQNLQLENLRMKLPKPPKPVSKMRMATPLLMQALPM

GALPQGPMQNATKYGNMTEDHVMHLLQNADPLKVYPPLKGSFPENLRHLKNTMETIDWKV

FESWMHHWLLFEMSRHSLEQKPTDAPPKVLTKCQEEVSHIPAVHPGSFRPKCDENGNYLP

LQCYGSIGYCWCVFPNGTEVPNTRSRGHHNCSESLELEDPSSGLGVTKQDLGPVPM

>sp|P04233|HG2A_HUMAN 98 PKPPKPVSKMRMATPL

MHRRRSRSCREDQKPVMDDQRDLISNNEQLPMLGRRPGAPESKCSRGALYTGFSILVTLL

LAGQATTAYFLYQQQGRLDKLTVTSQNLQLENLRMKLPKPPKPVSKMRMATPLLMQALPM

GALPQGPMQNATKYGNMTEDHVMHLLQNADPLKVYPPLKGSFPENLRHLKNTMETIDWKV

FESWMHHWLLFEMSRHSLEQKPTDAPPKVLTKCQEEVSHIPAVHPGSFRPKCDENGNYLP

LQCYGSIGYCWCVFPNGTEVPNTRSRGHHNCSESLELEDPSSGLGVTKQDLGPVPM

>sp|P04233|HG2A_HUMAN 98 PKPPKPVSKMRMATPLLMQA

MHRRRSRSCREDQKPVMDDQRDLISNNEQLPMLGRRPGAPESKCSRGALYTGFSILVTLL

LAGQATTAYFLYQQQGRLDKLTVTSQNLQLENLRMKLPKPPKPVSKMRMATPLLMQALPM

GALPQGPMQNATKYGNMTEDHVMHLLQNADPLKVYPPLKGSFPENLRHLKNTMETIDWKV

FESWMHHWLLFEMSRHSLEQKPTDAPPKVLTKCQEEVSHIPAVHPGSFRPKCDENGNYLP

LQCYGSIGYCWCVFPNGTEVPNTRSRGHHNCSESLELEDPSSGLGVTKQDLGPVPM

>sp|P04233|HG2A_HUMAN 98 PKPPKPVSKMRMATPLLMQALP

MHRRRSRSCREDQKPVMDDQRDLISNNEQLPMLGRRPGAPESKCSRGALYTGFSILVTLL

LAGQATTAYFLYQQQGRLDKLTVTSQNLQLENLRMKLPKPPKPVSKMRMATPLLMQALPM

GALPQGPMQNATKYGNMTEDHVMHLLQNADPLKVYPPLKGSFPENLRHLKNTMETIDWKV

FESWMHHWLLFEMSRHSLEQKPTDAPPKVLTKCQEEVSHIPAVHPGSFRPKCDENGNYLP

LQCYGSIGYCWCVFPNGTEVPNTRSRGHHNCSESLELEDPSSGLGVTKQDLGPVPM

>sp|P04233|HG2A_HUMAN 108 RMATPLLMQAL

MHRRRSRSCREDQKPVMDDQRDLISNNEQLPMLGRRPGAPESKCSRGALYTGFSILVTLL

LAGQATTAYFLYQQQGRLDKLTVTSQNLQLENLRMKLPKPPKPVSKMRMATPLLMQALPM

GALPQGPMQNATKYGNMTEDHVMHLLQNADPLKVYPPLKGSFPENLRHLKNTMETIDWKV

FESWMHHWLLFEMSRHSLEQKPTDAPPKVLTKCQEEVSHIPAVHPGSFRPKCDENGNYLP

LQCYGSIGYCWCVFPNGTEVPNTRSRGHHNCSESLELEDPSSGLGVTKQDLGPVPM

>sp|P04233|HG2A_HUMAN 108 RMATPLLMQALPMGAL

MHRRRSRSCREDQKPVMDDQRDLISNNEQLPMLGRRPGAPESKCSRGALYTGFSILVTLL

LAGQATTAYFLYQQQGRLDKLTVTSQNLQLENLRMKLPKPPKPVSKMRMATPLLMQALPM

GALPQGPMQNATKYGNMTEDHVMHLLQNADPLKVYPPLKGSFPENLRHLKNTMETIDWKV

FESWMHHWLLFEMSRHSLEQKPTDAPPKVLTKCQEEVSHIPAVHPGSFRPKCDENGNYLP

LQCYGSIGYCWCVFPNGTEVPNTRSRGHHNCSESLELEDPSSGLGVTKQDLGPVPM

>sp|P04233|HG2A_HUMAN 111 TPLLMQALPMGALPQ

MHRRRSRSCREDQKPVMDDQRDLISNNEQLPMLGRRPGAPESKCSRGALYTGFSILVTLL

LAGQATTAYFLYQQQGRLDKLTVTSQNLQLENLRMKLPKPPKPVSKMRMATPLLMQALPM

GALPQGPMQNATKYGNMTEDHVMHLLQNADPLKVYPPLKGSFPENLRHLKNTMETIDWKV

FESWMHHWLLFEMSRHSLEQKPTDAPPKVLTKCQEEVSHIPAVHPGSFRPKCDENGNYLP

LQCYGSIGYCWCVFPNGTEVPNTRSRGHHNCSESLELEDPSSGLGVTKQDLGPVPM

>sp|P04233|HG2A_HUMAN 111 TPLLMQALPMGALPQG

MHRRRSRSCREDQKPVMDDQRDLISNNEQLPMLGRRPGAPESKCSRGALYTGFSILVTLL

LAGQATTAYFLYQQQGRLDKLTVTSQNLQLENLRMKLPKPPKPVSKMRMATPLLMQALPM

GALPQGPMQNATKYGNMTEDHVMHLLQNADPLKVYPPLKGSFPENLRHLKNTMETIDWKV

FESWMHHWLLFEMSRHSLEQKPTDAPPKVLTKCQEEVSHIPAVHPGSFRPKCDENGNYLP

LQCYGSIGYCWCVFPNGTEVPNTRSRGHHNCSESLELEDPSSGLGVTKQDLGPVPM

>sp|P04233|HG2A_HUMAN 66 TTAYFLYQQQGRLDK

MHRRRSRSCREDQKPVMDDQRDLISNNEQLPMLGRRPGAPESKCSRGALYTGFSILVTLL

LAGQATTAYFLYQQQGRLDKLTVTSQNLQLENLRMKLPKPPKPVSKMRMATPLLMQALPM

GALPQGPMQNATKYGNMTEDHVMHLLQNADPLKVYPPLKGSFPENLRHLKNTMETIDWKV

FESWMHHWLLFEMSRHSLEQKPTDAPPKVLTKCQEEVSHIPAVHPGSFRPKCDENGNYLP

LQCYGSIGYCWCVFPNGTEVPNTRSRGHHNCSESLELEDPSSGLGVTKQDLGPVPM

>sp|P04233|HG2A_HUMAN 66 TTAYFLYQQQGRLDKLTV

MHRRRSRSCREDQKPVMDDQRDLISNNEQLPMLGRRPGAPESKCSRGALYTGFSILVTLL

LAGQATTAYFLYQQQGRLDKLTVTSQNLQLENLRMKLPKPPKPVSKMRMATPLLMQALPM

GALPQGPMQNATKYGNMTEDHVMHLLQNADPLKVYPPLKGSFPENLRHLKNTMETIDWKV

FESWMHHWLLFEMSRHSLEQKPTDAPPKVLTKCQEEVSHIPAVHPGSFRPKCDENGNYLP

LQCYGSIGYCWCVFPNGTEVPNTRSRGHHNCSESLELEDPSSGLGVTKQDLGPVPM

>sp|P04233|HG2A_HUMAN 188 WLLFEMSRHSLEQKP

MHRRRSRSCREDQKPVMDDQRDLISNNEQLPMLGRRPGAPESKCSRGALYTGFSILVTLL

LAGQATTAYFLYQQQGRLDKLTVTSQNLQLENLRMKLPKPPKPVSKMRMATPLLMQALPM

GALPQGPMQNATKYGNMTEDHVMHLLQNADPLKVYPPLKGSFPENLRHLKNTMETIDWKV

FESWMHHWLLFEMSRHSLEQKPTDAPPKVLTKCQEEVSHIPAVHPGSFRPKCDENGNYLP

LQCYGSIGYCWCVFPNGTEVPNTRSRGHHNCSESLELEDPSSGLGVTKQDLGPVPM

>sp|P24385|CCND1_HUMAN 198 NPPSMVAAGSVVAAV

MEHQLLCCEVETIRRAYPDANLLNDRVLRAMLKAEETCAPSVSYFKCVQKEVLPSMRKIV

ATWMLEVCEEQKCEEEVFPLAMNYLDRFLSLEPVKKSRLQLLGATCMFVASKMKETIPLT

AEKLCIYTDNSIRPEELLQMELLLVNKLKWNLAAMTPHDFIEHFLSKMPEAEENKQIIRK

HAQTFVALCATDVKFISNPPSMVAAGSVVAAVQGLNLRSPNNFLSYYRLTRFLSRVIKCD

PDCLRACQEQIEALLESSLRQAQQNMDPKAAEEEEEEEEEVDLACTPTDVRDVDI

>sp|P45880|VDAC2_HUMAN 233 AAKYQLDPTASISA

MATHGQTCARPMCIPPSYADLGKAARDIFNKGFGFGLVKLDVKTKSCSGVEFSTSGSSNT

DTGKVTGTLETKYKWCEYGLTFTEKWNTDNTLGTEIAIEDQICQGLKLTFDTTFSPNTGK

KSGKIKSSYKRECINLGCDVDFDFAGPAIHGSAVFGYEGWLAGYQMTFDSAKSKLTRNNF

AVGYRTGDFQLHTNVNDGTEFGGSIYQKVCEDLDTSVNLAWTSGTNCTRFGIAAKYQLDP

TASISAKVNNSSLIGVGYTQTLRPGVKLTLSALVDGKSINAGGHKVGLALELEA

>sp|P29218|IMPA1_HUMAN 62 YPSHSFIGEESVAAGEK

MADPWQECMDYAVTLARQAGEVVCEAIKNEMNVMLKSSPVDLVTATDQKVEKMLISSIKE

KYPSHSFIGEESVAAGEKSILTDNPTWIIDPIDGTTNFVHRFPFVAVSIGFAVNKKIEFG

VVYSCVEGKMYTARKGKGAFCNGQKLQVSQQEDITKSLLVTELGSSRTPETVRMVLSNME

KLFCIPVHGIRSVGTAAVNMCLVATGGADAYYEMGIHCWDVAGAGIIVTEAGGVLMDVTG

GPFDLMSRRVIAANNRILAERIAKEIQVIPLQRDDED

>sp|P05538|HB2X_HUMAN 210 SLQSPITVEWRAQSESAQSKMLSGIGGFVL

MSWKMALQIPGGFWAAAVTVMLVMLSTPVAEARDFPKDFLVQFKGMCYFTNGTERVRGVA

RYIYNREEYGRFDSDVGEFQAVTELGRSIEDWNNYKDFLEQERAAVDKVCRHNYEAELRT

TLQRQVEPTVTISPSRTEALNHHNLLVCSVTDFYPAQIKVRWFRNDQEETAGVVSTSLIR

NGDWTFQILVMLEITPQRGDIYTCQVEHPSLQSPITVEWRAQSESAQSKMLSGIGGFVLG

LIFLGLGLIIRHRGQKGPRGPPPAGLLH

>sp|P13762|HB2K_HUMAN 250 FRNQKGHSGLQP

MVCLKLPGGSCMAALTVTLTVLSSPLALAGDTQPRFLEQAKCECHFLNGTERVWNLIRYI

YNQEEYARYNSDLGEYQAVTELGRPDAEYWNSQKDLLERRRAEVDTYCRYNYGVVESFTV

QRRVQPKVTVYPSKTQPLQHHNLLVCSVNGFYPGSIEVRWFRNSQEEKAGVVSTGLIQNG

DWTFQTLVMLETVPRSGEVYTCQVEHPSMMSPLTVQWSARSESAQSKMLSGVGGFVLGLL

FLGTGLFIYFRNQKGHSGLQPTGLLS

>sp|P13762|HB2K_HUMAN 248 IYFRNQKGHSGLQP

MVCLKLPGGSCMAALTVTLTVLSSPLALAGDTQPRFLEQAKCECHFLNGTERVWNLIRYI

YNQEEYARYNSDLGEYQAVTELGRPDAEYWNSQKDLLERRRAEVDTYCRYNYGVVESFTV

QRRVQPKVTVYPSKTQPLQHHNLLVCSVNGFYPGSIEVRWFRNSQEEKAGVVSTGLIQNG

DWTFQTLVMLETVPRSGEVYTCQVEHPSMMSPLTVQWSARSESAQSKMLSGVGGFVLGLL

FLGTGLFIYFRNQKGHSGLQPTGLLS

>sp|P13762|HB2K_HUMAN 123 RVQPKVTVYPSKTQP

MVCLKLPGGSCMAALTVTLTVLSSPLALAGDTQPRFLEQAKCECHFLNGTERVWNLIRYI

YNQEEYARYNSDLGEYQAVTELGRPDAEYWNSQKDLLERRRAEVDTYCRYNYGVVESFTV

QRRVQPKVTVYPSKTQPLQHHNLLVCSVNGFYPGSIEVRWFRNSQEEKAGVVSTGLIQNG

DWTFQTLVMLETVPRSGEVYTCQVEHPSMMSPLTVQWSARSESAQSKMLSGVGGFVLGLL

FLGTGLFIYFRNQKGHSGLQPTGLLS

>sp|P13762|HB2K_HUMAN 123 RVQPKVTVYPSKTQPLQH

MVCLKLPGGSCMAALTVTLTVLSSPLALAGDTQPRFLEQAKCECHFLNGTERVWNLIRYI

YNQEEYARYNSDLGEYQAVTELGRPDAEYWNSQKDLLERRRAEVDTYCRYNYGVVESFTV

QRRVQPKVTVYPSKTQPLQHHNLLVCSVNGFYPGSIEVRWFRNSQEEKAGVVSTGLIQNG

DWTFQTLVMLETVPRSGEVYTCQVEHPSMMSPLTVQWSARSESAQSKMLSGVGGFVLGLL

FLGTGLFIYFRNQKGHSGLQPTGLLS

>sp|P01914|HB2C_HUMAN 243 GAGLFIYFRNQKGHS

MVCLRLPGGSCMAVLTVTLMVLSSPLALAGDTRPRFLEEVKFECHFFNGTERVRLLERRV

HNQEEYARYDSDVGEYRAVTELGRPDAEYWNSQKDLLERRRAAVDTYCRHNYGVGESFTV

QRRVQPKVTVYPSKTQPLQHHNLLVCSVNGFYPGSIEVRWFRNGQEEKTGVVSTGLIQNG

DWTFQTLVMLETVPQSGEVYTCQVEHPSVMSPLTVEWRARSESAQSKMLSGVGGFVLGLL

FLGAGLFIYFRNQKGHSGLPPTGFLS

>sp|P01913|2B32_HUMAN 247 FIYFRNQKGHSGLQPTGFLS

MVCLKLPGGSSLAALTVTLMVLSSRLAFAGDTRPRFLELLKSECHFFNGTERVRFLERHF

HNQEEYARFDSDVGEYRAVRELGRPDAEYWNSQKDLLEQKRGQVDNYCRHNYGVGESFTV

QRRVHPQVTVYPAKTQPLQHHNLLVCSVSGFYPGSIEVRWFRNGQEEKAGVVSTGLIQNG

DWTFQTLVMLETFPRSGEVYTCQVEHPSVTSPLTVEWSARSESAQSKMLSGVGGFVLGLL

FLGAGLFIYFRNQKGHSGLQPTGFLS

>sp|P01913|2B32_HUMAN 248 IYFRNQKGHSGLQPTGFL

MVCLKLPGGSSLAALTVTLMVLSSRLAFAGDTRPRFLELLKSECHFFNGTERVRFLERHF

HNQEEYARFDSDVGEYRAVRELGRPDAEYWNSQKDLLEQKRGQVDNYCRHNYGVGESFTV

QRRVHPQVTVYPAKTQPLQHHNLLVCSVSGFYPGSIEVRWFRNGQEEKAGVVSTGLIQNG

DWTFQTLVMLETFPRSGEVYTCQVEHPSVTSPLTVEWSARSESAQSKMLSGVGGFVLGLL

FLGAGLFIYFRNQKGHSGLQPTGFLS

>sp|P01913|2B32_HUMAN 248 IYFRNQKGHSGLQPTGFLS

MVCLKLPGGSSLAALTVTLMVLSSRLAFAGDTRPRFLELLKSECHFFNGTERVRFLERHF

HNQEEYARFDSDVGEYRAVRELGRPDAEYWNSQKDLLEQKRGQVDNYCRHNYGVGESFTV

QRRVHPQVTVYPAKTQPLQHHNLLVCSVSGFYPGSIEVRWFRNGQEEKAGVVSTGLIQNG

DWTFQTLVMLETFPRSGEVYTCQVEHPSVTSPLTVEWSARSESAQSKMLSGVGGFVLGLL

FLGAGLFIYFRNQKGHSGLQPTGFLS

>sp|P20039|2B1B_HUMAN 72 DVGEFRAVTELGRPDEEY

MVCLRLPGGSCMAVLTVTLMVLSSPLALAGDTRPRFLEYSTSECHFFNGTERVRFLDRYF

YNQEEYVRFDSDVGEFRAVTELGRPDEEYWNSQKDFLEDRRAAVDTYCRHNYGVGESFTV

QRRVHPKVTVYPSKTQPLQHHNLLVCSVSGFYPGSIEVRWFRNGQEEKTGVVSTGLIHNG

DWTFQTLVMLETVPRSGEVYTCQVEHPSVTSPLTVEWRARSESAQSKMLSGVGGFVLGLL

FLGAGLFIYFRNQKGHSGLQPRGFLS

>sp|Q30134|2B18_HUMAN 66 YVRFDSDVGEY

MVCLRLPGGSCMAVLTVTLMVLSSPLALAGDTRPRFLEYSTGECYFFNGTERVRFLDRYF

YNQEEYVRFDSDVGEYRAVTELGRPSAEYWNSQKDFLEDRRALVDTYCRHNYGVGESFTV

QRRVHPKVTVYPSKTQPLQHHNLLVCSVSGFYPGSIEVRWFRNGQEEKTGVVSTGLIHNG

DWTFQTLVMLETVPRSGEVYTCQVEHPSVTSPLTVEWSARSESAQSKMLSGVGGFVLGLL

FLGAGLFIYFRNQKGHSGLQPTGFLS

>sp|P13760|2B14_HUMAN 62 HQEEYVRFDSDVGEYRAV

MVCLKFPGGSCMAALTVTLMVLSSPLALAGDTRPRFLEQVKHECHFFNGTERVRFLDRYF

YHQEEYVRFDSDVGEYRAVTELGRPDAEYWNSQKDLLEQKRAAVDTYCRHNYGVGESFTV

QRRVYPEVTVYPAKTQPLQHHNLLVCSVNGFYPGSIEVRWFRNGQEEKTGVVSTGLIQNG

DWTFQTLVMLETVPRSGEVYTCQVEHPSLTSPLTVEWRARSESAQSKMLSGVGGFVLGLL

FLGAGLFIYFRNQKGHSGLQPTGFLS

>sp|P13760|2B14_HUMAN 124 VYPEVTVYPAK

MVCLKFPGGSCMAALTVTLMVLSSPLALAGDTRPRFLEQVKHECHFFNGTERVRFLDRYF

YHQEEYVRFDSDVGEYRAVTELGRPDAEYWNSQKDLLEQKRAAVDTYCRHNYGVGESFTV

QRRVYPEVTVYPAKTQPLQHHNLLVCSVNGFYPGSIEVRWFRNGQEEKTGVVSTGLIQNG

DWTFQTLVMLETVPRSGEVYTCQVEHPSLTSPLTVEWRARSESAQSKMLSGVGGFVLGLL

FLGAGLFIYFRNQKGHSGLQPTGFLS

>sp|P13760|2B14_HUMAN 124 VYPEVTVYPAKT

MVCLKFPGGSCMAALTVTLMVLSSPLALAGDTRPRFLEQVKHECHFFNGTERVRFLDRYF

YHQEEYVRFDSDVGEYRAVTELGRPDAEYWNSQKDLLEQKRAAVDTYCRHNYGVGESFTV

QRRVYPEVTVYPAKTQPLQHHNLLVCSVNGFYPGSIEVRWFRNGQEEKTGVVSTGLIQNG

DWTFQTLVMLETVPRSGEVYTCQVEHPSLTSPLTVEWRARSESAQSKMLSGVGGFVLGLL

FLGAGLFIYFRNQKGHSGLQPTGFLS

>sp|P13760|2B14_HUMAN 51 ERVRLLERCIYNQE

MVCLKLPGGSCMTALTVTLMVLSSPLALAGDTRPRFLWQLKFECHFFNGTERVRLLERCI

YNQEESVRFDSDVGEYRAVTELGRPDAEYWNSQKDLLEQRRAAVDTYCRHNYGVGESFTV

QRRVEPKVTVYPSKTQPLQHHNLLVCSVSGFYPGSIEVRWFRNGQEEKAGVVSTGLIQNG

DWTFQTLVMLETVPRSGEVYTCQVEHPSVTSPLTVEWRARSESAQSKMLSGVGGFVLGLL

FLGAGLFIYFRNQKGHSGLQPTGFLS

>sp|Q8NC54|KCT2_HUMAN 173 RGYMEIEQSVKSFK

MAAAVPKRMRGPAQAKLLPGSAIQALVGLARPLVLALLLVSAALSSVVSRTDSPSPTVLN

SHISTPNVNALTHENQTKPSISQISTTLPPTTSTKKSGGASVVPHPSPTPLSQEEADNNE

DPSIEEEDLLMLNSSPSTAKDTLDNGDYGEPDYDWTTGPRDDDESDDTLEENRGYMEIEQ

SVKSFKMPSSNIEEEDSHFFFHLIIFAFCIAVVYITYHNKRKIFLLVQSRKWRDGLCSKT

VEYHRLDQNVNEAMPSLKITNDYIF

>sp|Q13571|LAPM5_HUMAN 56 GYLRIADLISSF

MDPRLSTVRQTCCCFNVRIATTALAIYHVIMSVLLFIEHSVEVAHGKASCKLSQMGYLRI

ADLISSFLLITMLFIISLSLLIGVVKNREKYLLPFLSLQIMDYLLCLLTLLGSYIELPAY

LKLASRSRASSSKFPLMTLQLLDFCLSILTLCSSYMEVPTYLNFKSMNHMNYLPSQEDMP

HNQFIKMMIIFSIAFITVLIFKVYMFKCVWRCYRLIKCMNSVEEKRNSKMLQKVVLPSYE

EALSLPSKTPEGGPAPPPYSEV

>sp|Q13571|LAPM5_HUMAN 236 LPSYEEALSLPSKTP

MDPRLSTVRQTCCCFNVRIATTALAIYHVIMSVLLFIEHSVEVAHGKASCKLSQMGYLRI

ADLISSFLLITMLFIISLSLLIGVVKNREKYLLPFLSLQIMDYLLCLLTLLGSYIELPAY

LKLASRSRASSSKFPLMTLQLLDFCLSILTLCSSYMEVPTYLNFKSMNHMNYLPSQEDMP

HNQFIKMMIIFSIAFITVLIFKVYMFKCVWRCYRLIKCMNSVEEKRNSKMLQKVVLPSYE

EALSLPSKTPEGGPAPPPYSEV

>sp|Q13571|LAPM5_HUMAN 236 LPSYEEALSLPSKTPE

MDPRLSTVRQTCCCFNVRIATTALAIYHVIMSVLLFIEHSVEVAHGKASCKLSQMGYLRI

ADLISSFLLITMLFIISLSLLIGVVKNREKYLLPFLSLQIMDYLLCLLTLLGSYIELPAY

LKLASRSRASSSKFPLMTLQLLDFCLSILTLCSSYMEVPTYLNFKSMNHMNYLPSQEDMP

HNQFIKMMIIFSIAFITVLIFKVYMFKCVWRCYRLIKCMNSVEEKRNSKMLQKVVLPSYE

EALSLPSKTPEGGPAPPPYSEV

>sp|Q13571|LAPM5_HUMAN 236 LPSYEEALSLPSKTPEG

MDPRLSTVRQTCCCFNVRIATTALAIYHVIMSVLLFIEHSVEVAHGKASCKLSQMGYLRI

ADLISSFLLITMLFIISLSLLIGVVKNREKYLLPFLSLQIMDYLLCLLTLLGSYIELPAY

LKLASRSRASSSKFPLMTLQLLDFCLSILTLCSSYMEVPTYLNFKSMNHMNYLPSQEDMP

HNQFIKMMIIFSIAFITVLIFKVYMFKCVWRCYRLIKCMNSVEEKRNSKMLQKVVLPSYE

EALSLPSKTPEGGPAPPPYSEV

>sp|Q13571|LAPM5_HUMAN 235 VLPSYEEALSLPSKTPEG

MDPRLSTVRQTCCCFNVRIATTALAIYHVIMSVLLFIEHSVEVAHGKASCKLSQMGYLRI

ADLISSFLLITMLFIISLSLLIGVVKNREKYLLPFLSLQIMDYLLCLLTLLGSYIELPAY

LKLASRSRASSSKFPLMTLQLLDFCLSILTLCSSYMEVPTYLNFKSMNHMNYLPSQEDMP

HNQFIKMMIIFSIAFITVLIFKVYMFKCVWRCYRLIKCMNSVEEKRNSKMLQKVVLPSYE

EALSLPSKTPEGGPAPPPYSEV

>sp|Q13571|LAPM5_HUMAN 234 VVLPSYEEALSLPSKTPE

MDPRLSTVRQTCCCFNVRIATTALAIYHVIMSVLLFIEHSVEVAHGKASCKLSQMGYLRI

ADLISSFLLITMLFIISLSLLIGVVKNREKYLLPFLSLQIMDYLLCLLTLLGSYIELPAY

LKLASRSRASSSKFPLMTLQLLDFCLSILTLCSSYMEVPTYLNFKSMNHMNYLPSQEDMP

HNQFIKMMIIFSIAFITVLIFKVYMFKCVWRCYRLIKCMNSVEEKRNSKMLQKVVLPSYE

EALSLPSKTPEGGPAPPPYSEV

>sp|Q13571|LAPM5_HUMAN 234 VVLPSYEEALSLPSKTPEG

MDPRLSTVRQTCCCFNVRIATTALAIYHVIMSVLLFIEHSVEVAHGKASCKLSQMGYLRI

ADLISSFLLITMLFIISLSLLIGVVKNREKYLLPFLSLQIMDYLLCLLTLLGSYIELPAY

LKLASRSRASSSKFPLMTLQLLDFCLSILTLCSSYMEVPTYLNFKSMNHMNYLPSQEDMP

HNQFIKMMIIFSIAFITVLIFKVYMFKCVWRCYRLIKCMNSVEEKRNSKMLQKVVLPSYE

EALSLPSKTPEGGPAPPPYSEV

>sp|P03992|HB25_HUMAN 73 DSDVGVYRAVTPQGRPD

MSWKKALRIPGDLRVATVTLMLAMLSSLLAEGRDSPEDFVFQFKGMCYFTNGTERVRLVT

RYIYNREEYARFDSDVGVYRAVTPQGRPDAEYWNSQKEVLEGTRAELDTVCRHNYEVAFR

GILQRRVEPTVTISPSRTEALNHHNLLVCSVTDFYPGQIKVRWFRNDQEETAGVVSTPLI

RNGDWTFQILVMLEMTPQRGDVYTCHVEHPSLQSPITVEWRAQSESAQSKMLSGVGGFVL

GLIFLGLGLIIRQRSQKGLLH

>sp|P03992|HB25_HUMAN 73 DSDVGVYRAVTPQGRPDA

MSWKKALRIPGDLRVATVTLMLAMLSSLLAEGRDSPEDFVFQFKGMCYFTNGTERVRLVT

RYIYNREEYARFDSDVGVYRAVTPQGRPDAEYWNSQKEVLEGTRAELDTVCRHNYEVAFR

GILQRRVEPTVTISPSRTEALNHHNLLVCSVTDFYPGQIKVRWFRNDQEETAGVVSTPLI

RNGDWTFQILVMLEMTPQRGDVYTCHVEHPSLQSPITVEWRAQSESAQSKMLSGVGGFVL

GLIFLGLGLIIRQRSQKGLLH

>sp|P03992|HB25_HUMAN 73 DSDVGVYRAVTPQGRPDAEY

MSWKKALRIPGDLRVATVTLMLAMLSSLLAEGRDSPEDFVFQFKGMCYFTNGTERVRLVT

RYIYNREEYARFDSDVGVYRAVTPQGRPDAEYWNSQKEVLEGTRAELDTVCRHNYEVAFR

GILQRRVEPTVTISPSRTEALNHHNLLVCSVTDFYPGQIKVRWFRNDQEETAGVVSTPLI

RNGDWTFQILVMLEMTPQRGDVYTCHVEHPSLQSPITVEWRAQSESAQSKMLSGVGGFVL

GLIFLGLGLIIRQRSQKGLLH

>sp|P03992|HB25_HUMAN 75 DVGVYRAVTPQGRP

MSWKKALRIPGDLRVATVTLMLAMLSSLLAEGRDSPEDFVFQFKGMCYFTNGTERVRLVT

RYIYNREEYARFDSDVGVYRAVTPQGRPDAEYWNSQKEVLEGTRAELDTVCRHNYEVAFR

GILQRRVEPTVTISPSRTEALNHHNLLVCSVTDFYPGQIKVRWFRNDQEETAGVVSTPLI

RNGDWTFQILVMLEMTPQRGDVYTCHVEHPSLQSPITVEWRAQSESAQSKMLSGVGGFVL

GLIFLGLGLIIRQRSQKGLLH

>sp|P03992|HB25_HUMAN 75 DVGVYRAVTPQGRPD

MSWKKALRIPGDLRVATVTLMLAMLSSLLAEGRDSPEDFVFQFKGMCYFTNGTERVRLVT

RYIYNREEYARFDSDVGVYRAVTPQGRPDAEYWNSQKEVLEGTRAELDTVCRHNYEVAFR

GILQRRVEPTVTISPSRTEALNHHNLLVCSVTDFYPGQIKVRWFRNDQEETAGVVSTPLI

RNGDWTFQILVMLEMTPQRGDVYTCHVEHPSLQSPITVEWRAQSESAQSKMLSGVGGFVL

GLIFLGLGLIIRQRSQKGLLH

>sp|P03992|HB25_HUMAN 75 DVGVYRAVTPQGRPDA

MSWKKALRIPGDLRVATVTLMLAMLSSLLAEGRDSPEDFVFQFKGMCYFTNGTERVRLVT

RYIYNREEYARFDSDVGVYRAVTPQGRPDAEYWNSQKEVLEGTRAELDTVCRHNYEVAFR

GILQRRVEPTVTISPSRTEALNHHNLLVCSVTDFYPGQIKVRWFRNDQEETAGVVSTPLI

RNGDWTFQILVMLEMTPQRGDVYTCHVEHPSLQSPITVEWRAQSESAQSKMLSGVGGFVL

GLIFLGLGLIIRQRSQKGLLH

>sp|P03992|HB25_HUMAN 75 DVGVYRAVTPQGRPDAE

MSWKKALRIPGDLRVATVTLMLAMLSSLLAEGRDSPEDFVFQFKGMCYFTNGTERVRLVT

RYIYNREEYARFDSDVGVYRAVTPQGRPDAEYWNSQKEVLEGTRAELDTVCRHNYEVAFR

GILQRRVEPTVTISPSRTEALNHHNLLVCSVTDFYPGQIKVRWFRNDQEETAGVVSTPLI

RNGDWTFQILVMLEMTPQRGDVYTCHVEHPSLQSPITVEWRAQSESAQSKMLSGVGGFVL

GLIFLGLGLIIRQRSQKGLLH

>sp|P03992|HB25_HUMAN 154 FYPGQIKVRWFRNDQEET

MSWKKALRIPGDLRVATVTLMLAMLSSLLAEGRDSPEDFVFQFKGMCYFTNGTERVRLVT

RYIYNREEYARFDSDVGVYRAVTPQGRPDAEYWNSQKEVLEGTRAELDTVCRHNYEVAFR

GILQRRVEPTVTISPSRTEALNHHNLLVCSVTDFYPGQIKVRWFRNDQEETAGVVSTPLI

RNGDWTFQILVMLEMTPQRGDVYTCHVEHPSLQSPITVEWRAQSESAQSKMLSGVGGFVL

GLIFLGLGLIIRQRSQKGLLH

>sp|P03992|HB25_HUMAN 74 SDVGVYRAVTPQGRPDAE

MSWKKALRIPGDLRVATVTLMLAMLSSLLAEGRDSPEDFVFQFKGMCYFTNGTERVRLVT

RYIYNREEYARFDSDVGVYRAVTPQGRPDAEYWNSQKEVLEGTRAELDTVCRHNYEVAFR

GILQRRVEPTVTISPSRTEALNHHNLLVCSVTDFYPGQIKVRWFRNDQEETAGVVSTPLI

RNGDWTFQILVMLEMTPQRGDVYTCHVEHPSLQSPITVEWRAQSESAQSKMLSGVGGFVL

GLIFLGLGLIIRQRSQKGLLH

>sp|P01920|HB24_HUMAN 35 SPEDFVYQFKGMCYF

MSWKKALRIPGGLRVATVTLMLAMLSTPVAEGRDSPEDFVYQFKGMCYFTNGTERVRLVT

RYIYNREEYARFDSDVGVYRAVTPLGPPAAEYWNSQKEVLERTRAELDTVCRHNYQLELR

TTLQRRVEPTVTISPSRTEALNHHNLLVCSVTDFYPAQIKVRWFRNDQEETTGVVSTPLI

RNGDWTFQILVMLEMTPQRGDVYTCHVEHPSLQNPIIVEWRAQSESAQSKMLSGIGGFVL

GLIFLGLGLIIHHRSQKGLLH

>sp|P28067|2DMA_HUMAN 171 FGPTFVSAVDGLSFQ

MGHEQNQGAALLQMLPLLWLLPHSWAVPEAPTPMWPDDLQNHTFLHTVYCQDGSPSVGLS

EAYDEDQLFFFDFSQNTRVPRLPEFADWAQEQGDAPAILFDKEFCEWMIQQIGPKLDGKI

PVSRGFPIAEVFTLKPLEFGKPNTLVCFVSNLFPPMLTVNWHDHSVPVEGFGPTFVSAVD

GLSFQAFSYLNFTPEPSDIFSCIVTHEIDRYTAIAYWVPRNALPSDLLENVLCGVAFGLG

VLGIIVGIVLIIYFRKPCSGD

>sp|P13763|HB2Q_HUMAN 60 NREEFVRFDSDVGEFR

MMVLQVSAAPRTVALTALLMVLLTSVVQGRATPENYLFQGRQECYAFNGTQRFLERYIYN

REEFVRFDSDVGEFRAVTELGRPDEEYWNSQKDILEEERAVPDRMCRHNYELGGPMTLQR

RVQPRVNVSPSKKGPLQHHNLLVCHVTDFYPGSIQVRWFLNGQEETAGVVSTNLIRNGDW

TFQILVMLEMTPQQGDVYTCQVEHTSLDSPVTVEWKAQSDSARSKTLTGAGGFVLGLIIC

GVGIFMHRRSKKVQRGSA

>sp|O00584|RNT2_HUMAN 138 SLELYRELDLNSVLL

MRPAALRGALLGCLCLALLCLGGADKRLRDNHEWKKLIMVQHWPETVCEKIQNDCRDPPD

YWTIHGLWPDKSEGCNRSWPFNLEEIKDLLPEMRAYWPDVIHSFPNRSRFWKHEWEKHGT

CAAQVDALNSQKKYFGRSLELYRELDLNSVLLKLGIKPSINYYQVADFKDALARVYGVIP

KIQCLPPSQDEEVQTIGQIELCLTKQDQQLQNCTEPGEQPSPKQEVWLANGAAESRGLRV

CEDGPVFYPPPKKTKH

>sp|O43752|STX6_HUMAN 74 NPRKFNLDATELSIRKA

MSMEDPFFVVKGEVQKAVNTAQGLFQRWTELLQDPSTATREEIDWTTNELRNNLRSIEWD

LEDLDETISIVEANPRKFNLDATELSIRKAFITSTRQVVRDMKDQMSTSSVQALAERKNR

QALLGDSGSQNWSTGTTDKYGRLDRELQRANSHFIEEQQAQQQLIVEQQDEQLELVSGSI

GVLKNMSQRIGGELEEQAVMLEDFSHELESTQSRLDNVMKKLAKVSHMTSDRRQWCAIAI

LFAVLLVVLILFLVL

>sp|P01906|HA26_HUMAN 24 EDIVADHVASY

MILNKALLLGALALTAVMSPCGGEDIVADHVASYGVNFYQSHGPSGQYTHEFDGDEEFYV

DLETKETVWQLPMFSKFISFDPQSALRNMAVGKHTLEFMMRQSNSTAATNEVPEVTVFSK

FPVTLGQPNTLICLVDNIFPPVVNITWLSNGHSVTEGVSETSFLSKSDHSFFKISYLTFL

PSADEIYDCKVEHWGLDEPLLKHWEPEIPAPMSELTETLVCALGLSVGLMGIVVGTVFII

QGLRSVGASRHQGLL

>sp|P01908|HA21_HUMAN 98 IVIKRSNSTAATN

MILNKALMLGALALTTVMSPCGGEDIVADSVAQLGVNLYQSYGPSGQYSHEFDGDEEFYV

DLERKETVWQLPLFRRFRRFDPQFALTNIAVLKHNLNIVIKRSNSTAATNEVPEVTVFSK

SPVTLGQPNTLICLVDNIFPPVVNITWLSNGHSVTEGVSETSFLSKSDHSFFKISYLTFL

PSADEIYDCKVEHWGLDEPLLKHWEPEIPTPMSELTETVVCALGLSVGLVGIVVGTVLII

RGLRSVGASRHQGPL

>sp|P01908|HA21_HUMAN 97 NIVIKRSNSTAATNEV

MILNKALMLGALALTTVMSPCGGEDIVADSVAQLGVNLYQSYGPSGQYSHEFDGDEEFYV

DLERKETVWQLPLFRRFRRFDPQFALTNIAVLKHNLNIVIKRSNSTAATNEVPEVTVFSK

SPVTLGQPNTLICLVDNIFPPVVNITWLSNGHSVTEGVSETSFLSKSDHSFFKISYLTFL

PSADEIYDCKVEHWGLDEPLLKHWEPEIPTPMSELTETVVCALGLSVGLVGIVVGTVLII

RGLRSVGASRHQGPL

>sp|P01908|HA21_HUMAN 97 NIVIKRSNSTAATNEVPEVTVFS

MILNKALMLGALALTTVMSPCGGEDIVADSVAQLGVNLYQSYGPSGQYSHEFDGDEEFYV

DLERKETVWQLPLFRRFRRFDPQFALTNIAVLKHNLNIVIKRSNSTAATNEVPEVTVFSK

SPVTLGQPNTLICLVDNIFPPVVNITWLSNGHSVTEGVSETSFLSKSDHSFFKISYLTFL

PSADEIYDCKVEHWGLDEPLLKHWEPEIPTPMSELTETVVCALGLSVGLVGIVVGTVLII

RGLRSVGASRHQGPL

>sp|P62258|1433E_HUMAN 57 RASWRIISSIEQKEE

MDDREDLVYQAKLAEQAERYDEMVESMKKVAGMDVELTVEERNLLSVAYKNVIGARRASW

RIISSIEQKEENKGGEDKLKMIREYRQMVETELKLICCDILDVLDKHLIPAANTGESKVF

YYKMKGDYHRYLAEFATGNDRKEAAENSLVAYKAASDIAMTELPPTHPIRLGLALNFSVF

YYEILNSPDRACRLAKAAFDDAIAELDTLSEESYKDSTLIMQLLRDNLTLWTSDMQGDGE

EQNKEALQDVEDENQ

>sp|P41273|TNFL9_HUMAN 105 GPLSWYSDPGLAGVS

MEYASDASLDPEAPWPPAPRARACRVLPWALVAGLLLLLLLAAACAVFLACPWAVSGARA

SPGSAASPRLREGPELSPDDPAGLLDLRQGMFAQLVAQNVLLIDGPLSWYSDPGLAGVSL

TGGLSYKEDTKELVVAKAGVYYVFFQLELRRVVAGEGSGSVSLALHLQPLRSAAGAAALA

LTVDLPPASSEARNSAFGFQGRLLHLSAGQRLGVHLHTEARARHAWQLTQGATVLGLFRV

TPEIPAGLPSPRSE

>sp|P05536|HA27_HUMAN 24 EDIVADHVASYGVNL

MILNKALMLGSLALTTVMSPCGGEDIVADHVASYGVNLYQSYGPSGQYTHEFDGDEQFYV

DLGRKETVWCLPVLRQFRFDPQFALTNIAVHKHNLNSLIKRSNSTAATNEVPEVTVFSKS

PVTLGQPNILICLVDNIFPPVVNITWLSNGHSVTEGVSETSFLSKSDHSFFKISYLTLLP

SAEESYDCKVEHWGLDKPLLKHWEPEIPAPMSELTETVVCALGLSVGLVGIVVGTVFIIR

GLRSVGASRHQGPL

>sp|P05536|HA27_HUMAN 39 YQSYGPSGQYTHEFD

MILNKALMLGSLALTTVMSPCGGEDIVADHVASYGVNLYQSYGPSGQYTHEFDGDEQFYV

DLGRKETVWCLPVLRQFRFDPQFALTNIAVHKHNLNSLIKRSNSTAATNEVPEVTVFSKS

PVTLGQPNILICLVDNIFPPVVNITWLSNGHSVTEGVSETSFLSKSDHSFFKISYLTLLP

SAEESYDCKVEHWGLDKPLLKHWEPEIPAPMSELTETVVCALGLSVGLVGIVVGTVFIIR

GLRSVGASRHQGPL

>sp|P01903|2DRA_HUMAN 86 ANIAVDKANLEIMTKR

MAISGVPVLGFFIIAVLMSAQESWAIKEEHVIIQAEFYLNPDQSGEFMFDFDGDEIFHVD

MAKKETVWRLEEFGRFASFEAQGALANIAVDKANLEIMTKRSNYTPITNVPPEVTVLTNS

PVELREPNVLICFIDKFTPPVVNVTWLRNGKPVTTGVSETVFLPREDHLFRKFHYLPFLP

STEDVYDCRVEHWGLDEPLLKHWEFDAPSPLPETTENVVCALGLTVGLVGIIIGTIFIIK

GVRKSNAAERRGPL

>sp|P01903|2DRA_HUMAN 207 APSPLPETTENVVCALG

MAISGVPVLGFFIIAVLMSAQESWAIKEEHVIIQAEFYLNPDQSGEFMFDFDGDEIFHVD

MAKKETVWRLEEFGRFASFEAQGALANIAVDKANLEIMTKRSNYTPITNVPPEVTVLTNS

PVELREPNVLICFIDKFTPPVVNVTWLRNGKPVTTGVSETVFLPREDHLFRKFHYLPFLP

STEDVYDCRVEHWGLDEPLLKHWEFDAPSPLPETTENVVCALGLTVGLVGIIIGTIFIIK

GVRKSNAAERRGPL

>sp|P01903|2DRA_HUMAN 207 APSPLPETTENVVCALGLTV

MAISGVPVLGFFIIAVLMSAQESWAIKEEHVIIQAEFYLNPDQSGEFMFDFDGDEIFHVD

MAKKETVWRLEEFGRFASFEAQGALANIAVDKANLEIMTKRSNYTPITNVPPEVTVLTNS

PVELREPNVLICFIDKFTPPVVNVTWLRNGKPVTTGVSETVFLPREDHLFRKFHYLPFLP

STEDVYDCRVEHWGLDEPLLKHWEFDAPSPLPETTENVVCALGLTVGLVGIIIGTIFIIK

GVRKSNAAERRGPL

>sp|P01903|2DRA_HUMAN 81 AQGALANIAVDKANLEI

MAISGVPVLGFFIIAVLMSAQESWAIKEEHVIIQAEFYLNPDQSGEFMFDFDGDEIFHVD

MAKKETVWRLEEFGRFASFEAQGALANIAVDKANLEIMTKRSNYTPITNVPPEVTVLTNS

PVELREPNVLICFIDKFTPPVVNVTWLRNGKPVTTGVSETVFLPREDHLFRKFHYLPFLP

STEDVYDCRVEHWGLDEPLLKHWEFDAPSPLPETTENVVCALGLTVGLVGIIIGTIFIIK

GVRKSNAAERRGPL

>sp|P01903|2DRA_HUMAN 81 AQGALANIAVDKANLEIMT

MAISGVPVLGFFIIAVLMSAQESWAIKEEHVIIQAEFYLNPDQSGEFMFDFDGDEIFHVD

MAKKETVWRLEEFGRFASFEAQGALANIAVDKANLEIMTKRSNYTPITNVPPEVTVLTNS

PVELREPNVLICFIDKFTPPVVNVTWLRNGKPVTTGVSETVFLPREDHLFRKFHYLPFLP

STEDVYDCRVEHWGLDEPLLKHWEFDAPSPLPETTENVVCALGLTVGLVGIIIGTIFIIK

GVRKSNAAERRGPL

>sp|P01903|2DRA_HUMAN 80 EAQGALANIAVDKA

MAISGVPVLGFFIIAVLMSAQESWAIKEEHVIIQAEFYLNPDQSGEFMFDFDGDEIFHVD

MAKKETVWRLEEFGRFASFEAQGALANIAVDKANLEIMTKRSNYTPITNVPPEVTVLTNS

PVELREPNVLICFIDKFTPPVVNVTWLRNGKPVTTGVSETVFLPREDHLFRKFHYLPFLP

STEDVYDCRVEHWGLDEPLLKHWEFDAPSPLPETTENVVCALGLTVGLVGIIIGTIFIIK

GVRKSNAAERRGPL

>sp|P01903|2DRA_HUMAN 79 FEAQGALANIAVDKA

MAISGVPVLGFFIIAVLMSAQESWAIKEEHVIIQAEFYLNPDQSGEFMFDFDGDEIFHVD

MAKKETVWRLEEFGRFASFEAQGALANIAVDKANLEIMTKRSNYTPITNVPPEVTVLTNS

PVELREPNVLICFIDKFTPPVVNVTWLRNGKPVTTGVSETVFLPREDHLFRKFHYLPFLP

STEDVYDCRVEHWGLDEPLLKHWEFDAPSPLPETTENVVCALGLTVGLVGIIIGTIFIIK

GVRKSNAAERRGPL

>sp|P01903|2DRA_HUMAN 83 GALANIAVDKANLEIMTKRSN

MAISGVPVLGFFIIAVLMSAQESWAIKEEHVIIQAEFYLNPDQSGEFMFDFDGDEIFHVD

MAKKETVWRLEEFGRFASFEAQGALANIAVDKANLEIMTKRSNYTPITNVPPEVTVLTNS

PVELREPNVLICFIDKFTPPVVNVTWLRNGKPVTTGVSETVFLPREDHLFRKFHYLPFLP

STEDVYDCRVEHWGLDEPLLKHWEFDAPSPLPETTENVVCALGLTVGLVGIIIGTIFIIK

GVRKSNAAERRGPL

>sp|P01903|2DRA_HUMAN 238 IIKGVRKSNAAERRG

MAISGVPVLGFFIIAVLMSAQESWAIKEEHVIIQAEFYLNPDQSGEFMFDFDGDEIFHVD

MAKKETVWRLEEFGRFASFEAQGALANIAVDKANLEIMTKRSNYTPITNVPPEVTVLTNS

PVELREPNVLICFIDKFTPPVVNVTWLRNGKPVTTGVSETVFLPREDHLFRKFHYLPFLP

STEDVYDCRVEHWGLDEPLLKHWEFDAPSPLPETTENVVCALGLTVGLVGIIIGTIFIIK

GVRKSNAAERRGPL

>sp|P01903|2DRA_HUMAN 33 IQAEFYLNPDQSGEF

MAISGVPVLGFFIIAVLMSAQESWAIKEEHVIIQAEFYLNPDQSGEFMFDFDGDEIFHVD

MAKKETVWRLEEFGRFASFEAQGALANIAVDKANLEIMTKRSNYTPITNVPPEVTVLTNS

PVELREPNVLICFIDKFTPPVVNVTWLRNGKPVTTGVSETVFLPREDHLFRKFHYLPFLP

STEDVYDCRVEHWGLDEPLLKHWEFDAPSPLPETTENVVCALGLTVGLVGIIIGTIFIIK

GVRKSNAAERRGPL

>sp|P01903|2DRA_HUMAN 33 IQAEFYLNPDQSGEFMFD

MAISGVPVLGFFIIAVLMSAQESWAIKEEHVIIQAEFYLNPDQSGEFMFDFDGDEIFHVD

MAKKETVWRLEEFGRFASFEAQGALANIAVDKANLEIMTKRSNYTPITNVPPEVTVLTNS

PVELREPNVLICFIDKFTPPVVNVTWLRNGKPVTTGVSETVFLPREDHLFRKFHYLPFLP

STEDVYDCRVEHWGLDEPLLKHWEFDAPSPLPETTENVVCALGLTVGLVGIIIGTIFIIK

GVRKSNAAERRGPL

>sp|P01903|2DRA_HUMAN 85 LANIAVDKANLEIMTKR

MAISGVPVLGFFIIAVLMSAQESWAIKEEHVIIQAEFYLNPDQSGEFMFDFDGDEIFHVD

MAKKETVWRLEEFGRFASFEAQGALANIAVDKANLEIMTKRSNYTPITNVPPEVTVLTNS

PVELREPNVLICFIDKFTPPVVNVTWLRNGKPVTTGVSETVFLPREDHLFRKFHYLPFLP

STEDVYDCRVEHWGLDEPLLKHWEFDAPSPLPETTENVVCALGLTVGLVGIIIGTIFIIK

GVRKSNAAERRGPL

>sp|P01903|2DRA_HUMAN 70 LEEFGRFASFEAQG

MAISGVPVLGFFIIAVLMSAQESWAIKEEHVIIQAEFYLNPDQSGEFMFDFDGDEIFHVD

MAKKETVWRLEEFGRFASFEAQGALANIAVDKANLEIMTKRSNYTPITNVPPEVTVLTNS

PVELREPNVLICFIDKFTPPVVNVTWLRNGKPVTTGVSETVFLPREDHLFRKFHYLPFLP

STEDVYDCRVEHWGLDEPLLKHWEFDAPSPLPETTENVVCALGLTVGLVGIIIGTIFIIK

GVRKSNAAERRGPL

>sp|P01903|2DRA_HUMAN 179 LPSTEDVYDCRVE

MAISGVPVLGFFIIAVLMSAQESWAIKEEHVIIQAEFYLNPDQSGEFMFDFDGDEIFHVD

MAKKETVWRLEEFGRFASFEAQGALANIAVDKANLEIMTKRSNYTPITNVPPEVTVLTNS

PVELREPNVLICFIDKFTPPVVNVTWLRNGKPVTTGVSETVFLPREDHLFRKFHYLPFLP

STEDVYDCRVEHWGLDEPLLKHWEFDAPSPLPETTENVVCALGLTVGLVGIIIGTIFIIK

GVRKSNAAERRGPL

>sp|P01903|2DRA_HUMAN 82 QGALANIAVDKANLE

MAISGVPVLGFFIIAVLMSAQESWAIKEEHVIIQAEFYLNPDQSGEFMFDFDGDEIFHVD

MAKKETVWRLEEFGRFASFEAQGALANIAVDKANLEIMTKRSNYTPITNVPPEVTVLTNS

PVELREPNVLICFIDKFTPPVVNVTWLRNGKPVTTGVSETVFLPREDHLFRKFHYLPFLP

STEDVYDCRVEHWGLDEPLLKHWEFDAPSPLPETTENVVCALGLTVGLVGIIIGTIFIIK

GVRKSNAAERRGPL

>sp|P01903|2DRA_HUMAN 82 QGALANIAVDKANLEIM

MAISGVPVLGFFIIAVLMSAQESWAIKEEHVIIQAEFYLNPDQSGEFMFDFDGDEIFHVD

MAKKETVWRLEEFGRFASFEAQGALANIAVDKANLEIMTKRSNYTPITNVPPEVTVLTNS

PVELREPNVLICFIDKFTPPVVNVTWLRNGKPVTTGVSETVFLPREDHLFRKFHYLPFLP

STEDVYDCRVEHWGLDEPLLKHWEFDAPSPLPETTENVVCALGLTVGLVGIIIGTIFIIK

GVRKSNAAERRGPL

>sp|P01903|2DRA_HUMAN 158 SETVFLPREDHLFRKFHYLPFLP

MAISGVPVLGFFIIAVLMSAQESWAIKEEHVIIQAEFYLNPDQSGEFMFDFDGDEIFHVD

MAKKETVWRLEEFGRFASFEAQGALANIAVDKANLEIMTKRSNYTPITNVPPEVTVLTNS

PVELREPNVLICFIDKFTPPVVNVTWLRNGKPVTTGVSETVFLPREDHLFRKFHYLPFLP

STEDVYDCRVEHWGLDEPLLKHWEFDAPSPLPETTENVVCALGLTVGLVGIIIGTIFIIK

GVRKSNAAERRGPL

>sp|P01903|2DRA_HUMAN 78 SFEAQGALANIAVDKA

MAISGVPVLGFFIIAVLMSAQESWAIKEEHVIIQAEFYLNPDQSGEFMFDFDGDEIFHVD

MAKKETVWRLEEFGRFASFEAQGALANIAVDKANLEIMTKRSNYTPITNVPPEVTVLTNS

PVELREPNVLICFIDKFTPPVVNVTWLRNGKPVTTGVSETVFLPREDHLFRKFHYLPFLP

STEDVYDCRVEHWGLDEPLLKHWEFDAPSPLPETTENVVCALGLTVGLVGIIIGTIFIIK

GVRKSNAAERRGPL

>sp|P01903|2DRA_HUMAN 182 TEDVYDCRVEHWGLD

MAISGVPVLGFFIIAVLMSAQESWAIKEEHVIIQAEFYLNPDQSGEFMFDFDGDEIFHVD

MAKKETVWRLEEFGRFASFEAQGALANIAVDKANLEIMTKRSNYTPITNVPPEVTVLTNS

PVELREPNVLICFIDKFTPPVVNVTWLRNGKPVTTGVSETVFLPREDHLFRKFHYLPFLP

STEDVYDCRVEHWGLDEPLLKHWEFDAPSPLPETTENVVCALGLTVGLVGIIIGTIFIIK

GVRKSNAAERRGPL

>sp|P01903|2DRA_HUMAN 110 VPPEVTVLTNSPVELREP

MAISGVPVLGFFIIAVLMSAQESWAIKEEHVIIQAEFYLNPDQSGEFMFDFDGDEIFHVD

MAKKETVWRLEEFGRFASFEAQGALANIAVDKANLEIMTKRSNYTPITNVPPEVTVLTNS

PVELREPNVLICFIDKFTPPVVNVTWLRNGKPVTTGVSETVFLPREDHLFRKFHYLPFLP

STEDVYDCRVEHWGLDEPLLKHWEFDAPSPLPETTENVVCALGLTVGLVGIIIGTIFIIK

GVRKSNAAERRGPL

>sp|Q9P0L0|VAPA_HUMAN 80 LQPFDYDPNEKSK

MASASGAMAKHEQILVLDPPTDLKFKGPFTDVVTTNLKLRNPSDRKVCFKVKTTAPRRYC

VRPNSGIIDPGSTVTVSVMLQPFDYDPNEKSKHKFMVQTIFAPPNTSDMEAVWKEAKPDE

LMDSKLRCVFEMPNENDKLNDMEPSKAVPLNASKQDGPMPKPHSVSLNDTETRKLMEECK

RLQGEMMKLSEENRHLRDEGLRLRKVAHSDKPGSTSTASFRDNVTSPLPSLLVVIAAIFI

GFFLGKFIL

>sp|P60174|TPIS_HUMAN 237 LKPEFVDIINAKQ

MAPSRKFFVGGNWKMNGRKQSLGELIGTLNAAKVPADTEVVCAPPTAYIDFARQKLDPKI

AVAAQNCYKVTNGAFTGEISPGMIKDCGATWVVLGHSERRHVFGESDELIGQKVAHALAE

GLGVIACIGEKLDEREAGITEKVVFEQTKVIADNVKDWSKVVLAYEPVWAIGTGKTATPQ

QAQEVHEKLRGWLKSNVSDAVAQSTRIIYGGSVTGATCKELASQPDVDGFLVGGASLKPE

FVDIINAKQ

>sp|P11226|MBL2_HUMAN 174 IQNLIKEEAFLGITDEKTEG

MSLFPSLPLLLLSMVAASYSETVTCEDAQKTCPAVIACSSPGINGFPGKDGRDGTKGEKG

EPGQGLRGLQGPPGKLGPPGNPGPSGSPGPKGQKGDPGKSPDGDSSLAASERKALQTEMA

RIKKWLTFSLGKQVGNKFFLTNGEIMTFEKVKALCVKFQASVATPRNAAENGAIQNLIKE

EAFLGITDEKTEGQFVDLTGNRLTYTNWNEGEPNNAGSDEDCVLLLKNGQWNDVPCSTSH

LAVCEFPI

>sp|Q15056|IF4H_HUMAN 57 DIDAIFKDLSIRSVR

MADFDTYDDRAYSSFGGGRGSRGSAGGHGSRSQKELPTEPPYTAYVGNLPFNTVQGDIDA

IFKDLSIRSVRLVRDKDTDKFKGFCYVEFDEVDSLKEALTYDGALLGDRSLRVDIAEGRK

QDKGGFGFRKGGPDDRGMGSSRESRGGWDSRDDFNSGFRDDFLGGRGGSRPGDRRTGPPM

GSRFRDGPPLRGSNMDFREPTEEERAQRPRLQLKPRTVATPLNQVANPNSAIFGGARPRE

EVVQKEQE

>sp|P09326|CD48_HUMAN 88 DPQSGALYISKVQKEDNSTYI

MCSRGWDSCLALELLLLPLSLLVTSIQGHLVHMTVVSGSNVTLNISESLPENYKQLTWFY

TFDQKIVEWDSRKSKYFESKFKGRVRLDPQSGALYISKVQKEDNSTYIMRVLKKTGNEQE

WKIKLQVLDPVPKPVIKIEKIEDMDDNCYLKLSCVIPGESVNYTWYGDKRPFPKELQNSV

LETTLMPHNYSRCYTCQVSNSVSSKNGTVCLSPPCTLARSFGVEWIASWLVVTVPTILGL

LLT

>sp|P09326|CD48_HUMAN 129 DPVPKPVIKIEKIED

MCSRGWDSCLALELLLLPLSLLVTSIQGHLVHMTVVSGSNVTLNISESLPENYKQLTWFY

TFDQKIVEWDSRKSKYFESKFKGRVRLDPQSGALYISKVQKEDNSTYIMRVLKKTGNEQE

WKIKLQVLDPVPKPVIKIEKIEDMDDNCYLKLSCVIPGESVNYTWYGDKRPFPKELQNSV

LETTLMPHNYSRCYTCQVSNSVSSKNGTVCLSPPCTLARSFGVEWIASWLVVTVPTILGL

LLT

>sp|P09326|CD48_HUMAN 129 DPVPKPVIKIEKIEDMDD

MCSRGWDSCLALELLLLPLSLLVTSIQGHLVHMTVVSGSNVTLNISESLPENYKQLTWFY

TFDQKIVEWDSRKSKYFESKFKGRVRLDPQSGALYISKVQKEDNSTYIMRVLKKTGNEQE

WKIKLQVLDPVPKPVIKIEKIEDMDDNCYLKLSCVIPGESVNYTWYGDKRPFPKELQNSV

LETTLMPHNYSRCYTCQVSNSVSSKNGTVCLSPPCTLARSFGVEWIASWLVVTVPTILGL

LLT

>sp|P09326|CD48_HUMAN 63 DQKIVEWDSRKSKYF

MCSRGWDSCLALELLLLPLSLLVTSIQGHLVHMTVVSGSNVTLNISESLPENYKQLTWFY

TFDQKIVEWDSRKSKYFESKFKGRVRLDPQSGALYISKVQKEDNSTYIMRVLKKTGNEQE

WKIKLQVLDPVPKPVIKIEKIEDMDDNCYLKLSCVIPGESVNYTWYGDKRPFPKELQNSV

LETTLMPHNYSRCYTCQVSNSVSSKNGTVCLSPPCTLARSFGVEWIASWLVVTVPTILGL

LLT

>sp|P09326|CD48_HUMAN 62 FDQKIVEWDSRKSK

MCSRGWDSCLALELLLLPLSLLVTSIQGHLVHMTVVSGSNVTLNISESLPENYKQLTWFY

TFDQKIVEWDSRKSKYFESKFKGRVRLDPQSGALYISKVQKEDNSTYIMRVLKKTGNEQE

WKIKLQVLDPVPKPVIKIEKIEDMDDNCYLKLSCVIPGESVNYTWYGDKRPFPKELQNSV

LETTLMPHNYSRCYTCQVSNSVSSKNGTVCLSPPCTLARSFGVEWIASWLVVTVPTILGL

LLT

>sp|P09326|CD48_HUMAN 62 FDQKIVEWDSRKSKYF

MCSRGWDSCLALELLLLPLSLLVTSIQGHLVHMTVVSGSNVTLNISESLPENYKQLTWFY

TFDQKIVEWDSRKSKYFESKFKGRVRLDPQSGALYISKVQKEDNSTYIMRVLKKTGNEQE

WKIKLQVLDPVPKPVIKIEKIEDMDDNCYLKLSCVIPGESVNYTWYGDKRPFPKELQNSV

LETTLMPHNYSRCYTCQVSNSVSSKNGTVCLSPPCTLARSFGVEWIASWLVVTVPTILGL

LLT

>sp|P09326|CD48_HUMAN 62 FDQKIVEWDSRKSKYFE

MCSRGWDSCLALELLLLPLSLLVTSIQGHLVHMTVVSGSNVTLNISESLPENYKQLTWFY

TFDQKIVEWDSRKSKYFESKFKGRVRLDPQSGALYISKVQKEDNSTYIMRVLKKTGNEQE

WKIKLQVLDPVPKPVIKIEKIEDMDDNCYLKLSCVIPGESVNYTWYGDKRPFPKELQNSV

LETTLMPHNYSRCYTCQVSNSVSSKNGTVCLSPPCTLARSFGVEWIASWLVVTVPTILGL

LLT

>sp|P09326|CD48_HUMAN 62 FDQKIVEWDSRKSKYFES

MCSRGWDSCLALELLLLPLSLLVTSIQGHLVHMTVVSGSNVTLNISESLPENYKQLTWFY

TFDQKIVEWDSRKSKYFESKFKGRVRLDPQSGALYISKVQKEDNSTYIMRVLKKTGNEQE

WKIKLQVLDPVPKPVIKIEKIEDMDDNCYLKLSCVIPGESVNYTWYGDKRPFPKELQNSV

LETTLMPHNYSRCYTCQVSNSVSSKNGTVCLSPPCTLARSFGVEWIASWLVVTVPTILGL

LLT

>sp|P09326|CD48_HUMAN 92 GALYISKVQKEDNSTYI

MCSRGWDSCLALELLLLPLSLLVTSIQGHLVHMTVVSGSNVTLNISESLPENYKQLTWFY

TFDQKIVEWDSRKSKYFESKFKGRVRLDPQSGALYISKVQKEDNSTYIMRVLKKTGNEQE

WKIKLQVLDPVPKPVIKIEKIEDMDDNCYLKLSCVIPGESVNYTWYGDKRPFPKELQNSV

LETTLMPHNYSRCYTCQVSNSVSSKNGTVCLSPPCTLARSFGVEWIASWLVVTVPTILGL

LLT

>sp|O43760|SNG2_HUMAN 134 NPKDVLVGADSVRAAITF

MESGAYGAAKAGGSFDLRRFLTQPQVVARAVCLVFALIVFSCIYGEGYSNAHESKQMYCV

FNRNEDACRYGSAIGVLAFLASAFFLVVDAYFPQISNATDRKYLVIGDLLFSALWTFLWF

VGFCFLTNQWAVTNPKDVLVGADSVRAAITFSFFSIFSWGVLASLAYQRYKAGVDDFIQN

YVDPTPDPNTAYASYPGASVDNYQQPPFTQNAETTEGYQPPPVY

>sp|P01916|HB2S_HUMAN 73 RHNYELDEAVTLQ

NYLFQGRQECYAFNGTQRFLERYIYNREEFARFDSDVGEFRAVTELGRPAAEYWNSQKDL

LEEKRAVPDRVCRHNYELDEAVTLQRRVQPKVNVSPSKKGPLQHHNLLVCHVTDFYPGSI

QVRWFLNGQEETAGVVSTNLIRNGDWTFQILVMLEMTPQQGDVYICQVEHTSLDSPVTVE

WKAQSDSARSKTLTGAGGFVLGLIICGVGIFMHRRSKKVQRGSA

>sp|P16035|TIMP2_HUMAN 187 QAKFFACIKRSDGSCAWYR

MGAAARTLRLALGLLLLATLLRPADACSCSPVHPQQAFCNADVVIRAKAVSEKEVDSGND

IYGNPIKRIQYEIKQIKMFKGPEKDIEFIYTAPSSAVCGVSLDVGGKKEYLIAGKAEGDG

KMHITLCDFIVPWDTLSTTQKKSLNHRYQMGCECKITRCPMIPCYISSPDECLWMDWVTE

KNINGHQAKFFACIKRSDGSCAWYRGAAPPKQEFLDIEDP

>sp|P16035|TIMP2_HUMAN 187 QAKFFACIKRSDGSCAWYRGAAPPKQEF

MGAAARTLRLALGLLLLATLLRPADACSCSPVHPQQAFCNADVVIRAKAVSEKEVDSGND

IYGNPIKRIQYEIKQIKMFKGPEKDIEFIYTAPSSAVCGVSLDVGGKKEYLIAGKAEGDG

KMHITLCDFIVPWDTLSTTQKKSLNHRYQMGCECKITRCPMIPCYISSPDECLWMDWVTE

KNINGHQAKFFACIKRSDGSCAWYRGAAPPKQEFLDIEDP

>sp|O75629|CREG1_HUMAN 68 WGALATISTLEAVR

MAGLSRGSARALLAALLASTLLALLVSPARGRGGRDHGDWDEASRLPPLPPREDAARVAR

FVTHVSDWGALATISTLEAVRGRPFADVLSLSDGPPGAGSGVPYFYLSPLQLSVSNLQEN

PYATLTMTLAQTNFCKKHGFDPQSPLCVHIMLSGTVTKVNETEMDIAKHSLFIRHPEMKT

WPSSHNWFFAKLNITNIWVLDYFGGPKIVTPEEYYNVTVQ

>sp|P19397|CD53_HUMAN 122 IHRYHSDNSTKAAWD

MGMSSLKLLKYVLFFFNLLFWICGCCILGFGIYLLIHNNFGVLFHNLPSLTLGNVFVIVG

SIIMVVAFLGCMGSIKENKCLLMSFFILLLIILLAEVTLAILLFVYEQKLNEYVAKGLTD

SIHRYHSDNSTKAAWDSIQSFLQCCGINGTSDWTSGPPASCPSDRKVEGCYAKARLWFHS

NFLYIGIITICVCVIEVLGMSFALTLNCQIDKTSQTIGL

>sp|P19397|CD53_HUMAN 121 SIHRYHSDNSTKAAWD

MGMSSLKLLKYVLFFFNLLFWICGCCILGFGIYLLIHNNFGVLFHNLPSLTLGNVFVIVG

SIIMVVAFLGCMGSIKENKCLLMSFFILLLIILLAEVTLAILLFVYEQKLNEYVAKGLTD

SIHRYHSDNSTKAAWDSIQSFLQCCGINGTSDWTSGPPASCPSDRKVEGCYAKARLWFHS

NFLYIGIITICVCVIEVLGMSFALTLNCQIDKTSQTIGL

>sp|P20339|RAB5A_HUMAN 123 SPNIVIALSGNKADLANK

MASRGATRPNGPNTGNKICQFKLVLLGESAVGKSSLVLRFVKGQFHEFQESTIGAAFLTQ

TVCLDDTTVKFEIWDTAGQERYHSLAPMYYRGAQAAIVVYDITNEESFARAKNWVKELQR

QASPNIVIALSGNKADLANKRAVDFQEAQSYADDNSLLFMETSAKTSMNVNEIFMAIAKK

LPKNEPQNPGANSARGRGVDLTEPTQPTRNQCCSN

>sp|P61018|RAB4B_HUMAN 83 GALLVYDITSRETYN

MAETYDFLFKFLVIGSAGTGKSCLLHQFIENKFKQDSNHTIGVEFGSRVVNVGGKTVKLQ

IWDTAGQERFRSVTRSYYRGAAGALLVYDITSRETYNSLAAWLTDARTLASPNIVVILCG

NKKDLDPEREVTFLEASRFAQENELMFLETSALTGENVEEAFLKCARTILNKIDSGELDP

ERMGSGIQYGDASLRQLRQPRSAQAVAPQPCGC

>sp|P15814|IGLL1_HUMAN 119 FPPSSEELQANKATLVCL

MRPGTGQGGLEAPGEPGPNLRQRWPLLLLGLAVVTHGLLRPTAASQSRALGPGAPGGSSR

SSLRSRWGRFLLQRGSWTGPRCWPRGFQSKHNSVTHVFGSGTQLTVLSQPKATPSVTLFP

PSSEELQANKATLVCLMNDFYPGILTVTWKADGTPITQGVEMTTPSKQSNNKYAASSYLS

LTPEQWRSRRSYSCQVMHEGSTVEKTVAPAECS

>sp|Q9NRW1|RAB6B_HUMAN 78 LIPSYIRDSTVAVVV

MSAGGDFGNPLRKFKLVFLGEQSVGKTSLITRFMYDSFDNTYQATIGIDFLSKTMYLEDR

TVRLQLWDTAGQERFRSLIPSYIRDSTVAVVVYDITNLNSFQQTSKWIDDVRTERGSDVI

IMLVGNKTDLADKRQITIEEGEQRAKELSVMFIETSAKTGYNVKQLFRRVASALPGMENV

QEKSKEGMIDIKLDKPQEPPASEGGCSC

>sp|P01033|TIMP1_HUMAN 101 NRSEEFLIAGKL

MAPFEPLASGILLLLWLIAPSRACTCVPPHPQTAFCNSDLVIRAKFVGTPEVNQTTLYQR

YEIKMTKMYKGFQALGDAADIRFVYTPAMESVCGYFHRSHNRSEEFLIAGKLQDGLLHIT

TCSFVAPWNSLSLAQRRGFTKTYTVGCEECTVFPCLSIPCKLQSGTHCLWTDQLLQGSEK

GFQSRHLACLPREPGLCTWQSLRSQIA

>sp|P01033|TIMP1_HUMAN 101 NRSEEFLIAGKLQDGLLH

MAPFEPLASGILLLLWLIAPSRACTCVPPHPQTAFCNSDLVIRAKFVGTPEVNQTTLYQR

YEIKMTKMYKGFQALGDAADIRFVYTPAMESVCGYFHRSHNRSEEFLIAGKLQDGLLHIT

TCSFVAPWNSLSLAQRRGFTKTYTVGCEECTVFPCLSIPCKLQSGTHCLWTDQLLQGSEK

GFQSRHLACLPREPGLCTWQSLRSQIA

>sp|P01033|TIMP1_HUMAN 102 RSEEFLIAGKLQDGLL

MAPFEPLASGILLLLWLIAPSRACTCVPPHPQTAFCNSDLVIRAKFVGTPEVNQTTLYQR

YEIKMTKMYKGFQALGDAADIRFVYTPAMESVCGYFHRSHNRSEEFLIAGKLQDGLLHIT

TCSFVAPWNSLSLAQRRGFTKTYTVGCEECTVFPCLSIPCKLQSGTHCLWTDQLLQGSEK

GFQSRHLACLPREPGLCTWQSLRSQIA

>sp|P01033|TIMP1_HUMAN 103 SEEFLIAGKLQDGLL

MAPFEPLASGILLLLWLIAPSRACTCVPPHPQTAFCNSDLVIRAKFVGTPEVNQTTLYQR

YEIKMTKMYKGFQALGDAADIRFVYTPAMESVCGYFHRSHNRSEEFLIAGKLQDGLLHIT

TCSFVAPWNSLSLAQRRGFTKTYTVGCEECTVFPCLSIPCKLQSGTHCLWTDQLLQGSEK

GFQSRHLACLPREPGLCTWQSLRSQIA

>sp|P61006|RAB8A_HUMAN 160 NAFFTLARDIKAKMD

MAKTYDYLFKLLLIGDSGVGKTCVLFRFSEDAFNSTFISTIGIDFKIRTIELDGKRIKLQ

IWDTAGQERFRTITTAYYRGAMGIMLVYDITNEKSFDNIRNWIRNIEEHASADVEKMILG

NKCDVNDKRQVSKERGEKLALDYGIKFMETSAKANINVENAFFTLARDIKAKMDKKLEGN

SPQGSNQGVKITPDQQKRSSFFRCVLL

>sp|P51149|RAB7A_HUMAN 92 APNTFKTLDSWRD

MTSRKKVLLKVIILGDSGVGKTSLMNQYVNKKFSNQYKATIGADFLTKEVMVDDRLVTMQ

IWDTAGQERFQSLGVAFYRGADCCVLVFDVTAPNTFKTLDSWRDEFLIQASPRDPENFPF

VVLGNKIDLENRQVATKRAQAWCYSKNNIPYFETSAKEAINVEQAFQTIARNALKQETEV

ELYNEFPEPIKLDKNDRAKASAESCSC

>sp|P51149|RAB7A_HUMAN 186 FPEPIKLDKNDRAKASA

MTSRKKVLLKVIILGDSGVGKTSLMNQYVNKKFSNQYKATIGADFLTKEVMVDDRLVTMQ

IWDTAGQERFQSLGVAFYRGADCCVLVFDVTAPNTFKTLDSWRDEFLIQASPRDPENFPF

VVLGNKIDLENRQVATKRAQAWCYSKNNIPYFETSAKEAINVEQAFQTIARNALKQETEV

ELYNEFPEPIKLDKNDRAKASAESCSC

>sp|Q9BY32|ITPA_HUMAN 40 IDLPEYQGEPDEISIQK

MAASLVGKKIVFVTGNAKKLEEVVQILGDKFPCTLVAQKIDLPEYQGEPDEISIQKCQEA

VRQVQGPVLVEDTCLCFNALGGLPGPYIKWFLEKLKPEGLHQLLAGFEDKSAYALCTFAL

STGDPSQPVRLFRGRTSGRIVAPRGCQDFGWDPCFQPDGYEQTYAEMPKAEKNAVSHRFR

ALLELQEYFGSLAA

>sp|Q9BY32|ITPA_HUMAN 39 KIDLPEYQGEPDEISIQK

MAASLVGKKIVFVTGNAKKLEEVVQILGDKFPCTLVAQKIDLPEYQGEPDEISIQKCQEA

VRQVQGPVLVEDTCLCFNALGGLPGPYIKWFLEKLKPEGLHQLLAGFEDKSAYALCTFAL

STGDPSQPVRLFRGRTSGRIVAPRGCQDFGWDPCFQPDGYEQTYAEMPKAEKNAVSHRFR

ALLELQEYFGSLAA

>sp|P17900|SAP3_HUMAN 180 RLGCIKIAASLKGI

MQSLMQAPLLIALGLLLAAPAQAHLKKPSQLSSFSWDNCDEGKDPAVIRSLTLEPDPIIV

PGNVTLSVMGSTSVPLSSPLKVDLVLEKEVAGLWIKIPCTDYIGSCTFEHFCDVLDMLIP

TGEPCPEPLRTYGLPCHCPFKEGTYSLPKSEFVVPDLELPSWLTTGNYRIESVLSSSGKR

LGCIKIAASLKGI

>sp|P17900|SAP3_HUMAN 165 TGNYRIESVLSS

MQSLMQAPLLIALGLLLAAPAQAHLKKPSQLSSFSWDNCDEGKDPAVIRSLTLEPDPIIV

PGNVTLSVMGSTSVPLSSPLKVDLVLEKEVAGLWIKIPCTDYIGSCTFEHFCDVLDMLIP

TGEPCPEPLRTYGLPCHCPFKEGTYSLPKSEFVVPDLELPSWLTTGNYRIESVLSSSGKR

LGCIKIAASLKGI

>sp|P17900|SAP3_HUMAN 164 TTGNYRIESVLSSSGKR

MQSLMQAPLLIALGLLLAAPAQAHLKKPSQLSSFSWDNCDEGKDPAVIRSLTLEPDPIIV

PGNVTLSVMGSTSVPLSSPLKVDLVLEKEVAGLWIKIPCTDYIGSCTFEHFCDVLDMLIP

TGEPCPEPLRTYGLPCHCPFKEGTYSLPKSEFVVPDLELPSWLTTGNYRIESVLSSSGKR

LGCIKIAASLKGI

>sp|P01116|RASK_HUMAN 164 RQYRLKKISKEEKTPGC

MTEYKLVVVGAGGVGKSALTIQLIQNHFVDEYDPTIEDSYRKQVVIDGETCLLDILDTAG

QEEYSAMRDQYMRTGEGFLCVFAINNTKSFEDIHHYREQIKRVKDSEDVPMVLVGNKCDL

PSRTVDTKQAQDLARSYGIPFIETSAKTRQRVEDAFYTLVREIRQYRLKKISKEEKTPGC

VKIKKCIIM

>sp|P05090|APOD_HUMAN 77 LNQELRADGTVNQ

MVMLLLLLSALAGLFGAAEGQAFHLGKCPNPPVQENFDVNKYLGRWYEIEKIPTTFENGR

CIQANYSLMENGKIKVLNQELRADGTVNQIEGEATPVNLTEPAKLEVKFSWFMPSAPYWI

LATDYENYALVYSCTCIIQLFHVDFAWILARNPNLPPETVDSLKNILTSNNIDVKKMTVT

DQVNCPKLS

>sp|P05090|APOD_HUMAN 79 QELRADGTVNQIEG

MVMLLLLLSALAGLFGAAEGQAFHLGKCPNPPVQENFDVNKYLGRWYEIEKIPTTFENGR

CIQANYSLMENGKIKVLNQELRADGTVNQIEGEATPVNLTEPAKLEVKFSWFMPSAPYWI

LATDYENYALVYSCTCIIQLFHVDFAWILARNPNLPPETVDSLKNILTSNNIDVKKMTVT

DQVNCPKLS

>sp|P05090|APOD_HUMAN 79 QELRADGTVNQIEGE

MVMLLLLLSALAGLFGAAEGQAFHLGKCPNPPVQENFDVNKYLGRWYEIEKIPTTFENGR

CIQANYSLMENGKIKVLNQELRADGTVNQIEGEATPVNLTEPAKLEVKFSWFMPSAPYWI

LATDYENYALVYSCTCIIQLFHVDFAWILARNPNLPPETVDSLKNILTSNNIDVKKMTVT

DQVNCPKLS

>sp|Q07020|RL18_HUMAN 17 EPKSQDIYLRLLVKLYRFLARRTNST

MGVDIRHNKDRKVRRKEPKSQDIYLRLLVKLYRFLARRTNSTFNQVVLKRLFMSRTNRPP

LSLSRMIRKMKLPGRENKTAVVVGTITDDVRVQEVPKLKVCALRVTSRARSRILRAGGKI

LTFDQLALDSPKGCGTVLLSGPRKGREVYRHFGKAPGTPHSHTKPYVRSKGRKFERARGR

RASRGYKN

>sp|P61224|RAP1B_HUMAN 61 TEQFTAMRDLYMKN

MREYKLVVLGSGGVGKSALTVQFVQGIFVEKYDPTIEDSYRKQVEVDAQQCMLEILDTAG

TEQFTAMRDLYMKNGQGFALVYSITAQSTFNDLQDLREQILRVKDTDDVPMILVGNKCDL

EDERVVGKEQGQNLARQWNNCAFLESSAKSKINVNEIFYDLVRQINRKTPVPGKARKKSS

CQLL

>sp|Q10589|BST2_HUMAN 125 HKLQDASAEVERLRR

MASTSYDYCRVPMEDGDKRCKLLLGIGILVLLIIVILGVPLIIFTIKANSEACRDGLRAV

MECRNVTHLLQQELTEAQKGFQDVEAQAATCNHTVMALMASLDAEKAQGQKKVEELEGEI

TTLNHKLQDASAEVERLRRENQVLSVRIADKKYYPSSQDSSSAAAPQLLIVLLGLSALLQ

>sp|Q969H8|CS010_HUMAN 138 TEEFEVTKTAVAHRPG

MAAPSGGWNGVGASLWAALLLGAVALRPAEAVSEPTTVAFDVRPGGVVHSFSHNVGPGDK

YTCMFTYASQGGTNEQWQMSLGTSEDHQHFTCTIWRPQGKSYLYFTQFKAEVRGAEIEYA

MAYSKAAFERESDVPLKTEEFEVTKTAVAHRPGAFKAELSKLVIVAKASRTEL

>sp|Q9UDX5|MTP18_HUMAN 134 IPIIIHPIDRSVD

MSEPQPRGAERDLYRDTWVRYLGYANEVGEAFRSLVPAAVVWLSYGVASSYVLADAIDKG

KKAGEVPSPEAGRSARVTVAVVDTFVWQALASVAIPGFTINRVCAASLYVLGTATRWPLA

VRKWTTTALGLLTIPIIIHPIDRSVDFLLDSSLRKLYPTVGKPSSS

>sp|P46783|RS10_HUMAN 122 ADRDTYRRSAVPPGAD

MLMPKKNRIAIYELLFKEGVMVAKKDVHMPKHPELADKNVPNLHVMKAMQSLKSRGYVKE

QFAWRHFYWYLTNEGIQYLRDYLHLPPEIVPATLRRSRPETGRPRPKGLEGERPARLTRG

EADRDTYRRSAVPPGADKKAEAGAGSATEFQFRGGFGRGRGQPPQ

>sp|P62937|PPIA_HUMAN 20 VSFELFADKVPKTAEN

MVNPTVFFDIAVDGEPLGRVSFELFADKVPKTAENFRALSTGEKGFGYKGSCFHRIIPGF

MCQGGDFTRHNGTGGKSIYGEKFEDENFILKHTGPGILSMANAGPNTNGSQFFICTAKTE

WLDGKHVVFGKVKEGMNIVEAMERFGSRNGKTSKKITIADCGQLE

>sp|P10124|SRGN_HUMAN 133 LRSLDRNLPSDSQDLGQHGLE

MMQKLLKCSRLVLALALILVLESSVQGYPTQRARYQWVRCNPDSNSANCLEEKGPMFELL

PGESNKIPRLRTDLFPKTRIQDLNRIFPLSEDYSGSGFGSGSGSGSGSGSGFLTEMEQDY

QLVDESDAFHDNLRSLDRNLPSDSQDLGQHGLEEDFML

>sp|P10124|SRGN_HUMAN 31 QRARYQWVRCNPDSNS

MMQKLLKCSRLVLALALILVLESSVQGYPTQRARYQWVRCNPDSNSANCLEEKGPMFELL

PGESNKIPRLRTDLFPKTRIQDLNRIFPLSEDYSGSGFGSGSGSGSGSGSGFLTEMEQDY

QLVDESDAFHDNLRSLDRNLPSDSQDLGQHGLEEDFML

>sp|P10124|SRGN_HUMAN 32 RARYQWVRCNPDSNS

MMQKLLKCSRLVLALALILVLESSVQGYPTQRARYQWVRCNPDSNSANCLEEKGPMFELL

PGESNKIPRLRTDLFPKTRIQDLNRIFPLSEDYSGSGFGSGSGSGSGSGSGFLTEMEQDY

QLVDESDAFHDNLRSLDRNLPSDSQDLGQHGLEEDFML

>sp|P10124|SRGN_HUMAN 28 YPTQRARYQWVRCNPDSNS

MMQKLLKCSRLVLALALILVLESSVQGYPTQRARYQWVRCNPDSNSANCLEEKGPMFELL

PGESNKIPRLRTDLFPKTRIQDLNRIFPLSEDYSGSGFGSGSGSGSGSGSGFLTEMEQDY

QLVDESDAFHDNLRSLDRNLPSDSQDLGQHGLEEDFML

>sp|P68036|UB2L3_HUMAN 43 NPPYDKGAFRIEINFPAEYPFKPP

MAASRRLMKELEEIRKCGMKNFRNIQVDEANLLTWQGLIVPDNPPYDKGAFRIEINFPAE

YPFKPPKITFKTKIYHPNIDEKGQVCLPVISAENWKPATKTDQVIQSLIALVNDPQPEHP

LRADLAEEYSKDRKKFCKNAEEFTKKYGEKRPVD

>sp|P62277|RS13_HUMAN 135 LPPNWKYESSTASA

MGRMHAPGKGLSQSALPYRRSVPTWLKLTSDDVKEQIYKLAKKGLTPSQIGVILRDSHGV

AQVRFVTGNKILRILKSKGLAPDLPEDLYHLIKKAVAVRKHLERNRKDKDAKFRLILIES

RIHRLARYYKTKRVLPPNWKYESSTASALVA

>sp|P69905|HBA_HUMAN 27 AEALERMFLSFPTTKT

MVLSPADKTNVKAAWGKVGAHAGEYGAEALERMFLSFPTTKTYFPHFDLSHGSAQVKGHG

KKVADALTNAVAHVDDMPNALSALSDLHAHKLRVDPVNFKLLSHCLLVTLAAHLPAEFTP

AVHASLDKFLASVSTVLTSKYR

>sp|P09228|CYTT_HUMAN 61 DEYYRRLLRVLRAREQIV

MAWPLCTLLLLLATQAVALAWSPQEEDRIIEGGIYDADLNDERVQRALHFVISEYNKATE

DEYYRRLLRVLRAREQIVGGVNYFFDIEVGRTICTKSQPNLDTCAFHEQPELQKKQLCSF

QIYEVPWEDRMSLVNSRCQEA

>sp|P84243|H33_HUMAN 111 CAIHAKRVTIMPKDIQLA

MARTKQTARKSTGGKAPRKQLATKAARKSAPSTGGVKKPHRYRPGTVALREIRRYQKSTE

LLIRKLPFQRLVREIAQDFKTDLRFQSAAIGALQEASEAYLVGLFEDTNLCAIHAKRVTI

MPKDIQLARRIRGERA

>sp|P06314|KV404_HUMAN 68 QPPKLLIYWASTRE

MVLQTQVFISLLLWISGAYGDIVMTQSPDSLAVSLGERATINCKSSQSILYSSDNKNYLA

WYQQKPGQPPKLLIYWASTRESGVPDRFSGSGSGTDFTLTISSLQAEDVAVYYCQQYYNL

PWTFGQGTKVEIKR

>sp|Q01628|IFM3_HUMAN 86 DRKMVGDVTGAQAYA

MNHTVQTFFSPVNSGQPPNYEMLKEEHEVAVLGAPHNPAPPTSTVIHIRSETSVPDHVVW

SLFNTLFMNPCCLGFIAFAYSVKSRDRKMVGDVTGAQAYASTAKCLNIWALILGILMTIL

LIVIPVLIFQAYG

>sp|Q01628|IFM3_HUMAN 90 VGDVTGAQAYASTAK

MNHTVQTFFSPVNSGQPPNYEMLKEEHEVAVLGAPHNPAPPTSTVIHIRSETSVPDHVVW

SLFNTLFMNPCCLGFIAFAYSVKSRDRKMVGDVTGAQAYASTAKCLNIWALILGILMTIL

LIVIPVLIFQAYG

>sp|Q01628|IFM3_HUMAN 54 VPDHVVWSLFNTL

MNHTVQTFFSPVNSGQPPNYEMLKEEHEVAVLGAPHNPAPPTSTVIHIRSETSVPDHVVW

SLFNTLFMNPCCLGFIAFAYSVKSRDRKMVGDVTGAQAYASTAKCLNIWALILGILMTIL

LIVIPVLIFQAYG

>sp|Q16553|LY6E_HUMAN 36 LKPTICSDQDNYCVT

MKIFLPVLLAALLGVERASSLMCFSCLNQKSNLYCLKPTICSDQDNYCVTVSASAGIGNL

VTFGHSLSKTCSPACPIPEGVNVGVASMGISCCQSFLCNFSAADGGLRASVTLLGAGLLL

SLLPALLRFGP

>sp|P20366|TKN1_HUMAN 111 ALNSVAYERSAMQNYE

MKILVALAVFFLVSTQLFAEEIGANDDLNYWSDWYDSDQIKEELPEPFEHLLQRIARRPK

PQQFFGLMGKRDADSSIEKQVALLKALYGHGQISHKRHKTDSFVGLMGKRALNSVAYERS

AMQNYERRR

>sp|P04431|KV123_HUMAN 65 APKLLIYAASSL

MDMRVPAQLLGLLLLWLRGARCDIQMTQSPSSLSASVGDRVTITCRASQSISNYLNWYQQ

KPGKAPKLLIYAASSLQSGVTSRFSGSGSGTDFTLTISSLQPEDSATYYCQQSYSTLITF

GQGTRLEIK

>sp|P04431|KV123_HUMAN 61 KPGKAPKLLIYAASSL

MDMRVPAQLLGLLLLWLRGARCDIQMTQSPSSLSASVGDRVTITCRASQSISNYLNWYQQ

KPGKAPKLLIYAASSLQSGVTSRFSGSGSGTDFTLTISSLQPEDSATYYCQQSYSTLITF

GQGTRLEIK

>sp|Q96A08|H2B1A_HUMAN 70 DIFERIASEASRLA

MPEVSSKGATISKKGFKKAVVKTQKKEGKKRKRTRKESYSIYIYKVLKQVHPDTGISSKA

MSIMNSFVTDIFERIASEASRLAHYSKRSTISSREIQTAVRLLLPGELAKHAVSEGTKAV

TKYTSSK

>sp|Q8N257|H2B3B_HUMAN 67 VNDIFERIASEASRLAHYN

MPDPSKSAPAPKKGSKKAVTKAQKKDGKKRKRGRKESYSIYVYKVLKQVHPDTGISSKAM

GIMNSFVNDIFERIASEASRLAHYNKRSTITSREVQTAVRLLLPGELAKHAVSEGTKAVT

KYTSSK

>sp|Q9GZQ8|MLP3B_HUMAN 93 TPISEVYESEKDEDGFLY

MPSEKTFKQRRTFEQRVEDVRLIREQHPTKIPVIIERYKGEKQLPVLDKTKFLVPDHVNM

SELIKIIRRRLQLNANQAFFLLVNGHSMVSVSTPISEVYESEKDEDGFLYMVYASQETFG

MKLSV

>sp|P61769|B2MG_HUMAN 82 FYLLYYTEFTPTEKDEYA

MSRSVALAVLALLSLSGLEAIQRTPKIQVYSRHPAENGKSNFLNCYVSGFHPSDIEVDLL

KNGERIEKVEHSDLSFSKDWSFYLLYYTEFTPTEKDEYACRVNHVTLSQPKIVKWDRDM

>sp|P61769|B2MG_HUMAN 84 LLYYTEFTPTEK

MSRSVALAVLALLSLSGLEAIQRTPKIQVYSRHPAENGKSNFLNCYVSGFHPSDIEVDLL

KNGERIEKVEHSDLSFSKDWSFYLLYYTEFTPTEKDEYACRVNHVTLSQPKIVKWDRDM

>sp|P61769|B2MG_HUMAN 84 LLYYTEFTPTEKDEY

MSRSVALAVLALLSLSGLEAIQRTPKIQVYSRHPAENGKSNFLNCYVSGFHPSDIEVDLL

KNGERIEKVEHSDLSFSKDWSFYLLYYTEFTPTEKDEYACRVNHVTLSQPKIVKWDRDM

>sp|P61769|B2MG_HUMAN 85 LYYTEFTPTEKD

MSRSVALAVLALLSLSGLEAIQRTPKIQVYSRHPAENGKSNFLNCYVSGFHPSDIEVDLL

KNGERIEKVEHSDLSFSKDWSFYLLYYTEFTPTEKDEYACRVNHVTLSQPKIVKWDRDM

>sp|P61769|B2MG_HUMAN 24 TPKIQVYSRHPAEN

MSRSVALAVLALLSLSGLEAIQRTPKIQVYSRHPAENGKSNFLNCYVSGFHPSDIEVDLL

KNGERIEKVEHSDLSFSKDWSFYLLYYTEFTPTEKDEYACRVNHVTLSQPKIVKWDRDM

>sp|P61769|B2MG_HUMAN 24 TPKIQVYSRHPAENG

MSRSVALAVLALLSLSGLEAIQRTPKIQVYSRHPAENGKSNFLNCYVSGFHPSDIEVDLL

KNGERIEKVEHSDLSFSKDWSFYLLYYTEFTPTEKDEYACRVNHVTLSQPKIVKWDRDM

>sp|P61769|B2MG_HUMAN 24 TPKIQVYSRHPAENGK

MSRSVALAVLALLSLSGLEAIQRTPKIQVYSRHPAENGKSNFLNCYVSGFHPSDIEVDLL

KNGERIEKVEHSDLSFSKDWSFYLLYYTEFTPTEKDEYACRVNHVTLSQPKIVKWDRDM

>sp|P61769|B2MG_HUMAN 24 TPKIQVYSRHPAENGKS

MSRSVALAVLALLSLSGLEAIQRTPKIQVYSRHPAENGKSNFLNCYVSGFHPSDIEVDLL

KNGERIEKVEHSDLSFSKDWSFYLLYYTEFTPTEKDEYACRVNHVTLSQPKIVKWDRDM

>sp|P61769|B2MG_HUMAN 24 TPKIQVYSRHPAENGKSN

MSRSVALAVLALLSLSGLEAIQRTPKIQVYSRHPAENGKSNFLNCYVSGFHPSDIEVDLL

KNGERIEKVEHSDLSFSKDWSFYLLYYTEFTPTEKDEYACRVNHVTLSQPKIVKWDRDM

>sp|P61769|B2MG_HUMAN 83 YLLYYTEFTPTEKD

MSRSVALAVLALLSLSGLEAIQRTPKIQVYSRHPAENGKSNFLNCYVSGFHPSDIEVDLL

KNGERIEKVEHSDLSFSKDWSFYLLYYTEFTPTEKDEYACRVNHVTLSQPKIVKWDRDM

>sp|P61769|B2MG_HUMAN 87 YTEFTPTEKDEY

MSRSVALAVLALLSLSGLEAIQRTPKIQVYSRHPAENGKSNFLNCYVSGFHPSDIEVDLL

KNGERIEKVEHSDLSFSKDWSFYLLYYTEFTPTEKDEYACRVNHVTLSQPKIVKWDRDM

>sp|P01764|HV303_HUMAN 95 KNTLYLQMNSLRAEDTA

MEFGLSWLFLVAILKGVQCEVQLLESGGGLVQPGGSLRLSCAASGFTFSSYAMSWVRQAP

GKGLEWVSAISGSGGSTYYGDSVKGRFTISRDNSKNTLYLQMNSLRAEDTAVYYCAK

>sp|P60520|GBRL2_HUMAN 75 AIFLFVDKTVPQSSLT

MKWMFKEDHSLEHRCVESAKIRAKYPDRVPVIVEKVSGSQIVDIDKRKYLVPSDITVAQF

MWIIRKRIQLPSEKAIFLFVDKTVPQSSLTMGQLYEKEKDEDGFLYVAYSGENTFGF

>sp|P60520|GBRL2_HUMAN 70 LPSEKAIFLFVDKTVPQSS

MKWMFKEDHSLEHRCVESAKIRAKYPDRVPVIVEKVSGSQIVDIDKRKYLVPSDITVAQF

MWIIRKRIQLPSEKAIFLFVDKTVPQSSLTMGQLYEKEKDEDGFLYVAYSGENTFGF

>sp|P14174|MIF_HUMAN 33 KPPQYIAVHVVPDQ

MPMFIVNTNVPRASVPDGFLSELTQQLAQATGKPPQYIAVHVVPDQLMAFGGSSEPCALC

SLHSIGKIGGAQNRSYSKLLCGLLAERLRISPDRVYINYYDMNAANVGWNNSTFA

>sp|P14174|MIF_HUMAN 33 KPPQYIAVHVVPDQL

MPMFIVNTNVPRASVPDGFLSELTQQLAQATGKPPQYIAVHVVPDQLMAFGGSSEPCALC

SLHSIGKIGGAQNRSYSKLLCGLLAERLRISPDRVYINYYDMNAANVGWNNSTFA

>sp|P14174|MIF_HUMAN 33 KPPQYIAVHVVPDQLM

MPMFIVNTNVPRASVPDGFLSELTQQLAQATGKPPQYIAVHVVPDQLMAFGGSSEPCALC

SLHSIGKIGGAQNRSYSKLLCGLLAERLRISPDRVYINYYDMNAANVGWNNSTFA

>sp|P14174|MIF_HUMAN 91 SPDRVYINYYDMNAAN

MPMFIVNTNVPRASVPDGFLSELTQQLAQATGKPPQYIAVHVVPDQLMAFGGSSEPCALC

SLHSIGKIGGAQNRSYSKLLCGLLAERLRISPDRVYINYYDMNAANVGWNNSTFA

>sp|P14174|MIF_HUMAN 15 VPDGFLSELTQQLAQA

MPMFIVNTNVPRASVPDGFLSELTQQLAQATGKPPQYIAVHVVPDQLMAFGGSSEPCALC

SLHSIGKIGGAQNRSYSKLLCGLLAERLRISPDRVYINYYDMNAANVGWNNSTFA

>sp|P01834|KAC_HUMAN 3 AAPSVFIFPPSDEQLK

TVAAPSVFIFPPSDEQLKSGTASVVCLLNNFYPREAKVQWKVDNALQSGNSQESVTEQDS

KDSTYSLSSTLTLSKADYEKHKVYACEVTHQGLSSPVTKSFNRGEC

>sp|P01834|KAC_HUMAN 36 AKVQWKVDNALQSGN

TVAAPSVFIFPPSDEQLKSGTASVVCLLNNFYPREAKVQWKVDNALQSGNSQESVTEQDS

KDSTYSLSSTLTLSKADYEKHKVYACEVTHQGLSSPVTKSFNRGEC

>sp|P01834|KAC_HUMAN 36 AKVQWKVDNALQSGNS

TVAAPSVFIFPPSDEQLKSGTASVVCLLNNFYPREAKVQWKVDNALQSGNSQESVTEQDS

KDSTYSLSSTLTLSKADYEKHKVYACEVTHQGLSSPVTKSFNRGEC

>sp|P01834|KAC_HUMAN 36 AKVQWKVDNALQSGNSQ

TVAAPSVFIFPPSDEQLKSGTASVVCLLNNFYPREAKVQWKVDNALQSGNSQESVTEQDS

KDSTYSLSSTLTLSKADYEKHKVYACEVTHQGLSSPVTKSFNRGEC

>sp|P01834|KAC_HUMAN 36 AKVQWKVDNALQSGNSQE

TVAAPSVFIFPPSDEQLKSGTASVVCLLNNFYPREAKVQWKVDNALQSGNSQESVTEQDS

KDSTYSLSSTLTLSKADYEKHKVYACEVTHQGLSSPVTKSFNRGEC

>sp|P01834|KAC_HUMAN 36 AKVQWKVDNALQSGNSQES

TVAAPSVFIFPPSDEQLKSGTASVVCLLNNFYPREAKVQWKVDNALQSGNSQESVTEQDS

KDSTYSLSSTLTLSKADYEKHKVYACEVTHQGLSSPVTKSFNRGEC

>sp|P01834|KAC_HUMAN 35 EAKVQWKVDNALQSGN

TVAAPSVFIFPPSDEQLKSGTASVVCLLNNFYPREAKVQWKVDNALQSGNSQESVTEQDS

KDSTYSLSSTLTLSKADYEKHKVYACEVTHQGLSSPVTKSFNRGEC

>sp|P01834|KAC_HUMAN 35 EAKVQWKVDNALQSGNS

TVAAPSVFIFPPSDEQLKSGTASVVCLLNNFYPREAKVQWKVDNALQSGNSQESVTEQDS

KDSTYSLSSTLTLSKADYEKHKVYACEVTHQGLSSPVTKSFNRGEC

>sp|P01834|KAC_HUMAN 35 EAKVQWKVDNALQSGNSQ

TVAAPSVFIFPPSDEQLKSGTASVVCLLNNFYPREAKVQWKVDNALQSGNSQESVTEQDS

KDSTYSLSSTLTLSKADYEKHKVYACEVTHQGLSSPVTKSFNRGEC

>sp|P01834|KAC_HUMAN 79 EKHKVYACEVTHQGLS

TVAAPSVFIFPPSDEQLKSGTASVVCLLNNFYPREAKVQWKVDNALQSGNSQESVTEQDS

KDSTYSLSSTLTLSKADYEKHKVYACEVTHQGLSSPVTKSFNRGEC

>sp|P01834|KAC_HUMAN 79 EKHKVYACEVTHQGLSSP

TVAAPSVFIFPPSDEQLKSGTASVVCLLNNFYPREAKVQWKVDNALQSGNSQESVTEQDS

KDSTYSLSSTLTLSKADYEKHKVYACEVTHQGLSSPVTKSFNRGEC

>sp|P01834|KAC_HUMAN 79 EKHKVYACEVTHQGLSSPV

TVAAPSVFIFPPSDEQLKSGTASVVCLLNNFYPREAKVQWKVDNALQSGNSQESVTEQDS

KDSTYSLSSTLTLSKADYEKHKVYACEVTHQGLSSPVTKSFNRGEC

>sp|P01834|KAC_HUMAN 81 HKVYACEVTHQGL

TVAAPSVFIFPPSDEQLKSGTASVVCLLNNFYPREAKVQWKVDNALQSGNSQESVTEQDS

KDSTYSLSSTLTLSKADYEKHKVYACEVTHQGLSSPVTKSFNRGEC

>sp|P01834|KAC_HUMAN 81 HKVYACEVTHQGLS

TVAAPSVFIFPPSDEQLKSGTASVVCLLNNFYPREAKVQWKVDNALQSGNSQESVTEQDS

KDSTYSLSSTLTLSKADYEKHKVYACEVTHQGLSSPVTKSFNRGEC

>sp|P01834|KAC_HUMAN 81 HKVYACEVTHQGLSSPV

TVAAPSVFIFPPSDEQLKSGTASVVCLLNNFYPREAKVQWKVDNALQSGNSQESVTEQDS

KDSTYSLSSTLTLSKADYEKHKVYACEVTHQGLSSPVTKSFNRGEC

>sp|P01834|KAC_HUMAN 80 KHKVYACEVTHQG

TVAAPSVFIFPPSDEQLKSGTASVVCLLNNFYPREAKVQWKVDNALQSGNSQESVTEQDS

KDSTYSLSSTLTLSKADYEKHKVYACEVTHQGLSSPVTKSFNRGEC

>sp|P01834|KAC_HUMAN 80 KHKVYACEVTHQGL

TVAAPSVFIFPPSDEQLKSGTASVVCLLNNFYPREAKVQWKVDNALQSGNSQESVTEQDS

KDSTYSLSSTLTLSKADYEKHKVYACEVTHQGLSSPVTKSFNRGEC

>sp|P01834|KAC_HUMAN 80 KHKVYACEVTHQGLS

TVAAPSVFIFPPSDEQLKSGTASVVCLLNNFYPREAKVQWKVDNALQSGNSQESVTEQDS

KDSTYSLSSTLTLSKADYEKHKVYACEVTHQGLSSPVTKSFNRGEC
[truncated: 2,802 more chars]
